# 34th Annual Meeting & Pre-Conference Programs of the Society for Immunotherapy of Cancer (SITC 2019): part 2: National Harbor, MD, USA. 10 November 2019

**DOI:** 10.1186/s40425-019-0764-0

**Published:** 2019-11-06

## Poster Presentations

### Biomarkers, Immune Monitoring, and Novel Technologies

#### P501 Dietary deprivation of non-essential amino acids improves anti-PD-1 immunotherapy in murine colon cancer

##### Zehui Li, PhD, Grace Yang, PhD, Shuang Zhou, PhD, Xin Wang, MD, PhD, Xiyan Li, PhD

###### Filtricine, Inc., Santa Clara, CA, United States

####### **Correspondence:** Xin Wang (jimmyxinwang@filtricine.com), Xiyan Li (xiyanli@filtricine.com)

**Background**

Cancer cells require outside supply of some non-essential amino acids (NEAAs) to survive. Dietary deprivation of select (NEAAs) can slow down the growth of multiple solid tumors in mice, creating a new non-drug strategy in cancer treatment. However, deprivation of NEAAs could negatively impact the immune activation, an essential process for immunotherapy, because fast cell proliferation poses a higher demand for building blocks such as NEAAs. It is not clear whether dietary NEAA deprivation could be combined with immunotherapy for better safety-efficacy profiles.

**Methods**

In this study, we tested the effects of NEAA-deprived diets and checkpoint inhibitor anti-PD-1 and anti-PD-L1 in colon cancer using syngeneic mouse model (Balb/c) bearing tumors of mouse colorectal cancer cell line CT-26. Three diets were tested, including a natural rodent diet Teklad ENVIGO Global 16% Protein Rodent Diet (control 1), a formulated NEAA-complete diet COMPLETE (control 2, using amino acid mix in place of protein), and a formulated NEAA-deprived diet FTN203 (treatment, using amino acid mix in place of protein). Both COMPLETE and FTN203 have the same nutritional structures, contain 17% w/w protein equivalent, and are isocaloric. After tumor size-based randomization, these diets were provided to mice ad libitum throughout the whole test. Each of these diets was used alone or combined with anti-PD-1 antibody (i.p., twice per week for 2 weeks) or anti-PD-L1 antibody (i.v., twice per week for 2 weeks).

**Results**

We found 1) On day 24 post tumor implantation, NEAA-deprived diet FTN203 significantly reduced tumor growth when used alone, compared to the group fed with Teklad ENVIGO (by 81%, P=0.0054, unpaired t-test after Welch correction) and COMPLETE (by 81%, P=0.013), respectively; 2) The efficacy of FTN203 is comparable with that of anti-PD-1 or anti-PD-L1 in tumor growth and median survival; 3) FTN203 did not negate the efficacy of anti-PD-1 or anti-PD-L1 immunotherapy antibody when combined; 4) FTN203 significantly improved the efficacy of anti-PD-1 by further reducing the tumor growth (by 80% on day 26, P=0.046) and increasing the median survival (by 5 days or 14%, Log-rank test P= 0.031), against the combo of COMPLETE and anti-PD-1; 5) None of the mono or combo treatments caused body weight loss.

**Conclusions**

Our data supports the use of dietary NEAA deprivation to improve the efficacy of anti-PD-1 or anti-PD-L1 immunotherapy for colorectal cancer without noticeable side effects. With further development, dietary NEAA deprivation may become the promising foundation for a broad spectrum of cancer therapies.

**Ethics Approval**

The study CA-XLI-6 was approved by the CRO's Ethics Board under IACUC approval number 19-015.9.

#### P502 In vitro and in vivo RRx-001 synergy with regorafenib and in vivo attenuation of regorafenib-induced toxicity

##### Bryan Oronsky, MD PhD^1^, Tony Reid, MD PhD^2^, Corey Carter, MD^2^, ^2^, Pedro Cabrales, PhD^3^

###### ^*1*^*EpicentRx Inc, La Jolla, CA, United States*; ^*2*^*EpicentRx, Inc., La Jolla, CA, United States* ; ^*3*^*University of California San Diego (UCSD), La Jolla, CA, United States*

####### **Correspondence:** Christopher Larson (clarson@epicentrx.com)

**Background**

In the Phase 3 CORRECT study, which led to the approval of the multi-kinase inhibitor, Regorafenib, in 3rd/4th line metastatic colorectal cancer, the OS was 6.4 months and the PFS was 1.9 months compared to an OS of 5.0 months and a PFS of 1.7 months for placebo. However, Regorafenib is very poorly tolerated with a Grade 3/4 drug related adverse event rate of 54%, mostly due to hand-foot skin reactions, fatigue and diarrhea, resulting in frequent dose reductions and discontinuations and a general reluctance among GI oncologists to administer it. RRx-001 is a minimally toxic macrophage repolarizing agent in Phase 3 clinical trials that is associated with a reduced side effect profile from these chemotherapy agents. Recent studies have demonstrated the inhibitory impact of M2 macrophages on the activity of tyrosine kinases suggesting that the repolarization of macrophages by RRx-001 may enhance the activity of TKIs.

**Methods**

These experiments determined whether combination therapy with RRx-001 and regorafenib not only enhanced anticancer activity in vitro with HCT-116 and HCT-15 colorectal cell lines and in vivo with HCT 116 and HCT 15 xenografts but also attenuated the toxicity of regorafenib in these two xenografts.

**Results**

The results from these experiments demonstrate that 1) RRx-001 + regorafenib is more effective than either agent alone both in vitro and in vivo and that 2) the addition of RRx-001 to regorafenib attenuates the toxicity of regorafenib in vivo.

**Conclusions**

A clinical trial is planned to investigate the translational potential of the RRx-001 + regorafenib combination. Future experiments will determine whether RRx-001 also enhances the activity and decreases the toxicity of other tyrosine kinase inhibitors such as sorafenib, sunitinib, dasatinib, imatinib, lapatinib, and cabozantinib, all of which possess similar efficacy and safety profiles, not only in colorectal cancer but also other tumor types.

#### P503 Local treatment with adenovirus expressing TNF-α and IL-2 proteins promotes abscopal effect in mice receiving anti-PD-1 immunotherapy

##### Dafne Quixabeira, MSc, Victor Cervera-Carrascon, MS, Joao Santos, MS, Riikka Havunen, Akseli Hemminki, MD, PhD

###### University of Helsinki, Helsinki, Finland

####### **Correspondence:** Akseli Hemminki (akseli.hemminki@helsinki.fi)

**Background**

Treatment of metastatic solid tumors remains a challenge in oncology, especially in the context of locally given therapies. Some responses in non-treated metastases were described in the middle of the last century after using local radiotherapy in breast cancer patients. This phenomenon was named abscopal effect[1]. Nowadays, this effect is known to be elicited by systemic adaptive immune responses against tumor cells, following local treatment. Of note, abscopal effect remains somewhat limited in the clinic mainly due to the suppressive microenvironment surrounding cancer cells. The systemic use of immune checkpoint inhibitors, such as anti-CTLA-4 (anti cytotoxic T-lymphocyte-associated protein 4) and anti-PD-1 (anti programmed cell-death protein 1), has shown promise in the treatment of various tumor types but only a minority of patients respond[2]. Oncolytic virotherapy is an interesting strategy that has re-emerged in the last few decades. It is able to prime the host immune system against tumor epitopes, generating anti-tumor immunity and thus theoretically complementing anti-PD-1 and anti-PDL-1 therapy in an appealing manner[3,4]. The challenge with oncolytic immunotherapy is that not all metastases can usually be injected. Therefore, it is of importance to study if local treatment can induce distant responses. While our previous work has shown synergy between oncolytic adenovirus and anti-PD-1, here we sought to establish if local adenovirus injection can impact also non-injected tumors in pre-clinical models in the context of systemic anti-PD-1 therapy.

**Methods**

We utilized a murine melanoma model (B16.OVA) where each mouse had bilateral tumors. All mice received anti-PD-1 systemically, but only one tumor received local virotherapy with the non-replicative adenovirus. Both tumors were then studied and compared for possible systemic effects following local treatment, including immune responses and anti-tumor efficacy.

**Results**

Improvement in the overall survival was seen in the group receiving both therapies (anti-PD-1 plus virus) compared to the monotherapy groups. In particular, treatment with anti-PD-1 plus virus was the only group that presented a complete tumor regression of both tumors. Likewise, this therapeutic group presented significantly better tumors control in non-injected tumors than the other treatment groups. Further information, including full tumor growth and immunological mechanism-of-action data will be presented.

**Conclusions**

These two cancer immunotherapies seem to be a promising approach that could increase survival upon clinical translation. It is of importance that systemic anti-tumor effects result from local injection of adenovirus coding TNF- α and IL-2, even in the absence of virus replication.

**Acknowledgements**

We thank the Biomedicum FACS Core facility and the Animal the Finnish Centre for Laboratory Animal Pathology (FCLAP) at the University of Helsinki, for their expert assistance. This is study was supported by Jane and Aatos Erkko Foundation, HUCH Research Funds (EVO), Sigrid Juselius Foundation, Finnish Cancer Organizations, University of Helsinki, The Finnish Society of Sciences and Letters, and TILT Biotherapeutics Ltd.

**Ethics Approval**

All experiments described here have been approved by The Gene Technology Board of Finland, by the Experimental Animal Committee of the University of Helsinki and the Provincial Government of Southern Finland, under the license number ESAVI/7755/04.10.07/2016.

**References**

1. Mole RH. Whole Body Irradiation—Radiobiology or Medicine? Br J Radiol. 1953; 305: 234-241.

2. Demaria S, Kawashima N, Yang AM, Devitt ML, Babb JS, Allison JP, Formenti SC. Immune-Mediated Inhibition of Metastases after Treatment with Local Radiation and CTLA-4 Blockade in a Mouse Model of Breast Cancer. Clin Cancer Res. 2005; 15:728-34.

3. Havunen R, Santos JM, Sorsa S, Rantapero T, Lumen D, Siurala M, Airaksinen AJ, Cervera- Carrascon V, Tähtinen S, Kanerva A, and Hemminki A. Abscopal Effect in Non-injected Tumors Achieved with Cytokine-Armed Oncolytic Adenovirus. Mol Ther Oncolytics. 2018; 11: 109–121.

4. Cervera-Carrascon V, Siurala M, Santos JM, Havunen R, Tähtinen S, Karell P, Sorsa S, Kanerva A, Hemminki A.TNFa and IL-2 armed adenoviruses enable complete responses by anti-PD-1 checkpoint blockade. Oncoimmunology. 2018; 9;7(5):e1412902. doi: 10.1080/2162402X.2017.1412902. eCollection 2018.

#### P504 Arginase therapy combines effectively with checkpoint blockade or agonist anti-OX40 immunotherapy to control tumor growth

##### Annah Rolig, Ph D^1^, Scott Rowlinson, PhD^2^, Melissa Kasiewicz, BS^1^, Mark Badeaux, PhD^2^, William Redmond, PhD^1^ , Giulia Agnello, PhD^2^, Leslie Priddy, BS^2^, Danlee Enzler, BS^2^, Jason Wiggins, BS^2^, Christopher Daige^2^, Jessica Van Cleef^2^

###### ^*1*^*Earle A. Chiles Research Institute, Portland, OR, United States* ; ^*2*^*Aeglea Biotherapeutics, Inc., Austin, TX, United States*

####### **Correspondence:** William Redmond (William.Redmond@providence.org)

**Background**

Metabolic dysregulation is a hallmark of cancer; as such, many tumors exhibit auxotrophy for various nutrients as they are unable to meet the demand for these metabolites through endogenous production. Arginine auxotrophic tumors, for instance, are highly sensitive to depletion of systemic arginine. Pegzilarginase, a human arginase 1 enzyme engineered to have superior stability and enzymatic activity relative to the native human arginase 1 enzyme, depletes systemic arginine by converting it to ornithine and urea. Therapeutic administration of pegzilarginase in the setting of arginine auxotrophic tumors exerts direct anti-tumor activity by starving the tumor of exogenous arginine. Pegzilarginase monotherapy has shown significant anti-tumor activity in various arginine auxotrophic human and murine tumor models [1]. We hypothesized that in addition to direct effects, pegzilarginase treatment indirectly augments anti-tumor immunity through increased antigen presentation, thus making pegzilarginase a prime candidate for combination therapy with immuno-oncology (I-O) agents.

**Methods**

Tumor-bearing mice (CT26; MC38; MCA-205) received pegzilarginase (3 mg/kg; 1x/wk; ip) in combination with aPD-L1 (10mg/kg; 2x/wk; ip), aCTLA-4 (10mg/kg; 2x/wk; ip), or agonist aOX40 mAb (250ug, 2x; ip). The activation status of CD4+, CD8+, and NK cells in the tumor (7, 10, and 17 days post-treatment) was evaluated by flow cytometry. Effects on immune subsets were evaluated by flow cytometry and single cell RNA sequencing of sorted CD45+ cells from tumors 3 days post-treatment. Data represents the result of 2-3 independent experiments (n=9-18/group). Statistical significance was determined using a 1-way ANOVA with a p-value cut-off of 0.05.

**Results**

Pegzilarginase monotherapy increased MHC expression on antigen presenting cells (APCs) and the frequency of intratumoral CD8+ T cells relative to controls (p<0.01). Further, administration of pegzilarginase in combination with I-O agents, including aPD-L1 (p<0.01) and aOX40 (p<0.05), resulted in increased therapeutic benefit compared to I-O agents alone, including an increase in complete tumor regression. Combination therapy evinced the greatest number of activated intratumoral CD8+ T cells and elevated systemic levels of IFN-gamma.

**Conclusions**

Combination pegzilarginase/immunotherapy induces robust anti-tumor immunity characterized by increased intratumoral CD8+ T cells and M1-polarized macrophages. Our data suggests two potential mechanisms of synergy between pegzilarginase and I-O agents: 1) Increased intratumoral MHC expression on APCs and tumor antigen presentation; and 2) Increased presence of M1-like anti-tumor macrophages. These data support the clinical evaluation of T cell agonists and/or checkpoint inhibitors in conjunction with pegzilarginase for the treatment of patients with cancer.

**Ethics Approval**

All mice were maintained under specific pathogen-free conditions in the Providence Portland Medical Center animal facility. Experimental procedures were performed according to the National Institutes of Health Guide for the Care and Use of Laboratory Animals and in accordance with the Earle A. Chiles Research Institute Institutional Animal Care and Use Committee (Animal Welfare Assurance No. A3913–01).

**References**

1. Agnello G et al. Depleting blood arginine with AEB1102 (Pegzilarginase) exerts additive anti-tumor and synergistic survival benefits when combined with immunomodulators of the PD-1 pathway. SITC Annual Meeting, 2017, #275.

#### P505 Abscopal immunity achieved via in situ vaccination using a novel combination of cryoablation and Interleukin-12

##### Maura Vrabel, Francis Gillam, PhD, Jared Hopkins, Khue Nguyen, PhD, David Zaharoff, PhD

###### NC State University, Raleigh, NC, United States

####### **Correspondence:** David Zaharoff (dazaharo@ncsu.edu)

**Background**

Clinically, cryoablation is used to treat various types of cancers including certain prostate, liver and kidney tumors. Because it liberates massive amounts of tumor antigen, cryoablation can potentially serve as an in situ vaccine that is patient- and tumor-specific. However, numerous studies report that cryoablation alone rarely provides abscopal immunity [1]. Therefore, we hypothesized that direct injection of an immunomodulator (IM) following cryoablation (cryo) can induce tumor-specific immunity, controlling both treated and untreated tumors.

**Methods**

MB49 cells were purchased from ATCC. Female C57BL/6 mice were purchased from Jackson Laboratory. Primary tumor implantation, 3x105 cells were injected subcutaneously (s.c.) in the right flank. Rechallenge, 3x105 cells (s.c.) were implanted in the left flank of cured mice. Bilateral model, 3x105 and 1.5x105 cells were s.c. injected in the right and left flanks respectively on the same day. Tumor volume calculation, 0.5*a*b2 given perpendicular long (a) and short (b) dimensions. Tumors, 200-500 mm3 ,were cryoablated with three cycles of freeze/thaw using Visual-ICETM Cryoablation System (Galil Medical/BTG). Unless noted otherwise, an IM was directly injected into the tumor bed two days post cryo. R848 (Resiquimod) 10 μg/mouse, DMXAA 500 μg/mouse, and Interleukin-12 (IL-12) 1 μg/mouse in 1.5% (w/v) chitosan acetate (CS) dissolved in dPBS.

**Results**

Cryoablation of a single primary tumor resulted in a tumor-free survival rate of 40%. The addition of R848 or DMXAA did not significantly enhance survival, while adding CS/IL-12 resulted in a 90% survival rate (Figure 1). Cured mice were rechallenged with live MB49 cells and then given no further treatment. As of day 70 post rechallenge, the number of tumor free mice out of total survivors were cryo alone (3/4), cryo + CS/IL-12 (5/7), cryo + R848 (2/5), and cryo + DMXAA (1/4). In the bilateral model, the median survival of the cryo + CS/IL-12 group was significantly longer (p = 0.0016) at 49.5 days compared to 26 days for cryo alone (Figure 2). Furthermore, one mouse treated with cryo + CS/IL-12 experienced a complete response up to 90 days post tumor implantation.

**Conclusions**

IL-12 formulated with chitosan and delivered after cryoablation is superior in treating not only a primary tumor, but also in inducing an abscopal effect on a distant, untreated tumor. Of note, neither DMXAA nor R848 was formulated in a delivery vehicle, as we have yet to optimize these agonists. This work supports further study of cryoablation and IL-12 for in situ vaccination and cancer immunotherapy.

**Acknowledgements**

This work is supported by BTG, the NC State University Provost’s Fellowship, and startup funds provided by the College of Engineering at NC State University.

**Ethics Approval**

The study was approved by the NC State University's Ethics Board, approval number 17-052.

**References**

1. Abdo J, Cornell DL, Mittal SK, Agrawal DK. Immunotherapy Plus Cryotherapy: Potential Augmented Abscopal Effect for Advanced Cancers. Front Oncol. 2018;8:85.

**Fig. 1:**
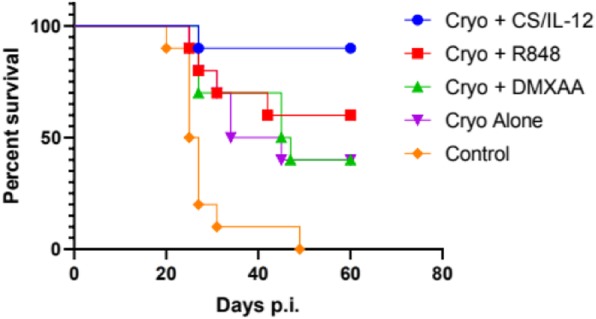
**(abstract P505).** Survival of single primary tumor model

**Fig. 2:**
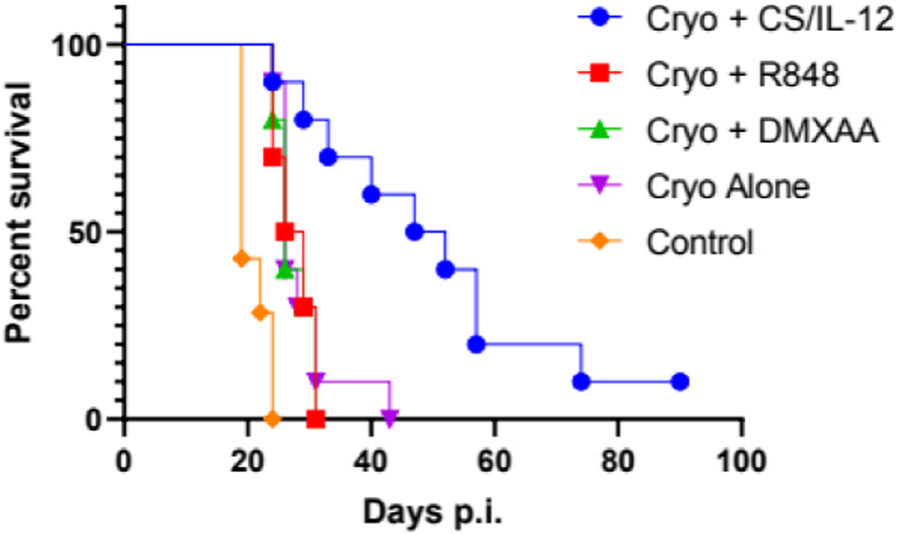
**(abstract P505)** Survival of bilateral model

#### P506 Targeting inflammation in the tumor micro-environment to improve radiation and immunotherapy

##### Debashree Basudhar, PhD, Veena Somasundaram, PhD, Robert Cheng, David Scheiblin, Erika Palmieri, William Heinz, Noemi Kedei, Jinqiu Chen, Daniel McVicar, PhD, Stephen Lockett, David Wink, Lisa Ridnour, PhD

###### National Cancer Institute, Frederick, MD, United States

####### **Correspondence:** Lisa Ridnour (ridnourl@mail.nih.gov)

**Background**

The presence of tumor infiltrating lymphocytes in breast cancer (BC) is associated with improved survival. More recently a study found that increased CD8 cells and Th17 cells are specifically associated with triple negative breast cancer (TNBC) patients, a highly aggressive subclass of breast cancer. However, they undergo functional reprogramming in the tumor micro-environment (TME) evident from decreased IFN-*γ* and granzyme B. These immune escape mechanisms contribute to inability of the immune system to control tumor progression. Thus modulation of TME is necessary to effectively target the tumor.

Radiation therapy (RT) is commonly used in cancer patients including BC. Focal radiation limits systemic side effects and acts as immune modulator. Currently there is no approved immunotherapy available for BC patients, early data from several ongoing clinical trials show activity in various subclasses of BC including TNBC. It is reported that PD-L1 is high in 20% of TNBCs and COX2 may be involved in its regulation in tumor-infiltrating myeloid cells. This led us to hypothesize that modulation of inflammation associated biomarkers in the TME would increase the efficacy of RT and immunotherapy by amplifying anti-tumor immunity.

**Methods**

We investigated the effect of nitric oxide synthase2 (NOS2) or cycloxygenase2 (COX2) inhibition using commercially available inhibitor on radiation and αPD-L1 induced tumor growth delay and lung metastases in murine model of TNBC using 4T1 cell line implanted in flank of Balbc mice. Change in immune cell populations in the TME was investigated using confocal microscopy, CO-Detection by indEXing (CODEX) technology and flow-cytometry. TME associated metabolic changes was investigated using seahorse bioanalyzer and LC-MS/MS. We also measured the levels of inflammation associated cytokines in serum using LEGENDplex.

**Results**

We found that RT induced inflammation associated biomarkers NOS2 and COX2 in the TME, specifically in the tumor cells. We have shown that co-expression of pro-inflammatory enzymes NOS2 and COX2 is a powerful prognostic indicator of poor outcome (HR=21) among ER– patients which in turn drive major oncogenic pathways. We demonstrated that co-treatment with COX2 inhibitor led to tumor growth delay and reduced metastases. COX2 inhibition along with RT induced proliferation of cytotoxic CD8, dendritic cells and pro-inflammatory macrophages while Treg activity was reduced. Furthermore, CODEX showed correlation between decreased macrophage population and increased CD8 infiltration suggesting role of macrophages in inhibiting CD8 infiltration.

**Conclusions**

We demonstrated that co-treatment of COX2 inhibitor with radiation and immunotherapy effectively activated the immune system by changing the TME to support tumor clearance.

#### P507 Radiation-induced modulation of TREX1, a checkpoint for innate immune-sensing of DNA damage

##### Maud Charpentier, PhD, Claire Vanpouille-Box, PhD, Camille Daviaud, Sandra Demaria, MD

###### Weill Cornell Medicine, New York, NY, United States

####### **Correspondence:** Sandra Demaria (szd3005@med.cornell.edu)

**Background**

We have shown in both murine tumor models and patients that focal tumor radiotherapy synergizes with CTLA-4 blockade to induce systemically effective anti-tumor immunity in otherwise resistant tumors [1,2,3]. Mechanistically, radiation-induced viral mimicry is critical for priming of anti-tumor CD8+ T cells, and is triggered by cytosolic DNA that stimulates cancer-cell intrinsic interferon type I release [2,3]. The exonuclease TREX1 abrogates this response by clearing cytosolic DNA thus acting as a central regulator of RT-induced anti-tumor immunity and we demonstrated that Trex1 gene expression is upregulated by radiation doses of >12Gy per fractions in most carcinoma cells [2]. Thus, we investigated the mechanisms responsible for inducing Trex1 expression in irradiated cancer cells.

**Methods**

The mouse mammary carcinoma TSA cells were irradiated in vitro with 8 or 20 Gy, followed by chromatin immunoprecipitation (ChIP) assay to determine if the transcription factor cFos was bound to Trex1 promoter region 4 hours after irradiation. A doxycycline-inducible shRNA was used to knockdown cFos expression in TSA cells.

**Results**

The transcription factor c-Fos was found to be phosphorylated in TSA cells treated with a radiation dose that induces TREX1 upregulation (20Gy), but not in 8Gy-treated cells. c-Fos has been previously implicated in the induction of Trex1 in response to UV irradiation [4]. Therefore, it was a good candidate as a regulator of Trex1 in 20 Gy-treated TSA cells. Consistently, a ChIP assay demonstrated that 20 Gy but not 8Gy irradiation induced cFos binding to Trex1 promoter. shRNA-mediated c-Fos knockdown abrogated Trex1 upregulation induced by after 20 Gy and restored cytosolic DNA accumulation and type I interferon pathway activation.

**Conclusions**

Overall, these data identify cFos as the transcription factor that regulates Trex1 gene expression in a radiation dose-dependent way. We are currently investigating the signals upstream of cFos. Results of this work will identify potential actionable targets to increase radiation-induced anti-tumor immunity.

**Acknowledgements**

Grant support: NIH R01CA201246

**References**

1. Demaria et al., 2005 Immune-mediated inhibition of metastases after treatment with local radiation and CTLA-4 blockade in a mouse model of breast cancer. Clin Cancer Res.

2. Vanpouille-Box, C. et al. 2017. DNA exonuclease Trex1 regulates radiotherapy-induced tumour immunogenicity. Nat Commun

3. Formenti, S. C. et al. 2018. Radiotherapy induces responses of lung cancer to CTLA-4 blockade. Nat Med 24: 1845-1851.

4. Christmann M. et al.,2010 Three prime exonuclease I (TREX1) is Fos/AP-1 regulated by genotoxic stress and protects against ultraviolet light and benzo(a)pyrene-induced DNA damage. Nucleic Acids Res. 38:6418-32.

#### P508 Combination of a radiation-enhancing nanoparticle, radiotherapy, and immune checkpoint inhibitors for treating metastasized lung cancer in mice

##### Yun Hu^1^, James Welsh^1^ , Sébastien Paris^2^, Angelica Cortez^1^

###### ^*1*^*MD Anderson Cancer Center, Houston, TX, United States*; ^*2*^*Nanobiotix, Paris, France*

####### **Correspondence:** James Welsh (jwelsh@mdanderson.org)

**Background**

Radiotherapy has been routinely used to treat lung cancer alone or in combination with chemotherapy or surgery. Despite the tremendous benefits of radiotherapy in localized tumor control, it is not very effective for treating metastasized lung cancers. In addition, the application of radiotherapy is limited by its damage to the surrounding healthy tissues.

**Methods**

In this study, we combined radiotherapy with checkpoint inhibitor immunotherapy and a radio-enhancing nanoparticle NBTXR3 (R3) to extend the application of radiotherapy to treating metastasized lung cancer in mice.

**Results**

In both the 344SQ-anti-PD-1 sensitive and 344SQ-anti-PD-1 resistant lung cancer models, we found that the combination of anti-PD1 and radiotherapy-induced robust immune responses, which resulted in significantly better control of both the local tumor (implanted on the right leg of the mice) and the metastasis (implanted on the left leg of the mice) than either immunotherapy or radiotherapy alone. In addition, we found that R3 significantly enhanced the efficacy of combined radiotherapy and immunotherapy in the two lung tumor models to control the local tumor and metastasis. The results also showed that with the supplement of R3, radiation plus anti-PD-1 achieved similar control of the metastasis under 3x8Gy compared to 3X12Gy, indicating that the treatment benefits acquired at a high dosage of radiation can now be achieved at lower dosages with the presence of R3. We also found that the triple combination of R3, radiotherapy, and anti-PD-1 significantly reduced the number of metastases in lungs compared to other combinations of these therapies. The immunoprofiling of the tumors after treatment showed that triple combination therapy reduced the population of regulatory T cells and M2 macrophages, resulting in favorable conditions for immune-mediated cancer cell killing.

**Conclusions**

In conclusion, R3 considerably enhances the the ability of the combination of radiotherapy and immunotherapy to control both the local tumor and the metastasis in both the 344SQ-anti-PD-1 sensitive and 344SQ-anti-PD-1 resistant lung cancer models. In addition, R3 allows a lower dosage of radiation to achieve similar treatment outcome compared to a higher dosage of radiation.

#### P509 Myeloid cell-selective STAT3 inhibition sensitizes head and neck cancers to radiation therapy and stimulates T-cell-dependent tumor regression

##### Dayson Moreira, PhD^2^, Marcin Kortylewski, PhD^2^ , Sagus Sampath^2^, Haejung Won^2^, Chongkai Wang^2^, Peter Lee, MD^2^, Ellie Maghami^2^, Erminia Massarelli, MD, PhD, MS^2^, William Spanos, MD^2^, Seok White^2^

###### ^*1*^*Beckman Research Institute at City of Hope, Duarte, CA, United States* ; ^*2*^*Sanford Health, Sioux Falls, SD, United States*

####### **Correspondence:** Marcin Kortylewski (mkortylewski@coh.org)

**Background**

Therapeutic and immunogenic effects of radiation therapy (RT) are often constrained by tolerogenic activity of the tumor microenvironment. We recently found that combined modality therapy (CMT), using RT with concurrent cisplatin or cetuximab treatment, triggers potentially tolerogenic STAT3 signaling in circulating myeloid immune cells in patients with stage III-IV head and neck squamous cell carcinoma (HNSCC). Here, we extended these studies to tumor-containing lymph nodes biopsies collected from the same patients before and after two-week CMT. The clinical observations were later verified in feasibility studies testing the effect of STAT3 targeting on sensitivity to radiation therapy in HPV-positive and HPV-negative HNSCC tumor models in mice.

**Methods**

The STAT3 activity in immune populations infiltrating HNSCC tumors was analyzed in patients' derived fixed or frozen fine-needle biopsies. We used the multicolor immunofluorescent analysis and also gene expression analysis using Nanostring assay. To assess the effect of targeting STAT3 in the identified tolerogenic myeloid cell populations, we used TLR9-targeted STAT3 antisense oligonucleotides (CpG-STAT3ASO) in combination with local tumor irradiation.

**Results**

Using the multicolor immunofluorescent analysis, we found a significant increase in the percentage of CD163+ M2 macrophages with activated STAT3 and a concurrent reduction in a number of CD8+ T-cells in tumors post-treatment. Similarly, the Nanostring analysis of frozen tumor-containing lymph nodes biopsies detected significant increase in the percentage M2 macrophages (CD163, CD206), with the upregulation of Th2 (IL4, IL5) and immunosuppressive regulators (ARG1, IL10, LIF, TGFB1). These results suggested that STAT3 activation in tumor-associated myeloid cells can dampen immunogenic effects of CMT. To test this hypothesis, we combined single-dose localized RT with intratumoral injections of myeloid cell-specific STAT3 antisense oligonucleotide (CpG-STAT3ASO) against three mouse models of HPV-positive (mEERL) or HPV-negative (MOC2 and mEERL/sh) head and neck cancers. The combination of both strategies resulted in a strong synergistic effect with frequent eradication of mEERL and MOC2 tumors in at least half of treated mice. Regardless of the HPV status, effects of RT/CpG-STAT3ASO therapy were associated with reduced numbers of tumor-associated M2 macrophages and increased percentage of CD8+ T-cells and/or CD8:Treg ratio in MOC2 and mEERL tumors. In addition, we observed recruitment of M1 macrophages with enhanced expression of MHC class II and costimulatory molecules in tumor-draining lymph nodes after RT/CpG-STAT3ASO treatments.

**Conclusions**

Our clinical observations and animal study results suggest that myeloid cell-targeted inhibition of STAT3 together with CpG-mediated immunostimulation dramatically augment the outcome of radiation therapy against HPV-positive and HPV-negative HNSCC.

**Ethics Approval**

The clinical protocol including the relevant informed consent form was approved by the institutional review board at City of Hope (IRB-14255) and the study was conducted in accordance with the amended Declaration of Helsinki and the International Conference on Harmonization Guidelines.

#### P510 Treatment of murine mammary carcinoma with focal radiation and immune checkpoint inhibition results in reduced immune suppression and enhanced cytotoxic immune cell activity

##### Philip Lapinski, PhD, Maryland Franklin, PhD , David Draper, PhD, Scott Wise, BS

###### MI Bioresearch, Ann Arbor, MI, United States

####### **Correspondence:** Maryland Franklin (mfranklin@mibioresearch.com)

**Background**

Despite the success of immune checkpoint blockade in treating many types of cancers, there remain many that are insensitive to these treatments. We have previously shown that focal radiation can sensitize the immunologically “cold” 4T1 tumor model to immune checkpoint inhibition. Combination treatment with radiation and anti-CTLA4 antibody resulted in decreased numbers of infiltrating regulatory T cells as well as B cells in the tumors. While numbers of CD8+ T cells were not increased in tumors that received combination treatment, the cells displayed a more activated phenotype. We hypothesized that combination treatment decreased numbers of regulatory cell types and led to increased cytotoxic activity of CD8+ T cells, Natural Killer (NK) cells, and Natural Killer T (NKT) cells in the tumor microenvironment.

**Methods**

We orthotopically implanted 4T1 mammary carcinoma cells into BALB/c mice and treated with anti-mCTLA4 antibody, focal radiation, or both. Tumors were analyzed by flow cytometry, utilizing the MI-CompLeukocyte™ panels with absolute cell counting, as well as the MI-T Effector/Memory™ panel and a custom intracellular cytokine panel.

**Results**

The 4T1 tumor model is typically unresponsive to immune checkpoint inhibition. As expected, we saw a synergistic effect of combination treatment on tumor burden compared with radiation alone (77.1% vs. 42.3% TGI, respectively), suggesting that radiation primes the tumors to be more responsive to checkpoint inhibition. Our analysis showed that there was a 2.4-fold increase in the percentage of CD8+ T effector memory cells with a corresponding 9.4-fold decrease in CD8+ T central memory cells. The percentage of T effector memory cells was inversely correlated with tumor volume in the combination group, suggesting that this cell type could be directly responsible for the decreased tumor burden. Consistent with the increased effector memory phenotype, the percentage of CD8+ T cells secreting Granzyme B was increased 2-fold with combination treatment compared to radiation alone. Combination treatment also increased the percentage of NKT cells that secreted Interferon-γ, suggesting that the increased inflammatory cell phenotype is a broader phenomenon and not specific to CD8+ T cells.

**Conclusions**

Taken together, these data suggest that synergistic treatment with anti-mCTLA4 and focal radiation can induce the tumor immune microenvironment to change to a more inflammatory, and less suppressive, state than either treatment alone. Reduction in numbers of regulatory T cells and B cells resulted in increased percentages CD8+ T cells and NKT cells with a phenotype consistent with improved anti-tumor activity.

**Ethics Approval**

This study was approved by the IACUC at MI Bioresearch under Protocol MI05 - Subcutaneous Tumor Models for Evaluation of Test Agents.

#### P511 Mechanistic insights into combination low dose targeted radionuclide and checkpoint blockade treatment to turn a “cold” tumor “hot”

##### Ravi Patel, MD, PhD, Reinier Hernandez, PhD, Peter Carlson, BS, Ryan Brown, Luke Zangl, Amber Bates, Ian Arthur, Justin Jagodinsky, Joseph Grudinski, Amy Erbe, PhD, Jamey Weichert, Paul Sondel, MD, PhD, Zachary Morris, MD, PhD, Ravi Patel, MD, PhD

###### University of Wisconsin, Madison, WI, United States

####### **Correspondence:** Zachary Morris (zmorris@humonc.wisc.edu)

**Background**

Radiation therapy (RT) to enhances the efficacy of immune checkpoint inhibition (ICI) in multiple immunologically “cold” preclinical tumor models[1,2]. However, the optimal approach to delivering immunostimulatory RT has not been established. We have developed a novel strategy to deliver immunostimulatory low dose (LD) RT to all tumor sites in the setting of metastatic disease by using targeted radionuclide therapy (TRT), with a tumor-selective alkyl-phosphocholine (90Y-NM600). Here we evaluate the mechanistic basis for an observed cooperative interaction between LDTRT and ICI in an immunologically cold murine melanoma model that does not respond to ICI alone.

**Methods**

Mice were engrafted with B78 melanoma tumors, 80-90 mm3, and were randomized to the following treatment groups: vehicle only (VO), anti-CTLA4 (C4), 90Y-NM600 TRT (2.5 Gy tumor dose), external beam RT (EBRT, 2.5 or 12 Gy), or combination TRT+C4. To compare LDRT delivered via EBRT or TRT or to high dose EBRT, tumors were harvested 1, 7, and 14 days after RT. We performed flow cytometry (FC) and qPCR on these specimens to evaluate immune cell infiltrates and gene expression. To determine treatment effects, tumors treated with VO, C4, TRT, or TRT+C4 were harvested on Day 25 after initiation of therapy. Immune infiltrates were quantified by FC in these samples, cytokine levels via multiplex ELISA, and T cell clonal analysis via T cell receptor (TCR) sequencing.

**Results**

Comparison of RT dose effects demonstrated increased ratios of CD8+ T cells to CD4+FoxP3+ regulatory T cells in the TRT group, compared to the EBRT groups at Day 1, as well as increased myeloid cell (CD11b+) and NK cell infiltrate with TRT. Gene expression analysis showed peak apoptosis markers on Day 1, peak type I interferon signaling (ISG) and chemokine expression on Day 7. High dose EBRT had significantly greater ISG expression than LD-RT (p <0.05). Following combined treatment with TRT + C4, tumors showed significantly greater CD8+ T cells and γδ T cells, compared to single agent or untreated control groups. Multiplex ELISA showed increased concentrations of multiple cytokines with the notable exception of IL-10 in tumors from mice receiving TRT+C4 (Figure 1). In contrast to prior studies with high dose EBRT, LDTRT stimulated increased TCR clonal expansion but did not increase TCR diversity when combined with C4 ICI.

**Conclusions**

Our results demonstrate a cooperative therapeutic interaction between TRT+ICI in a murine tumor model that is functionally immunologically cold and does not respond to ICI alone.

**Acknowledgements**

Research material support for this work was provided by Bristol Myers Squibb. This work was supported by the National Institute of Health under the following awards U01CA233102, the University of Wisconsin Carbone Cancer Center Support Grant P30 CA014520, RSNA Fellow Award, ASCO YIA, and the Bentson Translational Research Fellowship.

**References**

1. Twyman-saint victor C, Rech AJ, Maity A, et al. Radiation and dual checkpoint blockade activate non-redundant immune mechanisms in cancer. Nature. 2015;520(7547):373-7.

2. Patel, RB, Czapar, AE, Fiering, S et al. Radiation therapy combined with cowpea mosaic virus nanoparticle in situ vaccination initiates immune-mediated tumor regression. ACS Omega 2018. 3(4):3702-3707

**Ethics Approval**

The study was approved by the University of Wisconsin institutional animal care and use committee protocol 005670.

**Fig. 1 (abstract P511).**
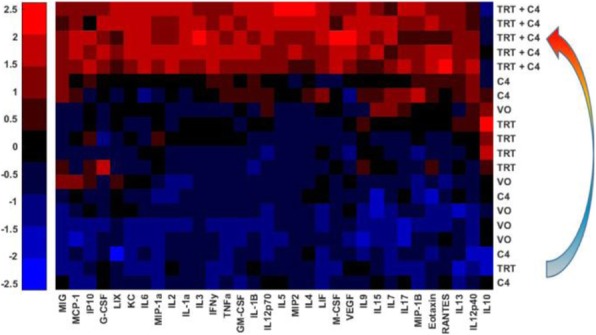
TRT + ICI turns an immunological “cold” tumor “hot”

#### P512 Efficacy of combined radio- and immuno-therapies vs. radiotherapy alone in the treatment of brain metastases: a systematic review and meta-analysis

##### Kirill Peskov^1^, Veronika Voronova^1^, Svetlana Lebedeva^2^, Marina Sekacheva^2^, Gabriel Helmlinger, PhD^3^

###### ^*1*^*M&S Decisions LLC, Moscow, Russia* ; ^*2*^*Sechenov First Moscow State Medical Univ, Moscow, Russian Federation* ; ^*3*^*AstraZeneca, Waltham, MA, United States*

####### **Correspondence:** Gabriel Helmlinger (gabriel.helmlinger@astrazeneca.com)

**Background**

Combination of radiation with immuno-therapy had shown promising results in preclinical experiments as well as in clinical trials. Information on clinical efficacy of immune checkpoint inhibitors (ICI) combined with radiotherapy (RT) is available from numerous retrospective analyses, mainly focused on the treatment of brain metastases. The interpretation of retrospective clinical meta data, however, may be complicated due to variability in the design of studies and confounding factors. The aim of the present research was to systematically review and perform a meta-analysis comparing efficacy profiles of mono-RT vs ICI-RT combination in the treatment of brain metastases. The analysis also aimed at identifying factors affecting treatment outcomes.

**Methods**

The meta-analysis was performed using the Preferred Reporting Items for Systematic Review and Meta-Analyses (PRISMA) guideline. Studies with at least one ICI-RT arm and including patients with brain metastases were curated via a systematic literature search. Information on 1-year overall survival (OS) and 1-year local control (LC) was extracted and analyzed; additionally, if studies included mono-RT arms, risk ratios (RR) for the aforementioned endpoints were calculated and analyzed given mono-RT as an active control. Random-effects meta-regression models were tested to evaluate the impact of different factors, such as combination treatment sequencing or type of ICI and RT on outcomes.

**Results**

In total, information on OS and LC was reported in, respectively, 26 and 6 studies, with 50 and 13 arms, featuring 2528 and 525 patients. The corresponding information for mono-RT arms was available from, respectively, 12 and 4 studies with 2021 and 463 patients enrolled. Higher 1-year was observed in ICI-RT treatments vs mono-RT, with corresponding incidence rates were 59% [95% CI: 54-64%] vs 32% [95% CI: 25-40%] (P

Concurrent treatment was associated with higher 1-year OS comparing to sequential regimen with incidence rates 69% [95% CI: 60-78%] vs 52% [95% CI: 45-58%] (P=0.039); efficacy of ICI-RT treatment was not affected by ICI and RT type.

**Conclusions**

RT and ICI combinations were associated with improved 1-year OS vs mono-RT. The highest benefit in outcomes was determined for cohorts with concurrent ICI-RT treatment regimen.

#### P513 Radiation therapy as a tool to optimize immunomodulation by cell cycle inhibitors in HR+ breast cancer

##### Giulia Petroni, Aitziber Buqué Martinez, Maurizio Di Liberto, Takahiro Yamazaki, PhD, Norma Bloy, Ai Sato, Selina Chen-Kiang, Silvia Formenti, MD, Lorenzo Galluzzi

###### Weill Cornell Medical College, New York, NY,, United States

####### **Correspondence:** Lorenzo Galluzzi (log3001@med.cornell.edu)

**Background**

Hormone receptor+ (HR+) breast cancer (BC) is the most frequent cause of BC death [1], and immunotherapy with immune checkpoint blockers (ICBs) in HR+ BC patients has been disappointing [2]. Conversely, recent results from MONALEESA 7 trial demonstrate a robust survival advantage for the combination of cyclin dependent kinase 4 (CDK4)/CDK6 inhibitors and hormonotherapy over hormonotherapy alone [3]. However, not all patients benefit from hormonotherapy + CDK4/CDK6 inhibition [4]. This implies that additional strategies are needed to improve disease outcome in this patient population. In this context, focal radiation therapy (RT) stands out as a promising therapeutic partner for several reasons, including (1) both RT and CDK4/CDK6 inhibitors have cytostatic/cytotoxic effects; and (2) both RT and CDK4/CDK6 inhibitors can have immunostimulatory effects [5,6]. We therefore decided to investigate the potential synergy between RT, and CDK4/CDK6 inhibitors, with a particular focus on administration schedule, ultimately aiming to inform the design of a clinical trial testing the combination of these therapeutic agents in patients with HR+HER2- BC.

**Methods**

The synergy between RT and palbociclib (a highly selective CDK4/CDK6 inhibitor approved for the treatment of BC in humans) was tested: (1) in vitro, in 2 human BC cell lines with differential sensitivity to palbociclib and RT (MCF7 and MBA-MD-231 cells), to evaluate the effects on cell cycle distribution, cell death, cell senescence and IFN secretion; (2) in vivo, in a unique endogenous model of mammary carcinogenesis that recapitulate multiple immunobiological features of human HR+HER2- BC.

**Results**

We identified the lowest doses of RT and palbociclib that mediate robust short-term cytostatic/cytotoxic effects in MDA-MB-231 and MCF7 cells. Optimized dose combinations and sequencing experiments pointed to RT followed by palbociclib as the approach with superior therapeutic potential. This largely reflect the ability of palbociclib to block in G1 cells escaping the G2/M arrest mediated by RT. Both RT and palbociclib were active in endogenous carcinomas driven in mice by slow-release progesterone pellets combined with an oral carcinogen, but failed to mediate disease eradication. Ongoing experiments are elucidating the impact of combination and sequencing in vivo.

**Conclusions**

Our preliminary results suggest that RT should precede CDK4/6 inhibition to achieve superior disease control. Altogether, these experiments will determine an optimal approach to combine CDK4/CDK6 inhibitors with RT in the context of endocrine therapy that has an elevated potential for clinical translation.

**Acknowledgements**

GP is supported by Fondazione Umberto Veronesi. ABM supported by a Breakthrough Level 2 grant from the US Department of Defense (DoD), Breast Cancer Research Program (BRCP) [#BC180476P1. LG is supported by a Breakthrough Level 2 grant from the US Department of Defense (DoD), Breast Cancer Research Program (BRCP) [#BC180476P1], by a startup grant from the Dept. of Radiation Oncology at Weill Cornell Medicine (New York, US), by industrial collaborations with Lytix (Oslo, Norway) and Phosplatin (New York, US), and by donations from Phosplatin (New York, US), the Luke Heller TECPR2 Foundation (Boston, US) and Sotio a.s. (Prague, Czech Republic).

**References**

1. Pan H, Gray R, Braybrooke J, Davies C, Taylor C, McGale P, Peto R, Pritchard KI, Bergh J, Dowsett M, Hayes DF; EBCTCG, 20-Year Risks of Breast-Cancer Recurrence after Stopping Endocrine Therapy at 5 Years. N Engl J Med. 2017; 377(19):1836-1846.

2. Rugo HS, Cortes J, Awada A, O'Shaughnessy J, Twelves C, Im SA, Hannah A, Lu L, Sy S, Caygill K, Zajchowski DA, Davis DW, Tagliaferri M, Hoch U, Perez EA, Change in Topoisomerase 1-Positive Circulating Tumor Cells Affects Overall Survival in Patients with Advanced Breast Cancer after Treatment with Etirinotecan Pegol. Clin Cancer Res. 2018;24(14):3348-3357.

3. Im SA, Lu YS, Bardia A, Harbeck N, Colleoni M, Franke F, Chow L, Sohn J, Lee KS, Campos-Gomez S, Villanueva-Vazquez R, Jung KH, Chakravartty A, Hughes G, Gounaris I, Rodriguez-Lorenc K, Taran T, Hurvitz S, Tripathy D, Overall Survival with Ribociclib plus Endocrine Therapy in Breast Cancer. N Engl J Med. 2019;381(4):307-316.

4. Pandey K, An HJ, Kim SK, Lee SA, Kim S, Lim SM, Kim GM, Sohn J, Moon YW, Molecular mechanisms of resistance to CDK4/6 inhibitors in breast cancer: A review. Int J Cancer. 2019;145(5):1179-1188.

5. Ameratunga M, Kipps E, Okines AFC, Lopez JS, To Cycle or Fight-CDK4/6 Inhibitors at the Crossroads of Anticancer Immunity. Clin Cancer Res. 2019;25(1):21-28.

6. Sahu AD, S Lee J, Wang Z, Zhang G, Iglesias-Bartolome R, Tian T, Wei Z, Miao B, Nair NU, Ponomarova O, Friedman AA, Amzallag A, Moll T, Kasumova G, Greninger P, Egan RK, Damon LJ, Frederick DT, Jerby-Arnon L, Wagner A, Cheng K, Park SG, Robinson W, Gardner K, Boland G, Hannenhalli S, Herlyn M, Benes C, Flaherty K, Luo J, Gutkind JS, Ruppin E, Genome-wide prediction of synthetic rescue mediators of resistance to targeted and immunotherapy. Mol Syst Biol. 2019;15(3):e8323.

**Ethics Approval**

The animal study was approved by Weill Cornell Medicine's IACUC; approval number 2019-0022.

#### P514 Molecularly targeted radionuclide therapy modulates the composition of the murine prostate cancer microenvironment

##### Hemanth Potluri, BA, Reinier Hernandez, PhD, Christopher Zahm, PhD, Joseph Grudzinski, PhD, Christopher Massey, Jamey Weichert, PhD, Douglas McNeel, MD, PhD

###### UW-Madison, Madison, WI, United States

####### **Correspondence:** Douglas McNeel (dm3@medicine.wisc.edu)

**Background**

Prostate cancer responds poorly to checkpoint blockade due to its immunologically “cold” microenvironment with low CD8+ T cell infiltration along with relatively high numbers of myeloid-derived suppressor cells (MDSCs) and regulatory CD4+ T cells (Tregs) [1–3]. Our lab has studied DNA vaccines as a simple method of increasing antigen-specific CD8+ T cell infiltration [4,5]. To further increase CD8+ T cell infiltration and deplete suppressive populations, external beam radiation (EBRT) can be used [6]. However, it is often infeasible to irradiate all metastases using EBRT. Molecularly targeted radionuclide therapy (MTRT) is a method of delivering radioisotopes radiation selectively to all sites of disease [7]. The effects of MTRT’s effects on the tumor immune microenvironment are not yet well described. In this study, we examined the effect of 90Y-NM600 MTRT on immune populations within murine prostate tumors and assessed whether MTRT combined with antigen-specific vaccination can improve anti-tumor response.

**Methods**

6-week old male FVB mice (n=3 per group) were implanted with subcutaneous (s.c.) MyC-CaP murine prostate tumors then given a single intravenous injection of either 50 (3.1 Gy) or 250 (15.5 Gy) μCi of 90Y-NM600. Mice were euthanized at day 0, 1, 4, 7, 14, and 21 post MTRT administration. Tumors were harvested and examined by multiparametric flow cytometry and immunohistochemistry for effects on immune populations. In a second study, mice (n=5 per group) were implanted with s.c. MyC-CaP tumors then given five weekly treatments of either DNA vaccine or vehicle as well as a single administration of 125 μCi (7.8 Gy) of 90Y-NM600. These mice were followed for tumor growth.

**Results**

CD8+ T cell tumor infiltration increased by 13% at Day 14 compared to Day 0 (p=.039) following MTRT administration in both dose groups while GR-1+ cells (including MDSCs) transiently decreased by 31% at Day 4 (p=.007). Recovering CD8+ T cells showed high expression of the checkpoints PD-1, CTLA-4, and LAG-3. CD4+ T cells were depleted following MTRT administration and did not recover by Day 21. MTRT combined with vaccination resulted in decreased tumor growth compared to MTRT alone.

**Conclusions**

Our data indicates that MTRT disrupts the suppressive tumor microenvironment of prostate tumors by depleting suppressive populations and increasing CD8+ T cell infiltration. High expression of exhaustion markers on CD8+ T cells suggests that combination of MTRT and vaccination with checkpoint blockade may further improve anti-tumor response.

**Acknowledgements**

UW-Madison Medical Scientist Training Program: GM008692

UW-Madison Institute for Clinical and Translational Research Predoctoral TL1 Program: TR002375

University of Wisconsin Carbone Cancer Center Support Grant P30 CA014520

Archeus Technologies for kindly providing the NM600

**References**

1. Danaher P, Warren S, Lu R, Samayoa J, Sullivan A, Pekker I, et al. Pan-cancer adaptive immune resistance as defined by the Tumor Inflammation Signature (TIS): results from The Cancer Genome Atlas (TCGA). J Immunother Cancer 2018;6:63. doi:10.1186/s40425-018-0367-1.

2. Parker KH, Beury DW, Ostrand-Rosenberg S. Myeloid-Derived Suppressor Cells: Critical Cells Driving Immune Suppression in the Tumor Microenvironment. Adv Cancer Res 2015;128:95–139. doi:10.1016/bs.acr.2015.04.002.

3. Flammiger A, Weisbach L, Huland H, Tennstedt P, Simon R, Minner S, et al. High tissue density of FOXP3+ T cells is associated with clinical outcome in prostate cancer. Eur J Cancer Oxf Engl 1990 2013;49:1273–9. doi:10.1016/j.ejca.2012.11.035.

4. Olson BM, Johnson LE, McNeel DG. The androgen receptor: a biologically relevant vaccine target for the treatment of prostate cancer. Cancer Immunol Immunother CII 2013;62:585–96. doi:10.1007/s00262-012-1363-9.

5. Olson BM, Gamat M, Seliski J, Sawicki T, Jeffery J, Ellis L, et al. Prostate Cancer Cells Express More Androgen Receptor (AR) Following Androgen Deprivation, Improving Recognition by AR-Specific T Cells. Cancer Immunol Res 2017;5:1074–85. doi:10.1158/2326-6066.CIR-16-0390.

6. Demaria S, Bhardwaj N, McBride WH, Formenti SC. Combining radiotherapy and immunotherapy: a revived partnership. Int J Radiat Oncol Biol Phys 2005;63:655–66. doi:10.1016/j.ijrobp.2005.06.032.

7. Grudzinski JJ, Hernandez R, Marsh I, Patel R, Aluicio-Sarduy E, Engle J, et al. Preclinical Characterization of 86/90Y-NM600 in a variety of murine and human cancer tumor models. J Nucl Med 2019:jnumed.118.224808. doi:10.2967/jnumed.118.224808.

#### P515 Defining the TCR repertoire and functional differentiation of tumor-infiltrating T cells in mice responding to radiotherapy and CTLA-4 blockade

##### Nils-Petter Rudqvist, PhD^1^, Claire Lhullier, PhD^1^, Erik Wennerberg, PhD^1^, Jennifer Sims, PhD^2^, Sandra Demaria, MD^1^

###### ^*1*^*Weill Cornell Medicine, New York, NY, United States* ; ^*2*^*Memorial Sloan Kettering Cancer Center, New York, NY, United States*

####### **Correspondence:** Sandra Demaria (szd3005@med.cornell.edu)

**Background**

Focal radiotherapy (RT) in combination with CTLA-4 blockade activates T-cells that reject tumors in both mice and patients unresponsive to CTLA-4 blockade alone [1, 2]. In metastatic non-small cell lung cancer patients dynamic changes in the blood TCR repertoire were strongly associated with response [1]. In the 4T1 mouse model of triple negative breast cancer we found higher intratumoral CD8+/CD4+ ratio and CD8+ T cell clonality in tumors of mice treated with RT+CTLA-4 blockade compared to each treatment alone [3]. However, the functional state of expanded intratumoral T cells remained unknown. The purpose of this study was to identify the intratumoral T cell landscape associated with response to RT+CTLA-4 blockade [4].

**Methods**

For single cell sequencing (SC-seq) and bulk TCR sequencing (TCR-seq) experiments, mice were inoculated in one or both flanks with 50,000 4T1 cells, respectively. To obtain pre-treatment samples in each individual mouse, when applicable, one tumor was removed 2 days before treatment with RT (3x8 Gy) and/or anti-CTLA-4 antibody (9H10 Ab, 3x200 μg i.p.), and the treated tumor was resected 1 day after last antibody administration. TCR-seq was performed using the iRepertoire platform, and 5’ gene expression, VDJ, and feature barcoding SC-seq was performed on sorted CD3+CD4+ and CD3+CD8+ T cells using the 10X Genomics chromium system.

**Results**

In tumors treated with RT alone or in combination with anti-CTLA-4, both the TCRα and TCRβ repertoires increased in clonality, whereas a smaller increase in TCRβ clonality was found after anti-CTLA-4 monotherapy. Increased divergence between pre- and post-treatment samples was found after RT alone or in combination with anti-CTLA-4 but only correlated with treatment effect in mice treated with the combination (Pearson correlation coefficients: TCRα = 0.85, TCRβ = 0.93). Compared to untreated tumors, tumors treated with RT+anti-CTLA-4 were enriched with an oligoclonal population of Ifng+Cd69+Tnf+Cd8+ and Ifng+Cd4+ T cells, and had decreased proportions of both Foxp3+Ox40+Cd25+Cd4+ (Tregs) and Pd1+Gzmb+Cd8+ (likely terminally differentiated/exhausted) T cells. In contrast, tumors treated with RT alone were enriched with T cells exhibiting the terminally differentiated/exhausted phenotype, which may explain why the TCR repertoire divergence between baseline and post-treatment tumors correlated with treatment effect only for mice treated with RT+anti-CTLA-4.

**Conclusions**

Overall, these results suggest that RT promotes the differentiation of the intratumoral TCR repertoire but in the absence of CTLA-4 blockade it does not drive the expansion and functional differentiation of T cell phenotypes necessary for immune-mediated tumor rejection.

**Acknowledgements**

Grant support: R01CA198533

**References**

1. Formenti SC, Rudqvist NP, Golden E, Cooper B, Wennerberg E, Lhuillier C, et al. Radiotherapy induces responses of lung cancer to CTLA-4 blockade. Nat Med. 2018;24(12):1845-51.

2. Vanpouille-Box C, Demaria S, Formenti SC, Galluzzi L. Cytosolic DNA Sensing in Organismal Tumor Control. Cancer Cell. 2018;34(3):361-78.

3. Rudqvist NP, Pilones KA, Lhuillier C, Wennerberg E, Sidhom JW, Emerson RO, et al. Radiotherapy and CTLA-4. Blockade Shape the TCR Repertoire of Tumor-Infiltrating T Cells. Cancer Immunol Res. 2018;6(2):139-50.

#### P516 Radiation-driven changes in immunomodulatory DNA cargo of exosomes produced by cancer cells

##### Sheila Spada, PhD, Sandra Demaria, MD , Nils-Petter Rudqvist, PhD, Tuo Zhang, PhD

###### Weill Cornell Medicine, New York, NY, United States

####### **Correspondence:** Sandra Demaria (szd3005@med.cornell.edu)

**Background**

Radiation therapy (RT) used at immunogenic doses (8GyX3) leads to cytosolic DNA accumulation which activates interferon type I (IFN-I) production via cGAS/STING pathway in cancer cells [1-3]. RT-induced IFN-I is critical for activation of systemically effective anti-tumor immune responses in combination with immune-checkpoint blockade therapy [1]. We have recently demonstrated that tumor-derived exosomes (TEX) produced by irradiated (8GyX3) TSA murine carcinoma cells (RT-TEX) contain more dsDNA compared to TEX derived from untreated cancer cells (UT-TEX). Interestingly, the dsDNA transferred by RT-TEX but not UT-TEX induces upregulation of costimulatory molecules CD40, CD80 and CD86, and STING-dependent IFN-I pathway activation in recipient dendritic cells. Furthermore, in vivo, RT-TEX elicit tumor-specific CD8+T-cell responses and protect mice from tumor development [4]. Here, we tested the hypothesis that not only quantitative but also qualitative differences may exist between the dsDNA cargo of RT-TEX and UT-TEX.

**Methods**

TEX were isolated 48 hours after last dose of radiation or mock treatment using ultracentrifugation. Internal DNA from TEX and from the parent TSA cells cytosolic fraction was analyzed by (1) semi-quantitative PCR using primers for mitochondrial 12S and 16S ribosomal subunits, NADH dehydrogenase subunit 1 (ND1) and genomic 18S ribosomal subunit genes; (2) DNA length was determined using Agilent Bioanalyzer; (3) DNA sequencing was performed using the Illumina HiSeq4000 system (paired end 50).

**Results**

Genomic and mitochondrial DNA were identified in both TEX and TSA cytosolic fraction regardless of RT. DNA length analysis demonstrated an enrichment of DNA fragments between 60 and 250 bp in RT-TEX compared to UT-TEX, and in the cytosolic fraction of irradiated TSA cells. These results are especially intriguing in light of the recent report that cGAS is optimally activated by dsDNA in this length range [5]. Whole-genome sequencing is ongoing to determine the genomic versus mitochondrial origin of the differentially represented DNA fragments.

**Conclusions**

Our data suggest that the immunostimulatory DNA cargo of RT-TEX has unique features that reflect molecular changes occurring in radiation-treated cells. The impact of these differences on the DNA-mediated activation of IFN-I pathway in innate immune cells that uptake TEX is under investigation. Identification of a “DNA signature” associated with TEX ability to activate cGAS could provide a blood-based biomarker for the immunogenic tumor response to radiotherapy.

**Acknowledgements**

Supported by American-Italian Cancer Foundation Fellowship, The Chemotherapy Foundation, R01CA201246 and Cornell University Seed Grants for Intercampus Collaborative Projects

**References**

1.Vanpouille-Box C, Alard A, Aryankalayil MJ, Sarfraz Y, Diamond JM, Schneider RJ, Inghirami G, Coleman CN, Formenti SC, Demaria S. DNA exonuclease Trex1 regulates radiotherapy-induced tumour immunogenicity. Nat Commun. 2017 Jun 9;8:15618

2.Mackenzie KJ, Carroll P, Martin CA, Murina O, Fluteau A, Simpson DJ, Olova N, Sutcliffe H, Rainger JK, Leitch A, Osborn RT, Wheeler AP, Nowotny M, Gilbert N, Chandra T, Reijns MAM, Jackson AP. cGAS surveillance of micronuclei links genome instability to innate immunity. Nature. 2017 Aug 24;548(7668):461-465

3.Harding SM, Benci JL, Irianto J, Discher DE, Minn AJ, Greenberg RA. Mitotic progression following DNA damage enables pattern recognition within micronuclei. Nature. 2017 Aug 24;548(7668):466-470

4.Diamond JM, Vanpouille-Box C, Spada S, Rudqvist NP, Chapman JR, Ueberheide BM, Pilones KA, Sarfraz Y, Formenti SC, Demaria S. Exosomes Shuttle TREX1-Sensitive IFN-Stimulatory dsDNA from Irradiated Cancer Cells to DCs. Cancer Immunol Res. 2018 Aug;6(8):910-920

5.Du M, Chen ZJ. DNA-induced liquid phase condensation of cGAS activates innate immune signaling. Science. 2018 Aug 17;361(6403):704-709. doi:10.1126/science.aat1022

#### P517 Potent anti-tumor effect of systemically administered nanoparticles containing a TLR7 agonist in combination with radiotherapy

##### Camilla Stavnsbjerg, MSc^1^, Esben Christensen, MSc^1^, Rasmus Münter, MSc^1^, Andreas Kjær, MD, PhD^2^, Svetlana Panina, MD, PhD^3^, Martin Bak, PhD^3^, Jonas Henriksen, PhD^1^, Simon Jensen, PhD^3^, Anders Hansen, DVM, PhD^1^, Thomas Andresen, PhD^1^

###### ^*1*^*Technical University of Denmark, Kgs. Lyngby, Denmark* ; ^*2*^*Rigshospitalet & University of Copenhagen, Copenhagen, Denmark*; ^*3*^*MonTa Biosciences Aps, Kgs. Lyngby, Denmark*

####### **Correspondence:** Thomas Andresen (tlan@dtu.dk)

**Background**

TLR7 agonists are potent immune activators that are highly attractive for anti-cancer immunotherapy with potential to act synergistically with other cancer treatments such as vaccines, radiotherapy, and chemotherapy. The limited tolerability of systemically administered TLR7 agonists makes administration problematic at therapeutically active concentrations. Drug delivery systems may increase tolerability by optimizing biodistribution and cellular uptake thereby allowing for administration of doses that are therapeutic at the target site.

The current study investigated lipid nanoparticles containing a TLR7 agonist (1V270) in combination with local radiotherapy (to induce antigen release) for an in situ vaccination effect in murine cancer models.

**Methods**

Biophysical properties of the nanoparticles were characterized and anti-tumor efficacy was investigated in three murine syngeneic subcutaneous cancer models (CT26, MC38, and B16-F10). Treatments were initiated when tumors reached a mean size of ~100 mm3. Nanoparticles were given intravenously as multiple doses in combination with fractionated radiotherapy of the tumor region. The optimal formulation and dosing schedule were based on in vivo efficacy studies. Tolerability of the treatment was evaluated based on body weight, hematology, and blood biochemistry as well as cytokine levels in plasma measured 2h and 6h post injection. Antibody responses against the nanoparticles were also investigated. The effect on the tumor microenvironment was evaluated by flow cytometry.

**Results**

Nanoparticles containing 1V270 displayed a synergistic effect with local radiotherapy and all (8/8) mice carrying CT26 tumors displayed complete tumor rejection. In the MC38 model, tumor growth delay was observed and 3/9 mice demonstrated complete tumor rejection. No anti-tumor effect was observed in the poorly immunogenic B16-F10 model. Immunologic memory against tumor antigens was confirmed by tumor-rechallenge with 80-100% tumor-rejection rate in both CT26 and MC38. Improved tolerability, compared to other lipid nanoparticles, was demonstrated. The tumor microenvironment analysis demonstrated that the TLR7 agonist formulation induced infiltration of a large number of innate immune cells and supported the formation of activated tumor-specific CD8 T cells.

**Conclusions**

The novel immunotherapeutic nanoparticle formulation induced a potent anti-tumor immune response; resulting in very potent efficacy in vivo against immunogenic tumors. Furthermore, we demonstrated improved tolerability in mice, which will be further investigated in larger animals. These results are highly encouraging for advancing potent immune stimulating therapy into the clinic.

**Ethics Approval**

The study was approved by the Danish Animal Experiments Inspectorate.

#### P518 Testing a new platform for precision use of radiation therapy to induce in situ vaccination in breast cancer

##### Samantha Van Nest, PhD, Tasnim Anika, Adriana Irizarry, Laura Martin, Giorgio Inghirami, Silvia Formenti, MD, Sandra Demaria, MD , Samantha Van Nest, PhD

###### Weill Cornell Medicine, New York, NY, United States

####### **Correspondence:** Sandra Demaria (szd3005@med.cornell.edu)

**Background**

Immune checkpoint inhibition targeting PD-1/PD-L1 has shown promise in breast cancer but is largely limited to triple negative breast cancer (TNBC).[1] This limitation is primarily due to inherently low levels of tumour infiltrating lymphocytes (TILs), particularly in HR+ disease.[2] Focal radiation therapy (RT) has been shown to generate anti-tumour T cells and increase TILs using mouse models of breast cancer (BC).[3,4] Mechanistically, radiation-induced increase in cytosolic DNA leads to activation of cGAS/STING pathway and production of IFN-훃, which is essential for priming of anti-tumour CD8+ T cells.[5] This process is under the control of TREX1, and dependent on RT dose and fractionation.[5] Inter-tumor variability in optimal RT dose for activating the IFN-I pathway and heterogeneity in immunological response amongst BC subtypes highlight the importance of precision use of RT.[6] We are testing the hypothesis that improved in vitro and in vivo assays allow testing of the pro-immunogenic response to radiation in individual BC patients.

**Methods**

Spheroids were established from human adenocarcinoma cell lines MCF-7 (ER+,PR+,HER2-) and MDA-MB-231 (TNBC) by seeding 1,000 cells in a mixture of 2% Matrigel in DMEM. Patient-derived tumour xenograft (PDTX) was dissociated and organoids formed using 66% Matrigel in media. Single radiation doses between 2Gy and 20Gy as well as fractionated doses of 8Gyx3 were delivered, samples harvested at 24 hours post-treatment and RNA extracted. RT-qPCR was used to interrogate expression of key IFN-I response genes (IFN**β**1, MX1, CXCL10, IFNAR1) as well as TREX1.

**Results**

Spheroids and organoids were successfully established using human cell lines and PDTX, respectively (Figure 1). IFN-I pathway activation by single radiation doses ranging from 6 to 10Gy was detected in MCF-7, MDA-MB-231 and in PDTX, whereas single doses of 15 to 20Gy enhanced the expression of TREX1 and dampened IFN-I activation. Similar results were obtained in an in vivo irradiated PDTX and its in vitro-irradiated organoids. We are currently establishing and testing additional pairs of organoids and PDTX from the same BC tumor to determine the most time and cost-efficient platform.

**Conclusions**

Developing a platform that utilizes pre-treatment tumor tissue from a patient to determine the functional response to different RT doses and aid in the selection of the best combination treatment with anti-PD-1/PD-L1 is a critical step towards precision use of RT to enhance responses to immunotherapy. Our data suggest that organoids and PDTX could both be used to test the functional response of an individual patient tumor to various RT doses.

**Acknowledgements**

The authors which to acknowledge financial support from the Breast Cancer Research Foundation (award BCRF-18-053).

**References**

1. Emens LA, Breast Cancer Immunotherapy: Facts and Hopes. Clin Cancer Res. 2018; 24(3):511-520.

2. Denkert C, von Minckwitz G, Darb-Esfahani S, Lederer B, Heppner B, Weber KE et al. Tumour-infiltrating lymphocytes and prognosis in different subtypes of breast cancer: a pooled analysis of 3771 patients treated with neoadjuvant therapy. Lancet Oncol. 2018;19(1):40-50.

3. Demaria S, Kawashima N, Yang AM, Devitt ML, Babb JS, Allison JP et al. Immune-Mediated Inhibition of Metastases after Treatment with Local Radiation and CTLA-4 Blockade in a Mouse Model of Breast Cancer. Clin Cancer Res. 2005;11(21):728-734.

4. Demaria S, Golden E, Formenti SC. Role of Local Radiation Therapy in Cancer Immunotherapy. JAMA Oncol. 2015;1(9):1325-1332.

5. Vanpouille-Box C, Alard A, Aryankalayil M, Sarfraz Y, Diamond J, Schneider R et al. DNA exonuclease Trex1 regulates radiotherapy-induced tumour immunogenicity. Nat Commun. 2017;8:15618.

6. Vanpouille-Box C, Formenti, SC, Demaria S. Toward Precision Radiotherapy for Use with Immune Checkpoint Blockers. Clin Cancer Res. 2018;24(2):259-265.

**Ethics Approval**

All animal procedures were approved by the Institutional Animal Care and Use Committee (IACUC) at Weill Cornell Medicine.

**Fig. 1 (abstract P518).**
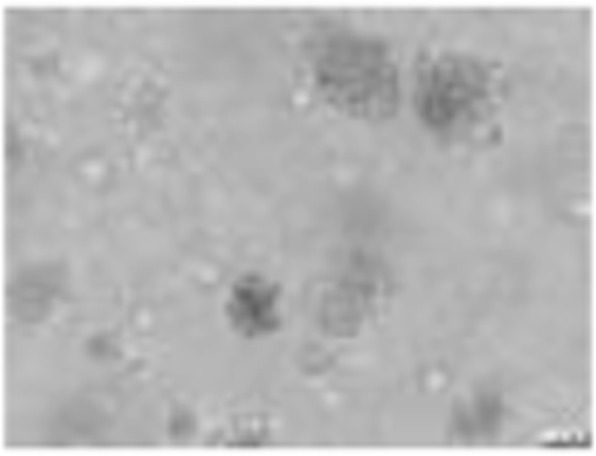
Patient-Derived Breast Cancer Organoids

#### P519 Immunogel - an intratumoral multi-drug sustained release technology that polarizes the tumor microenvironment towards a proinflammatory and immunosupportive state

##### Trine Engel, PhD^1^, Jennifer Jørgensen, PhD^1^, Lars Ringgaard, PhD^1^, Fredrik Melander, PhD^1^, Sophie Jensen, MSc^1^, Linda Bruun, PhD^1^, Camilla Stavnsbjerg, MSc^1^, Andreas Tue Jensen, PhD^1^, Julianna Thuróczy, DVM, PhD, Dipl ECAR^2^, Lajos Balogh, DVM, PhD^2^, Frederikke Fliedner, MSc^3^, Martin Bak, PhD^1^, Andreas Kjær, MD, PhD^3^, Jonas Henriksen, PhD^1^, Anders Hansen, DVM, PhD^1^, Thomas Andresen, PhD^1^

###### ^*1*^*Technical University of Denmark, Kgs. Lyngby, Denmark* ; ^*2*^*Animal Health Center Budafok, Budapest, Hungary* ; ^*3*^*Rigshospitalet & University of Copenhagen, Copenhagen, Denmark*

####### **Correspondence:** Thomas Andresen (tlan@dtu.dk)

**Background**

Polarizing the tumor microenvironment (TME) towards an inflamed and immunosupportive type is highly attractive for anti-cancer therapies. However, critical challenges are recognized for optimally modulating the TME. Firstly, immune activating drugs are poorly tolerated and secondly, the plastic and reactive TME must be continuously manipulated. Moreover, multiple pathways must be stimulated to recruit and activate immune cells. Here we present a well-tolerated multi-drug intratumoral delivery technology (Immunogel), which has the potential to transform the TME and support immunological anti-cancer activity by ensuring a local sustained drug release. The Immunogel can easily be injected with small gauge needle technology, which has been demonstrated in clinical cancer patients using advanced high-precision image-guided technologies.

**Methods**

The Immunogel was formulated to provide sustained release of a TLR7/8 agonist (NT03-gel) and combined sustained release of a TLR7/8 agonist and TGFβ-inhibitor (NT04C-gel). Therapeutic efficacy and immune-modulating properties were evaluated in syngeneic cancer models. Drug release kinetics and tolerability was demonstrated in preclinical rodents and laboratory canines.

**Results**

The NT03-gel demonstrated a sustained intratumoral drug release of 31% after 24 hours and 81% after 7 days. Combination of NT03-gel and immunogenic cell death inducing chemo- or radiotherapy in the CT26 cancer model induced tumor rejection in >60% compared to 0% of treatment controls. TME analysis demonstrated that NT03-gel in combination with chemotherapy reduced intratumoral levels of immunosuppressive myeloid subsets and improved the cytotoxic T cell (cT) to regulatory T cell ratio more than nine-fold compared to controls. Additionally, a high increase of strongly activated CD137+ cTs and intratumoral infiltration of central memory cTs was identified. NT03-gel and radiotherapy increased intratumoral levels of T cell attracting cytokines and chemokines. Encouragingly, NT03-gel significantly enhanced anti-PD-1 therapy, indicating that the immune modulating properties of NT03-gel are attractive for improving immune-checkpoint therapy. The potential of multitarget therapy was demonstrated for NT04C-gel, which in combination with radiotherapy induced 100% tumor rejection. Furthermore, 18FDG PET/CT and lung metastasis scoring demonstrated that NT04C-gel in combination with low-dose radiotherapy of the primary tumor significantly reduced lung metastasis in the metastatic 4T1 cancer model. Importantly, the NT03-gel and NT04C-gel were well tolerated in laboratory beagles during a three-week evaluation period.

**Conclusions**

By enabling intratumoral sustained drug release, the Immunogel has the ability to polarize the TME towards an inflamed immunosupportive state, which is key for improving anti-cancer immunotherapies.

**Ethics Approval**

All experimental procedures involving animals were approved by The Danish Animal Experiments Inspectorate.

#### P520 Matching-adjusted indirect comparison of pembrolizumab plus axitinib versus nivolumab plus ipilimumab for the first-line treatment of advanced/ metastatic renal cell carcinoma

##### Yufei Wang, Yichen Zhong, PhD, Shahrul Mt-Isa, Rodolfo Perini, MD, Oluwakayode Adejoro, MD, MPH

###### Merck, Kenilworth, NJ, United States

####### **Correspondence:** Oluwakayode Adejoro (oluwakayode.adejoro@merck.com)

**Background**

To date, pembrolizumab plus axitinib (P+A) and nivolumab plus ipilimumab (N+I) are the only immunotherapy combination therapies demonstrating superior overall survival (OS) compared to sunitinib in Phase III clinical trials in subjects diagnosed with advanced/metastatic renal cell carcinoma (mRCC). No head-to-head trial has compared the efficacy of P+A versus N+I in mRCC.

**Methods**

An anchored matching-adjusted indirect comparison (MAIC) was conducted using individual patient-level data (IPD) from KEYNOTE-426 and pseudo-IPD (digitized KM curves) in the published data from CHECKMATE-214. To adjust for differences in baseline potential effect modifiers between these trials, data for subjects from the KEYNOTE-426 were re-weighted to match the baseline characteristics reported in CHECKMATE-214. Progression-free survival (PFS) and OS were compared. The analyses were conducted in the intention-to-treat (ITT) populations and repeated in subjects with intermediate and poor risk disease (by International Metastatic Renal Cell Carcinoma Database Consortium [IMDC] criteria).

**Results**

After matching, P+A resulted in a lower hazard of disease progression or death than N+I (HR=0.81; 95% CI 0.63, 1.03; p-value = 0.090), and a lower hazard of death (HR=0.71; 95% CI 0.49, 1.03; p-value = 0.069) than N+I in the ITT populations. In the intermediate and poor risk populations, P+A resulted in a lower hazard of disease progression or death (HR=0.84; 95% CI 0.63, 1.12; p-value = 0.238) and a lower hazard of death (HR=0.75; 95% CI 0.50, 1.11; p-value = 0.148) than N+I.

**Conclusions**

Following the adjustment of cross-trial differences, P+A demonstrated lower hazards in the PFS and OS compared to N+I in both the ITT, and intermediate and poor risk populations. In the absence of direct comparison and sufficiently powered studies, these analyses provide valuable insights on the relative efficacy of P+A versus N+I for patients, physicians, and payers.

#### P521 MEK inhibition enhances oncolytic herpes virus immunotherapy

##### Praveen Bommareddy, MS, PhD^1^, Andrew Zloza, MD, PhD^2^, Samuel Rabkin, PhD^3^, Howard Kaufman, MD, FACS^3^

###### ^*1*^*Rutgers University, Woburn, MA, United States* ; ^*2*^*Rush University, Chicago, IL, United States* ; ^*3*^*Masachusetts General Hospital, Boston, MA, United States*

####### **Correspondence:** Howard Kaufman (Howard.Kaufman@replimune.com)

**Background**

Talimogene laherparepvec (T-VEC) is an oncolytic HSV-1 approved for the treatment of melanoma. We previously showed MEK inhibition enhances T-VEC-mediated immunogenic cell death in human melanoma cell lines in a BRAF mutation-independent manner. In this study we sought to understand how MEK inhibition and T-VEC promote host anti-tumor immunity.

**Methods**

Immunocompetent C57/BL6 mice bearing D4M3A melanoma tumors were treated with an adapted T-VEC encoding murine GM-CSF (mT-VEC;106 PFU) given by intra-tumoral administration biweekly with or without MEK inhibitor trametinib (0.1 mg/kg) by oral gavage for 2 weeks. Tumor growth was measured by calipers and, in some experiments, animals were monitored for survival or collection of tumor specimens for biomarker assessment. In independent experiments, treatment was evaluated in mice following depletion of specific immune cell subsets (CD8+ T cells, CD4+ T cells, macrophages and Batf3+ DCs). Flow cytometry analysis was performed on tumor-infiltrating lymphocytes using various markers of immune activation and analyzed by FloJo software (v 10.4). RNA was collected from tumors and subjected to Nanostring gene expression analysis. Statistical comparisons between groups were determined using Student’s t test and Kaplan-Meier method was used to estimate survival. P

**Results**

In an immunocompetent murine HSV-1-sensitive D4M3A melanoma model we observed a significant decrease in tumor growth and increased survival following the addition of trametinib to T-VEC therapy. Therapeutic activity was associated with recruitment of CD8+ T cells into the tumor microenvironment and was dependent on both CD8+ T cells and Batf3+ DCs, as anti-tumor activity was lost when CD8+ T cells, but not CD4+ T cells or macrophages were depleted, and in treated Batf3-/- mice. Characterization of the CD8+ T cells revealed early recruitment of HSV-specific CD8+ T cells followed by melanoma antigen-specific (gp100 and TRP2) CD8+ T cell responses. Gene expression analysis demonstrated that combination therapy induced an immune-inflamed signature supportive of lymphocyte recruitment and activation within the local tumor microenvironment. Combination therapy enhanced PD-L1 expression providing rationale for addition of PD-1 blockade in vivo. In this murine model, triple combination therapy with trametinib, T-VEC and anti-PD-1 was associated with significant improvement in therapeutic effectiveness and resulted in 80% overall survival. Finally, re-challenge with D4M3A in surviving mice demonstrated complete protection suggesting development of long-term anti-tumor memory.

**Conclusions**

Oncolytic virus immunotherapy is improved by MEK inhibition and appears to sensitize tumors to immune checkpoint blockade. These studies support clinical translation of this combination approach into the clinic.

**Ethics Approval**

The study was approved by Rutgers University IACUC.

#### P522 Preclinical development of M4112, an IDO1/TDO2 dual selective and orally bioavailable small molecule inhibitor, and combination with avelumab, for treatment of solid tumors

##### Jieqing Chen, MD^1^, Chia Lin Chu, PhD^1^, Feng Jiang, PhD^1^, Chunxiao Xu, PhD^1^, Yanping Zhang^1^, Sireesha Yalavarthi^1^, Molly Coop^1^, Hong Wang^1^, Yanyan Wang^1^, Bettina Hanschke^2^, Anindya Siddharta^2^, Sen Zhang^1^, Amit Deshpande^1^, Bartholomew Naughton, PhD^1^, Filippos Porichis^1^, Tai-An Lin, PhD^1^, Joern-Peter Halle^2^, Tilo Senger^2^, Brian Sherer^2^, Jacques Moisan^1^

###### ^*1*^*EMD Serono R&D Institute, Inc, Billerica, MA, United States* ;^*2*^*Merck KGaA, Darmstadt, Germany*

####### **Correspondence:** Jacques Moisan (jacques.moisan@emdserono.com)

**Background**

Indoleamine 2,3-dioxygenase (IDO1) and tryptophan 2,3-dioxygenase (TDO2) are key immunoregulatory enzymes that catabolize the essential amino acid tryptophan (Trp) to kynurenine (Kyn), an immunosuppressive metabolite. Kyn accumulation exerts immunosuppressive effects by preventing effector T-cell differentiation and enhancing regulatory T-cell (Treg) activity, thereby creating and sustaining a hostile tumor microenvironment and providing a mechanism of tumor escape from anti–PD-1/PD-L1 blockade. Preclinical data show IDO1 inhibitors have single-agent antitumor activity. In human cancers, tumor expression of IDO1 and TDO2 is distinct and does not completely overlap. Therefore, dual inhibition of IDO1 and TDO2 could provide a broader antitumor effect in a wider range of tumors. The preclinical rationale and testing of M4112, a highly selective, dual inhibitor of IDO1/TDO2 is presented and shows enhancement of immune function and antitumor activity in various in vitro and in vivo models.

**Methods**

One-way, mixed lymphocyte reaction (MLR) assays were performed on human peripheral blood mononuclear cells and mature dendritic cells treated with different concentrations of M4112 in combination with avelumab, a fully human anti–PD-L1 IgG1 antibody, to measure interferon-gamma (IFN-gamma) levels [1]. Naive mice or CT26-KSA and MC38 colon carcinoma mouse models were used for pharmacokinetic and pharmacodynamic and antitumor activity studies. Measurements of M4112, Kyn, and Trp levels in the plasma, liver, and tumor were performed after treatment with M4112 alone or in combination with avelumab. The CANScript tumor explant platform, which predicts potential antitumor effects using an M score [2], was used on 20 fresh human head and neck squamous cell carcinoma (HNSCC) tumor samples. Supernatants were measured for Kyn, Trp, and cytokine levels after 72 hours of M4112 or avelumab treatment alone and in combination.

**Results**

In combination with avelumab, M4112 treatment increased IFN-gamma production, indicating enhanced T-cell activation. Kyn levels and the Kyn/Trp ratio were also reduced in mouse and human tumor explant models. Treatment with M4112 in combination with avelumab led to reduced tumor volumes in syngeneic tumor models. In addition, M4112 exhibited antitumor effects in 4 (20%) and 7 (35%) of 20 HNSCC tumor explants as a single agent and in combination with avelumab, respectively.

**Conclusions**

The dual-selective IDO1/TDO2 inhibitor, M4112, in combination with avelumab enhanced antitumor activity in mouse and human tumor models and normalized systemic Kyn/Trp levels. Thus, M4112 represents a promising therapeutic agent targeting both the IDO1 and TDO2 immunosuppressive pathways.

**References**

1. Hamilton G, Rath B. Avelumab: combining immune checkpoint inhibition and antibody-dependent cytotoxicity. Expert Opin Biol Ther. 2017;17(4):515-523.

2. Majumder B, et al. Predicting clinical response to anticancer drugs using an ex vivo platform that captures tumour heterogeneity. Nat Commun. 2015;6:6169.

#### P523 Immunotherapy plus gene therapy: A tumor-targeting nanomedicine carrying the TP53 gene crosses the blood–brain barrier and enhances anti-PD-1 immunotherapy in mouse models of glioblastoma

##### Joe Harford, PhD^1^, Sang-Soo Kim, PhD^2^, Manish Moghe^2^, Caroline Doherty^1^, Esther Chang, PhD^2^

###### ^*1*^*SynerGene Therapeutics, Inc., Potomac, MD, United States* ; ^*2*^*Georgetown Univ Med Ctr, Washington, DC, United States*

####### **Correspondence:** Esther Chang (change@georgetown.edu)

**Background**

Glioblastomas are among the most lethal of cancers [1], and antibodies against the programmed cell death protein 1 (anti-PD-1) represent a promising immunotherapeutic strategy. However, significant numbers of glioblastoma patients fail to respond to anti-PD-1 [2,3]. Recent studies suggest that p53 participates in immune regulation [4,5], and we have shown that SGT-53, a novel nanomedicine for TP53 gene therapy, can enhance both innate and adaptive immune responses against a variety of tumor types [6,7]. SGT-53 is a liposome encapsulating the normal TP53 gene that targets tumors via an antibody fragment recognizing the transferrin receptor. SGT-53 has completed Phase I & Ib trials [8.9] is now in Phase II clinical trials. Not only does systemically administered SGT-53 target cancer cells with specificity, but it can also actively ferry its payload across the endothelial cells that constitute the blood-brain barrier (BBB) [10]. We therefore hypothesized that adding SGT-53 to anti-PD-1 would be more effective against glioblastomas than checkpoint blockade monotherapy .

**Methods**

We evaluated whether SGT-53 could augment anti-PD-1 therapy for murine glioblastomas (GL261) grown subcutaneously or intracranially in syngeneic mice. We examined a number of biological markers of immune responses and monitored expression of immune-relevant genes after mice bearing glioblastomas were treated with SGT-53 or anti-PD-1 or the combination of these therapeutic agents.

**Results**

Adding SGT-53 to anti-PD-1 immunotherapy resulted in increased intratumoral infiltration of immune effector cells and altered expression of a number of genes related to immune responses. SGT-53 triggered changes in both innate and adaptive immune responses and caused immunogenic changes that included elevated tumor expression of CRT, FAS, PD-L1, ICAM1 and MHC I. SGT-53 also markedly modified the previously immunosuppressive microenvironment of tumors. Importantly, the combination treatment restored T-cell effector function and increased CTLs within tumor (Figure 1), which was correlated with inhibition of subcutaneous and intracranial tumor growth (Figures 2 & 3, respectively) as well as a survival benefit in mice bearing intracranial glioblastomas.

**Conclusions**

Tumor-targeted p53 gene therapy via SGT-53 was able to augment anti-PD-1 immune checkpoint blockade and convert mouse syngeneic glioblastoma tumors that were unresponsive (immunologically “cold” tumors) into tumors that were more responsive to anti-PD-1 immunotherapy. These findings suggest that SGT-53 has potential to enhance the efficacy of checkpoint blockade and thereby provide for improved outcomes for patients with glioblastomas. Our observations provide compelling motivation to test the combination of SGT-53 and a checkpoint inhibitor in the context of a clinical trial for glioblastoma.

**Acknowledgements**

This work was supported by the NCI grant (5R01CA132012-02 to E.H.C.); and a research grant from SynerGene Therapeutics, Inc. This study was conducted at the Lombardi Cancer Center Shared Resource facilities that are partially supported by NCI (P30-CA051008). Some of the data to be presented at SITC have recently been published [11].

**References**

1. Wen PY, Kesari S. Malignant gliomas in adults. N Engl J Med 2008;359:492–507.

2. Lim M, Xia Y, Bettegowda C, et al. Current state of immunotherapy for glioblastoma. Nat Rev Clin Oncol 2018;15:422–42.

3. Kim ES, Kim JE, Patel MA, et al. Immune check-point modulators: an emerging antiglioma armamentarium. J Immunol Res 2016;2016:4683607.

4. Menendez D, Shatz M, Resnick MA. Interactions between the tumor suppressor p53 and immune responses. Curr Opin Oncol 2013;25:85–92.

5. Cui Y, Guo G. Immunomodulatory function of the tumor suppressor p53 in host immune response and the tumor microenvironment. Int J Mol Sci 2016;17:E1942.

6. Moore EC, Sun L, Clavijo PE, et al. Nanocomplex-based TP53 gene therapy promotes anti-tumor immunity through TP53- and STING-dependent mechanisms. Oncoimmunology 2018;7: e1404216.

7. Kim SS, Harford JB, Moghe M, et al. Combination with SGT-53 overcomes tumor resistance to a checkpoint inhibitor. Oncoimmunology 2018;7: e1484982.

8. Senzer N, Nemunaitis J, Nemunaitis D, et al. Phase I study of a systemically delivered p53 nanoparticle in advanced solid tumors. Mol Ther 2013;21:1096–103.

9. Pirollo KF, Nemunaitis J, Leung PK, et al. Safety and efficacy in advanced solid tumors of a targeted nanocomplex carrying the p53 gene used in combination with docetaxel: a phase 1b study. Mol Ther 2016;24:1697–706.

10. Kim SS, Rait A, Kim E, et al. A nanoparticle carrying the p53 gene targets tumors including cancer stem cells, sensitizes glioblastoma to chemotherapy and improves survival. ACS Nano 2014;8:5494–514.

11. Kim SS, Harford JB, Moghe M, Slaughter T, Doherty C, Chang EH. A tumor-targeting nanomedicine carrying the p53 gene crosses the blood–brain barrier and enhances anti-PD-1 immunotherapy in mouse models of glioblastoma. Intl J Cancer (2019) Epub 2019/06/27.

**Ethics Approval**

All animal studies was performed under an IACUC-approved protocol in compliance with the Animal Welfare Act, PHS Policy, and other Federal statutes and regulations relating to animals in the AAALAC-accredited animal facility at Georgetown University, which adheres to principles stated in the Guide for the Care and Use of Laboratory Animals, National Research Council .

**Fig. 1 (abstract P523).**
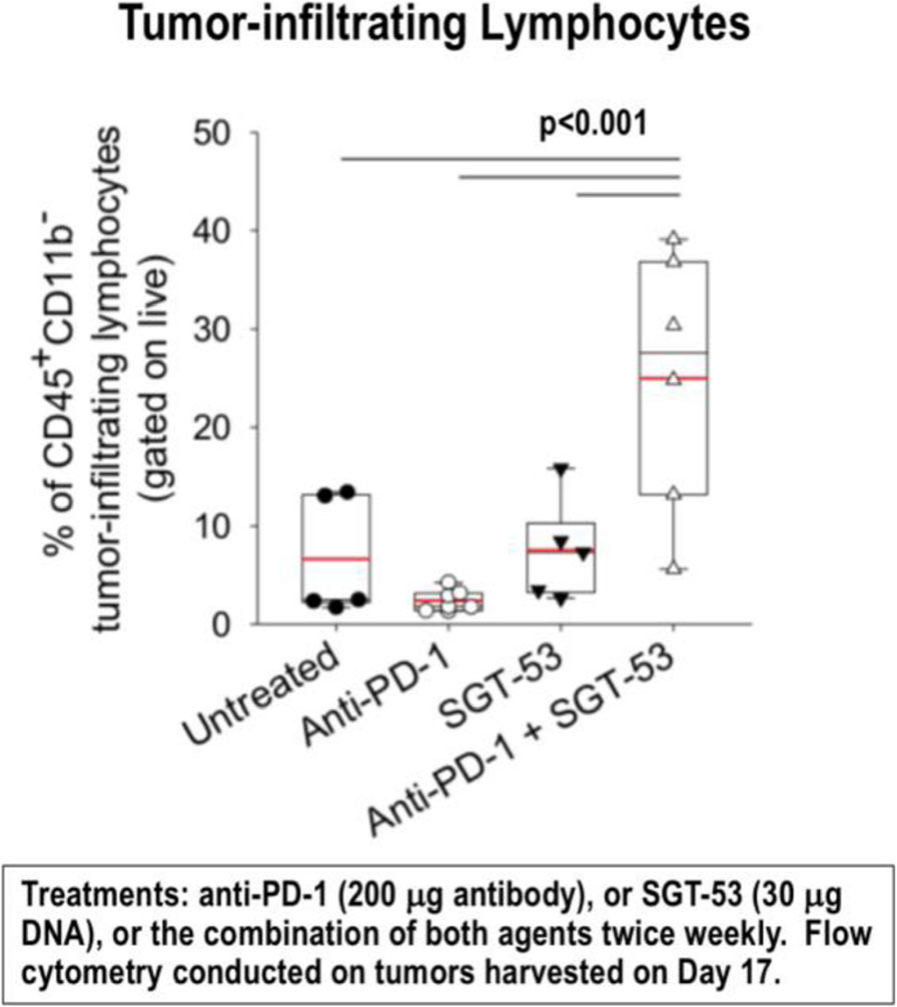
See text for description

**Fig. 2 (abstract P523).**
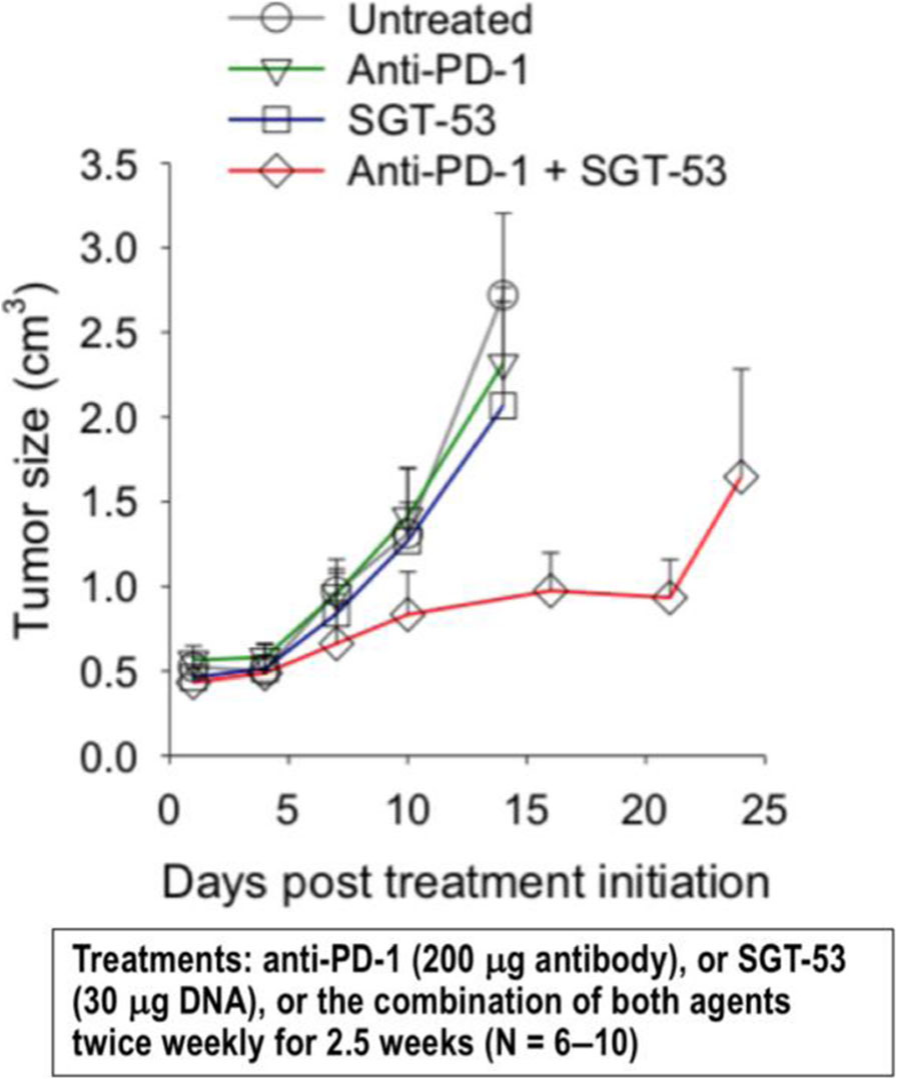
See text for description

**Fig. 3 (abstract P523).**
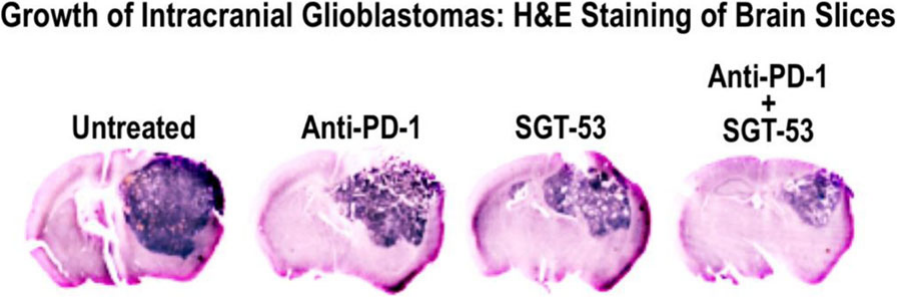
See text for description

#### P524 CRISPR CAS9 mediated BRCA1 knockout modulates the tumor infiltrating lymphocytes landscape and cytokine profile in a murine breast cancer model

##### Julia Schuler^1^ , Anya Avrutskaya^1^, Kanstantsin Lashuk^1^, Mariette Heins, PharmD^2^, Cordula Tschuch^1^, Astrid Jensen^1^, Gerhard Kelter^1^, Anne-Lise Peille^1^, Maycee Robinson^1^, William Durham^1^, Armin Mayer^1^, Anne-Marie Zuurmond^1^

###### ^*1*^*Charles River Discovery, Freiburg, Baden-Wurttembe, Germany* ; ^*2*^*Charles River Den Bosch, Discovery, Groningen, Netherlands*

####### **Correspondence:** Julia Schuler (julia.schueler@crl.com)

**Background**

Around 10% of breast cancer cases are attributed to genetic disorders like mutations in BRCA-1/2 genes. Targeted therapy of BRCA-deficient cancers has been achieved using poly(ADP-ribose) polymerase (PARP) inhibitors, which block BRCA-independent DNA repair.

**Methods**

To study the effect of this common mutation on tumor biology in general and specifically the immunological landscape of the tumor microenvironment, we knocked out the BRCA1 gene in the murine EMT6 breast cancer cell line. Subsequently, we analyzed the tumor infiltrating lymphocyte (TIL) population and the cytokine profiles in serum of tumor bearing animals.

**Results**

The percentage of TILs (=CD45+ cells) was similar in both lines (33.0% vs 29.6%, BRCA1-/- vs BRCA1wt ). Nevertheless, a more detailed analysis of the TIL subpopulations revealed higher percentages for gMDSC and M2 macrophages in the EMT6/BRCA1-/- line. The analysis of 23 cytokines showed a significant down regulation of 22 cytokines in the EMT6/BRCA1-/- bearing animals. Solely, G-CSF was significantly upregulated in the mutated cell line (multiple t-test, p< 0.001). With the objective to elucidate more in detail the biology behind the differences in cytokine levels and TIL distribution, we investigated the influence of different PARP and checkpoint (CP) inhibitors on both parameters. Both lines showed distinct TIL and cytokine profiles under treatment. In the EMT6/BRCA1-/- the TIL population decreased under PARP inhibitor monotherapy (Talazoparib) but increased significantly under treatment with CP inhibitors anti CTLA-4 and anti PD-1 in monotherapy as well as in combination with Talazoparib (Kruskall-Wallis test, p< 0.005). In contrast, the TIL frequency in the EMT6/BRCA1wt model was similar in all treatment arms. Differences in the TIL composition were mainly detected in the NK and gMDSC cell fraction which were upregulated under treatment including Talazoparib specifically in the EMT6/BRCA1-/- model. The TIL analysis in the EMT6/BRCA1wt tumors showed no differences in the individual subpopulations although the observed antitumoral activity of anti CTLA-4 as well as anti PD-1 in this model (tumor reduction of >60% as compared to untreated control). In line with the enhanced NK cell numbers under therapy was the fact that serum levels of RANTES and IL-2 were enhanced under treatment in EMT6/BRCA1-/- bearing animals.

**Conclusions**

Further studies using microdialysis as read-out will give additional information on the influence of the BRCA1 gene knockout and the resulting modulation of the tumor microenvironment and will help to elucidate the tumor biology and clinical relevance behind these findings.

**Ethics Approval**

The study was approved by the local authorities (Regierungspräsidium Freiburg; Germany) approval number G17-78

#### P525 Dose-dependent sensitization of combined anti-angiogenic and PD-1 blockade in breast cancer

##### Wen Jiang, MD, PhD^1^, Yifan Wang, PhD^1^, Betty Kim^2^, Jieqiong Liu, MD, PhD^3^

###### ^*1*^*The University of Texas Southwestern Medical Center, Dallas, TX, United States* ; ^*2*^*MD Anderson Cancer Center, Houston, TX, United States* ; ^*3*^*Sun Yat-sen Memorial Hospital, Guangzhou, China*

####### **Correspondence:** Jieqiong Liu (liujieqiong@163.com)

**Background**

Cancer immunotherapy using immune checkpoint inhibitors has significantly improved the clinical outcomes of patients with multiple aggressive and advanced-stage cancers. Despite its enormous successes, the overall response rates of immune checkpoint inhibitor as a monotherapy remain suboptimal, especially in treating breast cancers. In order to improve the efficacy of cancer immunotherapies, there is an increased interest in combining immune checkpoint inhibitors with targeted agents to enhance antitumor effects. Previous studies have shown that anti-angiogenic drugs that suppress tumor vasculatures can improve the therapeutic effect of immune checkpoint blockades in preclinical models of human cancer. However, the exact mechanism of action of such synergism is unclear and how the two treatment modalities should be administered in terms of dosing and sequencing to produce the optimal response remains unknown. In the present study, we investigate the antitumor effect of combined blockade of vascular endothelial growth factor receptors 2 (anti-VEGFR2) and programmed death (PD-1) in murine breast cancer models as well as in a phase II trial.

**Methods**

We tested the combination of anti-PD-1 with different dose of VEGFR2-targeting antibody in syngeneic breast cancer mouse models. Tumor infiltrated immune cell subsets were profiled by flow cytometry. A cytokine array was carried out to identify mechanism of the synergy between the two modalities. The efficacy of this combination was further evaluated in advanced triple-negative breast cancer (TNBC) patients.

**Results**

Antibody targeting VEGFR2 sensitizes breast tumors to PD-1 blockade in a dose-dependent manner. Although both conventional and low-dose anti-VEGFR2 antibody treatments normalize tumor vessels, low-dose VEGFR2 blockade results in more robust immune cells infiltration and activation, and promotes the secretion of osteopontin (OPN) by CD8+ T cells. OPN subsequently induces tumor cell production of TGF-ß, which in turn upregulates PD-1 expression on immune cells. In advanced TNBC patients, combined low-dose anti-VEGFR2 and anti-PD-1 treamtnets demonstrated excellent tolerability and efficacy with a doubling of progression free survival as compared to historical anti-PD-1 trials. Tissue analyses further revealed that higher OPN and TGF-β expressions were correlated with improved clinical responses in patients.

**Conclusions**

Taken together, these results demonstrate that a dose-dependent synergism exists between anti-angiogenic therapy and immune checkpoint blockade, thus providing important insights into identifying the optimal strategies to combine immunotherapy with molecular targeted agents.

**Trial Registration**

NCT03394287

**Ethics Approval**

All patient-related procedures were performed with the approval of the Internal Review and the Ethics Boards of the Sun Yat-sen Memorial Hospital. All animal studies were reviewed and approved by the Institutional Animal Care and Use Committee of UT Southwestern Medical Center and Sun Yat-sen University.

#### P526 Combination cabozantinib and nivolumab treatment in patients with refractory metastatic renal cell carcinoma (mRCC)

##### Emily Kinsey, MD , Landon Brown, MD, Chester Kao, MD, Patrick Healy, Michael Harrison, MD, Megan McNamara, MD, Andrew Armstrong, MD, Sundhar Ramalingham, MD, Daniel George, MD, Tian Zhang, MD

###### Duke University Medical Center, Durham, NC, United States

####### **Correspondence:** Emily Kinsey (enkinsey@gmail.com)

**Background**

The treatment landscape has drastically changed in mRCC, moving from anti-VEGF therapies to an immunotherapeutic approach in the first line setting for IMDC intermediate or poor risk mRCC. Combining anti-VEGF and anti-PD-1 or anti-PDL-1 therapies have shown survival improvements in mRCC, leading to approvals for first-line axitinib-pembrolizumab and axitinib-avelumab [1,2]. Cabozantinib and nivolumab (cabo/nivo) is also a safe option in phase 1 trials with some durable responses [3]. We evaluated the outcomes of patients who received cabo/nivo for mRCC refractory to immunotherapy alone.

**Methods**

A retrospective analysis was performed of patients with mRCC treated with ipilimumab and nivolumab (ipi/nivo) and subsequently with cabo/nivo at Duke Cancer Center between September 2017 and February 2019. Patient outcomes were collected including demographic information, treatment details, responses, and frequency of adverse events. The cohort of patients treated with the cabo/nivo combination is presented here.

**Results**

Eighty-six patients were treated with ipi/nivo for mRCC and of these patients, 34 patients also received cabozantinib, either alone or in combination with nivolumab. Nine patients received cabozantinib prior to ipi/nivo, and 14 patients received cabozantinib after ipi/nivo, and 10 patients received combination therapy with cabo/nivo. Of the ten patients who received combination cabo/nivo, 2 were favorable risk, 6 were intermediate risk, and 2 were poor risk. One patient had progressive disease, 2 patients had stable disease, 3 patients had a partial response (50% responses), and 4 patients were unevaluable due to insufficient follow up. Eight of 10 patients had treatment ongoing at the time of data collection.

**Conclusions**

Progression on immunotherapy alone did not appear to confer resistance to cabo/nivo treatment for five of the six patients who had a disease response assessment. Phase III studies COSMIC-313 and Alliance A031704 (PDIGREE) are ongoing to evaluate cabozantinib in combination or in sequence to ipilimumab immunotherapy.

**References**

1. Motzer RJ, Penkov K, Haanen J, Rini B, Albiges L, Campbell MT, et al. Avelumab plus Axitinib versus Sunitinib for Advanced Renal-Cell Carcinoma. N Engl J Med [Internet]. 2019;380(12):1103–15.

2. Rini BI, Plimack ER, Stus V, Gafanov R, Hawkins R, Nosov D, et al. Pembrolizumab plus Axitinib versus Sunitinib for Advanced Renal-Cell Carcinoma. N Engl J Med [Internet]. 2019;NEJMoa1816714.

3. Nadal R, Mortazavi A, Stein M, Pal SK, Davarpanah N, Parnes HL, et al. Final results of a phase I study of cabozantinib (cabo) plus nivolumab (nivo) and cabonivo plus ipilimumab (Ipi) in patients (pts) with metastatic urothelial carcinoma (mUC) and other genitourinary (GU) malignancies. Ann Oncol. 2017;28(supplement 5):846O.

**Ethics Approval**

This study was approved by the Duke IRB (#Pro00101984)

#### P527 IgE-based therapeutic strategy against pancreatic cancer

##### Kamiya Mehla, PhD^1^, Kamiya Mehla^2^, Ragupathy Madiyalakan, PhD^3^, Christopher Nicodemus, MD^4^, Thomas Caffrey^2^, Michael Hollingsworth, PhD^2^ , Kelly O' connell^2^

###### ^*1*^*University of Nebraska Medical Centre, Omaha, NE, United States*; ^*2*^*unmc, Omaha, NE, United States* ; ^*3*^*Oncoquest Inc., Edmonton,, AB, Canada* ; ^*4*^*AIT strategies, Charlestown, MA, United States*

####### **Correspondence:** Michael Hollingsworth (mahollin@unmc.edu)

**Background**

Pancreatic ductal adenocarcinoma (PDAC) is a lethal disease, accounting for 338,000 new cases worldwide with a 5-year survival rate of less than 10%. Despite progress with new treatments, PDAC remains indomitable. New therapeutic strategies that are tumor-specific, relieve tumor-associated immunosuppression and modulate tumor stroma for the efficient delivery of chemotherapeutics are urgently required. Abundant evidence suggests a reciprocal relationship between allergies and pancreatic cancer. Distinct immune surveillance in allergic individuals might enhance immune protection against cancer [1-2]. We investigated a novel immunotherapeutic strategy to trigger a tumor antigen-specific allergic phenotype in a pre-clinical model of pancreatic cancer.

**Methods**

Using a combination of humanized anti-MUC1.IgE, anti-PD-L1, and PolyICLC, we investigated the efficacy of this unique triple combination in a pre-clinical model of pancreatic cancer using transgenic mice expressing human MUC1 and FcεRI (hMUC1/hFcεRI). IgE antibody against prostate-specific antigen (anti-PSA.IgE) and anti-MUC1.IgG was employed as controls. In parallel, transgenic mice for only hMUC1 or hFcεRIα were utilized as additional controls. We also performed immuno-depletion studies and analysis of tumor-infiltrating lymphocyte (TIL) from treated mice to investigate the role of innate (NK cells) and adaptive immune players (CD8 T cells) in providing protective benefits of derived from the combination therapy. In parallel, we performed a phospho-proteomic screen to elucidate the mechanism through which our proposed therapy harnesses NK cell-mediated anti-tumor immune response against pancreatic tumors.

**Results**

We noted that combined immunotherapy with a humanized IgE directed towards a tumor antigen expressed on pancreatic adenocarcinoma (MUC1), PolyICLC, and anti-PD-L1 induces antigen-specific cellular immune responses that mediate rejection of tumor cells in a pre-clinical model of pancreatic cancer. Our initial mechanistic studies suggest that effector mechanisms for anti-MUC1.IgE includes induction of ADCC by NK cells and activation of antigen-specific CD8 cytotoxic T lymphocytes (CTLs). Furthermore, we noted that the anti-MUC1. IgE-combination treatment promotes intra-tumoral NK cell activation and increased CD103+ DCs proportions inside the tumor, which were reversed upon NK cell depletion. Also, we noted the activation of SMAD1, but not SMAD3, pathways in NK cells post-treatment with anti-MUC1.IgE-based combination.

**Conclusions**

Taken together, this is the first study to show that specific stimulation of the IgE/FcεRIα axis in combination with PolyICLC and anti-PD-L1 can activate CD8 T cell and NK cell effector pathways and provide long-lasting tumor-protective benefits against pancreatic cancer. Our study provides preliminary evidence for the clinical applicability and rapid translation of an anti-MUC1.IgE based combination therapy against pancreatic cancer.

**References**

1. Gomez-Rubio P, Zock Jp, RAVA M et al. Reduced risk of pancreatic cancer associated with asthma and nasal allergies. Gut. 2017; 66 (2): 314-322.

2. Huang BZ, Le Marchand L, et al. Atopic allergic conditions and pancreatic cancer risk: Results from the Multiethnic Cohort Study. Int J Cancer. 2018;142 (10):2019-2027.

**Ethics Approval**

All animal studies were performed in accordance with the Institutional Animal Care and Use Committee guidelines (Approved IACUC protocol no: 15-123-02FC).

#### P528 Combination antibody treatment targeting PD-L1 and 4-1BB leads to reduced anti-tumor immunity against CT26 tumors in B-cell deficient JH-/- mice

##### Zhengming Yan, Heather Llewellyn, Kerry Kelleher, Bernadette Pascual, Angela Stauffer, Ying Ding, Xiaorong Li, Mark Ozeck, Jeffrey Toste, Han Yang, Haikuo Zhang, Sripad Ram, PhD , Cathy Zhang

###### Pfizer, Inc., San Diego, CA, United States

####### **Correspondence:** Sripad Ram (sripad.ram@gmail.com), Cathy Zhang (Cathy.Zhang@pfizer.com)

**Background**

The role of B cells in tumor immunity is poorly understood due to their conflicting roles that augment and suppress anti-tumor responses. For example, B cells can augment T-cell responses against tumor challenge as antigen presenting cells, while B cells can also blunt anti-tumor responses by developing neutralizing antibodies against therapeutic antibodies. In the context of targeted immunotherapy using antibody-based therapeutics, an important question arises as to whether B cells are necessary for eliciting an anti-tumor immune response.

**Methods**

We use two different clones of anti-PD-L1 antibody, 10F9G2 and MIH5, which are rat IgG2b and rat IgG2a, respectively, in conjunction with a mouse anti-mouse 4-1BB antibody. CT26 tumors were implanted subcutaneously in BALB/c mice or genetically B-cell deficient JH-/- mice. Binding affinity measurements were performed on BIACore. For flow cytometry, tumor, spleen and lymph nodes were harvested at different times after the last dose, dissociated and assayed for different immune cell markers. For single-cell RNASeq, mouse PBMCs were isolated and analyzed using the 10x Genomics platform.

**Results**

In vivo and in vitro pharmacokinetic studies revealed that MIH5 clone had better bioavailability than the 10F9G2 clone which is attributed to its higher binding affinity to FcRn. Combination treatment with the MIH5 clone resulted in superior antitumor efficacy when compared to using the 10F9G2 clone in wild type and JH-/- mice. However, for a given combination, JH-/- mice showed consistently poorer efficacy than wild type mice. More specifically, in the absence of B cells CD8+ T-cell expansion in the tumor was diminished and memory T-cell development was systemically impaired. We also report that B cells are required for reciprocal crosstalk activation between 4-1BB and PD-L1 signaling pathways which could augment the synergistic response of combination therapy. Results from single cell RNASeq suggest that B cells promote T-cell mediated tumor immunity through the production of cytokines that increase antigen processing and presentation, and support T-cell activation, proliferation and differentiation.

**Conclusions**

Our results reveal an underappreciated role of B cells in T-cell targeted cancer immunotherapy. They also underscore the importance of serum persistence of therapeutic antibodies in mediating anti-tumor immunity.

**Ethics Approval**

All experimental animal procedures complied with the Guide for the Care and Use of Laboratory Animals (Institute for Laboratory Animal Research, 2011) and were approved by the Pfizer Global Research and Development Institutional Animal Care and Use Committee (IACUC)

#### P529 A tumor microenvironment-recognizable polymeric conjugate for antigen delivery and cancer immunotherapy

##### Jung Min Shin, Seok Ho Song, Jae Ah Lee, Jae Hyung Park, PhD

###### Sungkyunkwan University, Suwon-si, Korea

####### **Correspondence:** Jae Hyung Park (jhpark1@skku.edu)

**Background**

Cytotoxicity T cells play central roles in destroying cancer cells in cancer immunotherapy, which is similar to immunological rejection [1]. However, in many cancer types, cancer cells are able to avoid recognition of cytotoxic T cells because they present self-antigen on the cell surface [2]. In this study, we designed polymeric conjugate which can cause foreign-antigen presentation by delivering foreign antigen to the cancer cells for recognition of cancer cell by cytotoxic T cells.

**Methods**

We conjugated ovalbumin (OVA), a model foreign antigen, to biocompatible hyaluronic acid (HA) via simple reductive amination method. The surface of HA was coated with polyethylene glycol (PEG) containing tumor microenvironment-sensitive linker. The polymeric conjugate was then used to demonstrate in vitro and in vivo foreign antigen presentation and cancer cell death by cytotoxic T cells.

**Results**

The OVA contents in the polymeric conjugate were 13.2 wt.% determined by BCA assay. Cellular uptake study revealed that the polymeric conjugate can deliver antigen into the cancer cells and cause antigen presentation as well. In the tumor-bearing mice model, the polymeric conjugate has been shown to accumulate at significant levels in tumor tissue, thus leading to effective in vivo antigen presentation.

**Conclusions**

In this study, we synthesized model foreign antigen-bearing polymeric conjugate. The polymeric conjugate showed a significant anticancer effect in tumor-bearing mice model. Therefore, it is suggested that the foreign antigen delivery system might be an alternative for cancer immunotherapy.

**References**

1. Friedl P, den Boer AT, Gunzer M. Tuning immune responses: diversity and adaptation of the immunological synapse. Nat Rev Immunol. 2005;5:532-545.

2. Garcia-Lora A, Algarra I, Garrido F. MHC class I antigens, immune surveillance, and tumor immune escape. J Cell Physiol. 2003;195:346-55.

#### P530 Blockade of IDO/TDO downstream effectors restricts a Treg-macrophage suppressive axis and resistance to anti-PD-1 therapy

##### Luis Campesato, PhD^1^, Sadna Budhu, PhD^1^, Jeremy Tchaicha, PhD^2^, Mathieu Gigoux, PhD^1^, Ivan Cohen^1^, Levi Mangarin, BS^1^, David Redmond^1^, Stephane Pourpe^1^, Cailian Liu, MD^1^, Roberta Zappasodi, PhD^1^, Dmitriy Zamarin, MD, PhD^1^, Mark Manfredi, PhD^2^, Karen McGovern, PhD^2^, Taha Merghoub, PhD^1^ , Jedd Wolchok, MD, PhD^1^ , Luis Campesato, PhD^1^

###### ^*1*^*Memorial Sloan Kettering Cancer Center, New York, NY, United States*; ^*2*^*Kyn Therapeutics, Cambridge, MA, United States*

####### **Correspondence:** Taha Merghoub (merghouT@mskcc.org), Jedd Wolchok (wolchokj@mskcc.org)

**Background**

Although immune checkpoint blockade (ICB) may result in clinical benefit for a subset of cancer patients, multiple mechanisms of resistance can impair optimal response. The catabolism of Tryptophan (Trp) by the enzymes IDO or TDO is a frequent phenomenon that plays a suppressive role in tumor immunity. Accumulation of the Trp metabolite L-Kynurenine (Kyn) was shown to act as agonist of the aryl hydrocarbon receptor (AHR) and that its activation promotes immunological tolerance.

**Methods**

Here by using pre-clinical models of melanoma and analysis of clinical samples we sought to characterize the mechanisms of immune suppression associated with the AHR pathway and to evaluate its potential as therapeutic target.

**Results**

RNAseq analysis of human cancers revealed an up-regulation of Kyn-degrading enzymes in patients responsive to PD-1 blockade. Also, we observed a correlation between the expressions of Kyn-AHR pathway genes with a TGF-β immune signature and markers associated with immunotherapy resistance (PD-1, FOXP3, CD206). We found that the overexpression of IDO or TDO in a pre-clinical model of melanoma (B16-F10) promoted CD8+ T cell dysfunction and accumulation of M2-TAMs and Tregs with an activation of the AHR pathway and enhanced suppressive function. Moreover, IDO-Kyn-AHR-mediated tumor progression was dependent on an interplay between Tregs and TAMs, which could be reversed by selective inhibition of the AHR. Treatment of IDO/TDO-expressing but not wild-type tumor models with a selective AHR antagonist delayed tumor growth, and its efficacy was further improved with PD-1 blockadeRNAseq analysis of human cancers revealed an up-regulation of Kyn-degrading enzymes in patients responsive to PD-1 blockade. Also, we observed a correlation between the expressions of Kyn-AHR pathway genes with a TGF-β immune signature and markers associated with immunotherapy resistance (PD-1, FOXP3, CD206). We found that the overexpression of IDO or TDO in a pre-clinical model of melanoma (B16-F10) promoted CD8+ T cell dysfunction and accumulation of M2-TAMs and Tregs with an activation of the AHR pathway and enhanced suppressive function. Moreover, IDO-Kyn-AHR-mediated tumor progression was dependent on an interplay between Tregs and TAMs, which could be reversed by selective inhibition of the AHR. Treatment of IDO/TDO-expressing but not wild-type tumor models with a selective AHR antagonist delayed tumor growth, and its efficacy was further improved with PD-1 blockade

**Conclusions**

In summary, our findings demonstrate that targeting the Kyn pathway through AHR inhibition could overcome key suppressive mechanisms and sensitize otherwise immune resistant tumors to PD-1 blockade.

**Ethics Approval**

All mice were maintained in microisolator cages and treated in accordance with the NIH and American Association of Laboratory Animal Care regulations. All mouse procedures and experiments for this study were approved by the MSKCC Institutional Animal Care and Use Committee.

#### P531 A tumor PD-L1-NLRP3 inflammasome signaling axis drives adaptive resistance to anti-PD-1 immunotherapy

##### Balamayooran Theivanthiran, PhD^1^, Kathy Evans, BS^1^, Nicholas DeVito, MD^1^, Michael Plebanek, PhD^1^, Michael Sturdivant, BS^1^, Alisha Holtzhausen, PhD^2^, Luke Wachsmuth, BS^1^, April Salama, MD^1^, Yubin Kang, MD^1^, David Hsu, MD, PhD^1^, Justin Balko, PhD, PharmD^3^, Douglas Johnson, MD, MSCI^3^, Mark Starr, BS^1^, Andrew Nixon, PhD^1^, Brent Hanks, MD, PhD^1^

###### ^*1*^*Duke University Medical Center, Durham, NC, United States* ; ^*2*^*University of North Carolina, Chapel Hill, NC, United States* ; ^*3*^*Vanderbilt University Medical Center, Nashville, TN, United States*

####### **Correspondence:** Brent Hanks (brent.hanks@duke.edu)

**Background**

An in-depth understanding of immune escape mechanisms in cancer are likely to lead to innovative advances in immunotherapeutic strategies. However, our understanding of these mechanisms driving both primary and secondary immunotherapy resistance remains incomplete. While many groups have described the role of NOD-, LRR- and pyrin domain-containing protein-3 (NLRP3) as a sensor for pathogen-derived danger signals by antigen-presenting cells in the innate immune system, relatively little is known about the contribution of NLRP3 to tumorigenesis and its role in modulating tumor responses to immunotherapy has not been explored

**Methods**

Several pre-clinical syngeneic and transgenic tumor models, an autologous humanized mouse tumor model, and clinical plasma and tumor specimens derived from advanced melanoma patients were utilized to investigate the genetic and cellular alterations of tumors escaping anti-PD-1 antibody immunotherapy.

**Results**

Using several pre-clinical tumor models, we have determined that granulocytic myeloid-derived suppressor cells (PMN-MDSCs) accumulate within progressing tumors undergoing treatment with anti-PD-1 antibody (ab) immunotherapy. This PMN-MDSC recruitment effect was found to be due to an autocrine Wnt5a-dependent upregulation of CXCR2 chemokines in tumor tissues. After additional study, we determined that this pathway was induced by HSP70-TLR4 stimulation and occurred in response to cytolytic CD8+ T cell activation. Using genetic and pharmacologic approaches we have found that this tumor-intrinsic signaling cascade is triggered by a PD-L1-dependent activation of the NLRP3 inflammasome via a STAT3-PKR signaling axis. This mechanism requires CD8+ T cell activation and IFN-gamma expression. However, treatment with chemotherapy or anti-CTLA-4 ab immunotherapy failed to induce a similar effect. Genetic ablation and pharmacologic inhibition of NLRP3 suppresses PMN-MDSC recruitment and enhances the efficacy of ant-PD-1 ab immunotherapy in an autochthonous model of BRAF(V600E) melanoma. We then sought to validate this pathway in clinical specimens where we found elevations in various myeloid markers and CXCR2 chemokines in tumor tissues as well as an increase in plasma HSP70 levels in non-responding melanoma patients progressing through anti-PD-1 ab immunotherapy.

**Conclusions**

This work reveals a tumor-intrinsic PD-L1-NLRP3 inflammasome signaling pathway triggered by CD8+ T cell activation that ultimately drives adaptive resistance to anti-PD-1 immunotherapy by promoting the recruitment of PMN-MDSCs to the tumor bed. The identification of the key PD-L1-STAT3-PKR-NLRP3 signaling axis responsible for this mechanism provides insight into promising strategies for augmenting the efficacy of checkpoint inhibitor immunotherapy as well as for monitoring the management of cancer patients undergoing these therapies. NLRP3 inhibitors represent promising agents for future anti-PD-1 combination clinical trials.

**Ethics Approval**

This study utilized clinical specimens derived from ongoing tissue acquisition protocols approved by the Institutional Review Boards at Duke University Medical Center (IRB approval number Pro00059349) and Vanderbilt University Medical Center (IRB approval number 100178).

#### P532 Increased adiposity reduces the response rate to a combinatorial CTLA-4 based therapy in diet-matched, renal tumor-bearing mice without substantially altering intra-tumoral T cell profiles

##### William Turbitt, PhD^1^, Shannon Boi^2^, Rachael Orlandella, BS^1^, Justin Gibson, BS^1^, Lyse Norian, PhD^1^

###### ^*1*^*The University of Alabama at Birmingham, Birmingham, AL, United States* ; ^*2*^*St. Jude Children's Research Hospital, Memphis, TN, United States*

####### **Correspondence:** Lyse Norian (lnorian@uab.edu)

**Background**

Associations between modifiable factors (e.g., adiposity) and immunotherapeutic efficacy remain uncertain. Preclinical studies suggest increased adiposity improves response rates to immune checkpoint blockade; however, few studies control for diet effects between chow-fed lean and high-fat diet-induced obese (DIO) mice. We found previously that DIO reduces the efficacy of an immunotherapy consisting of replication-deficient adenovirus (Ad) encoding tumor-necrosis factor-related apoptosis-inducing ligand (TRAIL) (AdT) plus class B oligonucleotides (CpG1826) in renal tumor-bearing mice [1]. To eliminate the compounding effects of diet and explore if therapy response can be improved in DIO mice, the current study evaluated response rates to AdT/CpG combined with anti-cytotoxic T-lymphocyte-associated protein 4 (CTLA-4) in diet-matched obese-resistant (OBR) versus DIO mice.

**Methods**

BALB/c mice were administered a high-fat diet for 20-weeks. Following diet administration, mice were characterized as either OBR or DIO, as previously described [2]. DIO mice possess hallmarks of obesity, including increases in systemic glucose, insulin, and leptin; whereas, OBR mice display phenotypic characteristics similar to chow-fed, lean controls. Mice were injected orthotopically with Renca-Luc renal cancer cells into the left kidney. At day seven post-tumor challenge, mice were randomized to receive saline or AdT/CpG injected intra-tumorally, followed by isotype control (n=7-9/group) or anti-CTLA-4 therapy (n=12/group). At day 21 post-tumor challenge, immunogenetic profiling of whole-tumors was performed via nanoString and tumor-infiltrating lymphocytes were characterized by flow cytometry.

**Results**

Both OBR and DIO therapy-treated mice had significant reductions in tumor weight compared to therapy-free controls (66% reduction for OBR; p=0.001 versus 55% reduction for DIO; p=0.007). However, therapy was effective in 75% of OBR mice compared to 50% of DIO mice. Over 30 differentially expressed genes were shared between OBR and DIO therapy-treated responders compared to therapy-free controls. A third of these “responder” genes were related to T cell function or migration (e.g., *Cd8a, CD274, Icos, Icosl, Zap70, Cxcr3, Xcr1, Il16*). Cellular analyses demonstrated that OBR therapy-treated responder mice had a greater percentage of tumor-infiltrating activated (CD44+) CD8+ T cells compared to DIO therapy-treated responder mice (p=0.004); however, CD8+ T cell IFNγ production and PD-1 expression were comparable between these groups.

**Conclusions**

When controlling for diet, increased adiposity reduced the response rate to combinatorial anti-CTLA-4 based therapy. However, both OBR and DIO therapy-treated responder mice shared immunogenetic and T cell profiles independent of weight status. To improve treatment strategies, future studies are needed to understand the mechanistic drivers of the differential response rates to immunotherapy observed with obesity.

**Acknowledgements**

Research reported in this abstract was supported by The National Institutes of Health (NIH) Grant #5R01CA181088 to LAN. WJT is supported by The Research Training Program in Basic and Translational Oncology (T32CA183926), The University of Alabama at Birmingham School of Medicine Department of Medicine, Division of Hematology and Oncology, Birmingham, AL. RMO is supported by The Cancer Prevention and Control Program (T32CA047888), The University of Alabama at Birmingham, Birmingham, AL. JTG is supported by The Training Program in Cell, Molecular, and Developmental Biology (T32GM008111), The University of Alabama at Birmingham, Birmingham, AL.

**References**

1. James, B.R., Tomanek-Chalkley, A., Askeland, E.J., Kucaba, T., Griffith, T.S., Norian, L.A. Diet-Induced Obesity Alters Dendritic Cell Function in the Presence and Absence of Tumor Growth. J Immunol. 2012; 189:1311-1321.

2. Boi, S.K., Buchta, C.M., Pearson, N.A, Francis, M.B., Meyerholz, D.K., Grobe, J.L., Norian, L.A. Obesity Alters Immune and Metabolic Profiles: New Insight from Obese-Resistant Mice on High-Fat Diet. Obesity. 2016; 24(10):2140-2149.

**Ethics Approval**

All animal procedures were approved by the Institutional Animal Care and Use Committee (IACUC) at the University of Alabama at Birmingham, approval number 20233.

### Combination Treatments

#### P533 The genomic architecture of serous carcinomas shapes the tumor microenvironment and modulates responses to targeted and immunotherapies

##### Sonia Iyer, PhD^1^, Shuang Zhang^2^, Anniina Farkkila^3^, David Pepin^4^, Raghav Mohan^4^, Sean Smith^5^, Tian Xia^1^, Ferenc Reinhardt^6^, Tony Chavarria^6^, Esmee Hoefsmit^6^, Shailja Pathania^7^, Yunlan Zhou^3^, Kevin Elias^8^, Benjamin Neel^2^, Robert Weinberg, PhD^1^

###### ^*1*^*Whitehead Institute for Biomedical Research, Cambridge, MA, United States* ; ^*2*^*NYU-Langone Medical Center, New York, NY, United States* ; ^*3*^*Dana-Farber Cancer Institute, Boston, MA, United States* ; ^*4*^*Massachusetts General Hospital, Boston, MA, United States* ; ^*5*^*Massachusetts Institute of Technology, Cambridge, MA, United States* ; ^*6*^*Whitehead Institute for Biomedical Resea, Cambridge, MA, United States* ; ^*7*^*University of Massachusetts, Boston, MA, United States* ; ^*8*^*Brigham and Women's Hospital, Boston, MA, United States*

####### **Correspondence:** Sonia Iyer (iyers@wi.mit.edu), Robert Weinberg (weinberg@wi.mit.edu)

**Background**

The cornerstone of the existing treatment of high-grade serous ovarian cancer (HGSOC) is DNA-damaging chemotherapy; however, practically all patients eventually develop the progressive disease and the 5-year survival is only 40%. Immunotherapy would seem to be an attractive alternative treatment to chemotherapy, yet existing immunotherapies perform poorly in ovarian cancer, with only ~10% of patients responding to checkpoint-blockade. Why this is the case remains poorly understood and there is a pressing need to understand the underlying biology of immune evasion in ovarian cancer. Unfortunately, the preclinical tools required to explore the relationship between the types of DNA damage repair deficiencies and immune evasion have been lacking. Hence, we have modeled the biology of ovarian cancer using patient-relevant mutational landscapes in an immune-proficient, syngeneic-mouse model to help us identify the contribution of common driver mutations to the immune repertoire in the tumor microenvironment, and thus to responses of HGSOC tumors to immunotherapy.

**Methods**

We hypothesize that the immune composition and gene expression signatures of the resulting tumors will vary based on the combination of genetic alterations and the DNA repair proficiency of the transformed cells. To this end, we have engineered novel syngeneic mouse models from murine fallopian tube epithelium using CRISPR/Cas9 technology. These tumors capture the most common combinations of co-occurring mutations observed in patients. These models can identify the contribution of common driver mutations to the heterotypic interactions between cancer and stromal/immune compartments and examine how DNA repair proficiency contributes to immunogenicity.

**Results**

To validate the DNA repair proficiency of the transformed cells, we measured Rad51 nuclear focus formation after ionizing radiation (IR) and PARP-inhibitor and DNA-damaging-agent sensitivity. The HR-deficient cell lines had significantly fewer Rad51 nuclear foci and were more sensitive to PARP-inhibition in comparison to HR-proficient cells. Initial immune /stromal analysis using flow cytometry, scRNAseq transcriptomic and immunofluorescence analysis revealed substantial differences in the myeloid and T-cell regulatory compartments between HR-proficient and -deficient primary and metastatic tumors and within the ascitic fluid. Preliminary results also suggest that inhibition of the DNA damage response, checkpoint kinase 1 in combination with immune checkpoint inhibitors, potentiates antitumor effects and augments cytotoxic T-cell infiltration.

**Conclusions**

These results reveal how common mutational drivers determine the microenvironment of the tumor and its response to treatment. Understanding the genetic basis of these complex cellular interactions will be critical to better tailor combinations of existing targeted treatments and immunotherapies in ovarian cancer to fight this devastating disease.

### Data Sharing, Handling, and Access

#### P535 The automated subset assignment pipeline (ASAP) for data-driven clustering and automated analysis of flow cytometry immunophenotyping

##### Isabelle Solman, MS, Ann Mongan, PhD, Lisa Blum, PhD

###### Pharmacyclics LLC, an AbbVie Company, Sunnyvale, CA, United States

####### **Correspondence:** Lisa Blum (lblum@pcyc.com)

**Background**

Characterization of heme malignancies, particularly in response to therapies, requires detailed monitoring and analysis of changes to patient immune subsets. New high-dimensional cytometry technologies have expanded the depth of data that can be collected, and several clustering algorithms have been described to improve the consistency and efficiency of associated data analysis. However, few studies have described a pipeline that is user friendly to immunologists and compared such results to validated data generated with manual gating by experts in the field. Here, we detail a flow analysis pipeline, ASAP, and demonstrate its precision and accuracy on data from a published study of over 200 clinical samples [1].

**Methods**

ASAP was built on several existing R packages (flowCore, flowPeaks, ggcyto, uwot) and makes use of marker enrichment modeling (MEM) scores for cell subset classification [2]. The pipeline performs data formatting and manages intermediate inputs and outputs to provide a seamless user experience. Starting with 3 input files defining panel information, preprocessing settings, and fcs files, ASAP performs preprocessing which includes data transformation, applying compensation, and removal of debris, doublets, and dead cells. Processed files are then clustered by cell markers and assigned to subsets based on MEM scores. ASAP automates the tabulation and visualization of user-defined and novel cell types. Results produced by ASAP were benchmarked with results from manual gating of a 16-color panel [1].

**Results**

Results produced by ASAP are highly concordant with those produced by expert manual gating, r2=0.98 (Figure 1). This high correlation is observed for major immune subsets with strong marker expression, nearly independent of cell frequency, ranging from 1%–90%. Analytical variability from 14 repeated acquisitions of the same human peripheral blood mononuclear cell sample on multiple days was low (interquartile range <10% median frequencies) and within the expected inter-experiment variability (Figure 2). The typical runtime of ASAP is less than 30 minutes for a study of 20 samples at 200,000 events/sample, and less than 5 hours for a large study of 150 samples at 3 million events/sample. In comparison, the manual analysis and quality control of the same dataset would require approximately 500 hours.

**Conclusions**

ASAP enables the automated analysis of flow cytometry data from large studies with precision, accuracy, and reproducibility comparable to the gold standard of expert manual gating. The automation of such a pipeline allows high consistency of gating and immune cell classification, which facilitates objective evaluation of patient response over time.

**Acknowledgements**

Funding source: Pharmacyclics LLC, an AbbVie Company

**References**

1. Solman I, You H, Taylor M, Dubovsky JA, Kipps TJ, Burger JA, Lal I, Chang BY, James DF, Hill JS. Ibrutinib vs chlorambucil: immunophenotypic and quantitative impacts on circulating immune cells in chronic lymphocytic leukemia (CLL). J Clin Oncol. 2017;35:7524-7524.

2. Diggins KE, Greenplate AR, Leelatian N, Wogsland CE, Irish JM. Characterizing cell subsets using marker enrichment modeling. Nat Methods. 2017;14:275-278.

**Fig. 1 (abstract P535).**
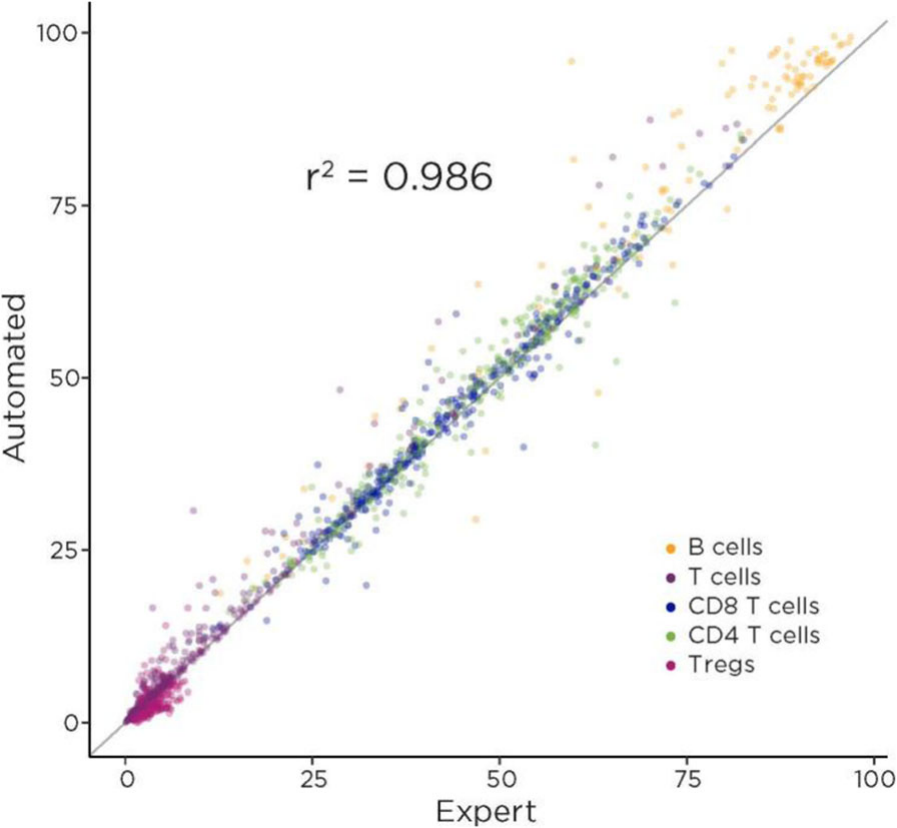
ASAP concordance with manual gating

**Fig. 2 (abstract P535).**
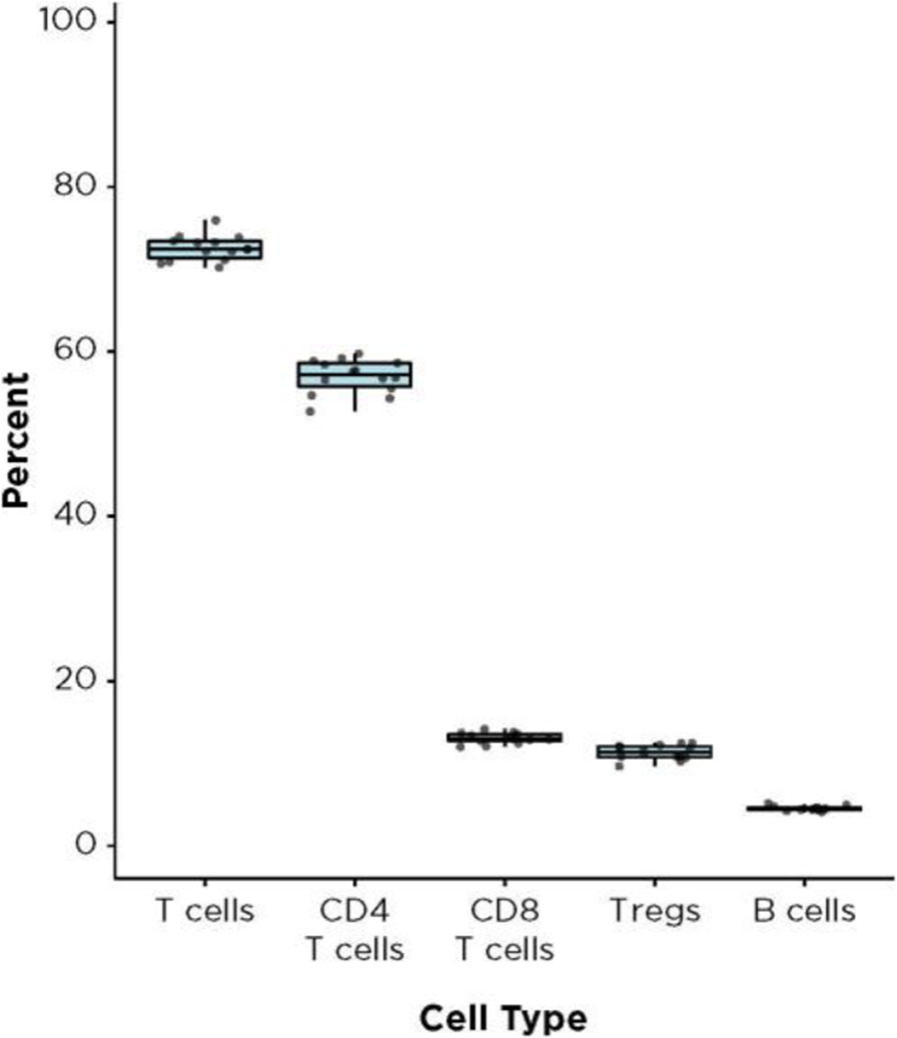
Reproducibility of ASAP for a repeat-run sample

### Education and Treatment Management

#### P536 Orbital tumor board: life saver or time waster?

##### Irfan Jeeva, MBBS, MRCOphth (UK), FRCOphth (UK), FEBO (EU), CCT, Ayesha Butt, Medical Student

###### Aga Khan University Hospital, Karachi, Pakistan

####### **Correspondence:** Ayesha Butt (ayeshabutt21@outlook.com)

**Background**

Orbital tumour board is a multidisciplinary approach towards ophthalmic cancer management, where relevant experts collaborate to manage patients holistically. Studies corroborate the notion that tumor boards positively affect patient outcomes. However, there is a scarcity of literature on the influence of orbital tumor boards globally and about tumor boards in general in Pakistan. There is no existing literature about orbital tumor boards in Pakistan. This study aims to assess the impact of tumor boards in managing ophthalmic cancers.

**Methods**

A retrospective review of data was carried out on cases that were presented in the orbital tumor board of Aga Khan University Hospital, the first orbital tumour board in Pakistan, from its commencement in August 2017 to May 2018.

**Results**

Out of a total of 80 patients in the study, 40%(n=32)were female and 60%(n=48) were male. The age of the patients ranged from 4 months to 78 years, with mean of 30 years. For analysis we divided the patients in three groups according to the nature of the diagnosis. Group 1 included those who had malignant tumours 40%(n=32), group 2 were those with benign tumours 30%(n=24) and group 3 was the non tumour group who were eventually diagnosed with other conditions 30%(n=24). Overall, 31.25% (n= 25) patients had a change in management plan, which was recorded, which included 10 patients in the malignant group, 9 in the benign group and 6 in the non tumour group.

**Conclusions**

A systematic review of literature has concluded that 54% of studies stated a change in management plans of at least 10% of patients post tumor board meetings. In our cohort, 31.25% of the patients had their treatment modified after tumour board discussion. The study shows that the management of ophthalmic cancers is influenced positively by tumour board discussions.

#### P537 A case of subacute cutaneous lupus erythematosus with adjuvant nivolumab for resected stage IV melanoma

##### Pragya Singh, MD^2^, Jeff Harvell^3^, Suraj Venna, MD FAAD^3^, Sekwon Jang, MD^3^, Logan Rhea, DO^2^

###### ^*1*^*Inova Fairfax Hospital Internal Medicine Residency Program, Falls Church, VA, United States* ; ^*2*^*Inova Health System, Falls Church, VA, United States* ; ^*3*^*Inova Schar Cancer Institute, Falls Church, VA, United States*

####### **Correspondence:** Logan Rhea (logan.rhea@inova.org)

**Background**

Nivolumab is a fully human immunoglobulin-G4 (IgG4) monoclonal antibody that selectively inhibits PD-1 activity, and can have various side effects. Drug-induced subacute cutaneous lupus erythematosus (SCLE), a unique drug-induced lupus erythematosus (DILE) syndrome, and is rare. Here, we report a case of SCLE after adjuvant nivolumab for resected stage IV melanoma.

**Methods**

A 77-year-old man with history of Stage I melanoma of the forehead, developed solitary pulmonary metastasis after 7 years which was resected with free margins. He began treatment with adjuvant nivolumab 480 mg every 28 days. Approximately two weeks after his 4th dose, he developed a pruritic rash limited to the trunk (Figure 1). This rash was believed to be a non-specific dermatitis related to nivolumab, and was treated with 6-day course of methylprednisolone. Although his symptoms initially improved, the rash progressed and he developed papules on the arms (Figure 2), along with polycyclic patches on the extensor forearms bilaterally (Figure 3).

**Results**

Skin biopsies were obtained, revealing interface dermatitis with focal epidermal pallor and necrosis, along with occasional eosinophils. The histologic differential included drug-induced erythema multiforme or DILE. Laboratory testing noted elevated erythrocyte sedimentation rate, C-reactive protein, positive antinuclear antibody (ANA), elevated anti-Ro and elevated anti-La antibody titers. Classic drug-induced lupus and systemic lupus erythematosus markers were negative.

**Conclusions**

Our case illustrates the development of DILE with SCLE morphology in a patient without previous history of autoimmune disease with nivolumab. DILE includes several lupus-like syndromes that develop with certain medication use, and resolve once discontinued. Histopathologic and autoantibody formation differ among DILE syndromes, though virtually all patients will have a positive ANA. The diagnosis is clinical. Chemotherapeutic agents are scarcely documented to cause DILE[1].

SCLE is a unique cutaneous variant of DILE. Skin lesions are generally annular, polycyclic or papulosquamous, and typically involve the upper trunk and upper extremity extensor surfaces[1]. Autoantibody production in SCLE is typical, and associated with high anti-Ro antibodies titers[2]. Anti-Ro antibodies are neither specific for Sjogrens, nor SCLE, additionally seen in malignancy and autoimmune liver injury. Symptoms may occur at any point during therapy. Treatment, like all DILE, is discontinuation of offending medication. Additional supportive treatments include photoprotection and topical corticosteroids. Lesions are expected to resolve in weeks, without scarring[3].

Our patient’s diagnosis was made clinically, with support of pathology and autoantibody panels. Nivolumab has been documented to cause SCLE in literature at least twice[4,5], however previous patients developed also other immune-related side effects.

**References**

1. Lamond NW, Younis T, Purdy K, Dorreen MS. Drug-induced subacute cutaneous lupus erythematosus associated with nab-paclitaxel therapy. Curr Oncol. 2013;20(5):e484-7.

2. Lowe G, Henderson CL, Grau RH, Hansen CB, Sontheimer RD. A systematic review of drug-induced subacute cutaneous lupus erythematosus. Br J Dermatol. 2011;164:465–72.

3. Srivastava M, Rencic A, Diglio G, et al. Drug-induced, Ro/ssapositive cutaneous lupus erythematosus. Arch Dermatol. 2003;139:45–9.

4. Liu, Rose C et al. “Subacute cutaneous lupus erythematosus induced by nivolumab.” The Australasian journal of dermatology. 2018;59(2): e152-e154 .

5. Zitouni N, Arnault JP, Dadban A, Attencourt C, Lok C, Chaby G. Subacute cutaneous lupus erythematosus induced by nivolumab. Melanoma Research. 2019; 29(2):212-215.

**Consent**

Consent was obtained from the patient for publication of this abstract.

**Fig. 1 (abstract P537).**
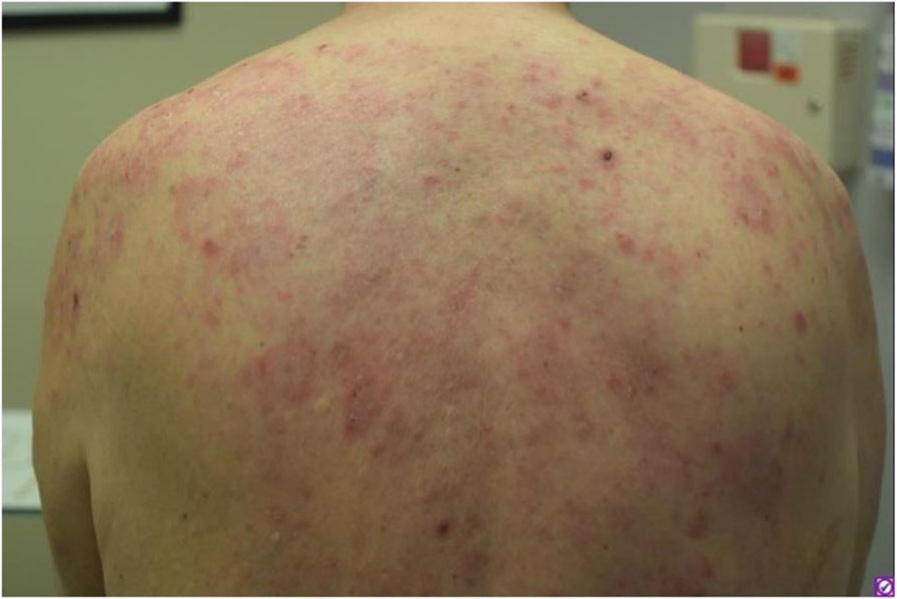
See text for description

**Fig. 2 (abstract P537).**
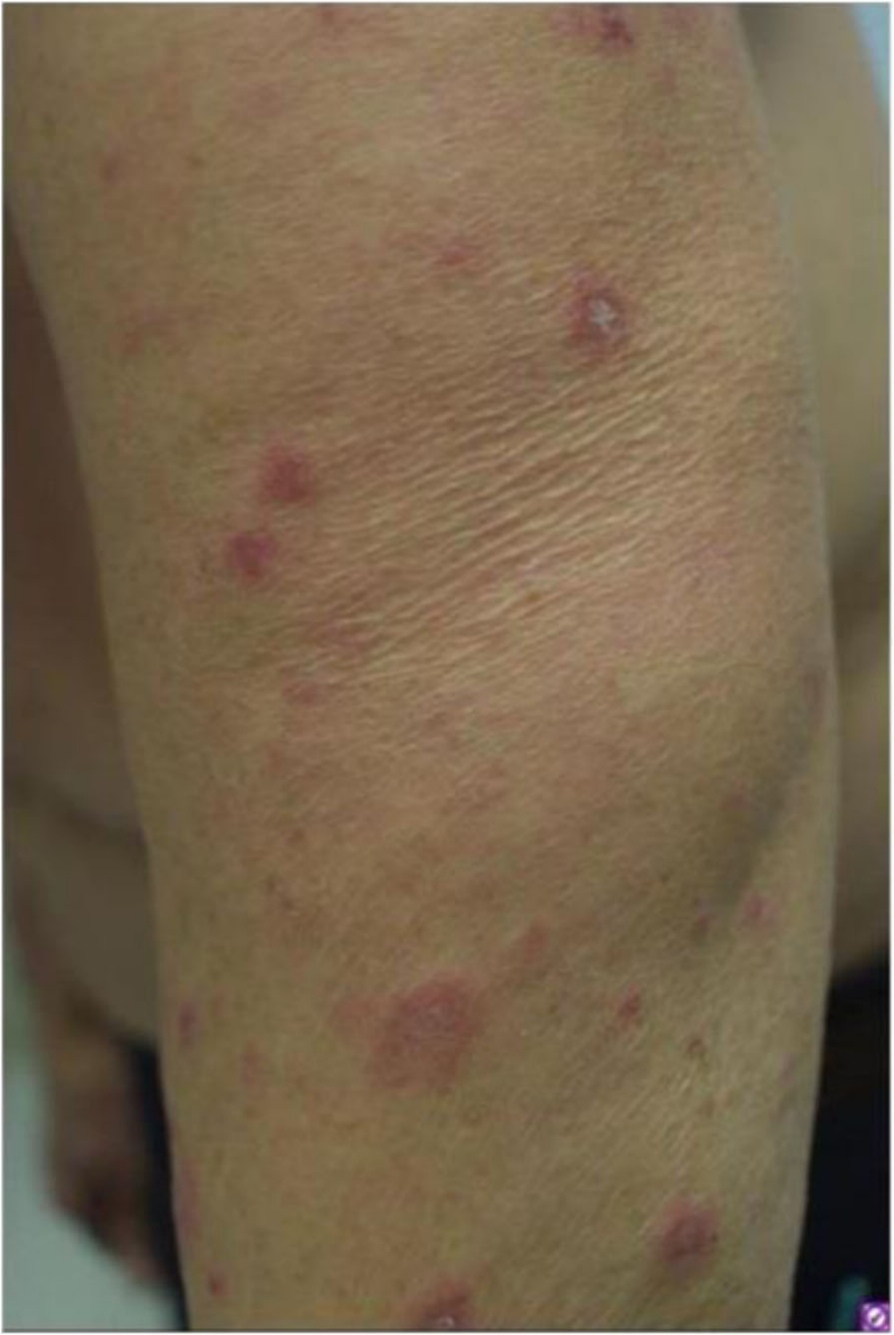
See text for description

**Fig. 3 (abstract P537).**
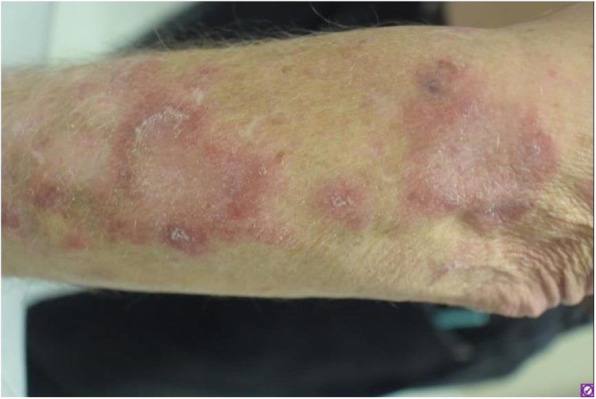
See text for description

#### P538 Outcomes of a newly formed multidisciplinary retinoblastoma service

##### Ayesha Butt, Medical Student , Irfan Jeeva, MBBS, MRCOphth (UK), FRCOphth (UK), FEBO (EU), CCT

###### Aga Khan University Hospital, Karachi, Pakistan

####### **Correspondence:** Ayesha Butt (ayeshabutt21@outlook.com)

**Background**

Retinoblastoma is the most common intraocular malignancy of childhood, accounting for 3 - 4% of all pediatric neoplasms. Mortality associated with retinoblastoma in the developed world is 3% to 5% whereas in the developing world it is 40%–70%. There is scarcity of research about retinoblastoma in Pakistan. We aim to study the presentation of retinoblastoma and impact of multidisciplinary management and tumor board discussions on its treatment at a tertiary care hospital in Pakistan

**Methods**

Retrospective review of retinoblastoma cases from May 2016 to April 2018 at Aga Khan University Hospital.

**Results**

A total of 35 eyes of 26 patients were studied with 9 presenting with bilateral retinoblastoma. 57.7% (n=15) of the population were males. The age range was 1 - 84 months (median: 14.5 months). The children presented from 3 different countries with most (76.9%, n=20) belonging to medium, upper medium or upper socioeconomic class.

The most prevalent symptom was leucocoria (38.5%, n=10) followed by decreased visual response (15.4%, n=4), squint (15.4%, n=4) and positive family history (15.4%, n=4). Most of the eyes (57.1%, n=20) were Group E on presentation, followed by Group D (20%, n=7) and 17.1% (n=6) Group B. One eye each presented with Group C and A. All the eyes that presented with Group A or B were of patients who had bilateral disease with advanced stage in the other eye. 54.3% (n=19) of the eyes were treated with a combination therapy including enucleation, focal lasers and systemic chemotherapy, 20% (n=7) with only focal lasers and 17% (n=6) with only enucleation. 42.3% (n=11) of the children presented with heritable traits. One patient (3.8%) did not survive

**Conclusions**

At our center we see Retinoblastoma in children with diverse background and majority presented with advanced disease. Multimodal therapy improved outcome. Awareness needs to be created for early detection and referral especially in the developing world.

#### P539 Management of melanoma: education improving clinical decisions of oncologists

##### Haleh Kadkhoda, MS^1^, Jeffrey Weber, MD, PhD^2^, Charlotte Warren^1^, KINJAL PARIKH, PharmD^1^, Ann Carothers, MEd^1^

###### ^*1*^*Medscape Oncology, Houston, TX, United States*; ^*2*^*NYU Langone, New York, NY, United States*

####### **Correspondence:** KINJAL PARIKH (kparikh@medscape.net)

**Background**

The evolving melanoma treatment algorithm has highlighted the role of immune checkpoint inhibitors in treating patients with this cancer. From the first drug approval in 2011 through today, many changes have occurred in treatment approaches. Selecting the most appropriate therapy with implementation of monitoring strategies for managing adverse events is vital. The goal of this study was to determine if participation in an educational activity can improve the proficiency of oncologists on the application of checkpoint inhibitors in adjuvant and metastatic melanoma.

**Methods**

An online continuing medical education (CME)-certified interactive, text-based activity developed by Medscape Education Oncology and the Society for Immunotherapy of Cancer included 2 patient cases, which served as the foundation for interactive questions. Educational design included a “test, then teach” approach to elicit cognitive dissonance, with evidence-based feedback provided following each learner response. A repeated pairs pre-/post-assessment study design with 3 case-based questions and 1 confidence question was used. A chi-square test assessed differences from pre- to post-assessment. P values .26 is extensive). The activity was launched online 2/25/19 and data were collected through 5/8/19.

**Results**

Participation in education resulted in statistically significant improvements and an extensive educational effect for oncologists (n=62; P < .001; V =.342). An average of 41% correctly responded to pre-assessment questions, increasing to 75% post-assessment. 40% of oncologists reported greater confidence prescribing immunotherapy in melanoma. Significant improvements in competency were observed in the following areas:

• Selection of an evidence-based regimen for patients with melanoma in the adjuvant setting (24% vs 73%, P < 0.001, V = 0.484, Figure 1)

• Identification of the most appropriate regimen for patients with metastatic melanoma following molecular testing (47% vs 74%, P <.01, V = 0.280, Figure 2)

• Implementing team-based strategies for monitoring and managing irAEs (52% vs 77%, P <.01, V = 0.269, Figure 3)

**Conclusions**

This online interactive, case-based, CME-certified educational activity resulted in significant gains in oncologist competency in identifying and selecting appropriate treatments for patients with melanoma in the adjuvant and metastatic setting, and in implementing monitoring and management strategies (Figure 4). These results demonstrate the effectiveness of on-demand education in improving the translation of clinical knowledge into practice scenarios while improving clinician confidence.

**Acknowledgements**

Supported by an educational grant from: Bristol-Myers Squibb Company, Incyte Education, and Merck & Co, Inc.

**Fig. 1 (abstract P539).**
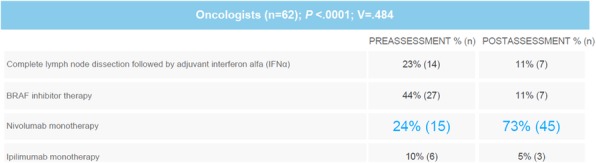
Selecting Evidence-Based Regimens

**Fig. 2 (abstract P539).**
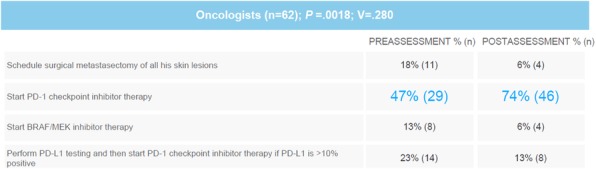
Competency with Molecular Profiling Treatment Selection

**Fig. 3 (abstract P539).**
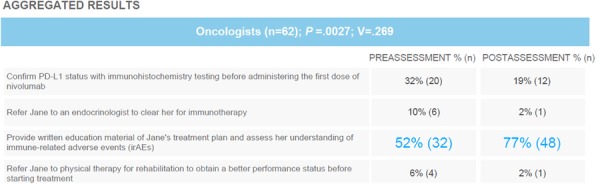
Competency Implementing Team-Based Strategies for irAEs

**Fig. 4 (abstract P539).**
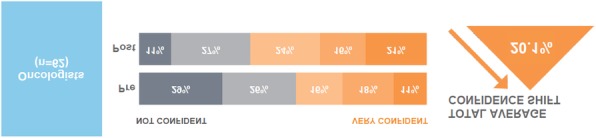
Confidence Prescribing Immunotherapy in Melanoma

#### P540 Understanding oncologist preferences for chemotherapy and immunotherapy monotherapy and combinations for first-line treatment of metastatic non-small cell lung cancer: a discrete choice experiment

##### Candice Yong, PhD^1^ , Brian Seal, RPh, MBA, PhD^1^ , Kathleen Beusterien, MS^2^, M. Janelle Cambron-Mellott, PhD^2^, Martine Maculaitis, PhD^2^, Kelly Clapp^2^, Emily Mulvihill, MBA^2^, Ion Cotarla, MD, PhD^1^, Ranee Mehra, MD^3^

###### ^*1*^*AstraZeneca, Gaithersburg, MD, United States* ; ^*2*^*Kantar, New York, NY, United States* ; ^*3*^*University of Maryland, Baltimore, MD, United States*

####### **Correspondence:** Candice Yong (candice.yong@astrazeneca.com), Brian Seal (brian.seal@astrazeneca.com)

**Background**

While platinum-based chemotherapy doublets were the first-line (1L) standard of care for metastatic non-small cell lung cancer (mNSCLC), the treatment landscape has recently shifted with the approval of immune checkpoint inhibitors. With the availability of these newer treatment options, it is important to elucidate the risk:benefit decision-making with regards to efficacy and toxicities that underlies oncologists’ real-world practice in mNSCLC. This study aimed to quantify preferences for attributes associated with immunotherapy and chemotherapy for mNSCLC among US oncologists.

**Methods**

Oncologists were recruited via a healthcare research panel to complete a cross-sectional online survey. Preferences were assessed in two separate discrete choice experiments (DCEs) where oncologists chose their preferred option in a series of tasks showing two alternative treatment profiles. The profiles varied based on six attributes identified in qualitative interviews with oncologists (Table 1). In both DCEs, the attributes were based on 1L treatment of patients with PD-L1 tumor cell (TC) >=1% and PD-L1 TC >=50% expression, respectively. A Hierarchical Bayes model was applied to estimate preference weights for each level of the treatment profile attributes.

**Results**

Oncologists (N=216) with an average of 16 years in practice participated; 69% were in community settings, and 31% were in academic settings. Attribute preferences were similar for patients with PD-L1 TC >=1% and PD-L1 TC >=50% (Figures 1a-1b). Improvements in OS were more important relative to improvements in any other attributes evaluated. Reducing the risk of neutropenia was second most important. The preference data showed a willingness to make trade-offs between efficacy and toxicity. For example, for both PD-L1 patient groups, oncologists would require an increase in OS by 5 months to accept an increase in risk of neutropenia from <1% to 25% and an increase in OS by 4 months to accept an increase in risk of pneumonitis from <1% to 5% (all grades) and <1% to 3% (Grade 3/4).

**Conclusions**

This study provides key insights into the trade-offs that oncologists are willing to make between efficacy and toxicities when selecting 1L treatment for patients with mNSCLC. Results suggest that improvements in OS were highly valued by oncologists and required to offset increases in the risk of toxicities, particularly neutropenia and pneumonitis. These findings were observed irrespective of patient PD-L1 status, which supports the consistency of oncologist preferences despite the differences in OS that have been observed for patients with PD-L1 TC >=1% or PD-L1 TC >=50% treated with immunotherapy regimens.

**Acknowledgements**

The authors acknowledge Oliver Will, PhD, and Bernadette Hallissey, PhD, for their assistance with data analysis

**Ethics Approval**

This study was reviewed by Pearl Institutional Review Board (Indianapolis, IN, US) and determined to be exempt.

**Table 1 (abstract P540).**
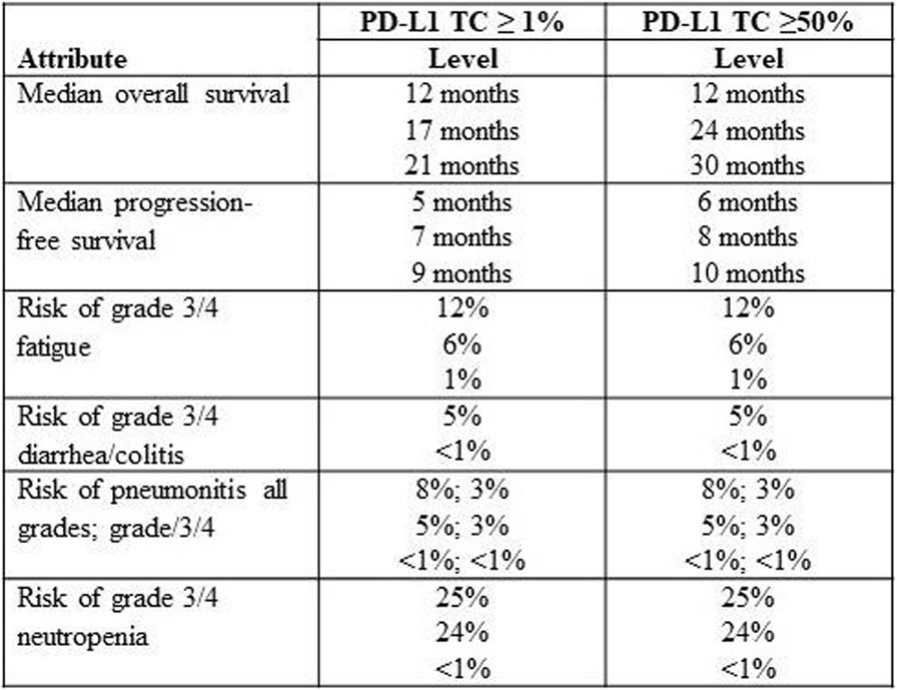
Attributes and levels used for each DCE

**Fig. 1 (abstract P540).**
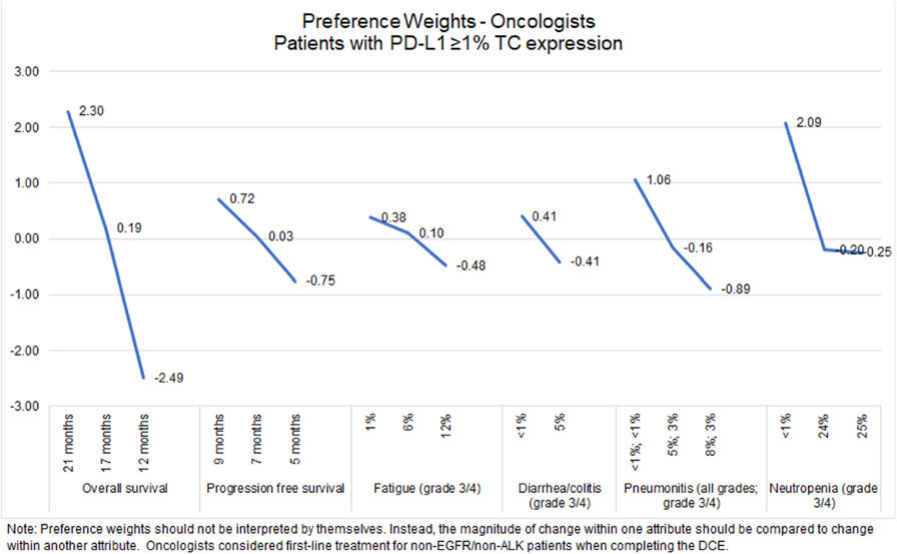
See text for description

#### P541 Informing and empowering cancer caregivers about immunotherapy: results from a psychoeducational workshop on immunotherapy

##### Maria Gonzalo^1^, Claire Saxton, MBA^1^ , Alex Swales, MSW^1^, Alexandra Zaleta, PhD^2^

###### ^*1*^*Cancer Support Community, Washington DC, United States* ; ^*2*^*Cancer Support Community, Philadelphia, PA, United States*

####### **Correspondence:** Claire Saxton (csaxton@cancersupportcommunity.org)

**Background**

Providing education and support for caregivers of cancer patients improves physical, psychosocial, and quality of life outcomes for both caregivers and patients.[1-4] As the use of immunotherapy continues to expand, it is important that caregivers have access to education and community resources to support their loved ones in making informed decisions, recognizing side effects, and getting optimal care. This analysis explores gains from Cancer Support Community’s Frankly Speaking about Cancer (FSAC): Immunotherapy program, a national evidence-based educational program created for cancer patients and caregivers.

**Methods**

One thousand two hundred thirty-two adults attending FSAC: Immunotherapy psychoeducational workshops nationwide from 2014-2018 completed a post-workshop survey. Participants rated their pre and post-workshop immunotherapy knowledge (“How knowledgeable were/are you about immunotherapy before/after this workshop?” 1= Not at all, 5= Very much) as well as their level of confidence speaking to their doctor (“Before/After this workshop I spoke with/feel more confident in speaking with my doctor about immunotherapy:” 1=Strongly disagree, 5=Strongly agree). 23% of respondents were non-professional caregivers; the remainder included cancer patients (66%), health care professionals (8%), and others (3%). This analysis focuses on 282 non-professional caregivers who self-reported background characteristics and workshop-specific outcomes (81% response rate). Descriptive analyses and paired samples t-tests were conducted to assess workshop outcomes.

**Results**

Of the 282 caregivers, 54% were spouses/partners, 31% family, and 15% friends. Caregivers were primarily White (72%) and female (70%); average age was 55 (s.d.= 17.6 years; range: 19-85 years). 56% of caregivers reported this was their first informational workshop. While 46% reported that immunotherapy treatment was an option for their loved one, only 9% reported speaking with their doctor about immunotherapy and its potential side effects. Post- workshop, 47% caregivers reported ‘high’ or ‘very high’ immunotherapy knowledge vs. pre-workshop (11%), a statistically significant difference in perceived knowledge (t(210)=24.05, p<.01). Caregivers also reported an increase in confidence in discussing immunotherapy and its side effects with their doctors, with 71% reporting confidence (agree/strongly agree) in speaking to their doctor about immunotherapy post-workshop vs. 24% pre-workshop (t(83)=5.8, p<.01), and 64% feeling more confident in talking with their doctor about immunotherapy’s side effects post-workshop vs. 32% pre-workshop (t(98)=8.9, p<.01). The workshop was well-received, with 87% recommending the workshop to others.

**Conclusions**

Results suggest that the FSAC: Immunotherapy program increases caregivers’ perceived knowledge about immunotherapy and their perceived confidence in talking to their doctor about immunotherapy. Our results highlight the potential benefits of immunotherapy education and support for cancer caregivers.

**Acknowledgements**

These workshops and analysis were funded in part by generous support from AstraZeneca, Bristol-Myers Squibb, and Merck.

**References**

1. Northouse L, Williams AL, Given B, McCorkle R. Psychosocial care for family caregivers of patients with cancer. J Clin Oncol. 2012;30(11):1227-1234.

2. Harvey A, Amsellem M, Suarez R. Informational and emotional support utilization and needs of lung cancer patients and caregivers: Results from a national education program. J Canc Educ 2014;29(Suppl 1).

3. Harvey A, Amsellem M, Suarez R. Information and emotional support utilization among cancer caregivers: results from a national sample of education program attendees. Psycho-Oncology 2015;24(Suppl. 2):160.

4. Saxton C, Amsellem M, Suarez R. Caregiver participation in a psychoeducational cancer support program: results from a national sample. J Canc Educ 2016;31(Suppl 1).

**Ethics Approval**

This study was conducted under IRB-exempt protocols [category 45 CFR 46.101(b) 2].

### Immune Cell Biology

#### P542 Case of an outstanding anti-cancer immune response in a microsatellite stable metastatic colon cancer

##### Thomas Duhen, PhD^1^ , Carmen Ballesteros Merino, PhD^1^, Venkatesh Rajamanickam^1^, Brady Bernard, PhD^1^, Bernard Fox, PhD^1^, Eric Anderson, MD, PhD^2^, Philippa Newell, MD^1^

###### ^*1*^*Providence Cancer Institute, Portland, OR, United States* ; ^*2*^*Knight Cancer Institute, OHSU, Portland, OR, United States*

####### **Correspondence:** Thomas Duhen (thomas.duhen@providence.org)

**Background**

Microsatellite stable colorectal cancers are known to harbor low mutation burden, a limited immune infiltration of the tumor and thereby respond poorly to immunotherapy.

**Methods**

Here we describe a case of microsatellite stable metastatic colon cancer with an outstanding anti-cancer response. Briefly, the patient was a 53-year-old man with a T4bN2aM1 metastatic colon cancer diagnosed in 2012. The primary tumor was resected at time of diagnosis and the patient underwent several cycles of chemotherapy from 2012 to 2017 in an effort to treat the multiple lesions present in the liver considered unresectable at time of surgery. In 2018, the patient’s CEA levels began to rise and he underwent a left hepatectomy to remove a slow growing mass.

**Results**

Analysis of the liver resection by flow cytometry revealed a large number of immune cells infiltrating the tumor, a phenotype distinct from the other colorectal liver metastases (CRLMs) we analyzed to date. Most importantly, more than 60% of the infiltrating immune cells were CD8 T cells, one third of which co-expressed the markers CD39 and CD103, a phenotype characteristic of tumor-reactive T cells. Those CD39+CD103+ CD8 T cells expressed high levels of PD-1 and CTLA-4 and were highly proliferative in vivo as demonstrated by Ki-67 staining. Using multiplex IHC, we confirmed that this pattern was unique to the recurring CRLM as very few CD8 T cells were found in the primary tumor. Sequencing of the CDR3 region of the TRB genes expressed by the CD8 T cells isolated from the CRLM supported the flow cytometry data, with the presence of one unique clonotype accounting for one-third of all the CDR3 sequences in the CD8 T cells, indicative of a strong local expansion. We are now performing whole-exome and RNA sequencing on both tissues in order to identify the mutations recognized by those tumor-infiltrating CD8 T cells and determine if those mutations were already present in the primary tumor or emerged during the metastatic transformation process.

**Conclusions**

This case shows that even cancers with low tumor mutation burden can lead to strong immune responses overtime and this knowledge should be considered to establish patient’s treatment plans in the future.

**Ethics Approval**

All subjects signed written informed consent approved by the Providence Portland Medical Center Institutional Review Board (IRB protocol no. 06-108A).

**Consent**

Consent was obtained for publication of this abstract.

#### P543 A transcriptional signature of exhausted CD8+ T cells predicts relapse-free outcome in ER+ breast cancer patients

##### Colt Egelston, PhD^1^, Weihua Guo^2^, Christian Avalos^2^, Min Hui Lim^2^, Diana Simons^2^, Peter Lee, MD^2^

###### ^*1*^*Beckman Research Institute, City of Hope, Duarte, CA, United States*; ^2^City of Hope, Duarte, CA, United States

####### **Correspondence:** Peter Lee (plee@coh.org)

**Background**

Estrogen receptor positive (ER+) breast cancer tumors are generally poorly immune infiltrated tumors as compared to triple negative breast cancer (TNBC). In TNBC the presence of tumor infiltrating lymphocytes (TILs) is predictive of response to chemotherapy and associates favorably with patient survival. However, in ER+ breast cancer the relationship between T cell infiltration and disease is less clear. Exhausted CD8+ T cells has been described in murine models of chronic disease and recently several human carcinomas. Here we profile human breast tumors for the functional, phenotypic, and transcriptional profile of exhausted CD8+ T cells and their correlation with survival in patients.

**Methods**

Fresh surgical tumor specimens were obtained from consented patients. Single cell suspensions were analyzed by flow cytometry for immune phenotyping and functional assessment of cytokine production by T cells. Single cell sorted T cells were subjected for whole transcriptome RNA sequencing and bulk sorted T cells were submitted for T cell receptor repertoire sequencing by Adaptive Biotechnologies. Transcriptional profiles generated from single cell sequencing were utilized for signature score analysis of outcomes in public TCGA and Metabric data sets.

**Results**

Exhausted CD8+ T cells were identified in both ER+ and TNBC. Functional and phenotypic analysis identified T cells to have a reduced capacity for IFNγ, TNFα, and IL-2 production. Further phenotyping of exhausted CD8+ T cells revealed a loss of expression of both the IL-7 receptor alpha (CD127) and KLRG1, suggesting terminal differentiation of these cells. Single cell and T cell receptor sequencing revealed distinct transcriptional and clonal signatures of exhausted CD8+ T cells. T cell receptor analysis demonstrated increased oligoclonality of exhausted CD8+ T cells as compared to other CD8+ TILs. Finally we demonstrate that a transcriptional signature of exhausted CD8+ denotes relapse-free survival in ER+ breast cancer patients.

**Conclusions**

This work is the first to demonstrate a T cell subset beneficial to survival outcomes of ER+ breast cancer patients. Immunotherapeutic interventions to expand exhausted CD8+ T cell populations may offer improved overall survival in breast cancer patients.

**Ethics Approval**

Fresh tissues were obtained from patients who gave institutional review board (IRB)-approved written informed consent prior to inclusion in the study (City of Hope IRB 05091, IRB 07047, and IRB 14346).

#### P544 The generation of memory CD8 T cells is regulated by intracellular Galectin-3

##### William Redmond, PhD, Mohammad Farhad, MS

###### Earle A. Chiles Research Institute, Portland, OR, United States

####### **Correspondence:** Mohammad Farhad (farhad@ohsu.edu)

**Background**

Background: CD8 T cells are a critical component of anti-tumor immunity and enhancing their function can promote tumor elimination. The levels of extracellular galectin-3 (Gal-3), a carbohydrate binding protein, are increased during tumor progression in many cancers and higher levels of Gal-3 are associated with a poor prognosis. Interestingly, intracellularly, Gal-3 functions through protein-protein interactions and is upregulated in T cells following T cell receptor (TCR) stimulation in the presence of IL-2. However, the function and mechanisms by which intracellular Gal-3 regulates CD8 T cell responses are poorly understood.

**Methods**

Wild type (WT) or Gal-3-/- OT-I CD8 T cells were adoptively transferred into WT mice and then stimulated with soluble ovalbumin protein along with an agonist anti-OX40 mAb or rIL-2/IL-2 mAb complexes (IL-2c). The frequency, phenotype, and effector/memory status of the adoptively transferred CD8 T cells was determined in the peripheral blood (days 7 and 14) and spleen (day 29) by flow cytometry.

**Results**

In the current study, we demonstrate that Gal-3-deficient CD8 T cells exhibited no defects in early (36 hrs) activation or proliferation. In contrast, Gal-3-/- CD8 T cells exhibited decreased survival and a reduced capacity to develop into memory cells following stimulation with cognate antigen plus agonist anti-OX40 mAb or IL-2 in vivo. Decreased survival of Gal-3-/- T cells was associated with increased apoptosis (Annexin V) and occurred in a cell-intrinsic manner. Additional studies are underway to evaluate the extent to which Gal-3 regulates T cell-mediated anti-tumor immunity.

**Conclusions**

Together, these data implicate intracellular Gal-3 as a critical mediator of OX40-mediated CD8 T cell survival and memory formation following antigen exposure.

#### P545 Expression of retention integrins by human CD4+ T cells

##### Kevin Lynch, MD, Marit Melssen, MSc, Walter Olson, PhD, Craig Slingluff

###### University of Virginia, Charlottesville, VA, United States

####### **Correspondence:** Craig Slingluff (cls8h@virginia.edu)

**Background**

T-cell retention in epithelial tumors is mediated by transmembrane proteins (CD49a, CD49b, CD103) known as retention integrins (RI)[1]. Expression of RI correlates with cytotoxicity of CD8+ T-cells in human epithelial tumors [2-3] and with immunosuppression by regulatory T-cells in murine models [4]. Recently, we reported that RI expression by CD8+ cells was modified by exposure to IL2, IL4, IL10, and TGFβ, as well as by T-cell receptor (TCR) stimulation [5]. TGFβ has also been shown to upregulate CD103 in human CD4+ memory cells [6] but investigation into RI expression by CD4+ cells has otherwise been sparse. We hypothesized that expression of RI by human CD4+ cells would be modulated by cytokines and TGFβ in a pattern similar to CD8+ cells.

**Methods**

Peripheral blood mononuclear cells isolated from five human donors were stimulated with mouse anti-CD3 and anti-CD28 for 24 hours. Cells were then cultured with TGBβ (5 ng/mL), IL2 (1000 CU/mL), IL4 (20 ng/mL), or IL10 (100 ng/ml) for six days. Integrin expression was quantified by flow cytometry. Data generated by flow cytometry was analyzed using FlowJo software (Tree Star), and results were compared using Student’s two-tailed T test.

**Results**

As shown in Table 1, TGFβ upregulated expression of CD49a and CD103 in TCR stimulated (TCR+) CD4+ cells relative to TCR+ controls (p = 0.02,

**Conclusions**

When comparing the expression of RI by CD4+ cells to our prior findings in CD8+ cells[5], we noted both common themes and significant differences. TGFβ upregulates CD49a and CD103 on both TCR+CD8+ and TCR+CD4+ cells. In contrast, the effects of IL2 and IL4 differ based on cell type. IL2 upregulates CD49a in TCRnegCD8+ cells but downregulates CD49a in TCR+CD4+ cells. IL4 decreased CD49a expression by TCR+ CD8+ cells, but decreased CD49a expression only in TCRnegCD4+. This heterogenous response to cytokine stimulation emphasizes the complexity of RI regulation and raises the question of whether regulation of RI expression is CD4+ subtype specific. This data supports the need for further investigation into the regulation of RI expression by CD4+Th1, CD4+Th2, and CD4+T regulatory cell populations.

**References**

1. Konkel J, Zhang D, Zanvit P, Chia C, Zangarle-Murray T, Jin W, Wang S, Chen W. Transforming Growth Factor-beta Signaling in Regulatory T cells Controls T Helper-17 Cells and Tissue Specific Immune Responses. Immunity. 2017; 18:660-674.

2. Boutet M, Gautheir L, Leclerc M, Gros G, de Montpreville V, Theret N, Donnadieu E, Mami- Chouaib F. TGFbeta Signaling Intersects with CD103 Integrin Signaling to Promote T-Lymphocyte Accumulation and Antitumor Activity in the Lung Tumor Microenvironment. Cancer Res. 2016; 76(7):1757-69.

3. Ling K, Dulphy N, Bahl P, Salio M, Maskell K, Piris J, Warren B, George B, Mortensen N, Cerundolo V. Modulation of CD103 Expression on Human Colon Carcinoma-Specific CTL. J Immunology. 2007; 178:2908-2915.

4. Anz D, Mueller W, Golic M, Kunz W, Rapp M, Koelzer V, Ellermeir J, Ellwart J, Schnurr M, Bourquin C, Endres S. CD103 is a hallmark of tumor infiltrating regulatory T cells. International Journal of Cancer. 2011; 129: 2417-2426.

5. Melssen M, Olson W, Wages N, Capaldo B, Mauldin I, Mahmutovic A, Hutchison C, Melief C, Bullock T, Engelhard V, Slingluff C. Formation and phenotypic characterization of CD49a, CD49b, CD103 expressing CD8 T cell populations in human metastatic melanoma. Oncoimmunology. 2018; 7(10): 10.1080/2162402X.2018.1490855

6. Watanabe R, Gehad A, Yang C, Scott L, Teague J, Schlapbach C, Elco C, Huang V, Matos T, Kupper T, Clark R. Human skin is protected by four functionally and phenotypically discrete populations of resident and recirculating memory T cells. Sci Transl Med. 2015; 7:279ra39

**Ethics Approval**

This study was approved by the UVA IRB-HSR (IRB 10598).

**Table 1 (abstract P545).**
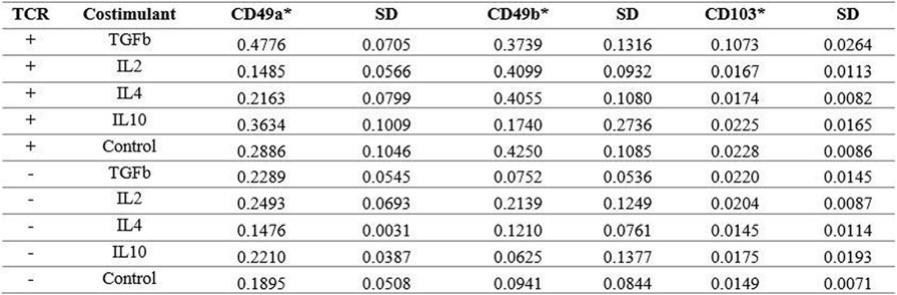
Expression of Integrins by CD4+ T cells

#### P546 Immunoprofiling myeloid-derived suppressor cells in non-small cell lung carcinomas using multiplex immunofluorescence and image analysis approaches

##### Edwin Parra, MD, PhD , Mei Jiang, Cara Haymaker, PhD, Jiexin Zhang, PhD, Tong Li, Carmen Behrens, MD, Chantale Bernatchez, Auriole Tamegnon, Renganayaki Pandurenga, Jack Lee, PhD, John Heymach, Cesar Moran, MD, Ignacio Wistuba, MD

###### MD Anderson Cancer Center, Houston, TX, United States

####### **Correspondence:** Edwin Parra (erparra@mdanderson.org)

**Background**

Myeloid-derived suppressor cells (MDSCs) are a heterogeneous population of cells that consists of myeloid progenitor cells and immature granulocytes or polymorphonucleares (PMN), immature macrophages (M), and immature dendritic cells (DCs). MDSCs play a critical role in tumor-associated immunosuppressive function, which is important in the effective immunotherapies for cancer. The aim of this study was analyze of full range of MDSCs to investigate their role in the microenvironment of non-small cell lung carcinomas (NSCLCs).

**Methods**

TMAs containing 247 patients with stage II/III NSCLCs (adenocarcinomas, ADC=162; squamous cell carcinomas, SCC=85) were analyzed using a multiplex immunofluorescence panel contained CD68, CD11b, Arg-1, CD66b, CD33, CD14, and a pancytokeratin (Figure 1). Densities of different phenotypes and neighbor distances were correlated with clinicopathologic features and outcomes.

**Results**

Using the median density of MDSCs we identified overall higher densities of those cells in SCC than ADC, with significant P value of total granulocytic (CD66b+CD11b+, P=0.016) cells, PMN-MDSCs (CD11b+CD66b+CD33+, P=0.021), and M-MDSCs (CD11b+Agr1+CD14+CD33+, P=0.009, Figure 1 and Table 1). In ADCs the densities of M2 macrophages (CD68+Arg1+CD11b+) were higher in solid pattern than other histologic patterns (acinar, lepidic, papillary, P=0.038); and the total macrophages CD68+ were higher in stage III than in stage I and II (P=0.075), Figure 2. Current smokers was showed higher densities of PMN-MDSC than never-smokers (P=0.031). In addition, EGFR mutant tumors had fewer M2 macrophages, total granulocytic and M-MDSCs than WT patient tumors (P=0.009, P=0.009 and, P=0.043; respectively, Figure 2). A 10μm distant radius from malignant cells compared with the other distant analyzed showed higher densities of CD68+ and CD68+CD11b+ myeloid cells in smaller and EGFR mutant tumors than in larger (P=0.024 and P=0.028; respectively) and WT (P=0.039 and P=0.034; respectively) tumors in ADC. Similarly, higher densities of PMN-MDSCs were observed in stage II tumors than stage I and III in SCC (P=0.029) at this distant. Patients with ADC and a high number of total granulocytic cells (P=0.081) showed worse prognosis than patients with lower total granulocytic cells.

**Conclusions**

While SCCs showed the highest densities of MDSCs as compared to ADCs, our data suggest MDSCs are related with clinicopathologic characteristics that can increase the risk of tumor progression, such as solid pattern, advanced stage, smoking status and prognosis. Additional studies need to be done to understand much better the role of MDSCs in lung cancer.

**Ethics Approval**

The study received approval from the MD Anderson Cancer Center Institutional Ethics Review Board. Tthe Lung Cancer Moon Shot projects through the National Cancer Institute’s Cancer Center Support Grant (P30CA016672), and a grant from the Cancer Prevention and Research Institute of Texas (MIRA-RP160688).

**Table 1 (abstract P546).**
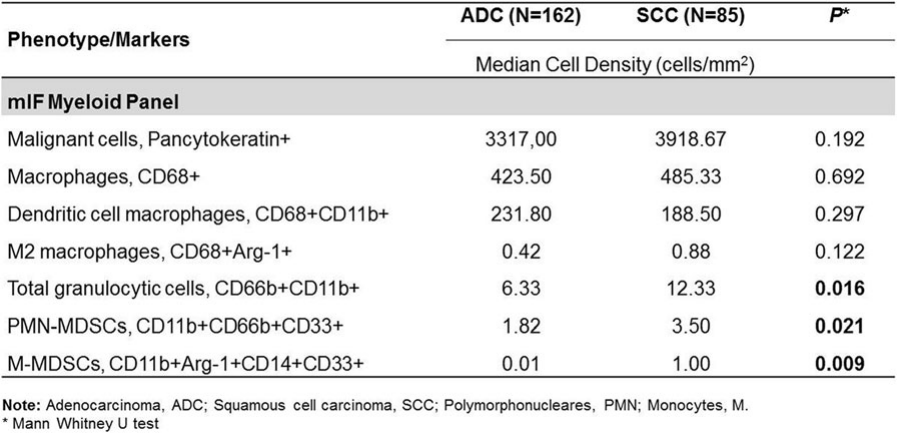
See text for description

**Fig. 1 (abstract P546).**
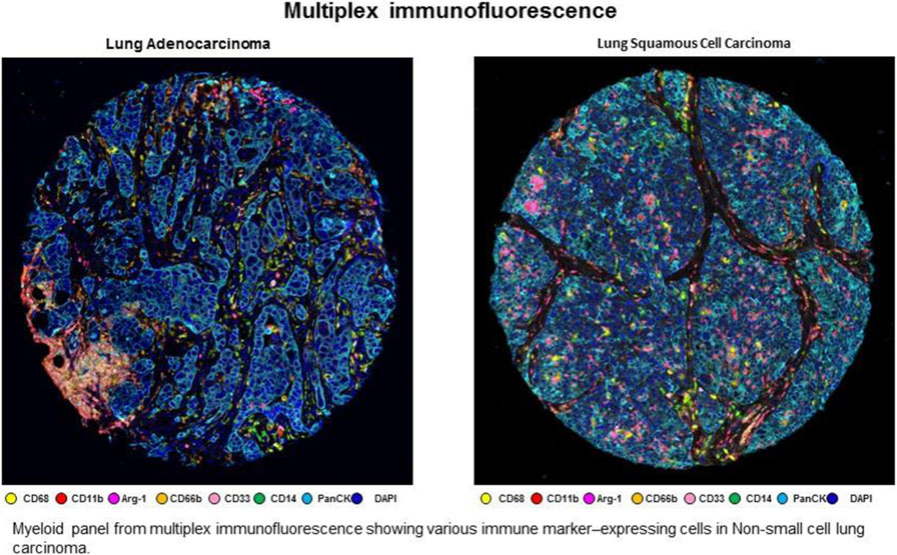
See text for description

**Fig. 2 (abstract P546).**
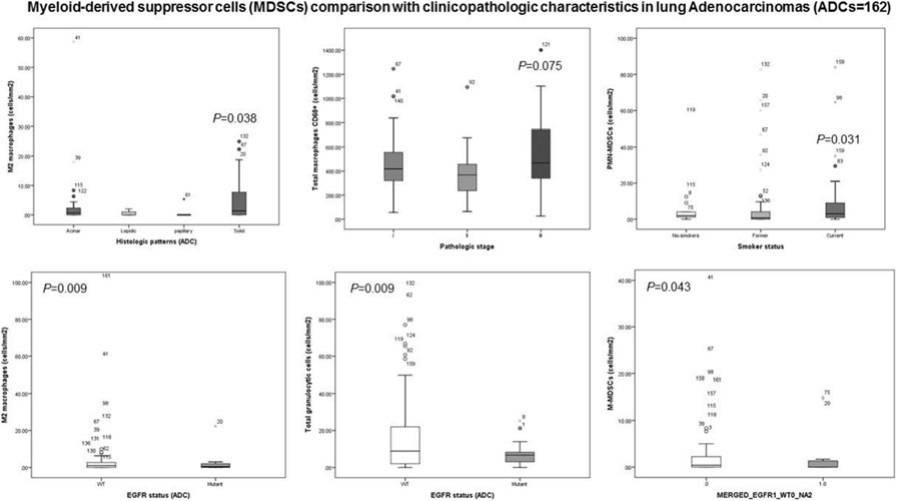
See text for description

#### P547 Distinct B cell signatures and tertiary lymphoid structures are driven by two etiologies in head and neck cancer

##### Ayana Ruffin, MS, Anthony Cillo, PhD, Sayali Onkar, Sheryl Kunning, Robert Ferris, MD, PhD, Dario Vignali, PhD, Tullia Bruno, PhD

###### University of Pittsburgh, Pittsburgh, PA, United States

####### **Correspondence:** Tullia Bruno (tbruno@pitt.edu)

**Background**

Current FDA-approved immunotherapies aim to reinvigorate CD8+ T cells, but the contribution of the humoral arm of the immune response in human cancer remains poorly understood [1,2]. B cells can mediate anti-tumor immunity by presenting antigen , producing tumor-specific antibodies and immunomodulatory cytokines [3-5]. Head and neck squamous cell carcinoma (HNSCC) can be induced by human papillomavirus (HPV+) and carcinogens (HPV-), and the immune infiltrate in the two etiologies has been reported to be distinct, in particular, more B cells in HPV+ HNSCC patients [6]. Further, the presence of B cells in HNSCC correlates with increased patient survival [6]. Our study seeks to differentiate B cell phenotype and function in HPV+ and HPV- HNSCC and identify putative immunotherapeutic targets.

**Methods**

We utilized a multi-level approach to categorize B cells in HNSCC patients. Single cell RNA sequencing (scRNAseq) was performed on CD45+ tumor infiltrating lymphocytes (TIL) and peripheral blood leukocytes (PBL) from HPV+ and HPV- HNSCC patients. Data was analyzed using the Seurat R toolkit in R studio. HNSCC TIL and PBL were stained via multiparameter flow cytometry panel using the Cytek Aurora to allow for unbiased analysis of B cell subsets via computational spectral unmixing. Paraffin embedded slides from HNSCC primary tumors were utilized for single parameter immunohistochemistry of CD20 (n=25 each for HPV + and HPV - disease), and multispectral immunofluorescence was performed on matched tumors from scRNAseq for CD20, CD4, CD8, CD68, PanCK, DAPI and Foxp3.

**Results**

We demonstrated distinct trajectories for B cells in HPV+ and HPV- disease. B cell signatures in HPV- HNSCC patients were predominantly memory B cells and plasma cells, while the signatures in HPV+ HNSCC were naïve and germinal center B cells. We quantified B cells and CD4 T cells in tertiary lymphoid structures (TLS), and the presence of germinal center-rich TLS was associated with HPV+ disease. GC-rich TLS within the tumor bed of HPV+ patients correlated with increased overall survival. Independent of HPV status, we have identified an atypical B cell signature increased in the HNSCC TME compared to healthy and inflamed tonsil tissue with no cancer. This atypical B cell subpopulation is also elevated in the periphery of patients with advanced, metastatic disease.

**Conclusions**

Characterization of B cell phenotype and function in HNSCC is important for devising new therapeutic options for patients. Development of therapeutics to enhance B cell responses in the TME should be prioritized as a compliment to T-cell mediated therapies.

**References**

1.Economopoulou, P., Kotsantis, I. & Psyrri, A. The promise of immunotherapy in head and neck squamous cell carcinoma: combinatorial immunotherapy approaches. ESMO Open 1, e000122 (2016).

2. Yuen, G. J., Demissie, E. & Pillai, S. B lymphocytes and cancer: a love-hate relationship. Trends Cancer 2, 747–757 (2016).

3. León, B., Ballesteros-Tato, A., Misra, R. S., Wojciechowski, W. & Lund, F. E. Unraveling effector functions of B cells during infection: the hidden world beyond antibody production. Infect Disord Drug Targets 12, 213–221 (2012).

4. Bruno, T. C. et al. Antigen-Presenting Intratumoral B Cells Affect CD4+ TIL Phenotypes in Non-Small Cell Lung Cancer Patients. Cancer Immunol Res 5, 898–907 (2017).

5. Germain, C. et al. Presence of B cells in tertiary lymphoid structures is associated with a protective immunity in patients with lung cancer. Am. J. Respir. Crit. Care Med. 189, 832–844 (2014).

6. Wood, O. et al. Gene expression analysis of TIL rich HPV-driven head and neck tumors reveals a distinct B-cell signature when compared to HPV independent tumors. Oncotarget 7, 56781–56797 (2016).

**Ethics Approval**

This study was approved by the local Institutional Review Board under protocol UPCI 99-069, and patients provided informed consent.

#### P548 Beyond a cell metabolite: glutamate receptor signaling improves antitumor T cell immunity

##### Anil Shanker, PhD , Maria Teresa De Aquino, PhD^2^, Thomas Hodo, MS^2^, Roman Uzhachenko, PhD MD^2^

###### *Meharry Medical College School of Medicine, Nashville, TN, United States* ; ^*2*^*Meharry Medical College, Nashville, TN, United States*

####### **Correspondence:** Anil Shanker (ashanker@mmc.edu)

**Background**

The interaction between the central nervous system and immune system underscores an intricate neuro-immune network.

**Methods**

We were intrigued by the expression of glutamate receptors (GluRs), particularly ionotropic GluR3 (AMPAR subtype 3, GluA3) and metabotropic GluR1 on T-lymphocytes, which were upregulated following TCR activation. We blocked group I metabotropic GluRs and AMPA by specific antagonists during T-cell stimulation and analyzed various parameters of T-cell functions in vitro and tumor-bearing mice.

**Results**

We found a delayed CD8+T-cell activation exhibited by downregulated CD25, CD69 and CD44 molecules, resulting in impaired proliferation and cytolytic activity against orthotopic mammary tumors expressing a cognate low-avidity antigen in vivo or specific peptide-loaded target cells in vitro. While GluR+CD8+T-cell frequency ranges about 30% of total CD8+T-cells, treatment with glutamate receptor antagonists profoundly affects T-cell functions. These effects are mediated by transient reduction in phosphorylation of molecules such as Lck/Akt as well as calcium, indicating interaction between TCR and GluR.

**Conclusions**

Data suggest a costimulatory effect of GluRs on T-cell function. Therefore, optimized glutamate agonists may overcome immunosuppression in tumor microenvironment and boost antitumor T-cell therapy.

#### P549 Identifying the host immune response in adult undifferentiated pleomorphic sarcoma

##### Rosanna Wustrack, MD, Evans Shao, Ross Okimoto, Lawrence Fong, MD

###### University of California San Francisco, San Francisco, CA, United States

####### **Correspondence:** Rosanna Wustrack (rwustrack@gmail.com)

**Background**

Undifferentiated Pleomorphic Sarcoma (UPS) is the most common STS subtype in older adults. Despite advancements in systemic treatment for other solid tumors, 5- and 10-survival has remained stagnant for advanced stage UPS. With the development of immune-based therapeutic strategies, it is becoming increasingly clear that the microenvironment plays a critical role in the surveillance and potential elimination of nascent tumor cells [1]. In STS for example, subtype specific effects of checkpoint inhibition were observed in patients with UPS, suggesting that UPS derived antigens modulate tumor-specific adaptive immune responses [2]. While lymphocyte subtypes have been quantified in large panels of STS patients, these studies are hampered by the diverse heterogeneity and relatively small numbers of UPS patients analyzed [3]. We aimed to identify tumor infiltrating immune cell subset and tumor specific antigens in UPS using a robust and well-validated multi-parameter flow cytometry platform and determine if there was any correlation between the immune phenotype and other recognized prognostic factors such as tumor size and depth.

**Methods**

All patients undergoing resection of grade 2 or 3 UPS were eligible for inclusion. We performed single cell multi-parameter flow cytometry using 15 immune-based markers.

tSNE plots were used to demonstrate the relative immune landscape within UPS tumors. The relative frequency of tumor infiltrating lymphocytes were then correlated with clinical outcomes data.

**Results**

From January 1, 2017, to July 31, 2019, 20 resected UPS specimens were collected and successfully immunoprofiled using well-validated multi-parameter flow based cytometry. We identified an increased frequency of CD8+ tumor infiltrating lymphocytes in smaller tumors (8cm) tumors representing 25.82% and 15.01% respectively (Figure 2).

**Conclusions**

We were able to successfully identify the tumor infiltrating immune cell subsets in 20 freshly resected extremity undifferentiated pleomorphic sarcoma specimens. Lymphocytes represented a greater proportion of the immune cell population in smaller tumors compared to larger tumors; specifically, CD8+ T cells were seen at a lower frequency in the tumors greater than 8cm compared to those less than 8cm. As patients with high grade tumors greater than 8cm historically have had worse outcomes, this data shows that the host immune environment may play a role in these clinical outcomes and could be a future target for sarcoma immunotherapy (Figure 3).

**References**

1. Ribatti D. The concept of immune surveillance against tumors. The first theories. Oncotarget. 2017;8(4):7175–7180. doi:10.18632/oncotarget.12739.

2. Tawbi HA, Burgess M, Bolejack V, et al. Pembrolizumab in advanced soft-tissue sarcoma and bone sarcoma (SARC028): a multicentre, two-cohort, single-arm, open-label, phase 2 trial. Lancet Oncology. 2017;18(11):1493–1501. doi:10.1016/S1470-2045(17)30624-1.

3. D'Angelo SP, Shoushtari AN, Agaram NP, et al. Prevalence of tumor-infiltrating lymphocytes and PD-L1 expression in the soft tissue sarcoma microenvironment. 2015;46(3):357–365. doi:10.1016/j.humpath.2014.11.001.

**Ethics Approval**

The study was approved by the UCSF Committee on Human Subject Research, approval number 15-16384

**Fig. 1 (abstract P549).**
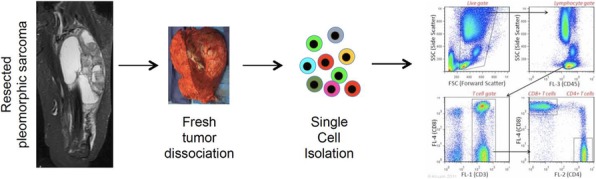
See text for description

**Fig. 2 (abstract P549).**
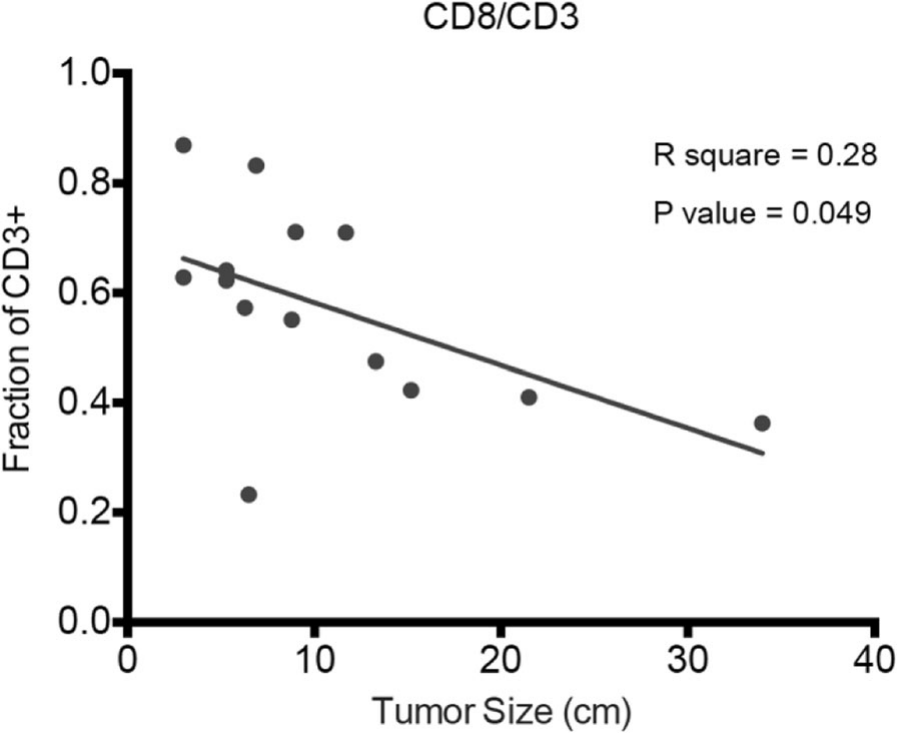
See text for description

**Fig. 3 (abstract P549).**
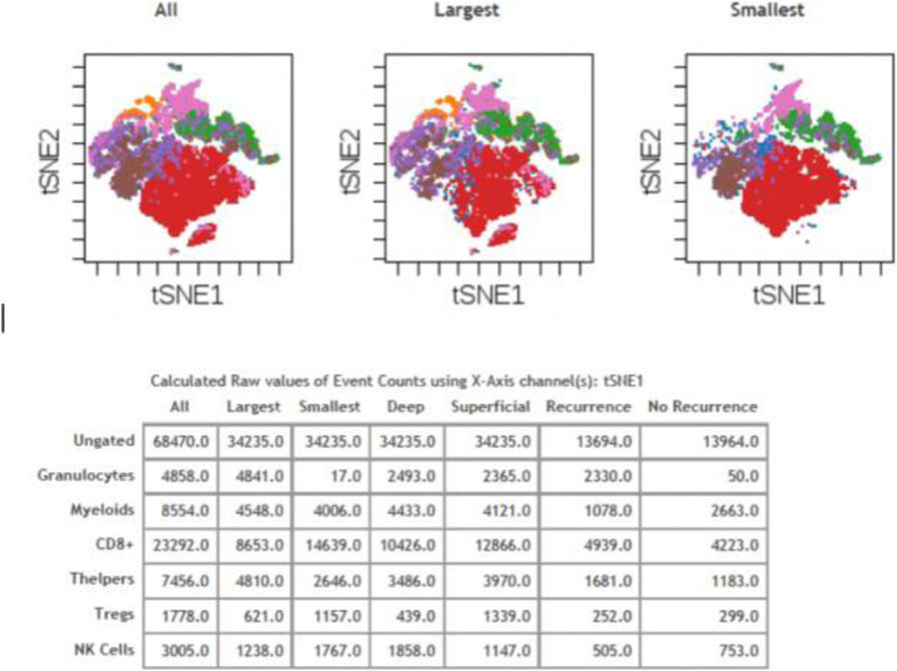
See text for description

#### P550 An Immune-CRISPRomics platform enabling genome-scale and pair-wise combination in vivo T-cell function screens enables comprehensive identification of novel therapeutic targets

##### Isabelle Le Mercier, PhD, Jason Merkin, Sean Keegan, Conor Calnan, Anja Hohmann, PhD, Nick Colletti, Eric Fagerberg, Sol Shenker, Caroline Bullock, Christopher Wrocklage, Noah Tubo, PhD, Tianlei Xu, Matt Noyes, Rami Rahal, Sean Arlauckas, Aria Pearlman Morales, Frank Stegmeier, PhD, Louise Cadzow, Mike Schlabach, PhD, Gregory Kryukov, Micah Benson, PhD

###### KSQ Tx, Lebanon, NH, United States

####### **Correspondence:** Micah Benson (mbenson@ksqtx.com)

**Background**

Although immunotherapy with PD-1/PD-L1 antagonists has significantly advanced patient care, the majority of cancer patients currently do not benefit from checkpoint inhibitor therapies. To identify novel targets for the treatment of PD-1 insensitive cancers, we developed a novel Immune-CRISPRomics platform that enabled genome-wide CRISPR/Cas9 screens in primary T cells in an in vivo setting.

**Methods**

In vivo genome-wide CRISPR/Cas9 screens were conducted by introducing sgRNA libraries into Cas9-expressing TCR-Tg T cells, followed by adoptive transfer into tumor-bearing hosts. Targets enabling enhanced TCR-Tg T cell function in the context of the tumor microenvironment were identified and validated by follow-on efficacy studies.

**Results**

Our CRISPRomics platform identified clinically active molecules, such as PD-1, and also predicted recent clinical failures. In addition, we identified multiple targets that enhanced anti-tumor T-cell function similar to or better than PD-1 as a monotherapy. The anti-tumor activity of targets was assessed across a collection of PD-1 sensitive and PD-1 refractory syngeneic tumor models. One of the targets identified, IO-7, was found to possess robust activity across multiple PD-1 refractory models. We found that inhibition of IO-7 leads to long-term T-cell memory and prevents tumor growth upon re-challenge. Moreover, mechanistic follow-up studies demonstrate that IO-7 inhibition leads to an expansion of central memory T-cell subsets, which have been implicated in driving the durable clinical response of checkpoint inhibitors. We then extended these findings further with an in-vivo double CRISPR screen to examine more than 2,000 pairwise combinations of active sgRNAs in-vivo. These combination studies identified CD8 T-cell gene pairs that when inactivated gave anti-tumor activity that exceeded any single gene inactivation from the primary screen and greatly exceeded the efficacy of PD-1 monotherapy.

**Conclusions**

We describe an Immune-CRISPRomics platform which 1) identified multiple functional targets with therapeutic promise and 2) functionally screened combinations of these targets to reveal the most potent T-cell target combinations for the treatment of PD-1 resistant solid tumors.

#### P551 Bone marrow infiltrating lymphocytes possess a resident memory, stem-like phenotype that may account for enhanced anti-tumor efficacy in multiple myeloma–implications for adoptive T cell therapy

##### Luca Biavati, MD^1^, Megan Heimann, PhD candidate^1^, Elizabeth Zawidzka^1^, Amy Thomas^1^, Ervin Griffin^1^, Danielle Dillard^1^, Bruno Paiva^2^, Ivan Borrello, MD^1^

###### ^1^Johns Hopkins University, Baltimore, MD; ^2^Universidad de Navarra, Pamplona, Spain

####### **Correspondence:** Ivan Borrello (iborrell@jhmi.edu)

**Background**

In multiple myeloma (MM), marrow infiltrating lymphocytes (MILs) have shown increased anti-tumor reactivity and proliferative capacity compared to their peripheral blood counterparts. Despite the recent identification of a core signature for tissue resident memory T cells (TRM), a definitive bone marrow-resident T cell phenotype has yet to be identified.

**Methods**

Here, we used multiparametric flow cytometry to perform single-cell analysis on CD8+ T cells from MM patients to identify those subsets that are enriched in the bone marrow (BM) compared to peripheral blood (PB).

**Results**

The activation and tissue retention marker CD69 identified a population of CD8+ MILs that is virtually absent in PB and displays peculiar phenotypic and functional features compared to its CD69-negative counterpart. Despite the virtual absence of CD103 expression, CD69+ MILs displayed increased levels of CXCR6 and variable levels of CD101, thereby sharing resemblance with the canonical TRM phenotype. Interestingly, CD101 has been associated with both tissue residency and exhaustion. As such, its variable expression may depend on MM burden. CD69+ MILs consistently displayed high levels of the bone marrow homing chemokine receptor, CXCR4 and low/absent expression of the follicular marker, CXCR5. The expression pattern of these extracellular markers further characterizes the BM-resident phenotype and suggests a unique role for the BM microenvironment in anti-tumor immunity. Analysis of exhaustion and senescence markers clearly separated the CD69+ MILs population from both PBL and CD69- T cells because of intermediate expression of exhaustion markers such as TIGIT, TIM3, and PD1, increased CD27, and markedly reduced CD57 levels. This unique phenotype is associated with preserved cytotoxic activity and proliferative capacity. In human T cells, TCF1 and T-bet have a reciprocal gradient of expression. Stem-like features are associated with increased TCF1 and reduced T-bet levels. Interestingly, CD69+ MILs were enriched for TCF1+ and T-bet low T cells suggesting the presence of a stem-like subset in the BM of MM patients. As a whole, CD69+ MILs are distinct from their PB and CD69- counterparts as they uniformly displayed higher levels of TCF1, CD27, CXCR6 and high CXCR4 levels and reduced expression of both CD57 and T-bet.

**Conclusions**

In conclusion, we identified a subset of MILs that appears to display a stem-like/resident phenotype. Studies are ongoing to determine whether this population can be exploited and/or enriched to improve the efficacy of adoptive T cell therapy with in MM, especially in the post-transplant setting where activated MILs therapy has already been proven feasible.

#### P552 A translational platform to assess immunomodulation of the tumor microenvironment

##### Louise Brackenbury, PhD^1^, Robert Nunan^2^, Anna Willox^2^, Michelle Yap^2^, Laura McMillan^2^, Louise Brackenbury^2^, S. Rhiannon Jenkinson, PhD^2^

###### ^*1*^*Charles River Portishead, Bristol, United Kingdom*; ^*2*^*CRL Portishead, Bristol, United Kingdom*

####### **Correspondence:** S. Rhiannon Jenkinson (rhiannon.jenkinson@crl.com)

**Background**

The tumor microenvironment (TME) is a complex network, consisting of the tumor, blood vessels, stromal and immune cells, and soluble factors. The immune system plays an important role in combating tumor growth and various tractable immuno-oncology targets have been identified. We have developed a range of primary human assays to model the TME in vitro, this provides a multi-cellular platform in which to study the mode of action of novel immune-modulators and to identify biomarkers before moving into the clinic. This platform models multiple aspects of anti-tumor immune responses and has been validated with standard of care therapeutics. The assays include: T cell or NK cell-mediated tumor killing, myeloid/macrophage assays, exhausted T cells, Th1/Th17/iTreg differentiation and regulatory T cell suppression assays.

**Methods**

The ability of therapeutics to reverse or inhibit T cell exhaustion was performed using primary human cells. T cell-mediated tumor killing using Pembrolizumab and Ipilimumab (PI) as controls, or 3D tumour cell spheroid ADCC assays (Trastuzumab control) were performed using an IncuCyte. Myeloid/macrophage cells were differentiated from monocytes in tumor-conditioned media (TCM), phenotyped and tested for their phagocytic capability using anti-CD47 as a control. The TME is associated with increased Treg numbers and low frequency of Th1 or Th17 cells. Many therapeutics aim to shift the balance away from Treg, towards Th1 or Th17 cells. Naïve CD4+ T cells were therefore differentiated into iTreg, in the presence or absence of a USP7 inhibitor to examine if iTreg differentiation altered.

**Results**

PI enhanced tumour cell killing in a 2D co-culture assay. Trastuzumab drove enhanced ADCC-mediated killing in a 3D spheroid format. TCM drove the generation of macrophage which had a phenotype similar to tumour associated macrophage, CD25lo, CD127lo, CD184hi, CD80lo, CD163hi, CD68lo and MHCIIlo, produced IL-10 and VEGF and were suppressive. Reduced iTreg generation, without significant alteration in Th1/17 frequency was observed in the presence of the benchmark compound and resulting iTreg were less suppressive compared to non-treated cells. nTreg suppression assays were performed and the USP7 inhibitor had a partial effect.

**Conclusions**

We present an immuno-oncology platform to model the TME in human cells which enables a rapid assessment of the immunomodulatory capacity of therapeutics. The 3D assays represent a more complex system in which to support translational drug discovery, sitting alongside T cell-mediated tumor killing, myeloid/macrophage assays, Th1/Th17/ iTreg differentiation and nTreg assays. Together these assays help to define diverse aspects of the immune response in the TME.

**Ethics Approval**

The study was approved by HRA NRES South West, Bristol (UK), approval number 15/SW/0029.

#### P553 Platelets as immune suppressors in anti-cancer immune responses

##### Ana Micaela Carnaz Simoes, MSc, Morten Holmström, PhD, Mads Andersen, PhD, Per Thor Straten, PhD

###### Center for Cancer Immune Therapy (CCIT), Herlev Hospital, Copenhagen, Denmark

####### **Correspondence:** Ana Micaela Carnaz Simoes (ana.micaela.carnaz.simoes@regionh.dk)

**Background**

Platelets (PLTs) are well-known players during cancer progression. For several cancers, an increased number of circulating PLTs correlates with poor prognosis. Not only can these cells modulate angiogenesis and directly bind cancer cells to facilitate the metastatic process, but they can also protect cancer cells from immune attack by mechanisms that are poorly understood [1,2]. Studies in the autoimmunity field, have shown that PLTs form aggregates with T cells, downregulating T cell activation, proliferation and interferon-ɣ production [3,4]. Nevertheless, no similar studies have been conducted in the context of cancer.

**Methods**

We investigated the presence of circulating PLT-lymphocytes aggregates in solid and haematological cancers. Furthermore, we assessed the effect of PLT binding on T cell proliferation and phenotype. To that purpose, cryopreserved peripheral blood mononuclear cells were analyzed by multicolor flow cytometry and fluorescent microscopy for PLT-T cell aggregates, as well as CD4 and CD8 subpopulations. Lastly, to evaluate the effect PLT binding on T cell anti-tumor reactivity, in vitro cytotoxic response was continuously monitored for 40 hours, using the xCELLigence technology.

**Results**

Our preliminary results show that, compared to healthy donors, cancer patients have an increased number of PLT-lymphocyte aggregates, specially CD8+ T cells. Furthermore, PLTs seem to bind preferentially to a specific T cell phenotype and modulate T cell tumor reactivity. Data will be further discussed in detail at the meeting.

**Conclusions**

These results suggest that binding of PLTs have an impact on T cell activity, which could greatly influence the immune response against tumors. However, further studies are needed to confirm our preliminary results.

**References**

1. Borsig L. The role of platelet activation in tumor metastasis. Expert Rev Anticancer Ther. 2008;8(8):1247-1255. doi:10.1586/14737140.8.8.1247.

2. Bambace NM, Holmes CE. The platelet contribution to cancer progression. J Thromb Haemost. 2011;9(2):237-249. doi:10.1111/j.1538-7836.2010.04131.x.

3. Zamora C, Canto E, Nieto JC, et al. Functional consequences of platelet binding to T lymphocytes in inflammation. J Leukoc Biol. 2013;94(3):521-529. doi:10.1189/jlb.0213074.

4. Zamora C, Cantó E, Nieto JC, et al. Binding of Platelets to Lymphocytes: A Potential Anti-Inflammatory Therapy in Rheumatoid Arthritis. J Immunol. 2017;198(8):3099-3108. doi:10.4049/jimmunol.1601708.

#### P554 Divergent fates of antigen-specific CD8+ T cell clones in mice with acute myeloid leukemia

##### Xiufen Chen, PhD^1^, Blake Flood, BS^1^, Brendan MacNabb^1^, Bruce Blazar, MD^2^, Justin Kline, MD^1^

###### ^*1*^*The University of Chicago, Chicago, IL, United States*; ^*2*^*University of Minnesota, Minneapolis, MN*

####### **Correspondence:** Justin Kline (jkline@medicine.bsd.uchicago.edu)

**Background**

We have previously demonstrated that high-affinity, leukemia-specific CD8+ T cells are efficiently deleted in leukemia-bearing hosts in a manner that depends on cross-presentation of the antigen by splenic Batf3-lineage dendritic cells [1 ,2].

**Methods**

Here, we generated a T cell receptor transgenic mouse strain (Tg101) that expresses TCR-α and -β chains from a CD8+ T cell clone specific for a native H-2Kb-restricted antigen on C1498 leukemia cells.

**Results**

In naïve mice, Tg101 thymocytes predominantly develop along the CD8+ lineage, and exist as naïve CD8+ T cells in peripheral lymphoid organs. Tg101 T cells do not recognize other H-2Kb tumor lines in vitro, suggesting that their cognate antigen may indeed be leukemia-specific. In leukemia-bearing mice, high-affinity 2C CD8+ T cells specific for the H-2Kb-restricted model antigen, SIYRYYGL, rapidly encounter antigen (day 3-4) via its cross-presentation by splenic CD8alpha+ DCs, proliferate briefly, and are largely deleted by day 14. Conversely, antigen encounter by Tg101 CD8+ T cells in mice with C1498 leukemia is delayed (day 7-8), and primarily occurs in the liver - a prominent site of leukemia progression. Subsequently, Tg101 T cells expand, upregulate expression of co-inhibitory receptors (PD-1, LAG-3, TIM-3 and TIGIT), adopt a transcriptional program enriched for upregulation of known genes associated with T cell anergy/exhaustion (Cblb, Egr2/3, Ctla4 Pcdc1, Tox) and produce effector cytokines poorly, all consistent with the acquisition of a dysfunctional state. Antigen-experienced Tg101 cells isolated from leukemia bearing mice fail to expand upon transfer into secondary C1498 cell-challenged mice, suggesting that the dysfunctional state induced in Tg101 T cells becomes irreversible at some point. Surprisingly, antigen encounter by Tg101 T cells in leukemia-bearing mice occurs independently of CD11c+ cells, suggesting that cross-presentation of the Tg101 antigen is not required for in vivo proliferation. Ex vivo cross-presentation assays also fail to demonstrate detectable cross-presentation of the Tg101 antigen by DCs subsets isolated from leukemia-bearing mice. Rather, in vivo Tg101 T cell proliferation is strikingly blunted in mice harboring H-2Kb-/- C1498 AML cells, suggesting that the antigen is directly presented to Tg101 T cells.

**Conclusions**

Collectively, our results reveal that CD8+ T cell tolerance is effectively generated in leukemia-bearing animals. However, the nature of the leukemia antigen and the context in which it is presented are critical factors in dictating the underlying mechanism of tolerance that ensues. A search for the Tg101 antigen and investigation of the epigenetic landscape of naïve versus tolerized Tg101 T cells are ongoing.

**Acknowledgements**

Janet D. Rowley Discovery Fund

**References**

1.Zhang L, Chen X, Liu X, Kline DE, Teague RM, Gajewski TF, Kline J. CD40 ligation reverses T cell tolerance in acute myeloid leukemia. Journal of Clinical Investigation. 2013 May 123: 1999-2010.

2. Kline DE, MacNabb BW, Chen X, Chan WC, Fosco D, Kline J. CD8α+ Dendritic Cells Dictate Leukemia-Specific CD8+ T Cell Fates. J Immunol. 2018 Dec 15;201(12):3759-3769

**Ethics Approval**

The study was approved by The University of Chicago Ethics Board, approval number ACUP 71945.

#### P555 Neuropilin-1 stabilizes human tregs in cancer patients thereby potentiating their suppressive function

##### Christopher Chuckran, BS, Anthony Cillo, PhD, Ashwin Somasundaram, MD, Jessica Moskovitz, MD, John Kirkwood, MD, Francesmary Modugno, PhD, Robert Edwards, MD, Robert Schoen, MD, Robert Ferris, MD, PhD, Tullia Bruno, PhD, Dario Vignali, PhD

###### University of Pittsburgh, Pittsburgh, PA, United States

####### **Correspondence:** Dario Vignali (dvignali@pitt.edu)

**Background**

Regulatory T cells (Tregs) maintain peripheral tolerance;[1] however, in cancer, Tregs dampen anti-tumor immunity, contributing to disease progression.[2,3] Neuropilin-1 (NRP1) is required for intratumoral Treg stability and Treg-specific knockout of NRP1 attenuates tumor growth.[4] Transcriptional analyses revealed that intratumoral NRP1-deficient Foxp3+ Tregs develop an effector phenotype, characterized by interferon-gamma (IFNγ) production and decreased suppression of conventional T cells.[5] Whereas NRP1 is constitutively expressed on mouse Tregs, expression on human Tregs is activation-driven and thus may be modulated by immune processes. Furthermore, the role of NRP1 in maintaining human Treg stability amidst proinflammatory signals is not known. We hypothesize that (1) surface NRP1 expression marks highly suppressive human Tregs, (2) NRP1 expression is driven by proinflammatory signals in the tumor microenvironment, and (3) NRP1 ligation is required for maximal suppressive function.

**Methods**

Phenotypic profiling of peripheral blood (PBL) and tumor infiltrating lymphocytes (TILs) from head and neck squamous cell carcinoma (HNSCC), melanoma, non-small cell lung, ovarian, and colorectal cancer patients was conducted by flow cytometry. Treg function was evaluated in vitro by a micro-scale suppression assay, which measures the ability of Tregs to suppress CD8+ T cell proliferation. Paired with our phenotyping, we cultured Tregs under various stimulatory conditions to query drivers of NRP1 expression. These included numerous cytokine conditions, T cell receptor stimulation, co-culture with antigen presenting cells, as well as blockade of specific costimulation/inhibitory receptors.

**Results**

NRP1+ Tregs are greatly enriched in cancer patient PBL and TIL across numerous malignancies, and the degree of enrichment negatively correlates with disease-free survival in HNSCC. NRP1+ Tregs upregulate a module of functional receptors that mark highly suppressive intratumoral Tregs, including TIGIT, ICOS, and CCR8, as well as a network of TNFRSF members. Correspondingly, NRP1+ Tregs suppress cytotoxic T cell proliferation to a greater degree than their NRP1- counterparts, which can be inhibited using blocking antibodies. NRP1+ Tregs are enriched in vitro in low interleukin-2 (IL-2) conditions as well as upon T cell activation. Proinflammatory cytokine exposure, such as IFNγ, increases NRP1 expression in a subset of cancer patient samples.

**Conclusions**

NRP1+ Tregs constitute a more suppressive human Treg subset based on their enhanced regulatory phenotype and function. Given the increased expression of proliferation and survival markers, especially under destabilizing conditions, NRP1 confers a survival advantage to these Tregs, allowing them to persist and function amidst such signals. Therefore, destabilizing intratumoral Tregs with NRP1 blockade may complement other T cell therapies such as anti-PD1 blockade.

**References**

1. Vignali, D. A. A., Collison, L. W. & Workman, C. J. How regulatory T cells work. Nat. Rev. Immunol. 8, 523–532 (2008).

2. Tanaka, A. & Sakaguchi, S. Regulatory T cells in cancer immunotherapy. Cell Res. 27, 109–118 (2017).

3. Liu, C., Workman, C. J. & Vignali, D. A. A. Targeting regulatory T cells in tumors. FEBS J. 283, 2731–2748 (2016).

4. Delgoffe, G. M., Woo, S.-R., Turnis, M. E., Gravano, D. M., Guy, C., Overacre, A. E., Bettini, M. L., Vogel, P., Finkelstein, D., Bonnevier, J., Workman, C. J. & Vignali, D. A. A. Stability and function of regulatory T cells is maintained by a neuropilin-1–semaphorin-4a axis. Nature 501, 252–256 (2013).

5. Overacre-Delgoffe, A. E., Chikina, M., Dadey, R. E., Yano, H., Brunazzi, E. A., Shayan, G., Horne, W., Moskovitz, J. M., Kolls, J. K., Sander, C., Shuai, Y., Normolle, D. P., Kirkwood, J. M., Ferris, R. L., Delgoffe, G. M., Bruno, T. C., Workman, C. J. & Vignali, D. A. A. Interferon-γ Drives T reg Fragility to Promote Anti-tumor Immunity. Cell 169, 1130–1141.e11 (2017).

**Ethics Approval**

This study was approved by the local Institutional Review Board under protocol UPCI 99-069, and patients provided informed consent.

#### P556 Sex-based differences of the intratumoral immune-infiltrate

##### Fabio Conforti^1^, Laura Pala^1^, Vincenzo Bagnardi^2^, Eleonora Pagan^2^, Giuseppe Viale^1^, Tommaso De Pas, MD^1^, Elisabetta Penacchioli^1^, Paola Queirolo^1^, Emilia Cocorocchio^1^, Pier Francesco Ferrucci, MD^1^, Filippo de Marinis^1^, Adriana Albini^1^, Richard Gelber^1^, Aron Goldhirsch^4^, Fabio Conforti, MD^1^

###### ^*1*^*European Institute of Oncology, Milan, Italy*; ^*2*^*University of Milan-Bicocca,, Milan, Italy* ; ^*4*^*Multimedica, Milan, Italy*

####### **Correspondence:** Fabio Conforti (fabio.conforti@ieo.it)

**Background**

We previously demonstrated that patients’ sex is a variable that influences the response to anticancer immunotherapy: in RCTs comparing immunotherapy-containing regimens to standard treatments in several solid tumors, men obtained larger survival benefit than women when treated with anti-CTLA4 or anti-PD-1 drugs as monotherapy, whereas women experienced larger benefit from anti-PD-1 or PD-L1 combined with chemotherapy.[1,2]

We hypothesized that such heterogeneity of response is due to differences in the molecular mechanisms that govern anticancer immune-response in men and women.

**Methods**

We analyzed 7 public datasets containing data on genome-wide RNA expression analysis of NSCLC samples.[3-9]

We used gene-expression data to estimate the abundance of 64 different cell-types in the microenvironment of each tumor sample, using single sample gene-set enrichment analysis (ssGSEA) according to a previously validated computational algorithm.[10,11]

The Enrichment Scores (ES) of the gene-sets specifically associated with each of the 64 different cell-types were first calculated in tumors of men and women and then compared using a multivariable linear model that adjusted for other covariates including patients’ age, stage at diagnosis, tumor-histotype and smoking status.

Finally, we performed a meta-analysis of the adjusted ES sex-related differences obtained in each single dataset using a random-effects model.

The false discovery rate (FDR) was used to correct for multiple comparisons.

**Results**

2590 tumors were analyzed: 1529 tumors (60%) were from men and 1047 (40%) from women. (table1) The majority of patients (95%) had stage I-III tumors, and did not receive immunotherapy.

Figure. 1 shows results obtained in each dataset as well as the pooled meta-analytic results. (Figure 1)

In the pooled analysis, the immune cell-types found enriched in the tumor microenvironment of women at a FDR cut-off

1)Dendritic cells;

2)CD4+ T-cells (including naive and central memory T-cells);

3)B-cells (including Memory and Class-switched memory B-cells),

4)Eosinophils;

5)Mast-cells;

6)Granulocyte-monocyte progenitor cells (GMP);

Immune-cells found enriched in the tumors of women at FDR cut-off

1)Regulatory T-cells;

2)Gamma-delta T-cells;

3)Natural killer T-cells;

4)Macrophages (both M1 and M2);

5) CD4+ effector memory T-cells;

The only immune cell-type found enriched in the tumor infiltrates of men at FDR

**Conclusions**

We found relevant sex-based differences in the cell-type composition of the immune-infiltrate of NSCLC.

The higher levels of immune-suppressive cells, such as GMP, Macrophage-M2 and Regulatory T-cells observed in tumors of women may explain lower efficacy of anti-CTLA4 or anti-PD1 given as monotherapy, and the need to add chemotherapy to counteract their inhibitory effects.

**References**

1. Conforti F, Pala L, Bagnardi V, et al. Cancer immunotherapy efficacy and patients' sex: a systematic review and meta-analysis. Lancet Oncol. 2018; 19:737-746.

2. Conforti F, Pala L, Bagnardi V, et al. A. Sex-based heterogeneity in response to lung cancer immunotherapy: a systematic review and meta-analysis. J Natl Cancer Inst. 2019 May 20. pii: djz094. doi: 10.1093/jnci/djz094. [Epub ahead of print].

3. Cancer Genome Atlas Research Network. Comprehensive molecular profiling of lung adenocarcinoma. Nature 2014; 511:543-50.

4. Cancer Genome Atlas Research Network. Comprehensive genomic characterization of squamous cell lung cancers. Nature. 2012; 489:519-25.

5. Schabath MB, Welsh EA, Fulp WJ, et al. Differential association of STK11 and TP53 with KRAS mutation-associated gene expression, proliferation and immune surveillance in lung adenocarcinoma. Oncogene 2016; 35:3209-16.

6. Shedden K, Taylor JM, Enkemann SA, et al. Gene expression-based survival prediction in lung adenocarcinoma: a multi-site, blinded validation study. Nat Med. 2008; 14:822-7.

7. Rousseaux S, Debernardi A, Jacquiau B, et al. Ectopic activation of germline and placental genes identifies aggressive metastasis-prone lung cancers. Sci Transl Med. 2013;5:186ra66.

8. Sato M1, Larsen JE, Lee W, et al. Human lung epithelial cells progressed to malignancy through specific oncogenic manipulations Mol Cancer Res. 2013;11:638-50.

9. Noro R, Ishigame T, Walsh N, et al. A Two-Gene Prognostic Classifier for Early-Stage Lung Squamous Cell Carcinoma in Multiple Large-Scale and Geographically Diverse Cohorts. J Thorac Oncol. 2017; 12:65-76.

10. Aran D, Hu Z, and Butte A, xCell: digitally portraying the tissue cellular heterogeneity landscape. Genome Biology. 2017; 18:220.

11. Subramanian A, Tamayo P, Mootha V, et al. Gene set enrichment analysis: A knowledge-based approach for interpreting genome-wide expression profiles. PNAS 2005; 102:15545-15550.

**Table 1 (abstract P556).**
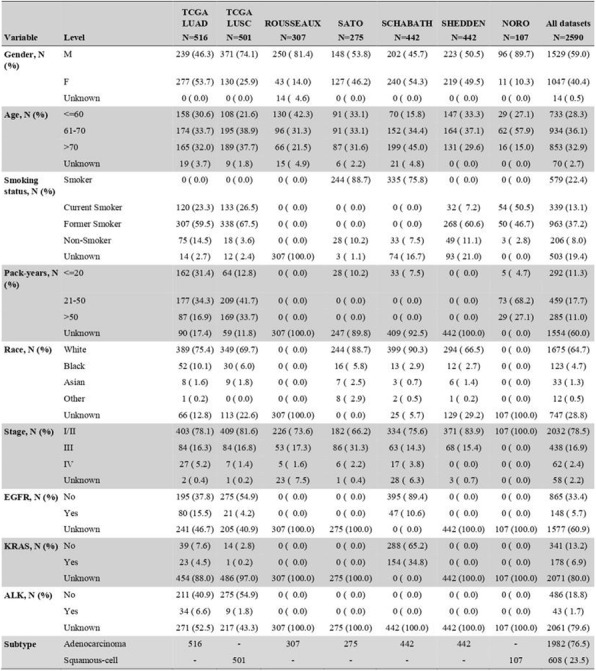
See text for description

**Fig. 1 (abstract P556).**
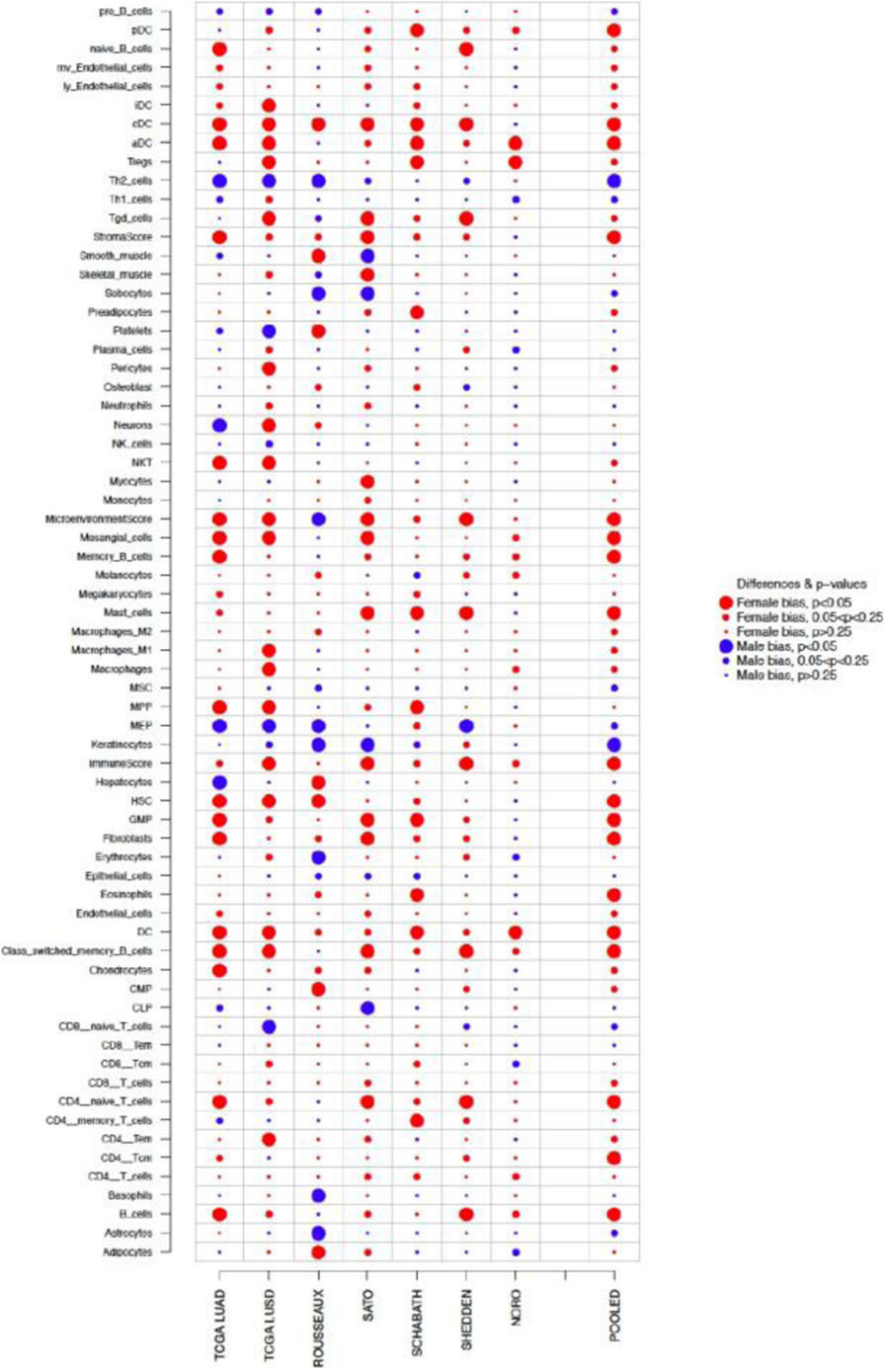
See text for description

#### P557 A2bR contributes to adenosine-mediated immunosuppression, which is relieved by the dual A2aR/A2bR antagonist AB928

##### Daniel DiRenzo, PhD, Kristen Zhang, BS, Sean Cho, PhD, Sachie Marubayashi, Dana Piovesan, MSc, Jesus Banuelos, Joanne Tan, PhD, Akshata Udyavar, PhD, Jenna Jeffrey, PhD, Manmohan Leleti, PhD, Jay Powers, PhD, Matthew Walters, PhD

###### Arcus Biosciences, Inc., Hayward, CA, United States

####### **Correspondence:** Matthew Walters (mwalters@arcusbio.com)

**Background**

The tumor microenvironment (TME) generates high levels of adenosine which binds to A2aR and A2bR receptors on immune cells to inhibit their activity. We have previously shown that AB928, a dual A2aR/A2bR antagonist, blocks the immunosuppressive effects of adenosine in human cultured cells and mouse syngeneic tumors. Herein, we describe studies to assess the contribution of A2bR to adenosine-mediated immunosuppression.

**Methods**

CD14+ monocytes were differentiated with IL-4/GM-CSF for 6 days to generate monocyte-derived dendritic cells (moDC). Cells were taken for NanoString analysis or placed in a mixed lymphocyte reaction (MLR) with CD4+ T cells. PBMCs were activated with CD2/3/28 beads +/- NECA or CGS-21680 for 24 hours and supernatants run on a CXCL5 ELISA.

**Results**

Using publicly-available gene expression databases, we identified that T cells and other non-myeloid cells predominantly express the A2a adenosine receptor. In contrast, most myeloid cells express both A2aR and A2bR with cross-presenting CLEC9A+ dendritic cells (DCs) having the highest levels of A2bR. This pattern of adenosine receptor expression makes DCs a prime target for dual adenosine receptor antagonism in the TME. Experiments in vitro showed that moDC have the highest levels of A2bR expression compared to other cultured cells such as monocytes and macrophages. As expected, adenosine-differentiated moDC showed a decreased ability to stimulate IFN-γ secretion in a MLR. Notably, AB928 attenuated IFN-γ suppression by adenosine and showed significantly greater rescue than a comparable A2aR-specific antagonist. NanoString gene expression profiling identified 39 genes (>2.0 fold change, p <0.05) regulated by adenosine in moDC. These gene expression changes were largely rescued with AB928 but not significantly changed with the A2aR-specific antagonist. Importantly, stimulation of primary human DCs with the dual A2aR/A2bR agonist NECA reproduced several of the adenosine-driven gene expression changes observed in moDC.

To further highlight the importance of A2bR, NECA or the A2aR-specific agonist CGS-21680 were added to human PBMC cultures. Both agonists significantly upregulated the ligand CXCL5 with equivalent rescue by AB928 and the A2aR-specific antagonist when stimulated by CGS-21680. However, upon NECA stimulation, AB928 showed significantly greater inhibition of CXCL5 compared to the A2aR-specific antagonist (86% vs 72% of control, p<0.01), demonstrating that A2bR signaling in PBMCs significantly contributes to gene expression changes.

**Conclusions**

Collectively, these studies demonstrate an important role for A2bR in adenosine-mediated immunosuppression and provide a mechanistic rationale for stimulation of anti-tumor immune responses with the dual adenosine receptor antagonist AB928, which is currently undergoing evaluation in several Phase 1/1b trials.

#### P558 Novel gene expression programs of immune-tumor-stroma identified in inflamed head and neck cancer microenvironment profiling using single cell RNA sequencing (scRNAseq)

##### Cornelius Kürten, MD^1^, Aditi Kulkarni^2^, Lazar Vujanovic, PhD^2^, Xueer Chen^3^, Anthony Cillo, PhD^2^, Xinghua Lu^3^, Robert Ferris, MD, PhD^2^

###### ^*1*^*University of Essen, Essen, Germany*; ^*2*^*UPMC Hillman Cancer Center, Pittsburg, United States*; ^*3*^*University of Pittsburgh*, *Pittsburg, United States*

####### **Correspondence:** Robert Ferris (ferrisrl@upmc.edu)

**Background**

Immunotherapy has significantly advanced HNSCC treatment. However, mechanisms of resistance to immunotherapy mediated by intrinsic and extrinsic factors produced in the Tumor microenvironment (TME) formed by stroma, cancer and immune cells leads to a relatively low response rate of only 15-20%. The emerging technology of single-cell RNA sequencing can be employed to investigate heterogeneous cell populations within the TME by interrogating the transcriptome of individual cells. This allows for transcriptomic mapping of the HNSCC TME at the single cell level.

**Methods**

17 HNSCC specimens were processed for scRNAseq with matched peripheral blood leukocytes (PBL) from treatment-naïve HPV+ and HPV- patients. Following manual and enzymatic tumor dissociations, cell suspensions were sorted into CD45+ (tumor infiltrating leukocytes: TIL) and CD45- (tumor and associated stromal) cells. 10x Genomics 3’ single cell libraries were sequenced on a NextSeq500 (Illumina). Cellular transcriptomes from all samples were aggregated and normalized using the CellRanger pipeline. Downstream bioinformatic analyses were performed using the Scanpy package.

**Results**

We identified 30 different TME cell clusters, with 22 clusters formed by immune cells (PBL and TIL) while CD45- non-immune cells formed 8 clusters. In the immune cell compartment, therapeutically and prognostically relevant cell types were identified (cytotoxic T cells, NK cells, regulatory T cells). Sub-clustering revealed important cell states such as activation, senescence and exhaustion in these immune cell types. Inflamed tumors (n=8) showed a marked increase in the number of tumor-infiltrating CD8+ T cells (43%) versus non-inflamed tumors (17%). Immune checkpoint receptor levels were elevated in CD8+ T cells from inflamed tumors. Novel gene expression signatures representing IFNγ, IFNα and previously described allograft rejection were uniquely associated with these CD8+ T cells, while TNF, apoptosis and hypoxia signaling were predominant in CD8+ T cells from non-inflamed tumors. Modeling putative interactions between immune and fibroblasts, endothelial cells and cancer cells identified potential therapeutic targets to manipulate the immune-stroma-cancer interaction in the TME.

**Conclusions**

Heterogeneous cell populations of immune, stroma and cancer cells can be found in the TME of HNSCC. Transcriptomic characterization using single-cell RNA sequencing allows to identify diverse cell states as well as to model putative cell-to-cell interactions.

**Ethics Approval**

The study was approved by the University of Pittsburgh’s Institutional Review Board (#99-069).

#### P559 Metastatic osteosarcoma utilizes multiple inhibitory checkpoint molecules and immunosuppressive myeloid cells to inhibit the cytotoxic effect of tumor-infiltrating lymphocytes

##### John Ligon, MD, Teniola Oke, Woonyoung Choi, Gady Cojocaru^2^, Ludmila Danilova, Megan Fong, Adam Levin, Daniel Rhee, Carol Morris, Nicholas Siegel, Emily Hsiue, Christian Meyer, David McConkey, Robert Anders, MD, PhD, Drew Pardoll, MD, PhD, Nicolas Llosa, MD

###### Johns Hopkins University SOM, Baltimore, MD, United States; ^*2*^*Compugen, Holon, Israel*

####### **Correspondence:** John Ligon (jligon1@jhmi.edu)

**Background**

Patients with metastatic osteosarcoma (OS) have 5 year overall survival <25% [1]. We aim to understand the immune architecture of the tumor microenvironment (TME) of OS, with the goal of harnessing the immune system as a major therapeutic strategy for the treatment of patients with OS.

**Methods**

Immunohistochemistry (IHC) slides from 66 formalin-fixed paraffin-embedded OS tissue blocks were digitally analyzed. Laser capture microdissection and RNA extraction was performed on 13 OS specimens, and gene expression profiles were obtained utilizing RNA seq and analyzed. Tumor infiltrating lymphocytes (TILs) were isolated from 25 freshly obtained OS specimens and analyzed by multiparameter flow cytometry (MFC).

**Results**

Digital image analysis of IHC demonstrated significantly higher infiltrating immune infiltrating cells in the pulmonary metastases compared to primary bone tumors, particularly at the tumor-normal lung interface. There is increased expression of multiple immune checkpoint molecules at the interface, including programmed cell death 1 (PD-1), T cell immunoglobulin mucin-3 (TIM-3) and lymphocyte activation gene 3 (LAG-3) (Figure 1). Gene expression profiling revealed increased CD8 T cell and resting CD4 memory T cell signature at the interface compared to the tumor interior, and a strong M2 macrophage signature in both regions. Genes related to neutrophil and myeloid cell chemotaxis and known to be associated with polymorphonuclear myeloid-derived suppressor cells (PMN-MDSCs) were shown to be highly expressed in the interface region (Figure 2). MFC revealed that TILs isolated from pulmonary metastases had higher expression of PD-1 and other immune checkpoint molecules, and these TILs were more capable of producing the effector molecules IFN-gamma and granzyme B (Figure 3).

**Conclusions**

Pulmonary metastatic OS lesions are more highly infiltrated with immune cells than primary bone lesions, particularly at the interface. These immune cells are capable of producing cytotoxic cytokines and proteases by MFC, suggesting that these TILs may be tumor-specific and capable of acting against the tumor. The inability of these immune cells to penetrate further into the tumor interior may represent an adaptive immune response by the tumor through a combination of repellent cytokines and inhibition of TILs via upregulation of multiple immune checkpoint molecules and recruitment of PMN-MDSCs. Treatment with a combination of therapies targeting both inhibitory immune checkpoint molecules and the immunosuppressive myeloid compartment may unleash these immune cells and allow them to destroy metastatic OS tumor cells.

**References**

1. Mirabello L, Troisi RJ, Savage SA. Osteosarcoma incidence and survival rates from 1973 to 2004: Data from the surveillance, epidemiology, and end results program. Cancer. 2009;115(7):1531-1543.

**Ethics Approval**

This study was approved by Johns Hopkins University's Ethics Board, approval number FWA00005752.

**Fig. 1 (abstract P559).**
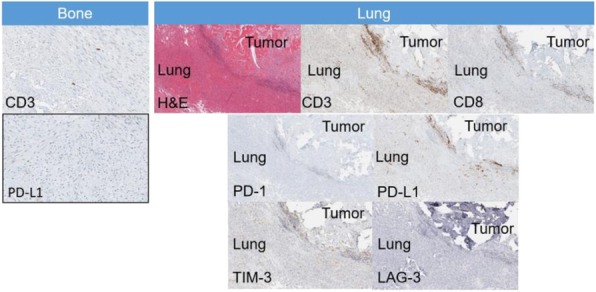
Pulmonary metastases have high infiltration of immune cells

**Fig. 2 (abstract P559).**
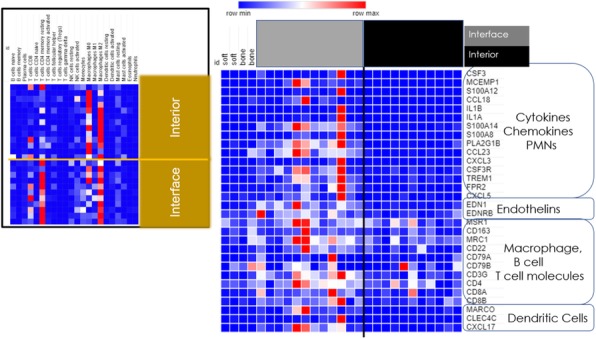
Gene expression analysis of interface compared to interior

**Fig. 3 (abstract P559).**
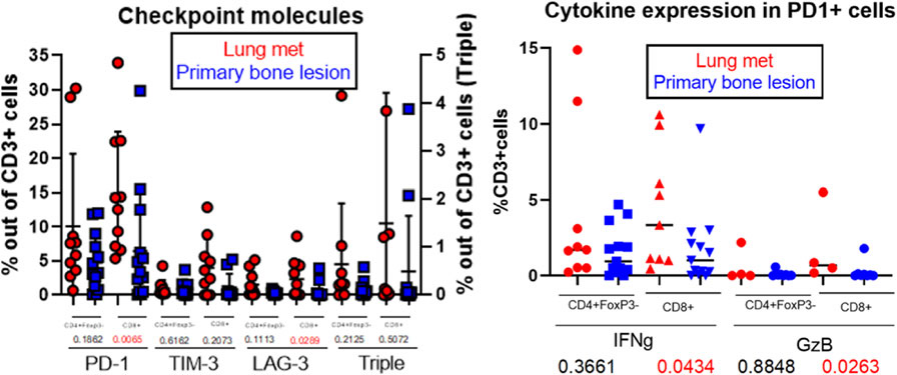
Metastasis TILs express higher checkpoints and cytokines

#### P560 Cancer patients can be categorized by a uniform and mutually exclusive pattern of expression of PD-1 or PD-L1 on their tumor infiltrating T lymphocytes

##### Aurelien Marabelle, MD, PhD, Séverine Mouraud

###### Gustave Roussy, Villejuif, France

####### **Correspondence:** Séverine Mouraud (severine.mouraud@gustaveroussy.fr)

**Background**

We reported that PDL-1 expression on circulating CD8+ T cells was a predictor of bad prognosis and of resistance to CTLA4 blockade in melanoma [1]. PD-L1 expression is currently assessed by IHC stainings and implemented into clinical practice for selecting cancer patients eligible to anti-PD(L)1 immunotherapies. However, PD-L1 IHC stainings have a low sensitivity compared to flow cytometry and fall short in identifying the very nature of PD-L1 expressing cells in the TME. We aimed at better defining the PD-1/PD-L1 expression in the Tumor Micro Environment (TME) by using flow cytometry.

**Methods**

We analyzed 132 fresh tumors by flow cytometry: primitive tumor samples of Urothelial Carcinoma (UC; n=45), liposarcoma (n=19), Ovarian Cancers (OC; n=24), Kidney carcinoma (n=17), Head and Neck Squamous Cell Carcinoma (HNSCC; n=11), Thyroid (n=8) and liver metastases of Colorectal Cancers (CRC; n=8). We evaluated membrane expression of PD-1, PD-L1, TIM-3, LAG-3, MHC-I and MHC-II in the TME using a BD Fortessa X20 and analyzed the data with the Kaluza software.

**Results**

We found a high variability of tumor infiltrating leukocytes across individuals (from 0% to 90% of CD45+ cells) and across tumor types (median range from 4% for CRC metastasis to 20% for OC and NSCLC primitive tumor samples). However, the level of T-cell infiltrates (CD3+) (TILs) was consistently around 60% across histologies with an equal proportion of CD4+ and CD8+ T-cells (except for UC and HNSCC with a CD4/CD8 ratio= 2). Unsupervised analysis of immunophenotyping data could not cluster patients by histological type or by the expression of TIM-3, LAG-3, MHC-I or MHC-II on T cells. More surprisingly, we found that cancer patients could be grouped by clusters with mutually exclusive patterns of PD-1 and PD-L1 expression on T-cells. Indeed, patients were either PD-1POS/PD-L1NEG (18% of patients), PD-1NEG/PD-L1POS (45% of patients), or PD-1NEG/PD-L1NEG (30% of patients) on their tumor-infiltrating T-cells with an identical phenotype between CD4+ and CD8+ T-cells.

**Conclusions**

Patients' TIL are either PD-1POS or PD-L1POS or PD-1NEG/PD-L1NEG. This pattern is uniform across all TILs and mutually exclusive across patients, independently from their tumor histology. This result could have a major implication in terms of anti-tumor activity of anti-PD(L)1 therapies and patient selection for clinical trials and routine practice.

**References**

1. Jacquelot N, Roberti MP, Enot DP, Predictors of responses to immune checkpoint blockade in advanced melanoma. Nat Commun. 2017; 8(1):592.

**Ethics Approval**

All samples were collected in accordance with French Law (MR-004).

#### P561 Formation and function of and CD49b+ CD8 T cells CD49a+ in a murine breast cancer model

##### Marit Melssen, MSc^1^, Robin Lindsay^1^, Amanda Briegel^1^, Anthony Rodriguez^1^, Salwador Cyranowski^1^, Cornelis Melief, MD, PhD^2^, Sjoerd van der Burg^2^, Craig Slingluff, Jr^1^, Victor Engelhard^1^

###### ^1^University of Virginia, Charlottesville, VA, United States; ^*2*^*Leiden University, Leiden, Netherlands*

####### **Correspondence:** Victor Engelhard (vhe@virginia.edu)

**Background**

Integrins CD49a and CD49b can mediate retention of lymphocytes in peripheral tissues by binding to collagens type IV and type I, respectively. Their expression is upregulated on CD8+ tumor infiltrating lymphocytes (TIL) compared to circulating lymphocytes and density of CD49a+ TIL is associated with improved patient outcome. Little is known about how expression of these integrins is regulated in vivo and the functional capacity of the cells expressing them. We hypothesized that CD49a and CD49b expression evolves over time as T cells differentiate in the tumor microenvironment (TME) and that integrin expression is associated with T cell function, by directly supporting effector function or localization.

**Methods**

To address these hypotheses, CD8 TIL from an implantable breast carcinoma model, BRPKp110, were for expression of CD49a, CD49b and functional markers. Where indicated, mice were treated daily with 5ug/mouse FTY720. For transfer experiments, OT1 cells were activated in vivo with ovalbumin, polyIC and anti-CD40 vaccination. After 5d, CD49b+ cells were purified and transferred into tumor-bearing mice.

**Results**

In early stage tumors (d14), T cells were predominantly CD49b single positive (SP) or CD49aCD49b double positive (DP). Later (d23), CD49b SP cells largely disappeared, and CD49a SP cells appeared, while DP cells remained unchanged. After treatment with FTY720, to block further T cell infiltration, the switch from CD49b SP to CD49a SP cells was even more pronounced, suggesting the change is not due to newly infiltrating CD49a SP cells. Regardless of timepoint, CD49b SP cells were antigen-responsive, whereas DP and CD49a SP cells had features of exhaustion. To test whether the switch in integrin expression depends on antigen engagement or environmental factors, we transferred activated CD49b SP OT1 cells into OVA-negative BRPKp110 tumor-bearing mice. In these tumors, CD49b SP cells upregulated CD49a, followed by downregulation of CD49b (Figure 1). Preliminary in vitro experiments showed that restimulation of T cells in presence of CD49a/CD49b ligands, collagen type I or IV, strongly inhibit IFNγ expression, suggesting a potentially direct negative role for CD49a or CD49b in anti-tumor immunity.

**Conclusions**

Together, our data suggest that in CD49b SP CD8 TIL evolve over time to upregulate CD49a, followed by downregulation of CD49b, as TIL gain an exhausted phenotype. Furthermore, the switch in expression is dependent on the microenvironment, not antigen-specific differentiation. Future experiments will assess which environmental factors play a role in CD49a upregulation, and how processes of integrin expression relate to the antigen-dependent generation of exhaustion.

**Fig. 1 (abstract P561).**
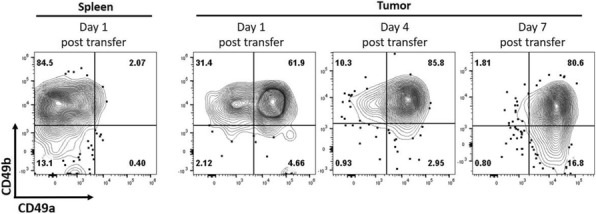
CD49a and CD49b expression on transferred OT1 cells

#### P562 BTN2A, a new immune-checkpoint targeting Vgamma9Vdelta2 T cell cytotoxicity

##### Carla Cano, PhD^1^, Christine Pasero^1^, Aude de Gassart^1^, Sophie Agaugué^1^, René Hoet^1^, Emmanuel Scotet^2^, Erwan Mortier^2^, Agnès Quemeneur^2^, Chirine Rafia^1^, Antoine Briantais^3^, Anne-Charlotte Le Floch^3^, Daniel Olive, MD, PhD,^4^

###### ^*1*^*IMCHECK THERAPEUTICS, Marseille , France*; ^*2*^*INSERM UMR1232 & CNRS ERL6001 CRCINA IR, Nantes, France* ; ^*3*^*Centre de Recherche en Cancerologie de Marseille, Marseille, France;*^*4*^*Centre de Recherche en Cancérologie de Marseille- Institut Paoli Calmettes, Marseille, France*

####### **Correspondence:** Daniel Olive (daniel.olive@imcheck.fr)

**Background**

Anti-tumoral response of Vg9Vd2 T cells requires sensing of phosphoantigens accumulated in malignant cells through binding of butyrophilin 3A(BTN3A). Moreover, an unknown partner located in human Chr6 was shown to be mandatory to BTN3A-mediated Vg9Vd2 T cell activation in murine models. Here, we identified butyrophilin 2A (BTN2A), which is located to Chr6, as a requirement for BTN3A-mediated Vg9Vd2 T cell cytotoxicity against cancer cells.

**Methods**

CRISPR-Cas9-mediated inactivation of BTN2A1/2A2 isoforms was performed in Daudi, K562 and HEK-293T cells. Vg9Vd2 T cells expanded from healthy PBMCs were co-cultured with wild-type or BTN2AKO cells +/- BrHPP(1 μM), HMBPP(0.1 μM) or zoledronate(45 μM), or anti-BTN2A mAb, and Vg9Vd2 T cell degranulation (%CD106αβ+ cells), and intracellular TNFα and IFNγ assessed after 4h. Mouse T cell hybridoma 53/4 expressing TCRVg9Vd2-MOP were co-cultured overnight with NIH3T3 murine fibroblasts transfected with BTN3A- and/or BTN2A-encoding plasmids +/-HMBPP(10 μM), or increasing doses of HMBPP or anti-BTN3 20.1 mAb. BTN2A transcript expression in normal vs. tumoral tissue was analyzed using GEPIA tool. Anti-BTN2A mAb staining was performed on human samples of primary AML, cervical and pancreatic carcinoma and assessed by flow cytometry.

**Results**

Degranulation and intracellular IFNg/TNFa (n=6) were abolished in Vg9Vd2 T cells co-cultured with BTN2AKO Daudi, K562 and HEK-293T cells compared to wild-type, in all conditions tested including anti-BTN3 20.1. Murine cells do not express no BTN2A1 or BTN3A orthologs and are unable to activate human Vg9Vd2 T cells. Ectopic expression of BTN2A and BTN3A combination but neither BTN2A or BTN3A alone in murine NIH3T3 cells, allows triggering of IL-2 secretion in mouse 53/4-TCRVg9Vd2-MOP reporter cells in presence of HMBPP or 20.1 mAb in dose-dependent manner. Anti-BTN2A mAb was able to suppress Vg9Vd2 T cell degranulation/cytokine secretion against cancer cell lines and activation of mouse 53/4-TCRVg9Vd2-MOP reporter by BTN2A/BTN3A-expressing NIH3T3 in a dose-dependent manner. BTN2A transcript was significantly up-regulated in pancreatic, ovarian and cervical carcinoma vs. normal tissue. Extracellular BTN2A protein was detected in primary hematological and solid tumors.

**Conclusions**

Here, we show that BTN2A is mandatory for BTN3A-mediated function in human Vg9Vd2 T cells. Moreover, concomitant BTN2A and BTN3A expression empowers murine T cells with activation through Vg9Vd2 TCR, opening new roads for mouse models of Vg9Vd2 T cell anti-tumoral responses. We describe an anti-BTN2A able to suppress Vg9Vd2 T cell function, and we show BTN2A expression in primary tumors. These results are relevant for understanding Vg9Vd2 T cell antitumoral immunity triggered by phosphoantigens and amino-bisphosphonates.

#### P563 Profiling regulatory and cytotoxic T cell relationships in the tumor microenvironment of non-small cell lung cancer FFPE samples

##### Bonnie Phillips, PhD, Mark Burton, HTL ASCP, Katir Patel, PhD, Mael Manesse, PhD, Sean Downing, PhD

###### Ultivue, Cambridge, MA, United States

####### **Correspondence:** Sean Downing (sean.downing@ultivue.com)

**Background**

Immune cell infiltration and exhaustion has been a key question within the field of immunotherapy research. In particular, regulatory T cells (T-regs) has been identified as one of the cell types responsible for modulation of the immune response. Spatial resolution through multiplex immunofluorescence allows researchers to identify cell types only identifiable through multiple-marker expression and their behavior and relationships with other cells. In this work, we set out to identify three main cell types and their interactions in the tumor microenvironment of non-small cell lung cancer samples: cytotoxic T cells, T-regs, and tumor cells. We used the UltiMapper I/O T-reg panel (CD4, CD8, FoxP3, pan-Cytokeratin) for characterization of cell phenotypes and additional testing to assess the reproducibility of the assay for downstream studies.

**Methods**

Two sets of 5 serial sections from four different tissue sets (one human tonsil and three different NSCLCs) were stained with the UltiMapper I/O T-reg panel (CD4, CD8, FoxP3, pan-Cytokeratin) on the Leica Biosystems BOND RX autostainer. Whole-slide images were acquired using the ZEISS AxioScan.Z1, without the need for linear unmixing. Analysis was performed using Indica Labs HALO® software Indica Labs. Samples were analyzed for immunophenotyping and spatial mapping. Coefficients of variation (CVs) were calculated based on resulting data. All image acquisition and analysis was performed using the same analysis template for each sample type.

**Results**

Detection of single-marker and multi-marker phenotypes were identified. T-regs were identified by CD4+/FoxP3+. Cytotoxic T cells were identified by CD8+. Tumor regions were mapped with pan-Cytokeratin+. Cell population comparisons and proximity analysis was used to compare T-helper:cytotoxic T cell, T-reg:cytotoxic T cell, and cytotoxic T cell:tumor cell ratios. Overall CV’s were less than 15% across all markers across serial sections. This included the total cell counts and average signal intensity.

**Conclusions**

The results presented here indicate that the UltiMapper I/O T-reg panel offers robust and reproducible multiplex immunofluorescence data for the detection and characterization of immune cell infiltration and T-cell regulation. Histological standards for coefficients of variation in IHC based assays are typically

#### P564 How tumor-specific CD8 T cell activation in draining lymph nodes supports the anti-tumor CD8 T cell response

##### Nataliya Prokhnevska, BS, Rajesh Valanparambil, Caroline Jansen, BS, Viraj Master, MD, PhD, Martin Sanda, Haydn Kissick

###### Emory University, Atlanta, GA, United States

####### **Correspondence:** Haydn Kissick (haydn.kissick@emory.edu )

**Background**

CD8 T cells are a critical part of the immune response to tumors. With the increased use of immunotherapies to treat many cancers understanding what leads to highly CD8 T cell infiltrated tumors is crucial in the improvement of these therapies. Especially since the number of CD8 T cells within the tumor has been shown to predict the response to anti-PD1 blockade and survival. Recent work in CD8 T cell immunology described how CD8 T cells respond to chronic diseases, finding two subsets of CD8 T cells within tumors. One is a stem-like CD8 T cell and the other is an effector CD8 T cell with cytotoxic capabilities [1]. Understanding how tumor-specific CD8 T cells activate and differentiate to produce an effective CD8 T cell response with both stem-like CD8 T cells and effector CD8 T cells is crucial to determining why certain tumors are highly infiltrated by CD8 T cells while others are not.

**Methods**

To understand how tumor-specific CD8 T cells respond to prostate cancer we have made a prostate cancer model which expresses the viral LCMV glycoprotein (GP) which acts a tumor-specific antigen. Using this model, we have used this model to study tumor-specific CD8 T cell activation by adoptively transferring LCMV GP specific TCR transgenic P14 CD8 T cells into TRAMPC1-LCMV-GP bearing mice.

**Results**

We have data showing that to have a highly infiltrated there must be a population of stem-like CD8 T cells which are capable to differentiating into effector CD8 T cells and supporting the CD8 T cell response against the tumor. We have found when tumor-specific CD8 T cells are activated they acquire an undifferentiated but activated program, upregulating CD44, PD1 but retaining high TCF1 and CD62L expression (Fig 1, C,E). They also upregulate effector molecules such as CD43 slower and completely fail to upregulate CX3CR1 (Fig 1, C). These undifferentiated activated CD8 T cells do not acquire a typical effector program that is seen in an acute viral infection such as LCMV Armstrong (Fig 1, A,B), and proliferate slower than P14 CD8 T cells activated in LCMV Armstrong (Fig 1, D).

**Conclusions**

Tumor-specific CD8 T cells do not acquire an effector program after activation and instead gain an undifferentiated but activated phenotype. Based on this we hypothesize that tumor-specific CD8 T cells are activated in the TDLN and differentiate to become the stem-like CD8 T cells within the tumor, promoting the anti-tumor CD8 response.

**References**

1. Im, S. J., et al. "Defining Cd8+ T Cells That Provide the Proliferative Burst after Pd-1 Therapy." Nature 537.7620 (2016): 417-21. Print.

**Fig. 1 (abstract P564).**
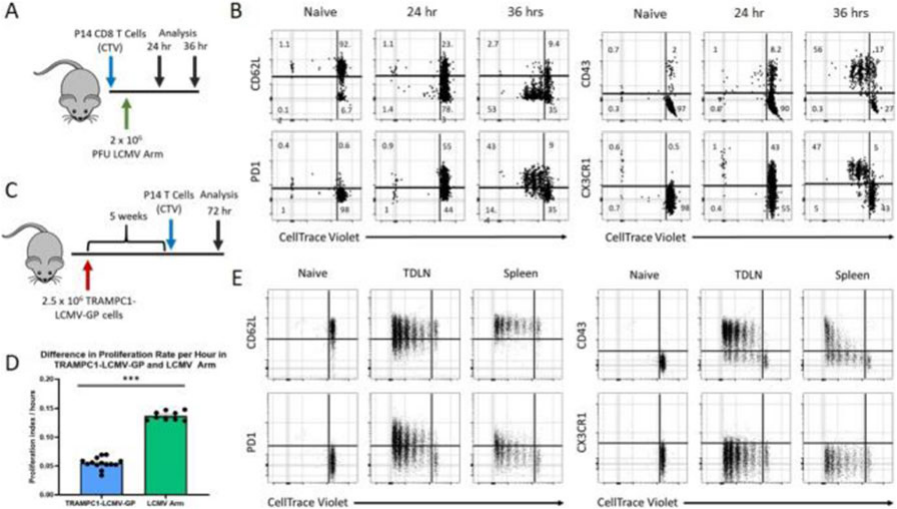
CD8 T cell activation in viral infection and tumor model

#### P565 B cells serve as primary antigen presenting cells for DNA vaccines

##### Ichwaku Rastogi, BE, MS, Douglas McNeel, MD, PhD , Ichwaku Rastogi, BE, MS

###### University of Wisconsin Madison, Madison, WI, United States

####### **Correspondence:** Douglas McNeel (dm3@medicine.wisc.edu)

**Background**

DNA vaccines, relative to other vaccine strategies, are an attractive approach for cancer treatment given their safety, easy manipulation, scalability, stability and economical manufacturing. While a DNA vaccine has been approved for canine melanoma, early phase clinical studies have been generally disappointing [1]. Further studies to understand their mechanism of action, in order to improve their immunogenicity, are therefore needed. We have previously found that passive uptake of DNA by dendritic cells (DC) and macrophages led to degradation, while passive uptake of DNA by B cells led to transcription of encoded genes and ultimately activation of antigen-specific CD8+ T cells.

**Methods**

We used splenic B cells from wild type C57/Bl6 mice, DCs from Flt3L-treated splenocytes from C57/Bl6 mice, CD8 T-cells from OT-1 mice carrying the T-cell receptor specific for the SIINFEKL peptide epitope, and DNA encoding ovalbumin. Antigen-presenting cells (APCs) were treated with different cytokines for in vitro activation and to evaluate antigen presentation following passive uptake of DNA. The activation and proliferation of antigen-specific CD8+ T-cells was then analyzed by flow cytometry and IFN-γ release by ELISA.

**Results**

We found that B cells are the only APC subtype that could take up DNA and transcribe it to mRNA, but they could not present the antigen themselves to CD8+ T cells. B cells require direct contact with DCs to cross present the antigen, and only co-culture of B with DC led to T-cell activation and proliferation. We also found that CD4+ T cells, including antigen-non-specific CD4+ T cells, act as helper cells to promote this presentation, and this function could be replaced by directly providing IL-4.

**Conclusions**

B cells serve as primary antigen-presenting cells for DNA vaccine, but their ability to subsequently activate CD8+ T cells is mediated through DC. Further evaluation of this mechanism may lead to novel vaccine delivery approaches that could improve the immunogenicity of DNA vaccines.

**References**

1. Wahren B. & Liu M. (2014) DNA Vaccines: recent developments and the future. Vaccines 2, 785

#### P566 Does loss of BAP-1 influences the activation of non-canonical NFκB pathway in uveal melanoma with inflammation?

##### Mithalesh Singh^1^, Lata Singh, PhD^2^, Seema Kashyap^3^, Neelam Pushker, MD^3^, Seema Sen, MD^3^, Rachna Meel, MD^3^

###### ^*1*^*AII India Institute of Medical Sciences, New Delhi, India* ; ^*2*^*University of California, Irvine, CA, United States;*^*3*^*Dr.R.P.Centre for ophthalmic Sciences, New Delhi, India*

####### **Correspondence:** Seema Kashyap (dr_skashyap@hotmail.com)

**Background**

Inflammation in Uveal Melanoma (UM) is linked to a bad prognosis. Infiltration with an inflammatory infiltrate increases with disease progression but does not seem to inhibit metastasis. Genetic predictors for metastatic tumor behavior is the loss of BRCA1-associated protein 1 (BAP1) expression. NF-κB is a principal coordinator of innate immunity and inflammation and has emerged as an essential endogenous tumor promoter. We hypothesize that genetic changes not only influence the immunological microenvironment but also drive metastasis in UM and that NC-NFκB proteins are the consequence of a highly-inflammatory profile.

**Methods**

In our study, seventy-five patients are recruited for a period of 4 years (2013-2017). Based on the expression of CD3 (infiltrating lymphocytes) and CD68 (infiltrating macrophages), we divided our study cohort into two categories: UM with inflammation and UM without inflammation. Expression of BAP-1 and NC-NFκB proteins (RelB & p52/NFκB2) was evaluated using immunohistochemistry and western blotting. Real-time PCR was performed on 60 frozen tumor samples. To detect the presence of p52/RelB heterodimer by Co-immunoprecipitation was performed on five each case of the inflammation and non-inflammation group of UM. BAP-1 sequencing was performed on 10 cases.

**Results**

In the inflammation group, activation of NC-NFκB proteins found in 82% and 64% of cases while the loss of BAP-1 was observed in 82% of cases. Loss of BAP-1 protein along with activation of NC-NFκB proteins was seen in the 70% of cases of the inflammation group. Loss of BAP-1 along with activation of C-NFκB proteins was statically significant with inflammatory factors such as CD34+ (p=0.036), IL-6 (p=0.012), LBD>15mm (p=0.031) and epithelioid cell type (p=0.027). Our western blotting results supporting the immunohistochemistry results. In the inflammation group fold-change value of RelB(5.21) & NFκB2 (4.65) genes was reduced to 2.85 (RelB) & 2.34 (NFκB2) gene in the non-inflammation group. Mutation of BAP-1 was more frequently seen in the inflammation group than the non-inflammation group. Loss of BAP-1, along with the activation of NC-NFκB proteins, was associated with reduced metastasis-free survival and overall survival (p

**Conclusions**

Our study showed that in an inflammation group loss of BAP-1 showed the synergistic role with the activation of NC-NFκB proteins and are the poor prognostic indicator of overall and metastasis-free survival. By predicting those patients who have a poor prognosis might help to differentiate which patients require more aggressive local treatments and which of those systemic treatments.

**Ethics Approval**

The study was approved by the institutional ethics committee, All India Institute of Medical Sciences (IESC/T-417/2015).

**Consent**

Written informed consent was obtained from the patient for publication of this abstract and any accompanying images. A copy of the written consent is available for review by the Editor of this journal

#### P567 Characterizing myeloid-derived suppressor cells in the tumor microenvironment of colorectal cancer FFPE samples

##### Angela Vasaturo, PhD, Katir Patel, PhD, Mael Manesse, PhD, Sean Downing, PhD

###### Ultivue, Milan, Italy

####### **Correspondence:** Sean Downing (sean.downing@ultivue.com)

**Background**

Myeloid-derived suppressor cells (MDSCs) are developed to protect the host, and in the context of immuno-oncology these cells protect tumor cells through suppression of immune cell activity. MDSCs are not present in healthy environments, and only rise during pathological conditions associated with inflammation. Two main types of MDSCs have been studied in depth: monocytic MDSCs (M-MDSCs) and polymorphonuclear MDSCs (PMN-MDSCs). M-MDSCs are an intermediate state along the path of differentiation from myeloid cells to monocytes. PMN-MDSCs are an intermediate state along the path of differentiation from myeloid cells to neutrophils. Much of the research into MDSCs has been collected through flow cytometry because of the ability to immunophenotype single cells with multiple markers. Within tissue, research has been limited because of the current multiplex immunohistochemistry technologies’ inability to detect co-expression of multiple markers on single cells and measure a range of expression for dynamic markers. Here we assess the UltiMapper I/O MDSC panel (CD11b, CD14, CD15, HLA-DR) for characterization in a colorectal cancer cohort and for reproducibility studies.

**Methods**

A set of tissue microarrays (TMAs) containing multiple colorectal carcinoma samples was stained with the UltiMapper I/O MDSC kit. Staining was conducted on the Leica Biosystems BOND RX autostainer. Multiplex images were acquired with the ZEISS Axio Scan.Z1 slide scanner. TMAs were analyzed for cell phenotyping and spatial distribution using Indica Labs HALO software. Coefficients of variation (CVs) were calculated based on resulting data.

**Results**

Detection of co-expression of multiple markers in the panel and range of expression for HLA-DR was identified. M-MDSC were identified by CD11b+/CD14+/CD15-/HLA-DR- cells. PMN-MDSCs are CD11b+/CD14-/CD15+/HLA-DR-/low. Overall CV’s were less than 15% across all markers across serial sections. This included total cell counts and average signal intensity. Differences in densities of MDSCs were measured in various colorectal carcinoma samples.

**Conclusions**

Given the robustness and high reproducibility of the UltiMapper I/O MDSC panel, the identification and assessment of MDSC cell types in tissue is reliably attainable. This allow researchers to apply this technology to cohorts of colorectal cancer to assess the utility of measuring MDSCs in the context of immunotherapy-related studies and the overall immunosuppressive landscape.

#### P568 Terminally exhausted CD8+ T cells potentiate the tolerogenic tumor microenvironment as functional suppressors

##### Paolo Vignali, BA, McLane Watson, BS, Ashley Menk, MS, Nicole Scharping, PhD, Kristin DePeaux, BS, Greg Delgoffe, PhD

###### The University of Pittsburgh School of Medicine, Pittsburgh, PA, United States

####### **Correspondence:** Greg Delgoffe (delgoffeg@upmc.edu)

**Background**

Blockade of co-inhibitory ‘checkpoint’ molecules, PD-1 and CTLA-4, has induced impressive clinical responses in advanced tumors; yet only in a subset of patients. Limited success with checkpoint blockade therapy suggests other cell extrinsic or intrinsic mechanisms may be dampening an effective immune response. Cytotoxic CD8+ T cells (CTL) encountering chronic antigen and metabolic restriction can differentiate to a terminally exhausted state (Texh), marked by hyporesponsiveness and metabolic, epigenetic, and transcriptional dysfunction. While enrichment of this population in tumor is a negative prognostic factor, it remains unclear whether Texh are simply non-functional or instead possess tolerogenic or suppressive properties. Transcriptional profiling of tumor-infiltrating CD8+PD-1int (progenitor exhausted) CTL versus CD8+PD-1hiTIM-3+ terminally differentiated Texh reveals that exhausted cells express a pattern of genes associated with immune suppression. We hypothesize that Texh potentiate the suppressive microenvironment of solid tumor by autoregulation and inhibition of local immune responses.

**Methods**

Texh and were isolated from B16 melanoma by expression of inhibitory receptors (IR) and assayed in tandem in microsuppression assay with progenitor exhausted and CD4+FoxP3+ regulatory T cells. Murine melanoma clones with inhibited oxidative metabolism were generated by CRISPR-Cas9 and validated for ablated mitochondrial respiration by extracellular flux analysis. Enforced expression of CD39 in effector T cells was attained by murine retroviral vector delivery. Deletion of CD39 was accomplished through tamoxifen-inducible Cre recombinase expression in CD8+ T cells (E8icre-ERT2Entpd1flox/flox).

**Results**

When sorted directly from tumor, terminally exhausted (PD-1hiTim3+) Texh, but not PD-1 intermediate, progenitor exhausted CD8+ T cells, induce marked suppression of T cell effector responses, comparable to suppression from Foxp3+ Treg cells sorted from the same environment. Suppressive activity correlated with exposure to hypoxia, and exhausted T cells sorted from tumors engineered to be less hypoxic had significantly less suppressive capacity. Our data suggest hypoxia induces HIF1a-dependnet expression of the cell surface ectonucleotidase, CD39, which depletes extracellular ATP, contributes to generation of immunosuppressive adenosine, and has been previously associated with terminal exhaustion. CD39 overexpression on effector T cells can confer a diminished phenotype, and preliminary data using CD8-specific CD39 deletion suggests it confers the regulatory phenotype in tumor-infiltrating exhausted T cells.

**Conclusions**

Our data support a model that as CTL progress to terminal exhaustion, hypoxic exposure upregulates CD39, providing Texh a mechanism to suppress proinflammatory processes. These findings suggest Texh are not solely dysfunctional but are rather deleterious to anti-tumor immunity and may need to be drastically reprogrammed or deleted in order to alleviate immunosuppressive functions.

#### P569 Immune tumor microenvironment dictates clinical outcome of oropharyngeal carcinoma upon standard therapy

##### Marij Schoenmaekers-Welters, PhD, Saskia Santegoets, Chantal Duurland, Vanessa van Ham, Ilina Ehsan, Sjoerd van der Burg, PhD

###### Leiden University Medical Center, Leiden, ZA, Netherlands

####### **Correspondence:** Marij Schoenmaekers-Welters (M.J.P.Schoenmaekers-Welters@lumc.nl)

**Background**

Apart from anogenital cancers, high-risk human papillomavirus type 16 (HPV16) can induce oropharyngeal squamous cell carcinomas (OPSCC). Interestingly, patients with HPV16-induced OPSCC respond better to standard (chemo)radiotherapy compared to HPV-negative OPSCC patients. We previously demonstrated that intratumoral HPV16-specific type 1 T-cell responses contributes to this improved outcome. To further decipher the impact of tumor immune contexture on standard therapy in HPV16-negative and HPV16+ OPSCC we performed in-depth high-dimensional studies.

**Methods**

HPV16 status was determined by GP5+/6+ PCR and p16ink4a staining. Archived formalin-fixed paraffin-embedded tumor tissues were used for multicolor immunofluorescent (IF) staining. Prior to therapy tumor samples were dispersed into single cells and analyzed by CyTOF, flow cytometry and single cell RNAseq. HPV16-specific T-cell reactivity was determined by proliferation and cytokine release using IL-2-expanded tumor-infiltrating lymphocyte (TIL).

**Results**

Intratumoral HPV16-specific T cells were detected in 5/9 HPV16+ and in 0/4 HPV16- OPSCC. The immune cell composition of HPV16+ tumors differed between T-cell non-reactive (IR-) and reactive (IR+) OPSCC as determined by CyTOF analysis. HPV16+IR- OPSCC were strongly infiltrated by B cells while HPV16+IR+ contained mainly T cells with a highly activated (CD38+, HLA-DR+ and/or PD1+) effector memory phenotype and more CD4+CD161+ and CD8+CD103+ T cells. Analysis of 75 HPV16+ OPSCC patients in the TCGA database demonstrated that high expression of CD4 (log-rank p=0.003), CD8 (log-rank p=0.007) or CD161 (log-rank p=0.0007), but not CD103, was associated with better overall survival. Importantly, flow cytometry analysis showed that these intratumoral CD161+ T cells produced higher levels of pro-inflammatory cytokines than their CD161-negative counterparts (p<0.0001).

**Conclusions**

HPV16+ OPSCC patients respond better to standard therapy due to infiltration of highly activated and functional HPV-specific effector memory T cells that express CD161 and/or Tbet into the tumor. These type 1-oriented T-cell inflamed OPSCC were concomitantly infiltrated with Tbet+Tregs, associated with improved survival but may hamper full control of tumor growth.

**Acknowledgements**

This study was financially supported by grants from the Dutch Cancer Society (2014–6696 and 2016–10726).

**Ethics Approval**

This study was approved by the Leiden University Medical Center Ethics Board (P07-112 and P08-197).

#### P570 Assay the interaction of 1000s of individual T cells and antigen-presenting cells for CAR-T or TCR screening in 24hrs

##### Mark White, PhD

###### Berkeley Lights Inc, Emeryville, CA, United States

####### **Correspondence:** Mark White (mark.white@berkeleylights.com)

**Background**

Human T cells represent a highly heterogenous population that can recognize a wide variety of antigens through their T cell receptor. Dissecting how diverse populations of single T cells respond to antigenic stimulus is key to understanding immunity and disease. Berkeley Lights has developed a novel platform to assay the interaction of 1000s of individual T cells and antigen-presenting cells in an automated fashion in 24hrs. The T Cell Phenotype and Functional Analytics workflow enables simultaneous detections of T cell surface markers like CD137 and IFNɣ secretion at the single-cell resolution. Following analysis, individual clones can be selected and recovered alive for downstream expansion or genomic analysis.

**Methods**

On the Lightning, BLI’s proprietary light technology was used to load IFNɣ capture beads into NanoPens with volumes less than 1 nL. Next, single antigen-specific T cells were loaded and followed by antigenic peptide-pulsed T2 cells. In a small subset of the chip, T2 cells were loaded that were pulsed with an irrelevant peptide to serve as a negative control. Following overnight incubation, chips were assayed for IFNɣ on the capture beads and CD137 upregulation on T cells.

**Results**

Linking complex cellular phenotypes to genotype can be applied to a diverse range of cell interactions and applications including CAR construct screening and validation as well as TCR and antigen discovery and validation. In addition, the functional analysis of primary samples like TILs with low cell numbers based on multiple parameters is now possible

#### P571 NGS evaluation of colorectal cancer reveals interferon gamma dependent expression of immune checkpoint genes and novel genes associated with immune suppression

##### Lai Xu, Amy Rosenberg, Lorraine Pelosof

###### FDA, Bethesda, MD, United States

####### **Correspondence:** Amy Rosenberg (amy.rosenberg@FDA.hhs.gov)

**Background**

Colorectal cancers (CRCs) have very low response rates to current immunotherapeutic approaches with the exception of the microsatellite instability high (MSI-H) phenotype. To investigate the basis for such poor responsiveness, we evaluated expression levels of 6 well-known immune checkpoint genes (ICPs), as well as potential novel immune checkpoint related genes (ICPRGs).

**Methods**

The biological connectivity of 6 known ICPs with IFNγ and its downstream genes was examined by NGS in 79 stringently procured and processed CRC and their normal, patient matched intestinal epithelium pairs. Identification of novel IFNγ-induced molecules with potential ICPRG activity was sought.

**Results**

In our study, the 6 “classical” ICPs were statistically upregulated and correlated with IFNγ and 183 additional immune related genes in IFNγ positive (FPKM>1) tumors (Figure 1). By ICP coexpression analysis, we identified 3 IFNγ-induced genes (IFI30, GBP1 and GBP4) as potential novel ICPRGs. These 3 genes were statistically upregulated compared to normal tissue, expressed at relatively high abundance (average FPKM = 115) across 79 CRC pairs compared to the abundance of the 6 ICPs (average FPK = 12; Table 1 ) and correlated with expression of IFNγ and 172 immune-related genes in IFNγ positive tumors (Figure 2). Further evaluation of the TCGA database revealed commonality of IFNγ dependent expression of 6 ICPs and the 3 potential ICPRGs in 638 CRCs, 103 skin cutaneous melanomas (SKCMs), 1105 breast cancers (BCs), 184 esophageal cancers (ESCs), 416 stomach cancers (STCs) and 501 lung squamous carcinomas (LUSC).

**Conclusions**

The expression of higher abundance and novel ICPRG genes, including IFI30, GBP1 and GBP4 requires further evaluation of protein expression levels in tumors because these genes have the potential to be druggable targets for immunotherapy of CRC as well as other tumors.

**Fig. 1 (abstract P571).**
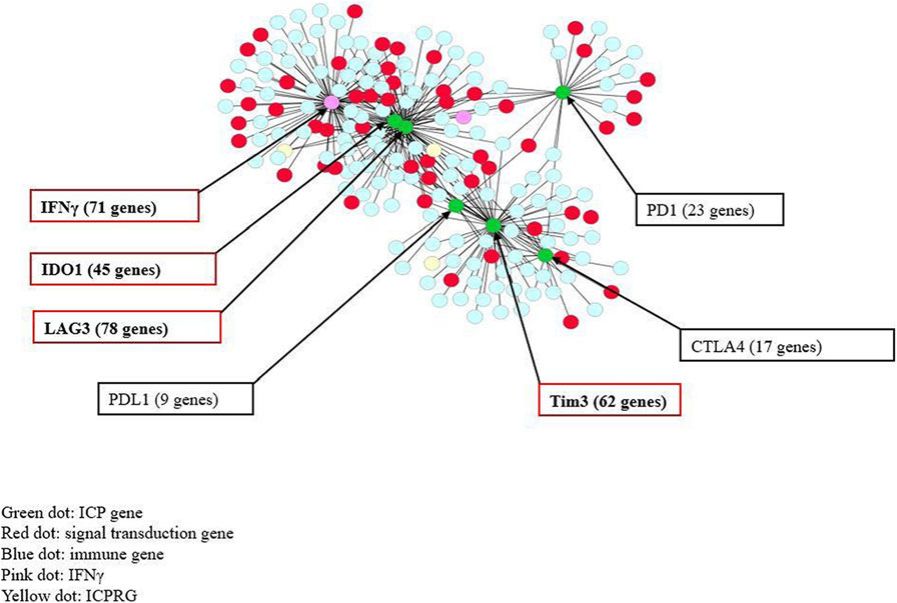
ICP coexpression network

**Table 1 (abstract P571).**
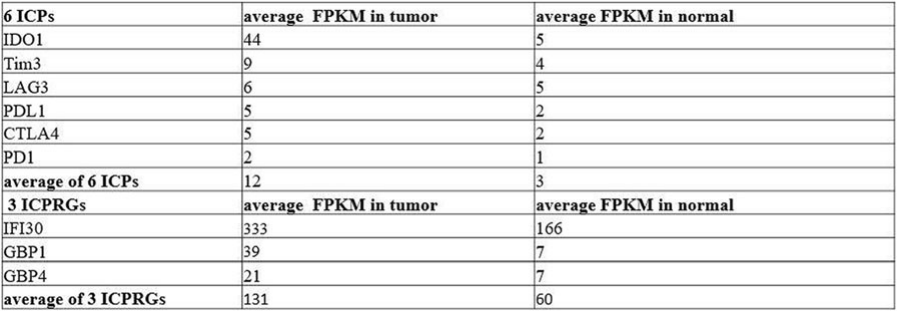
FPKM of 6 ICPs and 3 ICPRGs in 79 CRC pairs

**Fig. 2 (abstract P571).**
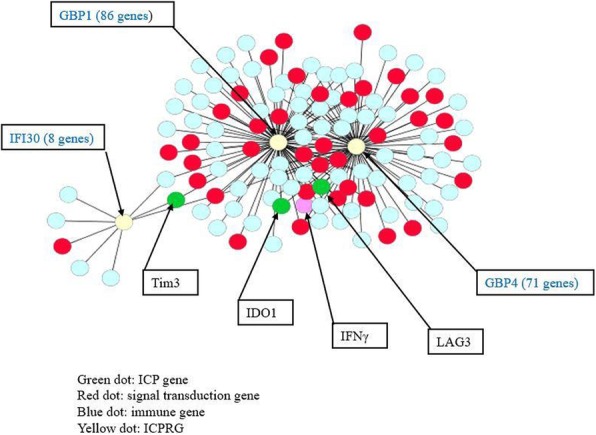
ICPRG coexpression netweork

#### P572 IFNgamma-induced nitric oxide cell death is inhibited by L-arginine depletion in renal cell carcinoma

##### Arnold Zea, PhD^1^, Charity Silvester^1^, Paula Datri, BS^2^

###### ^*1*^*Louisiana State University HSC, New Orleans, LA, United States*; ^*2*^*Louisiana Cancer Research Center, New Orleans, LA, United States*

####### **Correspondence:**Arnold Zea (azea@lsuhsc.edu)

**Background**

In mammals, NO is generated by nitric oxide synthases (NOS 1, 2, 3). All type of NOS catabolizes L-arginine (L-Arg) to produce L-citrulline and nitric oxide (NO) the later known to have anti-tumor activity. L-Arg metabolizes arginase (ARG2) to synthesize polyamines necessary for tumor growth. NOS2 is commonly up-regulated by inflammatory mediators, and it produces NO as long as the molecule is intact and its substrate L-Arg is available. Because ARG2 and NOS2 compete for L-Arg, the metabolism of this amino acid will become a crucial component in modulating cell growth, cell death and resistance in renal cell carcinoma. We hypothesize that L-Arg availability in renal carcinoma cells expressing ARG2 regulates the activity and expression of NOS2 protein as a mechanism that induces resistance against IFNgamma-induced tumor activity.

**Methods**

300,000 Renca cells per well were plated in 6 well plates in RPMI media containing 1,000; 2,000; 4,000 and 6,000 micro-molar of L-Arg and culture for 48h. Then, the cells were stimulated with100U/ml of IFNgamma and cultured for an additional 24 h. At this time, supernatants and cells were harvested and tested for NOS2 protein by immunoblot and NOS2 activity by Greiss Assay (nitrite formation). Supernatants were collected every day during the length of the experiments, to determine L-Arg levels by High-Performance Liquid Chromatography (HPLC). As a control for the experiments, we used CL-19 renal carcinoma cells responsive IFNgamma stimuli.

**Results**

Here, we report that decreased availability of L-Arg in Renca cells blocked the expression of NOS2 protein and NO-induction after stimulation with IFNgamma. Furthermore, the addition of L-Arg to the cell cultures reverted the process. INFgamma receptor, NOS2 gene expression, ARG2 protein expression did not change under the different experimental conditions.

**Conclusions**

The lack of IFNgamma-induced responses could likely be due to the tumor’s ability to develop strategies of tumor escape and define a distinct mechanism by which L-Arg can regulate the activity of its associated NOS2 enzyme. Our plan, include testing if, in this phenomena, the inhibition of NOS2 expression and activity by L-Arg depletion occurs via inhibition of translation of NOS2 mRNA.

**Acknowledgements**

This work is supported by the Stanley S. Scott Cancer Center Funds, and NIH-R25GM121189, NCI-1P20CA202920-01AI grants.

#### P574 Remodeling translation primes CD8+ T cell anti-tumor immunity

##### Jessica Thaxton, PhD, MsCR, Kiley Lawrence, Katie Hurst, Lauren Ball, PhD, Lee Leddy, MD, MsCR, Dongjun Chung, PhD

###### Medical University of South Carolina, Charleston, SC, United States

####### **Correspondence:** Jessica Thaxton (Thaxton@musc.edu)

**Background**

The requisites for translation in T cells are poorly understood and how translation shapes the anti-tumor efficacy of T cells is unknown.

**Methods**

We used multiple innovative tools to dissect how translation shapes T cell lineages and impacts tumor growth. LC-MS/MS proteomics, cytokine conditioning, immunblotting, and a novel FACS-based assay of mRNA translation were employed to elucidate the molecular components that control translation in T cells. A novel in vitro tumor microenvironment assay was developed to measure the impact of the microenvironment on T cell translation. Adoptive transfer of T cells to tumor-bearing mice was employed to elucidate how translation is impacted in T cells in tumors, and fresh tumor infiltrating lymphocytes (TILs) from cancer patients were assessed to measure translation in human TILs. Pharmacological modulation of translation was used to test that remodeling protein synthesis is a new strategy to augment T cell-mediated tumor control.

**Results**

Here we demonstrate that memory T cells are primed by metabolic energy sensor AMP-activated protein kinase (AMPK) to undergo diminished translation relative to effector T cells. However, we show that memory T cells exhibit a remarkable capacity to enhance translation in tumors that effector T cells are unable to duplicate. Study of modulation of translation for applications in cancer immunotherapy revealed that translation is suppressed in CD8 TILs in multiple human tumor types, and that direct ex vivo pharmacological inhibition of the translation elongation step primes powerful T cell anti-tumor immunity by remodeling metabolic properties of T cells.

**Conclusions**

Our work elucidates the new finding that paths to translation shape CD8 T cell anti-tumor capability. We show that metabolic energy sensors shape protein synthesis in T cells and control anti-tumor immunity, and we demonstrate that direct modulation of translation is a powerful and unique strategy to shape the efficacy of T cells to combat tumor growth.

#### P575 Targeting the stress response Kinase GCN2 to restore immunity in the tumor microenvironment

##### Lisa Marshall, MS, Buvana Ravashankar , Lavanya Adysumilli, Deepika Kaveri, Deepa Pookot, Thant Zaw, Raashi Sreenivasan, Mikhail Zibinsky, Jeffrey Jackson, Grant Shibuya, Paul Leger, Anqi Ma, Anton Shakhmin, Andrew Ng, Omar Robles, Oezcan Talay, Delia Bradford, Christophe Colas, Scott Jacobson, Jerick Sanchez, Justy Guagua, Steve Wong, Martin Brovarney, Angela Wadsworth, Goerge Katibah, Gene Cutler, David Wustrow, Paul Kassner, Dirk Brockstedt, Lisa Marshall, MS

###### RAPT Therapeutics, South San Francisco, CA, United States

####### **Correspondence:** Buvana Ravashankar (bravashankar@rapt.com)

**Background**

The tumor microenvironment (TME) is characterized by deficiencies in oxygen and key nutrients, such as glucose and amino acids, resulting in an overall immune suppressive environment. Stromal cells and myeloid-derived suppressor cells (MDSC) within the tumor create a nutrient-poor environment that inhibits immune function and supports tumor growth. GCN2 (general control nonderepressible 2), a stress response kinase, plays a key role in sensing and modulating the response to amino acid deprivation. GCN2 activation in T cells leads to an induction of the integrated stress response pathway and subsequently to T cell anergy and apoptosis.

**Methods**

Culturing primary mouse or human immune cells under low nutrient conditions activates the GCN2 pathway limiting T cell proliferation and function. Treatment of these nutrient-deprived T cells with GCN2i resulted in rescue of CD8+ T cell proliferation and effector functions as measured by flow cytometry. In addition, GCN2 inhibition in MDSC alone fully reversed CD33+MDSC-induced T cell suppression and effector functions. Using murine syngeneic tumor models, we demonstrate that the pharmacologic inhibition of GCN2 in-vivo leads to an observed anti-tumor effect.

**Results**

Furthermore, GCN2 inhibition induced tumor specific immunity by increased antigen-specific T cell frequency and increased cytokine production. Our GCN2i is currently being evaluated to further elucidate the immune contribution in the tumor microenvironment.

**Conclusions**

The GCN2/eIF2α pathway is activated in immune cells during amino acid deprivation, and this induces a functional suppression of the immune response. Our results demonstrate that inhibition of GCN2 is an attractive approach for relieving T cell suppression and promoting anti-tumor activity, demonstrating GCN2 as a promising therapeutic target for the treatment of cancer.

**Ethics Approval**

The study was approved by RAPT Therapeutics Ethics Board, approval number FL0002

#### P576 Tumor-infiltrating regulatory T cell function is metabolically supported through lactate-mediated symbiosis with the tumor microenvironment

##### McLane Watson, BS^1^, Paolo Vignali, BA^1^, Steven Mullet^1^, Natalie Rittenhouse^1^, Ashley Menk, MS^1^, Ryan Whetstone, MS, PhD^1^, Brett Morrison^2^, Jeffrey Rothstein^2^, Amanda Poholek^1^, Stacy Wendell, PhD^1^, Greg Delgoffe, PhD^1^

###### ^1^University Of Pittsburgh, Pittsburgh, PA, United States; ^2^Johns Hopkins, Baltimore, MD, United States

####### **Correspondence:** Paolo Vignali (pdv7@pitt.edu), Greg Delgoffe (gdelgoffe@pitt.edu)

**Background**

Regulatory T (Treg) cells are vital for maintaining immune homeostasis and preventing autoimmunity but represent a major barrier to robust cancer immunity as the tumor microenvironment (TME) actively recruits, activates, and promotes their differentiation. Tumor cells have deregulated cellular metabolism leading to a metabolite-depleted, hypoxic, and acidic TME. Highly glycolytic tumor infiltrating CD8 T cells are in direct competition with the tumor for glucose and oxygen which impairs their effector function. In contrast, Treg cells maintain their high suppressive function within the TME. Further, studies of in vitro induced and directly ex vivo Treg cells reveal a distinct metabolic profile compared to effector T cells. Thus, it may be that the altered metabolic landscape of the TME and the increased activity of intratumoral Treg cells are linked.

**Methods**

Fluorescent glucose tracers, extracellular flux analysis, isotopic flux analysis, and RNA sequencing were used to determine the metabolic profile of murine lymph node and B16 tumor resident Treg cells. Mice harboring a Treg specific deletion of the lactate transporter Slc16a1 (MCT1) were used to determine the in vivo significance of Treg cell lactate uptake.

**Results**

Here we show that Treg cells, especially tumor derived, do not robustly engage in glycolysis or consume glucose. Transcriptomic and functional analysis of glucose consuming Treg cells revealed a diminished Treg cell signature and reduced suppressive capacity. While both glucose low and high consuming Treg cells expressed glycolysis pathway genes, low glucose consuming Treg cells had enriched expression of the lactate transporter Slc16a1 (MCT1) and lactate hydrogenous a (Ldha). Isotopic flux analysis revealed Treg cells take up lactate to generate TCA cycle intermediates and phosphoenolpyruvate (PEP) which we show is critical for intratumoral Treg cell proliferation. Using B16 melanoma bearing mice harboring a Treg specific deletion of lactate transporter MCT1, we demonstrate MCT1 is dispensable for peripheral Treg cell function but required intratumorally resulting in slowed tumor growth and prolonged survival. Analysis of tumor infiltrate demonstrated increased proliferation and cytokine production by intratumoral effector T cells and decreased suppressive capacity of MCT1-deficient intratumoral Treg cells.

**Conclusions**

Taken together these data support a model in which Treg cells are metabolically flexible to utilize ‘alternative’ metabolites to support their function in nutrient poor environments like that of the tumor. Targeting lactate metabolism via MCT1 directed antibodies or inhibitors or lowering tumor lactate levels provides a promising therapeutic strategy to impair intratumoral Treg suppressive capacity and enhance anti-tumor immunity.

**Ethics Approval**

Animal work was done in accordance with the Institutional Animal Care and Use Committee of the University of Pittsburgh (protocol #17071235).

#### P577 HDACi differentially affect distinct T-cell subsets and cytokine production

##### Nisha Holay, BS^1^, Uma Giri^2^, Carla Van Den Berg, PharmD^1^, Gail Eckhardt, MD^2^, Todd Triplett, PhD^1^

###### ^*1*^*University of Texas: Austin, Austin, TX, United States*; ^*2*^*Dell Medical School, Austin, TX, United States*

####### **Correspondence:** Todd Triplett (todd.triplett@gmail.com)

**Background**

The alteration of epigenetic status by HDACs, regulates chromatin structure and transcription by controlling protein access to DNA through the removal of acetyl groups. In addition, HDACs are now known to regulate non-histone proteins as well. Histone deacetylase inhibitors (HDACi) are a class of agents that modulate the acetylation status of histones and non-histone proteins, that are used clinically to treat cancer. Recently, HDACi have been shown to rely on CD8+ T-cells during monotherapy and enhance anti-tumor immune responses when given with immunotherapy. However, this is counterintuitive as majority of early studies have found HDACi are largely immunosuppressive. While, prior HDACi studies on immune function has primarily been on bulk T-cells, various T-cell subsets exist and play diverse roles in immune responses, including naive T-cell activation and immunological memory responses, which may be differentially impacted. Understanding the role of HDACi on T-cell subsets will lead to important biological insights and may aid in the development of future drug design.

**Methods**

Normal PBMCs isolated from healthy individuals were subjected to HDAC Inhibitors with varying specificities: Panobinostat, Entinostat, Vorinostat and OKI-005 and stimulated with PMA/Ionomycin and protein export was blocked with BFA. Cells were fixed and permeabilized after 18 hours and stained with markers: CD4, CD8, CD95, CD31, and CCR7 to delineate naïve and memory subtypes.

**Results**

To determine HDACi effects on T-cells, I evaluated cytokine production on bulk CD4 and CD8 T-cells, and observed a decrease in IL-2, consistent with previous literature that HDACi is negatively impacting T-cells overall. However, TNFa also significantly increased, showing HDACi effects are not only due to cytotoxicity, but cytokines are being differentially affected. Interestingly, the cytokine change effect was across both CD4 and CD8 T-cells and when delineated into naïve versus memory subsets, the naïve T-cells were much more impacted than memory cells, exhibiting more drastic decreases of IL-2 and increases in TNFa. Naïve T-cells also exhibited higher acetylation levels of total acetylated lysine and di-acetyl H3 than memory cells 24 hours after drug treatment.

**Conclusions**

Naïve T-cells comprise a large portion of T-cells from PBMCs, which may explain why previous studies with HDACi show immunosuppressive effects. However, anti-tumor immunity largely relies on tumor infiltrating lymphocytes, which consist of memory T-cells. Thus, memory T-cells’ reduced susceptibility to HDACi could explain enhanced anti-tumor immune responses seen previously. Further investigation into the effect of HDACi on distinct T-cell subsets could aid in rationally designing future immunotherapy strategies in cancer.

**Acknowledgements**

This work was funded by the CPRIT SCHOLAR IN CANCER RESEARCH Grant #RR160093 awarded to Dr. S. Gail Eckhardt. Special thanks to Anna Capasso, Todd Triplett, Carla Van Den Berg, the personnel of the Developmental Therapeutics Lab for their guidance and support and OnKURE Therapeutics.

#### P578 In vitro assessment of the impact of chemotherapy agents on immune cell function

##### Dana Banas, Dawn Stetsko, Miye Jacques, Sium Habte, Matthew Loya, Xiaodong Wang, Krishna Vasudevan, DVM, PhD, Laurence Menard, PhD, Julie Carman, PhD

###### Bristol-Myers Squibb, Princeton, NJ, United States

####### **Correspondence:** Julie Carman (julie.carman@bms.com)

**Background**

Immunotherapy has transformed cancer treatment, providing durable responses for a subset of patients. Improved responses were recently observed for combinations of checkpoint inhibitors with chemotherapy regimens as compared to either therapy alone [1]. Chemotherapeutics are thought to enhance anti-tumor responses by increasing tumor antigens via immunogenic cell death and by modulating the tumor microenvironment. In addition to killing tumor cells, these agents may also directly impact immune cell function. In order to understand the impact of different classes of chemotherapeutic agents on immune cell function, a panel of compounds was profiled using several in vitro functional assays of human cells.

**Methods**

T cells and monocytes were isolated from the whole blood of 6-8 healthy donors. Monocytes were differentiated in vitro to immature dendritic cells by culture with GM-CSF and IL-4 for 7 days. T-cell proliferation was assessed by stimulating isolated T cells with antibodies to CD3 and CD28 for 3 days. Proliferation was measured by uptake of 3H-thymidine. T-cell cytokine production was assessed in an allogeneic mixed lymphocyte culture. Immature dendritic cells were cultured with T cells from HLA-mismatched donors for 5 days. IFNγ in the supernatant was assessed using ELISA. Dendritic-cell maturation was assessed by culturing immature dendritic cells with CD40L overnight. Surface markers (CD80, CD86, CD83, CD32, CD40, HLA-DR, PD-L1) were assessed using flow cytometry.

**Results**

Consistent with their anti-proliferative activity, several classes of chemotherapy agents potently inhibited anti-CD3 plus anti-CD28–driven T-cell proliferation. These included microtubule targeting agents (paclitaxel, docetaxel, eribulin), gemcitabine, and doxorubicin. Alkylating agents (cisplatin, carboplatin, oxaliplatin) and CDK4/6 inhibitors (abemaciclib, palbociclib) had moderate anti-proliferative activity. PARP inhibitors (rucaparib, olaparib), pemetrexed, and fluorouracil each had weak activity. Although pemetrexed and fluorouracil had weak anti-proliferative activity, they did inhibit IFNγ production in the allogeneic mixed lymphocyte culture. Gemcitabine, doxorubicin, CDK4/6 inhibitors, and some of the microtubule targeting agents (paclitaxel, docetaxel) also had strong activity in this assay. Alkylating agents weakly inhibited IFNγ production. In the dendritic-cell maturation assay, eribilin and doxorubicin were the only compounds among those tested that impacted expression of maturation and activation markers.

**Conclusions**

Many chemotherapeutic agents inhibit T-cell proliferation and IFNγ production, with IC50 values in the range of concentrations achieved at therapeutic exposures. These data suggest that several of these agents could also negatively impact T-cell–driven anti-tumor responses. This understanding may help guide treatment selection and scheduling of optimal combination therapeutics with checkpoint inhibitors to broaden anti-tumor responses.

**Acknowledgements**

Bristol-Myers Squibb.

**References**

1. Gandhi L, Rodríguez-Abreu D, Gadgeel S, et al. Pembrolizumab plus chemotherapy in metastatic non-small-cell lung cancer. N Engl J Med. 2018;378:2078-2092.

**Ethics Approval**

The protocol was approved by site institutional review boards or independent ethics committees and conducted according to Good Clinical Practice guidelines, per the International Conference on Harmonisation. Volunteers provided written informed consent based on Declaration of Helsinki principles.

#### P579 Predictive and prognostic relevance of CD8+ tumor-infiltrating lymphocyte density combined with PD-L1 expression in locally advanced rectal cancer patients receiving preoperative chemoradiotherapy

##### Changhoon Song, MD^1^, Yoonjin Kwak^2^, Hye Seung Lee^1^, Sung-Bum Kang^1^, Jae-Sung Kim^1^

###### ^*1*^*Seoul National University Bundang Hospital, Seongnam, Korea, Republic of* ; ^*2*^*Seoul National University Hospital, Seoul, Korea, Republic of*

####### **Correspondence:** Hye Seung Lee (mw9195@snubh.org), Jae-Sung Kim (jskim@snubh.org)

**Background**

The classification of tumor microenvironments based on the presence of tumor infiltrating lymphocytes (TILs) and programmed cell death ligand-1 (PD-L1) expression has been used to predict the efficacy of immune checkpoint blockade in several cancer types. Few studies have evaluated the predictive and prognostic role of CD8 and PD-L1 coexpression in locally advanced rectal cancer.

**Methods**

CD8+ TIL density and tumor PD-L1 expression were assessed by immunohistochemistry using pretreatment biopsies from one-hundred and eighty nine consecutive patients with locally advanced rectal cancer who had preoperative chemoradiotherapy (CRT). Response to CRT was determined based on Dworak tumor regression grade (TRG); tumors with complete (TRG 4) or near-complete (TRG 3) regression were grouped as good responders, and those with TRG 0 to TRG 2 as poor responders.

**Results**

High CD8 TILs showed borderline significant association with better disease-free survival (DFS). Although PD-L1 expression alone was not correlated with DFS, the combination of PD-L1-positive and CD8 low group identified a subtype with poorer DFS. In multivariate analysis, this classification based on combination of CD8 and PD-L1 was independent prognostic factors even when considering the clinical parameters (p =0.007). Among pathologic node-positive patients, high CD8 TILs compared to low were significantly associated with higher rate of good responders (29.2% vs 5.6%, p = 0.023).

**Conclusions**

A combination of tumor PD-L1 expression and low CD8+ TIL density was significantly associated with unfavorable survival in locally advanced rectal cancer patients who underwent preoperative CRT. It is proposed that PD-L1 expression in combination with CD8+ TIL density could be a useful predictive and prognostic biomarker in these patients.

**Ethics Approval**

The study was approved by Seoul National University Bundang Hospital's Ethics Board, approval number B-1707-406-306.

#### P580 Immunogenomic landscape of neuroendocrine prostate cancer

##### Bhavneet Bhinder, MS^1^, Olivier Elemento, PhD^1^, Himisha Beltran, MD^2^ , Alison Ferguson^1^

###### ^1^Weill Cornell Medicine, New York, NY, United States; ^2^Dana Farber Cancer Institute, New York, NY, United States

####### **Correspondence:** Olivier Elemento (ole2001@med.cornell.edu), Himisha Beltran (hip9004@med.cornell.edu)

**Background**

Prostate cancer (PCa) shows a modest clinical benefit from immunotherapy. Neuroendocrine prostate adenocarcinoma (NEPC) is a histological subtype of advanced PCa that arises clonally from castrate resistant prostate adenocarcinoma (CRPC) as a mechanism of resistance, but shares pathological, clinical, and molecular features with small cell lung carcinoma (SCLC). We studied the immunogenomics landscape of NEPC compared to other prostate malignancies and SCLC to identify mechanisms of immune evasion and potential immunological targets in NEPC.

**Methods**

We evaluated RNA-seq data from a 190 patient cohort comprised of benign prostate (n=29), localized prostate adenocarcinoma (PCa; n=68), hormone-naïve metastatic prostate adenocarcinoma (mPCa; n=11), CRPC (n=54), NEPC (n=25) with follow-up data, and SCLC (n=28) [1]. Whole exome sequencing available for 234 cases was used to quantify the mutations and copy number load in the cohort. Unsupervised hierarchal clustering followed by a consensus clustering approach was applied to the expression profiles to determine T-cell immune status for each sample i.e., inflamed or depleted.

**Results**

Immune cluster analysis revealed a predominantly T-cell depleted immune status across prostate subtypes, where in all de novo NEPC cases belonged exclusively to the immune depleted cluster. Poor overall survival (OS) was associated with the inflamed compared to the depleted immune cluster (HR=2.62; p= 0.002; adjusted for subtype). Significantly higher genomic copy number gains were found in T-cell depleted compared to inflamed cases (p= 0.001); however, no difference in total mutation load was seen. Further analysis of NEPC showed a relatively lower expression of cytokines, cytolytic activity, checkpoint, antigen processing machinery, and HLA presentation genes in NEPC (p<0.001).

**Conclusions**

NEPC is characterized by a T-cell depleted tumor microenvironment similar to other metastatic prostate cancer subtypes but with higher PDL1 expression, which is comparable to SCLC. The inverse correlation between OS and infiltration, as well as the novel expression changes in cytokines and checkpoint markers support further investigation into the potential immunological targets for NEPC.

**References**

1. Rudin CM, Durinck S, Stawiski EW, Poirier JT, Modrusan Z, Shames DS, Bergbower EA, Guan Y, Shin J, Guillory J, et al., Comprehensive genomic analysis identifies SOX2 as a frequently amplified gene in small-cell lung cancer. Nat Genet. 2012;44:1111-1116.

#### P581 Identification of prime master regulators for immunologic constant of rejection at pan-cancer scale using network analysis

##### Raghvendra Mall, PhD^1^, Mohamad Saad, PhD^1^, Jessica Roelands, Master^2^, Wouter Hendrickx, PhD^2^, Michele Ceccarelli^3^, Davide Bedognetti, MD, PhD^2^

###### ^1^Hamad Bin Khalifa University, Doha, Qatar; ^2^Sidra Medicine, Doha, Qatar ; ^3^AbbVie Biotherapeutics, Chicago, IL, United States

####### **Correspondence:** Davide Bedognetti (dbedognetti@sidra.org)

**Background**

Network analysis in biology has led to discovery of molecular and genetic interactions, biomarkers of disease, and therapeutic targets. Large-scale biomedical data availability offers a unique opportunity to assess the effect and impact of key cellular determinants for an observed phenotype. In this work, we consider the phenotype associated with the expression of the immunologic constant of rejection (ICR) gene set. This gene set consists of 4 major pathways observed in immune rejection of cancerous tissue, th1 signaling, chemokine production, effector function and immune regulation Higher expression of these genes is associated with tumor infiltrating lymphocytes and an active immune engagement, often correlated with prolonged survival. [1–6]

**Methods**

Consensus clustering of tumor samples based on ICR gene expression divides them into three classes, ICR high, medium and low. The ICR high phenotype is associated with better survival in a subset of cancer types which we refer to as ICR enabled (N=8). In other cancer types we observe the opposite. (ICR Disabled, N=4) [7]

Reverse engineering the gene regulatory networks (TF-target) for each cancer subtype from transcriptional profiles (RNA sequences) of tumors obtained via the cancer genome atlas (TCGA) was performed using a machine learning technique called RGBM. We then perform functional gene set enrichment analysis to discover differential active master regulators.

**Results**

Our aim is to identify key transcription factors (TF) i.e. proteins binding in the regulatory regions of the DNA for specific target genes and regulating their expression, which are differentially activate between ICR High and ICR Low samples which are molecular signatures of hot or cold immune response respectively. We unravel common differentially active TFs or master regulators for these phenotypes over the 12 selected cancer subtypes, where the ICR can be shown to have an impact on survival. We found genes such as IRF1, STAT1, CXC10, CTLA4 to be common master regulators which are positively active for the ICR High phenotype and a validation of our proposed approach as these genes are part of the ICR gene signature. Master regulators, L3MBTL1, HDAC11, SALL2 etc., are positively active for the ICR Low phenotype across the 12 cancers.

**Conclusions**

Downstream analysis of the master regulators specific to the ICR Low phenotype resulted in identification of the NOTCH signaling pathway, transcriptional regulation of oncogene TP53 and several other cancer related signaling pathways that need to be investigated further to understand mechanisms of poor immune response (ICR Low).

**References**

1. Wang, E., Worschech, A. & Marincola, F. M. The immunologic constant of rejection. Trends Immunol 29, 256–62 (2008).

2. Spivey, T. L. et al. Gene expression profiling in acute allograft rejection: challenging the immunologic constant of rejection hypothesis. J Transl Med 9, 174 (2011).

3. Bedognetti, D., Tomei, S., Hendrickx, W., Marincola, F. M. & Wang, E. Toward the Identification of Genetic Determinants of Responsiveness to Cancer Immunotherapy. in Developments in T Cell Based Cancer Immunotherapies (eds. Ascierto, P. A., Stroncek, D. F. & Wang, E.) 99–127 (Springer International Publishing, 2015). doi:10.1007/978-3-319-21167-1_5

4. Bedognetti, D., Hendrickx, W., Ceccarelli, M., Miller, L. D. & Seliger, B. Disentangling the relationship between tumor genetic programs and immune responsiveness. Curr. Opin. Immunol. 39, 150–158 (2016).

5. Hendrickx, W. et al. Identification of genetic determinants of breast cancer immune phenotypes by integrative genome-scale analysis. OncoImmunology 0, 00–00 (2017).

6. Bertucci, F. et al. The immunologic constant of rejection classification refines the prognostic value of conventional prognostic signatures in breast cancer. Br. J. Cancer (2018). doi:10.1038/s41416-018-0309-1

7. Roelands, J. et al. Genomic landscape of tumor-host interactions with differential prognostic and predictive connotations. bioRxiv 546069 (2019). doi:10.1101/546069

#### P582 Characterization of abscopal effect in cryoablation-treated kidney cancer patients

##### Taigo Kato, MD, PhD^1^, Motohide Uemura^1^, Kazuma Kioytani^2^, Norio Nonomura^1^

###### ^1^Osaka University, Osaka, Japan; ^*2*^*Japanese Foundation for Cancer Research, Tokyo, Japan*

####### **Correspondence:** Taigo Kato (kato@uro.med.osaka-u.ac.jp)

**Background**

Cryoablation is one of treatment modalities for kidney cancer and is expected to induce strong local and systemic T cell-mediated immune reactions. So far, in some cases, these immune reactions cause the elimination of distant tumor (abscopal effect). However, the molecular mechanisms of the immune response, particularly for T cells that play fundamental roles in attacking cancer cells, still remain unclear.

**Methods**

In this study, we collected tumor tissues and blood samples from 22 kidney cancer patients, before cryoablation and at 3 months after cryoablation. We applied a next generation sequencing approach to characterize T cell receptor beta (TCRB) repertoires using RNAs isolated from samples.

**Results**

TCRB repertoire analysis revealed expansion of certain T cell clones in tumor tissues by cryoablation. We also found that proportions of abundant TCRB clonotypes (frequency of ≥ 1%) were significantly increased in the post-cryoablation tissues than those of pre-cryoablation tumors. Interestingly, some of these TCRB clonotypes were increased in peripheral blood. Expression analysis of immune-related genes in the tissues of pre- and post-cryoablation showed significantly elevated transcriptional levels of CD8, CD4, GZMA, and CD11c with higher CD8/FOXP3 ratio in the post-cryoablation tissues.

**Conclusions**

Our findings revealed that cryoablation could induce strong immune reactions in tumors with oligoclonal expansion of anti-tumor T cells, which circulate systemically and induce abscopal effect in distant tumor.

#### P583 Building a translational pathway using pharmacodynamic and syngeneic tumour models in conjunction with gene expression to enable the development of cancer immune therapies

##### Louise Brackenbury, PhD^1^, Tommaso Iannitti^2^, Robert Nunan^2^, Louise Harvey, BSc^2^, Clio Andreae, PhD, Bsc (Hons)^2^, Louise Brackenbury^2^, S. Rhiannon Jenkinson, PhD^2^

###### ^1^Charles River Portishead, Bristol, United Kingdom; ^2^CRL Portishead, Bristol, United Kingdom

####### **Correspondence:** S. Rhiannon Jenkinson (rhiannon.jenkinson@crl.com)

**Background**

In order to develop therapeutics which drive the immune system to target tumour cells and eliminate tumour growth, sophisticated in vitro and in vivo models are required. We have developed models which enable us to combine data from human immune cell in vitro assays and murine pharmacodynamic (PD) and syngeneic tumour models to evaluate which pathways a therapeutic is hitting and whether it is effective at inhibiting tumour growth in vivo. We show here the powerful combination of using TCR transgenic T cell adoptive transfer and syngeneic tumour models with flow cytometry and Nanostring profiling of gene expression within the tumour microenvironment (TME) to determine the effect of therapeutic intervention.

**Methods**

To determine the effect of checkpoint inhibitors, mice bearing a defined population of ovalbumin (OVA)-specific T cells were challenged either with antigen (PD model), or an OVA expressing tumour (EG7 or MC38). In the PD model, activation, proliferation and differentiation of these cells into CTL were assessed. Using the OVA-expressing tumour models, comprehensive TIL analysis was carried out to determine how changes to T cell activation impacted on the wider TME and whether a model tumour vaccine could improve T cell responses. Nanostring analysis of gene expression using the murine immune-oncology 360 panel enabled a deeper analysis of the TME and acted as a powerful tool for the identification of potential PD biomarkers. Tumour killing assays were also performed with human cell lines and PBMC using clinical antibodies targeting the same pathways.

**Results**

In the PD model, anti-PD-L1 caused robust upregulation of CD25, IFNγ and granzyme B by antigen-specific CD8+ T cells. Anti-CTLA-4 exerted a similar but less potent effect. Immunisation with peptide increased T cell infiltration in the syngeneic model and this correlated with enhanced T cell functional capacity. Nanostring analysis of tumours saw raised CTL, NK cell and neutrophil gene expression within tumours from peptide immunised mice. Clinical therapeutics targeting PD1 or CTLA-4 drove enhanced tumour killing using human cell-based assays.

**Conclusions**

These models allow testing of novel therapeutics alongside benchmark reagents. The PD model provides a screening platform to assess enhancement of T cell function before moving into syngeneic models. Nanostring gene expression profiling may direct which in vitro assays should be used to map the immunological mechanisms underlying immune modulation. The use of in vitro and in vivo PD/efficacy models better enable assessment of novel cancer immune therapeutics and allow early identification of potential biomarkers.

**Ethics Approval**

The study was approved by HRA NRES South West, Bristol (UK), approval number 15/SW/0029.

#### P584 Functional cytomic targeting of PD-L1 at the surface of living cells to investigate resistance to immunotherapy in multiple myeloma

##### Jolene Bradford, MLS, SCYM(ASCP)^1^, Laura Rico^2^, Jordi Juncà^2^, Jorge Bardina^2^, Àngel Bistué-Rovira^2^, Michael Ward^1^, Jordi Petriz^2^

###### ^*1*^*Thermo Fisher Scientific, Eugene, OR, United States* ; ^*2*^*Josep Carreras Leukaemia Research, Badalona (Barcelona), Spain*

####### **Correspondence:** Jordi Petriz (jpetriz@carrerasresearch.org)

**Background**

Multiple myeloma (MM) is characterized by accumulation of malignant plasma cells in the bone marrow (BM) with an associated immunosuppressive BM microenvironment. Programmed Death-Ligand 1 (PD-L1) has been shown to suppress immune responses through the interaction with Programmed Death-1 (PD-1). In MM, PD-L1 is overexpressed in MM plasma cells and in Myeloid Derived Suppressor Cells (MDSCs). PD-1 is expressed in T-cells. The interaction between PD-L1 and PD-1 decreases TCR-mediated proliferation and cytokine production. PD-L1 plays an important role in tumor immune evasion and drug resistance, and is considered a therapeutic target. However, some MM individuals do not respond to treatments with PD-L1 or PD-1 inhibitors. The aim of this study was to design and evaluate a direct functional screening assay to identify MM MDSCs PD-L1+ using flow cytometry, and its potential use in MM management.

**Methods**

Samples were labeled with PE-PD-L1, APC-CD11b, PE-Cy7-CD33, and FITC-HLA-DR (Invitrogen™ eBioscience) to detect MDSCs using no-lyse no-wash methods. Vybrant™ DyeCycle™ Violet Stain (Thermo Fisher) was used to discriminate nucleated cells from erythrocytes and debris. Samples were analyzed using Attune™ NxT Flow Cytometer (Thermo Fisher). PD-L1 expression was studied in n=35 MM subjects, with and without bone marrow stimulation with PMA (Merck), for 10 minutes at 37ºC. PD-L1 cell surface expression was compared with cytoplasmic expression (n=11 subjects). Kinetics of PD-L1 expression were also studied over time. Competitive experiments in the presence of Durvalumab (0 ng/μL to 250 ng/μL) were used to study its interaction with PD-L1.

**Results**

PD-L1 expression was found dramatically increased after stimulation (n=33 subjects, 94.3%) ranging from 2 to 650 times. PD-L1 cytoplasmic levels were found to be undetectable. PD-L1 expression was found to be higher after 1 to 5 minute stimulation, with a progressive decrease up to 1 hour. Co-incubation with Durvalumab resulted in different inhibitory PD-L1 immunofluorescent profiles.

**Conclusions**

PD-L1 reactivity appears to result from complex interactions that can only be detected with minimal sample perturbation. Since PD-L1 is not found at the cytoplasmic level, PD-L1 may reveal steric changes in response to stimulation, even for a short period of time. These changes may be associated with a PD-L1 immunoregulatory mechanism that may affect therapies targeting the PD-1/PD-L1 checkpoint. Assessment of PD-L1 folding may help to develop better treatment strategies or to predict therapy resistance. No-lyse no-wash methodologies in combination with functional assays show promise as an emerging strategy to model conformational changes in the target site.

**Ethics Approval**

All procedures were performed in accordance with the internal protocols of the laboratory, which were authorized by the HGTiP’s Ethical Committee, in accordance with current Spanish legislation, by the Departament de Medi Ambient i Habitatge (file #1899) of the Autonomous Government of Catalonia (Generalitat de Catalunya).

#### P585 Extracellular matrix gene expression and cytotoxic T lymphocyte infiltration in the tumor microenvironment in non-small cell lung cancer

##### Na Li, PhD, Hongzhe Sun, Xin Wang, Ying Zhou, Zhifu Zhang, Anushka Dikshit, PhD, Courtney Anderson, PhD, Xiao-Jun Ma

###### Advanced Cell Diagnostics, Shanghai, China

####### **Correspondence:** Xiao-Jun Ma (xiao-jun.ma@bio-techne.com)

**Background**

Immunotherapy has proven to be a powerful anti-tumor therapy by harnessing the body’s own immune system to target and kill tumor cells. However, immunotherapy is not successful in all cancer patients due to both intrinsic non-responsiveness and adaptive resistance. Developing predictive biomarkers and understanding mechanisms of resistance are major goals of the immuno-oncology community. The extracellular matrix (ECM), an important factor for promoting tumor growth and metastasis, may also act as a physical barrier to prevent immune cell infiltration and promote tumor immune escape. Components of the ECM such as COL11A1, COL4A1, and LOXL2 have been shown to be associated with cancer progression. Furthermore, new data suggests that TGFβ activation leads to up-regulation of ECM genes in cancer-associated fibroblasts and immune suppression. However, it remains poorly understood which cells in the tumor microenvironment (TME) are the sources of ECM gene expression and how they are related to tumor infiltrating cytotoxic T lymphocytes (CTLs).

**Methods**

In this study, we employed a highly sensitive and specific RNAscope in situ hybridization (ISH) duplex assay to directly visualize the tissue distribution of cells expressing COL4A1, COL11A1, LOXL2, and TGFB1 in relation to tumor infiltrating CTLs in non-small cell lung carcinoma (NSCLC). NSCLC tissue microarrays (TMAs) consisting of 63 independent patient FFPE tumor samples were analyzed using this ISH assay with the following probe combinations: Hs-CD8/Hs-IFNG, Hs-CD4/Hs-FOXP3, Hs-LOXL2/Hs-COL4A1, and Hs-TGFB1/Hs-COL11A1.

**Results**

We observed COL4A1 expression in both tumor and tumor-associated stromal cells in different samples. In contrast, COL11A1 was only expressed in tumor-associated stromal cells. Interestingly, high COL4A1 expression was associated with high CD8+ T cell infiltration, whereas high COL11A1 expression was associated with poor CD8+ T cell infiltration. In addition, tumor expression of TGFB1 was positively correlated with COL11A1 expression. These data depict a complex landscape of ECM gene expression and their relationship to T cell infiltration in the tumor and TME.

**Conclusions**

Taken together, these results demonstrate that the RNAscope assay provides a powerful approach to directly examine the interactions between tumor, ECM, and T cell immune infiltration and offers advantages over immunohistochemistry (IHC) for identifying the cellular sources of secreted proteins such as ECM components in the TME.

#### P586 Comprehensive immune profiling of clinical samples from subjects with advanced recurrent epithelial ovarian cancer treated with a novel T cell activating therapy, DPX-Survivac

##### Brennan Dirk, PhD, Heather Torrey, PhD, Yogesh Bramhecha, PhD, Olga Hrytsenko, PhD, Stéphan Fiset, Marianne Stanford, PhD

###### IMV, Dartmouth, Canada

####### **Correspondence:** Marianne Stanford (mstanford@imv-inc.com)

**Background**

DPX-Survivac is a novel lipid-based formulation designed to elicit de novo cytotoxic T cell response to survivin-expressing tumours. DeCidE1 trial is assessing the clinical activity of DPX-Survivac in combination with low dose intermittent cyclophosphamide with or without epacadostat in advanced ovarian cancer patients. To understand the underlying mechanism of action of this immunotherapy, we performed immune-profiling of PBMC and tumour samples from this study.

**Methods**

Pre-treatment and longitudinally collected on-treatment PBMCs were used to assess survivin-specific T cell response by ex vivo IFN-γ ELISPOT and in vitro MHC multimer staining. Immune-phenotyping was performed on a subset of samples using multi-parametric flow cytometry and tSNE analyses. Pre- and on-treatment tumour biopsies were subjected to whole-transcriptome RNA-sequencing to assess changes in immune infiltration. TCR repertoires were assessed in tumours by TCRb ImmunoSEQ analysis.

**Results**

Treatment with DPX-Survivac induced systemic survivin-specific T cell response in nearly all evaluable subjects as assessed by ELISPOT. On-treatment enrichment in systemic survivin-specific T cells was also detected after in vitro expansion and tetramer analysis, confirming activated T cells are functional and proliferative. Immunophenotyping of PBMC did not show substantial increases in the expression of immunosuppressive markers (CTLA-4, PD-1, B7-H3) within T cell population in subjects that responded to the treatment, further suggesting that induced T cells remained active over time. RNA-profiling of immune cells within the tumour micro environment of subjects demonstrating clinical response revealed on-treatment overexpression of genes related to T cell activation and cytolysis as well as enrichment of B cell and NK cell specific signatures, suggesting the strong potential of treatment to induce tumor-infiltration and activation of cytotoxic cells. Infiltration of T cells into tumor post treatment did not correlate with highest levels of systemic survivin-specific T cells, suggesting migration of circulating activated T cells into tumours. In contrast to tumor infiltration analyses, minimal changes in circulating B cell populations were observed on-treatment. Analysis of TCRβ repertoire in pre- and on-treatment tumours demonstrated that DPX-Survivac therapy can promote proliferation and tumor-infiltration of new T cell clones.

**Conclusions**

DPX-Survivac combinational therapy induces robust and sustained survivin-specific responses and promotes T cell infiltration of tumours, without a loss in functionality. The infiltration immune cells beyond T cells has been demonstrated in tumor tissue yet was not consistently detected in PBMCs; a finding that emphasizes the need for immune-profiling of both blood and tumor to gain a full understanding of the mechanism of action of novel immunotherapies and combinations.

**Ethics Approval**

The study was approved by Dalhousie University's research ethics board, approval number 2018-4659

#### P587 Collagen density regulates the activity of tumor-infiltrating T cells

##### Dorota Kuczek, MSc^1^, Anne Mette Hvid Larsen, MSc^1^, Marco Carretta, PhD^1^, Adrija Kalvisa, MSc^2^, Majken Siersbæk^2^, Ana Micaela Carnaz Simoes, MSc^1^, Anne Roslind^1^, Lars Engelholm^3^, Marco Donia, MD, PhD^1^, Inge Marie Svane, MD^1^, Per Straten^1^, Lars Grøntved^2^, Daniel Hargboel Madsen^1^

###### ^*1*^*Copenhagen University Hospital Herlev, Herlev, Denmark*; ^*2*^*University of Southern Denmark, Odense, Denmark*; ^*3*^*University of Copenhagen, Copenhagen, Denmark*

####### **Correspondence:** Daniel Hargboel Madsen (daniel.hargboel.madsen@regionh.dk)

**Background**

Tumor progression is accompanied by extensive remodeling of the surrounding extracellular matrix leading to the formation of a tumor-specific ECM, which is often more collagen-rich and of increased stiffness. High collagen-density within tumors is known to promote tumor progression, however, it is unknown if these pro-tumorigenic effects involve modulation of T cell activity. To investigate if a high-density tumor-specific ECM could influence the ability of T cells to kill cancer cells, we studied how T cells respond to 3D culture in different collagen densities.

**Methods**

T cells cultured in 3D conditions surrounded by a high or low collagen density were imaged using confocal fluorescent microscopy, and the effects of the collagen density on T cell survival, proliferation and differentiation were examined using flow cytometry. Similarly, cancer cell proliferation in 3D was also evaluated. Using immunohistochemistry, triple-negative breast cancer specimens were analyzed for the number of infiltrating CD8+ T cells and for the collagen density. The influence of collagen density on T cell cytotoxicity was examined using chromium-51 release assay. Whole-transcriptome analyses were used to study in detail the effects of collagen density on T cells and computational analysis was used to identify transcription factors involved in the collagen density-induced gene regulation. Observed changes were confirmed using qRT-PCR analysis.

**Results**

Using 3D collagen gels of varying density, we identified that T cells respond to their ECM environment and that T cell proliferation is significantly reduced in a high-density matrix, while cancer cell proliferation is unaffected. Consistently, the number of infiltrating CD8+ T cells in mammary tumors with high collagen-density was reduced, indicating it can play a role in regulating T cell abundance in human breast cancer. Additionally, whole-transcriptome analysis of 3D-cultured T cells revealed distinct transcriptional profiles depending on the surrounding collagen-density. Specifically, a high-density matrix induced downregulation of cytotoxic activity markers and upregulation of regulatory T cell markers. Importantly, these transcriptional changes were accompanied by an impaired ability of tumor-infiltrating T cells cultured in a high-density matrix to kill autologous melanoma cells.

**Conclusions**

Our study identifies a new immune modulatory mechanism, which could be essential for suppression of T cell activity in the tumor microenvironment.

#### P588 Development of in vitro immune effector function assays to better approximate the in vivo behavior of biotherapeutics and cell therapies

##### Chris Langsdorf, BS

###### Thermo Fisher Scientific, Eugene, OR, United States

####### **Correspondence:** Chris Langsdorf (chris.langsdorf@thermofisher.com)

**Background**

With continued growth in development and approval of biologic drugs and cell therapies, comes a need for more robust and reliable cell-based assays and analysis systems. Here we describe new methods to assess the specific killing of target cells by antibody-mediated complement-dependent cytotoxicity (CDC), antibody-dependent cellular cytotoxicity (ADCC) by natural killer cells, T cell killing, and antibody-dependent cellular phagocytosis (ADCP).

**Methods**

These methods are demonstrated with single-cell analysis models compatible with flow cytometry and image-based analysis. Additionally, we have transitioned these assays into more biologically-relevant models such as tumor spheroids and whole blood. T and NK cell penetration and killing of breast and lung cancer spheroids was evaluated using quantitative confocal microscopy. Ramos Burkitt's lymphoma cells were opsonized with Rituximab, spiked into human whole blood, and complement- and NK-dependent killing evaluated using flow cytometry. Ramos cells opsonized with Blinatumomab were spiked into human whole blood and bispecific-dependent T cell killing evaluated with flow cytometry.

**Results**

T and NK cells were imaged at single-cell resolution as they penetrated and killed tumor spheroids in an antibody-dependent manner. Whole blood analysis with flow cytometry was able to clearly distinguish target cells from white blood cells, and clearly indicated the percent of target cells killed in an antibody-dependent fashion.

**Conclusions**

Penetration and potency of immune effector cells can be evaluated using whole-spheroid imaging. Flow cytometry provides a robust method to directly measure T, NK, and complement-dependent killing of cancer cells in human whole blood.

#### P589 IL-15 deficient colon carcinomas have decreased cytolytic lymphocytes and skewed myeloid cell populations

##### Kimberly Schluns, PhD^1^, Chih-Chien Chou, PhD^1^, Rosa Santana-Carrero^2^, R. Eric Davis, PhD^1^

###### ^1^University of Texas MD Anderson Cancer Center, Houston, TX, United States; ^2^UT MD Anderson Cancer Center/UT Health Graduate School of Biomedical Sciences, Houston, TX, United States

####### **Correspondence:** Kimberly Schluns (kschluns@mdanderson.org)

**Background**

Loss of Interleukin (IL)-15 expression by colon tumors strongly correlates with classification as microsatellite stable, decreased tumor infiltrating T cells, increased metastasis, and poor responses to immunotherapy. To better understand how IL-15 expression regulates tumor-associated immune cells and demonstrate the importance of IL-15 expressed by tumor cells, we developed a mouse model system to examine this by generating MC-38 tumor cells deficient in IL-15 using Clustered Regularly Interspaced Short Palindromic Repeats (CRISPR)/Cas9 system. The MC-38 cells are ideal for this as the tumor cells are the dominant source of sIL-15 complexes in the tumor microenvironment (TME) [1].

**Methods**

After one round of CRISPR/Cas9 deletion of IL-15, we have generated MC-38 cells that lack one IL-15 allele (i.e IL-15+/- MC-38 cells) and express decreased levels of soluble IL-15 complexes. A second round of deletion has led to complete deletion of IL-15 in MC-38 cells (i.e. IL-15-/- MC-38). IL-15+/- and IL-15-/- MC-38 cells and wildtype (Wt) MC-38 cells were implanted subcutaneously into Wt mice and tumor growth measure overtime. Additionally, immune cells in tumors were analyzed at earlier time points. RNAseq of tumor-associated myeloid cells was conducted in Wt MC-38 tumors treated with neutralizing IL-15 antibody.

**Results**

Surprisingly, deletion of one IL-15 allele was sufficient to affect tumor growth as IL-15+/- MC-38 tumors grew faster than Wt MC-38 tumors but had similar growth kinetics in vitro. IL-15+/- MC-38 tumors harbored less CD8 T cells and NK cells than Wt tumors whereas numbers of CD4 T cells were similar. Interestingly, these tumors also had increased CD11b+Ly6G+ and CD11b+Ly6ChiLy6G- myeloid cells (putative MDSCs). Implanted IL-15-/- MC-38 cells also progressed faster than Wt MC-38 cells. Gene expression analysis of myeloid cells showed that blocking IL-15 in tumors increased expression of CXCL4, CD206, Arginase 1, and tryptophan 2,3-dioxygenase and decreased IL-12β and IL-6.

**Conclusions**

These results demonstrate that even partially decreasing the levels of IL-15 in the TME negatively impacts the numbers of CD8 T cells and NK cells and promotes differentiation of putative MDSCs. These results suggest that IL-15 expressed by the tumor cells is a key attribute creating a microenvironment that supports cytolytic lymphocytes by not only acting directly on lymphocytes but also indirectly by skewing the myeloid cells towards pro-inflammatory activities. Overall, the IL-15-/- MC-38 cells is a model that can be used to identify the mechanisms contributing to the poor therapeutic responses observed in human colon carcinomas.

**References**

1. Santana Carrero, R.M. et al. IL-15 is a component of the inflammatory milieu in the tumor microenvironment promoting antitumor responses. 2019. Proc Natl Acad Sci U S A. 116(2):599-608.

**Ethics Approval**

All animal procedures were conducted in accordance with the animal care and use protocols (00000851-RN02) approved by the IACUC at the UT MD Anderson Cancer Center.

#### P590 The use of in vitro T cell assays to accelerate cancer development of immunotherapy and immune checkpoint inhibitors

##### Amin Osmani^1^, Thibault Jonckheere^2^, Sofie Pattijn^2^, Mayuko Oh^2^, Thibaut Janss^1^

###### ^1^ImmunXperts, Gosselies, Belgium; ^2^ImmunXperts Sa, Gosselies, Belgium

####### **Correspondence:** Amin Osmani (amin.osmani@immunXperts.com)

**Background**

During the last years, significant advancement has been made in the clinical application of cancer immunotherapies. Molecules directed against immune checkpoints and other agonists show great promise for treatment of a variety of malignancies. Next to CTLA-4 and PD-1 blockade, a wide range of therapeutics with the potential to reverse the tumor-induced suppression are under development.

**Methods**

Mixed lymphocyte reaction assays using both innate cells and lymphoid cells mimic a real physiological T cell response and are widely used for the potency screening of candidate therapeutics. The use of different allogenic donor combinations can provide additional information on the profile of the responding population. Other T cell assays such as antigen-specific CMV recall activation assays or SEB activation assays can be used to evaluate the ability of test molecules to promote T cell responses.

Next to these activation assays, the antigen-specificity within a naïve or pre-exposed T cell population can be evaluated by using T cell enrichment assays, which can be set-up using different formats depending on the origin of test molecules. Additionally, it might also be beneficial to generate peptide-specific T cell pools or clones to be used for functional testing e.g. in the context of tumor-associated antigens (TAA).

**Results**

All these in vitro T cell assays are optimized and fine-tuned for the screening of certain types of molecules. An important factor for sensitive and reproducible assays and consistent results is the quality of the primary immune cells. Therefore, all PBMC donor preparations are quality controlled and HLA-typed, and optimized procedures are used to generate functional subpopulations such as dendritic cells and T cell subpopulations.

**Conclusions**

Early evaluation of the effectiveness of candidate therapeutics and combination therapies can be assessed using mouse models and in vitro bioassays with mouse or human immune cells.

#### P591 In vitro functional suppressive bioassays for evaluating candidate therapeutic effectiveness in immuno-oncology

##### Amin Osmani^1^, Thibaut Janss^2^, Juliette Lamy^2^, Thibault Jonckheere^2^, Severine Giltaire^2^, Sofie Pattijn^2^

###### ^1^ImmunXperts, Gosselies, Belgium; ^2^ImmunXperts SA, Gosselies, Belgium

####### **Correspondence:** Amin Osmani (amin.osmani@immunXperts.com)

**Background**

The increasing interest in the tumour microenvironment leads to focus on new bioassays to represent all the players of the cancer immune response. Some of these players like Tumour Associated Macrophages (TAM), Myeloid Derived Suppressor Cells (MDSC) regulatory T cells (Treg) play an important role by downregulating the anti-tumour response. Their regulation mechanisms constitute an important target for new therapeutics. In order to study these mechanisms in a human model, suppressive bioassays, mimicking the suppressive action of these cells on T cells activations, were developed.

**Methods**

Macrophages possess important active and regulatory functions in both innate and adaptive immune responses. Classical activated macrophages, also classified as M1-like macrophages, comprise immune effector cells with an acute inflammatory phenotype while the alternatively activated M2-like macrophages have suppressive and healing capacities. Tumor associated macrophages (TAMs) are present at high densities in solid tumors and share many characteristics with so called M2 macrophages.

**Results**

Although distinguished classification and in vitro generation and polarization of M1- and M2-like macrophages is challenging, in vitro assays can be a first step to screen the effect of the test molecules on the phenotype and function of the macrophages. For example, macrophage precursors display extraordinary plasticity in response to exogenous and endogenous stimuli which can lead them to M2-polarized macrophages or towards the M1-activated status. Using in vitro polarization and functional macrophage assays, one can screen molecules with the potential to influence M1 and M2 like macrophage generation and polarization. Next to that, the effect of the test molecules on the function of the macrophages can be evaluated using a macrophage suppressive assay. Here the ability of the molecules to reverse the stimulating effect of the M1-macropahges or suppressive effect of the M2-macrophages can be determined.

Myeloid-derived suppressor cells (MDSC) and regulatory T cells (Treg) can also be found in the tumour microenvironment and present a highly suppressive phenotype. Their role in relation to cancer development and progression has shown to be of great importance. Therefore, the ability of molecules to reverse the suppressive function of the MDSC or Treg on immune effector cells can be evaluated in vitro using these cell-type specific suppressive bioassays.

**Conclusions**

The use of the bioassays contributes to a better understanding of the tumour microenvironment and the steps needed to generate an anti-tumour response by the immune system will help to assess the functional potential of new drugs, design clinical trials and ultimately discover relevant biomarkers.

#### P592 TGFβR1 antagonism improves radiation efficacy by enhancing CXCR3 dependent tumor recruitment of CD8+ T cells

##### Andrew Gunderson, PhD^1^, Tomoko Yamazaki, PhD^1^, Kayla McCarty, BS^1^, Nathaniel Fox^1^, Michaela Phillips, BA^1^, Alejandro Alice, PhD^1^, Tiffany Blair^1^, Mary McCormick, RN^1^, Andrea Burt, RN^1^, Iliana Gonzalez, BS^1^, Mark Whiteford, MD^2^, David O'Brien^2^, Rehan Ahmad^2^, Maria Kiely, MD^2^, Amanda Hayman, MD, MPH^2^, Rui Li, MD, PhD^1^, Todd Crocenzi, MD^1^, Michael Gough, PhD^1^, Marka Crittenden, MD, PhD^1^, Kristina Young, MD, PhD^1^

###### ^*1*^*Earle A. Chile Research Institute, Portland, OR, United States;*^*2*^*The Oregon Clinic, Portland, OR, United States* ; ^*3*^*Earle A. Chiles Research Institute, Portland, OR, United States*

####### **Correspondence:** Kristina Young (kristina.young@providence.org)

**Background**

Transforming growth factor beta (TGFβ) is a multipotent cytokine capable of promoting tumors in part through immunosuppression. With the development of clinically relevant immunotherapy, inhibition of TGFβ is a widely explored therapeutic strategy to enhance the efficacy of both traditional cytotoxic therapies and immunotherapies.

**Methods**

Based on our previous work, we initiated a Phase II trial of a TGFβ type I receptor (TGFβRI or ALK5) inhibitor in combination with neoadjuvant chemoradiation in patients with locally advanced rectal adenocarcinoma (NCT02688712). We sought to better understand the mechanism of action of TGFβRI inhibition in this setting using preclinical CT26 and MC38 colorectal cancer models and cell type-conditional Alk5-deficient transgenic mice. The ALK5 inhibitor was administered P.O. b.i.d. in two-1 week cycles with drug respite of 1 week between cycles. 5Gy x 5 consecutive daily fractions of CT image guided radiation was delivered between ALK5 inhibitor cycles with concurrent 5-FU (25mg/kg i.p. t.i.w.). To interrogate the actions of TGFβR1 signaling on specific cell types, we evaluated MC38 tumor growth and response to radiation in three separate conditional Alk5 knock-out mice under control of cell-type specific promoters: CD8Cre-ALK5flox/flox, LysMCre-ALK5flox/flox, and Foxp3Cre-ALK5flox/flox to ablate ALK5 expression in CD8+ T cells, macrophages and regulatory T cells, respectively.

**Results**

No improvement in tumor growth or radiation response was seen in animals with Treg or macrophage specific ALK5 deletion. However, CD8Cre-ALK5flox/flox (ALK5ΔCD8) mice largely rejected MC38 tumors in a CD8+ T cell dependent manner and demonstrated increased sensitivity to radiation therapy associated with superior CD8+ T cell effector phenotypes, and reduced threshold for TCR stimulated activation. Alk5-deficient CD8+ T cells had diminished SMAD2/3 occupancy at the CXCR3 promoter, increased CXCR3 expression and enhanced migration to CXCL10. Importantly, neutralization of CXCR3 in vivo blocked the rejection of tumors in ALK5ΔCD8 mice.

**Conclusions**

These results indicate that TGFβ signaling represses CXCR3-dependent trafficking of CD8+ T cells into tumors and can be reversed to improve tumor immunity alone and in combination with radiation therapy.

**Trial Registration**

NCT02688712

**Ethics Approval**

This clinical study was approved by Providence Health & Services IRB, approval number 15-050. The animal modeling was performed under the approval of our institution's IACUC Protocol #37, in our OLAW certified animal facility (Assurance #D16-00526).

#### P593 Rituximab treatment induces long lasting potentiation of degranulation responses by NK cells in non-Hodgkin lymphoma patients

##### Dmitry Zhigarev, MS^1^, Alexander Macfarlane^1^, Mowafaq Jillab^1^, R. Katherine Alpaugh, PhD^1^, Adam Cohen, MD^3^, Kerry Campbell^1^

###### ^*1*^*Fox Chase Cancer Center, Philadelphia, PA, United States;*^*2*^*Pirogov National Research Medical Univ., Philadelphia, PA, United States* ; ^*3*^*University of Pennsylvania, Philadelphia, PA, United States*

####### **Correspondence:** Kerry Campbell (kerry.campbell@fccc.edu)

**Background**

Rituximab is widely used to treat B-cell non-Hodgkin lymphomas (NHL). The antibody depletes B cells in patients by several mechanisms, including antibody-dependent cellular cytotoxicity (ADCC) by NK cells and monocytes, complement-mediated cell death, and signaling apoptosis. We tested degranulation responses by NK cells in peripheral blood from B-cell NHL patients before, during and after rituximab treatment.

**Methods**

Seventy-five patients with B-cell NHL [follicular lymphoma or marginal zone lymphoma] were monitored for immune phenotype and NK cell function in peripheral blood, as well as 54 healthy volunteers. All blood donors provided informed consent in accordance with our Institutional Review Board. Thirteen NHL patients were treated with rituximab and provided blood samples immediately before the first treatment, during the treatment period and three months after treatment. Peripheral blood mononuclear cells (PBMC) were isolated on Ficoll. Degranulation assays were performed by exposing PBMC to 721.221 EBV-transformed B cell line (natural cytotoxicity) for two hours and staining for LAMP-1 (CD107a). Degranulation was quantified by flow cytometry as percentage of LAMP-1+ CD56dim (mature, cytolytic) NK cells.

**Results**

NK cell degranulation responses were not significantly different between healthy donors or pretreatment samples from NHL patients. Within the patients treated with rituximab, a significant increase in the degranulation response of CD56dim NK cells was observed between the pretreatment and during treatment samples when assayed under natural cytotoxicity conditions (p

**Conclusions**

Our results provide evidence that a treatment course of rituximab can potentiate degranulation activity of peripheral blood NK cells under natural cytotoxicity conditions for at least 3 months after the end of therapy. At the same time NK cells become more resistant to activation-induced death during these natural cytotoxicity responses, which may contribute to the improved functional response.

**Acknowledgements**

We thank the FCCC Biosample Repository for coordinating patient consenting and sample acquisition and support from the FCCC Keystone Program, FCCC Boards of Associates, and NIH grants CA083859 (KSC) and CA06927 (FCCC).

**Ethics Approval**

The study was approved by the Fox Chase Cancer Center Ethics Board, approval numbers 99-802 and 99-839

#### P594 Targeting immune-suppressive myeloid cell pathways for the treatment of cancer

##### Jesus Banuelos, Susan Lee, PhD, Tim Park, Nell Narasappa, MSc, Dana Piovesan, MSc, Yu Chen, Jenna Jeffrey, PhD, Jarek Kalisiak, Kimberline Gerrick, Hema Singh, Annette Becker, PhD, Jie Chen, Sean Cho, PhD, Bryan Handlos, Akshata Udyavar, PhD, Steve Young, PhD, Jay Powers, PhD, Matthew Walters, PhD, Jo Tan

###### Arcus Biosciences

####### **Correspondence:** Jesus Banuelos (jbanuelos@arcusbio.com)

**Background**

Elevated levels of myeloid-derived suppressor cells (MDSCs) have been associated with a blunted response to PD-(L)1 inhibitors in vivo and suppressive myeloid cells have been shown to inhibit T cell responses in vitro. Suppressive myeloid cells mediate their immunosuppressive effects via multiple mechanisms, including expression of immune checkpoint protein ligands, activation of the PI3Kγ pathway, and release of arginase-1 (ARG1), an arginine-depleting enzyme. The significant role of myeloid cells in dampening anti-tumor immunity makes them an attractive target for immunotherapy. To that end, we have developed potent and selective inhibitors against PI3Kγ and ARG1.

**Methods**

Recombinant and endogenous ARG1 from human granulocytic lysates were used to determine AB474 potency. The ability of AB474 (ARG1 inhibitor), to rescue recombinant ARG1-mediated inhibition of T cell activation was assessed in human T cells isolated from healthy donors. The ability of PI3Kγ inhibition to stimulate immune cells was assessed in CD14+ monocytes or monocyte-derived M1 polarized macrophages and confirmed using mixed lymphocyte reactions. Anti-tumor activity was determined using syngeneic tumor models

**Results**

Using sorted immune cells from healthy donor PBMC, we determined that PI3Kγ expression is highest in monocytes, whereas PI3Kδ expression is higher in lymphocytes. Inhibition of PI3Kγ significantly (p

Elevated levels of ARG1 were observed in cancer patients compared to healthy donors. In particular, ARG1 was highly expressed in polymorphonuclear-MDSCs while lower levels were seen in monocytic-MDSCs. AB474 inhibited the activity of both recombinant and endogenous ARG1 with an IC50 of 15 nM and 20 nM, respectively. Consistent with this observation, AB474 reversed ARG1-mediated suppression of human CD8+ T cell activation. Additionally, single agent AB474 significantly (p

**Conclusions**

Myeloid cells are associated with blunted responses to checkpoint protein blockade. High expression of both PI3Kγ and ARG1 within suppressive immune subsets contributes to the dampening of pro-inflammatory responses. Inhibiting these targets can reduce immunosuppression, enhance anti-tumor responses and act in concert with immune checkpoint blockade and other immunotherapies.

#### P595 Gene editing and protein expression in innate immunity cells by electroporation

##### Jian Chen, PhD, George Sun

###### Celetrix LLC, Manassas, VA, United States

####### **Correspondence:** Jian Chen (jchen@celetrix.com)

**Background**

The interest in the therapeutic potentials of the innate immune system is growing as we gained more knowledge of role of the innate immune system in fighting cancer. Natural killer (NK) cells and macrophages are capable of attacking tumor cells directly and dendritic cells are important for antigen presentation. Macrophages are also important for modulation of tumor microenvironment that presents both obstacles and opportunities to fighting tumor cells. Gene expression or gene editing in the innate immune cells are not only important for studying gene functions in these cells, but also important for potential clinical applications.

**Methods**

Using our novel electroporation technology, we have found that the innate immunity cells can all be modified by protein expression plasmid vectors or CRISPR-Cas9 mediated gene editing.

**Results**

Primary NK cells, primary bone marrow monocytes and peripheral blood monocytes need relatively high voltage for electroporation. Primary mouse peritoneal macrophage needs lower voltage and expanded NK cells as well as mature dendritic cells need lowest voltage. Using the same electroporation buffer, we were able to successfully electroporate these different cells using different voltages. The transfection efficiency for plasmids can reach 80% and Cas9 ribonucleorprotein (RNP) with gRNA can also be electroporated successfully.

**Conclusions**

Our capability to modify all of the major types of the innate immunity cells by electroporation is a significant step that could contribute to harnessing the power of the innate immunity system for immunotherapy of cancer.

#### P596 Targeted IRAK-M degradation as a novel approach in cancer immunotherapy overcoming innate-driven immunosuppression

##### Kanae Gamo, MS , Naomi Kitamoto, Tomoaki Hayashi, Yoshihide Tomata, Yusuke Tominari

###### FIMECS, Inc., Fujisawa, Japan

####### **Correspondence:** Kanae Gamo (kanae.gamo@fimecs.com)

**Background**

IRAK-M has an important role in tightly controlling innate immune responsiveness to preserve immune homeostasis by acting as a negative feedback regulator of TLR/IL-1R signaling pathway. Targeting IRAK-M, which expression is restricted to myeloid cells, would be potentially limiting adverse events against non-target tissues. From supporting evidence for the role of IRAK-M in innate immunosuppressive capacity of myeloid cells in tumor microenvironment, we have generated compounds targeting IRAK-M as an effective cancer-immunotherapy strategy by converting tumors to more inflamed state. Since IRAK-M is a pseudokinase which lacks kinase activity, it is considered 'undruggable’ that could not be targeted pharmacologically by the conventional small molecule. Therefore, we have developed heterobifunctional degrader molecules comprising IRAK-M-binding moiety linked to proprietary E3 binders to eliminate IRAK-M protein.

**Methods**

Optimization of IRAK-M degraders have conducted by applying RaPPIDS(TM) which is a proprietary divergent degrader synthetic platform and identified multiple preclinical candidates within a year. We measured the activity of degradation mechanisms in vitro by analyzing degradation rates, protein turnover and proteasome dependence. To assess the effect of IRAK-M degrader on myeloid-derived suppressor cells (MDSCs), we performed MDSC suppression assays in a co-culture model with CD8+ T cells. One of the drug candidates was profiled extensively in different syngeneic models. Subsets of tumors and spleen from treated mice were analyzed ten days following dosing initiation for myeloid cell populations and phenotype.

**Results**

We demonstrated a dose- and time-dependent degradation of IRAK-M protein in THP1 human monocytic leukemia cells with degrader treatment in a proteasome-dependent manner. MDSC suppression assays revealed that IRAK-M degrader could release the suppressive function of MDSC on both IFNγ production by CD8+ T cells as well as T cell proliferation. When examined in mouse models, IRAK-M degrader showed significant anti-tumor activity in several syngeneic models at tolerated doses and schedules. In the 4T1-HA model, we found increase of LPS-induced TNFα production in white blood cells from compound injected mice associated with degradation of IRAK-M protein as pharmacodynamic effect. FACS analysis showed that IRAK-M degradation translated into increased infiltration of M1-like activated macrophage into tumors and spleen. Advanced profiling of the drug candidate is ongoing in IND-enabling studies.

**Conclusions**

In summary, we demonstrated IRAK-M degrader as a novel and promising immuno-modulatory therapeutic modality and the great potential of shifting the balance between tolerance and immunity by releasing immunosuppressive network activity, leading to a more favorable tumor microenvironment to enhance host immunity.

#### P597 Myeloid-specific inflammasome signaling promotes tumor growth

##### Cara Lang^1^, Young Kim, MD^2^

###### ^1^Vanderbilt University, Nashville, TN, United States; ^2^Vanderbilt University Medical Center, Nashville, United States

####### **Correspondence:** Young Kim (y2.kim@vumc.org)

**Background**

Tumor infiltrating myeloid cells, such as Myeloid Derived Suppressor Cells (MDSCs) and Tumor Associated Macrophages (TAMs), are important mediators of immune evasion in cancer, and are associated with worse overall survival and progression free survival in various types of solid tumors. However their procarcinogenic functions in the tumor microenvironment are unclear. Here, we propose that the inflammasome signaling pathway is a key T cell independent mechanism of myeloid-mediated tumor growth.

**Methods**

In this work, we use various adoptive transfer models to generate bone marrow specific inflammasome knockout mice and utilized a B16 melanoma model to assess changes in tumor growth.

**Results**

We first performed single cell sequencing of Head and Neck Squamous Cell Carcinoma (HNSCC) tumors, and we found IL-1β expression is restricted to the tumor infiltrating myeloid cell populations. From our working hypothesis that myeloid inflammasome signaling can promote T-cell independent tumor growth, we initially showed that caspase 1 is important for promoting tumor growth in a myeloid specific manner. However, the mechanisms by which myeloid-intrinsic inflammasome activation promotes T-cell independent tumor growth are not well understood. Here we utilize a B16 melanoma tumor growth model to show that NLRP3 dependent caspase 1 activation is driving tumor growth in vivo, while AIM2 has no effect on tumor growth. In addition, we have found that circulating T cells in tumor bearing inflammasome knockout mice have higher expression levels of the checkpoint molecule PD-1. We utilized a C57Bl/6 mouse model of B16 melanoma to demonstrate that combination of inflammasome blockade and checkpoint blockade further reduces tumor burden in vivo.

**Conclusions**

This work will have broad implications across all solid tumor types and will allow for the understanding of how myeloid-specific production of IL-1β promotes tumor growth. In addition, the proposed work will contribute to our basic knowledge of inflammasome activation within the tumor microenvironment.

#### P598 Glioblastoma-associated myeloid cells promote regulatory T and B-cell function via transfer of microvesicle

##### Catalina Lee-Chang, PhH^1^, Aida Rashidi^1^, Jason Miska^2^, Peng Zhang^1^, Katarzyna Pituch^1^, David Hou^1^, Irina Balyasnikova, PhD^1^, Maciej Lesniak, MD^1^

###### ^1^Northwestern University, Chicago, IL, United States; ^2^Northwestern Unversity, Chicago, IL, United States

####### **Correspondence:** Catalina Lee-Chang (catalina.leechang@northwestern.edu)

**Background**

Recent clinical trials in brain tumor immunotherapy have gathered tremendous attention while at the same time establishing a favourable safety profile and preliminary efficacy. Immunotherapy is a promising approach in other malignancies but needs to be tailored specifically to glioblastoma (GBM) given the highly immunosuppressive tumor microenvironment [1, 2]. A hallmark of GBM microenvironment is the massive infiltration of bone marrow-derived tumor-associated myeloid cells (TAMCs) and regulatory T-cells (Tregs) [3-7]. We recently identified the existence of perivascular regulatory B-cells (Bregs) that blocked the activation of cytotoxic CD8+ T-cells in a subset of GBM patients. Even though tremendous progress has been made to understand GBM-mediated immunosuppression [8-10], there is still limited knowledge regarding the mechanisms by which the tumor selects, maintains and promotes immunosuppressive cells in detriment of anti-tumor immunity. A deeper comprehension of these process will propel the rational design of successful immunotherapies for GBM. Recent studies in our laboratory where tumor-infiltrating myeloid cells were specifically depleted using antibody-targeting drug delivery via nanoparticles in preclinical settings, pointed out to the almost total dependency of Tregs and Bregs on TAMCs to maintain their cellular abundance and immunosuppressive functions. The data presented here highlight the biological relevance of TAMC-derived microvesicle in controlling remotely regulatory lymphocytes.

**Methods**

Microvesicles were isolated by ultracentrifugation. Microvesicle uptake by lymphocytes was assessed by flow cytometry using fluorescent lipophilic dye.

**Results**

TAMC can control both Bregs and Tregs via microvesicle production. Microvesicle uptake by Tregs and Bregs was assessed using CD163+ TAMCs from GBM tumors. Proteomics and immunophenotype analysis of TAMC-derived microvesicles revealed that inactive TGFb and PD-L1 are transported as “cargos”. Our data showed that microvesicle-TGFb controls Tregs and Bregs cellular abundance. On the other hand, microvesicle-PDL1 are transfer and recycled via endocytosis by target cells, conferring them with immunosuppressive properties.

**Conclusions**

Glioma TAMCs utilize microvesicles to remotely promote Tregs and Bregs survival and immunosuppressive functions. This data suggest TAMC-mediated immunosuppression extends beyond the tumor vicinity limits.

**References**

1. Vega EA, Graner MW, Sampson JH. Combating immunosuppression in glioma. Future Oncol 2008;4(3):433-42 doi 10.2217/14796694.4.3.433.

2. Wainwright DA, Nigam P, Thaci B, Dey M, Lesniak MS. Recent developments on immunotherapy for brain cancer. Expert Opin Emerg Drugs 2012;17(2):181-202 doi 10.1517/14728214.2012.679929.

3. Wintterle S, Schreiner B, Mitsdoerffer M, Schneider D, Chen L, Meyermann R, et al. Expression of the B7-related molecule B7-H1 by glioma cells: a potential mechanism of immune paralysis. Cancer Res 2003;63(21):7462-7.

4. Raychaudhuri B, Rayman P, Ireland J, Ko J, Rini B, Borden EC, et al. Myeloid-derived suppressor cell accumulation and function in patients with newly diagnosed glioblastoma. Neuro Oncol 2011;13(6):591-9 doi 10.1093/neuonc/nor042.

5. Fecci PE, Mitchell DA, Whitesides JF, Xie W, Friedman AH, Archer GE, et al. Increased regulatory T-cell fraction amidst a diminished CD4 compartment explains cellular immune defects in patients with malignant glioma. Cancer Res 2006;66(6):3294-302.

6. El Andaloussi A, Lesniak MS. An increase in CD4+CD25+FOXP3+ regulatory T cells in tumor-infiltrating lymphocytes of human glioblastoma multiforme. Neuro Oncol 2006;8(3):234-43 doi 10.1215/15228517-2006-006.

7. El Andaloussi A, Han Y, Lesniak MS. Prolongation of survival following depletion of CD4+CD25+ regulatory T cells in mice with experimental brain tumors. J Neurosurg 2006;105(3):430-7 doi 10.3171/jns.2006.105.3.430.

8. Nduom EK, Weller M, Heimberger AB. Immunosuppressive mechanisms in glioblastoma. Neuro Oncol 2015;17 Suppl 7:vii9-vii14 doi 10.1093/neuonc/nov151.

9. Kamran N, Kadiyala P, Saxena M, Candolfi M, Li Y, Moreno-Ayala MA, et al. Immunosuppressive Myeloid Cells' Blockade in the Glioma Microenvironment Enhances the Efficacy of Immune-Stimulatory Gene Therapy. Mol Ther 2017;25(1):232-48 doi 10.1016/j.ymthe.2016.10.003.

10. Kamran N, Chandran M, Lowenstein PR, Castro MG. Immature myeloid cells in the tumor microenvironment: Implications for immunotherapy. Clin Immunol 2016 doi 10.1016/j.clim.2016.10.008.

**Ethics Approval**

IRB N° STU00202003, AICUC protocol N° IS00002459

#### P599 Unveiling tumor-associated macrophages (TAMs) heterogeneity and plasticity by a fully automated sequential chromogenic multiplex assay

##### Anna Martirosyan, Dr, Jacques Fieschi, PhD

###### HalioDx, Marseille, France

####### **Correspondence:** Jacques Fieschi (jacques.fieschi@haliodx.com)

**Background**

Among the various immune cells recruited to the tumor microenvironment (TME), tumor-associated macrophages (TAMs) are particularly abundant and play a central role at all stages of cancer progression. TAMs represent a heterogeneous population in terms of cell morphology, functions, and tissue localization. Activated M1 macrophages express pro-inflammatory cytokines and drive potent antitumoral Th1 responses. Alternatively, activated M2 macrophages induce Th2 cells and enhance cancer progression and metastasis. The specific polarization of TAM may therefore skew the TME toward tumor rejection (M1) or favor immune suppression (M2). An additional level of complexity is conferred by their ability to shift between M1 and M2 subtypes (plasticity), depending on microenvironmental stimuli. Albeit the extensive literature on TAMs protumoural function and poor prognosis, some studies have shown that these cells possess an antitumour activity and their increase correlates with patient’s survival. Noteworthy, emerging studies suggest that TAMs are important in tumor metastases formation by regulating epithelial mesenchymal transition of colorectal cancer (CRC) cells. Until now, the most commonly used marker to identify human macrophages was the pan-macrophage marker CD68. The latter overlooks the prognostic potential of macrophage subsets with different functions in the TME. Therefore, there is a strong and urgent need to dissect TAMs heterogeneity in tumors using more refined approaches such as multiplex technology.

**Methods**

Here we assessed the presence, abundance and localization of TAMs subsets within the CRC microenvironment by using the Brightplex® solution, an automated sequential chromogenic multiplex assay. A unique combination of biomarkers (CD11b, CD68, CD86, CD163, CD204, CD206, and CD64) was developed to assess M1 and M2 TAMs heterogeneity and plasticity on a single FFPE tumor tissue section by preserving tissue’s spatial contexture.

**Results**

Briefly, a tissue section was sequentially stained, digitized, unstained and re-stained with antibodies targeting the seven markers. Images of the whole slide were then analyzed by digital pathology and the detection of positive cells was performed for each marker independently. In addition, tissue segmentation tools were used to assess TAMs densities in parenchyma, tumor stroma and invasive margin regions. This new panel of the Immunoscore® Suppressor Cells family enhances the ability to unravel the tumor microenvironment.

**Conclusions**

Deciphering the heterogeneity and plasticity of TAMs populations in CRC could help to identify new reliable prognostic markers to improve patient’s stratification and design more individualized therapeutic approaches.

#### P600 AL008 enhances myeloid anti-tumor function by coupling SIRPα antagonism with Fc receptor-mediated activation

##### Andrew Pincetic, PhD, Jerry Yang, Isaiah Deresa, We-Hsien Ho, Hua Long, Daniel Maslyar, Spencer Liang, Arnon Rosenthal

###### Alector, South San Francisco, CA, United States

####### **Correspondence:** Arnon Rosenthal (ar@alector.com)

**Background**

The SIRPα-CD47 axis represents a myeloid checkpoint in cancer. Recent clinical data with CD47-targeted therapies that competitively block the interaction with SIRPα show clinical responses in some patients with hematologic and solid cancers when combined with anti-tumor antigen antibodies. However, known limitations associated with targeting CD47, such as clearance of red blood cells (RBCs) and disruption of the SIRPγ-CD47 interaction important for T cell activation, likely hinder the anti-tumor immune response necessary for robust clinical benefit. A therapeutic advantage may be realized by targeting SIRPα and promoting immunostimulatory pathways that drive anti-tumor immunity. Here, we describe the discovery and characterization of a SIRPα-specific antibody, AL008, a first-in-class inhibitor of SIRPα that simultaneously antagonizes SIRPα signaling and stimulates Fcγ receptor (FcγR) activation.

**Methods**

Multiple functional assays were performed to ascertain the mechanism of action of AL008 and to benchmark activity against anti-CD47/anti-SIRPα antibodies in the clinic. For example, in vitro phagocytosis assays determined whether AL008 promoted engulfment of various tumor cell lines by human macrophages. Mixed leukocyte reactions (MLR) assessed the effect of AL008 on human dendritic cell-mediated T cell function. Furthermore, AL008 was administered to non-human primates to obtain pharmacokinetic and immunotoxicology profiles. Anti-tumor activity and pharmacodynamic effects of AL008 were examined in tumor-bearing mouse models expressing human SIRPα and human CD47.

**Results**

AL008 is a non-competitive inhibitor of SIRPα that triggers SIRPα internalization and degradation from the cell surface and simultaneously activates FcγR. Unlike other competitive antagonist antibodies to SIRPα, which require combination with opsonizing antibodies to promote tumor cell phagocytosis, AL008 stimulates tumor cell engulfment by macrophages in the absence of opsonizing antibody due to intrinsic FcγR activation. Because of its restricted binding specificity towards SIRPα, AL008 promotes dendritic cell-mediated stimulation of T cell proliferation, which contrasts with anti-CD47 antibodies and other anti-SIRPα/γ cross-reactive antibodies. Despite FcγR engagement by AL008, in vitro ADCC assays and in vivo studies in non-human primates verify that AL008 does not deplete myeloid cells or RBCs, which anticipates a significantly improved safety profile in patients. Lastly, inhibition of tumor growth by AL008 in mouse models coincides with downregulation of SIRPα and induction of activation markers on tumor-associated macrophages.

**Conclusions**

AL008 potentiates the anti-tumor effector functions of macrophages and dendritic cells in vitro, as well as in vivo models. This dual mechanism of AL008, which couples the reversal of inhibitory signal with immunostimulation, provides a novel therapeutic strategy for targeting myeloid cells for immune activation.

**Ethics Approval**

All in vivo studies were reviewed and approved by Alector Institutional Animal Care and Use Committee (IACUC).

#### P601 Expression of an immune checkpoint receptor VSIG4 defines new subsets of mouse peritoneal macrophages

##### Kanako Lewis, PhD, Chau Nguyen, Robert Navert, Ryan Gerhart, Sonja Zahner, Castle Funatake, Homero Sepulveda, Dariusz Stepniak

###### Thermo Fisher Scientific, Waltham, MA, United States

####### **Correspondence:** Dariusz Stepniak (dariusz.stepniak@thermofisher.com)

**Background**

V-set and Immunoglobulin Domain Containing 4 (VSIG4), also known as Complement Receptor of the Immunoglobulin superfamily (CRIg), is a cell surface receptor structurally related to the B7 family of immune regulatory proteins. Expression of VSIG4 on macrophages renders them less responsive to LPS. In addition, VSIG4 promotes immune tolerance by attenuating early T cell activation and supporting the induction and maintenance of Foxp3 in T cells. Expression of VSIG4 on tumor-infiltrating macrophages suggests that it may be implicated in immune evasion.

**Methods**

Using our new VSIG4 (CRIg) antibody (clone NLA14), we demonstrate that Large Peritoneal Macrophages (LPM) in mouse consist of two distinct subsets, the VSIG4+ LPM and VSIG4- LPM.

**Results**

VSIG4+ LPM co-express Arginase 1 more frequently than the VSIG4- LPM, particularly in C57BL/6 mice. Further comparative analysis of the two LPM populations using a panel of macrophage specific antibodies revealed that VSIG4+ and VSIG4- LPM show a similar pattern of expression that is distinct from Small Peritoneal Macrophages (SPM). This phenotypic diversity within the LPM most likely reflects differences in function and activation status. In vitro cultured macrophages show a spontaneous decrease in the expression of VSIG4, and addition of proinflammatory factors, including IFN-γ and LPS, further reduces VSIG4 expression.

**Conclusions**

Characterization of VSIG4+ LPM might provide an opportunity to better understand the immunoregulatory role of VSIG4 in tumor infiltrating macrophages.

#### P602 A distinctive lineage negative cell population produces IL-17A in cutaneous squamous cell carcinoma

##### Lillian Sun, BS^1^, Jennifer Ko, MD and PhD^2^, Allison Vidimos, MD^2^, Shlomo Koyfman, MD^2^, Brian Gastman, MD^2^

###### ^1^Case Western Reserve University, Solon, OH, United States; ^2^Cleveland Clinic, Cleveland, OH, United States

####### **Correspondence:** Brian Gastman (gastmab@ccf.org)

**Background**

IL-17A is a key pro-inflammatory cytokine indicated in multiple pathologies including tumor progression via suppression of anti-tumor immunity. While IL-17A is a signature cytokine of CD4+ T helper cells (Th17 cells), IL-17A is also produced by other cell types, including CD8+ T cells and type 3 innate lymphoid cells (ILC3s) in the mucosal surfaces. It remains unclear what are the key cellular sources of IL-17A in cutaneous squamous cell carcinoma.

**Methods**

Human cutaneous squamous cell carcinoma were collected from patients undergoing surgery to remove skin tumors that were diagnosed as SCCs by a dermatopathologist. Specimen collection and processing was approved by the Institutional Review Board of the Cleveland Clinic Foundation. For flow cytometry analysis, fresh tumors were cut into small pieces and dissociated with collagenase IV/hyaluronidase, followed by staining with antibodies against CD45 (BioLegend, clone 2D1,368529), Linage (BD, lin3, 643510) and IL-17A (Bioligand, Clone BL168, 512319). For immunohisotochemistry/fluorescence staining, tissues were fixed with 10% formalin overnight and then kept in 70% ethanol at 4 °C until processed into paraffin tissue blocks by Imaging Core at Cleveland Clinic Lerner Research Institute.

**Results**

We detect CD45+Lin-(CD3-CD14-CD19-CD2-)IL-17A+ cells in the cutaneous squamous cell carcinomas (cSCCs) by flow cytometry of the cell suspensions prepared from tumor tissues. We found CD3-IL-17+ cells in tumor tissue of skin cSCCs by immunohistochemistry staining of serial sections of SCCs from both immunocompetent and immunocompromised patients (e.g. transplant patients on iatrogenic long-term immunosuppressive therapy). In some cases, the CD3-IL-17+ cells consist of over 90% of the total IL-17+ cells in the tumor tissue. Furthermore, these CD3-IL-17+ cells are negatively stained for SMA, CD11b and CD19, suggesting that they are unlikely to be fibroblast, myeloid cells or B cells.

**Conclusions**

Taken together, we found a population of lineage negative IL-17A producing cells present in the cSCCs, which share the “CD45+Lin-” features with innate lymphoid cells. We are actively identifying this cell population. This study suggests that IL-17A can be produced by immune cell populations other than T cells in skin SCCs.

#### P603 Deciphering immune checkpoint interactions between immune and non-immune cells in head and neck squamous cell carcinoma by single-cell RNA sequencing

##### Lazar Vujanovic, PhD^1^, Aditi Kulkarni^2^, Cornelius Kurten, MD^3^, Patricia Santos, PhD^1^, Umamaheswar Duvvuri^1^, Seungwon Kim, MD^1^, Anthony Cillo, PhD^1^, Robert Ferris, MD, PhD^2^

###### ^*1*^*University of Pittsburgh, Pittsburgh, PA, United States;*^*2*^*UPMC Hillman Cancer Center*, *Pittsburgh, PA, United States;*^*3*^*Universitätsklinikum Essen, Essen, Germany*

####### **Correspondence:** Robert Ferris (ferrisrl@upmc.edu)

**Background**

Resistance to the current generation of immunotherapies is mediated by complex relations between stromal, cancer and immune cells found within the tumor microenvironment (TME). Development of more efficacious drugs is predicated on improved understanding of these multi-spatial interactions. With emergence of new immune checkpoint receptor (ICR)-targeting therapies, a better understanding of topological expression of immune checkpoint ligand (ICL) on suppressive cell types in the TME may allow for improved strategies to treat cancer patients.

**Methods**

Single cell RNA sequencing (scRNAseq) was performed from head and neck squamous cell carcinoma (HNSCC) specimens (n=17) with matched blood from treatment-naïve patients. Immune and non-immune cells were enriched from tumor cell suspensions. Novel transcriptomic cell-to-cell interactions were predicted between heterogeneous cell populations. Histologic inflammation was corroborated with scRNAseq and multi-plex flow cytometry.

**Results**

Major cell type clusters (immune, epithelial, fibroblast and endothelial cells) were identified. Expression patterns for PD-1, TIGIT, LAG-3 and TIM-3 ligands were evaluated on these suppressive TME cell types. While LGALS9/galectin-9 levels were expressed highly on tumor-associated macrophages (TAMs) from both inflamed and non-inflamed tumors, CD274/PD-L1 was only upregulated on TAMs from inflamed lesions. By modeling receptor-ligand interactions between CD8+ T cells and the rest of the major TME cell types, CD8+ T cells were predicted to form more ICR-ICL interactions with TAMs than with any other cell type. Flow cytometry confirmed the abundance of PD-L1 and galectin-9 on TAMs.

**Conclusions**

Our data suggest that in the setting of HNSCC, TAMs are the major contributors of ICL in the HNSCC TME. Strategies that selective target this immunosuppressive population may be necessary to break tolerance to PD-1-targeting therapies.

**Ethics Approval**

The study was approved by the University of Pittsburgh’s Institutional Review Board (#99-069).

#### P604 Hypoxia and TGF-β1 induce tissue resident memory phenotype cells from human peripheral blood T-cells *in vitro*

##### Farah Hasan, BSc, Rebecca Shaw, Yulun Chiu, Junmei Wang, Cassian Yee, MD

###### The University of Texas MD Anderson Cancer Center, Houston, TX, United States

####### **Correspondence:** Cassian Yee (cyee@mdanderson.org)

**Background**

Recent reports suggest that tissue resident memory cells (T_RM_) may play an important role in anti-tumor immunity. Specifically, the accumulation of CD103+ tumor infiltrating lymphocytes (TIL_RM_) is associated with favorable prognosis [1,2]. However, relatively little is known regarding T_RM_ differentiation and endogenous T_RM_ are difficult to isolate, impeding their basic study and translational application. Thus a means to generate T_RM_ *in vitro* would be desirable. TGF-β is known to be critical in establishing T_RM_ populations and attempts to identify other factors have focused on cytokines [3,4]. Oxygen tension is another factor that distinguishes the circulation from peripheral tissues; thus we hypothesized that hypoxia may contribute to a TGF-β1-induced T_RM_ phenotype in human CD8+ T-cells.

**Methods**

Naïve CD8+ T-cells from human peripheral blood were activated in normal cell culture conditions (20% O2) or hypoxia (2% O2) and then cultured in the presence of rhTGF-β1. Cells were then analyzed for expression of T_RM_-associated genes via quantitative real-time PCR (qPCR) and T_RM_-associated surface markers via flow cytometry. The individual contributions of hypoxia and TGF-β1 to induction of T_RM_ phenotype cells were assessed by performing *in vitro* differentiation experiments as described above with or without the addition of rhTGF-β1. CD69-CD103- cells generated in 20% O2, CD69+CD103- cells generated in 2% O2, and CD69+CD103+ cells generated in 2% O2 + TGF-β1 were sorted before RNA-sequencing (*n*=3).

**Results**

We demonstrate that differentiation of human CD8+ T-cells in hypoxia and TGF-β1 *in vitro* led to the development of a T_RM_ phenotype, characterized by an increase in CD69+CD103+ cells expressing CD49a, CD101, and PD-1 – hallmarks of human T_RM_ (*n*=7, *p*_RM_ phenotype cells than the additive effects of either condition alone (*n*=4, *p*_RM_ and TIL_RM_ gene signatures in CD69+CD103+ cells generated in hypoxia and TGF-β1 (Figure 1).

**Conclusions**

Our findings identify a previously unreported cue for T_RM_ differentiation and enable a facile means of generating human T_RM_ phenotype cells *in vitro* with the potential for application to multiple existing Adoptive Cell Therapy (ACT) modalities. As hypoxia and TGF-β are common features of the tumor microenvironment our results indicate that these conditions may contribute to generation of CD103+ TIL *in vivo*.

**Acknowledgements**

This work was supported by the Parker Institute for Cancer Immunotherapy (PICI); C.Y. is a member of PICI

**References**

1. Smazynski J, Webb JR. Resident Memory-Like Tumor-Infiltrating Lymphocytes (TILRM): Latest Players in the Immuno-Oncology Repertoire. Front Immunol. 2018;9:1741. doi: 10.3389/fimmu.2018.01741.

2. Kim Y, Shin Y, Kang GH. Prognostic significance of CD103+ immune cells in solid tumor: a systemic review and meta-analysis. Sci Rep. 2019;9(1):3808. doi: 10.1038/s41598-019-40527-4.

3. Casey KA, Fraser KA, Schenkel JM, Moran A, Abt MC, Beura LK, et al. Antigen-independent differentiation and maintenance of effector-like resident memory T cells in tissues. J Immunol. 2012;188(10):4866-75. doi: 10.4049/jimmunol.1200402.

4. Skon CN, Lee JY, Anderson KG, Masopust D, Hogquist KA, Jameson SC. Transcriptional downregulation of S1pr1 is required for the establishment of resident memory CD8+ T cells. Nat Immunol. 2013;14(12):1285-93. doi: 10.1038/ni.2745.

**Ethics Approval**

This study was approved by University of Texas MD Anderson Cancer Center’s Institutional Review Board, approval number PA14-0105.

**Fig. 1 (abstract P604).**
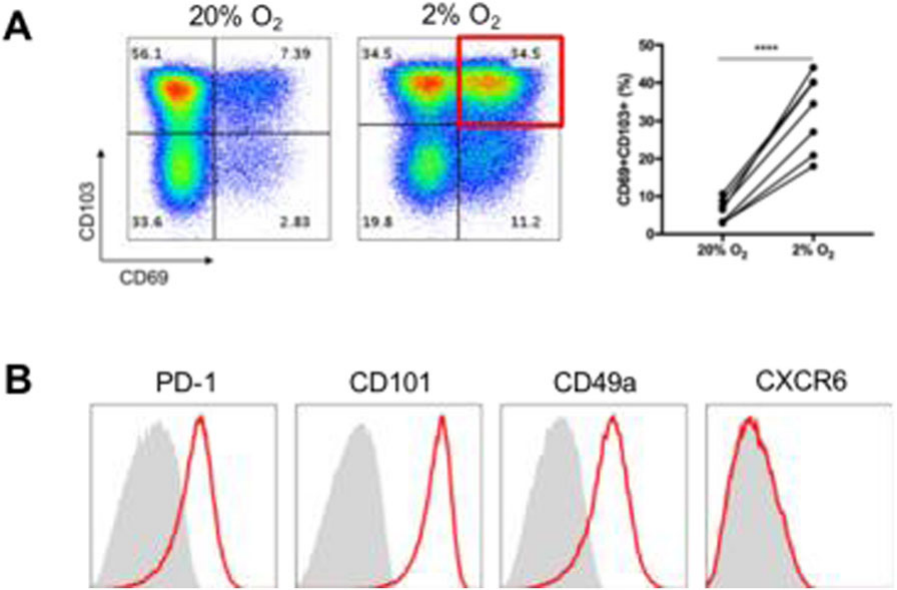
In vitro induction of tissue resident memory phenotype cells

#### P605 Severity of cytokine release syndrome as a predictor of infections after T-cell replete haploidentical hematopoietic cell transplantation (haploHCT)

##### Muhammad Bilal Abid, MD, MRCP (UK), Parameswaran Hari, MBBS, MD, MRCP, Aniko Szabo, Mehdi Hamadani, Mary Beth Graham, Saurabh Chhabra

###### Medical College of Wisconsin, Milwaukee, WI, United States

####### **Correspondence:** Saurabh Chhabra (schabra@mcw.edu)

**Background**

Severe cytokine release syndrome (CRS) results in an increased incidence of infections after T-cell engaging therapies. Several retrospective, single-center studies have shown infectious complications with haploidentical hematopoietic cell transplantation (haploHCT) but no data correlating infections with CRS that develops after haploHCT is available. [1-10]

**Methods**

We evaluated 78 consecutive adult haploHCT recipients at our center for the development of CRS, graded by Lee et al. criteria, and examined the incidence of infectious complications in correlation with CRS severity. All patients received haploHCT for hematologic malignancies between April 2012 and April 2018, using post-transplant cyclophosphamide (PTCy) and tacrolimus/MMF for GVHD prophylaxis. The incidence of infections was examined in two separate time periods in relation to the day of stem cell infusion (day 0): day 0-100 and day 101-180. The study cohort was further stratified by CRS grades into 2 groups: 53% of patients in cohort A, CRS grade 0-1 (n=41) and 47% in cohort B, CRS grade ≥2 (n=37).

**Results**

Overall, 81% patients developed infections (63/78) within 180 days. Fewer patients in cohort A developed an infection (71% vs. 92%; P=.018). 58% of these infections were of viral origin (104 episodes) and nearly half of those were CMV reactivations (46%). 38% infections were of bacterial origin (70 episodes) and 5% fungal. On multivariate analysis, higher-grade CRS (CRS grade ≥2) was independently associated with an increased frequency of bacterial infections (RR 2.25; 95% CI, 1.12 to 4.53; P=.023) and there was also evidence of higher overall infections (RR 1.6; 95% CI, 0.98 to 2.63; P=.057). Increasing frequency of overall and bacterial infections, including BSI and CLABSI, was observed as CRS became more severe (higher in grade 3 vs grade 2). CMV recipient positivity was independently associated with an increased frequency of overall infections (RR 1.98; 95% CI, 1.23 to 3.18; P=.0048) as well as an expectedly greater viral infections (RR 2.77; 95% CI, 1.60 to 4.78; P=.0003). 10 patients died of infection/sepsis-related causes and the cumulative incidence of infection-related mortality was higher in cohort B (Figures 1-3).

**Conclusions**

These findings suggest that the severity of CRS developing after haploHCT using PTCy-platform is associated with an increased frequency of bacterial infections, including BSI and CLABSI, and may lead to increased mortality. Pathogenesis of CRS after haploHCT remain an unmet need and potential mechanisms that may confer an increased frequency of infections are also discussed.

**References**

1. Marks DI, Abid MB. A Stem Cell Donor for Every Adult Requiring an Allograft for Acute Lymphoblastic Leukemia? Biol Blood Marrow Transplant. 2017;23(2):182-183.

2. Bashey A, Zhang X, Sizemore CA, et al. T-cell-replete HLA-haploidentical hematopoietic transplantation for hematologic malignancies using post-transplantation cyclophosphamide results in outcomes equivalent to those of contemporaneous HLA-matched related and unrelated donor transplantation. J Clin Oncol. 2013;31(10):1310-1316.

3. Abboud R, Keller J, Slade M, et al. Severe Cytokine-Release Syndrome after T Cell-Replete Peripheral Blood Haploidentical Donor Transplantation Is Associated with Poor Survival and Anti-IL-6 Therapy Is Safe and Well Tolerated. Biol Blood Marrow Transplant. 2016;22(10):1851-1860.

4. Lee DW, Gardner R, Porter DL, et al. Current concepts in the diagnosis and management of cytokine release syndrome. Blood. 2014;124(2):188-195.

5. Park JH, Romero FA, Taur Y, et al. Cytokine Release Syndrome Grade as a Predictive Marker for Infections in Patients With Relapsed or Refractory B-Cell Acute Lymphoblastic Leukemia Treated With Chimeric Antigen Receptor T Cells. Clin Infect Dis. 2018;67(4):533-540.

6. Hill JA, Li D, Hay KA, et al. Infectious complications of CD19-targeted chimeric antigen receptor-modified T-cell immunotherapy. Blood. 2018;131(1):121-130.

7. Cho C, Perales MA. Rapid identification of cytokine release syndrome after haploidentical PBSC transplantation and successful therapy with tocilizumab. Bone Marrow Transplant. 2016;51(12):1620-1621.

8. Slade M, Goldsmith S, Romee R, et al. Epidemiology of infections following haploidentical peripheral blood hematopoietic cell transplantation. Transpl Infect Dis. 2017;19(1).

9. Crocchiolo R, Bramanti S, Vai A, et al. Infections after T-replete haploidentical transplantation and high-dose cyclophosphamide as graft-versus-host disease prophylaxis. Transpl Infect Dis. 2015;17(2):242-249.

10. Fayard A, Daguenet E, Blaise D, et al. Evaluation of infectious complications after haploidentical hematopoietic stem cell transplantation with post-transplant cyclophosphamide following reduced-intensity and myeloablative conditioning: a study on behalf of the Francophone Society of Stem Cell Transplantation and Cellular Therapy (SFGM-TC). Bone Marrow Transplant. 2019.

**Ethics Approval**

The study was approved by Froedtert & the Medical College of Wisconsin Institutution‘s Ethics Board, approval number PRO00032170.

**Fig. 1 (abstract P605).**
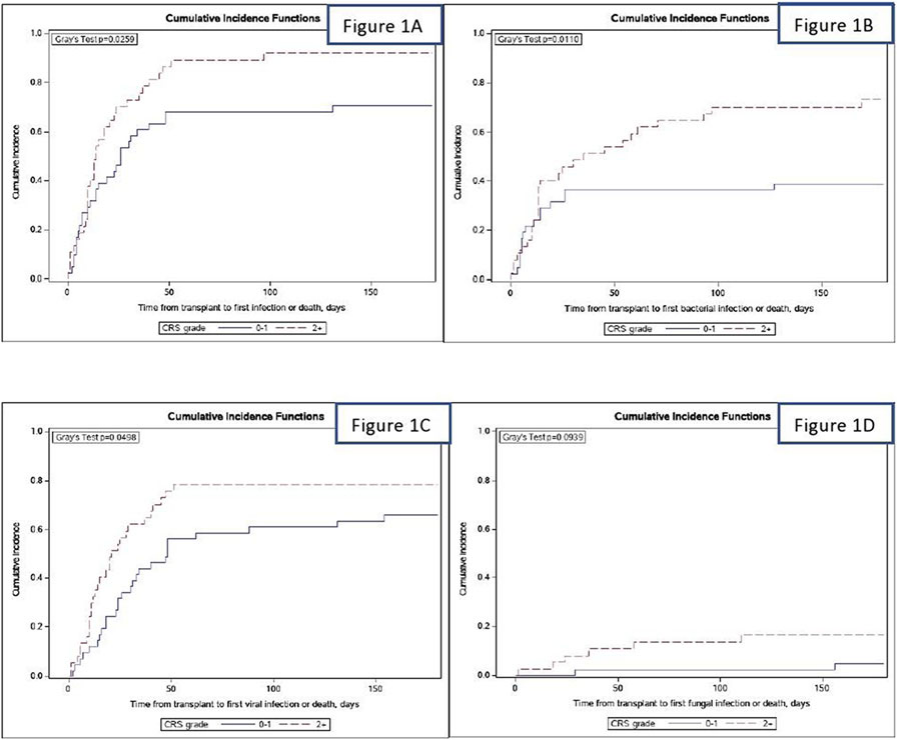
See text for description

**Fig. 2 (abstract P605).**
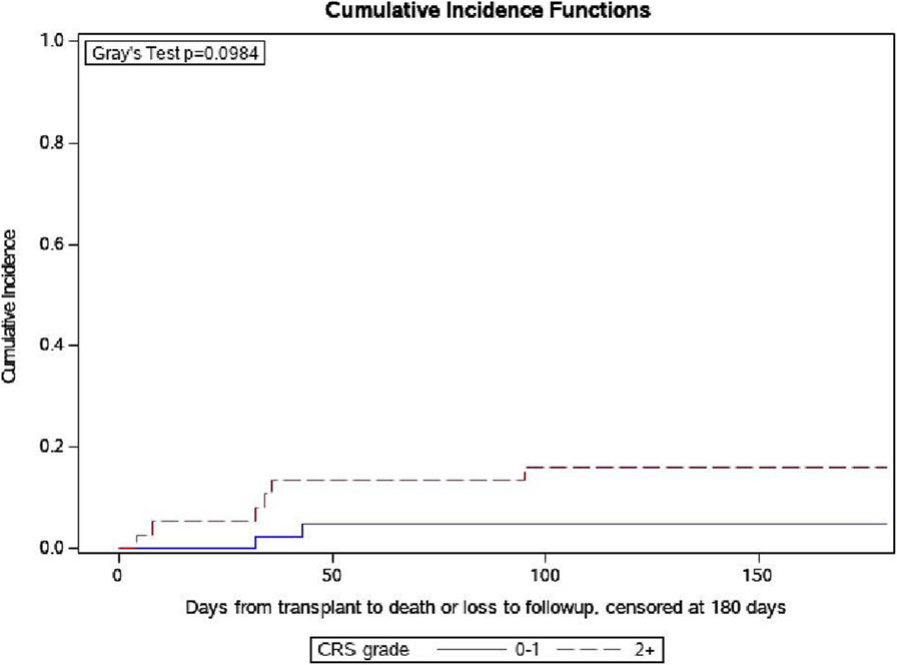
See text for description

**Table 1 (abstract P605).**
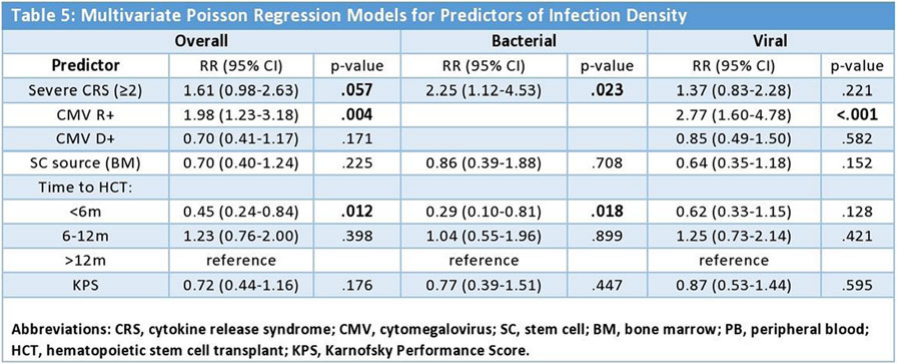
See text for description

#### P606 ICOSL on human dendritic cells is critical for priming T cell responses

##### Deena Maurer, MS^1,2^, Juraji Adamik, PhD^3^, Patricia Santos, PhD^2^, Jian Shi, MD^2^, Jennifer Taylor, PhD^2^, Michael Shurin, PhD^2^, John Kirkwood, MD^2^, Walter Storkus, PhD^2^, Lisa Butterfield, PhD^3^

###### ^*1*^*University of Pittsburgh School of Medicine, Pittsburgh, PA, United States;*^*2*^*University of Pittsburgh, Pittsburgh, PA, United States* ; ^*3*^*Parker Institute, San Francisco, CA, United States*

####### **Correspondence:**Lisa Butterfield (lbutterfield@parkerici.org)

**Background**

Metastatic melanoma patients successfully treated with combination immunotherapy using checkpoint blockade often display pre-existing antitumor immunity at time of initial treatment. Cancer vaccination can be used to promote anti-tumor T cell responses in patients lacking such immunity.

**Methods**

To generate optimal anti-tumor immune responses in melanoma patients, we previously developed a dendritic cell (DC)-based adenovirus-engineered vaccine encoding three common melanoma-associated antigens: Tyrosinase, MART-1, and MAGE-A6. This vaccine was used to treat 35 patients in a Phase 1 clinical trial. Immature DC were matured using IFN + LPS, and then transduced with the recombinant adenovirus yielding the vaccine. Patients received three intradermal injections of vaccine over one month. In addition to standard phenotyping and cytokine testing, a human genome RNA microarray was used to transcriptionally profile the DC vaccines produced for each patient. Cryopreserved patient and healthy donor monocytes were also examined in functional assays.

**Results**

Patient (matured and adenovirally-transduced) monocyte-derived DC expressed reduced levels of cell surface ICOSL (a costimulatory molecule) vs. DC generated from healthy donors (HD). Microarray analysis of patient cells revealed that ICOSLG mRNA gene expression was increased post-maturation and viral transduction, and differently expressed between HD and patient cells.

ICOSL surface protein showed a positive trend with the ability of adenovirally-transduced DC to elicit specific CD8+ T cell responses in vitro to Tyrosinase and MART-1. Using a CMV p65 antigen-based model, we observed reduced activation of naïve, CMV-specific CD8+ and CD4+ T cells in seronegative donors when primed using antigen-loaded autologous DC treated with anti-ICOSL antibody vs. an IgG control.

Microarray analysis further suggested that (matured and adenoviral-transduced) patient DC exhibit dysregulated NFĸB signaling vs. DC isolated from HD, and non-canonical NFκB signaling known to transactivate ICOSL. Pharmacologic inhibition of NFĸB signaling in DC from HD also resulted in decreased expression of surface ICOSL. We also observed that patient vaccine DC expression of NLRP2 (a natural NFĸB inhibitor), negatively correlated with clinical outcome.

**Conclusions**

Reduced levels of ICOSL on patient vaccine DC may limit their ability to prime anti-tumor immune responses in vitro (and in vivo). DC-expressed ICOSL appears important for specific T cell priming and is at least partially regulated by intrinsic canonical NFĸB signaling pathway. These data identify critical DC stimulatory pathways and inform the next generation DC-based vaccines capable of promoting superior anti-tumor benefits in advanced-stage melanoma patients.

**Acknowledgements**

This study utilized the UPMC Hillman Cancer Center’s Immunologic Monitoring and Cellular Products Laboratory shared facility, supported in part by award P30 CA047904.

This work is funded by the NIH SPORE IN SKIN CANCER Grant 4P50CA121973-09, the Melanoma Skin Cancer T32 Award 129297 (08/1/2017 – 07/31/2017), and the CTSI TL1 Pre-doctoral Scholar Fellowship, TL1 TR001858 (10/01/2018 – 09/30/2019).

**Trial Registration**

NCT01622933

**Ethics Approval**

The study was approved by the University of Pittsburgh's Ethics Board, approval number IRB12010416.

#### P607 Tumor derived IL-6 promotes early-stage myeloid derived suppressor cells (eMDSCs) via SOCS3 deficiency-related differentiation arrest in breast cancer

##### Wenwen Zhang, Jinpu Yu

###### Tianjin Tumor Hospital, Tianjin, China

####### **Correspondence:** Jinpu Yu (jinpu_yu@hotmail.com)

**Background**

Early stage myeloid-derived suppressor cells (eMDSCs) are a newly identified immunosuppressive cell subtype, but less is known about the generation of eMDSCs yet. In this study we elucidated that tumor-derived IL-6 manipulated the development and function of eMDSCs in vitro and vivo.

**Methods**

We constructed IL-6 knockdown 4T1 mammary tumor-bearing BALB/C models and myeloid lineage specific SOCS3 knockout C57BL/6 model. The system of eMDSCs induction was established, in which bone marrow cells were cocultured with 4T1 cells to induce eMDSCs in vitro.

**Results**

We identified a subpopulation of CD11b+Gr-1-F4/80-MHCII- eMDSCs in situ in IL-6 knockdown 4T1 mammary tumor-bearing mice models, which displayed more potent suppression on T cell immunity than conventional CD11b+Gr-1+ MDSCs. The proportion of eMDSCs in tumor tissues was highly correlated with the tumor size and the numbers of lung metastatic nodules. Bone marrow cells were co-cultured with 4T1 cells to induce eMDSCs in vitro, but IL-6R Ab treatment significantly decreased the proportion of eMDSCs. Tumor-derived IL-6 inhibited the expression of SOCS3 and persistently activated the JAK/STAT3 signaling pathways, which further caused the differentiation arrest of myeloid linkage and comparable immunosuppressive capacity of eMDSCs. The conditional myeloid SOCS3 knockout C57BL/6 (SOCS3mKO)mice was constructed to study SOCS3-related amplification of eMDSCs. The eMDSCs increased significantly in SOCS3mKO mice compared to SOCS3fl/fl mice, in which myeloid differentiation arrest was observed. The miRNA array screening indicated miR155-5p was the predominant regulator of SOCS3-related differentiation arrest in eMDSCs. We demonstrated that miR155-5p suppressed transcription factors C/EBPβ and PU.1, and thus promoted the development of eMDSCs. Transfecting miR155-5p inhibitor in myeloid precursors could efficiently reduce the number of eMDSCs and recover myeloid differentiation in vitro.

**Conclusions**

Tumor derived IL-6 activated the JAK/STAT3/ miR155-5p pathway in myeloid precursors by inducing SOCS3 deficiency, which consequently downregulated C/EBPβ and PU.1, induced myeloid differentiation arrest, and promoted the development of eMDSCs.

**Acknowledgements**

This work was supported by National Natural Science Foundation of China (grant numbers: 81872143, 81702280, 81472473, 81272360) and Key Project of Tianjin Health and Family Planning Commission (16KG126).

### Immune-Stimulants and Immune Modulators

#### P608 ATOR-1017, a 4-1BB antibody developed for tumor-directed immunotherapy of cancer

##### Doreen Werchau, BS^1^, Anna Rosen, MSc^2^, Mia Eriksson^1^, Sofia Järnum, PhD^1^, Christina Furebring, PhD^1^, Karin Enell Smith, PhD^1^, Karin Enell Smith, PhD^1^

###### ^1^Alligator Bioscience AB, Lund, Sweden; ^2^Alligator Bioscience, Lund, Sweden

####### **Correspondence:** Karin Enell Smith (kae@alligatorbioscience.com)

**Background**

ATOR-1017 is a Fcγ-receptor (FcγR) crosslinking dependent agonistic IgG4 antibody targeting the costimulatory receptor 4 1BB. 4-1BB is highly expressed on tumor infiltrating CD8+ T effector cells (T effs) in several cancer indications. By binding to 4-1BB, ATOR-1017 can enhance the activity of tumor reactive T effs and NK cells within the tumor and induce a potent anti-tumor attack. 4-1BB is an attractive target for immunotherapy, even though 4-1BB targeting antibodies in clinical development so far have been suffering from toxicity or poor efficacy. Therefore, ATOR-1017 was designed to improve tolerability and to enhance efficacy.

**Methods**

Human 4-1BB knock-in transgenic mice with established murine colon carcinoma MC38 tumors were used to demonstrate anti-tumor efficacy and immune cell infiltration with FACS after systemic treatment with ATOR-1017. In vitro, immune activation (IFN-γ production) of purified human immune cells from healthy donors with ATOR-1017 was analyzed using FcγR expressing cells for crosslinking in the presence of human IgG.

**Results**

ATOR-1017 reduced tumor growth and improved survival dose dependently in human 4-1BB knock-in transgenic mice with established tumors. No tumor growth was detected after tumor re-challenge in the complete responders previously treated with ATOR-1017, but in the naïve untreated control group only. These results demonstrated that ATOR-1017 induced a long-lasting immunological memory. The immunological response induced by ATOR-1017, leading to an efficient tumor eradication, consisted of a significant immune cell infiltration and a high T eff to T regulatory cell ratio within the tumor specifically but not in the spleens. In vitro it was demonstrated that immune activation with ATOR-1017 was blocked dose dependently in the presence of human IgG, due to saturation of the FcγRs which blocked crosslinking of ATOR-1017.

**Conclusions**

ATOR-1017 induced a potent tumor-directed immune response, leading to an efficient tumor eradication and survival and development of a long-lasting immunological memory. ATOR-1017 is FcγR crosslinking dependent with an activation profile that is expected to minimize the risk for inducing systemic immune activation and toxicity, by directing the immune response to the tumor tissue and tumor draining lymph nodes where 4-1BB as well as certain FcγRs are highly expressed. The FcγR crosslinking-dependency will also reduce the risk for immune activation in the circulation, due to the high concentration of endogenous circulating IgG which will compete with ATOR-1017 for binding to the FcγRs [1,2]. A first-in-human phase I study of intravenously administered ATOR-1017 is planned for 2019.

**References**

1. Preithner S, Elm S, Lippold S et al. High concentrations of therapeutic IgG1 antibodies are needed to compensate for inhibition of antibody-dependent cellular cytotoxicity by excess endogenous immunoglobulin G. Mol Immunol 2006; 43:1183-1193.

4. Eigenmann MJ, Karlsen TV, Krippendorff BF et al. Interstitial IgG antibody pharmacokinetics assessed by combined in vivo- and physiologically-based pharmacokinetic modelling approaches. J Physiol 2017; 595:7311-7330

**Ethics Approval**

The study was approved by Institutional Animal Care and Use Committee (IACUC) of China, approval number 10278L001.

#### P609 Fc-silenced bispecific antibodies targeting PD-L1 and 4-1BB combine checkpoint blockade and T-cell co-stimulation to promote anti-tumor activity

##### Alexander Muik, PhD^1^, Isil Altintas, PhD^2^, Friederike Gieseke^1^, Theodora Salcedo^2^, Saskia Burm^2^, Mustafa Diken^1^, Christian Grunwitz^1^, Danita Schuurhuis^2^, Sebastian Kreiter^1^, David Satijn^2^, Özlem Türeci^1^, Esther Breij, PhD^2^, Ugur Sahin^1^ , Maria Jure-Kunkel^2^

###### ^1^BioNTech SE, Mainz, Germany; ^2^Genmab, Utrecht, Netherlands

####### **Correspondence:** Ugur Sahin (ugur.sahin@biontech.de), Maria Jure-Kunkel (mjk@genmab.com)

**Background**

PD-1/PD-L1-targeting monoclonal antibodies (mAbs) have shown impressive responses in a wide range of tumors, although only in a subset of patients, leaving room for improvement. 4-1BB agonistic mAbs are potent T-cell co-stimulators and showed promising results in pre-clinical cancer models. However, no 4-1BB agonist has advanced beyond early phase II studies, due to dose-limiting hepatitis or lack of monotherapy efficacy. Fc-silenced bispecific antibodies (bsAbs) targeting PD-L1 and 4-1BB may overcome these limitations by combining immune checkpoint inhibition with conditional agonistic activity.

**Methods**

Fc-silenced PD-L1x4-1BB bsAbs (anti-mouse surrogate: mbsAb-PD-L1x4-1BB; anti-human: DuoBody®-PD-L1x4-1BB [GEN1046]) were obtained by controlled Fab-arm exchange of PD-L1 and 4-1BB mAbs. Activity of the mouse-specific mbsAb-PD-L1x4-1BB was tested in vivo in an ovalbumin-specific OT-1 T-cell adoptive transfer and vaccination model in C57BL/6 mice, and in efficacy studies in CT26 and MC38 tumor-bearing BALB/c and C57BL/6 mice, respectively. Tolerability was assessed in C57BL/6 mice and compared to treatment with a 4-1BB agonistic mAb. Induction of T-cell proliferation and cytokine production by DuoBody-PD-L1x4-1BB was analyzed in primary human T-cell assays in vitro and using patient-derived tumor-infiltrating lymphocytes (TILs) ex vivo. The non-clinical safety profile of DuoBody-PD-L1x4-1BB was assessed in cynomolgus monkeys.

**Results**

Surrogate mbsAb-PD-L1x4-1BB induced significant expansion of adoptively transferred OT-1 T-cells upon ovalbumin vaccination in proof-of-concept studies in vivo. In a therapeutic setting, mbsAb-PD-L1x4-1BB induced long-term tumor remission in up to 90% of CT26 and MC38 tumor-bearing mice, associated with expansion of tumor-specific CD8+ T-cells. mbsAb-PD-L1x4-1BB was well tolerated, while an Fc-active mouse 4-1BB agonistic mAb, alone or with a PD-L1-specific mAb, induced a significant increase in serum transaminase levels and liver immune infiltrates.

DuoBody-PD-L1x4-1BB enhanced T-cell proliferation and pro-inflammatory cytokine production in vitro, and was more potent than checkpoint blockade alone or the combination of Fc-silenced PD-L1- and 4-1BB-specific mAbs. 4-1BB agonist activity of DuoBody-PD-L1x4-1BB was strictly conditional and dependent on simultaneous binding of both targets. DuoBody-PD-L1x4-1BB increased TIL expansion ex vivo. DuoBody-PD-L1x4-1BB showed a favorable safety profile in cynomolgus monkeys at doses up to 30 mg/kg (1q3wx2).

**Conclusions**

Dual targeting of PD-L1 and 4-1BB with an Fc-silenced bsAb combines immune checkpoint blockade and conditional T-cell co-stimulation in one molecule. This distinctive mechanism of action results in potent anti-tumor activity without inducing hepatotoxicity in mice and enhances proliferation of human primary T-cells and TILs in vitro and ex vivo. The clinical safety of DuoBody-PD-L1x4-1BB is currently being assessed in patients with solid cancers in a first-in-human trial (NCT03917381).

**Ethics Approval**

Cynomolgus monkey studies were performed at Charles River Laboratories, Tranent, UK, in accordance with the EU legislations described in Directive 2010/63/EU. This study was licensed by UK Home Office under the Animals (Scientific Procedures) Act 1986 (approval number PBAD559F8, Toxicology of Pharmaceuticals, Protocol 15) and overseen by Genmab B.V.

All mice studies were performed by BioNTech SE at its research facilities in Germany, and the mice were housed in accordance with German federal and state policies on animal research. All experiments were approved by the regulatory authorities for animal welfare in Germany.

The use of tumor tissue resections was approved by BioNTech SE’s Ethics Board, approval number 837.309.12 (8410-F).

#### P610 Claudin18.2 bispecific T-cell engager for gastric cancer immunotherapy

##### Swarna Pandian, PhD^1^, Zusheng Li^1^, Eugene Chan, MD^1^, Robert Markelewicz, MD^1^, Timothy Wyant, PhD^2^, Sribalaji Lakshmikanthan, PhD^2^

###### ^1^Abpro Corporation, Woburn, MA, United States; ^2^AbproCorporation, Lexington, MA, United States

####### **Correspondence:** Sribalaji Lakshmikanthan (sribalajilakshmikanthan@gmail.com)

**Background**

Claudin18.2 (CLDN18.2), a gastric epithelial junction protein, has been discovered as a potential therapeutic target for stomach cancers. The aim of our study was to elucidate mechanism of action for ABP150, a CLDN18.2 specific CD3 engager. Specifically, we assessed the ability of ABP150 to be redirected to CLDN18.2 tumors followed by T-cell dependent killing.

**Methods**

CLDN18.2 specific clones were obtained by Abpro’s proprietary platform [1]. The clone selection criteria involved Claudin-isoform specific binding and off-target screening; HTS-flow cytometry based cross-reactive studies using ≥5000 other proteins expressed on HEK293T cell system. The bispecific T-cell engager for ABP150 antibody was engineered as described [1]. The built constructs were transiently transfected into HEK-293S cells grown in FreeStyle 293 Expression Medium followed by purification using Protein A IgG Purification Kit. Purified ABP150 was subjected to Size Exclusion Chromatography (SEC-HPLC) to assess its monomeric state. Relative binding of ABP150 to CLDN18.2 positive tumors and CD3 expressing Jurkat-T cell line was performed using flow cytometry. Following manufacturer’s protocol, T-cell activation and T-cell Dependent Cytotoxicity (TDCC) were examined by T Cell Activation Bioassay (NFAT) and CellTiter-Glo luminescent cell viability assay kits respectively.

**Results**

Out of 7 hybridomas, 2 clones, ABP150-1 and ABP150-2 were finalized based on relative binding affinity and specificity to CLDN18.2. Flow cytometric experiments demonstrated dose-dependent binding to CLDN18.2 positive cells exclusively. No binding to CLDN18.1 isoform was observed. Their ability to bind to T-cells were comparable to ABP100 [2, 3] suggesting functional monovalent binding to CD3. In the off-target screening experiments, both clones showed no cross-reactivity to extracellular membrane proteins. SEC-HPLC analysis displayed ≥95% of monomeric protein with no aggregations. In T-cell activation bioassay, both ABP150 clones presented a dose dependent T cell activation. Further, TDCC experiments resulted in dose dependent tumor killing only in the presence of CLDN18.2 expressing tumors. In absence of CLDN18.2 tumors, no toxicity was observed.

**Conclusions**

Lead CLDN18.2 clones, ABP150-1 and ABP150-2 as T-cell engagers showed CLDN18.2 specific binding and functionally weak CD3 binding (in the absence of target) resulting in strong TDCC response only in the presence of CLDN18.2 tumors. Taken together, we conclude that these two clones are expected to efficiently clear CLDN18.2 tumors without cytokine release syndrome.

**References**

1. Lakshmikanthan, S., et al., Novel tetravalent bispecific T-cell engaging antibodies for cancer immunotherapy. Journal of Clinical Oncology, 2019. 37, no. 15_suppl.

2. Boudot, A., et al., ABP-100: A Tetravalent Bispecific T-cell Engaging Antibody for HER2+ Solid Tumors. Annals of Oncology, 2018. Volume 29 Supplement 8.

3. Lopez-Albaitero, A., et al., Overcoming resistance to HER2-targeted therapy with a novel HER2/CD3 bispecific antibody. Oncoimmunology, 2017. 6(3): p. e1267891.

#### P611 Layer-by-layer nanoparticles for interleukin-12 delivery

##### Antonio Barberio, BSE, Sean Smith, Santiago Correa, Cathy Nguyen, Paula Hammond, Darrell Irvine, PhD, Mariane Melo, Talar Tokatlian

###### MIT, Cambridge, MA, United States

####### **Correspondence:** Paula Hammond (hammond@mit.edu)

**Background**

Interleukin-12 (IL-12) is a particularly compelling target for cytokine therapy, as it showed promise in preclinical tests. However, it failed in the clinic to high schedule dependent toxicities, including two patient deaths. An alternative approach is to deliver IL-12 in a nanoparticle (NP) delivery vehicle. Yet, cytokines pose a difficult challenge for NP delivery, as they require efficiently loading labile proteins, maintaining activity on cytokine membrane receptors from a NP that is often internalized, and achieving high association with tumors to prevent systemic leakage. Layer-by-layer (LbL) is a simple electrostatic method of layering polymers onto materials to alter the properties of the carrier. Through incorporation of different polymers, LbL offers the opportunity to address the challenges of cytokine NP delivery.

**Methods**

A single chain variant of IL-12 was loaded onto liposomal nanoparticles via Ni:HIS interactions and layered via the LbL process (Figure 1A). Particles were assessed for material properties in vitro including biological activity via their ability to stimulate an interferon-y response. Particles were further tested in vivo for their toxicity and efficacy in multiple tumor models.

**Results**

LbL IL-12 NPs with poly-L-arginine and poly-L-glutamic acid layers (PLE-IL-12-NPs) were able to efficiently load and deliver active cytokine. PLE-IL-12-NPs showed drastically reduced toxicity compared to carrier-free IL-12. Healthy mice treated with PLE-IL-12-NPs showed no differences in weight to PBS and unloaded NPs, while carrier-free IL-12 treated mice lost 10% of their body weight by the fifth dose when treated daily. Furthermore, PLE-IL-12-NP treated mice showed significantly less systemic cytokine response when compared to carrier-free IL-12. Importantly, PLE-IL-12-NPs also showed no loss in anti-tumor activity at these same doses when delivered against both subcutaneous MC38 and the more difficult to treat HM-1 ovarian cancer cell line. Finally, at significantly higher doses, carrier-free IL-12 again showed significant toxicity while the PLE-IL-12-NPs showed no differences to the controls and lead to pronounced antitumor efficacy in the MC38 model (Figure 1B,C).

**Conclusions**

PLE-IL-12-NPs show promise as an IL-12 therapy. Most importantly, PLE-IL-12-NPs were able to significantly reduce the toxicity of IL-12 therapy while maintaining its efficacy. Of noted importance, PLE-IL-12-NPs were also able to show efficacy in ovarian cancer, which has been refractory to many immunotherapies in the past. Finally, although the above results demonstrate activity with IL-12, the described particle uses no IL-12 specific methods and can likely be applied across many other cytokines or combinations

**Ethics Approval**

These experiments were approved by the Massachusetts Institute of Technology Committee on Animal Care (CAC).

**Fig. 1 (abstract P611).**
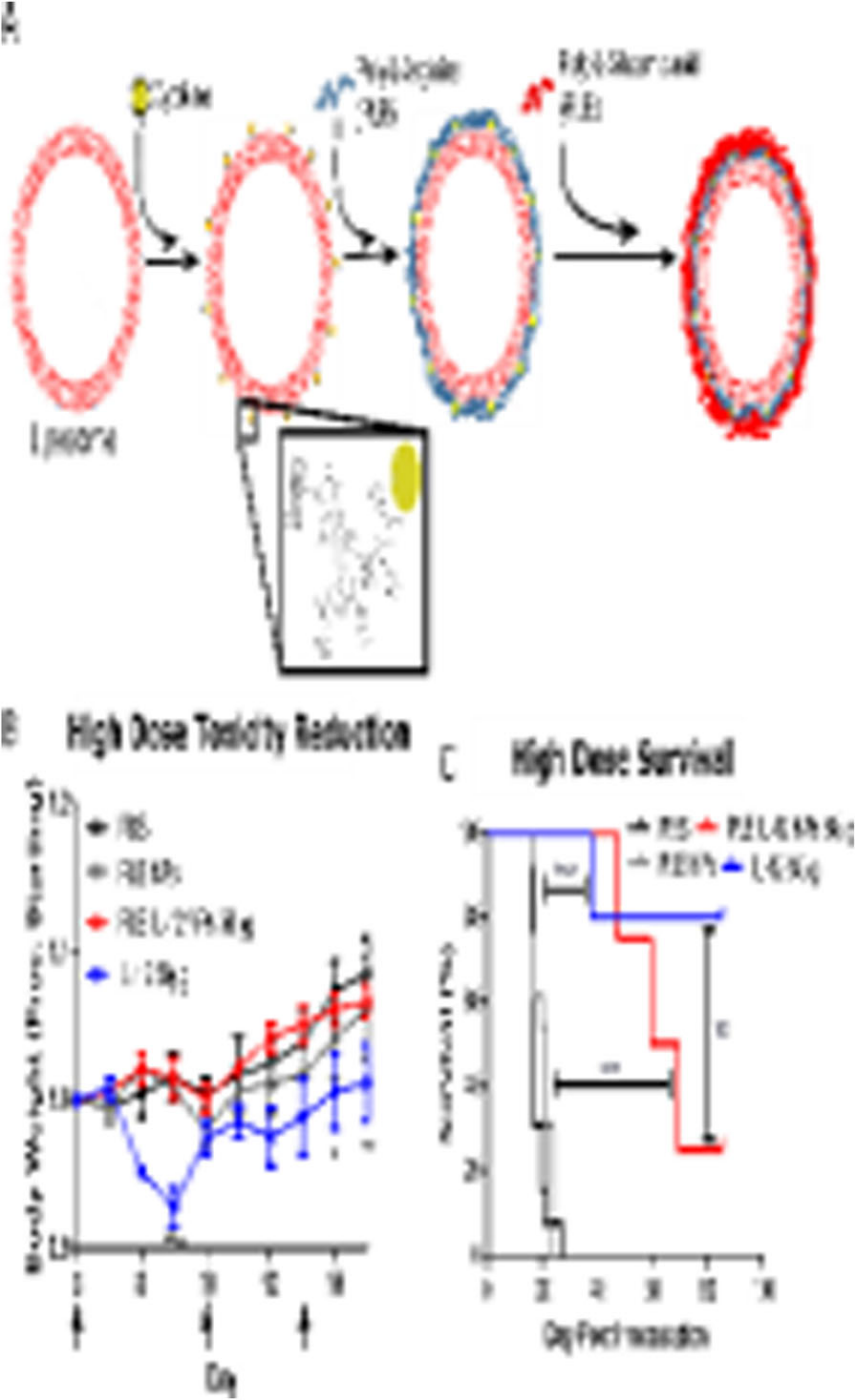
PLE-IL-12-NPs reduce toxicity and maintain efficacy

#### P612 Using a novel synthetic biology platform to generate a homogenous dipegylated IL-10 with native potency

##### Carolina Caffaro, PhD, Jerod Ptacin, PhD, Yelena Pavlova, Jasmine Nguyen, Kristine San Jose-Galli, Gavin Hong, Lina Ma, PhD, Nicole Acuff, Taylor Ismaili, MS, Kelsea Loescher, Ingrid Joseph, PhD, Jill Mooney, PhD, Joseph Leveque, MD, Marcos Milla, PhD

###### Synthorx, La Jolla, CA, United States

####### **Correspondence:** Marcos Milla (mmilla@synthorx.com)

**Background**

Upon TCR stimulation via tumor-specific antigen presentation, CD8+ cells upregulate the IL-10 receptor (IL-10R) and become responsive to this cytokine, leading to localized T effector cell (Teff) proliferation and tumor infiltration along with the release of IFN and cytolytic products. The presence of 13 lysine residues in IL-10 and participation of its N-terminal region in IL-10R1 and R2 engagement presents a challenge to conventional PEG strategies. To harness IL-10 potential as an immuno-oncology therapeutic, the generation of IL-10 variants with extended half-life is desired.

**Methods**

The Synthorx synthetic biology platform expands the genetic alphabet (EGA) by one base pair that generates new codons for site-specific incorporation of novel amino acids at any position within the polypeptide, which enables the design of biotherapeutics with improved drug properties. We have applied this platform to rapidly screen and identify residues within the IL-10 amino acid sequence for optimized PEG bioconjugation distal from the IL-10R binding sites, producing a series of single IL-10 variants that were then pegylated. Biochemical characterization of those pegylated variants by SPR and SEC-MALS together with in vitro and ex vivo potency profiling led to the identification of homogeneous Synthorin IL-10 dimers with extended in vivo half-life and minimal interference with IL-10 receptor engagement and activation.

**Results**

Analytical characterization of purified IL-10 Synthorins showed that they are homogeneous, and dimeric pegylated variants. Among them, THOR-1003 and THOR-1009 showed comparable in vitro potency to native IL-10 for proliferation of the human MC/9 cell line, and in a human recombinant cell assay directly measuring receptor activation, underscoring the importance of choosing the right site for pegylation on the agonist activity of IL-10. Other sites tested resulted in compounds exhibiting up to 1,000-fold decrease in potency in cell-based assays. THOR-1003 and THOR-1009 have been selected for further testing, focusing on the release of IFN and cytolytic enzymes from primary human CD8+ T cells, following stimulation with antigen-presenting cells displaying a CMV peptide epitope. Pharmacokinetics and pharmacodynamics profiling of these molecules in vivo is ongoing.

**Conclusions**

We have demonstrated that our synthetic biology platform can generate functional, pegylated Synthorin IL-10 variants that retain the folding and structural stability of native IL-10. Two of those Synthorins, THOR-1003 and THOR-1009, exhibit physiochemical properties similar to native IL-10, and functional properties consistent with a fully active agonist that can be dosed in vivo for durable pharmacodynamic effects.

#### P613 Discovery of pharmacologically differentiated interleukin 15 (IL-15) agonists employing a synthetic biology platform

##### Carolina Caffaro, PhD, Jerod Ptacin, PhD, Robert Herman, Lina Ma, PhD, David Chen, Ingrid Joseph, PhD, Marcos Milla, PhD

###### Synthorx, Inc, La Jolla, CA, United States

####### **Correspondence:** Marcos Milla (mmilla@synthorx.com)

**Background**

IL-15, distinctly from IL-2, induces proliferation and survival of CD8+ T memory (Tmem) cells, activates natural killer (NK) cells, and enhances antibody-driven cellular toxicity without the expansion of immunosuppressive regulatory CD4+ T cells (Treg). However, its high potency and resulting toxicity, combined with its short half-life, has limited its potential as an immunotherapeutic agent. We hypothesize that extension of the half-life of IL-15 could provide persistence of signaling for CD8+ Tmem expansion, while reducing potential Cmax-associated toxicities. Further modulation of the interaction with specific receptor chains (the private IL-15Rα and/or IL-2/15Rβ) by pegylation might enable tuning of drug exposure (AUC) for preferential stimulation of different cell subsets and therapeutic effects.

**Methods**

We applied the Synthorx Expanded Genetic Alphabet platform to the rational design of pegylated IL-15 compounds with extended half-life and differential interaction with the IL-15Rα and IL-2/15Rβ. In vitro biochemistry and cell bioassays and ex vivo profiling in mouse and human immune cells were used to select initial IL-15 Synthorin candidates for in vivo PK/PD studies.

**Results**

THOR-924 is a pegylated IL-15 Synthorin with similar binding affinity to IL-15Rα and IL-2/15Rβ, and comparable signaling potency, relative to native IL-15. PK/PD responses in mice, demonstrated an extended half-life with sustained induction of pSTAT5 and Ki67 resulting in strong proliferation of peripheral CD8+ Tmem and NK cells, without proliferation of Treg cells. 90-100% of peripheral CD8+ T cells were positive for both pSTAT5 and Ki67 after a single IV dose of 0.3 mg/kg. Additional IL-15 Synthorins, THOR-918 and THOR-908, showed overall reduced potency or specific blockade of IL-15 interaction with IL-15Rα, respectively, by SPR. This selective interaction accounts for the differential in vitro and ex vivo potency of these compounds compared to native IL-15 and THOR-924. Ongoing in vivo studies will shed light on the potential pharmacologic advantages of such activity-modulated IL-15 Synthorins.

**Conclusions**

IL-15 Synthorins are biochemically differentiated forms of IL-15 with extended half-life and optimized pharmacologic parameters for balanced engagement of IL-15R vs IL-2/15R inducing proliferation and cytotoxicity of CD8 T and NK cells in vivo without significant activation of immune-suppressive Treg cells or toxicities associated with dosing of native IL-15. They offer the potential to fulfill the promise of IL-15 as a clinical agent for therapeutic induction of immune responses in the treatment of solid tumors.

#### P614 Development of a cancer gene therapy approach based on encoding a single chain IL-12 transgene in an oncolytic T-SIGn virus vector

##### Sam Illingworth^1^, Manuela Zonca^1^, Ana Silva^1^, Alicia Belmonte^1^, Nalini Marino^1^, Carla Cerqueira^1^, Lorna Slater^1^, Katy West^1^, Sara Siebel^2^, Samantha Bucktrout^2^, Fred Ramsdell, PhD^2^, Paul Cockle^1^, Brian Champion, PhD^1^

###### ^1^PsiOxus Therapeutics Ltd, Abingdon, United Kingdom; ^2^Parker Institute Cancer Immunotherapy, San Francisco, CA, United States

####### **Correspondence:** Brian Champion (brian.champion@psioxus.com)

**Background**

T-SIGn virus vectors for cancer gene therapy are transgene-modified variants of enadenotucirev (EnAd), an Ad11p/Ad3 chimeric group B adenovirus, which retain all the functional and epithelial tumor-selectivity properties of EnAd while also mediating the selective expression of transgenes by infected tumor cells. We have created T-SIGn viruses expressing a single chain variant of human IL-12 as the primary transgene modality; production of IL-12 by virus-infected tumor cells could be an effective way of driving T-cell and NK cell activity within the tumor microenvironment to promote effective anti-tumor immunity.

**Methods**

T-SIGn virus constructs were generated to express either the p35 and p40 chains of human IL-12 separately or as a linked single chain molecule using a short linker sequence. To enable tumor-selective expression of IL-12, transgenes were placed under the control of the virus major late promoter (MLP) which is activated following initiation of genome replication in permissive cells. Tumor cell lines and primary cells derived by gentle disaggregation of surgically excised tumor samples from carcinoma patients were used to evaluate the production and activities of the different IL-12 viruses.

**Results**

ELISA assays measuring levels of the full IL-12 p70 molecule or the specific p40 chain in infected tumor cell supernatants showed that the virus encoding separate IL-12 chains produced p40 very effectively but very little p70 was detected. By contrast the virus encoding the single chain variant effectively produced p70 at levels >100ng/ml. Using a HEK-Blue IL-12 signalling reporter assay, this p70 protein was shown to be functionally active and thus folded correctly. Primary cells derived from excised patient tumor samples were also evaluated and shown to produce IL-12 when treated with the single chain IL-12 encoding virus. The functional activity of virus encoded IL-12 was then further demonstrated by its ability to enhance the production of IFNγ by T-cells and NK cells using PBMCs and PBMC-derived T-cell cultures as well as cultures of primary tumor cells.

**Conclusions**

In this study, we have shown that a functionally active single chain variant of human IL-12 can be effectively produced by tumor cells infected with a T-SIGn virus encoding this transgene payload. Ongoing studies are evaluating several different cytokines and chemokines encoded together with the single chain IL-12 transgene for their ability to enhance the activities of IL-12, either directly or indirectly via their ability to recruit IL-12 responsive immune cells in order to optimize the potential for clinical activity.

#### P615 Novel immune-cell targeted IL2v to deliver IL-2R signaling to tumor reactive T cells via PD-1 whilst blocking the PD-1 pathway

##### Laura Codarri Deak^1^, Valeria Nicolini^1^, Laura Lauener, Mrs^1^, Stefan Seeber, Dr^2^, Marine Richard^1^, Esther Bommer^1^, Maria Karagianni, Mrs^1^, Johannes Sam, Dr^1^, Ramona Schlenker, Dr^2^, Marisa Mariani, Dr^1^, Petra Schwalie, Dr^3^, Sylvia Herter, PhD^1^, Marina Bacac, Dr^1^, Inja Waldhauer, Dr^1^, Anne Freimoser-Grundschober, Dr^1^, Volker Teichgräber, Dr^1^, Christian Klein, Dr rer nat^1^ , Pablo Umana, PhD^1^

###### ^*1*^*Roche Innovation Center Zurich, Zurich, Switzerland;*^*2*^*Roche Innovation Center Munich, Munich, Germany* ; ^*3*^*Roche Innovation Center Basel, Basel, Switzerland*

####### **Correspondence:** Christian Klein (christian.klein.ck1@roche.com)

**Background**

Interleukin-2 (IL-2) has been the first effective cancer immunotherapy to treat metastatic melanoma and renal cell carcinoma for more than three decades. Although only a small fraction of patients benefits from such a treatment, the anti-tumor responses to IL-2 therapy tend to be complete, durable and the responding patients seem to be cured. Unfortunately, the applicability of IL-2 in the clinic is limited by its toxicity (i.e. capillary leak syndrome), unfavourable pharmacokinetic and bio-distribution in the lymphatic system rather than in the tumour, and the detrimental activation/expansion of regulatory T cells (Tregs) through IL2Ra (CD25) binding.

**Methods**

To overcome these limitations, we developed a novel immunocytokine that is constituted of a high affinity bivalent blocking anti-PD-1 antibody and a heterodimeric silent Fc-part fused to a monomeric IL-2 variant (IL2v).

**Results**

IL2v binding to CD25 is abolished, and therefore the binding to Tregs and endothelial cells is reduced. The anti-PD-1 antibody, having roughly 50-fold higher affinity than IL-2v, dictates the preferential cis-delivery of IL-2v to PD-1 positive T cells, such as antigen-experienced tumour-reactive T cells. In addition, the blocking of the PD-1 inhibitory pathway synergizes with the IL-2 signalling cascade translating into more potent T cell activation and effector functions than untargeted IL-2v. Due to reduced expression levels of PD-1 on Tregs compared to tumour-reactive T cells, PD1-IL2v binds preferentially to the latter rescuing them from Treg-mediated suppression. All these properties enabled PD1-IL2v to eradicate aggressive tumours and provide a significant survival benefit over combination of the parental molecules in the tested mouse models.

**Conclusions**

PD1-IL2v is a differentiated immune cell-targeted IL-2v which promotes an effective and long-term anti-tumor immune-response by delivering IL-2v to PD-1+ tumor-specific T cells

#### P616 A novel anti-tumor anti-PDL1-IL7 immunocytokine targeting lymphocytes

##### Feifei Cui, PhD, Lei Fang, Haijuan Gu, Yuanyuan Yang, Zhengyi Wang, Bingshi Guo

###### I-Mab Biopharma, Shanghai, China

####### **Correspondence:** Lei Fang (lei.fang@i-mabbiopharma.com)

**Background**

Lymphocyte counts in the peripheral immune system and tumor are correlated with positive clinical outcomes in PD-(L)1 immunotherapy. Interleukin 7 (IL7) is an immune homeostasis cytokine for T cells. Treatment with recombinant human IL7 preferentially expands recent thymic emigrants, naive and central memory T cells, but spares regulatory T cells. Here we constructed a series of immunocytokine L1I7 comprised of a PDL1 blocking mAb fused with an IL7 molecule with varying degree of attenuated potency. The aim of the study was to identify the right molecule that achieves enhanced biodistribution at the tumor site and an optimal balance between synergistic anti-tumor activity and an acceptable safety profile.

**Methods**

PDL1 binding affinity and antagonist function of L1I7 were evaluated by BiaCore and PD1/PDL1 cell-based functional assay, respectively. IL7 receptor (IL7R) binding and internalization were evaluated in human primary CD4+ T cells by flow cytometry (FACS). IL7 potency was evaluated by p-STAT5 signaling assay and human CD4+ T cell proliferation assay. The in vitro synergistic effect of L1I7 was evaluated in the human mixed lymphocyte reaction (MLR) assay. The in vivo tracking assay was conducted by injecting fluorescence-labeled L1I7 into HCC827-transplanted CD34+ hematopoietic stem cells humanized mice. The in vivo efficacy of L1I7 was investigated in B16F10 melanoma syngeneic mice model with surrogate L1I7. Absolute numbers of tumor-infiltrating CD4+ T and CD8+ T cells were analyzed by FACS.

**Results**

L1I7 series of immunocytokine maintained PDL1 binding and antagonist function when compared with PDL1 mAb. As compared with wildtype L1I7, L1I7 variants showed attenuated IL7 activity as evidenced by reduced IL7R binding/internalization, p-STAT5 activation and CD4+ T cell proliferation. Both wildtype and the attenuated variants were more effective in promoting CD4 T cell activation than anti-PDL1 or IL7 monotreatment in MLR. Similar with PDL1 mAb, L1I7 was enriched in the tumor site whereas IL7 was widely distributed in in vivo tracking experiment. In an anti-PDL1-resistant B16F10 tumor model, L1I7 showed superior efficacy as compared with either anti-PDL1, IL7 alone or in combination, which was correlated with an increased number of tumor-infiltrating CD4+ T and CD8+ T cells.

**Conclusions**

The novel immunocytokine L1I7 is designed to target poor and non-responders to PD-(L)1 treatment and even PD-(L)1 treatment relapsed patients. L1I7 represents potentially a best-in-class molecule with enhanced local immunostimulation achieved by concurrent PD-(L)1 blockade and IL7 stimulation. L1I7 is undergoing preclinical development with an aim to enter clinical studies in 2021.

#### P617 Delivery of interleukin-2 to the acidic tumor microenvironment by ultra-pH sensitive nanoparticles for immunotherapy

##### Xinliang Ding, PhD, Jason Miller, PhD, Ashley Campbell, Jonathan Almazan, Tian Zhao

###### OncoNano Medicine Inc., Dallas, TX, United States

####### **Correspondence:** Tian Zhao (tzhao@onconanomed.com)

**Background**

Immunotherapy has become a powerful strategy for cancer treatment. Immunomodulators such as interleukin-2 (IL-2) can induce anti-tumor immune responses, but their clinical applications are limited by unfavorable pharmacokinetic properties that can elicit serious dose-limiting toxicities (e.g. vascular leak syndrome). [1] We have developed an ultra-pH sensitive nanoparticle platform that targets the acidic tumor microenvironment (TME) with high specificity. At normal physiological pH, the nanoparticles exist as intact micelles, while at acidic tumor pH, they dissociate into single unimers, releasing the payload. The feasibility of selectively targeting the acidic TME by this platform has been successfully validated by imaging of different types of tumors in a Phase 1 clinical trial. [2-4] Using this platform, we have encapsulated and delivered IL-2 to the TME to improve its pharmacokinetic properties.

**Methods**

We have developed an ultra-pH sensitive nanoparticle (ONM-400) with similar pH sensitivity to ONM-100, which is being tested in a Phase 2 clinical trial for intraoperative imaging of tumors. Multiple strategies have been exploited to encapsulate IL-2 including mixing, single emulsion, acid/base titration, double emulsion/solvent evaporation, and covalent conjugation. The ONM-400•IL-2 complexes were purified by ultracentrifugation and characterized by dynamic light scattering and transmission electron microscopy. Encapsulation efficiency (EE) and drug loading (DL) were measured for each formulation using ELISA or gel electrophoresis. In addition, IL-2 was labeled with a near-infrared dye (IRDye® 800CW) to study the in vivo tumor accumulation and biodistribution after intravenous injection in mice bearing head and neck tumors using a LI-COR Pearl® camera.

**Results**

ONM-400 nanoparticles were able to encapsulate IL-2 with high loading. The EE and DL ranged from 81.8% to 35.7% and 8.2% to 3.5%, respectively. In vivo imaging of ONM-400•IL-2 showed significantly higher tumor accumulation and less bladder signal compared to free IL-2 at the same dose after IV injection (**Fig.** 1A). ONM-400•IL-2 provided longer and increased IL-2 exposure in tumor than free IL-2 which decreases overtime. Quantitative analysis of the tumor signal at different time points indicated an alteration of pharmacokinetics for IL-2 after encapsulation by the ONM-400 nanoparticle (**Fig.** 1B). Biodistribution at 24h further revealed a lower accumulation in kidney but higher accumulation in tumor for ONM-400•IL-2 versus free IL-2.

**Conclusions**

The ONM-400 nanoparticles can efficiently encapsulate IL-2 and alter its pharmacokinetics in vivo. These results suggest a great potential for using the ultra-pH sensitive nanoparticle technology to delivery immunomodulators to improve their efficacy and safety profile.

**References**

1. Riley R, June C, Langer R, Mitchell M. Delivery technologies for cancer immunotherapy. Nat Rev Drug Discov. 2019; 18: 175-196.

2. Steinkamp P, Voskuil F, Koller M, van der Vegt B, Doff J, Zhao T, Hartung J, Jayalakshmi Y, Sumer B, Gao J, Witjes M, van Dam G. Image guided surgery for tumor agnostic detection of solid tumors using the pH activated micellar imaging agent ONM-100: The Shine study. Cancer Res. 2019; 79 (13 Supplement): CT018.

3. Voskuil F, Steinkamp P, Koller M, van der Vegt B, Doff J, Zhao T, Hartung J, Jayalakshmi Y, Sumer B, Gao J, Witjes M, van Dam G. Image-guided surgery for tumor agnostic detection of solid tumors using the pH-activated micellar imaging agent ONM-100. J Clin Oncol. 2019; 37 (15 Supplement): 3068.

4. Steinkamp P, Voskuil F, Koller M, van der Vegt B, Doff J, Zhao T, Hartung J, Jayalakshmi Y, Sumer B, Gao J, Witjes M, van Dam G. Image guided surgery for tumor detection in breast cancer using the PH activated micellar tracer ONM-100: The SHINE study. Cancer Res. 2019; 79 (4 Supplement): P2-14-29.

**Ethics Approval**

All animal experiments were reviewed and approved by, and performed in accordance with, the UT Southwestern Institutional Animal Care and Use Committee under Animal Protocol Number: 2015-101065.

**Fig. 1 (abstract P617).**
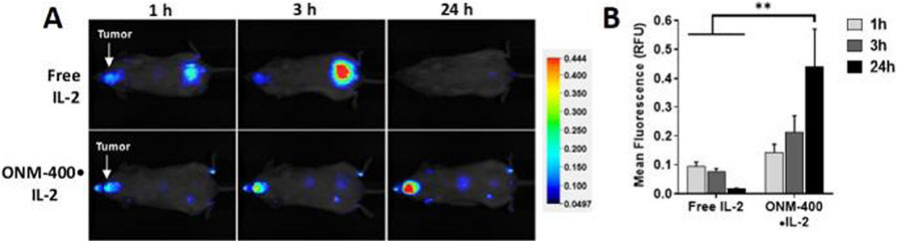
See text for description

#### P618 MK1169, a peptide unrelated to IL-2, is a potent IL-2Rβγc receptor agonist

##### William Dower, PhD, Steven Cwirla, BA, Blake Williams, MS, Praechompoo Pongtornpipat, MS, Prarthana Joshi, ME, Sandra Wang, BSc, Alice Bakker, BA, Michael Needels, PhD, Ronald Barrett, PhD

###### Medikine, Menlo Park, CA, United States

####### **Correspondence:** William Dower (bdower@medikine.com), Ronald Barrett (rbarrett@medikine.com)

**Background**

Efforts to modify IL-2 for immuno-oncology applications have focused on alterations that reduce interaction with the Rα subunit of the receptor complex via mutation, chemical modification, or complexation with antibodies or the Rα-ectodomain. IL-2Rβγc-agonists may also incorporate features to reduce side effects associated with peak drug levels, and to extend duration of action.

**Methods**

Peptides were selected from recombinant peptide libraries to identify molecules binding simultaneously to the β and γc subunits of the IL-2 receptor. Identified peptides were synthesized by both chemical and recombinant means, and evaluated for properties of IL-2Rβγc activation in IL-2 responsive cells lines and human lymphocytes.

**Results**

We report the discovery and optimization of small synthetic peptides, unrelated to IL-2, that simultaneously bind IL-2Rβ and γc subunits to induce IL-2R signaling. The peptides do not bind IL-2Rα and are therefore IL-2Rβγc-selective agonists with MW less than 5000D. Synthetic compound MK1169 activates the JAK-STAT (pSTAT5), MAPK (pERK1/2), PI3K (pAKT) pathways, and induces proliferation in cell lines NK92 (Rαβγc) and TF-1β (Rβγc). Agonism is attributable to direct activation of the IL-2Rβγc receptor: (1) conversion of IL-2Rβ-deficient parental TF-1 cells to the IL-2-responsive TF-1β line, by transfection of Rβ, imparts MK1169 responsiveness, and (2) neutralizing antibodies against IL-2 and IL-15 do not inhibit the agonist activity of MK1169. MK1169 exhibits potency similar to that of IL-2 in cell lines expressing Rβγc, but not Rα. MK1169 induces pSTAT5 in human lymphocyte subpopulations with potency bias similar to that of IL-2 variants that are attenuated in Rα binding. MK1169-treated human PBMCs cause PD-L1 up-regulation, and killing of tumor cell lines. MK1169 retains activity when fused to IgG and Fc molecules, and when pegylated; providing options for pharmacokinetic (PK) enhancement. We have also shown that fusions of MK1169 to pembrolizumab and cemiplimab exhibit full activity in both PD-1 inhibition and IL-2Rβγc agonist activity, indicating the feasibility of incorporating MK1169 into fusion proteins with dual pharmacology. The synthetic peptide, and peptide fused to the C-terminus of Fc exhibit promising in vitro serum stability, and the in silico predicted potential for immunogenicity is very low.

**Conclusions**

PK-enhanced MK1169 is an attractive alternative to engineered IL-2 variants. Dual pharmacology therapeutics incorporating MK1169 also offer promise for immuno-oncology. To our knowledge, this work is the first demonstration of small peptide agonists of a heterodimeric cytokine receptor.

**Ethics Approval**

The use of human PBMC in this study was authorized under Minimal Risk Research Related Activities at Stanford Blood Center (SQL 79075)

#### P619 NKTR-255, a polymer-conjugated IL-15 receptor agonist, enhances efficacy of therapeutic monoclonal antibodies with ADCC activity in solid tumor models

##### Saul Kivimae, PhD, Saul Kivimae, PhD, Takahiro Miyazaki, MS, Rhoneil Pena, Wildaliz Nieves, PhD, Andrew Moffett, Phi Quach, BS PhD, Lawrence Chinn, Marlene Hennessy, BS, Loui Madakamutil, Jonathan Zalevsky, PhD

###### Nektar Therapeutics, San Francisco, CA, United States

####### **Correspondence:** Takahiro Miyazaki (tmiyazaki@nektar.com)

**Background**

NKTR-255 is a polymer-conjugated IL-15 pathway agonist that retains binding affinity to the alpha subunit of the IL-15 receptor and exhibits reduced clearance. Sustained IL-15 pathway activation leads to expansion and activation of NK cells potentially enabling combinatorial immunotherapies including tumor targeting antibodies that mediate tumor killing by antibody dependent cellular cytotoxicity (ADCC). Here we investigate NKTR-255 induced enhancement of NK cell ADCC dependent tumor cell killing in vitro and in vivo in the tumor environment when combined with tumor-targeting monoclonal antibodies in solid tumors.

**Methods**

NKTR-255 dose dependent enhancement of NK cell cytotoxicity as a single agent or in combination with cetuximab was evaluated in vitro in co-cultures of purified human NK cells with HCT-116 or FaDu tumor cells. Target cell killing was evaluated by flow cytometry based viability scoring. In vivo tumor growth inhibition was measured in xenograft models with diverse histologies including colorectal (Colo25, HT29, HCT-116), lung (H1975), epidermal (A431), ovarian (SKOV3) and gastric (NCI-N87). Nude or SCID mice in Balb/c background were subcutaneously inoculated on the flank with tumor cells. NKTR-255 was administered intravenously weekly at 0.3mg/kg. Cetuximab or trastuzumab were administered at dose levels and schedules optimized for each tumor type with a once or twice a week schedule. Treatment was initiated at established ~100-150mm3 tumor volumes. Tumor growth inhibition was assessed by tumor size caliper measurements.

**Results**

NKTR-255 pre-treatment of NK cells led to enhanced cytotoxic activity against HCT-116 and FaDu cells in vitro. Additional enhancement of NK cell dependent killing of tumor cells was observed in the presence cetuximab antibody in the co-culture assay demonstrating NKTR-255 dependent enhancement of NK cell ADCC. In agreement with in vitro activity, NKTR-255 combination treatment with cetuximab or trastuzumab showed significant tumor growth inhibition in multiple models. Notably tumor models resistant to cetuximab single agent treatment (HT-29 and HCT-116) showed significant growth inhibition in combination treatment with NKTR-255. Trastuzumab targeted tumors showed significant enhancement of antibody mediated tumor growth inhibition in combination with NKTR-255 including 100% complete responses in the SKOV3 model.

**Conclusions**

NKTR-255 treatment of NK cells enhanced ADCC activity against tumor cells in vitro and in vivo efficacy of ADCC inducing antibodies in human solid tumor xenograft models. Furthermore, antibody single agent treatment resistant tumor models showed tumor growth inhibition in combination treatment with NKTR-255 suggesting potential for increasing response rates of therapeutic antibodies with ADCC mechanism of action.

**Ethics Approval**

All procedures in this study were reviewed and approved by Nektar Therapetuics Institutional Animal Care and Use Committee.

#### P620 Complementary effects of RNA encoded, extended half-life IL2 and IL7 synergize in modulating T cell responses and antitumoral efficacy

##### Lena Kranz^1^, Mathias Vormehr, PhD^1^, Jan Dieckmann, PhD^2^, Claudia Lindemann^2^, David Eisel^1^, Alexander Muik, PhD^1^, Sina Fellermeier-Kopf^1^, Friederike Gieseke^1^, Bodo Tillmann^3^, Sonja Witzel^3^, Marlen Lepper^2^, Mustafa Diken^3^, Sebastian Kreiter, MD^3^, Veronika Jahndel^2^, Özlem Türeci^2^, Ugur Sahin^2^

###### ^*1*^*BioNTech RNA Pharmaceuticals, Mainz, Germany;*^*2*^*BioNTech SE, Mainz, Germany* ; ^*3*^*TRON-Translational Oncology gGmbH, Mainz, Germany*

####### **Correspondence:** Ugur Sahin (ugur.sahin@biontech.de)

**Background**

Interleukin-2 (IL2) is known to support effector T cell differentiation, proliferation and survival. A particular challenge of IL2 for cancer treatment is the preferential stimulation of regulatory T cells (Tregs) constitutively expressing the high-affinity IL2 receptor alpha chain (CD25). Tregs can undermine the antitumoral activity of tumor-specific effector T cells only transiently expressing CD25. Interleukin-7 (IL7) is a homeostatic cytokine that plays an important role in lymphopoiesis, survival and memory formation, and can increase the expression of CD25 on antigen-specific T cells. Prompted by their complementary effects, we investigated the potential synergy between these two cytokines on the T cell level and on antitumoral activity.

**Methods**

We designed nucleoside-modified, RNA encoded, extended half-life IL2 and IL7, formulated as nanoparticles for intravenous delivery. The potency of IL2 and IL7 to enhance antitumoral activity of a liposome-formulated RNA vaccine [1] was determined for both cytokines separately and in combination. Therapeutic success and changes in blood T cell subset composition were determined in subcutaneous, syngeneic tumor-bearing mice.

**Results**

Using nanoparticle formulated, nucleoside-modified RNA coding for extended half-life IL2 and IL7, we demonstrated that IL2 and IL7 each increases the number and frequency of antigen-specific CD8+ T cells in combination with an antigen-specific RNA vaccine compared to the vaccine alone. While the effect on expansion of antigen-specific T cells of IL2 is strong and immediate, IL7 requires repetitive cycles to generate its maximal effect. In addition, IL7 turned out to expand CD8+ T cells with specificities for targets other than the vaccine-encoded antigen better than IL2. As expected, IL2 strongly expanded the number and frequency of Tregs. In contrast, IL7 reduced the fraction of Tregs among CD4+ T cells. The combination of IL2 with IL7 proved to be powerful in overcoming their individual shortcomings: The combination boosted the number of antigen-specific CD8+ T cells beyond the levels of IL2 alone, supported the expansion of CD8+ T cells not specific for the vaccine-encoded target, and further improved the ratio of antigen-specific CD8+ T cells over Tregs. These findings translated into superior antitumoral activity of the combination in subcutaneous mouse tumor models.

**Conclusions**

Based on the complementary mode of action and the promising preclinical data, clinical evaluation will be pursued.

**References**

1. Kranz, L. M., Diken, M., et al. Systemic RNA delivery to dendritic cells exploits antiviral defence for cancer immunotherapy. Nature 534, 396–401 (2016).

**Ethics Approval**

Experimental group sizes were approved by the regulatory authorities for animal welfare after being defined to balance statistical power, feasibility and ethical aspects. All mice were kept in accordance with federal and state policies on animal research at BioNTech SE.

#### P621 Nanoparticle-mediated tumor cell expression of mIL-12 via systemic gene delivery treats syngeneic models of murine lung cancers

##### Hye-Hyun Ahn, PhD^1^, Christine Carrington^2^, Heng-wen Liu^1^, Yizong Hu^1^, Christy Ng^1^, Hwanhee Nam^1^, Andrew Park^1^, Catherine Stace^3^, Will West, PhD^2^, Hai-Quan Mao, PhD^1^, Martin Pomper, MD, PhD^1^, Chris Ullman, PhD^2^ , IL Minn, PhD^1^

###### ^1^Johns Hopkins University, Baltimore, MD, United States; ^*2*^*Cancer Targeting Systems, Newton Centre, MA, United States* ; ^*3*^*Cancer Targeting Systmes, Newton Centre, MA, United States*

####### **Correspondence:**Chris Ullman (chris.ullman@cts-bioscience.com), IL Minn (iminn1@jhmi.edu)

**Background**

Therapies through systemic injection of recombinant immunomodulatory cytokines have not been successful due to low therapeutic index. Here we describe the use of a polycation-encapsulated DNA plasmid to locally deliver therapeutic cytokine genes under the control of the cancer-selective rat progression elevated gene 3 (PEG-3) promoter1,2 to tumors in the lungs of diseased mice. [1-3]

**Methods**

A cGMP grade polycationic carrier, in vivo-jetPEI® was chosen to formulate the plasmid DNA construct to aid rapid transition to clinical studies. Clinically compatible vectors were created that featured a CpG-free backbone containing a kanamycin resistant gene, scaffold/matrix attachment regions and CpG-free payload genes. Plasmids were then packaged into size-controlled nanoparticles with in vivo-jetPEI® using a scalable flash nanocomplexation (FNC) manufacturing method3 for production of lyophilized, shelf-stable nanoparticles with an excellent reproducibility under quality assurance and quality control standards.

**Results**

These well-defined pDNA/lPEI nanoparticles, delivered systemically, were well-tolerated in mice and accomplished significant reduction in levels of acute inflammatory cytokines compared to an equivalent CpG-containing plasmid. In vivo transfection of metastatic lesions in the lung by nanoparticles expressing firefly luciferase from the PEG-3 promoter could be detected bioluminescent imaging. Furthermore, intravenous delivery of nanoparticles expressing murine single-chain interleukin-12, under the control of PEG-3 promoter, significantly improved the survival of mice in two different models of lung cancer with no marked symptoms of systemic toxicity (Figure 1).

**Conclusions**

Increased survival in both an orthotopic and a metastatic model of lung cancer using clinically relevant nanoparticles raises the possibility of translation to human therapy.

**Acknowledgements**

This study is partially supported by a research contract from Cancer Targeting Systems, Inc. (CTS) and an NIH grant R01EB018358.

**References**

1. Bhang, H. E. et al. Tumor-specific imaging through progression elevated gene-3 promoter-driven gene expression. Nat. Med, 2011; 17(1), 123-129.

2. Minn, I. et al. Molecular-genetic imaging of cancer. Adv, Can. Res. 2014; 124:131-69.

3. J. L. Santos et al., Continuous production of discrete plasmid DNA-polycation nanoparticles using flash nanocomplexation. Small, 2016; 12(45), 6214-6222.

**Ethics Approval**

All animal experiments were performed in accordance with protocols approved by Animal Care and Use Committees of the Johns Hopkins University and of the Pennsylvania State University.

**Fig. 1 (abstract P621).**
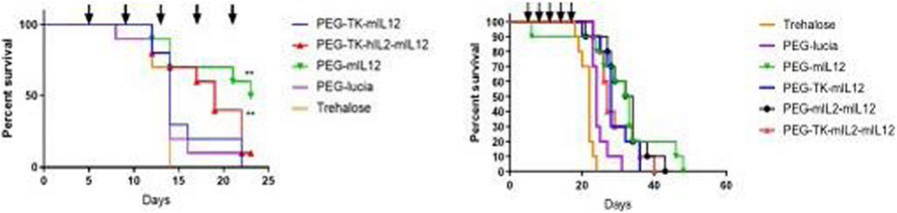
Expression of mIL-12 provides survival benefits

#### P622 Characterization and comparison of NKTR-255, a polymer-conjugated IL-15 versus IL-15 superagonist

##### Takahiro Miyazaki, MS , Mekhala Maiti, PhD, Sara Sheibani, Joanna Wilczek, Olga Zemska, Peiwen Kuo, PhD, Saul Kivimäe, Marlene Hennessy, BS, Phi Quach, BS PhD, Ping Zhang, MS PhD, Thomas Chang, PhD, Loui Madakamutil, Jonathan Zalevsky, PhD

###### Nektar Therapeutics, San Francisco, CA, United States

####### **Correspondence:** Takahiro Miyazaki (tmiyazaki@nektar.com)

**Background**

IL-15 is a cytokine that activates and provides survival benefit to T and NK cells and has great potential for the treatment of cancer. Exploiting the therapeutic value of native IL-15 has been challenging due to its unfavorable pharmacokinetic properties and poor tolerability. Several IL-15-based proteins aimed to overcome these problems are currently in development. NKTR-255 is a polymer-conjugated native IL-15 that is designed to retain a binding affinity to IL-15 receptor alpha (IL-15Ralpha), while the most clinically advanced IL-15-based protein is an IL-15 superagonist (IL-15 mutein/IL-15Ralpha-Fc complex). We investigate the pharmacological properties of NKTR-255 and the IL-15 superagonist to explore differentiations between the IL-15Ralpha dependent and independent cytokines.

**Methods**

For cellular receptor binding, IL-15Ralpha-overexpressing HEK-293 cells were incubated with rhIL-15, NKTR-255 or IL-15 superagonist to measure surface binding of the cytokines by flow cytometry. The biological activity of cell surface bound cytokines was measured as pSTAT5 induction following co-culture with human PBMCs. Surface expression of IL-15Rs was measured by flow cytometry at 20 min or 2 hr following incubation with each of the cytokines. In functional assays, human PBMCs were incubated with each of the cytokines for 1, 2 or 5 days for surface marker characterization, cytokine production or proliferation assay, respectively.

**Results**

NKTR-255 binds to surface IL-15Ralpha on the receptor-overexpressing HEK-293 cells. Maximal binding was similar to that of rhIL-15, while IL-15 superagonist showed negligible binding. In concordance, the HEK-293 cells incubated with NKTR-255 induced pSTAT5 on human PBMCs, while the HEK-293 cells incubated with IL-15 superagonist failed to induce pSTAT5. Similarly to rhIL-15, incubation of NKTR-255 with human whole blood reduced surface staining of IL-15Ralpha on T and NK cells; in contrast IL-15 superagonist reduced IL-2Rbeta staining. Each of the cytokines dose-dependently induced proliferation of both NK cells and T cells; the EC50 value of NKTR-255 was approximately 20-fold higher than the EC50 values of both rhIL-15 and IL-15 superagonist. Interestingly, NKTR-255-treated NK cells showed more rounds of division than IL-15 superagonist-treated NK cells at the highest concentration. The maximal responses of NKTR-255 for induction of surface CD107a expression in NK cells and IFNgamma production were also higher than those of IL-15 superagonist.

**Conclusions**

NKTR-255 is a novel IL-15Ralpha dependent molecule that provides enhanced PK and PD properties relative to the native IL-15 cytokine while IL-15 superagonist is a direct engager to IL-2Rbeta complex. These results indicate the potential of NKTR-255 pharmacology to capture the full spectrum of native IL-15 biology.

#### P623 Bempegaldesleukin in combination with local radiation and systemic checkpoint blockade induces a robust systemic anti-tumor immunity

##### Alexander Pieper, BS^1^, Alexander Rakhmilevich, MD, PhD^1^ , Dan Spiegelman^1^ , Ravi Patel, MD, PhD^1^ , Claire Baniel^1^ , Amy Erbe, PhD^1^ , Jacquelyn Hank, PhD^1^ , Deborah Charych, PhD^2^ , Willem Overwijk, PhD^3^ , Zachary Morris, MD, PhD^1^ , Paul Sondel, MD, PhD^1^

###### ^1^University of Wisconsin-Madison, Madison, WI, United States; ^*2*^*Third Rock Ventures, San Francisco, CA, United States*; ^*3*^*Nektar Therapeutics, San Francisco, CA, United States*

####### **Correspondence:**Alexander Rakhmilevich (rakhmil@humonc.wisc.edu), Dan Spiegelman (dspiegelman2@wisc.edu), Ravi Patel (rpatel55@wisc.edu), Claire Baniel (cbaniel@gmail.com), Amy Erbe (aerbe@wisc.edu), Jacquelyn Hank (hank@humonc.wisc.edu), Deborah Charych dcharych@nektar.com), Willem Overwijk (wwoverwijk@gmail.com), Zachary Morris(zmorris@humonc.wisc.edu), Paul Sondel (pmsondel@humonc.wisc.edu)

**Background**

Bempegaldesleukin (NKTR-214) is a first-in-class, CD122-preferential interleukin-2 (IL2) pathway agonist being investigated for its potential ability to leverage the clinically validated IL2 pathway and selectively stimulate an immune response, without overactivating the immune system. We have previously shown a synergistic interaction between local low dose radiation therapy (RT) and NKTR-214 in mice bearing moderate sized melanoma tumors. This combination is less effective, however, in the presence of larger tumors and/or systemic disease. Checkpoint blockade effectively disables immune inhibitory functions, thereby enabling a stronger anti-tumor immune response. We hypothesized the addition of checkpoint blockade to [RT]+[NKTR-214] would help overcome the immune suppression in difficult models, allowing for a stronger anti-tumor effect.

**Methods**

To generate a model of bulky unresectable disease, C57BL/6 mice were inoculated with B78 melanoma cells on the right flank and tumors were allowed to grow for ~7 weeks (average tumor size ~800mm3). Mice were randomized and treated with 12 Gy external beam RT to the flank tumor and intravenous (IV) NKTR-214 or [RT]+[NKTR-214]+[α-CTLA-4]. To create a model of heterogeneous and disseminated metastatic disease, C57BL/6 mice were inoculated with B78 cells on the right flank and tumors were allowed to grow for ~4 weeks (average tumor size ~185mm3). On the day the treatments began, mice were randomized and injected IV with B16 Luciferase+ melanoma cells. B16 is parental to B78 melanoma, sharing some common antigens. Mice were treated with: 1) PBS, 2) [NKTR-214]+[α-CTLA-4], 3) [RT]+[α-CTLA-4], 4) [RT]+[NKTR-214]+[α-CTLA-4], or 5) [RT]+[NKTR-214]+[α-CTLA-4]+[T-cell depleting antibodies (α-CD4+α-CD8)]. Tumor growth and survival were monitored. Metastatic disease burden was evaluated by luciferase imaging.

**Results**

In very large B78 tumors (~800mm3), [RT]+[NKTR-214]+[α-CTLA-4] caused greater tumor regression than [RT] +[NTKR-214]. In a model of disseminated metastatic disease, [RT]+[α-CTLA-4] slowed primary tumor progression but had little effect on metastatic disease, demonstrated by the group’s similar survival kinetics to the PBS control. Adding [NKTR-214] to [RT]+[α-CTLA-4] not only caused primary tumor regression but also resulted in greater overall survival than all other groups in the B78 primary/B16 metastasis study. Control of the lung metastasis by [RT]+[NKTR-214]+[α-CTLA-4] was T cell-mediated. Though [NKTR-214]+[α-CTLA-4] demonstrated relatively weak control of the primary tumor, this combination slowed metastatic tumor burden displaying a prolongation of survival compared to vehicle and [RT]+[α-CTLA-4].

**Conclusions**

Our preliminary results suggest that [RT]+[NKTR-214]+[α-CTLA-4] has the ability to mount a T cell dependent anti-tumor response capable of regressing large unresectable tumors and disseminated, heterogeneous metastatic disease in murine melanoma models.

#### P624 Layer-by-layer nanoparticles for the non-toxic delivery of interleukin-12 to orthotopic ovarian cancer

##### Sean Smith^1^, Antonio Barberio, BSE^2^, Darrell Irvine, PhD^2^, Santiago Correa^3^, Cathy Nguyen^2^, Mariane Melo^2^, Talar Tokatlian, PhD^2^, Paula Hammond^2^

###### ^1^Massachusetts Institute of Technology, Cambridge, MA, United States; ^*2*^*MIT, CAMBRIDGE, MA, United States* ; ^*3*^*Stanford University, Cambridge, MA, United States*

####### **Correspondence:** Paula Hammond (hammond@mit.edu)

**Background**

Ovarian cancer represents an area of great unmet clinical need, with the 5-year survival rate for high grade ovarian cancer at just 25% for those with advanced disease and with only ~ 10% of patients responding to checkpoint inhibition or other immune therapeutics [1]. Thus, there remains a pressing need to explore alternative strategies to potentiate immunotherapies in ovarian cancer. One attractive class of therapeutics that could play such a role are pro-inflammatory cytokines. However, many proinflammatory cytokines have been under-utilized in ovarian cancer and other solid tumors, largely due to toxicity concerns. In this study we develop a nanoparticle to deliver interleukin-12 (IL-12), one of the most potent and toxic cytokines, to an orthotopic model of murine ovarian cancer. The layer-by-layer (LbL) method was used to modify liposomes bearing IL-12 with a polymeric surface chemistry that covers the cytokine to limit its accessibility outside of the tumor environment, enhances ovarian cancer targeting, and prevents nanoparticle internalization.

**Methods**

IL-12 nanoparticles were constructed as shown in Figure 1A. Briefly, HIS-tagged, single-chain IL-12 was loaded onto the surface of liposomes bearing Ni headgroups. The liposomes were then layered with positively charged poly-L-arginine followed by poly-L-glutamine (PLE). These constructs (PLE-IL12-NP) were fully characterized and tested in vitro. For in vivo testing, female B6C3F1 mice were implanted intraperitoneally with 1MM HM-1 ovarian cancer cells. Beginning 7 days after implantation, mice were treated intraperitoneally 1x/day for five days with PLE-IL12-NP (5 μg). Mice were tracked for tumor burden via luciferase expression, survival, and signs of toxicity.

**Results**

Incubation with splenocytes confirmed that PLE-IL-12-NPs retained the activity of IL-12. Toxicity studies in mice showed that at 5 μg the PLE-IL-12-NPs reduced toxicity in terms of weight loss (Figure 2B) and systemic cytokine production (data not shown). Indeed, the weight of mice treated PLE-IL-12-NPs was indistinguishable from vehicle treated mice. Antitumor studies against established metastatic ovarian tumors showed that PLE-IL-12-NPs were just as efficacious as soluble IL-12 (Figure 2C) extending survival over both vehicle and unlayered cytokine-bearing liposomes.

**Conclusions**

PLE-IL-12-NPs offer a simple approach to ameliorate the toxicity of IL-12. Indeed, this approach offers promise for the systemic delivery of potent cytokines to hard-to-treat tumors such as ovarian cancer. Ongoing studies are exploring the biodistribution following additional routes of administration, the immunological mechanisms of these responses, and the combination of this approach with checkpoint inhibition.

**References**

1. Odunsi K. Immunotherapy in ovarian cancer. Ann Oncol. 2017;28:1-7

**Fig. 1 (abstract P624).**
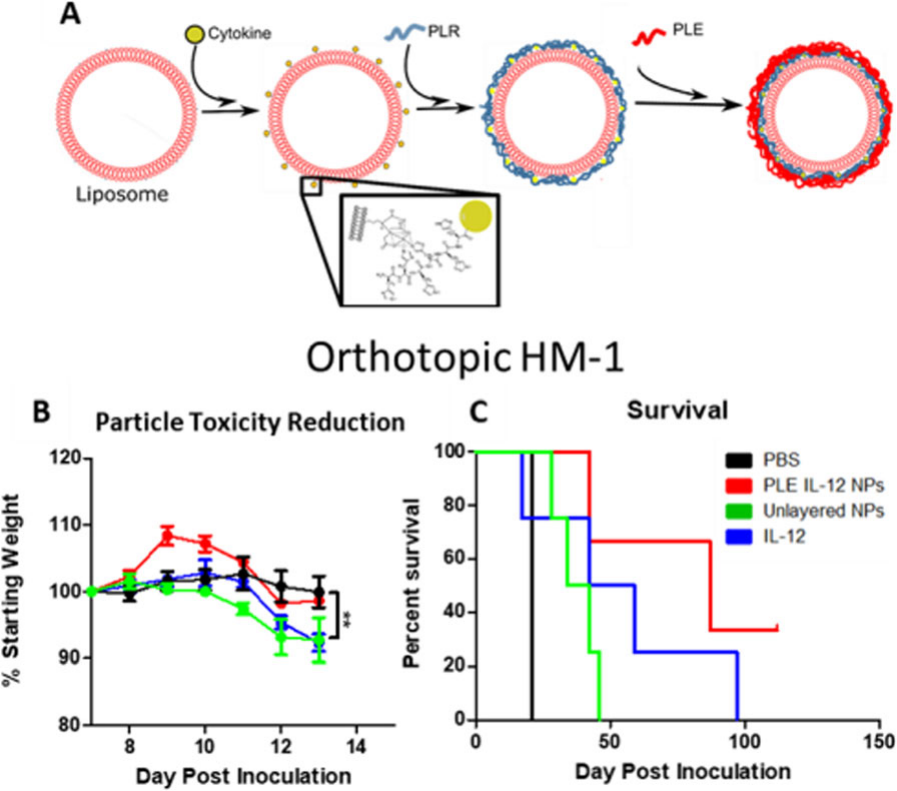
Layer-by-layer nanoparticles for IL-12 delivery

#### P625 KY1043, a novel PD-L1 IL-2 immunocytokine directed towards CD25, delivers potent anti-tumour activity in vitro and in vivo

##### Cassandra Van Krinks, PhD^1^, , Timothy Malcolm^1^, Morgane Lecointre^1^, Richard Brown^1^, Hanif Ali^1^, Hannah Craig^1^, Dirk Zahn^1^, Siobhan O'Leary^1^, Lucy Hepburn^1^, Rachael Kimber^1^, Nikole Sandy^2^, Alla Fane-Dremucheva^1^, Jamie Campbell, PhD, BS^3^, Volker Germaschewski, PhD, MSc^1^, Stephen Gillies, PhD^4^, , Matthew McCourt, BSc^1^

###### ^1^Kymab, Cambridge, United Kingdom; ^*2*^*Charles River Laboratories, Cambridge, United Kingdom*; ^*3*^*Abcam, Cambridge, United Kingdom* ; ^*4*^*Provenance Biopharmaceuticals, Carlisle, MA, United States*

####### **Correspondence:** Matthew McCourt (matthew.mccourt@kymab.com)

**Background**

IL-2 therapy has been approved for the use in certain cancers since 1989. The major limitation with this therapy is tolerability, with many patients experiencing grade 3 or higher adverse events. Approaches to improve this have focused on engineering molecules with selectivity towards the dimeric (CD122 and CD132) IL-2 receptor (IL-2R). However, this form of the receptor complex is widely expressed on peripheral naïve T cells as well as memory T cells and NK cells, potentially leading to unproductive stimulation of irrelevant cell populations and an increased possibility of systemic toxicity. KY1043 is a highly differentiated immunocytokine consisting of a neutralising anti-PD-L1 antibody, fused via its light chains to an attenuated IL-2, in which the balance of signaling has been adjusted to favour the trimeric form of the IL-2R, which contains CD25. We hypothesised that reduced binding to circulating cells may improve targeting to PD-L1+ cells in the tumour or lymphoid tissues while also targeting IL-2 to antigen-experienced T cells that express CD25 in the tumour microenvironment.

**Methods**

Tumour reactive HLA-A2+ CD4+ and CD8+ T cells were generated by co-culture with growth-arrested A375 cells, supplemented with IL-2 and IL-7. After 20 days, these tumour-directed T cells were cultured with fresh tumour cells in the presence of either KY1043, a non-targeted immunocytokine or the parental anti-PD-L1 antibody. Tumour cell growth was measured in real-time using the Incucyte S3™ system. In vivo, human PD-L1 knock-in (hPD-L1) mice bearing established MC38 hPD-L1 tumours were treated with KY1043.

**Results**

KY1043 induced T-cell-mediated killing of A375 tumour cells in vitro, with a higher potency compared to non-targeted immunocytokine or anti-PD-L1 alone, and comparable to that of anti-PD-L1 and equimolar amounts of recombinant native IL-2. In vivo, treatment with KY1043 led to a dose-dependent complete regression of hPD-L1-expressing MC38 tumours in hPD-L1 knock-in mice, with no significant adverse effects. Pharmacodynamic analysis confirmed proliferation of both effector (Teff) and regulatory (Treg) T cells in the periphery, with preferential expansion of Teff cells in the tumour. Immunological memory was demonstrated, as treated mice were resistant to re-challenge with MC38 but not to other tumours.

**Conclusions**

KY1043 induces potent T cell activation and can direct highly effective tumour killing in vitro and in vivo. These results challenge the dogma that selective binding to dimeric (CD122/CD132) IL-2R is required for tumour control and demonstrate that a CD25 directed immunocytokine may provide an advantageous therapeutic-index for the treatment of cancer.

**Acknowledgements**

We thank the Technology and Animal Sciences teams for support of knock-in mice and cell line generation, and the Antibody Discovery team for identification of the parental anti-PD-L1 antibody

**Ethics Approval**

This project was reviewed and approved by the Greater Manchester West Local Research Ethics Committee (LREC no. 2/NW/0553)

#### P626 Substantial improvement of cancer immunotherapy by an RNA encoded extended half-life Interleukin-2 variant

##### Mathias Vormehr, PhD^1^, Lena Kranz^1^, Alexander Muik, PhD^1^, Sina Fellermeier-Kopf^1^, Nadja Salomon^2^, Jan Diekmann^3^, Claudia Lindemann^3^, Sonja Witzel^2^, Marlen Lepper^3^, Friederike Gieseke^1^, Veronika Jahndel^3^, Özlem Türeci^3^, Ugur Sahin^3^

###### ^1^BioNTech RNA Pharmaceuticals GmbH, Mainz, Germany; ^*2*^*TRON – Translational Oncology at the University Medical Center of Johannes Gutenberg University, Mainz, Germany*; ^*3*^*BioNTech SE, Mainz, Germany*

####### **Correspondence:** Ugur Sahin (ugur.sahin@biontech.de)

**Background**

Interleukin-2 (IL2) supports the differentiation, proliferation, survival and effector functions of T cells [1–4]. Recombinant IL2 has been used for decades in the treatment of late stage malignant melanoma [5] and renal cell cancer [6,7]. Most patients with complete responses after IL2 treatment remain regression-free for more than 25 years after initial treatment, but overall response rates are low [8,9,7,5]. Recombinant IL2 treatment is complicated by its very short half-life and requirement of high and frequent dosing, which in turn potentiates side effects [10]. Furthermore, a particular challenge of IL2 for cancer treatment is the preferential stimulation of immunosuppressive regulatory T cells (Tregs), which can dampen anti-tumor immune responses and may counteract the beneficial effects of activated effector T cells.

**Methods**

We designed a nucleoside-modified RNA encoded IL2 variant with the goal of an extended half-life, reduced Treg bias and potent stimulation of effector T cells and NK cells. Activation and proliferation of human, cynomolgus or mouse T cells was characterized in vitro. IL2 variant serum levels after intravenous RNA nanoparticle administration were investigated in mice and cynomolgus monkeys. Subsequently, immune subset composition as well as therapeutic activity as monotherapy or in combination with cancer immunotherapies was determined in multiple syngeneic mouse tumor models.

**Results**

Characterization of the IL2 variant in vitro validated potent proliferation of CD8+ T cells, NK cells and a reduced Treg bias. Analysis of IL2 variant protein levels in blood of mice or non-human primates upon injection of RNA nanoparticles demonstrated drug exposure for several days. The IL2 variant mediated a substantial increase in murine CD8+ effector T cells and NK cell while Treg expansion was hardly detected in vivo. In syngeneic tumor-bearing mice, a spontaneous expansion of cancer (neo-epitope) specific T cells and a strong increase of the CD8+ T cell to Treg ratio translated into potent tumor control. When combined with T-cell vaccination anti-PD-1/PD-L1 checkpoint blockade, the IL2 variant significantly increased tumor specific T-cell expansion, further boosting anti-tumor efficacy.

**Conclusions**

We demonstrate substantial therapeutic activity of a RNA encoded, nanoparticle formulated IL2 variant in monotherapy or in combination with cancer immunotherapy. Patient recruitment for an open-label, clinical phase I/II, first-in-human trial is anticipated for the first half of 2020.

**References**

1. Gillis S, Smith KA: Long term culture of tumour-specific cytotoxic T cells. Nature 1977, 268:154–6.

2. Blattman JN, Grayson JM, Wherry EJ, Kaech SM, Smith KA, Ahmed R: Therapeutic use of IL-2 to enhance antiviral T-cell responses in vivo. Nat Med 2003, 9:540–7.

3. Bamford RN, Grant AJ, Burton JD, Peters C, Kurys G, Goldman CK, Brennan J, Roessler E, Waldmann TA: The interleukin (IL) 2 receptor beta chain is shared by IL-2 and a cytokine, provisionally designated IL-T, that stimulates T-cell proliferation and the induction of lymphokine-activated killer cells. Proc Natl Acad Sci U S A 1994, 91:4940–4.

4. Kamimura D, Bevan MJ: Naive CD8+ T cells differentiate into protective memory-like cells after IL-2 anti IL-2 complex treatment in vivo. J Exp Med 2007, 204:1803–12.

5. Atkins MB, Lotze MT, Dutcher JP, Fisher RI, Weiss G, Margolin K, Abrams J, Sznol M, Parkinson D, Hawkins M, et al.: High-dose recombinant interleukin 2 therapy for patients with metastatic melanoma: analysis of 270 patients treated between 1985 and 1993. J Clin Oncol 1999, 17:2105–16.

6. Rosenberg SA: Interleukin 2 for patients with renal cancer. Nat Clin Pract Oncol 2007, 4:497.

7. Klapper JA, Downey SG, Smith FO, Yang JC, Hughes MS, Kammula US, Sherry RM, Royal RE, Steinberg SM, Rosenberg S: High-dose interleukin-2 for the treatment of metastatic renal cell carcinoma : a retrospective analysis of response and survival in patients treated in the surgery branch at the National Cancer Institute between 1986 and 2006. Cancer 2008, 113:293–301.

8. Rosenberg SA: Raising the bar: the curative potential of human cancer immunotherapy. Sci Transl Med 2012, 4:127ps8.

9. Rosenberg SA, Yang JC, White DE, Steinberg SM: Durability of complete responses in patients with metastatic cancer treated with high-dose interleukin-2: identification of the antigens mediating response. Ann Surg 1998, 228:307–19.

10. Lotze MT, Chang AE, Seipp CA, Simpson C, Vetto JT, Rosenberg SA: High-dose recombinant interleukin 2 in the treatment of patients with disseminated cancer. Responses, treatment-related morbidity, and histologic findings. JAMA 1986, 256:3117–24.

**Ethics Approval**

Experimental group sizes were approved by the regulatory authorities for animal welfare after being defined to balance statistical power, feasibility and ethical aspects. All mice were kept in accordance with federal and state policies on animal research at BioNTech SE.

#### P627 Comparing the functionality of Proleukin® and Akron Interleukin-2 through an analysis of key T cell subsets

##### Claudia Zylberberg, PhD^1^, Claudia Zylberberg, PhD^2^, John Koreth, MBBS, DPhil^3^, Jerome Ritz, MD^3^

###### ^*1*^*Akron Biotechnology, Boca Raton, FL, United States;*^*2*^*Akron Biotech, Boca Raton, FL, United States* ; ^*3*^*Dana Farber Cancer Institute, Cambridge, MA, United States*

####### **Correspondence:** Claudia Zylberberg (czylberberg@akronbiotech.com)

**Background**

Recombinant human interleukin-2 (IL-2) promotes the proliferation of activated T cells and has long been used in vivo to stimulate and maintain the growth of effector T cells and increasingly, to expand T cells in vitro (including genetically engineered CAR-T cells) for adoptive cell therapy in patients with cancer. IL-2 was approved by the FDA for treatment of metastatic renal cancer in 1992 and for treatment of metastatic melanoma in 1998. However, its administration in the approved dose has been associated with serious side effects, including vascular toxicity. There have recently been efforts to address these issues through modifications to the molecule, through the development of fusion proteins, and through the development of low-dose formulations. Akron Biotechnology manufactures IL-2 for in vitro use. The product is embedded in the manufacturing process for several CAR T therapies. There are opportunities to leverage this IL-2 to treat patients suffering from acute and chronic graft versus host disease (GVHD) following allogeneic hematopoietic stem cell transplantation, as well as a range of other autoimmune diseases.

**Methods**

CyTOF (cytometry by time-of-flight mass spectrometry): antibody panel designed by Dana Farber Cancer Institute. There are three categories of target proteins: surface proteins, intracellular markers and signaling molecules. Different markers were checked after stimulation with Akron IL-2 or Proleukin® (15 minutes) and verified with lymphocyte subset and intrinsic signaling cascade.

**Results**

The research focused on three major T cell subset populations (Treg, Tcon and CD8 T cells). Upon stimulation with Akron IL-2 and Proleukin®, CD25, CD122, CD132, CTLA-4, PD-1, PD-L1 markers were checked for individual T cell subsets, observed via t-SNE plot. The data indicated that the antibody panel setting was accurate and reliable; each marker was expressed in the expected T cell subset. For example: CD25 was expressed exclusively in Tregs. The expression of pSTAT5, pSTAT3, and pSTAT1 was identical in Akron IL-2 and Proleukin®, controlling for exposure and dosage (Figure 1 includes summary of pSTAT5 results). These results confirm that Akron IL-2 and Proleukin® are identical in their ability to boost adaptive immune response.

**Conclusions**

The toxicity associated with high dose IL-2 has led many to seek alternatives that can achieve positive therapeutic outcomes while minimizing negative side effects. This study demonstrates that Akron's IL-2 is functionally similar to Proleukin®, validating its utility for the production of adoptive immunotherapies, and potentially for treatment of patients suffering from autoimmune diseases.

**Fig. 1 (abstract P627).**
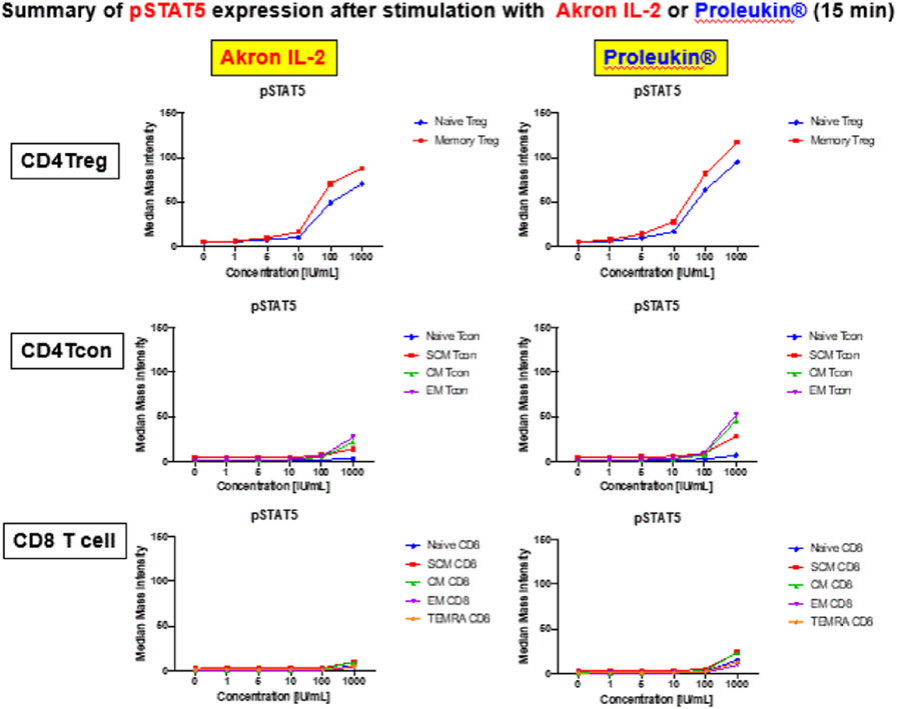
Summary of pSTAT5 Expression after Stimulation

#### P628 Inhibition of histone deacetylase enhances CD8 T-cell functionality and metabolic fitness resulting in strong anti-tumor response.

##### Pankaj Gaur, PhD^1^, Vivek Verma, PhD^1^, Rahul Nnadre^1^, Fatmah Alolaqi^1^, Peter Ordentlich, PhD^2^, Lei Wang, PhD^2^, Seema Gupta, PhD^1^, Samir Khleif, MD^1^

###### ^1^Georgetown University School of Medicine, Washington, DC, United States; ^2^Syndax Pharmaceuticals, Waltham, MA, United States

####### **Correspondence:** Samir Khleif (snk48@georgetown.edu)

**Background**

Tumor microenvironment (TME) often harbors epigenetic alterations mediated by histone acetyltransferases (HATs) and histone deacetylases (HDACs)[1, 2]. The epigenetic dysregulation of T-cells and enhanced numbers of immunosuppressive cells in the TME are associated with decreased anti-tumor effects. Hence, targeting the epigenetic modifications using modulators such as HDAC inhibitors (HDACi) provides the basis for a potential role for these agents in cancer immunotherapy. Earlier, we found that HDACi, entinostat reprograms the TME by increasing the number of central memory T-cells and enhancing the functionality of effector cells, resulting in enhanced anti-tumor responses. However, the mechanisms by which HADCi enhances the effector functions of CD8 cells are not well understood.

**Methods**

In the TC-1 mouse tumor model, entinostat (3mg/kg) was administered with a tumor-specific vaccine (E7-peptide; 3 doses one-week apart). Tumor growth and mice survival were recorded. Three-days after the second immunization, immune-responses were determined in the tumors. Mitochondrial mass of CD8 cells was estimated by incorporation of MitoFM dye, while glucose uptake rates were determined by 2-NBDG incorporation.

**Results**

We found that entinostat, in presence of optimal CD8 cell priming using tumor specific E7-peptide vaccine, significantly delayed the tumor growth and prolonged the mice survival. Immunologically, entinostat significantly enhanced the numbers of total and antigen-specific CD8+ T cells in the TME. Importantly, we observed a significant increase in the numbers of activated to total and antigen-specific granzyme B+ CD8+ T-cells in the TME when combined with the vaccine. The HDAC inhibition also augmented IFN-gamma production in CD8+ T cells. It is now widely appreciated that T-cell differentiation and effector functions are coupled to metabolic reprogramming. Therefore, using in-vitro assay systems, we checked the status of CD8+ T cells treated with the HDAC inhibitor. We found that HDAC inhibition in CD8 cells during antigen specific activation resulted in significantly increased mitochondrial mass. Activated CD8+ T effector cells are known to rely on glucose metabolism for their energy requirements. Interestingly, the glucose uptake rates of entinostat treated CD8 cells were not different than the non-treated cells indicating that glucose was not utilized differentially in entinostat treated cells. We are further in process of delineating the mechanisms of entinostat mediated enhancement of CD8 cells metabolism.

**Conclusions**

Overall, our study strongly supports the key role of epigenetic factors in regulating CD8+ T-cell effector and/or memory generation, and metabolic fitness. However, the epigenetic changes that accompany these functional T-cell stages in relation to metabolism remain to be understood.

**References**

1. Zhang, L. and P. Romero, Metabolic Control of CD8(+) T Cell Fate Decisions and Antitumor Immunity. Trends Mol Med, 2018. 24(1): p. 30-48.

2. McCaw, T.R., et al., Manipulating the epigenetic framework of T cells with histone deacetylase inhibitors for more robust and durable anti-tumor responses. The Journal of Immunology, 2018. 200(1 Supplement): p. 57.41-57.41.

#### P629 Therapeutic vascular normalization to promote tumor-associated tertiary lymphoid structures

##### Jessica Filderman, BS, Walter Storkus, PhD

###### University of Pittsburgh, Pittsburgh, PA, United States

####### **Correspondence:** Walter Storkus (storkuswj@upmc.edu)

**Background**

Tertiary lymphoid structures (TLS) are non-encapsulated immune cell aggregates that form at sites of chronic inflammation, including tumors in some cases. Recent studies have shown that the presence of TLS in human tumors is an indicator of positive clinical outcome. However, due to dysregulated angiogenesis, many tumors have poorly organized and leaky vasculature that impedes entry of immune effector cells into tumors and consequently TLS formation. Recently, pre-clinical studies have shown that low doses (well below maximum tolerate dose) of antiangiogenic agents normalize the tumor vasculature, leading us to hypothesize that treating tumors with low doses of these vascular normalizing (VN) therapies will improve immune cell infiltration and TLS formation within the tumor microenvironment (TME).

**Methods**

To test this hypothesis, tumor-bearing mice were treated intratumorally with VN agents. Five days post-treatment, tumors were digested into single cell suspensions and RNA was isolated and used for RT-PCR. Transcript levels of TLS-promoting factors (CCL19, CCL21, CXCL13) and markers of vascular normalization (HIF1A, GLUT1) and inflammation/immune cell infiltration (CXCL10, VCAM1, CD8A) were measured and compared to PBS treated controls. Additionally, primary cultures of murine T cells and DCs and the (BRAFV600E PTEN-/-) BPR melanoma cell line were treated in vitro with VN agents. Flow cytometry was then used to evaluate cell phenotype/function, while RT-PCR was used to evaluate transcript levels of TLS-promoting factors.

**Results**

We observed that the VN agents Dasatinib, Aduro, Bevacizumab, and agonist anti-TNFR1 antibody each induced global changes in the TME that are potentially supportive of immune cell infiltration and TLS formation. These changes include increases in transcript levels of CCL19, CCL21, and VCAM1. In vitro, Dasatinib induced DCs and BPR melanoma cells to express higher levels of co-stimulatory receptors, MHC class I and II, and TLS-promoting chemokines. However, while treatment with Aduro was able to enhance T cell activation, treatment with Dasatinib inhibited T cell activation, as measured by CD25 and CD69 expression.

**Conclusions**

VN agents induce global changes in immune cell infiltration and TLS-promoting factors in the TME. When these changes are evaluated on specific cell types, some agents were found to increase DC and T cell activation, while others have an inhibitory effect. This knowledge can be used to determine optimal combination immunotherapy designs in the cancer setting.

#### P630 Induction of antitumor immune response by selective HDAC6 inhibition

##### Chiara Ripamonti^1^, Gianluca Caprini^1^, Andrea Stevenazzi^1^, Barbara Vergani^1^, Luigi Aurisicchio^2^, Fabio Palombo, PhD^2^, Antonella Conforti^2^, Christian Steinkuhler^1^, Gianluca Fossati, Dr^1^

###### ^1^Italfarmaco SpA, Cinisello Balsamo, Italy; ^2^Takis Srl, Takis, Italy

####### **Correspondence:** Christian Steinkuhler (c.steinkuhler@italfarmaco.com), Gianluca Fossati (g.fossati@italfarmaco.com)

**Background**

The thorough understanding of the immune homeostatic mechanisms has led to the development of target-based immunotherapies. Still, the immunosuppressive tumor microenvironment limits the effectiveness of immunotherapy in several patients. To overcome this obstacle, new targets and combination therapies are the aim of numerous ongoing clinical trials. Histone deacetylase inhibitors have pleiotropic and promising activities as stimulators of anti-tumor immune response alone or in combination with immune checkpoint inhibitors and activators. HDAC6 orchestrates key processes in both innate and adaptive immune cells. HDAC6 null mice are viable and show few phenotypic alterations, indicating that HDAC6 inhibition is potentially safe in humans. We studied the effect of our potent and selective HDAC6 inhibitor, ITF3756, in a model of murine colon carcinoma in vivo and in a model of exhaustion in healthy human T cells in vitro.

**Methods**

BALB/c mice were injected s.c. with 1x106 CT26 cells and treated with anti-PD1 (i.p) gemcitabine (i.p) ITF3756, (os) or combinations. CD4 and CD8 T cells were depleted using specific antibodies given at the beginning of the experiment. Depletion was checked by flow cytometry. Human CD3+ cells were stimulated three times with anti-CD3/CD28 beads, during 7 days with ITF3756 added at the time of the first two stimulations. During the process, the expression of exhaustion, memory and effector T cells markers were analyzed by flow cytometry.

**Results**

In vivo, ITF3756 was as effective as an anti PD-1 antibody and their combination additionally reduced tumor growth. Spleen T cells of animals treated with ITF3756 responded ex vivo to immunogenic CT26-derived peptides, suggesting that the antitumor activity of the molecule was immune mediated. Indeed, ITF3756 was completely ineffective in SCID/beige mice bearing CT26 tumor. Selective in vivo depletion of CD4 and CD8 T cells indicated that the activity of ITF3756 strictly depends on both T cell subsets with a greater contribution of CD8 T cells.

In a model of in vitro human T cell exhaustion, ITF3756 countered the exhaustion process as evidenced by the reduced expression of PD-1 and Lag3. Furthermore, ITF3756 increased the CD8 T central memory and reduced the effector memory subsets.

**Conclusions**

This study shows in vivo and in vitro mechanisms whereby ITF3756 increases the immune response and constitutes a robust basis for the rational use of selective HDAC6 inhibitors either alone or in combination for the effective induction of antitumor immune response.

**Ethics Approval**

All the animal procedures (including housing, health monitoring, restrain, dosing, etc) and ethical revision were performed according to the current Italian legislation (Legislative Decree March 4th, 2014 n. 26) enforcing the 2010/63/UE Directive on the protection of animals used for biomedical research

#### P631 Immunotherapy plus gene therapy: a novel nanomedicine carrying the RB94 gene that suppresses tumor growth in non-small-cell lung cancer by inducing immunogenic cell death & modulating host immunity

##### Joe Harford, PhD^1^, Caroline Doherty^1^, Manish Moghe^2^, Antonina Rait, PhD^2^, Steven Metallo, PhD^3^, Kathleen Pirollo, PhD^2^, Esther Chang, PhD^2^, Sang-Soo Kim, PhD^1,3^

###### ^1^SynerGene Therapeutics, Inc., Potomac, MD, United States; ^*2*^*Georgetown Univ Med Ctr, Washington, DC, United States*; ^*3*^*Georgetown University, Washington, DC, United States*

####### **Correspondence:** Sang-Soo Kim (sangsoo.kim@georgetown.edu

**Background**

Lung cancer is the world's most common malignancy (>2 million new cases & ~1.7 million deaths annually) [1]. Current lung cancer treatments are woefully inadequate, and immunotherapy has had limited success with but 10-20% of patients with advanced non-small-cell lung cancer (NSCLC) showing durable responses [2]. This has led to a focus on combining immunotherapy with other treatment modalities, and we are exploring combining gene therapy and immunotherapy. scL-RB94, is a cationic liposome encapsulating the RB94 gene and using a single-chain antibody fragment recognizing the transferrin receptor to target tumors (Figure 1). RB94 protein is a potent tumor suppressor [3], and the scL delivery system targets tumors with exquisite specificity [4,5]. In a Phase I trial of scL-RB94 (aka SGT-94), we observed that systemically administered scL-RB94 delivers its payload to small bladder cancer metastases in the lungs resulting in tumor-specific expression of RB94 protein [6]. This ability of scL-RB94 to deliver the RB94 gene to tumor cells in the lungs prompted us to explore use of this investigational agent against NSCLC.

**Methods**

Because RB94 is active against human but not mouse tumor cells, we have utilized human NSCLC cell lines (H292 & H358) and subcutaneous xenografts of these cells in athymic mice. NSCLC tumor growth was monitored with and without scL-RB94 added to immunotherapy. Expression of markers for innate immunity were also assessed.

**Results**

Intravenous injection of scL-RB94 markedly inhibited growth of NSCLC xenografts (Figure 2) by inducing immunogenic cell death (ICD) (Figure 3). We observed enhanced tumor infiltration by NK cells (Figure 4) and elevated expression of NK activation ligands. scL-RB94 altered polarization of macrophages toward the M1 phenotype (Figure 5). HLA/A and TAP1/2 gene expression were upregulated (Figure 6), and expression of PD-L1 on the tumor cells was significantly elevated (Figure 7).

**Conclusions**

scL-RB94 renders NSCLC tumors more immunologically "hot" by triggering ICD and enhancing innate immunity via pleiotropic effects on multiple immune-relevant genes (Figure 8, Box A & B). We are currently exploring the effects of scL-RB94 on adaptive immune responses and in relieving tumor immunosuppression (Figure 8, Box C & D). We hypothesize that combining scL-RB94 with a checkpoint inhibitor will improve response rates compared to immunotherapy alone. With scL-RB94, it may also be possible to reduce the dose of the checkpoint inhibitor to mitigate toxicity. Our longer-term goal is to improve outcomes for NSCLC patients through more effective treatment that combines gene therapy and immunotherapy.

**References**

1. IARC Global Cancer Observatory web site (http://gco.iarc.fr/).

2. Moya-Horno I, Viteri S, Karachaliou N, Rosell R. Combination of immunotherapy with targeted therapies in advanced non-small cell lung cancer (NSCLC). Therap Advances Med Oncol. 2018;10:1758834017745012. Epub 2018/02/01.

3. Xu HJ, Xu K, Zhou Y, Li J, Benedict WF, Hu SX. Enhanced tumor cell growth suppression by an N-terminal truncated retinoblastoma protein. Proc Natl Acad Sci USA. 1994; 91:9837-9841.

4. Pirollo KF, Dagata J, Wang P, Freedman M, Vladar A, Fricke S, Ileva L, Zhou Q, Chang EH. A tumor-targeted nanodelivery system to improve early MRI detection of cancer. Molecular Imaging. 2006; 5: 41-52.

5. Pirollo KF, Zon G, Rati A, Zhou Q, Yu W, Hogrefe R, and Chang EH. Tumor targeting nanoimmunoliposome complex for short interfering RNA delivery. Human Gene Therapy. 2006; 17:117-124.

6. Siefker-Radtke A, Zhang XQ, Guo CC, Shen Y, Pirollo KF, Sabir S, et al. A Phase l Study of a Tumor-targeted Systemic Nanodelivery System, SGT-94, in Genitourinary Cancers. Molecr Therapy. 2016; 24:1484-1491.

**Ethics Approval**

All animal studies was performed under an IACUC-approved protocol in compliance with the Animal Welfare Act, PHS Policy, and other Federal statutes and regulations relating to animals in the AAALAC-accredited animal facility at Georgetown University, which adheres to principles stated in the Guide for the Care and Use of Laboratory Animals, National Research Council .

**Fig. 1 (abstract P631).**
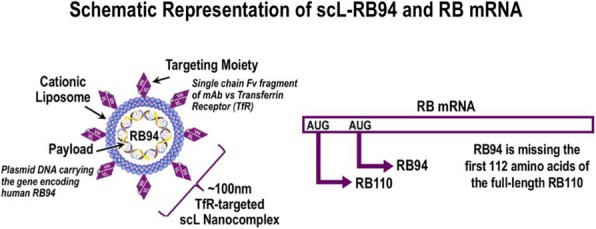
See text for description

**Fig. 2 (abstract P631).**
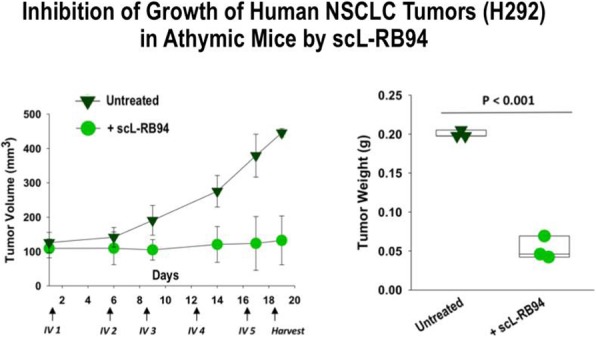
See text for description

**Fig. 3 (abstract P631).**
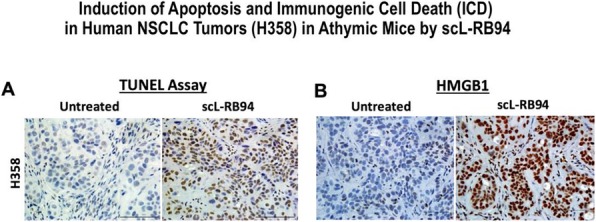
See text for description

**Fig. 4 (abstract P631).**
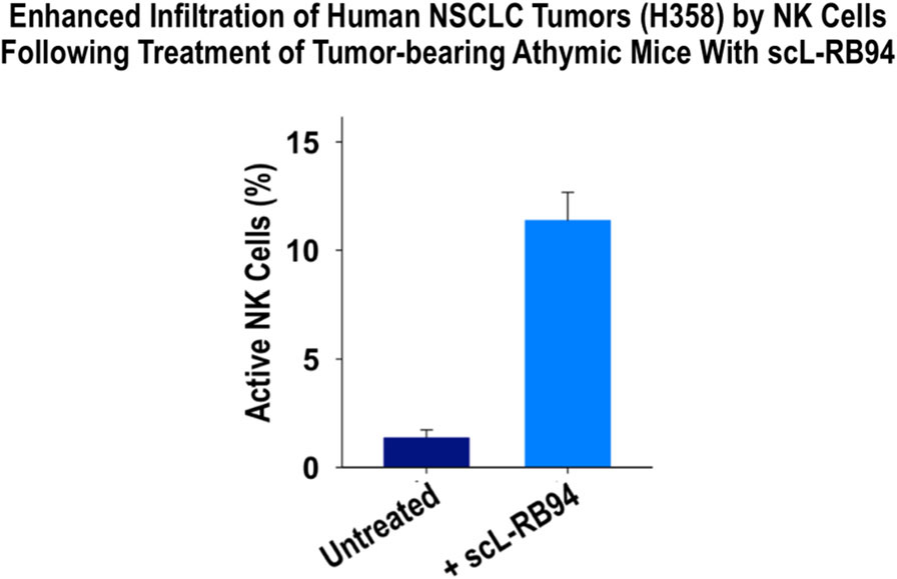
See text for description

**Fig. 5 (abstract P631).**
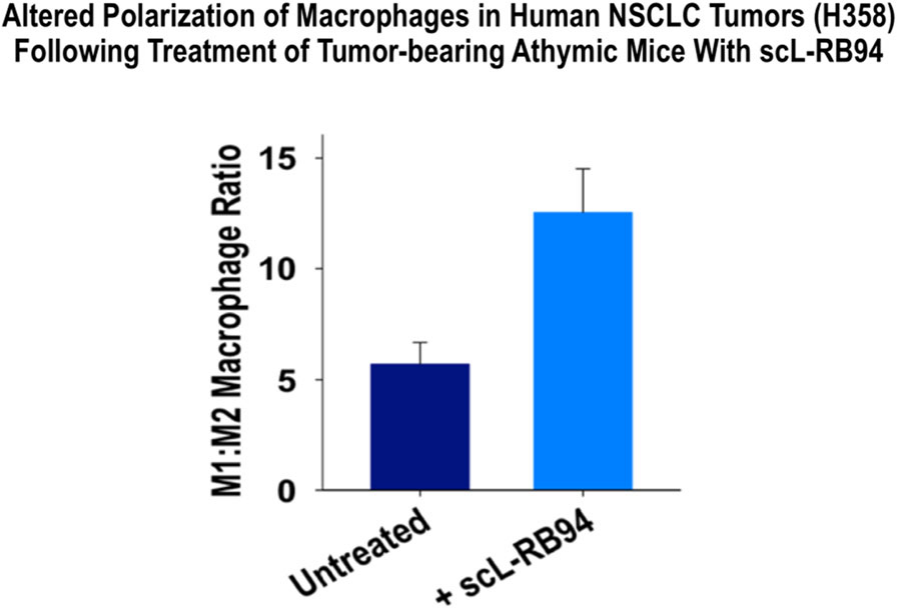
See text for description

**Fig. 6 (abstract P631).**
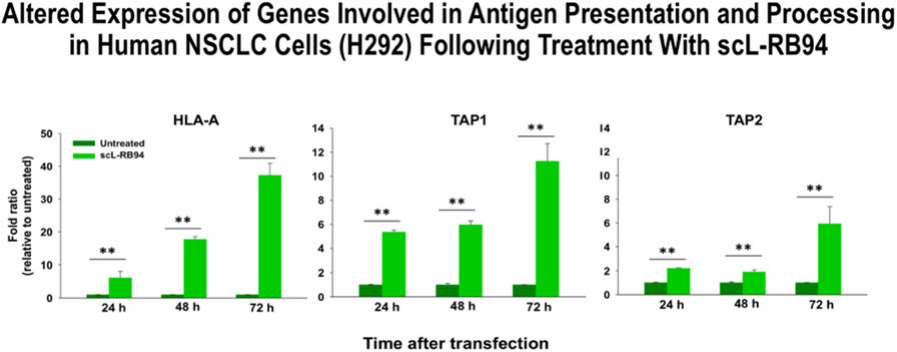
See text for description

**Fig. 7 (abstract P631).**
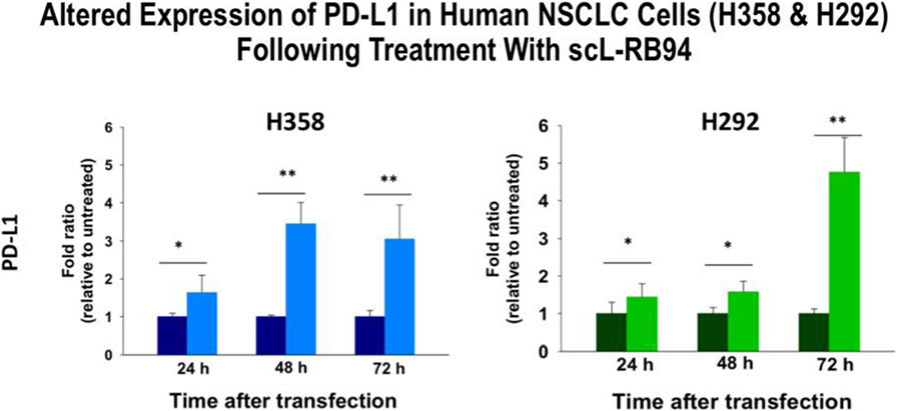
See text for description

**Fig. 8 (abstract P631).**
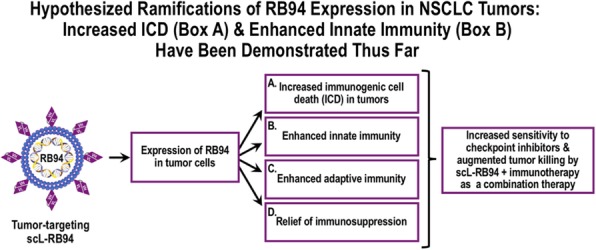
See text for description

#### P632 Acute myeloid leukemia induced T cell suppression can be reversed by inhibition of the MAPK pathway

##### Kaycee Moshofsky, BS, MCR, Kyle Romine, BS, Yoko Kosaka, PhD, Guanming Wu, Shannon McWeeney, PhD, Evan Lind, PhD

###### Oregon Health & Science University, Portland, OR, United States

####### **Correspondence:** Evan Lind (linde@ohsu.edu)

**Background**

Acute myeloid leukemia (AML) remains difficult to treat due to mutational heterogeneity and the development of resistance to treatment. Targeted agents, such as MEK inhibitors, may be incorporated into treatment, however, the impact of MEK inhibitors on the immune microenvironment in AML is not well understood. A greater understanding of the implications of MEK inhibition on immune responses may lead to greater understanding of immune evasion and more rational combinations with immunotherapies

**Methods**

AML murine model

Mice expressing FLT3-ITD under the endogenous FLT3 promotor were crossed to mice with the Tet2 gene flanked by LoxP sites and CRE recombinase under control of the LysM promotor. All mouse experiments were performed in accordance with IACUC protocol #IP00000907.

Primary patient samples

Peripheral blood and bone marrow samples were collected from patients with AML. Peripheral blood was collected from healthy donors. All participants gave informed consent to participate (OHSU eIRB# 4422).

Murine proliferation assays

Mononuclear cells were stained with CellTrace CFSE and plated with plate-bound anti-CD3. The mononuclear cells were treated with anti-PD1 and/or trametinib.

Primary patient sample proliferation assays

Mononuclear cells were labeled with CellTrace Violet. Cells were plated with anti-CD3. Cells were treated with combination of anti-PD1 and/or trametinib for 5 days.

**Results**

In the mouse model, trametinib increases T cell viability and restores T cell proliferation. Importantly, we report greater proliferation in the CD8+CD44+ effector subpopulation and impaired activation of CD8+CD62L+ naïve cells. Transcriptome analysis revealed trametinib sensitive samples have an inflammatory gene expression profile, and we also observe increased PD-L1 expression on trametinib sensitive samples. Finally, we demonstrate trametinib consistently reduces PD-L1 and PD-L2 expression in a dose-dependent manner on the myeloid population.

**Conclusions**

There are many clinical trials studying immunotherapies in AML. However, immunotherapies may fail as monotherapies in clinical trials if there is strong persisting immunosuppression in the tumor microenvironment or due to a lower mutational burden. Previous work in other cancers have demonstrated benefits of using MEK inhibition in combination with immunotherapies, including checkpoint inhibitors, adoptive T cell therapy, and viral therapy. Subpopulations of AML patients may have improved response to immunotherapies when used in combination with targeted agents. For this reason, we believe our data is useful in beginning to determine immune evasion pathways, such as MEK signaling, in AML in order to target these pathways with the combination of targeted inhibitors and immune based therapies, such as checkpoint inhibitors, bi-specifics, vaccines, or CAR T cell therapies.

**Acknowledgements**

This work was supported by grant U54CA224019 (E.F.L., S.K.M.) from the National Cancer Institute, grant U41HG003751 (G.W.) from the National Human Genome Research Institute, and by the Oregon Clinical and Translational Research Institute (OCTRI) grant TL1TR002371 (K.B.M.) from the National Center for Advancing Translational Sciences (NCATS) at the National Institutes of Health (NIH). The content is solely the responsibility of the authors and does not necessarily represent the official views of the NIH.

**Ethics Approval**

All mouse experiments were performed in accordance with IACUC protocol #IP00000907. All participants gave informed consent to participate in the study, which is approved by the Institutional Review Board at OHSU (eIRB# 4422).

#### P633 BPM31510, a metabolic modulating anti-cancer agent, demonstrates immune potentiating properties by promoting cytotoxic T cells and reversing indices of exhaustion and immunosuppression

##### Maria-Dorothea Nastke, PhD, Shiva Kazerounian, PhD, Anne Diers, PhD, Stephane Gesta, PhD, Vivek Vishnudas, PhD, Niven Narain, PhD, Rangaprasad Sarangarajan, PhD

###### Berg LLC, Framingham, MA, United States

####### **Correspondence:** Rangaprasad Sarangarajan (rangaprasad.sarangarajan@berghealth.com)

**Background**

Regulation of mitochondrial metabolism is crucial for immune cell differentiation and function. Therapeutic agents influencing mitochondrial metabolism can potentially impact immune-mediated tumor elimination to affect efficacy outcomes. BPM31510 is a Coenzyme Q10-containing lipid nanodispersion in clinical development for treatment of solid tumors. In a phase II study, patients treated with BPM31510 demonstrated signs of pseudo-progression suggesting immune-modulating properties associated with BPM31510 that was further investigated in a preclinical model.

**Methods**

A MC38 syngeneic mouse model was utilized to characterize tumor infiltrating lymphocytes by flow cytometry. Peripheral blood mononuclear cells (PBMCs) isolated from healthy donor leukopaks were treated ex vivo with increasing concentrations of BPM31510 to assess effects on immune cells and cytokine secretion. Phenotypic characterization of cells was assessed by flow cytometry using cell surface receptor staining CD3/CD8/CD4/PD-1/CTLA-4. Proliferation was assessed via EdU incorporation and cytolytic potential was assessed by intracellular staining for CD107a/b, both in combination with T cell markers. Extracellular cytokines IL-2, IFN-γ, and IL-10 were measured via ELISA.

**Results**

Consistent with the hypothesis, in a syngeneic mouse model, BPM31510 increased cytotoxic T cell frequency and cytotoxic T cell/regulatory T cell ratio in tumors. Ex vivo, treatment of PBMCs with BPM31510 lead to an increased frequency of viable CD3+ cells. Proliferation measurements by EdU-incorporation indicated enhanced cytotoxic T cell proliferation, and likewise, BPM31510 increased cytolytic potential of activated cytotoxic T cells, as indicated by measurement of plasma membrane-exposed lysosomal-associated membrane protein 1 (CD107a). Treatment of healthy donor PBMCs with BPM31510 decreased cell surface expression of inhibitory receptors PD-1 and CTLA-4 in cytotoxic and helper T cells. To investigate if BPM31510 regulates the cytokine milieu, cytokine secretion profiles of activated PBMCs treated with BPM31510 were established. Analysis of supernatants by multi-analyte ELISA platform revealed effector cytokines IL-2 and IFN-γ to be dose-dependently increased while IL-10, a key immune-regulatory cytokine, was decreased. Analysis of monocyte depleted PBMCs compared to monocytes alone demonstrated that the observed changes in IL-2 and IFN-γ secretion occur only when both lymphocytes and monocytes are present; however, monocytes alone were identified as the significant source for IL-10.

**Conclusions**

Collectively, the results identify and characterize immune-modulatory activity of BPM31510, particularly in the T cell compartment, specifically on the regulation of T cell exhaustion and immunosuppression in supporting an anti-cancer effect. The data support the utility of BPM31510 in ‘immunologically cold’ tumor types or in combination with immune checkpoint blockade strategies.

#### P634 Predicting radiation-induced immune trafficking and activation in localized prostate cancer

##### Simon Keam, Heloise Halse, ThuNgoc Nguyen, Catherine Mitchell, Franco Caramia, David Byrne, Sue Haupt, Georgina Ryland, Phillip Darcy, Shahneen Sandhu, MBBS FRACP, Piers Blombery, Ygal Haupt, Scott Williams , Paul Neeson, PhD

###### Peter MacCallum Cancer Center, Melbourne, Australia

####### **Correspondence:** Scott Williams (scott.williams@petermac.org)

**Background**

Localized prostate cancer (PCa) treated with high dose rate brachytherapy (HDRBT) has an excellent cure rate in low, but not in high grade group tumours. Localized PCa immune response to radiation damage is unknown. This study explored this question in order to build a rationale for combining HDRBT with immune checkpoint blockade to treat patients with localized higher grade score PCa and improve patient outcomes.

**Methods**

In this study we used ultrasound guided core biopsies collected from 24 men prior to, and 14 days post-HDRBT (10Gy). Serial FFPE sections were stained by H&E or OPAL, using two 7-plex panels to describe the density and tissue location of T cells (CD8+, CD4+ and CD4+ FoxP3+), B cells, macrophages, dendritic cells, PDL1+ and HMWCK+ cells. The H&E sections were examined by a uro-pathologist. RNA was extracted for RNAseq (3’ Lexogen) and Nanostring gene expression profiling (GEP) using the pan cancer immune gene set.

**Results**

HDRBT altered the immune context of PCa. We observed a significant increase post-HDRBT in CD4+FoxP3+ T cells and PDL1- macrophages in both the tumor and tumor stroma (Figure 1). All other immune subsets remained unchanged. All patient samples were grouped into one of three tumor inflammation signature (TIS) [1] clusters (1) high, (2) intermediate and (3) low TIS, Fig 2. Clusters one and two had increased expression of 14/16 and 8/16 TIS genes respectively; cluster three had low expression of all TIS genes. A pre-existing TIS signature was present in 8/24 patients; of the remaining patients 13/16 were converted from a low TIS to intermediate-high TIS gene signature by HDRBT. However, 3/24 PCa tumors did not develop a TIS gene signature post-HDRBT, these tumors had a significantly decreased levels of baseline type I and II IFN response genes, and decreased TNFalpha signalling compared to the 13/16 TIS HDRBT ‘responders’. Finally, we showed that patients with Gleason grade group score (GGG) 4-5 had a pre-existing TIS gene signature level which was not altered by HDRBT (Figure 2); they also exhibited elevated TGFbeta levels pre-HRDBT, that were increased further post-HDRBT.

**Conclusions**

Our study showed PCa has a heterogeneous immune context including a pre-existing TIS, which is further increased by HDRBT. GGG 4-5 patients expressed high TIS levels post-HDRBT, indicating that anti-PD-1/PDL1 antibodies may be effective in these patients; however TGFbeta remains an additional hurdle for radio-immunotherapy efficacy in these patients.

**References**

1. Ayers, M., et al., IFN-γ–related mRNA profile predicts clinical response to PD-1 blockade. The Journal of Clinical Investigation, 2017. 127(8): p. 2930-2940.

**Ethics Approval**

This study was approved by the Peter MacCallum Cancer Centre Human Research Ethics Committee, approval number 14/182

**Fig. 1 (abstract P634).**
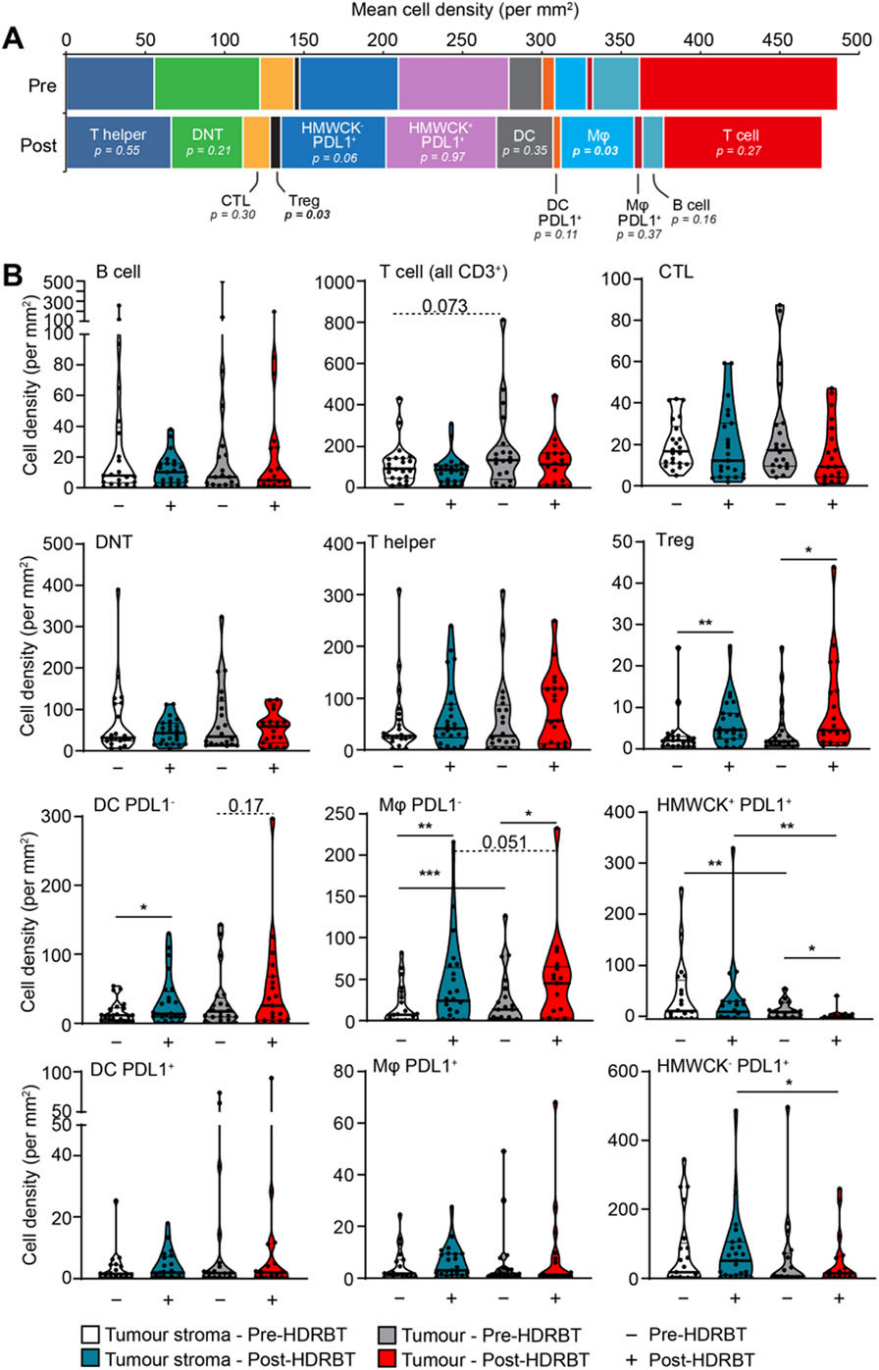
Radiotherapy induced immune changes in prostate cancer

**Fig. 2 (abstract P634).**
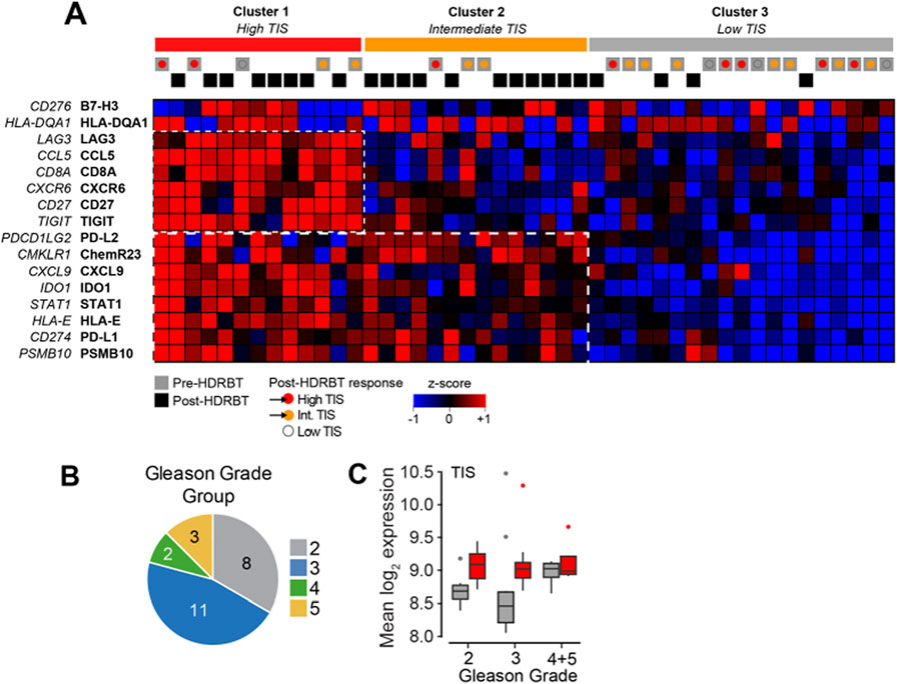
Radiotherapy induced PCa immune gene expression changes

#### P635 The impact of beta blockers on survival outcomes in non-small cell lung cancer patients treated with immune checkpoint inhibitors

##### Michael Oh, MD, Michael Oh, MD, Alex Guzner, Derek Wainwright, PhD, Nisha Mohindra, MD, Young Kwang Chae, MD, Amir Behdad, Victoria Villaflor, MD

###### Northwestern University Feinberg School of Medicine, Chicago, United States

####### **Correspondence:** Victoria Villaflor (victoria.villaflor@nm.org)

**Background**

Beta-blockers have been associated with anti-tumorigenic effects, potentially by reducing adrenergic-mediated stress responses. Preclinical studies have additionally shown that beta-blockade may enhance the efficacy of cancer immunotherapy. We investigated lung cancer patients who concomitantly used beta-blockers and immune checkpoint inhibitors, with the hypothesis that beta-blockade would positively impact clinical outcomes.

**Methods**

We retrospectively reviewed the health records of 104 patients who were treated at Northwestern University between January 2014 through August 2018 with immune checkpoint inhibitors for non-small cell lung cancer (NSCLC). Comparisons of overall survival (OS) and progression-free survival (PFS) were performed using Kaplan-Meier analysis with log-rank test, and a multivariate analysis was performed with a Cox regression model.

**Results**

Among 104 patients treated with immune checkpoint inhibitors for NSCLC, 27 of them were concomitantly prescribed beta-blockers. Use of beta-blockers was associated with increased PFS, with hazard ratio (HR) of 0.57 and 95% confidence interval (CI) of 0.35-0.93. There was not a significant increase in overall survival (OS) among patients who took beta-blockers (HR 0.63, 95% CI 0.35-1.12). In a regression model, beta blockers were identified as predictive of PFS, as were non-squamous histology, tumor PD-L1 positivity, and lower line of treatment. A subgroup analysis of patients with brain metastases (n=37) suggested greater overall survival among patients taking beta blockers (HR 0.17, 95% CI 0.06-0.54).

**Conclusions**

Beta-blocker use of at least 1 year was associated with improved PFS among patients treated with immune checkpoint inhibitors for NSCLC in our cohort of patients. This was a small study and these findings should be further validated in prospective clinical studies.

#### P636 Inhibitors of Ataxia-Telangiectasia Related (ATR) protein lead to innate immune pathway activation and enhanced response to immune therapy in prostate cancer

##### Patrick Pilie, MD, Zhe Tang, Sanghee Park, Cheng Wu, Zhenyang Dong, Timothy Yap, MD PhD, Paul Corn, Timothy Thompson

###### University of Texas MD Anderson Cancer C, Houston, TX, United States

####### **Correspondence:** Timothy Thompson (timthomp@mdanderson.org)

**Background**

In prostate cancer (PCa), androgen receptor (AR) signaling regulates the cell cycle and mitigates replication stress via upregulation of CDC6 and the TopB1-ATR-Chk1 pathway, the key axis in replication stress response (RSR)[1]. Preclinical studies and early phase clinical trials of DDR inhibitors, including PARP inhibitors, have shown synergy with immune checkpoint therapy, whereby DDR inhibitors lead to S-phase specific damage, accumulation of cytosolic DNA and activation of innate immune pathways[2]. DDR inhibitors have rapidly expanded, now including inhibitors of ATR[2]. However, the impact of these novel, potent, highly-specific inhibitors of ATR on immunocompetent PCa preclinical models has not been fully investigated. Furthermore, dynamic changes in DDR and innate immune gene expression profiles that result from various targeted therapies have not been reported. In this study we use preclinical, immunocompetent models of advanced PCa to investigate the efficacy, transcriptional targets, induction of immunostimulatory cytosolic DNA, and synergy with anti-PD-L1 therapy associated with ATR inhibition.

**Methods**

We used PCa cell lines derived from ras+myc—induced mouse PCa tumors (RM-1, RM-9 and RM-1-BM) to analyze ATRi (BAY1895344 or VX-970) effects on ATR-driven survival/proliferation, DNA damage, cGAS-STING signaling, and chemokine expression in comparison to olaparib. RM-1-BM xenograft model was used to assess the efficacy and safety of single agent ATR inhibitor, single-agent anti-PD-L1, and the combination in vivo.

**Results**

Treatment of the PCa cell lines with ATRis (BAY1895344 or VX-970) or a PARP inhibitor (olaparib) demonstrated dose-dependent (0.125-8μM) growth suppression and cytosolic DNA accumulation, while ATRis showed greater growth suppression than olaparib. ATRi and olaparib treatment generated similar levels of DNA damage (γH2AX); however, ATRi treatments resulted in significantly increased CCL5 and CXCL10 levels compared to olaparib (2-10 fold, P< 0.05), with BAY1895344 demonstrating superior induction of chemokine expression (P< 0.05). cGAS, STING, TBK1 or IRF3 siRNA knockdown significantly suppressed all of these responses (P< 0.05). The combination of BAY1895344 with anti-PD-L1 was safe and resulted in significantly greater tumor response than either single agent in RM-1-BM xenograft models.

**Conclusions**

ATR inhibition induces S-phase specific DNA damage, accumulation of cytosolic DNA, activation of downstream cGAS-STING signaling, and suppression of cancer growth in immunocompetent PCa models. ATRi had greater impact on innate immune pathway activation than olaparib, and the combination of ATRi with anti-PD-L1 was safe and resulted in significant efficacy in our model system. These results will directly inform biomarker-directed clinical trials of ATR inhibitors in combination with immune checkpoint blockade for patients with advanced solid cancers.

**Acknowledgements**

We would like to acknowledge members of the Bayer-MD Anderson Cancer Center Alliance, including but not limited to Dr. Antje Wengner, Dr. Dominik Mumberg.

**References**

1. Karanika S, Karantanos T, Li L, et al. Targeting DNA Damage Response in Prostate Cancer by Inhibiting Androgen Receptor-CDC6-ATR-Chk1 Signaling. Cell Rep. 2017; 18(8):1970-1981.

2. Pilie PG, Tang C, Mills GB, Yap TA. State-of-the-art strategies for targeting the DNA damage response in cancer.. Nat Rev Clin Oncol. 2019; 16(2):81-104.

**Ethics Approval**

The study, including animal work, was approved by MD Anderson Cancer Center's board, approval number #885 and #889.

#### P637 Immunomodulation of solid tumors by carbon-ion therapy versus conventional photon therapy

##### Catherine Spina, MD, PhD^1^, Chizuru Tsuruoka^2^, Wendy Mao^3^, Masaaki Sunaoski^2^, Mathew Chaimowitz^3^, Yi Shang^2^, Shizuko Kakinuma^2^, Charles Drake, MD, PhD^3^ , Catherine Spina, MD, PhD^3^

###### ^1^Columbia University Irving Medical Center, New York, NY, United States; ^2^NIRS-HIMAC, Chiba, Japan; ^3^Columbia University Irving Med Ctr., New York, NY, United States

####### **Correspondence:** Charles Drake (cgd2139@cumc.columbia.edu)

**Background**

Radiation-induced immunostimulation is of great interest as a strategy to reinvigorate or augment an immunotherapy-induced tumor-specific response. Evidence supports photon therapy (PhRT) as a tumor immunomodulator, pre-clinical data suggest that carbon-ion therapy (CiRT) may be more potent. Here we evaluate the immunomodulatory effects of CiRT, compared to biologically equivalent doses (BEDs) of PhRT in an orthotopic breast cancer mouse model.

**Methods**

Using the orthotopic 4T1 breast cancer mouse model, we evaluated the immunomodulatory effects of very low (0.4 Gy), low (1 Gy) and high (4 Gy) doses of CiRT (290 MeV/u, SOBP) delivered to mammary tumors at the Heavy Ion Medical Accelerator in Chiba (HIMAC), Japan compared to BEDs (RBE 2) of PhRT (SARRP, Xstrahl; 220 kV). Primary tumors were treated 7-days post implantation and harvested 72-hours later for flow cytometric characterization and protein cytokine analysis.

**Results**

At low doses, CiRT was lymphocyte sparing effect compared to PhRT. Only low and high-dose CiRT resulted in a significant (p

**Conclusions**

We demonstrate that at low doses, lymphocytes differ in their sensitivity to CiRT compared to PhRT -- CiRT being more lymphocyte-sparing. Very high-dose CiRT induced a more potent and mechanistically diverse of pro-inflammatory cytokine response, compared to PhRT. Combining less lymphotoxicity at low dose with more potent pro-inflammatory effects at high doses, CiRT may be a more optimal synergistic tool to augment an immunotherapy-induced, tumor-specific response.

#### P638 Integrin-mediated augmentation of antigenic immunity

##### Upendra Marathi, PhD^1^, Yared Hailemichael, PhD^2^, Nandadeva Lokugamage^3^, Michael Schotsaert^4^, Angela Choi^4^, Imran Chwdhury^3^, Nisha Garg^3^, Adolfo Garcia-Sastre^4^, Lionel Lewis^1^, Siddhartha De^1^, Peter Vanderslice, PhD^5^, Jeffrey Actor^6^, Darren Woodside, PhD^5^

###### ^*1*^*7 Hills Pharma, Houston, TX, United States*; ^*2*^*M.D. Anderson Cancer Center, Houston, TX, United States* ; ^*3*^*The University of Texas Medical Branch, Galveston, TX, United States* ; ^*4*^*Ichan School of Medicine at Mount Sinai, New York, NY, United States* ; ^*5*^*Texas Heart Institute, Houston, TX, United States* ; ^*6*^*UT Health, Houston, TX, United States*

####### **Correspondence:** Darren Woodside (dwoodside@texasheart.org)

**Background**

The use of immune stimulatory agents as systemic drugs to augment adjuvantation can be problematic due to elevated risks of toxicity and non-specific immune responses. Integrins αLβ2/ICAM-1 and α4β1/VCAM-1 are essential for antigen-specific immune responses. [1,2] In particular, these integrins stabilize receptor-mediated cell adhesion between Antigen Presenting Cells and naïve CD4+ T cells, providing a co-stimulatory signal required for effective antigen presentation.[3] 7HP349 is a first-in-concept, orally bioavailable, positive allosteric activator of these integrins.

**Methods**

To determine if systemic administration of 7HP349 in combination with known vaccines would increase adjuvantation, 7HP349 was co-administered with T. cruzi TcG2 and TcG4 vaccines against Chagas disease, inactivated vaccine against Influenza, and BCG vaccines against M. tuberculosis (Mtb) in mice. Moreover, 7HP349 as monotherapy in an immunogenic murine CT26 carcinoma model, and in combination with aCTLA-4/GVAX in a non-immunogenic murine B16 melanoma model.

**Results**

In the CT26 model, 7HP349 monotherapy significantly delayed tumor growth (Table 1). In the B16 tumor model, 7HP349 in combination with GVAX/aCTLA4 significantly increased long-term (>120 day) survival by 2.7-fold over GVAX/aCTLA4. In combination with T. cruzi TcG2/TcG4 vaccines, 7HP349 significantly decreased parasite burden in myocardial (~9.5-fold) and skeletal muscle (~5.3-fold) in challenged mice and increased splenic frequency of polyfunctional CD4+ and CD8+ T cells expressing IFN-gamma, perforin or granzyme B. In addition, in vitro stimulation with Tc antigens of splenocytes from vaccinated animals resulted in maximal release of TNFα, IFN-γ, IL-1β and IL-6 cytokines when 7HP349 was co-administered with vaccine. 7HP349 increased influenza H1N1 seroconversion and hemagglutinin inhibition titers by ~30 fold when co-administered with inactivated influenza vaccine. 7HP349 addition to a prime-boost immunization regimen with BCG reduced pulmonary mycobacterial burden and inflammation after Mtb challenge compared to vaccine alone. At the 0.5 and 1 mg/kg doses, the therapeutic plasma 7HP349 concentration range of AUC of 50-100 ng.h/mL, appears to be sufficient to engage αLβ2/ICAM-1 and α4β1/VCAM-1 and increase tumor immunity, checkpoint blockade, and T cruzi, BCG and H1N1 vaccination. 7HP349 exposures of >1,500 ng.h/mL for 28 days in rats and dogs in cGLP toxicology studies showed no adverse findings

**Conclusions**

7HP349 as systemic drug may facilitate immune priming against a variety of antigens by stabilizing αLβ2/ICAM-1 and α4β1/VCAM-1 interactions, with a high safety margin. 7HP349 oral drug product is IND ready. Phase I studies as monotherapy and combined with vaccine for influenza, or with immune checkpoint inhibitors in refractory solid tumors are planned.

**References**

1. Davignon D, Martz E, Reynolds T, Kürzinger K and Springer TA. Monoclonal antibody to a novel lymphocyte function-associated antigen (LFA-1): mechanism of blockade of T lymphocyte-mediated killing and effects on other T and B lymphocyte functions. The Journal of Immunology. 1981;127:590-595.

2. Shimizu Y, van Seventer GA, Horgan KJ and Shaw S. Costimulation of proliferative responses of resting CD4+ T cells by the interaction of VLA-4 and VLA-5 with fibronectin or VLA-6 with laminin. The Journal of Immunology. 1990;145:59-67.

3. Woodside DG, Teague TK and McIntyre BW. Specific inhibition of T lymphocyte coactivation by triggering integrin beta 1 reveals convergence of beta 1, beta 2, and beta 7 signaling pathways. The Journal of Immunology. 1996;157:700-706.

**Table 1 (abstract P638).**
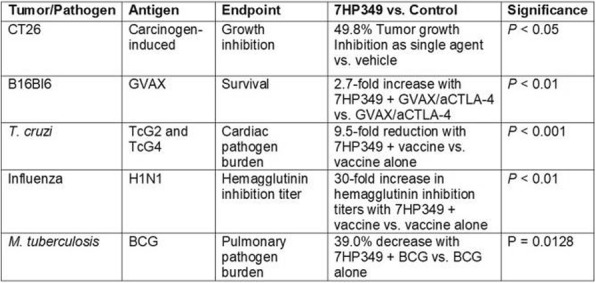
7HP349 augments antigenic immunity in mice

#### P639 Depth of tumor implantation predicts response to in situ vaccination in a syngeneic melanoma model - an important variable influencing response to immunotherapy

##### Peter Carlson, BS, Manasi Mohan, Mat Rodriguez, Vladimir Subbotin, Amy Erbe, PhD, Zachary Morris, MD, PhD, Alexander Rakhmilevich, MD, PhD, Paul Sondel, MD, PhD

###### University of Wisconsin School of Medicine, Madison, WI, United States

####### **Correspondence:** Paul Sondel (pmsondel@humonc.wisc.edu)

**Background**

An important component of research is ensuring rigor and reproducibility of work using animal models. We have identified a variable influencing the response of a syngeneic B78 melanoma flank tumor model to treatment with an in situ vaccine (ISV) regimen [1]. This study was prompted after two experimenters performing virtually identical studies were noted to obtain different results. Upon further evaluation, we observed different efficacy of ISV against B78 tumors when the tumors were implanted at different depths. Though the baseline in vivo immunogenicity of tumors can depend on depth of implantation, response to ISV as a function of tumor location, particularly in immunologically “cold” tumors, has not been thoroughly investigated. The goal of this study was to evaluate the difference in growth kinetics and response to ISV between identically sized melanoma tumors implanted at different depths in the skin.

**Methods**

We injected C57BL/6 mice with syngeneic B78 melanoma cells in the flank region. Half these mice were injected intradermally (ID), and half were injected subcutaneously (SQ). When tumors reached 190-230mm3, they were grouped into a ‘wave’ and treated according to our previously published in situ vaccine regimen: 12Gy local external beam radiation and intratumoral injections of hu14.18-IL2 immunocytokine [1]. Some mice from each wave were randomly assigned to be ‘untreated.’ Tumor volume and survival were monitored twice weekly for the duration of the experiment.

**Results**

Physical examination demonstrated that ID-implanted tumors were mobile upon palpation, while SQ-implanted tumors became fixed to the underlying fascia. Histologic examination identified a critical fascial layer, the panniculus carnosus, which separated ID and SQ tumors. SQ tumors reached the target tumor volume significantly faster compared to ID tumors. The majority of ID tumors exhibited either partial response or complete response to ISV, whereas the majority of SQ tumors did not. Direct comparison of ID vs. SQ tumors that reached target volume simultaneously further emphasized this difference in response to treatment. Immunohistochemistry evaluation of separately grown tumors demonstrated greater immune infiltrate following ISV in ID compared to SQ tumors.

**Conclusions**

We demonstrate that the physical fixed vs. mobile characterization of the tumors may be one method of ensuring implanted tumors are in the same tissue plane prior to initiation of treatment. Moreover, after controlling for strain of mouse, type, size, and growth rate of tumor, we demonstrate that depth of tumor implantation still has a dramatic effect on response to ISV.

**References**

1. Morris Z, Guy E, Werner L, Carlson P, Heinze C, Kler J, Busche S, Jaquish A, Sriramaneni R, Carmichael L, Loibner H, Gillies S, Korman A, Erbe A, Hank J, Rakhmilevich A, Harari P, Sondel P. Tumor-Specific Inhibition of In Situ Vaccination by Distant Untreated Tumor Sites. Cancer Immunol Res. 2018 Jul;6(7):825-834.

#### P640 Intratumoral application of the RNA-based TLR-7/-8 and RIG-I agonist CV8102 promotes a pro-inflammatory tumor microenvironment and causes a durable anti-tumor response

##### Michael Meister, PhD, Mohamed Habbeddine, PhD, Katja Fiedler, Johannes Lutz, Mallika Ramakrishnan, Regina Heidenreich

###### CureVac AG, Tübingen, Germany

####### **Correspondence:** Regina Heidenreich (regina.heidenreich@curevac.com)

**Background**

Intratumoral activation of innate immune signaling pathways is a promising approach to overcome the immunosuppressive tumor microenvironment and induce or restore anti-tumor immunity. CV8102, a non-coding RNA complexed with a cationic peptide that signals via TLR-7/-8 and RIG-I [1], has already been shown to exhibit immunomodulatory properties after intradermal injection [2]. Here, we investigated whether intratumoral injection of CV8102 alone or in combination with systemic anti-PD-1 treatment can also modulate the tumor microenvironment and enhance anti-tumoral responses.

**Methods**

Mice were injected subcutaneously with the syngeneic colon carcinoma cell line CT26 or the B cell lymphoma cell line A20 and established tumors were treated intratumorally with CV8102 alone or in combination with systemic anti-PD-1 antibodies. Anti-tumoral efficacy was determined in tumor bearing mice, which were treated twice weekly for three weeks. Additionally, cytokine concentrations, immune cell composition or gene expression within the tumor microenvironment, the draining lymph nodes or serum were analyzed at different time points post injection.

**Results**

Intratumoral administration of CV8102 led to a dose-dependent anti-tumoral response in syngeneic mouse models. Combination of local CV8102 delivery with systemic checkpoint inhibitors (CPIs) further improved the anti-tumoral response and enabled checkpoint inhibition efficacy in anti-PD-1 refractory tumor models. Gene expression profiling revealed activation of the innate immune compartment within the tumor microenvironment, reflected by up-regulation of genes involved in Type I interferon and anti-viral responses as well as RIG-I like, NOD-like and TNF signaling pathways. Accordingly, cytokine/chemokine analysis showed increased intratumoral concentrations of IFN-α/β, IL-6, TNF, MCP1, MIP1α/β and RANTES. Such effects were observed upon CV8102 single agent treatment and were more pronounced upon combination with systemic anti-PD-1 treatment. On the cellular level, intratumoral CV8102 monotherapy caused an increase in numbers and activation of innate immune cells like monocytes, neutrophils and NK cells within tumors and draining lymph nodes. Besides further enhancement of innate immunity, combination therapy synergistically enabled activation of the adaptive immune system and led to the infiltration of CD8+ T cells into the tumor microenvironment.

**Conclusions**

Our results demonstrate that intratumoral CV8102 delivery is a promising approach for local cancer immunotherapy. By modulating the tumor microenvironment CV8102 induces potent anti-tumoral immune responses and improves CPI efficacy in mice. An ongoing phase I trial is investigating intratumoral CV8102 either as a monotherapy or in combination with systemic anti-PD-1 treatment in various tumor entities (see abstract by Eigentler et al.).

**References**

1. Ziegler A, Soldner C, Lienenklaus S, Spanier J, Trittel S, Riese P, Kramps T, Weiss S, Heidenreich R, Jasny E, Guzmán CA, Kallen KJ, Fotin-Mleczek M, Kalinke U. A New RNA-Based Adjuvant Enhances Virus-Specific Vaccine Responses by Locally Triggering TLR- and RLH-Dependent Effects. J Immunol. 2017;198(4):1595-1605. doi: 10.4049/jimmunol.1601129

2. Heidenreich R, Jasny E, Kowalczyk A, Lutz J, Probst J, Baumhof P, Scheel B, Voss S, Kallen KJ, Fotin-Mleczek M. Int J Cancer. 2015 Jul 15;137(2):372-84. doi: 10.1002/ijc.29402

**Ethics Approval**

The study was approved by the Regierungspraesidium Tuebingen, Germany under Cur 07/18.

#### P642 Potent tumor-directed T cell activation and tumor inhibition induced by ALG.APV-527, a 4-1BB x 5T4 ADAPTIR™ bispecific antibody

##### Michelle H. Nelson, PhD^1^ , Robert Miller^2^, Robert Bader^2^, Doreen Werchau, BS^3^, Anneli Nilsson^4^, Lill Ljung^3^, Adnan Deronic, PhD^3^, Allison Chunyk^2^, Lena Schultz^3^, Catherine McMahan^2^, David Bienvenue^2^, Anna Dahlman^3^, Sara Fritzell^3,^ Maria Askmyr^2^, Gabriela Hernandez-Hoyos^2^

###### ^*1*^*Aptevo Therapeutics, Seattle, WA, United States;*^*2*^*Aptevo Therapeutics Inc., Seattle, WA, United States* ; ^*3*^*Alligator Bioscience AB, Lund, Sweden* ; ^*4*^*Alligator Bioscience, Lund, Sweden*

##### **Correspondence:** Michelle H. Nelson (nelsonm@apvo.com)

**Background**

4-1BB (CD137) is an activation-induced co-stimulatory receptor that regulates immune responses of activated CD8+ T and NK cells, by enhancing proliferation, survival, cytolytic activity and IFN-γ production. Its ability to induce potent anti-tumor CD8 and NK cell activity makes 4-1BB an attractive target for designing novel therapeutics for immuno-oncology. However, clinical development of a monospecific 4-1BB agonistic antibody was hampered by dose-limiting hepatic toxicities. ALG.APV-527 is a novel 4-1BB x 5T4 bispecific antibody developed to minimize systemic immune toxicities and enhance activity at the tumor site by stimulating 4-1BB function only when co-engaged with 5T4, a tumor-associated antigen. The combined preclinical dataset presented here provides an overview of the mechanism of action and the efficacy and safety profile of ALG.APV-527, supporting its advancement into the clinic.

**Methods**

ALG.APV-527 was built using the ADAPTIR™ platform with binding domains to 4-1BB and 5T4 generated using the ALLIGATOR-GOLD® human scFv library and subsequently optimized to increase binding affinity, function, stability and manufacturability. To assess function, induction of IFN-γ secretion by ALG.APV-527 was measured in PBMC cultures sub-optimally stimulated with anti-CD3 antibodies in the presence of 5T4+ tumor cells. To measure proliferation, PBMC were labelled with Cell TraceTM and T cells were gated using multicolor flow cytometry. For tumor inhibition studies, human 4-1BB knock-in mice were injected subcutaneously with murine bladder carcinoma MB49 cells transfected with human 5T4, and mice were treated therapeutically with ALG.APV-527. Preclinical safety and pharmacokinetics of ALG.APV-527 were evaluated in single and repeated dose toxicology studies in non-human primates (NHP).

**Results**

In primary PBMC assays, ALG.APV-527 induced a dose-dependent increase of IFN-γ production but only upon 5T4-mediated crosslinking. ALG.APV-527 enhanced CD8+ T cell and NK function and proliferation, preferentially over that of CD4+ T cells. Treatment with ALG.APV-527 led to rejection of established human 5T4-positive tumors at doses of 20 μg/mouse, in a syngeneic bladder carcinoma model. ALG.APV-527 is fully NHP cross-reactive and displayed antibody-like pharmacokinetic parameters in NHP studies. Furthermore, the drug was well tolerated after repeated administration at doses in excess of the expected human dose.

**Conclusions**

ALG.APV-527 induces 5T4-dependent CD8+ T cell and NK co-stimulation, and has potent anti-tumor activity in a therapeutic model. Based on preclinical data, ALG.APV-527 is a promising anti-cancer therapeutic for the treatment of a variety of 5T4-expressing solid tumors.

**Ethics Approval**

All studies were review and approved by the Internal Animal Care and Use Committee (IACUC) of Aptevo Therapeutics

#### P643 Harnessing pre-existing anti-viral immunity for tumor therapy

##### Cuburu Nicolas, PhD^1^, Lukas Bialkowski^2^, Sergio Pontejo, PhD^3^, Rina Kim, BS^2^, Alexander Bell^2^, Cynthia Thompson^2^, Douglas Lowy, MD^2^, John Schiller, PhD^2^

###### ^1^National Cancer Institutes, Bethesda, MD, United States; ^*2*^*National Cancer Institutes, NIH, Bethesda, MD, United States*; ^*3*^*NIAD, NIH, Bethesda, MD*

####### **Correspondence:** John Schiller (schillej@mail.nih.gov)

**Background**

The discovery that the tumor immune microenvironment can be used as biomarker of cancer progression and the advent of immunotherapies are revolutionizing cancer treatments. Notwithstanding these successes, there is still an urgent need for new therapeutics able to increase the proportion of clinical responses. Intratumoral delivery of immunotherapeutic agents constitutes a potential strategy to convert poorly immunogenic tumors into immunogenic tumors and to overcome acquired resistance to current immunotherapies.

**Methods**

Here we investigate a tumor antigen-agnostic intratumoral approach that harnesses preexisting antiviral T cell responses. To implement such approach, Human cytomegalovirus (hCMV) would be an ideal candidate as it is highly prevalent in human populations and induces polyfunctional T-cell responses that expand with age. In our experimental model, mice persistently infected with murine cytomegalovirus (mCMV) were challenged with TC-1 tumor cells expressing human papillomavirus E6 and E7 oncogenes or MC38 colon adenocarcinoma tumor cells. We investigated the effects of redirecting pre-existing mCMV-specific T cells by repeated intratumoral injection using minimal MHC class I and/or class II restricted mCMV peptide epitopes combined with polyI:C, a Toll-like receptor 3 and RIG-I-like receptor agonist.

**Results**

Intratumoral injection of mCMV MHC-I and MHC-II restricted peptides led to the expansion of CD8 and CD4 mCMV-specific T cells in situ and in blood. Intratumoral injection of mCMV MHC-I restricted peptides provoked arrest of TC-1 and MC38 tumor growth that sometimes resulted in complete tumor rejection. Interestingly, intratumoral injection of mCMV MHC-II restricted peptides with poly(I:C), without MHC-I peptides, also substantially delayed tumor growth. In addition, injection of mCMV MHC-II restricted peptides alone or in combination with mCMV MHC-I restricted peptides increased epitope spreading to MHC-I restricted tumor-derived epitopes, promoted clearance of established subcutaneous tumors, and conferred long-term protection against tumor re-challenge. Finally, analysis of the tumor microenvironment using whole tumor RNA gene expression profiling, flow cytometry and confocal microscopy revealed that intratumoral injection of mCMV MHC-I or MHC-II restricted epitopes caused local activation of adaptive and innate immune components.

**Conclusions**

Our results provide a proof-of-concept for a new generation of tumor antigen-agnostic immunotherapies based on pre-existing antiviral T cells that is potentially applicable to multiple tumor types. The approach induced rapid tumor regression, profound changes in the tumor immune microenvironment, epitope spreading, and long-term antitumor immunity. We are now using our model to decipher the contribution of CD4+ T cell to epitope spreading against tumor-derived epitopes and to evaluate this approach in spontaneous tumor models.

**References**

1. Aznar MA, Tinari N, Rullán AJ, Sánchez-Paulete AR, Rodriguez-Ruiz ME, Melero I. Intratumoral Delivery of Immunotherapy-Act Locally, Think Globally. J Immunol.2017 Jan 1;198(1):31-39.

2. Frank MJ, Reagan PM, Bartlett NL, Gordon LI, Friedberg JW, Czerwinski DK, Long SR, Hoppe RT, Janssen R, Candia AF, Coffman RL, Levy R. In Situ Vaccination with a TLR9 Agonist and Local Low-Dose Radiation Induces Systemic Responses in Untreated Indolent Lymphoma. Cancer Discov. 2018 Oct;8(10):1258-1269.

3. Ribas A, Medina T, Kummar S, Amin A, Kalbasi A, Drabick JJ, Barve M, Daniels GA, Wong DJ, Schmidt EV, Candia AF, Coffman RL, Leung ACF, Janssen RS. SD-101 in Combination with Pembrolizumab in Advanced Melanoma: Results of a Phase Ib, Multicenter Study. Cancer Discov. 2018 Oct;8(10):1250-1257.

#### P644 Collagen anchoring of intratumorally administered agonist antibodies improves efficacy and limits toxicities

##### Joseph Palmeri, BS, , Karl Wittrup, PhD

###### Massachusetts Institute of Technology, Cambridge, MA, United States

####### **Correspondence:** Karl Wittrup (wittrup@mit.edu)

**Background**

Agonist antibodies that target co-stimulatory receptors, including targets such as CD28, CD40, CD137, and OX40, have demonstrated impressive efficacy in preclinical cancer models. Broadly, drugging these receptors leads to immune cell proliferation, cytokine production, and enhanced effector function. In the clinic, however, the development of these antibody drugs has been hampered by dose-limiting toxicities which have limited their utility and prevented approval of any co-stimulatory receptor targeting agonist antibodies. Novel strategies are required to dose these drugs in a safe manner while still retaining their anti-tumor activity.

**Methods**

To contain the activity of agonist antibodies to the tumor microenvironment (TME), we have fused a collagen binding domain (CBD) to the Fc region of several agonist antibodies targeting a suite of co-stimulatory receptors. We have recently validated this collagen anchoring approach to improve the therapeutic index of cytokine therapies [1]. Fluorescent imaging was used to characterize intratumoral retention and the biodistribution of intratumorally injected antibodies. Survival studies were carried out to determine the efficacy of antibodies with and without CBDs in murine subcutaneous tumor models. We measured toxicity by quantifying serum protein levels as well as with histology in treated mice.

**Results**

Imaging studies revealed that antibody-CBD fusions were retained significantly longer in the TME compared to their non-CBD counterparts and experienced little to no detectable systemic dissemination. Intratumorally administered antibody-CBD fusions displayed comparable, or in some cases, improved efficacy when benchmarked against free antibody. These fusion proteins also caused markedly fewer signs of toxicity in murine models.

**Conclusions**

Intratumorally administered antibody-CBD fusions represent a new class of therapeutics with improved therapeutic indices compared to agonist antibodies that have already entered the clinic. Because intratumoral retention is dependent on collagen and not a tumor specific target, these drugs are tumor-agnostic and have the potential to impact large patient populations.

**References**

1. Momin et al., Anchoring of intratumorally administered cytokines to collagen safely potentiates systemic cancer immunotherapy. Sci Transl Med. 2019; 11; eaaw2614

#### P645 A tumor necrosis factor alpha mimetic as anti-tumor immune adjuvant

##### Ketki Velankar, MS, Ngoc Pham, BS, Ellen Gawalt, Wilson Meng, PhD

###### Duquesne University, Pittsburgh, PA, United States

####### **Correspondence:** Wilson Meng (meng@duq.edu)

**Background**

Intra-tumoral immunotherapy has emerged as an important modality of delivering anti-tumor immunomodulators. Rich in tumor-associated antigens and antigen-presenting cells, the tumor microenvironment is an apt locale for reprogramming the immune responses from a suppressive phenotype to one that is activating insofar anti-tumor cytotoxic T cell responses can be generated locally and systemically.

Herein we report a small peptide that can potentially serve as an adjuvant for enhancing antigen presentation in the tumor microenvironment. Our group discovered the peptide YTYQGKL, called pTNF, as a ligand of tumor necrosis factor receptor II (TNFRII) by screening phage display libraries on dendritic cells (DCs) [1]. The peptide binds to DCs in a concentration-dependant, TNFalpha-reversible manner, albeit at micromolar binding affinity. Subsequently, other groups have confirmed the DC-targeting property of pTNF[2,3]. In addition, it has been shown that pTNF upregulates costimulatory molecules on DCs [4,5]. This led us to hypothesize that pTNF can be used as an immune adjuvant in the context of cancer immunotherapy. To transform the low affinity ligand pTNF into a high avidity adjuvant, the peptide is appended with [AEAEAKAK]2, a self-assembling peptide (SAP) that drives oligomerization (**Fig.** 1a).

**Methods**

To test the capacity of pTNF_EAK in activating antigen presenting cells, BALB/c mice were injected with 100 mcg of the peptide in each footpad. After 48 hours, the draining lymph nodes were collected and analyzed using flow cytometry. The cells obtained from draining lymph nodes were restimulated ex vivo with a mouse renal adenocarcinoma (RENCA) cell line for 48hours. The cells were analyzed using flow cytometry and ELISA, quantifying interferon-gamma (IFN gamma) production upon in vitro restimulation.

**Results**

Preliminary results indicated that pTNF_EAK exhibited self-assembling characteristics based on Congo red staining (data not shown). Flow cytometric analyses showed upregulated CD40 on cells in pTNF_EAK treated draining lymph nodes compared to control footpads injected with saline (**Fig.** 1b). Upon restimulation in vitro with RENCA cells, pTNF_EAK treated lymph node-derived cells produced higher levels of (IFN gamma) compared to control cells (data not shown).

**Conclusions**

pTNF_EAK appears to upregulate the functions of antigen-presenting cells in draining lymph nodes in vivo.

**References**

1. Chamarthy SP, Jia L, Kovacs JR, Anderson KR, Shen H, Firestine SM, Meng WS. Gene delivery to dendritic cells facilitated by a tumor necrosis factor alpha-competing peptide. Molecular immunology. 2004 Jul 1;41(8):741-9

2. Margaroni M, Agallou M, Kontonikola K, Karidi K, Kammona O, Kiparissides C, Gaitanaki C, Karagouni E. PLGA nanoparticles modified with a TNFα mimicking peptide, soluble Leishmania antigens and MPLA induce T cell priming in vitro via dendritic cell functional differentiation. European Journal of Pharmaceutics and Biopharmaceutics. 2016 Aug 1;105:18-31.

3. Herringson TP, Altin JG. Convenient targeting of stealth siRNA-lipoplexes to cells with chelator lipid-anchored molecules. Journal of Controlled Release. 2009 Nov 3;139(3):229-38.

4. Caux C, Dezutter-Dambuyant C, Schmitt D, Banchereau J. GM-CSF and TNF-α cooperate in the generation of dendritic Langerhans cells. Nature. 1992 Nov;360(6401):258.

5. Caux C, Massacrier C, Vanbervliet B, Dubois B, Van Kooten C, Durand I, Banchereau J. Activation of human dendritic cells through CD40 cross-linking. Journal of Experimental Medicine. 1994 Oct 1;180(4):1263-72.

**Ethics Approval**

The study was approved by Duquesne University's IACUC, protocol number: 1806-04

**Fig. 1 (abstract P645).**
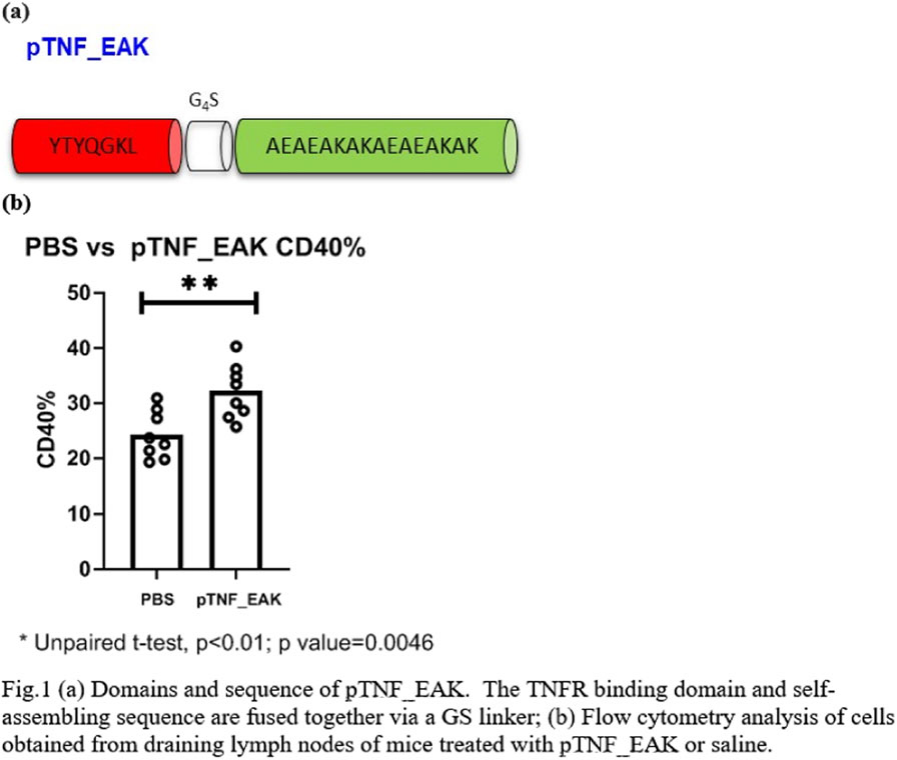
See text for description

#### P646 Reprogramming CD8 cells into metabolically fit stem cell memory with superior therapeutic activity by MEK1/2 manipulation.

##### Vivek Verma, PhD, Pankaj Gaur, PhD, Jose Lopez, Fatmah Alolaqi, Seema Gupta, PhD, Samir Khleif, MD

###### Georgetown University Medical Center, Washington, DC, United States

####### **Correspondence:** Samir Khleif (snk48@georgetown.edu)

**Background**

Exhaustion and short persistence are factors that limit the efficacy of endogenously generated or adoptively transferred effector T-cells. Metabolism in T-cells has been found to be a significant factor that contributes to their anti-cancer potential. Interestingly, tumor-induced metabolic insufficiency in immune cells renders these cells exhausted and hence ineffective in controlling the tumors. Hence the mechanisms that would prevent the metabolic degradation in immune cells would have strong anti-cancer potential. Renewable stem-cell-memory CD8+T-cells (Tscm) are metabolically fit cells that persist longer and produce stronger effector functions. Here, we determined the role of MAP kinase pathway in the generation of Tscm CD8 cells and their metabolic status.

**Methods**

Mice bearing TC-1 tumors (a mouse lung epithelial cell-line expressing human papillomavirus-specific E7-peptide) were treated with MEKi (selumetinib) in conjunction with tumor-specific E7-peptide vaccine, followed by determination of tumor growth rates and mice survival, and estimation of immune responses in the TME. Also, using in-vitro and in-vivo assay systems, we delineated the kinetics of MEKi-mediated Tscm generation and the molecular mechanisms of metabolic enhancement of CD8 cells.

**Results**

We found that MEKi treatment of tumor-bearing mice in conjugation with tumor-specific E7-vaccine resulted in a significant reduction in tumor growth and enhancement of mice survival. This treatment resulted in a strong increase in the effector functions and prevention of exhaustion in effector cells. We observed a significant increase in the number of Sca1+CD95+CD62L+CD44-CD8+ T-cells (Tscm cells) in the TME and under in-vitro conditions. Interestingly, we found that MEKi suppressed the cell-cycle progression in antigen-activated CD8 cells that led to increased mitochondrial biogenesis and fatty-acid oxidation (FAO) by modulating ERK1/2/CyclinD1/PGC1α/SIRT3 signaling pathway. FAO was crucial for MEKi-mediated induction of Tscm cells as its pharmacological inhibition or SIRT3KO prevented the Tscm generation. Further, ex-vivo MEKi-treated CD8+T-cells showed strong cell-activation, high antigen recall-responses and prolonged-survival, generating robust T-cells that significantly enhanced the efficacy of adoptive cell therapy (ACT) that was critically dependent upon MEKi-induced Tscm cells. Together, we show that MEKi-induced Tscm generation occurs as a result of a crosstalk between cell activation, cell-cycle progression and differentiation, and metabolic machinery.

**Conclusions**

Our data provide a novel strategy to reprogram CD8 cells into a self-regenerating stem cell phenotype with higher metabolic fitness and superior effector functions. This data also provide an effective approach to produce superior T-cells for in-vitro treatments such as ACT. Furthermore, the findings from this study provide a strategy for enhancement of treatment approaches using inhibitory molecules.

#### P647 Is intracellular STING expression a biomarker for oncolytic herpes virus immunotherapy?

##### Praveen Bommareddy, MS, PhD^1^, Samuel Rabkin, PhD^2^, Andrew Zloza, MD, PhD^3^, Howard Kaufman, MD, FACS^2^

###### ^*1*^*Rutgers University, Woburn, MA, United States;*^*2*^*Massachusetts General Hospital, Boston, MA* ; ^*3*^*Rush University, Chicago, IL, United States*

####### **Correspondence:** Howard Kaufman (Howard.Kaufman@replimune.com)

**Background**

Talimogene laherparepvec (T-VEC) is an oncolytic herpes simplex virus, type 1 (HSV-1) encoding granulocyte-macrophage colony stimulating factor (GM-CSF) and is approved for the treatment of melanoma. To date, there are no predictive biomarkers to identify patients likely to respond to T-VEC treatment, which is a high priority. Thus, we sought to identify intracellular factors associated with host anti-tumor immunity following T-VEC treatment.

**Methods**

Human melanoma cell lines were plated in 96-well plates (104 cells per well) and treated with T-VEC (MOI 0.001-1.0). At various time points, cell viability was assessed by standard MTS assay. To evaluate specific intracellular factors, knock out cell lines were generated by using CRISPR-Cas9 technology and assessed for oncolytic activity in vitro. For in vivo experiments, B6 mice bearing the HSV-1-sensitive D4M3A (3 x 105) melanoma bilateral flank tumor model was used. Mice were treated by injection in the right flank only with a T-VEC (6 x 106 PFU) once tumors reached 9-12 mm2 three times a week for two weeks. Tumor growth was measured by calipers daily for tumor growth and survival studies. In separate experiments, tumors were harvested at specified timepoints and flow cytometry analysis was performed to characterize immune cell populations and RNA was extracted for Nanostring gene expression analysis. Statistical comparisons between treatment groups were determined using the Student’s t test with and the Kaplan-Meier method was used to estimate survival. P

**Results**

We initially interrogated multiple human melanoma cell lines and found differential sensitivity to T-VEC-mediated oncolysis. Characterization of the cell lines suggested that STING expression, but not PKR or cGAS, was inversely associated with T-VEC-mediated cell lysis. This was confirmed using CRSPR-Cas9-generated STING knockout cells which reversed T-VEC resistance. Induction of host immunity was confirmed in vivo using a STING deficient melanoma tumor model, which is resistant to PD-1 blockade, and demonstrated significant delay in tumor growth and improved survival following treatment with T-VEC. Therapeutic responses were associated with recruitment of viral- and tumor antigen-specific CD8+ T cells and induction of a pro-inflammatory gene signature in both injected and un-injected tumors.

**Conclusions**

Intracellular STING was inversely associated with T-VEC-mediated lysis and suggests that STING may be a predictive biomarker for T-VEC responses. These data also support a role for T-VEC in restoring host immune responses in STING-deficient melanoma.

**Ethics Approval**

This study was approved by the Rutgers University IACUC.

#### P648 Therapeutic efficacy of oncolytic vaccinia virus requires infection and removal of deleterious host immune cells

##### Kristin DePeaux, BS, Greg Delgoffe, PhD, Saumendra Sarkar, PhD, Steve Thorne, PhD, Paolo Vignali, BA, Dayana Rivadeneira, PhD

###### University of Pittsburgh, Pittsburgh, PA, United States

####### **Correspondence:** Greg Delgoffe (delgoffeg@upmc.edu)

**Background**

Oncolytic virotherapy is an attractive immunotherapeutic approach as it can induce robust immune infiltration in immunologically cold tumors. While there is one OV approved for use by the FDA and many others in clinical trials, little is understood of their immunologic mechanism of action. The effect of OV treatment on the existing immune infiltrate in the tumor is especially understudied. Seven days after one intratumoral treatment of oncolytic vaccinia (vv) in a murine head and neck squamous cell carcinoma model we observed an influx of CD8 T cells into the tumor, including a large population of effector-memory like PD-1int cells. However, the suppressive PD-1hi Tim-3+ exhausted T cells (Texh) and Foxp3+ regulatory T cells (Treg) were missing. We hypothesized that vvDD may have the capacity to infect these inhibitory populations and lead to their selective deletion.

**Methods**

Infection and flow cytometric analyses of T cell populations were performed using both in vitro and in vivo modeling systems. An oncolytic Western Reserve strain of vaccinia virus (vv) was used at a multiplicity of infection of 10 for all in vitro experiments and at 2.5E6 PFU per mouse for in vivo experiments. T-reg specific BCL2 over-expressing mice were generated by crossing Rosa26-BCL2-IRES-GFP mice to Foxp3-YFP-cre mice.

**Results**

Analysis of the immune infiltrate of treated tumor-bearing mice revealed that Texh and Treg in the tumor were actively infected by virus. This may lead directly to the death of these infected T cells. This phenomenon was recapitulated in vitro under nutrient limited conditions. This was further investigated in vivo using a mouse model where the anti-apoptotic protein BCL2 was selectively overexpressed in Tregs. In this model the death of Tregs is prevented in the tumor. These mice were unable to clear tumor after vv treatment while all wild-type animals did.

**Conclusions**

These data suggest that the mechanism of action of oncolytic vaccinia also includes the direct ability of the virus to infect immune populations in the tumor. Infection of regulatory T cells in the tumor leads to their death. By preventing the death of these cells, treatment efficacy is lost, thus highlighting the importance of this mechanism during oncolytic vaccinia treatment.

#### P649 Targeting subtypes of circulating myeloid cells during oncolytic vaccinia virus therapy

##### Bora Lee^1^, Fen Jiang^2^, Mong Cho, MD, PhD^1^, Euna Cho^2^, Tae-Ho Hwang, PhD, DDS^1^

###### ^1^Pusan National University, Pusan, Korea, Republic of; ^2^Bionoxx Inc, Yangsan, Korea, Republic of

####### **Correspondence:** Tae-Ho Hwang (thhwang@pusan.ac.kr)

**Background**

Oncolytic virus (OV) is an emerging class of cancer treatment which has received increasing attention. The pharmacodynamics (safety and efficacy) of OV shows large inter-individual variability―the clinical outcomes of OV treatments can range from complete response to possibly treatment-related early death in different patients [1,2]. The innate immune system as well as adaptive immune system play critical roles in governing the OV treatment outcomes. This study aimed to investigate the therapeutic potential for the interaction between circulating myeloid cells, oncolytic vaccinia virus (OVV), and cancer cells; and to optimize the treatment outcomes of OTS-400 (OVV series with modified HSV-tk insertion).

**Methods**

The functional outcome of interaction between circulating immune cells, OVV, and cancer cells was evaluated quantitatively and qualitatively using a trans-well co-culture system and analyzed by fluorescence-activated cell sorting. After identification of subtypes of OV-disfavoring myeloid cells (OV-DMCs), candidate chemicals, including clinically used drugs, were screened to find the best modulators for OV-DMCs affecting clinical outcome of OTS-400 series in >3,000 tumor-bearing animals.

**Results**

Time to 90% lethality after intranasal administration of Western Reserve (107 pfu) vaccinia virus was significantly prolonged by inhibition of OV-DMCs, 30 days+; by stimulation, 7 days; and by no modulation, 11 days (Log-rank p-value

**Conclusions**

OV-DMCs play a critical role in the pharmacodynamics of OVV therapy in a variety of preclinical tumor models. Targeting OV-DMCs may significantly improve the treatment outcome for the upcoming clinical trials of the combination therapy of OTS-400 and PD-L1 inhibitor scheduled in early 2020.

**Acknowledgements**

This study was supported by a grant from the National R&D Program for Cancer Control, Ministry of Health and Welfare, Republic of Korea (HA16C0013) and National Research Foundation of Korea (NRF) grant funded by the Korea government (MSIP) (NRF-2015R1A5A2009656).

**References**

1.Park BH, Hwang T, Liu TC, Sze DY, Kim JS, Kwon HC, Oh SY, Han SY, Yoon JH, Hong SH et al: Use of a targeted oncolytic poxvirus, JX-594, in patients with refractory primary or metastatic liver cancer: a phase I trial. Lancet Oncol 2008, 9(6):533-542.

2.Breitbach CJ, Burke J, Jonker D, Stephenson J, Haas AR, Chow LQ, Nieva J, Hwang TH, Moon A, Patt R et al: Intravenous delivery of a multi-mechanistic cancer-targeted oncolytic poxvirus in humans. Nature 2011, 477(7362):99-102.

#### P650 Systemic immunity against disseminated subcutaneous and intracranial melanomas induced by oncolytic adenovirus Delta-24-RGDOX

##### Hong Jiang, PhD, Dong Ho Shin, Teresa Nguyen, BS, Frederick Lang, MD, Candelaria Gomez-Manzano, MD, Juan Fueyo

###### University of Texas MD Anderson Cancer Center, Houston, TX, United States

####### **Correspondence:** Juan Fueyo (jfueyo@mdanderson.org)

**Background**

Immune checkpoint blockade has revolutionized cancer therapy; however the therapeutic benefit is limited to only a subset of patients with immunogenic (“hot”) tumors and is compromised by immune-related adverse events. We have reported that intratumoral injection of oncolytic adenovirus Delta-24-RGDOX, an oncolytic adenovirus expressing immune co-stimulator OX40 ligand (OX40L), induces efficacious anti-glioma immunity and immune memory in syngeneic glioma mouse models. We hypothesized that localized treatment with the virus is effective against disseminated melanomas, including intracranial melanomas.

**Methods**

We tested the therapeutic effect of injecting Delta-24-RGDOX into primary subcutaneous (s.c.) tumors derived from luciferase-expressing B16-Red-FLuc cells in s.c./s.c. and s.c./intracranial (i.c.) melanoma models in C57BL/6 mice. The growth of treated s.c. and untreated disseminated s.c. and i.c. tumors, luciferase-expressing ovalbumin-specific (OT-I/Luc) T cells within the tumor were monitored via bioluminescence imaging. Cells were profiled for surface markers using flow cytometry.

**Results**

In both s.c./s.c. and s.c./i.c. models, three injections of Delta-24-RGDOX significantly inhibited the growth of both the virus-injected s.c. tumor and untreated distant s.c. or i.c. tumor, thereby prolonging survival. The surviving mice were protected from rechallenging with the same tumor cells. The virus treatment increased the presence of T cells and the frequency of effector T cells in the virus-injected tumor and mediated the same changes in T cells from peripheral blood, tumor-draining lymph nodes (TDLNs), spleens, and brain hemispheres with untreated tumor. Moreover, Delta-24-RGDOX decreased the frequency of exhausted T cells and regulatory T cells in the virus-injected and untreated i.c. tumors. Consequently, the virus promoted the in situ expansion of tumor-specific T cells and their migration to tumors expressing the target antigen.

**Conclusions**

Intratumoral injection of Delta-24-RGDOX induced an in situ antovaccination of the treated melanoma, resulting in systemic immune activity against the disseminated tumors. This is the first report demonstrating that localized treatment with an oncolytic virus in the subcutaneous tumor is able to reject intracranial tumors, suggesting Delta-24-RGDOX could be applied to patients with brain melanoma metastasis. Furthermore, given the immunogenicity, cancer-selectivity and localized application of the virus, Delta-24-RGDOX is expected to be efficacious in patients with immunosuppressive (“cold”) tumors as well and have an improved safety profile in cancer patients compared to immune checkpoint blockade treatment strategies.

#### P651 Oncolytic Vaccinia virus contains a potent CD80/CD86 ligand whose deletion confers higher tolerance, and potential synergy with immune arming

##### Eric Quemeneur, PharmD, PhD, Eric Quemeneur, PharmD, PhD, Patricia Kleinpeter, MSc, Jean Baptiste Marchand, PhD, Laetitia Fend

###### Transgene S.A., Illkirch-Graffenstaden, France

####### **Correspondence:** Eric Quemeneur (quemeneur@transgene.fr)

**Background**

Vaccinia virus (VACV) has been intensively used as oncolytic virus for the treatment of various types of cancers over the last years. Targeted gene deletion have enabled the selection of new VACV variants that retain tumor-specific replication, and oncolytic potential, but are safer for the surrounding healthy tissues. Recent examples of such deleted VACVs are TG6002, deltaJ2R(TK-)deltaI4L(RR-)-Fcu1 (Foloppe et al. 2019), and DeltaJ2R(TK-)DeltaF1L (Pelin et al. 2019). It is also well established that VACV secretes various factors interfering with major immune pathways, largely contributing to immune evasion for the virus. However, to our knowledge, none of these factors has already been used in the field of cancer immunotherapy.

**Results**

We here report the identification of M2L as a strong binder to both CD80, and CD86 co-stimulatory receptors. This binding antagonizes the interaction with their natural ligands CD28, and CTLA-4. M2L can also stabilize the interaction between CD80, and PD-L1 in vitro. We characterized M2L as a secreted homo-oligomeric protein (8 x 35 kDa), and could determine that apparent affinities are in the same range as natural ligands. It proved as active as CTLA-4, as an inhibitor for lymphocyte activation in a MLR assay.

M2L properties thus make it a potential new immuno-suppressive drug. Interestingly, M2L is largely conserved within the poxvirus family, and we could demonstrate that its ortholog from myxomavirus can also interact with CD86.

Expecting to reinforce the immunogenic properties of VACV, we engineered a triple-deleted Vaccinia virus (TD, DeltaJ2R, DeltaI4L, DeltaM2L). Oncolytic activity was not affected by the deletion of M2L, as assessed both in the regular mouse tumor xenograft models (HCT116), or in the syngeneic models (B16F10). We could demonstrate a better tolerance for the TD variant in a humanized model, where the CD80/CD86 pathway might be prominent in the neutralizing response.

**Conclusions**

Finally, the TD oncolytic backbone might be interesting for the development of our invirIO™ platform. Recent results with triple-deleted VACV expressing immune engagers will be presented.

**References**

1. Kleinpeter et al. By binding CD80 and CD86, the Vaccinia virus M2 protein blocks their interactions with both CD28 and CTLA4 and potentiates CD80 binding to PD-L1. J. Virol. 2019, 93: e00207-19

2. Foloppe et al. The enhanced tumor specificity of TG6002, an armed oncolytic Vaccinia virus deleted in two genes involved in nucleotide metabolism. Mol Ther Oncolytics 2019, 14: 1-14

3. Pelin et al. Deletion of apoptosis inhibitor F1L in Vaccinia virus increases safety and oncolysis for cancer therapy. Mol Ther Oncolytics 2019 (in press)

#### P652 The fully human antibody SRF617 is a potent enzymatic inhibitor of CD39 with strong immunomodulatory activity

##### Michael Warren, Sonia Das, Austin Dulak, Tauqeer Zaidi, Erik Devereaux, Jamie Strand, Benjamin Lee, MD PhD, Secil Koseoglu, Pamela Holland, PhD, Vito Palombella, PhD, Andrew Lake, PhD

###### Surface Oncology, Inc., Cambridge, MA, United States

####### **Correspondence:** Michael Warren (mwarren@surfaceoncology.com)

**Background**

The purine nucleoside adenosine dampens innate and adaptive immune responses under various inflammatory conditions [1]. In contrast, high levels of extracellular adenosine triphosphate (ATP), generated as a result of tissue damage or immunogenic cell death, can initiate proinflammatory responses [2]. Extracellular adenosine accumulates in cancerous tissues through the degradation of ATP and constitutes an important mechanism of tumor immune escape, induction of angiogenesis, and metastasis [3]. Reduction of ATP and subsequent increase of extracellular adenosine production in the tumor microenvironment (TME) depend on the concerted enzymatic activity of two ectonucleotidases, CD39 (ENTPD1) and CD73 (NT5E), which convert ATP to adenosine monophosphate (AMP), and AMP to adenosine, respectively. Targeting this enzymatic pathway by inhibition or loss of CD39 has been shown to have antitumor effects in multiple studies [4-7]. The preclinical characterization of SRF617, a fully human CD39 antibody that potently inhibits CD39-mediated conversion of ATP to AMP, is described.

**Methods**

A series of studies were conducted to examine the CD39 targeting properties of SRF617. Biolayer interferometry (BLI) and flow cytometry-based cellular binding assays were used to examine the affinity of SRF617 to both recombinant and cellular CD39. The malachite green phosphate detection assay was used to assess the enzymatic inhibitory properties of SRF617 on recombinant and cellular CD39. In vitro functional assays to observe SRF617 modulation of dendritic cell (DC) maturation and T-cell proliferation were performed. Antitumor activity of SRF617 was assessed in a MOLP-8 xenograft model with multiple pharmacodynamic endpoints including immune cell infiltration, target occupancy, and in situ CD39 activity.

**Results**

SRF617 displays single-digit nanomolar inhibition of CD39 enzymatic activity and binds tightly to CD39 on cells. In vitro, SRF617 enhances DC maturation and T-cell proliferation under high ATP conditions. In vivo, SRF617 has significant single-agent antitumor activity in the MOLP-8 xenograft model. In addition, SRF617 elevates ATP levels systemically in treated mice and increases tumor macrophage infiltration in MOLP-8 xenograft tumors. An in situ CD39 enzymatic assay demonstrates that SRF617 inhibits CD39 activity in the MOLP-8 tumor xenograft model and correlates with target occupancy.

**Conclusions**

These studies demonstrate that SRF617 blocks CD39 enzymatic activity, thereby reversing the anti-inflammatory/immunosuppressive TME caused by reduced extracellular ATP and the generation of adenosine. These findings support future clinical studies of SRF617 as an immune-modulating medicine in patients with cancer.

**References**

1. Antonioli L, Blandizzi C, Pacher P, Hasko G. Immunity, inflammation and cancer: a leading role for adenosine. Nat Rev Cancer. 2013;13(12):842-57.

2. Di Virgilio F, Sarti AC, Falzoni S, De Marchi E, Adinolfi E. Extracellular ATP and P2 purinergic signalling in the tumour microenvironment. Nat Rev Cancer. 2018;18(10):601-18.

3. Spychala J. Tumor-promoting functions of adenosine. Pharmacol Ther. 2000;87(23):161 73.

4. Feng L, Sun X, Csizmadia E, et al. Vascular CD39/ENTPD1 directly promotes tumor cell growth by scavenging extracellular adenosine triphosphate. Neoplasia. 2011;13(3):206-16.

5. Jackson SW, Hoshi T, Wu Y, et al. Disordered purinergic signaling inhibits pathological angiogenesis in cd39/Entpd1-null mice. Am J Pathol. 2007;171(4):1395-404.

6. Sun X, Wu Y, Gao W, et al. CD39/ENTPD1 expression by CD4+Foxp3+ regulatory T cells promotes hepatic metastatic tumor growth in mice. Gastroenterology. 2010;139(3):1030-40.2000;87(2 3):161-73.

7. Zhang H, Vijayan D, Li XY, et al. The role of NK cells and CD39 in the immunological control of tumor metastases. Oncoimmunology. 2019;8(6):e1593809.

**Ethics Approval**

Mice were used in compliance with protocols approved by the IACUC of Mispro Biotech Services, Cambridge, MA (#2017-03-21SUR-1).

#### P653 Labeling method for flow cytometric analysis of radioactive tumors following immunotherapy and molecular targeted radionuclide therapy (mTRT): demonstration of augmented immune infiltrate

##### Peter Carlson, BS, Manasi Mohan, Ravi Patel, MD, PhD, Lauren Nettenstrom, Dagna Sheerar, Kathryn Fox, Mat Rodriguez, Anna Hoefges, MS, Reinier Hernandez, PhD, Chris Zahm, PhD, Douglas McNeel, MD, PhD, Jamey Weichert, PhD, Zachary Morris, MD, PhD, Paul Sondel, MD, PhD

###### University of Wisconsin School of Medicine, Madison, WI, United States

####### **Correspondence:** Paul Sondel(pmsondel@humonc.wisc.edu)

**Background**

We are investigating low dose mTRT as an immunomodulator in combination with in situ tumor vaccines (ISV). Most shared resource flow cytometry facilities do not analyze radioactive samples. To study the immunomodulatory effect of mTRT, we evaluated different fixation and cryopreservation protocols allowing radioactive tumor samples to be stained, frozen and safely stored until radioactivity decayed to background, enabling flow analyses. Our aim is to identify a protocol which most accurately reflects the tumor immune microenvironment (TME) of a freshly analyzed sample.

**Methods**

We generated syngeneic B78 melanoma flank tumors in C57BL/6 mice. Cohorts of mice received: 1) no treatment; 2) our previously reported ISV regimen (12Gy local external beam radiation and intratumoral injections of hu14.18-IL2 immunocytokine [1,2]); or 3) a combination of ISV and 50μCi of 90Y-NM600 mTRT [3]. We then harvested, dissociated and labeled tumor infiltrating immune cells according to one of four protocols: 1) freshly labeled cells; 2) freezing cells before staining; 3) freezing cells just before fixation; or 4) freezing cells after staining and fixation. Cells were labeled with panels specific for adaptive and innate immune cells.

**Results**

All three staining protocols involving cryopreservation performed similarly to freshly stained cells. Protocols 2 and 3 resulted in increased PD1 expression compared to fresh cells, suggesting cryopreservation and thawing of unfixed cells impacts the detection of the activation state of the lymphocytes. Protocol 4 most closely resembled the labeling of “fresh” cells. In comparing untreated tumors to those treated with ISV, we analyzed half of each sample “fresh” and half using staining protocol #4. We observed the same immune stimulatory effect of in situ vaccination using both protocols, confirming that freezing cells after labeling and fixing can be used to evaluate changes in the TME. Lastly, we used protocol #4 on a time course of weekly harvested tumors following mTRT alone, in situ vaccine alone, or combination mTRT+ISV. We demonstrated greater immune infiltrate and an increased CD8/Treg ratio when combining mTRT with ISV than either alone.

**Conclusions**

We demonstrate that fixation and cryopreservation after labeling closely reproduces the analyses of freshly dissociated tumors. We confirm this by comparing tumors treated with several immunotherapies using both the traditional and modified protocols. Lastly, we use this protocol with radioactive specimens to demonstrate that low dose mTRT followed by ISV results in greater immune infiltrate and activation with this combination compared to ISV or mTRT alone.

**Acknowledgements**

Thank you to Archeus Technologies of Madison, WI for their kind gift of the NM600 mTRT agent.

**References**

1. Morris Z, Guy E, Werner L, Carlson P, Heinze C, Kler J, Busche S, Jaquish A, Sriramaneni R, Carmichael L, Loibner H, Gillies S, Korman A, Erbe A, Hank J, Rakhmilevich A, Harari P, Sondel P. Tumor-Specific Inhibition of In Situ Vaccination by Distant Untreated Tumor Sites. Cancer Immunol Res. 2018 Jul;6(7):825-834.

2. Morris Z, Guy E, Francis D, Gressett M, Werner L, Carmichael L, Yang R, Armstrong E, Huang S, Navid F, Gillies S, Korman A, Hank J, Rakhmilevich A, Harari P, Sondel P. In Situ Tumor Vaccination by Combining Local Radiation and Tumor-Specific Antibody or Immunocytokine Treatments. Cancer Res. 2016 Jul 1;76(13):3929-41.

3. Grudzinski J, Hernandez R, Marsh I, Patel R, Aluicio-Sarduy E, Engle J, Morris Z, Bednarz B, Weichert J. Preclinical Characterization of 86/90Y-NM600 in a variety of murine and human cancer tumor models. J Nucl Med. 2019 Apr 6.

#### P654 CD137 agonists as an adjunct to immune checkpoint inhibitors to overcome resistance in melanoma

##### Sreedevi Danturti, PhD, Lena Sophie Mayer, MD, Vesselin Tomov

###### University of Pennsylvania, Fountainville, PA, United States

####### **Correspondence:** Vesselin Tomov (vesselin.tomov@uphs.upenn.edu)

**Background**

Melanoma is currently the fifth most common cancer in men and sixth most common in women with an estimate of 7230 deaths from the disease in 2019 in the United States. Treatment with the immune checkpoint inhibitors anti-CTLA-4 and anti-PD-1 has tremendously improved outcomes for patients with metastatic disease, but although durable response can be observed in over 50% of the patients treated with monotherapy, resistance is still present. Studies suggest that combining therapies (e.g. immune checkpoint inhibitors, radiotherapy, and/or agonistic antibodies) are more effective than monotherapy. 4-1BB (CD137) is expressed on various cell populations including T cells, dendritic cells and macrophages. 4-1BB agonists have been proved to be effective in multiple murine tumor models and have had modest responses in early clinical trials. We hypothesize that 4-1BB agonists augment the anti-PD-1 anti-tumor immune response resulting in improved overall survival and decreased tumor burden in melanoma resistant to pre-treatment with a combinational therapy of anti-CTLA-4 and radiation

**Methods**

499 melanoma cells, derived from unirradiated B16-F10 tumors resistant to treatment with anti-CTLA-4 and radiation, were implanted on both flanks of C57BL6 mice. Treatment with 4-1BB agonists and/or anti-PD1 was given on day 10, 13 and 16 post-implantation. The anti-tumor response was assessed by both overall survival and tumor burden. Tumors were harvested on day 17-20 for protein, transcriptomic and flow cytometric immune profiling

**Results**

Dual immunotherapy with 4-1BB agonist and anti-PD-1 significantly increases overall survival, whereas there is no benefit from treatment with either antibody alone. With combinational treatment, complete remission was achieved in 45.8%, however, there is still resistance to immunotherapy with response and nonresponse. Investigating mechanisms of response and non-response to dual immunotherapy with 4-1BB and anti-PD-1, we identified cells from the innate and adaptive immune system to play important roles in the anti-tumor response. Our proteome array analysis revealed several proteins of the myeloid and lymphoid compartment to be upregulated in the non-responders. Transcriptomic profiling by Nanostring IO 360 pan cancer panel in the non-responders and responders to immunotherapy corroborated our findings and detected important genes of the myeloid and lymphoid compartment especially macrophages and CD8+ T cells respectively

**Conclusions**

Overcoming resistance to immunotherapy crucially depends on cells of the innate and adaptive immune system especially the macrophages and CD8+ T cells. Investigation of response mechanisms revealed potential targets that might be effective amplifiers of the anti-tumor response to overcome resistance after immunotherapy.

#### P655 EOS100850, an A2A receptor antagonist demonstrates efficacy in murine syngeneic tumor models regardless of adenosine concentrations

##### Romain Pirson, MSc, Anne Catherine Michaux, Diane Jamart, Julie Preillon, MSc, Lucas Chaible, Chiara Martinoli, PhD, Gregory Driessens, PhD , Erica Houthuys, PhD, Stefano Crosignani, PhD, Reece Marillier, PhD

###### iTeos Therapeutics, Gosselies, Brussels, Belgium

####### **Correspondence:** Gregory Driessens (gregory.driessens@iteostherapeutics.com)

**Background**

Extracellular adenosine, acting predominantly through the A2A receptor (A2AR), mediates immunosuppression which includes suppression of Th1 responses and cell-mediated cytotoxicity as well as increasing the activity of Tregs and MDSC. We have developed EOS100850/1, a novel, non-brain penetrant and highly selective inhibitor of A2AR with sub-nanomolar Ki. In vivo, EOS100850/1 significantly delays tumor growth in different syngeneic models when combined with aCTLA-4 or aPD-L1. In vitro, we have demonstrated that EOS100850/1 maintains its potency in high adenosine environment (>10uM) using human PDX models. However, these PDX were engrafted on to immunodeficient mice and therefore any possible contribution of the immune infiltrating cells for generation of adenosine was not taken into account. Thus, the first aim of this study was to determine the adenosine levels in immunocompetent tumor models. The second aim was to confirm that EOS100850/1 can mediate antitumor efficacy in vivo regardless of the ambient adenosine concentration in the tumor microenvironment (TME).

**Methods**

MCA205, CT26, A20, and EMT6 tumor cells were subcutaneously implanted into immunocompetent syngeneic mice. When tumors reached 100-200mm3 and again at 600-700mm3 tumor extracellular fluid was collected by microdialysis and the adenosine concentration was measured by LC/MS. In a second study, the antitumor effect of EOS100850/1 administered orally at 0.6mg/kg QD was evaluated in models with different level of adenosine concentration (MCA205 and CT26) in combination with oxaliplatin or doxorubicin dosed at 10 or 5 mg/kg, respectively.

**Results**

The adenosine concentrations in individual tumors ranged from 2 to 50uM across different models, while adenosine levels in the non-tumor region were always less than 1uM. For most tumors, size did not affect the concentration of adenosine, except for CT26, where there was a 2 fold increase when tumors grew from 100-200mm3 to 600-700mm3. MCA205 tumors displayed the highest adenosine concentration with an average of 33uM while CT26 was considered as intermediate with an average of 6uM. EOS100850/1, in combination with chemotherapy, demonstrated similar tumor growth inhibition in both models, regardless of the concentration of adenosine.

**Conclusions**

These data demonstrate that the adenosine concentrations vary in the tumor microenvironment of different syngeneic tumor models and that EOS100850/1 is able to inhibit the A2AR induced immunosuppression and therefore tumor growth in combination with chemotherapy regardless of the adenosine concentration in the TME.

**Acknowledgements**

We would like to thank the CRO Explicyte for performing the microdialysis experiments.

**Ethics Approval**

The study was approved by La S.A. Biopole ULB Charleroi, approval number BUC 2016-02

#### P656 PEGylated cationic liposomal-oxaliplatin display impressive anti-cancer efficacy by dual-targeting of immunosuppressive populations and cancer-cells

##### Lars Petersen, PhD^1^, Lars Ringgaard, PhD^1^, Fredrik Melander, PhD^1^, Ragnhild Østrem, PhD^1^, Kasper Kristensen, PhD^1^, Jonas Henriksen, PhD^1^, Dennis Elema, PhD^1^, Andreas Kjær, MD, PhD^2^, Thomas Andresen, PhD^1^

###### ^1^Technical University of Denmark, Kgs. Lyngby, Denmark; ^2^Rigshospitalet & University of Copenhagen, Copenhagen, Denmark

####### **Correspondence:** Thomas Andresen (tlan@dtu.dk)

**Background**

Liposomal drug delivery systems have historically focused on tumor accumulating properties. This is generally achieved by securing a long-circulating half-life of liposomes that then accumulate by the enhanced permeation and retention (EPR) effect. Several important therapeutic targets beyond the cancer cells have been identified in the tumor microenvironment. Pro-tumorigenic myeloid populations in the tumor microenvironment are potent suppressors of anti-cancer cytotoxic T-cells and their depletion improve therapeutic outcome [1,2]. Liposomal drug delivery systems that, not only induce immunogenic cell death of cancer cells but also eliminate potent immunosuppressive populations are therefore highly attractive.

**Methods**

Weakly cationic PEGylated Oxaliplatin (L-OHP) liposomes (R2) were formulated (POPC/cholesterol/DOTAP/chol-PEG/Chol, (52.5/36/7.5/4)) (size 120 nm, polydispersity 0.1, charge +10 mV). Stealth liposomes were formulated (DSPC/cholesterol/DSPE-PEG2000, (55/40/5)) (size 106, polydispersity 0.02, charge -10). Therapeutic efficacy was investigated in the syngeneic CT26 colorectal cancer cell-line and tumor model. In vivo therapeutic efficacy was performed and the tumor microenvironment was analyzed using flow cytometry. Liposome biodistribution and pharmacokinetics were investigated using rhodamine B labeled stealth and R2 liposomes. L-OHP levels in the tumors were measured using inductively coupled plasma mass spectrometry (ICPMS). Real-time in vivo biodistribution was evaluated by PET/CT using copper-64-radiolabeled stealth and R2 liposomes according to a previously described technology [3].

**Results**

The in vitro half-maximal inhibitory concentration (IC50) of CT26 cancer cells was comparable between R2 liposomes and free L-OHP (2.2 and 2.0 μM). Due to in vitro stability, IC50 value for stealth L-OHP liposomes could not be achieved even at the highest concentration evaluated. PET/CT scans and ICPMS demonstrated that the R2 liposomes had a shorter circulating half-life and significant lower tumor accumulation compared to stealth liposomes, but higher than free L-OHP. However, the R2 L-OHP liposomes displayed an impressive anti-cancer efficacy with >60% of treated CT26 tumor-bearing mice displaying a complete response (CR), while no CR’s were observed in the L-OHP stealth liposome treated group. Advanced flow cytometric analysis of the tumor microenvironment identified that both R2 and stealth liposomes accumulated in central immunosuppressive myeloid populations but only R2 L-OHP liposomes were able to eliminate these compared to stealth and free L-OHP.

**Conclusions**

Here we report the potent anti-cancer effect of weakly cationic PEGylated L-OHP liposomes that depletes suppressive populations and eradicates CT26 tumors. The data demonstrate how advanced drug delivery systems may provide important immune-modulating properties in addition to direct cancer-cell cytotoxicity.

**References**

1. Parker KH, Beury DW, Ostrand-Rosenberg S. Myeloid-Derived Suppressor Cells: Critical Cells Driving Immune Suppression in the Tumor Microenvironment. In: Advances in Cancer Research. Vol 128. NIH Public Access; 2015:95-139.

2. Iclozan C, Antonia S, Chiappori A, Chen D-T, Gabrilovich D. Therapeutic regulation of myeloid-derived suppressor cells and immune response to cancer vaccine in patients with extensive stage small cell lung cancer. Cancer Immunol Immunother. 2013;62(5):909-918.

3. Petersen AL, Binderup T, Rasmussen P, et al. 64Cu loaded liposomes as positron emission tomography imaging agents. Biomaterials. 2011;32(9):2334-2341.

**Ethics Approval**

The study has been approved by The Danish Animal Experiments Inspectorate.

#### P657 Selective T cells redirection proteins (STR) enhance the anti-tumor activity of checkpoint inhibitors (CPIs) and can lead to long-lasting immunity against the tumor

##### Meir Azulay, PhD

###### NeoTx LTD, Rehovot, Israel

####### **Correspondence:** Meir Azulay (meir@neotx.com)

**Background**

Tumor-targeted superantigens (TTS) such as Naptumomab Estafenatox (Nap) are fusion proteins that consist of genetically engineered Superantigens (Sag) linked to Fragment antigen binding (Fab) moieties directed to tumor-associated antigens. Unlike CD3-based T cell redirection approaches (e.g. BiTEs) which bind and activate all T cells, TTS only bind and activate subsets of T cells that contain certain TCR β variable (TRBV) regions, e.g. TRBV 7-9 [1] and are thus defined as STR. We previously reported the synergistic anti-tumor effect of combining CPI with our lead STR compound, Nap (5T4 targeted Sag) or its murine surrogate protein [2]. Here, we present new pre-clinical data showing that STR not only enhances the anti-tumor effect of CPIs, but also stimulates the overall immune response that could lead to long term immunity against the tumor.

**Methods**

The combination of Nap with PD-L1 inhibitor (durvalumab) was tested in vitro against high (MDA-MB 231) and low (RKO) 5T4-expressing cancer cell lines in the presence of human PBMCs. For the in vivo studies, mice bearing EpCAM transfected MC38 tumors were treated with TTS (consisting of a Fab against EpCAM), an anti-PD-1 antibody, or the combination. Tumor growth and survival were assessed and tumor recurrence following re-challenge was evaluated.

**Results**

Combination of Nap with durvalumab had synergistic anti-tumor effect against both high and low 5T4-expressing cancer cell lines. Concomitant treatment of MC38-EpCAM tumor bearing mice with TTS and anti-PD-1 achieved complete tumor rejection in 4 of 10 mice and significantly prolonged survival and delayed outgrowth of tumors compared to the monotherapies. All cured mice rejected re-challenge with MC38-EpCAM and parental MC38 tumors, indicating long-term memory responses.

**Conclusions**

Our studies show that combination of CPI with STR overcomes the limited effect of CPI monotherapy regardless of tumor antigen expression level. In addition, our in vivo studies demonstrate that the combination of STR with CPI may lead to long term durable responses not possible in most patients receiving single agent CPI therapy. Moreover, the ability of these “cured mice” to reject tumor re-challenge suggests that STRs cause release of secondary antigens that prime subsequent immune responses. Taken together, our data suggests that combining anti-PD(L)1 with STR may be a promising therapeutic strategy for patients with solid tumors. Clinical phase 1/2 trial was recently initiated investigating the combination of Nap with durvalumab in subjects with selected advanced or metastatic solid tumors [NCT03983954].

**Trial Registration**

NCT03983954

**References**

1. Hedlund G, Eriksson H, Sundstedt A, et. al. The Tumor Targeted Superantigen ABR-217620 Selectively Engages TRBV7-9 and Exploits TCR-pMHC Affinity Mimicry in Mediating T Cell Cytotoxicity. PLoS One. 2013; 8(10): e79082

2. Azulay M, Lifshits S, Friedmann A, et al. Naptumomab Estafenatox induces T cell recognition, turning anti-PD-1 unresponsive "cold" tumors into "hot" responsive tumors. Cancer Research. Jul 2018, 78 (13 Supplement) abstract # 2712 AACR Annual Meeting 2018; Chicago, IL; DOI: 10.1158/1538-7445.AM2018-2712

**Ethics Approval**

The study was approved by of the Institutional Animal Care and Use Committee (IACUC) of Tel Aviv University, approval number 01-18-008

#### P658 Co-delivery of synergistic immune agonists via systemic nanoparticles for interferon β-driven anti-tumor immunity and prevention of metastasis

##### Prabhani Atukorale, Chris Hoimes, MD, Efstathios Karathanasis

###### Case Western Reserve University, Westlake, OH, United States

####### **Correspondence:** Efstathios Karathanasis (stathis@case.edu)

**Background**

The generation of a robust antigen-presenting cell (APC) response at the site of a tumor is central to effective cancer immunotherapy. Systemic administration enables widespread access to the microvasculature of a primary tumor and metastases. We encapsulated two powerful synergistic immune agonists within a single ~50-nm liposomal immuno-nanoparticle (immuno-NP) and exploited systemic delivery to achieve efficient deposition within the APC-rich tumor/metastases perivasculature. We elected to co-deliver an agonist of the stimulator of interferon genes (STING) pathway and a Toll-like receptor 4 (TLR4) agonist on the same immuno-NP to mediate their co-uptake by the same APCs to ensure functional synergistic production of Type I inteferon β (IFNβ) by these target cells. Here, we investigated the treatment efficacy of immuno-NPs in reducing primary tumor burden and preventing metastasis in an orthotopic murine model of triple-negative breast cancer (TNBC) and established the mechanistic basis for this anti-tumor immunity.

**Methods**

Female BalbC mice were inoculated orthotopically in the mammary fat pad with 5x10^5 4T1 TNBC cells. Tumor burden was monitored longitudinally by bioluminescence measurements of luciferase-expressing tumor cells and physical caliper measurements. Cytokines, immune cell populations, and immuno-NP deposition was measured by ELISA, flow cytometry, and confocal microscopy, respectively. One-/two-way ANOVA and Student’s t-test were used for statistical analysis.

**Results**

Immuno-NPs carrying both STING/TLR4 agonists mediated a robust synergistic production of IFNβ from macrophages that was 10-fold increased from NPs carrying either agonist alone (**Fig.** 1a, with transmission electron micrograph of immuno-NPs shown in inset). Tumor-bearing mice treated with immuno-NPs had elevated levels of tumor dendritic cells (DCs), macrophages, and natural killer (NK) cells, compared to controls that included mice treated with clinically-approved immune checkpoint inhibitors anti-PD1 and anti-CTLA4 (**Fig.** 1b). Primary tumor sizes were 50-60% reduced in mice treated with immuno-NPs compared to controls (**Fig.** 1c) and metastasis was prevented (**Fig.** 1d). Significantly, treatment with immuno-NPs outperformed treatment with immune checkpoint inhibitors.

**Conclusions**

Nanoparticle-mediated co-delivery of synergistic STING/TLR4 agonists drives a significant therapeutic outcome in the treatment of TNBC and prevention of metastasis in the 4T1 orthotopic model. Systemic delivery enables accumulation of immuno-NPs within the APC-rich perivasculature of the tumor/metastases, which is pivotal for the mediation of a significant anti-tumor immune response.

**Acknowledgements**

This work partially supported by grants from the National Cancer Institute (R01CA177716, U01CA198892), Alex’s Lemonade Stand Foundation, and the Angie Fowler AYA Cancer Research Initiative.

**Fig. 1 (abstract P658).**
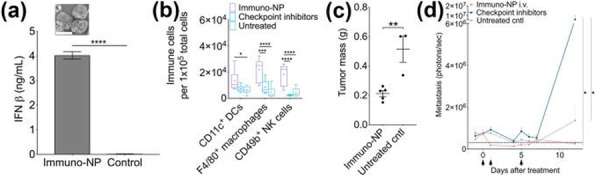
See text for description

#### P659 ENPP1 antagonists in combination with radiation or checkpoint inhibitors demonstrate antitumor activity in syngeneic mice models of pancreatic adenocarcinoma, neuroblastoma, TNBC, and colon cancer

##### Lingyin Li, PhD^1^, Mark Smith, PhD^1^, Betty Chang, PhD^2^

###### ^1^Stanford University, Stanford, CA, United States ; ^*2*^*Angarus Therapeutics, Cupertino, CA, United States*

####### **Correspondence:** Betty Chang (bchang@angarustx.com)

**Background**

Ectonucleotide pyrophosphatase 1 (ENPP1) was identified as the extracellular hydrolase for cGAMP, the natural ligand for STING [1,2]. Multiple lines of evidence suggest cancer cells produce soluble extracellular cGAMP, resulting in activation of CD14+ PBMCs, and subsequent production of IFNb in cell culture. Inhibition of ENPP1 and ionizing radiation further increase extracellular cGAMP concentrations and anti-cancer immunity in mice. Genetic knockout of ENPP1, in breast cancer cells or in the host, delayed tumor progression, demonstrating antitumor activity of ENPP1 deficiency. ENPP1 downregulation correlated with survival advantage in breast cancer patients. Here, systemic delivery of extracellular ENPP1 antagonists were evaluated in syngeneic mice tumor models alone and in combination with ionizing radiation and/or immune checkpoint inhibitors.

**Methods**

Novel SMI to ENPP1 were synthesized and characterized. ANG-1084 and ANG-1623, were identified as leads, and tested for potency, selectivity, stability, safety and PK. ANG-1623 was delivered at 50 mg/kg/d systemically for 7-28 days in models of pancreatic adenocarcinoma, neuroblastoma, orthotopic TNBC, and colon carcinoma.

**Results**

ANG-1084 and 1623 have IC50 of <0.5 nM against human ENPP1 with cGAMP as substrate at pH7.6, and are selective against hENPP2/3, PDEs, CEREP44 and stable in human and mouse microsomes. ANG-1623 had no hits in a 468-kinase-screen at 1 uM, and its protein binding was 66% and 55%, in human and mice, respectively. ANG-1084 and 1623 did not have CYP liability and hERG IC50 was >25 uM. In addition, both were superior in potency, selectivity, and hERG to the published ENPP1i QS-1. ANG-1623 at 50 mg/kg/day had single agent activity in the Panc02 model, with 63% TGI (p<0.001) and 81% TGI when combined with radiation (p<0.001, 5/15 tumor free). ANG-1623 in combination with radiation and/or checkpoint inhibitors had significant anti-tumor effects in Neuro-2a, orthotopic 4T1 or MC38 models.

**Conclusions**

Novel selective and potent small molecule inhibitors were designed to inhibit extracellular ENPP1. Lead molecule ANG-1623 has desirable properties of a drug and was subsequently tested in syngeneic mice tumor models: 4T1, Panc02, Neuro-2a, and MC38, where it was administered subcutaneously for 7 to 28 days. The drug was well tolerated and demonstrated significant anti-tumor activity alone or in combination with radiotherapy and/or anti-PD-L1. Since ENPP1 antagonists aim to preserve extracellular cGAMP produced by tumors either naturally or induced by ionizing radiation, and are delivered systemically, they may be promising alternatives to synthetic CDNs that are restricted to intratumoral injections of surface tumors, and pose challenges in optimal dose determinations.

**References**

1.Li, L et al., Hydrolysis of 2’3’-cGAMP by ENPP1 and design of nonhydrolyzable analogs. Nat Chem Biol 2014;10(12)1043-8.

2.Carozza, JA et al., 2’3’-cGAMP is an immunotransmitter produced by cancer cells and hydrolyzed by ENPP1. 2019; bioRxiv539312 doi: https://doi.org/10.1101/539312

#### P660 TNF-alpha suppresses the immunogenicity of STING-agonists

##### Anthony Desbien, PhD^1^ , Kelsey Sivick Gauthier^2^, Ed Lemmens^1^, Weiqun Liu, PhD^1^, Brian Francica, PhD^3^, Gabrielle Reiner^4^, Charles Cho^5^, Steven Bender^5^, Jeffry Mckenna, DPhil^6^, Yan Feng, PhD^6^, Lianzing Zheng, Phd^6^, Andrea van Elsas^1^, Sarah McWhirter^1^

###### ^*1*^*Aduro Biotech, Berkeley CA, United States;*^*2*^*Arcus Biosciences, Hayward, CA, United States* ; ^*3*^*Tempest Therapeutics, San Francisco, CA, United States* ; ^*4*^*University of California-Berkeley, Berkeley, CA, United States* ; ^*5*^*Genomics Institute Novartis, San Diego, CA, United States* ; ^*6*^*Novartis Institutes for BioMedical Res., Cambridge, MA, United States*

####### **Correspondence:** Anthony Desbien (TDESBIEN@ADURO.COM)

**Background**

The stimulator of interferon genes (STING)-pathway is a critical component of the adaptive immune response against tumors. Aduro Biotech is developing the synthetic cyclic dinucleotide (CDN) STING agonist, ADU-S100 (MIW815), as a cancer treatment.

**Methods**

A dose-response analysis of intratumorally administered STING agonists in syngeneic mouse tumor models was performed to determine immunogenicity and tumor control.

**Results**

Data show that low to mid-range doses caused injected tumor collapse and the activation and expansion of tumor-specific CD8 T cells. In contrast, overstimulation of the STING pathway with high-doses of ADU-S100 or more potent STING agonists failed to induce tumor-specific CD8 T cells and caused disruption of tumor draining lymph nodes. Blocking tumor necrosis factor-alpha (TNF-alpha) signaling restored induction of tumor-specific CD8 T cells during overstimulation of STING and preserved lymph node integrity.

**Conclusions**

These data demonstrate that the induction of anti-tumor CD8 T cells is inhibited by TNF-alpha during overstimulation of the STING pathway, suggesting that intervention in TNF-alpha signaling may broaden the therapeutic window of STING agonists.

**Ethics Approval**

All animals were used according to protocols approved by Institutional Animal Use Committee of

Aduro Biotech.

#### P661 Development and characterization of next generation small molecule STING agonists

##### Stefan Chmielewski, PhD, Magdalena Zawadzka, Jolanta Mazurek, Karolina Gluza, Katarzyna Wójcik-Jaszczyńska, Maciej Kujawa, Grzegorz Topolnicki, Grzegorz Ćwiertnia, Aleksandra Poczkaj, Eliza Zimoląg, Magdalena Mroczkowska, Agnieszka Gibas, Marcin Leś, Sylwia Sudoł, Marek Wronowski, Kinga Michalik, Katarzyna Banaszak, Katarzyna Wiklik, Federico Malusa, Michał Combik, Karolina Wiatrowska, Łukasz Dudek, Jose Alvarez, Anna Rajda, Maciej Rogacki, Faustyna Gajdosz, Aniela Gołas, Katarzyna Wnuk-Lipińska, Ewelina Gabor-Worwa, Charles Fabritius, Luigi Stasi, Peter Littlewood, Krzysztof Brzózka, Monika Dobrzańska

###### Selvita, Krakow, Poland

####### **Correspondence:** Monika Dobrzańska (monika.dobrzanska@selvita.com)

**Background**

Stimulator of Interferon Genes (STING) is a major player in the initiation of robust innate immune activation leading to initiation and enhancement of a tumor-specific adaptive immunity. Several clinical and pre-clinical studies employ modified cyclic dinucleotides, natural STING ligands. Yet, relative instability and chemical nature limit their use as systemic immuno-therapeutics. Herein, we present potent and selective non-nucleotide, non-macrocyclic, small molecule direct STING agonists, structurally unrelated to known chemotypes with potential for systemic administration.

**Methods**

Binding to recombinant STING protein was examined using FTS, MST, FP and crystallography studies. Phenotypic screen was performed in THP-1 Dual reporter cells. Human macrophages (HMDM) and dendritic cells (HMDC) were differentiated from monocytes (obtained from PBMC) in the presence of GM-CSF and GM-CSF/IL-4 for HMDM and HMDC, respectively. Human T cells were isolated from PBMC, activated with plate-bound anti-CD3/anti-CD28 and exposed to compounds followed by viability and proliferation assessment using flow cytometry. Mouse bone marrow-derived dendritic cells (BMDC) were obtained from C57BL/6 or STING Gt mice and differentiated with mIL-4 and mGM-CSF. Cell maturation marker expression and the presence of phosphorylated forms of the STING pathway proteins were assessed by flow cytometry and Western Blotting. BALB/c mice were injected with compounds and the cytokine release was measured in the plasma. Additionally, mice were inoculated with CT26 cells and the compound was administered followed by the regular tumor growth monitoring.

**Results**

Selvita’s agonists bind to all tested recombinant STING proteins: human, mouse, rat and monkey. Selvita’s compounds induce cytokine responses and upregulate DC maturation markers on BMDC in low nM range only in the presence of STING, confirming their high selectivity. They trigger pro-inflammatory cytokine release from human PBMC and HMDC and induce dendritic cell maturation regardless of the STING haplotype. In vitro repolarization of immuno-suppressive M2d macrophages into pro-inflammatory M1-like phenotype was demonstrated. The compounds have fine-tunable ADME properties with particularly good solubility, permeability, human plasma stability and plasma protein binding. Systemic in vivo administration led to robust upregulation of STING-dependent pro-inflammatory cytokines suggesting immune activation which translated into efficacy in vivo in subcutaneous CT26 model.

**Conclusions**

Selvita’s STING agonists activate STING-dependent signaling in both mouse and human immune cells promoting anti-tumor immunity. Treatment with Selvita’s STING agonists led to tumor growth inhibition by engagement of the immune cells. The compounds show good selectivity and in vitro ADME properties enabling development for systemic administration as a single agent or in combinations with immunotherapies or targeted agents.

#### P662 Inhibiting DNA methylation can reverse epigenetic silencing of STING in melanoma and enhance antitumor T-cell activity

##### Rana Falahat, PhD^1^, James Mulé, PhD^1^, Anders Berglund, PhD^1^, Patricio Perez-Villarroel^1^, Shota Aoyama, MD^1^, Shari Pilon-Thomas, PhD^1^, Glen Barber, PhD^2^

###### ^1^Moffitt Cancer Center, Tampa, FL, United States; ^*2*^*University of Miami Miller School of Medicine*

####### **Correspondence:** James Mulé (james.mule@moffitt.org)

**Background**

STING pathway is a major innate immune sensing mechanism for the detection of immunogenic tumors. We have recently shown that STING signaling is heterogeneously regulated across human melanoma cell lines. While intact activation of STING signaling in a subset of melanoma cell lines enhances their antigenicity, defects in STING pathway limit their sensitivity to lysis by human melanoma tumor infiltrating lymphocytes (TIL) [1]. In addition, we have reported a high incidence of STING gene methylation in a variety of tumors including melanoma [2]. Based on these findings, we hypothesized that reconstitution of STING expression through DNA demethylation would rescue STING signaling in STING-defective melanoma cell lines and could therefore promote antitumor T-cell activity.

**Methods**

Using methylation microarray analysis, we identified human melanoma cell lines with aberrant STING promoter methylation across a panel of 16 cell lines and subjected them to treatment with the DNA methyltransferase inhibitor 5-aza-2’-deoxycytidine (5AZADC). We next evaluated the induction of STING expression by immunoblot. We assessed phosphorylation of IRF3 and induction of IFN-beta and CXCL10 in 5AZADC-treated melanoma cells following their stimulation with the STING agonist 2’3’-cGAMP. We also co-cultured 5AZADC-treated melanoma cell lines with their HLA-matched human melanoma TIL in the presence or absence of 2’3’-cGAMP and assessed TIL production of IFN-γ.

**Results**

We identified 6 STING-negative human melanoma cell lines with high levels of STING promoter methylation. Immunoblot analysis revealed inhibiting DNA methylation by 5AZADC treatment can partially or completely restore STING expression in all 6 cell lines. Stimulation with 2’3’-cGAMP resulted in phosphorylation of IRF3 and induction of CXCL10 (~1200 pg/ml, p < 0.01) and IFN-beta (~900 pg/ml, p < 0.0001) in 3 of 6 5AZADC-treated cell lines. In co-culture studies 5AZADC-treated melanoma cells stimulated up to a 4-fold increase in TIL production of IFN-γ compared to untreated controls in the presence of 2’3’-cGAMP (510 to 2100 pg/ml, p < 0.05).

**Conclusions**

We provide evidence that DNA methylation is a major contributor to the suppression of STING signaling in melanoma. Reconstitution of STING expression through DNA demethylation can restore STING signaling in STING-defective melanoma cell lines and promote antitumor T-cell activity. Collectively, these observations argue that targeting epigenetic loss of STING in melanomas should be considered as a strategy to improve the efficacy of clinical interventions using STING agonists.

**Acknowledgements**

NCI P50 CA168536, Cindy and Jon Gruden Fund, Chris Sullivan Fund, V Foundation, Dr. Miriam and Sheldon G. Adelson Medical Research Foundation.

**References**

1. Falahat R, et al. Tumor cell-intrinsic STING signaling impacts antigenicity of melanoma and can promote antitumor T-cell activity. Cancer Immunology Research, 2019.

2. Konno H, et al. Suppression of STING signaling through epigenetic silencing and missense mutation impedes DNA damage mediated cytokine production. Oncogene, 2018; 37(15): 2037.

#### P663 Evaluating loss of cGAS and STING expression using a novel multiplex immunohistochemistry detection approach

##### Jeanette Rheinhardt^1^, Adam Markman^1^, Hideki Goda, PhD^2^, Etsuko Futaya^2^, Masaru Takahashi^2^, Hiroyuki Yokota^2^, Kenji Nishikawa^2^, Hisatake Okada^2^, Kenneth Bloom^1^, George Abe^1^, Joseph Krueger^1^, Apollina Goel, PhD^1^

###### ^1^Invicro, LLC, Boston, MA, United States; ^*2*^*Konica Minolta Inc. Bio-Healthcare unit, Tokyo, Japan*

####### **Correspondence:** Joseph Krueger (jkrueger@invicro.com)

**Background**

The cGAS-STING (cyclic GMP-AMP synthase-stimulator of interferon genes) pathway is becoming an important point of focus in the expanding landscape of next-generation immunotherapies. STING agonists can induce systemic anti-tumor immunity and thus may be also synergistic with other immunotherapies (i.e. immune checkpoint blockade, cancer vaccines, and CAR-T cell therapy). Importantly, a functional cGAS-STING pathway may be essential for response to oncolytic viral therapy. However, advanced stages of solid cancers (i.e. melanoma and colon adenocarcinoma) frequently show loss of STING or cGAS and would not be expected to respond to STING agonists. Since STING agonists have now entered clinical trials, there is an emerging need for predicting patient response which can accurately classify patients according to STING or cGAS expression. Here, we show application of a novel immunohistochemistry (IHC)-based approach using fluorescent nanoparticles (Quanticell™) to deliver highly sensitive and quantitative analysis of STING and cGAS within the tumor contexture.

**Methods**

cGAS and STING expression were simultaneously detected in melanoma tissues using our novel fluorescent nanoparticle IHC approach (Quanticell™). Serial sections were stained with chromogenic IHC to correlate between the expression of cGAS/STING and the patterns of tumor-infiltrating immune cells.

**Results**

Our results show that the Quanticell assay format enables a more objective and quantitative scoring of target proteins compared to standard IHC assay formats. The ability to multiplex cGAS and STING enables simultaneous quantitative analysis of these two critical markers to characterize distinct patient phenotypes with tumors associated with diverse immune contexture.

**Conclusions**

We show that our technique quantitatively measures cGAS and STING expression in cancer tissue biopsies with high sensitivity. The multiplexed IHC format has immediate value for predictive and prognostic application in STING agonist-based single/checkpoint inhibitor combination therapies.

**Acknowledgements**

Hemi Dimant, Ankit Gandhi, Stephan Collins, Mark Lawson.

#### P664 Development of small molecule STING agonists that can evoke host anti-cancer immune defences following local or systemic delivery

##### Nicole Haynes, PhD^1^, Theresa Connor^1^, Ben Morrow, PhD^2^, Judy Doherty^1^, Matthew Dennis, PhD^3^, Jonathan Hubert, PhD^2^, Sukhdeep Spall^4^, Anthony Cuzzupe^5^, Karen White, PhD^6^, Susan Charman, PhD, Professor^6^, Aaron Lock^7^, Vicky Avery, PhD, Professor^7^, Stewart Nuttall^3^, Tom Peat, PhD^3^, Olan Dolezal, PhD^3^, Pat Pilling^3^, Jezrael Revalde^8^, Tin Yow^8^, Greg Arndt^8^, Hendrik Falk, PhD^4^, Mark Devlin, PhD, COO^1^, Ian Street, PhD^4^, Poh Khoo, PhD^8^, Paul Stupple, PhD^2^, Brendon Monahan, PhD, CSO^1^

###### ^*1*^*Cancer Therapeutics CRC, Peter MacCallum Cancer Centre, Melbourne, Australia;*^*2*^*Cancer Therapeutics CRC, Monash University of Pharmaceutical Sciences, Melbourne, Australia* ; ^*3*^*Cancer Therapeutics CRC, CSIRO, Melbourne, Australia* ; ^*4*^*Cancer Therapeutics CRC, Walter and Elisa Hall Institute, Melbourne, Australia* ; ^*5*^*SYNthesis Research, Melbourne, Australia* ; ^*6*^*Monash University of Pharmaceutical Sciences, Melbourne, Australia* ; ^*7*^*Cancer Therapeutics CRC, Griffith Institute for Drug Discovery, Melbourne, Australia* ; ^*8*^*Cancer Therapeutics CRC, Children’s Cancer Institute, Melbourne, Australia*

####### **Correspondence:** Brendon Monahan (brendon@cancercrc.com)

**Background**

STING is a ubiquitously expressed innate immune sensor which is essential for the production of host defence-related proteins including type I interferons and proinflammatory cytokines that promote the recruitment and effector activity of innate and adaptive immune cells [1]. Targeted activation of STING and the associated release of type I interferons can enhance the sensitivity of antigen presenting cells to neoantigens and promote their ability to prime T cell-based anti-cancer immune responses [1,2]. This can lead to the establishment of T cell inflamed tumor microenvironments that are more susceptible to the anti-tumor effects of checkpoint blockade therapy [3]. The Cancer Therapeutics CRC (CTx) is developing direct small molecule STING agonists that can be administered systemically as an adjunct to immune checkpoint antibody therapy.

**Methods**

A phenotypic screen was run using an IRF3-luciferase reporter cell line looking for activation of Type I Interferon genes when screened against CTx small molecule libraries. Validated hits were deconvoluted by STING SPR to confirm direct STING binding. A mature medicinal chemistry program is underway with an established screening cascade including in vitro target engagement and primary cell functional assays, structural biology, in vivo PK/PD, and efficacy assays to determine mechanism(s) of action and demonstrate therapeutic index.

**Results**

CTx has developed potent drug-like small molecule STING agonist compounds that show direct STING binding. Cellular activity is dependent upon STING (inactive in STING-/- cells), independent of cGAS (active in cGAS-/- cells), and can be blocked by TBK1 inhibitors. Compounds show activity in primary mouse and human cells across a range of STING variants. Dose-dependent in vivo PD biomarker responses, including increased plasma IFN-β levels, were seen when administered intravenously (I.V) or intratumorally (I.T). In vivo, single agent efficacy of CTx STING agonists has been tested in multiple syngeneic mouse models of solid cancer. Treatment via I.T or I.V administration was well tolerated and evoked therapeutically beneficial immune-mediated anti-tumor responses. Successful induction of immunological memory, in response to STING agonist treatment was demonstrated in tumor-rechallenge experiments. In less responsive models, where intermediate efficacy was observed, increased tumor growth control could be achieved by co-treating mice with our STING agonist and anti-PD-1 antibody therapy.

**Conclusions**

CTx has a mature STING agonist program that has successfully produced direct STING activators that can promote primary DC function and elicit immune-mediated anti-tumor responses in mouse models of solid cancer.

**References**

1. Bose, D. cGAS/STING Pathway in Cancer: Jekyll and Hyde Story of Cancer Immune Response. Int J Mol Sci. 2017. 18:2456.

2. Vanpouille-Box, C. et al. Cytosolic DNA Sensing in Organismal Tumor Control. Cancer Cell. 2018. 34:361-378.

3. Woo, S.R., Corrales, L. & Gajewski, T.F. The STING pathway and the T cell-inflamed tumor microenvironment. Trends Immunol. 2015. 36:250-256.

#### P665 Enhancing IFN-β production through synergy between the STING and TLR pathways

##### Emily Higgs, BA, Thomas Gajewski, MD, PhD

###### University of Chicago, Chicago, IL, United States

####### **Correspondence:** Thomas Gajewski (tgajewsk@medicine.bsd.uchicago.edu)

**Background**

Previous work from our laboratory has shown that tumors activate the STING pathway in antigen-presenting cells. STING signaling leads to IFN-β production which is required for T cell priming through dendritic cells. While the best defined transcription factor for IFN-β gene expression is IRF3, other transcriptional regulators also contribute, including NF-κB. Enhancing STING activation and downstream IFN-β production has therapeutic potential, and STING agonists are currently being evaluated in clinical trials. However, innate immune activation in response to a pathogen rarely occurs by activating only one signaling pathway, and targeting multiple pathways may lead to more robust activation. Activating multiple innate immune pathways similar to a natural infection could improve the therapeutic efficacy of STING agonists.

**Methods**

In vitro experiments were performed using macrophages and bone marrow-derived dendritic cells stimulated with the STING agonist DMXAA alone or in combination with several TLR agonists. Cells were subjected to several downstream analyses, including qPCR, Western blotting, and immunofluorescent imaging to visualize transcription factor localization.

**Results**

Each innate immune agonist was titrated to identify the dose inducing maximal IFN-β expression. DMXAA induced greater peak IFN-β expression than any individual TLR agonist. After testing several combinations, we found that LPS + DMXAA induced significantly greater IFN-β transcription than the sum of either agonist alone. To explain this synergy, we assayed each step of STING pathway signaling. Initial activation leads to STING aggregation, and LPS did not increase STING aggregation beyond that induced by DMXAA. Additionally, LPS did not increase IRF3 phosphorylation nor IRF3 nuclear translocation in combination with DMXAA. Rather, LPS increased the phosphorylation and nuclear translocation of the NF-κB subunit p65. These results suggest that the synergy in IFN-β production is achieved through IRF3 activation downstream of STING signaling and NF-κB activation downstream of TLR4 signaling. Additional mechanistic experiments are ongoing.

**Conclusions**

TLR activation by LPS synergized with STING signaling to augment IFN-β production beyond the level of either agonist alone. Mechanistically, STING pathway activation preferentially signals through IRF3 whereas TLR4 pathway activation preferentially signals through NF-κB. Combining activation of these pathways could improve the innate immune response in a way that is therapeutically beneficial.

#### P666 Selective activation of antigen presenting cells by exoSTING enhances tumor antigen-specific immune response

##### Su Chul Jang, PhD, Kelvin Zhang, Nuruddeen Lewis, PhD, Tong Zi, Joanne Lim, Rane Harrison, Raymond Moniz, Katherine Kirwin, Chang Ling Sia, Christine McCoy, Kevin Dooley, Ke Xu, Jorge Sanchez-Salazar, Raymond Bourdeau, Agata Villiger-Oberbek, William Dahlberg, Shil Patel, Kyriakos Economides, Sriram Sathyanarayanan

###### Codiak Biosciences, Cambridge, MA, United States

####### **Correspondence:** Sriram Sathyanarayanan (sriram.sathy@codiakbio.com)

**Background**

Activation of the STING pathway by direct intra-tumoral administration of synthetic cyclic dinucleotide (CDN) agonists has shown anti-tumor activity in animal models and early stage human clinical trials. At higher doses CDNs induce local tissue damage due to non-selective uptake into cells at the injection site, including ablation of immune effector cells (CD8+ T cells and Antigen Presenting Cells [APC])[1]. CDN administration has been shown to result in a bell-shaped dose response curve, wherein anti-tumor effects are lost at high doses [1]. We have developed a novel engineered exosome therapeutic candidate, exoSTING™, which selectively activates the STING pathway in tumor-resident APCs demonstrating greater potency than free CDN and without immune ablation.

**Methods**

Exosomes were engineered to overexpress PTGFRN, an exosome surface protein, and loaded ex vivo with a proprietary CDN. In vivo pharmacodynamics (PD) and anti-tumor responses were demonstrated in a B16F10 syngeneic mouse model and ex vivo histo-culture of human tumor explants (head and neck squamous cell carcinoma [HNSCC] and non-small cell lung cancer [NSCLC] patients) was utilized to evaluate PD after intra-tumoral administration.

**Results**

ExoSTING™ was preferentially taken up by differentiated macrophages and dendritic cells. In vitro proliferation studies showed a 100-fold increase in potency of macrophage activation by exoSTING™ treatment compared to free CDN. In contrast, T-cells failed to take up exosomes and were not activated by exoSTING™. We have shown that intra-tumoral administration of exoSTING™ in B16F10 tumors preserves the viability of T-cells and APCs, reduces collateral tissue damage, and eventually recruits more T-cells into tumors, whereas free CDN induces immune cell ablation. NanoString gene expression analysis showed increased immune cell-related gene signatures in tumors after dosing with exoSTING™ but not free CDN exposed tumors. ExoSTING™ produced a systemic tumor-specific T-cell response as demonstrated by growth inhibition of non-injected distal tumors, which was further enhanced by combination with anti-PD1 checkpoint blockade. Activity of exoSTING™ was also examined in ex vivo human tumor histo-culture. ExoSTING™ induced IFNβ mRNA expression in a dose-dependent manner and induced more IFNβ, CXCL9, CXCL10, and IFNγ mRNA expression than equivalent amounts of free CDN.

**Conclusions**

ExoSTING™ is an engineered exosome therapeutic candidate that specifically targets the STING pathway in APCs in the tumor microenvironment, resulting in enhanced PD effects, avoidance of T cell uptake and enhanced anti-tumor immune responses compared to free CDN. Human clinical testing is planned to begin in the first half of 2020.

**References**

1. Sivick KE, Desbien AL, Glickman LH, et al. Magnitude of Therapeutic STING Activation Determines CD8+ T Cell-Mediated Anti-tumor Immunity. Cell Rep. 2018;25(11):3074-3085.

**Ethics Approval**

The study was approved by Codiak BioSciences' internal IACUC committee, approval number CB2017-001.

#### P667 Neoadjuvant STING agonists combined with systemic immunotherapy block metastatic recurrence in orthotopic breast tumors

##### Lauren Milling, BS, Darrell Irvine, PhD

###### Massachusetts Institute of Technology, Cambridge, MA, United States

####### **Correspondence:** Darrell Irvine (djirvine@mit.edu)

**Background**

Cyclic dinucleotides (CDN) – agonists of Stimulator of IFN genes – can initiate potent anti-tumor immunity due in part to activation of antigen presenting cells [1]. Recent studies have highlighted the role of CDN dose in governing the involvement of CD8+ T cells in anti-tumor immune response [2]. Intratumoral CDN co-therapy with systemic anti-PD-1 checkpoint blockade in bilateral 4T1 mammary carcinoma flank tumors results in regression of injected and distal tumors [2]. However, dual flank models largely ignore the role of the orthotopic tumor microenvironment, the natural process of metastatic development, and the impact of surgical resection – a predominant intervention in breast cancer [3].

**Methods**

We employed a surgical resection model of metastatic 4T1 mammary carcinoma to examine the effects of surgery and lung metastasis on CDN efficacy. 4T1-luciferase cells were inoculated in the mammary fat pad on Day 0, palpable tumors were then treated with immunotherapy starting on Day 6, any remaining primary tumor was surgically resected on Day 15, and mice were monitored for metastases by luciferase imaging. Intratumoral bisphosphorotioate 2’3’ c-di-AMP (CDN) was combined with systemic administration of mouse serum albumin IL-2 fusion protein (MSA-IL2) and anti-PD-1 followed by primary tumor growth measurements and monitoring for overall survival. AH1 tetramer staining of PBMC was utilized to monitor antigen-specific CD8+ T cells.

**Results**

In mice bearing established 4T1-luciferase tumors, administration of three CDN doses results in no cures if surgical resection is not performed. When administered prior to surgical resection CDN monotherapy yields a 20% cure rate and enhanced median overall survival compared to untreated mice (median survival 44.5 days vs 38 days, p=0.0026). Combination of CDN with MSA-IL2 and anti-PD-1 further improves survival, with 60% of mice surviving long-term. Using a tdTomato-expressing tumor line we find increased numbers of tdTomato+ CD103+ DCs in the tumor draining lymph node of CDN + MSA-IL2 + anti-PD-1-treated mice compared to CDN alone (p=0.016), indicative of enhanced antigen uptake. After surgical resection, triple combination-treated mice displayed higher levels of AH1-specific CD8+ T cells in the blood compared to CDN or CDN + anti-PD-1 treated mice. Upon rechallenge, AH1-specific T cells remain elevated in the combination group; however, complete rejection was not observed.

**Conclusions**

Our findings suggest that CDN monotherapy is less effective in the recurrent, orthotopic model of 4T1 mammary carcinoma than in flank tumors, but combination with systemic immunotherapy can improve immune control of both primary and metastatic disease.

**Acknowledgements**

The research reported was supported by the Howard Hughes Medical Institute, the Marble Center for Cancer Nanomedicine, and the NIGMS/NIH Interdepartmental Biotechnology Training Program (Grant# T32-GM008334).

**References**

1. Corrales L et al. Direct activation of STING in the tumor microenvironment leads to potent and systemic tumor regression and immunity. Cell Reports. 2015; 11:1018-1030.

2. Sivick K et al. Magnitude of therapeutic STING activation determines CD8+ T cell-Mediated anti-tumor immunity. Cell Reports. 2018; 25:3074-3085.

3. Al-Sahaf O et al. Surgical injury enhances the expression of genes that mediate breast cancer metastasis to the lung. Ann Surg. 2010; 252:1037-1043.

**Ethics Approval**

All mouse experiments were approved by MIT’s Committee on Animal Care, protocol# 0717-076-20.

#### P668 TTI-10001, a next generation small molecule STING agonist, demonstrates potent anti-tumor activity in mice following oral or intravenous administration

##### Natasja Nielsen Viller, PhD^1^, Peter Dove^2^, David Rosa^2^, Bolette Bossen, BSc^2^, Tran Truong^2^, Tapfuma Mutukura^2^, Debbie Jin^2^, Marilyse Charbonneau^2^, Laura Brinen^2^, Karen Dodge^2^, Gloria Lin^2^, Jeff Winston^2^, Robert Uger^2^, Malik Slassi^2^, Zezhou Wang^2^

###### ^1^Trillium Therapeutics, Mississauga, Ontario, Canada; ^2^Trillium Therapeutics Inc., Mississauga, Canada

####### **Correspondence:** Zezhou Wang (joe@trilliumtherapeutics.com)

**Background**

Stimulator of interferon genes (STING) is a master regulator of interferon (IFN)-mediated immune responses, and the cGAS-STING pathway plays an essential role in detection of aberrant cytosolic DNA fragments. The cascade of type I IFNs and pro-inflammatory cytokines that are produced following STING activation serve to bridge the innate and adaptive immune systems, and trigger potent anti-tumor immunity. STING is thus a promising therapeutic target in cancer and several STING agonists are currently in clinical development, though most are suitable only for intratumoral (i.t.) rather than systemic administration. We have previously shown that TTI-10001, a novel non-cyclic dinucleotide (CDN) small molecule STING agonist, potently induces the STING pathway *in vitro* and results in robust anti-tumor activity *in vivo* following i.t. administration. Here we report that TTI-10001 is well tolerated and demonstrates potent anti-tumor monotherapy activity in mice after oral or intravenous (i.v.) dosing.

**Methods**

TTI-10001 was administrated as a single dose (oral, i.v., or i.t.) to MC38 tumor-bearing mice and plasma or tumor samples were collected for pharmacokinetic (PK)/pharmacodynamic (PD) analysis. Repeat administration (oral, i.v., or i.t.) of TTI-10001 was used for maximum tolerated dose (MTD) studies or efficacy studies in single or dual flank MC38 tumor-bearing mice. Off-target effects were assessed using the SafetyScreen44™ panel.

**Results**

Mice dosed with TTI-10001 demonstrated favorable drug exposure with excellent oral bioavailability (85%) and a long tumor retention time (T1/2: ~ 4-8h) following all three routes of administration. Treatment with TTI-10001 induced dose-dependent expression of pro-inflammatory cytokines in tumors including interferon beta, tumor necrosis factor α, and interleukin 6. No significant off-target effects were observed *in vitro*. TTI-10001 was well tolerated in mice with no weight loss or overt morbidity after repeated systemic dosing. Potent, dose-dependent anti-tumor efficacy in the MC38 syngeneic mouse tumor model was observed when TTI-10001 was administered at doses below the MTD using all three modes of delivery.

**Conclusions**

TTI-10001, a novel non-CDN small molecule STING agonist, is well tolerated *in vivo* and displays an excellent PK/PD profile that results in potent anti-tumor activity by i.t., oral, and i.v. routes of administration. These data highlight the potential of TTI-10001 to achieve best-in-class status among next generation STING agonists.

**Ethics Approval**

All mouse experiments were approved by the University of Toronto animal care committee in accordance with the regulations of the Canadian Council on Animal Care (University of Toronto approved protocol # 20011874).

#### P669 Pharmacological evaluation of the ubiquitin ligase CBL-B as a small molecule, tumor immunotherapy target

##### Jennifa Gosling, MS, Christoph Zapf, Ryan Rountree, Chenbo Wang, Thomas Cummins, Frederick Cohen, Hiroko Tanaka, Dahlia Weiss, Mario Cardozo, Christopher Karim, May Tan, Austin Tenn-McClellan, Szerenke Kiss von Soly, Julie Sheung, Ketki Dhamnaskar, Katherine Kurylo, Neil Bence, Arthur Sands

###### Nurix, San Francisco, CA, United States

####### **Correspondence:** Arthur Sands (asands@nurix-inc.com)

**Background**

E3 ubiquitin ligases play critical roles in directing cellular protein fate by controlling the specificity of ubiquitin conjugation to substrate proteins and targeting them for cellular localization or degradation by the ubiquitin proteasome system. The E3 ubiquitin ligase CBL-B is expressed in immune cell lineages and negatively regulates activity of the T-cell receptor (TCR) through substrate proteins that impose a requirement for a costimulatory signal to mount a productive immune response upon TCR engagement. Mice deficient in Cbl-b or lacking CBL-B ligase activity demonstrate a tumor rejection phenotype mediated by CD8+ T cells (1,2). CD4+ and CD8+ T cells from mice deficient in Cbl-b have 5 to 10-fold enhanced secretion of IL-2 and IFN γ when stimulated ex vivo. Cbl-b deficient mice also demonstrate enhanced NK cell function (3). These data provide a genetic rationale for the development of a small molecule inhibitor of CBL-B ligase activity for use in patients with tumor-mediated immune suppression of effector T cells.

**Methods**

We have identified a series of small molecule inhibitors of CBL-B activity with biochemical potency at low nanomolar concentrations. CBL-B inhibitors enhanced in vitro T cell activation measured by cytokine secretion at low nanomolar concentrations in primary human and mouse T cells stimulated with anti-CD3/anti-CD28 or anti-CD3 alone.

**Results**

Furthermore, the compounds increased antigen recall responses in human PBMCs. CBL-B inhibitors also amplified T cell responses in models of anergy and exhaustion, with greater effects observed when combined with a checkpoint inhibitor. The CBL-B inhibitors also enhanced cytokine response in primary human NK cells measured by TNFα and IFNγ secretion. Oral dosing of an optimized CBL-B inhibitor enhanced anti-CD3 stimulated T cell activation in mouse CD4+ and CD8+ T cells, demonstrating a dose proportional pharmacodynamic effect.

**Conclusions**

These data support the continued advancement of small molecule oral CBL-B inhibitors for future development in immuno-oncology.

**References**

1. Loeser S, Loser K, Bijker MS, Rangachari M, van der Burg SH, Wada T Beissert S, Melief CJ, Penninger JM. Spontaneous tumor rejection by cbl-b-deficient CD8+ T cells. J Exp Med, 2007;204(4):879-91.

2. Paolino M, Thien CB, Gruber T, Hinterleitner R, Baier G, Langdon WY, Penninger JM. Essential role of E3 ubitquitin ligase activity in Cbl-b-regulated T cell functions.J Immunol. 2011 Feb 15;186(4):2138-47.

3. Paolino M, Choidas A, Wallner S, Pranjic B, Uribesalgo I, Loeser S, Jamieson AM, Langdon WY, Ikeda F, Fededa JP, Cronin SJ, Nitsch R, Schultz-Fademrecht C, Eickhoff J, Menninger S, Unger A, Torka R, Gruber T, Hinterleitner R, Baier G, Wolf D, Ullrich A, Klebl BM, Penninger JM. The E3 ligase Cbl-b and TAM receptors regulate cancer metastasis via natural killer cells. Nature. 2014 Mar 27;507(7493):508-12.

#### P670 Pharmacodynamic response in vitro and in vivo of novel orally administered toll-like receptor 7 agonists for systemic immunotherapy of cancer

##### James Appleman, PhD, Stephen Webber

###### Primmune Therapeutics, Inc., San Diego, CA, United States

####### **Correspondence:** James Appleman (jappleman@primmunerx.com)

**Background**

While ICPIs (immune checkpoint inhibitors) have fundamentally changed the practice of cancer therapy for tumors arising from many different tissues, ways to increase both response rate and durability are critically needed. Combining stimulators of innate immunity with activators of adaptive immunity should lead to better treatment outcomes. We have therefore created a series of novel orally administered, systemically acting Toll-like receptor 7 (TLR7) agonists for long-term combination therapy with enhancers of adaptive immunity like ICPIs. Our molecules incorporate a set of atypical properties critical to achieving this product profile [1].

**Methods**

Key assays in our testing cascade include:

• Cellular reporter assays and human PBMC assays to evaluate selectivity, potency and other characteristic aspects of cellular pharmacology (e.g. targeted cytokine and chemokine profiles)

• Characterization in cynomolgus monkeys

---Appropriate PK with active TLR7 agonist

---Efficient oral delivery into systemic circulation with prodrugs of the TLR7 agonist

---Targeted pharmacodynamic response at relevant oral dose (gating for candidate selection)

Additional descriptive assays include:

• Evaluation of potency and cellular pharmacology in monkey PBMCs

• In vitro predictors of pharmacokinetics and safety

• Pharmacodynamic response in rodents

• Antitumor activity in syngeneic rodent tumor models

**Results**

While TLR7 agonists having appropriate potency and specificity were discovered early in our program, systemic oral delivery even with an optimized prodrug approach was relatively poor. Modification of the lead TLR7 agonist itself increased efficiency of oral delivery by the prodrug from 14% for Compound A to 99% for Compound C (Figure 1) while also modestly increasing potency in PBMC assays. Surprisingly, Compound C is significantly less potent in monkey plasmacytoid dendritic cells (PDCs) than in human PDCs. Consequently doses of the prodrug of Compound C eliciting our targeted degree of immune induction in cynomolgus monkeys are higher than anticipated. In contrast, Compound B has equivalent potency in human and monkey PBMC assays. While either of these compounds may be suitable for development, we continue to investigate additional compounds that retain the preferred features found in Compounds B and C while having greater potency in cellular assays.

**Conclusions**

From our original starting point - a relatively weak TLR7 agonist with no oral bioavailability - we have invented a novel series of molecules that are designed to be dosed QOD continuously over a 24-month period to appropriately engage innate immunity at a level that is well-tolerated by the patient while increasing treatment response rate and durability.

**References**

1. Appleman J, Webber, S. Discovery of a series of novel toll-like receptor 7 agonists for systemic immunotherapy of cancer [abstract]. In: Proceedings of the Annual Meeting of the American Association for Cancer Research 2019. Mar 29 - Apr 3; Atlanta, GA. Abstract # 3262.

**Fig. 1 (abstract P670).**
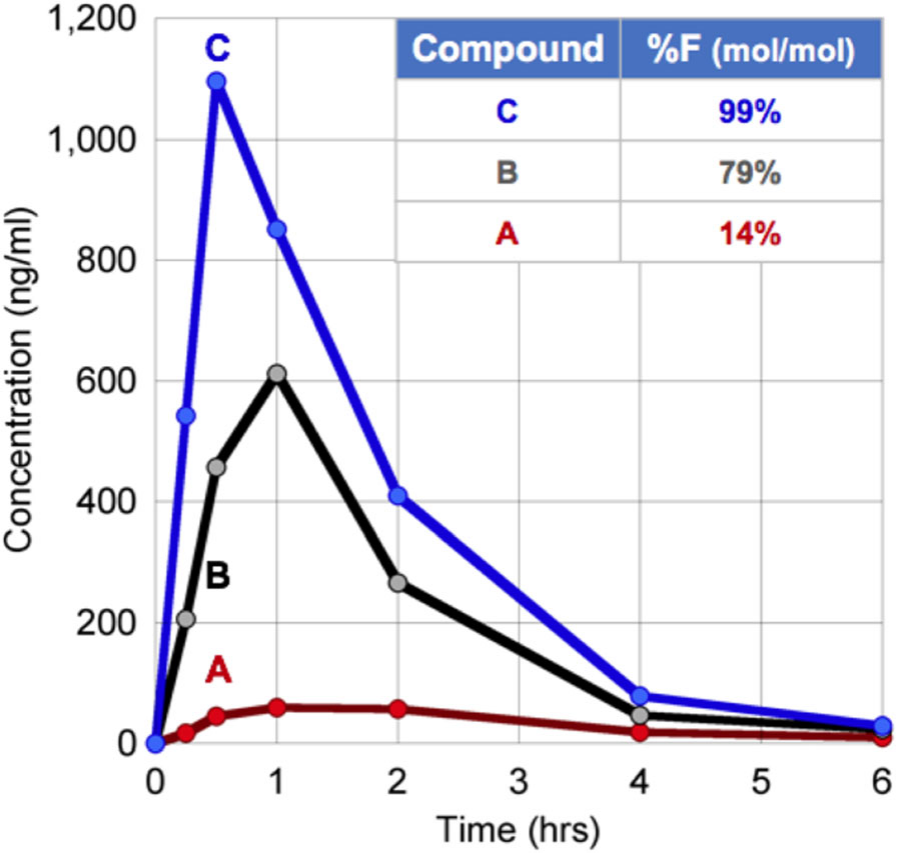
Efficient Oral Delivery of Novel TLR7 Agonists

#### P671 Delivery of TLR7 agonists by Deep-Primed™ T cells induces immune activation and improves anti-tumor activity in mice while circumventing systemic toxicity

##### Austin Boesch, PhD^1^, Vasily Rybakin^1^, Nathan Westcott^1^, Ji Young Hwang^1^, Kira Jørgensen^2^, Rasmus Lassen^1^, Martin Kraemer^2^, Martin Bak, PhD^2^, Gael Veiga^2^, Jonas Bruun, PhD^1^, Carlos Tassa^1^, Harrison Rodts^1^, Manny Sequeira^1^, Glenn Leary^1^, Santina Caruso^1^, Becker Hewes, MD^1^, Jonathan Fitzgerald, PhD^1^, Karsten Sauer, PhD^1^, Thomas Andresen, PhD^1^

###### ^1^Torque Therapeutics, Cambridge, MA, United States; ^2^Technical University of Denmark, Lyngby, Denmark

####### **Correspondence:** Austin Boesch (aboesch@torquetx.com)

**Background**

TLR7 agonists boost immune responses in the tumor microenvironment (TME), primarily through dendritic cell (DC) engagement, enhancement of antigen presentation and T cell co-stimulation. However, multiple TLR agonists have displayed unfavorable PK/PD profiles and considerable toxicities upon systemic administration. Instead, we designed a T cell mediated delivery system of TLR7 agonists that target TME and the lymphatic system to maximize efficacy while avoiding systemic toxicities. Torque’s Deep-Primed™ T cell technology enhances T cell function through tethering of immune modulators to the cell before adoptive cell transfer (ACT) and uses Torque’s multi-targeted T cells (MTC) platform that targets multiple tumor antigens. By transporting payload to antigen-expressing tissues, Deep-Primed™ MTC’s focus the effect of the immunomodulators on these tissues. Here, we show that Deep TLR Primed T cells delivering TLR7 agonists induce potent immune cell activation in the TME and elicit exquisite anti-tumor efficacy without overt toxicity.

**Methods**

TLR7 agonists and several liposomal formulations were screened for optimal T cell tethering and release, measured by HPLC. Formulations with desired characteristics were tethered to murine PMEL CD8 T cells specific for the B16-F10 melanoma antigen gp100 to generate Deep TLR Primed T cells. Following ACT into immunocompetent syngeneic tumor-bearing animals, the T cell product was evaluated for efficacy and immune cell activation.

**Results**

ACT with Deep TLR Primed PMEL T cells inhibited tumor growth significantly more than ACT of PMEL T cells alone or combined with systemically delivered TLR agonists. Deep TLR Primed cells displayed higher proliferation but lower PD-1 expression in blood and tumors and caused a trend towards an increase in CD8+ DC, plasmacytoid DC, and MDSC in the TME. Despite their remarkable efficacy, ACT of Deep TLR Primed PMEL T cells caused no treatment-specific weight loss and elicited substantially lower levels of plasma IFNg and IL-12 than systemic TLR7 agonist delivery.

**Conclusions**

Deep TLR Primed T cells display superior efficacy, PD, and safety compared to T cells alone or co-administered with systemic TLR agonists. Torque’s Deep-Primed™ technology allows the delivery of small molecules to the TME, with controlled doses. Compared to intratumoral delivery, agonist delivery via Deep-Primed™ tumor antigen-specific autologous T cells can target a wider variety of tumors, including distant metastases. It has the potential to improve agonist PK profile through sustained delivery over time in TME and draining lymph nodes, with limited systemic exposure.

#### P672 Activation of CD8+ T cells in the presence of multiple TLR agonists differently affects the expression of T-cell checkpoint receptors

##### Donghwan Jeon, MS, Christopher Zahm, PhD, Douglas McNeel, MD, PhD

###### University of Wisconsin-Madison, Madison, WI, United States

####### **Correspondence:** Douglas McNeel (dm3@medicine.wisc.edu)

**Background**

Expression of T-cell checkpoint receptors occurs following T-cell activation during exhaustion, and ligation of these receptors can impair T-cell function and anti-tumor immunity [1]. We previously found that T cells activated with cognate antigen upregulate the expression of PD-1, and the expression of PD-1 can be attenuated by the presence of specific Toll-like receptor (TLR) agonists [2,3]. This effect was mediated by IL-12 secretion from professional antigen presenting cells and resulted in CD8+ T cells with greater anti-tumor activity. In the current report, we sought to determine whether multiple TLR agonists can affect the expression of T-cell checkpoint receptors.

**Methods**

Splenocytes were isolated from OT-1 mice and stimulated with chicken ovalbumin peptide (SIINFEKL) in the presence of different combinations of TLR agonists for 4 days. Cells were collected daily and evaluated for expression of a marker of activation (4-1BB) and T-cell checkpoint receptors (PD-1, CTLA-4, CD160, CD244, LAG-3, TIM-3, TIGIT and VISTA) by flow cytometry. Purified dendritic cells were stimulated with these combinations of TLR agonists and evaluated for IL-12 release by ELISA.

**Results**

Activation of CD8+ T cells in the presence of specific TLR ligands resulted in decreases in expression of PD-1 and/or CD160 and increases in CTLA-4. These changes in T-cell checkpoint receptor expression were modestly affected when TLR ligands were used in combination. Similarly, stimulation of DC with several TLR agonists resulted in secretion of IL-12 that was modestly increased by combination treatment.

**Conclusions**

Multiple TLR agonists can modulate the expression of T-cell checkpoint receptors, notably PD-1 and CTLA-4. These data may provide rational combinations of TLR ligands that could be investigated as vaccine adjuvants, alone or in combination with T-cell checkpoint blockade, to improve the efficacy of anti-tumor vaccines.

**References**

1. Jin HT, Anderson AC, Tan WG, et al. Cooperation of Tim-3 and PD-1 in CD8 T-cell exhaustion during chronic viral infection. Proc Natl Acad Sci USA. 2010;107(33):14733-8.

2. Zahm CD, Colluru VT, Mcneel DG. Vaccination with High-Affinity Epitopes Impairs Antitumor Efficacy by Increasing PD-1 Expression on CD8 T Cells. Cancer Immunol Res. 2017;5(8):630-641.

3. Zahm CD, Colluru VT, Mcilwain SJ, Ong IM, Mcneel DG. TLR Stimulation during T-cell Activation Lowers PD-1 Expression on CD8 T Cells. Cancer Immunol Res. 2018;6(11):1364-1374.

#### P673 Targeted delivery system for toll-like receptor agonists to repolarize the immunosuppressive phenotype of tumor-associated macrophages

##### Soyoung Son, Hyewon Ko, Sol Shin, Jae Hyung Park, PhD

###### Sungkyunkwan University, Seoul, Korea, Republic of

####### **Correspondence:** Jae Hyung Park (jhpark1@skku.edu)

**Background**

Although immune checkpoint blockade therapy (ICT) contributed remarkably to elevated survival rate and durable tumor remission for the past decade, several limitations of ICT such as that only partial patients have corresponded to those therapeutic interventions have appeared. Therefore, recently, the need for combination therapies have been emerged to improve the response rate of ICT and elicit robust antitumor immunity. In the tumor microenvironment, multiple immunosuppressive immune cells (e.g. Treg, tumor-associated macrophage (TAM), MDSC) reside and they hamper antitumor immunity. Specifically, TAMs have been intensively studied to turn immunosuppressive microenvironment into an immunosupportive one, since they have plasticity in phenotypes (i.e. immunosupportive M1 macrophages and immunosuppressive M2 macrophages). In the tumor microenvironment, the dominant phenotype of TAMs are M2-like TAM, and they hinder the effector function of cytotoxic T lymphocytes (CTL) and NK cells. In this respect, we developed the polymeric nanocarrier for the targeted repolarization of M2-like TAMs toward tumoricidal M1-like TAMs to enhance the tumor targetability, reduce systemic adverse effects, and reinvigorate antitumor immunity.[1-3]

**Methods**

For in vitro studies, bone marrow-derived macrophages (BMDMs) were prepared as previously reported. To conduct in vivo studies, CT26-bearing mice were prepared and the tumor-infiltrating leukocytes were separated using MACS for further flow cytometric analysis.

**Results**

The polymeric nanocarrier (TR-NP) to target M2-like TAMs was successfully prepared by the layer-by-layer assembly, resulting in 145 nm of hydrodynamic diameter. TR-NPs targeting IL-4R alpha selectively demonstrated the enhanced uptake by M2-polarized BMDMs and effectively deliver the TLR agonists. As the main purpose of this study is to repolarize M2-like TAMs toward M1-like TAMs, we carried out in vitro flow cytometric analysis for macrophage markers. Upon treatment of TR-NPs, the expression levels of M2 markers (CD206, Arginase) decreased and the expression levels of M1 markers (iNOS, CD86, MHC II) increased, compared with untreated macrophages or free TLR agonist. Furthermore, we performed in vivo antitumor efficacy for combination therapy with PD-L1 antibody to assess the improvement of ICT. Consequently, TR-NP treatment showed an increased population of M1-like TAMs, CD8+ T cell in the tumor tissue, leading to effective combination therapy.

**Conclusions**

In this study, we designed the polymer nanocarrier that targets TAMs and repolarizes them toward M1-like macrophages. The TR-NPs successfully altered M2-like macrophages to anti-tumoral ones, thereby inhibiting the cancer cell proliferation. Consequently, TR-NP treatment provided immune-activating microenvironment by TAM repolarization and CTL infiltration in vivo, suggesting the potential as an immune adjuvant for combination therapy with ICT.

**Acknowledgements**

This work was financially supported by the Basic Science Research Programs (2018R1D1A1B07047950) of the National Research Foundation (NRF), Republic of Korea.

**References**

1. Pitt JM, Vetzou M, Daillere R, Roberti MP, Yamazaki T, Routy B, Lepage P, Boneca IG, Chamaillard M, Kroemer G, Zitvogel L. Resistance Mechanisms to Immune-Checkpoint Blockade in Cancer: Tumor-Intrinsic and -Extrinsic Factors. Immunity 2016;44:1255-1269.

2. van der Burg SH, Arens R, Ossendorp F, van Hall T, Melief CJ. Vaccines for established cancer: overcoming the challenges posed by immune evasion. Nat Rev Cancer. 2016;16:219-233.

3. Mantovani A, Marchesi F, Malesci A, Laghi L, Allavena P. Tumour-associated macrophages as treatment targets in oncology. Nat Rev Clin Ocol. 2017;14:399-416.

**Ethics Approval**

The study was approved by Sungkyunkwan University‘s Ethics Board, approval number SKKUIACUC2019-01-07-1.

#### P674 In situ vaccination using a tumor-binding polymeric glyco-adjuvant for the induction of anti-tumor immunity

##### Tiffany Marchell, David Wilson, Aaron Alpar, Jeffrey Hubbell

###### University of Chicago, Chicago, IL, United States

####### **Correspondence:** Jeffrey Hubbell (jhubbell@uchicago.edu)

**Background**

Difficultly translating therapeutic vaccination success from murine models to the clinical treatment of cancer has highlighted the need for vaccination strategies that may be more broadly applicable. To address this problem, we developed an in situ vaccination strategy to adjuvant tumor cells directly, utilizing the tumor itself as the antigen source in initiating tumor-reactive cellular responses. Our engineered vaccine is comprised of a novel polymeric TLR7-agonist (pTLR7) [1] chemically linked to tumor-binding antibodies. We hypothesized that following intratumoral administration, our pTLR7-(tumor-binding antibody) conjugates (pTLR7-tAbs) would bind to surface molecules expressed by tumors, thereby increasing persistence of the adjuvant within the tumor. Given the ease of conjugation of pTLR7 to various antibodies, our in situ pTLR7-tAb vaccination platform is tunable in specificity from highly tumor-specific to widely applicable, depending on the choice of antibody used. For generalizability between tumor models, we selected an anti-CD47 antibody to create anti-CD47-pTLR7 conjugates for testing our vaccination.

**Methods**

Efficacy studies were conducted in poorly immunogenic orthotopic murine tumor models of EMT6 breast cancer and B16F10 melanoma. Biodistribution and intratumoral retention studies were conducted using an in vivo imaging system (IVIS). Intratumoral T cell and antigen presenting cell numbers, frequencies, and activation phenotype were assessed via flow cytometry.

**Results**

Tumor retention studies tracking fluorescently labeled pTLR7-tAbs showed pTLR7-tAbs are retained within the tumor microenvironment (TME) 3-fold longer than isotype control antibody-pTLR7 constructs. Interestingly, following vaccination, pTLR7-tAb treated tumors showed increased numbers and activation of intratumoral antigen presenting cells (APCs). Assessment of tumor infiltrating lymphocytes (TIL) showed pTLR7-tAb vaccination stimulated tumor antigen TRP2-specific T cell responses in the B16F10 melanoma model and converted these immunologically ‘cold’ tumors into T cell inflamed tumors, with increased numbers of CD4+ and CD8+ T cells. Strikingly, we observed complete remission (CR) in 50% of ‘cold’ immune-excluded EMT6 breast cancer tumors and delayed tumor progression of B16F10 melanoma after treatment with pTLR7-tAbs (p

**Conclusions**

Together, these data support the use of our pTLR7-tAb platform for in situ vaccination and as a therapeutic tool for enhancing anticancer immunity.

**References**

1. Wilson DS, Hirosue S, Raczy M, Bonilla-Ramirez L, Jeanbart L, Wang R, Kwissa M, Franetich JF, Broggi MAS, Diaceri G, Quaglia-Thermes X, Mazier D, Swartz MA, Hubbell JA. Antigens reversibly conjugated to a polymeric glyco-adjuvant induce protective humoral and cellular immunity. Nat Mater. 2019; 18:175–185.

**Ethics Approval**

All studies with animals were carried out in accordance with procedures approved by the Institutional Animal Care and Use Committee at the University of Chicago.

#### P675 Multimodal intratumoral immunotherapy potentiates complete immunologic tumor elimination of breast cancer origin diffuse liver metastases

##### Erik Soule^1^, Jason Williams, MD^2^ , Jason Williams, MD^2^

###### ^1^University of Florida College of Medicine, Gainesville, FL, United States; ^*2*^*Williams Cancer Institute, Foley, AL, United States*

####### **Correspondence:** Jason Williams (drwilliams@cancerimmunebio.com)

**Background**

Depending on the origin of the primary lesion, patients with distant metastases of aggressive solid tumors have traditionally been deemed incurable. A complete abscopal effect, or cure, is a rare exception rather than the rule. This is secondary to cancer stem cells providing resilience to treatment and propensity to recur after remission [1]. Harnessing the potential of the immune system to recognize and destroy malignant cells harboring tumor antigens may allow stage 4 cancer to be completely eliminated from the body [2, 3]. This report describes a case of diffuse liver metastases from advanced breast cancer; eliminated immunologically by intratumoral injection of immunostimulatory pharmacotherapy. α-OX40 refers to a stimulatory monoclonal antibody with a dual mechanism of action on T cells designed to potentiate long-term immunity. Activating the OX40 protein triggers a sustained immune response in effector T cells while also potentiating the demise of regulatory T cells. Intratumoral α-OX40 was shown to work synergistically with intratumoral CpG, a toll-like receptor 9 agonist, in a mouse model of breast cancer [4]. To our knowledge, this is the first report of an α-OX40 antibody administered intratumorally, in vivo, in a human patient.

**Methods**

Two liver metastases were treated with combination immunotherapy suspended in montanide, an oil adjuvant. A combination of α-OX40, CpG, ipilimumab, and ketolorac was injected into two accessible, dominant, hepatic lesions under computed tomography guidance with general anesthesia. A CT scan was performed 6 weeks post-procedure, and a PET/CT was performed 12 weeks post-procedure. Tumor markers CA125, CA27.29, and CA15.3 were monitored weekly for 12 weeks post treatment.

**Results**

No suspicious uptake was noted anywhere in the body on the 12-week post-procedure PET/CT. Complete abrogation of all diffuse liver metastases was documented at that time. At 12 weeks post treatment tumor markers CA125 decreased by 98% to 35 from a baseline of 1767, CA27.29 decreased 93% to 112 from a baseline of 1511, and CA15.3 decreased 93% to 56 from a baseline of 821.

**Conclusions**

Intratumoral injection allows a much higher proportion of immunotherapy to directly interact with the tumor microenvironment compared to intravenous injection. With intratumoral dosing of multimodal immunotherapy an order of magnitude less than intravenous dosing, a patient with stage 4 breast cancer was able to achieve complete immunologic tumor elimination at 12 weeks follow-up. This durable abscopal effect remains complete 20 weeks post-treatment.

**References**

1. Kreso A, Dick JE. Evolution of the cancer stem cell model. Cell Stem Cell. 2014 Mar 06,;14(3):275-91.

2. Rosenberg MA, Williams J. Image guided cryoablation of cancer with intra-tumoral injection of anti-CTLA-4 and PD-1 immune check-point inhibitors. Journal for ImmunoTherapy of Cancer. 2015;3(2):P142.

3. Soule E, Bandyk M, Matteo J. Percutaneous ablative cryoimmunotherapy for micrometastaic abscopal effect: No complications. Cryobiology. 2018 06;82:22-6.

4. sagiv-Barfi I, Czerwinski DK, Levy R. In Situ Vaccination with a TLR9 Agonist and Anti-OX40 Antibody Leads to Tumor Regression and Induces Abscopal Responses in Murine Lymphoma. Blood. 2016;128(22):1847.

**Consent**

Written informed consent was obtained from the patient for publication of this abstract and any accompanying images. A copy of the written consent is available for review by the Editor of this journal.

#### P676 Intratumoral delivery of TransCon™ TLR7/8 Agonist provides potent anti-tumor activity as a monotherapy and in combination with IL-2 while minimizing systemic cytokine induction

##### Luis Zuniga, PhD^1^, Torben Leßmann^2^, Lars Holten-Andersen^3^, Nicola Bisek^2^, Joachim Zettler^2^, Sebastian Stark^2^, Frank Faltinger^2^, Oliver Kracker^2^, Samuel Weisbrod^2^, Robin Müller^2^, Tobias Voigt^2^, Kornelia Bigott^2^, Mohammad Tabrizifard^1^, Vibeke Breinholt^3^, Kennett Sprogøe^3^, Juha Punnonen^1^

###### ^*1*^*Ascendis Pharma, Inc., Palo Alto, CA, United States;*^*2*^*Ascendis Pharma GmbH, Heidelberg, Germany* ; ^*3*^*Ascendis Pharma A/S, Copenhagen, Denmark*

####### **Correspondence:** Juha Punnonen (jpn@ascendispharma.com)

**Background**

Local delivery of pattern recognition receptor agonists (PRRAs) to the tumor microenvironment (TME) stimulates innate immune sensors such as toll-like receptors (TLR) and can enhance antigen uptake and presentation, induce proinflammatory immune cell recruitment, and reverse tumor-associated immunosuppression (1, 2). Local delivery of PRRAs, such as STING or TLR agonists, has shown encouraging preclinical and clinical anti-tumor benefit (3-5). However, current approaches to deliver PRRAs to the TME suffer from the lack of local retention in the TME and rapid drug clearance, thus limiting anti-tumor benefit, promoting systemic treatment-related adverse events (e.g. cytokine storm), and necessitating frequent and often impractical dosing regimens. Additionally, systemic toxicity associated with current PRRA treatments may limit combination therapies (2, 6).

**Methods**

Given the need for safe and effective intratumoral therapies, we developed a sustained-release TransCon™ (refers to “transient conjugation”) TLR7/8 Agonist prodrug by conjugating resiquimod to a hydrogel via a TransCon linker to provide consistent intratumoral release of unmodified resiquimod.

**Results**

In vivo pharmacokinetics in rodents, following local injection of TransCon TLR7/8 Agonist, showed long-term resiquimod release over several weeks with minimal systemic exposure compared to an equivalent dose of unconjugated resiquimod. Intratumoral injection of TransCon TLR7/8 Agonist also demonstrated prolonged release. Furthermore, in a syngeneic tumor model, a single intratumoral injection of TransCon TLR7/8 Agonist mediated significant tumor growth inhibition and was associated with significantly lower systemic proinflammatory cytokine induction when compared to an equivalent dose of unconjugated resiquimod. Intratumoral delivery of TransCon TLR7/8 Agonist was well tolerated and mediated tumor growth inhibition in a dose-dependent fashion. Finally, in a bilateral syngeneic tumor model, TransCon™ TLR7/8 Agonist mediated significant tumor growth inhibition in both injected and non-injected tumors as a monotherapy and when combined with systemic IL-2 treatment.

**Conclusions**

These data provide strong evidence that a single dose of TransCon TLR7/8 Agonist can mediate long term local release of unmodified resiquimod with minimal systemic exposure and systemic pro-inflammatory induction compared to an equivalent dose of unconjugated resiquimod. Moreover, TransConTLR7/8 has potent anti-tumor effects as a monotherapy and in combination with cytokine therapy, suggesting further evaluation as a monotherapeutic and in combination with other immunotherapies is warranted. TransCon TLR7/8 Agonist represents a potentially novel PRRA therapy class that may overcome the shortcomings of existing PRRA treatments by providing a potent anti-tumoral response and reducing adverse events related to systemic drug exposure.

**References**

1. Aznar MA, Tinari N, Rullan AJ, Sanchez-Paulete AR, Rodriguez-Ruiz ME, Melero I. Intratumoral Delivery of Immunotherapy-Act Locally, Think Globally. J Immunol.2017;198:31-39.

2. Marabelle A, Tselikas L, de Baere T, Houot R. Intratumoral immunotherapy: using the tumor as the remedy. Ann Oncol.2017;28:xii33-xii43.

3. Rook AH, et al. Topical resiquimod can induce disease regression and enhance T-cell effector functions in cutaneous T-cell lymphoma. Blood.2015;126:1452-1461.

4. Clark CM, Furniss M, Mackay-Wiggan JM. Basal cell carcinoma: an evidence-based treatment update. Am J Clin Dermatol.2014;15:197-216.

5. Singh M, et al. Effective innate and adaptive antimelanoma immunity through localized TLR7/8 activation. J Immunol.2014;193:4722-4731.

6. Marabelle A, et al. Starting the fight in the tumor: expert recommendations for the development of human intratumoral immunotherapy (HIT-IT). Ann Oncol.2018;29:2163-2174.

#### P677 Warehouse approach for the development of personalized cancer vaccines by using Personal Antigen Selection Calculator (PASCal) without need for tumor biopsy

##### Zsolt Csiszovszki, PhD^1^, Levente Molnár^1^, Péter Páles^1^, József Tóth^1^, Orsolya Lőrincz^1^, Katalin Pántya^1^, Eszter Somogyi^1^, István Miklós^2^, Wolfgang Schönharting^3^, Sybille Urban^3^, Tim Röhnisch^4^, Mónika Megyesi^1^, Enikő Tőke^1^

###### ^1^Treos Bio Zrt., Budapest, Hungary; ^*2*^*Treos Bio Zrt. and MTA Rényi Institute, Veszprém, Hungary* ; ^*3*^*PMCR GmbH, Karlsruhe, Germany* ; ^*4*^*Interdisciplinary Oncology Center, Munich, Germany*

####### **Correspondence:** Enikő Tőke (eniko.toke@treosbio.com)

**Background**

Analysis of current data with cancer vaccines suggests that the lack of efficacy is likely due to their two primary design challenges: 1. Vaccines have little chance of destroying heterogeneous tumor cells since they rarely induce polyclonal T-cell responses; 2. Even when polyclonal T-cell responses have been successfully induced, they are often directed against tumors where the target is absent (not expressed). Recent mutated neoantigen-based vaccines (MNeoV) aim to solve this latter issue, however only about 10-20% of selected epitopes proved to induce CD8+ T-cell responses in patients. In addition, development of MNeoV for commercial use is challenging. To overcome these limitations, we developed PASCal for improved selection of peptides (epitopes) that induce T-cell responses targeted against heterogeneous tumor cells.

**Methods**

PASCal operates by 3 moduls: (1) validated epitope database containing 108 true HLA-epitope pairs (2) Expression frequency-based shared tumor antigen database established for 19 indications based on >96,000 tumor biopsies. (3) Validated algorithm for the identification of immunogenic peptides by the selection of personal epitopes (PEPIs) binding to multiple autologous HLA alleles.[1,2] Using PASCal, a library of 3,286 immunogenic 20mer peptides derived from 184 antigens associated with 19 cancer indications -based on 16,000 subjects’ HLA genotype (both class I&II alleles) was compiled. Personal vaccines were selected and tested for 3 HLA-genotyped metastatic cancer patients (with ovarian-, breast- and colorectal cancer). Immunogenicity of the vaccines was tested by IFN-γ ELISPOT. Signed informed consent were obtained from each patient, including allowance of publication.

**Results**

Personal cancer vaccines were selected to fulfill the following criteria: 12 immunogenic peptides derived from 12 different tumor-specific antigens frequently expressed in the patient’s disease type, with the expected number of expressed antigens on the patient’s tumor cells of at least 3 (by statistical estimation). CD8+ T-cell responses were induced by 97%, CD4+ T-cell responses by 85% of peptides, confirming aimed polyclonal T-cell responses. Long-lasting CD8+ T-cell responses were detected ex vivo 4.5 months, in vitro 14 months after last vaccination. Pre-existing T-cell reactivities were detected against at least 25% of vaccine antigens demonstrating their presence in the patients’ tumor, confirming the success of vaccine design strategy aiming to induce polyclonal T-cell responses against at least 3 antigens expressed by the tumor.

**Conclusions**

PEPIs outperform reported immunogenicity of mutated neoantigen-based personal vaccines and induced unprecedented immune responses in cancer patients. The „off-the-shelf” personalized approach with PASCal enables commercially scalable vaccine development, without need for tumor biopsy and on-demand manufacturing.

**References**

1. Hubbard JM, et al. J. Clin. Oncol., 37, 2019 (suppl; abstr 3557)

2. Toke ER, et al, J. Clin. Oncol., 37, 2019 (suppl; abstr e14295)

**Ethics Approval**

This study was performed in accordance to the Declaration of Helsinki (amendment 37). Signed informed consent were obtained from each patient, including allowance of publication.

**Consent**

Signed informed consent were obtained from each patient, including allowance of publication.

#### P678 Vaccine neoantigens empirically identified through the ex vivo ATLAS platform promote potent therapeutic responses to cancer in mice

##### Victoria DeVault, PhD, Hanna Starobinets, PhD, Sanmit Adhikari, Brendan Classon, PhD, Jessica Flechtner, PhD, Hubert Lam, PhD

###### Genocea Biosciences, Cambridge, MA, United States

####### **Correspondence:** Jessica Flechtner (jessica.flechtner@genocea.com)

**Background**

Neoantigens are mutated tumor antigens that make attractive targets for immunotherapy as they are recognized as foreign by the immune system. While considered as the optimal strategy for targeting immune tumor killing while minimizing risks of autoimmunity, identification of true neoantigens has been elusive due to poor predictive value of in silico selection. ATLAS™ is a T cell response profiling platform that does not use predictive methods, but instead employs high-throughput ex vivo screening of putative neoantigens using autologous antigen presenting cells and T cells. Antigens are characterized as stimulatory or inhibitory by significant up- or downregulation of inflammatory T cell cytokine secretion relative to controls. Neoantigen identification using an autologous cell assay allows differentiation of desired as well as potentially unwanted antigen-specific T cell responses that are biologically-relevant to the patient.

**Methods**

ATLAS screening of the B16F10 melanoma mutanome was performed, consisting of whole exome sequencing, non-synonymous mutation library construction, and screening of splenic CD8+ T cells from tumor-bearing C57BL/6 mice. Stimulatory or inhibitory neoantigens that modulated secretion of IFNγ and/or TNFα were identified and corresponding synthetic long peptides were evaluated in a therapeutic B16F10 tumor challenge model. Vaccine immune responses were measured by ELISpot, flow cytometry and immunohistochemistry, and animals were monitored for tumor growth and survival.

**Results**

ATLAS screening of B16F10 melanoma identified 66 stimulatory and 57 inhibitory neoantigens, the majority of which were not predicted by peptide binding affinity. Strikingly, when an adjuvanted antigen cocktail containing a top stimulatory neoantigen was therapeutically administered as monotherapy into tumor-bearing mice, tumor outgrowth was either significantly delayed or completely abrogated in all mice (n=12). Conversely, therapeutic immunization with adjuvanted inhibitory neoantigen peptides resulted in no benefit and in some cases led to a significant increase in tumor growth kinetics representative of tumor hyperprogression. Stimulatory antigen vaccination induced significant antigen-specific inflammatory T cell responses, whereas T cell responses to inhibitory antigens were variable. Immunohistochemistry analysis of hyperprogressing tumors indicate reduced infiltration of CD8+ T cells.

**Conclusions**

Currently, the only method to reliably and comprehensively identify neoantigen targets and rationally omit potentially deleterious antigens is ATLAS screening using autologous cells. These studies demonstrate proof of concept for ATLAS-based neoantigen vaccine selection and establish a model for further mechanistic study. In a recent interim analysis, 91% of vaccine neoantigens selected as stimulatory by ATLAS, elicited T cell responses in patients participating in the GEN-009 Phase 1/2 clinical trial of a personalized cancer vaccine (NCT03633110).

**Ethics Approval**

Animals were housed within a USDA registered, AAALAC accredited and OLAW assured (#A4591-01) animal facility in accordance with the Institutional Animal Care and Use Committee (IACUC).

#### P679 Scheduling androgen deprivation with AR-targeted vaccination affects anti-tumor efficacy in prostate cancer

##### Melissa Gamat-Huber, PhD, Douglas McNeel, MD, PhD

###### University of Wisconsin Madison, Madison, WI, United States

####### **Correspondence:** Douglas McNeel (dm3@medicine.wisc.edu)

**Background**

Androgen deprivation therapy is the primary treatment for recurrent and metastatic prostate cancer. Androgen deprivation can also modulate the immune system and cause effects such as T cell infiltration into the prostate, affect antigen processing and/or presentation and possibly affect T cell homeostasis. In previous studies, we have shown that immunizing tumor-bearing mice using a DNA vaccine against the androgen receptor significantly slowed down tumor growth compared to control. Furthermore, combining androgen deprivation with vaccination against the AR further augments the anti-tumor response compared to the AR vaccination alone. In this study, we hypothesized that the timing of DNA vaccination with respect to androgen deprivation treatment, may impact the efficacy of the anti-tumor effect.

**Methods**

We implanted the androgen-responsive mouse prostate tumor line MycCaP into 6 week old male FVB mice. The tumors were allowed to grow for up to 32 days, then mice were treated with androgen deprivation therapy (degarelix). Vaccines consisted of either the AR ligand binding domain within the pTVG4 backbone (pTVG-AR), or the pTVG4 backbone alone (control). Immunizations were either started one week after tumor implantation (Vx→ADT), or one day following androgen deprivation (ADT→Vx).

**Results**

Mice treated with AR vaccine before androgen deprivation (AR →ADT) had slower tumor growth compared to the control plasmid (pTVG4 →ADT) but not when vaccinated after ADT (ADT→Vx). Furthermore, when immunizations were given before androgen deprivation and continued after androgen deprivation, the emergence of castration resistance was further delayed.

**Conclusions**

These results have implications on the scheduling of androgen deprivation with immunotherapy for the treatment of prostate cancer, and correspond to similar results observed in human clinical trials with anti-tumor vaccines.

#### P680 Discovery of immunogenic ERV-derived antigens as targets for melanoma immunotherapy

##### Ray Jupp, PhD^1^, George Kassiotis^2^, Nicola Ternette^3^, George Young^1^, Duncan Howie^1^, Fabio Marino^1^, Matthew Davies^1^, Hayden Selvadurai^1^, Alvaro Sanchez^1^, Jonathan Dodd^1^, Laura Lozza^1^, Elizabeth Soilleux^4^, Peter Mason^5^, Kevin Pojasek^1^

###### ^1^Ervaxx, Ltd, Oxford, United Kingdom; ^*2*^*The Francis Crick Institute, London, United Kingdom*; ^*3*^*University of Oxford, Oxford, United Kingdom* ; ^*4*^*University of Cambridge, Cambridge, United Kingdom*; ^*5*^*Ervaxx, Inc, Somerville, MA, United States*

####### **Correspondence:** Kevin Pojasek (kpojasek@svhealthinvestors.com)

**Background**

Recent advances in immunotherapy have confirmed that adaptive immune responses can recognize and eliminate cancer cells. Past approaches using tumor-associated antigens have elicited poor immune responses and the cancer vaccine field has shifted to single nucleotide variant-derived neoantigens to avoid T-cell depletion by central tolerance. These highly personalized vaccines are beginning to show promise. We hypothesized that highly immunogenic target antigens exist within the cancer genome and become aberrantly expressed due to the epigenetic changes that accompany neoplasia. We utilized markers of endogenous retroviruses (ERVs), which are particularly abundant in repressed regions of DNA, to search for novel, high-value melanoma antigens, to circumvent the need for personalization.

**Methods**

We created a de novo pan-cancer transcriptome assembly with RNA-seq reads from 31 cancer types obtained from TCGA. Transcript sequences were subsequently filtered to identify those containing ERV elements. Next, they were subject to differential expression analysis selecting those expressed in the melanoma patients compared to normal tissues (utilizing GTEx data).

To discover bona fide antigens encoded by our melanoma-specific transcripts, we interrogated the ORFs from these transcripts using public and independently-generated mass spectrometry-based immunopeptidomics data from melanoma samples. HLA-bound peptides from these analyses matching the known proteome were removed, and those peptides solely mapping to our predicted ORF sequences were used to identify novel transcript-derived antigens specifically expressed and presented in primary melanoma tissue.

Confirmation of tumor cell-specific expression of antigen-encoding transcripts was carried out using RNAScope®. The ability of detected epitopes from our discovered antigens to elicit an adaptive immune response was assessed by characterization of antigen-specific T-cell responses from naïve donors.

**Results**

Our de novo assembly revealed the presence of approximately 100 melanoma-specific transcripts encoding over 2,000 potential antigens (ORFs). Interrogation of these ORFs against MS immunopeptidomic datasets mapped HLA-bound peptides to dozens of ORFs, demonstrating presentation of our novel antigens in multiple melanoma patient tissues.

RNAScope® revealed melanoma-specificity at the transcript level, with little to no transcript expression identified across normal tissues. Assessment of immunogenicity in naïve subject T cells revealed strongly reactive T cells that were able to kill peptide-pulsed APCs, indicating a lack of central-tolerance deletion of T cells specific for these ERV-derived peptides.

**Conclusions**

We have identified a number of novel melanoma-specific antigens that are shared among patients. T cells reactive for these antigens can be detected in naïve subjects, and thus these antigens show promise as candidates for development of off-the-shelf cancer vaccine-based immunotherapies.

**Acknowledgements**

The results presented here are based in part upon data generated by The Cancer Genome Atlas (TCGA) Research Network (http://cancergenome.nih.gov/); and the Genotype-Tissue Expression (GTEx) Project (supported by the Common Fund of the Office of the Director of the National Institutes of Health, and by NCI, NHGRI, NHLBI, NIDA, NIMH, and NINDS). Primary patient material utilized in this study was provided by the Cambridge University Hospitals NHS Trust Human Research Tissue Bank.

**Ethics Approval**

All work involving the use of human tissue was approved by the NHS Health Research Authority South Central - Oxford A Research Ethics Committee (reference number 04/Q1604/21), and North West - Haydock Research Ethics Committee (reference number 19/NW/0216).

#### P681 DNA methyltransferase inhibition immunosensitizes diffuse intrinsic pontine gliomas to peptide vaccine through augmentation of EphA2 and MHC I expression

##### Rajeev Kumar^1^, Lauren McCarl^1^, Alexandra Foster^1^, Shubhanchi Nigam^1^, Xinjuan Ma^1^, Marissa Campagna^1^, Alberto Broniscer^1^, Katherine Warren^2^, Ian Pollack^1^, Gary Kohanbash^1^

###### ^1^University of Pittsburgh, Pittsburgh, PA, United States; ^2^National Cancer Institute, Bethesda, MD, United States

####### **Correspondence:** Gary Kohanbash (gary.kohanbash2@chp.edu)

**Background**

Diffuse intrinsic pontine glioma (DIPG) is one of the most lethal pediatric brain tumors. DIPG is insensitive to chemotherapy and surgically inaccessible, creating an urgent need for novel therapeutic approaches. We have been evaluating peptide vaccine immunotherapies that target glioma-associated antigens (GAAs). Enhancing the expression of these immunogenic GAAs and MHC I on tumor cells may promote immune-mediated tumor recognition and killing following peptide vaccine immunotherapy. DNA methyltransferase (DNMT) inhibitors have been shown to augment the expression of MHC, tumor antigens, and other immunosensitizing molecules. Guadecitabine (SGI-110), a next-generation DNMT inhibitor, has been developed to prolong tumor cell exposure to its active metabolite, decitabine. In this study, we investigated whether SGI-110 can immunosensitize glioma cells to peptide vaccine immunotherapy by enhancing their surface expression of MHC I and a GAA, EphA2. [1-5]

**Methods**

We developed a novel C57BL/6-syngeneic DIPG model by culturing cells from a Sleeping Beauty de novo DIPG induced in a neonatal mouse using a K27M-mutated histone 3.3 plasmid and other oncogenic plasmids. Murine SB-DIPG-11 and human DIPG cell lines were treated with SGI-110 for 5 days. For vaccination, mice were intradermally injected with 100μg each of HBV core128-140 (MHC-II, lab restricted) and the GAA peptides hgp10025-33, mEphA2671-679, and mEphA2682-689, emulsified in 40μg of poly(I:C) and IFA. Control mice received HBV core128-140, also emulsified in poly(I:C) and IFA. Five days after tumor injection, mice received 2.0 mg/kg/day of SGI-110 for 6 days.

**Results**

Flow cytometry analysis showed that SB-DIPG-11 cells express both MHC I (H-2Kb/H-2Db) and EphA2 on their surface. In vitro, treatment of SB-DIPG-11 or C57BL/6-syngeneic GL261 cells with SGI-110 resulted in a dose-dependent increase of MHC I and EphA2 surface expression. In vivo, subcutaneous administration of SGI-110 in combination with an EphA2-targeted peptide vaccine significantly prolonged the survival of mice bearing orthotopic DIPG-like tumors compared with the control treatment. Stable EphA2 knockdown reverted the survival benefit of SGI-110 in combination with peptide vaccine. Analysis of dissociated tumor tissue by flow cytometry showed elevated levels of MHC I and EphA2 in mice receiving SGI-110 or SGI-110 and peptide vaccine.

**Conclusions**

Compared to control, combination therapy proved the most effective, significantly prolonging the survival of tumor-bearing mice. Based on these data, we have begun evaluating whether subcutaneous or intracerebroventricular administration of the SGI-110 is more effective in combination with peptide vaccine. Overall, SGI-110 may sensitize children with DIPG to peptide vaccine immunotherapy.

**Acknowledgements**

his research was supported by funding from the Pediatric Brain Tumor Foundation’s Project “All In” for DIPG, St. Baldrick’s Foundation, the National Cancer Institute, the National Institute for Biomedical Imaging and Bioengineering, the Pittsburgh Foundation’s Copeland Fund, and UPMC Children’s Hospital of Pittsburgh’s Scientific Program.

**References**

1. Hargrave, D., Bartels, U., Bouffet, E. Diffuse brainstem glioma in children: critical review of clinical trials. Lancet Oncol 2006; 7: 241-248.

2. Long, W., Yi, Y., Chen, S., Cao, Q., Zhao, W., Liu, Q. Potential New Therapies for Pediatric Diffuse Intrinsic Pontine Glioma. Front Pharmacol

2017; 8: 1-13.

3. Griffiths, E.A., Choy, G., Redkar, S., Taverna, P., Azab, M., Karpf, A.R. SGI-110: DNA Methyltransferase Inhibitor Oncolytic. Drugs Future 2013;

38: 535-543.

4. Srivastava, P., et al. Immunomodulatory action of the DNA methyltransferase inhibitor SGI-110 in epithelial ovarian cancer cells and xenografts.

Epigenetics 2015; 10: 237-246.

5. Wiesner, S.M., et al. De Novo Induction of Genetically Engineered Brain Tumors In Mice Using Plasmid DNA. Cancer Res 2009; 69: 431-439.

**Ethics Approval**

The study was approved by University of Pittsburgh Ethics Board.

#### P682 AI-augmented design of effective therapeutic cancer vaccines and adoptive cell therapies

##### Piotr Stepniak , Giovanni Mazzocco, MSc, Alexander Myronov, MSc, Iga Niemiec, MSc, Katarzyna Gruba, MSc, Piotr Skoczylas, MSc, Anna Sanecka-Duin, PhD, Michał Drwal, MSc, Jan Kaczmarczyk, PhD,

###### Ardigen S.A., Krakow, Poland

####### **Correspondence:** Piotr Stepniak (piotr.stepniak@ardigen.com)

**Background**

The strong involvement of neoantigens-driven CD8+ T-cells is considered a substantial element of immune-mediated tumor rejection. Neoantigens are, in fact, not subject to central immune tolerance and may constitute ideal agents for eliciting tumor-specific immune responses. For these reasons, neoantigens are rapidly gaining interest [1] as central components of personalized cancer vaccines (PCVs), as shown by the number of PCV clinical trials. One of the challenges in the design of PCVs is that only a small fraction of potential neoantigens arising from somatic mutations are immunogenic [2]. Several aspects including highly individual antigenic landscape, restricted number of targetable neoantigens per tumor, complex tumor subclonal structure, private neoantigen-specific T-cell repertoire, presence of inhibitory neoantigens, immune-escape mechanisms, etc. make the prediction of effective neoantigens a particularly challenging task.

Standard methods for the selection of neoantigens are typically based on peptide-HLA (pHLA) binding prediction algorithms. Advances in pHLA elution and mass spectroscopy [3] make it possible to identify pHLAs presented on the cell surface, which drives the development of algorithms for the prediction of the natural neoantigen presentation [4, 5]. Combining aspects such as pHLA binding affinity, neoepitope presentation, gene expression, etc. for the prediction of neoepitopes’ immunogenicity is essential for the design of therapeutic cancer vaccines and adoptive cell therapies.

**Methods**

We perform a benchmarking study of the available methods for vaccine design. To this end, we carry out a retrospective analysis on selected datasets [2] comprising WES and RNA-seq measurements, as well as validation of CD8+ response. We benchmark our approach - ArdImmuneVax - against other available methods.

**Results**

For each of the vaccine design tools, we assess the enrichment of immunogenic neoepitopes within the suggested vaccine compositions (e.g., top 20 peptides) using early-enrichment metrics [4]. We analyze the results in an indication-specific fashion in order to assess whether there are significant differences among cancer types. Finally, for each vaccine design method, we assess additional important properties of the resulting target neoepitopes, such as toxicity, tolerance, and peptides’ manufacturability.

**Conclusions**

The presented work provides insights into the vaccine design process by quantitatively and qualitatively comparing available tools for the selection of both cancer vaccine composition and targets for adoptive cell therapies.

**References**

1. Garcia-Garijo A, Fajardo CA, Gros A, Determinants for Neoantigen Identification. Front. Immunol. 2019;10:1392.

2. Parkhurst MR, et al., Unique Neoantigens Arise from Somatic Mutations in Patients with Gastrointestinal Cancers. Cancer Discov. 2019;9:1-14

3. Abelin JG, et al., Mass Spectrometry Profiling of HLA-Associated Peptidomes in Mono-allelic Cells Enables More Accurate Epitope Prediction. Immunity. 2017;46(2):315-326

4. Bulik-Sullivan B, et al., Deep learning using tumor HLA peptide mass spectrometry datasets improves neoantigen identification. Nat. Biotechnol. 2019;37:55-63

5. Zeng H, Gifford DK, DeepLigand: accurate prediction of MHC class I ligands using peptide embedding. Bioinformatics. 2019;35:i278-i283.

#### P683 Incomplete Freund’s adjuvant reduces arginase, enhances a Th1 dominant cytokine environment, and supports CD40 L expression

##### Karlyn Pollack, MD, Max Meneveau, MD, Alexander Koeppel, Samuel Young, Stephen Turner, Katia Sol-Church, MD, Ileana Mauldin, PhD, Craig Slingluff, MD

###### University of Virginia, Charlottesville, VA, United States

####### **Correspondence:** Craig Slingluff (CLS8H@hscmail.mcc.virginia.edu)

**Background**

Immunogenicity of cancer vaccines is impacted by adjuvants and schedule, but systematic assessments of their effects have not been performed. Montanide ISA-51, an incomplete Freund’s adjuvant (IFA), is used in many vaccine trials, but concerns have been raised about negative effects in murine studies [1, 2]. However, we found in humans that IFA enhances systemic immune responses over a toll-like receptor (TLR) agonist alone [3] and that repeat vaccination at one site (same site vaccination, SSV) creates tertiary lymphoid structures (TLS) in the vaccine site microenvironment (VSME) [4]. We hypothesized that one vaccine with peptides +IFA +pICLC or SSV x 3 with peptides in IFA will create an immunogenic milieu locally at the VSME, with activated and mature dendritic cells (DC), TLS-associated chemokines, and a Th1-dominant VSME.

**Methods**

Biopsies of the VSME were obtained from participants on two clinical trials (MEL48, NCT00705640; MEL58, NCT01585350) who were immunized with multiple melanoma peptides (MELITAC 12.1) in adjuvants comprised of IFA and/or the toll-like receptor (TLR)3-agonist pICLC. Biopsies were obtained either a week after one vaccine or a week after SSV x 3. Controls included normal skin and skin injected with IFA without peptides. Gene expression analysis was performed by RNAseq. Multiparameter immunofluorescence histology (mIF) was performed for selected markers.

**Results**

VSME samples were evaluated from 27 patients. One vaccine with peptides in pICLC + IFA enhanced expression of CD80, CD83, CD86 (p<0.01), CD40 and CD40L (p<0.0001) over normal skin; these effects were significantly enhanced for SSV with peptides + IFA (Figure 1). CD40L protein expression by CD4 T cells was confirmed by mIF (Figure 2). Vaccines containing pICLC increased expression of TBX21 (T-bet) but did not decrease GATA3 over normal skin, whereas SSV with peptides in IFA dramatically enhanced TBX21 and decreased GATA3, with high expression of IFNγ and STAT1 (Figure 3). SSV with peptides in IFA also dramatically reduced arginase-1 (ARG1) expression and enhanced expression of TLR adapter molecules TICAM-1 (TRIF) and MYD88 (Figure 4). Furthermore, SSV with IFA and peptides also enhanced expression of chemokines associated with TLS formation (not shown).

**Conclusions**

These findings suggest that SSV with peptides in IFA enhances CD40L expression by CD4 T cells, supports a Th1 microenvironment, with accumulation of activated and mature DC. Increased expression of TLR adaptor proteins after SSV with peptides in IFA might implicate effects of the skin microbiome. Reduced ARG1 may reflect diminished suppressive myeloid activity in the VSME.

**Trial Registration**

Samples from two clinical trials were used, including MEL48, NCT00705640; MEL58, NCT01585350.

**References**

1. Hailemichael, Y., et al., Cancer vaccine formulation dictates synergy with CTLA-4 and PD-L1 checkpoint blockade therapy. J Clin Invest, 2018. 128(4): p. 1338-1354.

2. Hailemichael, Y., et al., Persistent antigen at vaccination sites induces tumor-specific CD8(+) T cell sequestration, dysfunction and deletion. Nat. Med, 2013. 19(4): p. 465-472.

3. Melssen, M.M., et al., A multipeptide vaccine plus toll-like receptor agonists LPS or polyICLC in combination with incomplete Freund's adjuvant in melanoma patients. J Immunother Cancer, 2019. 7(1): p. 163.

4. Harris, R.C., et al., The vaccine-site microenvironment induced by injection of incomplete Freund's adjuvant, with or without melanoma peptides. J Immunother, 2012. 35(1): p. 78-88.

**Ethics Approval**

Patients were studied following informed consent, and with Institutional Board Review (IRB) (HSR-IRB 13498 and 15781, respectively) and FDA approval.

**Consent**

Written informed consent was obtained from patients for publication of this abstract and any accompanying images. A copy of the written consent is available for review by the Editor of this journal

**Fig. 1 (abstract P683).**
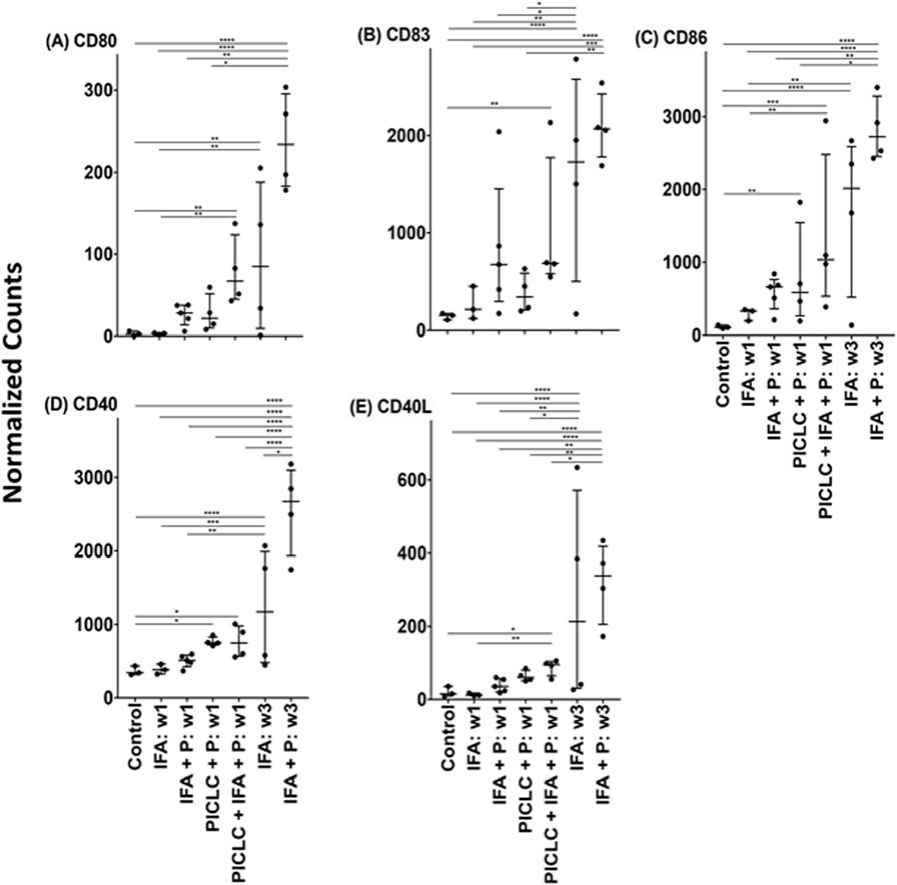
See text for description

**Fig. 2 (abstract P683).**
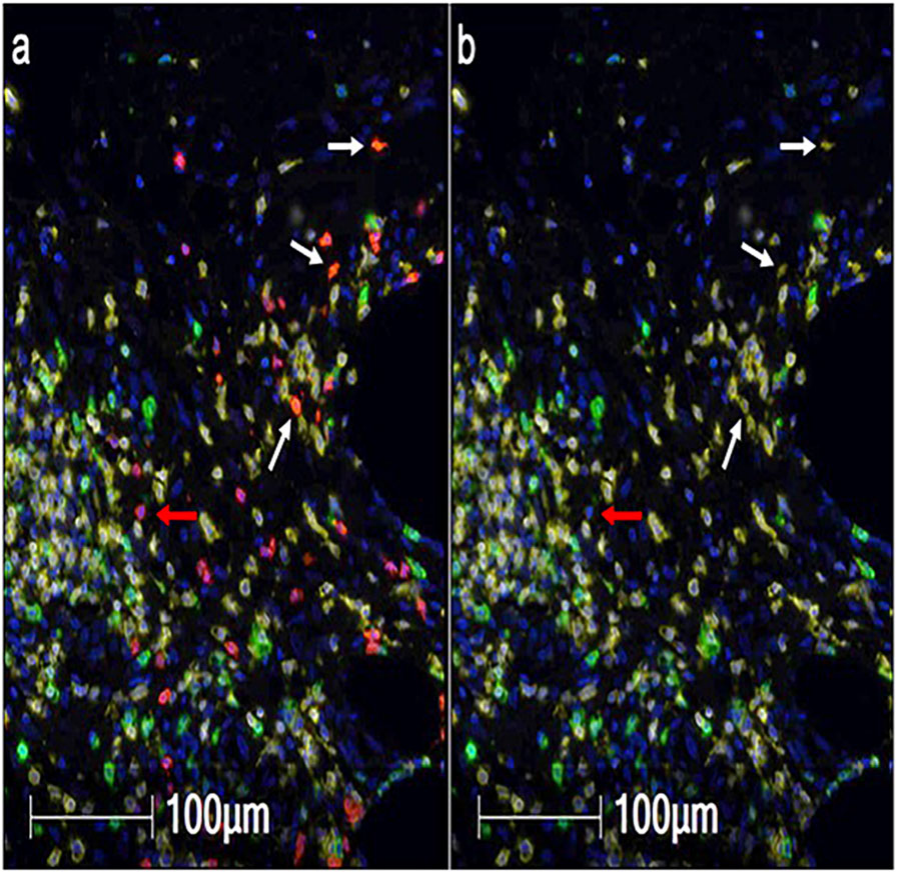
See text for description

**Fig. 3 (abstract P683).**
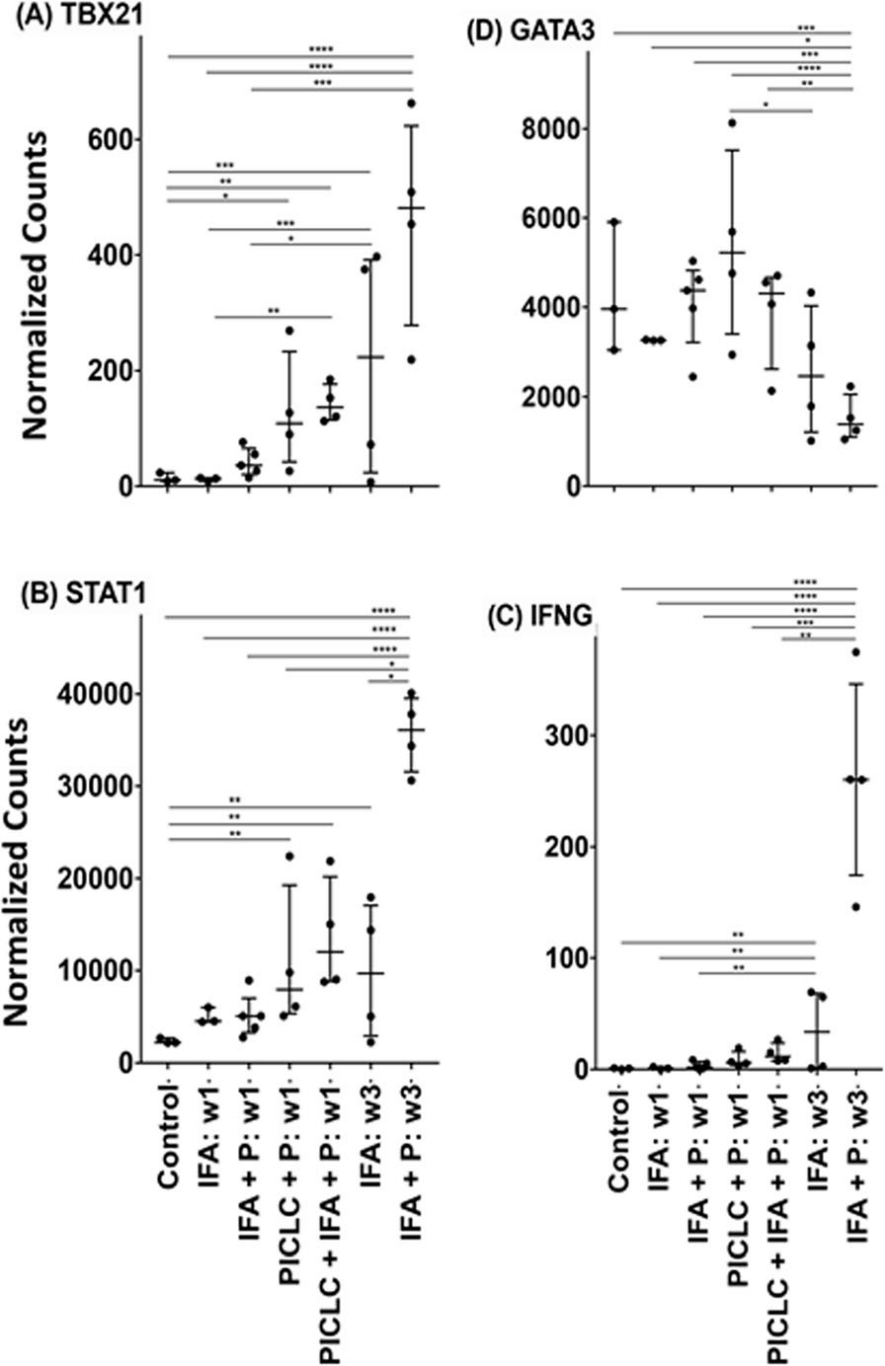
See text for description

**Fig. 4 (abstract P683).**
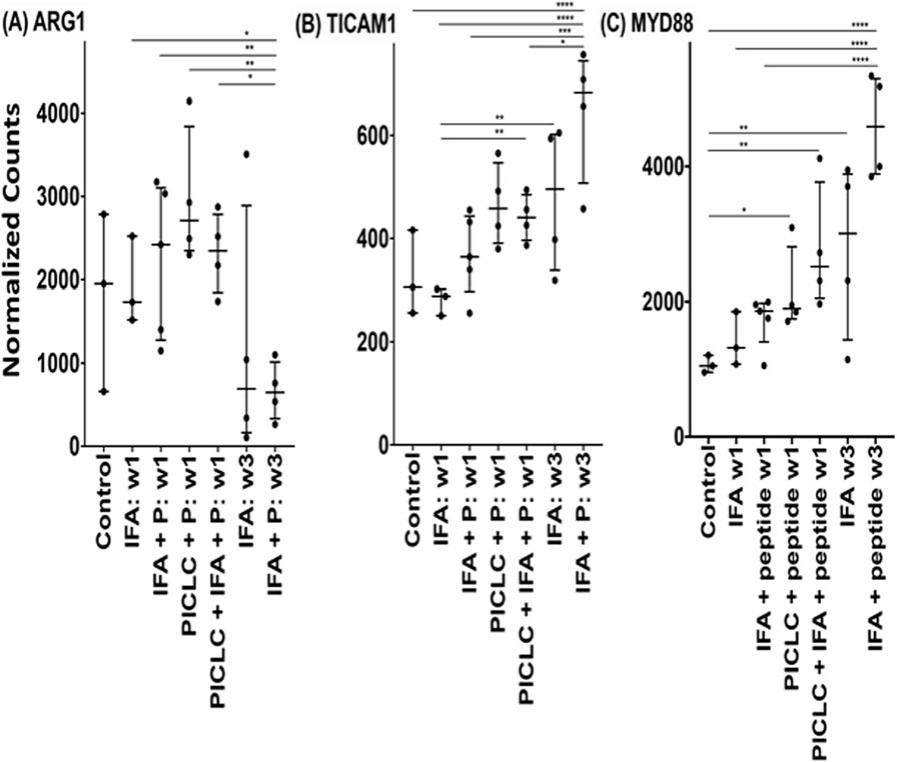
See text for description

#### P684 Highly efficient selection of tumor neoantigens improves therapeutic cancer vaccine efficacy

##### Guilhem Richard, PhD^1^, Gad Berdugo, MSc, MBA^1^ , Michael Princiotta, MS, PhD^1^, Leonard Moise, PhD^2^, Matthew Ardito, BA^2^, Christine Boyle^2^, Dominique Bridon^1^, William Martin, BA MD^2^, Anne de Groot, MD^2^

###### ^1^EpiVax Oncology, Providence, RI, United States; ^*2*^*EpiVax, Inc., Providence, RI*

####### **Correspondence:** Gad Berdugo (gberdugo@epivaxonco.com)

**Background**

Recent advances in whole exome sequencing have produced a renewed interest in therapeutic cancer vaccines targeting tumor-specific neoantigens. A central challenge to the development of successful therapeutic cancer vaccines is the identification of neoantigen sequences that elicit effector T cell responses able to control tumor growth while avoiding the induction of inhibitory immune pathways. In the study reported here, we present data highlighting the efficiency of neoantigen selection using the innovative Ancer™ screening platform and demonstrate antigen-specific responses and tumor control in a mouse tumor model.

**Methods**

The CT26 mutanome was evaluated with Ancer™, an innovative and automated neoantigen prediction platform that combines proprietary machine learning-based MHC class I and MHC class II neo-epitope identification tools with removal of inhibitory regulatory T cell (Treg) epitopes for optimal personalized cancer vaccine design. MHC class I- and MHC class II-restricted CT26 neoantigens, devoid of putative Treg epitopes, were ranked according to their immunogenic potential and tumor expression level. The immunogenicity and efficacy of the 20 highest Ancer-ranked neoantigens, delivered subcutaneously as a peptide pool with poly-ICLC (Oncovir) as adjuvant was evaluated in naïve and CT26 tumor-bearing BALB/c mice.

**Results**

T cell responses to individual peptide neoantigens were determined using ELISpot assay for IFNγ production. While around 20% of neoantigen sequences identified in studies using commonly available predictive algorithms are immunogenic, we found that up to 65% (13 out of 20) of the computationally predicted peptide sequences included in the CT26-Ancer™ vaccine were immunogenic (Figure 1). Responding T cells were both CD8+ and CD4+ and were shown to be multi-functional by flow cytometric analysis for IFNγ, TNFα and IL-2 production.

Tumor control was demonstrated by immunizing BALB/c mice implanted with syngeneic CT26 tumor cells with the CT26-Ancer™ vaccine. Average tumor volumes in CT26-Ancer™ immunized mice were significantly reduced by 45% (unpaired t test, p = 0.0156) and 38% (unpaired t test, p = 0.0291) at days 21 and 25 post implantation, respectively, compared to vehicle (poly-ICLC) immunized tumor-bearing animals (Figure 2).

**Conclusions**

The results presented here highlight the efficiency of the Ancer™ platform in selecting highly immunogenic neoantigen targets and that these responses are able to control tumor growth in a peptide formulation. Using advanced in silico methods to select antigenic targets for use in therapeutic cancer vaccines promises to improve the efficacy of tumor immunotherapy. While confined to mice, these studies further validate our personalized cancer vaccine process for future human clinical studies.

**Fig. 1 (abstract P684).**
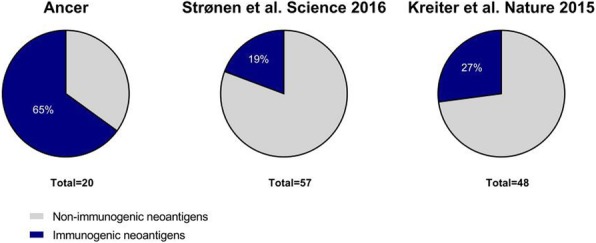
See text for description

**Fig. 2 (abstract P684).**
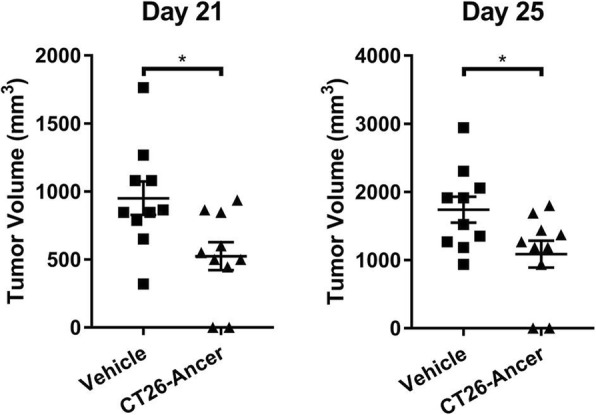
See text for description

#### P685 Inhibition of EZH2 enhances tumor immunogenicity through diversification of tumor antigen presentation

##### Jing Qiu, PhD, , Thomas Paul

###### Pfizer, San Diego, CA, United States

####### **Correspondence:** Thomas Paul (Thomas.A.Paul@pfizer.com)

**Background**

A central challenge to elicit effective anti-tumor T cell responses remains limited expression of productive tumor antigens or antigen presentation machinery. During immune editing, cancers utilize epigenetic silencing of cancer neo-antigens as well as components of the antigen presentation machinery to escape immunosurveillance[1]. As a result, therapeutic approaches that have the potential to reactive epigenetically silenced genes that enhance tumor immunogenicity are attractive drug combinations with checkpoint blockade[2].

EZH2 is an epigenetic modifier responsible for H3K27 histone methylation associated with the silencing of gene transcription. In cancer, dysregulation of EZH2 results in the redistribution of H3K27me3 to the promoters of genes involved in the regulation of cell cycle, cell survival, and cellular differentiation. Emerging studies have provided evidence for an additional role of EZH2 dysregulation in the transcriptional repression of genes involved in tumor immune pathways.

**Methods**

ChIP-seq, RNA-seq, and exome-seq was conducted on murine tumor cell lines treated with the EZH2 inhibitor PF-06821497. Peptides with mutations that are upregulated by EZH2 inhibitor treatment were computationally selected based on their predicted binding affinity to the corresponding MHC class I and class II expressed in each cell line. Presentation of peptide candidates were further validated by immunopeptidomics. Predicted peptides were synthesized and tested in pools for their ability to elicit antigen-specific T cell responses.

**Results**

In this study, we have identified a common mechanism of EZH2-mediated epigenetic suppression of genes involved in tumor immune recognition. Using epigenomic profiling, we demonstrate the involvement of EZH2 in the repression of genes involved in tumor antigen presentation including MHC class I and class II and numerous genes harboring mutations with potential to serve as neoantigens. Inhibition of EZH2 combined with IFN stimulation results in reduced H3K27me3 and transcriptional up-regulation of the MHC class II transactivator-CIITA and MHC class II genes. Moreover, by employing computational prediction tools, we observed a cluster of genes with somatic mutations that are transcriptionally re-activated by EZH2 inhibition. The re-expression of these genes is predicted to represent over 100 potential neoantigens with high binding affinity to MHC class I and class II molecules.

**Conclusions**

Our data illustrate the potential for targeting EZH2 to increase the immunogenicity of tumors through relieving epigenetic suppression of tumor antigen presentation machinery and silenced neoantigens. As EZH2 inhibitors are currently under clinical investigation, targeting EZH2 represents a promising combination approach with checkpoint inhibitors to elicit responses in poorly immunogenic tumors.

**References**

1. Schreiber RD, Old LJ, Smyth MJ. Cancer immunoediting: integrating immunity's roles in cancer suppression and promotion. Science. 2011; 331:1565-70.

2. Havel JJ, Chowell D, Chan TA. The evolving landscape of biomarkers for checkpoint inhibitor immunotherapy. Nat Rev Cancer. 2019; 19(3):133-150.

#### P686 The NCI PREVENT Program portfolio for precision cancer immunoprevention

##### Shizuko Sei, MD^1^, Shizuko Sei, MD^2^ , Shizuko Sei, MD^2^, Altaf Mohammed, PhD^2^, Jennifer Fox, PhD^2^, Romaine Fernando, PhD^2^, Daniel Boring, RPh, PhD^2^, Mark Miller, PhD^2^, Robert Shoemaker, PhD^2^

###### ^1^National Cancer Institute (NCI), Bethesda, MD, United States; ^*2*^*NCI, Bethesda, MD, United States*

####### **Correspondence:** Shizuko Sei (seis@mail.nih.gov)

**Background**

The NCI PREVENT Cancer Preclinical Drug Development Program (PREVENT) is a peer-reviewed program that supports the preclinical development of innovative cancer preventive interventions towards clinical applications (https://prevention.cancer.gov/major-programs/prevent-cancer-preclinical). One of the Program’s current focus areas is cancer immunoprevention. Recent advances in cancer immunotherapies have clearly demonstrated that the immune system can mount effective antitumor immune responses, if tumor-associated immunosuppression is overcome, for example, by immune checkpoint blockade. It is conceivable that cancer vaccines can arm the host with antitumor immunity that intercepts, arrests, and/or reverses tumorigenesis if delivered before or early in the tumorigenic process. PREVENT seeks to develop effective vaccines for precision cancer immunoprevention in high-risk groups in partnership with principal investigators (PIs) with innovative ideas.

**Methods**

PREVENT utilizes NCI contract resources to operate its agent development pipeline, which consists of three key stages: early proof of concept phase, secondary testing phase, and advanced preclinical development phase. Competitively selected contractor pools with necessary technical expertise are in place to implement approved projects under NCI oversight. Data and materials generated through PREVENT are returned to the applicant PIs for further agent development. A wide array of organ-specific preclinical cancer models is available to assess agent efficacy and toxicity.

**Results**

Since the Program’s inception, a total of 21 cancer preventive vaccine projects have been selected for support. Of those, two have progressed to the clinical trial stage with two additional projects in the advanced development phase. The PREVENT immunoprevention portfolio consists of a variety of vaccination approaches that target oncogenic drivers, early tumor associated antigens, and infectious agents known to induce cancers. These vaccines are developed for clinically identifiable high-risk cohorts, such as genetically predisposed populations (individuals with Lynch syndrome, BRCA1/2 mutations, and familial adenomatous polyposis, etc.) and those diagnosed with pre-cancerous lesions.

**Conclusions**

While the expanded knowledge base in tumor immunology has uncovered the potential hurdles for developing effective cancer immunoprevention strategies, it has also helped better understand the key determinants, including the selection of optimal target antigens, vaccine delivery strategies and schedules, immune biomarkers for vaccine immunogenicity, immune durability, and immune correlates of antitumor protection. Learning from and building on the success of prophylactic human papillomavirus vaccines in preventing cervical cancer, PREVENT offers partnership opportunities to researchers interested in developing novel cancer preventive vaccines for oncogenic pathogen-induced as well as cancers not linked to pathogens toward clinical applications.

#### P687 Novel vaccination strategies using tumour-independent antigens to induce anti-tumour immunity in solid tumours

##### Vinod Sommandas, Satwinder Singh, Erik Manting, Satwinder K. Singh

###### DCPrime b.v., Leiden, Netherlands

####### **Correspondence:** Satwinder K. Singh (s.singh@dcprime.com)

**Background**

Induction of optimal anti-tumour immune response is challenging as tumour antigens are self-antigens and antigen-specific receptors against self-antigens are negatively selected in the body. Therefore, anti-tumour immune responses that are present in the body harbour low affinity antigen-specific receptors that will not optimally eradicate tumours. The new insights on tumour-specific antigens (TSA) has led to personalised vaccines. However, not every tumour type has high mutation burden resulting in TSA and thus these tumour types cannot rely on these strategies. Also TSA are patient-specific and the broad application in cancer patients is limited. In this study we present a novel vaccination strategy which uses foreign (non-self) antigens to mount robust immune responses enabling solid tumour eradication. This strategy is based on two key elements: to induce immunity against a foreign antigen, and to mark the tumour as a target for the induced immune response with the same foreign antigen. To proof the concept, we selected keyhole limpet hemocyanin (KLH) as a foreign immunogenic neo-antigen which will serve both as vaccine composition and tumour marker to elicit effective anti-tumour immune response.

**Methods**

As solid tumour models, A375 melanoma or U87-MG glioblastoma cells were subcutaneously engrafted into CD34 humanised NCG. Mice received intraperitoneal (i.p.) vaccination with KLH. The tumour marking was done intratumourally (i.t.) by using DCOne® mDC loaded with KLH protein. Tumour reduction and immune responses induced against KLH were evaluated.

**Results**

We observed reduced tumour growth in both A375 and U87-MG tumour models in KLH vaccinated mice where KLH served as vaccine and i.t. injection of DCOne® mDC loaded with KLH protein for tumour marking compared to the mice injected with PBS. We observed that both in U87-MG and A375 mice, there was no significant difference regarding anti-KLH IgM concentration between treated groups and the PBS control group over time. Noteworthy, the mice from the KLH/KLH loaded DCOne® treated group produced significantly more anti-KLH IgG than the PBS control group at sacrifice.

**Conclusions**

These findings support the new concept that tumour independent antigens can be used to develop vaccines against solid tumours. Importantly, DCOne® cells can be used as a carrier for intratumoural delivery of foreign proteins.

#### P688 Lysosomal-associated membrane protein-1-targeting of a poly-neoepitope DNA vaccine elicits potent immune responses and inhibits tumor growth

##### Pratima Sinha, PhD^1^, Guilhem Richard^3^, Matthew Ardito, BA^4^, Leonard Moise, PhD^5^, Gad Berdugo, MSc, MBA^3^, Teri Heiland^2^

###### ^*1*^*Immunomic Therapeutics, Inc., Rockville, MD, United States;*^*2*^*Immunomic Therapeutics Inc., Rockville, MD, United States* ; ^*3*^*EpiVax Oncology, Inc., New York, United States* ; ^*4*^*EpiVax, Inc, Providence, RI, United States* ; ^*5*^*Institute for Immunology and Informatics, Providence, RI, United States*

####### **Correspondence:** Teri Heiland (THeiland@immunomix.com)

**Background**

Cancer vaccines have traditionally succeeded in preventing viral-induced malignancies, such as papilloma virus-associated cervical cancer and hepatitis B-associated hepatocellular carcinoma. Conceptually, tumor-specific protein coding mutations (neoantigens) are ideal targets for cancer immunotherapy. Neoantigens are not expressed in healthy tissues and therefore are exempt from central tolerance and can potentially be recognized by T cells to facilitate tumor rejection. However, every patient’s tumor possesses a unique set of mutations that must first be screened for optimal target selection, presenting a challenge for neoantigen vaccine development. We present here the merging of two innovative and complementary approaches for identifying neoantigens and delivering personalized cancer vaccines.

**Methods**

The CT26 mutanome was evaluated with Ancer™, an innovative and automated neoepitope prediction platform that combines proprietary machine learning-based MHC class I and MHC class II neoepitope identification tools with removal of inhibitory regulatory T cell (Treg) epitopes for optimal personalized cancer vaccine design. MHC class I- and MHC class II-restricted CT26 neoepitopes, devoid of putative Treg epitopes, were ranked according to their immunogenic potential and tumor expression level. The twenty most highly Ancer-ranked neoepitopes were subsequently introduced as a “string of beads” DNA vaccine into the UNITE (UNiversal Intracellular Targeted Expression) platform (poly-neoepitope UNITE vaccine). The UNITE platform is based in part on lysosomal targeting technology which results in enhanced antigen presentation and a balanced T cell response.

**Results**

We report that prophylactic vaccination with poly-neoepitope UNITE vaccine successfully induced IFNγ-producing Th1 cells, with complete rejection of CT26 tumors observed in 50% of mice. Mice rejecting tumors were also protected from rechallenge with CT26, demonstrating that effective antigen-specific memory was induced in these animals. In therapeutic vaccination studies, we observed CT26 tumor growth inhibition in 46% of animals immunized with the poly-neoepitope UNITE vaccine, as well as significantly prolonged survival, as compared to animals immunized with the control vector.

**Conclusions**

Therefore, targeting multiple mutations encoding the best set of CD4 and CD8 neoepitopes, as predicted by Ancer™, and using the UNITE platform may solve critical problems in current cancer immunotherapy development. Follow-up studies will include evaluation of the poly-neoepitope UNITE vaccine in combination with checkpoint inhibitors.

#### P689 Amphiphile-modifications target mKRAS-antigen and adjuvant to lymph nodes and enable polyfunctional mKRAS-specific immune responses with potent cytolytic activity

##### Martin Steinbuck, Peter DeMuth, PhD , Lochana Seenappa, MS

###### Elicio Therapeutics, Cambridge, MA, United States

####### **Correspondence:** Peter DeMuth (Pete.DeMuth@Elicio.com)

**Background**

Subunit vaccines targeting tumor antigens have shown limited capacity for expanding cytotoxic T-cells against tumors in the clinic. Especially in the case of KRAS-driven tumors, responses elicited by conventional vaccines have been exceedingly weak. For molecular immunogens including peptides and oligonucleotides, inefficient delivery to immune cells residing in the lymphatics is a significant challenge limiting their ability to induce cancer-directed immune responses of sufficient strength and functionality to impact tumors. Improving the targeting of immunogens to lymph nodes (LN), where resident immune cells potently orchestrate immunity, can substantially amplify their ability to induce effective tumor-directed immunity. Here, we demonstrate such an approach for significantly enhancing mKRAS-directed T-cell responses by precisely targeting antigens and adjuvants directly to the draining LN through a simple one-step conjugation to albumin-binding lipids. These amphiphilic conjugates (“Amphiphiles”) then ‘hitch-hike’ on albumin into the LNs where they elicit strong immune responses. LN accumulation of structurally optimized amphiphiles in mice is greatly improved over soluble equivalents.

**Methods**

C57BL/6J mice received two or more doses of benchmark or amphiphile-modified vaccines, comprised of mKRAS peptide and CpG adjuvant, subcutaneously injected into the tail base in two-week intervals. Immunological readouts were performed 7 days post dosing. For ELISpot analysis of IFNγ and Granzyme B production and flowcytometric bead array analysis of Th1/2 cytokines, splenocytes were harvested and re-stimulated with antigen overnight. In vivo, cytolytic capabilities of antigen-specific T-cells were evaluated by pulsing CFSE-stained splenocytes from naïve mice with mKRAS antigen and injecting these cells intravenously into immunized mice. Recovery of CFSE-labeled target cells from immunized mice was performed 24h later and analyzed flowcytometrically.

**Results**

We show robust immune responses that yield strong activation against all common mutations in the mKRAS protein compared to low or undetectable responses generated by soluble or benchmark treatments. Further, this response is composed of CD4+ as well as CD8+ T-cells resulting in the production of high levels of TH1-associated cytokines upon re-stimulation with mKRAS-specific peptides in vitro. In vivo, robust cytolytic function towards mKRAS-presenting targets can be measured in T-cells.

**Conclusions**

By targeting immunogens directly and precisely to the LNs, the Amphiphile platform can significantly amplify the potency of subunit vaccines. In the case of mKRAS, substantially improved cytolytic immune responses represent a promising therapeutic strategy for targeting mKRAS-driven tumor growth and survival in a large fraction of human tumors. Furthermore, this platform technology is simple, rapid and scalable for broad clinical application.

#### P690 Improving the immunogenicity of modified vaccinia virus Ankara (MVA) vaccine vector by deletion of the vaccinia E5R gene encoding a dominant cGAS inhibitor

##### Liang Deng, PhD, MD, Ning Yang, Yi Wang, MS, Peihong Dai, Stewart Shuman, MD PhD

###### Memorial Sloan Kettering Cancer Center, New York, NY, United States

####### **Correspondence:** Liang Deng (dengl@mskcc.org)

**Background**

Modified vaccinia virus Ankara (MVA) is a highly attenuated vaccinia strain that belongs to the poxvirus family. MVA is a promising vaccine vector for infectious diseases and cancers due to its immunogenicity, safety, and a large capacity to accommodate insertions of transgenes. However, MVA has certain disadvantages that need to be overcome to improve its immunogenicity and vaccine efficacy. Those include suboptimal induction of type I IFN in dendritic cell (DC) subsets, presumably due to the expression of a viral inhibitory protein(s) for the cGAS/STING-mediated cytosolic DNA-sensing pathway, as well as the induction of cell death in infected DCs [1,2].

**Methods**

To identify vaccinia viral inhibitors of the cGAS/STING pathway, we performed an unbiased screen of 70 vaccinia viral early genes in HEK293 T cells using a dual-luciferase system. We discovered that the vaccinia E5R gene encodes a dominant inhibitor of cGAS.

**Results**

Whereas MVA infection of bone marrow-derived DCs (BMDCs) induces cGAS degradation in a proteasome-dependent manner, recombinant MVA with deletion of the E5R gene (MVA∆E5R) infection fails to do so. MVA∆E5R infection of BMDCs induces much higher levels of cyclic GMP-AMP (cGAMP) production, IFNB gene expression, and IFN-beta protein secretion compared with live MVA or Heat-inactivated MVA. In addition to BMDCs, MVA∆E5R infection of plasmacytoid DCs (pDCs) and CD103+ DCs also activates the cGAS/STING/IFNB pathway very efficiently. Furthermore, MVA∆E5R infection of DCs triggers DC maturation and induces antigen cross-presentation by DCs in a cGAS-dependent manner. MVA∆E5R-infected DCs are much less prone to cell death compared with MVA-infected DCs, and they appear activated and healthy for more than five days in vitro cell culture. Using MVA∆E5R virus expressing mcherry under vaccinia synthetic early-late promoter, infected DCs appear to have robust transgene (mcherry) expression. We engineered a recombinant MVA∆E5R expressing chicken ovalbumin (OVA) as a model antigen. Vaccination with MVA∆E5R-OVA either through skin scarification or via intradermal injection generates stronger OVA-specific CD8+ T cell responses compared with MVA-OVA.

**Conclusions**

In this study, we discovered that the vaccinia E5R gene encodes a dominant cGAS inhibitor. Expression of E5 protein by MVA leads to proteasome-dependent degradation of cGAS. Deletion of the E5R gene from the MVA genome dramatically improves the immunogenicity of MVA, likely through stronger type I IFN induction, more robust DC maturation, and marked reduction of cell death compared with the parental virus.

**References**

1. Dai, P., W. Wang, H. Cao, F. Avogadri, L. Dai, I. Drexler, J. A. Joyce, X. D. Li, Z. Chen, T. Merghoub, S. Shuman, and L. Deng. (2014). Modified vaccinia virus Ankara triggers type I IFN production in murine conventional dendritic cells via a cGAS/STING-mediated cytosolic DNA-sensing pathway. PLoS Pathogen 10: e1003989. (PMCID: 3990710)

2. Dai, P., W. Wang, N. Yang, C. Serna-Tamayo, J.M. Ricca, D. Zamarin, S. Shuman, T. Merghoub, J.D. Wolchok, and L. Deng. (2017). Intratumoral delivery of inactivated modified vaccinia virus Ankara (iMVA) induces systemic antitumor immunity via STING and Batf3-dependent DCs. Science Immunology 2, eaal1713: 1-15.(PMCID: PMC5559204)

**Ethics Approval**

The study was approved by the Committee on the Ethics of Animal Experiments of Sloan Kettering Cancer Institute, approval number 19-01-002.

#### P691 Monoclonal microbial EDP1503 induces anti-tumor responses via gut-mediated activation of both innate and adaptive immunity

##### Humphrey Gardner, MD, Shubhra Kashyap, MS, Peter Sandy, PhD, Holly Ponichtera, PhD, Shannon Argueta, PhD, Chris Davitt, PhD, Michael Goldberg, PhD, Mark Carlson, MS, Maria Sizova, PhD, Valeria Kravitz, MS, Erin Troy, PhD, Sam Andrewes, MS, Mark Bodmer, PhD, Loise Francisco-Anderson, PhD

###### Evelo Biosciences, Cambridge, MA, United States

####### **Correspondence:** Loise Francisco-Anderson (loise@evelobio.com)

**Background**

Systemic immunity is regulated by interactions of commensal bacteria with immune cells in the gut. Enrichment of specific intestinal microbes has been shown to enhance the anti-tumor response to PD-1 blockade in both murine models and cancer patients. Here we report that oral administration of a monoclonal microbial, EDP1503, induces systemic anti-tumor immunity via the direct activation of antigen presenting cells (APCs) and natural killer (NK) cells with subsequent induction of T cell immunity.

**Methods**

The clinical efficacy of EDP1503 was investigated in subcutaneously implanted isograft tumor models as well an intravenous lung metastasis model. Mechanism of action was dissected by ex vivo analysis of the tumor microenvironment (TME) and gut-draining lymph nodes, and further interrogated by in vitro functional studies with murine and human cells.

**Results**

EDP1503 increases expression of costimulatory molecules on CD11c+ dendritic cells (DCs) within the mesenteric LNs with an accompanying increase in proinflammatory CD103+ DCs within tumor draining lymph nodes. Direct stimulation of DCs with EDP1503 results in the upregulation of the proinflammatory cytokines TNFa, IL-1B and IL-12. In addition, EDP1503 amplifies both myeloid and lymphocyte responses via production of DC-derived growth factors, M1 polarization of macrophages, and production of the lymphocyte-recruiting chemokines, CXCL9 and CXCL10. Mechanistically, EDP1503 triggers specific pattern recognition receptors, resulting in a proinflammatory signature from APCs and enhanced cytolytic activity by NK cells. Dissection of the tumor microenvironment reveals increased activated Ki67+ NK cells and CD8+ T cells producing IFNg. Moreover, in vivo, treatment of mice with EDP1503 results in decreased tumor volume, delayed tumor growth and reduction in the number of metastatic foci in a CD8T and NK dependent manner. The prominent anti-tumor effects of EDP1503 are further augmented by combination with anti-PD-1 neutralizing Abs.

**Conclusions**

Together, these data clearly demonstrate the ability of an orally delivered non-colonizing monoclonal microbe to enhance innate and adaptive anti-tumor immunity and substantiates the rationale for ongoing clinical trials. EDP1503 is currently in Phase 1b/2 studies (NCT03775850; NCT03595683) with enrollment open at multiple sites.

#### P692 Blockade of PGE2 production and signaling enhances the magnitude and selectivity of BCG-promoted immune responses in the human bladder cancer microenvironment

##### Omar Ibrahim, BPharm, Melissa Grimm, PhD, Weijan Jiang, Khurshid Guru, Gurkamal Chatta, MD, Pawel Kalinski, MD, PhD

###### Roswell Park Comprehensive Cancer Center, Buffalo, NY, United States

####### **Correspondence:** Pawel Kalinski (Pawel.Kalinski@roswellpark.org)

**Background**

Bacillus Calmette-Guérin (BCG) is commonly used in the treatment of non-muscle invasive urothelial cancer (bladder cancer; BlCa), but its benefit is typically transient and limited to a fraction of patients. We observed that the impact of BCG on human BlCa tissue involves undesirable induction of immunosuppressive factors and regulatory T(reg) cell- and myeloid-derived suppressor cell (MDSC)-attracting chemokines. Prompted by these observations, we evaluated the molecular pathways regulating the patterns of BCG-induced inflammation in BlCa tumor microenvironments (TME), and the feasibility of their modulation to selectively enhance local attraction and function of the desirable cytotoxic CD8+ T cells (CTLs) and counteract local MDSCs and Treg accumulation.

**Methods**

Human monocyte-derived macrophages and freshly resected human bladder cancer specimens were cultured ex vivo in the absence or presence of BCG, indomethacin (COX-1/2 blocker), celecoxib (selective COX-2 blocker) or selective EP4 blocker (ARY-007). The expression of inflammatory mediators and chemokine genes, secretion of chemokines and their cellular sources were analyzed by quantitative PCR, ELISAs and immunofluorescence microscopy. Migration of CTLs was evaluated ex vivo, using 24-transwell plates. Data were analyzed by Student’s t-test or Wilcoxon test. Spearman’s correlation is used for correlative analysis by using z-score downloaded from cBioportal.

**Results**

We observed that the BCG-driven-induction of MDSC- and Treg-attracting chemokines in human macrophages and whole BlCa explants was correlated with its induction of COX-2, the key enzyme controlling the PGE2 biosynthesis. Blockade of COX-2 activity using selective or non-selective COX-2 inhibitors (celecoxib, indomethacin) or interference with EP4-driven PGE2 signaling (using selective EP4 blocker, ARY-007) strongly elevated the production of CTL attractants (CXCL9, CXCL10 and CCL5), but suppressed the induction of MDSC/Treg attractants (CCL22, CXCL8, CXCL12), and several immunosuppressive mediators including COX-2, IL-10, NOS2 and IDO. Our functional studies demonstrated that the combination of BCG with PGE2 antagonism selectively enhances CTL attraction.

**Conclusions**

We observed that PGE2 represents a nodal point controlling the balance between desirable/immunostimulatory and undesirable/immunosuppressive components of BCG activity and that interference with PGE2 synthesis or it’s signaling improves the magnitude and character of the BCG-orchestrated immune response in BlCa TME. Our data suggest that PGE2 antagonism may be used to enhance the therapeutic effects of BCG treatment.

### Immuno-Conjugates and Chimeric Molecules

#### P693 Cooperative targeting of immunotherapy-resistant melanoma and lung cancer models by an AXL-targeting antibody-drug conjugate and immune checkpoint blockade

##### Julia Boshuizen, MD^1^, Nora Pencheva^2^, Oscar Krijgsman, PhD^1^, Maarten Janmaat^2^, Patricia Garrido Castro^2^, Elke Gresnigt-Van den Heuvel^2^, Andreas Lingnau^2^, Maria Jure-Kunkel^2^, Daniel Peeper, PhD^1^, Daniel Peeper, PhD^1^

###### ^1^Netherlands Cancer Institute, Amsterdam, Netherlands; ^2^Genmab,Utrecht, Netherlands

####### **Correspondence:** Daniel Peeper (d.peeper@nki.nl)

**Background**

Immune checkpoint blocking therapies (ICB) such as anti-PD1 antibodies have revolutionized the treatment of melanoma and lung cancer, leading to clinical benefit in approximately 50% and 19% of patients, respectively [1,2]. However, since ICB resistance remains a major challenge, novel treatment options are needed to combat these immunotherapy-refractory tumor fractions. We and others previously reported that the receptor tyrosine kinase AXL is a critical resistance marker to targeted therapy in melanoma and lung cancer [3,4], and that AXL-positive cell fractions can be effectively eliminated by an AXL-targeting antibody-drug conjugate (enapotamab vedotin, EnaV [5]). AXL may also mark immunotherapy-resistant tumor cell fractions [6], and antibody-drug conjugates may induce an inflammatory response, which could reinvigorate the immune response and may synergize with immune therapies [7]. Here we explored the potential of EnaV to cooperate with tumor-specific T cells and ICB.

**Methods**

We established a cohort of melanoma and lung cancer cell lines HLA- and antigen-matched to tumor-specific T cells using the MART-1 TCR system [8, 9]. These cell lines were treated with EnaV and matched cytotoxic T cells in vitro to evaluate their sensitivity. Furthermore, we tested whether EnaV could induce an inflammatory response in vitro and in vivo. Finally, we assessed any cooperative anti-tumor activity of EnaV, T cells, and anti-PD1 in human xenografts of melanoma and lung cancer.

**Results**

Several AXL-positive melanoma and lung cancer models showed a dose-dependent cooperative sensitivity to combined treatment with EnaV and tumor-specific T cells. EnaV had a profound effect on tumor models that were refractory to either tumor-specific T cells and/or ICB. Mechanistically, RNA and proteomic profiling revealed that EnaV treatment induced an inflammatory response in tumors, including markers of immunogenic cell death. Combining EnaV with tumor-specific T cells and anti-PD1 proved superior to either treatment alone in melanoma and lung cancer models. This effect was also seen in a model which showed no response to anti-PD1 treatment alone, suggesting that EnaV can create a de novo sensitization to anti-PD1.

**Conclusions**

Our findings demonstrate that EnaV and immunotherapy cooperatively exert a strong anti-tumor response in therapy-refractory melanoma and lung cancer models. Importantly, even in models fully refractory to either T cells and/or anti-PD1, EnaV treatment restores sensitivity to promote tumor elimination. Together, these results indicate that targeting AXL-positive, immunotherapy-resistant tumor fractions with EnaV enhances ICB sensitivity, thus warranting further investigations into this combination in a clinical setting.

**References**

1. Larkin J, Chiarion Sileni V, Gonzalez R, Grob J-J, Cowey CL, Lao CD, et al. Combined Nivolumab and Ipilimumab or Monotherapy in Untreated Melanoma. N Engl J Med. Massachusetts Medical Society; 2015 Jul 2;373(1):23–34.

2. Garon EB, Rizvi NA, Hui R, Leighl N, Balmanoukian AS, Eder JP, et al. Pembrolizumab for the treatment of non-small-cell lung cancer. N Engl J Med. Massachusetts Medical Society; 2015 May 21;372(21):2018–28.

3. Müller J, Krijgsman O, Tsoi J, Robert L, Hugo W, Song C, et al. Low MITF/AXL ratio predicts early resistance to multiple targeted drugs in melanoma. Nat Commun. Nature Publishing Group; 2014;5:5712.

4. Zhang Z, Lee JC, Lin L, Olivas V, Au V, LaFramboise T, et al. Activation of the AXL kinase causes resistance to EGFR-targeted therapy in lung cancer. Nat Genet. Nature Publishing Group; 2012 Aug;44(8):852–60.

5. Boshuizen J, Koopman LA, Krijgsman O, Shahrabi A, van den Heuvel EG, Ligtenberg MA, et al. Cooperative targeting of melanoma heterogeneity with an AXL antibody-drug conjugate and BRAF/MEK inhibitors. Nat Med. Nature Publishing Group; 2018 Feb;24(2):203–12.

6. Aguilera TA, Rafat M, Castellini L, Shehade H, Kariolis MS, Hui AB-Y, et al. Reprogramming the immunological microenvironment through radiation and targeting Axl. Nat Commun. Nature Publishing Group; 2016 Dec 16;7:1–14.

7. Müller P, Kreuzaler M, Khan T, Thommen DS, Martin K, Glatz K, et al. Trastuzumab emtansine (T-DM1) renders HER2+ breast cancer highly susceptible to CTLA-4/PD-1 blockade. Sci Transl Med. American Association for the Advancement of Science; 2015 Nov 25;7(315):315ra188–8.

8. Jorritsma A, Gomez-Eerland R, Dokter M, van de Kasteele W, Zoet YM, Doxiadis IIN, et al. Selecting highly affine and well-expressed TCRs for gene therapy of melanoma. Blood. 2007 Nov 15;110(10):3564–72.

9. Vredevoogd DW, Kuilman T, Ligtenberg MA, Boshuizen J, Stecker KE, de Bruijn B, et al. Augmenting Immunotherapy Impact by Lowering Tumor TNF Cytotoxicity Threshold. Cell. Elsevier Inc; 2019 Jul 9;:1–31.

**Ethics Approval**

The collection and use of human tissue was approved by the Medical Ethical Review Board of the Antoni van Leeuwenhoek. Animal experiments were approved by the animal experimental committee of the institute and performed according to Dutch law.

#### P694 Engineering extracellular matrix-binding checkpoint inhibitor antibodies to achieve localized cancer therapy

##### Jun Ishihara, PhD, Ako Ishihara, John Michael Williford, Jeffrey Hubbell

###### University of Chicago, Chicago, IL, United States

####### **Correspondence:** Jeffrey Hubbell (jhubbell@uchicago.edu)

**Background**

Cancer immunotherapies using checkpoint inhibitor antibodies (CPI) show considerable antitumor response in the clinic. However, a substantial number of patients suffered from treatment-related adverse events. A number of patients receiving combination CPI therapy could not continue with the therapy due to adverse events. Immunotherapies serve to activate immune responses, and as such, side-effects include the symptoms of systemic lymphocyte activation and auto-immune disease induction, which typically results from drug action in healthy organs. One strategy to address this problem is through localized drug delivery systems, thereby restricting their actions to the disease site. Here, we report two technologies of tumor localized CPI antibodies via conjugation to an extracellular matrix promiscuous binding peptide derived from placenta growth factor-2 (PlGF-2123-144) or a collagen-binding domain (CBD) derived from the blood protein von Willebrand factor (VWF) A3 domain. PlGF-2123-144 conjugated CPI retains at the injection site long-term; thus PlGF-2123-144-CPI is effective when injected peri-tumor. CBD-CPI harnesses the specific exposure of tumor stroma collagen to blood stream due to the abnormal leakiness of the tumor vasculature; thus CBD-CPI is effective when it is injected intravenously.

**Methods**

PlGF-2123-144 peptide was synthesized chemically. CBD recombinant proteins were produced in HEK-293F cells and purified by ÄKTA system. We have used a chemical cross linker to conjugate PlGF-2123-144 and CBD to CPI antibody. B16F10 and BrafPten melanoma, MMTV-PyMT breast cancer, CT26 models were used. 100μg each of CPI(anti-CTLA4+anti-PD-L1) was administered.

**Results**

We show that PlGF-2123-144-conjugated CPI localized to the injection site for long time, and limiting the CPI exposure to the body and toxicity including diabetes induction risk (AB). Also, PlGF-2123-144-CPI was more efficacious than unmodified CPI, injected at same dose and same way, when injected near tumor (C). PlGF-2123-144-CPI increased intratumoral effector CD8+ T cell number compares to CPI. Next we have tested CBD conjugated CPI, injected intravenously. Intravenously administered CBD protein accumulated mainly in tumors in an orthotopic MMTV-PyMT breast tumor model (D). CBD-conjugation to CPI decreased systemic toxicity, such as T cell infiltration into the liver (E). CBD-CPI significantly suppressed tumor growth compared to their unmodified forms in B16F10 melanoma, CT26 colon carcinoma and MMTV-PyMT breast cancer models (F). CBD-CPI increased tumor-infiltrating CD8+T cells; increases in the ratio of effector CD8+ T cells to T regulatory cells were observed.

**Conclusions**

Our data suggest that the tumor-matrix binding localized CPI technology can improve both safety and efficacy of CPI therapy, with high translational promise.

**References**

1.Ishihara et al., Sci Transl Med 9, eaan0401 (2017).

2. Ishihara, J., et al., Sci Transl Med, 11. Eaau3259 (2019).

**Fig. 1 (abstract P694).**
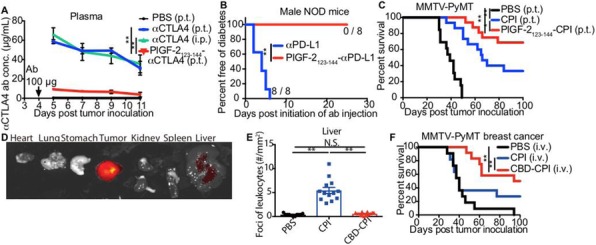
See text for description

#### P695 Tumor targeting of a STING agonist with an antibody-drug conjugate elicits potent anti-tumor immune responses

##### Naniye Malli Cetinbas, PhD, Kalli Catcott, PhD, Kenneth Avocetien, Keith Bentley, PhD, Tyler Carter, PhD, Chen-Ni Chin, PhD, Susan Clardy, PhD, Timothy Eitas, PhD, Brian Jones, PhD, Eoin Kelleher, Rebecca Mosher, MD, Mark Nazzaro, Barrett Nehilla, PhD, Marina Protopopova, PhD, Pamela Shaw, Kelly Slocum, LiuLiang Qin, Josh Thomas, PhD, Liping Yang, Dorin Toader, PhD, Marc Damelin, PhD, Jeremy Duvall, PhD, Raghida Bukhalid, PhD, Timothy Lowinger, PhD

###### Mersana Therapeutics, Cambridge, MA, United States

####### **Correspondence:** Raghida Bukhalid (rbukhalid@mersana.com), Timothy Lowinger (tlowinger@mersana.com)

**Background**

STimulator of Interferon Genes (STING) has emerged as an innate immune pathway capable of inducing anti-tumor immune activity through activation of antigen presenting cells and production of type I interferon, leading to T-cell priming and activation. In murine models, both intratumoral and intravenous administration of STING agonists have been shown to induce tumor regressions and generate immunological memory; clinical studies are underway. We hypothesize that a STING antibody-drug conjugate (ADC) – in which the STING agonist is conjugated to an antibody – will exhibit several advantages, including targeted delivery of STING agonist to desired cell types, an optimal pharmacokinetic profile, the convenience of systemic administration, and ultimately an improved therapeutic index.

**Methods**

We generated novel STING ADCs by developing a conjugation platform which consists of a STING agonist incorporated into a chemical scaffold for bioconjugation, designed to provide optimal drug-like properties. We studied the activity and mechanism of action of STING ADCs in monoculture and co-culture in vitro systems, as well as anti-tumor activity and pharmacodynamics in mice. Herein we report data with STING ADCs generated from antibodies to two therapeutic targets.

**Results**

The STING ADCs achieved a 100-fold higher potency than the corresponding free agonist in in vitro assays using THP-1 reporter cells. Similarly, in tumor cell/PBMC co-cultures, the STING ADCs exhibited 100-fold higher tumor cell-killing activity compared to free agonist, demonstrating the benefits of targeted delivery. Intravenous administration of STING ADCs regressed tumors in mouse models after a single dose of 1 mg/kg ADC in a target-dependent manner; in the same models, intravenously administered free agonist at a 50-fold higher dose resulted in only modest tumor growth delay. Importantly, a single intravenous administration of the STING ADC led to a significant increase in immune cell infiltration and tumor cell death within 72 hours and a significant increase in tumor-localized inflammatory cytokines within 12 hours, while levels of systemic cytokines remained relatively low.

**Conclusions**

We have developed a STING ADC platform and demonstrated target-dependent anti-tumor immune responses both in vitro and in vivo for two therapeutic targets.

**Ethics Approval**

Animal studies were conducted following the recommendations of the Guide for Care and Use of Laboratory Animals and in compliance with the Institutional Animal Care and Use Committees of Translational Drug Discovery and Charles River Discovery Services.

#### P696 Efficacy studies of a novel multi-targeted systemic therapy (αPD-L1/ODN1826) and deep-immune profiling of metastatic murine mammary adenocarcinoma in syngeneic immunocompetent models

##### Alan Epstein, MD, PhD^1^ , Alison Smith, MD, PhD candidate^2^

###### ^1^University of Southern California Keck S, Los Angeles, CA, United States; ^2^University of Southern California, Los Angeles, CA, United States

####### **Correspondence:** Alan Epstein (aepstein@usc.edu)

**Background**

While checkpoint inhibitors have found stunning success as single agent therapies, their clinical efficacy only extends to a subset of patients across multiple cancers. Even among likely responder preclinical models, response rates are often triggered by an early, unknown stochastic event. Ultimately, we do not know why some tumors respond and some do not. Previous work from our laboratory devised a strategy for dramatically improving systemic administration of an immune agonist of Toll-like receptor 9 (TLR9) known as CpG Oligodexoynucleotide (ODN) whose anti-tumor efficacy has been traditionally limited to intra- and peri-tumoral injections.[1,2] Through antibody-guided delivery of conjugated CpG ODN to the tumor microenvironment (TME), TLR9 activation promotes dendritic cell maturation thus upregulating cross-presentation of TME antigens.

**Methods**

Here we describe our latest multi-targeted approach which tackles immune suppression by overcoming mechanisms of acquired resistance to immunotherapy and synergizing therapeutic responses. This novel antibody immunoconjugate (AIC) targets murine TLR9 by tethering ODN1826 to a checkpoint inhibitor antibody against programmed death ligand 1 (αPD-L1) thereby stimulating and disinhibiting the immune system. Our studies of αPD-L1/ODN1826 showed superior in vivo efficacy over their free agent counterparts when delivered by intraperitoneal injection to D2F2 tumor-bearing BALB/cJ mice. All animals were treated humanely and in accordance with the guidelines of the USC’s Institutional Animal Care and Use Committee.

**Results**

Tumor growth inhibition was calculated by using the geometric mean of relative tumor volumes. The AIC demonstrated a statistically significant delay in tumor growth compared to single agents ODN1826 (-∆71.6%, p≤0.001) and αPD-L1 (p≤0.001) as well as compared to combination therapy ODN1826 + αPD-L1 (-∆51.8%, p≤0.01).

**Conclusions**

By transitioning to bioluminescence imaging techniques of tumor cells, we overcome the limitations of traditional tumor volume measurement by caliper and capture a fuller picture of both early therapeutic response events and metastatic disease. Additionally, subsequent deep immunophenotyping by Mass CyTOF combined with recent advancements in Digital Spatial Profiling, we can establish a more comprehensive method of tumor immune profiling. Elucidating the immune status of murine tumor models is a critical factor for predicting therapeutic response and understanding the mechanisms underlying immune suppression and activation.

**Acknowledgements**

This work was supported by Cancer Therapeutics, Inc., Los Angeles, CA.

**References**

1. Li, Z. et al. Generation of tumor-targeted antibody-CpG conjugates. J. Immunol. Methods 389, 45–51 (2013).

2. Jang, J. K. et al. Systemic delivery of chTNT-3/CpG immunoconjugates for immunotherapy in murine solid tumor models. Cancer Immunol. Immunother. 65, 511–23 (2016).

**Ethics Approval**

All animals were treated humanely and in accordance with the guidelines of the USC’s Institutional Animal Care and Use Committee under protocol 20265.

#### P697 Pre-clinical characterization of the mechanism of action of a CD25-targeted pyrrolobenzodiazepine dimer-based antibody-drug conjugate targeting regulatory T cells in solid cancers

##### Francesca Zammarchi, PhD^1^, Simon Chivers^2^, Patrick van Berkel, PhD^2^, Francesca Zammarchi, PhD^2^

###### ^1^ADC Therapeutics, London, United Kingdom; ^2^ADC Therapeutics Ltd. UK, London, United Kingdom

####### **Correspondence:** Francesca Zammarchi (francesca.zammarchi@adctherapeutics.com)

**Background**

Regulatory T cells (Tregs) play an important role in the establishment and progression of tumors and are considered a major obstacle to tumor eradication by immunotherapies [1]. Moreover, the intra-tumoral balance between Tregs and effector T cells (Teffs) appears to influence the outcome of immunotherapies [2], and poor prognosis in solid tumors is often associated with high tumor infiltration by Tregs and a low ratio of Teffs/Tregs [3].

**Methods**

Sur301 is an antibody-drug conjugate (ADC) composed of PC61, a rat monoclonal antibody directed against mouse CD25, conjugated to a pyrrolobenzodiazepine (PBD) dimer via a protease-cleavable linker, with a drug-to-antibody ratio of 2 [4]. We have previously shown that sur301 has potent and durable anti-tumor activity in vivo against CD25-negative immunogenic solid tumors with infiltrating CD25-positive Tregs and, when used at a sub-optimal dose, its activity is further enhanced in combination with PD-1 blockade [4]. Sur301 anti-tumor activity, either alone or combined with an anti-PD-1 antibody, was significantly reduced in the absence of CD8+ T cells, and when tumor-free survivors were re-challenged, they did not develop new tumors indicating that sur301 was able to induce tumor-specifi¬c protective immunity [4].

**Results**

In order to characterize the mode of action of sur301, we analysed the immunophenotype of Tregs and Teffs isolated from tumors, blood and spleens of mice bearing established MC38 tumors following a single dose of sur301 either alone or in combination with an anti-PD-1 antibody. Additionally, we performed a T cells dynamic study in non-tumor bearing immunocompetent mice over a period of 21 days. Spleen, lymph node, thymus and blood were analysed following a single dose of sur301 or an isotype control ADC. In both studies, sur301 resulted in strong but specific Tregs depletion, while Teffs were not affected by sur301. Interestingly, the substantial Tregs depletion observed in spleen, lymph node and blood of non-tumor bearing mice was accompanied by a temporary but significant elevation of thymic Tregs.

**Conclusions**

In conclusion, these new data show that sur301 is able to mediate potent Tregs depletion without affecting Teffs. Together with our previous data [4], these results suggest that sur301’s mode of action is at least in part mediated by Tregs depletion while Teffs are not affected. Translation of these pre-clinical data in the clinic is currently being investigated in a phase I trial evaluating the efficacy of camidanlumab tesirine (ADCT-301), a PBD-based ADC targeting human CD25, in patients with selected advanced solid tumors (NCT03621982).

**References**

1. Sasidharan Nair, V. and E. Elkord, Immune checkpoint inhibitors in cancer therapy: a focus on T-regulatory cells. Immunol Cell Biol, 2018. 96(1): p. 21-33.

2. Menetrier-Caux, C., et al., Targeting regulatory T cells. Target Oncol, 2012. 7(1): p. 15-28.

3. Arce Vargas, F., et al., Fc-Optimized Anti-CD25 Depletes Tumor-Infiltrating Regulatory T Cells and Synergizes with PD-1 Blockade to Eradicate Established Tumors. Immunity, 2017. 46(4): p. 577-586.

4. Zammarchi, F., et al., A CD25-targeted pyrrolobenzodiazepine dimer-based antibody-drug conjugate shows potent anti-tumor activity in pre-clinical models of solid tumors either alone

#### P698 Chimeric protein MICA-G129R Bridges Breast Cancer Cells and Natural Killer Cells

##### Hui Ding, PhD candidate, Yanzhang Wei

###### Clemson University, Clemson, SC, United States

####### **Correspondence:** Yanzhang Wei (ywei@clemson.edu)

**Background**

Major histocompatibility complex class I chain-related protein A (MICA) is a stress-induced ligand for the activating receptor NKG2D on natural killer (NK) cells. It is expressed on the cell surface of most cancer types at the early stages, but usually down-regulated or shed in advanced cancers leading to immune evasion [1,2]. Prolactin is an essential protein hormone for normal reproduction and maintenance of pregnancy, and contributes to pathogenesis of gynecologic malignancies [3]. The prolactin receptor (PRLR) was found over-expressed in many cancers, especially breast cancer [4]. G129R is a mutant of human prolactin with a single amino acid substitution. It still binds to PRLR but inhibits the growth of breast cancer cells [5]. We aim to create a chimeric protein with MICA and G129R, and use it to bridge breast cancer cells and NK cells. The MICA portion in the chimeric protein binds to the NKG2D receptor and activate NK cells, and the G129R portion targets to the PRLR on the breast cancer cells and leads NK cells to them.

**Methods**

A chimeric gene was created by cloning and fusing the cDNA of the extracellular domain of MICA and the cDNA of G129R into a plasmid, and then transfected into HEK293 cells to produce MICA-G129R protein. The MICA-extracellular-domain only protein and the G129R only protein were also generated as controls. When the proteins were added into the co-culture of NK cells (NK-92 cell line) and breast cancer cells (T47D cell line), the cytotoxicity was measured using a lactate dehydrogenase (LDH)-based assay, the granzyme B released by NK-92 cells was detected using ELISA, and the apoptosis of the T47D cells was examined using a caspase 3/7 luminescence assay.

**Results**

We successfully generated HEK293 stable clones to produce MICA-G129R, MICA and G129R proteins. The proteins were all confirmed using Western blot. The chimeric protein MICA-G129R was proved to be able to bind to NK-92 cells and T47D cells using immunofluorescent staining. The chimeric protein MICA-G129R significantly increased the cytotoxicity of the NK-92 cells to T47D cells in their co-culture. The granzyme B secretion of NK-92 cells was induced, and the caspase3/7 in the T47D cells were elevated in the co-culture of NK-92 and T47D with the presence of MICA-G129R.

**Conclusions**

The chimeric protein MIC-G129R can bridge NK cells and breast cancer cells, and induce the cytotoxicity of NK cells to kill the breast cancer cells.

**References**

1. Salih HR, Rammensee H-G, Steinle A. Cutting edge: down-regulation of MICA on human tumors by proteolytic shedding. J Immunol. 2002;169(8):4098-4102.

2. Duan S, Guo W, Xu Z, et al. Natural killer group 2D receptor and its ligands in cancer immune escape. Mol Cancer. 2019;18(1):29.

3. Bachelot A, Binart N. Reproductive role of prolactin. Reproduction. 2007;133(2):361-369.

4. Goffin V. Prolactin receptor targeting in breast and prostate cancers: New insights into an old challenge. Pharmacol Ther. 2017;179:111-126.

5. Chen WY, Ramamoorthy P, Chen N, Sticca R, Wagner TE. A human prolactin antagonist, hPRL-G129R, inhibits breast cancer cell proliferation through induction of apoptosis. Clin Cancer Res. 1999;5(11):3583-3593.

#### P699 DSP107—A first in class, bifunctional fusion protein targeting both innate and adaptive immunity

##### Adam Foley-Comer, MD^1^, Shirley Greenwald^1^, Ewa Cendrowicz^2^, Rinat Tabakman^1^, Shira Amsili^1^, Alexandra Aronin^1^, Liat Ben-Gigi-Tamir^1^, Elina Zorde-Khvalevsky^3^, Yosi Gozlan^1^, Michal Dranitzki-Elhalel^3^, Ayelet Chajut^1^ , Amnon Peled, PhD^3^, Edwin Bremer^2^

###### ^*1*^*Kahr Medical, Raanana, Israel;*^*2*^*University Medical Center, Groningen, Netherlands* ; ^*3*^*Hadassah Hebrew University Medical Center, Groningen, Netherlands*

####### **Correspondence:** Ayelet Chajut (ayelet@kahr-medical.com)

**Background**

Checkpoint inhibitors have transformed cancer treatment in certain patient subgroups. To improve response rates, considerable effort is now being invested developing novel immune-oncology (IO) drugs and exploring IO target combinations.

DSP107 is a bi-functional, trimeric protein consisting of the fused extracellular domains of human SIRPα and 4-1BBL that uniquely combines activation of both innate and adaptive immunity in a single product. Interaction of CD47, frequently overexpressed on cancer cells, with SIRPα on phagocytes transmits a “don’t eat me” signal. Binding of DSP107 to CD47 on tumor cells blocks the inhibitory signal delivered to phagocytes and cross-presents trimerized 4-1BBL to activated tumor antigen-specific T-cells that express the 4-1BB costimulatory receptor. Cross-presentation and trimerization of the 4-1BBL are required for optimal 4-1BB activation.

**Methods**

DSP107 was manufactured in CHO-S cells. Binding and affinity to CD47 and 4-1BB were evaluated by Biacore, FACS and ELISA. T-cell activation, proliferation, IFN-γ secretion, cytotoxicity and phagocytosis were confirmed in vitro using lymphoma, leukemia and carcinoma cell lines. In-vivo efficacy was tested against hCD47-MC38 tumors in h41BB-KI-C57BL/6 mice and against SUDHL4 (lymphoma) in humanized NSG mice. Safety was evaluated in a non-GLP, repeat-dose study in cynomolgus monkeys.

**Results**

DSP107 binds to CD47 and 4-1BB with high affinity (1.2 nM and 0.7 nM, respectively). Cell based reporter assays showed CD47 binding dependency for signaling through 4-1BB. Using in vitro assays, DSP107 amplified T-cell activation and proliferation, and augmented T-cell mediated elimination of cancer cells (0.5-5 nM). DSP107 also augmented macrophage-mediated phagocytosis of tumor cells as monotherapy and in combination with targeted monoclonal antibodies (6-12 nM). DSP107 led to statistically significant tumor growth inhibition (TGI) as monotherapy (lymphoma model; p

**Conclusions**

DSP107 is a bifunctional, fusion protein with a unique mechanism of action. In vivo, DSP107 was efficacious as monotherapy and in combination with anti PD-L1. Doses ≤ 50 mg/kg were safe and well tolerated in monkeys. IND-enabling studies are ongoing and clinical studies will commence in Q2/2020.

**Ethics Approval**

With reference to the study in h41BB-KI-C57BL/6 mice, the protocol and any amendment(s) or procedures involving the care and use of animals were reviewed and approved by the Institutional Animal Care and Use Committee (IACUC) of CrownBio prior to execution. During the study, the care and use of animals were conducted in accordance with the regulations of the Association for Assessment and Accreditation of Laboratory Animal Care (AAALAC).

With reference to the lymphoma model in humanized NSG mice, the study was approved by the Hebrew University Ethical Board, approval number MD-19-15821-5.

The protocol for the cynomolgus monkey study (Covance Study 8402826) was reviewed and approved by the Establishment Animal Welfare and Ethical Review Body. This study also complies with the permissions and conditions contained within Project Licence P98E2EBFD "Safety Testing of Medicinal Products Using Non-Human Primates" as authorised by the UK Home Office.

**Fig. 1 (abstract P699).**
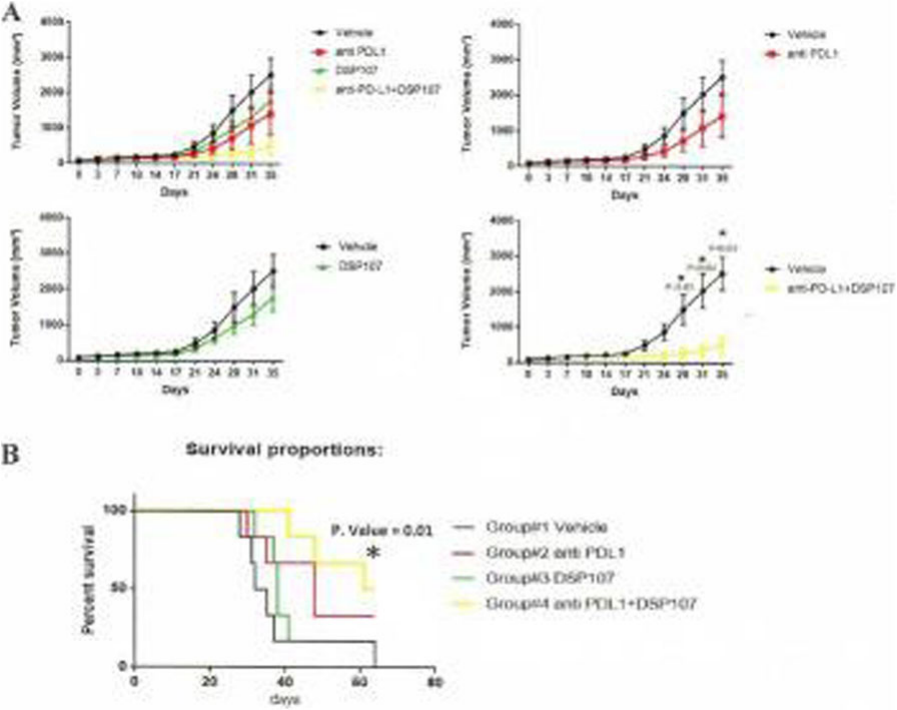
See text for description

#### P700 Combining CD27 costimulation and PD-1 blockade into a bispecific antibody improves T cell activation and anti-tumor activity over combination of individual antibodies

##### Laura Vitale, BS, Lawrence Thomas, PhD, Thomas O'Neill, BA, Jenifer Widger, BA, Laura Mills-Chen, Andrea Crocker, BS, Colleen Patterson, Anna Wasiuk, Eric Forsberg, James Boyer, Crystal Sisson, Jeffrey Weidlick, BS, Shannon Renn-Bingham, Ioannis Papayannopoulos, Russ Hammond, Joel Goldstein, PhD, Henry Marsh, Jr., Li-Zhen He, MD, Michael Yellin, MD, Tibor Keler, PhD, Tibor Keler, PhD

###### Celldex Therapeutics, Hampton, NJ, United States

####### **Correspondence:** Tibor Keler (tkeler@celldex.com)

**Background**

Strong scientific rationale and preclinical data support the combination of CD27 costimulation with PD-1 blockade. Synergy of these pathways for anti-tumor immunity has been demonstrated in several models. Studies of the effector T cell response suggest that these pathways converge to activate a non-redundant transcriptional program with anti-CD27 preferentially driving increased cell proliferation whereas anti-PD-1/L1 activates an effector-gene profile [1]. In a clinical study with varlilumab, a CD27 agonist antibody, three highly durable responses were observed in advanced cancer patients and had minimal adverse effects as monotherapy [2]. In combination with PD-1 blockade using nivolumab, durable responses were observed among patients with generally low response to PD-1 monotherapy and the combination was generally well tolerated, with no increase in toxicities observed over the individual agents.

**Methods**

We developed novel human antibodies to CD27 and PD-L1 from which the best pair was selected based on activity and manufacturability to engineer into a whole IgG-scFv bispecific antibody format. The bispecific antibody, CDX-527, was compared to parental antibodies in several in vitro assays of T cell activation. A surrogate bispecific using variable region sequences from the mouse cross-reactive PD-L1 antibody, avelumab, was generated for testing in human CD27 transgenic mice. A pilot study of CDX-527 was performed in cynomolgus macaques.

**Results**

CDX-527 was found to be a potent PD-1 inhibitor and a stronger CD27 agonist than anti-CD27 monoclonal antibodies. CDX-527 can interact simultaneously with CD27, PD-L1 and Fc receptors and has greater activity in mixed-lymphocyte reactions compared to the combination of the parental antibodies, indicating that cross-linking by both Fc receptors and PD-L1 leads to enhanced activity. This enhanced T cell activation requires concurrent T cell receptor activation. In huCD27 transgenic mice, the surrogate PD-L1xCD27 bispecific demonstrates more potent induction of antigen-specific T cell responses and antitumor activity than the combination of the parental antibodies. A pilot study of CDX-527 in cynomolgus macaques confirmed a good PK profile with a half-life of 5.3 days, increase in serum chemokines, with no adverse effects of treatment noted in clinical observations or clinical chemistry.

**Conclusions**

CDX-527 effectively combines PD-1 blockade and CD27 costimulation into one molecule that is more potent than combination of the parental antibodies. CDX-527 IND-enabling studies are ongoing with the IND submission planned for early next year.

**References**

1. Buchan SL, Fallatah M, Thirdborough SM, et al. PD-1 Blockade and CD27 Stimulation Activate Distinct Transcriptional Programs That Synergize for CD8 + T-Cell–Driven Antitumor Immunity. Clin Cancer Res. 2018;24:2383-2394

2. Burris HA, Infante JR, Ansell SM, et al. Safety and Activity of Varlilumab, a Novel and First-in-Class Agonist Anti-CD27 Antibody, in Patients With Advanced Solid Tumors. J Clin Oncol. 2017;35:2028-2036

**Ethics Approval**

Animals were sourced from IACUC-approved commercial sources. Murine studies were approved by the Celldex IACUC of Hampton, NJ or the Celldex IACUC of Needham, MA. The pilot primate study was approved by the Citox Lab USA IACUC of Stilwell, KS.

#### P701 A next generation bispecific antibody platform for effective tumor cell killing with minimal cytokine release

##### Udaya Rangaswamy, PhD, Harbani K Malik-Chaudhry, PhD, Brian Avanzino, PhD, Aarti Balasubramani, Andrew Boudreau, BA, Benjamin Buelow, Kevin Dang, BA, Laura Davison, PhD, Kathrine Harris, Sharon Hartstein, Brett Jorgensen, BA, Hannes Kehm, Yuping Li, Kyle Lorentsen, PhD, Harish Medlari, Duy Pham, BA, Kirthana Prabhakar, Ute Schellenberger, PhD, Harshad Ugamraj, BA, Nathan Trinklein, PhD, Roland Buelow, PhD, Suhasini Iyer, PhD

###### Teneobio, Menlo Park, CA, United States

####### **Correspondence:** Suhasini Iyer (siyer@teneobio.com)

**Background**

Bispecific T cell redirecting antibodies (T-BsAbs) targeting tumor cells and T cells are an increasingly popular form of cancer immunotherapy. The dual targeting redirects T cells to specifically cause cytotoxicity of tumor cells upon formation of an immunological synapse between the T cells and tumor cells, making this specificity an advantage over conventional monoclonal antibody therapy. Evidence from clinical data of Blinatumomab, the only FDA approved bispecific antibody, suggests that T-BsAbs are associated with cytokine release syndrome (CRS), similar to other T cell targeting therapies such as Muromonab or CAR-T, thus warranting a newer generation of bispecific antibodies which can limit CRS.

**Methods**

Antibodies against CD3 were developed by immunization of OmniFlic® animals. Anti-TAA (tumor associated antigen) antibodies (UniAbsTM) for various solid and liquid tumor antigens were developed by immunizations of transgenic humanized rats (UniRatsTM). Antibody discovery entailed antibody repertoire deep sequencing, high throughput gene assembly, cloning and screening. This strategy resulted in a novel class of anti-CD3 antibodies with varying affinities and different target epitopes. Bispecific antibodies were generated by knob in hole technology utilizing one such anti-CD3 domain, termed anti-CD3_F2B in combination with different anti-TAA arms. Specific anti-tumor activity of the T-BsAbs were evaluated by measuring cytotoxicity of tumor cells in the presence of T cells. Supernatants from these co-cultures were used to measure cytokines, namely IL-2 and IFNγ. In vivo studies were performed in a mouse xenograft model by injecting drug into NOG mice engrafted with human tumor cells and adoptively transferred human PBMCs (peripheral blood mononuclear cells).

**Results**

Our novel T-BsAbs targeting various solid and liquid tumor indications cause efficient tumor cell lysis, but with minimal cytokine release in respective in vitro tumor cell and T cell co-culture models. Additionally, one such molecule, anti-CD19xCD3 T-BsAb effectively cleared CD19+ tumor cells in NOG mice in the presence of adoptively transferred human PBMCs.

**Conclusions**

This next generation of T-BsAbs thus shows promise as an effective therapy against multiple cancer indications.

**Ethics Approval**

Animal studies were done in accordance with IACUC protocol # AUP-17-0331-M-2

#### P702 Informing rational immunotherapy combinations for enhancing therapeutic activity of Bi-Specific T cell Engager (BiTE®) antibody constructs in solid tumors

##### DEEPALI SAWANT, PhD, Brian Belmontes, Famke Aeffner, Olivier Nolan-Stevaux, Jackson Egen, Jason DeVoss, PhD

###### Amgen, South San Francisco, CA, United States

####### **Correspondence:** Jason DeVoss (jdevoss@amgen.com)

**Background**

Bi-specific T cell Engager (BiTE®) antibody constructs are synthetic cancer immunotherapies aimed at inducing polyclonal anti-tumor T cell responses by re-directing T cells to specific surface antigens expressed on tumors. BiTE® molecules are bispecific proteins with 2 linked scFvs; one targeting the CD3 surface receptor on T cells and the other targeting an antigen expressed on cancer cells. This class of immunotherapies is currently showing promising results in hematological malignancies, with Blincyto® (Blinatumomab) leading to complete remissions in 69% of patients with CD19+ B-ALL. While BiTE® antibody constructs are currently undergoing testing in solid tumors, only limited clinical data are available. However, multiple features of solid tumors, such as restricted T cell recruitment, limited BiTE® biodistribution, and an immunosuppressive microenvironment could potentially negatively influence BiTE® activity.

**Methods**

We have engineered a knock-in mouse expressing a chimeric mouse/human CD3e receptor recognized by BiTE® antibody constructs from the endogenous mouse CD3e genomic locus to elucidate the pharmacokinetic, pharmacodynamic and efficacy parameters governing BiTE® activity in preclinical models.

**Results**

We have evaluated tumor types with varying baseline T cell densities to understand the relationship between T cell density and BiTE® activity in a tumor. In addition, we have assessed the reliance of BiTE® activity on cytotoxic function of pre-existing tumor-resident T cells, recruitment of new T cells from the periphery, and local T cell proliferation across tumor models. These datasets are being employed to interrogate the immune determinants of efficacy that drive BiTE® activity.

**Conclusions**

These studies will allow defining key mechanisms that may limit BiTE® efficacy in solid tumors and informing optimal therapeutic combination strategies capable of maximizing the potential of BiTE® therapy in solid tumors.

**Ethics Approval**

All in vivo work was conducted under an IACUC approved protocol and in an AAALAC accredited facility.

#### P703 Co-stimulation of OX40 or LTβR reprograms exhausted lymphocytes to acquire an effector phenotype in the setting of combined TIGIT and checkpoint blockade

##### George Fromm, PhD^1^ , Suresh De Silva, PhD^2^, Arpita Patel, BA^2^, Kyung Jin Yoo^2^, Kellsey Johannes, BS^2^, Kaiwen Huang^2^, Taylor Schreiber, MD, PhD^2^

###### ^1^Shattuck Labs, Inc, Apex, NC, United States; ^2^Shattuck Labs, Durham, NC, United States

####### **Correspondence:** George Fromm (gfromm@shattucklabs.com)

**Background**

Recently, the role of TOX as a key transcription factor guiding T cells towards exhaustion have shed light on strategies to reprogram tumor infiltrating lymphocytes. While inhibition of TOX prevented acquisition of an exhausted phenotype, it did not result in acquisition of effector properties. This may explain why additional inhibition of TIGIT/LAG3/TIM3 in the setting of PD-1 blockade has not yielded marked clinical improvement. Instead, these results hint that while checkpoint blockade may be required to destabilize an exhausted phenotype, pushing exhausted lymphocytes toward an effector phenotype may require co-stimulatory signaling. To explore this, we developed two Agonist Redirected Checkpoint (ARC) constructs which combine TIGIT/PVR inhibition with either OX40L or LIGHT mediated co-stimulation, and compared the activity of these compounds to TIGIT/OX40/LTβR antibodies alone or with PD-1 or CTLA-4 checkpoint blocking antibodies.

**Methods**

Human TIGIT-Fc-OX40L and TIGIT-Fc-LIGHT (and the murine surrogates) were purified, and target binding assessed by ELISA, Octet, and cell-surface binding assays. Functional activity was confirmed using NFkB/NIK signaling, cytokine/chemokine stimulating, and immune cell activating assays. Anti-tumor efficacy of mouse TIGIT-Fc-OX40L and TIGIT-Fc-LIGHT was assessed alone and in combination with anti-PD1 and anti-CTLA4 in murine CT26 and B16.F10 tumors.

**Results**

Each respective domain of TIGIT-Fc-OX40L and TIGIT-Fc-LIGHT bound human and cyno PVR, OX40, LTbR, and HVEM with low nanomolar affinity. Due to the hexameric structure, the OX40L domain clustered OX40 receptors on the cell surface and stimulated NFkB signaling in a single-cell system lacking effector Fc receptors, and stimulated IL-2, TNFa, and IFNg secretion from human PBMCs in the presence of SEB. TIGIT-Fc-LIGHT uniquely induced non-canonical NFkB/NIK signaling, stimulated CCL2/CXCL8 expression and IL-8 secretion, and induced antigen-independent proliferation and differentiation of CD8-depleted human PBMCs. Both murine TIGIT-Fc-OX40L and TIGIT-Fc-LIGHT were effective at controlling CT26 and B16.F10 growth, and programed a memory response that mediated the rejection of secondary tumor re-challenge. Anti-tumor efficacy of both ARCs was enhanced when combined with checkpoint blockade of PD-1 or CTLA-4; resulting in complete tumor rejection. Lastly, both ARCs have concluded non-human primate dose-range finding studies and have demonstrated distinct on-target PD activity.

**Conclusions**

The addition of OX40L or LIGHT as co-stimulatory molecules dramatically improved the efficacy of checkpoint blockade to TIGIT both alone and in combination with PD-1 or CTLA-4 blockade. Pharmacodynamic evidence from NHP suggests that hexamerized OX40L and LIGHT activate OX40 and LTβR effectively, compared to existing data from agonist monoclonal antibodies to the same targets, and may uniquely reverse immune exhaustion.

#### P704 An Fc effector enhanced EGFR/cMet bispecific antibody (JNJ-61186372), mediates EGFR/cMet downmodulation and therapeutic efficacy through monocyte and macrophage trogocytosis

##### Smruthi Vijayaraghavan, PhD, Smruthi Vijayaraghavan, PhD , Lorriane Lipfert, Kristen Chevalier, Barbara Bushey, BS, Benjamin Henley, Ryan Lenhart, Jocelyn Sendecki, MS, Marilda Beqiri, Hillary Millar, MS, Kathryn Packman, PhD, Matthew Lorenzi, PhD, Sylvie Laquerre, Sheri Moores, PhD

###### Janssen R&D, Spring House, PA, United States

####### **Correspondence:** Smruthi Vijayaraghavan (svijayar@its.jnj.com)

**Background**

Agents targeting mutant EGFR in NSCLC have become standard of care but acquired resistance invariably develops due to new mutations in EGFR and activation of compensatory pathways such as cMET. JNJ-61186372 (JNJ-372) is an anti-EGFR and cMet bispecific low-fucose antibody (huIgG1) with enhanced Fc function designed to target tumors with activated EGFR and cMET signaling through a novel mechanism of action. An ongoing first-in-human study to assess the safety and efficacy of JNJ-372 in patients with advanced, treatment refractory NSCLC revealed JNJ-372 to have clinical activity in patients with diverse EGFR-mutated NSCLC, including Exon 20 mutations TKI resistance mutations (T790M, C797S) and resistance due to MET amplification. Our previous studies demonstrated that the Fc inactive version (IgG2sigma) of the EGFR/cMet antibody was significantly impaired in its ability to inhibit tumor growth compared to the Fc enhanced JNJ-372. The IgG2sigma variant also reduced the ability of the bispecific antibody to mediate downregulation of EGFR and cMet signaling. These observations suggested that the interaction of the Fc domain of the antibody with the Fc gamma receptors on innate immune cells may play a crucial role in the mechanism of action of JNJ-372.

**Methods**

To examine this hypothesis, we performed a comprehensive assessment of the Fc effector functions of JNJ-372, including effects on EGFR and cMet levels, downstream signal transduction, and role in mediating anti-tumor activity.

**Results**

Despite observing potent anti-tumor activity of JNJ-372 in EGFR mutant xenograft models, only modest anti-proliferative effects were observed in NSCLC cell lines in vitro. Interestingly, the in vitro addition of isolated human immune cells (PBMCs) notably enhanced JNJ-372-mediated EGFR and cMet downregulation, and dose-dependent tumor cell killing. Through depletion or enrichment of specific cell types, we demonstrated that monocytes and/or macrophages are necessary and sufficient for JNJ-372 Fc interaction-mediated EGFR/cMet downmodulation and that macrophages are required for in vivo efficacy. Finally, we show that the monocyte/macrophage-mediated down-modulation of EGFR/cMet signaling occurs through trogocytosis.

**Conclusions**

Collectively, these observations represent a novel Fc-dependent mechanism of action of JNJ-372 and support the continued clinical development in patients with aberrant EGFR and cMET signaling.

#### P705 A chimeric adenoviral vector overcomes pre-existing adenoviral immunity to elicit antitumor immunity to the colorectal cancer antigen GUCY2C

##### Jagmohan Singh, John Flickinger, Robert Carlson, Trevor Baybutt, Elinor Leong, Ellen Caparosa, Jamin Roh, Amanda Pattison, Jeffrey Rappaport, Joshua Barton, Tingting Yang, Scott Waldman, Adam Snook, PhD

###### Thomas Jefferson University, Philadelphia, PA, United States

####### **Correspondence:** Adam Snook (adam.snook@jefferson.edu)

**Background**

Previous studies examining vaccination against guanylyl cyclase C (GUCY2C) in colon cancer patients revealed pre-existing immunity to the adenovirus serotype 5 (Ad5) vaccine vector as a barrier to effective vaccination in patients, which was confirmed in animal models [1]. To overcome this challenge, we have generated an alternative vaccination approach using a chimeric viral vector composed of Ad5 in which the fiber molecule has been replaced with that from Ad35. The fiber is the major target for Ad5-neutralizng immunity and, in contrast to Ad5, Ad35 seroprevalence in human populations is very low. Therefore, we hypothesize that this chimeric vector (Ad5.F35) will be insensitive to Ad5-specific immunity, overcoming pre-existing immunity in patients.

**Methods**

The immunogenicity and antitumor efficacy, safety, and biodistribution of the Ad5.F35 vaccine (Ad5.F35-GUCY2C-S1) was evaluated in mouse models of metastatic colorectal cancer. Moreover, the sensitivity of Ad5 and Ad5.F35 vaccines to pre-existing Ad5 immunity was compared in animal models. Finally, the ability of patient sera to neutralize Ad5 and Ad5.F35 vector was compared.

**Results**

Like the previous Ad5 vaccination, and Ad5.F35 induced CD8+ T cell responses in adenovirus-naïve BALB/c mice. IFNγ ELISpot data showed that Ad5 and Ad5.F35 vaccination produced similar CD8+ T cell response and there was no significant difference in survival rates of Ad5- and Ad5.F35-vaccinated mice with metastatic colorectal cancer. Repeated administrations of Ad5.F35 resulted in no toxicity and little vector persistence in any examined tissues, demonstrating the safety of this approach. In unvaccinated patients, Ad5-specific neutralizing antibody titers were present in 50% (5/10) of patients, while those neutralizing Ad5.F35 were present in only 1/10 patients.

**Conclusions**

These studies demonstrate that a chimeric adenoviral vector composed of Ad5 with the Ad35 fiber is an advantageous alternative to Ad5 alone for vaccinating human populations. While Ad5 and Ad5.F35 vaccines produce comparable GUCY2C-specific immunity, antitumor efficacy, and safety profiles in adenovirus-naïve mice, Ad5.F35 is less sensitive to pre-existing Ad5-specific immunity. Planned clinical studies will examine the safety and efficacy of Ad5.F35-based vaccines in colon cancer patients.

**Acknowledgements**

The authors thank the Center for Cell and Gene Therapy, Baylor College of Medicine for assistance in Ad5.F35 vaccine manufacturing.

**References**

1. Snook AE, Baybutt TR, Xiang B, Abraham TS, Flickinger JC, Hyslop T, Zhan T, Kraft WK, Sato T, and Waldman SA. Split tolerance permits safe Ad5-GUCY2C-PADRE vaccine-induced T-cell responses in colon cancer patients. J. Immunother. Cancer. 2019;7, 104.

**Ethics Approval**

This study was approved the Thomas Jefferson University IRB (#13S.462) and IACUC (Protocol #02092).

**Consent**

No sensitive or identifiable patient data is included in this abstract.

#### P706 A TCR-CD3 bispecific fusion protein mediates increased presentation of peptide-HLA which associates with improved T cell activation and tumour cell killing

##### Duncan Gascoyne, PhD, Duncan Gascoyne, PhD, David Depoil, PhD, Karolina Rygiel, Nathaniel Davies, PhD, Cheryl McAlpine, MSN, Rupert Kenefeck

###### Immunocore, Abingdon, United Kingdom

####### **Correspondence:** Rupert Kenefeck (rupert.kenefeck@immunocore.com)

**Background**

Immune mobilising TCRs against cancer (ImmTAC) molecules are soluble bispecific fusion proteins comprised of affinity‐enhanced TCR and anti-CD3. ImmTAC molecules redirect T cells to kill tumour by binding to specific peptide HLA (pHLA) complexes on cell surface via the TCR moiety and recruiting T cells via anti-CD3.

Tebentafusp, an ImmTAC recognising gp100-derived pHLA, has monotherapy activity in advanced melanoma (NCT01211262) and pivotal studies are ongoing.

Here, using novel TCR-based labelling of individual surface pHLA molecules, we examine the effect of ImmTAC on peptide presentation and T cell response in vitro and explore tumour gene expression in tebentafusp treated patients.

**Methods**

pHLA epitope numbers were quantitated using a novel semi-automated single molecule pHLA counting technique (derived from [1]) and HLA levels determined by flow cytometry. PBMC response was measured by IFNy ELISpot and tumour killing (IncuCyte, xCELLigence or Phenix). To assess changes in tumour, paired baseline and on-treatment biopsies from tebentafusp-treated patients (n=9) (NCT01211262) were analysed for gene expression (NanoString).

**Results**

Activation of T cells, induced by ImmTAC co-incubated with 70 different tumour cell lines, correlated tightly with pHLA presentation as predicted by gene expression (n=70, R2=0.74) and with single molecule surface pHLA numbers based on TCR labelling (n=19, R2=0.86). Baseline pHLA numbers ranged from undetectable to ~500 per cell, with as few as 10-15 pHLAs per cell sufficient to drive T cell mediated killing.

The number of pHLA per tumour cell could be increased by incubation with T cells and ImmTAC, presumably due to release of T cell derived inflammatory cytokines [2], or by incubation alone with IFNβ (~3-fold, p

In patients treated with tebentafusp, comparison of on-treatment changes in tumour biopsies from partial response vs progressive disease patients, found HLA-associated antigen processing gene expression to be significantly enriched (p=0.006).

**Conclusions**

Using a state-of-the-art single molecule surface pHLA detection technology, T cell activation by ImmTAC is highly sensitive and correlates with pHLA number. T cell activation by ImmTAC further augments HLA expression and pHLA presentation indicating a feed-forward loop. Evidence of this is suggested by enrichment of HLA-associated antigen processing genes in tumour biopsies from patients responding to tebentafusp. By this mechanism, ImmTAC may promote overall tumour immunogenicity and epitope spreading, thus amplifying native T cell responses.

**Trial Registration**

Trial Registration Reference NCT01211262

**References**

1. Bossi G, Gerry AB, Paston SJ, Sutton DH, Hassan NJ, Jakobsen BK. Examining the presentation of tumor-associated antigens on peptide-pulsed T2 cells. OncoImmunology. 2013;2(11);e26840

2. Liddy N, Bossi G, Adams KJ, Lissina A, Mahon TM, Hassan NJ, Gavarret J, Bianchi FC, Pumphrey NJ, Ladell K, Gostick E, Sewell AK, Lissin NM, Harwood NE, Molloy PE, Li Y, Cameron BJ, Sami M, Baston EE, Todorov PT, Paston SJ, Dennis RE, Harper JV, Dunn SM, Ashfield R, Johnson A, McGrath Y, Plesa G, June CH, Kalos M, Price DA, Vuidepot A, Williams DD, Sutton DH, Jakobsen BK. Monoclonal TCR-redirected tumor cell killing. Nat. Med. 2012;18:980–7.

**Ethics Approval**

This study was approved by following institutions’ Ethics Boards:

Oxfordshire Research Ethics Committee; 10/H0604/47, Approved June 4, 2010.Mary Crowley Cancer Research Center; MCMRC IRB # 12-06, Approved March 16, 2012.

Human Investigation Committee, Yale University; HIC Protocol # 1302011504, Approved March 22, 2012.

IntegReview; Protocol No IMCgp100/01, Approved November 13, 2013.

Western Sydney Local Health District; HREC2012/7/4.1 (3552) AU RED HREC/12/WMEAD/237, Approved on October 24, 2012.

Western Institutional Review Board; Panel 1, Study Num 1147687, WIRB Pro Num 20141184, Approved July 15, 2014.

Memorial Sloan Kettering Cancer Center, Institutional Review Board; Protocol # 14-152, August 28, 2014.

#### P707 Matrix-binding IL-12 enhances tumor inflammation and drives complete remission in established cold murine tumors

##### Aslan Mansurov, BChen, Jeffrey Hubbell , Jun Ishihara, PhD

###### University of Chicago, Chicago, IL, United States

####### **Correspondence:** Jeffrey Hubbell (jhubbell@uchicago.edu)

**Background**

Checkpoint inhibitor (CPI) immunotherapy has achieved remarkable success in the clinic, yet its efficacy in poorly immunogenic, immune-excluded tumors has been modest. Interleukin (IL)-12 is an attractive cytokine capable of activating the innate and adaptive arms of immunity, yet immune-related adverse events have been a major barrier in its translation as an anticancer cytokine. To overcome this, we engineered collagen-binding IL-12 (CBD-IL-12), which, after intravenous administration, accumulates in the tumor stroma due to exposed collagen associated with disordered tumor vasculature.

**Methods**

N/A

**Results**

B16F10 melanoma is a poorly immunogenic and checkpoint-unresponsive tumor model. Single i.v. administration of CBD-IL-12 to mice bearing moderately-sized B16F10 tumors (~60 mm3 at the start of therapy) resulted in complete remission (CR) in 10 out of 15 mice, whereas none were cured by equimolar IL-12. EMT6 breast cancer is an immune-excluded, “cold” tumor model and is minimally responsive CPI therapy. In this model, single i.v. treatment with CBD-IL-12 achieved CR in 13 out of 15 treated mice, whereas equimolar i.v. IL-12 treatment achieved CR in 6 out of 15 mice. To investigate the pharmacodynamic mechanisms of enhancement of antitumor efficacy of CBD-IL-12, we sought to measure intratumoral (i.t.) IFNg kinetics in B16F10 melanoma. Single treatment with CBD-IL-12 resulted in sustained levels of i.t. IFNg for at least 4 days post-therapy, whereas i.t. levels of IFNg rapidly declined after treatment with unmodified IL-12, showing the effect of CBD-IL-12 retention within tumor matrix. Systemic administration of CBD-IL-12 to B16F10 tumor-bearing mice significantly decreased systemic toxicities, such as serum IFNg, ALT, and pancreatic damage (i.e., lipase, amylase) compared to equimolar IL-12. Ability to administer CBD-IL-12 systemically enables treatment of both primary and metastatic tumors. In the pulmonary metastatic model of B16F10, CBD-IL-12 decreased the tumor burden two-fold compared to equimolar unmodified IL-12. Treatment with CBD-IL-12 induced dramatic differences in both the innate and the adaptive immune compartments in the lungs of treated mice as assessed by flow cytometry. Additionally, we investigated whether CBD-IL-12 can synergize with CPI therapy in the CPI-unresponsive B16F10 melanoma model. Treatment of mice bearing large, established B16F10 tumors (~120 mm3) with only two cycles CBD-IL-12 + CPI cured 7 out of 12 mice, whereas no CR was observed in either monotherapy group, confirming the synergistic antitumor effects of CBD-IL-12 and CPI.

**Conclusions**

Collectively, our data show that CBD-IL-12 can safely cure large, established tumors without antigen-specific approach, greatly increasing the therapeutic index of IL-12.

**Ethics Approval**

All animal experiments performed in this work were approved by the Institutional Animal Care and Use Committee of the University of Chicago.

### Immunotherapy Toxicities

#### P708 Real world evidence of patient clinical profiles with advanced solid tumor malignancies receiving immune checkpoint inhibitors to investigate potential predictors for immune-related adverse events

##### Stephanie Berg, DO^1^, Joseph Clark, MD^1^, Jose Guevara^2^, Elizabeth Elliott, DO^1^, Stephanie Berg, DO^2^ , Michael Wesolowski^2^, Blaine Knox, MD^1^, Daniel Linden, DO^1^, Courtney Wagner, MD^1^

###### ^1^Loyola University Medical Center, Maywood, IL, United States; ^*2*^*Loyola University Chicago, Chicago, IL, United States*

####### **Correspondence:** Stephanie Berg (sberg2@luc.edu)

**Background**

Immune-related adverse events (irAEs) related to immune checkpoint inhibitors (ICI) may target any organ and originate from autoreactive T cells injuring host tissues. There is a need to uncover potential predictors for irAE development in patients (pts) receiving ICIs. Real-world evidence has been utilized previously to inform therapeutic development, outcomes research, and quality improvement [1]. Our study uses real world evidence in a prospective fashion to investigate potential characteristics which exist in pts that could predict irAEs occurrence. We predict that there may be specific clinical variables present before ICI administration that result in increased susceptibility to irAEs.

**Methods**

We designed a prospective study at Loyola University Medical Center to document clinical profiles of pts diagnosed locally advanced or metastatic solid tumors. Selected tumor types included: malignant melanoma (MM), renal cell carcinoma (RCC), small cell and non-small cell lung cancer, urothelial carcinoma, head and neck carcinoma, and merkel cell carcinoma. Only irAE events graded above 2 were verified according to CTCAE V5.0 criteria. Statistical analysis used involved univariable cox proportional hazards model and Type III Wald Chi square p-values are reported for overall variable significance.

**Results**

Pt enrollment and data collection began in March 2017 and ended May 2019. We have enrolled 124 patients on our study; at the time of analysis 98 patients had at least 6 months of follow up. The most commonly reported irAEs were: thyroid dysfunction (23%), hepatitis (14%), rash (11%), and colitis (7%). In our population the majority of these patients were men (69.4%), identified as white and non-Hispanic (82.5%), and most had MM (40%). Numerous clinical variables were utilized in univariable binary logistic regression models to try to predict irAE development (Table 1). Tobacco use (p = 0.06) was the only variable that had a trend towards statistical significance in unadjusted analyses (Table 2).

**Conclusions**

Our results do show a similar incidence of irAEs in our population (42.4%) as compared to other studies [2,3]. Previous data in NSCLC demonstrated that tobacco smokers commonly have higher PD-L1 tumor proportion scores and can have a higher response rate to ICIs than never smokers, but the authors did not remark on irAE occurrence [4]. In our study, we did see a trend regarding pts who are current or recent tobacco users tend to develop less irAEs than never smokers. This finding will need to be explored on further analyses studying irAEs.

**References**

1. Sherman RE, Anderson SA, Pan GJ, et al. Real-world Evidence-What Is it and What Can it Tell us? NEJM. 2016;375:2293-97.

2. Wang DY, Salem JE, Cohen JV et al. Fatal Toxic Effects Associated with Immune Checkpoint Inhibitors, A Systemic Review and Analysis. JANA Oncol. 2018;4(12):1721-28.

3. Shoushtari AN, Friedman CF, Navid-Azabaijani P, at al. Measuring toxic effects and time to treatment failure for nivolumab plus ipilimumab in melanoma. JAMA Oncol. 2018;4(1):98-101.

4. Norum J, Nieder C. Tobacco smoking and cessation and PD-1 inhibitors in non-small cell lung cancer (NSCLC): a review of the literature. ESMO Open. 2018;3:e000406

**Ethics Approval**

The study was approved by the Loyola University Medical Center institutional Review Board, approval number LU209364.

**Table 1 (abstract P708).**
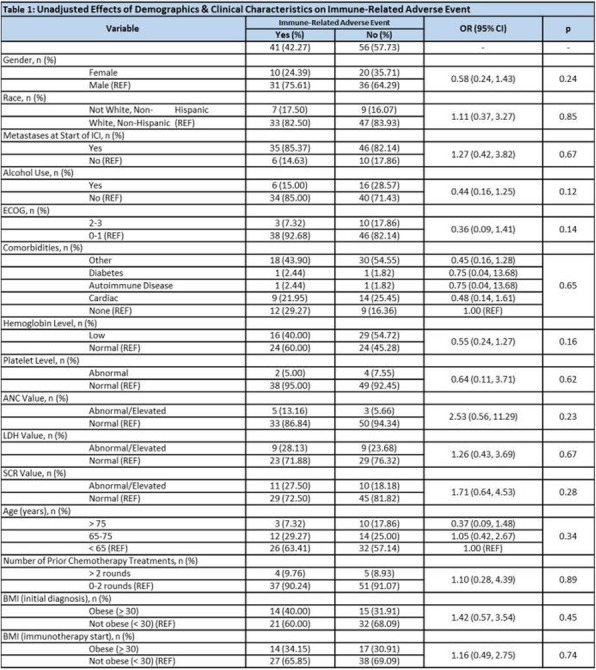
See text for description

**Table 2 (abstract P708).**
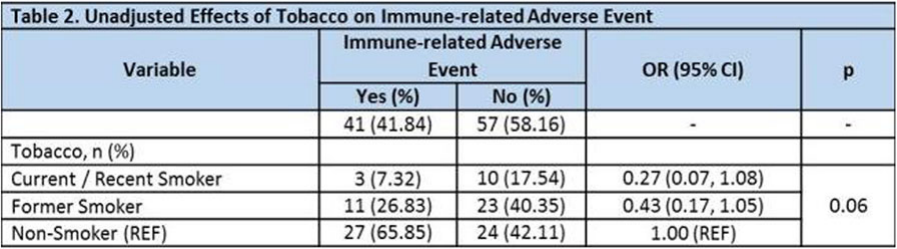
See text for description

#### P709 Immunotherapy adverse events predict treatment response

##### Diana Maslov, MD, MS^1^, Diana Maslov^1^, Katherine Thomas, MD, MS^2^, Victoria Simenson, MD^1^, Caitlin Sullivan, MD^2^, Alaa Mohammed, MPH^1^, Jessica Boyce^3^, Jonathan Lu, MD^3^, John Kucharczyk, MD^4^, Marc Matrana^3^

###### ^1^Ochsner Medical Center, Jefferson, LA, United States; ^*2*^*Louisiana State University, New Orleans, LA, United States* ; ^*3*^*Ochsner Clinical School, New Orleans, LA, United States* ; ^*4*^*New York University Winthrop Hospital, New York, NY, United States*

####### **Correspondence:** Diana Maslov (diana.maslov@ochsner.org)

**Background**

Immune checkpoint inhibitors have been approved in over 10 different types of malignancies. Immunotherapy blocks immunoinhibitory pathways, and allows for reversal of immunosuppression caused by malignant tumors [1]. Immunotherapy can cause immune-related adverse events (IrAE) including rash, pneumonitis, colitis, endocrinopathy, nephritis, adrenal insufficiency, hepatitis, and uveitis [2]. Limited data exists to predict which patients will have the greatest response to therapy. Additionally, the correlation between IrAE and immunotherapy effectiveness has not been well investigated. The aim of the current study is to determine the relationship between IrAE and immunotherapy efficacy.

**Methods**

Data was obtained using a retrospective chart review of Ochsner patients with metastatic cancer who received at least one cycle of immunotherapy (Nivolumab, Pembrolizumab, Atezolizumab, or Durvulamab) with no other drug combination between October 1st, 2014 and February 1st, 2019. Data collection included demographics, ECOG Performance Status recorded at the initiation of treatment, imaging results, time on treatment, best response to treatment, date of progression of disease, and presence or absence of IrAE while on treatment. Treatment response was analyzed using RECIST 1.1. Overall survival (OS) was calculated by the Kaplan-Meier survival method.

**Results**

A total of 456 patients were included for analysis. The median age was 67.6. The median time on immunotherapy for all patients was 112 months. Of the 456 patients, 175 (38.3%) had an IrAE while on immunotherapy. Hypothyroidism was the most prevalent adverse event (13.8%) followed by rash (10.5%). Out of 16 patients on Durvalumab, 12 had an IrAE (75%) (Table 1). The overall response rate (ORR) was 23.7% in the total population (Table 2). The development of IrAE correlate with OS and response rate. Of the patients with a reported IrAE, 58.3% had an overall response (OR), compared to 41.7% of those without IrAE (Table 2, Figure 1). Complete response (CR) occurred in 38 patients (8.3%), 21 (55.3%) of those with an IrAE, as compared to 17 (44.7%) of those without an IrAE. Partial Response occurred in 70 patients (15.4%), 42 (60.0%) of those with an IrAE as compared to 28 (40.0%) of those without an IrAE (Table 2, Figure 2). There is a significant increase in OS. At 24 months, 35 of 175 patients with IrAE were still on treatment compared to 26 out of 281 patients with no IrAE, p

**Conclusions**

Our results suggest that the presence of IrAE may represent a potential predictive indicator for treatment response to immune checkpoint inhibitors.

**References**

1. van den Bulk J, Verdegaal EME, de Miranda NFCC. Cancer immunotherapy: broadening the scope of targetable tumours. Open Biol. 2018; 8(6).

2. Ascierto et al. The role of immunotherapy in solid tumors: report from Campania Society of Oncology Immunotherapy (SCITO) meeting, Naples 2014. J Transl Med. 2014; 12:291.

**Table 1 (abstract P709).**
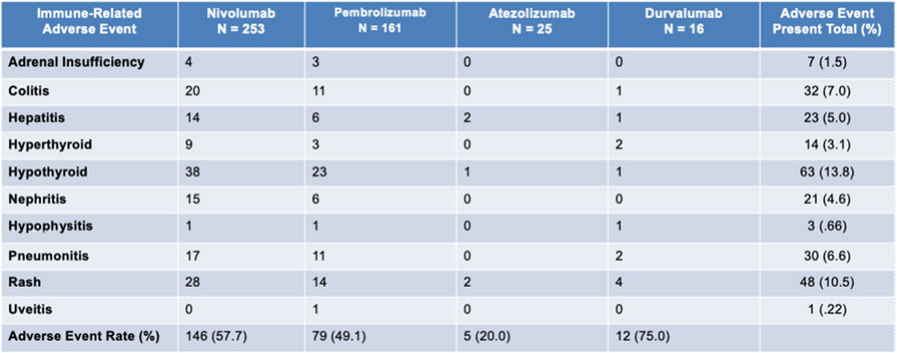
See text for description

**Table 2 (abstract P709).**
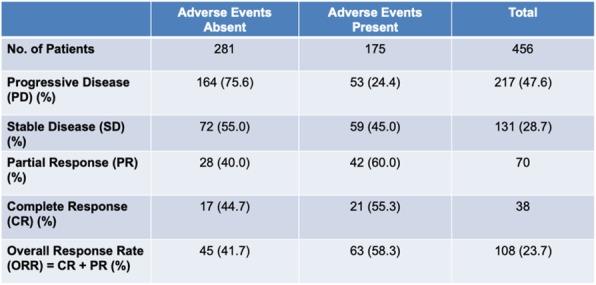
See text for description

**Fig. 1 (abstract P709).**
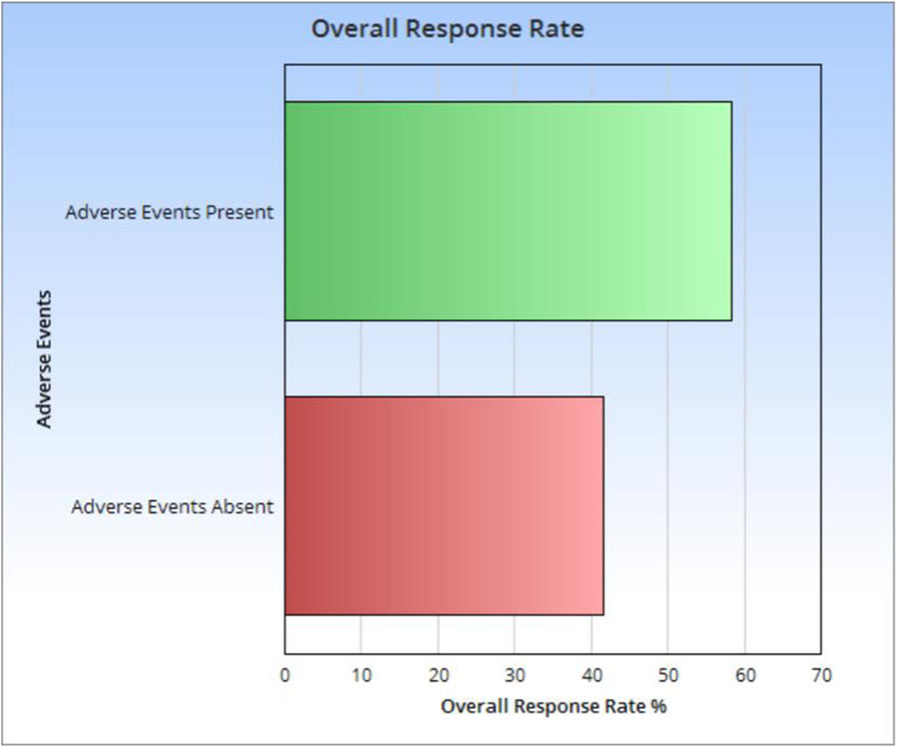
See text for description

**Fig. 2 (abstract P709).**
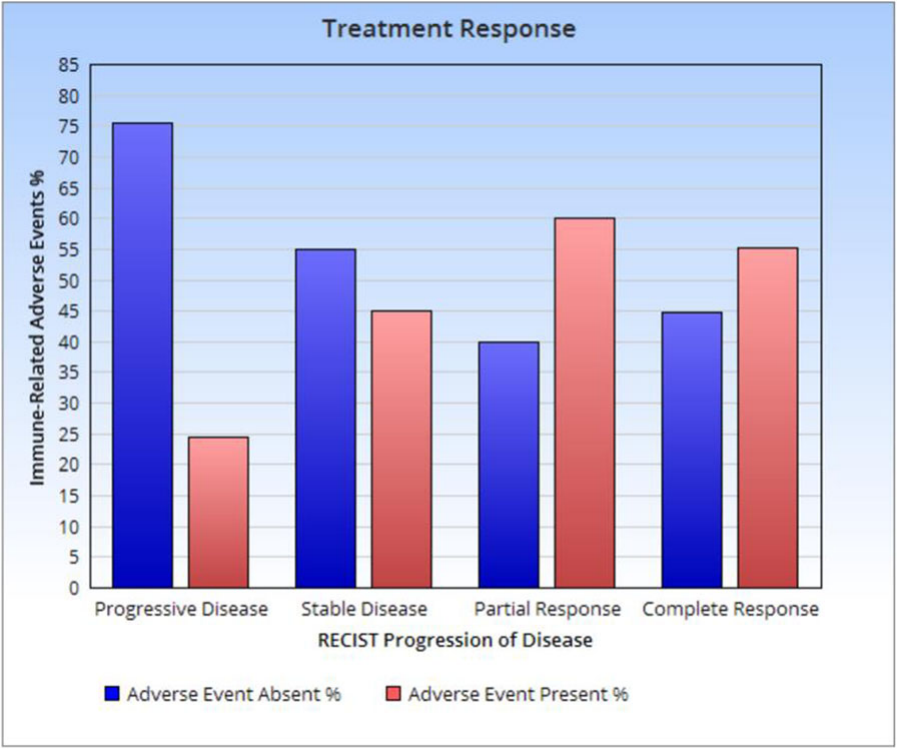
See text for description

**Fig. 3 (abstract P709).**
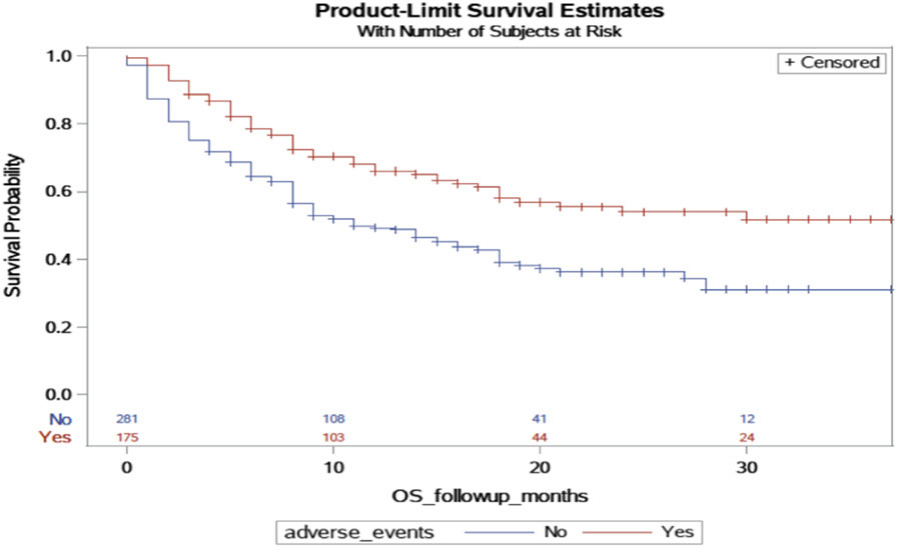
See text for description

#### P710 Immune checkpoint inhibitors increase the risk of hypothyroidism in patients with primary head and neck cancer compared to other cancers

##### Omar Alaber, MD, Apoorva Chandar, Prateek Mendiratta, MD, Monaliben Patel, MD, Chris Hoimes, MD, Pierre Lavertu, MD, Ankit Mangla, MD, Ankit Mangla, MD

###### Case Western Reserve University, Cleveland, OH, United States

####### **Correspondence:** Ankit Mangla (Ankit.Mangla@UHhospitals.org)

**Background**

Radiotherapy (RT) in patients with primary head and neck cancer (HNC) and those receiving immune checkpoint inhibitors (ICI) for any cancer, are independent risk factors for developing hypothyroidism. In this study, we sought to determine if the risk of hypothyroidism in patients with HNC who received RT, is increased by ICI compared to other primary malignancies (lung, gastrointestinal, skin, melanoma or urinary).

**Methods**

Data were obtained from a commercial de-identified database (Explorys, IBM, Inc.) that integrates electronic health records from 26 major integrated U.S. healthcare systems from 1999 to July 2019. Cases were defined as patients with a Systematized Nomenclature of Medicine Clinical Terms (SNOMED) diagnosis of primary HNC who received RT anytime between 1 and 90 days following their diagnosis and then received ICI (Nivolumab, Pembrolizumab, Atezolizumab, Durvalumab and/or Ipilimumab) 60 days after RT. Incidence of hypothyroidism was defined as anyone who had a new diagnosis of hypothyroidism at least 1 day following initiation of ICI. The comparison group comprised of patients with SNOMED diagnosis of primary lung, gastrointestinal, skin, melanoma or urinary cancer, who had received RT followed by ICI similar to cases and had a new diagnosis of hypothyroidism at least 1 day following initiation of ICI. All patients with previous/concurrent history of thyroid cancer, malignant lymphomas and hypothyroidism were excluded. Odds ratios (OR) and confidence intervals (CI) for the OR were calculated.

**Results**

We recorded 221,760 patients with an active diagnosis (within the last 5 years) of primary HNC, and 547,570 patients with an active diagnosis of a primary lung, gastrointestinal, skin, melanoma or urinary cancer in the database. Out of 260 HNC patients, 60 patients (23%) had a new diagnosis of hypothyroidism. Out of 1890 patients with other primary cancers, 290 (15%) had a new diagnosis of hypothyroidism. When compared to controls, cases had a 1.65 (CI = 1.2 – 2.3) times higher odds of developing hypothyroidism. Male cases and adults >65 years were significantly more likely to develop hypothyroidism.

**Conclusions**

The risk of developing hypothyroidism after ICI is higher in patients with HNC compared to other primary cancers. Men and older adults were more likely to become hypothyroid, but fewer numbers limit generalizability of our subgroup analyses. Other limitations included reliance on diagnosis coding, the retrospective nature of the study and lack of histological validation of SNOMED codes. Strengths include a strict definition of cases and controls and the inclusion of only incident hypothyroid patients.

**Fig. 1 (abstract P710).**
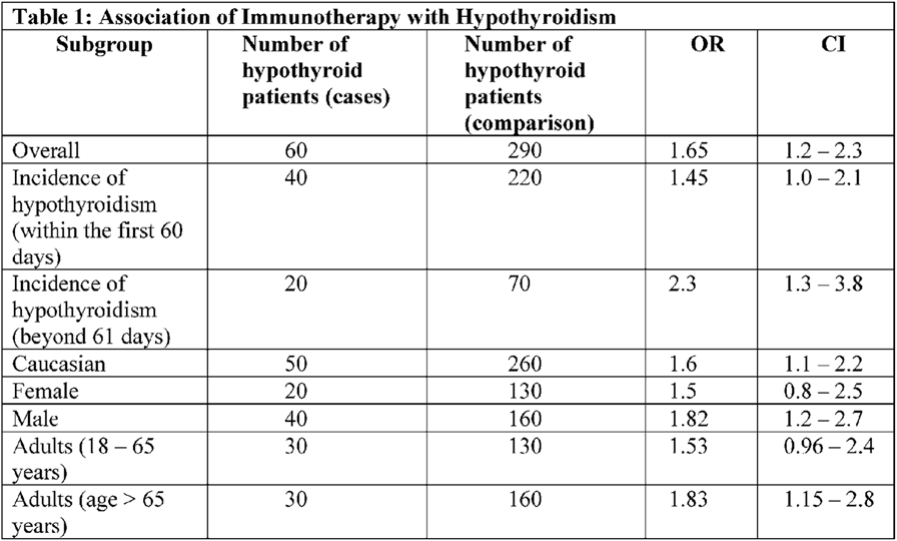
Association of Immunotherapy with Hypothyroidism

#### P711 Single-center analysis of safety and efficacy of immune checkpoint inhibitors (ICI) in non-hematologic malignancies: Effects of age and eosinophilia

##### Justin Moyers, MD^1^, Justin Moyers, MD^2^ , Dhanu Desai^3^, Jasmine Mitchell^1^, Jeffrey Ahn^1^, Steven Hardin^3^, Mie Mie Thinn^3^

###### ^*1*^*Loma Linda University HealthCare, Loma Linda, CA, United States;*^*2*^*Loma Linda University Medical Center, Loma Linda, CA, United States* ; ^*3*^*Veteran Affairs Loma Linda, Loma Linda, CA, United States*

####### **Correspondence:** Justin Moyers (jtmoyers@llu.edu)

**Background**

Trials conducted for ICI efficacy have largely included the young and healthy, while elderly patients have been excluded or under-represented.[1] In our clinic’s real-world population of predominantly elderly men, we aimed to compare the rate of clinically significant side effects and hospitalizations. There are no serologic markers for predicting response to ICI, but small series have suggested eosinophilia predicts improved survival.[2]

**Methods**

This is a retrospective observational study approved by the local IRB including all patients (n=147) who received ICI for non-hematologic malignancies from 2013-2019 at VA Loma Linda Healthcare System. Data were collected by chart review. Fisher’s exact test was performed on primary objectives of rate of hospitalization and grade 3-5 toxicities as graded by CTCAE between cohorts. The elderly cohort was defined as 70 years or greater, while younger cohort was defined as less than 70 years. Eosinophilia was defined as absolute count >5 x108/L on at least 2 occasions. Kaplan-Meier survival estimates were used to calculate secondary outcome of overall survival.

**Results**

There were 73 patients in elderly cohort and 74 in younger cohort. 28% of the older while 26% of the younger cohort (p=0.75) experienced immune-mediated grade 3-5 adverse events (AEs). During course of treatment, Elderly were hospitalized 50% vs 58% (p=0.36; HR=1.35) in younger population. AEs lead to a significantly increased rate of ICI discontinuation in 24% of the older population while only 10% in the younger cohort (p=0.02; HR=0.33). In metastatic melanoma (n=36) survival between the younger (n=21) and elderly (n=15) cohorts were similar with median OS of 24.9 and 27.8 months (p=0.77), respectively. Similarly, survival in those with NSCLC (n=51) survival were 16.3 and 16.9 months (p=0.86), respectively for younger (n=21) and elderly (n=30) cohorts. Results are summarized in Table 1.

An exploratory analysis examined presence of eosinophilia and survival amongst all ages. In metastatic melanoma (n=36), those with eosinophilia (n=12) had a median overall survival of 42.0 vs 8.2 months (p=0.03) (Figure 2). In metastatic non-small cell lung cancer (n=51) those with eosinophilia (n=11) had a trend towards improved survival 20.8 vs 9.8 months (p=0.08).

**Conclusions**

Elderly patients in our cohort did not experience more frequent grade 3-5 AEs or hospitalization, but more elderly patients discontinued treatment due to AEs. Intriguingly, a significant increase in overall survival was seen in those with eosinophilia during ICI therapy in metastatic melanoma.

**References**

1. Kanesvaran, R., R. Cordoba, and R. Maggiore, Immunotherapy in Older Adults With Advanced Cancers: Implications for Clinical Decision-Making and Future Research. Am Soc Clin Oncol Educ Book, 2018(38): p. 400-414.

2. Bernard-Tessier, A., et al., Immune-related eosinophilia induced by anti-programmed death 1 or death-ligand 1 antibodies. Eur J Cancer, 2017. 81: p. 135-137.

**Ethics Approval**

The study was approved by VA Loma Linda Healthcare System Institutional Review Board, protocol #1290.

**Table 1 (abstract P711).**
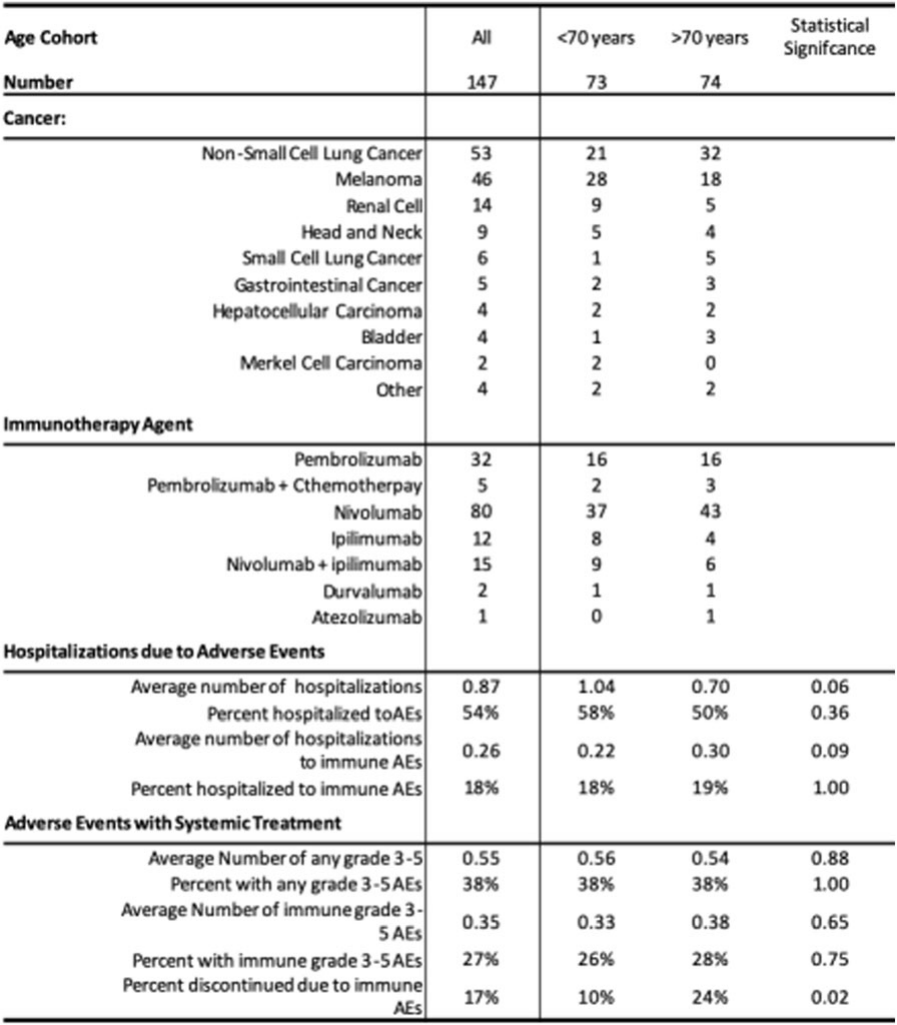
See text for description

**Fig. 1 (abstract P711).**
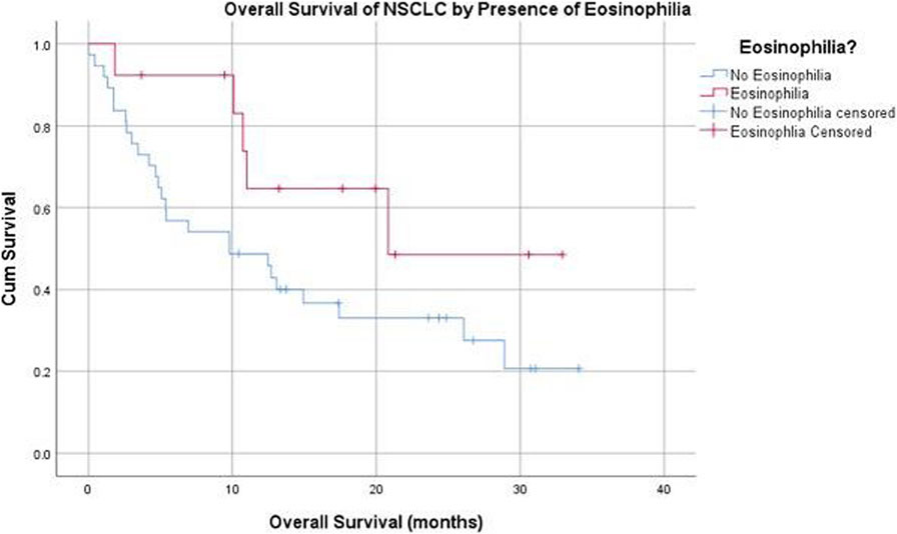
See text for description

#### P712 Conditional immune toxicity rate with immune checkpoint inhibitors

##### Pier Vitale Nuzzo, MD, PhD^1^, Gregory Russell Pond, MSc, PhD^2^, Amin Nassar, MD^2^, Sarah Abou Alaiwi, MD^2^, Ronan Flippot, MD^2^, Catherine Curran^2^, Kerry Kilbridge, MD^2^, Xiao Wei, MD^2^, Bradley Mcgregor, MD^2^, Lauren Harshman, MD^2^, Toni Choueiri^2^, Guru Sonpavde, MD^2^

###### ^1^Dana-Farber Cancer Institute, Boston, MA, United States; ^*2*^*McMaster University, Hamilton, Canada*

####### **Correspondence:** Guru Sonpavde (GuruP_Sonpavde@DFCI.HARVARD.EDU)

**Background**

Treatment with immune checkpoint inhibitors (ICIs) can result in immune-related adverse events (irAEs). irAE cumulative incidence (CI) rate is a measure that accounts for elapsed time since treatment initiation and estimates the risk of irAE development conditioned on time elapsed without experiencing an irAE. We sought to evaluate the CI of irAEs in metastatic urothelial carcinoma (mUC) and metastatic renal cell carcinoma (mRCC) patients treated with ICI.

**Methods**

We identified a cohort of mUC and mRCC patients who received ICI at Dana-Farber Cancer Institute (DFCI). The severity of irAEs was graded using CTCAE v.5.0. The CI of irAE was calculated accounting for the competing risk of death. Monthly landmark intervals were determined, and the monthly incidence and CI of irAEs at each landmark time was calculated based on the ratio of patients with irAEs after the landmark time if alive with no prior irAEs at landmark time.

**Results**

A total of 470 patients were treated between July 2013 and October 2018 [199 mUC (42.3%); 271 mRCC (57.7%); median age, 65 (22-91) years; 342 men (72.8%)]. Overall 341 (72.6%) received ICI monotherapy, 86 (18.3%) ICI+ other class of agents, and 43 (9.2%) combination ICI. 311 (66.2%) patients were treated with anti-PD1 and 159 (33.8%) with anti-PD-L1 agents. 207 (44%) received ICI as 1st line therapy and 263 (56%) as 2nd or later line therapy. IrAEs of any grade were observed in 186 patients (39.5%) overall at any point in time (Table). Common irAEs included hypothyroidism (n=42 [22.6%]), rash/pruritus (n=36 [19.4%]), colitis (n=35 [18.8%]), transaminitis (n=32 [17.2%]), and pneumonitis (n=14 [7.5%]). The risk of developing an irAE over time was as follows: 33.5% if no irAE within the 1st month (mo), 27.3% if no irAE in 3mo, 18.8% if no irAE in 6mo, and 16.4% if no irAE by 12mo. The monthly incidence of irAEs is shown in the Table. No difference was observed based on type of cancer (mUC vs mRCC) or agent (PD1 vs. PD-L1).

**Conclusions**

This is the first study to our knowledge that quantitates the incidence of developing irAEs with ICI conditioned on time elapsed without development. Although the incidence of irAEs decreased over time on therapy, irAEs require continuous vigilant monitoring because of the long tail in its incidence. Further evaluation of the conditional irAE rate is warranted in prospective studies in conjunction with evaluation of predictors of early and late irAEs.

**Ethics Approval**

The study was approved by Dana Faber Cancer Institutution’s Ethics Board, approval number 02-021 /17-000.

**Table 1 (abstract P712).**
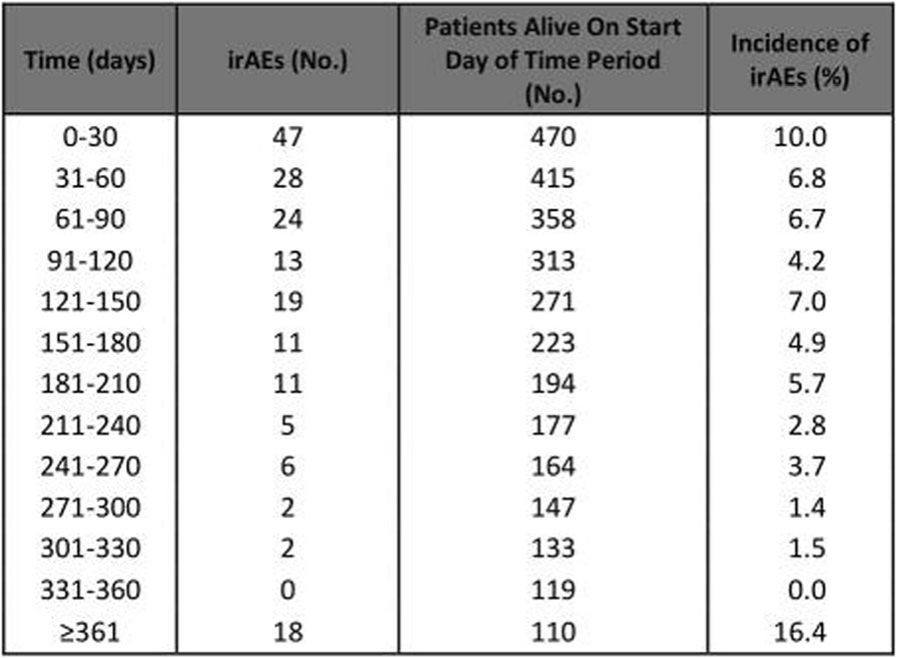
See text for description

#### P714 Immunological features of immune-related adverse events during anti-PD-1/PD-L1 immune checkpoint inhibition

##### Meghali Goswami, MPhil, Gege Gui, Katherine Lindblad, Jaydira Del Rivero, MD, Jennifer Marte, BS MD, Pradeep Dagur, PhD, Christin Destefano, MD, Julie Thompson, RN, Bogdan Popescu, MD, Laura Dillon, PhD, Cheryl Johnson, Steven Soldin, PhD, Catherine Lai, MD, MPH, James Gulley, MD, PhD, FACP, Christopher Hourigan, MD, PhD

###### National Cancer Institutes, Bethesda, MD, United States

####### **Correspondence:** Christopher Hourigan (hourigan@nih.gov)

**Background**

Immune checkpoint inhibition (ICI) against PD-1/PD-L1 has shown remarkable efficacy in many solid tumors. However, the PD-1 checkpoint is critical for peripheral tolerance, and endocrine toxicities are common immune-related adverse events (irAEs) of PD-1/PD-L1 inhibitors.

**Methods**

We characterized immune features in relapsed/refractory acute myeloid leukemia (AML) patients who developed irAEs during treatment with pembrolizumab and decitabine (PD-AML, NCT02996474). Two of 10 patients developed hypothyroidism, a known irAE. A third developed central diabetes insipidus, thought possibly related to pembrolizumab. TCRb sequencing was performed using the ImmunoSEQ platform (Adaptive Biotechnologies) on gDNA from longitudinal blood and bone marrow samples of AML patients. We performed 10x Genomics 5’ single-cell RNA / VDJ sequencing (scRNAseq) and cell surface protein profiling with oligo-conjugated antibodies (Biolegend) on FACS-purified CD3+ T cells from mononuclear cells of an AML patient at hypothyroidism diagnosis. In a cohort of 8 patients who developed hypothyroidism during anti-PD-1/PD-L1 treatment, including the 2 AML patients and 6 patients with solid tumors receiving avelumab on NCT01772004, we tested for anti-thyroid autoantibodies and performed HLA sequencing.

**Results**

We first queried for the presence of circulating anti-thyroid autoantibodies. At onset of hypothyroidism, 3/8 hypothyroidism patients had either elevated anti-thyroid peroxidase and/or anti-thyroglobulin autoantibodies.The 2 AML patients had near undetectable levels. Sequencing of HLA loci did not reveal any consistent overlap. We next examined TCR repertoire dynamics in AML patients during treatment. Through TCRb sequencing, we identified several expanded T cell clonotypes whose expansion coincided with irAE onset in all 3 AML irAE (Figure 1). To further explore genetic and proteomic signatures of these expanded clones in a representative AML hypothyroidism patient, 8578 T cells were sequenced with scRNAseq. All expanded TCRb clonotypes of interest were captured and paired TCRa chains identified. Most clones were CD8+ and expressed high levels of cell surface PD-1, TIM-3, HLA-DR, and CD27, with no CCR7 and moderate CD45RO, indicating an activated effector memory phenotype (Figure 2). These clonotypes had high PDCD1 and CD27 transcripts as well and an expression profile suggestive of activation, proliferation, and degranulation.

**Conclusions**

We performed a detailed characterization of immunological and molecular features accompanying the development of irAEs during anti-PD-1 ICI and identified specific T cell clonotypes that may have contributed to anti-PD1-induced irAEs in relapsed/refractory AML patients. Our data suggest that several immunological mechanisms may mediate irAEs. We believe irAEs offer a model system for discerning mechanisms of action of PD-1/PD-L1 inhibitors on T cells.

**Acknowledgements**

This research is supported in part by the Intramural Research Program of the NHLBI, NIH and the Center for Human Immunology, NIH.

**Ethics Approval**

NCT02996474 was approved by the NHLBI IRB (17-H-0026) and NCT01772004 by the NCI IRB (13-C-0063).

**Fig. 1 (abstract P714).**
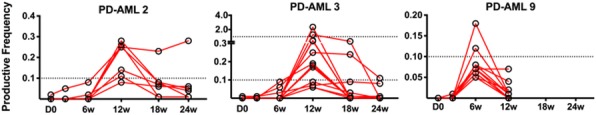
See text for description

**Fig. 2 (abstract P714).**
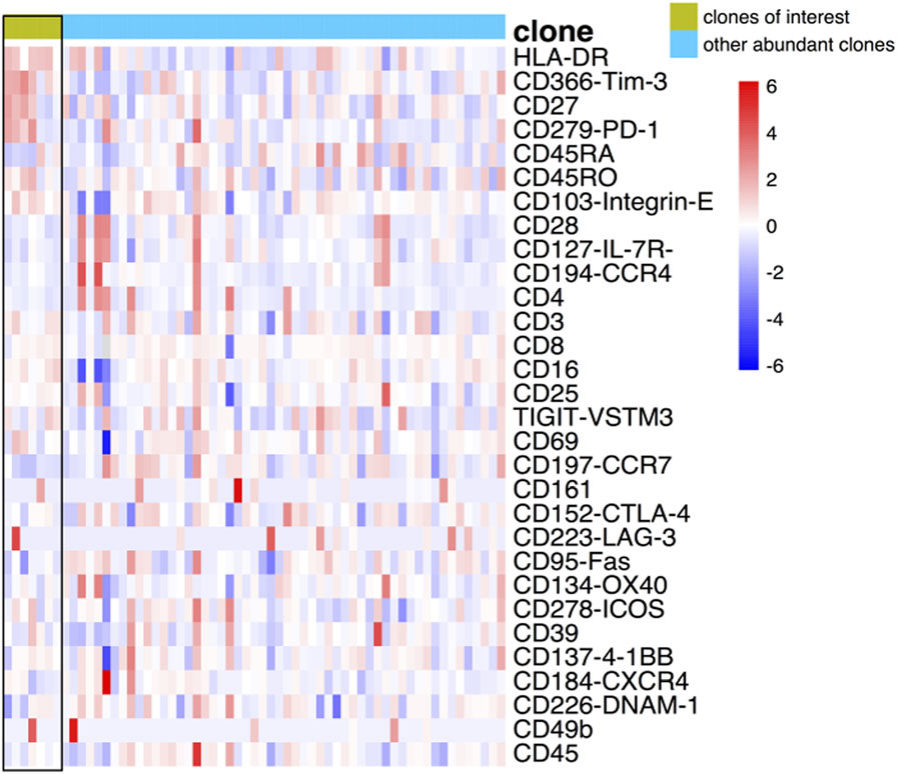
See text for description

#### P715 Strategies to mitigate immune-related adverse events of efficacious combination immunotherapies in preclinical models of cancer

##### Célia Jacoberger-Foissac, PhD, Stephen Blake, Jing Liu, Stacey Allen, Juming Yan, Heidi Harjunpaa, Mark Smyth, Michele Teng, PhD, Michele Teng, PhD

###### QIMR Berghofer Medical Research Institute, Brisbane, Australia

####### **Correspondence:** Michele Teng (michele.teng@qimrberghofer.edu.au)

**Background**

The clinical success of checkpoint inhibitors targeting CTLA-4 and PD-1/PD-L1 alone or in combination has paved the way for the development of new immunotherapy options and combination therapies. In addition to checkpoint inhibitors, agonistic antibodies against co-stimulatory receptors, such as CD40 or CD137, are currently being evaluated clinically. In preclinical studies, different agonistic antibodies have been shown to strongly complement the activity of checkpoint inhibitors to enhance T/NK-cell clearance of tumours. However, mouse models failed to predict the severe toxicities induced by anti-CD137 during early clinical trials, which can limit their clinical utility in combination therapies.

**Methods**

We recently described a model whereby transient depletion of T regulatory cells (Tregs) using the FoxP3-GFP-DTR mice, prior to treatment with immune modulating antibodies, increased physical and biochemical immune related adverse events (irAEs) similar to what is observed in humans.

**Results**

In this study, we demonstrated how Treg depletion can model clinical irAES and investigate methods to alleviate the toxicity induced by agonistic anti-CD40 and anti-CD137. First we demonstrated that biochemical irAEs such as increased inflammatory cytokines and liver toxicities were observed after transient Treg depletion and anti-CD40 and its severity varied depending on the mouse strain. We further demonstrated that treatment of these mice with anti-TNF reduced irAEs severity such as liver toxicity. Next, we demonstrated that Treg depletion specifically in the tumor in combination with systemic anti-CD137 suppressed tumour growth without inducing severe irAEs contrary to peripheral Treg depletion and anti-CD137 treatment where all mice died due to severe irAEs.

**Conclusions**

This study further highlights how T-reg depletion can be used to rapidly model clinically observed irAES and investigate methods to alleviate the toxicity of potent immunotherapies

#### P716 An in vivo PBMC humanized mouse model for determining checkpoint and bispecific antibody treatment related cytokine release syndrome

##### Chunting Ye^1^, Mingshan Cheng^1^, Michael Brehm^2^, Dale Greiner^2^, Leonard Shultz^1^, James Keck, PhD^1^, James Keck, PhD^1^

###### ^1^The Jackson Laboratory, Sacramento, CA, United States; ^*2*^*University of Massachusetts Medical Scho, Worcester, MA, United States*

####### **Correspondence:** James Keck (james.keck@jax.org)

**Background**

Although antibodies and CAR-T cell therapies have been successfully used for cancer treatment, they can have significant adverse effects such as cytokine release syndrome (CRS). The animal models and in vitro human PBMC assays presently in use unfortunately do not reliably predict the occurrence of CRS in patients. This significant gap between pre-clinical testing of novel therapeutics and clinical trials demonstrates a critical need for translational protocols that more accurately predict immune toxicity.

**Methods**

We have developed a rapid, sensitive and reproducible in vivo humanized mouse model for quantitating CRS. NSG mice and derivative strains are engrafted with human PBMCs for 6 days then treated with various therapeutics such as bispecifics, pembrolizumab, avelumab, atezolizumab, ipilimumab, and anti-CD28 or OKT3 antibodies to determine their cytokine release levels in the mouse 6 hours later. In addition to single agent therapies, drug combinations with pembrolizumab, lenalidomide, ATG and anti-CD28 were also performed

**Results**

At day 5 of PBMC engraftment, levels of human CD45+ cells ranged between 10 to 15% CD45+ human cells in peripheral blood consisting of approximately 70% CD3 T cells and 25% CD56 NK cells. All FACS tested cytokines, human IFN-γ, IL-2, IL-4, IL-6, IL-10 and TNF were upregulated 6 hours after injection with OKT3, ATG, anti-CD28, various checkpoint inhibitor and bispecific antibody drug treatments. Rectal temperatures of treated mice dropped from 37 to 38°C to about 36 °C at 6 post treatment. Clinical observations of mice showed health related issues with OKT3 and bispecific antibody treatment. Using multiple PBMC donors, variable cytokines release levels were evident with anti-CD28 treatment while most donors showed a high response to OKT3. The cytokine release levels were also determined to be consistent with a dose response or variable PBMC engraftment. The cytokine levels were also higher in some PBMC donors with drug combination studies such as pembrolizumab combined with lenalidomide or ATG; anti-CD28 combined with ATG. Direct comparison of the newly developed vivo model with a standard in vitro CRS assay demonstrated an increased sensitivity and accuracy in predicting CRS.

**Conclusions**

We have developed a rapid, sensitive and reproducible novel in vivo PBMC humanized mouse model that is able to differentiate human PBMC donors based on individual safety response to single agent and combination therapeutics of immune checkpoint inhibitors and possibly bispecific antibody and CAR-T therapy. Together these data indicate that our newly developed in vivo CRS assay has clinical relevance for future drug development.

**Ethics Approval**

The study(#16000) was approved by The Jackson Laboratory IACUC committee on 3/12/19 and expires 3/12/22

#### P717 Involvement of IL17 in a case of fatal neuroendocrine toxicity associated with dual immune checkpoint blockade

##### Luca Mazzarella, MD PhD^1^, Silvia Giugliano^2^, Paolo D'Amico^1^, Carmen Belli^1^, Bruno Duso^1^, Giuseppe Curigliano, MD, PhD^1^ , Maria Rescigno, MD, PhD^2^

###### ^1^IEO Istituto Europeo di Oncologia IRCCS, Milan, Italy; ^2^Istituto Humanitas, Rozzano (MI), Italy

####### **Correspondence:** Giuseppe Curigliano (giuseppe.curigliano@ieo.it)

**Background**

Severe neurological and endocrine toxicities are well recognized adverse events of immune checkpoint inhibitors (ICI). However, the underlying pathophyisology is poorly understood and classical circulating markers are often non-informative, making it difficult to obtain a precise diagnosis and to initiate timely and effective treatment.

**Methods**

We studied the case of a patient who developed fatal combined neurological and endocrine toxicity while on treatment with dual immune checkpoint blockade for his metastatic sarcomatoid mesothelioma. The syndrome was characterized by insulin-dependent diabetes, hypophisitis (see figure 1) with impairment of multiple endocrine axes and a myasthenia-like syndrome with predominant involvement of cranial districts. We tested in vitro dendritic cell (DC) activation upon incubation with patient's plasma. By cytokine bead array (CBA), we compared patients' levels of circulating cytokines (IFNg, IL4, IL5, IL6, IL13, IL17A and TGFb) with those in healthy subjects and cancer cases with comparable treatment history but no toxicity

**Results**

Incubation with patient's plasma resulted in upregulation of multiple DC activation markers, most prominently CD25 and CD11b. Levels of IL17A in patients' plasma (18.3 ± 5.6 pg/ml) were significantly higher than in healthy controls (1.9 ± 1.9 pg/ml) or cancer cases with no toxicity (1.3 ± 1.8 pg/ml) (p=0.04 by one-way ANOVA, figure 2)

**Conclusions**

This is the first clinical report of an association between ICI-related neuroendocrine toxicity and elevation of IL17A, a Th17-produced cytokine involved in numerous autoimmune disorders of the central nervous system. If confirmed in larger series, this findings may point to a role for IL17A as a diagnostic and therapeutic target in the management of severe neuroendocrine toxicities.

**Ethics Approval**

The study was approved by the IEO Institutution’s Ethics Board through its procedure for observational studies, approval number 2062.

**Fig. 1 (abstract P717).**
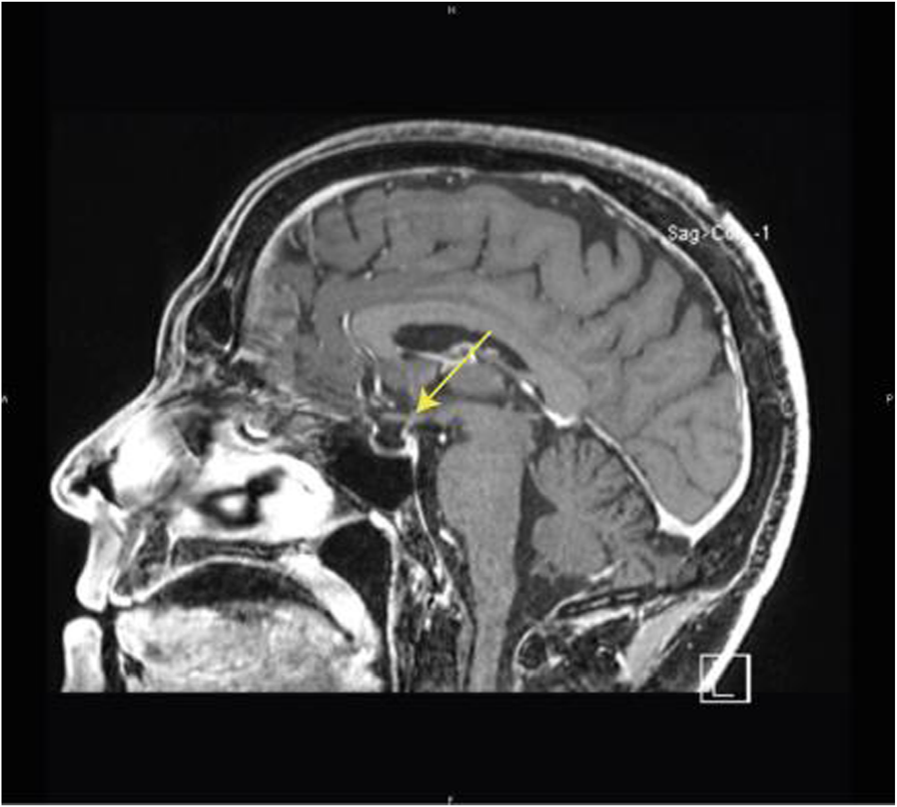
Brain RMN

**Fig. 2 (abstract P717).**
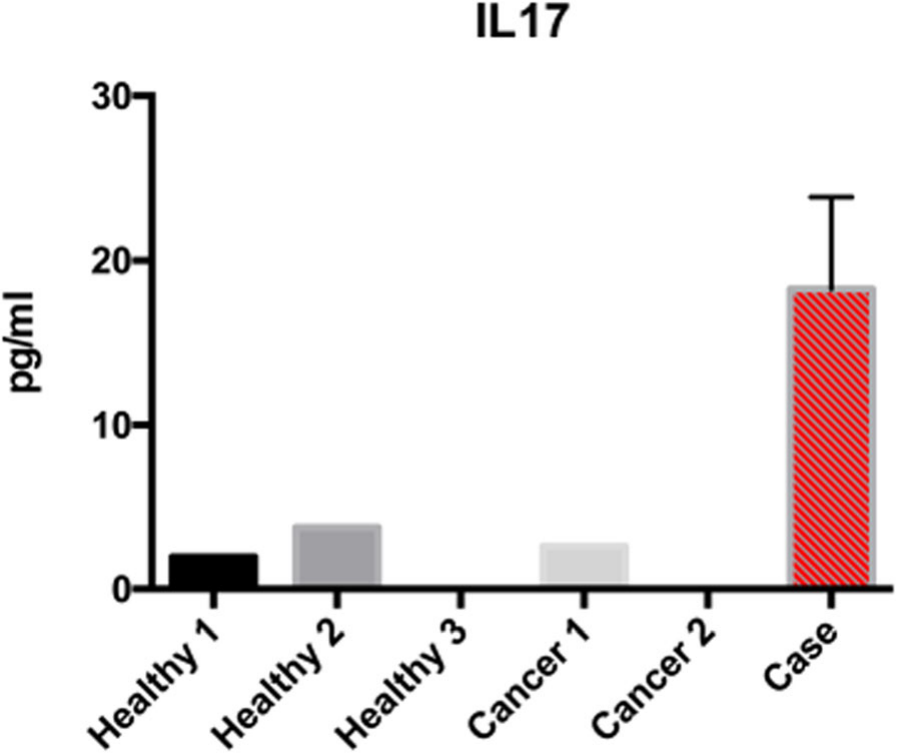
IL17A plasma levels

#### P718 A case of dual-mechanism hematologic immune-related adverse event in a patient with metastatic melanoma treated with combined nivolumab and ipilimumab

##### Daniel Olson, MD^1^, Daniel Olson, MD^1^ , Melissa Tjota, MD, PhD^1^, Padma Rajagopal, MD^1^, Girish Venkataraman, MD^1^, Jason Luke, MD, FACP^2^, Thomas Gajewski, MD, PhD^1^

###### ^1^The University of Chicago, Chicago, IL, United States; ^2^The University of Pittsburgh, Pittsburgh, PA, United States

####### **Correspondence:** Daniel Olson (dolson2@medicine.bsd.uchicago.edu)

**Background**

The combination of the immune checkpoint inhibitors (ICI) anti-CTLA-4 (ipilimumab) and anti-PD-1 (nivolumab) has become a mainstay of treatment for select patients with metastatic melanoma. The use of combination ICIs, while demonstrating higher levels of anti-tumor activity over either ICI alone, also results in more frequent immune-related adverse events (irAEs) [1]. While the CTLA-4 and PD-1 pathways regulate autoimmunity at different stages of an immune response, the exact mechanisms by which ICIs generate specific irAEs is not yet well-understood.

**Methods**

Here we report a case of simultaneous T cell-mediated red cell aplasia and antibody-mediated auto-immune hemolytic anemia (AIHA) in a patient receiving combined ICI therapy for metastatic melanoma.

**Results**

A 29-year-old woman with melanoma metastatic to the small bowel presented with worsening fatigue after completing four cycles of combination nivolumab and ipilimumab. Laboratory results were strongly suggestive of AIHA, including: a hemoglobin of 6.1g/dL, undetectable haptoglobin, elevated lactate dehydrogenase, and a positive Coombs test. However, the reticulocyte count was abnormally low for the degree of anemia (reticulocyte production index 0.0). She was given transfusion support and started on high-dose intravenous, then oral steroids. By the following visit, the hemoglobin fell to 5.5g/dL with ongoing reticulocytopenia. Intravenous steroids were again initiated and a bone marrow biopsy was performed; this demonstrated a substantial T cell infiltrate. The marrow core biopsy sections were hypercellular with an atypical lymphoid infiltrate. 25% of all cells stained as CD8+, and the CD4:CD8 ratio was abnormally shifted to 1:3, showing predominance of CD8+ T cells. Perforin was also increased suggesting a cytotoxic T cell phenotype. These observations were consistent with T cell-mediated destruction of the absent red cell precursors. Given lack of reticulocyte response to corticosteroids, T cell-directed immunosuppression was initiated with cyclosporine A along with continued steroid administration for AIHA. The reticulocyte count increased over the next week; within one month the hemoglobin level had normalized. A steroid taper was completed, and all immunosuppression was ceased within three months. Upon repeat imaging, the patient experienced a complete response to ICI therapy and remains in observation alone.

**Conclusions**

This report describes a rare case of two concurrent hematologic irAEs in a patient treated with combined ICI therapy: 1) auto-antibody mediated AIHA and, 2) CD8+ T cell-mediated red cell aplasia. Successful treatment resulted only after the second underlying mechanism of toxicity was uncovered. Prompt recognition of these unusual presentations of irAEs are now is now key to effective irAE management.

**References**

Wolchok JD, Chiarion-Sileni V, Gonzalez R, et al. Overall Survival with Combined Nivolumab and Ipilimumab in Advanced Melanoma. N. Engl. J. Med. 2017 Oct 5;377(14):1345–56.

**Ethics Approval**

Institutional Review Board protocol is not required at The University of Chicago for a single case report.

**Consent**

Individual patient consent was not applicable as no information in this report can be categorized as identifiable.

#### P719 CD8+ Tcell integrin α4β7expression: a potential predictor of severity and steroid sensitivity in checkpoint inhibitor induced colitis

##### Anna Olsson-Brown, MBChB (Hons), BSc (Hons)^1^, Joseph Sacco, MSc, MBChB, MRCP, Ph^1^, Anna Olsson-Brown, MBChB (Hons), BSc (Hons)^1^ , Anna Olsson-Brown, MBChB (Hons), BSc (Hons)^1^, Sandra Cachinho^1^, Carol Jolly^1^, Vanessa Fontana^1^, Rosemary Lord^2^, Mark Coles^3^, Munir Pirmohamed^1^

###### ^*1*^*University of Liverpool, Clatterbridge Cancer Centre, Liverpool, United Kingdom;*^*2*^*Clatterbridge Cancer Centre, University of Liverpool, Liverpool, United Kingdom* ; ^*3*^*University of Oxford, Oxford, United Kingdom*

####### **Correspondence:** Anna Olsson-Brown (acob@liv.ac.uk)

**Background**

Colitis a common immune related adverse events (irAEs) associated with immune checkpoint inhibitor (ICIs) therapy with severe toxicity occurring in up to 25% of patients receiving therapy[1]. It is one of the most complex irAEs to treat often requiring inpatient management and the use of additional immunosuppression beyond corticosteroids. The use of infliximab is widely accepted but use of gut specific agents such as the anti-integrin therapy vedolizumab is increasing. The rationale is that as a highly gut targeted therapy it presents the potential for therapeutic benefit without risking impact on oncological efficacy. However the evidence for this remains limited and patient selection unclear.

**Methods**

As part of an established observational study peripheral PBMCs were isolated from patients presenting with colitis secondary to ICI therapy for malignant melanoma. Samples were taken at the onset of colitis at the time of endoscopic investigation. Control patients had received at least 4 months of ICI therapy without any irAEs. Those with colitis were assessed for severity via endoscopic/ histological assessment and followed up to determine the duration of corticosteroids required. Mass Cytometry utilising a Fluidigm Helios (CyTOF3) platform was undertaken with a 16 marker panel including 12 cellular markers and 4 gut homing markers (Table 1). Cytometry analysis was undertaken using Cytobank software. Data was analysed using ANOVA in GraphPad Prisim v8.2.0.

**Results**

A total of 18 patient samples were analysed (12 colitis patients and 6 controls). Colitis patients were stratified by endoscopic/histological severity and duration of steroid therapy (80 days). The populations of CD8, CD4 and Treg populations were not found to be significantly different between affected patients and tolerant controls. With regards to the gut homing markers the expression of the α4β7 integrin heterodimer was found to be differentially expressed on peripheral CD45+CD3+CD8+ cells. There was a significant difference in expression in those found to have mild and severe colitis on investigation with those having severe endoscopy findings having an elevated α4β7 expression (p=0.0315). Additionally there was an increased expression of α4β7 in patients who required long-term steroids compared to those requiring short term steroids (p=0.0105)

**Conclusions**

This is preliminary data however the differential expression of Cd45+CD3+CD8+ α4β7 at the initiation of toxicity highlights its potential to predict steroid sensitivity. Given the increased expression of α4β7 in patients with severe investigative findings could endorse the use of anti-integrin directed therapy in patients with investigation-diagnosed severe ICI-induced colitis.

**Acknowledgements**

Dr Anna Olsson-Brown is a MRC Clinical Research Fellow based at the University of Liverpool and is supported by the North West England Medical Research Counical Fellowship Scheme in Clinical Pharmacology and Theraputics which is funded by the medical research council (Award Ref MR/N025989/1) Roche Pharma, Eli Lily and company limited, UCB Pharma, Novartis, the University of Liverpool and the University of Manachester. The authors would also like to acknowledge the support of the cell sorting and isolation facility, Technology Directorate, University of Liverpool.

**References**

1. Larkin J, Chiairon-Sileni V, Gonzalez R et al. Combined Nivolumab and Ipilimumab or Monotherapy in untreated melanoma. N Enjl J Med. 2015. 373:23-34

**Ethics Approval**

Patient samples and clinical data were collected under the observational study ‘A Mechanistic Investigation into Drug and Chemical Induced Hypersensitivity Reactions’ - REC Ref: 12/NW/0525 UKCRN ID: 13348

**Consent**

Data and samples taken as part of the ‘A Mechanistic Investigation into Drug and Chemical Induced Hypersensitivity Reactions’ - REC Ref: 12/NW/0525 UKCRN ID: 13348 and consent gained for entry into the trial.

**Table 1 (abstract P719).**
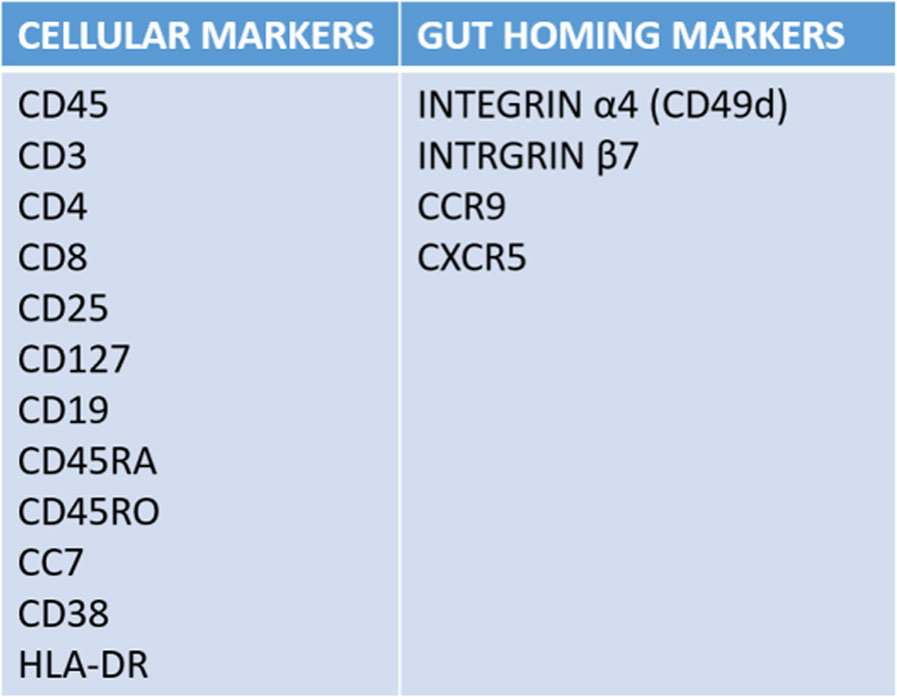
Mass Cytometry Panel

#### P720 The use of in vitro assays to reduce the risk of unwanted immunogenicity

##### Amin Osmani^1^, Thibault Jonckheere^2^, Sofie Pattijn^2^, Mayuko Oh^2^, Jana Schockaert, MS^2^

###### ^1^ImmunXperts, Charleroi, Belgium; ^2^ImmunXperts SA, Gosselies, Belgium

####### **Correspondence:** Amin Osmani (amin.osmani@immunXperts.com)

**Background**

Protein therapeutics represent a significant proportion of the currently available therapies for a broad spectrum of diseases and is one of the fastest growing markets. However, the development of new therapeutics comes with a series of challenges and risks.

**Methods**

One major hurdle in the therapeutic development is the risk for unwanted immunogenicity which can lead to decreased efficacy and safety concerns. Although a large proportion of oncology drug is recognized to induce an unwanted immune response, less is known about the consequences on safety and efficacy and potential adjuvant effect. Today, several tools are available to improve and accelerate therapeutic drug development in an early stage. A well balanced and rational development strategy can help to design less immunogenic drugs and reduce the number of clinical failures.

**Results**

Often used as a first step is an In silico T cell epitope prediction algorithm such as NetMHCpan which can be used to assess and compare the immunogenic potential of the lead candidates and guide de-immunization strategies. Further monitoring of the immunogenic risk can be performed using in vitro T cell proliferation assays to determine and rank the immunogenic risk of the test proteins or identify specific regions of concern. However, given the intrinsic capacity of certain immune check point blocking antibodies to stimulate T cells, assay protocols might need to be adapted and fine-tuned to allow the discrimination between a T-cell epitope driven response and a proliferative effect caused by ‘releasing the breaks of the immune system’. Next to the T cell assays, the MAPPS assay (MHC Associated Peptide Proteomics) is a useful assay to identify the actual number of T cell epitopes presented by professional antigen-presenting cells.

**Conclusions**

Another important step is the compilation of the different datasets and translation of the results into a comprehensive risk management plan, allowing further de-risking of test candidates and selection of the best candidates to move forward into humans. This step also allows to identify and discontinue the high-risk candidates as early as possible.

#### P721 Impact of antibiotic therapy on the development and response to treatment of immune checkpoint inhibitor-mediated diarrhea and colitis

##### Hamzah Abu-Sbeih, MD^1^, Lauren Herrera^2^, Tenglong Tang, MD^1^, Mehmet Altan^1^, Anne-Maria Chaftari^1^, Pablo Okhuysen^1^, Robert Jenq, MD^1^, Yinghong Wang, MD, PhD^1^

###### ^1^Univ of Texas MD Anderson Cancer Center, Houston, TX, United States; ^*2*^*Baylor College of Medicine, Houston, TX, United States*

####### **Correspondence:** Yinghong Wang (YWang59@mdanderson.org)

**Background**

The gut microbiome impacts the efficacy of immune checkpoint inhibitor (ICI) therapy and the development of ICI-mediated diarrhea and/or colitis (IMDC). Antibiotic therapy especially that with anaerobic activity, has profound effects on the gut microbiome. Therefore, we sought to assess the effect of antibiotics on the development of IMDC.

**Methods**

Patients who received ICI therapy from January 2016 to January 2018 were examined retrospectively. A Cox regression model was used to assess factors associated with overall survival.

**Results**

A total of 826 patients were included. Of these patients, 51.6% received inhibitors of programmed cell death protein-1 or its ligand, 32.0% received inhibitors of cytotoxic T-lymphocyte-associated antigen-4, and 16.5% received a combination of the two. IMDC developed in 52.5% of the patients after a median of 8 weeks. Overall, 569 patients (68.9%) received antibiotic therapy (Fig 1). Antibiotic use at any time was associated with reduced IMDC occurrence and recurrence rates but also with frequent hospitalization and intensive care unit admission for IMDC as well as increased IMDC severity. Compared with patients who received antibiotic therapy only before ICI therapy initiation, those receiving it after ICI had a higher IMDC rate and more often needed immunosuppressive therapy and hospitalization for IMDC. Antibiotics with anaerobic activity were included in 51% of the antibiotic therapy regimens and were associated with increased immunosuppressant use, hospitalization, intensive care unit admission for IMDC, and IMDC grades (Table 1). Forty-one patients received empiric prophylactic antibiotic therapy at IMDC onset. These patients more often needed immunosuppressive therapy, intravenous steroids, and infliximab/vedolizumab; had more frequent and longer hospitalization for IMDC and higher IMDC grades; and more frequently had IMDC recurrence than did patients who did not receive antibiotic therapy at the time of IMDC symptom onset (Table 2).

**Conclusions**

Whereas antibiotic therapy appeared to be protective against IMDC onset, use of antibiotics, especially those with anaerobic activity, after ICI therapy was associated with increased risk of severe IMDC.

**Ethics Approval**

This study was approved by University of Texas MD Anderson Cancer Center Institutional Review Board, IRB #PA18-0472.

**Consent**

Written informed consent was waived for this study given retrospective study.

**Fig. 1 (abstract P721).**
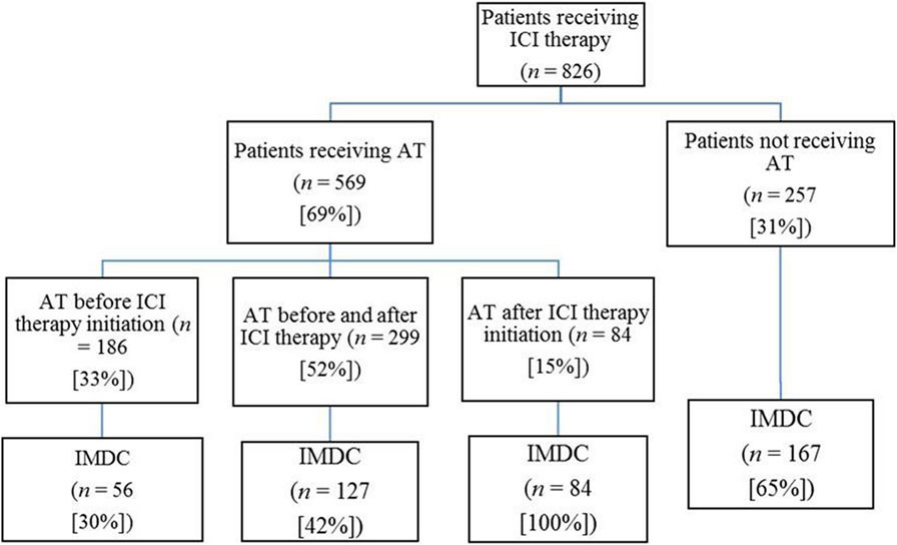
See text for description

**Table 1 (abstract P721).**
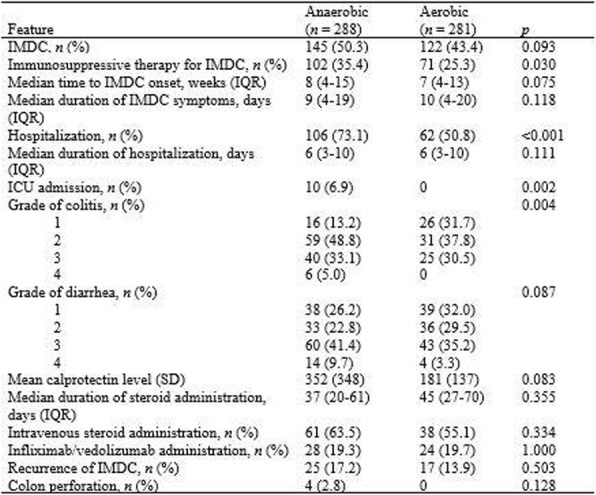
See text for description

**Table 2 (abstract P721).**
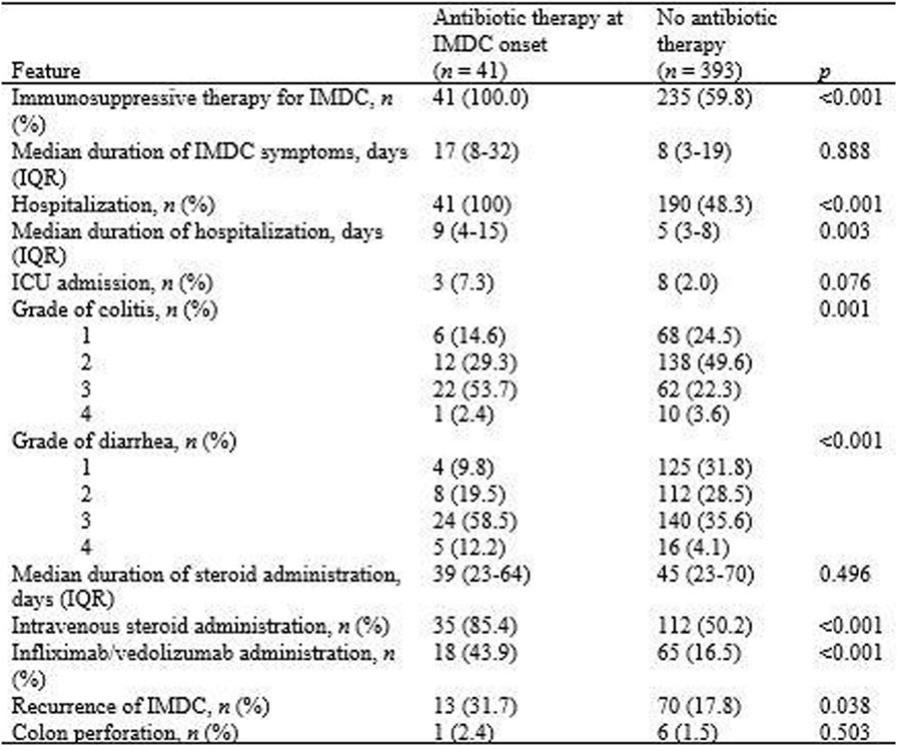
See text for description

#### P722 Opportunistic infection outcomes in patients receiving corticosteroids for immune checkpoint inhibitor toxicity

##### Michael Cook, MD, Michael Cook, MD, Michael Atkins, MD

###### MedStar Georgetown University Hospital, Washington, DC, United States

####### **Correspondence:** Michael Cook (mrc5282@gmail.com, mba41@georgetown.edu)

**Background**

Immune checkpoint inhibitor (ICI) therapy is commonly used in the treatment of advanced malignancy. Immune related adverse events (irAEs) are a common side effect from therapy, requiring high dose corticosteroids to limit morbidity[1,2]. Currently the NCCN recommends Pneumocystis jiroveci pneumonia (PJP) prophylaxis in patients treated with 20mg of prednisone equivalent for at least four weeks, but little is known about the incidence of PJP, opportunistic infections (OI), or non-opportunistic infections in this patient population.

**Methods**

We performed a retrospective analysis of patients treated with ICI therapy at five MedStar Health hospitals from January 2011 to April 2018. We identified 758 patients treated with ICIs; 113 were prescribed a minimum of 20mg of prednisone equivalent for more than 4 weeks for immune related adverse events. The incidence of use of non-corticosteroid immunosuppressive medication, prophylactic antibiotic usage, OI, non-OI infection, and infection outcome was analyzed.

**Results**

Of the 113 patients the most common cancers were melanoma (69) and non-small cell lung cancer (21). The most common ICI therapies implicated were nivolumab plus ipilimumab (35), nivolumab (28), ipilimumab (25), and pembrolizumab (20). The median starting steroid dose was 100mg of prednisone, and 25% of patients (n=28) were taking additional immunosuppressive medications (infliximab, n=15; mycophenolate mofetil, n=8; vedolizumab, n=2; tocilizumab, methotrexate, anti-thymoglobulin, 1 each). PJP prophylaxis with sulfamethoxazole/trimethoprim was given to 12% of patients (n=14). The incidence of patients with any documented infections, which required antimicrobial therapy during ICI treatment, was 19% (n=22). These infections included 4 patients (3%) with opportunistic infections (Table 1; 2 reactivations of non-disseminated varicella zoster infection (VZV), PJP, and Listeria monocytogenes endophthalmitis, 1 each). Additional non-OI included Clostridium difficile (n=5), oral candidiasis (n=4), hospital acquired/ventilator associated pneumonia (n=4), urinary tract infection (n=3) cellulitis (n=2), community acquired pneumonia, osteomyelitis/bacteremia, and sinusitis, 1 each.

**Conclusions**

Our findings suggests that the incidence of OI is low (< 3%) in patients treated with prolonged corticosteroid therapy alone for irAEs due to ICIs and raise questions about the value of prophylactic antibiotics in this population. Further, given the distribution of OI in this analysis, sulfamethoxazole/trimethoprim as the lone choice of prophylactic antimicrobial coverage could also be questioned. Further analyses are needed to validate above findings.

**References**

1. Brahmer JR, Lacchetti C, Schneider BJ, et al. Management of Immune-Related Adverse Events in Patients Treated With Immune Checkpoint Inhibitor Therapy: American Society of Clinical Oncology Clinical Practice Guideline. Journal of Clinical Oncology. 2018;36(17):1714-1768.

2. Weber JS, Kähler KC, Hauschild A. Management of immune-related adverse events and kinetics of response with ipilimumab. Journal of clinical oncology : official journal of the American Society of Clinical Oncology. 2012;30(21):2691-2697.

**Ethics Approval**

The study was approved by MedStar Georgetown University Hospital Ethics Board, approval number 2017-0559.

**Table 1 (abstract P722).**
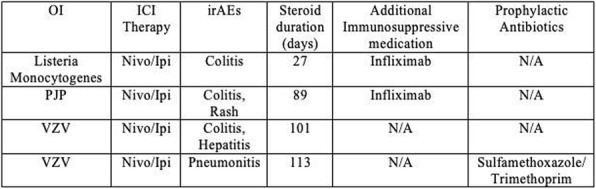
See text for description

#### P723 Interleukin-6 blockade to de-couple CTLA-4 blockade colitis from anti-tumor efficacy

##### Yared Hailemichael, PhD, Daniel Johnson, MD, Wai Foo, Kenneth Hess, BS, MS, PhD, Cara Haymaker, PhD, Chantal Saberian, MD, Salah Bentebibel, Elizabeth Burton, Yinghong Wang, MD, PhD, Scott Woodman, Patrick Hwu, MD, Adi Diab, MD

###### The University of Texas MD Anderson Cancer Center, Houston, TX, United States

####### **Correspondence:** Adi Diab (ADiab@mdanderson.org)

**Background**

Understanding of the immunobiology of checkpoint inhibitors (CPI) induced immune related toxicities, such as immune related enterocolitis, and how these compare to the immune signatures in tumors could lead to the development of strategies that de-couple autoimmunity from anti-tumor immunity. Interleukin 6 (IL-6), a pleiotropic-cytokine promoting tumor progression and essential for Th17 lineage commitment of naive CD4+T-cells has been implicated and a therapeutic target in a variety of autoimmune diseases. Here, we are presenting clinical and preclinical data on the impact of IL-6 blockade to de-couple autoimmunity and tumor immunity.

**Methods**

Total RNA from patient-matched colitis and normal colon FFPE tissue from patients [n=23] receiving CPI were profiled with the 770 gene NanoString nCounter PanCancer Immune Profiling Panel (NanoPCIP). The gene expression fold increase normal vs. irEC inflamed colonic tissue and baseline vs. on-treatment tumor samples from patients responding or non-responding per RECIST v1.1 to ipilimumab-based therapy were analyzed. To evaluate the effect of IL-6 blockade on anti-tumor activity, mice with B16.BL6 or CT-26 tumor received systemic anti-IL-6, anti-CTLA-4, anti-IL-6 and anti-CTLA-4 or placebo. Tumor sizes were measured and immune cell profiles were analyzed using cytof and flow cytometry and chemokine and cytokine levels were quantitated by Luminex.

**Results**

In patients with colitis, the highest significantly upregulated differentially expressed gene (DEG) in inflamed colon tissue encoded for IL-6 (+24.1) followed by genes for neutrophil and monocyte chemotactic molecules. Interestingly, IL-6 was also the highest upregulated DEG in non-responding tumors numerically. There was no overlap between DEGs upregulated in colitis and responding-tumors. Gene sets expression analysis indicated significantly higher Th17 than Th1 upregulation in colitis compared to normal colon. Further imaging cytof and IHC analyses of colitis tissue is on-going. In murine tumor models, the addition of IL-6 blockade to anti-CTLA-4 therapy significantly improved tumor shrinkage compared to anti-CTLA-4 alone. Correlating with the anti-tumor response, tumor-infiltrating immune cells analysis showed, increased CD8+ or Th1 (T-effector:Treg) ratio, reduced Th17 differentiation, contraction in macrophages and PD-L1High myeloid cells. Analysis of tumor supernatant revealed Th1-biased immune response and increased expression of T cell chemotactic molecules in mice treated with anti-CTLA-4 and anti-IL-6 compared with mice treated with anti-CTLA-4.

**Conclusions**

Our human-tissue data demonstrates that IL-6-mediated-inflammation may be more prevalent in colitis than in responding-tumors, and in mice blocking IL-6 enhances CPI anti-tumor response. Targeting IL-6 may ameliorate colitis without hindering anti-tumor immunity. A clinical trial evaluating concomitant IL-6 blockade with ipilimumab and nivolumab is being initiated.

**References**

1. Miossec, P., Korn, T. & Kuchroo, V.K. Interleukin-17 and type 17 helper T cells. N Engl J Med 361, 888-898 (2009).

**Ethics Approval**

The protocol was approved by The University of Texas MD Anderson IRB board protocol # PA19-0427

#### P724 Analysis of healthcare provider management of immune-related adverse events and concordance with NCCN Guidelines®

##### Megan Cartwright, PhD^1^, Megan Cartwright, PhD^1^, Krista Marcello, BA^1^, Jillian Scavone, PhD^2^, Kevin Obholz, PhD^1^, Timothy Quill, PhD^1^, John Thompson^3^

###### ^1^Clinical Care Options, LLC, Reston, VA, United States ; ^2^National Comprehensive Cancer Network®; ^*3*^*University of Washington, Seattle, WA, United States*

####### **Correspondence:** Megan Cartwright (mcartwright@clinicaloptions.com)

**Background**

Immune checkpoint inhibitors (ICIs) have dramatically altered the therapeutic landscape across oncology. However, ICIs are associated with a unique safety profile involving immune-related adverse events (irAEs) that require prompt recognition and management to ensure optimal patient safety. In 2017, we developed an online interactive decision support tool at www.clinicaloptions.com/immuneAEtool to provide healthcare providers (HCPs) case specific, evidence-based guidance on management of irAEs and reported substantial variances in practice compared with expert recommendations [1]. The National Comprehensive Cancer Network® (NCCN) publishes guidelines for managing irAEs in patients treated with ICIs across all organ systems [2]. In 2019, our interactive tool was updated to incorporate case-specific irAE management recommendations from the new NCCN Guidelines® [3]. Here, we report a comparison of HCP-reported planned irAE management strategies vs the corresponding NCCN recommendations provided via the 2019 irAE tool.

**Methods**

To use the online tool, HCPs enter the organ system affected and the grade or severity of the symptom along with their planned management strategy. The HCPs were then shown the NCCN management recommendation for that specific irAE scenario. After viewing the NCCN management recommendation, HCPs were asked if it affected their intended management approach.

**Results**

Between February and June 2019, 737 HCPs entered 972 unique case scenarios into the tool. 58% of cases were entered by clinicians who treat ≤ 20 patients/year with ICIs. The most frequently entered irAEs involved the gastrointestinal, dermatologic, and pulmonary systems (28%, 24%, and 19% of cases), similar to our previous practice analysis [1]. Overall, the planned irAE management strategy of HCPs matched the NCCN recommendations in 39% of cases, with the greatest divergence in cardiovascular and dermatologic irAE management (Figure 1). The proportion of cases in which the planned management strategies of HCPs matched the NCCN recommendations also varied by symptom grade/severity, with lower concordance (31%) observed for the management of grade 3/4 events compared with grade 1/2 events (43%). 48% of HCPs indicated that the NCCN recommendations provided by the tool changed their management plan.

**Conclusions**

These data suggest that many HCPs are challenged to optimally manage irAEs associated with ICI and are not managing their patients in concordance with the NCCN guidelines. Use of an online tool providing easy access to NCCN Guidelines recommendations improves patient care and safety. A detailed analysis of the tool, including case entries and planned management vs NCCN recommendations for each irAE and grade, will be presented.

**References**

1. Marcello K, Obholz KL, Quill TA, Weber JS. Variance from evidence-based management of immune-related adverse events among healthcare providers: analysis of an online management decision tool. 32nd Annual Meeting and Pre-Conference Programs of the Society for Immunotherapy of Cancer; November 8-12, 2017; National Harbor, Maryland. Abstract O33.

2. National Comprehensive Cancer Network. Management of Immunotherapy-Related Toxicities. V.2.2019 [http://www.nccn.org]. Accessed July 16, 2019.

3. NCCN Clinical Practice Guidelines in Oncology (NCCN Guidelines®) for Managing Immune Checkpoint Inhibitor–Related Toxicities: An Interactive Decision Support Tool [https://www.clinicaloptions.com/immuneAEtool]. Accessed July 16, 2019.

**Fig. 1 (abstract P724).**
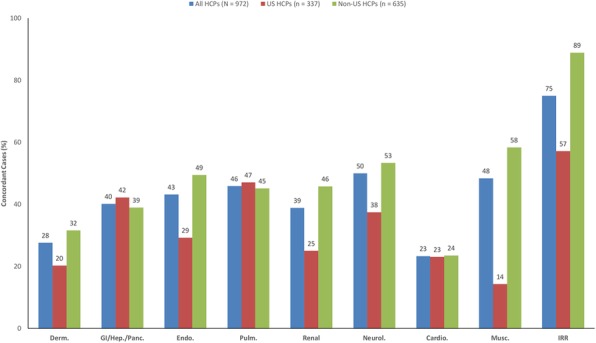
Cases managed concordant with guidelines

#### P725 Clinical features of immune checkpoint inhibitor-related adrenal insufficiency: A retrospective analysis

##### Qinwen Zhou, MD, Sandip Patel, MD, Qinwen Zhou, MD, Vala Hamidi

###### UCSD, San Diego, CA, United States

####### **Correspondence:** Qinwen Zhou (qzhou@ucsd.edu)

**Background**

Immune checkpoint inhibitors (ICIs) have revolutionized cancer treatment over the past decade. However, despite their increasing use in clinical practice, there are few established, evidence-based guidelines for the screening, diagnosis, and management of immune-related adverse events, particularly with regard to autoimmune endocrinopathies [1,2]. In particular, adrenal insufficiency is a toxicity associated with ICIs that is poorly characterized due to the nonspecific nature of its presentation, and can be life-threatening if not promptly recognized and treated [3]. Our aim is to better define the clinical, biochemical, and imaging features of ICI-related adrenal insufficiency (AI) to mediate earlier recognition and treatment.

**Methods**

We identified a total of 250 patients treated with either nivolumab, pembrolizumab, or ipilimumab/nivolumab combination therapy found to have a random cortisol level less than 3 or morning cortisol less than 5 during or following therapy. Of these, 60 patients had been on prednisone or dexamethasone at time of cortisol testing, and 6 had undergone adrenalectomy. These patients were excluded.

**Results**

184 patients met criteria for new onset AI on ICI therapy. Median time to onset was 17 weeks. 13% were appropriately diagnosed with adrenal insufficiency, 6% had subsequent workup with ACTH, 11% were treated with hydrocortisone, 2% were referred to endocrine. Of the undiagnosed patients, 5% had biochemical and clinical findings suggestive of adrenal insufficiency, including fatigue, nausea, hyponatremia, hypotension, and hyperkalemia, that was not further worked up in the setting of pembrolizumab and 10% in the setting of nivolumab. No anti-21 hydroxylase antibodies were drawn.

**Conclusions**

As ICIs are being used with increasing frequency, it is crucial for health care providers to recognize the characteristics of immune-mediated adrenal insufficiency and initiate the appropriate workup, treatment, and referrals. Based on our preliminary findings, we have found that a majority of cortisol levels are ordered as part of routine screening of ICI infusion but without proper work-up or follow up when levels meet criteria for AI. Few patients receive ACTH or antibody testing and are initiated on adrenal replacement or referred to endocrinology. Thus, we recommend a greater awareness and more systematic approach to diagnosing and managing AI in patients on ICIs.

**References**

1. Chang et al. “Endocrine Toxicity of Cancer Immunotherapy Targeting Immune Checkpoints.” Endocr Rev. 2019. 40(1):17-65.

2. Wang et al. “Immune-Related Adverse Events Associated with Anti-PD-1/PD-L1 Treatment for Malignancies: A Meta-Analysis.” Frontiers in Pharmacology. 2017. 8(730): 1-12.

3. Ariyasu et al. “Adrenal Insufficiency Related to Anti-Programmed Death-1 Therapy.” Anticancer Research. 2017. 37: 4229-4232.

**Ethics Approval**

UCSD Biorepository Protocol (HRPP# 090401)

UCSD ImmunoScape Immune Monitoring Protocol (HRPP# 150348) activated 3/12/15

### Machine Learning, Artificial Intelligence, and Computational Modeling

#### P726 Applying deep learning classification for tumor identification across immunohistochemical markers on serial sections to eliminate the need for image co-registration

##### Mark Anderson, BSc, Mark Anderson, BSc , Karen McClymont, Lorcan Sherry, PhD, Alison Bigley, CSci, FIBMS

###### OracleBio, North Lanarkshire, United Kingdom

####### **Correspondence:** Mark Anderson (mark.anderson@oraclebio.com)

**Background**

Artificial intelligence deep learning networks are being increasingly applied to resolve complex pattern recognition challenges in quantitative digital pathology. Khosravi[1], utilized deep learning to discriminate and classify tumors and associated subtypes using a range of immunohistochemically (IHC) labelled tumor sections, even where tumors were significantly heterogeneous.

Image co-registration is commonly employed in brightfield quantitative workflows where serial sections have been IHC labelled with different markers, one of which is a tumor marker such as pan-Cytokeratin, which is used to guide the tumor locations in the corresponding serial section. Frequently, alignment errors occur in particular at the periphery of tumor foci, where either tumor cells are missed or adjacent stroma is incorrectly classified as tumor.

Deep learning classifiers can be trained across a range of sections and markers to define tumor regions of interest (ROI), utilizing a range of features from each stained sample to generate a classifier, capable of identifying tumor ROI independent of the tissue, stain or marker.

Here we use serial tissue microarray (TMA) sections of gastric adenocarcinomas, labelled with pan-Cytokeratin and CD3, to exemplify the use of a deep learning approach to distinguish tumor foci without requiring serial section image co-registration.

**Methods**

Exemplar TMA serial sections of gastric adenocarcinomas were IHC labelled for pan-Cytokeratin and CD3, using DAB chromogen and counterstained with hematoxylin. The stained slides were digitized using a Zeiss scanner.

The sections were analyzed using Deeplabv3 network in Visiopharm Oncotopix Software. Classes were established for tumor, stroma and background, at x10 input magnification, using identical example training labels across the IHC images.

**Results**

Tumor and stroma were accurately identified across the serial sections using a single deep learning algorithm. Applying a Dice similarity coefficient confirmed a high-level correlation between manual ROI and deep learning classifications for the 2 markers.

**Conclusions**

The exemplar data demonstrated the application of a single classifier, across IHC images, enabling the identification of tumor and stroma, irrespective of marker. This approach has demonstrated that it is possible to accurately utilize multifaceted features, from different IHC markers on TMA tissue sections, for the accurate classification of tumor and stroma ROI. This approach provides an alternative method to the co-registration of images for tumor assignment across serial sections.

**References**

1. Khosravi P, Kazemi E, Imielinskid M, Elemento O and Hajirasouliha I. Deep Convolutional Neural Networks Enable Discrimination of Heterogeneous Digital Pathology Images. EBioMedicine 2018; 27: 317–328

#### P727 Single-cell systems neuroimmunology reveals immunosuppressive checkpoint blockade receptor expression correlates with ventricular stem cell niche contact in human glioblastoma

##### Todd Bartkowiak, PhD^1^, Sierra Barone, BS^1^, Allison Greenplate, PhD^1^, Justine Sinnaeve, BS^1^, Nalin Leelatian^1^, Akshitkumar Mistry, MD^2^, Caroline Roe, BS^1^, Bret Mobley, MD^1^, Lola Chambless, MD^2^, Reid Thompson, MD^2^, Kyle Weaver, MD^2^, Rebecca Ihrie, PhD^1^, Jonathan Irish, PhD^1^

###### ^1^Vanderbilt University, Nashville, TN, United States; ^2^Vanderbilt University Medical Center, Nashville, TX, United States

####### **Correspondence:** Rebecca Ihrie (rebecca.ihrie@vanderbilt.edu), Jonathan Irish (jonathan.irish@vanderbilt.edu)

**Background**

Glioblastomas make up more than 60% of adult primary brain tumors and carry a median survival of less than 15 months despite aggressive standard therapy. Immunotherapy, now standard of care for many solid tumors, offers an appealing alternative platform that may improve survival outcomes for patients with glioblastoma; however, predictive features that could inform responsiveness to different immunotherapeutic modalities remains to be elucidated. Recent studies have demonstrated that patients whose tumors show radiographic contact with the ventricular-subventricular zone (V-SVZ) have diminished survival outcomes compared to patients whose tumors do not contact the V-SVZ. We therefore hypothesized that V-SVZ contact may provide a unique, immunosuppressive microenvironment within the brain that promotes tumor growth by suppressing anti-tumor immunity that may be overcome via targeting the appropriate immune suppressive immune population or receptor target.

**Methods**

Primary glioblastoma tumors obtained in accordance with the Declaration of Helsinki and with institutional IRB approval (#131870, #030372, #181970) were disaggregated into single-cell suspensions. Radiographic contact with the V-SVZ was identified by MRI imaging and confirmed by a trained neurosurgeon. Multi-dimensional single-cell mass cytometry (CyTOF) then measured >30 immune identity markers in thirteen immune populations infiltrating human glioblastomas, including CD4 and CD8 T cells, γδ T cells, natural killer cells, B cells, microglia, peripheral macrophages, and myeloid-derived suppressors cells (MDSC). Advanced computational dimensionality-reduction tools including Citrus, t-SNE, FlowSOM, and MEM along with traditional biaxial gating strategies identified key differences in the abundance and phenotypes of immune infiltrates.

**Results**

On the basis of tumor contact with the V-SVZ, the Citrus and FlowSOM clustering algorithms computationally identified consequential distinctions in the abundance of five T cell, macrophage, and microglia subsets among glioblastomas. In addition, differential expression of five functional immune markers (three activating receptors and two inhibitory receptors) was observed in seven distinct immune cell subsets infiltrating tumors. Critically, the abundance of identified immune subsets and relative immune receptor expression levels correlated significantly with tumor contact status and patient outcomes. Biaxial gating analysis and parallel computational pipelines confirmed that comparable cell subsets could be identified with traditional approaches and unsupervised algorithmic analysis.

**Conclusions**

Single-cell mass cytometry in conjunction with the Citrus and FlowSOM clustering tools identified key differences in immune cell abundance between V-SVZ contacting and non-contacting glioblastomas. These results provide key insights into the immune microenvironment of glioblastomas and elucidate several clinically actionable immunotherapeutic targets that may be used to optimize treatment strategies for glioblastomas based on V-SVZ contact status.

**Ethics Approval**

This study was approved by the Institutional Review Board at Vanderbilt University, approval #131870, #030372, and #181970.

#### P728 Inference of immunotherapy response-predictive biomarkers in lung adenocarcinoma from hematoxylin and eosin (H&E) stained images

##### Kimary Kulig, PhD, MPH^1^, Cory Batenchuk, PhD^1^, Peter Cimermancic^1^, Huang-Wei Chang, PhD^1^, Eunhee Yi, MD^2^, Vanessa Velez^1^, Hardik Patel^1^, Ali Behrooz^1^, Kamilla Tekiela^1^, Robert Findlater^1^

###### ^1^Verily Life Sciences, South San Francisco, CA, United States; ^2^Mayo Clinic, Rochester, MN, United States

####### **Correspondence:** Kimary Kulig (kulig@verily.com)

**Background**

The current work-up for diagnosis, gene mutation, and programmed death ligand-1 (PD-L1) testing in metastatic NSCLC can exhaust an entire tumor specimen. Testing for tumor mutation burden (TMB), a candidate predictive biomarker for checkpoint inhibitor therapy, currently requires 10 tissue slides and ranges from 10 days to 3 weeks from sample acquisition to test result. As more CDx-restricted drugs are developed for lung adenocarcinoma, rapid, tissue-sparing tests are sorely needed. We investigated whether these biomarkers can be inferred from digital H&E images alone.

**Methods**

Whole slide (40x-magnified) H&E images of lung adenocarcinoma from TCGA were used to train a computational model to infer TMB and PD-L1 status. Modeling occurred in two layers: first, a neural network was trained to segment known histologic and other tumor microenvironment features from H&E images. Each feature was manually annotated and confirmed by two rounds of pathologist review. Second, these feature maps, along with ground truth biomarker labels and patient demographics, were analyzed by second-level models to infer biomarker status. Ground truth biomarker status of H&E-associated tumor samples included WES for TMB and reverse-phase protein array for PD-L1, per TCGA. TCGA samples (n=40) generating 40,000 feature annotations were used to train and evaluate the first layer model. Held-out TCGA samples were used to train (n=200) and test (n=194) the TMB and PD-L1 classification models.

**Results**

In a "hold-one-TCGA-site-out" approach using 40 images, the overall accuracy of the first model layer in classifying pathologist-confirmed histologic features was 98-99%. The two-layer model achieved an accuracy of 81% in predicting both TMB (AUC = 0.81) and PD-L1 status (AUC = 0.86) in the testing dataset.

**Conclusions**

Biomarker inference from digital H&E images has the potential to be highly accurate and to supplement or replace certain tissue-based tests. Use of known biological features makes the model interpretable and verifiable by pathologists. This method may enable testing in patients whose tumor tissue has been exhausted or who cannot undergo re-biopsy to enable biomarker testing. This technology can return test results within hours instead of days or weeks, enabling rapid treatment decision-making and start. Ongoing work to improve accuracy of the model, assess model correlation to PD-L1 by IHC and gene panel-derived TMB, and to evaluate the model’s correlation to clinical outcomes is in progress.

#### P730 An empirical framework for validating artificial intelligence-derived PD-L1 positivity predictions using samples from patients with urothelial carcinoma

##### Andrew Beck, MD, PhD^1^, Benjamin Glass^1^, Hunter Elliott^1^, Jennifer Kerner^1^, Aditya Khosla^1^, Abhik Lahiri^1^, Harsha Pokkalla^1^, Dayong Wang^1^, Ilan Wapinski^1^, George Lee, PhD^2^, Vipul Baxi, MS^2^, Cyrus Hedvat, MD, PhD^2^, Dimple Pandya^2^, Michael Montalto^2^

###### ^1^PathAI, Inc, Boston, MA, United States; ^2^Bristol-Myers Squibb, Princeton, NJ, United States

####### **Correspondence:** Ilan Wapinski(Ilan.wapinski@pathai.com)

**Background**

Assessing PD-L1 immunohistochemistry (IHC) expression may play a role in identifying patients likely to benefit from anti–PD-1/PD-L1 therapies in some advanced cancers, including urothelial carcinoma (UC). Studies have shown variable inter-observer agreement for pathologist assessment of PD-L1 expression, with generally higher levels of concordance for tumor cell scoring compared with immune cell scoring [1,2]. Thus, immune cell PD-L1 scoring may be more challenging to implement than scoring on tumor cells. We evaluated the performance of an artificial intelligence (AI)-based predictor of PD-L1 expression on tumor and/or immune cells in the tumor microenvironment and compared this with manual evaluations by a network of pathologists to identify whether the AI platform performed more consistently.

**Methods**

PD-L1 was assessed using the PD-L1 IHC 28-8 pharmDx assay (Dako, Agilent Technologies Co). The training set for this exploratory analysis consisted of 293 pretreatment samples from patients with advanced UC, commercially procured or from clinical trials of nivolumab [3,4]. From these, we obtained 105,514 annotations of tumor and immune cells from 43 pathologists. To establish a reference dataset for manual vs digital concordance using the platform (PathAI, not intended for diagnostic use), we generated 80 150x150-micron–sized “frames” sampled from 100 held-out clinical images, removing frames of inadequate tissue quality or with presence of artifacts. For each frame, we collected exhaustive annotations from 5 pathologists to produce quantitative estimates of PD-L1 positivity on all tumor and immune cells. Altogether, 66,049 annotations were collected and used to compute pathologist consensus scores for each frame. These scores were then correlated with each individual pathologist (inter-reader agreement) and with the PathAI-derived automated scores for evaluation of manual vs digital agreement.

**Results**

The PathAI platform showed significantly stronger correlation with pathologist consensus scores compared with scores generated by individual pathologists for quantifying PD-L1 positivity of lymphocytes (r-squared=0.744 vs 0.598) and macrophages (r-squared=0.68 vs 0.287). There was no difference in correlation with consensus between PathAI-derived and individual pathologist-derived assessment of positivity on tumor cells (r-squared=0.837 vs 0.857).

**Conclusions**

We validated performance of the PathAI platform for automated assessment of PD-L1 expression on tumor and immune cells and demonstrated that the AI-predictors perform similar to or better than pathologist-based scoring in all cell types tested, and especially for immune cells where manual correlation is low. These results suggest that AI-powered assessment represents a reproducible and potentially generalizable approach to interpretation of IHC assays.

**Acknowledgements**

Bristol-Myers Squibb and PathAI, Inc.

**Trial Registration**

NCT02387996, NCT01928394

**References**

1. Rimm DL, Han G, Taube JM, et al. A prospective, multi-institutional, pathologist-based assessment of 4 immunohistochemistry assays for PD-L1 expression in non-small cell lung cancer. JAMA Oncol. 2017;3:1051-1058.

2. Eckstein M, Wirtz RM, Pfannstil C, et al. A multicenter round robin test of PD-L1 expression assessment in urothelial bladder cancer by immunohistochemistry and RT-qPCR with emphasis on prognosis prediction after radical cystectomy. Oncotarget. 2018;9:15001-15014.

3. Sharma P, Retz M, Siefker-Radtke A, et al. Nivolumab in metastatic urothelial carcinoma after platinum therapy (CheckMate 275): a multicentre, single-arm, phase 2 trial. Lancet Oncol. 2017;18:312-322.

4. Sharma P, Callahan MK, Bono P, et al. Nivolumab monotherapy in recurrent metastatic urothelial carcinoma (CheckMate 032): a multicentre, open-label, two-stage, multi-arm, phase 1/2 trial. Lancet Oncol. 2016;17:1590-1598.

**Ethics Approval**

The protocol was approved by site institutional review boards or independent ethics committees and conducted according to Good Clinical Practice guidelines, per the International Conference on Harmonisation. Patients provided written informed consent based on Declaration of Helsinki principles.

#### P731 Identification and validation of shared neoantigens for cancer immunotherapy

##### Jennifer Busby, PhD, Melissa Rotunno, Tyler Murphy, William Brinton, Michael Zhong, Lina Kim, Abu Jalloh, Amanda Costa, Michael Fray, Aaron Yang, Meghan Hart, Matthew Davis, Rita Zhou, Elizabeth Maloney, Alexis Mantila, Karin Jooss, PhD, Aleksandra Nowicka, Christine Palmer, PhD, James Sun, Roman Yelensky, PhD , Jennifer Busby, PhD

###### Gritstone Onclogy, Inc, Cambridge, MA, United States

####### **Correspondence:** Roman Yelensky (ryelensky@gritstone.com)

**Background**

Neoantigens can be targeted with cancer immunotherapies, including cell therapy and immunization, but most neoantigens are private to each patient, necessitating fully personalized treatments. Identification of solid tumor-presented neoantigens that may be shared across patients holds great value for the generation of off-the-shelf therapies.

**Methods**

We pursued an immunopeptidomics strategy to identify which recurrent mutations in cancer lead to neoantigen peptides presented on the surface of tumor cells. 498 frozen resected tumor samples and 6 cell lines across 14 tumor types (including 139 NSCLC and 139 CRC) were subjected to transcriptome RNASeq. Transcriptomes were analyzed for expression of recurrent driver mutations and used to predict HLA peptide epitopes with our deep learning model EDGE [1]. Additionally, single HLA class I allele expressing K562 cells were transduced with vectors expressing shared mutations of interest. Predicted peptides were assayed in the tumors and cell lines by Class I HLA immunoprecipitation and targeted mass spec. For a subset of peptides, we evaluated if CD8 T-cell precursors are found in the naïve repertoire of HLA-matched donors.

**Results**

16 neoantigens (distinct HLA/mutation pairs) derived from 10 driver mutations were detected by MS in 27 tumors and 3 single allele cell lines. Only 1 of these neoantigens (KRAS G12V/HLA-A*11:01) was previously reported in patients. The driver mutation with most neoantigens was KRAS G12V, with peptides detected on 4 HLA alleles, accounting for ~40% of patients with the mutation. Importantly, HLA-A*02:01 was not found to present KRAS G12V. Other neoantigen peptides were detected in genes including KRAS, CTNNB1 and TP53. When combined with the few published neoantigens, the set was estimated to cover 9% of NSCLC, 11% of MSS-CRC, and 19% of pancreatic cancer patients, accounting for HLA restriction. Precursor CD8 T cells were detected for 6 out of 7 tested HLA/peptide pairs arising from mutations in KRAS and CTNNB1, recognized by a median of 46 TCR clonotypes, highlighting their immunogenic potential.

**Conclusions**

We analyzed immunopeptidomes of human tumors and cell lines and validated 15 novel shared neoantigens, the largest set to-date. The study enables off-the-shelf immunotherapies for significant fractions of patients and highlights the value of HLA screening, as mutations are presented by specific HLA alleles. Shared neoantigen targets identified in this study, among others, are currently being tested in patients in the SLATE clinical immunotherapy program. Patient coverage will likely expand as additional classes of neoantigens are identified and added to off-the-shelf immunotherapy products.

**References**

1. Bulik-Sullivan B, Busby J, et al. Deep learning using tumor HLA peptide mass spectrometry datasets improves neoantigen identification. Nature Biotech. 2018; 37: 55-63. (DOI: 10.1038/nbt.4313)

#### P732 Reverse causal inferencing on lung adenocarcinoma patients reveals a stem cell-like molecular subtype associated with pack year history

##### Renee Deehan, Sergey Korkhov, Alexis Foroozan, Scott Marshall, PhD, Renee Deehan, Nimisha Schneider

###### QuartzBio, a Precision for Medicine company, Plan-les-Ouates, Switzerland

####### **Correspondence:** Renee Deehan (renee.deehan-kenney@precisionformedicine.com)

**Background**

Advances in high throughput measurement technologies (-omics data) have made it possible, and increasingly affordable, to generate high complexity, high volume data for oncology research. Coordinate efforts in computational modeling and machine learning applications to biological data have yielded increasingly sophisticated methods to deeply characterize the mechanisms of disease pathogenesis and heterogeneity among patients; which are predicates to the rapid development and timely and safe administration of efficacious treatments. Open source repositories that catalog, harmonize and host -omics data collected from clinical or preclinical studies, generously donated by patients and researchers, provide revolutionary access to otherwise siloed data. The combination of these advancements enabled us to characterize the molecular phenotypic heterogeneity that exists within a lung adenocarcinoma cohort from The Cancer Genome Atlas [1].

**Methods**

We applied a reverse inferencing approach that systematically interrogates RNAseq measurements from tumor and control biopsies against our library of cause and effect gene networks curated from published experiments. If patterns observed in the data are significantly similar to those in a network, an inference about the directional activity of that network can be made. Our library was nucleated through an open sourced knowledge graph [2] and enhanced with updated and relevant knowledge using the Biological Expression Language framework [3].

**Results**

In LUAD tumor cells, we detected a pattern of gene signatures which indicated a tumor stem cell-like phenotype characterized by predicted decreases in the activity of pro-differentiation factors FOXP2 and PHOX2B and an increased response to hypoxia. Analysis of patients with heavy (>40) versus light (<10) pack-year burden suggested an augmented dedifferentiation profile in heavy smokers, including decreased SOX6, HNF1A and increased FAT1 signaling, which has also been recently implicated in resistance to immune checkpoint inhibitors in NSCLC patients [4]. Expression of PD-L1 has been associated with cumulative inhaled smoke exposure in lung cancer patients, and with mechanisms inferred from our analysis (e.g., increased MIR140 signaling) [5,6].

**Conclusions**

Our in silico analysis of lung cancer patient biopsies generated hypotheses implicating stem cell signaling in tumors, and a further stratification of this signal based on patient pack year burden. Given the implication of tumor stem cells in resistance to drug treatments in cancers, there is increased focus on immunotherapies as a mechanism to circumvent this resistance, and the role of PD-L1 in this milieu requires further examination. Understanding the molecular makeup of these subtypes is critical when considering clinical research treatment strategies in immuno-oncology.

**References**

1. The Cancer Genome Atlas. https://portal.gdc.cancer.gov/

2. Catlett N, et al., Reverse causal reasoning: applying qualitative causal knowledge to the interpretation of high-throughput data. BMC Bioinformatics. 2013;14-340

3. Open BEL. https://openbel.org

4. Fang W, et al., Comprehensive genomic profiling identifies novel genetic predictors of response to anti-PD-(L)1 therapies in non-small cell lung cancer. Clin Cancer Res. 2019 [epub ahead of print]

5. Villanueva N, Bazhenova L, New strategies in immunotherapy for lung cancer: beyond PD-1/PD-L1. Ther Adv Respir Dis. 2018;12

6. Xie W, et al., MiR-140 expression regulates cell proliferation and targets PD-L1 in NSCLC. Cell Physiol Biochem. 2018;46(2):654-663

#### P733 Quantitative systems pharmacology modeling of B-cell acute lymphoblastic leukemia treatment with bispecific T-cell engaging antibodies for investigation of bell-shaped dose response

##### Oleg Demin Jr, MSc, Oleg Demin Jr, MSc

###### InSysBio, Moscow, Russian Federation

####### **Correspondence:** Oleg Demin Jr (demin_jr@insysbio.com)

**Background**

Kinetics of bispecific therapeutic antibody bound with its targets (trimer complex) is more complex than simple monospecific antibody bound with one target. Theoretically, the dependence of concentration of trimer complex on dose is bell-shaped. The aim of this work is to investigate dose response behavior of bispecific T-cell engaging antibodies under physiological conditions on the basis of quantitative systems pharmacology (QSP) model of B-cell acute lymphoblastic leukemia (B-ALL) treatment with blinatumomab, CD19/CD3 bispecific T-cell engager.

**Methods**

Developed mathematical model consist of: (1) physiologically-based pharmacokinetic (PBPK) model of blinatumomab including binding to target receptors – CD3 on T cells and CD19 on leukemic cells; (2) adult B-ALL progression model including dynamics of normal cells (B cells and its precursors, neutrophils, platelets); (3) specific lysis of CD19+ cells (both normal and leukemic cells) by CD3+ T cells in presence of blinatumomab. Model describes immunological synapse between CD3+ T cells and CD19+ cells, and formation of trimer complex of blinatumomab with CD3 and CD19. Parameters of the model were identified on the basis of published in vitro and in vivo data on healthy subjects and adult relapse/refractory B-ALL patients, but not clinical data. Blinatumomab clinical data on pharmacokinetics (PK) and pharmacodynamics (PD) were used to validate the model.

**Results**

Model was able to reproduce clinical PK data on blinatumomab without fitting. PD of blinatumomab was validated against data on dynamics of CD19+ cells in blood of B-ALL patients. Model shows that there is no response at very low doses (up to 8 ug/m2/day) which correspond to clinical data where there is no response at doses up to 5 ug/m2/day [1]. The threshold numbers of trimer complexes in synapse for activation of specific lysis in presence of blinatumomab were 38 for CD4 T cells and 16 for CD8 T cells. The decrease in concentration of trimer complex (bell-shaped behavior) can be observed in the model only at doses above 10^6 ug/m2/day, whereas approved dose of blinatumomab is 15 ug/m2/day or 28 ug/day. Model analysis showed that under physiological conditions bell-shaped dose response can be observed only when density of target receptors is very low in comparison with amount of bispecific antibodies.

**Conclusions**

Developed model demonstrates capability of QSP modeling to reproduce treatment efficacy clinical data on the basis of in vitro data. Dose response behavior depends on properties of bispecific T-cell engaging antibody as well as target expression.

**References**

1. Bargou R, Leo E, Zugmaier G, Klinger M, Goebeler M, Knop S, Noppeney R, Viardot A, Hess G, Schuler M, Einsele H, Brandl C, Wolf A, Kirchinger P, Klappers P, Schmidt M, Riethmüller G, Reinhardt C, Baeuerle PA, Kufer P. Tumor regression in cancer patients by very low doses of a T cell-engaging antibody. Science. 2008;321(5891):974-7.

#### P734 Prediction of target occupancy in tumor after treatment with anti-PD1 antibodies using physiologically based pharmacokinetic modeling

##### Oleg Demin Jr, MSc, Oleg Demin Jr, MSc, Dmitry Shchelokov

###### InSysBio, Moscow, Russian Federation

####### **Correspondence:** Oleg Demin Jr (demin_jr@insysbio.com)

**Background**

Testing for target receptor occupancy in blood can be assessed in clinical studies. However, it is difficult to sample tumor tissues in patients to assess receptor occupancy (RO). The aim of this work is to develop physiologically based pharmacokinetic (PBPK) and RO model for therapeutic antibodies (Abs) against membrane-bound targets and test its predictive power on the basis of published clinical data on anti-PD1 Abs.

**Methods**

General structure of developed PBPK/RO model is similar to published minimal PBPK models, but there are some differences. Key features of developed model: (1) binding of Abs with membrane bound receptor, i.e., number of target receptor per cell, number of cell expressing target receptor and valency of Abs were taken into account; (2) internalization of target receptor; (3) uptake of Abs by endothelial cells, binding of Abs with FcRn, recycling of bound Abs, degradation of free Abs; (4) competition of Abs with endogenous IgG; (5) renal clearance of Abs which is equal to zero for IgG-based Abs. Physiological parameters were taken from published literature, other parameters were identified on the basis of in vitro and in vivo data. Clinical data on PK of anti-PD1 Abs (nivolumab, pembrolizumab, INCMGA00012 (MGA012), dostarlimab (TSR-042)), receptor occupancy in blood and tumor were used only for model validation.

**Results**

Model is able to reproduce clinical PK data on various doses and regimens of anti-PD1 Abs without fitting and data on PD1 occupancy in blood as well as PD1 occupancy in tumor after treatment with nivolumab [1]. Model shows that anti-PD1 Abs with dissociation constant less than 3 nM and at doses starting from 3 mg/kg will demonstrate PD1 occupancy within the range 90-100%. Mean steady state trough PD1 occupancy were predicted to be 99.5% after administration of 500 mg Q3W dostarlimab and 99.1% after administration of 500 mg Q3W of INCMGA00012, whereas less frequently administration of INCMGA00012 (500 mg Q4W) resulted in 98.6%.

**Conclusions**

Developed model is able to predict PK of anti-PD1 Abs and PD1 occupancy in blood and tumor without fitting of clinical data. This approach can be used for Abs targeting other receptors including bispecific Abs with different structure and properties.

**References**

1. Das R, Verma R, Sznol M, Boddupalli CS, Gettinger SN, Kluger H, Callahan M, Wolchok JD, Halaban R, Dhodapkar MV, Dhodapkar KM. Combination therapy with anti-CTLA-4 and anti-PD-1 leads to distinct immunologic changes in vivo. J Immunol. 2015;194(3):950-9.

#### P735 Accelerating biomarker discovery using AI powered multi-omics approaches

##### Madhuri Gadekar, MS Pharmacology and Toxicology, Mohan Uttarwar, Shruti Desai, Sanjay Khare

###### Indx.Ai, Cupertino, CA, United States

####### **Correspondence:** Sanjay Khare (sanjay.khare@indxtechnology.com)

**Background**

Since precision medicine is nascent field, majority of its requirements in respect of tools and technologies are in early development. It’s a move towards incrementally more effective targeted drugs, less uncertainty and more accuracy. The question today is not just about overall survival of the patient, but also Quality of Life the patient leads.

Oncology, precisely Immuno-oncology has been the most significant gainer of precision medicine and big data. But the challenges facing the community are myriad and magnificent

**Methods**

Data sharing and security is the most debated issue. Sharing of information across complex health care system remains the tantalizing challenge.

Targeted drugs in development patients with specific biomarkers that would predict the prognostic outcome of the drug, hence accurately and ethically recruiting patients for targeted therapies is a challenge, it’s no longer randomization in true sense.

Patient Management poses challenges regarding standardizing collection and transport of clinical samples, since it is rare that an individual lab/hospital possesses the expertise of different diagnostic tests undertaken.

With loads of data available, clinical practitioners are finding it hard to interpret the results of different diagnostic/exploratory tests.

Majority of the challenges faced have technical solutions that are designed, but the solutions are dipartite and need one stop destination.

**Results**

Cloud analytics is the most promising solution that helps in analyzing and aggregating data coming from different clinical assays faster enabling quicker diagnosis. Choices of programming frameworks, data analysis pipelines and development tools accompanied with flexible computational resource allocation makes cloud analytics the most deserving in this field.

Here we present interactive analytics using cloud infrastructure and distributed computing.

**Conclusions**

Icore is the faster, cheaper and smarter way of analyzing, aggregating and visualizing the data. The platform enables actionable intelligence across stakeholders in clinical research settings. It expedites the process of biomarkers discovery, analysis of drug resistance and exploration of adhoc research questions. Icore simplifies major bottlenecks of current research scenario from sample tracking to data analytics.

#### P736 Combining Multiplexed Ion Beam Imaging (MIBI) with Convolutional Neural Networks to accurately segment cells in human tissue

##### Noah Greenwald, BA^1^, ^1^Leeat Keren^1^, Shirley Greenbaum^1^, Mara Fong^1^, Gautam Chaudhry^1^, Zion Abraham^1^, Jackson Moseley^1^, David Van Valen^2^, Michael Angelo, MD, PhD^1^

###### ^1^Stanford University, Palo Alto, CA, United States; ^*2*^*Cal Tech, Pasadena, CA, United States*

####### **Correspondence:** Michael Angelo (mangelo0@stanford.edu)

**Background**

Multiplexed imaging is a rapidly growing field that promises to substantially increase the number of proteins that can be imaged simultaneously. We have developed Multiplexed Ion Beam Imaging by Time of Flight (MIBI-TOF), which uses elemental reporters conjugated to primary antibodies that are then quantified using a time of flight mass-spectrometer. This technique allows for more than 40 distinct proteins to visualized at once in the same clinical samples. This has already yielded significant insights into the interactions and relationships between the many different immune cell populations present in the tumor microenvironment. However, one of the remaining challenges in analyzing such data is accurately determining target protein expression values for each cell in the image. This requires the precise delineation of boundaries between cells that are often tightly packed next to one another. Current methods to address this challenge largely rely on DNA intensity to make these splits, and are thus mostly limited to nuclear segmentation.

**Methods**

We have developed a novel convolutional neural network to perform whole-cell segmentation from multiplexed imaging data. Rather than relying only on DNA signal, we use a panel of morphological markers. Our method integrates the information from these distinct proteins, allowing it to segment large cancer cells, small lymphocytes, and normal epithelium at the same time without requiring fine-tuning or manual adjustment.

**Results**

By combining our novel imaging platform with new computational tools, we are able to achieve extremely accurate segmentation of whole cells in tissue. Our approach compares favorably with many of the currently used tools for segmentation. We show that our improvements in accuracy come both from our novel imaging approach as well as algorithmic advances. We perform significantly better than traditional machine learning algorithms trained on the same dataset. Additionally, we show that our algorithm can be trained to identify cells across a range of cancer histologies and disease grades.

**Conclusions**

We have developed a robust and accurate approach to whole-cell segmentation in human tissues. We show the superiority over this method over current state of the art algorithms. The accurate segmentation generated by our approach will enable the analysis of complex tissue architectures with highly overlapping cell types, and will help to advance our understanding of the interactions between cell types in the diseased state.

#### P737 Model-based meta-analysis of the exposure-response and clinical efficacy across approved anti-PD(L)1 agents

##### Apurvasena Parikh, PhD^1^ , Apurvasena Parikh, PhD^1^, Sreeneeranj Kasichayanula, PHD^1^, Rajeev Menon^2^, Megan Gibbs^2^, Sven Mensing^3^, Benjamin Engelhardt^3^

###### ^*1*^*AbbVie Inc., Redwood City, CA, United States;*^*2*^*AbbVie Inc., North Chicago, IL, United States* ; ^*3*^*AbbVie Deutschland GmbH & Co KG, Ludwigshafen, Germany*

####### **Correspondence:** Apurvasena Parikh (apurvasena.parikh@abbvie.com)

**Background**

The objective of this model-based meta-analysis is to characterize the exposure-response (overall response rate [ORR]) across approved anti-PD(L)1 agents (nivolumab, pembrolizumab, atezolizumab, avelumab and durvalumab) to quantify the effect of drug, patient and trial characteristics on ORR, and assess the-relationship between ORR and efficacy outcomes such as 6-month progression-free survival (PFS) and 12-month overall survival (OS).

**Methods**

A clinical outcomes database was generated using published data for anti-PD(L)1 agents through March 2018. Reported exposures following Cycle 1 were normalized by the potency of the anti-PD(L)1 agents using respective IC50 values. Exposure-response (ORR) analysis was conducted using bayesian mixed effects modeling (R software), and included weighting by the number of subjects in each trial arm. Tumor type, number of prior therapies, ECOG performance, line of treatment, region, age, and gender were evaluated as model covariates. Impact of PD-L1 expression cut-off values was assessed to estimate the effect of PD-L1 expression on ORR. Correlation analyses were conducted between ORR, 6-month PFS and 12-month OS.

**Results**

Clinical data from 56 trials in approximately 11,000 subjects, and approximately 45 trials in 9,000 subjects across approved anti-PD(L)1s was included for the exposure-response and correlation analyses, respectively. A log-transformed linear model best described the flat exposure-response relationship, with tumor type as the most significant covariate (p < 0.001): the predicted ORR across the agents at approved dose ranges was 22% (95% CI: 16 – 30%) in non-small cell lung cancer. ORR appeared to increase by approximately 8 - 12% with greater PD-L1 expression (≥5% and ≥10% cut-offs respectively). ORR was correlated with 6-month PFS and 12-month OS (correlation coefficient, r = 0.6-0.7), and indicated a significant effect of tumor type on the correlation.

**Conclusions**

The model-based meta-analysis of exposure-response shows similar ORR depending on tumor type, across anti-PD(L)1s at the approved clinical doses. Improved efficacy was predicted with greater (i.e. ≥ 5%) PD-L1 expression.

#### P738 Artificial intelligence-based tumor purity assessment of digitized histology samples in multiple tumor types from clinical trials of nivolumab

##### George Lee, PhD, George Lee, PhD , Sujaya Srinivasan, Natallia Kalinava, Ariella Sasson, Vipul Baxi, MS

###### Bristol-Myers Squibb, Princeton, NJ, United States

####### **Correspondence:** George Lee (george.lee@bms.com)

**Background**

Tumor purity (TP) estimation is routinely performed for whole exome sequencing (WES) analysis to ensure signal is derived from cancer cells and not from other cells in the tumor microenvironment. Low TP has been shown to impact assessment of immunotherapy-related biomarkers including tumor mutational burden (TMB) [1]. While several methodologies exist to estimate TP by WES, a majority of studies do not compare against pathologist estimation, the gold standard. In this study, we utilize artificial-intelligence (AI)-based image analysis to estimate TP, and further benchmark two methodologies of TP estimation on WES against pathologist and AI estimates across 1509 pretreatment samples from patients with melanoma, non-small cell lung cancer, small cell lung cancer, or urothelial carcinoma enrolled in clinical trials of nivolumab.

**Methods**

To assess TP by WES, paired tumor-normal samples were processed by Sentieon [2] or Strelka [3] somatic variant callers with subsequent SciClone [4] tumor heterogeneity analysis. For comparison, we evaluated corresponding serial tumor sections of hematoxylin and eosin (H&E) and immunohistochemistry (IHC) digitized images for TP. A subset of H&E slides were read by pathologists, while scanned IHC images were read by deep-learning algorithms on the PathAI platform (Boston, MA) for each tumor type, to distinguish cancer cells from immune and other non-cancerous cell types based on morphology.

**Results**

For 280 samples whereby pathologist TP estimates were available, we found Pearson correlations of r=0.32 and r=0.276 for Sentieon and Strelka, respectively. Correlations improved with AI-based TP estimation, to r=0.439 (Sentieon) and r=0.321 (Strelka). Additionally, we analyzed the ability of WES-derived TP estimates to predict whether a sample had ≥60% TP as measured by pathologist or AI, according to TCGA sample acceptance guidelines [5]. Using pathologist-based TP, AUCs of 0.643 (Sentieon) and 0.625 (Strelka) were achieved. Using AI-based TP, AUCs improved to 0.753 and 0.714, respectively. Similar trends for Sentieon and Strelka were found when AI-based TP assessment was used to benchmark the larger dataset (n=1509), showing r=0.565 and 0.547, and AUCs of 0.784 and 0.777.

**Conclusions**

We demonstrated that AI-based image analysis can provide a more accurate assessment of TP compared with visual assessment by pathologist for all tumor types tested. Additionally, comparison with AI-based image analysis and pathologist estimates suggest that Sentieon TP estimates from WES may be more accurate than Strelka. Accurate assessment of TP by AI or AI-benchmarked WES may allow us to better identify patients suitable for immunotherapy via TMB and other biomarkers.

**Acknowledgements**

Bristol-Myers Squibb.

**Trial Registration**

NCT01928394, NCT02387996, NCT01673867, NCT02041533, NCT01844505

**References**

1. Rhee JK, Jung YC, Kim KR, et al. Impact of Tumor Purity on Immune Gene Expression and Clustering Analyses across Multiple Cancer Types. Cancer Immunol Res. 2017;6:87-97.

2. Saunders CT, Wong WS, Swamy S, et al. Strelka: accurate somatic small-variant calling from sequenced tumor-normal sample pairs. Bioinformatics. 2012;28:1811-1817.

3. Cibulskis K, Lawrence MS, Carter SL, et al. Sensitive detection of somatic point mutations in impure and heterogeneous cancer samples. Nat Biotechnol. 2013;31:213-219.

4. Miller CA, White BS, Dees ND, et al. SciClone: Inferring Clonal Architecture and Tracking the Spatial and Temporal Patterns of Tumor Evolution. PLoS Comput Biol. 2014;10:e1003665.

5. Aran D, Sirota M, Butte AJ. Systematic pan-cancer analysis of tumour purity. Nat Commun. 2015;6:8971.

**Ethics Approval**

The trial protocols were approved by site institutional review boards or independent ethics committees and conducted according to Good Clinical Practice guidelines, per the International Conference on Harmonisation. Patients provided written informed consent based on Declaration of Helsinki principles.

#### P739 Analysis of H&E images using deep learning for the prediction of distant metastatic recurrence in early stage melanoma

##### Michael Moore, BA^1^, Eric Robinson, BA^2^, Prathamesh Kulkarni, PhD^2^, Robyn Gartrell, MD^3^, Jaya Pradhan, DMD, MPH^1^, Emanuelle Rizk, BA^1^, Chen Yang, MD^4^, Acs Balazs, PhD^5^, Robert Phelps, MD^6^, Tammie Ferringer, MD^7^, Andrew Chen, BS^8^, Raúl Rabadán, PhD^1^, Bethany Rohr, MD^7^, Basil Horst, MD^9^, David Rimm, MD, PhD^10^, Jing Wang, MD^2^ , Yvonne Saenger, MD^1^

###### ^*1*^*Columbia University Irving Medical Cente, New York, NY, United States;*^*2*^*NYU Medical Center, New York, NY, United States*; ^*3*^*Columbia University/ New York Presbyterian, Newton, NJ, United States*; ^*4*^*Jiaotong University School of Medicine, Jiaotong, China*; ^*5*^*Karolinska Institute, Stockholm, Sweden* ; ^*6*^*Icahn School of Medicine at Mount Sinai, New York, NY, United States* ; ^*7*^*Geisinger Medical Center, Danville, PA, United States*; ^*8*^*Columbia University, New York, NY, United States* ; ^*9*^*University of British Columbia, Vancouver, Canada* ; ^*10*^*Yale University Medical Center, New Haven, CT, United States*

####### **Correspondence:** Jing Wang (jing.wang2@nyumc.org), Yvonne Saenger (yms4@cumc.columbia.edu)

**Background**

Patients with primary melanoma are at a high risk for recurrence, creating a need for biomarkers. Tumor infiltrating lymphocytes (TILs) have been previously shown to generally correlate with clinical outcome Using qmIF, we previously found that spatial relationships of immune cells within the tumor impact prognosis [1]. Herein we show that clustering of tumor and immune cells correlates with clinical outcome and find that nuclear size increases with tumor stage. Further, we applied a deep neural network (DNN) in H&E images that includes pre-defined features as well as raw image analysis to predict distant metastatic recurrence (DMR).

**Methods**

H&E’s and qmIF were evaluated on patients with stage I-III melanoma. QmIF data was analyzed using pair correlation function (PCF) to evaluate spatial relationships between cells. H&E images were processed using Qupath for nuclear segmentation and cell classification as well as the generation of data related to cell location, density, and clustering features to identify relevant tissue areas for training of network parameters. DNN architecture was composed of both convolutional neural network (CNN) and recurrent neural network (RNN) layers (Figure 1). The DNN model was trained on 108 patients from four independent institutions (Columbia, Mount Sinai, NYU and Geisinger) and validated on 115 patients from Yale and Geisinger. The DNN model analyzes image and feature information locally within an H&E image generated a prediction vote per region within the H&E image, and votes were averaged across all regions to produce an overall recurrence prediction score.

**Results**

PCF analysis of qmIF images finds that non-recurrent patients have higher degrees of clustering between tumor cells and CD8+ T Cells as well as tumor cells and HLA-DR+ macrophages (p=0.004). Prediction scores from the DNN correlated with DMR in both validation cohorts (Figure 2, AUC = 0.94 and 0.77 for Yale and Geisinger, respectively). A multivariable Cox proportional hazard model indicated that the recurrence prediction score was an independent prognostic factor for both Yale (HR = 2.54, 95% CI: 1.54-4.19, p<0.001***) and Geisinger (HR = 8.43, 95% CI: 2.58-27.51, p=0.001**).

**Conclusions**

Our DNN model incorporating features as well as raw image analysis produces an accurate prediction of DMR in H&E images of early stage melanoma, a readily available clinical pathology resource. Though larger studies are needed to further validate the DNN model, it may serve as a useful digital pathology tool to provide critical prognostic information and potentially aid in the selection of melanoma patients for adjuvant therapy.

**Acknowledgements**

This publication was supported by the National Institutes of Health through Grant Numbers R01FD006108 (Y.M. Saenger), R01NS100065 (J. Wang), and KL2TR001874 (R.D. Gartrell-Corrado). X.C is funded by the Medical Scientist Training Program (T32GM007367). R. Rabadán was supported by the NCI Center for Topology of Cancer Evolution and Heterogeneity (U54CA193313). The content is solely the responsibility of the authors and does not necessarily represent the official views of the NIH. This project also received funding from the Melanoma Research Alliance (Y.M. Saenger), Columbia University’s Irving Institute for Clinical and Translational Research (Y.M. Saenger), and Swim Across America (R.D. Gartrell-Corrado). D.L. Rimm was supported by funds from Navigate BioPharma (Novartis subsidiary), Yale SPORE in Lung Cancer (P50CA196530) and Yale Cancer Center (P30CA016359). The funding sources had no role in study design; collection, analysis, and interpretation of data; preparation of the manuscript; or the decision to submit for publication.

**References**

1. Gartrell RD, Marks DK, Hart TD, et al: Quantitative Analysis of Immune Infiltrates in Primary Melanoma. Cancer Immunol Res 6:481-493, 2018.

**Fig. 1 (abstract P739).**
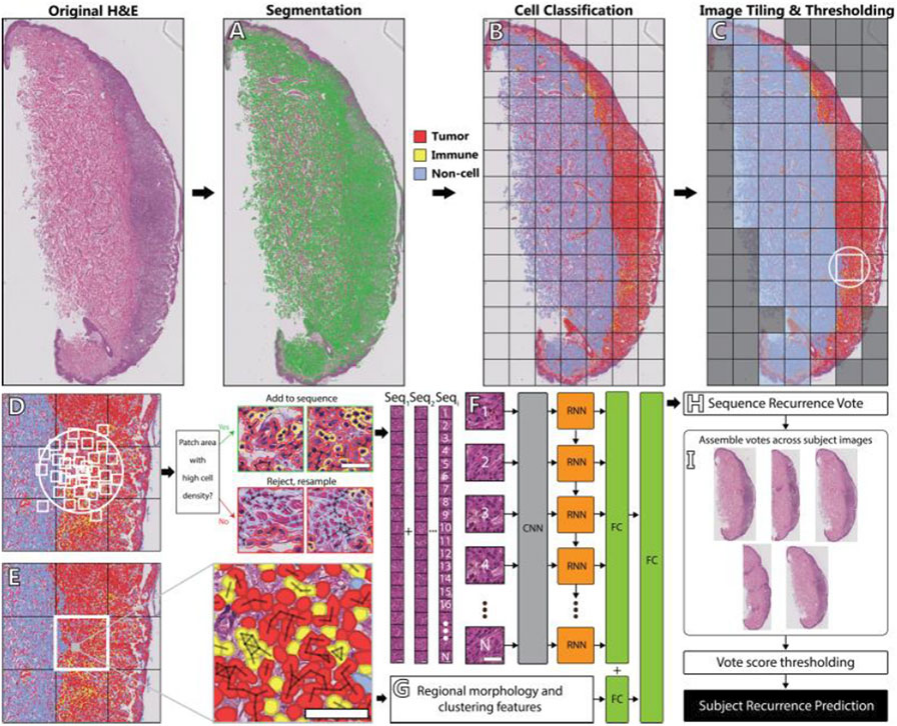
A detailed view of our approach

**Fig. 2 (abstract P739).**
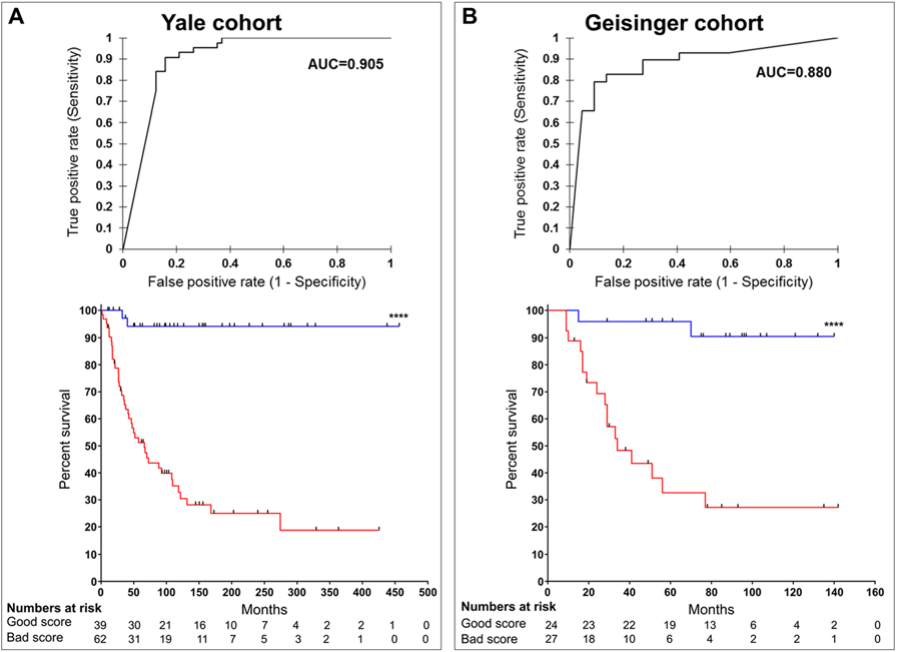
ROC and KM curves for the validation cohort

#### P740 Leveraging artificial intelligence to advance immuno-oncology drug development using functional ex-vivo 3D-tumor organoid platforms of fresh patient tissue samples

##### Jenny Kreahling, PhD, Daniel Santiago, PhD, Vijayendra Agrawal, PhD, Mibel Pabon, PhD, Soner Altiok, MD, PhD , Melba Page, PhD

###### Nilogen Oncosystems, Tampa, FL, United States

####### **Correspondence:** Soner Altiok (soner@nilogen.com)

**Background**

Traditionally, omics data analysis of biologic samples has been conducted in a single-level manner. However, integrative analysis offers an effective way to harness strength across multi-level omics data and can be more powerful than single-level analysis. In recent years we have developed complementary 3D ex vivo platforms utilizing unpropagated fresh patient tumor organoids with intact microenvironment to interrogate the efficacy of immuno-oncology drug and drug combinations. Here we aimed to develop a multiomic data integration and informatics platform to better understand the cellular and molecular mechanisms of pembrolizumab and its mode of action.

**Methods**

For the 3D-EX platform, tumor organoids were processed from fresh tumor tissue from NSCLC cancer patients obtained with IRB consent. Tumor organoids were then treated with pembrolizumab ex-vivo and treatment-mediated changes in tumor immune microenvironment were analyzed using multicolor flow cytometric analysis, multiplex cytokine assay as well as gene expression profiling by NanoString’s 770 gene Immune Panel. For the integrated multi-omics analyses, machine learning techniques included—but were not limited to—Wilcoxen Rank-Sum Test, clustering, and principal component analysis.

**Results**

Here we present the results from the integrative analyses of multiomic data generated from the ex vivo treatment of 11 fresh tumor samples in the ex vivo assays. We probed multiple biological layers in parallel, ranging from genome, epigenome, transcriptome, and proteome to phenome profiling. Our data revealed that integrative analyses using information across these data modalities deliver more comprehensive insights into the pembrolizumab mode of action and identified compensatory mechanisms likely contributing to resistance to the drug treatment. Furthermore, we identified potential molecular signatures of response to treatment.

**Conclusions**

Our results demonstrated that multiple types of omics data obtained from Nilogen’s comprehensive 3D ex vivo drug testing platforms can reveal cellular mechanisms that are active in individual tumors and may classify them into subtypes for response to drug treatment. As such, implementation of the functional 3D ex vivo assay combined with integrative multiomic data in clinical studies may enable us to ascertain which components of the tumor immune microenvironment to target within a patient.

#### P741 Prediction of biomarkers and combinational therapies for anti-PD1 therapy using a computational network approach

##### Chia Chin Wu, PhD, Andrew Futreal, Jianhua Zhang, Chia-Chin Wu

###### MD Anderson Cancer Center, Houston, TX, United States

####### **Correspondence:** Chia-Chin Wu (jzhang22@mdanderson.org)

**Background**

Despite the remarkable success, only a subset of patients can benefit from the anti-PD1 therapy. Therefore, there is a growing need to understand the resistance mechanisms for identifying predictive biomarkers and therapeutic targets for improving the therapy efficacy.

**Methods**

We hypothesized that aberrations of any gene close to MHC class I genes in a gene network are likely to deregulate MHC I pathway and affect tumor response to anti-PD1. First, the network guilt-by-association method [1] was used to calculate association scores of genes with the MHC I genes in the network. Second, pathways that enrich genes with high scores would be associated with the anti-PD1 response. Third, TCGA data was integrated with the top predicted genes to build up a signature-based response predictor of anti-PD1 for a specific cancer type. Forth, the drug target data [2] and immune infiltrate correlation data was mapped to the top predicted genes, to identify compounds that could enhance response to anti-PD1 for a cancer type.

**Results**

Our approach successfully identified genes (e.g. MYC) (Table 1) and pathways (e.g. WNT) (Fig. 1A) known to be associated with anti-PD1 response. Our prediction was further comprehensively validated by four lists of genes identified by the CRISPR screening (Fig. 1B). The integration analysis of TCGA data and our prediction reveals that our top 10% prediction significantly enrich genes whose expression are significantly correlated with the immune infiltrate score in most of cancer types (Figure 1C). We also found the mechanisms associated with anti-PD1 response would be tissue or lineage dependent (Figure 1D). The signature-based predictor built based on TCGA skin cutaneous melanoma (SKCM) data achieves an accuracy of AUC = 0.72 for 243 melanoma samples from 5 cohorts (Fig. 2A), which is slightly worse than IMPRES [3] (Fig. 2B) considering immune aspects different from ours. Integration of the predictions of ours and IMPRES improves the performance (Fig. 2C). In addition, most of targets of compounds that have been used or in clinical trial for combination treatment with anti-PD1 are in the top list of our prediction (AUC=0.83). By integrating drug target data, we further identified compounds, recently shown to be able to enhance tumor response to anti-PD1, such as inhibitors of GSK3B, CDK, and PTK2 (Table 1).

**Conclusions**

These results suggest that our approach is an effective method to identify genes/pathways associated with anti-PD1 and can be employed to screen potential drugs for combination regimens with anti-PD1.

**References**

1. Barabási AL, et al. Network medicine: a network-based approach to human disease. Nat Rev Genet. 2011; 12(1):56-68.

2. Griffith M, et al. DGIdb: mining the druggable genome. Nat Methods. 2013;10(12):1209-1210.

3. Auslander N, et al. Robust prediction of response to immune checkpoint blockade therapy in metastatic melanoma. Nat Med. 2018; 24(10):1545-1549.

4. Manguso RT, et al. In vivo CRISPR screening identifies Ptpn2 as a cancer immunotherapy target. Nature. 2017; 547:413-418.

5. Pan D, et al. A major chromatin regulator determines resistance of tumor cells to T cell-mediated killing. Science. 2018; 359(6377):770-775.

6. Patel SJ, et al., Identification of essential genes for cancer immunotherapy. Nature. 2017; 548(7669):537-542.

**Fig. 1 (abstract P741).**
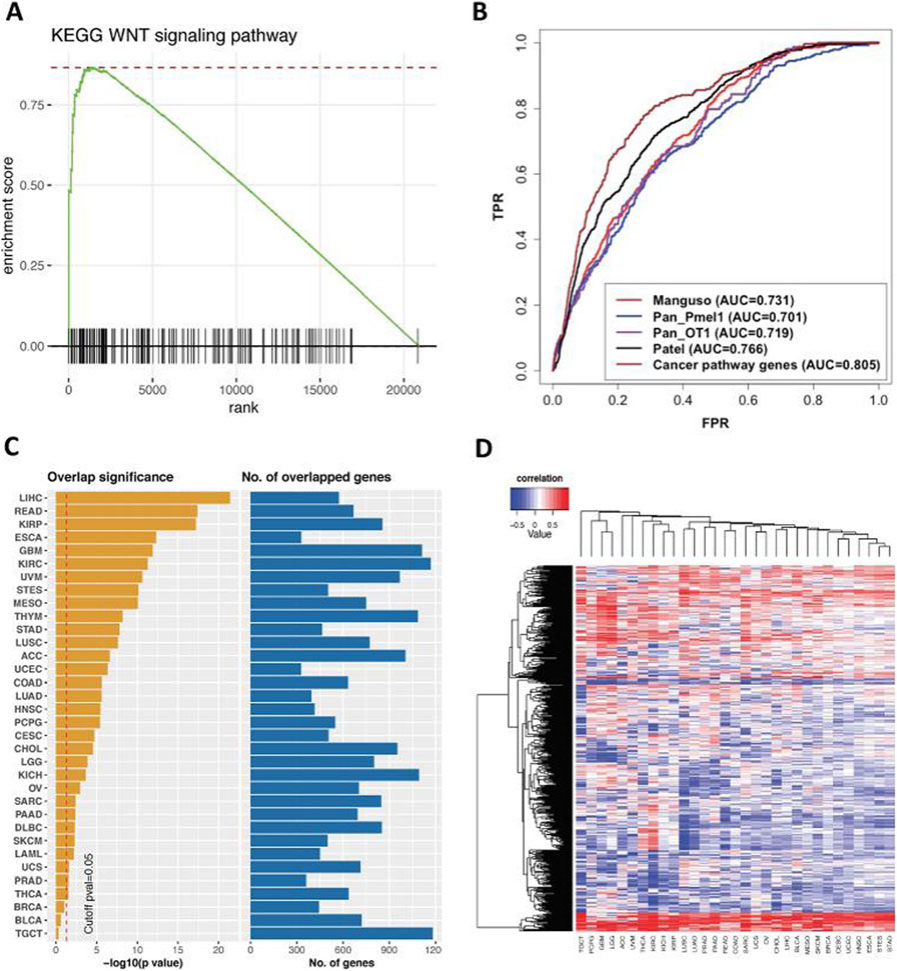
Prediction evaluation and mining TCGA Data

**Fig. 2 (abstract P741).**
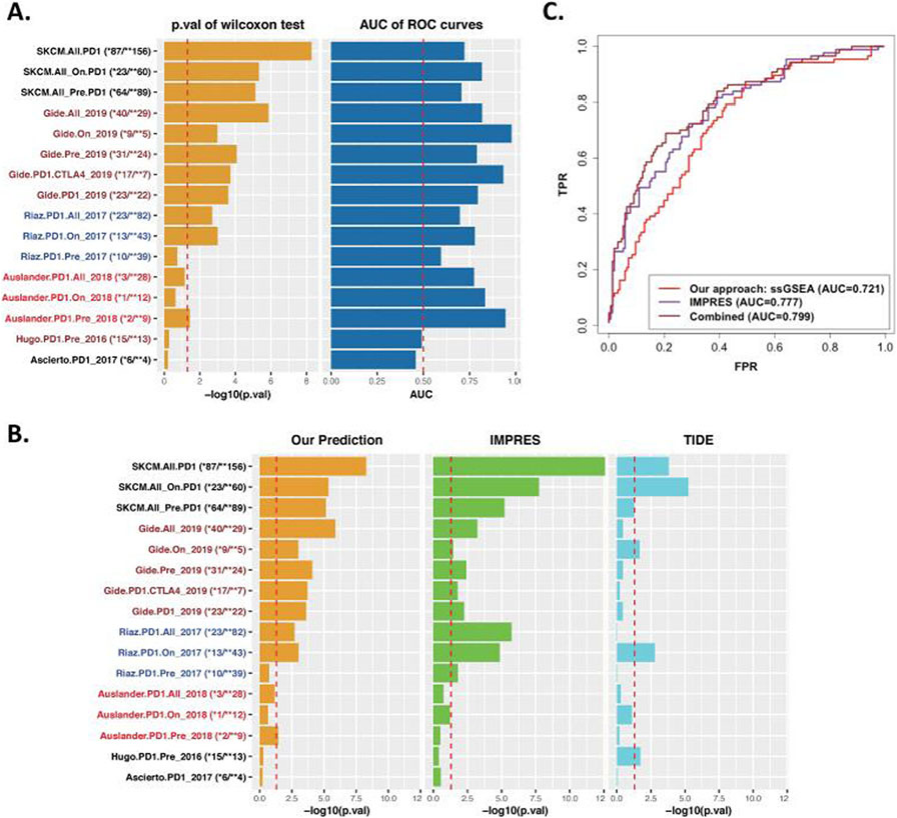
Performance of anti-PD1 response prediction

**Table 1 (abstract P741).**
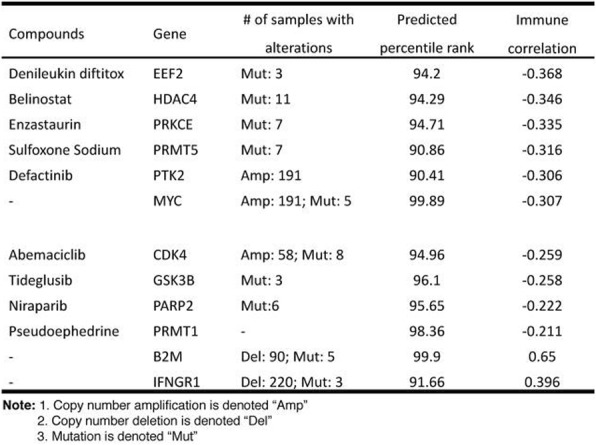
Some selected genes in the SKCM prediction

#### P742 Computational analysis of feedback regulations of TIM-3

##### JIAN ZHU, PhD^1^, JIAN ZHU, PhD^1^, Rebecca Zhu^3^

###### ^*1*^*OmicsHealth LLC, Bethesda, MD, United States;*^*2*^*OmicHealth LLC, Bethesda, MD, United States* ; ^*3*^*Winston Churchill High School, Potomac, MD, United States*

####### **Correspondence:** JIAN ZHU (jian.zhu@omicshealth.com)

**Background**

TIM-3 is an inhibitory immune checkpoint protein currently under clinical investigation. As many oncology targets, TIM3 is potentially regulated by feedback loops (FBL). Positive (+)FBLs sustain while negative (-)FBLs restrict TIM-3's expression and activity upon its induction. In the case of TIM-3 inhibition, (-)FBLs are capable of rescuing TIM-3's activity and confer therapeutic resistance. However, feedback regulations of TIM-3 have not been systematically investigated. In this study, a computational approach was developed based on literature mining and gene network analysis. The in silico models established rationales for further biomarker and combination studies in the context of TIM-3 inhibition.

**Methods**

As of July 2019, there were ~1,370 publications on TIM-3 in PubMed. Abstracts of these articles were downloaded and systematically analyzed. Information on molecular/cellular interactions together with results from preclinical and clinical studies was extracted, standardized and integrated into a graph-based database. A network-oriented querying and visualization system was established to model the interactions between cellular components, pathways and drugs.

**Results**

The computationally generated TIM-3 feedback network (Figure 1) comprises 60 nodes, 132 interactions and 855 possible FBLs (412 positive vs 443 negative). Factors contributing to (+)FBLs include up-regulation of Tregs, IL-35, TGFbeta, IL-10, BTK, SRC & HMGB1, and down-regulation of iNOS & COX2. On the other hand, IL-4, IL-2, STAT6, IL-18, PKCtheta, tryptophan, mTOR, c-Jun, IL-12, TNFalpha and CD4+ T cells are involved in (-)FBLs. Inhibition of TIM-3 may induce up-regulation of these factors which in turn up-modulate TIM-3. The gene network can be used to model drug combination effects. For example, TIM-3 inhibition activates mTOR which induces TIM-3, indicating that addition of mTOR inhibitors may enhance the efficacy of TIM-3 blockade. TIM-3 inhibition may increase tryptophan concentration through the down-regulation of Tregs, subsequently tryptophan up-regulates TIM-3 through activating PKCtheta. The addition of an IDO1 inhibitor will increase tryptophan and antagonize the immunomodulating effect of TIM-3 inhibition. It is of great interest to evaluate the combination effects of anti-TIM-3 and anti-PD-1, particularly to identify conditions rendering the combination ineffective. Anti-TIM-3 may increase the sensitivity of anti-PD-1 via up-regulation of IL-4, anti-PD-1 may up-regulate TIM-3 through IL-12 induction, the induction of TIM3- may lead to resistance to both anti-TIM-3 and anti-PD-1.

**Conclusions**

The cellular components involved in the TIM-3 FBLs are potential targets of drug combination and biomarkers for anti-TIM-3 therapies. FBLs proved to be useful tools to establish rationales and evaluate risk for preclinical and clinical development.

**Fig. 1 (abstract P742).**
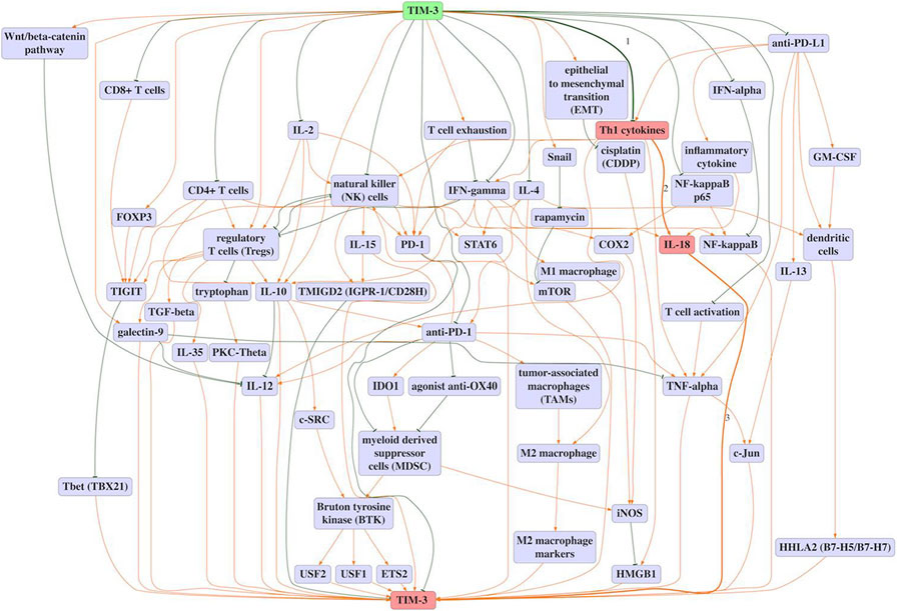
TIM-3 feedback network

#### P743 Impact of IDO1 expression and inhibition on the sensitivity of anti-PD-1

##### JIAN ZHU, PhD^1^, JIAN ZHU, PhD^1^, Rebecca Zhu^2^

###### ^1^OmicsHealth LLC, Bethesda, MD, United States; ^2^Winston Churchill High School, Potomac, MD, United States

####### **Correspondence:** JIAN ZHU (jian.zhu@omicshealth.com)

**Background**

Concurrent inhibition of IDO1 and PD-1 may have enhanced anti-tumor effects. However, inhibition of IDO1 failed to enhance efficacy of anti-PD-1 in late phase clinical trials. In this study, computational models were established to identify factors involved in interactions between these two types of agents.

**Methods**

Abstracts published on IDO1, PD-1, PD-L1, anti-PD1/PD-L1 were downloaded from PubMed, and analyzed by text mining. The information on interactions among gene, compound/therapy, cell/animal model, pathway/disease, and clinical study was extracted. Networks were constructed to simulate effects of IDO1 inhibition on anti-PD-1.

**Results**

The IDO1/anti-PD-1 network consists of 79 nodes, 227 interactions with 1277 possible regulatory paths from IDO1 to anti-PD-1. 675 paths are associated with agonism and 602 with antagonism between IDO1 inhibition and anti-PD-1. Up-regulation of TNF-alpha, IFN-gamma, type I immunity & CD8+ T cells, and down-regulation of Tregs, MDSC, JAK/STAT pathway, IL-6 and PD-L1 are associated with enhancement of anti-PD-1 by IDO1 inhibition. Besides, treatments with PARP inhibitor, EGFR inhibitor or radiation may enhance the combination efficacy of IDO1 and PD-1 inhibitors. Up-regulation of TIM-3 and NK cells are associated with reduced activities of anti-PD-1 upon IDO1 inhibition. PD-L1 is a predictive biomarker for anti-PD-1 response and its expression can be regulated by IDO1. The IDO1/PD-L1 network consists of 83 nodes, 204 interactions and 318 paths. Up-regulation of IL-6, AhR, MDSC, JAK/STAT, HIF1A, IL10, NF-kappaB & MYC was associated with induction of PD-L1 by IDO1.

**Conclusions**

As the network analyses revealed, inhibition of IDO1 could either up- or down-regulate PD-L1, and enhance or reduce efficacy of anti-PD-1. Multiple factors, other than PD-L1, play roles in determining the efficacy of combining IDO1 and PD-1 inhibitors.

**Fig. 1 (abstract P743).**
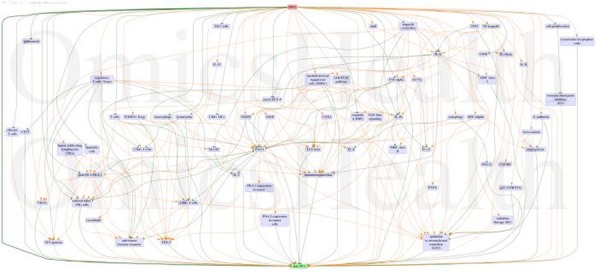
IDO1 - anti-PD-1 network

### Microbiome and Other Environmental Factors

#### P744 Physical fitness influences the composition of human T cell populations

##### Michael Gustafson, PhD^1^, Michael Gustafson, PhD^1^, Courtney Wheatley-Guy, PhD^1^, Svetlana Bornschlegl^2^, Allan Dietz, PhD^2^, Dennis Gastineau, MD^1^, Emmanuel Katsanis, MD^3^, Richard Simpson, PhD^3^, Bruce Johnson, PhD^1^

###### ^*1*^*Mayo Clinic Arizona, Phoenix, AZ, United States;*^*2*^*Mayo Clinic, Rochester, MN, United States* ; ^*3*^*University of Arizona, Tucson, AZ, United States*

####### **Correspondence:** Michael Gustafson (gustafson.michael@mayo.edu)

**Background**

Despite the remarkable success of cancer immunotherapies, a significant proportion of patients fail to respond. In order to overcome this, a number of ongoing approaches are being tested to address this need including combinatorial treatment strategies, biomarker identification to tailor optimal responses, and neoantigen discovery. One potential avenue that is emerging is the application of exercise to promote overall health. While the mechanisms of exercise-mediated improvements in health are certainly multi-factorial, a substantial amount of data suggests that physical activity can improve outcomes in cancer patients, in part by, improving immunological function. As such, we propose that physical activity may enhance the efficacy of immunotherapy by restoring the quantity and function of leukocyte subsets in cancer patients.

**Methods**

T cells and other immune cell populations were analyzed by flow cytometry from two different cohorts of healthy men[1, 2]. Physical fitness parameters including maximum rate of oxygen consumption (VO2max), physical activity, lean body mass, and BMI were assessed or measured and tested for correlations with T cell populations.

**Results**

Here, we provide evidence demonstrating that the compositions of human T cells circulating in peripheral blood are altered by physical fitness. In one cohort, late differentiated KLRG1+/CD57+/CD28- T cells were inversely associated with a cardiorespiratory fitness parameter, VO2max. In another we show several T cell populations that are associated with distinct fitness parameters. For example, in baseline peripheral blood samples, PD-1+ T cells were increased in sedentary subjects compared to active ones, numbers of CD8+ T cells and memory CD4+ T cells correlated with lean body mass, and a population containing stem cell memory CD8+ T cells were correlated with VO2max.

**Conclusions**

These results suggest that distinct T cell populations may be uniquely sensitive to both negative (age, obesity, and lack of physical activity) and positive (exercise) physiological inputs. These findings provide hypothesis-generating insights into how exercise might be utilized to overcome the immunosuppressive environment in cancer patients potentially improving their responses to immunotherapies.

**References**

1. Spielmann G, McFarlin BK, O'Connor DP, Smith PJ, Pircher H, Simpson RJ. Aerobic fitness is associated with lower proportions of senescent blood T-cells in man. Brain, behavior, and immunity. 2011;25(8):1521-9.

2. Gustafson MP, DiCostanzo AC, Wheatley CM, Kim CH, Bornschlegl S, Gastineau DA, et al. A systems biology approach to investigating the influence of exercise and fitness on the composition of leukocytes in peripheral blood. Journal for immunotherapy of cancer. 2017;5:30.

**Ethics Approval**

These studies were approved by the Institutional Review Board by their respective institutions, Mayo Clinic and University of Houston.

**Consent**

Written informed consent was obtained from the patient for publication of this abstract and any accompanying images. A copy of the written consent is available for review by the Editor of this journal.

#### P745 Tumor cell metabolism as a barrier to immunotherapy in melanoma

##### Ashley Menk, MS^1^, Greg Delgoffe, PhD^1^ , Yana Najjar, MD^2^, Ashley Menk, MS^1^, John Kirkwood, MD^2^

###### ^1^University of Pittsburgh, Pittsburgh, PA, United States; ^2^UPMC Hillman Cancer Center, Pittsburgh, PA, United States

####### **Correspondence:** Greg Delgoffe (delgoffeg@upmc.edu)

**Background**

Immunotherapy has been paradigm-shifting for melanoma, but durable responses only occur in a subset of patients. While we have previously shown that tumor-infiltrating T cells have repressed metabolic machinery, the environment itself is nutrient poor due to the deregulated metabolism of tumor cells. Recent studies have suggested that T cells compete with tumor cells for glucose and oxygen which may be limiting anti-tumor immunity. We hypothesize resistance to immunotherapy may be due to deficiencies in the metabolic makeup of the tumor microenvironment, and that we can infer that environment’s metabolism in patients by metabolically profiling tumor cells.

**Methods**

Melanoma patient samples were profiled by Seahorse analysis in parallel to flow cytometric analysis of tumor infiltrating lymphocytes (TIL). Murine melanoma cells were generated including RNAi constructs to specific metabolic pathways. Responses to PD-1 blockade and adoptive T cell immunotherapy were monitored and lymph node or TIL T cells were tested for effector function, metabolism, and localization by flow cytometry and immunofluorescence.

**Results**

Analysis of melanoma patient biopsies showed striking heterogeneity in the metabolism of tumor cells. Patients with more metabolically active tumors have more dysfunctional TIL, while tumors that were metabolically quiescent contained T cells with superior effector function. Profiling of patient tumor cells prior to PD-1 blockade therapy revealed that patients with metabolically oxidative tumors had poorer responses while patients with quiescent tumors experienced long-term response. To confirm the role of oxidative metabolism and further investigate the role of tumor cell metabolism, we generated murine melanoma lines in which glucose or oxidative metabolism was inhibited. Tumor cells in which oxidative metabolism showed an increased response to PD-1 blockade immunotherapy while tumor cells with inhibited glycolytic metabolism responded better to adoptive T cell therapy.

**Conclusions**

Our data suggest that the metabolic makeup of the tumor microenvironment, driven through deregulated oxidative or glycolytic metabolism of the tumor cell, determines whether T cells have a permissive microenvironment for effective immunotherapy, and that inhibiting tumor cell metabolism may be an attractive strategy to improve the efficacy of immunotherapy.

#### P746 Global metabolomics of advanced gastric cancer patients identifies signatures associated with response and clinical benefit from nivolumab

##### Carrie Brachmann, PhD^1^, Carrie Brachmann, PhD^1^, Daniel Cooper^2^, Kari Wong^2^, Ethan Grant^1^, Daniel Catenacci, MD^3^, David Cunningham^4^, Jean-Philippe Metges^5^, Eric Van Cutsem, MD, PhD^6^, Zev Wainberg, MD^7^, Dung Thai^1^, Pankaj Bhargava^1^, Emon Elboudwarej^1^, Ondrej Podlaha^1^, Wadud Khan^8^, Jennifer Wargo, MD, MMSc^8^, Manish Shah, MD^9^

###### ^*1*^*Gilead Sciences, Foster City, CA, United States;*^*2*^*Metabolon, Inc, Morrisville, NC, United States* ; ^*3*^*University of Chicago Medical Center, Chicago, IL, United States* ; ^*4*^*Sutton and London Hospital, London, United Kingdom* ; ^*5*^*Brest University Hospital* ; ^*6*^*University Hospitals Leuven & KU Leuven, Leuven, Belgium*; ^*7*^*UCLA School of Medicine, Santa Monica, CA*; ^*8*^*UT MD Anderson Cancer Center, Houston, TX, United States*; ^*9*^*Weill Cornell Medicine, NY Pres Hosp, New York, NY*

####### **Correspondence:** Carrie Brachmann (carrie.brachmann@gilead.com)

**Background**

The gut microbiome has been reported to impact the immune response to checkpoint blockade [1]. Circulating metabolites relate to both host and microbiome biology and were explored in an advanced gastric cancer population treated with nivolumab.

**Methods**

Plasma samples (pre-treatment, week 2, week 8 and end of study) from patients enrolled in a randomized phase 2 study in >=2nd line advanced gastric or gastroesophageal junction patients [2] were extracted for analysis on the LC/MS/MS and Polar LC platforms. 141 patients were treated and all received nivolumab. This analysis evaluates all patients as a single population, since andecaliximab failed to provide additional benefit [2-4]. ANCOVA analyses were performed for samples stratified by responder/non-responder status or patients that received clinical benefit versus those that did not receive clinical benefit and were adjusted for age and sex. Clinical benefit was defined as patients living >1 year post treatment or had a tumor response. Results with nominal p

**Results**

Metabolic profiling of human plasma detected 1364 metabolites. The majority of metabolic changes that occurred while the patients were on treatment were detected at end of study (151 and 35 significantly altered metabolites in non-responders and responders, respectively) when compared to the pre-treatment time point. Clinical benefit from checkpoint blockade was associated with higher levels of bacterial-derived indoles, host-derived tryptophan catabolites, lower primary and secondary bile acids, lower levels of lactate, and altered glutamine catabolism. Similar differences were observed when responders and non-responders were compared.

**Conclusions**

The association of microbial indoles and host-derived tryptophan catabolites with benefit suggests the activation of immunosuppressive mechanisms in non-responders and those who did not experience clinical benefit. Combined with the differences in secondary bile acids, these data suggest a role for the microbiome. Indeed, tryptophan catabolites can be produced by Clostridiales and enrichment of these bacteria are linked to improved response to anti-PD1 therapy [5]. In addition, differences in circulating lactate, glutamine, and glutamate between responders and non-responders suggested differences in tumor metabolic preferences, flexibility, or overall activity.

**Acknowledgements**

The authors gratefully acknowledge the patients and their families who participated in this study.

**Trial Registration**

Clinicaltrials.gov NCT02862535

**References**

1. Routy B, Le Chatelier E, et al. Gut microbiomer influences efficacy of PD-1-based immunotherapy against epithelial tumors

2. Shah M, Metges J-M, et al. A phase 2, open-label, randomized study to evaluate the efficacy and safety of andecaliximab combined with nivolumab versus nivolumab alone in subjects with unresectable or recurrent gastric or gastroesophageal junction adenocarcinoma. ASCO Gastrointestinal Cancers Symposium. 2019.

3. Metges J-M, Elboudwarej E, et al. Exploratory evaluation of baseline tumor biomarkers and their association with response and survival in patients with previously treated advanced gastric cancer treated with andecaliximab combined with nivolumab versus nivolumab. ASCO Gastrointestinal Cancers Symposium. 2019.

4. Brachmann C, Zhang Y, et al. Evaluation of intratumoral T cells in biopsies from advanced gastric patients treated with andecaliximab and nivolumab. ASCO Gastrointestinal Cancers Symposium. 2019.

5. Gopalakrishnan V, Spencer CN, et al. Gut microbiome modulates response to anti-PD-1 immunotherapy in melanoma patients. Science. 2018.

**Ethics Approval**

This study was approved by the institutional review board or independent ethics committee appropriate for each site.

#### P747 Leveraging the Second Genome platform to develop microbiome-derived cancer immunotherapies

##### Dhwani Haria, Jayamary Ravichandar, Lynn Yamamoto, Cheryl-Emiliane Chow, Bernat Raja, Joanna Dreux, Kareem Graham, Kathryn Iverson, Shoko Iwai, Sunit Jain, Yuliya Katlinskaya, Sabina Lau, Jina Lee, Michelle Lin, Paul Loriaux, Nicole Narayan, Erica Rutherford, Connor Skennerton, Thomas Weinmaier, Michi Wilcoxon, Yonggan Wu, Todd DeSantis, Toshihiko Takeuchi, Karim Dabbagh, Helena Kiefel, PhD , Helena Kiefel, PhD

###### Second Genome, South San Francisco, CA, United States

####### **Correspondence:** Helena Kiefel (helena@secondgenome.com)

**Background**

The gut microbiota has emerged as an important player in cancer pathology and increased evidence supports its influence on clinical response to immune checkpoint inhibitor (ICI) therapy. However, the specific gut bacteria-derived molecules responsible for the improved response to ICI therapy remain unknown. Second Genome has developed a unique discovery platform to identify, screen, and validate microbiome-derived peptides that promote response to cancer-immunotherapy.

**Methods**

Using our multi-technology meta-analysis of published datasets characterizing the baseline microbiome from melanoma patients treated with anti-PD-1, we have identified gut microbiota strains differentially abundant in responders versus non-responders that are concordant across multiple cohorts. Next, peptides from the strains associated with responder signatures were predicted from their genome sequences. In addition, we identified and selected predicted bacterially-derived peptides associated with responder signatures from assembled metagenomes. All predicted peptides were screened using phage display technology to select binders to immune cells known to play a role in the tumor microenvironment (TME).

**Results**

Selected microbiome-derived peptide binders were evaluated for activity in cell-based assays using isolated human primary T cells, dendritic cells, and macrophages. We show that several selected peptide binders induce secretion of proinflammatory cytokines and chemokines (e.g., IP-10, TNF) from human primary monocyte-derived dendritic cells, as well as effector cytokine secretion (e.g., IFNγ, IL-2) from human primary T lymphocytes. Additionally, we have identified microbiome-derived peptide binders with capacity to inhibit an M2-like phenotype in macrophages (decreased LPS-induced IL-10 secretion).

**Conclusions**

Collectively, our results demonstrate the potential of Second Genome’s discovery platform to identify and characterize novel immunomodulatory factors produced by the gut microbiota. Second Genome’s discovery platform offers a unique approach to identify novel agents with potential for use as therapeutics in cancer immunotherapy.

#### P748 A rationally-designed consortium of human gut commensals induces IFNg+ CD8+ T cells and enhances tumor immunity

##### Amanda Prince, PhD^1^, Johnny Lightcap^2^, Amit Reddy^2^, Sachin Deshmukh^2^, Steve McClellan^2^, Bruce Roberts, PhD^1^ , Rose Szabady^1^, Arthur Frankel^2^

###### ^1^Vedanta Biosciences, Inc., Cambridge, MA, United States; ^2^University of South Alabama, Mobile, AL, United States

####### **Correspondence:** Bruce Roberts (broberts@vedantabio.com), Arthur Frankel (afrankel@health.southalabama.edu)

**Background**

Efficacy of immune checkpoint inhibitor (ICI) therapy is associated with the composition of the gut microbiome, but the mechanism remains to be elucidated. Recent data from Tanoue et al. [1] demonstrated a consortium of 11 bacteria (VE800) induced IFNg production in intestinal CD8+ T cells and enhanced anti-PD-1 cancer activity in tumor models. Vedanta has developed VE800 as a microbiome therapeutic to enhance the efficacy of ICI therapy. Here, we aimed to further understand the requirements for VE800 efficacy in pre-clinical models and to understand how immune modulation by VE800 affects tumor immunity.

**Methods**

We used IFNg production in colonic T cells as a readout for VE800 anti-cancer efficacy. Mice were pre-treated with different antibiotics to establish a permissive niche for colonization and given VE800. To determine which bacteria were critical contributors to VE800 function, we created various combinations of individual VE800 bacterial strains and measured induction of IFNg+ CD8+ T cells in the intestines of germ-free mice. Consortia unable to induce IFNg+ CD8+ T cells were tested for their anti-tumor efficacy with ICI in the B16 tumor model. In this model, C57BL/6 mice were pre-treated with antibiotics to deplete endogenous microbiota, injected with B16, and treated with ICI on days 4, 8, and 12. VE800 was administered three times a week for three weeks prior to sacrifice to assess levels of tumor infiltrating cells.

**Results**

Our data suggest that bacterial density drives VE800 efficacy for induction of IFNg+ CD8 T cells. We further established that the complete VE800 consortium provides maximal IFNg induction, and that removal of specific strains impairs IFNg induction. Utilizing the B16 tumor model, colonization of VE800 strains was observed in tumor-bearing mice receiving the complete consortia. Additionally, the complete VE800 consortium restored ICI efficacy in antibiotic-treated tumor-bearing mice (Figure 1). However, bacterial consortia incapable of inducing IFNg+ CD8+ T cells do not promote anti-tumor immunity. Pre-treatment with antibiotics abolished the therapeutic effect of ICI, which was accompanied by a reduced level of tumor infiltrating immune cells indicating the important, supportive role played by gut microbiota.

**Conclusions**

Our data suggests VE800 induces IFNg+ CD8+ T cell induction and provides the necessary microbial component to promote anti-tumor immunity in combination with dual checkpoint inhibitor therapy in the multiple tumor models.

**References**

1. Tanoue T, Morita S, Plichta DR, Skelly AN, Suda W, Sugiura Y, Narushima S, Vlamakis H, Motoo I, Sugita K, Shiota A, Takeshita K, Yasuma-Mitobe K, Riethmacher D, Kaisho T, Norman JM, Mucida D, Suematsu M, Yaguchi T, Bucci V, Inoue T, Kawakami Y, Olle B, Roberts B, Hattori M, Xavier RJ, Atarashi K, Honda K. A defined commensal consortium elicits CD8 T cells and anti-cancer immunity. Nature. 2019 Jan 23.

**Fig. 1 (abstract P748).**
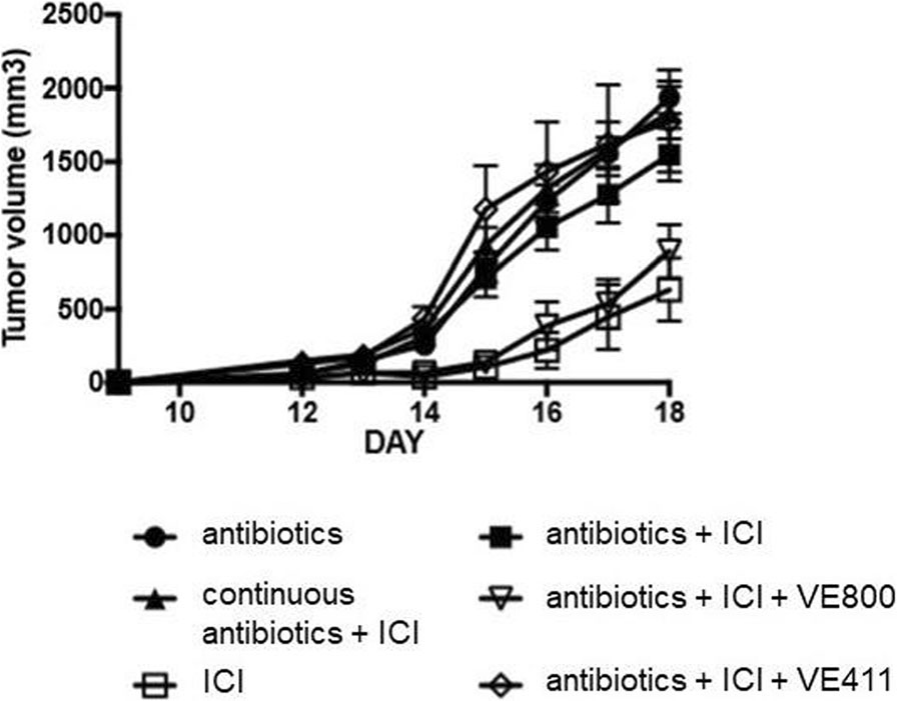
VE800 promotes anti-tumor responses in a B16 melanoma mouse

#### P749 Investigation of immune-microbiota interaction in lung cancer using genetically engineered mouse models

##### Chengcheng Jin, PhD, Tyler Jacks, PhD , Georgia Lagoudas

###### Massachusetts Institute of Technology, Cambridge, MA, United States

####### **Correspondence:** Tyler Jacks (tjacks@mit.edu)

**Background**

The development of lung cancer is closely associated with chronic inflammation, but the source of such inflammation has not been clearly defined and the contribution of specific cellular and molecular component of the immune system is yet to be elucidated. Pulmonary infections are very common in lung cancer patients and affect clinical outcomes, but these associations are not understood in molecular details. Although increasing evidence has linked commensal microbiota to oncogenesis and cancer response to therapy, the current literature has been mainly focused on the intestinal microbiota. It remains unclear how the distal intestinal microbiota and local airway microbiota act together to regulate the malignant transformation in the lung.

**Methods**

We interrogate the immune-microbiota interaction in lung cancer using an autochthonous genetically engineered mouse model of lung adenocarcinoma driven by oncogenic Kras and loss of p53. Lung and intestinal microbiome were characterized by 16S rDNA-based sequencing analysis. Antibiotics treatment and germ-free animals were used to examine the functional importance of microbiota in lung cancer development. Tumor-associated immune cells were profiled by flow cytometry, bulk and single-cell RNA-seq.

**Results**

We found lung cancer development was associated with increased total bacterial load and reduced bacterial diversity. This dysregulated local microbiota stimulated IL-1β and IL-23 production from myeloid cells, resulting in proliferation and activation of lung-resident Vγ6+Vδ1+ γδ T cells. These γδ T cells produced IL-17 to promote neutrophil infiltration and inflammation in the tumor microenvironment; they also expressed IL-22, amphiregulin and other effector molecules to directly enhance tumor cell proliferation. Mice rederived to germ-free condition or treated with combined antibiotics were profoundly protected from lung cancer initiation and progression, whereas intratracheal inoculation of bacterial mixture isolated from late-stage lung tumors robustly accelerated tumor growth in naïve animals. Bulk and single-cell RNA-seq based transcriptional profiling further revealed that microbiota induced robust gene expression changes in a number of tumor-associated immune cell populations. In addition, we also performed 16S FISH and multiplex IF to show the direct interaction between bacterial and immune cells in the native lung tumor microenvironment. Therapeutically, we found blocking the γδ T or its downstream IL-17, IL22 signaling all significantly suppressed tumor growth in the lung.

**Conclusions**

Our study provides strong evidence that local microbiota play a critical role in promoting lung cancer development by provoking tumor-associated inflammation. The key cellular and molecular mediators that we identified in this study may serve as effective targets in lung cancer treatment and prevention.

#### P750 Predicting response to anti-PD1 therapy from metagenomic sequencing data with machine learning

##### Jan Majta, MSc, Krzysztof Odrzywołek, Agata Szymanek, Monika Majchrzak-Gorecka, Szymon Wojciechowski, Zuzanna Karwowska, Kaja Milanowska

###### Ardigen, Krakow, Poland

####### **Correspondence:** Kaja Milanowska (kaja.milanowska@ardigen.com)

**Background**

Recent years have brought the breakthrough to the immune checkpoint therapy (ICT) research with the discovery of the role of gut microbiota (GM) in the treatment response rate. A number of publications describes not only the association, but more importantly the impact of intestinal communities on ICT response [1–3].

The published research points to the specific traits of GM that are assocated with its beneficial role. A common approach identified ‘responder gut’ with general metrics of GM like high diversity or richness, which can be associated with non-dysbiotic intestine. Furthermore, each study identified specific clades enriched in responder or non-responder group.

Nevertheless, the taxonomic profile of samples is insufficient to build a predictive model of ICT response [4]. Additionally, bacteria identified in a single cohort were not found significant in any other study [5]. It suggests that not the taxonomy, but the function should be a primarily analyzed property in microbiome research. More importantly, the design of microbiota-based treatment requires more specific features than diversity, e.g. a certain strain or function.

Therefore, we believe it is essential to develop an algorithm that combines high quality machine learning with interpretable, actionable results.

**Methods**

We carried out a classification study to identify responders to anti-PD1 treatment for metastatic melanoma, using shotgun metagenomic sequencing data from three published cohorts. We developed a novel algorithm - BiomeScout - to build cohort-specific feature space and predict therapy outcome for each patient.

**Results**

In analyzed cohorts we detected a function-associated signal and used it for R/NR classification. We benchmarked BiomeScout’s performance on the Houston cohort achieving AUC=0.76, outperforming already published results [4]. Moreover, BiomeScout selects a set of functional features along with their sequential representation, ready to be used as a biomarker or a component for Live Biotherapeutic Product (LBP) design.

**Conclusions**

Presented work advocates for the importance of function-oriented microbiome research in ICT. Furthermore, BiomeScout’s algorithms enables detection of a cross-cohort signal which is currently in testing phase.

**References**

1. Gopalakrishnan V, Spencer CN, Nezi L, Reuben A, Andrews MC, Karpinets TV, et al. Gut microbiome modulates response to anti-PD-1 immunotherapy in melanoma patients. Science. 2018;359: 97–103.

2. Matson V, Fessler J, Bao R, Chongsuwat T, Zha Y, Alegre M-L, et al. The commensal microbiome is associated with anti-PD-1 efficacy in metastatic melanoma patients. Science. 2018;359: 104–108.

3. Routy B, Le Chatelier E, Derosa L, Duong CPM, Alou MT, Daillère R, et al. Gut microbiome influences efficacy of PD-1-based immunotherapy against epithelial tumors. Science. 2018;359: 91–97.

4. Gharaibeh RZ, Jobin C. Microbiota and cancer immunotherapy: in search of microbial signals. Gut. 2019. pp. 385–388. doi:10.1136/gutjnl-2018-317220

5. Trinchieri G. The microbiome in cancer therapy. American Association for Cancer Research Annual Meeting 2019. Mar 29 - Apr 3 Atlanta, GA

#### P751 A targeted next generation sequencing assay to analyze and characterize the gut microbiome for health research

##### Anna McGeachy, PhD, Shrutii Sarda, David Merrill, Birgit Drews, Wing Lee, Heesun Shin, Janice Au-Young, Rajesh Gottimukkala, MS, Aren Ewing, Fiona Hyland

###### Thermo Fisher Scientific, South San Francisco, CA, United States

####### **Correspondence:** Fiona Hyland (fiona.hyland@thermofisher.com)

**Background**

The gut microbiota has emerged as a promising biomarker to assess the efficacy of immune-modulatory drugs in the CTLA-4 or PD-1 pathways. Targeted Next Generation Sequencing may provide a lower cost approach for characterizing the Gut Microbiome. Sequencing of 16S rRNA is one approach to characterize microbial diversity. However, 16S sequencing alone is often insufficient to gain species level information. Here we describe a Targeted Sequencing panel comprising both 16S and species-specific primers using Ion AmpliSeqTM technology. This assay can be used to understand the composition and diversity of the human gut microbiome at the species level.

**Methods**

Our improved assay includes customized primers to multiple hypervariable regions of the 16S region. However, 16S alone can still be insufficient in distinguishing highly homologous organisms such as Bacteroides and Lactobacillus. We have designed species-specific primers that provide high strain coverage and high specificity performance. We have tested primers for 75 species associated with human health and disease, from genera such as Bifidobacterium, Clostridium, Ruminococcus and Bacteroides. With this approach, we can analyze reads generated from sequencing to report taxonomic classifications and relative abundance for organisms in the sample with high specificity and sensitivity.

**Results**

We designed a multiplexed Ion AmpliSeqTM assay including 16S targets and species-specific markers targeting important microbial species. Using the Ion Genestudio S5™ system, We sequenced two mock community controls comprising of 20 and 12 bacterial strains. With our more comprehensive 16S design, we positively identified all the organisms at Genus level with 100% sensitivity. With the data generated from the species-specific primers, we identified all the targeted species in the ATCC samples at the Species level. We also created mixed population pools using DNA from clonal isolates of 40 different bacterial strains. We identified all 40 microbes at Genus level with 100% sensitivity using 16S alone. We improved the detection resolution to the Species level through the application of our species-specific primers.

**Conclusions**

Using the Ion AmpliSeq™ technology, we have created a highly sensitive and specific assay for robust characterization of microbiota. This highly multiplexed PCR approach enables an efficient and affordable means for conducting extensive analyses of the human microbiome having applications in the study of the etiology of phenotypic variability and the relationship to disease, resistance, and therapy.

#### P752 Antitumor activity of neoantigen cancer vaccine delivered by DNA electroporation is driven by the microbiota

##### Lucia Lione^1^, Erika Salvatori^1^, Antonella Conforti^2^, Gennaro Ciliberto^3^, Luigi Aurisicchio^4^, Fabio Palombo, PhD^1^ , Fabio Palombo, PhD^1^

###### ^*1*^*Takis, Rome, Italy;*^*2*^*Evvivax, Rome, Italy* ; ^*3*^*Regina Elena, Rome, Italy* ; ^*4*^*Biogem, Takis, Italy*

####### **Correspondence:** Fabio Palombo (palombo@takisbiotech.it)

**Background**

The microbiota has an impact on different aspects of cancer immunology. We reasoned that it might also influence cancer vaccine delivered by DNA-EP.

**Methods**

Mice were treated with antibiotics (ABX) for two weeks and vaccinated with a plasmid DNA encoding MC38 neoantigens delivered by electroporation (EP) [1]. Analysis of T cell immune responses was carried out with flow cytometry either in the peripheral blood or in the spleen. One week after last vaccination mice were challenged with MC38 cells. The microbiota was monitored in the feces by bacterial cultures and NGS analysis of 16 S.

**Results**

Unexpectedly, ABX treatment allowed a statistically significant reduction of tumor growth in mice vaccinated with DNA-EP. In these experimental conditions, the DNA vaccine is not effective in controlling mice untreated with ABX. NGS analysis of the microbiota composition revealed the presence of clostridiaceae in ABX treated mice.

**Conclusions**

Clostridiaceae have been associated with the production of specific short-chain fatty acids (SCFA) mainly butyrate and with the induction of T -reg. Very recently the metabolites produced by the intestinal microbiota have been also associated with memory potential of activated CD8 cells [2]. The observation that anti-tumor property of DNA-EP depends on the lack of normal mouse microbiota suggests new possibilities for improving this potent vaccine method

**References**

1. Aurisicchio L, Salvatori E, Lione L, Bandini S, Pallocca M, Maggio R, Fanciulli M, De Nicola F, Goeman F, Ciliberto G, Conforti A, Luberto L, Palombo F, Poly-specific neoantigen-targeted cancer vaccines delay patient derived tumor growth. J Exp Clin Cancer Res. 2019 Feb 14;38(1):78

2. Bachem A, Makhlouf C, Binger KJ, de Souza DP, Tull D2, Hochheiser K, Whitney PG, Fernandez-Ruiz D, Dähling S, Kastenmüller W, Jönsson J, Gressier E, Lew AM, Perdomo C, Kupz A, Figgett W, Mackay F, Oleshansky M, Russ BE, Parish IA, Kallies A, McConville MJ, Turner SJ, Gebhardt T, Bedoui S. Microbiota-Derived Short-Chain Fatty Acids Promote the Memory Potential of Antigen-Activated CD8+ T Cells. Immunity. 2019 Jun 26. pii: S1074-7613(19)30247-X

#### P753 Trial in progress: A phase I study of live biotherapeutic MRx0518 in the neoadjuvant setting for patients awaiting surgical removal of solid tumours

##### John Weinberg, MD^1^, John Weinberg, MD^1^ , Jonathan Krell, PhD, MRCP, MBChB, BSc^2^

###### ^1^4D pharma plc, Leeds, United Kingdom; ^2^Imperial College, London, London, United Kingdom

####### **Correspondence:**John Weinberg (john.weinberg@4dpharmaplc.com)

**Background**

The gut microbiome has emerged as a therapeutic approach to immune-stimulation treatment of solid tumours[1,2,3]. MRx0518 is a gut microbiome-derived, oral live biotherapeutic, identified as inducing an immunostimulatory response, particularly in stimulating immune cell populations and signalling pathways relevant for anti-cancer therapy. For example, the bacterial flagellin moiety leads to an NFκB-related cytokine responses4. Preclinical studies have demonstrated that MRx0518 reduced tumour growth in syngeneic models of kidney, lung and breast cancer. MRx0518 increased CD8 T cell infiltration into the tumour and decreased Tregs along with up-regulation of tumour TLR5.

This study, one of the first oncology trials conducted with live biotherapeutics, is a single-centre, double blind, neoadjuvant window study in patients with resectable solid tumours to evaluate the safety, tolerability and preliminary efficacy of MRx0518.

**Methods**

The trial consists of 2 parts. In Part A, 20 patients receive 1 capsule (bid) of MRx0518 for 2–4 weeks prior to tumour resection. In part B, 80 patients receive 1 capsule (bid) of MRx0518 and 20 patients receive 1 capsule (bid) of placebo for 2-4 weeks prior to resection.

The primary end points are safety and tolerability of MRx0518 (Parts A and B). Secondary end points include responses in respect of intra-tumoral markers compared to placebo, and clinical outcomes including overall survival. Exploratory end points include surrogate biomarkers of treatment effect, the effect on microbiota and impact on tumoral and circulating T-cell populations. Recruitment is ongoing. Clinical trial information: NCT03934827

Eligibility is limited to treatment-naïve patients with confirmed solid tumours amenable to primary surgery resection with a comparison of the diagnostic biopsy and surgical excision specimen to assess the biomarker response.

**Trial Registration**

www.clinicaltrials.gov

NCT03934827

**References**

1. Nelson KE, An Update on the Status of Current Research on the Mammalian Microbiome., ILAR K., 2015, 56: 163-168

2. Dzutsev A, Goldszmid RS, Viaud S et al., The role of the microbiota in inflammation, carcinogenesis, and cancer therapy., Eur J Immunol., 2015, 45: 17-31

3. Sivan A, Corrales L, Hubert N at al., Commensal Bifidobacterium promotes antitumor immunity and facilitates anti-PD-L1 efficacy., Science, 2015, 350: 1084-1089

4. Lauté-Caly DL, Raftis EJ, Cowie P et al., The flagellin of candidate live biotherapeutic Enterococcus gallinarum MRx0518 is a potent immunostimulant., Sci Rep., 2019, 9: 801-814

**Ethics Approval**

The study was approved by the East of England – Cambridge East Research Ethics Committee, reference 18/EE/0091.

#### P754 Using advanced imaging techniques to study the interactions among malignant cells, immune system, and microbiota in lung adenocarcinoma

##### Chen Zhao, MD^1^, Weizhe Li^1^, Chengcheng Jin, PhD^2^, Tyler Jacks^2^, Ronald Germain, MD, PhD^3^

###### ^*1*^*National Institutes of Health (NIH), North Bethesda, MD, United States;*^*2*^*Massachusetts Institute of Technology (MIT), Cambridge, MA, United States*; ^*3*^*National Institute of Allergy and Infectious Diseases (NIAID), Bethesda, MD, United States*

####### **Correspondence:** Ronald Germain (rgermain@nih.gov)

**Background**

Lung cancer is the leading cause of cancer-related mortality worldwide. Although immune checkpoint inhibitors have shown encouraging results in the treatment of lung adenocarcinoma, the response rate and overall survival are still low, especially in patients with low PD-L1 expression in the tumor site. Existing evidence suggests that interactions involving lung cancer cells, microbes, and immune cells in the tumor microenvironment affect tumor progression and patients’ responses to treatments.

**Methods**

Here we used advanced imaging techniques, such as histo-cytometry [1], Clearing-enhanced 3D (Ce3D) microscopy [2] and multiplex FISH, together with single-cell RNA sequencing and high-dimensional unsupervised spatial-temporal analysis, to study the tumor-immune microenvironment in an autochthonous genetically engineered mouse model of lung adenocarcinoma, which is driven by an activating mutation of Kras and a loss of function mutation of p53.

**Results**

These studies have revealed a role for local dysbiosis in tumor progression, a connection to interleukin-17 secreting gamma-delta T cells in the immune response to the tumor, and the presence in the tumor micro-environment of an unexpectedly heterogeneous population of neutrophils, a cell type known to have adverse effects in lung adenocarcinoma.

**Conclusions**

Together, these findings are beginning to produce a more complete picture of the three-way interaction among malignant cells, the immune system, and the microbiota in driving tumor progression, with implications for possible interventions to decrease tumor support and facilitate useful anti-tumor immunity.

**Acknowledgements**

This research was supported by the Intramural Research Programs of NIAID and NHLBI, NIH.

**References**

1. Gerner, M. Y., W. Kastenmuller, I. Ifrim, J. Kabat and R. N. Germain. Histo-cytometry: a method for highly multiplex quantitative tissue imaging analysis applied to dendritic cell subset microanatomy in lymph nodes. Immunity,2012, 37(2): 364-376.

2. Li, W., R. N. Germain and M. Y. Gerner. Multiplex, quantitative cellular analysis in large tissue volumes with clearing-enhanced 3D microscopy (Ce3D). Proc Natl Acad Sci U S A, 2017, 114(35): E7321-E7330.

#### P755 Microbiome spatial patterns as markers of cancer immune therapy response

##### Bokai Zhu, BS, Yunhao Bai, BS, Sizun Jiang, PhD , Xavier Rovira-Clave , Justin Sonnenburg, Garry Nolan

###### Stanford University, Stanford, CA, United States

####### **Correspondence:** Sizun Jiang (sizunj@stanford.edu), Xavier Rovira-Clave (xrovira@stanford.edu), Garry Nolan (gnolan@drowlab.com)

**Background**

In the past decade, numerous studies of microbiota have shown the tremendous impact of this symbiont community on human health. With the advancement of high-throughput sequencing technologies, the composition of microbiota communities, which used to be elusive, can now be quantified and categorized, even towards the resolution of single bacterial strains. More importantly, numerous therapeutical implications associated with the microbiome have been discovered and are under active exploration.

Accumulating evidence support that microbiota can specifically influence the efficacy of certain cancer immunotherapies: 1. Species of Bifidobacterium are positively correlated with anti-PD-1 therapy in mice; 2. Species of Bacteroidales can trigger Th1 response and promote DC maturation during anti-CTLA-4 therapy; 3. Antibiotic treatment remarkably decreases the effect of anti-CTLA-4 therapy. These discoveries have motivated various research projects, aiming to identify novel microbial-based intervention to enhance the efficacy of cancer immunotherapy in patients. One of the major confounding factors impeding a more detailed understanding of these microbial-host interactions is the lack of appropriate methodology to explore the microbial-tumor interactions and microbial anti-tumor activation of the immune system.

**Methods**

Mice harboring a humanized gut microbiome were sacrificed, and colon tissue collected. We then performed Multiplexed Ion Beam Imaging of these samples to understand the spatial structure of the microbiome and corresponding host responses by a combination of ISH and antibody staining. To establish a highly specific bacteria phylum specific ISH library, oligo probes were designed to detect specific bacteria groups and antibody targeting cell types markers or host response presented in the same location.

**Results**

We have optimized and validated a panel of ~ 20 antibodies either targeting cell type makers presented in the small intestine and colon, including immune cell (CD3, CD4 etc), intestinal epithelial cell (DCLK-1etc ), or targeting host functional marker including histone modification (H3K27me3, H3K27ac), inflammatory response (MPO, pSTAT3 etc) or mucus secretion (UEA-1). We have also built an oligo design pipeline to establish a highly specific oligo probe panel targeting the major Bacteria phylum in the human gut microbiome.

**Conclusions**

We introduce microMIBI, a multiplexed imaging technique that combines bacteria-specific ISH with host specific protein detection, with potentially >40 parameters (Figure 1). We can achieve co-detection of the spatial structure of bacteria and host protein expression. This allows us to understand the functions of specific bacteria neighborhoods and their interactions with the host tumor and immune system. We hope that these interactions will elucidate novel microbial markers to infer anti-cancer immunogenic outcomes.

**Acknowledgements**

We thank Matt Newgren for tireless technical support on the MIBI instrument, Rachel Finck, Xiao-Jun Ma and Bingqing Zhang for helpful discussions. B.Z. is supported by the Stanford Graduate Student fellowship S.J. was supported by a Stanford Dean’s Fellowship and the Leukemia & Lymphoma Society Career Development Program. X.R.-C. was supported by a long-term EMBO fellowship. This work was supported by grants from the FDA, NIH, Parker Institute for Cancer Immunotherapy, the Bill and Melinda Gates Foundation, as well as the Rachford and Carlota A. Harris Endowed Professorship to G.P.N.

**Consent**

Written informed consent was obtained from the patient for publication of this abstract and any accompanying images. A copy of the written consent is available for review by the Editor of this journal.

**Fig. 1 (abstract P755).**
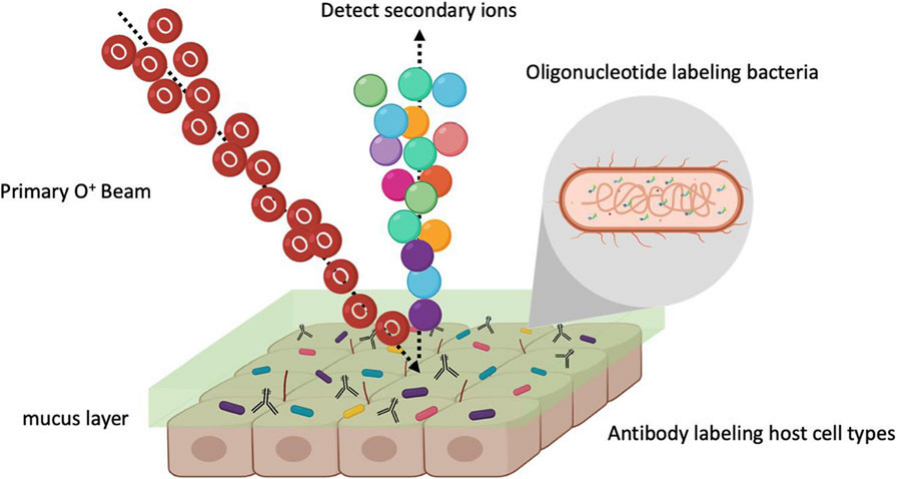
Schematic of the microMIBI

### Novel Single-Agent Immunotherapies

#### P756 HER2-targeting TLR7/8 immune-stimulating antibody conjugates elicit robust myeloid activation and anti-tumor immune responses in a TLR- and FcR- dependent manner

##### Joseph Gonzalez^1^, Cecelia Pearson^1^, Shelley Ackerman, PhD^1^, Justin Kenkel^1^, Po Ho^1^, Angela Luo^1^, Murray Nguyen^1^, Jason Paik, MD PhD^2^, Arthur Lee^1^, Richard Laura^1^, Hai Li^1^, Cindy Kreder^1^, Karla Henning^1^, Steve Chapin^1^, Bruce Devens^1^, Brian Safina^1^, David Jackson^1^, Edgar Engleman^2^, David Dornan, PhD^1^, Michael Alonso^1^

###### ^1^Bolt Biotherapeutics, Redwood City, CA, United States;^2^Stanford University, Palo Alto, CA, United States

####### **Correspondence:** Michael Alonso (malonso@boltbio.com)

**Background**

Antigen presenting cells (APCs) process tumor antigens and facilitate recruitment of tumor-specific T cells to generate a robust anti-tumor immune response. APCs are present in almost all tumor types, including those that are refractory to checkpoint inhibitors; however, the highly immunosuppressive tumor microenvironment (TME) often renders APCs unable to effectively process and present tumor antigens. To overcome this immunosuppressive TME and rescue the function of tumor-infiltrating APCs, we developed an immune-stimulating antibody conjugate (ISAC) through covalent attachment of a TLR7/8 agonist to a HER2-targeting monoclonal antibody.

**Methods**

For human in vitro assessment of ISACs, primary PBMCs or myeloid APCs were isolated from human healthy donor blood and activation was measured by flow cytometry, cytokine-bead array, and ELISA-based methods. Various ISACs were assessed in vivo using human HER2 expressing tumor xenograft models (HCC1954, JIMT1, and COLO205) as well as rHER2-expressing syngeneic tumor models (MMC and CT26-rHER2).

**Results**

In vitro co-cultures with human primary leukocytes and HER2-positive cancer cell lines revealed that the anti-HER2 ISAC potently activates APCs, leading to increased co-stimulatory molecule expression and secretion of pro-inflammatory cytokines. In myeloid-focused in vivo models utilizing HER2-positive human tumor cell line xenografts, which are resistant to trastuzumab, anti-HER2 ISAC treatment led to tumor regression and clearance. Importantly, antigen density and receptor occupancy were key factors dictating response to ISAC treatment. Further mechanistic studies revealed that anti-HER2 ISAC efficacy was TLR- and FcR- dependent. Cell-specific depletion studies also demonstrated a dependence on phagocytic cells for tumor regression and clearance. In syngeneic tumor models in which anti-HER2 ISAC treatment led to tumor clearance, animals rechallenged with the parental tumor cell line lacking HER2 antigen expression were protected from tumor growth. In addition, depletion of CD4 and CD8 T cells prior to rechallenge led to loss of immunological memory.

**Conclusions**

These data provide a strong rationale for pursuing an anti-HER2 TLR7/8 ISAC into the clinic for the potential treatment of HER2-positive cancers.

#### P757 Novel SIRP antibodies with differentiated characteristics for targeting innate immunity in cancer

##### Gabriela Andrejeva, PhD, Benjamin Capoccia, PhD, Michael Donio, MS, Ronald Hiebsch, BS, Robyn Puro, PhD, Myriam Bouchlaka, PhD, Daniel Pereira, PhD

###### Arch Oncology, St. Louis, MO, United States

####### **Correspondence:** Daniel Pereira (dpereira@archoncology.com)

**Background**

Unleashing the adaptive immune response from checkpoint blockade has shown therapeutic efficacy in oncology, although in a subset of patients. The ability of innate immune cells to acquire tumor-associated antigen and effectively present it to T cells is fundamental to a successful immune attack on cancer. Macrophage-mediated tumor cell phagocytosis can enact significant tumor clearance. In addition, macrophages represent the most abundant immune cell type in many solid tumors and are often linked to poor prognosis due to the ability of cancer cells to block phagocytosis and manipulate macrophages into facilitating malignant progression. The interaction of tumor CD47 with SIRPα on macrophages, dendritic cells and neutrophils presents such an immune blockade. We have previously presented data on AO-176, a clinical stage humanized anti-CD47 antibody that is highly differentiated among agents in this class of checkpoint inhibitors. Here we also evaluate a portfolio of novel anti-SIRP antibodies with differentiated properties as another approach to targeting the CD47/SIRPα axis.

**Methods**

We used solid-phase ELISA and cell binding assays to characterize target binding and blocking by our antibodies. Immunomodulatory effects were tested using in vitro phagocytosis, T cell stimulation by allogeneic dendritic cells and antigen-specific T cell assays.

**Results**

Here we present novel discovery-stage antibodies that either bind human SIRPα variant 1 or both variant 1 and variant 2, the two most common variants in the human population. Interestingly, our antibodies may or may not block the SIRPα-CD47 interaction, however, they are unique and differentiated among reported anti-SIRP antibodies in that they induce phagocytosis of several solid and hematologic tumor cell lines as single agents. They also exhibit enhanced phagocytosis when combined with tumor-opsonizing antibodies, including our highly differentiated anti-CD47 antibody, AO-176. Finally, while our antibodies also bind to SIRPγ, but differ in ability to block the interaction to CD47 in solid phase assays, they do not block SIRPγ in cell-based assays involving T cells. Consequently, they show no adverse effects on T cell proliferation or activation in allogeneic co-cultures of T cells with dendritic cells, and in a CMV antigen recall response assay.

**Conclusions**

Our novel anti-SIRP antibodies induce promising and differentiated in vitro single agent and combination phagocytosis and show no adverse effects on T cell functionality. These data support their future development, both as single agent antibodies and in combination with other anti-cancer drugs.

**Ethics Approval**

All human material was purchased from Astarte Biologics, INC., 22122 – 20th Ave SE, H-150, Bothell, WA 98021.

#### P758 COBRA: A novel conditionally active bispecific antibody that regresses established solid tumors in mice

##### Anand Panchal, Andisheh Bagheri, MS, Maia Vinogradova, Tim Chen, Russell Wall, Stéphanie Levon, Pui Seto, Jessica Krakow, Brian Hillier, Ying Zhu, Danielle Dettling, Jeremiah Degenhardt, Aakash Datt, Eilene Kwok, Lucy Quach, Patricia Culp, Chad May, Bob DuBridge

###### Maverick Therapeutics, Brisbane, CA, United States

####### **Correspondence:** Bob DuBridge (bdubridge@mavericktx.com)

**Background**

Despite clinical success with bispecific antibodies (bsAbs) targeting hematological malignancies (e.g., blinatumomab, a CD19xCD3 bsAb), efficacy in solid tumor indications remains a significant challenge. Because T-cell redirecting bsAbs are so potent, even low levels of cell surface target antigen expression on normal tissues may quickly become a safety liability and severely restrict the dose levels that can be achieved in patients. Additionally, identifying “clean” targets that are uniquely expressed on the tumor and not on normal tissues is very difficult at best, further narrowing the therapeutic landscape for T-cell redirected bispecifics.

To overcome these challenges, we have developed a novel recombinant bsAb platform called COBRA (Conditional Bispecific Redirected Activation). COBRAs are engineered to enable targeting of more widely expressed and validated tumor cell surface antigens by focusing T-cell engagement within the tumor microenvironment.

**Methods**

COBRA molecules are designed to bind to target antigen, which may be expressed on both tumor and normal cells, yet not engage T-cells unless exposed to a proteolytic microenvironment, which is common in tumors but not in normal healthy tissues. Once bound to the tumor target antigen, protease-dependent linker cleavage allows COBRAs to convert an inactive anti-CD3 scFv to an active anti-CD3 scFv binding domain. Upon conversion, COBRAs are then able to simultaneously co-engage T-cells and target antigen, resulting in a potent cytolytic T-cell response against the tumor cells. In addition, COBRAs are designed with a half-life extension moiety that is removed from the active molecule upon proteolytic cleavage. This allows for a sustained presence in the circulation of the inactive COBRA prior to tumor target binding, and more rapid clearance of unbound active COBRA molecules, thereby decreasing the potential for cytotoxic activity in normal tissues (Figure 1).

**Results**

Here we report the novel design of the COBRA molecule MVC-101 and demonstrate its ability to engage CD3 and Epidermal Growth Factor Receptor (EGFR) to elicit potent cytotoxic activity in vitro and in vivo (Figure 2, 3).

**Conclusions**

In T-cell dependent cellular cytotoxicity (TDCC) assays, we demonstrate that protease dependent activation increases the potency of MVC-101, yielding a therapeutic with sub-picomolar potency. We further demonstrate that administration of MVC-101 in mice with established xenografts results in protease cleavage dependent, T-cell mediated tumor regressions in multiple tumor models (Figure 4). Finally, MVC-101 has an extended half-life in vivo upon administration, and rapid clearance post proteolytic activation, resulting in an improved safety profile over conventional T-cell redirected bispecifics.

**Fig. 1 (abstract P758).**
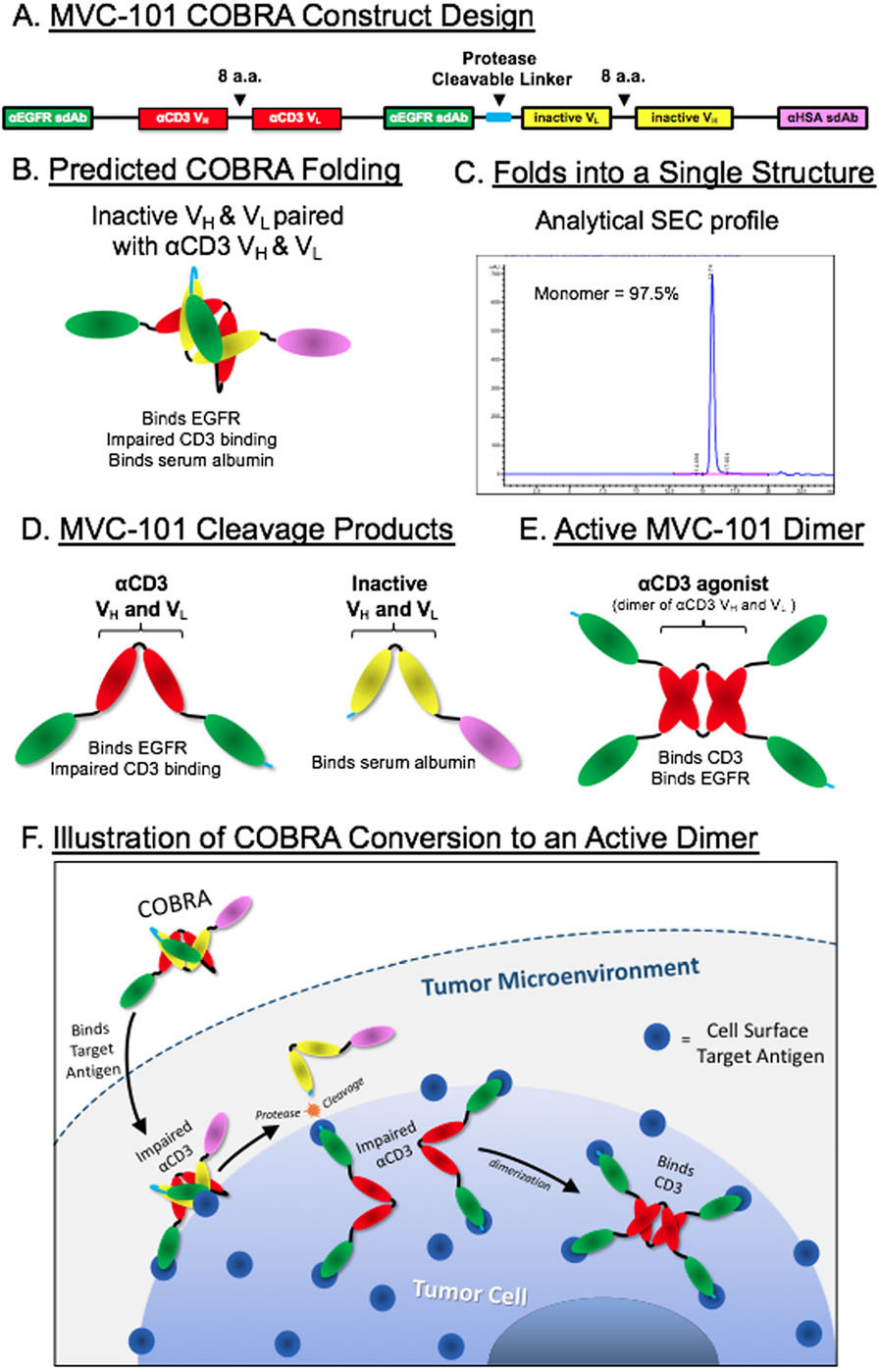
COBRA design and mechanism of action

**Fig. 2 (abstract P758).**
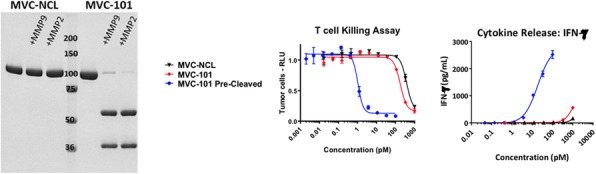
In vitro activity

**Fig. 3 (abstract P758).**
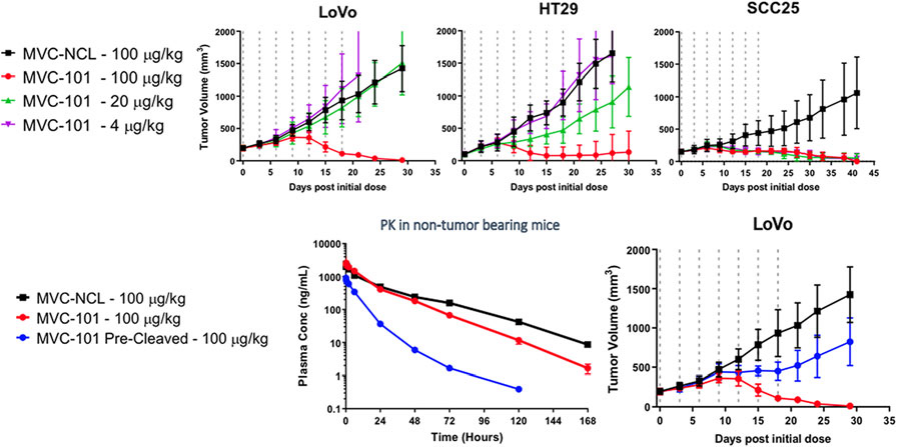
Tumor regressions in mice and PK

**Table 1 (abstract P758).**
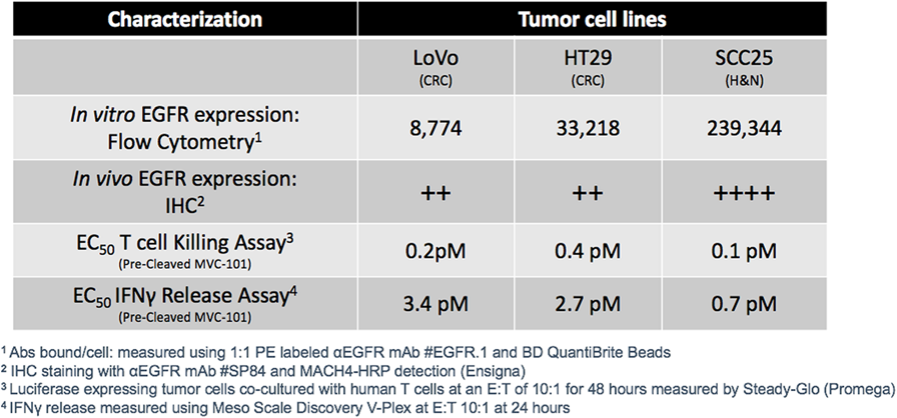
EGFR expression and potency

#### P759 DZD2269, a novel A2AR antagonist capable of overcoming high adenosine induced immunosuppression

##### Yu Bai, MSc , Xin Zhang, Jie Zheng, Yingchun Wang, Lingli Zhang, Zhenfan Yang

###### Dizal Pharmaceutical Co., Ltd, Shanghai, China

####### **Correspondence:** Yu Bai (Yu.Bai@dizalpharma.com), Zhenfan Yang (Pamela.Yang@dizalpharma.com)

**Background**

Extracellular adenosine is a potent immunosuppressive metabolite. While its level is normally low in circulating blood, it could reach over 1,000-fold higher in tumor microenvironment, at 50 to 100 μM [1-3]. Adenosine exerts its biological effect through adenosine receptors. Available evidence suggests that A2aR is the key mediator for its immune suppressive effect [4,5]. Several A2aR antagonists are in early clinical development. While potent at low adenosine level, they loss their activity totally when measured at the adenosine level found in tumor microenvironment. DZD2269 was designed to fully block A2aR in a wide range of adenosine concentrations. It has the right DMPK properties consistent with once daily oral dosing. Results from GLP toxicity studies support its clinical evaluation in healthy volunteers and cancer patients.

**Methods**

The binding assay was measured by testing the ability of DZD2269 to replace radio-labelled ligand for each adenosine receptors. The potency of A2aR antagonists and selectivity towards related receptors was determined by measuring cAMP levels or calcium influx change in adenosine receptor overexpressed CHO cells, following NECA or ADO stimulation. Reversed adenosine induced Immune suppression assay were quantified following compound treatment in the presence and absence of NECA in CD3/CD28 stimulated PBMC. NECA-stimulated mouse whole blood was utilized for measuring the p-CREB level. In addition, in vivo anti-tumor activity of DZD2269 alone or in combination with anti-CTLA4, radiotherapy and chemotherapy was evaluated in several syngeneic models.

**Results**

DZD2269 binds to human A2aR with a Ki of 5.4nM. Adenosine or its stable analogue NECA potently inhibited TNF-α, IFN-γ and IL-2 secretion from human T cells in a dose-dependent manner. DZD2269 effectively overcame 10uM NECA (equal to 100uM adenosine) induced immunosuppression and restored TNF-α, IFN-γ and IL-2 secretion. Adenosine inhibited CD8+ cell proliferation which could be reversed by DZD2269. The antitumor activities of DZD2269 was evaluated in several syngeneic mouse tumor models. DZD2269 monotherapy reduced tumor growth in these models. The most profound anti-tumor growth effects were found when DZD2269 was administered in combination with anti-CTLA4 Ab, radiotherapy and chemotherapy. Our results showed that p-CREB level could be a useful pharmacodynamic biomarker. It negatively correlated with DZD2269 exposure in animal models. Inhibition of p-CREB to basal level was required for achieving maximum efficacy.

**Conclusions**

DZD2269 is a potent, selective A2aR antagonist, which effectively reverses high adenosine induced immunosuppression. Its safety profile supports its clinical evaluation in healthy volunteers and cancer patients.

**References**

1. Vaupel P, Mayer A. Hypoxia-Driven Adenosine Accumulation: A Crucial Microenvironmental Factor Promoting Tumor Progression. Adv Exp Med Biol. 2016;876:177-183

2. Blay J, White TD, Hoskin DW. The extracellular fluid of solid carcinomas contains immunosuppressive concentrations of adenosine. Cancer Res 1997, 57(13):2602–2605

3. Spychala J. Tumor-promoting functions of adenosine. Pharmacol Ther. 2000 Aug-Sep;87(2-3):161-73.

4. Ohta A, Gorelik E, Prasad SJ, Ronchese F, Lukashev D, Wong MK, Huang X, Caldwell S, Liu K, Smith P, Chen JF, Jackson EK, Apasov S, Abrams S, Sitkovsky M. A2A adenosine receptor protects tumors from antitumor T cells. Proc Natl Acad Sci U S A, 2006 Aug 29; 103(35):13132-7.

5. Ohta A, Madasu M, Kini R, Subramanian M, Goel N, Sitkovsky M. A2A Adenosine Receptor May Allow Expansion of T Cells Lacking Effector Functions in Extracellular Adenosine-Rich Microenvironments. J Immunol. 2009 Nov 1;183(9):5487-93.

**Ethics Approval**

All in vivo studies were approved by Institutional Animal Care and Use Committee of Dizal

#### P760 Local modulation of T cell PD-1 using self-delivering RNAi as a potential immunotherapeutic

##### John Barrett, PhD , James Cardia, Melissa Maxwell, Mani Kadiyala, MS, Dingxue Yan, PhD, Winnie Tam, Simon Fricker, PhD, Gerrit Dispersyn, PhD

###### Phio Pharmaceuticals, Malborough, MA, United States

####### **Correspondence:** John Barrett (jbarrett@phiopharma.com)

**Background**

The ability of tumor cells to evade the immune system (immune escape) as well as their acquired resistance to anti-cancer drugs constitute important barriers to the successful management of cancer. Interaction between the Programmed Death Ligand 1 (PD-L1) on the surface of tumor cells with the Programmed Death-1 (PD-1) receptor on cytotoxic T lymphocytes leads to inactivation of these immune effectors and, consequently, immune escape. Restoration of anti-tumor immunity by blocking PD-1 signaling using antibodies has proven to be beneficial in cancer therapy. However, systemic anti-PD-1 therapy has been shown to induce Grade 3-4 colitis, pneumonitis and increased LFTs. The ability to elicit local inhibition of PD-1 should circumvent the systemic toxicity of current anti-PD-1 therapy.

Ph-762 is a chemically modified self-delivering RNA inhibitor containing an asymmetric duplex structure (≤ 15 base pair duplex) and a single-strand phosphorothioate tail specifically targeting PD-1 mRNA expression. Ph-762 is efficiently delivered to the immune cells without the need for specialized formulations or mechanical transfection as is observed with current RNAi’s.

**Methods**

PD-1 mRNA knockdown was assessed by qRT-PCR on total cellular RNA using Taqman probe sets. T cell phenotyping was performed using flow cytometry. T cell IFN-γ release was assessed via ELISA.

**Results**

Ph-762 rapidly and efficiently transfected T cells. Ph-762 elicited a concentration dependent silencing of primary human PD-1 mRNA levels, IC50 of ~ 0.4 μM, with silencing persisting for up to 7 days posttreatment while media alone and nontargeting control (NTC) did not affect PD-1 mRNA levels. This in turn resulted in a concentration dependent reduction in PD-1 surface protein expression (IC50 of ~ 0.4 μM). Ph-762 transfection did not alter T cell viability when compared to media control. Ph-762 CD3+CD8+ T cells were able to recognize allogenic tumors as demonstrated by increased IFN-γ production when co-cultured with melanoma cells. The increased CD3+CD8+ functionality resulted in enhanced tumor cell kill when compared to media alone or NTC. Similar effects were observed with mPh-762 (murine surrogate) in mouse T cells.

**Conclusions**

These findings support the hypothesis that local PD-1 mRNA silencing by Ph-762 is a viable approach for overcoming tumor-induced immunosuppression resulting in enhanced T cell functionality and tumor cell kill which warrants further investigation in patients.

#### P761 Tumor-targeted bacterial nanocells carrying a super-cytotoxic drug elicit an anti-tumor immune response, long-term survival, prolonged tumor remission, and resistance to tumor re-challenge

##### Himanshu Brahmbhatt, PhD

###### EnGeneIC Pty Ltd, Lane Cove West, Australia

####### **Correspondence:** Himanshu Brahmbhatt (hbrahmbhatt@engeneic.com)

**Background**

Despite efficacy of current immunotherapy in specific cancers, most tumors still develop resistance by exploiting different immune evasion pathways. Bacterial nanocells (designated as EDVs; EnGeneIC Dream Vector) can be loaded with a variety of chemotherapeutic drugs and targeted with antibodies directed to the cancer cell surface, to release a toxic payload intracellularly [1,2]. Here we present evidence that targeted EDVs packaged with the highly toxic drug PNU159682 (D682), resulted in an anti-tumor immune response and resistance to tumor re-challenge.

**Methods**

Syngeneic mouse tumor models, CT26Ep12.1 (colon) and 4T1 (breast) expressing EpCAM receptor were intravenously treated with saline, 1x10^9 EpCAM targeted EDVs (Ep-EDV), or 1x10^9 EpCAM targeted nanocells loaded with PNU-159682 (Ep-EDV-D682). Tumors were measured three times per week and tumors and spleen were isolated for immune cell analysis by flow cytometry. Separately, mice received 4 doses of saline, 2x10^9 Ep-EDV, or 2x10^9 Ep-EDV-D682 and tumor growth was monitored until tumors reached 1000 mm3 (endpoint). Mice were re-challenged on day 15 with a tumor inoculation on the other flank as was a Naïve cohort. Spleens were collected from control mice upon sacrifice and from surviving Ep-EDV-682 treated mice 50 days post initial tumor inoculation for flow cytometric analysis of memory T-cell populations.

**Results**

Immune cells isolated from CT26Ep12.1 and 4T1 mice treated with Ep-EDV-682 showed a significant increase in M1/M2 macrophages, activation of NK cells concurrently with a predominantly Th1 cytokine response. Dendritic cell maturation followed, generating anti-tumor CD8+ T-cells. Mice exhibited a significant increase in long term survival, with 40% CT26Ep12.1 and 35% 4T1 showing complete regression and no relapse respectively. On rechallenge with tumor cells, 0% of CT26EP12.1 and 25% 4T1 mice formed tumors compared to 100% of saline controls and a naïve cohort. T- cell populations in the spleens of surviving Ep-EDV-682 treated CT26Ep12.1 mice exhibited significant increases in total T-cell numbers (CD3+) including both CD4+ and CD8+ and both tumor models showed an increase in memory T-cell populations.

**Conclusions**

Targeted nanocells carrying a super-cytotoxic payload are capable of a dual attack on the tumor in syngeneic mouse models of colon and breast cancer. Firstly, tumor cell killing occurs via a very effective drug, followed by development of an anti-tumor immune response, resulting in long-term survival and resistance to re-challenge. The current study highlights the potential of targeted nanocells as a novel and versatile cancer cyto- immuno-therapeutic.

**Acknowledgements**

J. MacDiarmid and H. Brahmbhatt contributed equally to these studies

**References**

1. MacDiarmid JA, Mugridge NB, Weiss JC, Phillips L, Burn AL, Paulin RP, Haasdyk JE, Dickson KA, Brahmbhatt VN, Pattison ST, James AC, Bakri GA, Straw RC, Stillman B, Graham RM, Brahmbhatt H. Bacterially-derived 400nm particles for encapsulation and cancer cell targeting of chemotherapeutics. Cancer Cell 2007; 11:431-445.

2. MacDiarmid JA, Amaro-Mugridge NB, Madrid-Weiss J, Sedliarou I, Wetzel S, Kochar K, Brahmbhatt VN, Phillips L, Pattison ST, Petti C, Stillman B, Graham RM, Brahmbhatt H. Sequential treatment of drug-resistant tumors with targeted minicells containing siRNA or a cytotoxic drug. Nature Biotechnol. 2009; 27:643-651.

**Ethics Approval**

Mouse experiments were approved by the EnGeneIC Animal Ethics committee, under the following approval numbers, AEC 1/2016, AEC 14/2016, AEC 15/2016, AEC 11/2017, and AEC 05/2019.

#### P762 Antibodies to ILT3 (LILRB4) abrogate myeloid immunosuppression and combine with PD1 blockade to enable T cell activation and function

##### Philip Brandish, PhD , Jeanne Baker, Alan Byford, Michael Caniga, Craig Chappell, Holly Cherwinski, Daniel Cua, Laurence Fayadat-dilman, Brian Hall, Barbara Joyce-Shaikh, BS, Veronica Juan, Carl Mieczkowski, Anthony Palmieri, Yujie Qu, MD, Latika Singh, Peter Stiivers, Jie Zhang-Hoover

###### Merck, Boston, MA, United States

####### **Correspondence:** Philip Brandish (philip_brandish@merck.com)

**Background**

Accumulating clinical data suggest that infiltration of solid tumors with immunosuppressive myeloid cells is associated with a lower likelihood of response to T cell checkpoint immunotherapy. As such, modulation of the activation state and function of the myeloid compartment is potentially a critical next frontier in cancer therapy. Immunoglobulin-like transcript 3 (ILT3, also known as LILRB4) expressed on dendritic cells (DCs) has been previously implicated in the promotion of antigen-specific tolerance, and we have found increased expression of ILT3 on monocytic myeloid-derived suppressor cells (MDSCs) that are effectors of antigen-independent immune suppression.

**Methods**

To study the role of ILT3 in these aspects of immunity, we created antibodies that are highly potent and selective for ILT3 over other ILT family members.

**Results**

These antibodies effectively antagonized polarization by IL-10 to enhance dendritic cell activation state and enable T cell priming. Furthermore, in a mixed lymphocyte reaction using IL-10 polarized DCs and allogenic CD8+ T cells, anti-ILT3 combined with pembrolizumab afforded greater CD8+ T cell activation compared to either agent alone. Turning to MDSCs, we found that anti-ILT3 antibodies impaired the acquisition of a suppressive phenotype by monocytes co-cultured with cancer cells, resulting in diminished suppression of autologous CD8+ T cell activation. Finally, anti-ILT3 antibodies inhibited tumor growth and stimulated an increase in markers of immune activation in a humanized mouse model of cancer, the subject of a companion poster.

**Conclusions**

These data support the testing of anti-ILT3 antibodies in humans for the treatment of cancer.

**Ethics Approval**

All mice were maintained under specific pathogen-free conditions in accordance with Merck Research Laboratories (MRL) Institutional Animal Care and Use Committee (IACUC) regulations and were performed in accordance with guidelines in approved protocol P12217.

#### P763 Target Engagement and PK/PD quantification in humanized tumor model for anti-ILT3 mAb discovery

##### Alan Byford, MSc, Yujie Qu, MD, Xiaoyan Du, Daping Zhang, Yvonne Yannoni, Laurence Fayadat-dilman, Brian Hall, Jeanne Baker, Michael Meehl, Scott Pruitt, MD, PhD, Lily Moy, Philip Brandish, Jie Zhang-Hoover

###### Merck, Boston, MA, United States

####### **Correspondence:** Jie Zhang-Hoover (jie.zhang-hoover@merck.com)

**Background**

Target engagement (TE) and translational pharmacodynamics (PD) biomarkers are critical in drug discovery and development. We explored and compared multiple TE & PD readouts after anti-ILT3 mAb treatment in a humanized murine SK-MEL-5 tumor model: 1) ILT3 receptor occupancy on monocytic myeloid cells in the tumor and blood vs. total blood soluble ILT3 (sILT3) levels; 2) levels of activation markers HLA-DR and LOX-1 on monocytic myeloid cells that are associated with antigen presentation and activation of T cells.

**Methods**

Hu-NSG mice with subcutaneous SK-MEL-5 or Panc 08.13 tumors were treated with an anti-human ILT3 mAb. Cell surface ILT3 receptor occupancy and activation markers were quantitated by FACS analysis. Plasma anti-ILT3 mAb and total soluble ILT3 (sILT3) concentrations were measured using electrochemiluminescence immunoassay.

**Results**

TE assessed by ILT3 occupancy on the surface of CD14+ myeloid cells and by plasma total sILT3 levels was detected after single dose administration of the anti-ILT3 mAb ranging from 0.003 to 20 mg/kg. Levels of ILT3 receptor occupancy on CD14+ myeloid cells correlated with the dose administered and exposure levels of anti-ILT3 mAb in the plasma in both tumor models. ILT3 receptor occupancy in the tumor and total sILT3 in the blood were saturated in the mice treated with 10 mg/kg of anti-IL-3 mAb, while saturation of ILT3 receptor occupancy in the blood was reached at a lower dose (1 mg/kg). The percentage of tumor myeloid cell ILT3 receptor occupied correlated with total plasma sILT3 levels after mAb treatment. Expression of immune activation related markers LOX-1 and HLA-DR on CD14+ myeloid cells were upregulated in a time and exposure dependent manner in the SK-MEL-5 hu-NSG model that expresses high levels of ILT-3, but not in Panc 08.13 with low ILT3 expression.

**Conclusions**

High levels of ILT3 receptor occupancy in the tumor were required for the upregulation of immune activation markers in the humanized tumor model. HLA-DR could be used as an immune response PD biomarker for assessing anti-ILT3 mAb activity in vivo. A proof-of-concept was established for using total sILT3 in the blood as a surrogate for tumor TE after anti-ILT3 mAb treatment that could be used for human dose projection.

Activity of the anti-ILT3 mAb in vitro and tumor efficacy in vivo is described in a companion poster

**Ethics Approval**

The study was approved by Merck Institutional Animal Care and Use Committee, approval number 2022-200518-FEB.

#### P764 Directly targeting FOXP3 with AZD8701, a first-in-class high affinity antisense oligonucleotide to relieve immunosuppression in cancer

##### Charles Sinclair^1^, Alexey Revenko^2^, Alison Peter^1^, Molly Taylor^1^, Anisha Solanki, PhD^1^, Melissa Chapman^1^, James Yates^1^, Helen Angell, BSc PhD^1^, Frederick Goldberg^1^, Robert MacLeod^2^, Larissa Carnevalli, PhD^1^

###### ^1^AstraZeneca, Cambridge, United Kingdom; ^2^Ionis Pharmaceuticals, Carlsbad, CA, United States

####### **Correspondence:** Larissa Carnevalli (larissa.carnevalli@astrazeneca.com)

**Background**

Regulatory T cells (Treg) critically maintain immuno-suppression in the tumor microenvironment, representing an attractive immuno-oncology target. The Treg lineage is defined by expression of the FOXP3 transcription factor, which controls immune-suppressive functions.

**Methods**

We have discoverd a clinical candidate AZD8701, a next-generation antisense oligonucleotide inhibitor of FOXP3 (utilizing the Ionis Gen 2.5 cEt-modified ASO platform).

**Results**

AZD8701 treatment knocked down FOXP3 in primary human Tregs via free uptake (IC50 65nM), which was also associated with modulation of known FOXP3 target genes including 25-50% reduction in CTLA4, ICOS, CCR8 and GITR. Tregs treated with AZD8701 further exhibited reduced suppressive functions in in vitro suppression assays, which confirmed the functional effects of FOXP3 modulation. Finally, AZD8701 promoted dose-dependent knockdown of FOXP3 in humanized mice, including >50% FOXP3 knockdown at doses that are predicted to correspond to clinically achievable doses. To support the importance of FOXP3 in immuno-oncological settings, we characterized murine surrogate FOXP3 ASOs in the context of syngeneic tumour bearing mice. Murine FOXP3 ASOs similarly promoted >50% FOXP3 knockdown in mice and were well tolerated with no overt toxicological findings at high doses, over a maximum of 12 weeks of treatment. Murine FOXP3 ASOs significantly attenuated tumour growth in A20 and ID8-VEGF syngeneic models, which was associated with some complete tumour regressions. Moreover, we found that mouse surrogate FOXP3 ASOs promoted additive/enhanced therapeutic effects when combined with immune checkpoint blockade. Additionally, an increase in tumour infiltrated CD8+ T cells and activation biomarkers (e.g. Granzyme B) correlated with increase anti-tumour response in A20 tumour model.

**Conclusions**

In summary, AZD8701 represents a first-in-class clinical candidate to target Tregs in cancer in a highly selective manner, and may provide therapeutic benefit to patients either as a monotherapy or in combination with immune checkpoint blockade.

#### P765 First in class antibody targeting Galectin-9 promotes anti-tumor response against pancreatic and other solid cancers

##### Linxiao Chen, PhD^1^, Wei Wang, MD^1^, Akiko Koide, PhD^1^, Adrian Seifert, MD^2^, Lena Seifert^3^, Aleksandra Filipovic, MD, PhD^4^, George Miller^1^, Shohei Koide, PhD^1^

###### ^*1*^*NYU Langone, New York, NY, United States;*^*2*^*University Hospital Carl Gustav Carus, Dresden, Germany* ; ^*3*^*German Cancer Research Center, Heidelberg, Germany* ; ^*4*^*PureTech Health, London, United Kingdom*

####### **Correspondence:** Shohei Koide (Shohei.Koide@nyumc.org )

**Background**

Current immunotherapies have shown promise in treating multiple cancer types but success remains elusive for cancers like pancreatic, the majority of colorectal cancers as well as cholangiocarcinoma. Poor prognosis in these tumor types is invariably linked to immune dysfunction. Galectin-9, which is a fundamental immune modulator and global immune suppressor, was previously identified as a molecule that plays a significant role in orchestrating and maintaining a tumor-permissive immune environment. In light of this, we have studied the relevance of galectin-9 as a biomarker and have developed LYT-200, a therapeutic, fully human IgG4 antibody to target galectin-9.

**Methods**

A synthetic, human antibody library was screened via phage display using purified carbohydrate binding domains (CRDs) of human and mouse galectin-9. Blockade of galectin-9-receptor binding and signaling by antibodies was assayed in both biochemical and cell-based formats. Antibody efficacy was assessed in orthotopic pancreatic cancer and subcutaneous melanoma mouse models as single agent and in combination with anti-PD1 and chemotherapy. Patient-derived samples (FFPE tissues, organotypic tumor spheroids (PDOTs)), which recapitulate complex tumor architecture, and patient blood samples were analyzed by immunohistochemistry, flow cytometry and ELISA to measure galectin-9 expression and compare with healthy controls across tumor types. Additionally, PDOTs were treated with clinical anti-galectin-9 antibodies and the resulting immune profile was analyzed.

**Results**

Significantly higher levels of galectin-9 were detected across patient blood/tissue samples (n >100 patients) compared with healthy controls. Analyses of PDOTs showed high levels of galectin-9 on tumor, myeloid and T cells. Its high expression, for example in pancreatic adenocarcinoma, suggest that galectin-9 is a promising therapeutic target and a biomarker that correlates with disease stage. From a therapeutic perspective, LYT-200 was selected out of numerous antibody candidates as the lead clinical clone based on its high affinity (dissociation constant < 1 nM) to galectin-9 across species, high specificity, stability, and blocking galectin-9 interactions with its ligands in biochemical and cell-based assays. Treatment of tumor bearing mice with LYT-200 significantly reduced in tumor size (p

**Conclusions**

LYT-200, a first-in-class human antibody that inhibits tumor-permitting activity of Galectin-9 was developed. The data collectively reveal novel galectin-9 biology and demonstrate therapeutic efficacy of LYT-200. Blockade of galectin-9 using LYT-200 is an innovative and promising strategy for treating aggressive solid tumors.

#### P766 The distinct binding footprints of bispecific T cell receptors (TCR) and TCR-mimic antibodies underpin their altered pHLA selectivity

##### Rory Crean^2^, David Cole^1^ , Johanne Pentier^1^, Ben de Wet^1^, Angharad Lloyd^1^, Bent Jakobsen, PhD^1^, Christopher Holland^1^

###### ^1^Immunocore Ltd, Milton, Abingdon, United Kingdom; ^2^Bath University, Bath, United Kingdom

####### **Correspondence:** David Cole (david.cole@immunocore.com)

**Background**

Tumour-associated peptide-human leukocyte antigen complexes (pHLA) represent the largest pool of cell surface expressed cancer-specific epitopes, making them attractive targets for cancer therapies. Soluble bispecific molecules, that incorporate an anti-CD3 effector function, are being developed to redirect T cells against these targets using two different approaches. The first achieves pHLA recognition via affinity-enhanced versions of natural TCRs (e.g. ImmTAC® molecules), whereas the second harnesses an antibody-based format (TCR-mimic antibodies). For both classes of reagent, target specificity is vital considering the vast universe of potential pHLA molecules that can be presented on healthy cells.

**Methods**

Using structural, biochemical, functional and computational approaches, we assessed the selectivity of two novel classes of T cell redirecting pHLA-targeting bispecifics based on TCR-mimic antibodies or high-affinity TCRs. Reagents included were chosen based on their recognition of identical, or closely related, pHLA determinants of the NY-ESO-1157-165 cancer testis antigen-derived peptide, SLLMWITQC, presented by HLA-A*02:01 (A2-SLL), the MAGE-A1161-169/MAGE3-A3168-176 melanoma-associated antigen-derived peptides, EADPTGHSY (A1-EAD) and EVDPIGHLY (A1-EVD), presented by HLA-A*01:01, and the WT126-134 Wilms tumour antigen derived peptide, RMFPNAPYL, presented by HLA-A*02:01 (A2-RMF).

**Results**

We demonstrate that affinity-enhanced TCRs engage pHLA using a comparatively broad and balanced energetic footprint, with interactions distributed over several HLA and peptide side-chains. As ImmTAC molecules, these TCRs also retained pHLA-selectivity, with potent killing of antigen-positive cell lines and minimal off-target activity in cellular assays. Conversely, TCR-mimic antibodies tended to exhibit binding modes focussed more towards hotspots on the HLA surface and tolerate amino acid degeneracy. In cellular assays, the antibody-based bispecifics exhibited greater cross-reactivity to antigen-negative cell lines and as such, a reduced specificity window.

**Conclusions**

These findings extend our understanding of the basic principles that underpin pHLA-selectivity and exemplify a number of molecular approaches that can be used to probe the specificity of pHLA-targeting molecules, aiding the development of future reagents.

#### P767 Single-cell cytometry with the genesis system - A scalable solution for high-parameter epitope detection

##### Jon Bell, BS, MS^1^, Yad Deol, PhD, MS^1^, Shaun Connor, BS, MBA^1^, Tyler Burns, PhD^2^

###### ^1^Celsee, Inc., Hudson, OH, United States; ^2^Celsee, Berlin, Germany

####### **Correspondence:** Jon Bell (jon.bell@celsee.com)

**Background**

This poster illustrates an exciting new way to characterize cell populations far beyond traditional methods. Using Single-Cell Cytometry removes common detection limitations of both flow and mass cytometry. Here we describe Celsee’s ability to detect 60 different antibodies in a single run.

**Methods**

25,000 cells were isolated using Celsingle 250K Slide on the Genesis System and sample preparation protocol was performed. PBMCs were stained with 60 oligo-labeled antibodies (BioLegend, CA, USA) and sequencing was performed on the MiSeq (Illumina, CA, USA). Analysis method via Seurat (Satija Lab).

**Results**

We observed signal from all 60 antibodies across the population of thousands of cells analyzed. Plotting all cells together via dimensional reduction provides a global view of similar and different sub-populations present in our PBMC sample (Figure 1). Importantly, separation of populations such as T, B, and NK cells (Figure 2) were observed. The T cell populations showed both CD8 and CD4 are mutually exclusive as well as CD45RO and CD45RA (Figure 3). For the B cell population, we observed signal across multiple CD markers as expected (e.g. CD 19). As for NK Cells, expression of CD16, CD45RA, and CD8 are in concordance with our observations. Aggregating data across major cell types shows expression across all 60 antibodies tested (Figure 4).

**Conclusions**

These data highlight the simplicity and feasibility of multiplexing 60 antibodies in a single run with the Genesis System. Whether one wishes to analyze 10 markers or over 100, this application is highly scalable both in cell number and number of markers analyzed. Given the obvious difficulties in legacy flow cytometry and mass cytometry instruments, Single-Cell Cytometry is poised to deliver limitless protein detection with Celsingle technology making the Genesis System a practical and economical solution for cytometry related experimentation.

**Consent**

Yad Deol, Shaun Connor, Jonathan Bell, Tyler Burns

**Fig. 1 (abstract P767).**
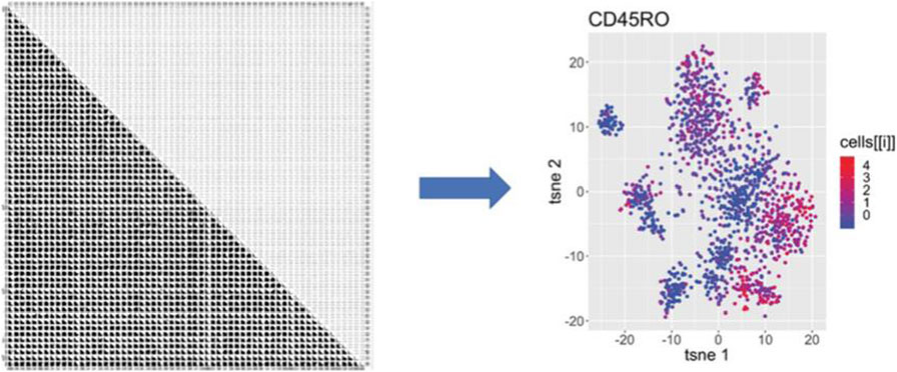
Single Cell Cytometry with the Genesis System

**Fig. 2 (abstract P767).**
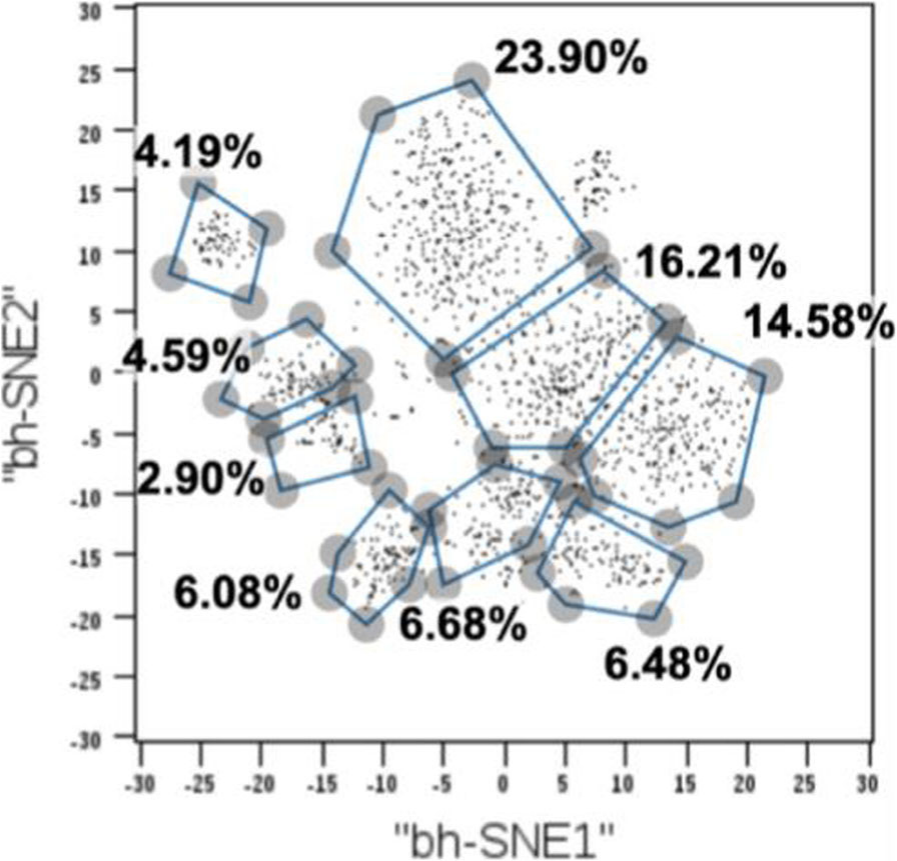
Single Cell Cytometry with the Genesis System

**Fig. 3 (abstract P767).**
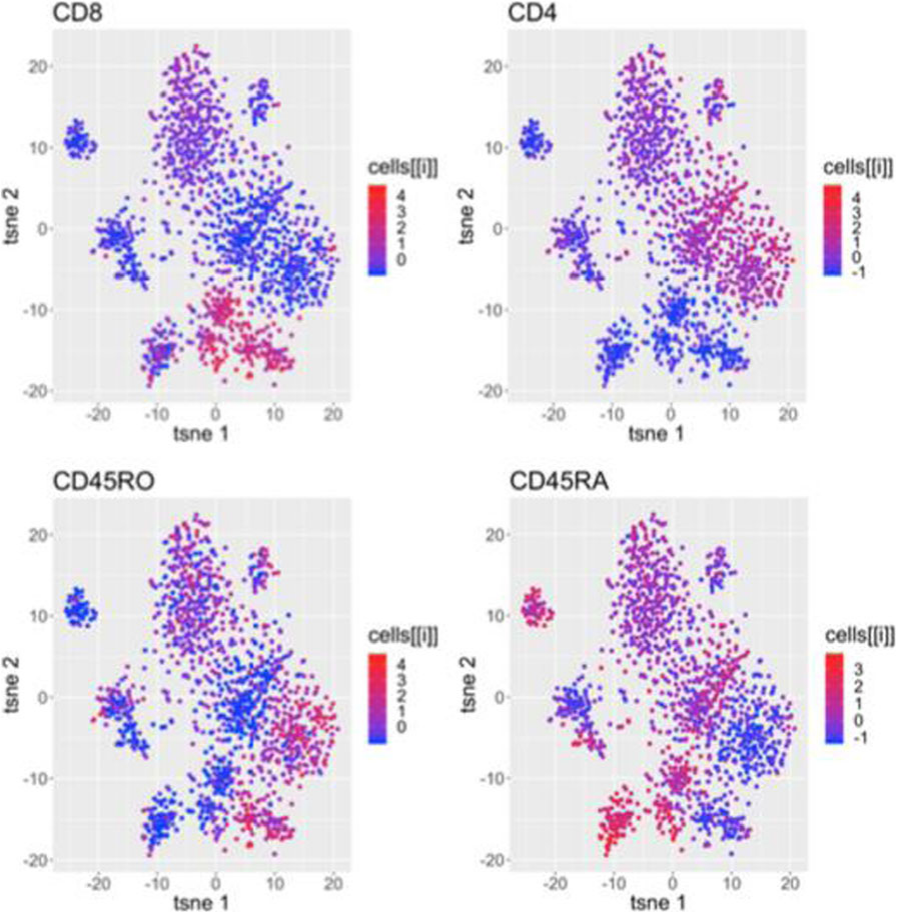
Single Cell Cytometry with the Genesis System

**Fig. 4 (abstract P767).**
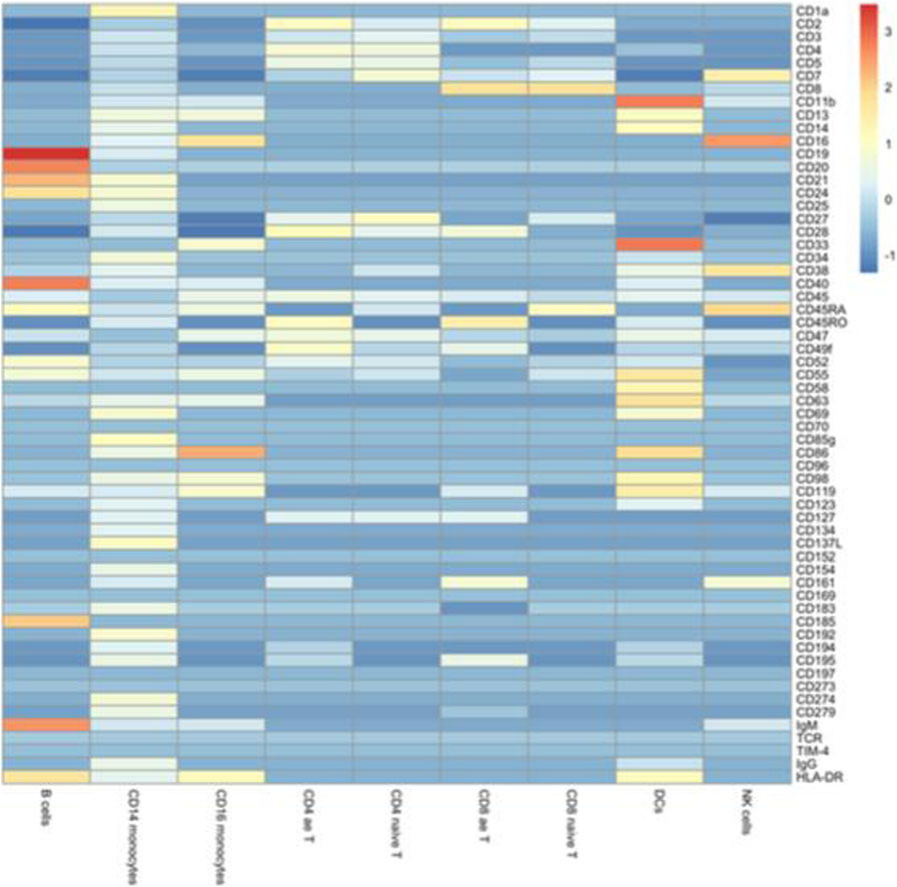
Single Cell Cytometry with the Genesis System

#### P768 Antagonism of signaling through the vasoactive intestinal peptide receptor as a novel immunotherapy in preclinical models of melanoma

##### Sruthi Ravindranathan, PhD^1^, Shuhua Wang, MD^1^, Rebecca Pankove, MS^1^, David Francis^2^, Susan Thomas, PhD, BS^2^, Rohan Dhamsania, BS^1^ , Brian Pollack^1^, Edmund Waller, MD, PhD, FACP^1^

###### ^1^Emory University, Atlanta, GA, United States; ^2^Georgia Institute of Technology, Atlanta, GA, United States

####### **Correspondence:** Rohan Dhamsania (rohan.dhamsania@emory.edu)

**Background**

Only 40% of the patients with metastatic melanoma respond to FDA-approved immune-checkpoint blockade therapies. 25%-40% of these patients relapse within 3 years of treatment [1]. Thus, it is imperative to explore more rigorous cancer immunotherapies.

Vasoactive Intestinal Peptide (VIP) is a 28-aminoacid immunosuppressive neuropeptide. Its potent immunosuppressive effects are shown in mouse models of leukemia and lymphoma, with downregulation of PD-1 and PD-L1 expression in activated T cells and dendritic cells respectively, following treatment with peptide antagonist of VIP (ANT-00) that competitively blocks binding of native VIP to VIP receptors [2]. ANT-00 treatment also significantly enhanced proliferation of splenic T cells in-vitro2. Interestingly, according to the cancer genome atlas (TCGA), metastatic melanoma patients with low VIP mRNA have better survival than patients with high VIP mRNA expression in tumor (Figure 1A). Thus, we hypothesize that pharmacological inhibition of VIP-signaling pathway would be efficacious in melanoma immunotherapy.

**Methods**

Murine melanoma cell lines: B16F10, B16F1, SM1 and D4M were cultured in DMEM-based media for 24 hours and cell-free supernatant was quantified for VIP concentration via VIP-specific competitive Enzyme Immunoassay (EIA). Presence of VIP receptors on B16F10 cells was confirmed using western blot. Cytotoxic effect of ANT-00 on B16F10 cells was analyzed using MTT assay. C57BL/6 mice inoculated with B16F10 cells were treated with ANT-00 before/after tumors were palpable. Treatment regimen involved daily subcutaneous administration of 0.1-10ug of ANT-00 or ANT-08 (a more potent VIP antagonist) throughout the duration of treatment. Phenotypic differences in immune infiltrates were analyzed via flow cytometry.

**Results**

All melanoma cell lines secrete low levels of VIP (Figure 1B). Although B16F10 cells express similar VIP receptors levels (Figure 2A), ANT-08 didn’t have direct cytotoxic effect on B16F10 cells (Figure 2B, 2C). ANT-08 dosed at 1-10ug/mouse/day-controlled tumor growth for up to 10 days of treatment, with the anti-tumor effect diminishing with subsequent treatment beyond 10 days (Figure 3E-G). Moreover, when B16F10 bearing mice were treated daily with 1.0ug of ANT-08 for 10 days after tumors were palpable, a significant improvement in survival was observed compared to PBS-treated control animals (p<0.01, Figure 4). Immunophenotyping of infiltrating lymphocytes in excised tumors revealed that ANT-08-treated group had significantly higher levels on proliferating CD4+ T cells (p=0.04) (Figure 4), supporting a role of VIP antagonism in activating anti-cancer immunity.

**Conclusions**

In preclinical melanoma models, pharmacological antagonism of VIP-signaling significantly increased survival rates (Figure 4) by activating adaptive T cell immunity. Thus, inhibiting VIP-signaling is a novel potential immunotherapy for melanoma.

**Acknowledgements**

I would like to thank Dr. Edmund Waller, Dr. Sruthi Ravindranathan and entire Waller for their constant support. I would like to acknowledge Laney Graduate School and Winship Cancer Institute of Emory University for providing me the platform to pursue cancer immunotherapy research.

**References**

1. Wherry EJ. T cell exhaustion. Nature Immunol. 2011, 12:492 -9

2. Li JM, Darlak KA, Southerland L, Hossain MS, Jaye DL, Josephson CD, Rosenthal H, Waller EK. VIPhyb, an antagonist of vasoactive intestinal peptide receptor, enhances cellular antiviral immunity in murine cytomegalovirus infected mice. PloS one. 2013;8(5)e6: 3381.

**Fig. 1 (abstract P768).**
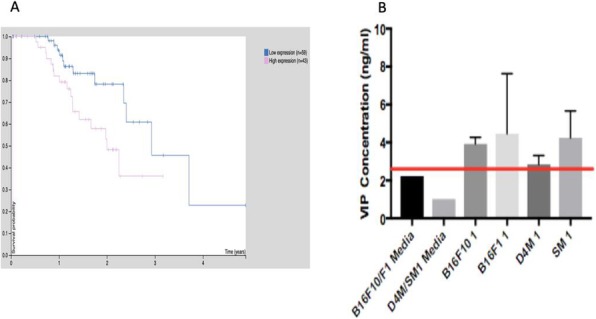
Low VIP levels in all murine melanoma cell supernatants.

**Fig. 2 (abstract P768).**
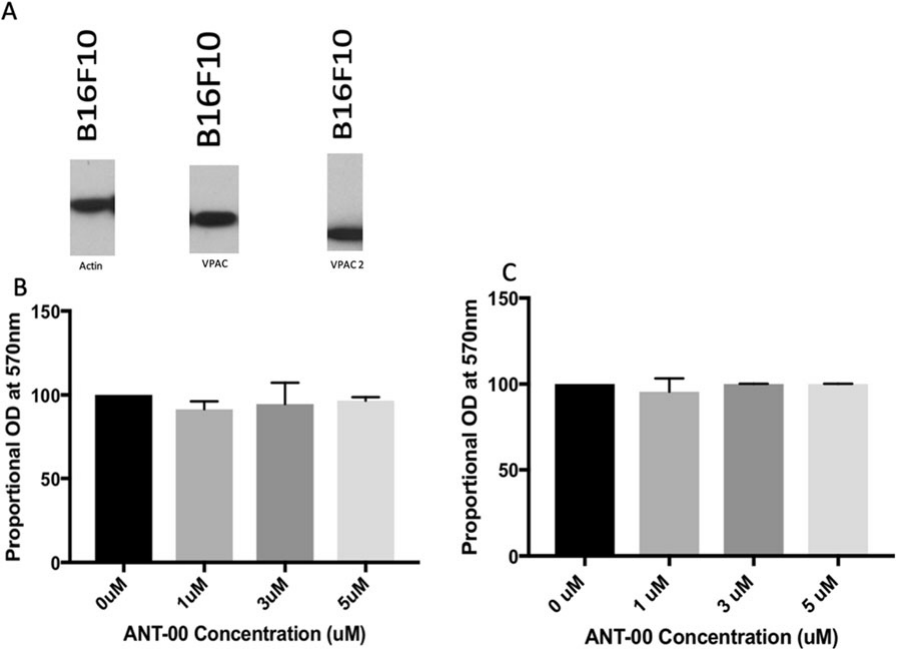
ANT-00 doesn’t have direct cytotoxic effects on B16F10 cells.

**Fig. 3 (abstract P768).**
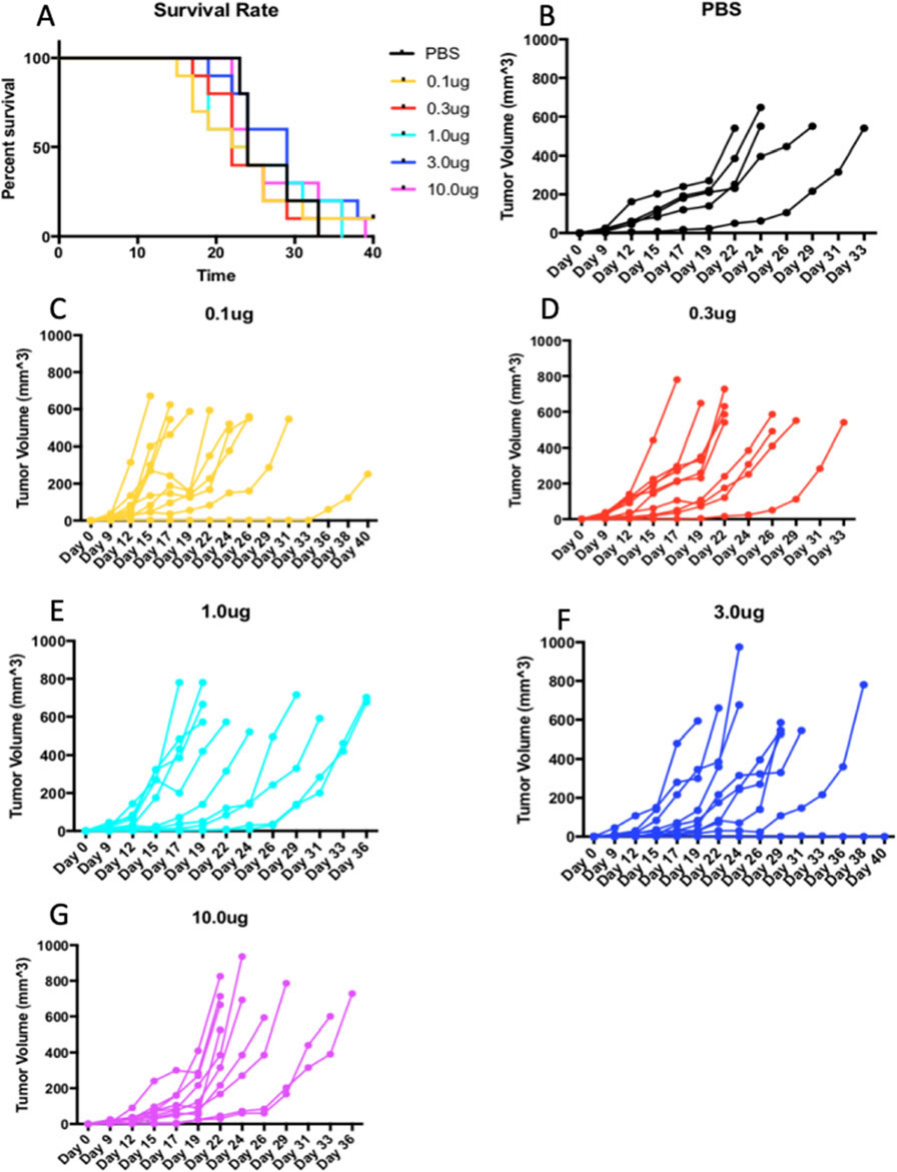
ANT-08 controlled tumor growth for 10 days of treatment

**Fig. 4 (abstract P768).**
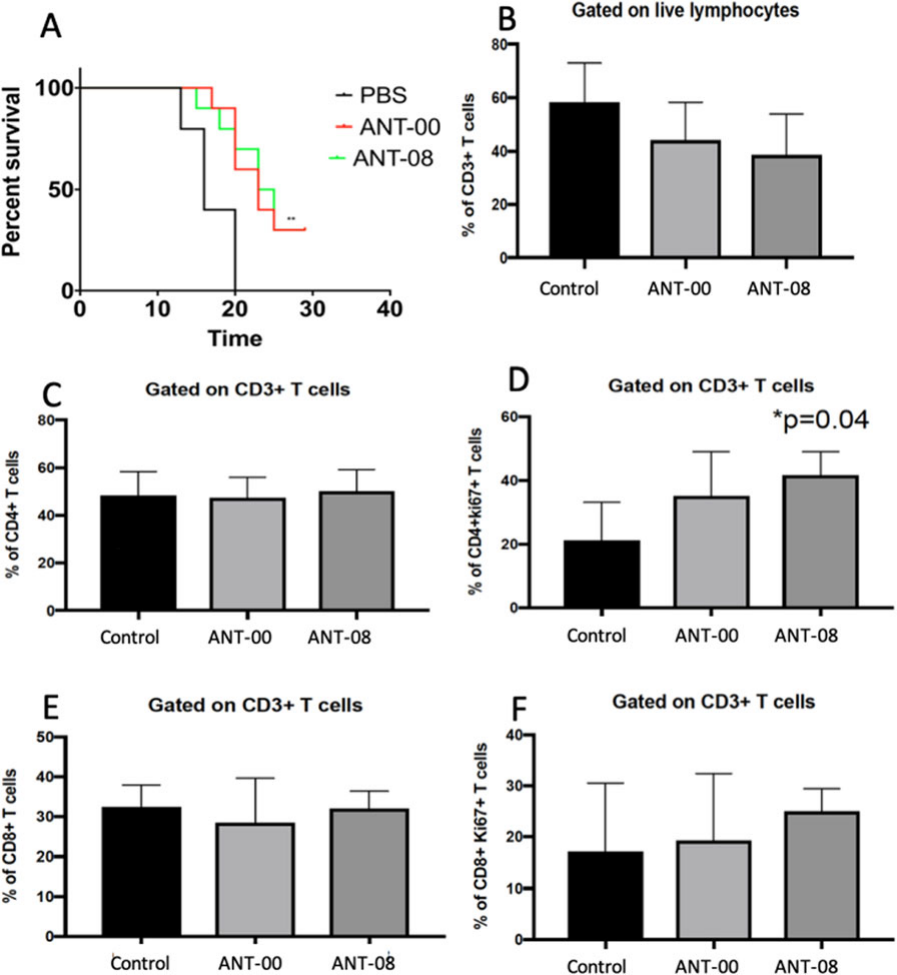
VIP antagonists significantly improved survival in mice

#### P769 Inhibition of arginase in combination with anti-PDL1 leads to increased infiltration and activation of CD8+ T cells, NK cells, and CD103+ dendritic cells in the mouse syngeneic MC38-OVA tumor model

##### Aatman Doshi, MS, Susan Cantin , Michael Secinaro, Yanjun Wang, Laura Prickett, Sharon Tentarelli, Scott Mlynarski, Eric Gangl, M. Raymond Finlay, Wenlin Shao, PhD, Alwin Schuller

###### Astrazeneca, Waltham, MA, United States

####### **Correspondence:** Susan Cantin (susan.cantin@astrazeneca.com)

**Background**

The tumor microenvironment (TME) has multiple mechanisms of immunosuppression, one being recruitment of arginase (ARG) expressing myeloid cells. ARG is an enzyme that catalyzes hydrolysis of L-arginine into urea and L-ornithine. L-arginine is a semi-essential amino acid required for optimal function of T cells and natural killer (NK) cells. Arginine depletion in the TME inhibits T cell and NK cell function resulting in immunosuppression [1]. Therefore, inhibition of ARG can reverse immunosuppression and optimize anti-tumor immunity.

**Methods**

Human CD8+ T cell and NK cell proliferation was evaluated in various concentrations of L-arginine by measuring CellTrace Violet (CTV) dilution in response to stimulation (anti-CD3/anti-CD28 stimulation for CD8’s; IL2/IL15 for NK’s.) Syngeneic mouse tumor models (MC38-OVA, LLC) were assessed for anti-tumor activity and immune system activation with an ARG inhibitor as a monotherapy and in combination with an anti-PDL1 checkpoint inhibitor. The ARG inhibitor was administered orally BID at 30 mg/kg, and anti-PDL1 was dosed with intraperitoneal injections 2QWx2 at 10 mg/kg. Four, ten and fourteen days post-treatment, phenotype and activation status of T cell, NK cell and CD103+ dendritic cell (DC) populations within tumor and lymph nodes (LN’s) were evaluated using multi-color flow cytometry. Furthermore, the functional response of CD8+ cytotoxic T cells was evaluated by ex vivo restimulation with phorbol 12-myristate-13-acetate (PMA) and ionomycin prior to analysis.

**Results**

Human CD8+ T cells and NK cells showed decreased proliferation with decreased L-arginine concentration. L-arginine threshold for optimal proliferation was determined to be ~30 uM for CD8+ T cell and ~ 9 uM for NK cells. Furthermore, CD8+ T cells showed dose dependent decreases in cytotoxic molecules (e.g. granzyme A, granzyme B, perforin) with decreased L-arginine. While ARG inhibitor monotherapy showed 30% tumor growth inhibition (TGI) in the MC38-OVA tumor model in vivo, combining with anti-PDL1 significantly improved efficacy (87% TGI), with 7/10 tumors regressing. An in vivo PD combinaton study of ARG inhibitor with anti-PDL1 showed increases in multiple tumor immune cell populations (~4-fold CD8+ T cells, 2-fold NK cells, 2-fold CD103+ DCs) and increased CD8 T cell activation. Moreover, ARG inhibitor resulted in an increase of IFNg and TNFa producing CD8+ T cells in the periphery (draining LN).

**Conclusions**

Our pre-clinical data demonstrates that an ARG inhibitor in combination with a checkpoint inhibitor can reverse tumor immunosuppression leading to strong immune activation and anti-tumor responses, suggesting arginase inhibitors could provide an opportunity to increase activity of checkpoint inhibitors clinically.

**References**

1. Oberlies J, Watzl C, Giese T, Luckner C, Kropf P, Muller I, Ho AD, Munder M. Regulation of NK Cell Function by Human Granulocyte Arginase. J Immunol 2009; 182:5259-5267

**Ethics Approval**

The study was approved by AstraZeneca's Institutional Animal Care and Use Committee (IACUC)

#### P770 A novel class of multi-specific antibodies targeting NKp30 on innate immune cells

##### Monia Draghi, PhD^1^ , Allison Nelson^1^, Amanda Oliphant^1^, Alan Leung^1^, Jay Vekeria^1^, Zach Frye^1^, Claire Chottin^2^, Sara Haserlat^1^, Rachel Rennard^1^, Caitlin Goshert^1^, Rachel McCrory^1^, Lucy Liu^1^, Kenneth Rogers^2^, Francois Villinger^2^, Piotr Bobrowicz^1^, Michael Schmidt^1^, Jennifer Watkins-Yoon^1^

###### ^1^Compass Therapeutics, Cambridge, MA, United States; ^*2*^*New Iberia Research Center, New Iberia, LA, United States*

####### **Correspondence:** Monia Draghi (monia.draghi@compasstherapeutics.com)

**Background**

Cancer immunotherapies have demonstrated remarkable clinical benefits and durable responses even for late-stage cancers. Most of the attempts of current therapies in development are focused on harnessing the adaptive immune system by unleashing antitumor T cell responses. However, there is emerging evidence that cancers develop multiple strategies to escape T cell recognition, hence approaches that do not require T cell recognition should be explored. Tumors can be effectively eradicated by natural killer (NK) cells and other innate-like lymphocytes such as gamma delta T cells that can elicit potent anti-tumor response in both mouse cancer models and patients. Therefore exploiting therapies that enhance NK and gamma delta T cells response for the treatment of cancer represents a promising and complementary approach to current existing immunotherapies.

**Methods**

Using our common light chain discovery platform we have developed a novel class of multi-specific antibodies that bind to one or two antigens at the surface of tumor cells and engage both CD16 and NKp30 activating receptors on NK and gamma deltaT cells.

**Results**

Unlike conventional monoclonal antibodies that require high levels of target antigen for efficacy, our TAAxNKp30 engagers can potently activate NK and gamma delta T cells to kill tumor cells that express high, medium and low levels of tumor antigen. Furthermore, our NKp30 engagers can activate NK cell subsets with low or null expression of CD16A without inducing off-target effect and NK-versus-NK toxicity. Compass NKp30xBCMA engagers show efficacy in vivo and when tested in cynomolgus monkeys, displays pharmacokinetics similar to monoclonal antibodies and a favorable toxicity profile as compared to BCMAxCD3 bispecific T cell engagers currently tested in clinical settings. Furthermore, transcriptomic profiling of NK cells co-cultured with BCMA positive tumor cells in the presence of NKp30xBCMA reveals a differentiated immune signature as compared to a BCMA-IgG1 monoclonal antibody.

**Conclusions**

Compass NKp30 engagers are highly differentiated from monoclonal antibodies for their capability to activate different subsets of NK and gamma delta T cells and induce killing of tumor cells expressing low levels of tumor associated antigen. Our multi-specifics antibodies show in vivo efficacy while maintaining a favorable toxicity profile as compared to T cell engagers. They are highly manufacturable, with expression, purity, stability and pharmacokinetics identical to monoclonal antibodies. Taken together, our results provide the rationale for developing multifunctional NKp30 engagers for the treatment of hematological and solid tumors.

#### P771 An anti-carcinoma monoclonal antibody (mAb) NEO-201 can also target and eliminate human immunosuppressive regulatory T cells (Tregs)

##### Kwong Tsang, PhD^1^ , Massimo Fantini, PhD^2^, Justin David^2^, M Maria Morelli, MD^3^, Christina Annunzata, MD^3^, Philip Arlen, MD^2^

###### ^*1*^*Precision Biologics/NIH, Bethesda, MD, United States;*^*2*^*Precision Biologics, Inc, Bethesda, MD, United States* ; ^*3*^*National Cancer Institute, Bethesda, MD, United States*

####### **Correspondence:** Kwong Tsang (ktsang13@gmail.com)

**Background**

NEO-201 is an IgG1 mAb reactive against many different carcinomas, but not reactive against most normal tissues. Functional analysis revealed that NEO-201 is capable of engaging innate immune effector mechanism (ADCC, CDC and enhancing NK activity) to kill tumor cells. Previous studies showed that NEO-201 attenuates growth of human tumor xenografts in mice, and demonstrates safety/tolerability in non-human primates with a transient decrease in neutrophils being the only adverse effect observed. A clinical trial evaluating NEO-201 in adults with chemo–resistant solid tumors is ongoing at the NIH Clinical Center. Preclinical evaluation showed that NEO-201 reacts against human regulatory T cells (Tregs) and here we further investigated the phenotypic and functional effects of NEO-201 on human Tregs in vitro.

**Methods**

PBMCs were collected from 5 normal donors and used for phenotypic and functional analysis. EasySep StemCell Treg isolation kits anti-biotin kits (biotin-labeled NEO-201 mAb) were used to isolate Tregs from PBMCs. Phenotypic analysis was conducted by flow cytometry for markers: CD4, CD25, CD127, FoxP3, CD15s, CD45RA, CCR4, NEO-201 antigen, CEACAM5 and CEACAM6. The ability of NEO-201-isolated Tregs to suppress autologous CD4+ T responder cell proliferation was assessed using a co-culture suppression assay and the ability of NEO-201 to mediate killing of opsonized Tregs was evaluated using a CDC assay.

**Results**

The % of NEO-201+ cells in the population of CD4+CD25highCD127negFoxP3+CD15s+CCR4+Tregs ranged from 60%-80%. NEO-201+Tregs were CD45RA negative. Isolated CD4+NEO-201+ Tregs were capable of suppressing CD4+ T responder cell proliferation, and NEO-201 mAb mediated CDC activity against Tregs.

**Conclusions**

NEO-201 reacts against human Tregs and can be used as a novel marker to identify and to purify Tregs. Tregs isolated using NEO-201 mAb were functionally suppressive and could be eliminated by CDC. This study demonstrates for the first time that this tumor-targeting mAb may also mediate through a novel mechanism down regulating Treg-mediated immunosuppression of anticancer immunity.

#### P772 Antagonism of TNFR2: Focus on novel antibodies with preference for tumor microenvironment Tregs and oncogenes expressed on the tumor

##### Michael Khodadoust, MD PhD^1^, Youn Kim, MD^1^, Michael Yang^2^, Heather Torrey, PhD^2^, Lisa Tran^2^, Denise Faustman, MD, PhD^2^

###### ^1^Stanford University School of Medicine, Palo Alto, CA, United States; ^2^Massachusetts General Hospital/Harvard Medical School, Charlestown, MA, United States

####### **Correspondence:** Denise Faustman (faustman@helix.mgh.harvard.edu)

**Background**

In diverse human and murine cancers, tumor necrosis factor receptor 2 (TNFR2) is heavily expressed in the tumor microenvironment on regulatory T cells (Tregs) and myeloid-derived suppressor cells. Clinical data consistently show TNFR2 overexpression in the tumor microenvironment on Tregs after checkpoint failures. TNFR2 also serves as an oncogene for direct tumor expansion. One major challenge to creating antagonistic antibodies to the TNF superfamily receptors has been that natural ligands such as TNF have a very high affinity and strongly promote agonism. Using sequential receptor peptide fragment mapping, we have made TNFR2 antagonistic antibodies in which a select region of this receptor creates antagonism resistant to TNF. Here we share new data on cell-specific and disparate TNFR2 expression in the tumor microenvironment and show that these novel antibodies preferentially target the tumor microenvironment, not peripheral Tregs.

**Methods**

We analyzed TNFR2 expression in humans using mean fluorescent intensity (MFI) of TNFR2 on Tregs, T effector (Teff) cells and primary tumor cells from subjects with lymphoma vs controls and analyzed over 1000 human cell lines from different primary tumor sites. We studied the effect of dominant TNFR2 antagonists on secreted soluble TNFR2 (sTNFR2), a marker of extreme agonism, by culturing human T cells with TNFR2 antagonistic antibodies. We also screened dominant TNFR2 antagonists for their ability to kill cancer cells, kill tumor cell lines, eliminate tumor-associated Tregs and induce Teff proliferation in subjects with advanced Stage IV cutaneous T cell lymphoma (CTCL, or Sézary syndrome) and cell lines of diverse tumor origins.

**Results**

The TNFR2 antagonists killed tumor-residing Tregs and cancer cells expressing the oncogene in vitro. This new antagonist class does not require antibody-dependent cellular cytotoxicity (ADCC). Framework class switching experiments show no need for Fc region function, and the construction of F(ab)2 fragments also still shows efficacy. Dominant TNFR2 antagonists rapidly stopped sTNFR2 secretion, a known serum biomarker of poor cancer prognosis.

**Conclusions**

Dominant TNFR2 antagonists kill both tumor-residing Tregs and cancer cells expressing the oncogene in human specimens but not normal circulating cells. TNFR2 antagonistic antibodies may represent a new cancer treatment approach.

**Ethics Approval**

A human studies protocol was approved by the Massachusetts General Hospital (MGH) Human Studies Committee (MGH-2001P001379) and Stanford University Human Studies Committee (IRB 5538 and IRB 13844).

#### P773 ONCR-177, an oncolytic HSV designed to safely and potently activate systemic anti-tumor immunity

##### Sonia Feau, Melissa Hayes, Brian Haines, Agnieska Denslow, Jacqueline Gursha, Daniel Wambua, Shreeya Khatiwada, Lingxin Kong, Jacob Spinale, Prajna Behera, Peter Grzesik, Jennifer Lee, Terry Farkaly, Edward Kennedy, Lorena Lerner, Christophe Queva

###### Oncorus, Cambridge, MA, United States

####### **Correspondence:** Christophe Queva (christophe.queva@oncorus.com)

**Background**

Oncolytic viruses (OVs) represent a new class of therapeutic agents for the treatment of cancers that poorly respond to immune checkpoint inhibitors. OV promote anti-tumor responses by a dual mechanism of action: the selective oncolysis of tumor cells, followed by the development of systemic anti-tumor immune responses [1].

**Methods**

ONCR-177 is a highly engineered recombinant oncolytic Herpes Simplex Virus (oHSV) designed to be a safe and efficacious therapy for the treatment of solid tumor indications. ONCR-177 expresses five transgenes selected for abscopal efficacy using an in vivo screen in syngeneic tumor models: IL-12 for NK and T cell activation, CCL4 and FLT3LG for expansion and recruitment of classical dendritic cells (cDC), and antagonists to two clinically validated immune checkpoint targets, PD-1 and CTLA-4, to counter T cell exhaustion. ONCR-177, or its mouse surrogate virus mONCR-171, was tested in a series of experiments designed to demonstrate local and systemic anti-tumor activity, and mechanisms of action.

**Results**

In in vitro co-culture studies, DCs loaded with ONCR-177 oncolysates were superior in stimulating MART1+ tumor antigen-specific T cells compared to lysates from the unarmed vector. Intra-tumoral administration of mONCR-171 resulted in durable local and distant complete tumor regressions and survival benefit across several syngeneic bilateral tumor models representing various degrees of HSV permissivity and baseline T cell infiltration, including models reported to be resistant to other oHSV therapies. Immune phenotyping by flow cytometry of injected or contralateral CT26 syngeneic tumors after a mONCR-171 intra-tumor (IT) treatment regimen demonstrated increased numbers of activated NK cells, CD8 and CD4 T cells, and cDCs. The proportion of Tregs decreased, resulting in elevated CD8/Treg ratios. The tumor Ag response against AH1 a peptide derived from envelope glycoprotein 70 (gp70) of endogenous murine leukemia virus (MuLV) and expressed by BALB/c-derived CT26 colorectal carcinomas [2] was recently assessed in order to measure specific anti-tumor response after mONCR-171 IT delivery. The proportion of AH1 specific T cells producing IFNɤ was significantly increased in mice treated with mONCR-171 compared to the base vector. The quality of the T cell response was enhanced, with an increase of polyfunctional T cells expressing both IFNɤ and TNF, demonstrating a strong potentiating effect of our payloads to the OV response.

**Conclusions**

In conclusion, ONCR-177/mONCR-171 potently activates local and systemic anti-tumor immune responses that result in durable responses, extended survival, and the elicitation of protective immunity. These encouraging preclinical data warrant the clinical investigation of ONCR-177 in patients with metastatic cancer.

**References**

1. Andtbacka RHI, Kaufman HL, Collichio F, Amatruda T, Senzer N, Chesney J, et al. Talimogene laherparepvec improves durable response rate in patients with advanced melanoma. J Clin Oncol. 2015;33(25):2780–8.

2. Huang AY, Gulden PH, Woods AS, Thomas MC, Tong CD, Wang W, et al. The immunodominant major histocompatibility complex class I-restricted antigen of a murine colon tumor derives from an endogenous retroviral gene product. Proc Natl Acad Sci. 2002;93(18):9730–5.

**Ethics Approval**

Whole blood was collected from healthy donors as described in the ethical protocol/amendment

IXP-001_V3 (Belgium; Reg. Nr. B6702014215858), protocol IXP-003_V1 (Belgium; Reg. Nr.

B707201627607) or protocol IXP-004_V1 (The Netherlands; Reg. Nr. NL57912.075.16).

#### P774 Intratumoral depletion of regulatory T cells using CD25 targeted photoimmunotherapy elicits anti-cancer immune activity and synergizes with PD1 checkpoint blockade in immunocompetent mouse models

##### Michelle Hsu, Stephanie Okamura, Daniele Bergeron, Estela Solis, Dany Gitnick, Roger Heim, Jerry Fong, PhD , Miguel Garcia-Guzman

###### Rakuten Medical, San Diego, CA, United States

####### **Correspondence:** Jerry Fong (jerry.fong@rakuten-med.com)

**Background**

Photoimmunotherapy (PIT) is an investigational cancer treatment platform that utilizes an antibody-dye (IRDye® 700DX, IR700) conjugate and illumination with a non-thermal, red-light laser for selective cell killing. These preclinical studies describe the intratumoral depletion of regulatory T cells (Tregs), a significant source of immune suppression, with an anti-CD25-IR700 conjugate using photoimmunotherapy (CD25 PIT). Anti-cancer activity and subsequent immune responses following CD25 PIT administered alone or in combination with anti-PD1 treatment were evaluated in immunocompetent mouse models.

**Methods**

Immunocompetent mice inoculated with CT26 tumors were treated with CD25 PIT alone or in combination with anti-PD1 treatment. Tumor volume was determined by caliper measurements in target (illuminated) and distal (non-illuminated) tumors. Target tumors were harvested and intratumoral immune responses were characterized by flow cytometry. To evaluate the activation of systemic immune responses, cytotoxic T lymphocyte assays were conducted with splenocytes derived from mice treated with CD25 PIT alone or in combination with anti-PD1. Tumor rechallenge studies were conducted to assess for a tumor-specific immune memory response. Anti-cancer responses to CD25 PIT combined with anti-PD1 treatment were also evaluated in CD8+ T cell-depleted mice.

**Results**

Single tumor bearing mice treated with saline, anti-PD1 antibodies, CD25 conjugate without PIT, CD25 PIT alone, or in combination with anti-PD1 antibodies, resulted in complete response (CR) rates of 0% (0/15), 8.3% (1/12), 0% (0/15), 50.0% (5/10), and 73.9% (17/23), respectively. CD25 PIT combined with anti-PD1 led to synergistic anti-cancer activity in target and distal tumors. All CR mice prevented formation of new tumors when inoculated with the same tumor type, but tumor growth occurred when inoculated with a different tumor type, suggesting a tumor specific immune memory response. Flow cytometry analysis of intratumoral immune responses revealed an increase of CD8+ T cells in the targeted tumor one week after PIT treatment, with a majority of CD8+ T cells lacking exhaustion markers (PD1-CTLA4-). CD25 PIT treatment also led to expansion of tumor antigen specific cytotoxic lymphocytes in the spleen. CD8+ T cell depletion abrogated the anti-cancer responses induced by CD25 PIT combined with anti-PD1 treatment.

**Conclusions**

Depletion of intratumoral Tregs by CD25 PIT resulted in the local activation of CD8+ T-cell mediated anti-cancer activity, and a systemic tumor specific immune memory response. CD25 PIT treatment combined with anti-PD1 therapy led to synergistic anti-cancer activity in target and distal tumors. These results support the exploratory evaluation of CD25 PIT in patients for the treatment of cancers that present with Treg immunosuppression.

**Ethics Approval**

All animal studies conducted followed the guidelines put out by the "Guide to the Care and Use of Laboratory Animals" by the National Research Council.

#### P775 Crosslink-independent CD137 agonism is associated with liver inflammation

##### Miguel Gaspar, PhD , John Pravin, Leonor Rodrigues, MSc, Alexander Koers, PhD, Mihriban Tuna, PhD, Neil Brewis, PhD

###### F-star Biotechnology ltd, Cambridge, United Kingdom

####### **Correspondence:** Miguel Gaspar (miguel.gaspar@f-star.com)

**Background**

Stimulating existing anti-cancer immune responses by activating T cell costimulatory receptors of the Tumor Necrosis Factor Receptor Superfamily (TNFRSF) is potentially the next significant stage of cancer immunotherapy. Clustering of TNFRSF members is required to initiate signal transduction and crosslinking of TNFRSF-specific antibodies via Fcg receptor (FcgR) interaction is typically required to mimic this effect. However, TNFRSF antibodies have been described which can initiate crosslink-independent agonism and signal transduction by simply binding to their receptor. CD137 is a costimulatory TNFRSF member expressed on activated T cells and interaction with CD137L results in increased T cell proliferation, survival and cytotoxic activity. Some agonist antibodies targeting CD137 have been shown to induce liver inflammation both in the clinic or in preclinical models, with the latter being associated with increased liver T cell infiltration.

**Methods**

To understand factors contributing to toxicity, different CD137 agonist antibodies were compared for their ability to activate T cells in the presence or absence of secondary crosslinking antibodies, used to mimic the effect of FcgR-mediated crosslinking in vivo. A bispecific antibody targeting OX40 and CD137 (FS120), with activity independent of FcgR-mediated crosslinking, was also used to test the ability of using binding to co-expressed receptors as an alternative mechanism of crosslinking. The anti-mouse CD137 agonist antibodies as well as an anti-mouse OX40/CD137 bispecific (FS120 surrogate) were also tested in vivo for their ability to induce liver T cell infiltration, activation and proliferation.

**Results**

The CD137 antibodies tested showed varying levels of in vitro potency in stimulating T cells and certain antibodies were able to induce crosslink-independent activation. The CD137 antibody clone present in urelumab, which induced hepatotoxicity in the clinic, showed crosslink-independent activity and was more potent as compared to the clone present in utomilumab, which was well tolerated in the clinic. An anti-mouse CD137 antibody (clone 3H3) also showed crosslink-independent activity and increased sustained liver T cell infiltration, activation and proliferation as compared to a crosslink-dependent clone (Lob12.3). FS120 surrogate showed activity in vitro which was not associated with increased liver T cell infiltration in vivo.

**Conclusions**

CD137 agonist antibodies associated with increased liver inflammation were found to be crosslink-independent, suggesting this factor may contribute to their hepatotoxicity risk. FS120 surrogate did not show increased liver T cell infiltration suggesting this has the potential to be a well-tolerated and effective mechanism of crosslinking and agonising CD137, as well as OX40, and is independent of FcgR-mediated crosslinking.

**Ethics Approval**

Murine studies were conducted under a U.K. Home Office License in accordance with the U.K. Animal (Scientific Procedures) Act 1986 and EU Directive EU 2010/63.

#### P776 Multifunctional natural killer cell engagers targeting NKp46 trigger protective tumor immunity

##### Laurent Gauthier, PhD^1^ , Ariane Morel, PhD^1^, Nadia Anceriz^1^, Benjamin Rossi^1^, Audrey Blanchard-Alvarez^1^, Gwendoline Grondin^1^, Sylvia Trichard^1^, Cédric Cesari^1^, Melody Sapet^1^, Frédéric Bosco^1^, Hélène Rispaud-Blanc^1^, Franceline Guillot^1^, Stéphanie Cornen^1^, Alain Roussel^2^, Béatrice Amigues^2^, Guillaume Habif^1^, Flavien Caraguel^1^, Sandrine Arrufat^1^, Romain Remark, PhD^1^, François Romagné^3^, Yannis Morel, PhD^1^, Eric Vivier^1^

###### ^*1*^*Innate Pharma, Marseille, France;*^*2*^*AFMB, Marseille, France* ; ^*3*^*MImAbs, Marseille, France*

####### **Correspondence:** Laurent Gauthier (laurent.gauthier@innate-pharma.fr)

**Background**

Over the last decade, various new therapies have been developed to promote anti-tumor immunity. Despite interesting clinical results in hematological malignancies, the development of bispecific killer cell engager antibody formats directed against tumor cells and stimulating anti-tumor T-cell immunity has proved challenging, mostly due to toxicity problems.

Interest has recently focused on the use of NK cells for therapeutic interventions, as these cells have anti-tumor properties. NK cells express several activating receptors that can be targeted to induce NK cell-mediated anti-tumor immunity, such as CD16, NKG2D, and the natural cytotoxicity receptors NKp30, NKp44 and NKp46. The full activation of NK cells has been shown to require the co-engagement of different cell surface receptors.

**Methods**

We report here the design, production and characterization of a new generation of trifunctional NK cell engagers (NKCEs) consisting of mAb fragments targeting the activating NK cell receptor NKp46 together with a Tumor Antigen (TA) and an Fc fragment, to promote antibody-dependent cell-mediated cytotoxicity (ADCC) via the activating receptor CD16 expressed on NK cells. The approach used was based on innovative IgG-Fc multispecific antibody formats and first-in-class agonist anti-NKp46 mAbs activating NK cells only when cross-linked by tumor cells, with no off-target effects.

**Results**

Trifunctional NKCEs were more potent in vitro than clinical therapeutic antibodies targeting the same tumor antigen. They had similar in vivo pharmacokinetics to full IgG antibodies, no off-target effects and efficiently controlled tumor growth in mouse models of solid and invasive tumors [1].

**Conclusions**

Trifunctional NKCEs targeting CD19, CD20 or EGFR as tumor antigens triggered tumor killing by human primary NK cells in vitro. In vivo, they induced NK cell accumulation in tumors and promoted tumor clearance in preclinical mouse models of solid and invasive cancers. Our multispecific technology provides a versatile platform with many different format options and the potential to co-engage up to three activating receptors on NK cells and two different tumor antigens on cancer cells. The trifunctional NKCEs reported here should favor NK cell targeting to the tumor microenvironment, in which NKp46 expression levels remain high in many tumor conditions, by contrast to CD16, NKG2D, NKp30 and NKp44. The co-targeting of NKp46 and CD16 led to full NK cell activation. Together with the stronger anti-tumor activity of these molecules in preclinical models than of gold standard mAbs, such rituximab, obinituzumab and cetuximab, these results support the clinical development of NKCEs for cancer immunotherapy, as a complement to existing immuno-oncology approaches.

**References**

1. Gauthier L, Morel A, Anceriz N, Rossi B, Multifunctional natural killer cell engagers targeting NKp46 trigger protective tumor immunity. Cell. 2019 ; 177:1701-1713.

**Fig. 1 (abstract P776).**
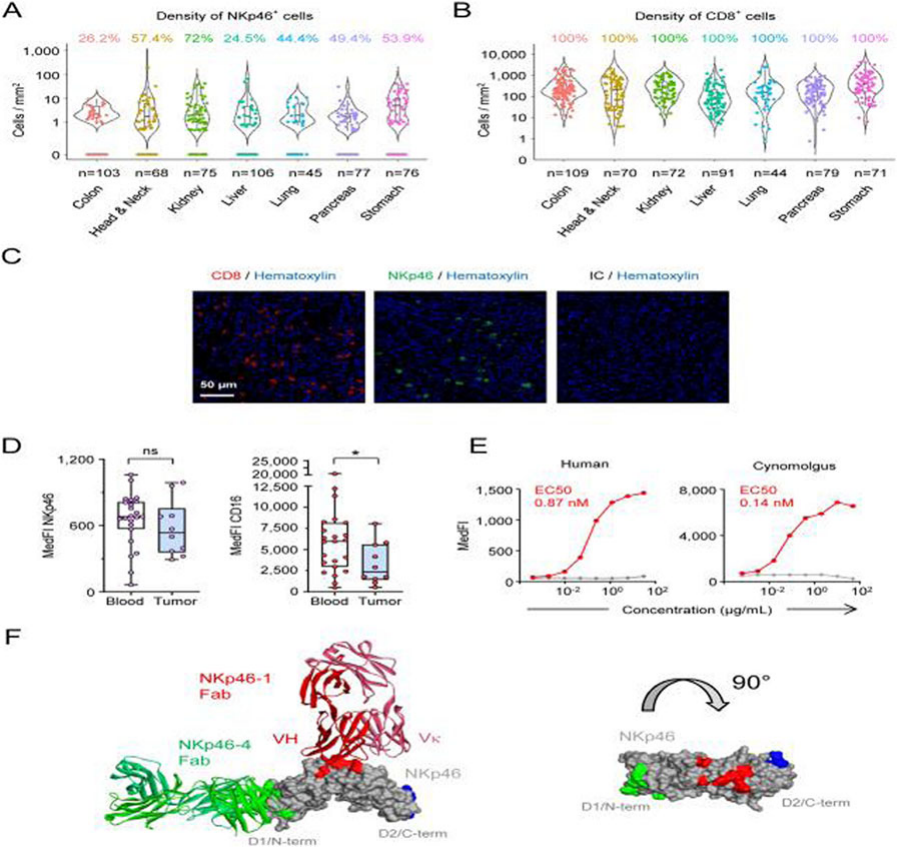
NKp46 is expressed in the tumor bed

**Fig. 2 (abstract P776).**
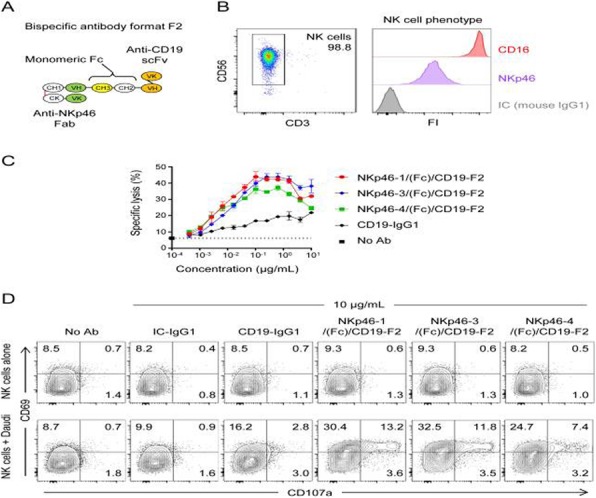
In vitro activity of anti-NKp46 antibodies targeting differed

**Fig. 3 (abstract P776).**
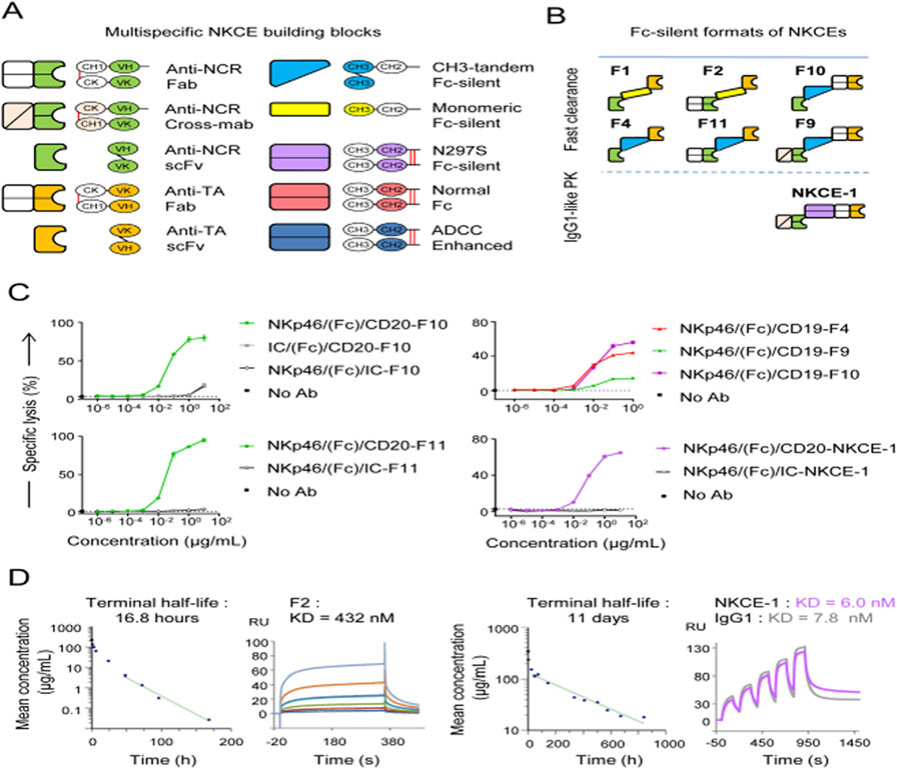
Development and comparison of silent-Fc NKCE mAb formats in

**Fig. 4 (abstract P776).**
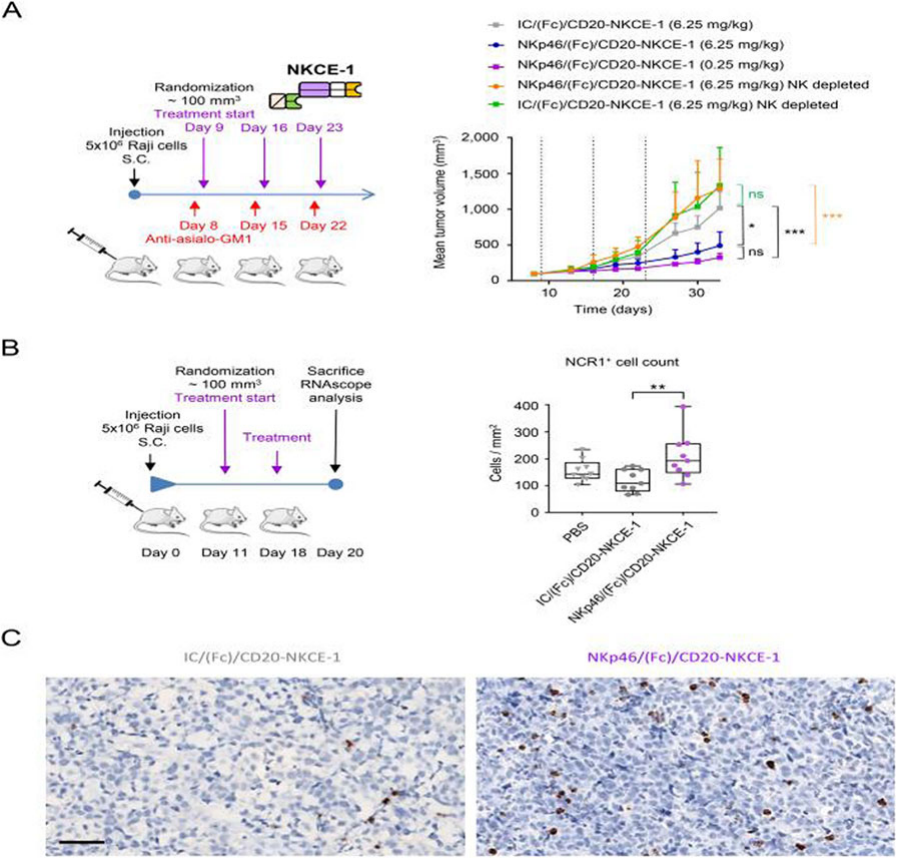
Bispecific NKCEs promote tumor growth control in vivo

**Fig. 5 (abstract P776).**
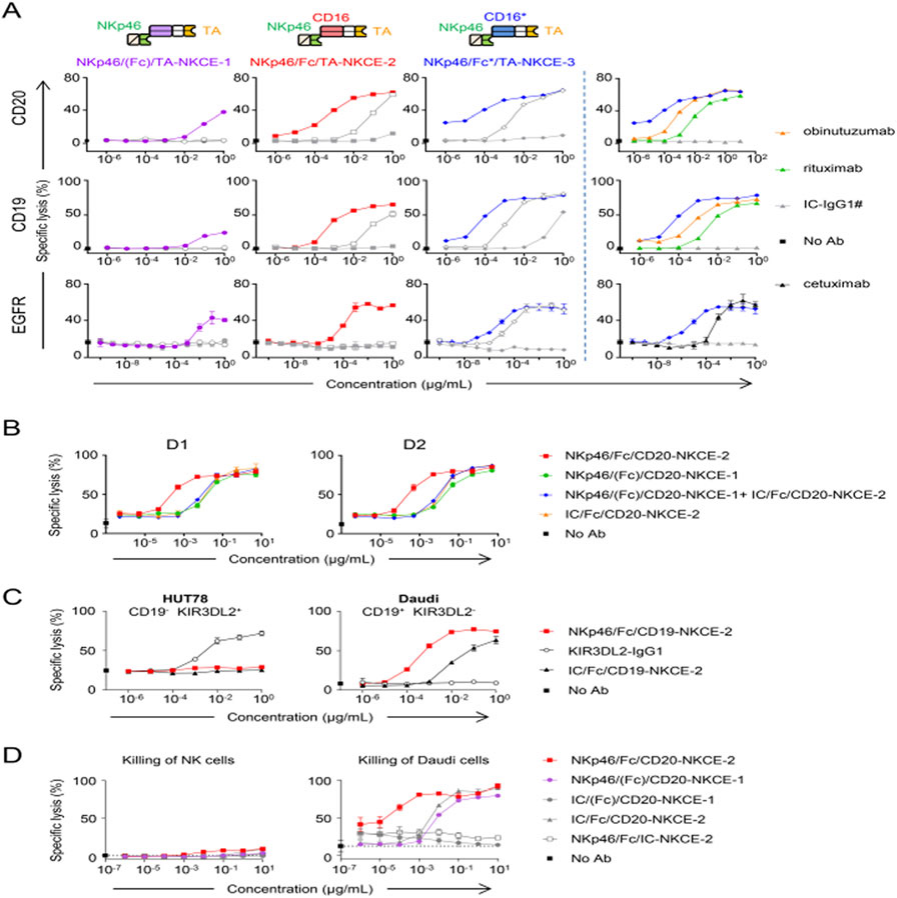
Trifunctional NKCEs promoting ADCC are more efficient than b

**Fig. 6 (abstract P776).**
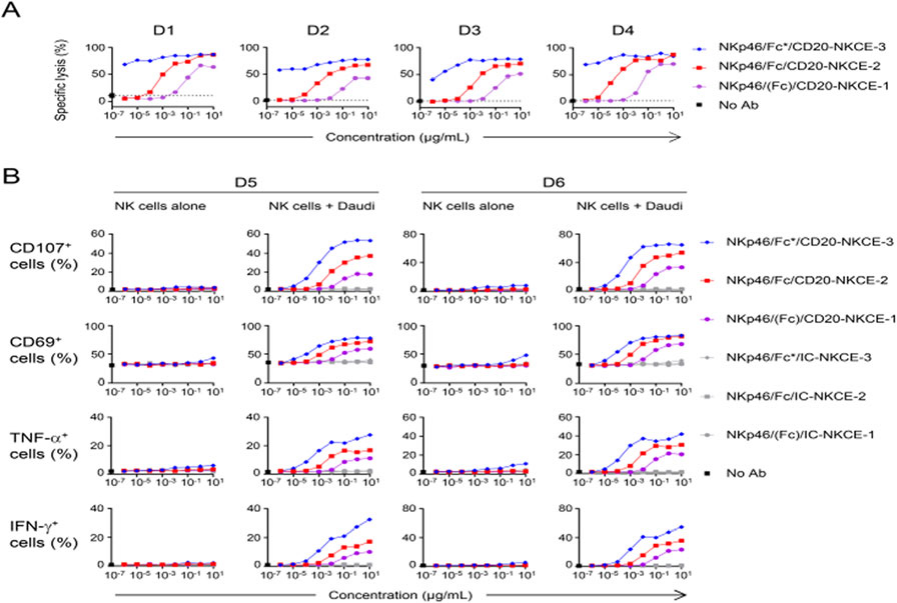
Trifunctional NKCEs promoting NK cell activation are more ef

**Fig. 7 (abstract P776).**
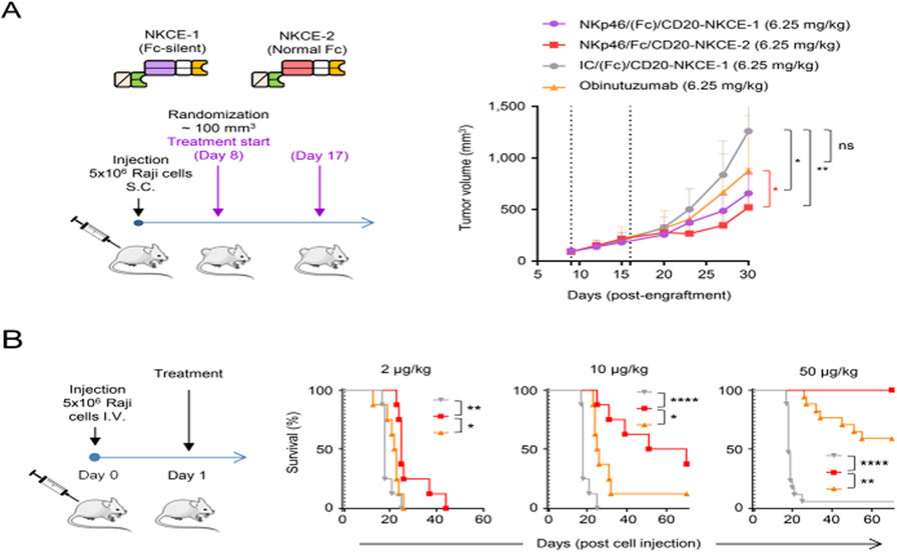
Trifunctional NKCEs promoting ADCC are more efficient than b

#### P777 Engineered monomeric JAG1 and multimeric DLL1 Notch ligand constructs enhance antitumor immunity by reducing exhaustion of T-effector memory cells

##### Uttam Laksmi Mounika Goruganthu, M Tech^1^, Anil Shanker, PhD^2^ , David Carbone, MD, PhD^1^ , Elena Tchekneva, MD^1^, Anneliese Antonucci^1^, Michael Koenig^1^, Longzhu Piao^1^, Anwari Akhter, PhD^1^, Parvathi Ranganathan^1^, Nicholas Long^1^, Thomas Magliery^1^, Jason Evans^1^, Rajeswara Arasada^1^, Pierre Massion, MD^2^, Mikhail Dikov, PhD^1^ , Roman Uzhachenko, PhD MD^2^, Portia Thomas^2^, Asel Biktasova, MD, PhD^2^, Duafalia Dudimah^2^, Maria Teresa De Aquino, PhD^2^

###### ^1^The Ohio State University, Columbus, OH, United States; ^2^Meharry Medical College and Vanderbilt University, Nashville, TN, United States

####### **Correspondence:** Anil Shanker (ashanker@mmc.edu), David Carbone (david.carbone@osumc.edu), Mikhail Dikov (mikhail.dikov@osumc.edu)

**Background**

Notch receptor-ligand interactions are an important regulation governing T-cell-mediated immune responses and are highly contextual. Dendritic cells (DC) expressing JAG1 on their cell surface can drive naïve T-cells to a regulatory phenotype (Treg), which are immunosuppressive and dampen antitumor immune responses, whereas DC-expressed DLL1/4 can support Th1 or cytotoxic T-cell responses, facilitating tumor regression. Activation of Notch receptors on T-cells requires polyvalent interactions with ligand-bearing DC, whereas soluble monovalent ligands act as competitive inhibitors and suppress Notch signaling. We exploited this mechanistic detail of Notch receptor-ligand interaction to design novel ligand-based reagents comprised of receptor binding domains that significantly improved antitumor immune responses.

**Methods**

We engineered monomeric soluble JAG1 (N-EGF3, sJAG1) and multimeric clustered DLL1 constructs and evaluated their immunological effects in lung and pancreatic cancer murine models. FACS was used for immunophenotyping. ELISpot was used to evaluate cytokine production. In vitro experiments were performed using T: DC (3:1 ratio) co-culture with allogeneic BMDC and CD3+CD28-labeled beads. Mice with CD11c (DC)-specific Dll1−/− and Jag2−/− were used to assess ligand-specific T-cell responses. Correlative studies were performed in tumor-infiltrating immune cells from primary human lung biopsies.

**Results**

Treating tumor-bearing mice with monovalent sJAG1 and multivalent DLL1 elicit antigen-specific T-cells, significantly reduce Treg frequency in spleen and lymph nodes, improve anti-tumor immunity, attenuate tumor growth, and increase IFN-γ production by splenocytes and improve mice survival compared to untreated mice. The frequency of effector CD8+T-cells increased upon addition of ligand-based constructs to T+DC co-culture in vitro. Remarkably, PD-1 expression by CD8+ T-effector memory (TEM) cells was reduced 3-fold with sJAG1 treatment indicating their reduced exhaustion and improved effector activity by selective ligand-mediated interventions. These observations were validated in tumor-bearing mice with DC-specific Dll1−/− and Jag2−/−. While DLL1 expression in mice was important for cytotoxic antitumor immune responses, JAG2 expression sustained a Th2 response. A highly significant correlation was observed between the proportion of JAG1 or JAG2-expressing tissue-resident DCs and the numbers of PD-1-expressing TEM cells in cells-infiltrating human lung tumors. Our study substantiates a pre-clinical rationale for incorporating selectively engineered Notch ligands to modulate antitumor T-cell immunity.

**Conclusions**

Data demonstrate efficacy of functional Notch ligand-based constructs in reducing exhaustion and improving anti-tumor T-cell responses. The findings provide a strong rationale for the clinical application of a new approach of Notch ligand-based therapeutics as single agents or in combination to enhance efficacy of current immunotherapies in improving outcomes of solid tumors refractory to checkpoint therapy.

**Ethics Approval**

The animal studies were performed at The Ohio State University Wexner Medical Center in compliance with the protocol approved by the Institutional Animal Care and Use Committee regulations. Lung cancer samples were obtained from patients who signed an informed consent at the Vanderbilt University Medical Center (VUMC). The human study was conducted on de-identified samples under Institutional Review Board (IRB) protocol 000616 from VUMC.

#### P778 ATRC-101: A first-in-class engineered fully human monoclonal antibody that targets a tumor-restricted ribonucleoprotein complex

##### Jeffrey DeFalco, PhD^1^, Daniel Emerling, PhD^1^, Amy Manning-Bog, PhD^1^, Gilson Baia, PhD^1^, Shaun Lippow, PhD^1^, Alexander Scholz, PhD^1^, Yanhong Zhu, MD^1^, Guy Cavet, PhD^1^, Wayne Volkmuth, PhD^1^, Ish Dhawan, PhD^1^, Jonathan Benjamin, MD, PhD^1^, William Robinson, MD PhD^2^, Tito Serafini, PhD^1^, Norman Greenberg, PhD^1^

###### ^1^Atreca, Redwood City, CA, United States; ^2^Stanford University, Palo Alto, CA, United States

####### **Correspondence:** Norman Greenberg (ngreenberg@atreca.com)

**Background**

The checkpoint inhibitor (CPI) class of immunotherapeutic drugs demonstrates a key role for active T cells in anti-tumor immune responses and positive clinical outcomes in cancer treatment. However, the role of B cells and their antibodies in anti-tumor immune responses is less clear. We propose that B cells might aid in tumor control by producing antibodies that target tumor antigens and thereby induce tumor cell lysis or prime anti-tumor T cell responses. To this end we built a proprietary technology called Immune Repertoire Capture® (IRC™) to characterize the active B cell response in patients whose immune systems are responding to disease [1]. We are now using IRC™ to discover antibodies that can identify novel targets and have established a screening and validation platform to identify those antibodies with the potential to become next generation therapeutics.

**Methods**

Our IRC™ technology was used to generate unbiased and virtually error-free, natively paired heavy and light chain sequences of antibodies expressed by individual B cells isolated from patient samples. We expressed plasmablast-sourced antibodies that, in part, had evidence of progressive affinity maturation and class switching and then performed in vitro and in vivo studies to further characterize each antibody for their (i) target identity, (ii) activity profile in vitro (iii) ability to cause tumor growth inhibition, regression and immunologic memory in vivo in a panel of model systems and (iv) safety profile in non-human primates.

**Results**

Using our methodology we now report on ATRC-101, a fully human IgG1/kappa engineered version of an antibody and identified by IRC™ from a plasmablast B cell isolated from a patient with NSCLC adenocarcinoma who had been treated, in part, with checkpoint inhibitor therapy. ATRC-101 binds to a novel target comprised of both a polyadenylate-binding protein (poly(A)-binding protein) and polyadenylated ribonucleic acid (poly(A) RNA). Despite the wide patterns of distribution and gene expression reported for poly(A) RNA-binding family members, thorough analyses of tissue sections via immunohistochemistry (IHC) and immunofluorescence (IF) demonstrate that target expression is remarkably tumor-associated in both human and mouse. In immunologically "cold" syngeneic mouse tumor models such as EMT6, ATRC-101 demonstrates robust and persistent anti-tumor activity as monotherapy. No safety signals are observed in non-human primates at dose levels up to and including 100 mg/kg in repeat dose studies.

**Conclusions**

Based on robust in vitro and in vivo data ATRC 101 is now being advanced to the clinic for evaluation in solid tissue malignancies.

**Acknowledgements**

We acknowledge the significant effort and contributions of our colleagues from the histology, in vivo pharmacology, in vitro pharmacology and target identification groups as well as our consultants. This include Iraz Aydin, Adam Abtahie, Jerald Aurellano, Felix Chu, Cathrin Czupalla, Gregg Espiritu Santo, Sheila Fernandez, Nicole Haaser, Benjamin Haugen, Dongkyoon Kim, Yvonne Leung, Alan Liu, Fengling Liu, Earth Light Lowe, Sumi May , Harbell Mike, Beatriz Millare, Xiao Peng, Judevin Sapugay, Yann Chong Tan , Steve Tobia, Nikhil Vad, Mauricio Velasco-Delgado, Jenny Wu, Danhui Zhang, Patricia Zuno, Douglas Miller, John Hill and Kevin Baker.

**References**

1. DeFalco, J., Harbell M., Manning-Bog, A., et al. Non-progressing cancer patients have persistent B cell responses expressing shared antibody paratopes that target public tumor antigens. Clinical Immunology 2018; 187, 37-45

**Ethics Approval**

The study was approved by WIRB (Western Institutional Review Board) on Jun11, 2013. The WIRB study number is 20130121.

#### P779 HER2/PD-L1/CD3/HSA tetra-specific antibody mediates cytolytic synapse-restricted PD-L1 blockade and potent depletion of HER2+ tumors

##### Tea Gunde, PhD^1^, Alexandre Simonin^1^, David Urech^1^ , Stefan Warmuth^1^, Christian Hess^1^, Matthias Brock^1^, Eva Oswald^2^, Julia Zeberer^1^, Dania Diem^1^, Dana Mahler^1^, Diego Morenzoni^1^, Simone Muntwiler^1^, Benjamin Küttner^1^, Robin Heiz^1^, Naomi Flückiger^1^, Sebastian Meyer^1^, Timothy Egan^3^

###### ^*1*^*Numab Therapeutics, Wädenswil, Switzerland;*^*2*^*Charles River Discovery Service Germany, Freiburg, Germany* ; ^*3*^*Numab US LLC, Northborough, MA, United States*

####### **Correspondence:** David Urech (d.urech@numab.com)

**Background**

TAA/CD3 bispecific antibodies are highly effective at depleting cancer cells and have achieved encouraging results in the clinical setting. However, two prominent issues occasionally attenuate their efficacy in patients: 1) pre-existing or treatment-emergent expression of PD-L1 on tumor cells and 2) drug-induced cytokine bursts leading to cytokine release syndrome (CRS). Addressing these two issues in the context of a single, conveniently dosed, next-generation molecule could improve rates, and durations, of patient responses while rendering CD3-mediated T cell-stimulation more tolerable. In service of this goal, we constructed a HER2/PD-L1/CD3/HSA tetra-specific MATCH4 molecule (ND022), whose αPD-L1 domain is affinity-tuned to necessitate HER2-binding by the molecule in order to block PD-1 signaling and whose αHSA domain not only extends serum half-life, but also potentially reduces cyto-kine release without surrendering antitumoral efficacy.

**Methods**

We produced a novel HER2/PD-L1/HSA tri-specific scMATCH3 (ND030) and ND022 to evaluate the con-tribution of the αPD-L1 domain to tumor cell-depletion and to confirm that PD-L1-blockade is restrict-ed to the cytolytic synapse. In a series of in vitro experiments, we applied these molecules to co-cultures of reporter T cells or human peripheral blood mononuclear cells (hPBMCs) and either HCC1954 (PD-L1+HER2+) or HCC827 (PD-L1+HER2−) cancer cell-lines. We then applied both molecules, in a controlled experiment, to humanized NOG mice xenografted with HCC1954 breast cancer cells to evaluate antitumoral efficacy in vivo.

**Results**

Both ND030 and ND022 strictly inhibit PD-1-signaling on T cells when such cells are co-cultured with a PD-L1+HER2+ cancer cell-line, but not on HER2− cells, confirming cytolytic synapse-restricted PD-L1-binding. Compared to a HER2/CD3 bispecific single-chain diabody, ND022 depletes HER2+ cells with equal potency. When anchored to HER2+ cells, ND030 less potently inhibits PD-1-signaling than nivolumab in vitro, but its tumor-directed PD-L1-blockade results in superior antitumoral efficacy in vivo. ND022 outperforms trastuzumab + nivolumab combination therapy in vivo, eliciting a complete response in 8/8 mice.

**Conclusions**

ND022 is a next-generation tetra-specific molecule with exquisite antitumoral efficacy in HER2+ cancer models. The molecule’s prophylactic αPD-L1 domain is inert in the absence of PD-L1/HER2 co-expression but potently blocks PD-L1 on PD-L1+HER2+ tumor cells, enabling safe administration to patients with both PD-L1+ and PD-L1− tumors.

#### P780 Selection and in vitro characterization of ABBV-368, a novel anti-OX40 agonist antibody

##### Fiona Harding, PhD^1^ , Marcia Stickler^1^, Margo Werner^1^, Jennifer Tran^1^, Nicole Belmar, BS^1^, Archana Thakur^1^, Enrico DiGiammarino^32^, David Powers^21^

###### ^1^AbbVie Biotherapeutics Inc., Redwood City, CA, United States; ^32^AbbVie, North Chicago, IL, United States

####### **Correspondence:** Fiona Harding (fiona.harding@abbvie.com)

**Background**

OX40 (CD134, TNFRSF4) is a type I transmembrane cell surface protein member of the TNF receptor superfamily transiently expressed on recently activated T cells, present at a low level on memory CD4 T cells, and is expressed on activated and intratumoral T regulatory cells. OX40 can also be expressed on B cells, CD8 T cells, neutrophils and NK and NKT cells following activation. OX40 ligand binding to OX40 results in signaling via TRAF family proteins and upregulation of pro-survival molecules such as Bcl-2, Bcl-XL and survivin. In addition, OX40 signaling in the absence of CD3 signaling may contribute to homeostasis of OX40 expressing memory cells. Targeting OX40 expressed by T regulatory cells has been shown to impact suppressive capacity and to lead to the active depletion of OX40 positive T regulatory cells from the tumor microenvironment. Finally, conversion of naïve CD4+ T cells into T regulatory cells is inhibited by OX40-mediated signals. We sought to create an anti-OX40 agonist activity with both T cell stimulatory activities and with demonstrated ability to inhibit T regulatory activity.

**Methods**

A set of anti-human hybridoma clones was created and screened based on binding by ELISA and NFkB bioactivity in a reporter cell-based assay. Four potential leads were humanized and tested for binding affinity by Biacore analysis, cross-reactivity to cynomolgus monkey OX40, reporter cell activity, IFN-g secretion in human cell based MLR assays, inhibition of Tregulatory cell conversion in vitro, and activity in human CD4 T cell activation assays.

**Results**

ABBV-368 was selected based on the best bioactivity in these assays. Interestingly, the lead candidate was found to have a modest affinity of 42 nM. ABBV-368 binds to the CRD II/III region and inhibits OX40L binding. In an ADCC reporter cell assay, ABBV-368 showed ADCC activity when target cells expressed high levels of OX40. ABBV-368 uniquely demonstrates an ability to signal NFkB in reporter cell lines in the absence of crosslinking. ABBV-368 was tested for bioactivity in human cell engrafted mouse models. ABBV-368 treatment resulted in excellent anti-tumor activity in a humanized mouse tumor model, enhanced GVHD in a human PBMC induced model and showed anti-tumor activity in a human OX40 transgenic mouse.

**Conclusions**

ABBV-368 is an anti-OX40 agonist with unique signaling activity that is efficacious in human cell in vitro and in vivo models and represents a promising clinical candidate currently being evaluated in several ongoing Phase I clinical trials (NCT03071757; NCT03893955; NCT03818542).

**Acknowledgements**

All authors are employees of AbbVie. The design, study conduct, and financial support for this research were provided by AbbVie. AbbVie participated in the interpretation of data, review, and approval of the publication.

#### P781 A new approach used to characterise off target peptide repertoires for T cell receptors that target the cancer testis antigen NY-ESO-1-HLA-A*02:01

##### Stephen Harper^1^ , Charlotte Coles, PhD^1^, Rachel Mulvaney^1^, Sunir Malla, PhD^1^ , Andrew Walker, PhD^1^, Kathrine Smith^2^, Angharad Lloyd^1^, Jane Harper^1^, Zoe Donnelan^1^, Andrew Knox^1^, Andrea Stacey^1^, Joseph Dukes^1^, Emma Baston^1^, Suzanne Griffin^2^

###### ^1^Immunocore Ltd, Milton, Abingdon, United Kingdom; ^2^GlaxoSmithKline, Stevenage, United Kingdom

####### **Correspondence:** Stephen Harper (stephen.harper@immunocore.com), Sunir Malla (Sunir.Malla@immunocore.com)

**Background**

T cell receptor (TCR) cross-reactivity or poly-specificity is thought to be essential for adequate recognition of the potential pathogenic repertoire. Furthermore, understanding a TCRs potential off-target repertoire forms an important part of an immunotherapeutic TCR safety profile. Understanding the extent to which TCRs targeting the same antigen overlap in their off-target peptide repertoires will further elucidate the molecular rules governing TCR cross-reactivity.

**Methods**

We characterised three newly isolated TCRs specific for the cancer testis antigen NY-ESO-1 a.a 157-165 presented on HLA-A*02:01. We describe TCR-pHLA crystal structures and use a novel single chain HLA phage display approach to determine peptide variant specificity profiles.

**Results**

We show that all three TCRs have different peptide specificity profiles, enabling recognition of distinct off-target peptides. Two of the TCRs have overlapping specificity profiles and engage the same central peptide feature, whereas a third TCR has a non-overlapping specificity profile. Although this third TCR engages NY-ESO-1-HLA-A*02:01, the interaction involves an alternate NY-ESO-1 peptide conformation, recognising an antigenic feature which would typically be buried and resulting in off-target peptides sharing little similarity with the cognate peptide.

**Conclusions**

We demonstrate that TCRs targeting the same antigen can achieve the necessary specificity to achieve self-tolerance despite recognising distinct peptide repertoires. This reconciles how an individual’s limited TCR repertoire, following negative selection in the thymus, is able to recognise a vastly larger antigenic pool. These findings significantly advance our understanding of TCR-antigen specificity and we anticipate that approaches such as these will play a crucial role in increasing the safety of TCR-based immunotherapeutic molecules in the clinic.

#### P782 A novel fully synthetic dual targeted Nectin-4/4-1BB *Bicycle®* peptide induces tumor localized 4-1BB agonism

##### Kristen Hurov, PhD , Punit Upadhyaya, Marianna Kleyman, Jessica Kublin, Tom Stephen, Jun Ma, Liz Repash, Julia Kristensson, Sophie Watcham, Liuhong Chen, Sailaja Battula, PhD, Johanna Lahdenranta, Kevin McDonnell, PhD, Nicholas Keen

###### Bicycle Therapeutics, Lexington, MA, United States

####### **Correspondence:** Kristen Hurov (kristen.hurov@bicycletx.com)

**Background**

4-1BB (CD137/TNFRSF9) is an inducible costimulatory receptor belonging to the TNF receptor superfamily. Despite compelling preclinical data, 4-1BB agonistic antibodies have been hampered by failure to delineate hepatotoxicity from efficacy in the clinic [1]. Next generation strategies are focused on bispecific approaches aimed at promoting target-mediated clustering of 4-1BB to limit systemic and liver toxicities [2,3]. *Bicycles®* represent a new class of drugs - fully synthetic, constrained bicyclic peptides that have antibody-like affinity and selectivity to their targets. Unlike traditional biologic approaches, the small size (~2 kDa) and tunable pharmacokinetic (PK) parameters of *Bicycles* enable superior tumor penetration and allow exploration into the relationship between pulsatile dosing and 4-1BB activation while de-risking hepatoxicity concerns due to a differentiated renal clearance mechanism combined with tumor-localized activation.

**Methods**

We envisioned clustering and activation of 4-1BB could be achieved by conjugating a 4-1BB binding *Bicycle* to a tumor antigen targeting *Bicycle*. The synthetic simplicity and highly modular nature of the *Bicycle®* platform enabled us to rapidly explore this hypothesis. The functional activity of the molecules was analyzed using a suite of relevant in vitro assays and animal models.

**Results**

Nectin-4/PVRL4 is highly expressed in numerous tumors, including bladder, pancreatic, and lung. Synthetic “bispecific” Nectin-4/4-1BB *Bicycles* exhibit extremely potent (EC50=~100 pM) and Nectin-4-dependent 4-1BB agonism in an engineered 4-1BB reporter system and induce robust production of pro-inflammatory cytokines when human peripheral blood mononuclear cells (PBMCs) are co-cultured with Nectin-4-expressing tumor cells. Importantly, 4-1BB agonism is also achieved in ex-vivo experiments utilizing patient-derived lung tumor samples with an intact immune microenvironment. We observed profound changes in immune markers, including IL-2, and increased CD8+ T cell proliferation only in lung tumors expressing Nectin-4. Excitingly, we have demonstrated *in vivo* anti-tumor activity in a Nectin-4 overexpressing syngeneic tumor model. Daily dosing at 1mg/kg led to tumor growth inhibition in 4 out of 5 animals including 2 complete responses despite the PK parameters predicting only transient target coverage.

**Conclusions**

These data indicate that the biological effect of the bispecific *Bicycle* agonist persists beyond the detection of the molecule in systemic circulation and suggests that continuous coverage of the target may not be necessary when activating 4-1BB with low molecular weight and highly tumor penetrant *Bicycles*. We have extended the *Bicycle* dual targeted approach to other tumor and immune cell targets and have found this concept to be extremely generalizable, paving the way for future novel non-biologic immune cell engaging immunotherapies.

**References**

1. Segal NH, Logan TF, Hodi FS, et al. Results from an integrated safety analysis of urelumab, an agonist anti-CD137 monoclonal antibody. Clin Cancer Res. 2017;23(8):1929-1936.

2. Pastor F, Kolonias D, McNamara JO 2nd, Gilboa E. Targeting 4-1BB costimulation to disseminated tumor lesions with bi-specific oligonucleotide aptamers. Mol Ther. 2011; 19(10):1878-1886.

3. Claus C, Ferrara, C, Xu W, et al. Tumor-targeted 4-1BB agonists for combination with T cell bispecific antibodies as off-the-shelf therapy. Sci Transl Med. 2019; 11(496): eaav5989.

**Ethics Approval**

Cryopreserved human dissociate tumor cells (DTCs) and PBMCs were purchased from approved vendors that follow stringent Institutional Review Board (IRB) and Ethics Committee compliance. The care and use of animals were reviewed and approved by the Institutional Animal Care and Use Committee (IACUC) of WuXi AppTec (Approval #ON01-003-2017V1) and conducted in accordance with the regulations of the Association for Assessment and Accreditation of Laboratory Animal Care (AAALAC).

#### P783 DuoBody®-CD3x5T4 shows potent preclinical anti-tumor activity in vitro and in vivo in a range of cancer indications

##### Kristel Kemper, PhD , Ellis Gielen, Laura Smits-de Vries, Sandra Verploegen, Mischa Houtkamp, Saskia Burm, Edward van den Brink, Rik Rademaker, Dennis Verzijl, Patrick Engelberts, Bart E.C.G. de Goeij, David Satijn, A. Kate Sasser, Esther Breij, PhD

###### Genmab, Utrecht, Netherlands

####### **Correspondence:** Kristel Kemper (kke@genmab.com)

**Background**

Promising clinical activity has been observed with multiple CD3 bispecific antibodies (bsAbs) in hematological malignancies. In solid cancers, a handful of trials assessing the clinical safety and preliminary efficacy of CD3 bsAbs are ongoing, yet clinical efficacy remains to be established. The tumor antigen 5T4 (trophoblast glycoprotein) is highly expressed in multiple solid tumors, while its expression in normal tissue is limited. Therefore, 5T4 is an attractive target for a CD3 bsAb approach. Here, we introduce DuoBody-CD3x5T4, a CD3 bsAb targeting 5T4-expressing tumor cells.

**Methods**

DuoBody-CD3x5T4 is an Fc-silenced IgG1 bispecific antibody that was obtained by controlled Fab-arm exchange of a humanized CD3ε and a human 5T4 monoclonal antibody. The capacity of DuoBody-CD3x5T4 to induce T-cell activation, T-cell-mediated cytotoxicity and production of inflammatory cytokines was analyzed in vitro using co-cultures of 5T4-expressing tumor cells and T cells isolated from healthy donors (effector:target cell ratio 4:1, 72 h). Efficacy of DuoBody-CD3x5T4 in vivo was tested in humanized mouse models using cell line-derived (CDX) and patient-derived xenografts (PDX) expressing 5T4.

**Results**

The expression of 5T4 in different tumor types was confirmed by flow cytometry across tumor cell lines, and immunohistochemistry in TMAs for patient-derived samples. Functional characterization in vitro showed that DuoBody-CD3x5T4 induced concentration-dependent T-cell activation, cytokine production and cytotoxicity in co-cultures of T cells and 5T4+ cell lines derived from various tumor types. EC50 values for T-cell-mediated cytotoxicity were in the subnanomolar range. Functional activity was dependent on crosslinking of CD3 on T cells with 5T4 on tumor cells, as control antibodies targeting either CD3 or 5T4 alone did not induce T-cell activation, cytotoxic activity or cytokine production. Furthermore, DuoBody-CD3x5T4 did not induce T-cell-mediated cytotoxicity of CRISPR-Cas9-generated 5T4-knockout tumor cells. Both CD4+ and CD8+ T cells could independently perform DuoBody-CD3x5T4-induced cytotoxicity. In addition, DuoBody-CD3x5T4 showed anti-tumor activity in vivo in 5T4+ CDX and PDX tumor models xenografted in humanized mice. Exploratory biomarker studies, including analysis of T-cell activation and cytokines in the blood after DuoBody-CD3x5T4 treatment, are currently ongoing in these in vivo models. Results will be shared.

**Conclusions**

DuoBody-CD3x5T4 is a bispecific antibody that induces CD4+ and CD8+ T-cell activation, cytokine production and T-cell-mediated cytotoxicity of 5T4-expressing tumor cells in vitro and in vivo. Due to its mechanism of action, the broad expression of 5T4 across cancer indications and limited expression in normal cells, DuoBody-CD3x5T4 is a promising novel drug candidate with potential anti-tumor effect across different solid tumors.

**Ethics Approval**

The CDX animal experiments performed are in compliance with the Dutch animal protection law (WoD) translated from the directives (2010/63/EU) and are approved by the Ethical committee of Utrecht. For the performed PDX animal experiments, all patients included had given written informed consent and were carried out in accordance with the German Animal Protection Law (LaGeSoBerlin, A0452/08). The studies were approved by the local Institutional Review Board of Charite University Medicine, Germany.

#### P784 SBT6050, a HER2-directed TLR8 agonist antibody conjugate, designed to overcome primary resistance to and synergize with checkpoint inhibition in HER2-expressing tumors

##### Kara Moyes, Jamie Brevik, Damion Winship, Ty Brender, Heather Metz, Yvette Latchman, PhD, Monica Childs, Michael Comeau, Jenny Chang, Sean Smith, Hengyu Xu, Ben Setter, Ray Carrillo, Li-Qun Fan, Phil Tan, Robert DuBose, Peter Baum, Valerie Odegard, PhD

###### Silverback Therapeutics, Seattle, WA, United States

####### **Correspondence:** Valerie Odegard (vodegard@silverbacktx.com)

**Background**

Tumor cell-extrinsic mechanisms, such as exclusion of cytotoxic T lymphocytes (CTL) and immunosuppression by tumor associated macrophages, myeloid-derived suppressor cells, and regulatory T cells, contribute to immunotherapy primary resistance. Activation and reprogramming of myeloid cells within the tumor microenvironment (TME) can overcome this resistance. SBT6050 is a novel therapeutic comprised of a potent TLR8 agonist conjugated to a HER2-directed monoclonal antibody, designed for systemic delivery and tumor-localized activation of myeloid cells. We have recently reported that treatment of tumor-bearing mice with a SBT6050 surrogate results in durable, single agent efficacy by activating intratumoral myeloid cells and increasing neoantigen specific CTLs. A sustained increase in intratumoral IFN-gamma, a potent inducer of PD-L1, was also observed. Here, we extend these studies to evaluate the efficacy and intratumoral pharmacodynamic activity of the SBT6050 surrogate as a single agent and in combination with anti-PD1 in tumor models with demonstrated primary resistance to checkpoint inhibition (CPI).

**Methods**

SBT6050 activation and reprograming of myeloid cells was evaluated in vitro using human myeloid cell:HER2-expressing tumor cell co-culture assays with flow cytometry and MSD-based ELISA read-outs. Efficacy and pharmacodynamic activity of the SBT6050 mouse surrogate alone or in combination with anti-PD1 was examined in vivo in HER2-expressing EMT6 and CT26 tumor models.

**Results**

SBT6050 potently activates human myeloid cells to produce pro-inflammatory and TH1 priming cytokines such as IL-12p40 and IFN-gamma in the presence of HER2-expressing tumor cells. The EMT6 and CT26 mouse tumor models demonstrate primary resistance to CPI, likely due to low TIL and abundant myeloid cells. Treatment of mice bearing these HER2-expressing tumors with the SBT6050 surrogate resulted in robust, durable single agent efficacy. In addition to increasing TIL infiltrate, the SBT6050 surrogate upregulates PD-L1 and MHCI in the TME. Similarly, in vitro, SBT6050 induces PD-L1 upregulation concomitantly with myeloid cell activation. Given these findings, the SBT6050 surrogate was evaluated in combination with anti-PD1. This combination enhanced the single agent efficacy observed with the SBT6050 surrogate. Intratumoral pharmacodynamic activity associated with combination therapy will also be presented.

**Conclusions**

These data show the potential for SBT6050 to reprogram an immunosuppressive TME and support the clinical evaluation of SBT6050 as a monotherapy and in combination with CPI in HER2-expressing tumors, including those with low pre-existing CTL infiltrate. SBT6050 is projected to enter the clinic in 2020.

#### P785 Exosome surface display of IL-12 results in tumor-retained pharmacology with superior potency and limited systemic exposure compared to recombinant IL-12

##### Katherine Kirwin, Chang Ling Sia, Sonya Haupt, Ke Xu, Kevin Dooley, Su Chul Jang, PhD, Bryan Choi, Adam Boutin, Christine McCoy, Scott Estes, Kyriakos Economides, Sriram Sathyanarayanan, Nuruddeen Lewis

###### Codiak BioSciences, Cambridge, MA, United States

####### **Correspondence:** Nuruddeen Lewis (nuruddeen.lewis@codiakbio.com)

**Background**

The promise of IL-12 as a treatment for cancer has never been fulfilled despite numerous clinical trials. Severe toxicity due to systemic exposure has impeded clinical development. Intratumoral (IT) administration of recombinant IL-12 (rIL-12) has been attempted to avoid toxicity, but significant systemic exposure was detected within 30 minutes [1]. To address these limitations, we have created exoIL-12™, a novel, engineered-exosome therapeutic that displays IL-12 on the surface of an exosome. ExoIL-12 has restricted pharmacology within the tumor upon IT administration resulting in superior in vivo efficacy without systemic exposure.

**Methods**

IL-12 was displayed on the surface of exosomes by genetic fusion to PTGFRN. Both human and mouse constructs were prepared. In vitro potency of rIL-12 and exoIL-12 was determined by IFNγ-release assays using primary human or mouse target cells as appropriate. Syngeneic mouse models (B16F10, MC38) were utilized to characterize anti-tumor immune responses of exoIL-12 or rIL-12 and comparisons were based upon matched IL-12 concentrations as measured by ELISA.

**Results**

The exoIL-12 constructs retained equivalent potency in vitro to rIL-12 as demonstrated by IFNγ-release using primary human PBMCs or murine splenocytes. Following IT injection, we found that exoIL-12 demonstrated significantly greater anti-tumor activity than rIL-12. In the MC38 model, exoIL-12 was 100-fold more potent than rIL-12 in tumor growth inhibition. Complete responses (CR) were observed in 63% of mice treated with exoIL-12; in contrast, rIL-12 resulted in 0% CRs at an equivalent IL-12 dose. Systemic immunity was observed as evidenced by dose-dependent increases in tumor antigen-specific CD8+ T cells in the spleen using p15E tetramers. Re-challenge studies of CR mice showed no tumor regrowth, suggesting immune-mediated rejection and memory T cell activity. In the B16F10 model, exoIL-12 achieved a 75% reduction in tumor growth and significantly reduced the number of lung nodules in mice intravenously challenged with B16F10 in addition to the effect on the primary tumor in the flank. Using a specific immunoassay for IL-12p70, rIL-12 was found to have a short tumor retention time of 3 h, whereas exoIL12 was still detectable 48 h post-injection. This resulted in exoIL-12 having prolonged IT IFNγ production.

**Conclusions**

ExoIL-12 is a novel cancer therapeutic candidate with tumor-retained pharmacological activity and superior potency as compared to the rIL-12.

**References**

1. Van Herpen CM, Huijbens R, Looman M, et al. Pharmacokinetics and immunological aspects of a phase Ib study with intratumoral administration of recombinant human interleukin-12 in patients with head and neck squamous cell carcinoma: A decrease of T-bet in peripheral blood mononuclear cells. Clin Cancer Res. 2003;9(8):2950-2956.

**Ethics Approval**

The study was approved by Codiak BioSciences' internal IACUC committee, approval number CB2017-001.

#### P786 Network meta-analysis (NMA) and matching-adjusted indirect comparison (MAIC) of pembrolizumab versus atezolizumab for second-line (2L) locally advanced/metastatic urothelial carcinoma (mUC)

##### Dave Gelb, Yichen Zhong, PhD, Rodolfo Fleury Perini, James Luke Godwin, Yizhen Lai, Stella Arndorfer^2^, Maria Lorenzi, MSc^2^, Haojie Li

###### Merck & Co., Inc., Kenilworth, IL, United States; ^*2*^*Precision Xtract, Oakland, CA, United States*

####### **Correspondence:** Haojie Li (haojie.li@merck.com)

**Background**

KEYNOTE-045 (ClinicalTrials.gov identifier: NCT02256436) demonstrated statistically and clinically meaningful improvements in overall survival (OS) in 2L mUC subjects treated with pembrolizumab versus investigator’s choice of vinflunine, docetaxel, or paclitaxel (median survival, 10.1 versus 7.3 months; P < 0.001). IMvigor 211 (ClinicalTrials.gov identifier: NCT02302807) showed no significant improvement in the primary endpoint of OS (median survival, 11.1 versus 10.6 months; P = 0.41) in 2L mUC subjects treated with atezolizumab versus investigator’s choice of chemotherapy. No head-to-head trial has compared the efficacy of pembrolizumab versus atezolizumab for 2L mUC patients.

**Methods**

To synthesize RCT evidence and indirectly compare the relative treatment effects of pembrolizumab versus atezolizumab, fixed-effect Bayesian network meta-analysis (NMA) was conducted to determine the relative efficacy of both treatments. Hazard ratios (HRs) for OS were estimated with 95% credible intervals (CrIs). Furthermore, an anchored matching-adjusted indirect comparison (MAIC) was conducted using individual patient-level data (IPD) from KEYNOTE-045 and published data from IMvigor 211. In the MAIC, to adjust for differences in baseline potential effect modifiers between these trials, data for subjects from KEYNOTE-045 were re-weighted to match the baseline characteristics reported in IMvigor 211.

**Results**

Pembrolizumab consistently showed favorable OS benefit over atezolizumab in both analytical approaches (NMA method: HR [95% CrI] = 0.83 [0.63-1.07]; MAIC method: HR [95% confidence interval] = 0.72 [0.53-0.97]).

**Conclusions**

These results suggest that pembrolizumab offers a better probability of prolonging OS compared to atezolizumab as 2L therapy for mUC patients. In the absence of direct comparison and sufficiently powered studies, these analyses provide valuable insight on the relative efficacy of pembrolizumab versus atezolizumab for patients, physicians and payers, using the best available methods and evidence.

#### P787 Novel humanized CD3ε mouse model for evaluation of bi-specific T-cell engager antibodies

##### Xiaofei Zhou, Quinn Li, Dirui Li, Rui Huang, Yuelei Shen

###### Biocytogen, Boston, MA, United States

####### **Correspondence:** Xiaofei Zhou (info@biocytogen.com)

**Background**

CD3ε is one of the key signaling subunits of the CD3 co-receptor of the T cell receptor (TCR) complex. This TCR complex plays critical role in mediating T cell activation and cellular immune response. In recent years, bispecific antibodies that target human CD3ε and redirect T cell cytotoxicity toward tumor cells has been extensively investigated and some have shown clinical success and promise. However, due to the relatively low homology between the extracellular domain of human CD3 and mouse CD3, many antibodies that recognize human CD3 do not cross-react with mouse CD3. Hence, a murine model suitable for directly evaluating CD3-specific therapy is needed.

**Methods**

To expedite the in vivo interrogation of human CD3-based therapeutics, Biocytogen generated the B-hCD3ε mice where the extracellular domain of human CD3ε replaces that of the mouse counterpart. Here we present evidence that these humanized CD3ε mice demonstrated normal T cell development compared with wild type C57BL/6 mice.

**Results**

B-hCD3ε mice responded well to anti-PD-1-mediated tumor suppression and exhibited T cell depletion by an anti-human CD3 antibody. In addition, when stimulated with anti-CD3 antibody in vitro, T cells of B-hCD3ε mice showed comparable level of production of cytokines to that by T cells of wild type mice. Finally, in efficacy test, bispecific anti-human CD3/CD19 antibody blinatumomab dose-dependently inhibited MC38-hCD19 tumor growth in B-hCD3ε mice.

**Conclusions**

Taken together, Biocytogen’s B-hCD3ε mice are validated and exhibit expected T cell profile, robust in vitro activity, and efficacious in vivo response to anti-human CD3 antibody and anti-human CD3 bispecific antibody. These humanized B-hCD3e mice present a useful model for accelerated in vivo evaluation of anti-human CD3 therapeutics.

#### P788 Development of ADG116, a novel antagonist anti-CTLA-4 human antibody for cancer immunotherapy

##### Guizhong Liu, PhD, Felix Fangyong Du, Kristine She, Peter Luo

###### Adagene, Suzhou, China

####### **Correspondence:** Peter Luo (peter_luo@adagene.com)

**Background**

CTLA-4 and CD28 exemplify a co-inhibitory and co-stimulatory T cell signaling axis by sharing common ligands CD80 and CD86, which regulate antigen-specific T cell immunity. CTLA-4 acts as a checkpoint to confine the magnitude of CD28 receptor mediated T cell activation and has been widely investigated as a target to boost antitumor immunity.

ADG116 is a fully human antagonistic anti-CTLA-4 IgG1 monoclonal antibody identified through our proprietary Phage display Dynamic Precision Library (DPL) technology. Different from the clinically approved Ipilimumab, ADG116 binds to a unique evolutionally conserved epitope on CTLA-4, which endows ADG116 a broad species cross-reactivity to CTLA-4 of human, monkey and rodent origins, and allows the extensive evaluation of its MOA and efficacy in various mouse cancer models. Compared to Ipilimumab, ADG116 in vitro is a weaker CTLA-4 checkpoint inhibitor in terms of its ligand relieving activity to co-stimulate CD28 receptor signaling, while ADG116 has much potent ADCC activity towards activated Treg cells but not effector T cells. In vivo, ADG116 alone exhibits robust dose-dependent single agent anti-tumor activity in established tumors and/or increases overall survival in syngeneic mouse models of liver, breast, lung and colon cancers, as well as induces durable antigen-specific memory immunity that protect animals from re-challenged tumor cells. The activity of ADG116 to enhance activation and inflammatory cytokine release of primed T cells in vitro and specifically deplete intra-tumoral Treg cells in vivo, is consistent with the MOA by which ADG116 acts through two distinct mechanisms: 1) functions as a checkpoint inhibitor to directly enhance effector T cell activation, and 2) functions as an ADCC targeting antibody to alleviate immunosuppression in tumor microenvironment through specific Treg depletion in tumors but not in peripheral tissues. Both mechanisms are involved in mediating its potent anti-tumor responses. In a human CTLA-4 knock-in mouse tumor model, ADG116 exhibits more potent anti-tumor efficacy than Ipilimumab, along with stronger intra-tumoral Treg depletion. Weekly repeat-dose GLP toxicology studies demonstrate a relatively good safety profile for ADG116 in rats and cynomolgus monkeys, respectively.

These pre-clinical results provide strong support for further clinical development of ADG116 as a novel anti-cancer immunotherapy agent for a broad spectrum of human malignancies. ADG116 is currently being evaluated in the phase 1 first in human study in patients with advance/metastatic cancers in US.

**Methods**

It is not a clinical trial abstract.

#### P789 Vaccination of high-risk neuroblastoma patients with anti-id antibody ganglidiomab to maintain protective immunity after passive immunotherapy with anti-GD2 antibody ch14.18/CHO

##### Hans Loibner, PhD^1^, Holger Lode, MD, PhD^2^ , Nikolai Siebert, PhD^2^, Sascha Troschke-Meurer, BS^2^, Oliver Mutschlechner^1^

###### ^1^HL Bioscience Research GmbH, Vienna, Austria; ^2^University Clinic Greifswald, Greifswald, Germany

####### **Correspondence:** Holger Lode (lode@uni-greifswald.de)

**Background**

Disialoganglioside GD2 is abundantly expressed on neuroblastoma (NB) with little expression on normal cells, thus is a suitable target for immunotherapy. Chimeric anti-GD2 antibodies ch14.18 (dinutuximab) and ch14.18/CHO (dinutuximab beta) were developed and are now marketed in US and Europe resp. for passive immunotherapy of high-risk NB. Active immunization based on GD2 following antibody therapy may induce a long-lasting protective immunity to further extend the therapeutic benefit of NB immunotherapy. Anti-idiotypic antibody ganglidiomab to ch14.18 was raised and shown to carry an internal image of GD2 in vitro and in murine models [1]. Here we describe results of a compassionate need program in NB patients vaccinated with alum adsorbed ganglidiomab, following ch14.18/CHO immunotherapy.

**Methods**

Between 2012 and 2014, six high-risk NB patients (2-8 y); 4 in CR, 2 with disease) following multimodal therapies including stem cell transplant and ch14.18/CHO immunotherapy were repeatedly treated under compassionate need with 0,5mg alum adsorbed ganglidiomab by subcutaneous vaccination (5-10 vaccinations every 2 weeks). Blood for serum measurements was withdrawn at various timepoints. Antibody response to ganglidiomab and GD2 by ELISA as well as complement lysis to a NB cell line was investigated. Last patient follow-up was in 2019.

**Results**

Vaccinations were well tolerated with mild transient local reactions only. In immune sera of all patients a marked induction of antibodies to ganglidiomab and to its variable regions was detected. In 3 of 6 patients also an induction of antibodies to GD2 was found. Induction of cytolytic activity via complement of GD2+ LAN1 NB cells was seen in 2 patients. All six patients were alive without evidence of disease at their last follow up in 2019, i.e. 5 years after start of vaccinations.

**Conclusions**

Repeated s.c. injections of alum-adsorbed ganglidiomab may evolve as additional well tolerated immunotherapy modality after ch14.18 antibody treatment to maintain a long-lasting immunity for prevention of progression or re-occurrence of disease. A clinical trial program is warranted to explore this hypothesis.

**References**

1. Lode HN, Schmidt M, Seidel D. Vaccination with anti-idiotype antibody ganglidiomab mediates a GD(2)-specific anti-neuroblastoma immune response. Cancer Immunol Immunother. 2013 62(6):999-1010

**Ethics Approval**

Compassionate need treatment

#### P790 The anti-tumor activity of an anti-CD39 antibody (ES002) in a multiple myeloma model is dependent on NK cells

##### Yangsheng Qiu, Qinglin Du, PhD, Meiling Sun, Rui Gao, PhD, Huimin Kong, Roumei Xing, Yufei Shi, Mengying Xu, Zhihao Wu, PhD, Hongtao Lu, PhD

###### Elpiscience Biopharma, Shanghai, China

####### **Correspondence:** Hongtao Lu (hongtaolu@elpiscience.com)

**Background**

CD39-CD73-adenosine pathway plays an important immuno-suppressive role within the tumor microenvironment (TME) as adenosine receptor signaling suppresses effector T cells and enhances immune inhibitory effects of Tregs and MDSCs. To overcome the immunosuppression by adenosine in TME, we choose to target CD39 for two main reasons: 1). CD39 plays a pivotal role in converting extracellular ATP into adenosine. Blocking CD39 enzymatic activity will not only lead to the inhibition of adenosine generation, but also maintain extracellular ATP which enhances T cell priming by dendritic cells (DCs). 2). CD39 is expressed highly in Tregs and exhausted T cells, inhibition of CD39 activity will likely suppress Treg inhibition and reinvigorate exhausted T cells.

**Methods**

We have generated a CD39 antibody, ES002, by traditional hybridoma technology using human CD39 overexpressing HEK293 cells as immunogen, and the antibody was subsequently humanized through complementarity determining region grafting. ES002 binding to CD39 and inhibition of ATPase activity were evaluated through protein-based and cell-based assays. The immunological function of ES002 was studied in an in vitro Elpiscience proprietary Immuno-assay platform (ImmunoShine). The in vivo efficacy of ES002 was investigated in a multiple myeloma CDX cancer model. The effector immune cells were each depleted to analyze their respective roles in tumor growth inhibition.

**Results**

ES002, a humanized anti-hCD39 antibody, binds to human and cynomolgus CD39 with sub-nanomolar binding affinity and effectively blocks CD39 ATPase activity. We found that CD39 was highly upregulated in Tregs within tumor microenvironment in several cancer types including NSCLC, HCC and CRC. ES002 reversed Treg inhibition of IFN-gamma production by effector T cells. ES002 enhanced the expression of costimulatory molecules on monocytes and dendritic cells activated by ATP. ES002-pretreated DCs can functionally enhance T cell proliferation and IFN-gamma production in an MLR assay. ES002 significantly suppressed tumor growth in a multiple myeloma CDX tumor model and depleting antigen presenting cells (APC) or NK cells in these animals completely abolished the anti-tumor activity of the antibody.

**Conclusions**

We have successfully developed a potentially best-in-class anti-CD39 therapeutic antibody with strong efficacy in tumor growth inhibition. The antibody appears to have dual functions. It suppresses Treg inhibition on effector T cells. It engages APC and NK cells for its antitumor efficacy in vivo.

#### P791 Anti-tumor activity of a small molecule inhibitor of the immuno-suppressive enzyme Interleukin 4 (IL-4)-Induced Gene 1 (IL4I1)

##### Andy MacKinnon, PhD , Deepthi Bhupathi, MS, Jason Chen^2^, Rosalyn Dang, Tony Huang, Weiqun Li, PhD, Yong Ma, Natalija Sotirovska, MS, Sandra Spurlock, Susanne Steggerda, PhD, Winter Zhang, Francesco Parlati, PhD

###### Calithera Biosciences, Inc., South San Francisco, CA, United States

####### **Correspondence:** Andy MacKinnon (amackinnon@calithera.com)

**Background**

Altered metabolism is an important mechanism by which tumors evade detection and destruction by the immune system. Metabolic control of immune responses can occur through depletion of essential nutrients or accumulation of toxic metabolic products in the tumor microenvironment that impair immune cell function and promote tumor growth. The secreted enzyme interleukin 4 (IL-4)-induced gene 1 (IL4I1) is an L-phenylalanine oxidase that catabolizes phenylalanine and produces phenyl-pyruvate and H2O2, an inhibitor of T cell proliferation. IL4I1 may promote cancer progression by suppressing the cytotoxic activity of T cells.

**Methods**

IL4I1 enzymatic activity was measured using an HRP-coupled enzyme assay. RNA in-situ hybridization was carried out on the RNAScope platform. Syngeneic mouse tumor models were used to evaluate the anti-tumor activity of small molecule IL4I1 inhibitor 220307. The level of phenyl-pyruvate in tumor homogenates was measured by LC/MS.

**Results**

Interrogation of The Cancer Genome Atlas (TCGA) revealed high expression of IL4I1 in multiple solid tumor types and correlated with reduced overall patient survival (HR = 1.5, p = 4.4 x 10-15). Expression of IL4I1 was further validated using RNA in-situ hybridization in human tumor microarrays. IL4I1 was found to be highly expressed in a subset of cells in several solid tumor types, with notably high expression in ovarian tumors and B cell lymphomas.

We identified 220307, a potent, orally-bioavailable small-molecule inhibitor of IL4I1 (IC50 = 274 nM). Twice-daily oral administration of 220307 resulted in single-agent tumor growth inhibition in several syngeneic mouse tumor models (e.g. B16-F10 and LLC) and when combined with immune checkpoint blockade, further reduced tumor growth. Oral dosing of tumor-bearing mice with 220307 reduced the levels of phenyl-pyruvate in the tumor, consistent with inhibition of IL4I1 enzymatic activity. The anti-tumor activity of 220307 was immune cell-mediated, since anti-tumor efficacy was abrogated in CD8-depleted mice or in SCID mice, and 220307 had no direct anti-proliferative activity on tumor cells grown in vitro (IC50 > 50 μM). These data support an immune-mediated anti-tumor effect of IL4I1 inhibition by 220307.

**Conclusions**

These results indicate that small-molecule inhibition of IL4I1 may represent a novel strategy for cancer immuno-therapy.

#### P792 A GUCY2C-CD3 bispecific engages T cells to induce cytotoxicity in gastrointestinal tumors

##### Divya Mathur, PhD , Adam Root, Bozena Bugaj-Gaweda, Xingzhi Tan, Wei Fang, Stephanie Bisulco, Jonathon Golas, Jessica Kearney, Erik Upeslacis, Johnny Yao, Edward Rosfjord, Chad Stevens, Lindsay King, Jatin Narula, Kerry Kelleher, Cynthia Rohde, Lioudmila Tchistiakova, Anhco Nguyen, Puja Sapra, PhD

###### Pfizer, Pearl River, NY, United States

####### **Correspondence:** Divya Mathur (divya.mathur@pfizer.com), Puja Sapra (puja.sapra@pfizer.com)

**Background**

Guanylyl Cyclase C (GUCY2C) is a regulator of intestinal homeostasis and is expressed in more than 90% of colorectal cancer (CRC), as well as in other gastrointestinal malignancies [1]. Target expression in normal tissues is largely restricted to the apical side of intestinal epithelial tight junctions, which could allow for preferential uptake of GUCY2C targeted biologics by tumors that have disrupted tight junction architecture. Here we demonstrate tumor selective uptake and potent efficacy with a half-life extended, GUCY2C-CD3 bispecific molecule that recruits CD3-positive T cells to induce cytotoxicity of GUCY2C expressing tumors in several CRC models in vivo. Additionally, to address immune evasion mechanisms, rational combinations of the bispecific are explored with immune checkpoint blockade agents, as well as by blocking angiogenesis, which has been reported to enhance T cell infiltration into tumors [2].

**Methods**

GUCY2C-CD3 mediated activity was evaluated in vivo in several cell line-and patient derived-xenograft models of CRC, using adoptive transfer of human T cells in established subcutaneous and orthotopic tumors. Immunohistochemistry was performed to demonstrate biodistribution of the drug and recruitment of activated T cells in GUCY2C expressing tumors vs normal tissues. We also performed CYTOF analyses on GUCY2C-CD3 treated tumors and tumor infiltrating lymphocytes (TILs) to identify markers of immune evasion. Combination studies with Anti-PD-1/PD-L1 checkpoint blockade agents, and with an Anti-VEGF-A antibody were performed using the adoptive transfer model.

**Results**

GUCY2C-CD3 bispecific showed preferential biodistribution to GUCY2C expressing xenograft tumors compared to normal tissue. The drug demonstrated potent dose-dependent efficacy in several CRC models with no signs of toxicity. Bispecific treated tumors not only showed recruitment of activated T cells to the tumor site, but also upregulated checkpoint mechanisms, such as PD-L1 on tumors and exhaustion markers on TILs. Consequently, Anti-PD-1/PD-L1 checkpoint blocking antibodies provided enhanced efficacy when combined with GUCY2C-CD3. Additionally, significant combination benefit was observed when GUCY2C-CD3 was combined with an Anti-VEGF-A blocking antibody.

**Conclusions**

Our preclinical data demonstrate that a GUCY2C-CD3 bispecific can selectively target colorectal tumors, including those with KRAS or BRAF mutations, which are difficult to treat with currently approved therapies. This bispecific shows combination benefits with T cell checkpoint blockade agents, as well as anti-angiogenesis agents, indicating that single agent activity with GUCY2C-CD3 can be further enhanced with mechanisms that address immune evasion.

**Acknowledgements**

We would like to thank Dr. John Hill, Ph.D. for his insightful discussions.

**References**

1. Birbe R, Palazzo JP, Walters R et al. Guanylyl cyclase C is a marker of intestinal metaplasia, dysplasia, and adenocarcinoma of the gastrointestinal tract. Hum Path. 2005; 36(2):170-9.

2. Allen E, Jabouille A, Rivera LB. Combined antiangiogenic and anti–PD-L1 therapy stimulates tumor immunity through HEV formation. Sci Transl Med. 2017; 9(385): eaak9679.

**Ethics Approval**

All procedures performed on animals were in accordance with regulations and established guidelines and were reviewed and approved by an Institutional Animal Care and Use Committee or through an ethical review process.

#### P793 ALPN-202, a conditional CD28 costimulator and dual checkpoint inhibitor, utilizes multiple mechanisms to elicit potent anti-tumor immunity superior to checkpoint blockade alone

##### Mark Maurer, BS , Chelsea Gudgeon, BS, Katherine Lewis, PhD, Sherri Mudri, BS, Stacey Dillon, PhD, Martin Wolfson, BS, Steven Levin, PhD, Kristine Swiderek, PhD, Stanford Peng, MD, PhD

###### Alpine Immune Sciences, Seattle, WA, United States

####### **Correspondence:** Mark Maurer (mark.maurer@alpineimmunesciences.com)

**Background**

Although immune checkpoint inhibitors (CPI) targeting the CTLA-4 and PD-1/PD-L1 pathways have demonstrated significant clinical activity in many cancers, many patients fail to respond, or they develop acquired resistance due at least in part to insufficient anti-tumor T cell activation and/or exhaustion. Based on this hypothesis, CPIs have been combined with T cell costimulation and shown to enhance and sustain anti-tumor responses preclinically. ALPN-202 is a variant CD80 vIgD™-Fc fusion protein that mediates PD-L1-dependent CD28 costimulation and inhibits the PD-L1 and CTLA-4 checkpoints (Figure 1). Studies were conducted to characterize the unique mechanism of action of this molecule and further elucidate CD80/PD-L1 biology.

**Methods**

X-ray crystallography and cell-based reporter assays were used to demonstrate the simultaneous binding of PD-L1 and CD28 to the single CD80 IgV domain of ALPN-202. PD-L1-dependent CD28 costimulation was evaluated using primary human T cells stimulated by antigen presenting cells (APC) with or without surface PD-L1. ALPN-202 anti-tumor activity was mechanistically evaluated in vivo using a human PD-L1-transduced MC38 tumor model, by combining ALPN-202 treatment with anti-PD-L1, anti-CTLA-4, or anti-CD28 blocking antibodies. Anti-tumor activity was evaluated by serial tumor volume measurements, and intratumoral immune responses were assessed by flow cytometry and/or RNA-Seq analyses.

**Results**

The CD80 vIgD:PD-L1 crystal structure was elucidated at a resolution of 3.15 Å, revealing a non-overlapping binding interface distinct from the previously published CD28:CD80 interaction (Figure 2). Using cell-based reporter and cytokine release assays, monomeric and dimeric CD80 vIgD domains elicited conditional CD28 costimulation which was blocked when combined with anti-PD-L1 or anti-CD28 antibodies. In vivo, ALPN-202 demonstrated activity superior to PD-L1 blockade alone, while coadministration with either anti-CD28 or anti-PD-L1 prevented ALPN-202-mediated conditional CD28 costimulation resulting in reduced anti-tumor activity and RNA signatures consistent with lower T cell infiltration and activation (Figures 3 and 4).

**Conclusions**

The CD80 IgV domain utilizes separate, non-competing epitopes to bind CD28 and PD-L1. Exploitation of this attribute via directed evolution-based discovery has yielded ALPN-202, a variant CD80 vIgD-Fc fusion protein capable of simultaneously engaging PD-L1 on tumor cells and CD28 or CTLA-4 on T cells, conferring the unique abilities to block both the PD-L1 and CTLA-4 checkpoints as well as eliciting conditional CD28 costimulation only in the presence of PD-L1. The significantly superior activity of ALPN-202 over CPI-only therapies in vitro and in vivo suggests that conditional CD28 costimulation, together with checkpoint inhibition, may result in meaningfully improved clinical anti-tumor responses.

**Ethics Approval**

Animal experiments and euthanasia protocols performed in this study were approved by the Alpine Immune Sciences Institutional Animal Care and Use Committee (IACUC protocol IR 17-01)

**Fig. 1 (abstract P793).**
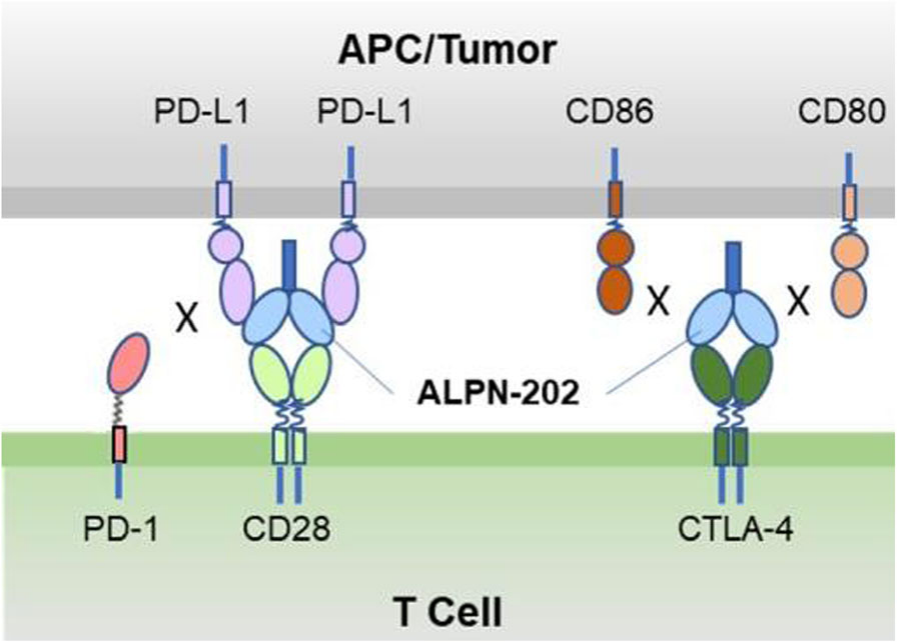
Schematic of Proposed ALPN-202 Mechanisms of Action

**Fig. 2 (abstract P793).**
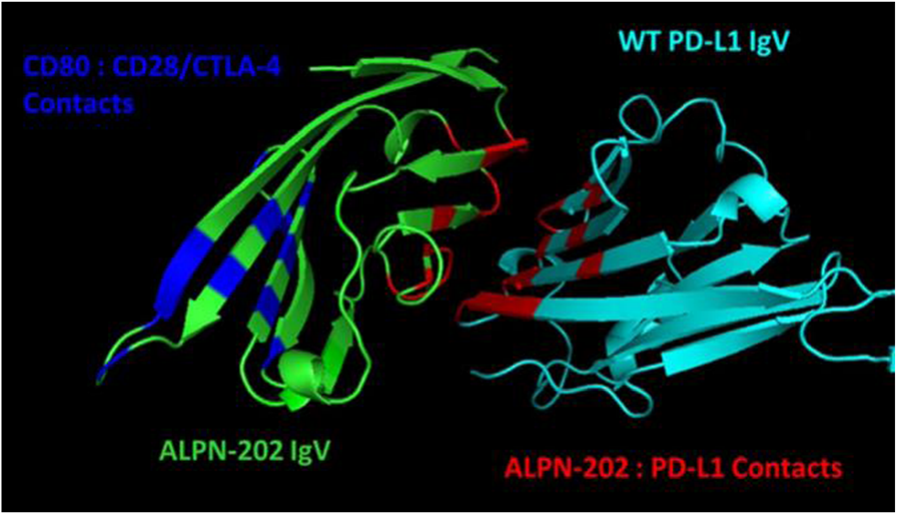
Crystal Structure of ALPN-202 IgV Bound to PD-L1 IgV

**Fig. 3 (abstract P793).**
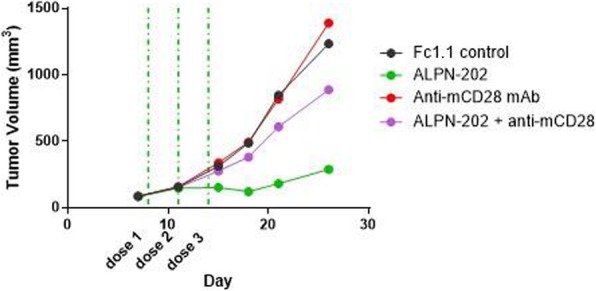
Anti-CD28 Reduces ALPN-202-Mediated Anti-Tumor Activity

**Fig. 4 (abstract P793).**
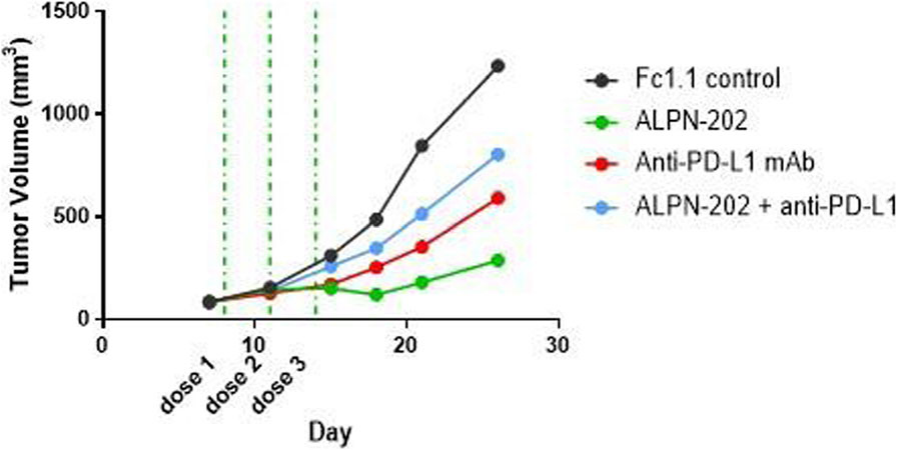
Anti-PD-L1 Reduces ALPN-202-Mediated Anti-Tumor Activity

#### P794 A novel fully synthetic dual targeted EphA2/4-1BB Bicycle® peptide induces tumor localized 4-1BB agonism

##### Sailaja Battula, PhD , Punit Upadhyaya, Marianna Kleyman, Jessica Kublin, Eric Haines, PhD, Jun Ma, liz Repash, Tom Stephen, Julia Kristensson, Liuhong Chen, Kristen Hurov, Johanna lahdenranta, Kevin McDonnell, PhD, Nicholas Keen

###### Bicycle, Lexington, MA, United States

####### **Correspondence:** Sailaja Battula (sailaja.battula@bicycletx.com)

**Background**

4-1BB (CD137) is a member of TNF superfamily involved in the activation of several immune cells including T cells and NK cells. Agonism of this receptor is a promising immunotherapeutic approach as there is ample evidence suggesting that the systemic administration of agonistic anti–4-1BB antibodies is effective pre-clinically [1] however it’s success in clinical trials is limited due to hepatotoxicity and Fcγ receptor–mediated hyperclustering. Bicycles® represent a new therapeutic modality - fully synthetic, constrained bicyclic peptides. We hypothesized that Bicycle 4-1BB agonists with rapid clearance, minimal liver exposure, and no Fc receptor interaction may induce 4-1BB mediated anti-tumor activity while avoiding liver toxicity. Furthermore, with an optimized 4-1BB-binding Bicycle in-hand, we are now developing a new set of tumor-specific immune cell agonist therapies to obtain potent activation of immune cells exclusively at the tumor site. The modular nature of Bicycle® platform allowed us to rapidly generate fully synthetic dual targeting Bicycles linking immune-agonising molecules to a tumor target such as Erythropoietin-producing hepatocellular A2 receptor (EphA2). EphA2 overexpression is observed in human cancers and correlates with tumor progression. Here, we present substantial preclinical data demonstrating the potent immunomodulatory activity of EphA2/4-1BB targeting Bicycles.

**Methods**

Tumor targeting EphA2/4-1BB Bicycles are chemically synthesized using our proprietary Bicycle platform and the molecules are tested for activity in various in vitro assays and relevant animal models.

**Results**

EphA2/4-1BB Bicycles engage EphA2 and 4-1BB simultaneously with nanomolar potency. EphA2/4-1BB Bicycles showed enhanced 4-1BB reporter signaling in co-culture assays consisting of cancer cell lines endogenously expressing EphA2 and 4-1BB Jurkat NF-kB/luc reporter cells. Moreover, these dual targeting Bicycles also potentiate cytokine secretion by human primary immune cells when co-cultured with EphA2-expressing cells. In addition, EphA2/4-1BB molecules demonstrate increased immune cell mediated cancer cell killing in T cell mediated cell killing assays. The agonistic activity of these molecules is fully dependent on tumor target expression, as such activity is absent in co-culture with target-null cell lines. In vivo testing of EphA2/4-1BB Bicycles in PBMC-humanized mice bearing HT29 xenografts showed an increased proportion of CD8+ T cells in tumor tissue but not in the circulation, suggesting a local tumor target specific activation of T cells without systemic 4-1BB agonism.

**Conclusions**

The unique ability of EphA2/4-1BB dual targeting Bicycles to precisely and potently activate immune cells in tumors without systemic immune activation is very promising and provides us a rationale for developing first-in-class Bicycles to target EphA2+ cancers.

**References**

1. Melero I, Shuford WW, Newby SA, Aruffo A, Ledbetter JA, Hellström KE, Mittler RS, Chen L. Monoclonal antibodies against the 4-1BB T-cell activation molecule eradicate established tumors. Nat Med. 1997; 3(6): 682-5

**Ethics Approval**

In vivo study work was approved by Jackson Laboratories' IACUC; Approval number :P569-4-A19-R1

Primary immune cells were purchased from approved vendors that follow stringent IRB and Ethics Committee compliance

#### P795 Anti-MUC1 monoclonal antibodies derived from the first non-viral prophylactic cancer vaccine clinical trial can target cancer cells and facilitate their immune-mediated elimination

##### Michelle McKeague, PhD, Jason Lohmueller, PhD, Eric Ricci, William Lu, Olivera Finn, PhD

###### University of Pittsburgh, Pittsburgh, PA, United States

####### **Correspondence:** Olivera Finn (ojfinn@pitt.edu)

**Background**

Monoclonal antibody therapies have transformed cancer treatment. These antibodies can be broadly characterized into two categories, immune modulating and direct targeting antibodies. Immune modulating antibodies modify immune cell function regardless of tumor antigen expression while direct targeting antibodies bind directly to antigens on tumor cells and mediate their anti-tumor functions through inhibiting signaling, targeting toxins or by additionally interacting with immune cells or complement through their Fc domains. A promising candidate for direct targeting antibodies for cancer therapy is Mucin-1 or MUC1, a protein over-expressed and hypoglycosylated on many epithelial tumors compared to normal cells. Hypoglycosylated MUC1 peptide was used in the first non-viral prophylactic cancer vaccine trial in healthy individuals at risk for colon cancer. A dozen antibodies were cloned from one individual who had a strong IgG response to the vaccine [1]. Importantly, in all patients who made antibodies to the vaccine, there have not been adverse events in >8 years, increasing the likelihood that these agents could be safely used as passive antibody therapies in patients that have cancer.

**Methods**

Each antibody was tested for its ability to bind to multiple different MUC1+ cancer cells and to interact in co-cultures with NK cells, macrophages, and complement to mediate antibody-dependent cellular cytotoxicity (ADCC), antibody-dependent cellular phagocytosis (ADCP) and/or complement-dependent cytotoxicity (CDC). Because MUC1 contains a variable number of tandem repeat (VNTR) domain containing 20-120 repeats, multiple antibodies can bind to a single MUC1 molecule. Different MUC1 constructs were tested of varying repeat lengths. Additionally, some constructs kept the extracellular domain tethered to the membrane, unable to be shed from the cell surface, as is the case for several endogenous mucins.

**Results**

Several of the antibodies could mediate ADCC and ADCP, although none of the antibodies mediated an appreciable amount of CDC. In contrast to published studies of anti-CD20 (rituximab) and anti-CD52 (alemtuzumab) the amount of antibody binding did not always correlate with increased antibody effector function.

**Conclusions**

Fully human MUC1 IgG1 antibodies generated in healthy individuals at risk for cancer were capable of mediating immune effector functions such as ADCC and ADCP and are candidates for further testing as human therapeutics. Because the amount of antibody binding did not always correlate with increased functionality, these results suggest properties other than antigen density control efficiency of direct targeting antibody tumor cell elimination and are currently under investigation.

**Acknowledgements**

Supported by grants from the NIH (T32 CA82084, F32 CA236457, R35 CA210039) and the American Cancer Society (PF LIB 125429)

**References**

1. Lohmueller JJ, Sato S, Popova L, et al. Antibodies elicited by the first non-viral prophylactic cancer vaccine show tumor-specificity and immunotherapeutic potential. Sci Rep. 2016;6:31740.

#### P797 Novel dual A2A/A2B receptor antagonist reverses adenosine-mediated immune suppression - in vitro and in vivo characterization

##### Michał Gałęzowski, Paulina Węgrzyn, Aneta Bobowska, Katarzyna Dziedzic, Magdalena Bońkowska, Karolina Grycuk, Joanna Szeremeta-Spisak, Marcin Nowogródzki, Grzegorz Satała, Alicja Obara, Iwona Łozińska-Raj, Przemysław Wyrębek, Marcelina Dudek, Anita Janiga, Jacek Reus, Marek Wronowski, Magdalena Zastawna, Grzegorz Statkiewicz, Maciej Rogacki, Mateusz Świrski, Jakub Woyciechowski, Kinga Michalik, Agnieszka Adamus, Karolina Wiatrowska, Natalia Literska, Aniela Gołas, Olga Haberkiewicz, Luigi Stasi, Peter Littlewood, Krzysztof Brzózka, Mateusz Nowak

###### Selvita, Kraków, Poland

####### **Correspondence:** Mateusz Nowak (mateusz.nowak@selvita.com)

**Background**

The immunosuppressive role of adenosine attracts attention of many researchers as an interesting target for anticancer therapy. High concentration of adenosine detected in many types of tumors affect different subpopulations of infiltrating immune cells e.g. T lymphocytes, NK cells, dendritic cells and macrophages. Adenosine acts on the immune cells mainly via A2A or A2B receptors and maximal therapeutic effect can be achieved by dual antagonism to both receptors. Here we present the data for equipotent dual A2A/A2B antagonists that exert nanomolar potency in tumor-like adenosine-rich environment.

**Methods**

We are demonstrating how our inhibitors restore adenosine depleted immune functionality in the series of functional in vitro assays in primary cells. This includes the cytokine release by activated CD4+ and CD8+ human T-lymphocytes (driven by A2A) and dendritic cells (driven by A2B). Additionally we have observed effective inhibition of surrogate PD biomarker phospho-CREB (cAMP response element-binding protein) tested in human whole blood assay. Murine syngeneic models are used to demonstrate antitumor efficacy.

**Results**

By head-to-head comparison of inhouse developed dual A2A/A2B antagonists to compounds currently pursued in clinical development we have demonstrated the best-in-class characteristics of our molecules. Our compounds maintain sub- or low nanomolar antagonistic potency in a high adenosine conditions on both A2A and A2B receptor. Multilevel pharmacological characterization spans whole range from receptor binding and cAMP accumulation assay in cell lines overexpressing target receptors to functional assays in primary human immune cells demonstrating the reversal of immune suppression induced by adenosine. Our compounds showed superior activity in antagonizing adenosine-mediated immunosuppression in T-cells (cytokines IL2 and TNFα in CD4+ and CD8+), NK cells (cytotoxicity against target cancer cells), moDCs (cytokines IL12 and TNFα, effect driven specifically by A2B receptors) and macrophages (suppression of adenosine induced VEGF release by M2). High activity of our compounds in human whole blood assay demonstrates that low nanomolar concentration is sufficient to fully antagonize adenosine. Therapeutic efficacy in combination with checkpoint inhibitors has been confirmed in syngeneic in vivo mouse models.

**Conclusions**

New class of dual A2A/A2B adenosine receptor antagonists combines two crucial activities which addresses distinct, cell type mediated mode of action of adenosine related immunosupression. The dual A2A/A2B antagonism profile as well as the ability to antagonize both receptors at very high adenosine conditions are key features differentiating current series from other adenosine receptor antagonist in clinical development.

**Acknowledgements**

Project supported by the European Regional Development Funds under the Measure 1.1.1. Operational Program - Smart Growth (POIR.01.01.01-00- 0987/16-00)

#### P798 Think globally act locally: Biodistribution of BETA-PRIME, a replication-competent type 5 adenovirus enhanced with a trap for the immunosuppressive cytokine, TGF-Beta

##### Christopher Larson, MD PhD , Bryan Oronsky, MD PhD, Tony Reid, MD PhD

###### EpicentRx, Inc., La Jolla, CA, United States

####### **Correspondence:** Christopher Larson (clarson@epicentrx.com)

**Background**

Transgene-enhanced oncolytic adenoviruses (OVs) represent a promising novel therapeutic option for the treatment of cancer especially for patients with chemotherapy- and immunotherapy-resistant, advanced, aggressive, and systemic disease. The precise mechanisms by which intratumorally injected OVs trigger an immune response and induce an abscopal effect on distant lesions remain undefined. BETA-PRIME, a type 5 adenovirus, which carries a TGF-beta trap transgene that binds to and neutralizes the immunosuppressive cytokine, TGF-beta, has been shown in an immunocompetent mouse model to eradicate both locally injected and non-injected tumors. Single dose biodistribution of the TGF-beta trap transgene was evaluated in tumor bearing mice, providing a possible explanation for its systemic activity.

**Methods**

In 129S1 immunocompetent mice bearing ADS-12 tumors (mouse lung carcinoma), BETA-PRIME was injected intratumorally and intravenously at the following dose levels in groups of 25 mice each (Table 1)

Group Dosed Article Route Dose Level (per animal) Term

Group 1 None - 0 Untreated

Group 2 Vehicle IT Vehicle 0.1mL IT Vehicle

Group 3 Vehicle IV Vehicle 0.2mL IV Vehicle (high volume)

Group 4 BETA-PRIME IT 5E8 VP /0.1 m IT Low Dose

Group 5 BETA-PRIME IT 5E9 VP /0.1 mL IT Medium Dose

Group 6 BETA-PRIME IT 5E10 VP /0.1 mL IT High Dose

Group 7 BETA-PRIME IV 1E11 VP /0.2 mL IV High Dose

Group 8 Vehicle IV 0.1 mL IV Vehicle (low volume)

Group 9 BETA-PRIME IV 5E9 VP /0.1 mL IV Low Dose

Soluble TGF-beta trap was detected in the serum using ELISA

**Results**

A single viral injection resulted in long-term TGF-beta trap expression of over a week in the serum from all doses of intravenous and intratumoral groups while in vehicle treated mice TGF-beta trap was not detected. In the high dose IV group the TGF-beta trap was detected at concentrations of 210-585 ng/ml while in high-dose intratumoral mice circulating TGFb trap was detected at concentrations of 4-13 ng/ml.

**Conclusions**

Despite local injection of BETA PRIME, systemic levels of the TGF-beta trap transgene were observed, which demonstrates that the virus follows the “think globally act locally” dictum and may in part account for evidence of regression of non-injected, distant tumor lesions.

**Table 1 (abstract P798).**
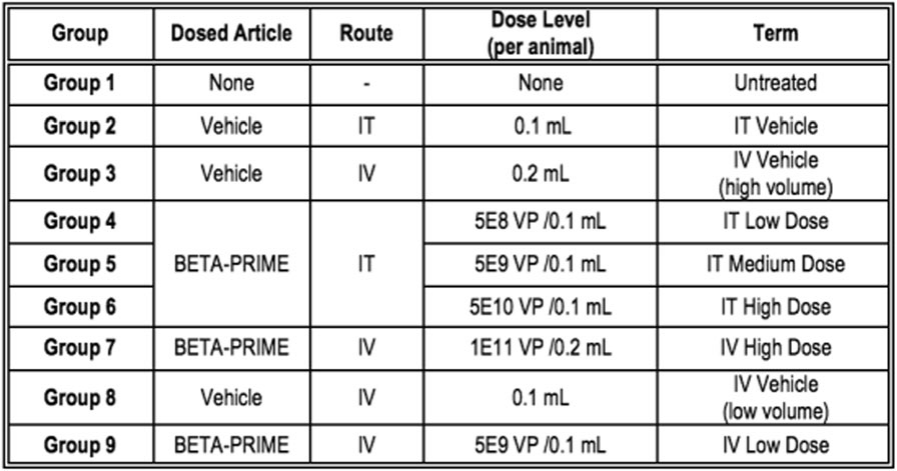
See text for description

#### P799 Innate leukocyte chemoattractant chemerin is an intrinsic tumor suppressor in prostate cancer

##### Zhongping Xu, PhD^1^, Woo Jae Shin, BA^1^, Kevin Kim^1^, Brian Zabel, PhD^2^, Russell Pachynski, MD^1^

###### ^1^Washington University School of Medicine,, St Louis, MO, United States; ^2^Stanford University, Palo Alto, CA, United States

####### **Correspondence:** Russell Pachynski (rkpachynski@wustl.edu)

**Background**

Chemerin (RARRES2) is an innate leukocyte chemoattractant known to recruit cells that express its chemotactic receptor (CMKLR1) along its concentration gradient. Chemerin downregulation has been shown in several tumor types, but not specifically investigated in prostate cancer. Our previous work has shown that augmented chemerin in the tumor microenvironment can suppress tumor growth by recruitment of immune effector cells. Here, we show in preclinical models of immune competent prostate cancer that chemerin can act as a potent tumor suppressor, and may play a role in immunosurveillance during tumorigenesis.

**Methods**

Transgenic Adenocarcinoma of the Mouse Prostate (TRAMP) C1 and C2 cells were lentivirally transduced with murine RARRES2. Monoclonal cell populations were obtained by limiting dilution. Constitutive chemerin secretion was evaluated by ELISA, and functional chemotaxis assays used to confirm the ability of secreted chemerin to recruit CMKLR1+ immune cells. In vitro cell proliferation and surface expression of markers (eg MHC, PDL1) were examined on control and chemerin-expressing tumor lines. Control or chemerin-expressing TRAMP lines were inoculated subcutaneously into male C57BL/6 mice. Tumor growth was serially assessed. To study the effect of chemerin/CMKLR1 on immunosurveillance of spontaneous tumorigenesis, B6 TRAMP WT mice were crossed with CMKLR1 knockout (KO) or RARRES2 KO mice.

**Results**

No significant difference in in vitro cell proliferation, nor differences in expression of CMKLR1, MHC class I, CD-1d, CD44, or PD-L1 levels was found between control or chemerin-expressing lines. Tumors were palpable (~50 mm3) in TRAMP C1 control after 4 weeks after inoculation, and tumors reached ~1000 mm3 at 8 weeks. Tumor growth was significant delayed in the chemerin-expressing TRAMP C1 cohort (< 0.04).

**Conclusions**

Here we show for the first time that modulation of chemerin, an innate leukocyte chemoattractant, can result in significant prostate tumor suppression using an immune competent preclinical model. Deficiency of chemerin/CMKLR1 in TRAMP transgenic mice leads to significant increase in prostate tumor growth, suggesting a role for immune surveillance. Profiling of tumor infiltrating leukocytes, immune subset depletion studies, and gene expression profiling is ongoing.

**Ethics Approval**

The study was approved by Washington University's IACUC institutional review board, approval number 20170174.

#### P800 Development of PY314, a monoclonal antibody that selectively depletes tumor-associated macrophages, for the treatment of solid tumors

##### Venkataramna Sriram, PhD, Mikhail Binnewies, Joshua Pollack, Erick Lu, Nadine Jahchan, Xiaoyan Du, Aritra Pal, Evan Greger, Kevin Baker, Michel Streuli, PhD , Len Reyno

###### Pionyr Immunotherapeutics, South San Francisco, CA, United States

####### **Correspondence:** Michel Streuli (michel.streuli@pionyrtx.com)

**Background**

The proportion of patients who benefit from checkpoint inhibitor (CPI) therapy is modest, and additional immune pathways need to be targeted to improve overall patient outcomes. The abundance of immune-suppressive, tumor-associated macrophages (TAMs) is thought to be a key CPI resistance mechanism. To target TAMs, we developed monoclonal antibodies (mAbs) that specifically bind the cell surface protein Triggering Receptor Expressed on Myeloid Cells 2 (TREM2).

**Methods**

We developed a lead anti-TREM2 mAb, termed PY314, that was designed to deplete TREM2-positive TAMs. PY314 binding to TREM2 was evaluated using surface plasmon resonance and cell binding assays. In vivo efficacy and pharmacodynamic studies were conducted in a variety of syngeneic mouse tumor models. Cellular and molecular characterization of post-treatment tumor immune infiltrates was performed using flow cytometry and RNA sequencing.

**Results**

The PY314 mAb binds with high affinity to human, cynomolgus and mouse TREM2. Administration of PY314m, a murinized version of PY314, to tumor-bearing mice resulted in the reduction of TAMs in a variety of mouse syngeneic tumor models, including the CT26 and EMT6 models. More specifically, PY314m depleted MHC class II-low, M2-like TAMs, which are pro-tumorigenic. In EMT6 tumors, PY314m single agent treatment significantly shifted the TAMs within the TME from a M2-like phenotype to an inflammatory M1-like phenotype, and treatment resulted in a 40% cure rate. PY314m treatment in combination with an anti-PD-1 mAb in mice harboring CT26 tumors, that are otherwise resistant to anti-PD-1 treatment, resulted in a ~40% cure rate. Analysis of intratumor immune cell infiltrates suggested that TAM modulation resulted in enhanced functional activity of T cells within the TME. These results demonstrate that PY314 in combination with anti-PD-1 can convert CPI-resistant tumors into treatment responsive tumors. Furthermore, tumor rechallenge studies using animals cured of their tumors demonstrated that the PY314m/anti-PD-1 mAb combination treatment resulted in secondary tumor rejection, suggesting the development of long-term immunological memory. In pilot, four-week, multi-dose mouse and non-human primate toxicokinetic studies, PY314 was generally well tolerated at all tested doses.

**Conclusions**

Collectively, the available preclinical and nonclinical data support PY314 immunotherapy, alone or in combination with a CPI, in cancer patients who are resistant or refractory to CPI therapies, to improve both the overall response rates as well as the durability of responses. First in human clinical testing will start in 2020.

#### P801 Development of the first in class immunotherapy targeting immuno-suppressive δ1 containing γδ T cells for the treatment of pancreatic ductal adenocarcinoma and other solid tumors

##### Tanya Panchenko, PhD^1^, Wei Wang^1^, Eric Denbaum^1^, Takamitsu Hattori^1^, Akiko Koide, PhD^1^, Aleksandra Filipovic, MD, PhD^2^, George Miller^1^, Shohei Koide^1^

###### ^1^NYU Langone, New York, NY, United States; ^2^PureTech Health, London, United Kingdom

####### **Correspondence:** Shohei Koide (Shohei.Koide@nyulangone.org)

**Background**

Targeting and engineering γδ T cells has recently emerged as an orthogonal therapeutic approach in oncology with capacity to modulate both innate and adaptive immune properties. In solid tumors such as pancreatic ductal adenocarcinoma (PDA), melanoma, glioblastoma, ovarian, and breast cancer, γδ1 T cells express immunosuppression-related molecules and possess a pro-tumorigenic capacity. We have shown that intra-tumoral γδ T cells from PDA, colorectal cancer (CRC) and hepatocellular carcinoma (HCC) potently suppress patients’ αβ T cells. To harness the therapeutic potential of γδ1 T cell blockade, we developed highly specific, fully human antibodies against δ1-subset of γδT cell receptor (γδ1-TCR).

**Methods**

We determined the amino acid sequences of tumor specific δ1-TCR chains from primary PDA, CRC and gastric cancer samples. Multiple γδ-TCR proteins were produced and used to screen a proprietary synthetic, human antibody library using phage display. Surface plasmon resonance and bead-based assays were used to measure binding affinity and specificity. Affinity maturation was performed to improve cross-reactivity to monkey γδ1-TCR. Cell based assays were used to evaluate antibody-dependent cell cytotoxicity and phagocytosis (ADCC and ADCP). The levels of γδ1-T cell infiltration was measured in patient tumors. Efficacy in reversing immunesuppression was assayed using patient-derived organotypic tumor spheroids (PDOTS, n = 32), which recapitulate complex tumor architecture.

**Results**

Because the tumor-derived δ1 chains showed diverse CDR3 sequences, we developed antibodies that bind diverse δ1 TCRs. Our first-in-class anti-δ1 antibodies had low nanomolar affinity to δ1 TCRs and showed no binding to δ2 TCRs. Our lead clinical candidate showed no preference for the γ chains of the TCR, which enables it to target diverse set of γδ1-TCRs. It had equivalent affinity for the human and cynomolgus monkey γδ1-TCRs and was potent in mediating cell based ADCC and ADCP. We showed that patient tumors can have a high δ1 T cell infiltration (up to 40% of the total T cell infiltrate). Our lead candidate achieved reproducible and robust efficacy in the up-regulation of pro-inflammatory T cell activation markers in PDOTS from a diverse set of gastrointestinal tumors. Furthermore, γδ knockout mice had an improved response to checkpoint inhibitors, anti-PD1 and anti-CTLA4, in melanoma and lung cancer models.

**Conclusions**

We have defined a novel therapeutic immuno-oncology strategy and translated it to develop a lead clinical candidate anti-δ1 monoclonal antibody. Our efficacious, novel immunotherapy has the potential to be transformative for the treatment of cancers where γδ1 T cells drive a pro-tumorigenic, immunosuppressive environment.

#### P802 Single cell immuno- and cancer marker profiling of non-small cell lung cancer tissue: Checkpoint marker expression on CD103+, CD4+ T-cells correlates with circulating tumor enumeration

##### Kaylan Handique, PhD^1^, Bruce Patterson, BS, MD^2^, Lianne McLean, BA^1^ , Will Chow, PhD^1^, Priya Gogoi, PhD^1^, Vishal Sharma, PhD^1^

###### ^1^Celsee, Inc., Ann Arbor, MI, United States; ^*2*^*IncellDx, San Francisco, CA, United States*

####### **Correspondence:** Lianne McLean (lianne.mclean@celsee.com)

**Background**

~40% of newly diagnosed lung cancer patients have tumors that have metastasized and non-small cell lung cancer (NSCLC) has a poor prognosis. Advances in treatment, such as targeted therapy and immunotherapy have significantly improved NSCLC patient outcome. In a collaborative study, between Celsee, IncellDx, Cytek Biosciences, and Qognit, single-cell immune and cancer marker profiling of primary tumor cells was employed to investigate possible signatures that might predict the presence or absence of circulating tumor cells (CTCs). This low- cost screening tool could be used to select patients for ongoing CTC monitoring for disease progression and response to therapy. A comprehensive study on 10 NSCLC was performed on patient tissue samples with paired blood samples.

**Methods**

Solid tissue biopsy samples were dissociated into single cells and stained with a total 26 immune, and cancer markers (aneuploidy) that were then analyzed with high-parameter flow cytometry. A total of 72 pairwise comparisons were assessed as biomarkers for their correlation with CTC number. Strong correlations were observed between CTC number and the frequency of PD-1 expressing lymphocytes. This was evident with the CD103+CD4+ T lymphocytes which are indicative of T- regulatory cells that are proximal to the tumor. (The PD-1/PD-L1 pathway is a target for NSCLC immunotherapy.) As part of the study, the lower limits of the reproducible detection (LOD) of the Genesis System was evaluated. 5 PD-L1+ CTCs were reproducibly detected in 4 mL of whole blood. This represents a capture rate as low as 1 in a 1,000,000 cells, which is much higher than the 50-60% capture rate observed with legacy systems.

**Results**

Correlations in CTC number and the frequency of immune PD-1+ lymphocytes with the PD-1+CD103+CD4+ T lymphocytes which are a biomarker for T- regulation cells, are proximal to the tumor. CTC number is correlated with the frequency of PD-L1+ cancer cells and cancer DNA markers. CTC number inversely correlated to the frequency of CD44+E-cadherin- cancer cells. Unsupervised clustering analysis based on the biomarker analysis separated the CTC negative patients from the CTC positive patients.

**Conclusions**

Profiling multiple immune and cancer markers on cancer samples with multi- parametric flow cytometry yielded protein expression information at the single cell level. Clustering analysis of the proteomic data revealed a signature driven by PD-1 expression on CD103+CD4+ T cells that was predictive of the presence of CTCs. High efficacy of cell capture and accuracy of the Genesis System make it ideal for CTC enumeration and monitoring (Figures 1-6).

**Acknowledgements**

Xiaoyang Wang1, Pin-I Chen1, Maria Jaimes2, Huimin Gu2, Keith Shults1, Santosh Putta3, Fariba Fazeli1, Janine Fernandez1, Vishal Sharma4, Will Chow4, Priya Gogoi4, Kalyan Handique4 and Bruce K Patterson1. 1 IncellDx Inc, San Carlos, CA 2 Cytek Biosciences Inc, Fremont, CA. 3 Qognit Inc, San Carlos, CA 4 Celsee Inc, Ann Arbor MI

**References**

Xiaoyang Wang1, Pin-I Chen1, Maria Jaimes2, Huimin Gu2, Keith Shults1, Santosh Putta3, Fariba Fazeli1, Janine Fernandez1, Vishal Sharma4, Will Chow4, Priya Gogoi4, Kalyan Handique4 and Bruce K Patterson1. 1 IncellDx Inc, San Carlos, CA 2 Cytek Biosciences Inc, Fremont, CA. 3 Qognit Inc, San Carlos, CA 4 Celsee Inc, Ann Arbor MI

**Fig. 1 (abstract P802).**
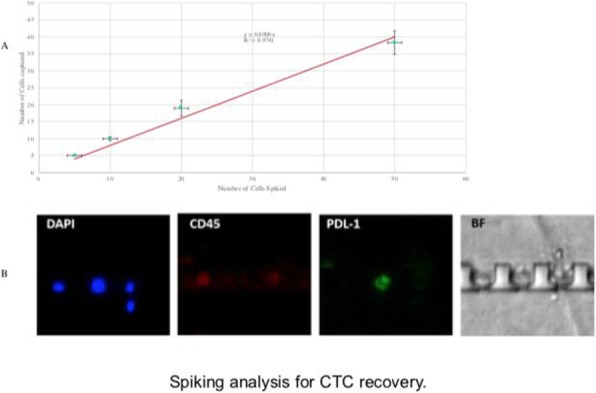
Single-Cell Immune and Cancer Marker Profiling

**Fig. 2 (abstract P802).**
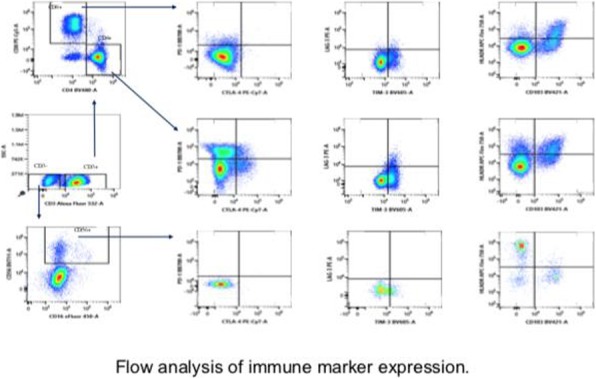
Single-Cell Immune and Cancer Marker Profiling

**Fig. 3 (abstract P802).**
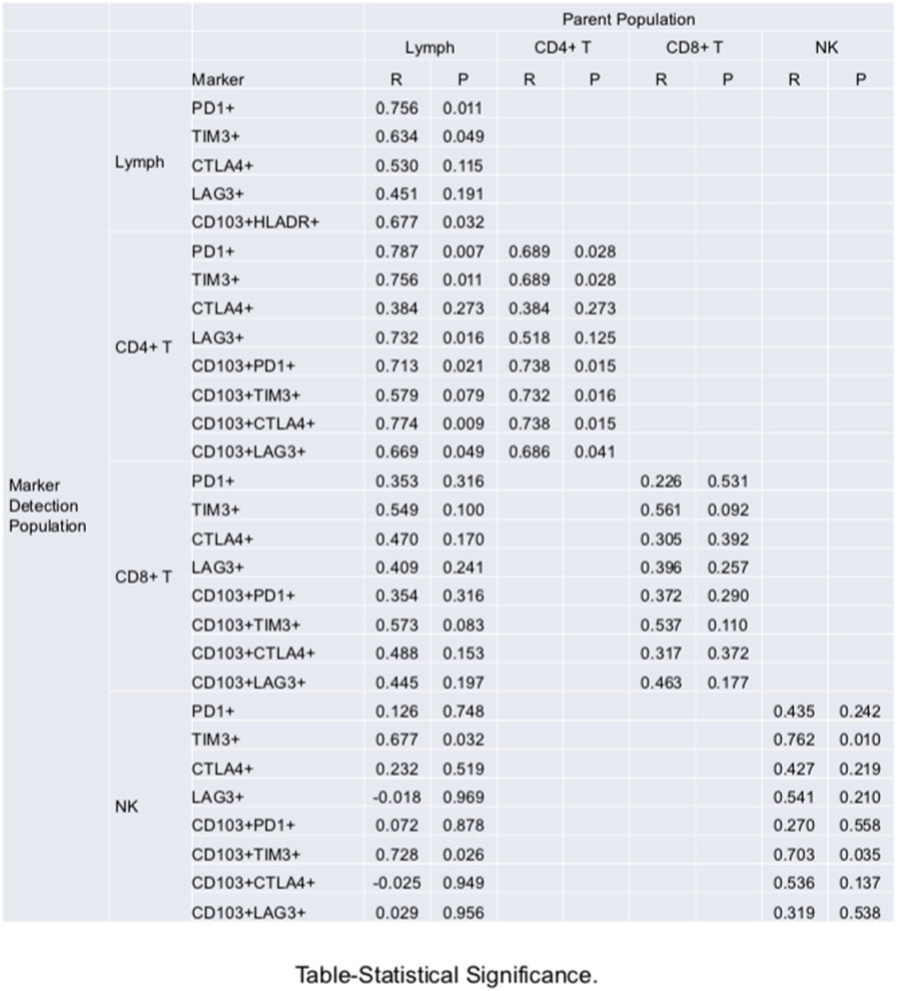
Single-Cell Immune and Cancer Marker Profiling

**Fig. 4 (abstract P802).**
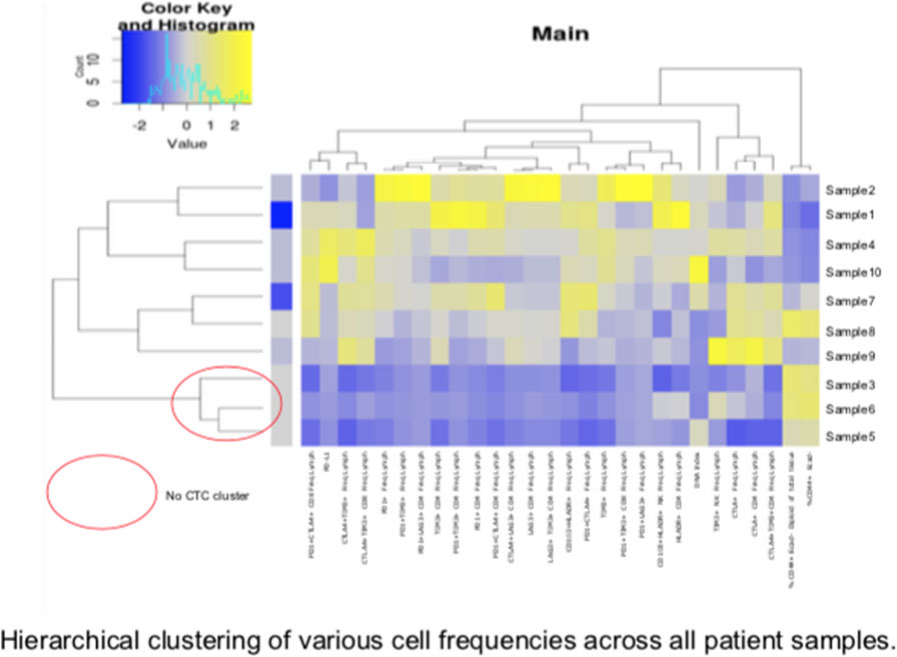
Single-Cell Immune and Cancer Marker Profiling

**Fig. 5 (abstract P802).**
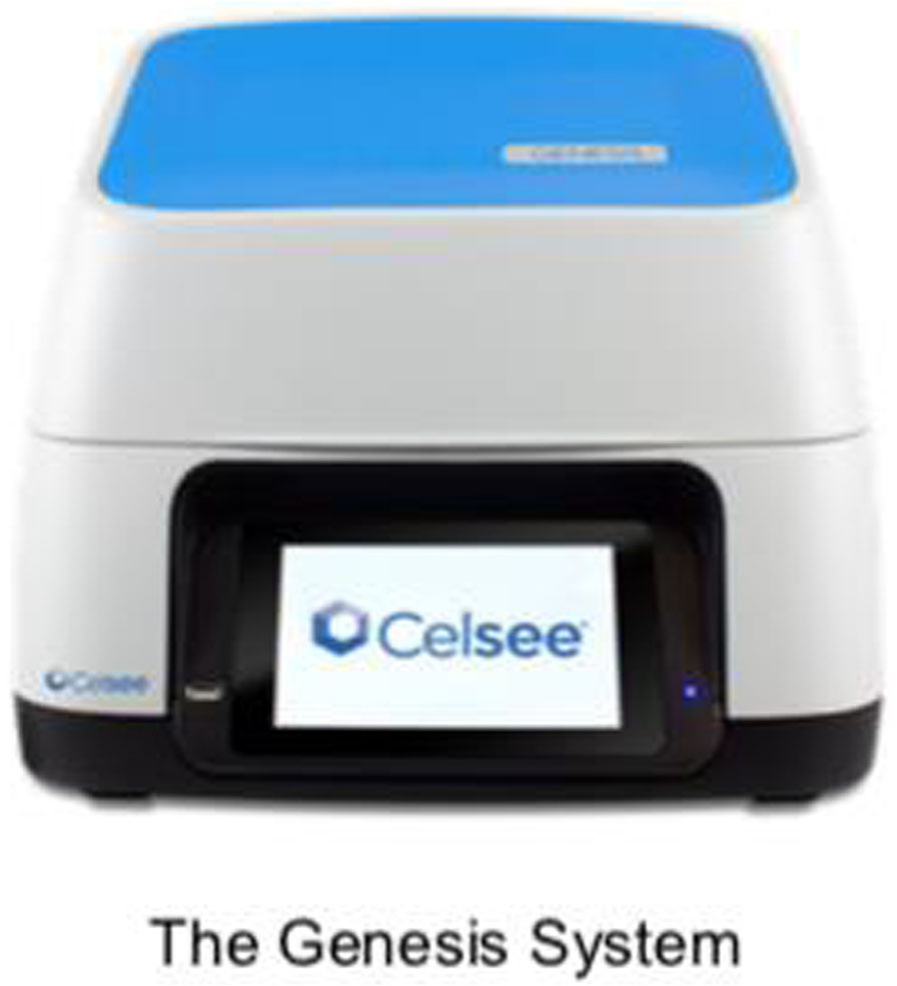
Single-Cell Immune and Cancer Marker Profiling

**Fig. 6 (abstract P802).**
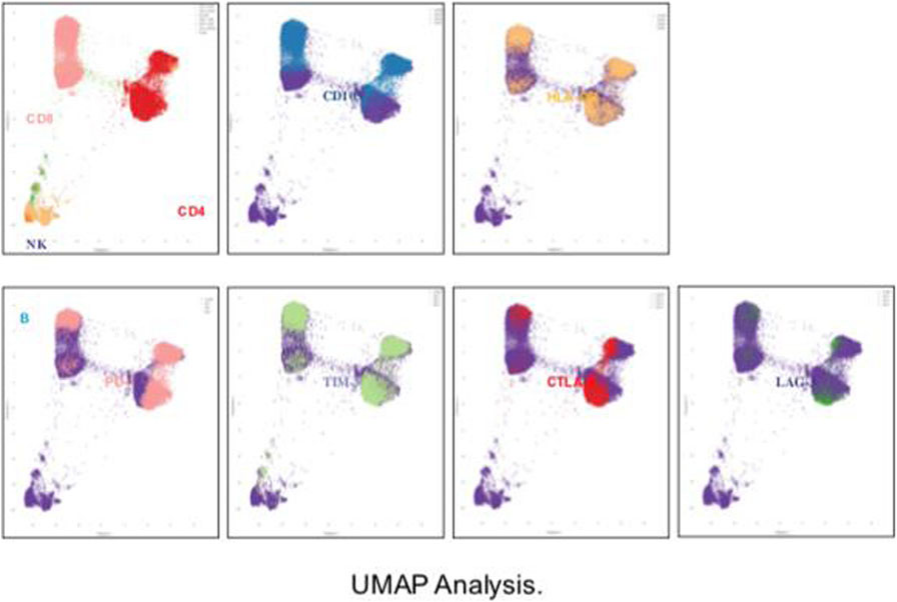
Single-Cell Immune and Cancer Marker Profiling

#### P803 CUE-101, a novel HPV16 E7:pMHC:IL-2:Fc fusion protein, enhances tumor antigen specific T cell activation for the treatment of HPV16-driven malignancies

##### Steven Quayle, PhD^1^ , Natasha Girgis, PhD^1^, Dharma Raj Thapa^1^, Zohra Merazga, MS^1^, Miguel Moreta^1^, Sandrine Hulot, PhD^1^, Alyssa Nelson, BS^1^, Lauren Kraemer, BS^1^, Dominic Beal, PhD^1^, Mark Haydock, BS^1^, Luke Witt, BS^1^, Jessica Ryabin, BS^1^, Jonathan Soriano, MSc^1^, Emily Spaulding, PhD^1^, John Ross, PhD^1^, Rodolfo Chaparro, PhD^1^, Ronald Seidel, PhD^1^, Anish Suri, PhD^1^, Saso Cemerski^1^, Kenneth Pienta, MD^2^, Mary Simcox, PhD^1^

###### ^1^Cue Biopharma, Inc, Cambridge, MA, United States; ^2^Johns Hopkins School of Medicine, Baltimore, MD, United States

####### **Correspondence:** Steven Quayle (squayle@cuebio.com)

**Background**

Human papilloma virus (HPV)-driven cancers cause significant morbidity and mortality worldwide. CUE-101 is a novel therapeutic fusion protein designed to selectively activate tumor antigen-specific (AgS) T cells to treat HPV16-driven cancers. CUE-101 is comprised of two human leukocyte antigen (HLA) molecules presenting an HPV16 E7 peptide, four affinity-attenuated human interleukin-2 (IL-2) molecules, and an effector attenuated human immunoglobulin G (IgG1) Fc domain.

**Methods**

Human E7-specific T cells (Astarte Biologics, Bothell, WA) and human PBMCs were tested to demonstrate cellular activity and specificity of CUE-101, while in vivo activity of CUE-101 was assessed in HLA-A2 transgenic mice. Anti-tumor efficacy with a murine surrogate (mCUE-101) was tested in the TC-1 syngeneic tumor model. Tetramer staining and flow cytometry identified cell populations and activation markers.

**Results**

The mutated IL-2 domains of CUE-101 exhibit reduced functional activity relative to wildtype IL-2. In AgS T cells, CUE-101 selectively binds and induces receptor signaling and concentration-dependent IFNγ secretion. From E7(11-20)-responsive PBMCs, CUE-101 selectively expands AgS CD8+ T cells in a concentration-dependent manner with minimal expansion of regulatory T cells. The AgS T cells expanded by CUE-101 treatment are polyfunctional cytotoxic T lymphocytes as shown by production of IFNγ, TNF-alpha, and cytotoxic granules in response to native peptide-loaded presenting cells. Furthermore, CUE-101 induces expansion of an oligoclonal T cell repertoire comparable to that expanded with peptide/IL-2. In vivo studies in HLA-A2 mice confirm that intravenous administration of CUE-101 elicits polyfunctional AgS cells from naïve and peptide-immunized mice without significantly altering the frequencies of other immune lineages. A CUE-101 surrogate demonstrates anti-tumor activity both alone and in combination with an anti-PD-1 antibody in the TC-1 model. Efficacy in this model was associated with expansion of E7 peptide-reactive T cells and establishment of immunologic memory as shown by tumor rejection upon rechallenge with TC-1 cells in the absence of additional dosing.

**Conclusions**

Consistent with its design, CUE-101 demonstrates selective expansion of an HPV16 E7(11-20)-specific population of cytotoxic CD8+ T cells in vitro and in vivo. These results support its potential for clinical efficacy in an ongoing Phase 1 trial (NCT03978689).

**Ethics Approval**

All animal studies were approved under SmartLabs Institutional Animal Care and Use Committee protocol MIL-100 and were performed in compliance with federal guidelines.

#### P804 In vivo efficacy of a PD-L1 targeted Engineered Toxin Body (ETB) comprised of direct cytotoxicity and T-cell mediated tumor targeting

##### Brigitte Brieschke, BS, Sara LeMar, Joseph Dekker, PhD, Garrett Cornelison, Asis Sarkar, Garrett Robinson, Jay Zhao, PhD, Aimee Iberg, PhD, Jack Higgins, PhD, Hilario Ramos, PhD, Erin Willert, PhD

###### Molecular Templates, Austin, TX, United States

####### **Correspondence:** Erin Willert (erinwillert@mtem.com)

**Background**

Engineered Toxin Bodies (ETBs) are comprised of a deimmunized Shiga-like toxin-A subunit (DI-SLTA) genetically fused to an antibody-based targeting domain. Multiple ETBs are now in clinical development. Our next evolution of the platform, antigen seeding technology (AST), retains the primary characteristics of these ETBs - self-internalization, routing to the cytosol, and enzymatic and irreversible inactivation of ribosomes by DI-SLTA - but in addition, can also deliver a viral antigen for presentation in complex with MHC-I to resident viral-specific cytotoxic T lymphocytes (CTLs). The fusion protein provides a powerful, dual mechanism of action to specifically target and destroy PD-L1 positive tumor and inhibitory immune cells.

**Methods**

In vitro activity of ETBs was measured by cell viability and kinetic imaging of labeled target cells in an effector T-cell co-culture assay. Repeat dosing studies in non-human primates (NHPs) and murine models harboring human PD-L1 positive patient derived tumors were used to evaluate tolerability and efficacy.

**Results**

ETBs targeting PD-L1 showed potent and specific activity across human tumor cell lines at picomolar concentrations in vitro. PD-L1 positive monocytes, but not PD-L1 negative lymphocytes, from human peripheral blood are targets of these ETBs. In co-culture assays, AST capable ETBs had a deeper response when target cells express both PD-L1 and the appropriate MHC-I allele in the presence than in the absence of antigen specific CTLs. In vivo, PD-L1 targeted ETBs induced tumor growth delay and regression and provided a survival benefit in multiple NSCLC PDX models with variable PD-L1 staining, as compared to controls. These ETBs were also tolerated in repeat dosing NHP studies.

**Conclusions**

The potent and direct activity of ETB mediated tumor cell destruction is demonstrated across tumor cell lines from multiple indications, on PD-L1 positive immune cells ex-vivo, and by tumor growth and survival benefits in PDX models. Potency differences observed in vitro across ETBs correlate with efficacy on low PD-L1 models. Additional in vivo models to further elucidate the potential of the exemplary AST capable ETBs are ongoing, and clinical trials are expected to initiate next year. The AST capable, PD-L1 targeted ETBs provide dual novel mechanisms to deplete PD-L1 positive cells and control malignancies. Both the DI-SLTA mediated direct cell kill and activation of viral-specific CTLs circumvents a need for high mutational burden or anti-tumor immune-cell infiltrate at the tumor site, providing a potential treatment option for patients whose disease has progressed after checkpoint therapy.

**Ethics Approval**

In vivo animal studies were approved by Champions Champions Oncology’s Institutional Animal Care and Use Committee (2017-TOS-001).

#### P805 SRF388, a first-in-class, fully human monoclonal antibody targeting IL 27, blocks the immunoregulatory effects of IL-27 in immune cells and demonstrates preclinical in vivo antitumor activity

##### Matthew Rausch, PhD , Jing Hua, Devapregasan Moodley, Kerry White, Katherine Walsh, Christine Miller, Jamie Strand, Pamela Holland, PhD, Vito Palombella, PhD, Jonathan Hill, PhD

###### Surface Oncology, Inc., Cambridge, MA, United States

####### **Correspondence:** Matthew Rausch (mrausch@surfaceoncology.com)

**Background**

IL-27, a heterodimeric member of the IL-12/IL-23 cytokine family, consists of two subunits, IL-27p28 and Epstein-Barr virus-induced gene 3 (EBI3). IL-27 signals through a heterodimeric receptor composed of glycoprotein (gp130) and IL-27 receptor subunit alpha (IL 27RA). Binding of IL-27 to its receptor induces signaling through the JAK-STAT pathway to alter immunoregulatory receptor and proinflammatory cytokine expression in immune cells, limiting the intensity and duration of T-cell responses during infection and cancer. Given the immunoregulatory function of IL-27, it was hypothesized that blockade of IL-27 signaling may represent a novel and promising strategy to treat cancer.

**Methods**

To explore the therapeutic potential of IL-27 blockade, SRF388, a first-in-class, fully human anti IL-27 monoclonal antibody that binds to the p28 subunit of IL-27 with high affinity, was developed. The ability of SRF388 to inhibit IL-27-induced phenotypic changes in human peripheral blood mononuclear cells (PBMCs) was assessed in vitro. To explore the ability of SRF388 to inhibit IL-27 signaling in vivo, mice overexpressing recombinant human IL-27 (rhIL 27) were treated with SRF388, and T cells were analyzed by flow cytometry. Finally, the antitumor activity of IL-27 blockade with SRF388 was assessed in mice.

**Results**

SRF388 prevented IL-27 from binding to IL-27RA, inhibited IL-27-induced STAT1 phosphorylation in human cell lines and primary T cells, and blocked IL-27-responsive immunoregulatory receptor expression in human PBMCs. Ectopic expression of rhIL 27 in mice promoted the expression of PD-L1, Tim-3, Tigit, and Lag-3 on murine T cells, a response that was fully inhibited by the administration of SRF388. Treatment of human PBMCs with rhIL-27 altered the expression of many of the same markers. Moreover, IL-27 counteracted the activity of PD-1 blockade in upregulating cytokine production by activated human PBMCs. Notably, SRF388 blocked all of these IL-27-mediated responses. Lastly, blockade of IL-27 signaling with SRF388 demonstrated antitumor activity in mice.

**Conclusions**

Collectively, these data demonstrate that IL-27 exerts multiple immunosuppressive effects and that SRF388, a first-in-class antibody targeting IL-27, can potentiate antitumor responses in cancers that rely on IL-27-mediated immune escape.

**Ethics Approval**

Mice were used in compliance with protocols approved by the IACUC of Mispro Biotech Services, Cambridge, MA (#2017-03-21SUR-1), Charles River Accelerator and Development Lab, Cambridge, MA (#CR-008), or Charles River Laboratories, Worcester, MA (#I023).

#### P806 Structural implications for GITR agonism in immunotherapies

##### Hamsell Alvarez, PhD , Bryan Rogers, MSc, Sarah Chan, Lance Bigelow, Samuel Cichanowicz, Marc Lake, Russell Judge, Ramesh Iyer, Kenton Longenecker

###### AbbVie, Redwood City, CA, United States

####### **Correspondence:** Hamsell Alvarez (hamsell.alvarezjares@abbvie.com)

**Background**

GITR is a member of the TNF superfamily. Multiple studies in mice have demonstrated that GITR ligation by an agonist antibody induces an enhanced anti-tumor immune response to tumors in various mouse models. In humans, the therapeutic effect of GITR agonist antibodies has been limited. The difference in bioactivity may be explained by the dimeric vs. trimeric organization of the endogenous forms of the mouse and human GITR-L, respectively. Our current study provides a structural framework for understanding how to achieve optimal GITR clustering using novel engineered GITR-L-based constructs that overcome the limited bioactivity induced by GITR antibody-based therapeutics in humans.

**Methods**

The human GITR/GITR-L complex was co-expressed, purified, crystallized, and structure solved by an initial molecular replacement solution using monomeric ELR1 as the search model (PDB: 3WVT). The resulting complex structure was used to design novel GITR-L constructs to maximize their co-stimulatory potential. Trimeric GITR-L was fused to an Fc-effector null heavy chain isotype, with increasing oligomeric organization up to a hexameric construct design. The constructs were tested in an anti-CD3 mediated human PBMC co-stimulation assay to evaluate bioactivity by testing cell viability, IL-2 and IFN-γ secretion.

**Results**

We described the first crystal structure of the human GITR/GITR-L complex at a resolution of 2.85 Å which revealed an unusual receptor-mediated non-covalent dimer of two GITR-GITR-L trimeric assemblies forming a hexagonal network. Trimeric GITR interacts with its trimeric GITR-L in the canonical cysteine-rich domain-dependent modality. Peptide mapping revealed an o-glycosylation site in the CRD1 subdomain of GITR which induced partial heterogeneity. Structure based alignment of GITR demonstrated good superposition with 4-1BB and OX40. The CRD3-mediated GITR homodimer interface contains a network of hydrophobic interactions at the c-terminus, which suggest a propensity for native oligomerization. Since free GITR-L or bivalent antibodies cannot create the optimized clustering demonstrated by the crystal structure, we sought to achieve enhanced bioactivity by engineering novel GITR-L constructs which achieve various levels of GITR clustering. T cell co-stimulation was shown to increase with higher GITR-L oligomeric organization. Superior in vitro bioactivity was observed with a hexameric GITR-L complex construct.

**Conclusions**

Our data suggests that GITR-L or bivalent antibody-mediated GITR agonism on T cells is critically dependent on the receptor’s structural conformational geometry. Guided by the crystal structure of the GITR/GITR-L, we found that optimized receptor organization induces enhanced bioactivity of human T cells in vitro. These novel engineered GITR-L moieties may represent a new strategy to design effective co-stimulatory biotherapeutics.

#### P807 Characterization of anti-tumor immune responses and effects on survival of neoadjuvant oncolytic virotherapy in spontaneous osteosarcoma

##### Kelly Makielski, DVM, MS, DACVIM^2^, Aishwarya Sathyanarayan, PhD^1^, Shruthi Naik, PhD^1^ , Jaime Modiano, VMD, PhD^2^ , Stephen Russell, MD, PhD^1^ , Michael Henson, DVM, PhD^2^, Kathleen Stuebner^2^, Flaviu Alexandru Tabaran^2^, Gerard O^2^, Andrea Eckert^2^, Donna Groschen^2^, Lauren Mills, PhD^2^, Milcah Scott, BS^2^, Aaron Sarver, PhD^2^, Michael Farrar^2^, Ingrid Cornax, PhD^2^

###### ^*1*^*Mayo Clinic, Rochester, MN, United States*; ^*2*^*University of Minnesota, Minneapolis, MN, United States*

####### **Correspondence:** Shruthi Naik(naik.shruthi@mayo.edu), Jaime Modiano (modiano@umn.edu), Stephen Russell (sjr@mayo.edu)

**Background**

There is a critical, unmet clinical need for effective therapies for osteosarcoma, a rare bone cancer that primarily affects adolescents and young adults. The rarity and heterogeneity of these cancers have hindered research in this field. Comparative oncology studies in naturally occurring osteosarcoma in companion dogs provide opportunities to advance development in a clinically realistic setting where the tumors resemble their human counterparts but where the barriers of rarity and cost are lowered. Vesicular stomatitis virus (VSV) is a rapidly replicating, robustly immunogenic oncolytic virus platform with demonstrated cytotoxicity in canine osteosarcoma cell lines. The aim of this study is to evaluate neoadjuvant VSV-IFNβ-NIS therapy for osteosarcoma using a comparative oncology approach, and characterize anti-tumor immune responses in spontaneous canine osteosarcoma.

**Methods**

Dogs with spontaneous osteosarcoma were enrolled in a veterinary clinical trial. Dogs were randomized to receive neo-adjuvant oncolytic VSV-IFNβ-NIS (109 TCID50/kg) or placebo followed by standard of care (SOC) amputation and carboplatin chemotherapy. Tumor tissues and peripheral blood mononuclear cells were collected pre-and post- VSV therapy to characterize local and systemic antiviral and antitumor immune responses.

**Results**

28 dogs were enrolled and treated on the study. Intravenous VSV-IFNβ-NIS was well-tolerated and post infusion symptoms included mild, transient fever and evidence of acute cytokine responses. Preliminary efficacy analyses show survival outcomes exceed that of historic control populations treated with standard of care. Tumor pathology indicated the presence of tumor necrosis and inflammation in VSV treated dogs. Patient specific osteosarcoma cell lines were isolated following surgical tumor resection and tested in vitro to characterize susceptibility to VSV infection and oncolysis. Experiments are underway to characterize T-cell responses against virus and tumor associated antigens. We will evaluate correlation of tumor pathology, in vitro tumor cell line susceptibility, and antiviral and antitumor immune responses to survival outcomes.

**Conclusions**

Neoadjuvant VSV treatment is well-tolerated, and shows preliminary evidence of biological activity and clinical efficacy. Updated results describing anti-tumor immunity that is attributable to oncolytic VSV and its effects on patient outcomes will be presented. These data will indicate if intravenous neoadjuvant VSV-IFNβ-NIS therapy improves clinical outcomes in canine osteosarcoma and inform clinical studies to evaluate this therapeutic approach as an addition to current chemotherapy protocols for osteosarcoma patients. These studies may shed light on the relative roles of viral oncolysis and immune responses in mediating tumor killing and indicate biomarkers that are indicative or predictive of clinical efficacy in heterogeneous cancer.

#### P808 A patient-derived anti-CD9 antibody induces tumor rejection and synergistically enhances anti-PD1 activity

##### Remko Schotte, PhD^1^ , Julien Villaudy^1^, Wouter Pos^1^, Koen Wagner^1^, Viviana Neviani^2^, Daniel Go^1^, Christien Fatmawati^1^, Els Verdegaal^3^, Susan van Hal^1^, Camille Bru^1^, Yvonne Claassen^1^, Pauline van Helden^1^, Sjoerd van der Burg, PhD^3^, Piet Gros^2^, Hergen Spits^1^

###### ^*1*^*AIMM Therapeutics, Amsterdam, Netherlands;*^*2*^*University Utrecht, Utrecht, Netherlands* ; ^*3*^*Leiden University Medical Center, Leiden, Netherlands*

####### **Correspondence:** Remko Schotte (rschotte@aimmtherapeutics.com)

**Background**

Adaptive immunity to cancer cells has been shown to form a crucial part of cancer immunotherapy. We investigated whether an anti-tumor antibody could be isolated from a patient with metastatic melanoma that was tumor-free for 6 years following adoptive transfer of ex vivo expanded autologous T cells [1].

**Methods**

Peripheral blood memory B cells were immortalized using AIMM’s immmortalisation technology (ectopic Bcl-6 and Bcl-xL expression) [2] and analyzed for the presence of tumor-reactive B cells.

**Results**

The AT1412 antibody was identified by virtue of its differential binding to melanoma cells as compared to healthy melanocytes. AT1412 was found to bind a cancer-selective palmitoylated form of the tetraspanin CD9. CD9 is a broadly expressed protein involved in multiple cellular activities. Crystal structure of the CD9 large extracellular loop in complex with an AT1412 Fab fragment revealed that AT1412 binds the CD9 epitope in an extended and unfolded conformation. In addition to melanoma, AT1412 binds other tumor types including gastric, colon- and pancreatic cancer.

AT1412 was shown to induce antibody dependent cellular cytotoxicity (ADCC) of cancer cells in both 2D and spheroid cell cultures. In addition, AT1412 demonstrated activation of monocytes, most likely via activation of CD9 on platelets-bound monocytes. Chemokines such as CCL5/RANTES and CCL2/MCP1 were induced by AT1412 activated platelets and monocytes, respectively.

In mice carrying a human immune system (HIS-mice) [3] AT1412 strongly enhanced tumor rejection of A375 and SKMEL-5 tumor cells. AT1412 treatment was shown to enhance tumor infiltration of CD8 T cells and macrophages. AT1412 efficacy was further synergistically enhanced when combined with an anti-PD1 antibody (nivolumab).

To address safety the effect of AT1412 on human platelet aggregation was studied. In contrast to other CD9 targeting antibodies, AT1412 was shown not to induce aggregation of human platelets. This was confirmed in a single-dose study in cynomolgus monkeys using increasing dose levels of AT1412. Although a transient drop in circulating platelet counts was observed upon antibody injection, AT1412 was well tolerated up to 10 mg/kg antibody (highest dose tested) and did not lead to other adverse events nor were any coagulation factors affected.

**Conclusions**

Taken together, applying AIMM’s proprietary B-cell immortalization technology [2] we isolated a tumor-selective anti-CD9 antibody (AT1412). AT1412 was shown to induce tumor rejection as a single agent and enhances the activity of anti-PD-1 antibodies. Preliminary data from single-dose administration in monkeys suggested that AT1412 can be safely administered. Preclinical development of AT1412 is currently ongoing.

**Acknowledgements**

Funding: Dutch Cancer Society, grant UVA 2010-4822

**References**

1. Verdegaal EME, Visser M, Ramwadhdoebé TH, van der Minne CE, van Steijn JAQMJ, Kapiteijn E, Haanen JBAG, van der Burg SH, Nortier JWR, Osanto S. Successful treatment of metastatic melanoma by adoptive transfer of blood-derived polyclonal tumor-specific CD4+ and CD8+ T cells in combination with low-dose interferon-alpha. Cancer Immunol Immunother. 2011 Jul;60(7):953–63.

2. Kwakkenbos MJ, Diehl SA, Yasuda E, Bakker AQ, van Geelen CMM, Lukens MV, van Bleek GM, Widjojoatmodjo MN, Bogers WMJM, Mei H, Radbruch A, Scheeren FA, Spits H, Beaumont T. Generation of stable monoclonal antibody-producing B cell receptor-positive human memory B cells by genetic programming. Nat Med. 2010 Jan;16(1):123–8.

3. van Lent AU, Centlivre M, Nagasawa M, Karrich JJ, Pouw SM, Weijer K, Spits H, Blom B, Legrand N. Chapter 6 In Vivo Modulation of Gene Expression by Lentiviral Transduction in “Human Immune System” Methods Mol Biol. 2010;87–115.

**Ethics Approval**

The protocol for the melanoma clinical study was approved by the Medical Ethics Committee of the Leiden University Medical Center and conducted in accordance with the Declaration of Helsinki. The patient gave written informed consent.

Blood from healthy individuals was obtained in accordance with national guidelines and oral informed consent.

Animal experiments were approved by the local ethical committee (AMC): AIMM122

#### P809 Preclinical development of a novel, allogeneic, OX40L-secreting, therapeutic cancer vaccine for use in combination with a gp96-secreting vaccine for solid tumors

##### Matthew Seavey, PhD , Jason Rose, MS, Patrick Dillon, PhD, Jayalakshmi Miriyala, MS, Vikas Tahiliani, PhD, Jeff Hutchins, PhD

###### Heat Biologics, Inc., Durham, NC, United States

####### **Correspondence:** Matthew Seavey (mseavey@heatbio.com)

**Background**

Heat Biologics is focused on developing and optimizing a next generation cellular vaccine platform that incorporates a tumor antigen chaperone (gp96-Ig) with costimulation into a single tumor cell line overexpressing a host of cancer associated neoantigens. Viagenpumatucel-L (HS-110), a human lung adenocarcinoma cell line, stably transfected to express gp96-Ig, is being tested in a phase 1/2 clinical trial (NCT#02439450) for NSCLC. A similar line was generated that complements HS-110, providing costimulation in the form of secreted OX40-Ig (HS-130).

**Methods**

As a model, mouse surrogates of each human product (mHS-110 and mHS-130) were generated to activate and expand adoptively transferred OVA-specific T cells (OT-1) responding to tumor challenge with B16F10 cell line over expressing OVA. To identify the best ratio of mHS-110 to mHS-130, multiple dose ratio and dose escalation studies were performed to measure T cell expansion (peripheral and intratumoral) in the context of tumor challenge.

**Results**

Combining mHS-110/gp96-Ig with mHS-130/OX40L-Ig at a ratio of 1 to 1.3, i.e, 339 ng/10^6 cells/24 hrs of gp96-Ig to 441 ng/10^6 cells/24 hrs of OX40L-Ig provided the optimal dose combination for long-term survival and expansion of tumor specific CD8+ T-cells, tumor infiltrating lymphocytes (TILs) and greatest tumor growth inhibition (TGI). The no observed effect level (NOEL) was determined to be 38 ng of gp96-Ig to 50 ng of OX40L-Ig with the minimum active biological effect level (MABEL) being 113 ng gp96-Ig to 147 ng OX40L-Ig. Dosing mice with mHS-130, HS-130 and an OX40 agonist as a positive control, the no observed adverse event level (NOAEL) was determined to be 20 mg/kg for the OX40 agonist antibody, 219.5 ng/animal or 0.01097 mg/kg of human OX40L-Ig/24 hrs for HS-130, and 387.2 ng/animal or 0.01936 mg/kg of mouse OX40L-Ig/24 hrs. The serum cytokines detected in response to cellular injection were those frequently associated with acute innate immune responses (CXCL10, MCP-3, CCL4, TNFalpha, CXCL1).

**Conclusions**

Best dose group combination mHS-110/gp96-Ig to mHS-130/OX40L-Ig was at a ratio of 1 to 1.3, at a dose rate of 339 ng/10^6 cells/24 hrs of gp96-Ig to 441 ng/10^6 cells/24 hrs of OX40L-Ig, with a NOEL of 38 ng gp96-Ig to 50 ng OX40L, a MABEL of 113 ng gp96-Ig to 147 ng OX40L-Ig. These results support the clinical translation of this approach of combining a T cell activation platform with costimulation.

**References**

1. Fromm G, de Silva S, Giffin L, Xu X, Rose J, Schreiber TH. Gp96-Ig/Costimulator (OX40L, ICOSL, or 4-1BBL) Combination Vaccine Improves T-cell Priming and Enhances Immunity, Memory, and Tumor Elimination. Cancer Immunol Res. 2016;4(9):766-778.

#### P810 An engineered bi-function fusion protein (CD86-Fc-NKG2a) to reverse NK cell tolerance of malignant cells

##### Casey Shuptrine, PhD, Taylor Schreiber, MD, PhD

###### Shattuck Labs Inc, Durham, NC, United States

####### **Correspondence:** Taylor Schreiber (tschreiber@shattucklabs.com)

**Background**

As clinical outcomes improve for immunotherapeutic regimens based on PD-1/L1 blockade, the proportion of cancer patients who develop resistance to PD-1/L1 blockade will increase. Some resistance mechanisms, including MHC-I and β2M downregulation, reflect the nature of PD-1/L1 as a T-cell centric checkpoint. Recent clinical success by groups targeting non-T cell immune cells has highlighted the potential of enhancing macrophage or NK cell targeted immunotherapies for the treatment of a wide variety of cancers. Future combinatorial approaches will likely utilize agents targeting multiple different immune cells to effectively arm both the adaptive and the innate immune systems. As a prime example of this, the anti-NKG2a monoclonal antibody Monalizumab, has shown promising preclinical and clinical anti-cancer effects, especially when combined with an ADCC competent antibody [1]. Therefore, targeting non-classical innate immune checkpoints has the potential to synergize with currently approved cancer therapies. Here we report the generation of a two-sided fusion protein, CD86-Fc-NKG2a, which was designed to provide competitive inhibition of the NK-centric HLA-E checkpoint, which providing co-stimulation to T cells via CD28.

**Methods**

Human and mouse variants of CD86-Fc-NKG2a were produced and characterized using a variety of biochemical assays to determine the correct molecular weight, subunit composition and binding affinity; molecular assays to characterize in vitro cell binding, in vitro functional activity, in vitro lysis capabilities; and anti-tumor efficacy in multiple syngeneic model systems.

**Results**

The NKG2a domain bound recombinant and cell-expressed HLA-E with high affinity. The CD86 domain bound recombinant CD28 and CTLA-4, with a higher affinity for CTLA-4. CD86-Fc-NKG2a also stimulated NF-kB signaling in CHO/CD28/NFkB-luciferase cells. When tested head-to-head in a therapeutic CT26 murine tumor model, mCD86-Fc-NKG2a demonstrated stronger anti-cancer activity compared to anti-NKG2a antibody controls. The combination of the CD86-Fc-NKG2a with anti-PD1 and anti-CTLA-4 antibodies was effective in controlling tumor growth in immune competent mice. In vitro cell lysis assays, with PBMCs and isolated NK cells, have further elucidated the effect of CD86-Fc-NKG2a on NK cell mediated tumor cell death.

**Conclusions**

The data presented here demonstrates the feasibility and function of a dual fusion protein, designed to competitively inhibit the NKG2a/HLA-E NK checkpoint pathway, while providing costimulation via CD86. CD86-Fc-NKG2a provided superior tumor control in comparison to NKG2a blocking antibodies, and was further improved in the setting of CTLA-4 or PD-1 blockade. Combined inhibition of NK and T cell checkpoint pathways may reduce the probability of PD-1 resistant disease due to downregulation of MHC-I or other antigen-presentation mechanisms.

**References**

1. Andre´ P, Denis C, Soulas C, et al. Anti-NKG2A mAb is a Checkpoint Inhibitor that Promotes Anti-tumor Immunity by Unleashing Both T and NK Cells. Cell. 2018 Dec 13; 175(7): 1731-1743.e.13.

#### P811 Utilizing novel oncolytic vaccinia virus for selective expression of immunotherapeutic payloads in metastatic tumors

##### Adrian Pelin^1^, Mike Huh, PhD^1^, Matthew Tang, PhD^1^, Fabrice Le Boeuf, PhD^1^, Brian Keller^1^, Jessie Duong^1^, Katherine Clark-Knowles, PhD^1^, Victoria Jennings, PhD^2^, Julia Petryk^1^, Alan Melcher^2^, Mathieu Crupi, PhD^1^, Ragunath Singaravelu, PhD^1^, Larissa Pikor, PhD^1^, Caroline Breitbach, PhD^3^, Steven Bernstein, MD^3^, Michael Burgess, MD, PhD^3^, John Bell, PhD^1^

###### ^*1*^*Ottawa Hospital Research Institute, Ottawa, Canada;*^*2*^*Leeds University, Leeds, United Kingdom*; ^*3*^*Turnstone Biologics, San Francisco, CA, United States*

####### **Correspondence:** John Bell (jbell@ohri.ca)

**Background**

Despite the profound clinical impact that immune checkpoint inhibitors (ICIs) have had, they benefit only a fraction of cancer patients due in part to the multitude of immunosuppressive networks within the tumor microenvironment (TME). Therefore, strategies to selectively re-program the TME from that of immunosuppression to immune stimulation are anticipated not only to improve the clinical efficacy of ICI, but also that of other immunotherapeutic approaches. Oncolytic viruses (OVs) encoded with rational combinations of immune modulatory agents (IMA) have the potential to be the ideal platforms for such TME reprogramming. To be successful however, OVs must be engineered to allow for intravenous delivery and tumor selective infection, replication and transgene expression to deliver clinically-relevant concentrations of IMA directly to TME. Finally, as viruses are evolutionarily designed to subvert the immune response, such immunosuppressive components of the virus must be removed to optimize their ability as immunotherapeutic agents.

**Methods**

Using a combination of functional genomics and bio-selection strategies, we generated a novel Vaccinia Virus (VACV) backbone, denoted as SKV, having augmented oncolytic activity, improved tumor selectivity and less immunosuppression compared to wild-type VACV. We next engineered SKV to encode three potent immune modulators; anti-CTLA4 antibody, membrane tethered IL12p35 and FLT3L. Various immunocompetent syngeneic mouse tumor models and human xenograft tumor models were used to characterize this engineered virus, TBio-6517.

**Results**

SKV generates more potent anti-tumor effects and shows enhanced synergy with ICIs compared to that of other clinical VACV candidates. SKV increases tumor T-cell infiltration while decreasing the number of Treg cell and g-MDSC levels. Tumor-selective transgene expression has been demonstrated in murine tumor models in which therapeutic payload concentrations were achieved within the tumor (e.g. >7.5 ng/mL FLT3L) without any detectable transgene product detected in the systemic circulation. Expression of the therapeutic payloads in TBio-6517 increased survival and tumor control compared to the SKV backbone control in syngeneic mouse tumor models, including humanized CTLA-4 immune checkpoint knock-in mice.

**Conclusions**

Our new best-in-class recombinant VACV robustly stimulates systemic anti-tumor immunity and has a substantially improved pre-clinical safety profile compared to other VACV clinical candidates. Additional ongoing toxicity and efficacy studies are being conducted prior to clinical evaluation of the novel virus construct.

**Ethics Approval**

This study was approved by the University of Ottawa’s ACVS Animal Care Committee.

#### P812 Development of PY159, a monoclonal antibody that repolarizes tumor-associated inhibitory myeloid cells, for the treatment of solid tumors

##### Linda Liang, Michel Streuli, PhD , Chris Chan, Aritra Pal, PhD, Erick Lu, Joshua Pollack, Mikhail Binnewies, Xiaoyan Du, Aritra Pal, Vladi Juric, Evan Greger, Kevin Baker, Len Reyno

###### Pionyr Immunotherapeutics, South San Francisco, CA, United States

####### **Correspondence:** Michel Streuli (michel.streuli@pionyrtx.com)

**Background**

To improve the proportion of patients who benefit from checkpoint inhibitor (CPI) therapy additional immune pathways likely need to be targeted. Tumor-associated macrophages (TAMs), myeloid-derived suppressor cells (MDSCs), and tumor-associated neutrophils (TANs) exhibit a spectrum of functional phenotypes ranging from immunosuppressive M2-like macrophages or N2-like neutrophils that promote tumor growth to pro-inflammatory M1-like macrophages and N1-like neutrophils that promote anti-tumor immunity. Therapies that shift the balance of inhibitory myeloid cells towards a more pro-inflammatory phenotype are expected to positively impact anti-tumor immune responses and convert CPI-resistant tumors into CPI-sensitive tumors.

**Methods**

Human whole blood, peripheral blood mononuclear cells, isolated myeloid cells, or dissociated tumor cells were treated with monoclonal antibodies (mAbs) and analyzed by flow cytometry and Meso Scale Discovery. In vivo efficacy and pharmacodynamic studies were conducted in MC38, CT26, and EMT6 tumor models. Cellular and molecular characterization of tumors was performed using flow cytometry and RNA sequencing.

**Results**

We identified Triggering Receptor Expressed on Myeloid Cells 1 (TREM1) as a target on TAMs, TANs, and MDSCs, and developed anti-human and anti-mouse TREM1 mAbs, termed PY159 and PY159m, respectively. These mAbs activated downstream signaling pathways, induced secretion of a highly selective subset of pro-inflammatory factors, and increased the expression of HLA-DR and CD40, which play critical roles in antigen presentation and T cell activation. Molecular profiling of PY159m-treated syngeneic tumors demonstrated activation of innate and adaptive immune pathways, suggesting that PY159 therapy repolarizes TREM1-positive myeloid cell into pro-inflammatory cells. In vivo, PY159m had anti-tumor activity in a number of syngeneic tumor models. PY159m in combination with anti-PD-1 converted anti-PD-1 resistant tumors into treatment-sensitive tumors. Furthermore, mice cured of their tumors by PY159m combination therapy were resistant to tumor re-challenge suggesting that targeting myeloid cells promotes long-term immunological memory. A survey of the immune infiltrates by both flow cytometry and RNA expression from a variety of indications showed that TREM1-positive myeloid cells were present at a high frequency in all tested tumors. In a pilot, single-dose non-human primate tolerability/pharmacokinetic (PK) study PY159 was generally well tolerated at all tested doses.

**Conclusions**

The available preclinical and nonclinical data support PY159 immunotherapy, alone or in combination with a CPI, in cancer patients who are resistant or refractory to CPI therapies, to improve both the overall response rates as well as the durability of responses. First in human clinical testing will commence in 2020.

#### P813 TAS0313, a novel cocktail vaccine of multivalent HLA-A2-, A24- and A3 superfamily-restricted epitopes, demonstrates a synergistic antitumor effect with anti-PD-1 antibody

##### Yuki Tanaka, MS , Hiroshi Wada, MS, Risa Goto, Toshihiro Osada, Keisuke Yamamura, Satoshi Fukaya, Kazuhisa Minamiguchi, Koichi Ikizawa, Teruhiro Utsugi

###### Taiho Pharmaceutical Co.,Ltd,, Tsukuba, Ibaraki, Japan

####### **Correspondence:** Yuki Tanaka (yuuki-tanaka@taiho.co.jp)

**Background**

Many peptide vaccines have failed in clinical trials due to insufficient efficacy. Therefore, improving the efficacy of peptide vaccines is essential for cancer vaccine development. Long peptide vaccines are expected to have a better antitumor effect than short epitope vaccines. We thus developed three novel long peptide vaccines, TAS0314, TAS0315 and TAS0316, composed of 12 HLA-A2-, A24- or A3 superfamily-restricted cytotoxic T lymphocyte (CTL) epitopes with arginine linkers. The purpose of this preclinical study was to evaluate the immunological characteristics of TAS0313, a cocktail vaccine of TAS0314, TAS0315 and TAS0316, using HLA knock-in (KI) mice.

**Methods**

We immunized HLA-A2, -A24 and -A31 KI mice with TAS0313, and evaluated the epitope-specific CTL induction in lymph nodes using an interferon-γ ELISPOT assay. The antitumor effect against B16F10 tumor cells expressing HLA-A24 and TAS0314 epitope was evaluated using HLA-A24 KI mice treated with either TAS0313 monotherapy or TAS0313 in combination with anti-PD-(L)1 monoclonal antibody (mAb). Epitope-specific CTLs in tumors were measured by epitope-specific HLA tetramer staining.

**Results**

TAS0313 induced all of the detectable epitope-specific CTLs in the HLA KI mice. Prophylactic immunization with TAS0314 significantly inhibited subcutaneous tumor growth on day 14 after tumor inoculation (Vehicle: 359.59 mm3; TAS0314: 127.07 mm3).

Combination treatment with TAS0314 and anti-PD-1 mAb significantly prolonged the survival of mice when compared to TAS0314 or anti-PD-1 mAb monotherapy. The tumor-free rate was 53.33% in the combination TAS0314 and anti-PD-1 mAb group, 13.33% in the TAS0314 monotherapy group, and 0.00% in the anti-PD-1 mAb monotherapy group.

Immunization with TAS0314 induced epitope-specific CTL infiltration into tumors (5.06% of the CD8 T cells). Furthermore, combination treatment with TAS0314 and anti-PD-1 mAb remarkably increased the CTL infiltration (16.8% of the CD8 T cells).

**Conclusions**

We demonstrated that TAS0313 can enhance CTL induction and antitumor effects in vivo. In addition, the combination therapy of TAS0313 and anti-PD-1 mAb resulted in a synergistic antitumor effect by increasing the infiltration of epitope-specific CTLs into tumors. Based on these results, Phase I/II studies evaluating the efficacy of combination therapy with pembrolizumab in urothelial carcinoma patients and monotherapy in glioblastoma patients are currently underway.

**Ethics Approval**

All animal studies were approved by the Taiho Institutional Animal Care and Use Committee.

#### P814 Antibody-interferon-gamma fusion protein for the treatment of B-cell lymphomas

##### Alex Vasuthasawat, Reiko Yamada King, Kham Trinh, Neiki Rokni, Sherie Morrison, PhD, John Timmerman, MD

###### UCLA, Los Angeles, CA, United States

####### **Correspondence:** John Timmerman (jtimmerman@mednet.ucla.edu)

**Background**

The interferons, including IFNα/IFNβ (type I) and IFNγ (type II) are essential mediators of anti-cancer immunity. To achieve efficient targeting of IFNs to tumor sites, we have developed antibody (Ab)-IFN fusion protein technology [1]. A fusion protein targeting human CD20 (anti-huCD20-huIFNα) exhibited stronger direct anti-proliferative effects, complement-dependent cytotoxicity (CDC), Ab-dependent cell-mediated cytotoxicity (ADCC), and in vivo potency against B-cell lymphoma xenograft models compared to the parent Ab rituximab. Based on these results, a phase I, first-in-human, dose-escalation trial of anti-huCD20-huIFNα for B-cell non-Hodgkin lymphoma is now underway (NCT02519270). We now report on the construction and characterization of anti-CD20 fusions containing IFNγ, the IFN species secreted by effector CD8+ T cells, NK cells, and NKT cells.

**Methods**

The VH and VL regions from antibody 2B8 recognizing human CD20 were engineered in recombinant form with human IgG1 constant regions, and fused at the C-terminus with mIFNgamma. Tumor cell proliferation in vitro was measured by [3H]-thymidine incorporation, ADCC by LDH release using mouse splenocyte effectors, CDC by PI exclusion, and in vivo tumor growth using the huCD20-expressing syngeneic mouse B cell lymphoma 38C13-huCD20. Tumor-infiltrating lymphocytes were measured by flow cytometry.

**Results**

Anti-hCD20-mIFNγ suppressed the in vitro proliferation of 38C13-huCD20 lymphoma cells by up to 70% (at 1 nM). Anti-hCD20-mIFNg also showed enhanced ADCC against lymphoma cells compared with unfused, parent antibody (16-20% at E:T ratio of 20:1, versus 9-12%, respectively) (Figure 1), while CDC was identical to unfused antibody. In vivo efficacy with 70-80% cures was demonstrated in mice bearing established subcutaneous 38C13-huCD20 tumors, with systemic (i.v.) injection of 100 mcg anti-hCD20-mIFNγ fusion protein on days 5, 6, 7, and 9 after tumor inoculation, in repeated experiments. Mechanistic studies showed that depletion of natural killer (NK) cells (using anti-asialo-GM1) significantly abrogated tumor clearance (p=0.01), while depletion of macrophages (clodronate liposomes) had lesser, borderline effects (p= 0.05) (Figure 2), and depletion of complement (cobra venom factor) or T cells (CD4+ or CD8+) had no significant effects on tumor eradication. Subcutaneous mouse B cell lymphomas treated with intratumoral injections of anti-hCD20-mIFNγ displayed increased tumor-infiltrating CD8+ T cells (mean 20.6% versus 5% in PBS-treated controls, p=0.008), and CD4+ T cells (mean 15.3% versus 6.6%).

**Conclusions**

Anti-hCD20-mIFNγ fusion protein has in vitro and in vivo efficacy in a syngeneic, immunocompetent model of B cell lymphoma, with NK cells and possibly macrophages implicated in the mechanism(s) of tumor eradication.

**References**

1. Xuan C, Steward KK, Timmerman JM, Morrison SL. Targeted delivery of interferon-alpha via fusion to anti-CD20 results in potent antitumor activity against B-cell lymphoma. Blood. 2010 Apr 8;115(14):2864-71.

**Fig. 1 (abstract P814).**
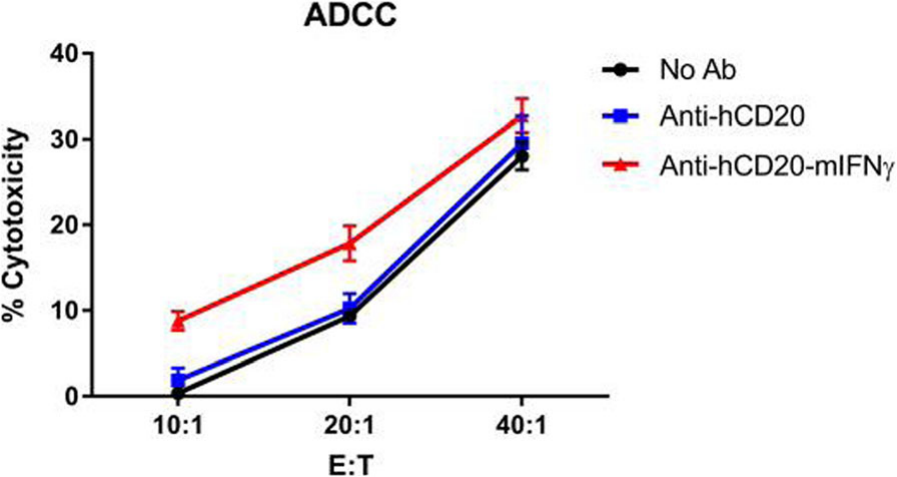
See text for description

**Fig. 2 (abstract P814).**
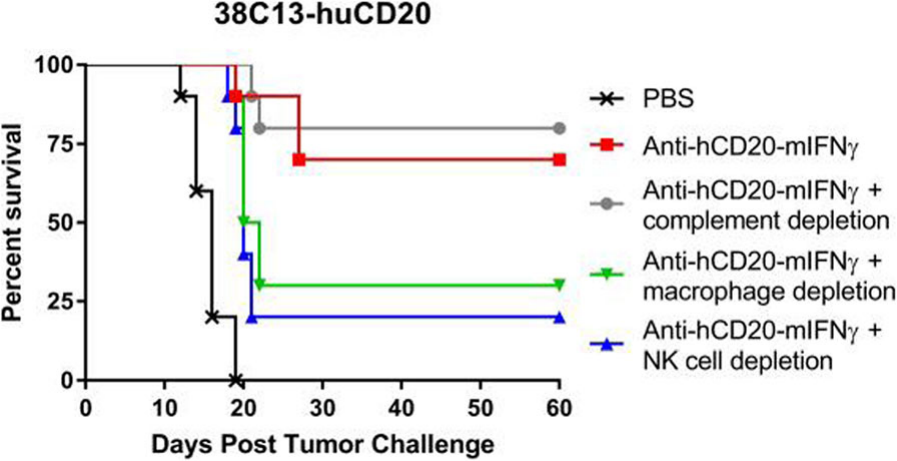
See text for description

#### P815 Targeting multiple immune checkpoint proteins with novel small molecule inhibitors of Sec61-dependent cotranslational translocation

##### Jennifer Whang, PhD^1^, Janet Anderl^1^, Andrea Fan^1^, Christopher Kirk^1^, Eric Lowe^1^, Dustin McMinn^1^ , Beatriz Millare^1^, Meera Rao^1^, Jack Taunton, PhD^2^

###### ^1^Kezar Life Sciences, South San Francisco, CA, United States; ^2^University of California, San Francisco, CA, United States

####### **Correspondence:** Dustin McMinn (dmcminn@kezarbio.com)

**Background**

Antibody-based immunotherapies have demonstrated improved anti-tumor responses and success in the clinic. Nevertheless, a majority of patients either do not respond or develop resistance to single agent checkpoint inhibitors, thus driving exploration into the rational design of combination therapies. Expression of secreted and transmembrane proteins, including checkpoint molecules, involves cotranslational translocation, which is facilitated by the ribosome-signal recognition particle-Sec61 complex [1]. Amino-terminal signal sequences unique to each protein direct nascent polypeptides through the Sec61 channel into the endoplasmic reticulum for expression and function. While many Sec61-targeting compounds have demonstrated anti-tumor effects by broadly inhibiting protein translocation, some compounds have been identified to exert signal sequence-specific blockade [2]. Here we present small molecule inhibitors of Sec61 capable of selectively targeting multiple co-inhibitory immune checkpoint proteins.

**Methods**

Target protein inhibition was assayed using HEK293 cells overexpressing constructs comprised of signal sequences fused to a luciferase reporter. Resistance to novel Sec61 inhibitors was assessed in cell lines overexpressing wild-type or R66I-mutant Sec61, which was previously shown to confer resistance to published Sec61 inhibitors [3]. Tumor cells or T cells were incubated with compound in vitro, and surface expression of immune checkpoint proteins was examined by flow cytometry. T cell functions were examined in vitro following anti-CD3/anti-CD28 stimulation or in T cell co-culture systems. Cytokine production was measured by electrochemiluminescence-based assay. CD73 enzymatic activity was measured in cell-based assays using the AMP-Glo™ assay.

**Results**

We identified a series of compounds that selectively block Sec61-dependent cotranslational translocation of select immune checkpoint proteins. In particular, the compounds more readily inhibited PD-1, B7H3, CD47, CD73, and CD96 compared to other co-inhibitory molecules, such as TIGIT, TIM3 and CTLA-4. Following in vitro compound treatment, decreased PD-1 and CD96 surface expression was observed on activated T cells. Furthermore, the compounds enhanced T cell production of interleukin-2 and interferon gamma in mixed lymphocyte reactions. Select inhibitors also down-regulated surface expression of CD73 on SKOV3 tumor cells. Consistent with this result, compound treatment of SKOV3 cells reduced CD73 hydrolysis of AMP, suggesting these inhibitors may rescue CD73-mediated suppression of T cell functions.

**Conclusions**

Small molecules targeting Sec61-dependent cotranslational translocation provide a new approach for blocking multiple immune checkpoint proteins with a single agent. We have identified novel Sec61 inhibitors that have the capacity to regulate T cell functions and/or the tumor microenvironment. Based on these data, in vivo studies will further elucidate the efficacy of Sec61 inhibitors as immunotherapeutics.

**References**

1. Park E, Rapoport, TA. Mechanisms of Sec61/SecY-mediated protein translocation across membranes. Annu Rev Biophys. 2012; 41:1-20.

2. Van Puyenbroeck V, Vermeire K. Inhibitors of protein translocation across membranes of the secretory pathway: novel antimicrobial and anticancer agents. Cell Mol Life Sci. 2018; 75:1541-1558.

3. MacKinnon AL, Paavilainen VO, Sharma A, et. al. An allosteric Sec61 inhibitor traps nascent transmembrane helices at the lateral gate. eLIFE. 2014; 3:e01483.

#### P816 DRP-104 induces durable responses in vivo by inhibiting tumor glutamine addiction, remodeling the tumor microenvironment and stimulating both the innate & adaptive immune systems

##### Yumi Yokoyama, PhD, Michael Nedelcovych, PhD, Robert Wild, PhD

###### Dracen Pharmaceutical, New York, NY, United States

####### **Correspondence:** Robert Wild (rwild@dracenpharma.com)

**Background**

Glutamine is an essential amino acid for rapidly proliferating cancer cells, thus depriving the same fuel from immune cells and contributing to tumor immune evasion. DRP-104 was designed as a novel prodrug of the broad acting glutamine antagonist 6-Diazo-5-oxo-L-norleucine (DON). DRP-104 is inert in its prodrug form, affords high levels of plasma and gastro-intestinal (GI) tissue stability; has high tumor cell permeability and preferential tumor versus plasma/GI tissue distribution for DON. Here we sought to (1) characterize in vitro biochemical and biological activities, (2) identify the immune-modulatory mechanism of action, and (3) evaluate in vivo anti-tumor efficacy of DRP-104 as a single agent.

**Methods**

Anti-tumor and immunomodulatory effects of DRP-104 were evaluated in vitro using biochemical, cellular and functional assays. Tumor distribution was assessed by LC-MS/MS. Metabolomic analysis was performed by Metabolon. Immuno-phenotyping was performed in tumor and blood samples by flowcytometry, Nanostring, and Luminex. Tumor growth inhibition and survival analysis was performed in syngeneic tumor models.

**Results**

DRP-104 and its active moiety DON showed glutamine dependent inhibition of cancer cell growth in vitro, correlating with broad glutamine pathway inhibition. In vivo, DRP-104 is preferentially converted to DON in tumors and has minimal plasma/GI tissue distribution. Metabolomic profiling in tumor samples showed widespread changes indicative of disruption of tumor anabolism and canonical cancer metabolism pathways. In addition, glutamine and various other amino acids were significantly increased while several immune-suppressive metabolites were decreased. DRP-104 treatment resulted in substantial and broad changes in various immune cell infiltrates, such as increased TIL, T, Tmem, NK and NKT cells. T cells were more proliferative and less exhausted, NK cells showed increased activation markers and TAMs were polarized to M1 phenotype. DRP-104 treatment resulted in significant reduction in PD-L1 expression on tumors, macrophages and dendritic cells. Pro-tumorigenic proteins, such as VEGF and IL-8, were decreased. Finally, DRP-104 showed significant tumor growth inhibition including curative effects as a single agent in mouse syngeneic tumor models.

**Conclusions**

DRP-104 is a novel, broad acting glutamine antagonist with potent single agent anti-tumor activity. DRP-104 treatment causes not only wide-spread shutdown of glutamine pathways resulting in dramatic metabolic remodeling of the TME but also results in enhanced infiltration and function of immune cells involving both innate and adaptive immunity. This unique mechanism of action suggests DRP-104 may function as a novel metabolic checkpoint inhibitor and supports clinical development of DRP-104 to potentially treat a wide variety of cancers.

#### P817 Potent ERAP1 inhibitors modify the immunopeptidome and generate novel neoantigens

##### Jamie Ware^1^, Kris Clark^1^, Carmen Tong^1^, Jason Shiers^1^, Elisa Lori^2^, Emma Reeves^2^, Henry Leonard^3^, Alihussein Remtulla^3^, Nicola Ternette^4^, Edd James, PhD^2^, Martin Quibell^5^, Lesley Young, PhD^5^, Peter Joyce^5^

###### ^*1*^*Sygnature Discovery, Nottingham, United Kingdom;*^*2*^*University of Southampton, Southampton, United Kingdom* ; ^*3*^*Charles River Laboratories, Portishead, United Kingdom* ; ^*4*^*University of Oxford, Oxford, United Kingdom* ; ^*5*^*Grey Wolf Therapeutics Ltd, Oxford, United Kingdom*

####### **Correspondence:** Peter Joyce (peter.joyce@greywolftherapeutics.com)

**Background**

Prior research demonstrates tumours with low neoantigen burden respond poorly to T-cell checkpoint blockade, with neoantigen-specific T-cell responses important to tumour regression [1]. Endoplasmic reticulum aminopeptidase 1 (ERAP1) is an enzyme that trims peptides loaded into classical and nonclassical MHC molecules [2]. Inhibition of ERAP1 creates neoantigens, resulting in improved immunogenicity, generation of CD8 T cell responses and tumor growth inhibition [3,4]. Presentation of novel peptides also leads to the activation of NK cells through KIRs and NKG2A in vitro and in vivo [5,6]. We report the generation and activity profile of novel, potent and selective ERAP1 inhibitors as novel oral immunotherapy agents.

**Methods**

A suite of in vitro assays was established to profile the potency, affinity and selectivity of several lead series and select lead candidates. In vitro and ex vivo CETSA measurements were used to measure target engagement. Pharmacodynamic measurements included Class I MHC expression in CT26 [3] and SIINFEKL/H-2Kb in CAG-Ova Tg mice by FACS [7]. Immunopeptidomics [8] were applied to both human and mouse cell lines treated with ERAP inhibitors to profile peptide changes and identify new peptides or changes in peptide levels as a consequence of ERAP inhibition.

**Results**

Optimised lead series potently inhibit ERAP1 across key species and ERAP1 haplotypes. In addition to a correlation between lead series enzymatic activity and ERAP1 affinity, biochemical potency translates into cell-based target engagement measurements in mouse and human cell lines and PBMC. ERAP1 modulation (siRNA /lead compounds) dose dependently increases overall length of the immunopeptidome and changes a proportion of the repertoire resulting in presentation of novel neoantigens and tumour associated antigens. In vitro treatment of CT26 downregulates Ld, Kd and Dd in line with published data from ERAP knockout mice [2]. Leads with optimised PK, bioavailability and target engagement were administered orally to CAG-OVA transgenic mice downregulating SIINFEKL/H-2Kb in a dose dependent manner confirming published in vitro data [9] and demonstrating in vivo PD. Profiling is ongoing in in vivo tumor growth inhibition and PD studies, and human primary CD8 and NK assays to select a lead candidate.

**Conclusions**

We have identified potential first in class, potent and species cross reactive small-molecule inhibitors of ERAP1 that modify the immune peptidome creating neoantigens. Extensive preclinical development is ongoing to generate a transformative therapeutic that could be used as monotherapy or in combination with other immunotherapies such as checkpoint blockade.

**References**

1. Rizvi N, Hellmann MD, Snyder A, et al. Mutational landscape determines sensitivity to PD-1 blockade in non–small cell lung cancer. Science. 2015;348(6230):124-128.

2. Shastri N, Nagarajan N, Lind KC, et al. Monitoring peptide processing for MHC class I molecules in the endoplasmic reticulum. Curr Opin Immunol. 2014;26:123-127.

3. James E, Bailey I, Sugiyarto G, et al. Induction of protective antitumor immunity through attenuation of ERAAP function. J Immunol. 2013;190(11):5839-5846.

4. Mpakali A, Maben Z, Stern LJ, et al. Molecular pathways for antigenic peptide generation by ER aminopeptidase 1. Mol Immunol. 2018. doi: https://doi.org/10.1016/j.molimm.2018.03.026. [Epub ahead of print].

5. Cifaldi L, Monaco EL, Forloni M, et al. Natural Killer Cells Efficiently Reject Lymphoma Silenced for the Endoplasmic Reticulum Aminopeptidase Associated with Antigen Processing. Cancer Res. 2010;71(5):1597-1606.

6. Cifaldi L, Romania P, Falco M, et al. ERAP1 Regulates Natural Killer Cell Function by Controlling the Engagement of Inhibitory Receptors. Cancer Res. 2015; 75:824-834.

7. Ehst BD, Ingulli E, Jenkins MK. Development of a novel transgenic mouse for the study of interactions between CD4 and CD8 T cells during graft rejection. Am J Transplant. 2003; 3(11):1355-1362.

8. Purcell AW, Ramarathinam SH, Ternette N. Mass spectrometry–based identification of MHC-bound peptides for immunopeptidomics. Nat Protoc. 2019;14(6):1687-1707.

9. York IA, Brehm MA, Zendzian S, et al. Endoplasmic reticulum aminopeptidase 1 (ERAP1) trims MHC class I-presented peptides in vivo and plays an important role in immunodominance. Proc Natl Acad Sci USA. 2006;103(24):9202-9207.

#### P818 EGFR Exon-20 insertion-based neoantigen stimulated effective T-cell immunity against non-small cell lung cancer

##### Jinpu Yu , Dandan Liu

###### Tianjin Tumor Hospital, Tianjin Shi, China

####### **Correspondence:** Jinpu Yu (jinpu_yu@hotmail.com)

**Background**

Certain Epidermal growth factor receptor (EGFR) mutations are targets of tyrosine kinase inhibitors (TKIs) and predictive biomarkers of clinical response to TKI therapy. However, NSCLC patients with EGFR exon-20 insertions respond poorly to TKI therapy. Considering higher sequence divergence correlated with more immunogenic of neoantigen, we investigated whether EGFR exon-20 insertion-based neoantigen peptide could stimulate effective T-cell immunity against NSCLC.

**Methods**

The immunogenic T cell epitopes of EGFR exon-20 insertions coding peptide were predicted using multiple public bioinformatic algorithms referring on their expression level and major histocompatibility complex (MHC) class I-binding capacity. The most immunogenic EGFR exon-20 insertion-based neoantigen peptides were synthesized to validate its efficacy to induce specific anti-tumor T-cell immunity in vitro.

**Results**

Ninety-one types of insertions in EGFR exon-20 were identified, among which two types occurred with considerable high mutation frequencies: V769_D770insASV (19.02%) and D770_N771insSVD (19.2%). Both EGFR exon-20 insertions coding peptide displayed higher MHC class I binding score than other insertions, which implied more immunogenicity to induce effective T-cell immunity. Therefore, PBMCs of a NSCLC patient with V769_D770insASV insertion in EGFR was collected and primed with V769_D770insASV coding long peptide(E-ASV) in vitro for 14d, and specific anti-tumor CD8+ T cells were induced and amplified which produced significant high level of interferon-γ (IFN-γ) upon E-ASV re-stimulation.

**Conclusions**

E-ASV could induce cytotoxic T lymphocyte (CTL) responses in vitro, and EGFR exon-20 insertion-based neoantigen peptide might become an alternative immunotherapeutic approach for NSCLC.

**Acknowledgements**

This work was supported by National Natural Science Foundation of China (grant numbers: 81872143, 81702280, 81472473, 81272360) and Key Project of Tianjin Health and Family Planning Commission (16KG126).

#### P819 Regulation of isatuximab-mediated antibody-dependent cellular cytotoxicity of multiple myeloma cells: Rationale for combining isatuximab with anti-PD-1 / anti-TGFβ to enhance anti-myeloma activity

##### Anlai Wang^1^ , Zhili Song^1^, Guang Yang^1^, Rita Greco^1^, Joachim Theilhaber^1^, Elvis Shehu^1^, Beatriz Ospina^1^, Fangxian Sun^1^, Yu-Tzu Tai^2^, Kenneth Anderson, MD^2^, Chen Zhu^1^, Marielle Chiron, PhD^1^, Francisco Adrián^1^

###### ^1^Sanofi Oncology Research, Boston, MA, United States; ^2^Dana-Farber Cancer Institute, Boston, MA, United States

####### **Correspondence:** Anlai Wang (anlai.wang@sanofi.com)

**Background**

CD38 is a transmembrane protein with relatively low expression levels on immune cells and some non-hematological tissues. Under disease conditions, CD38 is expressed in a number of hematological malignancies. In particular, high levels of CD38 expression are found in malignant cells from patients with multiple myeloma (MM) and germinal center B-cell diffuse large B-cell lymphoma (DLBCL) [1,2]. Therefore, eliminating these tumor cells by targeting CD38 is an attractive therapeutic option. Isatuximab is an investigational anti-CD38 monoclonal antibody with promising clinical activity in patients with relapsed/refractory multiple myeloma (RRMM) [3, 4]. Antibody-dependent cellular cytotoxicity, mediated by natural killer (NK) cells, is regarded as a key mechanism of isatuximab anti-myeloma activity [5–7]. MM cells are known to suppress NK cells through the production of immunosuppressive cytokines and the induction of checkpoint inhibitor PD-1/PD-L1 expression [8, 9].

**Methods**

To determine potential mechanisms that may affect the efficacy of isatuximab, we conducted in vitro studies using a panel of CD38 expressing MM and DLBCL cell lines, as well as primary MM cells from patients. MM cells were exposed to isatuximab and peripheral blood mononuclear cells (PBMCs) that had been cultured in the presence or absence of the MM cells. The expression of the checkpoint inhibitor PD-1 and PD-L1 was measured at regular intervals in NK cells co-cultured with MM cells. To investigate whether cytokines present in the MM tumor environment could induce PD-L1 expression on MM cells or affect isatuximab antitumor activity we treated MM cell lines with recombinant IL-6, TGFβ1, and IFNα.

**Results**

PD-1 expression on PBMCs was induced shortly after co-culture with MM cells. The activity of isatuximab-mediated ADCC was decreased in MM cell exposed PMBCs, suggesting that PD-1 expression on NK cells plays a critical role in restricting the functionality of NK cells and isatuximab-mediated ADCC. We also observed decreased cytolytic activity and isatuximab-mediated ADCC against MM cells following NK cell exposure to TGFβ1. PD-1 expression was unaltered by TGFβ1 exposure.

**Conclusions**

Our observations demonstrate that ADCC is a key mechanism for the antitumor activity of isatuximab and provides a rationale supporting the testing of isatuximab in combination with anti-TGFβ or anti-PD1 antibodies to determine whether isatuximab-mediated killing of CD38-expressing tumor cells is improved.

**Acknowledgements**

Study funded by Sanofi. Medical writing support provided by Aspire Scientific (Bollington, UK), funded by Sanofi.

**References**

1. Lin P, Owens R, Tricot G, Wilson CS. Flow cytometric immunophenotypic analysis of 306 cases of multiple myeloma. Am J Clin Pathol. 2004;121:482-8.

2. Alizadeh AA, Eisen MB, Davis RE, Ma C, Lossos IS, Rosenwald A, et al. Distinct types of diffuse large B-cell lymphoma identified by gene expression profiling. Nature. 2000;403(6769):503-11.

3. Martin T, Strickland S, Glenn M, et al. Phase I trial of isatuximab monotherapy in the treatment of refractory multiple myeloma. Blood Cancer J 2019;9:41.

4. Richardson PG, Attal M, Rajkumar SV, et al. A phase 3 randomized, open-label, multicenter study comparing isatuximab, pomalidomide, and low-dose dexamethasone versus pomalidomide and low-dose dexamethasone in patients with relapsed/refractory multiple myeloma (RRMM). J Clin Oncol; 37(15):suppl 8004-8004.

5. Deckert J, Wetzel MC, Bartle LM, et al. SAR650984, a novel humanized CD38-targeting antibody, demonstrates potent antitumor activity in models of multiple myeloma and other CD38+ hematologic malignancies. Clinical cancer research : an official journal of the American Association for Cancer Research. 2014;20(17):4574-83.

6. Jiang H, Acharya C, An G, et al. SAR650984 directly induces multiple myeloma cell death via lysosomal-associated and apoptotic pathways, which is further enhanced by pomalidomide. Leukemia. 2016;30(:399-408.

7. Moreno L, Perez C, Zabaleta A, et al. The Mechanism of Action of the Anti-CD38 Monoclonal Antibody Isatuximab in Multiple Myeloma. Clin Cancer Res. 2019;25:3176-3187.

8. Hallett WH, Jing W, Drobyski WR, et al. Immunosuppressive effects of multiple myeloma are overcome by PD-L1 blockade. Biol Blood Marrow Transplant. 2011;17(8):1133-45.

9. Pratt G, Goodyear O, Moss P. Immunodeficiency and immunotherapy in multiple myeloma. Br J Haematol 2007;138(5):563-579.

### Other

#### P820 Analysis of synergy between early-incorporation immunotherapy and extracranial radiotherapy in stage IV non-small cell lung cancer

##### Corbin Jacobs, MD, Parth Chodavadia, BS, Frances Wang, MS, Joseph Salama, MD, Christopher Kelsey, MD, Jeffrey Clarke, MD, Neal Ready, MD, PhD, Jordan Torok, MD

###### Duke University, Durham, NC, United States

####### **Correspondence:** Jordan Torok (jordan.torok@duke.edu)

**Background**

Emerging evidence suggests that combining radiotherapy (RT) and immunotherapy (IT) may enhance outcomes for metastatic non-small cell lung cancer (NSCLC) patients. However, there remains limited data on the immunomodulatory effects of extracranial RT. This study examined real-world practice patterns, predictors of survival, and comparative effectiveness of extracranial radioimmunotherapy (RT+IT) versus early-incorporation IT alone (defined as receiving IT within 120 days of diagnosis) based on sequencing, irradiated site, and dose-fractionation in patients with metastatic NSCLC.

**Methods**

Patients diagnosed with stage IV NSCLC between 2004-2016 treated with IT alone or RT+IT were identified in the National Cancer Database. The Cochrane-Armitrage trend test assessed for significant trends in practice patterns over time. OS was analyzed using Cox proportional hazards models and the Kaplan-Meier method. Propensity score matching with respect to diagnosis year, age, race, sex, comorbidity index, insurance status, facility type, histology, organs with metastasis, and chemotherapy receipt was performed to account for baseline imbalances. Biologically effective doses (BED) were stratified based on the median (30Gy/10 fractions=39Gy10), and SBRT was defined as above median BED in ≤5 fractions.

**Results**

Early-incorporation IT utilization increased from 0.3% in 2010 to 13.2% in 2016 among patients with stage IV NSCLC (p<0.0001). Rates of RT+IT also increased from 38.8% in 2010 to 49.1% in 2016 among those who received IT (p<0.0001). On unadjusted analysis, patients who received RT+IT demonstrated worse OS compared to patients who received IT alone (11.2 versus 13.2 months median OS, log-rank p<0.0001). Increasing BED was associated with improved OS, and SBRT+IT was associated with improved OS compared to IT alone (25 versus 13.2 months median OS, log-rank p<0.0001). There were no significant differences in OS based on sequencing of IT relative to RT (log-rank p=0.4333) or irradiated site (log-rank p=0.1395). On multivariate analysis, factors associated with improved OS included chemotherapy receipt (HR 0.86, p=0.0058), treatment at academic facilities (HR 0.83, p<0.0001), and SBRT (HR 0.60, p=0.0009); after propensity-score matched multivariate analysis, patients who received above median BED (excluding SBRT) also showed improved OS (HR 0.85, p=0.0485).

**Conclusions**

Utilization of RT+IT in metastatic NSCLC is increasing. SBRT+IT was associated with improved OS on multivariate analysis, and RT+IT in propensity-score matched patients receiving ≥39Gy10 was associated with improved OS on multivariate analysis. There were no significant differences in OS based on RT+IT sequencing or site irradiated. Whether these observations reflect patient selection or possible immunomodulatory benefits of RT is unclear and warrants further study.

**Ethics Approval**

The Institutional Review Board deemed this study exempt from review given the de-identified nature of the data.

#### P821 Induction of anti-tumor immune responses in ovarian cancer with c-MET inhibitor Capmatinib

##### Maureen Drakes, PhD^1^ Swati Mehrotra, MD^1^, Ronald Potkul, MD^1^, M.Sharon Stack, PhD^3^, Patrick Stiff, MD^1^

###### ^1^Loyola University Chicago, Maywood, IL, United States; ^3^University of Notre Dame, South Bend, IN, United States

####### **Correspondence:** Maureen Drakes (mdrakes@luc.edu)

**Background**

Ovarian cancer is generally asymptomatic until the advanced stages. The majority of patients will eventually die of their disease [1]. Our goal is to investigate novel therapies which can improve this outcome.

c-mesenchymal-epithelial transition receptor (c-MET) is a receptor tyrosine kinase which is upregulated in ovarian cancer. The disease promoting properties of c-MET in tumors is primarily due to the overexpression of the wild type gene, and binding to its ligand hepatocyte growth factor (HGF) [2,3]. In our laboratory, we found c-MET expression in 52/ 54 (96 %) ovarian cancer patient sections (28/ 54; 52% with high expression). c-MET signaling controls tumor growth, invasion, resistance to therapy, the maintenance of cancer stem cells, and immune suppressive pathways, which may all contribute to worsening of ovarian cancer [4,5,6].

**Methods**

The c-MET/ HGF pathway is often studied in the context of its tumorigenic properties, but the focus of this research was to study its potential to regulate T cell immune responses in ovarian cancer, with the future goal of using this molecule in combination therapy design. We hypothesize that attenuation of the c-MET/ HGF pathway may induce anti-tumor effector T cell immune responses, thereby overcoming underlying immune suppression in ovarian cancer. Ovarian cancer was induced in C57BL/6 (H-2Kb) mice by intraperitoneal injection of syngeneic ID8-RFP ovarian tumor cells. We studied the efficacy of a c-MET inhibitor Capmatinib (INCB28060) monotherapy on disease outcome by measuring immune responses in spleen cells.

**Results**

c-MET inhibitor Capmatinib treatment of mice resulted in an average ascites volume of 4.34 ml (Figure 1), and vehicle treatment 9.2 ml (> 2 fold decrease, n=5/ group). The average weight increase was 6.5 mg with Capmatinib treatment, versus 9.8 mg vehicle mice (n=5, p=0.06, borderline significant by t-test). We measured the anti-tumor potential of spleen cells stimulated with anti-CD3 antibody. A higher percent of CD8+ T cells secreted anti-tumor cytotoxic molecule granzyme B from Capmatinib treated mice (62.8%), in comparison with (49.6) vehicle (Table 1). Additionally, in cells of Capmatinib treated mice, 3 of 5 (60%) released greater than 5000 pg/ml granzyme B (Figure 2), compared with only 1 of 5 (20%) from vehicle treated mice.

**Conclusions**

With these anti-tumor responses, the performance of Capmatinib for ovarian cancer treatment is promising. Pre-clinical studies are ongoing with this inhibitor alone and in combination with anti-programmed death-1(PD-1) antibody for better outcome, and studies are planned to use this treatment with conventional therapy.

**References**

1. Siegel R, Miller K, Jemal A. CA Cancer J. Clin. Cancer Statistics. 2019; 69: 7-34

2. Sawada K, Radjabi A, Shinomiya N, Kistner E, Kenny H, Becker A.R, Turkyilmaz M, Salgia R, Yamada S, Vande Woude G, et al. C-Met Overexpression is a Prognostic Factor in Ovarian Cancer and an Effective Target for Inhibition of Peritoneal Dissemination and Invasion. Cancer Res. 2007; 67: 1670-1679.

3. Zhang Y, Xia M, Jin K, Wang S, Wei H, Fan C, Wu Y, Li X, Li X, Li G, et al. Function of the C-Met Receptor Tyrosine Kinase in Carcinogenesis and Associated Therapeutic Opportunities. Mol. Cancer. 2018; 17:45.

4. Trusolino L, Comoglio P. Scatter-Factor and Semaphorin Receptors: Cell Signalling for Invasive Growth. Nat. Rev. Cancer 2002; 2: 289-300.

5. Bardelli A, Corso S, Bertotti A, Hobor S, Valtorta E, Siravegna G, Sartore-Bianchi A, Scala E, Cassingena A, Zecchin D. et al. Amplification of the MET Receptor Drives Resistance to Anti-EGFR Therapies in Colorectal Cancer. Cancer Discov. 2013; 3: 658-673.

6. Sierra J, Tsao M. C-MET as a Potential Therapeutic Target and Biomarker in Cancer. Ther. Adv. Med. Oncol. 2011; 3; 21-35.

**Ethics Approval**

Studies were conducted with the approval of our IACUC board (protocol LU 205167).

**Table 1 (abstract P821).**
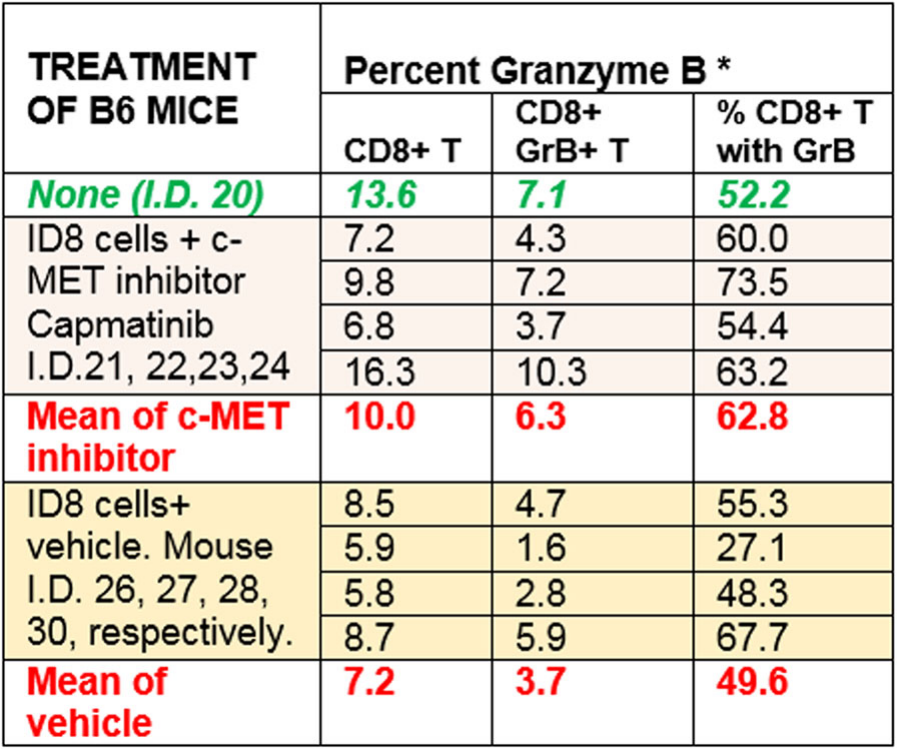
See text for description

**Fig. 1 (abstract P821).**
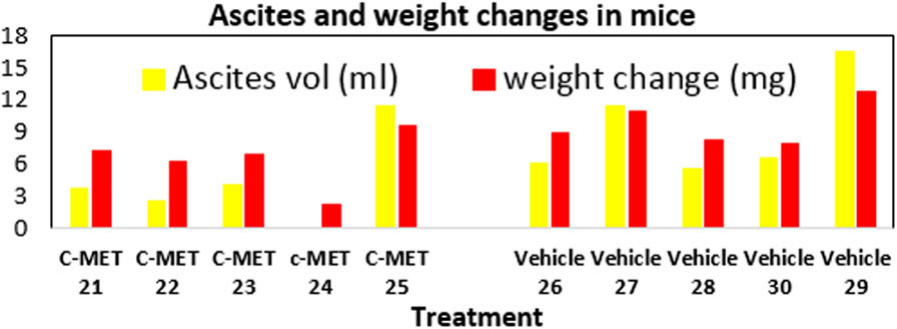
See text for description

**Fig. 2 (abstract P821).**
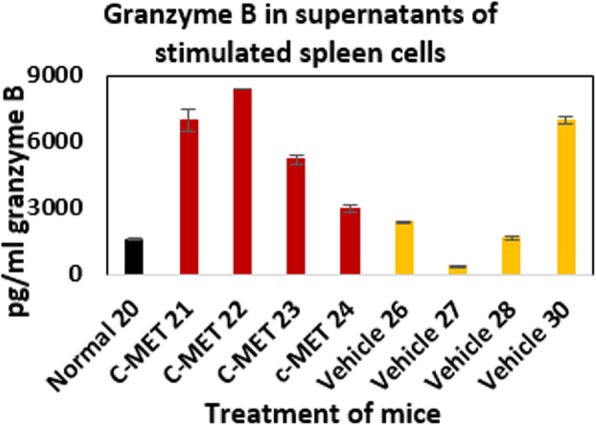
See text for description

#### P822 Evidence that a germline genetic variant impairs a robust anti-tumor immune response in a substrain of C57BL/6 mice

##### Jessica Fessler, BS, Vyara Matson, PhD, Thomas Gajewski, MD, PhD

###### University of Chicago, Chicago, IL, United States

####### **Correspondence:** Thomas Gajewski (tgajewsk@medicine.bsd.uchicago.edu)

**Background**

Among the factors that influence response to immunotherapy, germline genetics has been linked to outcome [1,2]. Identifying genetic variants in murine models that influence antitumor immunity may prove important to understanding how germline differences impact immunotherapy efficacy more generally, and could identify novel therapeutic targets.

C57BL/6 mice represent an important model system for in vivo experimentation, but strain-level discrepancies have prompted recent scrutiny. Mice housed under different conditions or from different vendors can harbor distinct commensal communities, resulting in profound phenotypic differences [3,4]. Additionally, genetic drift over decades of breeding at different facilities has led to the designation of “substrains” within the C57BL/6 classification with some genetic differences already defined.

**Methods**

Mice obtained from Envigo, Taconic, and Jackson labs were injected subcutaneously with various C57BL/6-derived cell lines including the melanoma line B16.F10.SIY (B16.SIY) expressing a model antigen to quantify tumor-specific immune response [5]. Tumor growth was measured over time, and T cell responses and phenotyping measured in the spleen and tumor. Fecal 16S rRNA gene amplicon sequencing was performed. Whole-genome sequencing was performed from DNA isolated from bulk mouse splenocytes and aligned to the C57BL/6J genome.

**Results**

Tumors grew significantly faster in mice purchased from the vendor Envigo compared to mice acquired from either Jackson or Taconic, and showed a significantly impaired antitumor CD8+ T cell response. Manipulation of Envigo microbiota through co-housing with either Taconic or Jackson mice, or oral gavage of fecal material did not influence tumor control suggesting little to no contribution of the microbiota. To determine whether a genetic abnormality unique to Envigo mice may account for their weak anti-tumor immune response, we bred Envigo mice to either Jackson or Taconic mice and studied tumor control in the F1 and F2 generation. We found that both the Envigo-Jackson F1 and Envigo-Taconic F1 uniformly resemble the slower growing Jackson and Taconic tumors. The Envigo-Jackson or the Envigo-Taconic F2 generation, however, showed variable control, following Mendelian inheritance with 25% of the F2 exhibiting Envigo-level fast tumor growth. Using bone marrow chimeras, we found that the inferior Envigo tumor control tracked with the hematopoietic compartment rather than with the host. Whole-genome sequencing revealed Envigo-specific polymorphisms and genetic variants distinct from both Jackson and Taconic mice. Detailed genetic analysis is on-going.

**Conclusions**

Our results indicate that mice from Envigo likely harbor a germline variant that impairs their anti-tumor immune response. Current work is focused on identifying the variant mediating inferior anti-tumor immunity.

**References**

1. Chowell D, Morris LGT, Grigg CM, et al. Patient HLA class I genotype influences cancer response to checkpoint blockade immunotherapy. Science. 2018;359(6375):582-587.

2. Chat V, Ferguson R, Simpson D, et al. Autoimmune genetic risk variants as germline biomarkers of response to melanoma immune-checkpoint inhibition. Cancer Immunol Immunother CII. 2019;68(6):897-905.

3. Ivanov II, Atarashi K, Manel N, et al. Induction of intestinal Th17 cells by segmented filamentous bacteria. Cell. 2009;139(3):485-498.

4. Sivan A, Corrales L, Hubert N, et al. Commensal Bifidobacterium promotes antitumor immunity and facilitates anti–PD-L1 efficacy. Science. 2015;350(6264):1084-1089.

5. Blank C, Brown I, Peterson AC, et al. PD-L1/B7H-1 Inhibits the Effector Phase of Tumor Rejection by T Cell Receptor (TCR) Transgenic CD8+ T Cells. Cancer Res. 2004;64(3):1140-1145.

#### P823 The Cancer Moonshot Immuno-Oncology Translational Network

##### Alan Hutson, PhD , Song Liu, Martin Morgan, Kunle Odunsi, MD, PhD

###### Roswell Park Comprehensive Cancer Center, Buffalo, NY, United States

####### **Correspondence:** Alan Hutson (alan.hutson@roswellpark.org)

**Background**

Immunological destruction of tumors is a multistep, coordinated process that can be targeted at several critical points along the cancer immunity cycle to elicit tumor rejection, and to prevent emergence of cancer. Despite regulatory approval of many immune-based interventions in the past decade, a number of barriers remain to be addressed in order to fully harness the therapeutic potential of the immune system and improve outcomes for cancer patients. In 2016, the Cancer Moonshot Blue Ribbon Panel convened by the NIH recommended to “create a translational science network devoted to immunotherapy” [1].

**Methods**

The Immuno-Oncology Translational Network (IOTN) was created in 2018 to improve the efficacy, durability, and safety of immunotherapy across the spectrum of human cancers, and develop immunoprevention approaches that will prevent cancers before they occur [2]. The consortium structure of the IOTN will enable rapid translation of basic discoveries to translation and clinical application by fostering highly collaborative team science. The NIH program stewardship of the IOTN is led by a trans-NIH adult immunotherapy implementation team, which provides scientific and programmatic oversight to ensure the IOTN meets the goals defined by the Cancer Moonshot Blue Ribbon Panel.

**Results**

The IOTN currently consists of 12 research groups spanning immunoprevention, novel therapeutic targets, cell-based therapies, neoantigen and TCR approaches, multimodal therapies, immuno-suppression and immuno-responsiveness. These studies are conducted in diverse tumor types, including brain, breast, colon, liver, lung, oral, ovarian, prostate and skin cancers. These efforts include a Cellular Immunotherapy Data Resource (CIDR) program and a Data Management and Resource-Sharing Center (DMRC). CIDR provides a data registry for collecting outcomes of patients receiving cellular immunotherapies. DMRC provides overall administrative, software development, data analytical and data coordinating support to facilitate research integration across the IOTN, as well as outreach to other Cancer Moonshot initiatives and the larger scientific community. To synergistically tackle scientific gaps that cannot be addressed by individual research projects, the IOTN has formed six working groups: 1) Bioinformatics and Computational Biology, 2) Cancer Antigens, 3) Immune Mechanisms, 4) Immuno-radiotherapy, 5) Immuno-prevention, and 6) Translational and Cellular Therapy. The IOTN will expand in 2019 to include additional immunoprevention and immunotherapy research projects, as well as new initiatives including immuno-engineering centers and immune-related adverse events research projects.

**Conclusions**

The major anticipated deliverable of the IOTN collaborative consortium is the acceleration of clinical translation of basic discoveries to overcome barriers that limit the efficacy of immunotherapy across the spectrum of human cancers.

**Acknowledgements**

This work is supported by an IOTN Moonshot grant U24CA232979-01.

**References**

1. Jacks, T., Jaffee, E. and Singer, D. Blue Ribbon Panel Co-Chairs (2016) Cancer Moonshot Blue Ribbon Panel Report. National Cancer Institute, Bethesda, MD.

2. McShea, K. (Sept 26, 2018) Roswell Park Shoots for the moon and hits mark with $6 million dollar award. The Buffalo News. B1, B12.

#### P824 Development of anti-Globo H bispecific antibody for breast cancer therapy

##### Yow-Sien Lin, PhD, Jei-Hwa Yu, Chia-Cheng Wu

###### Development Center for Biotechnology, Taipei, Taiwan, Province of China

####### **Correspondence:** Chia-Cheng Wu (chiachengwu@dcb.org.tw)

**Background**

In women, breast cancer is the most common cancer and the first leading cancer-caused deaths worldwide. Up to 30% of the breast cancer patients overexpressed HER2, and trastuzumab is the most commonly prescribed HER2-targeted therapy among those patients. Besides HER2, a tumor-associated glycan Globo H has also been shown to be overexpressed in breast cancer patients, and its prevalence is ~61% of total patients. Therefore, targeting Globo H as therapeutics will benefit more breast cancer patients. Here, we developed an anti-Globo H bispecific antibody (BsAb) for breast cancer therapy.

**Methods**

The humanized anti-Globo H mAb was incorporated into the asymmetric IgG-like bispecific antibody platform developed by our institute. The anti-Globo H moiety targets the Globo H+ breast cancer and the anti-CD3 moiety recruits and activates T cell. The in vitro T cell activation and cytokine secretion was examined. The anti-tumor efficacy of this novel T cell engager was evaluated in the HCC1428 breast cancer xenograft animal model.

**Results**

Asymmetric IgG-like BsAb usually generates mispairing species. The anti-Globo H BsAb developed from our platform showed a high ratio of correct pairing. Besides that, it activates various cytokines in a target cell-dependent manner which elevates its safety profile. The BsAb eradicates the Globo H+ breast cancer in vitro and in vivo.

**Conclusions**

In summary, the asymmetric IgG-like anti-Globo H bispecific antibody possesses great efficacy in killing breast cancer both in vitro and in vivo. The toxicology study of this novel biologics will be evaluated before filing IND for clinical trials.

#### P825 Increased and co-localized CD4+FOXP3+ and CD8+ T cells is a strong independent prognostic indicator in gastric cancer

##### Minyu Wang^1^, Yu-Kuan Huang^1^, Joe Kong^1^, Yu Sun^1^, Daniela Tantalo^1^, Han Xian Aw Yeang^1^, Le Ying^2^, Feng Yan^3^, Dakang Xu^4^, Heloise Halse^1^, Natasha di Costanzo^1^, Ian Gordon^5^, Catherine Mitchell^1^, Laura Mackay^5^, Rita Busuttil^1^, Paul Neeson, PhD^1^, Alex Boussioutas^1^

###### ^*1*^*Peter MacCAllum Cancer Center, Melbourne, Australia;*^*2*^*Hudson Institute of Medical Research, Melbourne, Australia* ; ^*3*^*Monash University, Melbourne, Australia* ; ^*4*^*Shanghai Jiao Tong University, Shanghai, China* ; ^*5*^*The University of Melbourne, Melbourne, Australia*

####### **Correspondence:** Alex Boussioutas (alex.boussioutas@petermac.org)

**Background**

Immune checkpoint inhibitor (anti-PD1) therapy is approved for use in MSI(hi) and EBV+ advanced metastatic gastric cancer (GC) patients; however it is not known which GC patient sub-group with primary disease would best respond in the adjuvant setting. To address this issue, we assessed the immune context data of primary GC using high dimensional data analysis in a clinically comprehensive cohort of gastric cancer patients.

**Methods**

Using a clinically annotated primary GC cohort [1], we analyzed the immune context of each tumor using multiplex immunohistochemistry (mIHC) and transcriptome analysis (Affymetrix array). We assessed immune cell type, density and spatial relationships with tumor cells; immune cells identified included T cells (CD8+, CD4+, CD4+FoxP3+ and CD8-CD4-) and CD3-CD56+ cells. We correlated the mIHC, transcriptome analysis and clinical data to define a GC immune signature associated with good clinical outcome. Using mass cytometry, we also investigated systemic immune changes associated with the good vs poor GC outcome patients (Figure 1A-E).

**Results**

Increased GC patient survival correlated with infiltration of the tumor core by significantly increased T cells, in particular CD4+FOXP3+ T cells (Figure 1F). In the GC tumor core of patients with a good clinical outcome, spatial analysis showed increased CD4+FOXP3+ T cells were closely associated with CD8+ T cells, and not tumor cells (Figure 2A-B). We confirmed the same finding in a separate validation cohort (Figure 2C). Furthermore, increased tumor core CD8+ and CD4+FOXP3+ T cells (CD8highCD4FOXP3high) was an independent predictor for prolonged GC patient survival, regardless of established clinical variables (Figure 2D-E). Gene ontology analysis revealed CD8highCD4FOXP3high GC was strongly associated with an interferon-gamma (INF-γ) gene signature (Figure 3A-B), this INF-γ gene signature was also predictive of better survival in a combined analysis of publicly available GC datasets (Fig 3C), increased GC tumor PDL1 expression (Figure 3D) and has also been predictive of response to anti-PD-1 therapy [2]. Finally, systemic immune system changes were observed in CD8highCD4FOXP3high GC patient tumors, including significantly increased effector and central memory CD4+ T cells, and decreased PDL1+ dendritic cells (Figure 4A-C). Finally, a model depicts primary GC immune context features associated with good vs poor clinical outcome (Figure 4D).

**Conclusions**

Our study showed a new biomarker (CD8highCD4FOXP3high) in primary GC patients for good clinical outcome, this biomarker was prognostic across all GC molecular sub-groups. Importantly, the biomarker associated gene signature indicates there is potential for immunotherapy strategies in the adjuvant setting to improve GC patient outcomes.

**References**

1. Pattison, S., et al., Early relapses after adjuvant chemotherapy suggests primary chemoresistance in diffuse gastric cancer. PLoS One. 2017; 12(9): p. e0183891.

2. Ayers, M., et al., IFN-γ–related mRNA profile predicts clinical response to PD-1 blockade. J Clin Invest. 2017; 127(8): p. 2930-2940.

**Ethics Approval**

This study was approved by the Peter MacCallum Cancer Center Human Research Ethics committee, approval number 12/25

**Fig. 1 (abstract P825).**
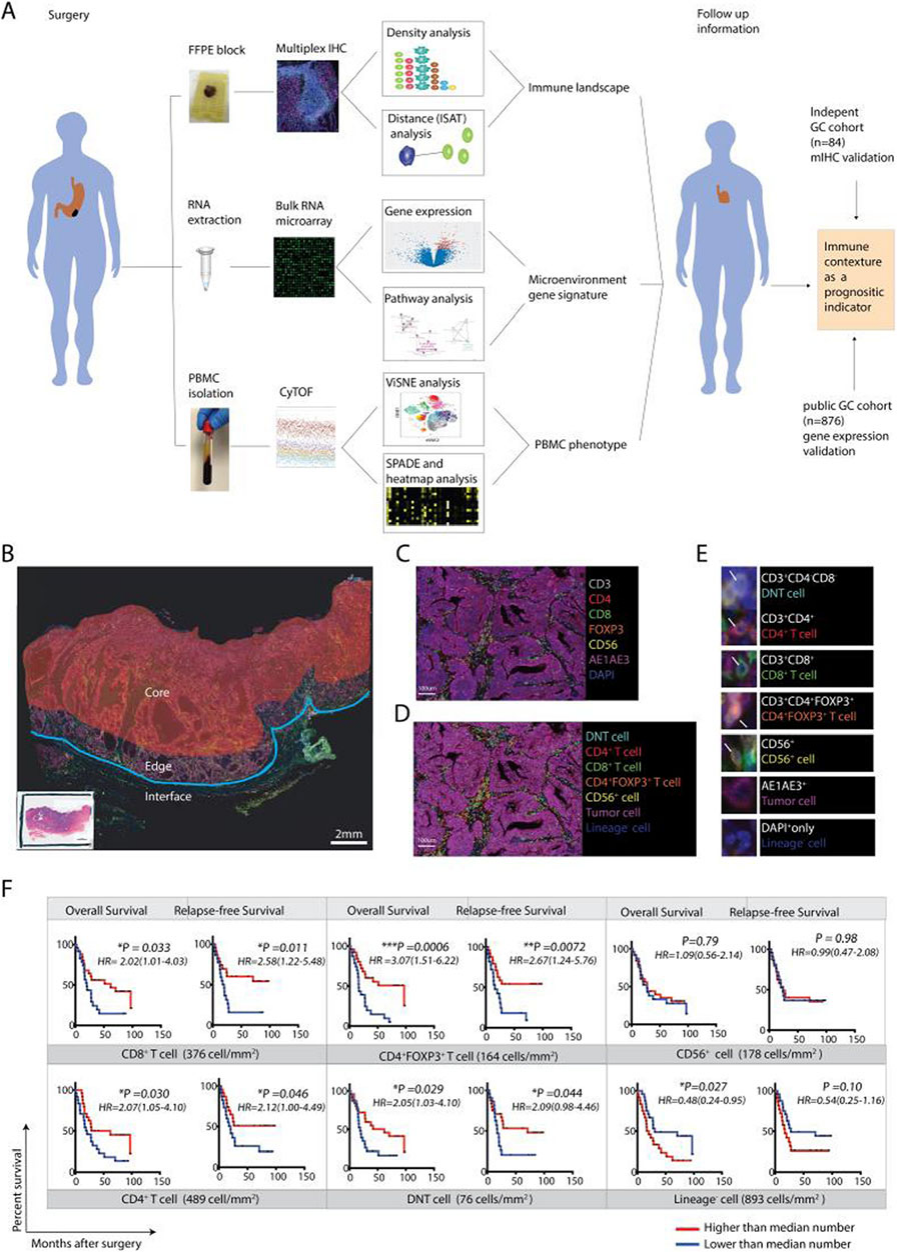
CD4+FoxP3+ T cells indicate good outcome in GC

**Fig. 2 (abstract P825).**
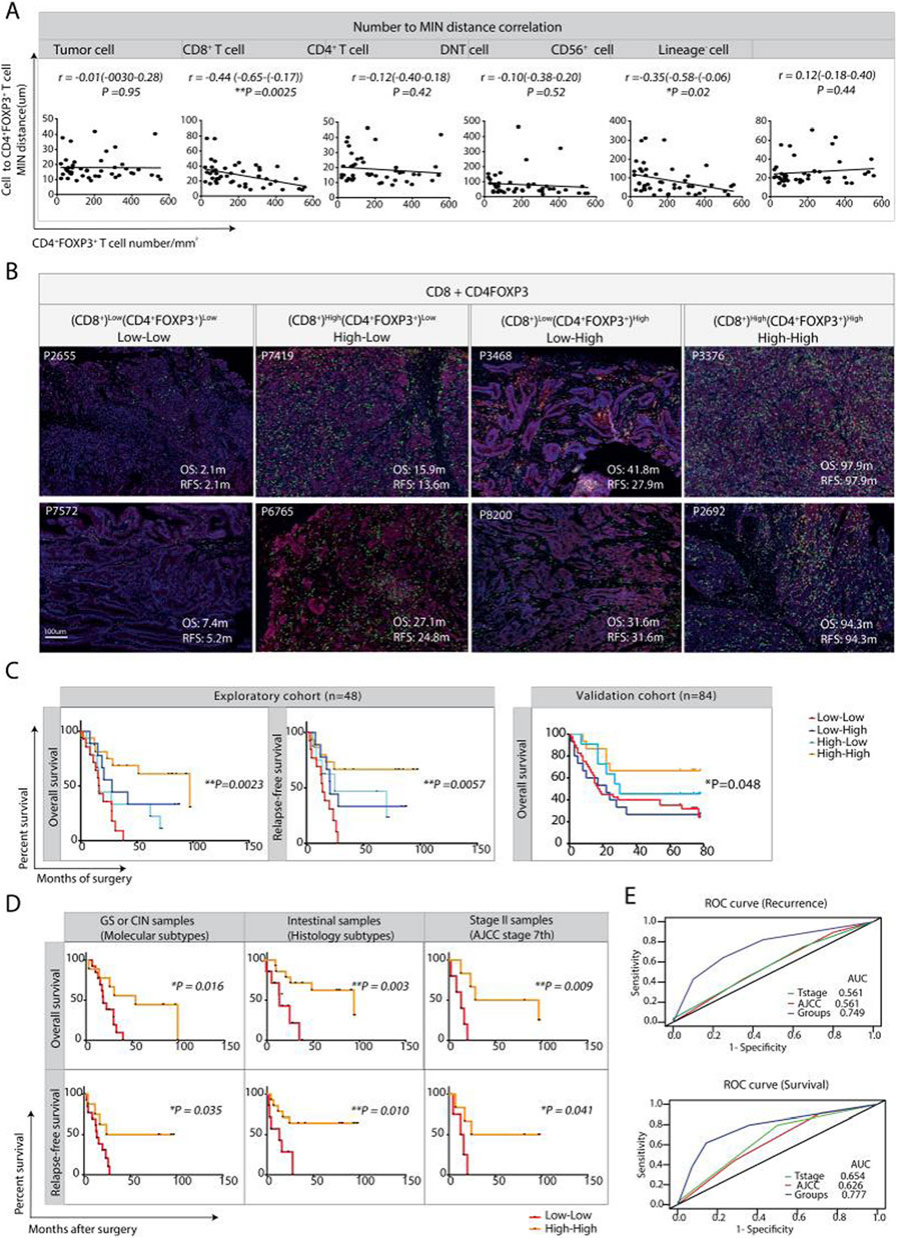
CD8+FoxP3CD4 biomarker defines good outcome patients

**Fig. 3 (abstract P825).**
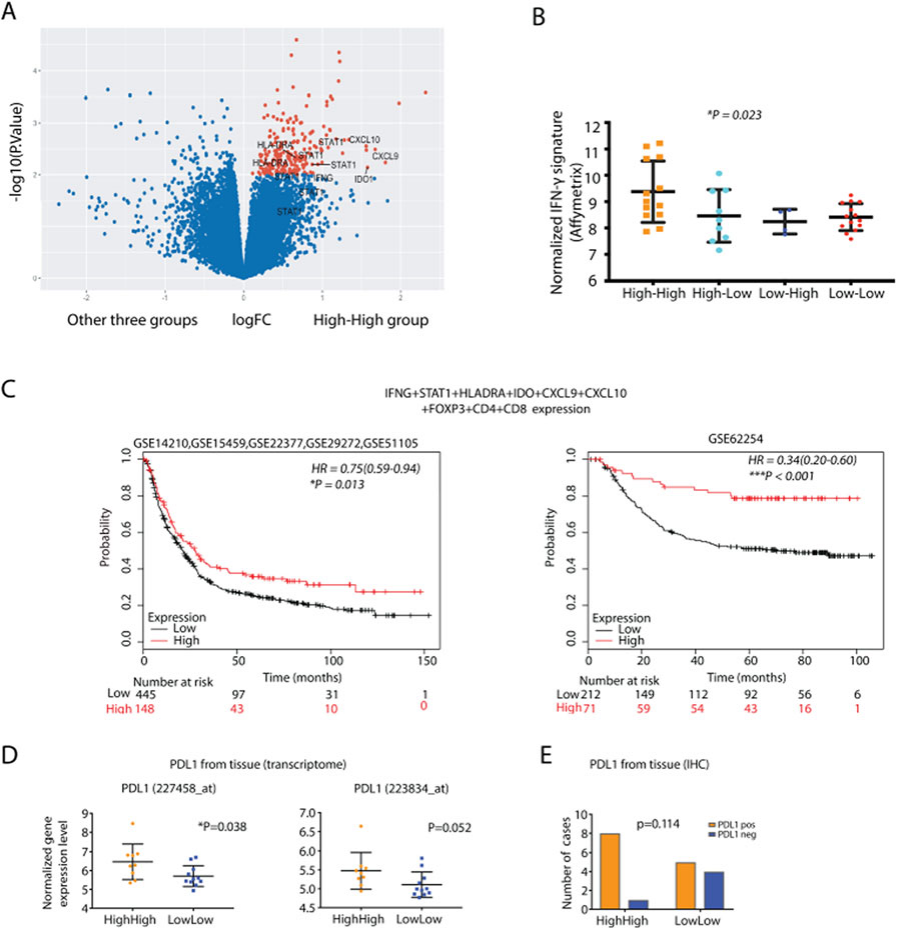
CD8+FoxP3CD4 tumours have a T cell inflamed gene signature

**Fig. 4 (abstract P821).**
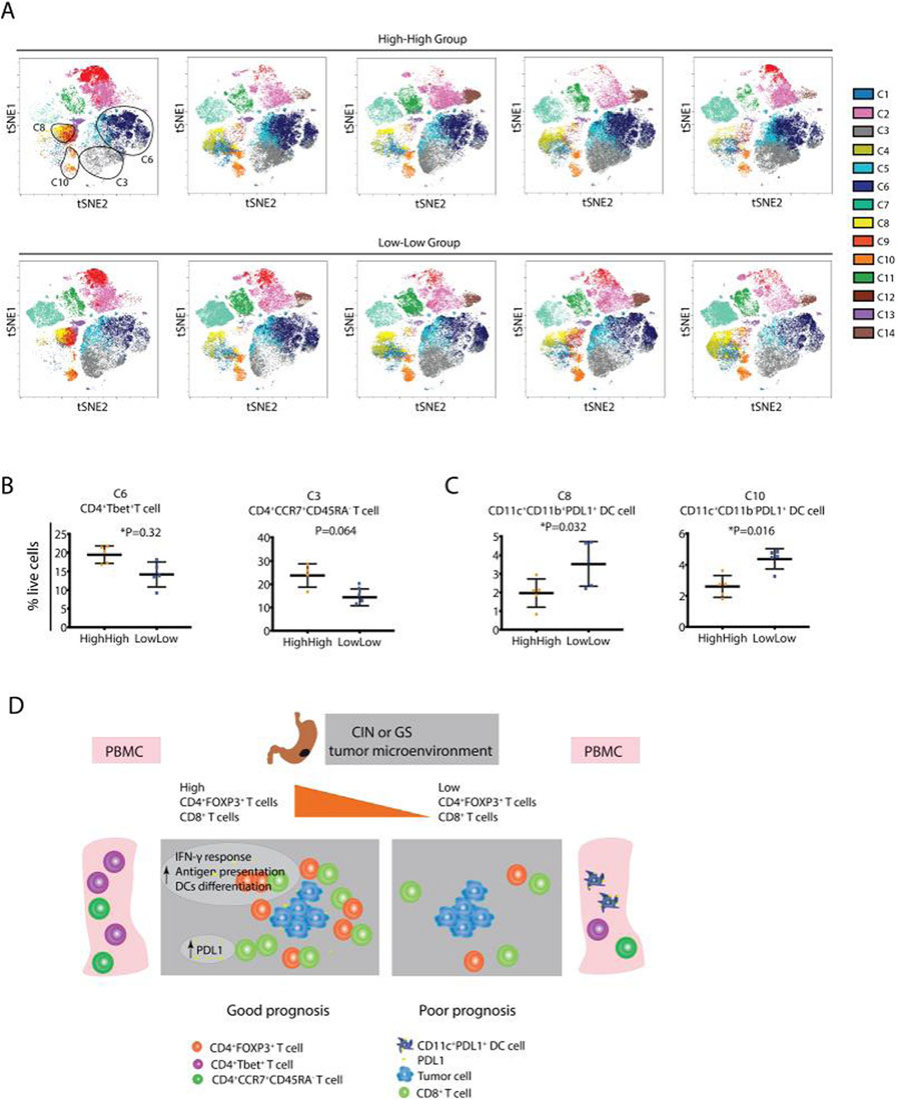
Systemic immunity in GC patients with a good outcome

#### P826 Preliminary results of a Phase 2 study of INCMGA00012 in patients with squamous carcinoma of the anal canal (SCAC) who have progressed following platinum-based chemotherapy (NCT03597295)

##### Sheela Rao^1^, Jaume Capdevila^2^, Duncan Gilbert^3^, Stefano Kim^4^, Laetitia Dahan^5^, Talal Kayyal^6^, Marwan Fakih, MD^7^, Anne Demols^8^, Mark Cornfeld, MD, MPH^9^, Chuan Tian, PhD^9^, Melissa Catlett^9^, Jean-Philippe Spano^10^

###### ^*1*^*Royal Marsden NHS Foundation Trust, London, United Kingdom;*^*2*^*Vall d'Hebron University Hospital, Barcelona, Spain* ; ^*3*^*Royal Sussex County Hospital, Brighton, United Kingdom* ; ^*4*^*Centre Hospitalier Universitaire de Besançon, Besançon, France* ; ^*5*^*CHU La Timone, Marseille, France* ; ^*6*^*Renovatio Clinical Consultants, LLC, Houston, TX, United States* ; ^*7*^*City of Hope National Medical Center, Duarte, CA, United States* ; ^*8*^*Hôpital Erasme, Brussels, Belgium* ; ^*9*^*Incyte Corporation, Wilmington, DE, United States* ; ^*10*^*Pitie-Salpetriere Hospital, Paris, France*

####### **Correspondence:** Sheela Rao (Sheela.Rao@rmh.nhs.uk)

**Background**

Background: A standard treatment for refractory advanced SCAC has not yet been established; however, preliminary Phase 1b/2 results with PD-1 inhibitors showed promising activity [1, 2]. INCMGA00012 is a humanized IgG4 monoclonal antibody that recognizes human PD-1 and has demonstrated acceptable tolerability with evidence of clinical activity in a Phase 1 study of solid tumors (NCT03059823) [3, 4, 5, 6]. This trial is evaluating the safety and efficacy of INCMGA00012 in patients with locally advanced or metastatic SCAC, including those with well-controlled HIV infection.

**Methods**

Methods: This is a single-arm Phase 2 trial in patients ≥18 years old with disease progression during or following 1 or 2 prior systemic treatments for metastatic SCAC, which must have included a platinum-based treatment. Patients with well-controlled HIV disease are eligible. All patients have measurable disease per RECIST v1.1. Approximately 81 patients will be enrolled. INCMGA00012 is administered as a flat dose at the recommended phase 2 dose (RP2D) of 500 mg Q4W for up to 2 years. The primary endpoint of the study is overall response rate (ORR) according to RECIST v 1.1. HIV control will be assessed as an exploratory endpoint.

**Results**

Results: As of July 22 2019, 32 patients have received at least one dose of INCMGA00012. Median (range) age was 64 (39-81) years. The majority of patients were female (68.8%), white (90.9%), and had ECOG performance status of 0 (28.1%), 1 (68.8%), or 2 (3.1%). Three patients were known to be HIV-positive. Median time on treatment was 2 months (0.14, 10.25). Updated safety and efficacy data will be reported at the meeting.

**Conclusions**

Conclusions: Immunotherapy holds promise as a treatment for platinum-refractory SCAC. INCMGA00012 is an investigational PD-1 inhibitor with favorable pharmacology that is being developed for SCAC and a wide range of solid tumors. Preliminary results from this large Phase 2 trial will be presented. This trial will provide important clinical data for a rare tumor type with limited treatment options.

**Acknowledgements**

Clinical trial management was provided by Tristan Richard (Incyte Corporation, Wilmington, DE)

**Trial Registration**

NCT03597295

**References**

1. Morris VK, Salem ME, Nimeiri H, et al. Nivolumab for previously treated unresectable metastatic anal cancer (NCI9673): a multicentre, single-arm, phase 2 study. Lancet Oncol. 2017; 18(4):446-453.

2. Ott PA, Piha-Paul SA, Munster P, et al. Safety and antitumor activity of the anti-PD-1 antibody pembrolizumab in patients with recurrent carcinoma of the anal canal. Ann Oncol. 2017; 28(5):1036-1041.

3. Lakhani N, Mehnert J, Rasco D, et al. A phase 1 study of the safety, tolerability, and pharmacokinetics (PK) of MGA012 (anti-PD-1 antibody) in patients with advanced solid tumors. J Immunother Cancer. 2017; 5(Suppl 2):Abstract P249.

4. Mehnert J, Joshua A, Lakhani N, et al. First-in-human Phase 1 study of INCMGA00012 in patients with advanced solid tumors: interim results of the cohort expansion phase. Abstract P669 presented at: The Society for Immunotherapy of Cancer; November 7-11, 2018; Washington, DC.

5. Condamine T, Owens S, Feldman P, et al. Pharmacodynamic correlates in a phase I study of INCMGA00012, a PD-1 antagonistic monoclonal antibody. Presented at: AACR Annual Meeting 2019, Atlanta, GA. Poster CT-085.

6. Chen X, Wang P, Kaul S, Sumrow B, Yeleswaram S. Assessment of flat dosing strategy for INCMGA00012 in patients with advanced tumors. Presented at: AACR Annual Meeting 2019; Atlanta, GA. Abstract LB268.

**Ethics Approval**

The study was approved by institutional review boards or independent ethics committees of participating institutions.

#### P827 Phase 1 study of the CD40 agonist monoclonal antibody (mAb) CDX-1140 alone and in combination with CDX-301 (rhFLT3L) in patients with advanced cancers

##### Rachel Sanborn, MD^1^, Nashat Gabrail, MD^2^ , Mark O'Hara, MD^3^ , Nina Bhardwaj, MD, PhD^4^ , Michael Gordon, MD^5^ , Ralph Hauke, MD^6^ , Rodolfo Bordoni, MD^7^ , Danny Khalil, MD PhD^8^ , Tracey Rawls^9^ , Laura Vitale, BS^9^ , Richard Gedrich^9^ , Thomas Hawthorne^10^ , Tibor Keler, PhD^9^ , Eirc Forsberg, BS^9^ , Lawrence Thomas, PhD^11^ , Michael Yellin, MD^9^

###### ^1^Providence Cancer Institute, Portland, OR, United States; ^2^Gabrail Cancer Center, Canton, OH, United States ; ^3^Hospital of the University of PA, Philadelphia, PA, United States ; ^4^Icahn School of Medicine at Mount Sinai, New York, NY, United States ; ^5^HonorHealth Research Institute, Scottsdale, AZ, United States ; ^6^Nebraska Cancer Specialists, Omaha, NE, United States ; ^7^Northside Hospital, Inc., Marietta, GA, United States ; ^8^Memorial Sloan Kettering Cancer Center, New York, NY, United States ; ^9^Celldex Therapeutics, Inc., Beacon, NY, United States ; ^10^CelldexTherapeutics, Inc., Hampton, NJ, United States ; ^11^Celldex Therapeutics, Inc, Needham, MA, United States

####### **Correspondence:** Nashat Gabrail (cantondesk@gabrailcancercenter.com), Mark O'Hara (mark.ohara@uphs.upenn.edu), Nina Bhardwaj (nina.bhardwaj@mssm.edu), Michael Gordon (michael.gordon@honorhealth.com), Ralph Hauke (rhauke@nebraskacancer.com), Rodolfo Bordoni (rodolfo.bordoni@wellstar.org), Danny Khalil (dannykhalil@gmail.com), Tracey Rawls (trawls@celldex.com), Laura Vitale (lvitale@celldex.com), Richard Gedrich (rgedrich@celldex.com), Thomas Hawthorne (thawthorne@celldex.com), Tibor Keler (tkeler@celldex.com), Eirc Forsberg (eforsberg@celldex.com), Lawrence Thomas (lthomas@celldex.com), Michael Yellin (myellin@celldex.com)

**Background**

CD40 plays multiple roles in anti-tumor immune responses, including within the tumor microenvironment (TME). Agonist anti-CD40 mAbs have demonstrated anti-tumor activity in pre-clinical and clinical studies. CDX-1140 is a human IgG2 anti-CD40 mAb with unique characteristics selected to achieve optimal agonist activity at doses associated with TME penetration. CDX-1140 may synergize with CDX-301, a dendritic cell growth factor known to increase CD141+ DCs critical for tumor antigen cross-presentation.

**Methods**

A Phase 1 dose-escalation (DE)/expansion study evaluating safety, PK, PD, and clinical activity of CDX-1140 monotherapy (advanced solid tumors/NHL) and in combination with CDX-301 (advanced solid tumors) (NCT03329950). DE evaluates CDX-1140 0.01-3.0 mg/kg (monotherapy) and 0.09-3.0 mg/kg (combination) Q4W. In combination DE, CDX-301 75 mcg/kg sc daily x 5 is given prior to the first two CDX-1140 infusions. Biomarker assessments include characterizing effects on peripheral blood immune cells and cytokines and on the TME in paired tumor biopsies. Backfill and/or expansion cohorts will further explore the activity of monotherapy and combination.

**Results**

CDX-1140 monotherapy DE through 3.0 mg/kg across 10 tumor types (n=32; 30 solid tumor/2 NHL) is complete without reaching a MTD. Treatment related AEs (>20%) were arthralgia, nausea and fatigue. Transient and mostly low-grade increases in liver transaminases were seen in some patients. Treatment-related SAEs in ≥ 1 monotherapy patient included: fatigue (n=2; both grade 3), cytokine release syndrome (n=2; grade 2 and 3), pneumonitis (n=2; both grade 3), nausea (n=1; grade 3) and pyrexia (n=1; grade 1). CDX-1140 exposure increased with dose (T1/2 ranged from 0.4-3.0 days). Serum cytokine increases and changes in peripheral immune cells demonstrated immune activation consistent with CD40 agonism. Of the clinically evaluable monotherapy patients (n=24), four had SD lasting up to 5.4 months.

In combination, 14 patients have been dosed through 0.72 mg/kg of CDX-1140 and DE is ongoing. Treatment related AEs (>20%) have been myalgia and fatigue; with no treatment related SAEs. Enhancement of some serum cytokine levels, e.g., IL-12p40, compared to CDX-1140 monotherapy, was observed. A patient with gastro-esophageal cancer treated in combination (CDX-1140 0.36 mg/kg) has an unconfirmed PR, with 41% reduction in target lesions (liver and lymph node).

**Conclusions**

To date, CDX-1140 has shown the expected PK, PD, and safety profile up to 3 mg/kg. Combination with CDX-301 has been well tolerated, with evidence of enhanced biological and clinical activity. Analyses are planned to explore the treatment effects on the TME. Additional combinations, including with checkpoint blockade, are planned.

**Trial Registration**

NCT03329950

**Ethics Approval**

The study was approved by the following IRBs: Providence Health and Services (Oregon) (#2017000532), WIRB for Gabrail Cancer Center Research (#1188814), University of Pennsylvania (# 828733), Mount Sinai School of Medicine (#18-00202), WIRB for Honor Health (#1180854), WIRB for Nebraska Cancer Specialist (#1189918), WIRB for North Side Hospital (#1249506), Memorial Sloan Kettering Cancer Center (#18-225A[2])

#### P828 Cytokine release syndrome (CRS) following treatment with tebentafusp, a novel bispecific TCR–anti-CD3 directed against gp100, in patients with advanced melanoma

##### Alexander Shoushtari, MD^1^, Mark Middleton^2^, Neil Stevens^3^, Thomas Evans^4^, Jeffrey Infante^5^, Mario Sznol, MD^6^, Alan Anthoney^7^, Avinash Gupta^8^, Victoria Woodcock^2^, Tina Wiseman^9^, Cheryl McAlpine, MSN^10^, Chris Cohen^10^ , Omid Hamid, MD^12^

###### ^1^Memorial Sloan Kettering Cancer Center, New York, NY, United States; ^2^University of Oxford, Oxford, United Kingdom ; ^3^University of Birmingham, Birmingham, United Kingdom ; ^4^University of Glasgow, Glasgow, United Kingdom ; ^5^Janssen, Spring Hill, PA, United States ; ^6^Yale Cancer Center, New Haven, CT, United States ; ^7^Leeds Teaching Hospitals NHS Trust, Leeds, United Kingdom ; ^8^The Christie NHS Foundation Trust, Manchester, United Kingdom ; ^9^Immuncore Ltd, Conshohocken, PA, United States ; ^10^Immunocore Ltd, Milton, United Kingdom ; ^11^The Angeles Clinic and Research Institute, Los Angeles, CA, United States

####### **Correspondence:** Chris Cohen (chris.cohen@immunocore.com)

**Background**

In early clinical studies, tebentafusp (IMCgp100; a TCR-anti-CD3 bispecific ImmTAC against melanocyte-associated lineage antigen gp100) showed monotherapy activity in advanced melanoma with mechanism-related changes in circulating cytokines and T cells [1]. Grouping of cytokine-mediated AEs across bispecific clinical programs has been inconsistent. In FIH study IMCgp100-01, investigators reported infrequent, severe CRS (1%) and separately reported other cytokine-mediated AEs. To understand kinetics, outcome and biologic correlates of cytokine-mediated AEs, we reviewed IMCgp100-01 using the Lee grading criteria [2], which groups these AEs under the umbrella term CRS.

**Methods**

84 HLA-A2+ advanced melanoma patients received tebentafusp. Per protocol, patients did not receive corticosteroids as CRS prophylaxis. CRS was evaluated post-hoc according to Lee [2]. Circulating cytokines were measured in serum taken over the first 24-48 hours (n=69).

**Results**

The most frequent treatment-related AEs (any grade) per investigator that are also cytokine mediated per Lee criteria were mostly mild to moderate and included fever (57%), fatigue (42%), nausea (40%), headache (23%). Investigators separately reported 3 cases of CRS (G1=2, G3=1). Based on sponsor review using Lee criteria, we find a similar incidence of any grade CRS (60%), the majority of which is mild (45%) generally transient fever, fatigue, nausea, headache; moderate (10.7%); with low severe (3.6%) and no G4/G5.

CRS generally started within 24 hours of tebentafusp administration, typically resolved within 3 days, and most commonly occurred after the first dose with decreased frequency and severity after subsequent doses. Patients were able to continue tebentafusp treatment despite CRS (2 patients discontinued due to CRS). Treatment of ≥G2 CRS included intravenous fluids (19 patients), intravenous steroids (7 patients), oxygen (5 patients), and epinephrine (1 G3 patient). No patients received tocilizumab. Six patients achieved a partial response (three each had G2 and G1 CRS). ≥G2 CRS was not associated with age, baseline absolute lymphocyte count, or disease burden. Consistent with the hypothesized mechanism of action supported by preclinical data, tebentafusp induced transient increases in peripheral cytokines within hours of administration, which tended to be greater in patients with higher grade CRS.

**Conclusions**

We confirmed the low incidence of severe CRS reported by investigators and regrouped cytokine-mediated AEs under the umbrella of CRS. Without mandatory corticosteroid pre-treatment, CRS was generally low grade, reversible with standard management including IVF and short course corticosteroids, decreased in frequency and severity after initial doses and infrequently led to discontinuation. Patients with higher grade CRS tended to have greater increases in serum cytokines.

**Trial Registration**

NCT01211262

**References**

1. Middleton MR, Steven NM, Evans JT, et al. Relationship between clinical efficacy and AEs of IMCgp100, a novel bispecific TCR–anti-CD3, in patients with advanced melanoma. Presented at ASCO 2019. Abstract 9523

2. Lee DW, Gardner R, Porter DL, et al. Current concepts in the diagnosis and management of cytokine release syndrome. Blood. 2014;124:188–195.

**Ethics Approval**

This study was approved by following institutions’ Ethics Boards:

Oxfordshire Research Ethics Committee; 10/H0604/47, Approved June 4, 2010.

Mary Crowley Cancer Research Center; MCMRC IRB # 12-06, Approved March 16, 2012.

Human Investigation Committee, Yale University; HIC Protocol # 1302011504, Approved March 22, 2012.

IntegReview; Protocol No IMCgp100/01, Approved November 13, 2013.

Western Sydney Local Health District; HREC2012/7/4.1 (3552) AU RED HREC/12/WMEAD/237, Approved on October 24, 2012.

Western Institutional Review Board; Panel 1, Study Num 1147687, WIRB Pro Num 20141184, Approved July 15, 2014.

Memorial Sloan Kettering Cancer Center, Institutional Review Board; Protocol # 14-152, August 28, 2014.

#### P829 The pattern of expression of hMENA isoforms impacts tumor immune microenvironment of early node-negative non-small cell lung cancer

##### Sheila Spada, PhD, Francesca Di Modugno, PhD, Anna Di Carlo, Isabella Sperduti, Enzo Gallo, Mariangela Panetta, Belinda Palermo, Francesca De Nicola, Lorenzo D'Ambrosio, Barbara Antoniani, Paola Trono, Francesco Facciolo, Paolo Visca, Paola Nistico', MD

###### Regina Elena National Cancer Institute, Rome, Italy

####### **Correspondence:** Paola Nistico' (paola.nistico@ifo.gov.it)

**Background**

Actin cytoskeleton dynamics act as platforms for gene regulation and key signaling transduction pathways involved in the cross-talk among tumor cells and cellular and non-cellular components of the tumor microenvironment (TME). The actin regulatory protein hMENA undergoes tissue specific splicing, generating two alternatively expressed isoforms hMENA11a and hMENAΔv6 that respectively inhibit or increase cell invasiveness [1], TGFβ [2] and β1 integrin signaling and the secretion of several key extracellular matrix (ECM) proteins [3] and is crucial in TGFβ mediated-EMT [2]. Early node-negative NSCLC patients show a prolonged disease-free survival when expressing high tumor hMENA11a/low stromal FN1[3]. Recently, tertiary Lymphoid Structures (TLS), sites of transient lymphoid neo-genesis and determinants of antitumor immunity, have been associated with a favorable clinical outcome in NSCLC patients [4].

**Methods**

The pattern of hMENA isoform expression, TLS presence and localization and stromal fibronectin were evaluated in 94 primary tumors of node negative NSCLC patients by immunohistochemistry. We evaluated the role of hMENA isoform expression on lymphotoxin beta receptor (LTBR), by gain and loss of function, RNA-SEQ and QRT-PCR.

**Results**

We found that TLS localized within the tumor area (TLS-AT) are predictive of a longer disease free survival in N0 NSCLC patients, whereas the presence of peritumoral TLS (TLS-PT) does not associate with the patient survival, but with higher tumor grade. TLS-AT presence is significantly correlated with hMENA11a expression in tumor cells, whereas the presence of TLS-PT correlates with the absence of hMENA11a. We evidenced that a low level of stromal FN along with the expression of hMENA11a, associated with TLS-AT.

Searching for the mechanisms involved, we found that the hMENA11a knock out in NSCLC cells modulates the expression of several genes: down-regulates the TLS regulator LTβR and up-regulates FN1. Finally, we found that the depletion hMENA11a in the H1650 NSCLC cells induces the reduction of the chemokine CXC-chemokine ligand 13 (CXCL13) a critical cytokine induced after LTβ-LTβR signalling [5] [6].

**Conclusions**

Our findings indicate that the alternative splicing of hMENA is crucial in the reciprocal signaling between tumor cells and their immune microenvironment, by participating in tertiary lymphoid structure neo-genesis and spatial distribution.

**Acknowledgements**

This work is supported by AIRC

**References**

1. Di Modugno F, Iapicca P, Boudreau A, Mottolese M, Terrenato I, Perracchio L, Carstens RP, Santoni A, Bissell MJ, Nisticò P. Splicing program of human MENA produces a previously undescribed isoform associated with invasive, mesenchymal-like breast tumors. Proc Natl Acad Sci U S A. 2012; 109(47):19280-5.

2. Melchionna R, Iapicca P, Di Modugno F, Trono P, Sperduti I, Fassan M, Cataldo I, Rusev BC, Lawlor RT, Diodoro MG, Milella M, Grazi GL, Bissell MJ, Scarpa A, Nisticò P. The pattern of hMENA isoforms is regulated by TGF-β1 in pancreatic cancer and may predict patient outcome. Oncoimmunology. 2016; 5(12):e1221556.

3. Di Modugno F, Spada S, Palermo B, Visca P, Iapicca P, Di Carlo A, Antoniani B, Sperduti I, Di Benedetto A, Terrenato I, Mottolese M, Gandolfi F, Facciolo F, Chen EI, Schwartz MA, Santoni A, Bissell MJ, Nisticò P. hMENA isoforms impact NSCLC patient outcome through fibronectin/β1 integrin axis. Oncogene 2018; 37: 5605-5617.

4. Dieu-Nosjean MC, Giraldo NA, Kaplon H, Germain C, Fridman WH, Sautès-Fridman C. Tertiary lymphoid structures, drivers of the anti-tumor responses in human cancers. Immunol Rev. 2016;271(1):260-75.

7. Dejardin E, Droin NM, Delhase M, Haas E, Cao Y, Makris C, Li ZW, Karin M, Ware CF, Green DR. The lymphotoxin-beta receptor induces different patterns of gene expression via two NF-kappaB pathways. Immunity 2002; 17: 525-535.

8. Tang H, Zhu M, Qiao J, Fu YX. Lymphotoxin signalling in tertiary lymphoid structures and immunotherapy. Cell Mol Immunol 2017; 14: 809-818.

**Ethics Approval**

This study was approved by the ‘Comitato Etico Centrale IRCCS Lazio’; approval number: ifo_058.IFO_AOO.REGISTRO UFFICIALE.U.0012817 20-11-2017

#### P830 Optimization of RG1-VLP vaccine performance in mice with novel TLR4 agonist-based adjuvants

##### Jason Marshall, PhD , Athina Zacharia, PhD

###### Leidos, Inc., Frederick MD, United States

####### **Correspondence:** Jason Marshall (jason.marshall2@nih.gov)

**Background**

We are testing in vitro 2 candidate vaccine adjuvants (lipid A structures) that are generated by using bacterial enzymatic combinatorial chemistry (BECC). We have selected two BECC compounds (438 and 470) designed by Dr. Bob Ernst at the University of Maryland, Baltimore to be used as adjuvants to the RG1-VLP vaccine for the prevention of human papillomavirus (HPV) infection and associated cancers. Adjuvantation of the RG1-VLP vaccine formulated with aluminum hydroxide and the BECC compounds could result in a more effective HPV vaccine that induces more robust and longer-lasting protection, increases HPV strain-specific coverage, allows fewer injections and decreases manufacturing costs.

**Methods**

Mice were vaccinated 3X IM with 2 ug RG1-VLP + 25 ug of BECC438 or BECC470 formulated +/- Alhydrogel (alum) and compared to alum alone or PHAD, a commercial TLR4L. HPV16 L1 subunit and RG1 L2 epitope-specific Ab titers were measured in the sera by ELISA.

Neutralizing Ab titers were measured using a cell-based assay in which antisera from vaccinated mice were used to inhibit in vitro infection of LoVo-T cells with HPV pseudovirions expressing alkaline phosphatase.

In study 2, blood was collected at earlier time points to determine if the addition of BECC compounds induced significant humoral immunity at earlier time points.

After 3X IM vaccinations with RG1-VLPs + alum or alum/BECC, spleens were harvested and HPV16 L1 VLP-specific IFN-g ELISPOT was performed on splenocytes.

**Results**

BECC compounds improve HPV16-L2-specific Ab production in mice

BECC compounds improve neutralizing Ab production to several HPV strains

BECC compounds accelerate anti-HPV humoral responses

BECC compounds accelerate appearance of HPV-neutralizing Abs

Induction of T cell responses by BECC

**Conclusions**

Combining the adjuvants Alhydrogel and BECC compounds increases the magnitudes of antibodies specific to HPV16 L1 and the RG1-specific L2 epitope as well as of neutralizing titers to HPV16/18/39/6.

BECC470 outperforms BECC438 as well as standard TLR4 agonists PHAD and 3D-PHAD to augment anti-HPV humoral responses in several key assays.

BECC470 accelerates the appearance of L1 and L2 antibody levels after 2 vaccinations to equal or exceed levels achieved by Alhydrogel alone after 3 vaccinations.

The presence of BECC470 also induces a substantial T cell response specific to HPV16 L1 VLPs that is absent with Alhydrogel alone.

Adjuvant optimization of the RG1-VLP vaccine with TLR4 agonist BECC compounds may lead to VLP dose sparing and an accelerated schedule, resulting in enhanced protective immunity while saving time and cost of goods.

### Regulatory, Financial, and Access Considerations

#### P831 Inclusion of people living with HIV in anti-PD(L)1 clinical trials: Results of an NCI-driven initiative to broaden eligibility in clinical trial development

##### Joshua Reuss, MD, Diana Stern, MD^2^, Ramya Ramaswami^3^, Kathryn Lurain, MD^3^, Helen Chen, MD^3^, Howard Streicher, MD^3^, Ravie Kem, RPh, Pharm D, MPH^3^, Richard Little, MD^3^, Elad Sharon, MD, MPH^3^

###### ^1^Johns Hopkins University School of Medicine, Baltimore, MD, United States ; ^*2*^*Yale New Haven Health System, Bridgeport, CT, United States* ; ^*3*^*NIH/NCI, Bethesda, MD, United States*

####### **Correspondence:** Elad Sharon (sharone@mail.nih.gov)

**Background**

Certain cancers occur at higher rates in people living with HIV (PLWH) as compared with the general population and are a leading cause of mortality among PLWH in developed countries [1]. Anti-programmed death (ligand) 1 (PD-(L)1) therapies are approved to treat cancers that affect PLWH, however HIV is a common exclusion criterion in clinical trials. Collaborative efforts by multiple professional groups have led to recently published guidelines on modernizing clinical trial eligibility to include PLWH [2,3]. In 2007 the National Cancer Institute (NCI) Cancer Therapy Evaluation Program (CTEP) began efforts to include PLWH in clinical trials culminating with changes to its formal protocol templates in early 2018. We reviewed CTEP approved final protocols for anti-PD(L)1 agents and compared HIV-specific eligibility criteria in the initial letter of intent (LOI) with the final protocol to evaluate the effectiveness of CTEP policy to include PLWH in immunotherapy clinical trials.

**Methods**

LOIs including anti-PD(L)1 agents (nivolumab, pembrolizumab, atezolizumab, durvalumab) submitted to CTEP with study approval between January 2014 and May 2018 were included in this analysis. Only LOIs approved for protocol development that proceeded to final activated clinical trials were included. Studies designed specifically for PLWH were excluded. We compared the frequency of PLWH inclusion at initial LOI submission and final approved protocol.

**Results**

Eighty-seven studies were included in this analysis; 39 included nivolumab, 23 pembrolizumab, 19 atezolizumab, and 6 durvalumab. Sixty-eight trials were pilot, phase I, phase I/II, or phase II. Nineteen studies were phase II/III or III. At the LOI-stage, 14 of 87 proposals (16%) included PLWH. Eleven of 68 pilot/I/II LOIs (16%) and 3 of 19 phase II/III or III LOIs (16%) included PLWH. After CTEP policy intervention, 61 of 87 final approved protocols (70%) included PLWH. Forty-seven of 68 pilot/I/II protocols (69%) and 14 of 19 phase II/II or III protocols (74%) included PLWH. Also, of 36 LOIs that initially excluded PLWH, 24 (67%) included PLWH in their final approved protocols.

**Conclusions**

CTEP advocacy had a meaningful impact on inclusion of PLWH in anti-PD(L)1 clinical trials independent of study phase. These data demonstrate that collaborative efforts lead to inclusion of underrepresented populations in clinical trials and suggest interventions for other underrepresented populations including patients with other chronic viral infections, brain metastases, and end-organ dysfunction can be effective. These data also demonstrate that efforts to include PLWH have not yet reached parity with other comorbid conditions, such as diabetes or heart disease.

**References**

1. Silverberg MJ, Lau B, Achenbach CJ, Jing Y, Althoff KN, D'Souza G, Engels EA, Hessol NA, Brooks JT, Burchell AN, Gill MJ, Goedert JJ, Hogg R, Horberg MA, Kirk GD, Kitahata MM, Korthuis PT, Mathews WC, Mayor A, Modur SP, Napravnik S, Novak RM, Patel P, Rachlis AR, Sterling TR, Willig JH, Justice AC, Moore RD, Dubrow R. Cumulative incidence of cancer among persons with HIV in North America: A cohort study. Ann Intern Med. 2015;163(7):507-18.

2. Persad GC, Little RF, Grady C. Including persons with HIV infection in cancer clinical trials. J Clin Oncol. 2008;26(7):1027-32.

3. Uldrick TS, Ison G, Rudek MA, Noy A, Schwartz K, Bruinooge S, Schenkel C, Miller B, Dunleavy K, Wang J, Zeldis J, Little RF. Modernizing clinical trial eligibility criteria: Recommendations of the american society of clinical oncology-friends of cancer research HIV working group. J Clin Oncol. 2017;35(33):3774-80.

#### P832 Systematic literature review (SLR) of intravenously (IV) administered oncology therapies for which a subcutaneous (SC) formulation has also been developed

##### Brad Schenkel, MSc^1^ , Elise Wu^1^, Ali McBride^2^, Marília Hernani^3^, Robin Wyn^3^, Klaudia Kornalska^3^

###### ^*1*^*Bristol Myers-Squibb, Lawrence Township, NJ, United States;*^*2*^*The University of Arizona Cancer Center, Tucson, AZ, United States* ; ^*3*^*Adelphi Values PROVE, Bollington, United Kingdom*

####### **Correspondence:** Brad Schenkel (Brad.Schenkel@bms.com)

**Background**

SC administration of immuno-oncology (I-O) therapies is being explored as a less-invasive and more-convenient alternative to IV administration. However, further research is needed to fully understand the additional benefits that the SC route can offer. This review aimed to evaluate comparative data on the efficacy, safety, and pharmacokinetics/pharmacodynamics (PK/PD) of these two administration methods in oncology, along with differences in time requirements, costs, and patient reported outcomes.

**Methods**

Literature published from 2009–2019 was identified through OVID®-indexed databases and supplemented with conference proceedings, recursive searching, and additional clinicaltrials.gov searches. Studies of any design that provided comparative data between IV and SC formulations on the following outcomes were included: efficacy, safety, PK/PD, HRQoL, time savings, adherence, persistence, preference, satisfaction, and cost.

This SLR focused on the following anti-cancer therapies, for which both IV and SC formulations are either being developed in phase 3 clinical trials or are approved by either the FDA or EMA: alemtuzumab, atezolizumab, blinatumomab, bortezomib, daratumumab, pembrolizumab, pertuzumab, rituximab, and trastuzumab.

**Results**

A total of 100 publications met the inclusion criteria. Most publications reported on trastuzumab (38), rituximab (26), or bortezomib (25); fewer publications reported on alemtuzumab (3), daratumumab (3), rituximab/trastuzumab (4), and alemtuzumab/rituximab/trastuzumab (1).

In 48 publications, SC had non-inferior efficacy relative to IV formulations; statistically significant differences were reported in only 3 publications. The relative tolerability of SC and IV formulations was also similar across 68 publications. However, in 9 publications, SC trastuzumab and rituximab appeared to be associated with higher incidence of (mostly low-grade) injection-site reactions compared to IV infusion. When pharmacokinetic outcomes were reported, overall systemic exposure to therapy was similar between formulations.

SC administration was also associated with time savings, based on 39 publications, with reduced health-care professional (HCP) time and patient chair time being major factors. In combination with other factors, such as reduced drug wastage, this led to overall cost savings with SC administration.

In 33 publications that considered preference, SC formulations were associated with higher treatment satisfaction and were preferred by patients and HCPs. Data on HRQoL, however, were rarely reported.

**Conclusions**

Our SLR suggests that SC and IV formulations had generally similar efficacy and tolerability profiles, while SC administration led to substantial cost and time savings and was usually preferred by patients and HCPs. Therefore, SC I-O therapy presents several advantages over IV, and switching from IV to SC administration could be beneficial from multiple stakeholder perspectives.

### Timing of Immunotherapy Administration

#### P833 Timing of IL-2 treatment delivery after transplantation of B16 melanoma in a murine model differentially influences tumour growth

##### Suzanne Edwards, BSc, Brendon Coventry, MD PhD

###### University of Adelaide, Adelaide, SA, Australia

####### **Correspondence:** Brendon Coventry (brendon.coventry@adelaide.edu.au)

**Background**

The immune system is a dynamic homeostatic entity which fluctuates in response to antigenic stimulation but provides overall stability, with the capacity to generate an active immune response when required. The nature of this fluctuation is currently poorly understood, but has likely significant ramifications for when antigen is delivered for vaccination, or when other immunomodulatory agents are administered to influence immune system function. Immune reactivity usually commences at first exposure to antigen, including first exposure to tumour antigens. We aimed to investigate whether there was any difference on tumour growth compared with controls according to when Interleukin-2 (IL-2) was administered after tumour transplantation (Day 0).

**Methods**

B16 F0 melanoma was transplanted into C57BL/6 mice and a peri-tumoural dose of 20,000 IU of IL-2 (or PBS vehicle control) was given on different days after transplantation to observe for any effect on tumour growth determined by the timing of the day of dose delivery. IL-2 or PBS control treatments were administered to comparator groups on days 4, 5, 6, 7, 8, 9 and 10, and again on day 14 after the day of transplantation (Day 0). A linear mixed-effects model compared ‘tumour size’ (logarithmic transformation) versus interaction of Day (categorical version) and control/treatment group, controlling for repeated measurements over time.

**Results**

IL-2 exerted a repeatable effect observed only on Day-7 after transplantation causing statistically significant reduced tumour growth over comparable control (PBS) treated animals. Control untreated tumours were over double the size of tumours treated with IL-2 administered at Day-7. The extent of this effect was not obtainable for IL-2 injection administered under identical conditions but on other days either before or after Day-7 after transplantation, indicating that a potential ‘window of sensitivity’ to immunotherapy treatment might exist.

**Conclusions**

There appears to be a differential in-vivo effect on control of tumour growth depending on the day of delivery of IL-2 after transplantation and first antigen exposure. This unusual timing effect requires further study and might have important implications for the timing of therapy delivery and could explain some of the differential variable effects observed with human immunotherapies.

### Tumor and Stromal Cell Biology

#### P834 The actin regulatory protein hMENA is a key player in the paracrine TGF-β mediated crosstalk between tumor cells and cancer associated fibroblasts

##### Roberta Melchionna^1^, Paola Trono^1^, Francesca Di Modugno, PhD^1^, Anna Di Carlo^1^, Sheila Spada, PhD^1^, Giuliana Falasca^1^, Isabella Sperduti^1^, Barbara Antoniani^1^, Daniel D'Andrea^2^, Paolo Visca^1^, Gian Luca Grazi^1^, Francesco Facciolo^1^, Aldo Scarpa^3^, Paola Nistico', MD^1^

###### ^*1*^*Regina Elena National Cancer Institute, Rome, Italy;*^*2*^*Imperial College London, London, United Kingdom*; ^*3*^*ARC-NET University of Verona, Verona, Italy*

####### **Correspondence:** Paola Nistico' (paola.nistico@ifo.gov.it)

**Background**

The bi-directional communication mediated by paracrine loop between cancer cells and cancer associate fibroblasts (CAFs) critically affects tumor growth and aggressiveness.

TGF-β is a key regulator of epithelial mesenchymal transition (EMT), an inducer of CAF activation and extracellular matrix (ECM) production, suggesting its mechanistic role in cancer cell-CAF crosstalk.

Recently, stromal-TGFβ signaling has been associated with T cell exclusion and poor response to immune checkpoint PD-1/PD-L1 blockade, suggesting the relevance of identifying stromal-derived TGF-β mediated pathways [1,2].

The actin regulatory protein hMENA has been implicated in the metastatic progression of several human cancers. Our group identified two tissue specific splicing-derived isoforms, the epithelial hMENA11a and mesenchymal hMENAΔv6 [3]. The expression of hMENAΔv6, along with the lack of hMENA11a, is crucial for TGFβ-mediated EMT [4], and the pattern of hMENA isoform expression represents a prognostic factors in PDAC and early stage NSCLC patients [4,5,6].

We have recently identified a role for hMENA/hMENAΔv6 in CAF activation and function and herein, we aimed at identifying their role in the TGFβ-mediated crosstalk between cancer cells and CAFs in NSCLC and PDAC

**Methods**

The role of hMENA isoforms in TGF-β-CAF activation was analyzed by loss and gain of function experiments. To analyze hMENA-driven regulation of TGF-β ligands (TGF-β1,TGF-β2, and TGF-β3) we employed the Bio-Plex Pro™.

To study the role of hMENA expression in CAFs on cancer cell signaling pathway activation, we cultured cell lines with sihMENA-CAF-conditioned media and analyzed their effect on the phosphorylation of AKT,ERK,STAT1/3 by immunoblotting

**Results**

We found that hMENA/hMENAΔv6 overexpression identify a subset of CAFs with pro-tumoral functions in NSCLC and PDAC. hMENA/hMENAΔv6 silencing in CAFs downregulated the expression of TGF-βR1 and 2, inhibited the TGF-β signaling activation, in turn impairing the autocrine TGF-β- mediated CAF activation and functions.

Moreover, hMENA/hMENAΔv6 silencing significantly decreased the secretion of the three TGF-β ligands, modulated TGF-β-mediated cytokine/chemokines secretion and affect the paracrine signaling pathways involved in cancer cell invasiveness. Reciprocally, hMENA silencing in NSCLC cells specifically regulates the expression and secretion of TGFβ-2 and impairs the activation of CAF-TGF-β signaling mediated by tumor cell secretoma.

**Conclusions**

In conclusion these novel findings provide the evidence for a crucial role of hMENA and its tissue specific splicing program as regulator of TGF-β-mediated cross-talk between tumor cells and CAFs. We highlight the importance of inhibiting hMENA-mediated signaling pathways also in view of the new advance of combined immunotherapy and TGF-β inhibitors in the clinical practice

**Acknowledgements**

This supported by AIRC IG (19822) and AIRC 5x1000 (P609)

**References**

1. Mariathasan, S., Turley, S.J., Nickles, D., Castiglioni, A., Yuen, K., Wang, Y., Kadel Iii, E.E., Koeppen, H., Astarita, J.L., Cubas, R., et al.. TGFβ attenuates tumor response to PD-L1 blockade by contributing to exclusion of T cells. Nature. 2018; 554: 544–548.

2. Tauriello, D.V.F., Palomo-Ponce, S., Stork, D., Berenguer-Llergo, A., Badia-Ramentol, J., Iglesias, M., Sevillano, M., Ibiza, S., Can˜ ellas, A., Hernando-Momblona, X., et al.. TGFβ drives immune evasion in genetically reconstituted colon cancer metastasis. Nature. 2018; 554: 538–543.

3. Di Modugno F, Iapicca P, Boudreau A, Mottolese M, Terrenato I, Perracchio L, Carstens RP, Santoni A, Bissell MJ, Nisticò P. Splicing program of human MENA produces a previously undescribed isoform associated with invasive, mesenchymal-like breast tumors. Proc Natl Acad Sci U S A. 2012 Nov 20; 19280-5.

4. Melchionna R, Iapicca P, Di Modugno F, Trono P, Sperduti I, Fassan M, Cataldo I, Rusev BC, Lawlor RT, Diodoro MG, Milella M, Grazi GL, Bissell MJ, Scarpa A, Nisticò P, The pattern of hMENA isoforms is regulated by TGF-β1 in pancreatic cancer and may predict patient outcome. Oncoimmunology. 2016; 12;5(12).

5. Di Modugno F, Spada S, Palermo B, Visca P, Iapicca P, Di Carlo A, Antoniani B, Sperduti I, Di Benedetto A, Terrenato I, Mottolese M, Gandolfi F, Facciolo F, Chen EI, Schwartz MA, Santoni A, Bissell MJ, Nisticò P. hMENA isoforms impact NSCLC patient outcome through fibronectin/β1 integrin axis. Oncogene. 2018 Jun 15.

6. Bria E, Di Modugno F, Sperduti I, Iapicca P, Visca P, Alessandrini G, Antoniani B, Pilotto S, Ludovini V, Vannucci J, Bellezza G, Sidoni A, Tortora G, Radisky DC, Crinò L, Cognetti F, Facciolo F, Mottolese M, Milella M, Nisticò P, Prognostic impact of alternative splicing-derived hMENA isoforms in resected, node-negative, non-small-cell lung cancer. Oncotarget. 2014; 30;5(22):11054-63.

**Ethics Approval**

This study was approved by the ‘Comitato Etico Centrale IRCCS Lazio’; approval number ifo_058.IFO_AOO.REGISTRO UFFICIALE.U.0012817 20-11-2017 and number CE/594/11 del 3/11/2011

#### P835 The role of sialylation in stromal cell mediated immunosuppression in the colorectal tumour microenvironment

##### Hannah Egan, BSC, Kevin Lynch, PhD, Niamh Leonard, MSc, Michael O, Laurence Egan, MD, Thomas Ritter, Oliver Treacy, PhD, Aideen Ryan, PhD

###### National University of Ireland (NUIG), Galway, North Campus, Ireland

####### **Correspondence:** Aideen Ryan (aideen.ryan@nuigalway.ie)

**Background**

Immunosuppressive tumour microenvironments (TME) reduce the effectiveness of immunotherapies for the treatment of cancer [1]. Hyper-sialylation of glycoproteins and glycolipids has been linked to increased immune evasion, drug resistance, tumour invasiveness and metastasis in cancer [2]. Increased levels of sialic acid on the surface of cancer cells has been associated with an increase in cancer cell survival via the modulation of immunosurveillance [3]. Non haematopoietic stromal cells (MSCs), the precursor to cancer associated fibroblasts (CAFs) play a vital role in tumour progression by enhancing a tumour promoting microenvironment [4]. This study investigates if MSC/CAF sialylation contributes to enhanced immunosuppression in the colorectal tumour microenvironment

**Methods**

Using a Balb/c immunogenic, syngeneic model of colorectal cancer, we characterised silayltransferase expression and the cell surface sialic acid profile of tumour associated MSCs exposed to tumour cell secretome, using RNA sequencing and lectin-based flow cytometry respectively. Immunosuppression was determined using an ex vivo CFSE based co-culture system. T-lymphocytes were co-cultured with conditioned MSCs and T-lymphocyte proliferation and activation was assessed by flow cytometry. To investigate the role of sialic acid in MSC-mediated immunosuppression, tumour conditioned MSCs (CAFs) were treated with a sialyltransferase inhibitor (SI). SI treated tumour conditioned MSCS were co-cultures with activated lymphocytes and phenotype, proliferation and activation were assessed by multicolour flow cytometry.

**Results**

Our results show overexpression of silayltransferase expression and α2,6-sialic acid cell surface expression on MSCs/CAFs exposed to tumour cell secretome for 72 hours. Tumour conditioned stromal cells overexpress Siglec ligands as determined by SIglec E Fc chimera binding.. We observed that inflammatory tumour conditioned MSCs significantly suppressed activation and proliferation of CD4+ and CD8+ T-cells. Siglec E expression was enhanced on co-cultured CD8+ T cells suggestive of sialic acid dependent inactivation. Inhibiting sialic acid on cell surface using SI inhibitor over a 72h period, confirmed that this effect was dependent on sialylation. Siglec E expression was inhibited, resulting in enhanced effector function following SI treatment. These effects were also observed in a model of multiple myeloma, indicating that MSC sialylation is not restricted to the colorectal tumour microenvironment.

**Conclusions**

These results indicate for the first time that non-haematopoietic stromal cells in the tumour-microenvironment express high levels of sialic acid which contribute to their ability to suppress activated T-effector cells. Understanding how glycosylation of stromal cells, and more specifically sialylation is regulated and functions to enhance immunosuppression in the TME could uncover novel immune checkpoints to reactivate anti-tumour immunity

**References**

1. Tang H, Qiao J, Fu YX. Immunotheray and the tumour microenvironment. Cancer Lett. 2016; 370(1): 85-90.

2. Bull C, den Brok MH, Adema GJ. Sweet escape: sialic acids in tumor immune evasion. Biochim Biophys Acta. 2014; 1846: 238-246.

3. Bull C, Boltje TJ, Balnege Nr, Weischer SM, Wassink M, van Gemst JJ, Bloemendal VR, Boon L, van der Vlag J, Heise T, den Brok MH, Adema GJ, Sialic Acid Blockade Suppresses Tumor Growth by Enhancing T-cell-Mediated Tumor Immunity. Cancer Res. 2018; 78: 3574-3588.

4. O'Malley G, Treacy O, Lynch K, Naicker S.D, Leonard NA, Lohan P, Dunne PD, Ritter T, Egan LJ, Ryan AE Stromal Cell PD-L1 Inhibits CD8(+) T-cell Antitumor Immune Responses and Promotes Colon Cancer. Cancer Immunol Res. 2018; 6: 1426-1441.

**Ethics Approval**

The study was approved by Animal Care research Ethics Committee, NUI Galway

#### P836 Immunopathological properties of the fibroblastic niche for cancer stem cells and the niche-targeted immunotherapy

##### Toshihiko Torigoe, MD, PhD , Yoshihiko Hirohashi, MD, PhD

###### Sapporo Medical University, Sapporo, Japan

####### **Correspondence:** Toshihiko Torigoe (torigoe@sapmed.ac.jp)

**Background**

Cancer stem-like cells (CSCs) are a small subpopulation of cancer cells that have unique characteristics such as tumor-initiating capacity and cellular stress tolerance. These cells have a huge impact on the field of cancer therapy since they are resistant to standard chemoradiotherapy and responsible for disease recurrence [1, 2].

**Methods**

We have studied the immunopathological properties of CSCs derived from colon, breast, and gynecological cancers to develop CSC-targeted immunotherapy. The following characteristics were revealed in the present study.

**Results**

#1 CSC-specific antigens: CSCs expressed several germ cell-specific genes, and some of them had crucial roles in the maintenance of tumor initiation capacity. Importantly, they were highly immunogenic since antigen-specific cytotoxic T-cells (CTLs) were efficiently induced from peripheral blood lymphocytes. The CTLs exerted specific cytotoxicity against CSCs. Adoptive transfer of the CTLs efficiently protected tumor growth in mouse models.

#2 Fibroblastic niche for CSCs: CSCs recruited fibroblasts, which enhanced the tumor initiation capacity and immune resistance of CSCs. Gene expression analysis revealed that fibroblast growth factor 4 (FGF4) and FGFR2, a receptor for FGF4 were involved in the fibroblastic niche of CSCs. siRNA-mediated knockdown of FGFR2 abrogated the pro-tumorigenic character of the fibroblasts.

#3 Innate immune resistance of CSCs: Some CSCs constitutively expressed PD-L1 on the cell surface through the activation of PI3K/AKT signaling.

**Conclusions**

Based on these unique properties of CSCs, we propose a niche-targeted combination immunotherapy for cancer.

**References**

1. Tabuchi Y, Hirohashi Y, Hashimoto S, Mariya T, Asano T, Ikeo K, Kuroda T, Mizuuchi M, Murai A, Uno S, Kawai N, Kubo T, Nakatsugawa M, Kanaseki T, Tsukahara T, Saito T, Torigoe T. Clonal analysis revealed functional heterogeneity in cancer stem-like cell phenotypes in uterine endometrioid adenocarcinoma. Exp Mol Pathol. 2019; 106:78-88.

2. Yasuda K, Torigoe T, Mariya T, Asano T, Kuroda T, Matsuzaki J, Ikeda K, Yamauchi M, Emori M, Asanuma H, Hasegawa T, Saito T, Hirohashi Y, Sato N. Fibroblasts induce expression of FGF4 in ovarian cancer stem-like cells/cancer-initiating cells and upregulate their tumor initiation capacity. Lab Invest. 2014; 94:1355-69.

**Ethics Approval**

The experiments were conducted with the approval of the Sapporo Medical University Ethics Review Board, approval number 282-134 and 282-1055.

#### P837 Roles of nitric oxide synthase and cyclooxygenase inhibition in potentiating immune response in murine models of 4T1 mammary tumor

##### Veena Somasundaram, PhD, Anne Gilmore, Debashree Basudhar, Erika Palmieri, David Scheiblin, William Heinz, Robert Cheng, Lisa Ridnour, PhD, Stephen Lockett, Daniel McVicar, PhD, David Wink

###### National Cancer Institute, NIH, Frederick, MD, United States

####### **Correspondence:** David Wink (wink@mail.nih.gov)

**Background**

Anticancer versus protumor behavior of tumor microenvironment (TME) largely depends on tumor cell-macrophage interactions which are regulated by nitric oxide (NO) in mouse models as well as patients. NO induces diverse effects in cancer cells and TME based on its localized flux. High expression of inducible nitric oxide synthase (NOS2) and co-expression with high cyclooxygenase 2 (COX2) is associated with poor prognosis in ER- breast cancer patients and pharmacological inhibition of these two proteins reduces primary tumor growth in nude mouse tumor models [1, 2]. However, the role of the immune system in this effect is largely unknown. As tumors are nutrient deprived, we hypothesized that NO flux dependent metabolic changes in TME could determine the outcome of the tumor-immune system crosstalk.

**Methods**

4T1 mammary tumor model in wild type (wt) and Nos2 knockout (Nos2-/-) BALB/c mice was utilized to study the effects of host Nos2 and Cox2 on tumorigenesis and pulmonary metastasis. Bone marrow derived macrophages (BMDMs) from wt and Nos2-/- mice were stimulated with cytokines to produce low, medium and high NO flux and effects on metabolism were studied using seahorse bioanalyzer and microscopy. Effects of varying NO flux on local oxygen tension (pO2) was analyzed using a novel, in vitro chamber system that forms cell-generated hypoxic and metabolic gradients by restricting the diffusive exchange of oxygen and metabolites to a monolayer of cells– analogous to diffusion between capillaries and nearby tissue.

**Results**

Absence of Nos2 in the immune system reduces pulmonary metastasis while pharmacological inhibition of Cox2 delays tumor growth preferentially in Nos2-/- mice. High NO flux delayed onset of hypoxia and improved local pO2. Nos2high cells harbored depolarized mitochondria and were Cox2low. Also, Nos2 and Cox2 expression in 4T1 mouse tumors was spatially distinct, and paracrine Nos2/Cox2 regulation by prostaglandin E2 (Cox2 product) and NO, respectively modulated inflammatory status and treatment response.

**Conclusions**

High levels of NO such as those from inflammatory murine macrophages regulate several effects in neighboring cells due to a cumulative effect of the metabolic changes occurring in Nos2high single cells through localized extracellular NO flux. Maintenance of higher levels of NO requires Nos2 and Cox2, which are spatially separated in cultured cells and tumor tissues. Importantly, while Cox inhibition reduces primary tumor volume, Nos2 inhibition reduces lung metastasis suggesting that the spatial localization of Nos2 and Cox2 may provide diagnostic and therapeutic options for targeting these aggressive cell niches.

**Acknowledgements**

This work was supported by the NIH Intramural Research Programs Cancer and Inflammation Program, Optical Microscopy and Image Analysis Laboratory and the NIH Post-Baccalaureate Intramural Research Training Award Program at the National Cancer Institute. This project was funded in whole or in part with Federal funds from the National Cancer Institute, NIH, under Contract HHSN261200800001E.

**References**

1. Basudhar D, et al. Coexpression of NOS2 and COX2 accelerates tumor growth and reduces survival in estrogen receptor-negative breast cancer. Proc Natl Acad Sci U S A. 201;, 114(49):13030-13035.

2. Glynn SA, et al. Increased NOS2 predicts poor survival in estrogen receptor-negative breast cancer patients. J Clin Invest. 2010; 120(11):3843-3854.

#### P838 Disrupting hypoxic tumor niches to sensitize pancreatic cancer to immunotherapy

##### Arthur Liu, BS, Michael Curran, PhD

###### The University of Texas MD Anderson Cancer Center, Houston, TX, United States

####### **Correspondence:** Michael Curran (MCurran@mdanderson.org)

**Background**

Despite the clinical success of immune checkpoint blockade therapy in providing durable responses in advanced cancers such as melanoma, its use as a monotherapy in patients with pancreatic cancer has shown little efficacy [1]. We have previously shown that hypoxia in the tumor microenvironment acts as a major barrier to host immunity, and that the hypoxia-activated prodrug TH-302 (Evofosfamide) potentiates CTLA-4/PD-1 blockade therapy [2]. Mechanistically, TH-302 decreased tumor hypoxia and increased accumulation of CD31+ endothelial cells, suggestive of improved oxygen supply and normalization of tumor vasculature. While TH-302 initiates a cellular remodeling process that diminishes tumor hypoxia, it remains unclear to what extent this involves alterations in tumor angiogenesis directly versus indirectly, and thus whether hypoxia reduction could be improved through concurrent anti-angiogenic therapy.

**Methods**

We utilized TH-302 and a vascular endothelial growth factor receptor-2 (VEGFR-2) blocking antibody (DC101) to assess the relationship between blockade of angiogenesis and the hypoxia reduction activity of TH-302. Using an orthotopic, transplantable model of pancreatic cancer, we investigated the use of DC101 and TH-302 to impact tumor hypoxia, vessel normalization, and infiltration of CD8+ T cells.

**Results**

We found that orthotopic transplanted pancreatic tumors contain hypoxic tumor niches that lack T cell infiltration. High-dose DC101 (40mg/kg) in combination with TH-302 (50mg/kg) significantly increased the frequency of tumor infiltrating CD8+ T cells relative to mice treated with TH-302 alone. Preliminary data suggests that compared to low-dose DC101 (10mg/kg), the combination of 40mg/kg DC101 and TH-302 increases the percentage of hypoxia-exposed CD8+ T cells and is associated with expansion of intercellular adhesion molecule 1 (ICAM-1)+ expressing CD31+ cells. Additionally, combination DC101 and TH-302 promote major histocompatibility complex (MHC)-II presentation and simultaneously reduces programmed death-ligand 2 (PD-L2) expressing endothelium.

**Conclusions**

We found that the combination of DC101 and TH-302 promotes infiltration of CD8+ T cells. This is in part associated with re-activation of tumor endothelium to a pro-inflammatory state favoring T cell adhesion and extravasation. These results indicate that combining TH-302 mediated tissue remodeling with VEGFR-2 blockade can enhance both reduction of hypoxia and induction of tumor immunity in a refractory model of pancreatic cancer.

**References**

1. Royal RE, Levy C, Turner K, et al. Phase 2 Trial of Single Agent Ipilimumab (Anti-CTLA-4) for Locally Advanced or Metastatic Pancreatic Adenocarcinoma. J Immunother. 2010;33:828-33.

2. Jayaprakash P, Ai M, Liu A, Budhani P, Bartkowiak T, Sheng J, et al. Targeted hypoxia reduction restores T cell infiltration and sensitizes prostate cancer to immunotherapy. J Clin Invest. 2018;128(11):5137-49.

#### P839 DNA demethylating agent sensitizes lung cancer cells to immune attack of γδ T cells

##### Rueyhung Weng, PhD, Chien-Ting Lin, Hsuan-Hsuan Lu, Chong-Jen Yu, Hsing-Chen Tsai

###### National Taiwan University Hospital, Taipei, Taiwan, Province of China

####### **Correspondence:** Hsing-Chen Tsai (htsai@ntu.edu.tw)

**Background**

Decitabine, a DNA demethylating agent, is known to induce a viral mimicry interferon response and facilitate T cell killing in a major histocompatibility complexes (MHC)-dependent manner. Abundant evidence has indicated that tumor may evade immune attack through downregulation of surface proteins including MHC molecules. It remains unclear whether and how decitabine may alter surface immune-related molecules and augment other types of immunotherapy.

**Methods**

We treated human lung cancer cells with decitabine at clinically-relevant low doses, and isolated cell surface proteins using biotin-affinity purification. To obtain a comprehensive picture of surface proteins altered by decitabine, we employed an unbiased high-throughput approach using Stable Isotope Labeling by Amino acids in Cell culture (SILAC)-based quantitative proteomics. We performed in vitro functional assays and in vivo animal studies to examine γδ T cell-mediated anti-tumor immunity enhanced by decitabine. We conducted CRIPSR-knockout and overexpression experiments to uncover key decitabine-mediated immune molecules on lung cancer cells for γδ T cell cytolysis.

**Results**

A total of 351 surface proteins in A549 cells were upregulated in decitabine-treated cells. Gene ontology analysis of these proteins revealed γδ T lymphocyte activation as the top enriched immune pathway, which was known to exert anti-tumor immunity in an MHC-unrestricted manner. We further demonstrated that pretreatment of various lung cancer cell lines with decitabine markedly enhanced γδ T cell killing in coculture assays. Of all decitabine-induced proteins, we found that intercellular adhesion molecule 1 (ICAM1) was a key molecule involved in the cancer-γδ T cell interaction. Through over-expression and knockdown experiments, we further demonstrated that ICAM1 protein levels on the cancer cell surface correlated with the susceptibility of decitabine-treated cancer cells to γδ T cell killing. Combination treatment of decitabine followed by adoptive transfer of γδ T cells in lung cancer xenograft mouse models showed much better therapeutic efficacy than either treatment alone. Moreover, higher ICAM1 levels were observed in tumor tissues responsive to the therapy.

**Conclusions**

Our data demonstrated sensitization effects of decitabine to γδ T cell therapy in lung cancer cells through ICAM1 upregulation. This provides novel molecular basis for coupling DNA demethylation agents with MHC-unrestricted γδ T immunotherapy in the treatment of lung cancer.

**Ethics Approval**

The study was approved by National Taiwan University College of Medicine and College of Public Health Institutional Animal Care and Use Committee (IACUC), approval number 20180077.

#### P840 Characterization of syngeneic tumor models and their microenvironment for immunotherapeutic approaches

##### Marco Carretta, PhD^1^, Marie Louise Thorseth^1^, Dennis Agardy^1^, Silvia Redondo Garcia^2^, Anne Mette Hvid Larsen, MSc^1^, Dorota Kuczek, MSc^1^, Daniel Madsen^1^

###### ^1^CCIT - Herlev Hospital, Herlev, Denmark; ^2^Universidad de Granada, Granada, Spain

####### **Correspondence:** Daniel Madsen (daniel.hargboel.madsen@regionh.dk)

**Background**

The recent success of immunotherapeutic approaches has demonstrated that the immune system plays a critical role in fighting cancer. Syngeneic mouse tumor models retain intact immune systems and therefore are particularly relevant for studying immunologically based targeted therapies, either alone or in combination with other drugs. Although developed decades ago, the immunologic and molecular characteristics of syngeneic mouse tumor models and their microenvironment have been poorly investigated. In this work, we set out to characterize a panel of commonly used murine syngeneic tumor models covering all the major cancers in order to investigate the correlations between different parameters and improve preclinical model selection for target validation and drug development.

**Methods**

We are currently characterizing 5 syngeneic tumor models in C57BL/6 mice (B16, LL2, PAN02, EO771, MC38) and their microenvironment by flow cytometry and RNA sequencing. For flow cytometry analysis we use antibody panels to identify various myeloid and lymphoid cells and a panel to characterize the composition of other tumor associated cells such as cancer associated fibroblasts (CAFs) and endothelial cells. Furthermore, we monitor tumor sizes and measure the relative physical stiffness of tumor tissues by shear rheology. Lastly, we sort CAFs and perform RNA sequencing.

**Results**

The flow cytometry analysis revealed striking differences across the different models, especially in relation to the ratio of CD45+ cells within the tumor. Models such as B16F10 and EO771 displayed particularly low immune infiltration levels while, in contrast, we have found that the PANO2 model has a significantly higher level of CD45+ cells compared to all the other models. The MC38 and LL2 models displayed intermediate levels of immune infiltration but are associated with increased levels of myeloid immunosuppressor cells such as mMDSC. Furthermore, we have focused our attention on the extracellular matrix components and found positive correlation between the percentage of CAFs and the relative physical stiffness of tumor tissues for models such as EO771 and PANO2. Ongoing RNAseq analysis of the tumors and CAFs will hopefully provide significant insights in the biology of these tumors and their microenvironment.

**Conclusions**

Thus far, we can conclude that the characterization of syngeneic tumor models and their microenvironment reflects the immunologic diversity observed in the clinic and provides a resource to select relevant models to study and test the efficacy of immunotherapies in vivo.

#### P841 NCG/CRL as a novel (Charles River) humanized mouse model for pre-clinical oncology studies: immunophenotypic characterization and performance monitoring

##### Christoph Eberle, PhD, Jenny Rowe, PhD, Robert Mihalek, PhD, Stephen Festin

###### Charles River Laboratories, Wilmington, MA, United States

####### **Correspondence:** Stephen Festin (steve.festin@crl.com)

**Background**

The NCG (NOD-Prkdcem26Cd52Il2rgem26Cd22/NjuCrl) model was created by simultaneous CRISPR/Cas9 editing of the Prkdc and Il2rg loci in the NOD/Nju mouse. NCG mice are severely immunodeficient enabling xenotransplantation of human immune cells for studying human immune responses to new therapies. In this study we characterized the hematopoietic output of humanized mice.

**Methods**

Charles River NCG mice (n=14) were inoculated with cord blood derived human CD34+ stem cells. Following engraftment body weights, general health status and survival was monitored. Additionally, peripheral blood (PB) and selected tissues were analyzed for the presence and composition of human immune cells by multicolor flow cytometry.

**Results**

NCG mice remain humanized up to 34 weeks post-engraftment. Over time the mean hCD45+ levels in PB can reach 83.67%, whereas on average between 1.85-14.22% of single live cells were residual mCD45+ leukocytes. Circulating human lymphocyte subsets (total T, CD4, CD8, DP, B, NK, iNKT, CD30) could be quantified in PB at Days 92, 124, 180 and 236 post-engraftment. Study survival rate was 100% up to Day 226 with 63% surviving up to Day 250. Current studies are ongoing to monitor CD34+ donor variability. Of note, at Days 124 and 180 the average proportion of mature B-cells was lower compared to other subsets and time points. Individual mice (n=4) suspected of graft-versus-host disease (GvHD) showed increased CD30 expression on T-cells and higher CD4:CD8 ratios, when compared to non-GVHD mice (n=7), coupled with changes in clinical signs. Bone marrow, PB, spleen, liver and lung tissues were collected at Days 166 (n=3) and 223 (n=3) post-engraftment. Phenotyping by flow cytometry defined human immune cells in each tissue compartment that retained the ability to differentiate into common lymphoid (total and proliferating T, CD4, CD8, Tregs) and myeloid populations (CD11b, DC, M1, M2 macrophages). Distribution of both lineages was preserved without subset loss up to 31 weeks post-engraftment. Likewise, splenic T-lymphocytes secreted cytokines (IFN-γ, IL-2, TNF-α) after PMA/Ionomycin stimulation.

**Conclusions**

The Charles River NCG mouse has been developed as a new humanized model with stable graft function. Phenotypic data acquired by flow cytometry demonstrate reconstitution of human immune cells. These repopulate and maintain lymphoid and myeloid differentiation, where T-lymphocytes can also generate polyfunctional responses. Both lineages are detectable in the periphery and infiltrate mouse tissues. This new model needs to be further evaluated for testing human immunotherapies in pilot tumor cell line studies.

#### P842 The pattern of expression of hMENA isoforms impacts tumor immune microenvironment of early node-negative non-small cell lung cancer

##### Paola Nistico', MD , Francesca Di Modugno, PhD, Sheila Spada, PhD, Anna Di Carlo, Isabella Sperduti, Enzo Gallo, Mariangela Panetta, Belinda Palermo, Francesca De Nicola, Lorenzo D'Ambrosio, Barbara Antoniani, Paola Trono, Francesco Facciolo, Paolo Visca

###### Regina Elena National Cancer Institute, Rome, Italy

####### **Correspondence:** Paola Nistico' (paola.nistico@ifo.gov.it)

**Background**

Actin cytoskeleton dynamics act as platforms for gene regulation and key signaling transduction pathways involved in the cross-talk among tumor cells and cellular and non-cellular components of the tumor microenvironment (TME). The actin regulatory protein hMENA undergoes tissue specific splicing, generating two alternatively expressed isoforms hMENA11a and hMENAΔv6 that respectively inhibit or increase cell invasiveness [1], TGFβ [2] and β1 integrin signaling and the secretion of several key extracellular matrix (ECM) proteins [3] and is crucial in TGFβ mediated-EMT [2]. Early node-negative NSCLC patients show a prolonged disease-free survival when expressing high tumor hMENA11a/low stromal FN1[3]. Recently, tertiary Lymphoid Structures (TLS), sites of transient lymphoid neo-genesis and determinants of antitumor immunity, have been associated with a favorable clinical outcome in NSCLC patients [4].

**Methods**

The pattern of hMENA isoform expression, TLS presence and localization and stromal fibronectin were evaluated in 94 primary tumors of node negative NSCLC patients by immunohistochemistry. We evaluated the role of hMENA isoform expression on lymphotoxin beta receptor (LTBR), by gain and loss of function, RNA-SEQ and QRT-PCR.

**Results**

We found that TLS localized within the tumor area (TLS-AT) are predictive of a longer disease free survival in N0 NSCLC patients, whereas the presence of peritumoral TLS (TLS-PT) does not associate with the patient survival, but with higher tumor grade. TLS-AT presence is significantly correlated with hMENA11a expression in tumor cells, whereas the presence of TLS-PT correlates with the absence of hMENA11a. We evidenced that a low level of stromal FN along with the expression of hMENA11a, associated with TLS-AT.

Searching for the mechanisms involved, we found that the hMENA11a knock out in NSCLC cells modulates the expression of several genes: down-regulates the TLS regulator LTβR and up-regulates FN1. Finally, we found that the depletion hMENA11a in the H1650 NSCLC cells induces the reduction of the chemokine CXC-chemokine ligand 13 (CXCL13) a critical cytokine induced after LTβ-LTβR signalling [5] [6].

**Conclusions**

Our findings indicate that the alternative splicing of hMENA is crucial in the reciprocal signaling between tumor cells and their immune microenvironment, by participating in tertiary lymphoid structure neo-genesis and spatial distribution [7, 8].

**Acknowledgements**

This work is supported by AIRC

**References**

1. Di Modugno F, Iapicca P, Boudreau A, Mottolese M, Terrenato I, Perracchio L, Carstens RP, Santoni A, Bissell MJ, Nisticò P. Splicing program of human MENA produces a previously undescribed isoform associated with invasive, mesenchymal-like breast tumors. Proc Natl Acad Sci U S A. 2012; 109(47):19280-5.

2. Melchionna R, Iapicca P, Di Modugno F, Trono P, Sperduti I, Fassan M, Cataldo I, Rusev BC, Lawlor RT, Diodoro MG, Milella M, Grazi GL, Bissell MJ, Scarpa A, Nisticò P. The pattern of hMENA isoforms is regulated by TGF-β1 in pancreatic cancer and may predict patient outcome. Oncoimmunology. 2016; 5(12):e1221556.

3. Di Modugno F, Spada S, Palermo B, Visca P, Iapicca P, Di Carlo A, Antoniani B, Sperduti I, Di Benedetto A, Terrenato I, Mottolese M, Gandolfi F, Facciolo F, Chen EI, Schwartz MA, Santoni A, Bissell MJ, Nisticò P. hMENA isoforms impact NSCLC patient outcome through fibronectin/β1 integrin axis. Oncogene 2018; 37: 5605-5617.

4. Dieu-Nosjean MC, Giraldo NA, Kaplon H, Germain C, Fridman WH, Sautès-Fridman C. Tertiary lymphoid structures, drivers of the anti-tumor responses in human cancers. Immunol Rev. 2016;271(1):260-75.

7. Dejardin E, Droin NM, Delhase M, Haas E, Cao Y, Makris C, Li ZW, Karin M, Ware CF, Green DR. The lymphotoxin-beta receptor induces different patterns of gene expression via two NF-kappaB pathways. Immunity 2002; 17: 525-535.

8. Tang H, Zhu M, Qiao J, Fu YX. Lymphotoxin signalling in tertiary lymphoid structures and immunotherapy. Cell Mol Immunol 2017; 14: 809-818.

**Ethics Approval**

This study was approved by the ‘Comitato Etico Centrale IRCCS Lazio’; approval number Ifo_058.IFO_AOO.REGISTRO UFFICIALE.U.0012817 20-11-2017

#### P843 Oral cancers expressing Glycoprotein-A repetitions predominant (GARP) show poor prognosis

##### Rieneke van de Ven, PhD , Jan Kloosterman, Bsc, Irene Nauta, MSc, Sonja Ganzevles, BSc, Elisabeth Bloemena, Prof Dr, C. René Leemans, MD, PhD, Ruud Brakenhoff, MD, PhD

###### Amsterdam UMC, VU University Amsterdam, Cancer Center Amsterdam, Amsterdam, Netherlands

####### **Correspondence:** Rieneke van de Ven (R.vandeven@vumc.nl)

**Background**

While immune checkpoint inhibitors targeting the programmed-death receptor-1 in recurrent and/or metastatic head and neck squamous cell carcinoma (HNSCC) (all regions) showed an encouraging 11% increase in 2-year overall survival (OS) (from 6-17%) [1], those data at the same time stress the need for the identification of additional, targetable, suppressive pathways in this disease. Glycoprotein-A repetitions predominant (GARP), a docking receptor for the latent transforming growth factor-beta (TGF-β) complex, facilitates TGF-β activation. Since TGF-β is a known immune-suppressive cytokine, we assessed GARP expression on oral squamous cell carcinoma (OSCC) specimen and analyzed whether its expression relates to clinical outcome.

**Methods**

Formalin-fixed paraffin embedded OSCC surgical resection specimen collected between 2005-2014 at our institute (n=56) were stained with anti-human GARP (clone: Plato-1) or an IgG2b isotype control antibody. DAB was used for visualization. Sections were scanned on a Vectra Polaris scanner, and images were analyzed using Phenochart. For some patient samples (n=38) CD45 immune infiltrate data within the tumor region were available. All patient materials were handled and used following the medical ethical guidelines as described in the Code of Conduct for Proper Secondary Use of Human Tissue of the Dutch Federation of Biomedical Scientific Societies or written informed consent was obtained in accordance with the Declaration of Helsinki. Graphpad Prism 8 software was used for statistical analyses.

**Results**

GARP expression was detected in 57% of OSCC (32/56). The majority of the GARP+ tumors (n=24) displayed a marginal expression pattern for GARP, with GARP being expressed on tumor cells located at the tumor invasive border. A diffuse expression pattern, with all tumor cells expressing GARP, was observed in 19% (n=6) of GARP-positive tumors. Two tumors had both marginal and diffuse expression. CD45+ immune infiltration within the total tumor area (tumor core plus invasive margin) was similar for GARP-negative and GARP-positive tumors. Survival analysis showed a significantly reduced 5-year OS for GARP-positive versus GARP-negative tumors with a hazard ratio of 2,90 (95% CI: 1,30 – 6,46; p<0.02 by Log-rank Mantel-Cox test). Though low in number in the current cohort, patients with tumors with only diffuse expression had an even poorer outcome compared to GARP-negative and marginal-GARP expressing tumors.

**Conclusions**

We found clear expression of GARP on OSCC tumor cells in over fifty percent of the patient samples. Expression of GARP on OSCC was linked with a poor prognosis. Ongoing research is focused on determining the immune suppressive effects of tumor-derived GARP expression.

**References**

1. Ferris RL et al., Nivolumab vs investigator’s choice in recurrent or metastatic squamous cell carcinoma of the head and neck: 2-year long-term survival update of CheckMate 141 with analyses by tumor PD-L1 expression. Oral Oncol. 2018; 81: 45-51.

**Ethics Approval**

All patient materials were handled and used following the medical ethical guidelines as described in the Code of Conduct for Proper Secondary Use of Human Tissue of the Dutch Federation of Biomedical Scientific Societies or written informed consent was obtained in accordance with the Declaration of Helsinki.

#### P844 Investigating the immunologic landscape and tumor-specific target identification in human clear cell renal carcinoma

##### Nivedita Chowdhury, MS^2^, Aleksandar Obradovic^2^, David Aggen, MD, PhD^2^, Michael Johnson^1^, Charles Drake, MD, PhD^2^

###### ^1^Johns Hopkins Medical Institution, New York, NY, United States; ^2^Columbia University Medical Center, New York, NY, United States

####### **Correspondence:** Charles Drake (cgd2139@cumc.columbia.edu)

**Background**

Evaluation of the mutational landscape of cancers has revealed significant correlation between mutation burden and response to immunotherapy in cancers such as melanoma and non-small cell lung carcinoma[1,2]. Clear cell renal carcinoma (RCC) is considered an exception as it has a low SNV (single nucleotide variant) burden and yet, a similar response rate. Factors within the tumor microenvironment (TME) play a critical role in regulating response to targeted immunotherapy. While it is well-accepted that ccRCC is characterized by T-cell infiltrates (TILs), differential activation states and effect of immunomodulatory cells remain unexplored. In this multi-institutional study, we applied next-generation sequencing technologies to ccRCC patient specimens to determine the immune cells within the TME as well as explore alternative sources of tumor-specific mutations.

**Methods**

Whole exome sequencing and RNA sequencing was performed on matched normal and tumor tissue from ten ccRCC patients (nuclear grades I-IV) undergoing surgical resection. Peripheral blood and tumor tissue were processed and cryopreserved for later use. We also sequenced approximately 20,000 immune cells at the single-cell level from tumor and matched adjacent normal tissue for two of these patients, using the 10X Genomics platform. SNVs and insertion/deletions (indel) were annotated and tumor/normal analysis was performed using DRAGEN’s somatic variant caller. Multi-dimensional reduction of single-cell data along with multiple iterations of PAM clustering with feature selection was implemented using iterClust algorithm.

**Results**

The visualization of immune-repertoire of RCC revealed a higher fraction of lymphoid infiltrate (~70-75%) than myeloid in a relatively stable ratio across both patients, with tumor containing a slightly higher fraction of lymphoid cells. Cross-referencing with known immune markers elucidated clusters of functionally exhausted cells, activated T cells as well as a distinct population of monocytes expressing high levels of complement factors C1QA, B, C and ApoE, exclusively in the tumor tissue.

The Indel analysis on the ten patients detected higher number of total insertions/deletions in tumor leading to the formation of ~3000 frameshift mutations and 186 gene fusion events. Tumor/normal analysis also identified expression of several endogenous retroviral (ERV) env proteins including ERVMER34-1, ERVV-1 and ERV3-1. Further analysis of transcriptionally active ERVs and their functional role is currently ongoing.

**Conclusions**

This study demonstrates the abundance of immune cell subtypes in the ccRCC TME, identifying a tumor-specific myeloid population. Additionally, detection of tumor-specific somatic variants beyond point mutations provide alternative sources of potential neoantigens in low mutation burden tumors such as renal cancer.

**References**

1. Rizvi NA, Hellmann MD, Snyder A, Kvistborg P, Makarov V, Havel JJ, Lee W, Yuan J, Wong P, Ho TS, Miller ML, Rekhtman N, Moreira AL, Ibrahim F, Bruggeman C, Gasmi B, Zappasodi R, Maeda Y, Sander C, Garon EB, Merghoub T, Wolchok JD, Schumacher TN, Chan TA. Mutational landscape determines sensitivity to PD-1 blockade in non-small cell lung cancer. Science. 2015; 348(6230): 124-8.

2. Johnson DB, Frampton GM, Rioth MJ, Yusko E, Xu Y, Guo X, Ennis RC, Fabrizio D, Chalmers ZR, Greenbowe J, Ali SM, Balasubramanian S, Sun JX, He Y, Frederick DT, Puzanov I, Balko JM, Cates JM, Ross JS, Sanders C, Robins H, Shyr Y, Miller VA, Stephens PJ, Sullivan RJ, Sosman JA, Lovly CM. Targeted Next Generation Sequencing Identifies Markers of Response to PD-1 Blockade. Cancer Immunol Res. 2016 Nov; 4(11):959-967.

**Ethics Approval**

This study was approved by the Institutional Review Boards of the Johns Hopkins Medical Institutions (Protocol#IRB00033839) and of Columbia University Medical Center (Protocol#AAAO5706).

#### P845 Targeting of Lactate Dehydrogenase C and its effects on the tumor immune microenvironment: two birds with one stone

##### Adviti Naik, PhD, Julie Decock, PhD, Aljazi Al-Khalifa

###### Qatar Biomedical Research Institute, Doha, Qatar

####### **Correspondence:** Julie Decock (jdecock@hbku.edu.qa)

**Background**

Cancer immunotherapy, in particular immune checkpoint blockade, has shown great promise in the treatment of advanced cancer. However, response rates remain low and only a subset of patients experience clinical benefit. One of the major challenges remains the persistence of a specific anti-tumor immune response within an immunosuppressive tumor microenvironment. Lactate dehydrogenase C (LDHC) is a promising novel target as it features a highly tumor-specific expression with an important role in metabolic reprogramming. Therapeutic targeting of LDHC could inhibit tumor growth by dysregulation of aerobic glycolysis, while releasing the anti-tumor immune response from the acidic immunosuppressive microenvironment with little off-target effects. In this *in vitro study*, we investigated the potential benefit of targeting LDHC in relation to its function in triple negative breast cancer (TNBC) cells and modulation of the tumor immune microenvironment.

**Methods**

Transcriptomic data from TCGA and METABRIC were mined for *LDHC* gene expression in breast cancer. A loss-of-function TNBC cell line model was established to study the effect of LDHC targeting on clonogenic ability, genomic stability and cellular senescence. In addition, immune modulation by LDHC was assessed using the ProteomeProfiler cytokine antibody array and through evaluation of PD-L1 tumor cell expression.

**Results**

Interrogation of TCGA and METABRIC RNA-seq data revealed that *LDHC* was markedly upregulated in TNBC compared to non-TNBC (Figure 1A), with a significant poorer overall and disease-specific survival in TNBC patients with higher LDHC expression (Figure 1B). Silencing of LDHC increased polyploidy (Figure 2A-B) as a likely result of microtubule dysfunction and mitotic slippage (Figure 2C) followed by increased cellular senescence (Figure 2C) and reduced clonogenicity (Figure 2D). Together these findings indicate that targeting LDHC could significantly reduce tumor burden. Furthermore, LDHC silencing induced substantial dysregulation of immunosuppressive cytokines illustrating the potential immunomodulatory effects of LDHC targeting (Figure 3A-B). For instance, LDHC silencing reduced the expression of factors involved in M2-macrophage polarization (CD14, Serpin E1, CHI3L1, IGFBP2, IL-10), inhibition of tumor infiltration of T cells and macrophages (MIF, CHI3L1, IL-10) or myeloid-derived suppressor cell infiltration (MIF, CD14). Moreover, LDHC silencing increased the ratio of non-glycosylated/glycosylated PD-L1 with primarily nuclear localization, thus negatively affecting the stability and immunosuppressive function of PD-L1 (Figure 3C-D).

**Conclusions**

Our findings demonstrate a positive correlation between LDHC expression and worse survival in specifically triple negative breast cancer. We provide in vitro evidence of the therapeutic potential of targeting LDHC to mitigate cancer cell growth and survival in addition to improving immunosurveillance.

**Acknowledgements**

This work was supported by a grant from the Qatar Biomedical Research Institute (grant number VR94), awarded to Dr Julie Decock.

**Fig. 1 (abstract P845).**
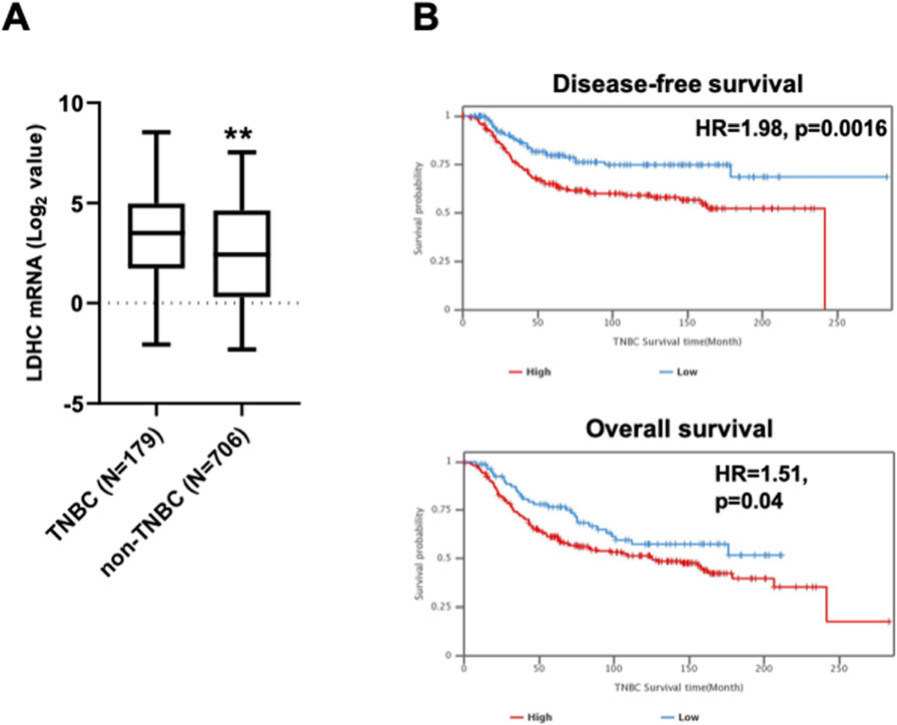
LDHC expression and triple negative breast cancer

**Fig. 2 (abstract P845).**
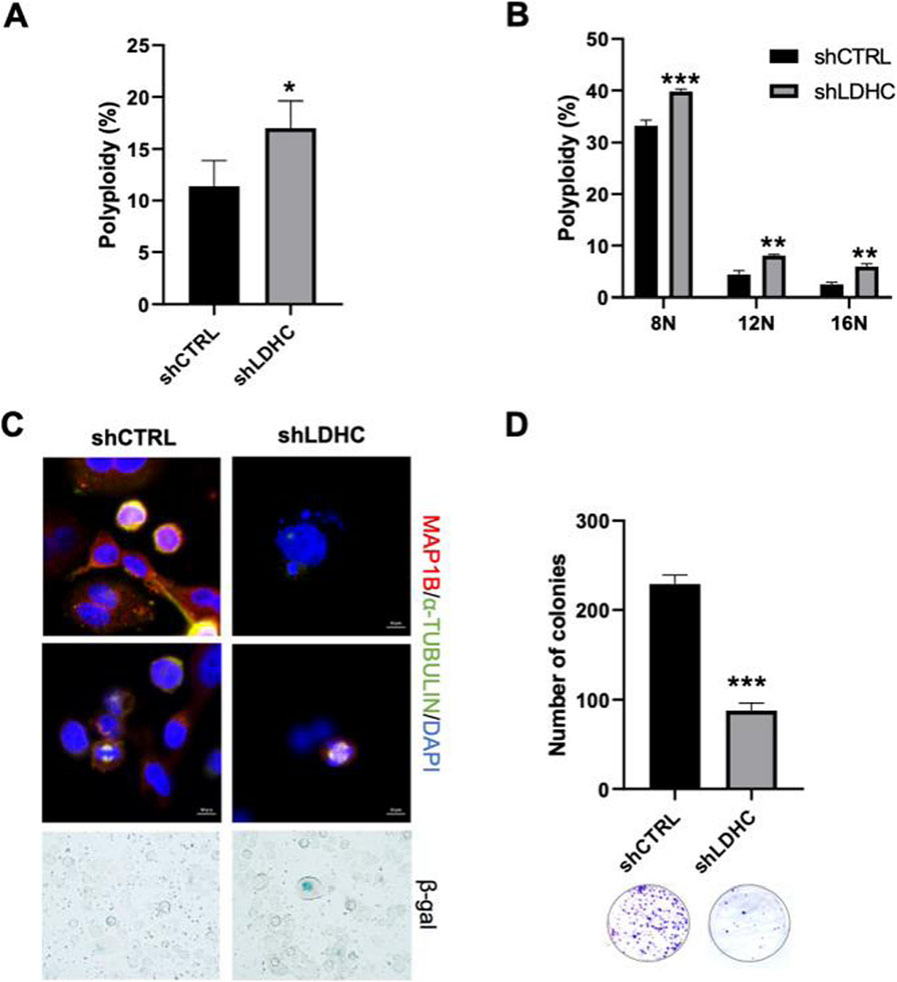
LDHC and cancer cell survival

**Fig. 3 (abstract P845).**
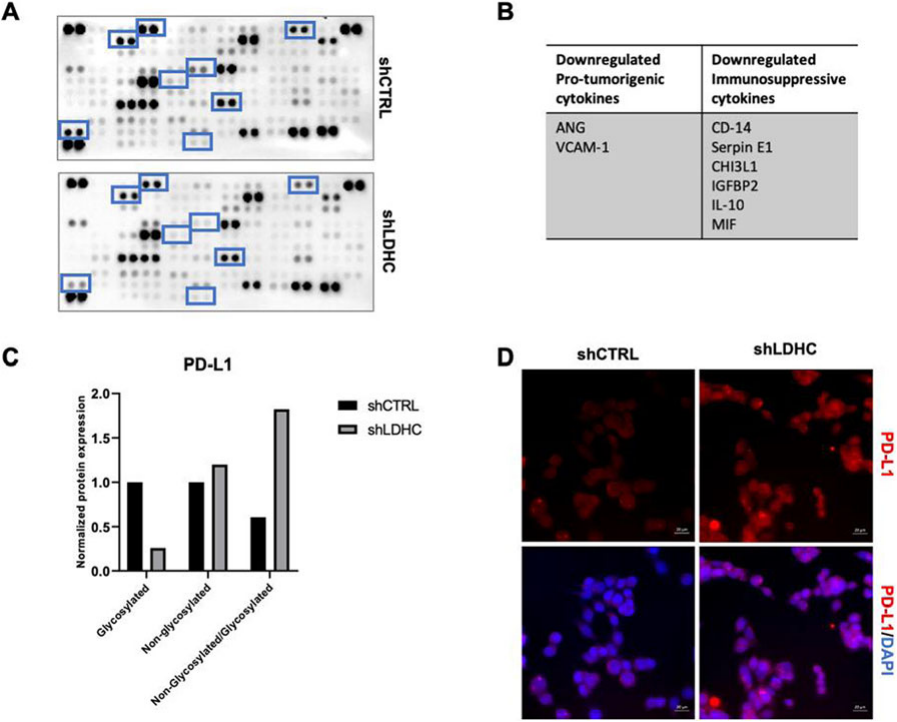
LDHC and tumor microenvironment modulation

#### P846 Tumor-infiltrating B cells control the progression of oropharyngeal squamous cell carcinoma via cell-to-cell interactions with CD8+ T cells

##### Kamila Hladíková^1^ , Vladimír Koucký^1^, Jan Bouček^2^, Jan Laco^3^, Marek Grega^2^, Aleš Ryška^3^, Radek Špíšek^1^, Anna Fialová^1^

###### ^1^Sotio a.s., Prague, Czech Republic; ^*2*^*University Hospital Motol, Prague, Czech Republic* ; ^*3*^*University Hospital Hradec Králové, Hradec Králové, Czech Republic*

####### **Correspondence:** Kamila Hladíková (hladikova@sotio.com)

**Background**

Standard treatment of oropharyngeal squamous cell carcinoma (OPSCC) is associated with high morbidity, whereas immunotherapeutic approaches using PD-1:PD-L1 checkpoint blockade only show moderate response rates in OPSCC patients. Therefore, the development of a complex therapeutic protocol combining checkpoint inhibition with other targets is needed for optimal responses. The importance of tumor-infiltrating B cells (TIL-Bs) in shaping antitumor immunity has been underestimated.

**Methods**

In this study, we assessed the density and distribution of TIL-Bs in FFPE samples from 2 independent cohorts of OPSCC patients. Than we assessed the phenotype of TIL-Bs by means of flow cytometry in fresh samples immediately after radical surgery. The acquired data were corelated with the clinical outcome of the patients.

**Results**

Here, we observed that CD20+ TIL-Bs and CD8+ T cells formed non-organized aggregates with clearly interacting cells and the densities of both intraepithelial CD20+ B cells and B/CD8+ T cell interactions have prognostic significance for the overall survival of OPSCC patients. Furthermore, a high density of TIL-Bs was associated with an activated B cell phenotype and a high density of direct B cell/CD8+ T cell interactions significantly correlated with the abundance of HPV16-specific CD8+ T cells.

**Conclusions**

Our results indicate that high abundance of TIL-Bs and high density of direct B cell/CD8+ T cell interactions can preselect patients with excellent prognosis who would profit from less invasive treatment. Furthermore, including B cells as an additional target in novel immunotherapeutic protocols may help to establish the desired sustained antitumor T cell responses in OPSCC patients.

**Acknowledgements**

This study was financially supported by the Grant Agency of Charles University, project number 344216.

**Ethics Approval**

All patient studies were conducted in accordance with Declaration of Helsinki and approved by an institutional review board, namely the Ethics Committee of the University Hospital Hradec Kralove and the Ethics Committee of the University Hospital Motol.

#### P847 Macrophages-induced H19 up-regulation activate the miR-193b/MAPK1 axis and promotes cell aggressiveness in hepatocellular carcinoma

##### Jinpu Yu , Yingnan Ye

###### Tianjin Tumor Hospital, Tianjin Shi, China

####### **Correspondence:** Jinpu Yu (jinpu_yu@hotmail.com)

**Background**

Dysregulation of long noncoding RNA (lncRNA) H19 has been implicated in hepatocellular carcinoma (HCC), but the concrete regulatory mechanism is lack of research. Our study aims to systematically study the role and regulation of H19 in HCC and find prognostic biomarkers and potential targets for HCC.

**Methods**

We mined gene expression profiles of 457 HCC samples from TCGA and TJMUCH cohorts (GSE116174 and GSE116182) and further validated in 64 FFPE HCC tissues. Genetically modified HCC cell lines HepG2 were applied to investigate the pathological functions of lncRNA H19 and its interaction with corresponding target miRNAs and mRNAs.

**Results**

LncRNA H19 overexpression in situ was significantly correlated with poor prognosis of HCC patients, which induced EMT, promoted stemness and accelerated invasion of HCC cells in vitro. Co-expression network analysis indicated lncRNA H19 negatively correlated with miR-193b and positively correlated with MAPK1 gene, which implicated that lncRNA H19 served as a sponge molecule to hijack miR-193b and protect MAPK1 from miR-193b-mediated gene knockdown. Forced overexpression of H19 attenuated miR-193b-mediated inhibition on multiple driver oncogenes (EGFR, KRAS, PTEN and IGF1R) and MAPK1 gene, thus triggered EMT and stem cell transformation in HCC. LncRNA H19 positively correlated with CD68+ TAMs in situ which induced H19 overexpression and activated H19-centered ceRNA network to promote HCC aggressiveness in vitro.

**Conclusions**

TAMs-induced lncRNA H19 promotes HCC aggressiveness via triggering and activating the miR-193b/MAPK1 axis, mediates the crosstalk between HCC and immunological microenvironment, and causes poor clinical outcomes. LncRNA H19 is a valuable predictive biomarker and potential therapeutic target in HCC.

**Acknowledgements**

This work was supported by National Natural Science Foundation of China (grant numbers: 81872143, 81702280, 81472473, 81272360) and Key Project of Tianjin Health and Family Planning Commission (16KG126).

**Ethics Approval**

This study was approved by the Ethics Committee of Tianjin Medical University (No. TMUhMEC 2012018). All experiments were performed in accordance with the principles of the Declaration of Helsinki and written consent was obtained from all patients.

#### P848 MiR-17 Cluster Promoted M2-polarized tumor associated macrophages related cell aggressiveness via inducing the imbalance of TGF-β1 /BMP7 pathways in hepatocellular carcinoma

##### Yingnan Ye, Junya Ning, Jinpu Yu

###### Tianjin Tumor Hospital, Tianjin, China

####### **Correspondence:** Jinpu Yu (jinpu_yu@hotmail.com)

**Background**

TGF-β1 and BMP7 are important members of TGF-β superfamily, which have similar downstream transduction pathways and interfere mutually. The antagonistic effects between both pathways has been widely studied in kidney disease and bone formation, and less studied in HCC. This study is intended to elucidate the crosstalk between TGF-β1/ BMP-7 pathways in the progression and invasion of HCC.

**Methods**

The immunohistochemistry staining method was used to exam the expressions of TGFBR2, TGFBR1, BMP7, BMPR2 and ACVR1 proteins in 64 HCC samples. Genetically modified HCC cell lines HepG2 and Hep3B were applied to investigate the related molecule mechanisms of antagonistic effects of TGF-β1 and BMP7 pathway in HCC.

**Results**

In 64 HCC samples, the expression of TGFBR2 was significantly negatively correlated with ACVR1 expression. TGFBR2-ACVR1+ HCC patients showed higher portal vein invasion and shorter survival time than non-TGFBR2-ACVR1+ HCC patients, which implied that down-regulated TGFBR2 and up-regulated ACVR1 dramatically promoted HCC invasion and metastasis in vivo. We separately knocked down TGFBR2 and overexpressed ACVR1 in HCC cell lines HepG2 and Hep3B cells, and found that down-regulated TGFBR2 and up-regulated ACVR1 dramatically promoted HCC invasion via enhancing EMT and stemness in vitro. The global miRNA profiling analysis indicated that miR17 cluster (including miR-17, miR-18a, miR-19a, miR-19b, miR-20a and miR-92a) are the key triggers to cause the imbalance of TGF-β1/BMP7 pathways. The overexpression of MIR17HG inhibited the TGF-β pathway, but activated the BMP7 pathway simultaneously, which promoted HCC invasion and progression in vitro and vivo. The multicolor immunofluorescence staining assay indicated that the recruitment of M2-polarized tumor associated macrophages (M2-TAMs) in HCC tissues positively correlated with the imbalance of the TGF-β1/ BMP-7 pathways and poor clinical outcome. And co-culture of HCC cells with TAMs dramatically enhanced the levels of miR17 cluster and promoted HCC EMT.

**Conclusions**

In summary, the miR-17 cluster promoted M2-TAMs related cell aggressiveness via inducing the imbalance of TGF-β1/BMP7 pathways in HCC.

**Acknowledgements**

This work was supported by National Natural Science Foundation of China (grant numbers: 81872143, 81702280, 81472473, 81272360) and Key Project of Tianjin Health and Family Planning Commission (16KG126).

**Ethics Approval**

This study was approved by the Ethics Committee of Tianjin Medical University (No. TMUhMEC 2012018). All experiments were performed in accordance with the principles of the Declaration of Helsinki and written consent was obtained from all patients.

### Various

#### P849 Melanoma-associated vitiligo skin maintains resident memory CD8 T cells with specific TCR profiles contributing to long-term tumor protection

##### Jichang Han, Aleksey Molodtsov, Yanding Zhao, Keisuke Shirai, Mary Jo Turk, Christina V. Angeles

###### Dartmouth College, Lebanon, NH, United States

####### **Correspondence:** Christina V. Angeles (christina.v.angeles@hitchcock.org)

**Background**

Vitiligo, an immune-related adverse effect (irAE) which targets normal melanocytes in the skin, is associated with significantly improved overall survival in metastatic melanoma patients treated with immunotherapy [1]. The presence of tissue resident memory T cells (TRM) correlates with better prognosis in multiple cancer types and our lab previously showed that the generation of potent TRM responses against melanoma cells depends on the induction of autoimmune vitiligo [2,3]. The present studies test the hypothesis that TRM in melanoma patient’s vitiligo skin shares the same TCR clonotypes with tumor infiltrating lymphocytes (TILs) which indicates that TRM provides durable, long-term tumor protection.

**Methods**

Seven melanoma survivors who received immunotherapy with the irAE of vitiligo were recruited to the study. Genomic DNA was extracted from matched skin, tumor and blood specimens and TCRβ chain deep sequencing was performed by Adaptive Biotechnologies. Fresh matched tumor, skin and blood samples from four patients were collected, and paralleled single cell RNA (scRNA) and single cell TCR (scTCR) sequencing were performed using the 10X platform. Gene expression profiles were analyzed using the Seurat V3.0 package. Matched TCR clonotypes were identified as those TCRβ chain sequences in bulk level TCR sequencing, or paired TCRα, β chain sequences in scTCR sequencing, that are the same between different tissues.

**Results**

TRM-like CD8 T cells were found in all melanoma patients’ vitiligo skins. Instead of a single population, one of the TRM-like CD8 T cell populations had highly upregulated IFN-γ, TNF, CXCR3, GZMA, GZMB, NKG7 and PFN1 genes (TRM-IFNG), while the other population expressed high levels of AP-1 transcription factors (TRM-AP1). Matched T-cell clonotypes were found among tumor, skin and blood and phenotypes. These clonotype-matched T cells included both TRM-like and circulating memory T cells. Tumor-skin matched TCR repertoire had significantly higher clonal expansion level than tumor-blood matched TCR repertoire. The TCR repertoire of TRM-like T cells with matched clonotypes were more clonally expanded compared to that of the circulating memory T cells. Focusing on the TRM-like population, TRM-IFNG had a higher enriched level of matched T-cell clonotypes between tumor and skin compared to the TRM-AP1 population. Using TCGA metastatic melanoma patients’ datasets, patients with TRM-IFNGhigh TRM-AP1high gene expression profiles had better prognosis compared to patients with TRM-IFNGlow TRM-AP1high gene expression profiles.

**Conclusions**

These data suggest that TRM-like CD8 T cells with TCR clonotypes matched between tumor and skin maintains a tumor protecting population in long-term melanoma survivors’ vitiligo skin.

**References**

1. Hua C, Boussemart L, Mateus C. Association of Vitiligo With Tumor Response in Patients With Metastatic Melanoma Treated With Pembrolizumab. JAMA Dermatol. 2016; 152:45-51
2. Malik BT, Byrne KT, Vella JL. Resident memory T cells in the skin mediate durable immunity to melanoma. Sci Immunol. 2017; 2
3. Molodtsov, A., Turk, M.J. Tissue Resident CD8 Memory T Cell Responses in Cancer and Autoimmunity. Front Immunol. 2018; 9:2810

**Ethics Approval**

This study was approved by Dartmouth College/Dartmouth Hitchcock Medical Center's Committee for the Protection of Human Subjects, approval number 29821

#### P850 Metabolically reprogram MDSCs by targeting Pim kinases to overcome resistance to PD-1 blockade immunotherapy

##### cYao Chen^2^, Paytsar Topchyan^2^, Aimin Jiang, PhD^3^, Yiliang Chen^1^, Roy Silverstein^2^, Weiguo Cui^1^ , Gang Xin^1^

###### ^*1*^*Blood Center of Wisconsin, Milwaukee, WI, United States;*^*2*^*Medical College of Wisconsin, Milwaukee, WI, United States*; ^*3*^*Roswell Park Cancer Institute, Buffalo, NY, United States*

####### **Correspondence:** Weiguo Cui (Weiguo.Cui@BCW.edu)

**Background**

Despite the unprecedented success of immune checkpoint blockade (ICB) therapy, many patients still respond poorly, owing to multiple resistance mechanisms. Among these mechanisms, myeloid cells, constitute the largest populations of immune cells in the tumor microenvironment, and are considered as one of the major barriers for successful therapy and the promoters for immune evasion and metastasis.). Despite the frequent clinical observation that patient with high levels of tumor-infiltrating myeloid cells usually fail to respond to ICB therapy and correlate with worse prognosis (ref), little is known about the crucial mechanisms governing these cells. Consequently, limited approaches have been developed to effectively abolish the intratumoral myeloid cells to restore sensitivity to ICB therapy.

**Methods**

To address these knowledge gaps, we employed single-cell sequencing (scRNA-seq) to assess transcriptional and metabolic changes in myeloid cells that associated with resistance to PD-1 blockade in preclinical cancer models.

**Results**

Our study revealed that the resistant tumor was dominated by immunosuppressive myeloid cells enriched for a fatty acid metabolism gene expression signature, which can be used as biomarker to distinguish responders from non-responders. Mechanistically, our data suggested that this fatty acid metabolism in intratumoral myeloid cells was regulated by Pim1, a serine/threonine kinase. Supporting this notion, genetic deletion of Pim1 in myeloid cells repressed PPARγ pathway activity, which in turn substantially abrogated the fatty acid oxidation and immunosuppressive function. More intriguingly, using AZD1208 to pharmacologically inhibit Pim kinase disrupted the myeloid cell-mediated immunosuppressive microenvironment and promoted T cell-mediated tumor regression, working synergistically with anti-PD-L1 therapy in various solid tumor models. Additionally, sensitivity to ICB in non-responders can be restored by selective treatment with AZD1208.

**Conclusions**

Overall, we have identified Pim1 as a metabolic modulator in MDSCs that may cause ICB resistance and can be therapeutically inhibited to sensitize non-responsive tumors to ICB therapy.

**Fig. 1 (abstract P850).**
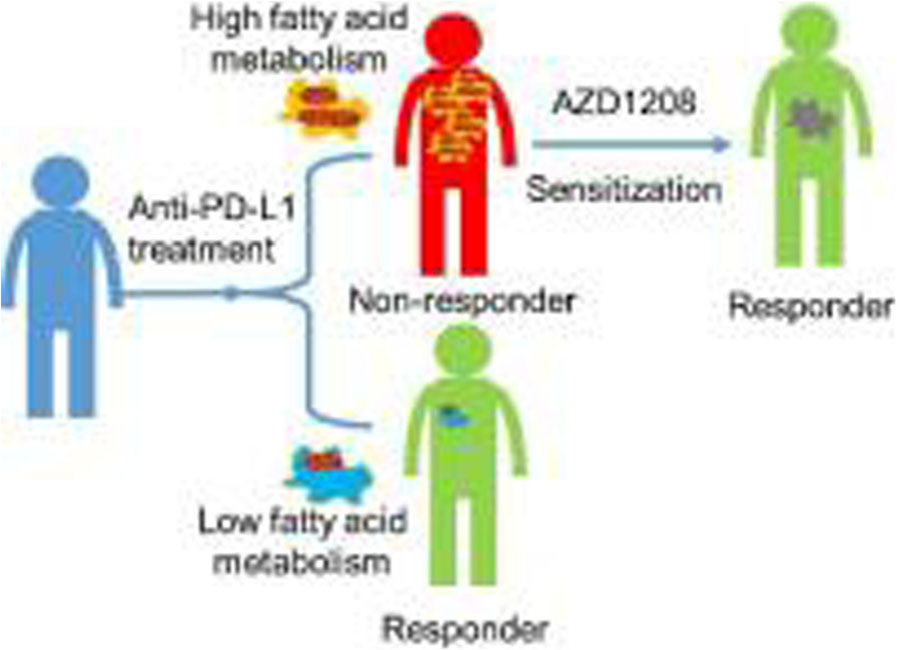
A precision medicine approach for overcome resistance to ICB

## Oral Presentations

### Biomarkers, Immune Monitoring, and Novel Technologies

#### O1 Flexible Copper-64-nanoparticle-based cell labeling system allows for in vivo tracking of adoptively transferred T-cells by PET/CT

##### Hólmfridur Halldórsdóttir, MSc^1^, Lars Ringgaard, PhD^1^, Martin Bak, PhD^1^, Ditte Jæhger, MSc^1^, Esben Christensen, MSc^1^, Fredrik Melander, PhD^1^, Mie Hübbe, MSc^1^, Rasmus Eliasen, PhD^1^, Frederikke Fliedner, MSc^2^, Andreas Kjaer, MD, PhD^3^, Anders Hansen, DVM, PhD^1^, Jonas Henriksen, PhD^1^, Thomas Andresen, PhD^1^

###### ^*1*^*Technical University of Denmark, Kongens Lyngby, Denmark;*^*2*^*University of Copenhagen, Copenhagen, Denmark*; ^*3*^*Rigshospitalet & University of Copenhagen, Copenhagen, Denmark*

####### **Correspondence:** Thomas Andresen (tlan@dtu.dk)

**Background**

To fully exploit the potential of adoptive cell transfer (ACT) therapy, an improved understanding of in vivo T-cell trafficking and engraftment following transfer is paramount. In vivo tracking of adoptively transferred cells by PET/CT represents an attractive imaging technology for ACT due to the quantitative capacity with high spatial and temporal resolution [1]. We have developed a flexible copper-64-micelle-based cell labeling system, which allows for prolonged cell tracking.

**Methods**

The labeling protocol was optimized for T-cell copper-64-micelle labeling in vitro and >90% loading efficiency was achieved with intact cell viability. Stability of the copper-64-micelles labeled T-cells was evaluated by co-incubating the T-cells with celltrace stained splenocytes for three and 24 hours. Following co-incubation, cells were FACS-sorted and copper-64 activity in population determined. The radiolabeled T-cell population displayed a 10-14-fold higher copper-64 activity level compared to celltrace+ splenocytes. In vivo, labeled T cell biodistribution and tumor accumulation in mice were investigated using PET/CT imaging in relation to radiation and various cancer immunotherapies. PET/CT scans were performed at multiple time-points up to 40 hours after ACT.

**Results**

Whole-body irradiated mice displayed significantly higher copper-64 activity (4.6 +/- 0.1% of injected dose) in the thymus compared to controls (2.1 +/- 0.1% of injected dose). Additionally, there was correspondence between T cell number of the injected clone in selected organs and radioactivity. This demonstrates that the signal is T-cell specific and retained in vivo in T-cells over time. We investigated the activity of labeled T-cells in tumors treated with an intratumoral sustained release depot of a TLR7 agonist (TLR7 Immunogel). The TLR7 Immunogel has been demonstrated to increase tumor protein levels of chemokines; CXCL-10, IFN-gamma, and TNF-alpha, with importance for T-cell accumulation. PET/CT imaging showed that accumulation of ACT labeled T-cells was increased by pre-treating tumors with TLR7 Immunogel.

**Conclusions**

This flexible radiolabeling technology provides a novel method for tracking T-cells in vivo over several days. The flexibility of the system offers labeling of multiple murine and human cell populations, for high-resolution in vivo cell tracking.

**References**

1. Tumeh PC, Radu CG, Ribas A. PET Imaging of Cancer Immunotherapy. J Nucl Med [Internet]. 2008;49(6):865–8. Available from: http://jnm.snmjournals.org/cgi/doi/10.2967/jnumed.108.051342

**Ethics Approval**

The study has been approved by the The Danish Animal Experiments Inspectorate.

#### O2 Combining transcriptomic and tissue-based immune biomarkers to improve recurrence prediction in stage II-III melanoma

##### Robyn Gartrell, MD^2^, Andrew Chen, BS^3^, Emanuelle Rizk, BA^2^, Douglas Marks, MD^4^, Margaret Bogardus, BA^3^, Basil Horst, MD^5^, Rui Chang, MD^6^, Anthea Monod, PhD^7^, Raúl Rabadán, PhD^2^, Yvonne Saenger, MD^2^

###### ^1^Columbia University/ New York Presbyterian, Newton, NJ, United States; ^2^Columbia University Irving Medical Cente, Newton, NJ, United States; ^3^Columbia University, New York, NY, United States ; ^4^New York University Langone Health, Mineola, NY, United States; ^5^University of British Columbia, Vancouver, Canada; ^6^University of Arizona, Tucson, AZ, United States; ^7^Tel Aviv University, Tel Aviv, Israel

####### **Correspondence:** Yvonne Saenger (yms4@cumc.columbia.edu)

**Background**

Biomarkers that predict clinical response in stage II-III melanoma are critical to helping clinicians weigh benefits of immunotherapy against potential toxicities. Individual biomarkers are often insufficient to clearly stratify patients, thus the combination of transcriptomic and tissue-based immune biomarkers may help identify more precise and predictive biomarkers. We previously found that the ratio of CD8+ cytotoxic T lymphocytes (CTLs) to CD68+ macrophages and melanoma immune profile (MIP), a 53 immune gene signature, both predict recurrence and disease specific survival (DSS) in patients with primary stage II-III melanoma [1,2].

**Methods**

We performed quantitative multiplexed immunofluorescence (qmIF) and nanoString analysis on a cohort of 78 patients with stage II-III melanoma and known DSS who were diagnosed at Columbia University Irving Medical Center (CUIMC) between 2000 and 2012. MIP was computed based on gene expression using nanoString, and the CTL/macrophage ratio was found using qmIF. We assessed the similarity of each sample’s gene expression profile to the cellular subtype signatures from LM22, the reference standard for CIBERSORT. Differential expression was evaluated using t-test with Benjamini-Hochberg (BH) correction. We used Spearman correlation to evaluate the association of the top immune genes (>1 fold change) with the CTL/macrophage ratio. We combined the CTL/macrophage ratio with MIP using the log-rank (Mantel-Cox) test to compare low- to medium-risk, low- to high-risk, and medium- to high-risk patients.

**Results**

We found that recurrent patients had decreased M1 and increased M2 macrophage signatures relative to non-recurrent patients (p=0.042 and p=0.048, respectively) using CIBERSORT. The M1 macrophage signature further correlated with density of HLA-DR+ macrophages as assessed by qmIF (p=0.022). Next, we evaluated the differential expression of genes in MIP identifying 9 with >1.0 log-fold change and significant by BH. We then evaluated the correlation of these 9 genes with stromal density of CD3+, CD8+, and CD68+, as well as the ratio of CTL/macrophage as determined by qmIF. Importantly, the CTL/macrophage ratio had no correlation with these 9 genes, allowing for combination of MIP with the CTL/macrophage ratio. This composite biomarker significantly distinguished low- and medium-risk patients from high-risk patients (Figure 1, p=0.0031 and p=0.0258, respectively).

**Conclusions**

Combining multiple modalities to stratify patients into risk groups is a critical step to improving the management of melanoma patients. Here, we find that a composite biomarker consisting of the MIP signature and CTL/macrophage ratio successfully stratifies patients into three risk groups predictive of DSS, thus highlighting the potential use of combination biomarkers for adjuvant therapy.

**Acknowledgements**

The authors of this publication were supported by the National Institutes of Health through Grant Numbers R01FD006108 (Y.M. Saenger) and KL2TR001874 (R.D. Gartrell-Corrado) A.X.C is funded by the Medical Scientist Training Program (T32GM007367). Yvonne Saenger is also supported by funding from the Melanoma Research Alliance and by an Irving Assistant Professorship at Columbia University’s NIH/NCATS CTSA Program hub: UL1TR001873. Robyn Gartrell-Corrado is also supported by Swim Across America. R. Rabadán, A. Monod, and A. Chen have been supported by the NCI Center for Topology of Cancer Evolution and Heterogeneity (U54CA193313). A. Monod wishes to acknowledge the support of the New Frontiers in Research Fund--Fonds Nouvelles Frontières en Recherche (SSHRC-NFRF-FNFR, Government of Canada) NFRE-2018-00431. The content is solely the responsibility of the authors and does not necessarily represent the official views of the NIH. The funding sources had no role preparation of the manuscript or the decision to submit for publication.

**References**

1. Gartrell RD, Marks DK, Hart TD, et al: Quantitative Analysis of Immune Infiltrates in Primary Melanoma. Cancer Immunol Res 6:481-493, 2018

2. Gartrell RD, Marks DK, Rizk EM, et al: Validation of Melanoma Immune Profile (MIP), a Prognostic Immune Gene Prediction Score for Stage II-III Melanoma. Clin Cancer Res, 2019

**Ethics Approval**

This study was approved by Columbia University Irving Medical Center’s (CUIMC) Institutional Review Board, Protocol AAAO2758

**Fig. 1 (abstract 02).**
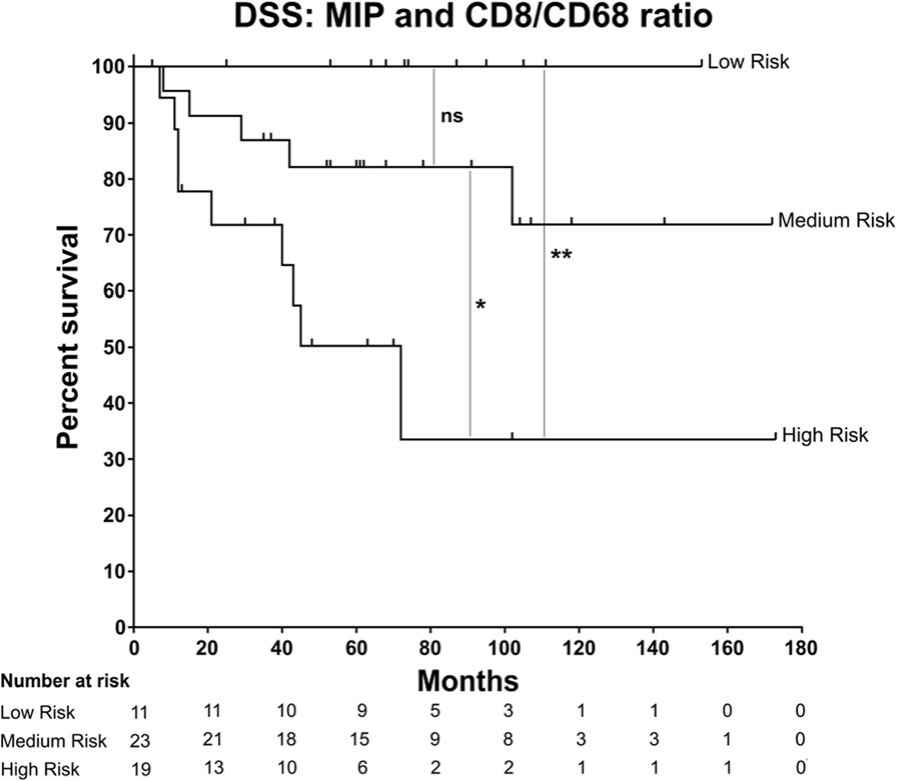
Combination of MIP and CD8/CD68 ratio predicts DSS

#### O3 Immune infiltration correlates with TP53 mutational status in a multi-cohort acute myeloid leukemia study

##### Sergio Rutella, MD, PhD, FRCPath^1^ , Jayakumar Vadakekolathu, PhD^1^, Stephen Reeder, BS^1^, Jenny Ashforth^1^, Melissa Courtney^1^, Amanda Coutts, PhD^1^, Tressa Hood, MS^2^, Sarah Church, PhD^2^, Clare Coveney, PhD^1^, Jan Davidson-Moncada, MD, PhD^3^, Jorn Meinel, MD^4^, Marc Schmitz, MD^4^, Francesco Marincola, MD^5^, Martin Bornhauser, MD^4^

###### ^*1*^*Nottingham Trent University, Nottingham, United Kingdom;*^*2*^*NanoString Technologies, Inc., Seattle, WA, United States;*^*3*^*MacroGenics, Inc., Rockville, MD, United States*; ^*4*^*University Hospital Carl Gustav Carus, Dresden, Germany*; ^*5*^*Refuge Biotechnologies, Half Moon Bay, CA, United States*

####### **Correspondence:** Sergio Rutella (sergio.rutella@ntu.ac.uk)

**Background**

Acute myeloid leukemia (AML) is a molecularly and clinically heterogeneous hematological malignancy. Although immunotherapy may be an attractive modality to exploit in AML, the ability to predict the groups of patients and the types of cancer that will respond to immune targeting remains limited [1-2]. Herein, we aimed to determine whether prognostic molecular lesions, including TP53 mutations, correlate with the AML immune milieu and identify a patient subgroup that may benefit from immunotherapy approaches.

**Methods**

The Cancer Genome Atlas (TCGA) discovery cohort consisted of RNA-sequencing data from 162 AML patients with complete cytogenetic and clinical annotation. The second public cohort consisted of RNA-sequencing data from 281 AML patients treated on the Beat AML Master Trial. Immune cell type-specific and biological activity scores were computed as previously published [3]. GeoMx(TM) Digital Spatial Profiling (DSP) [4] was used to characterize the expression of 31 immuno-oncology-related proteins in FFPE bone marrow (BM) samples from 10 patients with newly-diagnosed AML (SAL cohort).

**Results**

Immune gene sets stratified TCGA-AML patients into two distinct clusters expressing genes associated with adaptive immune responses and an interferon (IFN)-gamma-dominant tumor microenvironment (TME) and gene modules capturing BM infiltration with monocytes, macrophages and other myeloid cells. Patients with adverse-risk molecular lesions, including somatic TP53 and RUNX1 mutations, clustered in the immune-infiltrated subgroup (Fig. 1A-B). In contrast, the majority of TCGA-AML cases with favorable/intermediate-risk molecular lesions were classified as immune-depleted. Similarly, 16 out of 17 TP53-mutated Beat AML cases (validation cohort) expressed high levels of CD8 transcripts, markers of cytotoxicity and downstream IFN signaling genes compared to TP53 wild-type (WT) cases. We next analyzed protein profiles in BM specimens from a third independent AML series (SAL cohort) using GeoMx DSP (Fig. 1C). We identified four microenvironmental protein signatures (SIG1-4). Abnormalities in SIG3 genes (i.e., mRNA up-regulation, gene amplification, deep deletion and mis-sense mutations of PD-L1, FoxP3, cytotoxicity molecules, PTEN and BCL2) were predominantly observed in TCGA patients with IFN-gamma-dominant AML and correlated with higher tumor mutational burden, adverse cytogenetic features, TP53 mutational status and worse clinical outcomes (Fig. 1D). Mechanistically, IFN-gamma inhibited chemotherapy-induced cell death in TP53-mutated Kasumi-1 AML cells treated with PRIMA-1 [6], a TP53 reactivator (Fig. 1E).

**Conclusions**

Congruent with previous reports in breast cancer with genetic instability [5], this study provides evidence for a correlation between IFN-gamma-dominant AML subtypes and TP53 mutations, an established adverse prognosticator, and could support the implementation of immunotherapy clinical trials in this specific patient subgroup.

**Acknowledgements**

Grant support: Qatar National Research Fund (#NPRP8-2297-3-494), Roger Counter Foundation (Dorset, UK) and John and Lucille van Geest Foundation to S.R.

**References**

1. Vadakekolathu J, et al. Immune gene expression profiling in children and adults with acute myeloid leukemia identifies distinct phenotypic patterns. Blood. 2017; 130: 3942A.

2. Rutella S, et al. Capturing the complexity of the immune microenvironment of acute myeloid leukemia with 3D biology technology. J Clin Oncol. 2018; 36: 50A.

3. Danaher P, et al. Gene expression markers of Tumor Infiltrating Leukocytes. J Immunother Cancer. 2017; 5: 18.

4. Rutella S, et al. Digital spatial profiling of bone-marrow infiltrating immune cells in acute myeloid leukemia. J Immunother Cancer. 2018; 6 (Suppl. 1): P113.

5. Hendrickx W, et al. Identification of genetic determinants of breast cancer immune phenotypes by integrative genome-scale analysis. Oncoimmunology. 2017; 6: e1253654.

**Ethics Approval**

The study was approved by Technical University of Dresden's Ethics Board.

**Fig. 1:**
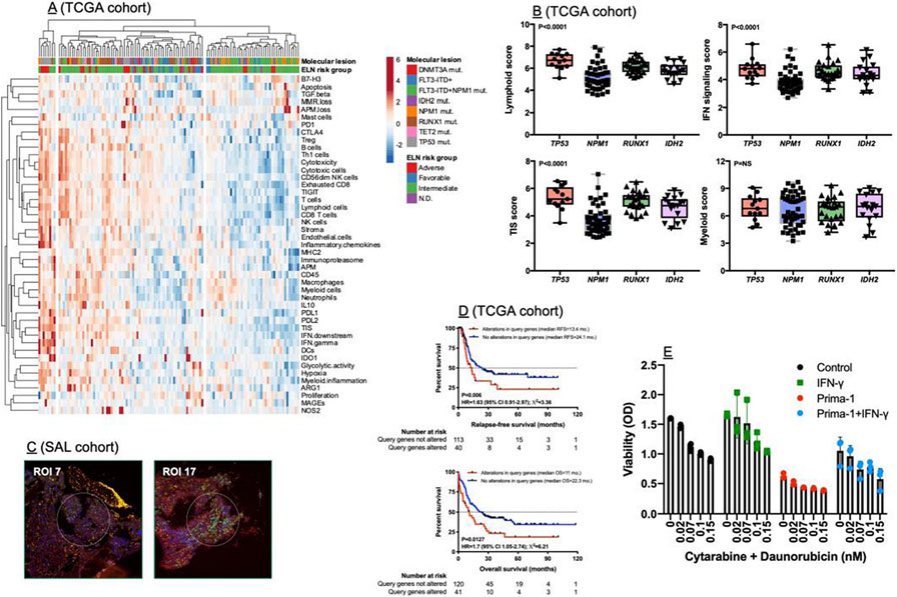
**(abstract 03).** See text for description

### Cellular Therapies

#### O4 Preclinical CAR-T cell target safety, biodistribution, and tumor infiltration analysis using in situ hybridization technology

##### Helly Xiao Yan Pimentel^1^, Helen Jarnagin^1^, Hailing Zong^1^, Courtney Todorov^1^, Kenneth Ganley^2^, Fay Eng^2^, Kevin Friedman, PhD^2^, Molly Perkins^2^, Shannon Grande, PhD^2^, Bingqing Zhang^1^, Christopher Bunker^1^, Xiao-Jun Ma^1^, Courtney Anderson, PhD^1^ , James Rottman^2^

###### ^1^Advanced Cell Diagnostics/Bio-Techne, Newark, CA, United States; ^2^Bluebird Bio, Cambridge, MA, United States

####### **Correspondence:** Courtney Anderson (courtney.anderson@bio-techne.com)

**Background**

Chimeric antigen receptor (CAR) T cell therapy has proven to be highly effective in treating hematologic malignancies, and major efforts are being made to achieve similar efficacy in solid tumors. The greater potency of CAR-T cells compared to antibody therapeutics demands a more stringent CAR-T target safety assessment to avoid adverse events resulting from “on-target/off-tumor” activity. Furthermore, it is critical to track and monitor CAR+ T cells within the context of intact tissue and tumor to understand the mechanisms underlying off-tumor toxicity and efficacy in tumor killing.

**Methods**

We employed the RNAscope in situ hybridization (ISH) technology to assess target expression specificity and to track CAR-T cell distribution and activation in xenograft and host tissues using the RPMI-8226 xenograft mouse model.

**Results**

For the CAR-T target candidates, BCMA and ROR1, RNA ISH revealed that BCMA was only expressed in the xenograft tumor and in no mouse organs, while ROR1 was found to be expressed at low levels in mouse lung and liver, as well as in the xenograft tumor. Duplex RNA ISH assays with probes targeting the CAR 3’ UTR and either IFNG or GZMB mRNA allowed for highly sensitive and specific detection of CAR-T cells and their activation state in both tumor and normal tissues from vehicle, anti-BCMA CAR-T cell, or anti-ROR1 CAR-T cell treated mice. Activated anti-BCMA CAR-T cells expressing GZMB and IFNG were found only in the xenograft tumor, where BCMA was expressed. In contrast, activated anti-ROR1 CAR-T cells were found almost exclusively in mouse lung and liver, with very few anti-ROR1 CAR-T cells being found in the xenograft tumor, consistent with the previously observed pulmonary and hepatic toxicity of anti-ROR1 CAR T cells that was not predicted by IHC analysis of ROR1 protein presumably due to lack of antibody sensitivity. Lastly, a multiplex ISH-IHC approach confirmed the presence of activated BCMA CAR-T cells in the xenograft tumor through simultaneous detection of the BCMA CAR 3’ UTR, IFNG, GZMB, and CD3.

**Conclusions**

These data demonstrate how the RNAscope assay can be utilized for CAR-T cell efficacy and safety/toxicity assessment in preclinical models by detecting very low levels of target antigen expression in off-tumor tissues and monitoring CAR-T cell pharmacodynamics and activation in tumor models and can also be applied for assessing TCR-T cell activity in tumors.

#### O5 The road-map to tumor-infiltrating lymphocyte (TIL) therapy: Understanding genetic alterations for improved patient treatment

##### Caitlin Creasy, MS^1^, Yuzhong Meng, PhD^2^, Tatiana Karpinets, MS^1^, Cara Haymaker, PhD^1^, Marie Andrée Forget, PhD^1^, Chip Stewart, PhD^3^, Carlos Antonio Torres Cabala, MD^1^, Shari Pilon-Thomas, PhD^4^, Amod Sarnaik, MD^4^, Levi Garraway, MD, PhD^3^, Patrick Hwu, MD^1^, Rodabe Amaria, MD^1^, Rameen Beroukhim, MD, PhD^3^, Chantale Bernatchez^1^

###### ^*1*^*UT MD Anderson Cancer Center, Houston, TX, United States;*^*2*^*Harvard Medical School, Boston, MA, United States*; ^*3*^*Broad Institute, Cambridge, MA, United States*; ^*4*^*Moffitt Cancer Center, Tampa, FL, United States*

####### **Correspondence:** Chantale Bernatchez (CBernatchez@mdanderson.org)

**Background**

Treatment of metastatic melanoma has vastly improved over the past decade due to the rise of immunotherapy. Adoptive cell therapy (ACT) of tumor-infiltrating lymphocytes (TIL) has yielded a 40-50% response rate in this subset of patients. Although this living therapy yields durable responses, it is unknown why some patients do not respond or are not afforded lasting protection. Previously, we have demonstrated that phenotypic differences of the infused TIL, LDH levels, as well as number and type of prior systemic therapies impact responses. To gain further insight into the role of genetic alterations and response to TIL therapy, we interrogated pre-treatment tumor tissue from TIL treated patients.

**Methods**

Tumor samples were collected from 74 patients from MD Anderson Cancer Center (MDACC) and 10 patients from Moffitt Cancer Center. A subset of patients from MDACC also had tumor biopsies collected at recurrence (n=6). Whole exome sequencing (WES), and RNA sequencing, were assessed along with response, progression free survival (PFS), and overall survival (OS).

**Results**

WES on pre-treatment tumor tissue first revealed that a high neoantigen burden is predictive of long-term OS, but is not associated with PFS or response to treatment. Interestingly, when compared with mutation rates found in TCGA, we found mutations of the genes KCNQ2 and SFTA3 were enriched in the overall patient cohort from both centers (1.9 vs 13% for KCNQ2 and 0.6 vs 6% for SFTA3). To our knowledge, this observation was never reported prior and could be linked to the difference in stage of the disease between TCGA (mostly stage III) and our cohort (stage IV). When assessing the clonal evolution in the biopsies collected at recurrence vs the matched pre-treatment tumor tissue, no common driver DNA alteration could be identified, however changes in the prevalence of tumor subclones could be observed. RNA sequencing analysis revealed three genes significantly enriched in patients experiencing long-term PFS, long-term OS, and response to therapy (PDE1C, RTKN, and NGFR), while one gene enriched with the inverse outcomes (ELFN1).

**Conclusions**

Using genetic information, we uncovered potential biomarkers for response to TIL ACT in metastatic melanoma which can be assessed prior to treatment. This analysis also highlights potential routes of immune escape.

**Trial Registration**

Trial NCT00338377 was conducted at UT MD Anderson Cancer Center and trials NCT01005745, NCT01701674, and NCT01659151 were conducted at Moffitt Cancer Center.

**Ethics Approval**

This study was approved by UT MD Anderson Cancer Center's and Moffitt Cancer Center's Ethics Board.

#### O6 Enhanced presence of a myeloid-like NK cell subpopulation is associated with MHC class I-deficient tumor escape

##### Yimin Du, PhD^1^, Curtis Perry, MD, PhD^2^, William Damsky, MD, PhD^2^, Katherine Hastings, PhD^2^, Patricia Rodriguez-Morales^1^, Dan Chen, PhD^1^, Kacie Traina^1^, Ivy Phung^3^, Ting Zhou^2^, Aaron Ring, MD, PhD^2^, Katerina Politi, PhD^2^, Susan Kaech, PhD^1^

###### ^*1*^*Salk Institute for Biological Studies, La Jolla, CA, United States;*^*2*^*Yale University, New Haven, CT, United States* ; ^*3*^*University of California San Diego, La Jolla, CA, United States*

####### **Correspondence:** Susan Kaech (skaech@salk.edu)

**Background**

Recent advances in immunotherapies (e.g. anti-PD-1 or –CTLA-4) aiming to enhance anti-tumor immunity have shown promising results in cancer patients. However, resistance to these therapies is a major challenge we face today [1]. An emerging mechanism of cancer resistance to immune checkpoint inhibitors is impairment of major histocompatibility class I (MHC-I) antigen presentation through disruption of B2M (encoding a critical component of MHC-I) [2]. While it is intuitive that compromised CD8 T-cell responses are directly responsible for defective control of tumors with low or absent MHC-I, it is critical that we better understand why other immune cells, especially natural killer (NK) cells, also fail to suppress the growth of these types of tumors, as they are known for their rapid and effective response targeting infected cells with low or absent MHC-I expression [3].

**Methods**

Using CRISPR/Cas9-mediated genetic knockout of B2M in our murine melanoma model, as well as single-cell RNA sequencing (sc-RNAseq) and flow cytometry, we were able to effectively probe the phenotype and function of tumor-infiltrating NK cells.

**Results**

Our preliminary sc-RNAseq analysis indicated that B2M-/- (B2M-KO) tumoral NK cells appear to acquire a “neo-phenotype” accompanied by increased expression of myeloid cell-like genes (Figure 1). We hypothesize these genetic changes underlie the functional impairments in MHC-I-deficient tumoral NK cells. Further validation using both standard (Figure 2) and imaging-based flow cytometry indicated that B2M-KO tumoral NK cells exhibit enhanced co-expression of CSF1R and C5AR, two molecules that are critical for myeloid survival and differentiation and are known to disrupt effector CD8 T-cell and NK cell functions. Moreover, our analysis revealed impaired effector and cytotoxic capacity of C5AR/CSF1R co-expressing NK sub-population. Additionally, treating tumor-bearing B6 mice with a CSF1R inhibitor led to delayed B2M-KO tumor growth that is NK-dependent (Figure 3).

**Conclusions**

We hereby demonstrate enhanced presence of a myeloid-like NK sub-population in the context of B2M or MHC-I deficiency in our murine melanoma model, for which such phenotype is linked to compromised anti-tumor immunity. Future works will seek to find out if myeloid-like phenotypes are observed in tumoral NK cells in human cancer patients. Our research, therefore, has significant implications in cellular and therapeutic targeting for CD8 T-cell resistant tumors.

**References**

1. Brahmer J, Reckamp KL, Baas P, Crino L, Eberhardt WE, Poddubskaya E, Antonia S, Pluzanski A, Vokes EE, Holgado E, Waterhouse D, Ready N, Gainor J, Aren Frontera O, Havel L, Steins M, Garassino MC, Aerts JG, Domine M, Paz-Ares L, Reck M, Baudelet C, Harbison CT, Lestini B, Spigel DR. Nivolumab versus Docetaxel in Advanced Squamous-Cell Non-Small-Cell Lung Cancer. N Engl J Med. 2015; 373:123-35.

2. Zaretsky JM, Garcia-Diaz A, Shin DS, Escuin-Ordinas H, Hugo W, Hu-Lieskovan S, Torrejon DY, Abril-Rodriguez G, Sandoval S, Barthly L, Saco J, Homet Moreno B, Mezzadra R, Chmielowski B, Ruchalski K, Shintaku IP, Sanchez PJ, Puig-Saus C, Cherry G, Seja E, Kong X, Pang J, Berent-Maoz B, Comin-Anduix B, Graeber TG, Tumeh PC, Schumacher TN, Lo RS, Ribas A. Mutations Associated with Acquired Resistance to PD-1 Blockade in Melanoma. N Engl J Med. 2016; 375:819-29.

3. Ljunggren HG, Karre K. In search of the 'missing self': MHC molecules and NK cell recognition. Immunol Today. 1990; 11:237-44.

**Ethics Approval**

The study was approved by Salk Institute's Institution Review Board (IRB), approval number 10086.

**Fig. 1 (abstract 06).**
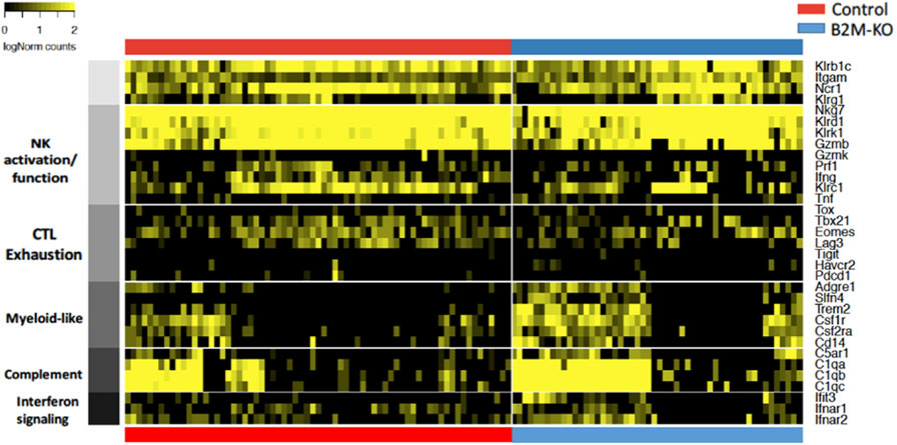
B2M-KO tumoral NK cells have significant myeloid signatures

**Fig. 2 (abstract 06).**
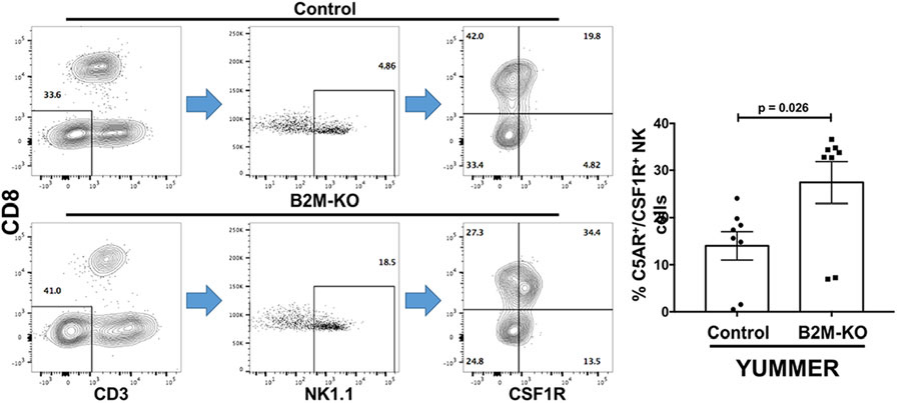
B2M-KO tumoral NK cells have higher C5AR/CSF1R co-expression

**Fig. 3 (abstract 06).**
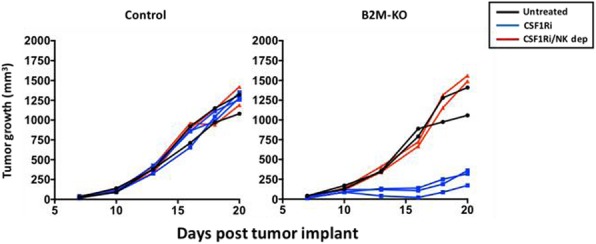
Anti-CSF1R treatment leads to delayed B2M-KO tumor growth

#### O7 P-BCMA-ALLO1 — a nonviral, allogeneic anti-BCMA CAR-T therapy with potent antitumor function for the treatment of multiple myeloma

##### Maximilian Richter, PhD, Stacey Cranert, PhD, Yening Tan, MS, Min Tong, MS, Christine Domingo, BS, Elvira Argus, PhD, Samad Ibitokou, PhD, Jenessa Smith, PhD, Christopher Martin, PhD, Xinxin Wang, PhD, Burton Barnett, PhD, Eric Ostertag, MD, PhD, Julia Coronella, PhD, Devon Shedlock, PhD

###### Poseida Therapeutics, San Diego, CA, United States

####### **Correspondence:** Devon Shedlock (shedlock@poseida.com)

**Background**

Chimeric antigen receptor (CAR)-T therapy has shown great promise against relapsed/refractory multiple myeloma (MM) by targeting the B cell maturation antigen (BCMA), an example being Poseida’s P-BCMA-101 product candidate. Nevertheless, the need to manufacture an individualized product from highly variable patient material, lengthy vein-to-vein time and high manufacturing cost remain challenges of autologous CAR-T. Employing the nonviral piggyBac® (PB) DNA Modification System in combination with the high-fidelity Cas-CLOVER™ (CC) Site-Specific Gene Editing System , we have developed P-BCMA-ALLO1, an off-the-shelf allogeneic CAR-T cell product candidate aimed at overcoming the limitations of autologous therapy.

**Methods**

For manufacture of P-BCMA-ALLO1 from healthy donor T cells, the PB system is used to stably deliver the transgene, while the CC system is used to knockout expression of T cell receptor (TCR) and MHC, while avoiding unwanted off-target cutting. We also use a proprietary “booster molecule” to increase the yield, enabling the production of hundreds of patient doses from a single manufacturing run. P-BCMA-ALLO1 final product is >95% CAR+, >99.5% TCR-KO and is comprised of a high percentage of desirable TSCM cells (CD45RA+, CD62L+), similar to P-BCMA-101, contributing to safety and durability.

**Results**

Preclinical evaluation of P-BCMA-ALLO1 demonstrated potent efficacy in coculture assays against multiple BCMA+ cell lines. In addition, P-BCMA-ALLO1 secreted IFNγ and mediated robust (70-90%) killing of cell targets. Moreover, product generated from multiple different donors showed strong potency in an NSG xenograft model implanted with RPMI-8226, an aggressive human MM-derived tumor model. Untreated animals showed rapid tumor progression and succumbed to disease within the standard 5-7 weeks. In contrast, mice treated with P-BCMA-ALLO1 from any donor showed reduction of tumor burden below the limit of detection by caliper within ~2 weeks and remained tumor-free for the duration of the study. TCR-KO P-BCMA-ALLO1 cells were readily detectable in the blood of tumor-bearing animals, demonstrating good durability in mice.

**Conclusions**

Altogether, P-BCMA-ALLO1 exhibited potent effector function in preclinical studies and will be rapidly advanced into the clinic for the treatment of MM.

#### O8 The Thrombospondin-1/CD47 signaling axis serve as potential markers of immune checkpoint blockade response modulating immune cell bioenergetics in the tumor microenvironment.

##### Elizabeth Stirling, Adam Wilson, Mitra Kooshki, Lilya Yamaleyeva, Guanxu Jin, Wei Zhang, Lance Miller, MS, PhD, Pierre Triozzi, MD, David Soto-Pantoja, PhD

###### Wake Forest School of Medicine Comprehensive Cancer Center, Winston Salem, NC, United States

####### **Correspondence:** David Soto-Pantoja (dsotopan@wakehealth.edu)

**Background**

CD47 is a widely expressed receptor that controls innate and adaptive immunosurveillance by engaging its counter receptor SIRPα or by binding to its ligand Thrombospondin-1 (TSP1). TCGA data shows that high expression of TSP1 is associated with low survival probability of melanoma patients and its expression is elevated in metastatic melanoma versus localized lesions.

**Methods**

TSP1 expression was examined by single cell RNA sequencing in melanoma patient’s peripheral blood mononuclear cells (PBMCs) and was validated by ELISA . Expression of CD47 was examined by Flow cytometry. T cell antigen-specific responses were determined using isolated CD8+ T cells from Pmel-1 transgenic mice in an in vitro cell impedance platform. Cellular bioenergetics was measured using a Seahorse® bioanalyzer. Mechanisms of TSP1/CD47 regulation of the tumor microenvironment were examined in the B16 melanoma model and oxygen tension was measured by photoacoustic imaging.

**Results**

scRNAseq shows that TSP1 expression localizes to the myeloid population (CD163+, CD11b+) and it is induced in patients after the first cycle of anti-PD1 therapy suggesting that TSP1 could serve as a potential marker of immune checkpoint blockade (ICB) response (N=16, *p

**Conclusions**

Our data indicates TSP1/CD47 expression serves as markers of ICB response and targeting this pathway may impact cell bioenergetics to reduce tumor burden.

**Acknowledgements**

DSP is supported by The NCI Career Development Award (1K22CA181274) and ERS is supported by a NIGMS T32 (GM127261).

**Ethics Approval**

Human subject studies were approved by the institutional review board (IRB) and animal studies were approved by the Institutional Animal Care and Use Committee, Wake Forest Health Sciences.

#### O9 Enhancing affinity of CD22-directed CAR T cells increases activation signaling and in vivo response to CD22lo leukemia

##### Zachary Walsh, BA, Mark Kohler, MD, PhD, Terry Fry, MD

###### Children's Hospital Colorado, Aurora, CO, United States

####### **Correspondence:** Terry Fry (terry.fry@ucdenver.edu)

**Background**

T cells modified with a chimeric antigen receptor (CART) targeting CD22 have demonstrated exciting efficacy in clinical trials, inducing remission in 70% of patients with relapsed and refractory B-precursor acute lymphoblastic leukemia (B-ALL) including those resistant to CD19 targeted immunotherapy [1,2]. However, follow-up data from these trials has demonstrated that a considerable percentage of patients will relapse following CD22-directed CAR T cell therapy (CD22-CART), commonly with decreased leukemic expression of CD22.

**Methods**

In this study, we investigated the mechanistic connection of antigen density to CART function measured by the phosphoproteomic signature upon CAR stimulation. To model a spectrum of leukemic CD22 expression, we used previously-generated variants of the NALM6 B-ALL cell line which are modified to stably express varying levels of CD22. We studied CD22-CART signaling in the presence of these cell lines using phospho-flow cytometry. We further evaluated CD22-CART efficacy in vivo using a murine xenograft model of refractory B-ALL.

**Results**

We found that decreased CD22 antigen density on target leukemia cells is associated with a diminished percentage and intensity of ERK phosphorylation in CD22-CART, an indicator of T cell activation. These findings were corroborated by an observed decrease in the T cell activation markers PD-1 and CD69 as leukemic CD22 expression decreased. In an attempt to improve CD22 CART efficacy in the setting of low antigen density, we explored multiple mechanisms to improve CD22-CART signaling. Using a high affinity variant of the CD22-CAR (CD22-CARTV1), we noted increased in both the p-ERK intensity and percentage of p-ERK-positive CART in response to CD22lo leukemia in vitro, which led to improved in vivo leukemic clearance and CAR persistence. Additionally, using the BRAF inhibitor Vemurafenib, we were able to increase the intensity of p-ERK in CD22-CART. Finally, we noted that PD1hi and CD69hi CD22-CART demonstrated a higher percentage of p-ERK after antigen stimulation, suggesting that expression of these markers may be predictive of CART response.

**Conclusions**

Together, our results indicate the promise of the high affinity CD22-CARTV1 for the treatment of CD22lo leukemia and suggest that modification to CART design and targeted therapies to enhance signaling are viable, clinically relevant strategies to improve CART efficacy and persistence.

**References**

1. Fry TJ, Shah NN, Orentas RJ, Stetler-Stevenson M, Yuan CM, Ramakrishna S, et al. CD22-targeted CAR T cells induce remission in B-ALL that is naive or resistant to CD19-targeted CAR immunotherapy. Nat Med. 2018; Jan;24(1):20–8.

2. Ramakrishna S, Highfill SL, Walsh Z, Nguyen SM, Lei H, Shern JF, et al. Modulation of Target Antigen Density Improves CAR T Cell Functionality and Persistence. Clinical Cancer Research. 2019;25(17):5329–41.

**Ethics Approval**

This study was approved by the University of Colorado’s Institutional Animal Care and Use Committee, approval number 00751.

**Fig. 1:**
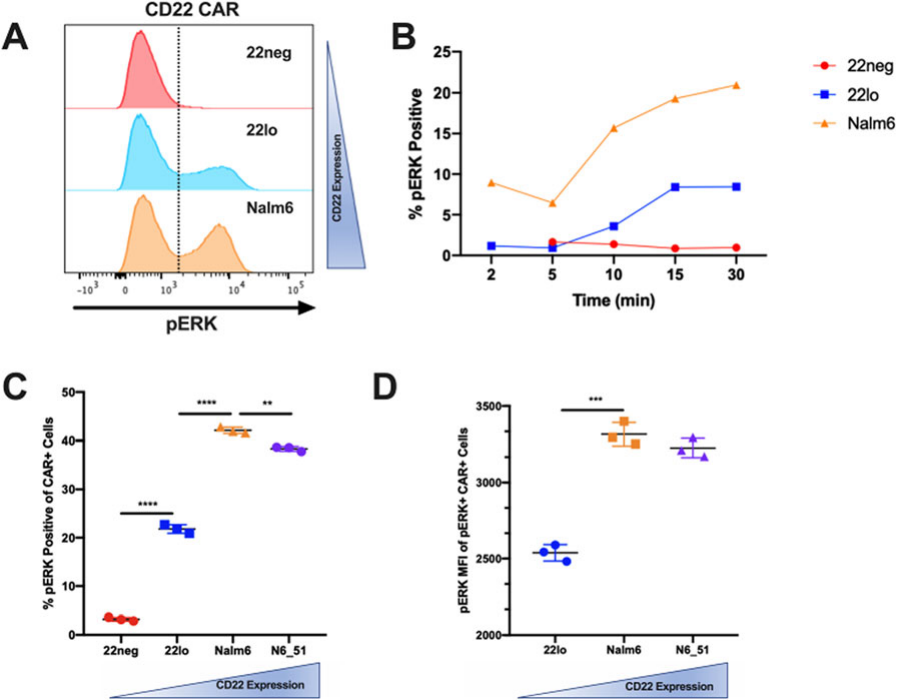
**(abstract 09).** See text for description

**Fig. 2:**
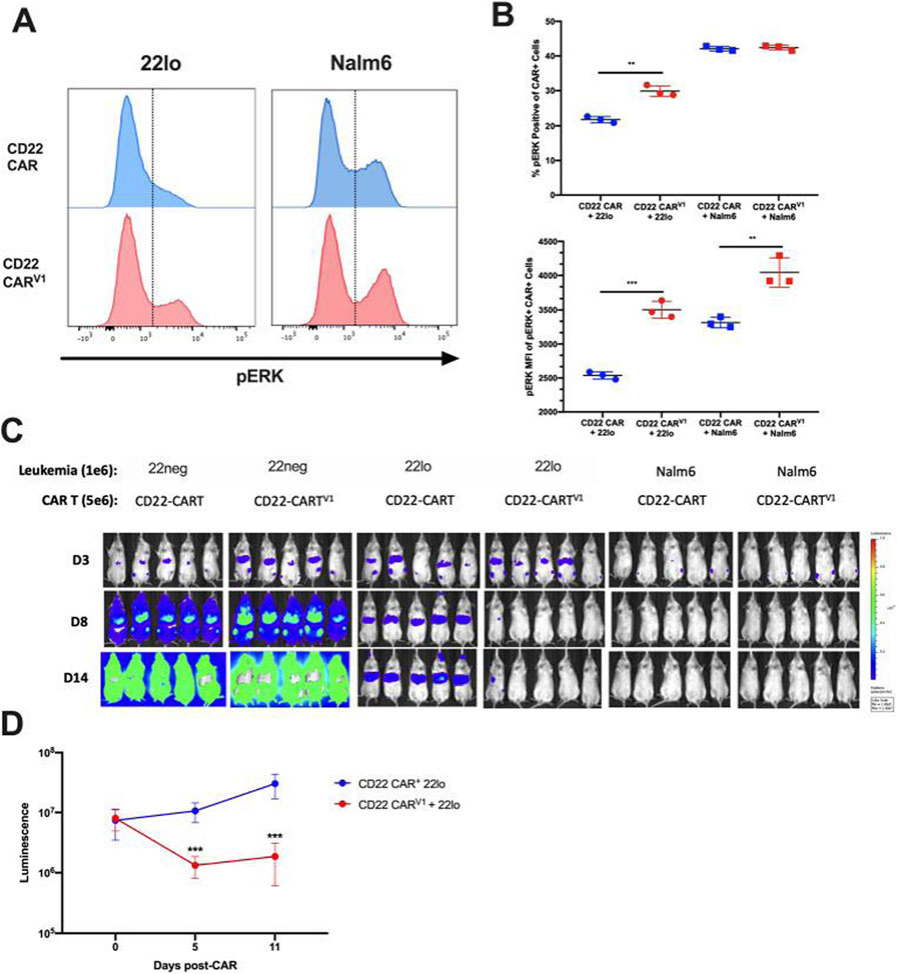
**(abstract 09).** See text for description

#### O10 Licensing of CAR-T cell persistence and function by STING agonist in breast cancer

##### Nuo Xu^1^, Jonathan Serody, MD^2^, Nicholas Restifo, MD^2^, Douglas Palmer^2^

###### ^1^University of North Carolina at Chapel Hill, Chapel Hill, NC, United States; ^2^National Cancer Institute, Bethesda, MD, United States

####### **Correspondence:** Jonathan Serody (jonathan_serody@med.unc.edu)

**Background**

Cancer therapy has been revolutionized by immunotherapy in recent research or clinic[1-4]. Two forms of immunotherapy have demonstrated significant clinical activity: antibodies targeting checkpoint proteins[5] and adoptive cellular therapy (ACT). Among ACT, chimeric antigen receptor (CAR) engineered T cell has gained great success targeting hemopoietic malignancy[6-8]. These receptors take advantage of scFv from antibody specific to antigen expressed on tumor surface and activate the T cells targeting tumor cells once interact with tumor cells[9-13].

Despite promising clinic outcome in hemopoietic tumor therapy, CAR-T cell therapeutic performance in solid tumor patients is less satisfactory[14,15]. The potential challenge includes the difficulty of CAR-T to migrate and persist in solid tumor where the immunosuppressive tumor microenvironment (TME) greatly restricts the function of CAR-Ts. One significant obstruct for the study of CAR-T cells for solid tumor treatment is the lack of reliable orthotopic tumor models that can be implanted orthotopically into immunocompetent animal and treated using CAR-Ts.

**Methods**

Here, we took the advantage of orthotopic NT2 mammary tumor in FVB-Neu mice, a model in which T cells develop central tolerance to Neu-expressing tumors, similar to human patients presenting with HER-2+ breast tumors[16]. Neu expressing tumors can be targeted by anti-Neu CAR-Ts we developed from antibody hybridoma, which is similar to the HER-2/neu-specific CAR T cells currently used in clinical trials.

**Results**

We demonstrated conventional method expanded CAR-T with IL-7 and IL-15 (7/15 CAR-Ts) failed to induce in vivo tumor growth control in established mammary tumors regardless of approaches to alter the TME. However, CAR transduced CD4 and CD8 T cells cultured under Th17 condition (Th/Tc17 CAR-Ts) has better persistence and expansion in vivo compared to 7/15 CAR-Ts. Furthermore, the enhanced cytolysis of Th/Tc17 CAR-Ts was licensed by locally STING agonist treatment, and lead to short term tumor growth control. Finally, the combination of TME modulation by myeloid-derived suppressive cells (MDSCs) depletion led to profound tumor regression in mice receiving Th/Tc17 CAR-Ts with STING administration, and the tumor resolution was accompanied with cytokine release like symptom that caused mortality in animals with the fastest tumor shrinkage.

**Conclusions**

In conclusion, we found STING agonist administration can enhance the therapeutic effect of Th/Tc17 CAR-T in vivo against breast tumor in immunocompetent host and further combinational of MDSC depletion can lead to tumor clearance with CRS like symptom. Our work provides significant new insight into approaches to improve CAR-T efficacy for solid tumor, which can be rapidly translated into clinic.

**References**

1 Humphries, C. Adoptive cell therapy: Honing that killer instinct. Nature 504, S13-15, doi:10.1038/504S13a (2013).

2 Rapoport, A. P. et al. Restoration of immunity in lymphopenic individuals with cancer by vaccination and adoptive T-cell transfer. Nature medicine 11, 1230-1237, doi:10.1038/nm1310 (2005).

3 Klebanoff, C. A., Rosenberg, S. A. & Restifo, N. P. Prospects for gene-engineered T cell immunotherapy for solid cancers. Nature medicine 22, 26-36, doi:10.1038/nm.4015 (2016).

4 Speiser, D. E. Hit parade for adoptive cell transfer therapy: the best T cells for superior clinical responses. Cancer Discov 3, 379-381, doi:10.1158/2159-8290.CD-13-0064 (2013).

5 Korman, A. J., Peggs, K. S. & Allison, J. P. Checkpoint blockade in cancer immunotherapy. Adv Immunol 90, 297-339, doi:10.1016/S0065-2776(06)90008-X (2006).

6 Neelapu, S. S. et al. Axicabtagene Ciloleucel CAR T-Cell Therapy in Refractory Large B-Cell Lymphoma. The New England journal of medicine 377, 2531-2544, doi:10.1056/NEJMoa1707447 (2017).

7 Maude, S. L. et al. Tisagenlecleucel in Children and Young Adults with B-Cell Lymphoblastic Leukemia. The New England journal of medicine 378, 439-448, doi:10.1056/NEJMoa1709866 (2018).

8 Brudno, J. N. et al. T Cells Genetically Modified to Express an Anti-B-Cell Maturation Antigen Chimeric Antigen Receptor Cause Remissions of Poor-Prognosis Relapsed Multiple Myeloma. Journal of clinical oncology : official journal of the American Society of Clinical Oncology 36, 2267-2280, doi:10.1200/JCO.2018.77.8084 (2018).

9 Gorochov, G., Lustgarten, J., Waks, T., Gross, G. & Eshhar, Z. Functional assembly of chimeric T-cell receptor chains. Int J Cancer Suppl 7, 53-57 (1992).

10 Gross, G. & Eshhar, Z. Endowing T cells with antibody specificity using chimeric T cell receptors. FASEB J 6, 3370-3378 (1992).

11 Finney, H. M., Lawson, A. D., Bebbington, C. R. & Weir, A. N. Chimeric receptors providing both primary and costimulatory signaling in T cells from a single gene product. J Immunol 161, 2791-2797 (1998).

12 Imai, C. et al. Chimeric receptors with 4-1BB signaling capacity provoke potent cytotoxicity against acute lymphoblastic leukemia. Leukemia 18, 676-684, doi:10.1038/sj.leu.2403302 (2004).

13 Carpenito, C. et al. Control of large, established tumor xenografts with genetically retargeted human T cells containing CD28 and CD137 domains. Proceedings of the National Academy of Sciences of the United States of America 106, 3360-3365, doi:10.1073/pnas.0813101106 (2009).

14 Beatty, G. L. et al. Activity of Mesothelin-Specific Chimeric Antigen Receptor T Cells Against Pancreatic Carcinoma Metastases in a Phase 1 Trial. Gastroenterology 155, 29-32, doi:10.1053/j.gastro.2018.03.029 (2018).

15 Tchou, J. et al. Safety and Efficacy of Intratumoral Injections of Chimeric Antigen Receptor (CAR) T Cells in Metastatic Breast Cancer. Cancer immunology research 5, 1152-1161, doi:10.1158/2326-6066.CIR-17-0189 (2017).

16. R.T. Reilly, M.B. Gottlieb, A.M. Ercolini, J.P. Machiels, C.E. Kane, F.I. Okoye, et al. HER-2/neu is a tumor rejection target in tolerized HER-2/neu transgenic mice, Cancer Res, 60 (August (13)) (2000), pp. 3569-3576

### Checkpoint Blockade Therapy

#### O11 Comparing anti-tumor and anti-self immunity in a patient with melanoma receiving immune checkpoint blockade

##### Shuming Chen, PhD, Abha Soni, Tracee McMiller, John-William Sidhom, MSE, Farah Succaria, MD, Alan Berger, Jody Hooper, Drew Pardoll, MD, PhD, Evan Lipson, Janis Taube, MD, MSC, Suzanne Topalian, MD

###### Johns Hopkins University School of Medicine, Baltimore, MD, United States

####### **Correspondence:** Shuming Chen (schen72@jhmi.edu)

**Background**

Immune checkpoint blockade has become a pillar of oncology for its effectiveness in treating some patients with advanced cancers. However, ≤90% of patients develop minor to severe immune-related adverse events (irAEs), marked by normal tissue inflammation and significantly associated with a higher tumor regression rate [1]. Glucocorticoids often administered to manage irAEs may blunt anti-tumor immunity. This study was undertaken to investigate the functional relationship between anti-tumor and anti-self immunity that could uncover more refined approaches to both irAE management and tumor control.

**Methods**

Under the Johns Hopkins Legacy Gift Rapid Autopsy Program, multiple biopsy samples were collected over 6 years from diagnosis to death in a melanoma patient experiencing both tumor regression and irAEs after receiving anti-PD-1 and anti-CTLA-4. Fourteen specimens including tumor, inflamed and non-inflamed normal tissues were collected. Laser-capture microdissected (LCM) anti-tumor and anti-self inflammatory infiltrates from eight FFPE samples were subjected to gene expression profiling (GEP) by multiplex qRT-PCR for 123 immune-related genes; infiltrates from six of these samples underwent TCRVB sequencing and deep learning analysis. Expression of 14 immune cell subset and regulatory molecules was assessed in 12 samples by immunohistochemistry (IHC).

**Results**

GEP revealed genes abundantly expressed across all samples, including those involved in MHC and antigen presentation (B2M, CD74, HLA-DRA), IFN signaling (STAT1, CXCL9), innate immunity (LYZ, DDX58/RIG-I), and immunosuppression (TGFB1, TIGIT, LGALS9/galectin-9, HAVCR2/Tim-3). Some genes were poorly expressed across all samples, including those related to lineage differentiation or function of neutrophils (CEACAM8/CD66b, CSF2/GM-CSF), Th2 cells (IL4, IL13), and Th17 cells (IL17, IL22, IL22RA2, IL23A). Inflammatory infiltrates in normal tissues significantly over-expressed genes associated with regulatory T cells (FOXP3, CD4, TGFB1), immunosuppression (SOCS1, CSF1R, IL27&EBI3, KLRG1), and the COX2 pathway (IL1B, PTGER1/EP1, PTGER4/EP4). Focal LCM&GEP was more informative than quantitative whole slide IHC analysis. TCRseq revealed relatedness among samples from normal tissue inflammation, however dominant clones were not shared with tumor infiltrates.

**Conclusions**

Rapid autopsy in an individual with melanoma allowed a unique opportunity to study the effects of immune checkpoint blockade on tumor regression and normal tissue inflammation in multiple organs. We found enhanced immunosuppression in inflamed normal tissues, reflecting intrinsic homeostatic mechanisms. TCRseq indicated that anti-tumor and anti-self immunity may be parallel processes caused by distinct T cell clones. Individual genes upregulated in irAEs, such as IL1B, EP1 and EP4 (prostaglandin receptors), are targetable with existing drugs and may present new opportunities to specifically suppress irAEs while promoting anti-tumor immunity.

**Acknowledgements**

Supported by the Johns Hopkins Bloomberg~Kimmel Institute for Cancer Immunotherapy, NCI R01CA142779, Melanoma Research Alliance, Bristol-Myers Squibb, Moving for Melanoma of Delaware, Laverna Hahn Charitable Trust, and the Barney Foundation.

**References**

1. Topalian SL, Hodi FS, Brahmer JR, et al. Five-year survival and correlates among patients with advanced melanoma, renal cell carcinoma, or non-small cell lung cancer treated with nivolumab. JAMA Oncol. 2019; doi:10.1001/jamaoncol.2019.2187.

**Ethics Approval**

The study was approved by the Johns Hopkins Institutional Review Board, approval number NA_00036610.

#### O12 B-cells activated by checkpoint blockade immunotherapy and radiation improve overall survival in squamous cell carcinomas

##### Sangwoo Kim, BA, Sarek Shen, Sayuri Miyauchi, Philip Sanders, Loren Mell, MD, J. Silvio Gutkind, Ezra Cohen, MD, Joseph Califano, Andrew Sharabi, MD, PhD

###### University of California, San Diego, CA, United States

####### **Correspondence:** Andrew Sharabi (sharabi@ucsd.edu)

**Background**

Many studies examining tumor immunology and the effects of radiation therapy (RT) and PD-1 checkpoint blockade immunotherapy (CBI) have focused on T-cells or myeloid cells. However, B-cells are a critical arm of the adaptive immune system and generate antibodies targeting tumors for eradication. Nevertheless, the role of B-cells in response to RT or CBI and resultant patient outcomes remains largely unknown. Here we studied B-cells in HPV-associated squamous cell carcinomas and identified dramatic changes induced by RT and CBI, and novel B-cell markers associated with improved overall survival.

**Methods**

For human studies we analyzed two independent databases of tumor RNA-seq from over 800 head and neck (HNSCC) and cervical cancer patients, including a prospective validation cohort. We performed proteomics array on samples from patients treated with CBI and RT to analyze antigen specific B-cell responses. For murine studies syngeneic head and neck models were used including MEER and AT-84. C3H and C57B/6 mice with palpable flank tumors were treated with combinations of RT and CBI. B-cell surface markers in the spleen, lymph nodes, and tumor were analyzed by flow cytometry. BCR sequencing was performed and analyzed using ImmunoSeq platform. Single-cell RNA sequencing (scRNA seq) was performed using the 10X Genomics platform and analyzed using Seurat v3.

**Results**

RT and CBI induced dramatic changes in systemic B-cell activation, T1 and T2- type B-cells, memory cells, plasma cells, and antigen specificity. B-cell depletion significantly increased tumor growth using HNSCC models. BCR sequencing identified that CBI and RT increase the maximum productive frequency and clonality, and modified CDR3 length. scRNA seq of sorted B-cells identified striking increases in B-cell germinal center development after CBI and RT. Proteomics analysis revealed remarkable increases in IgG antibody levels in patients with objective responses after CBI and RT. Finally, analysis of tumor RNA sequencing data from two independent databases of over 800 patients identified CD19 and IGJ as novel B-cell specific prognostic biomarkers for 3-year overall survival (OS; 3-year OS hazard ratio = 0.545, P < 0.001).

**Conclusions**

RT and CBI activate multiple B-cell subpopulations, modulate B-cell germinal center formation and maturation, and enhance B-cell mediated antigen-specific immune responses. Furthermore, we discovered CD19 and IGJ as novel B-cell prognostic biomarkers for 3-year overall survival in patients with HPV-associated squamous cell carcinomas. These findings establish a key role for B-cells in HPV-associated tumor biology and reveal a novel B-cell mediated immune mechanism by which PD-1 blockade may improve patient outcomes.

**Ethics Approval**

Patients in the JHU cohort were recruited under protocol NA_00-36235, which was approved by the JHU IRB. Patients in the University of California, San Diego (UCSD) cohort were recruited under IRB approved protocol UCSD HRPP 151570 (ClinicalTrials.gov Identifier: NCT02843165).

#### O13 Overcoming resistance to anti-PD1 therapy by targeting Bone Morphogenetic Proteins

##### Maria Cortez, PhD^1^, Fatemeh Maspourour^1^, Cristina Ivan, PhD^1^, Jie Zhang^1^, Ahmed Younes^1^, Yue Lu^1^, Marcos Estecio^1^, Hampartsoum Barsoumian^1^, Hari Menon^1^, Mauricio da Silva Caetano^1^, Rishab Ramapriyan^1^, Jonathan Schoenhals^2^, Xiaohong Wang^1^, Ferdinandos Skoulidis^1^, George Calin^1^, Patrick Hwu, MD^1^, James Welsh^1^

###### ^*1*^*The University of Texas MD Anderson Cancer, Houston, TX, United States* ; ^*2*^*UT Southwestern, Dallas, TX, United States*

####### **Correspondence:** Maria Cortez (macortez@mdanderson.org)

**Background**

Although anti-PD1 antibodies have produced durable control in some patients, most do not respond to this therapy and even those who do often develop resistance. The mechanisms underlying immunosuppression and resistance to PD1 inhibitors in lung cancer are not well understood, raising fundamental questions about the drivers of this resistance and how to enhance antitumor immune function to overcome treatment resistance. Here, we report that overexpression of bone morphogenetic protein (BMP)-7, a member of the TGF-beta superfamily, represents a new mechanism for resistance to anti-PD1 therapy in preclinical models and in patients with disease progression on immunotherapies.

**Methods**

Paraffin-embedded tissues from mouse and patients treated with pembrolizumab or ipilimumab were examined via IHC. BMP7 and cytokines/chemokines serum levels were analyzed by Elisa. Macrophages and CD4+T cells were isolated using magnetic beads and used in co-culture experiments. Anti-PD1-resistant 344SQ murine lung cancer cells (p53R172HΔg/+K-rasLA1/+) and parental cells were inoculated into syngeneic 129Sv/Ev mice, which were treated with anti-PD1, follistatin, anti-CTLA-4 or anti-PDL1. Tumor tissues were analyzed as follows: methylation status by reduced representation bisulfite sequencing and pyrosequencing methylation assay; protein levels by reverse-phase protein array. Tumor-infiltrating lymphocytes were isolated for flow cytometry and gene expression analyses using Nanostring. Prism 8.0 software and R were used for statistical analyses.

**Results**

BMP7 was upregulated in mouse and human tumors resistant to immunotherapies, and BMP7 levels were higher not only in blood from mice bearing resistant tumors but also in pretreatment blood from patients with disease progression on anti-PD1. BMP7 specifically regulated p38α expression via SMAD1 activation in mouse and in cancer patients with progression on immunotherapies. BMP7 is secreted by tumor cells and acts on immune cells, promoting downregulation of p38α and p38α-regulated cytokines and chemokines IL-1alpha, IL-1beta, TNF-alpha, RANTES, IFN-gamma, and IL-2. We found increased numbers of CD4+ and CD8+ IFN-gamma+ T cells and decreased percentages of M2 macrophages in BMP7-knockdown tumors treated with anti-PD1 compared with control. Knockdown of BMP7 or its neutralization via follistatin in combination with anti-PD1, anti-CTLA4 or anti-PDL1 extended survival and re-sensitizes resistant tumors to immunotherapies.

**Conclusions**

Our studies revealed that BMP7 impinged effector T cell functions while favoring the generation of immunosuppressive cells precluding response to immunotherapy. Knockdown of BMP7 or its neutralization via follistatin in combination with anti-PD1 re-sensitizes resistant tumors to immunotherapies. Collectively, our results support that targeting BMP7 may represent a new approach to overcome resistance to checkpoint blockade in cancer.

**Ethics Approval**

All analyses were approved by the UT MD Anderson Cancer Center institutional review board (protocols 2014-1020 and 2013-0882). All mouse studies were approved by the Institutional Animal Care and Use Committee (IACUC) of The University of Texas MD Anderson Cancer Center before their initiation; animal care was provided according to IACUC standards, and all mice had been bred and were maintained in our own specific pathogen-free mouse colony.

#### O14 Immune selection for IFN-g signaling mutant cancer cells involves tumor heterogeneity and clonal cooperation

##### Thomas Gajewski, MD, PhD^1^ , Shuyin Li, PhD^1^, , Jason Williams^1^, Emily Higgs^1^, Alexandra Cabanov^1^, Xiaozhong Wang^2^, Haochu Huang^1^

###### ^*1*^*University of Chicago, Chicago, IL, United States* ; ^*2*^*Northwestern University, Chicago, IL, United States*

####### **Correspondence:** Thomas Gajewski (tgajewsk@medicine.bsd.uchicago.edu), Haochu Huang (haochu.huang@gmail.com)

**Background**

Immune checkpoint blockade therapies have transformed the landscape of cancer care. Durable clinical responses have been observed in a subset of patients with historically difficult-to-treat cancer types. Despite these successes, many patients do not respond, indicating a primary resistance. Additionally, a significant proportion of patients develop progressive/recurrent disease after an initial clinical benefit, known as secondary resistance, a setting in which tumors may arise under a strong immune selection pressure. Further work is needed to elucidate and target secondary mechanisms of resistance, which may in turn improve treatment outcomes for patients.

**Methods**

To identify genes critical to immunotherapy resistance, B16.SIY cells were transduced with a genome-scale gRNA lentivirus to generate loss of function mutants. In vitro-primed CD8+ T cells isolated from 2C/Rag2–/– TCR transgenic mice specific for the SIY antigen were co-cultured with transduced B16.SIY tumor cells. Resistant mutants were identified by sequencing the gRNAs of survival clones. Ifngr2 and Jak1 mutants were identified from the screen. Based on these results, we generated Ifngr2 and Jak1 deficient mutants using CRISPR-Cas9 gene editing. To investigate the role of genes in tumor growth and immune responses in vivo, the mutants were transplanted into B6 mice and the mice were subsequently treated with anti-PDL1 antibody. The tumor microenvironments were analyzed by flow cytometry.

**Results**

The genome-wide CRISPR screen identified Ifngr2 and Jak1 mutants resistant to CD8+ T cell killing in vitro. However, when implanted into mice, these mutant tumors paradoxically were better controlled immunologically and the anti-tumor CD8+ T cell response was augmented. This phenotype mapped to defective PD-L1 upregulation on mutant tumor cells which improved the anti-tumor efficacy of CD8+ T cells. To reconcile these observations with clinical reports of anti-PD-1 resistance linked to emergence of IFN-γ signaling mutants, wild-type and mutant tumor cells were mixed prior to implantation and anti-PD-L1 treatment, to recapitulate tumor cell heterogeneity. Under these conditions, IFN-γ signaling mutant tumor cells indeed grew out.

**Conclusions**

Our results illustrate the complexity of functions for IFN-γ in anti-tumor immunity and demonstrate that intra-tumor heterogeneity and clonal cooperation can contribute to patterns of immunotherapy resistance.

#### O15 Replication stress response defects predict response to immune checkpoint blockade across multiple cancer lineages

##### Daniel McGrail, PhD, Patrick Pilie, MD, Truong Nguyen Anh Lam, Hui Dai, MD, Eric Jonasch, MD, Shiaw-Yih Lin, PhD

###### MD Anderson Cancer Center, Houston, TX, United States

####### **Correspondence:** Shiaw-Yih Lin (sylin@mdanderson.org)

**Background**

Immune checkpoint blockade (ICB) has provided robust, durable responses for a subset of patients. Initial studies focused on high-mutation burden, or “mutator,” cancers identified potential protein, mutational, and gene expression biomarkers for patient response. While emerging clinical data indicates ICB may also contribute to treatment of “non-mutator” cancers, such as renal cell carcinoma and glioblastoma, we and others have found established clinical biomarkers exhibit minimal activity at predicting response in non-mutator patient cohorts.

**Methods**

To analyze predictive biomarkers for ICB response, we gathered 11 non-mutator patient cohorts across 5 different tumor lineages treated with ICB, along with molecular profiling data including gene expression profiling, PD-L1 immunohistochemistry, tumor mutation burden (TMB), and relevant clinical information. Mechanistic studies using flow cytometry, microscopy, and quantitative cellular fractionation were performed using human and mouse cell lines. Pre-clinical mouse models of ICB treatment were used to validate ICB response mechanisms and analyze immune populations with multispectral imaging.

**Results**

We demonstrate accurate prediction of ICB response in all 11 non-mutator patient cohorts spanning 5 tumor lineages using a gene expression signature indicative of replication stress response (RSR) defects. Across these cohorts the average area under the receiver-operator characteristic (ROC) curve was 0.82 (range 0.72-1.00). No other evaluated biomarker, including mutation/neoantigen burden, PD-L1 immunostaining, and the T cell inflamed gene expression signature, was able to predict outcomes across these cohorts. Pre-clinical studies show that RSR defect-high tumor cells undergo RPA exhaustion downstream of aberrant origin firing, leading to accumulation of immunostimulatory cytoplasmic DNA. Consistent with this, pharmacological induction of RSR defects induced accumulation of cytoplasmic DNA and sensitivity to ICB, whereas suppression of origin firing reduced cytoplasmic DNA causing ICB resistance. Both multispectral immunostaining of preclinical models and in silico analysis of patient cohorts failed to uncover any conserved relationship between infiltrating immune populations and response to ICB.

**Conclusions**

The RSR defect gene signature can accurately identify patients who will benefit from ICB across numerous non-mutator tumor lineages, and the benefits of ICB may be further expanded by pharmacological induction of the RSR defect phenotype.

**Ethics Approval**

All samples used were collected in accordance with their respective institutes' Ethics Boards. All animal studies were approved by IACUC at The University of Texas MD Anderson Cancer Center.

### Clinical Trial Completed

#### O16 Avelumab and cetuximab in combination with FOLFOX in patients with previously untreated metastatic colorectal cancer (MCRC) – Final Results of the phase II AVETUX trial (AIO-KRK-0216)

##### Mascha Binder^2^, Eray Goekkurt^3^, Sylvie Lorenzen^4^, Jorge Riera-Knorrenschild^5^, Reinhard Depenbusch^6^, Thomas Ettrich^7^, Steffen Doerfel^8^, Salah Al-Batran^9^, Meinolf Karthaus^10^, Uwe Pelzer^11^, Donjete Simnica^2^, Lisa Waberer^9^, Axel Hinke^12^, Carsten Bokemeyer^13^, Susanna Hegewisch-Becker^14^, Joseph Tintelnot, MD^13^

###### ^*1*^*University Cancer Center Hamburg, Hamburg, Germany*; ^*2*^*University Hospital Halle, Halle, Germany* ; ^*3*^*Hematology-Oncology Practice Hamburg (HO, Hamburg, Germany* ; ^*4*^*Rechts der Isar Hospital, Technical Univ, Munich, Germany* ; ^*5*^*University Clinics Giessen-Marburg, Marbug, Germany* ; ^*6*^*Private Practice Onkodoc GmbH Gütersloh, Guetersloh, Germany* ; ^*7*^*Center for Internal Medicine I, Universi, Ulm, Germany* ; ^*8*^*Private Practice Onkozentrum Dresden, Dresden, Germany* ; ^*9*^*Institute of Clinical Cancer Research, K, Frankfurt, Germany* ; ^*10*^*Neuperlach Clinic, Neuperlach, Germany* ; ^*11*^*Charite, Berlin, Germany* ; ^*12*^*CCRC, Duesseldorf, Germany* ; ^*13*^*University Medical Center Hamburg-Eppend, Hamburg, Germany* ; ^*14*^*Hematology-Oncology Practice Hamburg, Hamburg, Germany*

####### **Correspondence:** Eray Goekkurt (goekkurt@hope-hamburg.de)

**Background**

Single agent PD-1/L1 inhibition is efficacious in MCRC patients (pts) with high microsatellite instability (MSI-H). For the vast majority of MCRC pts with MS stable (MSS) phenotype the role of immunotherapy still needs to be determined. Based on the induction of immunogenicity by chemotherapy, particular oxalipatin and immunogenic cell death by cetuximab in combination with chemotherapy the standard 1st line regimen FOLFOX and cetuximab in combination with avelumab was evaluated.

**Methods**

The single arm phase II AVETUX trial combined mFOLFOX6 and cetuximab with avelumab (10mg/kg day 1 from cycle 2 onwards) in RAS/BRAF wildtype (local lab) MCRC pts. Primary endpoint was 12 month progression-free survival rate. Secondary endpoints included overall response rate (ORR), tolerability, overall survival and translational research evaluating tissue including PD-L1 expression (tumour/immune cells) and serial ctDNA. Efficacy analyses were done by intention to treat (ITT).

**Results**

Overall 43 pts were enrolled. Median age was 61 (range 29-82), 14 pts (33%) were female and 39 (91%) left sided. 30 pts (70%) had liver mets and 17 (40%) liver mets only. 2 pts were MSI-H, one MSI-low and 40 MSS. Besides immediate and otherwise unexplained fever in 4 pts treatment was well tolerated and avelumab was not associated with unexpected adverse events to standard FOLFOX/cetuximab. Central tissue review found 4 pts to be ineligible due to low frequent KRAS or BRAF mutation (15-31%). Thus, ITT included 39 pts. The ORR was 79.5%, including 6 complete (CR) and 25 partial responses (PR). Further 5 stable diseases were noted, thus disease control rate was 92.3%; 2 pts had progression and 1 was not evaluable. Early tumor shrinkage (ETS) rate (≥20% after 8 weeks) was 79.5% (1 CR, 27 PR and 3 SD with ≥20% -

**Conclusions**

The AVETUX regimen was feasible producing a high rate of responses in MSS pts mainly occurring within the first 8 weeks. The noted ORR/ETS of 79.5% warrants further evaluation in a randomized trial.

**Trial Registration**

NCT03174405

**Ethics Approval**

The study protocol was approved by the local ethics committees (PVN 5447), and was also subject to authorization by the competent authority.

**Consent**

Written informed consent was obtained from the patient for publication of this abstract. A copy of the written consent is available for review by the Editor of this journal.

#### O17 A novel enantio-specific cationic lipid R-DOTAP + HPV16 E6 & E7 antigens induces potent antigen-specific CD8+ T cell responses in-vivo in subjects with CIN and high-risk human papillomavirus infection

##### Lauren Wood, MD^1^, Lance Edwards, MD^2^, Daron Ferris, MD^3^, Nicole Nevadunsky, MD^4^, Sara Isani, MD^4^, Greg Conn, PhD^1^, Frank Bedu-Addo, PhD^1^, Mark Einstein, MD^5^

###### ^*1*^*PDS Biotechnology, Berkeley Heights, NJ, United States* ; ^*2*^*Suffolk Obstetrics and Gynecology, Port Jefferson, NY, United States* ; ^*3*^*Augusta University, Augusta, GA, United States* ; ^*4*^*Montefiore Medical Center, Bronx, NY, United States* ; ^*5*^*Rutgers-NJMS, Newark, NJ, United States*

####### **Correspondence:** Lauren Wood (lwood@pdsbiotech.com)

**Background**

Persistent human papillomavirus (HPV) 16 DNA infection is associated with up to 60% of cervical cancers as well the high grade cervical intraepithelial neoplasia (CIN) that precedes them. Current treatment strategies focus on excision of these virally mediated precancerous lesions and cancers, which lead to preterm birth and other issues highlighting an unmet clinical need for medical therapeutics. Therapeutic vaccines to date have been limited in their ability to generate high levels of HPV16-specific CD8 cytolytic T cells (CTL) in vivo. Here we describe a phase I dose escalation study of PDS0101, a novel multi-functional HPV16-specific immunotherapy which acts via antigen cross presentation and induction of type I interferons [1] in women with high-risk (hr) HPV infection and CIN. (NCT02065973)

**Methods**

Twelve females (median age 33 years, range 24-51), with confirmed CIN1 and hrHPV DNA infection received PDS0101 administered subcutaneously at doses of 1.0, 3.0 and 10.0 mg of R-DOTAP with a fixed 2.4 mg dose of HPV16 peptide mix. Vaccines were administered at Days 1, 22 and 43, with immune monitoring pre-treatment, 14 days after each vaccination and at Day 133. HPV16-specific CD8+ T-cell induction was assessed by Granzyme B ELISPOT. All subjects were monitored for safety and tolerability of vaccination.

**Results**

Four of 12 subjects (33%) had HPV16, with 8 subjects (67%) having two or more HPV genotypes. A total of 15 HPV genotypes were documented (mean 2.5 genotypes/subject). Two subjects had high pre-existing HPV responses. The remaining 10 subjects had low HPV16-specific T-cell responses pre-vaccination: 100% (10/10) developed vaccine-induced HPV16-specific T-cell responses on day 14: 9 of 10 (90%) had IFN-γ responses while 6 of 9 (67%) had granzyme B responses. Comparable CD8 T cell reactivity was observed in the 3.0 and 10.mg R-DOTAP cohorts as assessed by mean fold-increase over baseline as shown in Table 1. All doses were well tolerated with the most common toxicity of self-limiting local injection site reactions that resolved within several days.

**Conclusions**

PDS0101 is associated with induction of potent HPV16-specific CD8 T cells with a granzyme B cytolytic profile and is safe and well tolerated. The 3.0 mg R-DOTAP dose has been selected for further studies in subjects with high grade CIN2/3 and HPV16DNA infection as well as HPV16-related head and neck cancer.

**Trial Registration**

NCT02065973

**References**

1. Gandhapudi SK, Ward M, Bush JP et al. Antigen Priming with Enantiospecific Cationic Lipid Nanoparticles Induces Potent Antitumor CTL Responses through Novel Induction of a Type I IFN Response. J Immunol 2019;202:3524-3536.

**Ethics Approval**

The investigators from each respective institution obtained approval of the study from a properly constituted institutional review board (!RB) prior to study initiation. These included Schulman IRB (now Advarra IR), BRANY IRB (#IRB00000080, #IRB00010793) and Georgia Regents University IRB (now Augusta University IRB).

**Table 1 (abstract 017).**
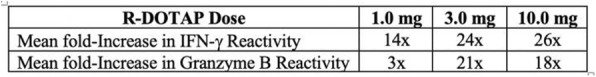
See text for description

#### O18 Neoantigen vaccination targeting shared epidermal growth factor receptor (EGFR) mutations induces clinical and immunological responses in non-small cell lung cancer patients

##### Gregory Lizee, PhD^1^, Fenge Li^1^, Ligang Deng^2^, Amjad Talukder^1^, Kyle Jackson^1^, Arjun Katailiha^1^, Sherille Bradley^1^, Heather Sonnemann^1^, Qingwei Zhou^2^, Caixia Chen^2^, Chong Huo^2^, Yulun Chiu^1^, Matthew Stair^3^, Weihong Feng^4^, Aleksander Bagaev^5^, Nikita Kotlov^5^, Viktor Svekolkin^5^, Ravshan Ataullakhanov^5^, Natalia Miheecheva^5^, Felix Frenkel^5^, Yaling Wang^2^, Minying Zhang^1^, Jason Roszik, PhD^1^, Ling Han^6^, Shuo Zhou^7^, Yan Zhang^8^, Zhenglu Wang^9^, Marie-Andree Forget^1^, Chantale Bernatchez^1^, Cassian Yee, MD^1^, Patrick Hwu, MD^1^, Xueming Du^4^, Gregory Lizee, PhD^1^

###### ^*1*^*MD Anderson Cancer Center, Houston, TX, United States* ; ^*2*^*Tianjin HengJia Biotech. Co., Ltd., Tianjin, China* ; ^*3*^*Our Lady Medical Center, Baton Rouge, United States* ; ^*4*^*Tianjin Beichen Hospital, Tianjin, China* ; ^*5*^*BostonGene Corporation, Boston, United States* ; ^*6*^*Tianjin Beichen Hosipital, Tianjin, United States* ; ^*7*^*Fujian Medical University, Shanghai, China* ; ^*8*^*Nankai University, Tianjin, China* ; ^*9*^*Tianjin First Central Hospital, Tianjin, China*

####### **Correspondence:** Gregory Lizee (glizee@mdanderson.org)

**Background**

Neoantigen (NeoAg) peptides derived from protein-coding somatic mutations displayed at the tumor cell surface by human leukocyte antigen (HLA) molecules demonstrate exquisite tumor specificity and can elicit tumor-specific T cell-mediated immunity to facilitate tumor rejection [1]. NeoAg vaccines have shown great promise for inducing such antitumor T-cell responses in multiple cancer types [2-3]. Based on our previous case study of a non-small cell lung cancer (NSCLC) patient who experienced regression of multiple lung tumors following NeoAg vaccination [4], we initiated a phase Ib clinical trial to immunize 24 additional advanced-stage NSCLC patients, with primary endpoints being to determine safety and feasibility, and secondary endpoints to assess patient survival in addition to the immunogenicity of NeoAg vaccine peptides.

**Methods**

Personalized peptide vaccines were designed based on mutational profiling of a panel of 508 cancer-associated genes. Vaccine peptides were chosen primarily based on the highest predicted binding affinity of mutation-encoding NeoAgs to each patient’s individual HLA class I and class II allotypes. Each patient was immunized subcutaneously with a unique mixture of short and long NeoAg peptides dissolved in saline along with topically-applied Imiqimod to provide co-stimulation. The 24 patients each received a minimum of 12 weekly immunizations followed by clinical response evaluation by CT scans taken 3 to 4 months following the initiation of vaccination. Patients with EGFR mutations (n=16) were given the option of continuing EGFR inhibitor (EGFRi) therapy concurrent with NeoAg vaccination, despite previously experiencing disease progression on EGFRi.

**Results**

Following NeoAg vaccination, 7 RECIST-based objective clinical responses were observed, with all responders being amongst the 16 patients harboring EGFR mutations. Immune monitoring demonstrated robust EGFR NeoAg-specific T cell responses in 5 responding patients, with 3 patients generating dominant CD8+ T-cell responses against a single NeoAg peptide encompassing the widely shared L858R driver mutation. Importantly, patients that continued receiving EGFRi therapy showed significantly extended overall survival compared to patients who stopped EGFRi prior to immunization (13.8 vs. 7.6 months, P=0.038) . Molecular and cellular analyses showed that EGFRi impacted multiple immunomodulatory pathways in EGFR-mutated lung tumor cells to favor immune cell infiltration and HLA-mediated antigen presentation, providing a potential mechanism of synergy with NeoAg vaccination.

**Conclusions**

These findings identify shared EGFR mutations as immunogenic NeoAgs with therapeutic potential, and suggest that EGFRi in combination with NeoAg vaccination may facilitate improved clinical outcomes for patients with NSCLC, even in the setting of prior EGFRi failure.

**Trial Registration**

This clinical trial was approved by the internationally-recognized Chinese Clinical Trials Registry (ChiCTR) (Trial#: ChiCTR-IIR-16009867, “Phase I clinical research of personalized neoantigen peptide vaccine for non-small cell lung cancer". Weblink: http://www.chictr.org.cn/showproj.aspx?proj=16736)

**References**

1. Schumacher, T. N. & Schreiber, R. D. Neoantigens in cancer immunotherapy. Science. 348, 69-74, 487 (2015).

2. Ott PA, Hu Z, Keskin DB, et al. An immunogenic personal neoantigen vaccine for patients with melanoma. Nature. 517 13, 217-221 (2017).

3. Hilf N, Kuttruff-Coqui S, Frenzel K, et al. Actively personalized vaccination trial for newly diagnosed glioblastoma. Nature. 521 565, 240-245 (2019).

4. Li F, Chen C, Ju T, et al. Rapid tumor regression in an Asian lung cancer patient following personalized neo-epitope peptide vaccination. Oncoimmunology. 5, e1238539 (2016).

**Ethics Approval**

This study has been performed in accordance with the Declaration of Helsinki and was approved by the Ethics Committee of Tianjin Beichen Hospital, Tianjin, China.

#### O19 Final results from a phase 2 study using off-the-shelf activated natural killer (aNK) cells in combination with N-803, an IL-15 superagonist, in patients with metastatic Merkel cell carcinoma (MCC)

##### Shailender Bhatia, MD^1^, Candice Church, PhD^2^, Kelly Paulson, MD, PhD^1^, Robert Pierce, MD^3^, Paul Nghiem, MD, PhD^1^, John Lee, MD^4^, Bridget Adcock, BS^5^, Patrick Soon-Shiong, MD^4^, Sunandana Chandra, MD^6^

###### ^*1*^*University of Washington; Fred Hutchinson Cancer Research Center, Seattle, WA, United States* ; ^*2*^*University of Washington, Seattle, WA, United States* ; ^*3*^*Fred Hutchinson Cancer Research Center, San Diego, CA, United States* ; ^*4*^*ImmunityBio, Inc, Culver City, CA, United States;*^*5*^*ImmunityBio, Inc., Culver City, CA, United States* ; ^*6*^*Northwestern University Feinberg School of Medicine, Chicago, IL, United States*

####### **Correspondence:**Shailender Bhatia (sbhatia@uw.edu), Sunandana Chandra (sunandana.chandra@northwestern.edu)

**Background**

Metastatic MCC, a virus-associated cancer, is associated with high objective response rates (ORRs) to PD-1/PD-L1 blockade. Unfortunately, ~50% of patients are refractory due to MHC-I downregulation and other mechanisms of immune evasion. Allogeneic aNK cells are cytotoxic to tumor cells with deficient MHC-I, and N-803 increases the number and cytotoxicity of NK and memory CD8+ T cells. aNK plus N-803 therapy could reverse refractoriness to immune checkpoint inhibitors (ICI) in MCC (and other immunogenic cancers).

**Methods**

In this phase 2 trial, aNK monotherapy (2×10^9 cells/m^2, expanded on-site) was administered intravenously on days 1 and 2 of each 2-week cycle; a subsequent protocol amendment added subcutaneous N-803 (10 μg/kg) on day 1 prior to aNK infusion. Key eligibility criteria included unresectable stage III or IV MCC, and ≤ 2 prior systemic therapies. Endpoints included ORR by RECIST 1.1, progression-free survival, safety, and immunologic changes in the tumor microenvironment.

**Results**

Seven subjects were enrolled; 3 received aNK monotherapy, and 4 received aNK plus N-803. Two of 7 subjects had objective responses (ORR=29%; one partial response [PR] and one unconfirmed complete response [CR]); one patient had stable disease lasting 5.5 months. Treatment was safe, without immune-related adverse events (AEs), treatment-related AEs of grade ≥ 3, or serious AEs. Intriguing changes were noted clinically in superficial tumors in several patients within hours of aNK infusion. One heavily pretreated subject, who was refractory to pembrolizumab despite high PD-L1 expression, experienced an impressive clinical and radiologic CR at ~4 months with aNK monotherapy before experiencing PD at 6 months, resulting in treatment discontinuation. Rechallenging with pembrolizumab after aNK therapy resulted in a durable CR ongoing at 36 months. Serial tumor biopsies after aNK treatment revealed increased CD8+ and CD4+ T-cell infiltration, and increased expression of markers for immune cells, antigen presentation genes, and interferon gamma genes. Another subject treated with combination therapy had initial pseudoprogression, resulting in treatment discontinuation, but subsequently had an impressive PR (>85% stable regression, ongoing at 22 months) without additional treatment.

**Conclusions**

aNK (with/without N-803) therapy was well tolerated, and led to clinical benefit in 3 of 7 patients, including reversal of ICI-refractoriness in one patient. This trial provides proof of concept for aNK-based therapy in MCC and supports an upcoming registrational trial (NCT03853317) using cryopreserved NK cells (not requiring on-site expansion) plus N-803 plus avelumab in patients with advanced MCC refractory to treatment with ICIs.

**Trial Registration**

NCT02465957

**Ethics Approval**

This study was approved by Western IRB; approval number 1156905.

#### O20 A pilot study of engineered adenovirus ONCOS-102 in combination with pembrolizumab in checkpoint inhibitor refractory advanced or unresectable melanoma

##### Alexander Shoushtari, MD^1^, Anthony Olszanski, MD, RPh^2^, Thomas Hornyak, MD^3^, Jedd Wolchok, MD, PhD^1^, Sylvia Vetrhus^4^, Karianne Risberg Handeland^4^, Lukasz Kuryk, PhD^4^, Magnus Jaderberg, MD^4^

###### ^*1*^*Memorial Sloan Kettering Cancer Center, New York, NY, United States* ; ^*2*^*Fox Chase Cancer Center, Phildelphia, PA, United States* ; ^*3*^*University of Maryland, Baltimore, MD, United States* ; ^*4*^*Targovax ASA, Oslo, Norway*

####### **Correspondence:** Alexander Shoushtari (shoushta@mskcc.org)

**Background**

Many patients (pts) with advanced melanoma experience disease progression despite receiving immune checkpoint inhibitors (ICI). Resistance to ICIs may be due to an immunosuppressive tumor microenvironment. Oncolytic viruses have the unique ability to both prime and boost immune responses. ONCOS-102 is a granulocyte-macrophage colony stimulating factor (GM-CSF) expressing oncolytic adenovirus (Ad5/3-D24-GMCSF). A prior phase I study demonstrated both innate and adaptive immune responses in various solid tumors. The aim of this study is to assess the safety, clinical and immune responses in pts with advanced melanoma who have progression despite prior PD-1 blockade.

**Methods**

Eligible pts had advanced or unresectable melanoma progressing after PD-1 blockade. Pts received 3 intra tumoral injections of ONCOS-102 at 3x1011 viral particles on days 1, 4 and 8 followed by pembrolizumab (2mg/kg or 200mg flat dose) on day 22 and then every 3 weeks until week 27. Biopsies were performed at baseline, Week 3 and Week 9. Primary objective was safety of the treatment. Secondary objectives: Objective Response Rate (ORR, defined as CR or PR), Clinical Benefit Rate (CBR, defined as PR, CR or SD) by RECIST1.1 at 27 weeks, and changes in immune cell subsets.

**Results**

9 pts were enrolled. Median age 73 years (40-87), 4/9 female, AJCC stage III (6 pts) and stage IV (3 pts). All pts had prior anti-PD1. 4 pts had previous anti-CTLA4, 3 T-VEC, 2 BRAFi/MEKi, 1 IL-2 and 1 TLR9 agonist. RECIST1.1 confirmed responses were seen in 3/9 pts (33%) with one CR and two PR. CBR was also 33%. The most common AEs were chills and fever associated with adenovirus replication. There were no Dose Limiting Toxicities. 5 SAEs were seen in 3 pts, including one pt with infectious colitis related to ONCOS-102. Innate immune activation defined as >2-fold increase in pro-inflammatory cytokines and circulating CD8+ and PD1+CD8+ T cells was seen in all pts. Multiplex IHC in 7 paired tumor biopsies confirmed lesional infiltration of CD8+ T-cells in all pts and PD1+CD8+ T-cells in 6/7 pts. IFNy ELISPOT revealed 2 patients with de novo tumor specific T-cells (MAGE-A1, NY-ESO-1) and 2 patients with MAGE-A1 and/or NY-ESO-1 at baseline that increased during the study.

**Conclusions**

ONCOS-102 combined with pembrolizumab is tolerable and appears to induce systemic and lesional immune activation that can lead to objective responses in anti-PD-1 refractory melanomas. An expansion cohort is currently enrolling pts to receive up to 24 weeks of ONCOS-102 injections.

**Trial Registration**

ClinicalTrials.gov NCT02879669

**Ethics Approval**

This study was approved by the IRBs of Memorial Sloan Kettering Cancer Center, Fox Chase Cancer Center and the University of Maryland Greenebaum Comprehensive Cancer Center.

#### O21 Safety and clinical activity of avelumab (MSB0010718C), an anti-programed death-ligand 1 (PD-L1) antibody, in recurrent thymic epithelial tumors (TETs)

##### Arun Rajan, MD^1^ , Haobin Chen^1^, Chen Zhao, MD^1^, Shannon Swift^1^, Andrew Mammen^2^, Alessandra Brofferio^3^, Emerson Padiernos^1^, Eva Szabo, MD^1^, Udayan Guha^1^, Raffit Hassan, MD^1^, Seth Steinberg^1^, Yo-Ting Tsai^1^, Renee Donahue, PhD^1^, Jeffrey Schlom^1^, James Gulley, MD, PhD, FACP^1^

###### ^*1*^*National Cancer Institute, Bethesda, MD, United States* ; ^*2*^*National Institutes of Arthritis and Musculoskeletal and Skin Diseases, Bethesda, MD, United States* ; ^*3*^*National Heart, Lung, and Blood Institute, Bethesda, MD, United States*

####### **Correspondence:** Arun Rajan (rajana@mail.nih.gov)

**Background**

TETs (composed of thymomas and thymic carcinomas) are PD-L1-expressing tumors with low tumor mutation burden, and particularly with thymomas, defective immune tolerance that increases the risk of paraneoplastic autoimmunity. We have previously demonstrated activity of avelumab with an increased incidence of skeletal and cardiac muscle inflammation in a small cohort of thymoma patients. The present study aims to confirm the safety and activity of avelumab and evaluate cyclosporine A (CsA) for secondary prophylaxis against immune myositis and isolated troponin elevation.

**Methods**

Patients with recurrent TETs and no active autoimmune disease were eligible and received avelumab 10 mg/kg intravenously q2weeks. Primary objectives were evaluation of safety (by CTCAE v4.0) and response rate (by RECIST v1.1). Patients developing non-life-threatening immune-related adverse events (irAEs) resumed avelumab after appropriate management. CsA was used for secondary prophylaxis against musculoskeletal irAEs at the lowest effective dose. Immune activation was assessed by flow cytometry and ELISA. This study is approved by the NCI Institutional Review Board and registered in clinicaltrials.gov (NCT03076554).

**Results**

Twenty-three subjects (12 thymoma, 11 thymic carcinoma; 10 female; median age 56 yrs) were enrolled between April 2017 and October 2018. Twenty-two subjects were evaluable for response. Among thymoma patients: partial response: 2 (17%), stable disease: 10 (83%). Among thymic carcinoma patients: partial response: 2 (20%), stable disease: 6 (60%), progressive disease: 2 (20%). After a median potential follow-up of 18.6 months, median progression-free survival for thymoma patients was 6.4 months (95% CI: 3.7 months – not estimable) and 14.7 months (95% CI: 1.3 months – not estimable) for patients with thymic carcinoma. irAEs occurred in 12 (52%) patients (thymoma 58%, thymic carcinoma 45%). Most common irAEs of any grade included myositis (17%), sicca syndrome (13%), myocarditis and thyroiditis (9% each). Avelumab rechallenge with CsA secondary prophylaxis was feasible in 3 of 4 (75%) patients with myositis with no further issues. irAEs caused treatment discontinuation in 3 (13%) patients. No treatment-related deaths occurred. All patients had high pre-treatment soluble PD-L1 levels compared with healthy donors. Low baseline B-cell counts correlated with clinical response and occurrence of some irAEs.

**Conclusions**

Avelumab is safe and active in TET patients. CsA can prevent recurrence of immune myositis. This is the first reported use of CsA for this indication with implications on the feasibility of immunotherapy for patients with thymoma and/or underlying autoimmunity. This trial is being amended to increase accrual ceiling with a phase II design.

**Acknowledgements**

This research was supported in part by the Intramural Research Program of the NIH, National Cancer Institute, Center for Cancer Research.

**Trial Registration**

Clinicaltrials.gov ID: NCT03076554

**Ethics Approval**

This study is approved by the NCI Institutional Review Board and registered in clinicaltrials.gov (NCT03076554).

### Clinical Trial In Progress

#### O22 Phase II trial of therapeutic vaccine consisting of autologous dendritic cells loaded with autologous tumor cell antigens from self-renewing cancer cells in patients with newly diagnosed glioblastoma

##### Daniela Bota^1^, David Piccioni, MD, PhD^2^, Renato LaRocca, MD^3^, Christopher Duma, MD^4^, Robert Aiken, MD^5^, Santosh Kesari, MD, PhD^6^, Jose Carrillo, MD^6^, Thomas Taylor, PhD^1^, Candace Hsieh^7^, Gabriel Nistor, MD^7^, Robert Dillman, MD, FACP^7^

###### ^*1*^*University of California Irvine, Orange, CA, United States* ; ^*2*^*University of California San Diego, San Diego, CA, United States* ; ^*3*^*Norton Hospital, Louisville, KY, United States* ; ^*4*^*Hoag Hospital, Newport Beach, CA, United States* ; ^*5*^*Rutgers University, NewBrunswick, NJ, United States* ; ^*6*^*John Wayne Cancer Institute, Santa Monica, CA, United States* ; ^*78*^*AiVita Biomedical, Inc., Irvine, CA, United States*

####### **Correspondence:** Robert Dillman (bob@aivitabiomedical.com)

**Background**

Despite advances in surgery, radiation therapy, and chemotherapy, glioblastoma (GBM) remains a highly lethal disease, and has been relatively refractory to anti-checkpoint therapy, presumably because of limited host anti-tumor immune responses. Treatment with an effective vaccine could increase immune responses and improve survival.

**Methods**

This phase II trial was approved by the Western IRB. AV-GBM-1, patient-specific dendritic cell vaccines loaded with autologous tumor antigens from self-renewing cancer cells, is administered as an adjunctive therapy after completion of standard optimal therapy which includes maximum safe surgical resection followed by concurrent treatment with radiation therapy and temozolomide chemotherapy. Key eligibility criteria are a diagnosis of primary GBM, age 70 or less at the time of tumor harvest, successful establishment of a short-term cancer cell line, successful leukapheresis collection of monocytes, and a Karnofsky Performance Status of 70 or greater at the time of enrollment, which takes place just prior to starting chemoradiation. Tumor is collected at the time of surgery from which a short-term cell line is derived. Dendritic cells are produced by incubating peripheral blood monocytes with GM-CSF and IL-4. The antigen source is a lysate of irradiated tumor cells from the cell culture. After completion of concurrent chemoradiation, vaccine doses admixed with GM-CSF are injected subcutaneously at week 1, 2, 3, 8, 12, 16, 20, and 24 for up to eight doses. The objective is to achieve a 75% survival rate15 months from enrollment, which would be a 50% increase over results achieved with standard therapy alone.

**Results**

Cell line success rate for submitted GBM samples is 39/41 with 3 in progress. A satisfactory leukapheresis product has been obtained for 34/35 patients, but was repeated for 4. 30 of a planned 55 patients have been enrolled for intent-to-treat. 24 have started treatment; 3 have completed all 8 doses, 3 discontinued early for disease progression, 18 are currently in treatment. 121 doses have been administered in total. Proteomic analysis of blood samples obtained at baseline and at the time of each vaccine injection, has demonstrated Th1, Th2, and Th17 immune responses in 7/8 patients. No significant toxicity directly attributed to the vaccine has been reported.

**Conclusions**

Although logistically complex, this patient-specific vaccine approach is feasible. Furthermore, the vaccine has been well-tolerated and has induced immune responses in nearly all patients analyzed to date. [NCT03400917]

**Trial Registration**

ClinicalTrials.Gov . NCT03400917

**Ethics Approval**

The study was approved the Western Institutional Review Board, approval number 20182582

#### O23 Sitravatinib in combination with nivolumab demonstrates clinical activity in platinum-experienced patients with urothelial carcinoma (UC) who progressed on prior immune checkpoint inhibitor (CPI)

##### Pavlos Msaouel, MD, PhD^1^, Arlene Siefker-Radtke, MD^1^, Randy Sweis, MD^2^, Amir Mortazavi^3^, Nicholas Vogelzang, MD^4^, Ulka Vaishampayan, MD^5^, Thomas Bradley, MD^6^, Manojkumar Bupathi^7^, Luke Nordquist, MD, FACP^8^, David Shaffer^9^, Joel Picus^10^, Jeffrey Yorio, MD^11^, Shifeng Mao^12^, Gurjyot Doshi^13^, Daniel Spitz^14^, Sunil Gandhi^14^, Daniel Chong^15^, Arash Rezazadeh Kalebasty^16^, James Christensen^17^, Peter Olson^17^, Demiana Faltaos, PharmD, PhD^17^, Ronald Shazer^17^, Maria Winter^17^, Delia Alvarez^17^, Hirak Del-Torossian^17^, Jonathan Rosenberg, MD^18^

###### ^*1*^*The University of Texas MD Anderson Cancer Center, Houston, TX, United States* ; ^*2*^*The University of Chicago Medicine, Chicago, IL, United States* ; ^*3*^*The Ohio State University Comprehensive Cancer Center, Columbus, OH, United States* ; ^*4*^*Comprehensive Cancer Centers of Nevada, Las Vegas, NV, United States* ; ^*5*^*Barbara Ann Karmanos Cancer Institute, Detroit, MI, United States* ; ^*6*^*Northwell Health - Monter Cancer Center, Lake Success, NY, United States* ; ^*7*^*Rocky Mountain Cancer Centers, Littleton, CO, United States* ; ^*8*^*Urology Cancer Center and GU Research Network, Omaha, NE, United States* ; ^*9*^*New York Oncology Hematology, Albany, NY, United States* ; ^*10*^*Washington University in St. Louis School of Medicine, St. Louis, MO, United States* ; ^*11*^*Texas Oncology-Austin, Austin, TX, United States* ; ^*12*^*Allegheny General Hospital, Pittsburgh, PA, United States* ; ^*13*^*Texas Oncology-Memorial City, Houston, TX, United States* ; ^*14*^*Florida Cancer Specialists & Research Institute, West Palm Beach, FL, United States* ; ^*15*^*Virginia Cancer Specialists, Fairfax, VA, United States* ; ^*16*^*Norton Cancer Institute, Louisville, KY, United States* ; ^*17*^*Mirati Therapeutics, Inc., San Diego, CA, United States* ; ^*18*^*Memorial Sloan-Kettering Cancer Center, New York, NY, United States*

####### **Correspondence:** Jonathan Rosenberg (rosenbj1@mskcc.org)

**Background**

Sitravatinib is a spectrum-selective TKI which targets TAM receptors (Tyro3, Axl, MerTK), split family receptors (VEGFR2 and c-Kit) and c-Met. Inhibition of these receptor tyrosine kinases may enhance antitumor activity by reducing Type 2 tumor-associated macrophages, regulatory T cells and myeloid derived suppressor cells and enhancing a T cell-mediated anti-tumor immune response. Given these pleiotropic immune-enhancing effects, we hypothesized that the combination of sitravatinib with nivolumab will restore or enhance CPI clinical activity in pts with immunotherapy-resistant UC.

**Methods**

Study 516-003 objectives include evaluation of safety and efficacy in patients (pts) with advanced or metastatic UC. Cohort 1 includes UC pts who have disease progression on or after CPI and were previously treated with platinum-based chemotherapy. Sitravatinib is administered orally, daily, in 28-day cycles; nivolumab is administered intravenously, either 240 mg every 2 weeks or 480 mg every 4 weeks. A target sample size of 17 pts with futility based on the Predictive Probability Design (PPD) is used, with the option to expand if clinically active. Other objectives include pharmacokinetics (PK) and correlative biomarkers.

**Results**

The 516-003 study met its prespecified measure of clinical activity in Cohort 1 with 3 confirmed responses in the first 17 evaluable patients, warranting expansion of enrollment, which is ongoing. As of 26 July 2019, Cohort 1 has enrolled 25 pts with 21/25 having had > 1 on-study tumor assessment and 13/25 having had > 2 on-study tumor assessments. Sixteen out of 21 pts have demonstrated tumor reductions. Six out of 21 pts have achieved a partial response (PR): 3 confirmed (including 1 unconfirmed Complete Response on 2nd tumor assessment) and 3 unconfirmed. Clinical benefit rate (CBR), defined as confirmed response or SD on > 2 study assessments with treatment duration > 14 wks, was observed in 9/13 pts. Among pts with reported adverse events (AEs), treatment-related AEs (≥20% of pts; all-grades in decreasing frequency) included fatigue, diarrhea, decreased appetite, dysphonia, increased alanine aminotransferase, nausea, increased lipase, palmar-plantar erythrodysesthesia syndrome (PPE), increased aspartate aminotransferase, hypertension, dysgeusia, proteinuria, and vomiting. Grade 3 treatment-related AEs in > 1 pt included hypertension, fatigue, diarrhea, increased lipase, and PPE. There were no Grade 4 or 5 treatment-related AEs. Updated safety, PK and efficacy data will be presented.

**Conclusions**

The combination of sitravatinib with nivolumab is clinically active with manageable side effects in pts with advanced or metastatic UC who progressed on CPI and were previously treated with platinum-based chemotherapy.

**Ethics Approval**

The study was centrally approved by WIRB, IRB tracking number 20181442.

#### O24 Phase II trial of concurrent Nivolumab and radiation therapy in chemotherapy ineligible muscle invasive bladder cancer [NUTRA trial NCT03421652]

##### Saby George, MD, FACP^2^, Lance Heilbrun^3^, Jordan Maier, MD^4^, Brenda Dickow^4^, Michael Kuettel, MD^2^, Arthur Frazier, MD^4^, Stacey Suisham, RN BSN OCN CcRP^4^, Prahlad Parajuli, PhD^4^, Nitin Vaishampayan, MD^4^, Ulka Vaishampayan, MD^4^

###### ^*1*^*Wayne State University and Barbara Ann K, Detroit, MI, United States* ; ^*2*^*Roswell Park, Buffalo, NY, United States* ; ^*3*^*Wayne State University, Detroit, MI, United States* ; ^*4*^*Karmanos Cancer Center, Detroit, MI, United States*

####### **Correspondence:** Ulka Vaishampayan (vaishamu@karmanos.org)

**Background**

Concurrent chemotherapy and radiation is a bladder sparing strategy for management of muscle invasive bladder cancer (MIBC). However many patients are not candidates for chemotherapy due to comorbid conditions, performance status, or having received prior chemotherapy with progression. We are conducting a study in a chemotherapy ineligible patient population with the primary objective of comparing the 12-month rate of progression-free (PFS) survival achieved with the combination of nivolumab, a programmed death (PD-1) inhibitor, and radiation therapy in localized/locally advanced urothelial cancer patients, to a historical control reference rate.

**Methods**

Eligible patients have MIBC, are not candidates for standard chemoradiation strategy due to any of the following; performance status of 2, creatinine clearance < 60ml/min, cardiac disease, neuropathy, or intolerance to previous treatment. Creatinine clearance >40ml/min, normal marrow and liver function were required. The primary endpoint is progression free survival rate at 12 months. Nivolumab is started within 3 days of radiation therapy and is administered at the dose of 240 mg intravenously every 2 weeks for a maximum of 6 months. Radiation therapy is per standard of care for bladder cancer. Imaging and cystoscopy and biopsy evaluation is required at months 3, 6 and 12 and then annually until progression.

**Results**

14 evaluable patients; median age 71 years- (range 54 to 95 years) 12 males and 2 females, 3 African American and 11 Caucasian have been enrolled. 11 patients have completed radiation therapy. 3 patients had prior intravesical therapy and 2 had prior systemic chemotherapy with cisplatin and gemcitabine. 2 patients were clinical T3 /T4 and one patient had pelvic LNpathy. The regimen was well tolerated with Nivolumab and radiation therapy. No severe treatment related adverse events were noted. 5 patients needed steroids due to immune mediated adverse events. 8 patients are evaluable after one assessment and 3 patients demonstrated complete remission. PDL-1 expression is being tested. Preliminary immune evaluation results indicate an improvement in CD4:CD8 ratio, natural killer (NK) cell expansion as well as enhanced expression of T cell and NK cell activation markers .

**Conclusions**

Concurrent nivolumab and radiation therapy is tolerable and showed promising efficacy in an elderly population with multiple comorbidities. Immune correlates data will be available for the treated patients and will be reported.

**Trial Registration**

The Clinical trials.gov no NCT03421652

**Ethics Approval**

The study was approved by Wayne State University's Ethics Board, approval number 083417MP2F

#### O25 Dominant negative PD1 armored CART cells induce remission in relapsed or refractory Non-Hodgkin Lymphoma (NHL) patients

##### Cheng fei Pu, Lei Xiao

###### Innovative Cellular Therapeutics, Shanghai, China

####### **Correspondence:** Lei Xiao (xiaolei@ictbio.com)

**Background**

The chimeric antigen receptor (CAR) T cell treatment has been demonstrated as an effective therapy to treat relapsed/refractory B cell malignancy. However, tumor microenvironment influences and affects the efficacy of CAR T treatment. For example, programmed death ligand 1/2 (PDL1/2) may inhibit the CAR T cells via interaction with up-regulated programmed cell death protein 1 (PD1) after T cells activation, suppressing the tumor-killing capability of the CAR T cells.

Here, we generated CAR T expressed an anti-CD19 CAR molecule and a dominant-negative PD1 molecule. Compared with conventional CART cells, these “armored” CART cells showed the enhanced capability of tumor-killing and more “memory-like” phenotypes after multiple-round tumor challenging . These results suggest dominant-negative PD1 molecules may protect CART cells from exhaustion in the tumor microenvironment.

**Methods**

We designed a CAR lentivirus vector that consisted of a humanized CD19-specific single-chain variable fragment (scFv), a 4-1BB costimulatory domain, and a CD3ζ signaling domain. Patient’s CD3 T cells were transduced with CAR lentivirus. Quality controls for fungi, and endotoxin etc were performed. After infusion, serial peripheral blood samples were collected, and the expansion and cytokine release of CART cells were detected. The evaluation of response level for patients were performed at month 1,month 3,and month 6 by PET/CT.

**Results**

Further, we reported the findings of a clinical trial for six relapsed or refractory B-cell non-Hodgkin lymphoma (NHLs) patients treated using our armored CAR T cells. These six patients failed multiple rounds of chemotherapy and radiotherapy. In the clinical trial, the patients were infused with autologous CAR T cells range from 1×10e6/kg to 8×10e6/kg. PET/CT showed significant tumor shrinkage and SUV max declines in all six patients, and the ongoing responses were monitored . The best overall response rate (ORR)was 100% .

**Conclusions**

The results of these six patients in the clinical trial showed that our armored CAR T cells achieved the significant anti-bulky lymphoma response while causing limited and tolerated cytokine release syndrome and central nervous system toxicity. Thus, dominant-negative PD1 molecules may increase CAR T cells persistence in patients, enhancing the efficacy of CAR T cells for treating blood cancer. Finally, dominant-negative PD1 can be used as a platform technology and may be applied to other adoptive cellular immunotherapies such as TCR-T or TIL in the treatment of solid tumors. We are continuing to monitor current patients and recruit more patients for the clinical trial.We are continuing to recruit more patients for the clinical trial.

#### O26 A phase II study of bemcentinib (BGB324), a first-in-class selective AXL inhibitor, in combination with pembrolizumab in patients with advanced NSCLC: Updated analysis

##### Matthew Krebs, MD PhD^1^ , Paal Brunsvig, MD PhD^2^, Åslaug Helland^2^, Nuria Vinolas Segarra^3^, Santiago Ponce Aix, MD^4^, Enric Carcereny Costa, MD^5^, Manuel Dómine Gomez, MD, PhD^6^, Jose Manuel Trigo Perez^7^, Edurne Arriola^8^, Rosario Garcia Campelo^9^, James Spicer, MD, PhD^10^, Jonathan Thompson, MD MS^11^, Ana Laura Ortega Granados^12^, Robert Holt, PhD^13^, James Lorens, PhD^13^, Muhammad Shoaib, MD^13^, Abdul Siddiqui^13^, Emmett Schmidt, MD PhD^14^, Michael Chisamore, PhD^14^, Enriqueta Felip, MD PhD^15^

###### ^*1*^*Christie NHS Foundation Trust and The University of Manchester, Manchester, United Kingdom* ; ^*2*^*Oslo University Hospital, Oslo, Norway* ; ^*3*^*Hospital Clinic, Barcelona, Spain* ; ^*4*^*Hospital 12 de Octubre, Madrid, Spain* ; ^*5*^*Catalan Institute of Oncology, Badalona, Spain* ; ^*6*^*Hospital Universitario, Madrid, Spain* ; ^*7*^*Hospital Virgen de la Victoria, Málaga, Spain* ; ^*8*^*Hospital del Mar, Barcelona, Spain* ; ^*9*^*University Hospital A Coruña, A Coruña, Spain* ; ^*10*^*King's College London, Guy's Hospital, London, United Kingdom* ; ^*11*^*Medical College of Wisconsin, Milwaukee, WI, United States* ; ^*12*^*Complejo Hospitalario de Jaén, Jaén, Spain ;*^*13*^*BerGenBio ASA, Oxford, United Kingdom* ; ^*14*^*Merck & Co., Inc., Kenilworth, NJ, United States* ; ^*15*^*Hospital Universitari Vall d'Hebron, Barcelona, Spain*

####### **Correspondence:** Matthew Krebs (matthew.krebs@christie.nhs.uk)

**Background**

AXL is an RTK implicated in EMT, immune suppression and resistance to anti-PD1 therapy. Bemcentinib (BGB324), a first-in-class, oral, selective and potent AXL kinase inhibitor, enhances anti-PD1 efficacy in pre-clinical models.

**Methods**

This is a PhII single-arm, 2-stage study with bemcentinib (200mg/d) and pembrolizumab (200 mg/q3wk) for previously-treated, IO-naïve patients with Stage IV lung adenocarcinoma. Primary endpoint was ORR according to RECIST 1.1 with pre-defined minimum requirement of 18% RR in the first Stage (n=24) to proceed to Stage 2. Secondary endpoints included DCR, PFS, OS and safety. Updated biomarker analysis and correlations with clinical endpoints will be presented, including quantification of tumor and immune cell AXL, PD-L1 protein expression, and genetic and transcriptome analysis.

**Results**

Enrolment for both stages is complete. As of July 2019, 50 patients have been dosed with the combination. Median age 65 years (range: 39-82), 60% male. All patients received one prior line of platinum-based chemotherapy or an approved EGFR/ALK-directed therapy. AXL status was available for 33 patients: 19 AXL-positive, 14 AXL-negative. PD-L1 status was available for 42 patients: 4 TPS >50%, 16 TPS 1-49%, and 22 TPS <1%.

Most common TEAEs (occurring in >25% of patients) were asthenia/fatigue (54%, 18% grade 3+), transaminase increases (46%, 20% grade 3+), diarrhoea (32%, no grades 3+) and decreased appetite (28%, no grade 3+). All cases of transaminase increase were reversible and managed with concomitant administration of systemic corticosteroids and interruption of study treatments, or Grade 1 (mild) events.

To date, 43 patients were radiologically evaluable, with 10 PRs observed (ORR 23%). 17 AXL-positive patients were radiologically evaluable, with 6 responses observed (35%), as opposed to 2 (15%) in the 13 radiologically evaluable AXL-negative patients. Of the 10 responders, 4 had a TPS 50% (1 AXL-positive); 2 did not have a PD-L1 status available (1 AXL-positive, 1 unknown).

In Stage 1, mPFS was 4.0 months overall (95% CI 1.9 –NR) and 5.9 months in AXL-positive pts (n=10; 3.0 - NR). mPFS for the entire cohort continues to mature and will be reported at the meeting.

**Conclusions**

Promising clinical activity has been seen, particularly in pts with AXL-positive disease and including patients with PD-L-negative tumours. Overall, bemcentinib in combination with pembrolizumab was well tolerated. Updated results will be reported at the meeting, including PFS for all Cohort A patients as well as further biomarker data.

**Acknowledgements**

The authors would like to thank all patients and their caretakers for participating in this trial.

**Trial Registration**

NCT03184571

**References**

1. Schoumacher M, Burbridge M. Key Roles of AXL and MER Receptor Tyrosine Kinases in Resistance to Multiple Anticancer Therapies. Current Oncology Reports, 2017.

2. Hugo et al, Cell (2016)

3. Gausdal G, et al. Abstract 566: BGB324, a selective small molecule inhibitor of the receptor tyrosine kinase AXL, enhances immune checkpoint inhibitor efficacy. Cancer Res. 2016.

**Ethics Approval**

This study was approved by all relevant institutions, including London Bridge Research Ethics Committee (UK), REC-South East (Norway), Drug Research Ethics Committee of the University Hospital Clinic of Barcelona (Spain), MCW/FH Institutional Review Board #4, Medical College of Wisconsin (USA).

#### O27 Clinical response to Tumor Infiltrating Lymphocytes (TIL) in stage 4 Non-small Cell Lung Cancer (NSCLC) correlates with Neoantigen-Specificity: a phase I trial

##### Chao Wang, PhD^1^, Jamie Teer^1^, Jiqiang Yao, PhD^1^, Anadon Galindo Carmen M^1^, David Noyes^1^, Eric Toloza^1^, John Mullinax, MD^1^, Ana Landin^1^, Jhanelle Gray^1^, Tawee Tanvetyanon^1^, Andreas Saltos^1^, Linda Kelley, PhD^1^, Bin Fang, PhD^1^, John Koomen, PhD^1^, Amod Sarnaik, MD^1^, Sungjune Kim, MD PhD^1^, Shari Pilon-Thomas, PhD^1^, Jose Conejo-Garcia, MD, PhD^1^, Scott Antonia, MD PhD^2^, Eric Haura, MD^1^ , Ben Creelan, MD, MS^1^

###### ^*1*^*Moffitt Cancer Center, Tampa, FL, United States* ; ^*2*^*Duke University, Durham, NC, United States*

####### **Correspondence:**Eric Haura (Eric.Haura@moffitt.org), Ben Creelan (Ben.Creelan@moffitt.org)

**Background**

Neoantigens are generated by non-synonymous somatic mutations and could be recognized by self T cells and elicit neoantigen specific T cell responses[1]. Scientists have shown that adoptive transfer of tumor infiltrating lymphocytes could induce durable remissions for patients with epithelial cancers[2,3]. We hypothesized that TIL therapy may induce remissions in anti-PD1-refractory patients. Therefore, we initiated a phase I trial to evaluate the safety and preliminary activity of TIL therapy in anti-PD1-refractory non-small cell lung cancer (NSCLC) patients and characterize the corresponding neoantigen specific T cells.

**Methods**

Eligible patients received TIL harvest, followed by 4 cycles of nivolumab, with 2 serial CT scans. Patients with enlarging or new tumors proceeded to receive lymphodepletion with cyclophosphamide/fludarabine (Cy/Flu), TIL infusion and IL-2. Tumors from these patients were resected, morselized and tested using whole exome sequencing and RNA sequencing. Tumor fragments were cultured and only reactive ones were pooled and expanded for TIL infusion. Predicted neoantigen epitopes were prioritized using customized algorithms and synthesized for validation assays. Reactive neoantigens were selected using ELISA/ELISpot assay and neoantigen specific T cells were identified according to their TCRVβ sequences.

**Results**

Of 15 patients enrolled to date, 14 had successful TIL expansion. Of 12 patients who received TIL therapy, 3 have achieved a confirmed partial response (PR). The median overall survival (OS) for all enrolled patients has not yet been reached. We have discovered neoantigen-specific T cells and followed the number of neoantigen specific T cells using TCRVβ sequencing. Neoantigen specific T cell clonotypes increased significantly after TIL therapy, and were retained at a relatively high level for a long time. However, some neoantigen recognition was lost in the TIL of a new recurrent tumor, indicating acquired resistance to TIL treatment may occur in some patients. In an example, allelic editing of a neoantigen was detected in a recurrent tumor, showing that fitness pressure may be exerted upon tumor cell clones after TIL treatment.

**Conclusions**

Adoptive cell transfer with TIL and nivolumab for NSCLC had acceptable toxicity and durable responses in this ongoing trial. TIL therapy can increase the number of neoantigen specific T cells, which mediated antitumor effects in responders.

**Acknowledgements**

This work has been supported in part by the Molecular Genomics Core, Tissue Core and Flow Cytometry Core at the H. Lee Moffitt Cancer Center & Research Institute, a comprehensive cancer center designated by the National Cancer Institute and funded in part by Moffitt’s Cancer Center Support Grant (P30-CA076292). This work is funded by SU2C grant.

**Trial Registration**

NCT03215810

**References**

1. Yarchoan M, Johnson BA 3rd, et al. Targeting neoantigens to augment antitumour immunity. Nat Rev Cancer. 2017; 17: 209-222.

2. Lu YC, Yao X, et al. Efficient identification of mutated cancer antigens recognized by T cells associated with durable tumor regressions. Clin Cancer Res. 2014; 20:3401-3410.

3. Yossef R, Tran E, et al. Enhanced detection of neoantigen-reactive T cells targeting unique and shared oncogenes for personalized cancer immunotherapy. JCI Insight. 2018; 3: e122467.

**Ethics Approval**

The study was approved by Chesapeake IRB, approval number Pro00021984.

**Fig. 1:**
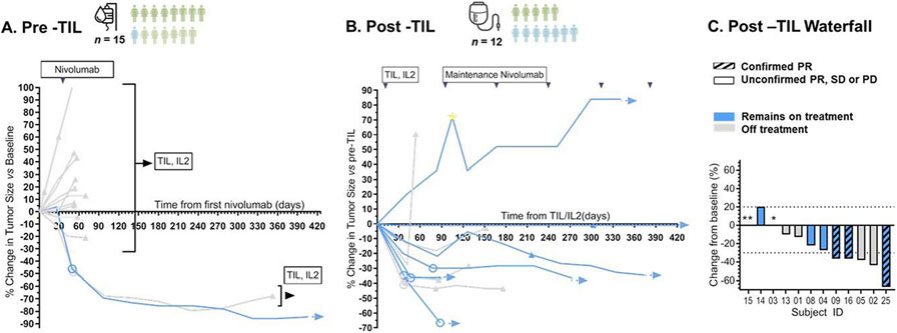
**(abstract 027).** See text for description

**Fig. 2:**
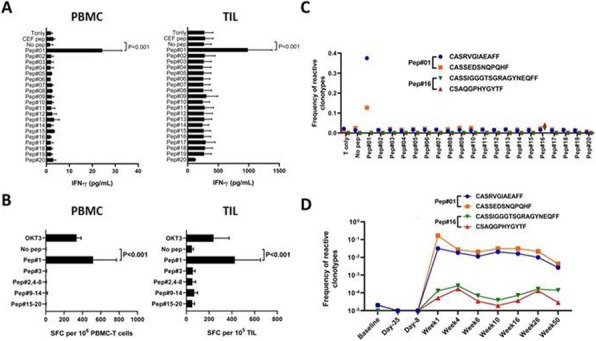
**(abstract 027).** See text for description

#### O28 First-in-class small molecule CA-170 targeting VISTA: A report on efficacy outcomes from a cohort of 12 malignant pleural mesothelioma (MPM) patients in study CA-170-101

##### Marjorie Zauderer, MD, MS, FACP^1^ , Joshua Brody, MD^2^, Thomas Marron, MD PhD^2^, Simon Pacey^3^, Robert Martell^4^, Hongwei Wang^4^, James Spicer, MD, PhD^5^

###### ^*1*^*Memorial Sloan Kettering Cancer Center, New York, NY, United States* ; ^*2*^*Mount Sinai, New York, NY, United States* ; ^*3*^*The University of Cambridge, Cambridge, United Kingdom* ; ^*4*^*Curis, Inc., Lexington, KY, United States*; ^*5*^*King's College London, London, United Kingdom*

####### **Correspondence:** Marjorie Zauderer (zauderem@mskcc.org)

**Background**

MPM is an aggressive disease with few therapeutic options. VISTA, a distinct immune checkpoint protein separate from the PD-1/PD-L1 pathway, is involved in inhibition of anti-tumor responses and is expressed on infiltrating lymphocytes and tumor cells in approximately 90% of MPM patients. CA-170, a small molecule that targets both VISTA and PD-L1, has shown anti-tumor activity in multiple in vivo models and early phase trials provided evidence of a favorable safety/PK profile with signs of anti-tumor activity in immune checkpoint inhibitor (iCPI)-naïve patients. CA-170-101 is a Phase 1 dose finding study in advanced solid tumors and lymphomas in which a cohort of 12 MPM patients was evaluated for anti-tumor activity.

**Methods**

MPM patients were enrolled into two pharmacologically active dose levels of 200 or 1200 mg BID. The primary objectives were to determine safety, tolerability and RP2D, and secondary objectives included pharmacokinetics and anti-tumor activity, PFS and ORR. Patients had histologically-confirmed disease, ≥1 prior therapy including pemetrexed-platinum doublet, no prior ICI, measurable disease per RECIST, ECOG 0-1, life expectancy >3 months, and adequate organ function. Paired tumor biopsies were obtained when clinically feasible.

**Results**

As of July 2019, 12 patients with MPM were treated with CA-170. 11 of 12 patients have discontinued due to disease progression (or death due to disease progression); 1 patient remains on study treatment at 1200 mg BID. As of the data cut-off, 5 of 11 patients evaluable for response (>1 post-baseline tumor assessment) had stable disease per RECIST. One patient remains on study treatment for over 21 weeks with stable disease. An additional 4 patients were on study drug for over 12 weeks. No partial or complete responses were reported according to RECIST or immune-related Response Criteria. Serious immune-related adverse events were not observed in CA-170-101. The safety profile of CA-170 is distinct from other known iCPIs monoclonal antibodies.

**Conclusions**

Evidence of immune-modulating activity was observed in non-clinical experiments of peripheral blood and tumor tissue specimens. Modest anti-tumor activity was observed in patients with resistant/refractory mesothelioma. CA-170 was well tolerated and shows favorable clinical PK. Clinical trial information: NCT02812875.

**Trial Registration**

NCT02812875

**Ethics Approval**

This study involves human subjects and complies with the principles found in the Declaration of Helsinki. The study protocol was approved by the appropriate IRB/IEC at each institution.

#### O29 Phase I study of multisite stereotactic body radiotherapy plus nivolumab and urelumab or nivolumab and cabiralizumab in patients with advanced solid tumors

##### Corey Foster, MD, MS^1^, Jason Luke, MD, FACP^2^, Robyn Hseu, CCRP, MLIS^1^, Linda Janisch^1^, Gini Fleming, MD^1^, Steven Chmura, MD, PhD^1^

###### ^*1*^*The University of Chicago Medicine, Chicago, IL, United States* ; ^*2*^*University of Pittsburgh Medical Center, Pittsburgh, PA, United States*

####### **Correspondence:** Steven Chmura (schmura@radonc.uchicago.edu)

**Background**

Stereotactic body radiotherapy (SBRT) and immune checkpoint inhibition have been combined safely with preliminary suggestion of enhanced treatment effect. Agonism of CD137 or depletion of immunosuppressive myeloid cells by anti-colony stimulating factor 1 receptor (CSF1R) antibodies have been shown in preclinical studies to enhance each therapy and overcome resistance. The safety and efficacy of SBRT plus multiple checkpoint antibodies is unknown.

**Methods**

This is a single-institution, phase I study of multisite SBRT to 1-4 metastases with nivolumab (nivo) (480 mg IV every 4 weeks) and non-randomized administration of either (1) urelumab (ure) (CD137 agonist, 8 mg IV every 4 weeks) or (2) cabiralizumab (cab) (anti-CSF1R, 4 mg/kg IV every 2 weeks). Key eligibility criteria include ECOG 0-1 and progression on standard therapies. The primary endpoint is dose-limiting toxicity (DLT) defined as grade 3+ according to the CTCAEv4 occurring ≤3 months from the start of treatment. The recommended organ-specific SBRT doses will be associated with a ≤33% rate of DLTs. Secondary endpoints include RECIST response, progression-free survival, and overall survival. Pre- and post-SBRT biospecimens are collected for molecular correlative studies.

**Results**

Fifty-one eligible patients with 21 different tumor types including primarily non-immunotherapy responsive histologies have been enrolled and treated as of May 2019. Twenty-eight patients are evaluable for DLTs at 3 months with 3 (11%) experiencing ≥1 DLT. DLTs were observed in 1/6 (17%), 1/7 (14%), and 1/6 (17%) patients receiving SBRT to metastases in the peripheral lung, central lung, and abdominal/pelvic organs, respectively, while 7 patients receiving SBRT to the liver and 2 patients receiving SBRT to osseous metastases did not experience DLTs. Three of 11 (27%) patients receiving nivo+cab and 0/17 patients receiving nivo+ure with SBRT experienced DLTs. DLTs have included grade 3 maculopapular rash (n=1) and grade 4 creatine phosphokinase elevation (n=2). Of 29 treated patients evaluable for modified RECIST target lesion response, 1 (3%, 0 cab/1 ure) experienced a complete response, 5 (17%, 2 cab/3 ure) experienced a partial response, 9 (31%, 4 cab/5 ure) experienced stable disease, and 14 (48%, 6 cab/8 ure) experienced progressive disease. Enrollment and follow-up continue with closure to accrual anticipated in August 2019.

**Conclusions**

Multisite SBRT plus nivo+cab or nivo+ure preliminarily demonstrates acceptable toxicity with suggestion of anti-tumor activity worth ongoing exploration.

**Trial Registration**

ClinicalTrials.gov, NCT03431948

**Ethics Approval**

This study was approved by the University of Chicago Medicine Internal Review Board; approval number IRB17-1317.

#### O30 A Phase 1 study of AK104, a tetrameric bispecific antibody that targets PD-1 and CTLA-4 in patients with advanced solid tumors

##### Ben Markman, MBBS FRACP^1^, Ben Markman, MBBS FRACP^1^, Ben Tran^2^, Hui Gan, MBBS PhD^3^, Amy Prawira, MD^4^, Jermaine Coward^5^, Xiaoping Jin^6^, Baiyong Li^6^, Max Wang^6^, Yu Xia^6^, Jayesh Desai, MBBS FRACP^2^ , Jayesh Desai, MBBS FRACP^2^, Michael Millward^7^

###### ^*1*^*Monash Health and Monash University, Melbourne, Australia* ; ^*2*^*Peter MacCallum Cancer Centre, Melbourne, Australia* ; ^*3*^*Austin Health, Melbourne, Victoria, Australia* ; ^*4*^*St Vincent’s Hospital Sydney, Toronto, ON, Canada* ; ^*5*^*Icon Cancer Care, Brisbane, Australia;*^*6*^*Akeso BioPharma, Inc. Zhongshan, Anhui, China;*^*7*^*Linear Clinical Research, Nedlands, Australia*

####### **Correspondence:** Jayesh Desai (jayesh.desai@petermac.org)

**Background**

AK104 is a tetrameric PD-1/CTLA-4 bispecific antibody, based on the Akeso Tetrabody platform [1]. A number of studies have shown that combined PD-1 and CTLA-4 blockade is associated with a higher response rate in multiple tumor types. Toxicity associated with anti-CTLA-4 agents in particular is dose limiting, thereby limiting full potential for PD-1 and CTLA-4 combination therapy. AK104 introduces novel T cell targeting mechanisms of action that may provide an improved therapeutic index and a favorable toxicity profile compared to PD-1 and CTLA-4 combination therapy.

**Methods**

A multicenter, Phase I, open-label dose escalation and expansion study in solid tumors (NCT03261011) began in Oct 2017, evaluating the safety, efficacy and recommended phase 2 dose of AK104 administered IV every 2 weeks (q2w). For dose escalation, pts were enrolled at dose cohorts of 0.2, 0.5, 1.0, 2.0, 4.0, 6.0 and 10.0 mg/kg. Pharmacodynamic studies examined Ki67 expression on peripheral T-cells and receptor occupancy (RO).

**Results**

As of 8 July 2019, 40 pts, median age 66.5 years [31-85], have received AK104 at doses of 0.2 mg/kg (n = 1), 0.5 mg/kg (n = 3), 1.0 mg/kg (n = 6), 2.0 mg/kg (n = 3), 4.0 mg/kg (n = 3), 6.0 mg/kg (n = 21) and 10.0 mg/kg q2w (n = 3). Median number of doses was 4 (1–26). Treatment-related adverse events (TRAEs) occurred in 62.5% of pts. G3 TRAEs occurred in 10% [4/40], there were no G4 TRAEs. TRAEs leading to treatment discontinuation occurred in 7.5% [3/40]). Most frequent TRAEs were rash (23%), infusion reaction (15%), fatigue (15%), nausea (13%), and pruritus (10%)(Figure 1). Of 21 evaluable pts treated at doses ≥ 2 mg/kg, ORR was 28.6% (6/21) and disease control rate (DCR) was 47.6% (10/21) (Figure 2). Six responders remain on treatment (Gastric cancer, TNBC, mesothelioma, and large-cell neuroendocrine carcinoma each and 2 MSI-H CRC). Peripheral CD4+ T-cells demonstrated dose dependent increases in Ki-67 expression at D8.

**Conclusions**

AK104 can be given safely in patients up to 10.0 mg/kg and has demonstrated encouraging anti-tumor activity with ORR 28.6% when dosed >2mg/kg in a mixed patient population. Enrolment is currently ongoing at 10.0 mg/kg and in dose expansion cohorts. Updated data, including PK, Ki67, RO and other biomarkers based on pair biopsies will be presented. The mechanism underlying improved safety profile of tetrameric PD-1/CTLA-4 bispecific AK104 will be discussed.

**Trial Registration**

NCT03261011

**References**

1. Li B, Huang Z, Pang X, Zhong T, Chen N, Wang M, Jin X, Xia D, Zhang P, Xia Y. Bispecific antibodies with an anti-PD-1 backbone for cancer therapy generate enhanced immune activity. Cancer Res 2018:78 (13 Suppl): Abstract 3827.

2. National Institute for Health And Care Excellence, 2018, Single technology appraisal for nivolumab with ipilimumab for untreated metastatic renal cell carcinoma [ID1182]. Website: https://www.nice.org.uk/guidance/ta581/documents/committee-papers

**Ethics Approval**

The study was approved by the above-mentioned institutions’ Ethics Committees, approval number 2017-07-538 (Bellberry Human Research Ethics Committee) and HREC/17/MH/229 (Melbourne Health Human Research Ethics Committee).

**Fig. 1 (abstract 030).**
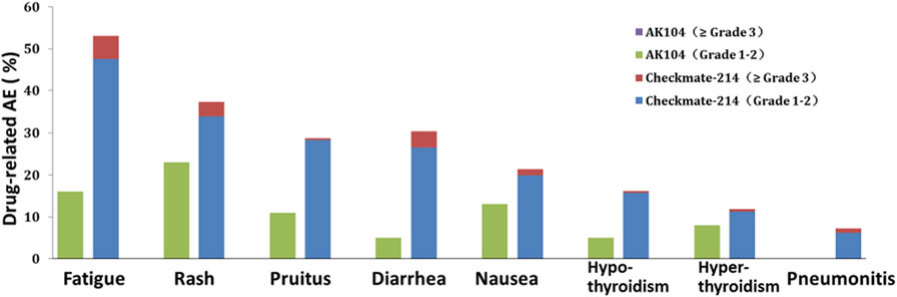
See text for description

**Fig. 2 (abstract 030).**
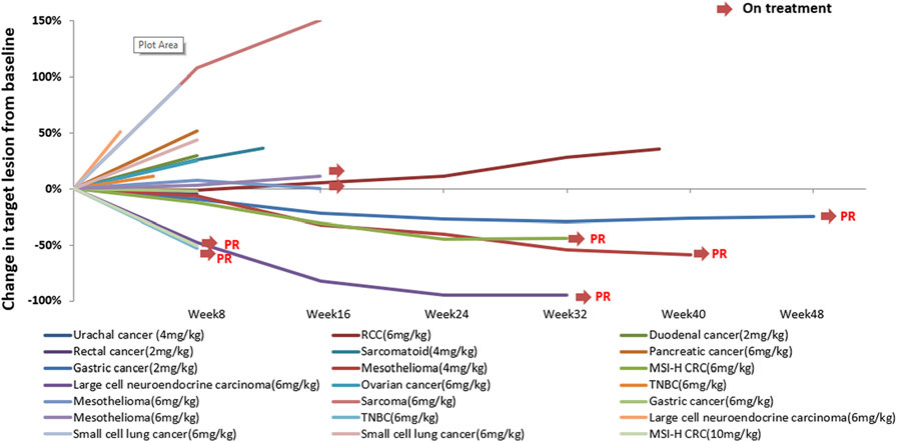
See text for description

#### O31 Intratumoral INT230-6 injection into solid tumors kills tumors and induces immune cell infiltration leading to abscopal responses and prolonged disease control in multiple refractory cancer types

##### Jacob Thomas, MD^1^, Anthony Olszanski, MD, RPh^2^, Nilofer Azad, MD^3^, Lewis Bender, MS, MA, MBA^4^, Ian Walters, MD^4^ , Giles Whalen, MD^5^, Diana Hanna, MD^1^, Vinay Duddalwar, MD^1^, Phillip Cheng, MD^1^, Kevin King, MD^1^, Lilian Siu, MD^6^, Anthony El-Khoueiry, MD^1^

###### ^*1*^*USC, Los Angeles, CA, United States* ; ^*2*^*Fox Chase Cancer Center, Philadelphia, PA, United States* ; ^*3*^*Johns Hopkins, Chevy Chase, MA, United States* ; ^*4*^*Intensity Therapeutics, Westport, CT, United States* ; ^*5*^*UMass, Worcester, MA, United States* ; ^*6*^*PMH, Toronto, ON, Canada*

####### **Correspondence:** Ian Walters (iwalters@intensitytherapeutics.com)

**Background**

INT230-6 is a novel formulation of cisplatin(CIS) and vinblastine(VIN) with an amphiphilic cell penetration enhancer(SHAO), designed specifically for intratumoral (IT) administration. Nonclinical experiments indicate that the drug rapidly diffuses into cells including into the nucleus with dispersion throughout tumors. Cell death, dendritic cell influx, antigen presentation and T-cell engagement result.

**Methods**

This phase 1/2 study evaluated five injections of INT230-6 into multiple superficial or deep tumors. Dose was determined by tumor volume with intrapatient dose escalation permitted. Patients were treated every two weeks with injections of up to 50% new fluid volume at each visit (potential for 200% additional fluid volume administered prior to the first tumor imaging assessment). Blood samples were analyzed by flow cytometry, and on study tumor samples analyzed by multiplex immunohistochemistry. Conventional CT/MRI tumor assessments were performed every 2 months.

**Results**

Thirty-eight heavily pretreated subjects (median 3 prior therapies (range 0-9)) have been dosed. Median age was 59 (42-76). Fourteen different cancer types were accrued. PK analysis revealed limited exposure of CIS and VIN in blood and supports that the majority of INT230-6’s active agents are retained in the tumor (consistent with low incidence of systemic AE’s despite IT doses of INT230-6 containing twice the amount of VIN for a standard 3.5 mg/m2 IV dose). There were no DLTs. The majority of drug-related AEs were grade 1/2. The most frequent treatment-related adverse event was injection-site pain (44%). Circulating CD8+ T-cells increased above baseline in most patients, with 40% of evaluable patients having a marked >30% increase. Paired biopsies were available in 5 patients and revealed >70% reduction in viable tumor cells in 3 out of 5 cases. Injected lesions demonstrated infiltration of CD4 and CD8 T-cells and reduction in FoxP3 Tregs. Several injected lesions showed asymptomatic tumor increases on the early scans (often with decreased contrast uptake), which decreased below baseline on subsequent scans while others showed tumor reduction already at the 2 month time point. The protocol suggested to continue treatment despite radiographic progression until clinical deterioration. Ten non injected tumors in 7 patients showed tumor shrinkage.

**Conclusions**

INT230-6 is well tolerated when administered intratumorally at doses given to date. Pharmacodynamic assessments support a notable reduction in viable cancer cells and increases in CD4/CD8 T-cell infiltrates. RECIST assessments may be compromised due to large drug-volume injections and immune cell infiltration. However, imaging suggests variable necrosis and tumor reductions in both injected and non-injected lesions.

**Trial Registration**

NCT 03058289

**Ethics Approval**

The study was approved by USC Institutution‘s Ethics Board, approval number HS-16-00843 as well as the other centers in the study.

#### O32 Single agent anti-tumor activity in PD-1 refractory NSCLC: phase 1 data from the first-in-human trial of NC318, a Siglec-15-targeted antibody

##### Anthony Tolcher^1^, Omid Hamid, MD^2^, Jeffrey Weber, MD, PhD^3^, Patricia LoRusso, DO^4^, Kathryn Shantz^5^, Kevin Heller, MD^5^ , Kevin Heller, MD^5^, Martin Gutierrez, MD^6^

###### ^*1*^*Next Oncology, San Antonio, TX, United States* ; ^*2*^*The Angeles Clinic, Los Angeles, CA, United States* ; ^*3*^*NYU Langone, New York, NY, United States* ; ^*4*^*Yale University, New Haven, CT, United States* ; ^*5*^*NextCure, Beltsville, MD, United States* ; ^*6*^*Hackensack University, Hackensack, NJ, United States*

####### **Correspondence:** Kevin Heller (hellerk@nextcure.com)

**Background**

Siglec-15 (S15) is a member of the Siglec family (Sialic acid-binding Immunoglobulin Lectins), a distinct subgroup of immunoglobulin (Ig) superfamily proteins involved in discriminating self and non-self-immune regulation [1]. Recent studies have shown that S15 mediates suppression of T cell proliferation and negatively regulates T cell function. The expression of S15 and PD-L1 are mutually exclusive in NSCLC and other cancers, which supports S15 as a potential target for the treatment of cancer patients who are refractory to PD-1 directed therapies. NC318 is a first-in-class monoclonal antibody that blocks S15-mediated immune suppression and prevents tumor growth by normalizing T cell function and restoring anti-tumor immunity in the tumor microenvironment [2].

**Methods**

NC318-01 is a multi-center, first-in-human, phase 1/2, open-label, non-randomized study to determine the safety and tolerability, define the maximum tolerated dose or pharmacologically active dose, and assess the preliminary efficacy of NC318. The phase 1 component uses a 3+3 dose escalation design to determine the recommended phase 2 dose (RP2D) of NC318. Primary endpoints include safety and tolerability. Secondary endpoints include assessment of pharmacokinetics, response rate (measured every 8 weeks), progression-free survival, and overall survival. Exploratory analyses include investigation of biomarkers associated with treatment benefit.

**Results**

As of August 2019, 43 patients have been dosed across 6 dose cohorts (8mg - 800mg Q2W). The most common tumor types enrolled included: 10 NSCLC, 7 ovarian, 6 melanoma, 3 breast, and 3 CRC. NC318 has been well tolerated with no DLTs. The most common treatment-related AEs included diarrhea (4- grade 1 and 2-grade 2), pruritis (3- grade 1 and 1- grade 2), rashes (grade 1 and 2), arthralgias (2- grade 1), elevated amylase (grade 1, 2, and 3), and elevated lipase (grade 3 and 4). Tumor responses were evaluable in 32 patients; 11 patients have not reached their first assessment, and their efficacy data will be reported at the conference. Single agent activity has been seen in NSCLC including 1 CR (ongoing at 41 weeks), a PR (ongoing at 14 weeks), 1 stable disease with tumor reduction (ongoing for 26 weeks), and 2 with stable disease. NSCLC BORR: 2/7 or 29%, DCR: 5/7 or 71%.

**Conclusions**

NC318 has been well tolerated across multiple dose levels and has shown encouraging anti-tumor activity when administered as monotherapy. Single agent anti-tumor activity was observed in 5 of 7 NSCLC subjects refractory to PD-1 therapies.

**Acknowledgements**

We would like to thank the patients and their families for participating in this clinical trial.

**Trial Registration**

Clinical trial information: NCT03665285

**References**

1. Macauley MS, Crocker PR, Paulson JC. Siglec-mediated regulation of immune cell function in disease. Nat Rev Immunol. 2014;14(10):653-666.

2. Wang J, Sun J, Liu, L, Flies D, Nie X, Toki M, et al. Siglec-15 as an immune suppressor and potential target for normalization cancer immunotherapy. Nat. Med. 2019; 25, 656–666.

**Ethics Approval**

Next Oncology’s Ethics Board, IntegReview, approved 18Sep18

Hackensack University Medical Center’s Ethics Board, WIRB, approved 4Oct2018

Angeles Clinic’s Ethics Board, WIRB, approved 11Nov18

NYU School of Medicine’s Ethics Board, approved 8Oct18

Yale University’s Ethics Board, WIRB, approved 25Apr19

#### O33 Immune enrichment and functional T-cell receptor (TCR) frequencies predict response to immune checkpoint blockade (ICB) in selected fusion-associated sarcomas

##### Akash Mitra, BS^1^, Neeta Somaiah, MD, MBBS^1^, Anthony Conley, MD^1^, Behrang Amini^1^, Heather Lin^1^, Beatriz Sanchez Espiridion^1^, Celia Garcia-Prieto^1^, Grace Mathew^1^, Latasha Little, BS^1^, Xingshi Song^1^, Curtis Gumbs, BS^1^, Cara Haymaker, PhD^1^, Jianhua Zhang^1^, Zachary Cooper, PhD^2^, Michael Oberst, PhD^2^, Chantale Bernatchez^1^, Vinod Ravi^1^, Dejka Araujo, MD^1^, Maria Zarzour^1^, John Livingston^1^, Christina Roland, MD, MS, FACS^1^, Najat Daw, MD^1^, Jaime Rodriguez-Canales, MD^3^, Alexander Lazar, MD, PhD^1^, Wei-Lien Wang^1^, Ignacio Wistuba, MD^1^, Jean-Charles Soria, MD, PhD^3^, Robert Benjamin^1^, Shreyaskumar Patel^1^, Patrick Hwu, MD^1^, Phillip Andrew Futreal, PhD^1^

###### ^*1*^*MD Anderson Cancer Center, Houston, TX, United States* ; ^*2*^*Oncology R&D , AstraZeneca, Gaithesburg, MD, United States* ; ^*3*^*Oncology R&D, AstraZeneca, Gaithersburg, MD, United States*

####### **Correspondence:** Phillip Andrew Futreal (afutreal@mdanderson.org)

**Background**

Metastatic sarcomas represent a significant therapeutic challenge considering the few systemic treatment options and suboptimal survivorship. Tumor mutational burden (TMB), tumor-infiltrating lymphocytes, and PD-L1 expression have been identified as biomarkers of response to ICB in certain cancers; however, their role in sarcoma remains limited. We conducted a Phase-II trial (NCT02815995) in pre-treated patients with metastatic sarcoma (mixed histologies) using durvalumab (anti-PD-L1) in combination with tremelimumab (anti-CTLA-4), and identified measures of diversity in the TCR in responders through molecular profiling.

**Methods**

We obtained tumor biopsies at baseline and after 6 weeks of treatment and performed whole-exome, TCR and RNA-sequencing, along with multiplexed-immunofluorescence (mIF). Preliminary analysis was conducted using alveolar soft part sarcoma (n=9) and synovial sarcoma (n=5) cohorts, comprised of 7 samples derived from responders and 7 samples from non-responders. TMB was calculated using mutations filtered against matched peripheral blood. TCR sequencing was conducted using high template producing samples. Differentially expressed genes (DEG) were assessed between responders and non-responders using baseline samples with adequate tumor purity. Further immune deconvolution was conducted using MCP-counter in baseline and on-treatment samples. mIF was conducted on a panel of T-cell and myeloid cell markers.

**Results**

TMB was not found to be predictive of response within these patients. Transcriptomic profiling through targeted immune gene assessment using MCP-counter revealed higher CD8 T-cell (p=0.0055) and NK-cell proportions (p=0.0086) in responders. Correspondingly, tumor shrinkage was found to be correlated with activated cytotoxic CD8+ T-cell density by mIF at baseline. The most significantly-enriched DEGs in responders at baseline included CCL20, CXCL10, CXCL11 and BTLA (p

**Conclusions**

Together, these results highlight the potential significance for multi-lineage immune cell enrichment containing high-frequency functional oligoclonal TCRs as prognostic and predictive factors for response to ICB in sarcoma.

**Trial Registration**

NCT02815995

**Ethics Approval**

The study was approved by MD Anderson's Ethics Board, protocol number 2015-1071.

#### O34 Phase II trial of neoadjuvant nivolumab (Nivo) and intra-tumoral (IT) CMP-001 in high risk resectable melanoma (MEL): Preliminary results

##### Diwakar Davar, MD^1^, Arivarasan Karunamurthy^2^, Douglas Hartman^2^, Mignane Ka^1^, Carmine Menna^1^, Julie Burkette^1^, Joe-Marc Chauvin, PhD^1^, Lucas Rubuffet^1^, Shuowen Zhang^1^, Quanquan Ding^1^, Amy Rose^1^, Kellie Sellitto^1^, Yana Najjar, MD^1^, Matthew Holtzman^1^, James Pingpank^1^, Shaum Sridharan^4^, Umamaheshwar Duvvuri^4^, Hagop Youssoufian^5^, Barry Labinger^5^, Arthur Krieg^5^, John Kirkwood, MD^1^, Hassane Zarour, MD^1^

###### ^*1*^*Hillman Cancer Center, Pittsburgh, PA, United States* ; ^*2*^*University of Pittsburgh, Pittsburgh, PA, United States* ; ^*3*^*University of Pittsburgh Medical Center, Pittsburgh, PA, United States* ; ^*4*^*Checkmate Pharmaceuticals, Boston, MA, United States*

####### **Correspondence:** Hassane Zarour (zarourhm@upmc.edu)

**Background**

Neoadjuvant PD-1 blockade produces major pathological responses (MPR) in ~30% of patients (pts) with high-risk resectable melanoma (MEL) with durable relapse-free benefit, and increased circulating activated CD8+ T cells [1,2]. CMP-001 comprises a CpG-A oligodeoxynucleotide packaged within a virus-like particle. It is designed to activate tumor-associated plasmacytoid dendritic cells (pDC) via TLR9 inducing an interferon-rich tumor microenvironment and anti-tumor CD8+ T cell responses. CMP-001 together with pembrolizumab resulted in objective, durable tumor responses with tolerable toxicities in PD-1 refractory MEL [3]. The goal of the present phase II study is to evaluate the pathological, clinical and immunological activities of Nivo and IT CMP-001 in the neoadjuvant setting in high-risk resectable MEL.

**Methods**

Pts with stage III B/C/D MEL were enrolled. Pre-operatively, CMP-001 was dosed at 5mg subcutaneous (SC, 1st), then 10mg IT (2nd-7th) weekly for 7 doses. Nivo was dosed 240mg q2weeks for 3 doses over a 7-week period. Post-operatively, Nivo was dosed 480mg q4weeks with CMP-001 5mg q4 weeks SC for 48 weeks. Primary endpoints included MPR rate, safety, and dose-limiting toxicities (DLT). Secondary endpoints were radiographic response, relapse-free survival (RFS) and overall survival (OS). Pathological response was assessed depending on residual volume of tumor (RVT) using prior cutoffs [4-6]: 0% (complete response, pCR); 0%<RVT<RVT50% (non-response, pNR). Radiographic response was assessed using RECIST v1.1. Sequential blood draws and tumor biopsies were collected. Tumor biopsies were evaluated for CD8+ T cell infiltrates and multiparameter flow cytometry evaluated circulating immune cells.

**Results**

20 pts have been enrolled, while 16 are evaluable for response. In 16 evaluable pts, disease sub-stage is IIIB (50%), IIIC (38%), IIID (12%). No DLTs were observed. Radiographic response seen in 50% (N=8), while 38% (N=6) had stable disease and 2 (13%) had progressive disease. MPR seen in 76% of pts with pCR 10 (63%) and pMR 2 (13%); while 1 pt (6%) had pPR and 3 pts (19%) had pNR. Responses to neoadjuvant therapy associated with increased CD8 T cell infiltrates and circulating PD1+/Ki67+ CD8+ T cells.

**Conclusions**

Neoadjuvant Nivo and IT CMP-001 has an acceptable toxicity profile and promising efficacy with 76% pCR/pMR in 16 evaluable pts which has prompted trial expansion. Median RFS is unreached in responders. Our findings suggest that Nivo and IT CMP-001 increases the clinical efficacy of PD-1 blockade alone with minimal additional toxicity in neoadjuvant MEL. Findings suggest that the proposed combination augments anti-melanoma CD8+ T cell immune responses in tumors and peripherally.

**Acknowledgements**

We thank Dr. Jagjit Singh and the pathology grossing room staff for their assistance and Checkmate Pharmaceuticals for funding and CMP-001.

**Trial Registration**

Clinical trial information: NCT03618641.

**References**

1. Amaria RN, Reddy SM, Tawbi HA, et al. Neoadjuvant immune checkpoint blockade in high-risk resectable melanoma. Nat Med. 2018 Nov;24(11):1649-1654.

2. Huang AC, Orlowski RJ, Xu X, et al. A single dose of neoadjuvant PD-1 blockade predicts clinical outcomes in resectable melanoma. Nat Med. 2019 Mar;25(3):454-461. doi: 10.1038/s41591-019-0357-y.

3. Milhem M, Gonzales R, Medina T, et al. Abstract CT144: Intratumoral toll-like receptor 9 (TLR9) agonist, CMP-001, in combination with pembrolizumab can reverse resistance to PD-1 inhibition in a phase Ib trial in subjects with advanced melanoma. In: Proceedings of the American Association for Cancer Research Annual Meeting 2018; 2018 Apr 14-18; Chicago, IL. Philadelphia (PA): AACR; Cancer Res 2018;78(13 Suppl):Abstract CT144.

4. Tetzlaff MT, Messina JL, Stein JE, et al. Pathological assessment of resection specimens after neoadjuvant therapy for metastatic melanoma. Ann Oncol. 2018 Aug 1;29(8):1861-1868.

5. Cottrell TR, Thompson ED, Forde PM, et al. Pathologic features of response to neoadjuvant anti-PD-1 in resected non-small-cell lung carcinoma: a proposal for quantitative immune-related pathologic response criteria (irPRC). Ann Oncol. 2018 Aug 1;29(8):1853-1860. doi: 10.1093/annonc/mdy218.

6. Stein JE, Soni A, Danilova L, et al. Major pathologic response on biopsy (MPRbx) in patients with advanced melanoma treated with anti-PD-1: evidence for an early, on-therapy biomarker of response. Ann Oncol. 2019 Apr 1;30(4):589-596. doi: 10.1093/annonc/mdz019.

**Ethics Approval**

The study was approved by University of Pittsburgh‘s Institutional Review Board, approval number MOD19040237-002.

**Consent**

Written informed consent was obtained from the patient for publication of this abstract and any accompanying images. A copy of the written consent is available for review by the Editor of this journal.

#### O35 Clinical activity of BEMPEG plus NIVO in previously untreated patients with metastatic melanoma: updated results from the phase 1/2 PIVOT-02 study

##### Adi Diab, MD^1^ , Igor Puzanov, MD, MSCI, FACP^2^, Michele Maio, MD, PhD^3^, Brendan Curti, MD^4^, Mehmet Bilen^5^, Karl Lewis, MD^6^, Scott Tykodi, MD, PhD^7^, Gregory Daniels, MD, PhD^8^, Alexander Spira, MD, PhD, FACP^9^, Chantale Bernatchez^1^, Salah Eddine Bentebibel^1^, Michael Wong, MD PhD FRCPC^1^, James Larkin, MD^10^, Ewa Kalinka, PhD, MD^11^, Sunny Xie^12^, Sue Currie^12^, Ute Hoch, PhD^12^, Wei Lin^12^, Mary Tagliaferri, MD^12^, Stina Singel^12^, Michael Hurwitz, MD, PhD^13^

###### ^*1*^*MD Anderson Cancer Center, Houston, TX, United States* ; ^*2*^*Roswell Park Cancer Institute, Buffalo, NY, United States* ; ^*3*^*Azienda Ospedaliera Universitaria Senese, Siena, Italy* ; ^*4*^*Providence Portland Medical Center, Portland, OR, United States* ; ^*5*^*Emory University Hospital, Atlanta, GA, United States* ; ^*6*^*University of Colorado, Denver, Aurora, CO* ; ^*7*^*Seattle Cancer Care Alliance, Seattle, WA, United States* ; ^*8*^*University of California, San Diego, La Jolla, CA, United States* ; ^*9*^*Virginia Cancer Specialists, Fairfax, VA, United States* ; ^*10*^*The Royal Marsden, London, United Kingdom* ; ^*11*^*Polish Mothers Memorial Hospital, Lodz, Poland* ; ^*12*^*Nektar Therapeutics, San Francisco, CA, United States* ; ^*13*^*Yale School of Medicine, New Haven, CT, United States*

####### **Correspondence:** Adi Diab (ADiab@mdanderson.org)

**Background**

Although checkpoint inhibitor(CPI) therapy has emerged as an effective treatment option for various cancers, there is an unmet need for therapies to produce more durable and deeper responses in metastatic melanoma. Safety and clinical activity of bempegaldesleukin(BEMPEG;NKTR-214), a CD-122 preferential IL-2 pathway agonist, plus the anti-PD1 CPI nivolumab(NIVO), was evaluated in PIVOT-02(NCT02983045), a multicenter phase 1/2 study in multiple solid tumor settings. At SITC2018, PIVOT-02 reported encouraging preliminary clinical activity and safety data in metastatic melanoma (ORR, 53%; CR, 24%)[1,2]. We plan to report updated results in 1L metastatic melanoma patients, and the first report of PFS.

**Methods**

41 patients with previously untreated stage IV metastatic melanoma received >1 dose of BEMPEG(0.006mg/kg) + NIVO(360mg) q3w. Patients were categorized by PD-L1 status. Response was assessed every 3 cycles by RECISTv1.1. Per protocol, ORR was evaluated in the efficacy-evaluable population (≥1 post-baseline scan) by independent central radiology review (N=38; 3 patients, non-efficacy-evaluable:1 unrelated treatment-emergent AE;2 patient decisions). Baseline immunohistochemistry(IHC) analysis for PD-L1 was performed (using Dako PD-L1 IHC 28-8 pharmDx) and defined as PD-L1 negative

**Results**

At a median follow-up of 12.7 months*, 38 patients were evaluable for efficacy. Table 1 shows BEMPEG plus NIVO was associated with clinical activity regardless of PD-L1 status. Confirmed ORR was 53% (20/38), and 34% (13/38) achieved a complete response. 42% (16/38) had 100% reduction in target lesions. Median time to response was 2 months, and median time to complete response was 7 months. Median duration of response was not reached (range:11mo-NR). BEMPEG plus NIVO was well tolerated, with TRAEs similar to those previously reported, with 14.6%(6) patients experiencing a ≥Grade 3 TRAE, and 9.8%(4) discontinuing treatment due to any TRAE. As of July 10, 2019, all 10 patients reported on treatment on March 29th, 2019 remain on treatment or achieved maximum response. At time of presentation, updated clinical results, including PFS, with ~18 months of follow-up will be reported.

**Conclusions**

BEMPEG plus NIVO is associated with robust clinical activity in 1L metastatic melanoma, as demonstrated by a high rate of durable responses that deepened over time. Based on these data, the FDA granted Breakthrough Therapy Designation for this combination therapy for patients with untreated unresectable or metastatic melanoma, and a Phase 3 trial evaluating the combination of BEMPEG plus NIVO vs NIVO alone in this setting is currently enrolling (NCT03635983).

**Trial Registration**

NCT02983045

**References**

1. Diab A, Tykodi S, Curti B, et al. Immune monitoring after NKTR‐214 plus nivolumab (PIVOT‐02) in previously untreated patients with metastatic stage IV melanoma oral presentation at SITC; November 7-11, 2018; Washington, DC, USA. Abstract #O4.

2. Hurwitz M, Cho DC, Balar, AV, et al. Baseline tumor-immune signatures associated with response to bempegaldesleukin (NKTR-214) and nivolumab. J Clin Oncol 2019;37:(suppl; abstr 2623).

**Ethics Approval**

The study was approved by each individual institution’s Ethics Board.

**Table 1 (abstract 035).**
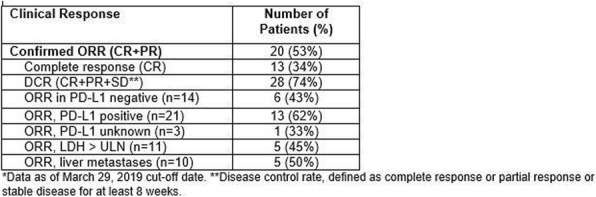
See text for description

#### O36 A phase I dose escalation and expansion study of intratumorally administered CV8102 as a single-agent or in combination with anti-PD-1 antibodies in patients with advanced solid tumors

##### Thomas Eigentler, Dr^1^, Juergen Krauss^2^, Jutta Schreiber, Dr^2^, Carsten Weishaupt^3^, Patrick Terheyden^4^, Lucie Heinzerling^5^, Peter Mohr^6^, Benjamin Weide, MD^7^, Sebastian Ochsenreither^8^, Juergen Becker, MD, PhD^9^, Franz-Georg Bauernfeind, Dr^10^, Peter Brossart^10^, Fatma Funkner^11^, Regina Heidenreich^11^, Sarah-Katharina Kays^11^, Anke Muth^11^, Tobias Seibel^11^, Birgit Scheel^11^, Oliver Schoenborn-Kellenberger^12^, Claudia Stosnach^11^, Angelika Daehling^11^, Tanja Strack^11^, Roman Korolkiewicz^11^, Ulrike Gnad-Vogt, MD^11^, Ulrike Gnad-Vogt, MD^11^, Ulrike Gnad-Vogt, MD^13^

###### ^*1*^*Universitätsklinikum Tübingen, Tuebingen, Germany* ; ^*2*^*NCT Heidelberg, Heidelberg, Germany* ; ^*3*^*Universitätsklinikum Muenster, Muenster, Germany* ; ^*4*^*Universitätsklinikum Schleswig Holstein, Lübeck, Germany* ; ^*5*^*Universitätsklinikum Erlangen, Germany* ; ^*6*^*Elbe Kliniken,Stabe, Germany* ; ^*7*^*Universitätsklinikum Tuebingen, Tuebingen, Germany* ; ^*8*^*Charite*, *Universitätsmedizin Berlin, Berlin, Germany* ; ^*9*^*Translational Skin Cancer Research, Essen, Germany* ; ^*10*^*Universitätsklinikum Bonn, Bonn, Germany* ; ^*11*^*CureVac AG, Tuebingen, Germany* ; ^*12*^*Cogitars, Heidelberg, Germany* ; ^*13*^*CureVav AG, Frankfurt, Germany*

####### **Correspondence:** Ulrike Gnad-Vogt (Ulrike.Gnad-Vogt@curevac.com)

**Background**

CV8102 comprises a non-coding RNA complexed with a cationic peptide signaling via TLR-7/-8 and RIG-I [1-2]. After intratumoral (IT) injection CV8102 was shown to activate T, NK cells at the injection site and draining lymph nodes. Dose-dependent antitumor activity and synergism with PD-1 blockade was shown in preclinical models (see abstract by Meister et al.).

The ongoing phase I trial is investigating IT CV8102 either as a single agent or in combination with systemic anti-PD-1 antibodies in patients (pts) with advanced melanoma (MEL), squamous cell carcinoma of the skin (cSCC) or head and neck (hnSCC) and adenoid cystic carcinoma (ACC).

**Methods**

Pts with MEL, cSCC, hnSCC or ACC not amenable to curative therapy are eligible for single agent CV8102. Pts with MEL or hnSCC indicated for anti-PD-1 therapy or stable/slowly progressing disease after ≥12 weeks of anti-PD-1 are eligible for the combination therapy.

Eight CV8102 IT injections are being administered over 12 weeks into superficial injectable lesions, while patients benefiting from the single agent therapy may receive further treatments. In an initial dose escalation part the maximum tolerated and recommended single agent/combination-doses (MTD, RD/RCD) will be defined. Tumor response according to RECIST 1,1/irRECIST and immune responses in the peripheral blood and tumor tissue (optional) are being assessed. After determination of the RD/RCD, expansion cohorts are planned in several indications.

**Results**

As of July 01, 2019, 18 pts have been treated with single agent CV8102 (25-300 μg) and 10 pts have received CV8102 (25-200 μg) in combination with anti-PD-1 antibodies. No dose limiting toxicities occurred so far and the majority of treatment emergent AEs were mild to moderate injection site reactions and flu-like symptoms.

After single agent CV8102 treatment one MEL pt achieved a complete response of injected and non-injected lesions and seven pts stable disease including two pts showing regressions of non-injected lymph node lesions (1 hnSCC and 1 MEL pt). Dose escalation is continuing and updated safety, efficacy and immune response data of the single agent and combination cohorts will be presented.

**Conclusions**

IT CV8102 single-agent and in combination with systemic anti-PD-1 antibodies appears well tolerated to date and showed evidence of single agent activity with shrinkage of injected and non-injected lesions. Updated results will be presented.

**Trial Registration**

NCT03291002

**References**

1. Ziegler A, Soldner C, Lienenklaus S, Spanier J, Trittel S, Riese P, Kramps T, Weiss S, Heidenreich R, Jasny E, Guzmán CA, Kallen KJ, Fotin-Mleczek M, Kalinke U. A New RNA-Based Adjuvant Enhances Virus-Specific Vaccine Responses by Locally Triggering TLR- and RLH-Dependent Effects. J Immunol. 2017;198(4):1595-1605. doi: 10.4049/jimmunol.1601129

2. Heidenreich R, Jasny E, Kowalczyk A, Lutz J, Probst J, Baumhof P, Scheel B, Voss S, Kallen KJ, Fotin-Mleczek M. A novel RNA-based adjuvant combines strong immunostimulatory capacities with a favorable safety profile. Int J Cancer. 2015 Jul 15;137(2):372-84. doi: 10.1002/ijc.29402

**Ethics Approval**

The study was approved by the Central Ethics Committee in Tuebingen, Germany under 785/2016AMG1

**Consent**

Written informed consent was obtained from the patient for publication of this abstract and any accompanying images. A copy of the written consent is available for review by the Editor of this journal

### Combination Immunotherapies

#### O37 PD1 blockade augments adoptive T cell therapy via endogenous T cells rather than direct enhancement of transferred T cells

##### John Davies, PhD, Farrah Karimipour, BS, Ling Zhang, PhD, Carylinda Serna, Christian Hinrichs, MD

###### NIH/NCI, Bethesda, MD, United States

####### **Correspondence:** Christian Hinrichs (hinrichs@nih.gov)

**Background**

The use of adoptive T cell therapy (ACT) has yielded promising clinical results, but improvements are needed. Transferred T cells experience prolonged antigen exposure during ACT and efficacy might improve if combined with PD1 axis blockade. However, limited data combining ACT with PD1 axis blockade has been published.

**Methods**

To assess whether ACT was improved with the combination of PD1 axis blockade, antigen-tagged retroviral constructs were used to generate B16-OVA and B16-gp33 tumors. These tumors were targeted by OT-I and P14 TCR transgenic T cells, respectively. After implanted tumors became palpable, mice underwent total body irradiation lymphodepletion and were subsequently treated with T cells and/or anti-PD1 antibody.

**Results**

Two independent T cell models demonstrated that ACT combined with anti-PD1 showed improved treatment compared to either therapy alone. To isolate the role of transferred T cells, mice lacking adaptive immunity (Rag1-KO) were utilized as tumor-bearing hosts. The efficacy of anti-PD1 as a monotherapy and the added benefit when combined with ACT were abolished in Rag1-KO hosts. These data were corroborated in mice specifically devoid of alpha-beta T cells (Trac-KO) as well as in mice containing T cells with a restricted TCR repertoire (irrelevant TCR transgenic). Antibody blockade has the confounding property of neutralizing PD1 systemically, altering signaling of the PD1 axis on both transferred cells and endogenous cells. The intrinsic role of the PD1 pathway on transferred T cells was further interrogated by engineering T cells with PD1-targeting miRNA or over-expression of PD1 without the cytoplasmic signaling domain. Both methods of intrinsic disruption of the PD1 pathway provided no added benefit to ACT in tumor-bearing wild-type animals. Another method of demonstrating that anti-PD1 activity was acting via endogenous T cells was utilizing PD1-KO T cells for ACT. PD1-KO T cell transfer combined with anti-PD1 conferred an added treatment benefit compared to either therapy alone. Moreover, endogenous T cells in the tumor increased in number after anti-PD1 treatment, while transferred T cells remained constant. Lastly, the efficacy of anti-PD1 therapy was lost when treating less immunogenic B16 tumors that were engineered with ubiquitin-tagged antigen constructs, rather than RFP-antigen constructs. This finding demonstrated that PD1-sensitive endogenous T cells were targeting antigens within RFP.

**Conclusions**

These data demonstrate that blocking the PD1 axis does not directly benefit adoptively transferred T cells. Rather, the benefit of anti-PD1 is dependent on the presence of endogenous T cells specific for immunogenic tumor antigens.

#### O38 A single-dose immunotherapy that remodels the tumor microenvironment for subsequent curative responses to checkpoint blockade

##### Chensu Wang, PhD^1^, Ang Cui^2^, Ayush Thomas^1^, Simon Liang^1^, Maurice Bukenya^1^, Heikyun Suh^1^, K. Dane Wittrup, PhD^1^, Nir Hacohen, PhD^2^, Darrell Irvine, PhD^1^

###### ^*1*^*Massachusetts Institute of Technology, Cambridge, MA, United States* ; ^*2*^*Broad Institute of MIT and Harvard, Cambridge, MA, United States*

####### **Correspondence:** Darrell Irvine (djirvine@mit.edu)

**Background**

Given that immune checkpoint blockade (ICB) therapy only benefits a subset of cancer patients, much effort is currently being invested in the search for combination treatments that could increase the “visibility” of tumors to the immune system in order to sensitize them to ICB therapy[1]. We previously reported an efficacious immunotherapy termed AIPV [2], containing a tumor-targeting antibody, half-life-extended IL-2, anti-PD-1 and a lymph-node-targeting vaccine. Here we show that a minimalist “priming” therapy derived from AIPV, administered once, rapidly remodels the tumor microenvironment and initiates an immune attack that leads to tumor eradication upon subsequent ICB therapy in multiple mouse tumor models.

**Methods**

To define the minimal AIPV-derived “priming” therapy, we explored all possible sub-combinations of the four components of AIPV and verified the result in a several mouse tumor models. To be broadly relevant for clinical translation, we substituted the tumor-specific antibody in AIPV to a pan-tumor-targeting antibody surrogate 2.5F-Fc, which recognizes integrins that are overexpressed by many human and murine tumors. We further analyzed tumor antigen release, tumor antigen uptake by dendritic cells (DCs), CD8+ T cell activation by DCs in tumor-draining lymph nodes (TDLNs), and the number of immune infiltrates in therapy-primed B16F10 tumors.

**Results**

We identified AIP as the minimally required combination to prime responsiveness to ICB treatment, which successfully cured more than 75% of large established syngeneic mouse tumors, including aggressive B16F10 melanoma and lung cancer models that harbor low tumor mutational burden and fail to respond to ICB therapy alone. In mechanistic studies, we found that compared to untreated and ICB-treated tumors, AIP-primed tumors showed higher levels of tumor cell death, which triggered more cross-presenting DCs to take up tumor antigens and activate CD8+ T cells in TDLNs, preceding a later wave of T cell infiltration. Moreover, the therapeutic benefit resulted from not only effector T cells but also innate immune cells, especially NK cells and macrophages that contributed to the initial tumor cell killing. Intriguingly, we found that NK cells secreted XCL1 and FLT3L to recruit and maintain cross-presenting DCs in the tumor, and depletion of NK cells completely abolished AIP-induced increases in the number of intratumoral cross-presenting DCs and activated T cells.

**Conclusions**

In summary, we identified a minimalist “priming” therapy that successfully synergizes with subsequent ICB treatment. Mechanistic studies indicate that the engagement of innate immune cells to initiate the “cancer-immunity-cycle” and expand cross-presenting DCs is important to the success of this combination therapy.

**Acknowledgements**

We thank the Koch Institute Swanson Biotechnology Center for technical support, specifically the applied therapeutics & whole animal imaging core facility, histology, microscopy and flow cytometry core facility.

**References**

1. Ribas A, Wolchok JD. Cancer immunotherapy using checkpoint blockade. Science. 2018;359(6382):1350-5.

2. Moynihan KD, Opel CF, Szeto GL, Tzeng A, Zhu EF, Engreitz JM, Williams RT, Rakhra K, Zhang MH, Rothschilds AM. Eradication of large established tumors in mice by combination immunotherapy that engages innate and adaptive immune responses. Nat. Med. 2016;22(12):1402.

#### O39 Soluble TNFα induced mucin 4 is a mediator of trastuzumab resistance and of an immunosuppressive tumor microenvironment in HER2+ breast cancer

##### Sofia Bruni, MS, Mara De Martino, PhD, Florencia Mauro, MS, Lucia Santa Maria de la Parra, Patricia Elizalde, PhD, Roxana Schillaci

###### Instituto de Biologia y Medicina Experimental - CONICET, Buenos Aires, Argentina

####### **Correspondence:** Roxana Schillaci (roxanaschillaci@gmail.com)

**Background**

About 13-20% of breast cancer (BC) patients are HER2 positive (HER2+) and receive trastuzumab (T), an anti-HER2 monoclonal antibody, but 40-60% of them relapse. We have demonstrated that tumor necrosis factor alpha (TNFα) induces the expression of the transmembrane glycoprotein mucin 4 (MUC4), that shields T epitope in HER2, impairing its antitumor effects. Also, we have shown that Etanercept (E), an inhibitor of soluble and transmembrane TNFα (sTNFα, tmTNFα), downregulated MUC4 expression and sensitized de novo T-resistant BC xenografts to T [1]. The aim of this work was to study the participation of MUC4 on T resistance in vivo, on T-mediated antitumor innate immune response, and to evaluate the role of sTNFα on MUC4 expression.

**Methods**

We used the T-resistant JIMT-1 cell line transduced with a doxycycline (Dox)-inducible MUC4 shRNA construct (JIMT-shMUC4). To block TNFα, we used E or the dominant negative-TNFα protein INB03 (DN). Nude mice bearing JIMT-1-shMUC4 tumors (~50 mm3), were randomly assigned to the experimental (+Dox 2mg/ml in drinking water) or control group (−Dox). Both groups were treated with IgG, T, E (all 5 mg/kg), DN (10 mg/kg), T+E or T+DN i.p. twice a week and tumor volume was monitored routinely. MUC4 expression was determined by Western Blot in tumor extracts. Tumor-infiltrating immune cells were evaluated by immunofluorescence and analyzed by flow cytometry.

**Results**

In control groups, only T+E and T+DN administrations were able to inhibit tumor growth (72% and 75%, respectively, P

**Conclusions**

This is the first report to show that sTNFα is responsible for MUC4 regulation and that MUC4 is the major player in TNFα-induced T resistance in vivo. In addition, MUC4 favors an immunosuppressive tumor microenvironment. We propose that patients with HER2+ and MUC4+ tumors should be treated with T and TNFα-blocking agents to avoid resistance.

**References**

1. Mercogliano MF, De Martino M, Venturutti L, Rivas MA, Proietti CJ, Inurrigarro G, Frahm I, Allemand DH, Gil Deza E, Ares S, Gercovich FG, Guzmán P, Roa JC, Elizalde PV, Schillaci R. TNFα-induced Mucin 4 Expression Elicits Trastuzumab Resistance in HER2-Positive Breast Cancer. Clin Cancer Res. 2017; 23:636-648.

**Ethics Approval**

The study was approved by IACUC from Instituto de Biologia y Medicina Experimental‘s Ethics Board, approval number 25/2017.

#### O40 Immunobiology and clinical activity of CPI-006, an anti-CD73 antibody with immunomodulating properties in a phase 1/1b trial in advanced cancers

##### Jason Luke, MD, FACP^1^, Jaime Merchan^2^, Lauren Harshman, MD^3^, Thomas Marron, MD PhD^4^, John Powderly, MD, CPI^5^, Minal Barve^6^, Patricia LoRusso, DO^7^, Melissa Johnson^8^, Andrew Hotson, PhD^9^, Rachel Gittelman, PhD^10^, Brian Munneke^9^, Joseph Buggy^9^, Stephen Willingham, PhD^9^, Emily Piccione, PhD^9^, Mehrdad Mobasher^9^, Richard Miller, MD^9^

###### ^*1*^*UPMC, Pittsburgh, PA, United States* ; ^*2*^*University of Miami, Miami, FL, United States* ; ^*3*^*Dana-Farber Cancer Institute, Boston, MA, United States* ; ^*4*^*Icahn School of Medicine at Mount Sinai, New York, NY, United States* ; ^*5*^*Carolina BioOncology Institute, Huntersville, NC, United States* ; ^*6*^*Mary Crowley Cancer Research Center, Dallas, TX, United States* ; ^*7*^*Yale University, New Haven, CT, United States* ; ^*8*^*Sarah Cannon Research Institute, Nashville, TN, United States* ; ^*9*^*Corvus Pharmaceuticals Inc, Burlingame, CA, United States* ; ^*10*^*Adaptive Biotechnologies, Seatle, WA, United States*

####### **Correspondence:** Richard Miller (rmiller@corvuspharma.com)

**Background**

CPI-006, a humanized IgG1 Fc gamma receptor binding-deficient anti-CD73 antibody blocks CD73 enzyme activity and has adenosine independent, immunomodulatory activity including activation of lymphocytes (expression of CD69, CD83, CD25), effects on T and B cell trafficking, and increases in expression of markers on antigen presenting cells (CD86, HLA-DR)[1]. We report immunologic, safety and preliminary efficacy data from a Phase 1/1b study, with CPI-006 monotherapy and combination with ciforadenant, an adenosine-A2A receptor (A2AR) antagonist (NCT03454451).

**Methods**

Dose escalation/expansion design with CPI-006 monotherapy (IV, q21 days) and combination with ciforadenant (100mg oral BID). Eligibility included patients with cancers progressed on standard therapies. Blood was evaluated for lymphocyte subsets and IgH sequencing was performed to assess B cell receptor (BCR) clonality; tumor biopsies evaluated by immunohistochemistry.

**Results**

25 patients with median 4 prior therapies enrolled (15 monotherapy, doses 1mg/kg to 18mg/kg; 10 combination, 1mg/kg to 12mg/kg). No DLT was observed and MTD not reached. Most common adverse events Gr 1/2 nausea, fatigue and infusion reactions. PBMC CD73 occupancy by CPI-006 for >21 days was seen at ≥ 6mg/kg (occupancy dose; OD, N=12 patients) and blockade of enzyme activity of CD73 in tumor was achieved at 12mg/kg. In blood of all 11 evaluable patients treated at ≥ OD, there was reduction in circulating CD73+ B cells (median −64%) at 0.5 hr with partial recovery by day 21. Returning B cells demonstrated upregulated HLA-DR with the proportion of memory B cells expanded (median +40% in CD27+, IgD−). No change in serum immunoglobulins was seen. Circulating T, NK cells and monocytes were reduced at 0.5 hr but recovered by 24 hrs. Memory CD4 T cell numbers in blood increased >20% in 5 of 10 patients tested with three of those patients showing reduction in tumor. Tumor regression was observed in 4 of 8 evaluable patients at ≥ OD (Figure 1) including best overall response of −28% and −21% in 2 patients with RCC and −18% in mCRPC. BCR analysis showed that treatment induced the generation and expansion of new B cell clones in blood of 2 of 3 patients studied including one of the responding RCC patients.

**Conclusions**

CPI-006 is an anti-CD73 antibody with immunomodulatory activity. Activation markers and changes in circulating lymphocytes are consistent with trafficking to nodal sites and sensitization of T and B cells. Preliminary evidence of anti-tumor activity has been observed without enrichment for tumor CD73 expression or adenosine gene signature.

**References**

1. Luke JJ, Powderly JD, Merchan JR, Barve MA, Hotson AN, Mobasher M, Kwei L, Luciano G, Buggy JJ, Piccione E, Miller RA. Immunobiology, preliminary safety, and efficacy of CPI-006, an anti-CD73 antibody with immune modulating activity, in a phase 1 trial in advanced cancers. J Clin Oncol. 2019;37 (suppl; abst 2505).

**Ethics Approval**

The study was approved by Western IRB, approval number 1-1066703-1.

**Fig. 1 (abstract 040).**
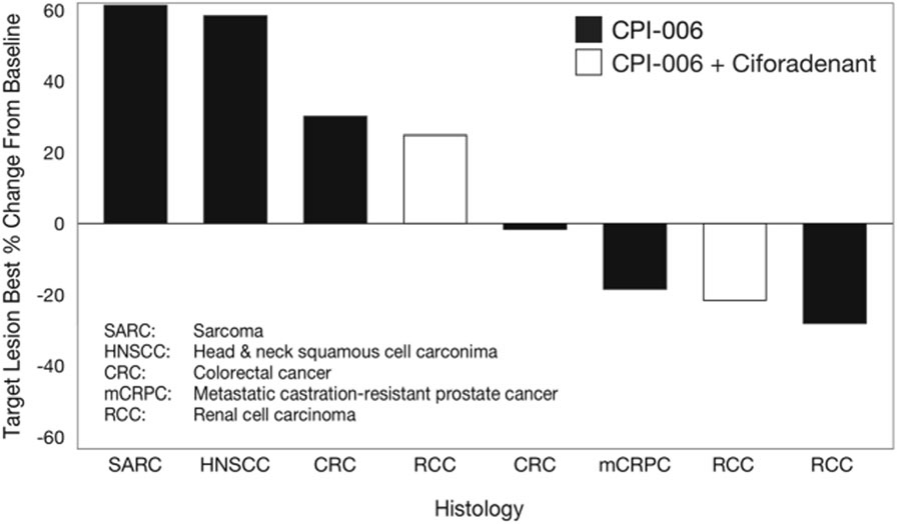
See text for description

#### O41 Vasoactive intestinal peptide: a novel checkpoint pathway in pancreatic ductal adenocarcinoma

##### Sruthi Ravindranathan, PhD, Shuhua Wang, MD, Brandon Ware, Mohammad Zaidi, MD, MS, Jingru Zhu, Rohan Dhamsania, BS, Susan Thomas, PhD, BS, Yiwen Li, MS, Bassel El-rayes, MD, Gregory Lesinski, PhD, MPH, Edmund Waller, MD, PhD, FACP

###### Emory University, Atlanta, GA, United States

####### **Correspondence:** Sruthi Ravindranathan (sravin5@emory.edu)

**Background**

Pancreatic ductal adenocarcinoma (PDAC) largely fails to respond to FDA-approved immunotherapy, with a 5-year survival rate of only 5-7 % [1]. Intriguingly, according to the cancer genome atlas (TCGA), human pancreatic cancers have exceptionally high levels of vasoactive intestinal peptide (VIP) mRNA expression in the tumor (Figure 1A). VIP is an immunosuppressive neuropeptide which when added to activated T cells decreases their proliferation and polarizes them towards the Th2 phenotype [2]. Thus, we hypothesized that VIP plays a crucial role in immune paralysis associated with PDAC and that pharmacologically inhibiting VIP signaling could enhance treatment response in PDAC tumors.

**Methods**

Levels of VIP in cell culture supernatant and serum of human or mouse blood was detected using VIP specific enzyme immunoassay. Subcutaneous or orthotopic mouse PDAC models were generated by injecting 1 million murine PDAC cells, MT5 or luciferase transfected KPC (KPC.luc), near the flank or on a matrigel into the tail of pancreas of immunocompetent C57BL/6 mice, respectively. Peptide VIP antagonists, ANT-00 or ANT-08 (more potent version) were subcutaneously administered every day to inhibit VIP signaling in-vivo followed by intraperitoneal administration of anti-PD1 or rat IgG2a (isotype) every 3 days for a duration of 10 days. Tumor growth was monitored by measurements with Vernier calipers or via IVIS imaging and in some cases confirmed with 9.4T MRI imaging. Immunological differences between the treatment groups were determined via flow cytometry and/or immunofluorescence staining of spleen or tumor.

**Results**

Our results show that peripheral blood of untreated human pancreatic cancer patients have significantly higher levels of VIP when compared to healthy volunteers (Figure 1B). Similarly, mouse PDAC cell lines also produce significantly high levels of VIP both in-vitro and in-vivo (Figure 1C&D). Treatment with ANT-00+anti-PD1 in subcutaneous MT5 and KPC models, resulted in significant improvement in median survival that was associated with increased CD8+ T cell infiltration into the TME (Figure 2). The treatment strategy also resulted in significantly reduced tumor burden in the orthotopic mouse models of PDAC (Figure 3). Additionally, splenocytes from ANT-08+anti-PD1 treated mice with orthotopic PDAC tumors had more IFN gamma-secreting CD8+ T cells and lower numbers of myeloid derived suppressor cells and plasmacytoid dendritic cells, when compared to control group (Figure 4).

**Conclusions**

Expression of high levels of VIP is a targetable mechanism of immune escape in PDAC. Inhibiting VIP signaling along with checkpoint blockade significantly improves treatment response in murine PDAC models.

**Acknowledgements**

Emory Core Laboratories: Center for Systems Imaging and Research Pathology Core for their expertise in mouse MRI imaging and mouse tissue sectioning and staining, respectively.

**References**

1. Rahib L, Smith BD, Aizenberg R, Rosenzweig AB, Fleshman JM, Matrisian LM: Projecting cancer incidence and deaths to 2030: the unexpected burden of thyroid, liver, and pancreas cancers in the United States. Cancer Res 2014, 74(11):2913-2921.

2. Ganea D, Hooper KM, Kong W: The neuropeptide vasoactive intestinal peptide: direct effects on immune cells and involvement in inflammatory and autoimmune diseases. Acta Physiol (Oxf) 2015, 213(2):442-452.

**Fig. 1 (abstract 041).**
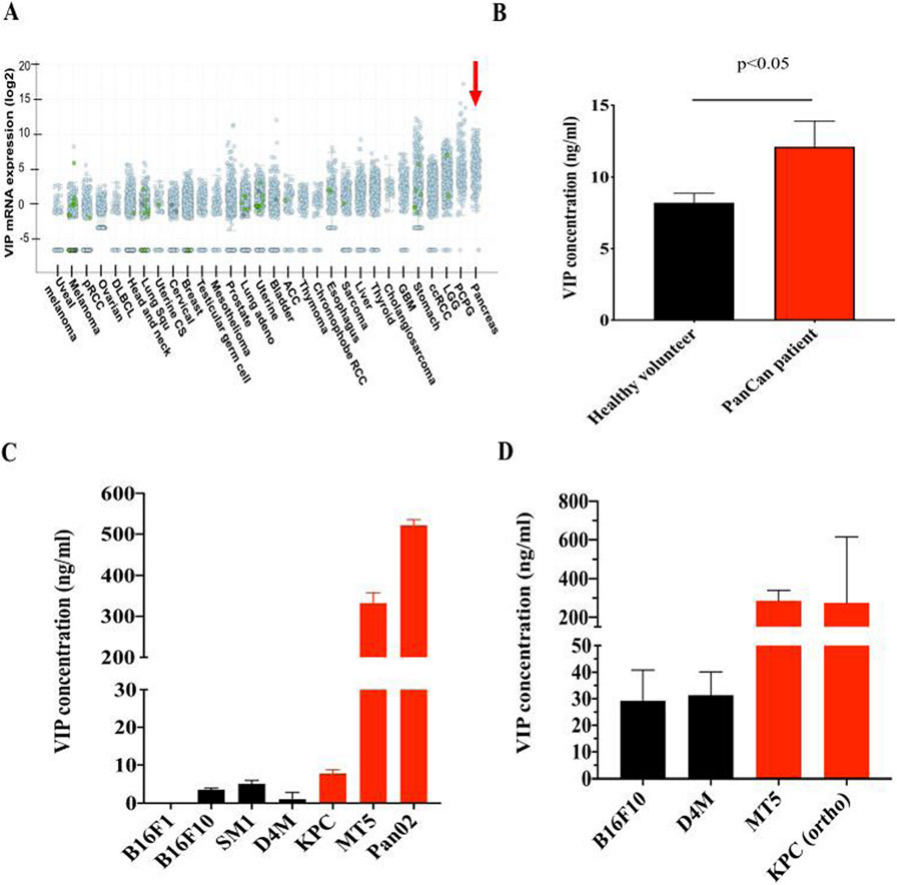
Elevated VIP levels in human and mouse pancreatic cancer.

**Fig. 2 (abstract 041).**
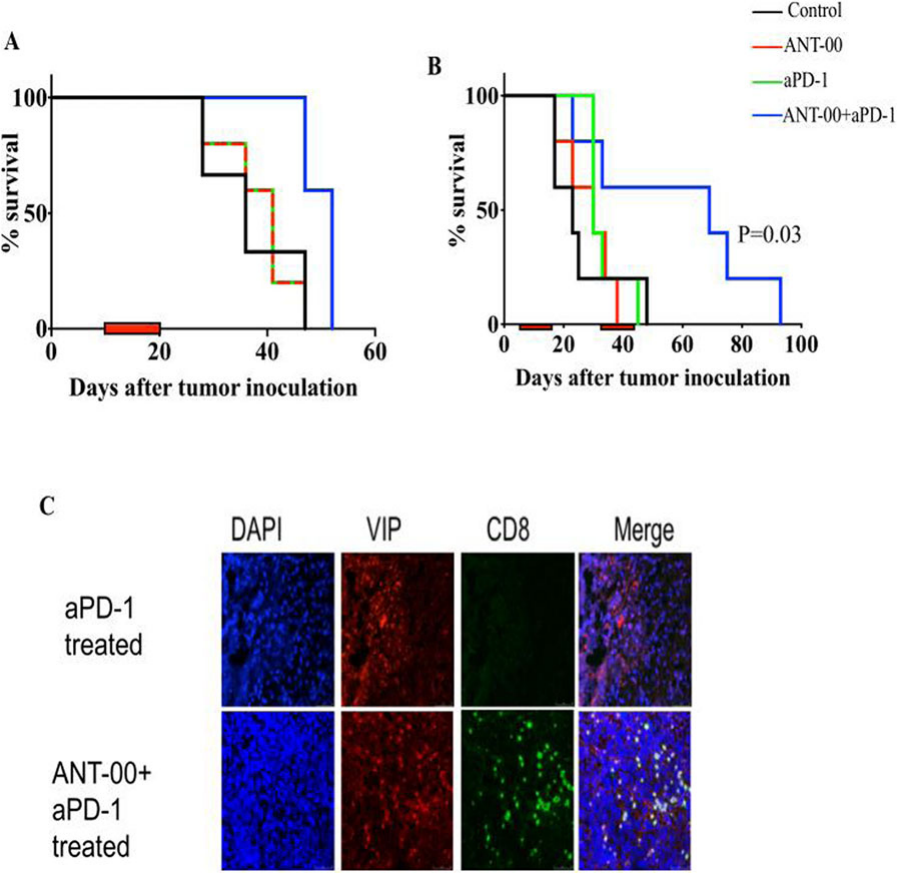
Enhanced survival via CD8+ T cell infiltration

**Fig. 3 (abstract 041).**
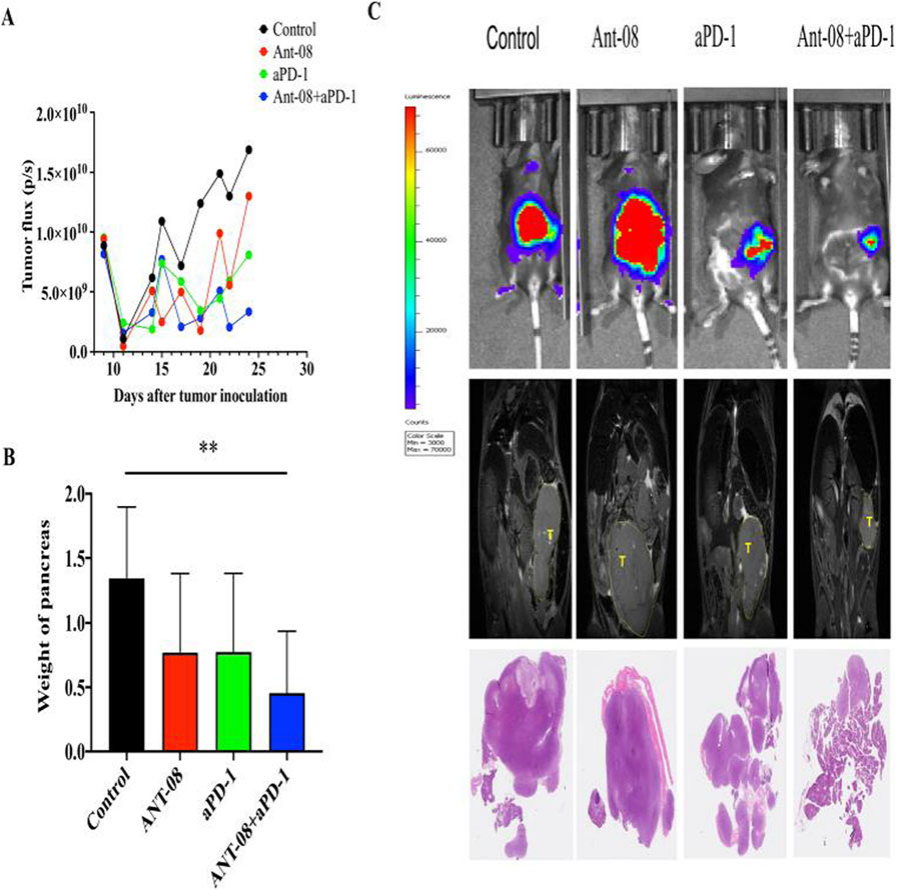
Decreased tumor growth and burden in orthotopic PDAC model

**Fig. 4 (abstract 041).**
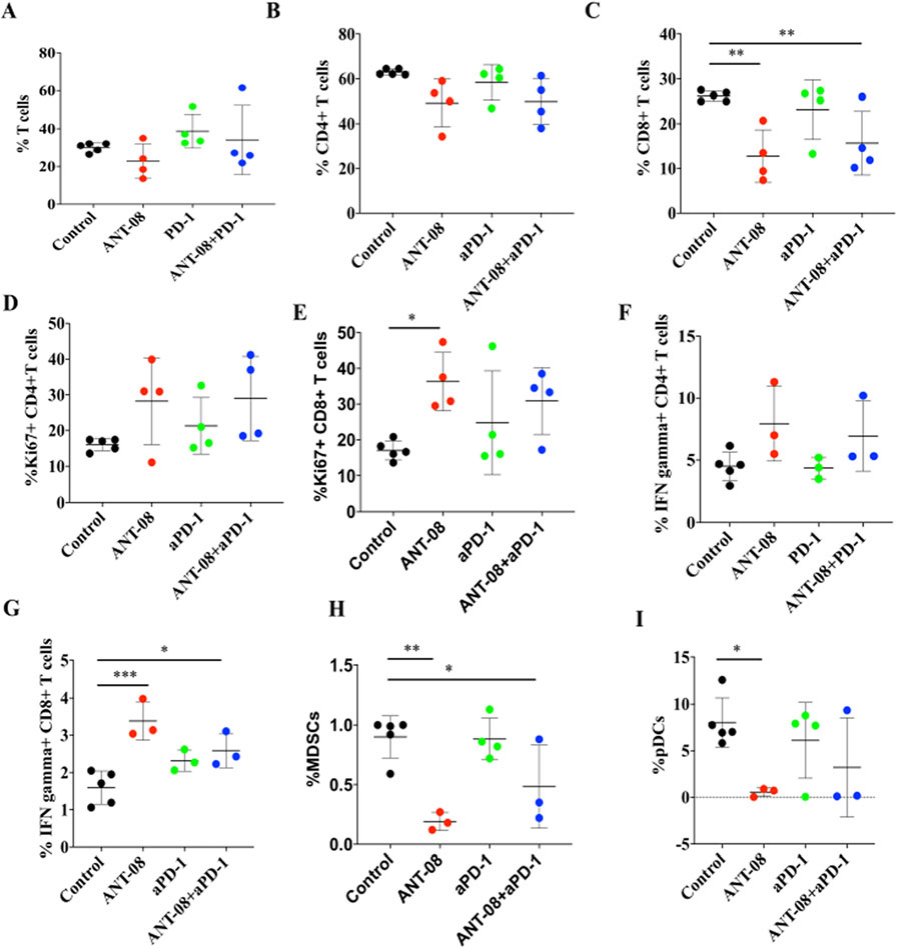
Analysis of immune cell populations in spleen

### Immune Cell Biology

#### O42 Transcriptional dissection reveals antitumor role of T follicular helper cells in head and neck cancer

##### Anthony Cillo, PhD, Dario Vignali, PhD

###### University of Pittsburgh, Pittsburgh, PA, United States

####### **Correspondence:** Dario Vignali (dvignali@pitt.edu)

**Background**

Head and neck squamous cell carcinoma (HNSCC) is caused by either infection with human papillomavirus (HPV+) or tobacco use (HPV-). Immunotherapy for metastatic or recurrent head and neck squamous cell carcinoma (HNSCC) is FDA-approved regardless of HPV status, but only ~15-20% of patients achieve a clinical response [1-2]. Many targets for CD8+ T cells are being investigated for clinical efficacy [3], but the potential of other cell types to enhance antitumor immunity has yet to be fully realized.

**Methods**

Single-cell RNAseq (scRNAseq) libraries (10X Genomics, 3’ v2) were generated by sorting CD45+ cells (i.e. all immune populations) from peripheral blood mononuclear cells (PBMC) and tumor infiltrating leukocytes (TIL) from immunotherapy naïve HNSCC patients. CD45+ cells from healthy donor PBMC and tonsil tissues from sleep apnea patients undergoing tonsillectomy were also used for scRNAseq. Clustering, differential gene expression analysis, and gene set enrichment analysis were used for scRNAseq analysis. Seven-color immunofluoresence (IF) was performed on the same tissues used for scRNAseq. A Kolmogorov-Smirnov test was used to determine enrichment scores from bulk mRNAseq from The Cancer Genome Atlas (TCGA), and log-rank tests were used for survival analyses.

**Results**

We identified 132,302 single cells from 6 healthy donor PBMC, 5 tonsils, 18 paired PBMC/TIL from HPV- patients, and 9 paired PBMC/TIL from HPV+ patients. Interestingly, B cells, CD4+ Tconv, and myeloid cells had divergent transcriptional profiles between HPV- and HPV+ TIL, while CD8+ T cells and CD4+ Treg were relatively similar. In HPV+ HNSCC, CD4+ Tconv expressed a T follicular helper (TFH) signature (including co-expression of CXCR5, PDCD1, ICOS and ASCL2), while those in HPV- HNSCC predominantly had an effector-memory signature. We next used the TFH signature derived from scRNAseq to generate a TFH enrichment score for HNSCC patients in TCGA. This analysis revealed better progression-free survival (PFS) in patients with a high versus low TFH signature (hazard ratio=0.39; p=0.0098). In multivariate analysis controlling for 9 covariates, high TFH signatures remained associated with better PFS (hazard ratio=0.041, p=0.02). Consistent with the scRNAseq data, IF analysis revealed co-localization of CD4+ Tconv and B cells in tertiary lymphoid structures, suggesting a functional role for TFH in HNSCC.

**Conclusions**

Our “top-down” single-cell transcriptional analysis of immune cells in HNSCC reveals an unappreciated hierarchy of immune heterogeneity. Immunotherapeutics should be tailored to unique properties of the tumor microenvironment, such as boosting the function of CD4+ TFH in HPV+ HNSCC.

**Acknowledgements**

We thank the Hillman Cancer Center Flow Cytometry Core for assistance with cell sorting; the University of Pittsburgh Heath Science Genomics Research Core for assistance with sequencing; the University of Pittsburgh Center for Research Computing for computational resources; and the University of Colorado Denver Human Immune Monitoring Shared Resource for assistance with IF imaging. We thank the University of Pittsburgh Cancer Immunology Training Program (T32 CA082084) and the Hillman Postdoctoral Fellowship for Innovative Cancer Research for funding support.

**References**

1. Ferris RL, et al. Nivolumab for Recurrent Squamous-Cell Carcinoma of the Head and Neck. N Engl J Med. 2016; 375:1856-1867.

2. Chow LQ, et al. Antitumor activity of pembrolizumab in biomarker-unselected patients with recurrent and/or metastatic head and neck squamous cell carcinoma: results from the phase Ib KEYNOTE-013 expansion cohort. J Clin Oncol. 2016; 34:3838-3845.

3. Tang J, Shalabi A, Hubbard-Lucey VM. Comprehensive analysis of the clinical immuno-oncology landscape. Ann Oncol. 2018; 29:84-91.

**Ethics Approval**

This study was approved by the local Institutional Review Board under protocol UPCI 99-069, and patients provided informed consent.

#### O43 Immune profiling of the tumor microenvironment in classic Hodgkin lymphoma using high-complexity mass cytometry is feasible and reveals significant multi-compartment heterogeneity between patients

##### El-ad David Amir, PhD^1^, Jose Villasboas, MD^2^, Kaitlyn McGrath, MS^2^, Stephen Ansell, MD, PhD^2^

###### ^*1*^*Astrolabe Diagnostics, Inc., Fort Lee, NJ, United States* ; ^*2*^*Mayo Clinic, Rochester, MN, United States*

####### **Correspondence:** Jose Villasboas (Villasboas@mayo.edu)

**Background**

Classic Hodgkin lymphoma (cHL) is a germinal-center derived B-cell lymphoma which is rich in non-malignant T cell infilitrates [1]. The malignant cHL cells manipulate the tumor microenvironment, which in turn has the potential to be used as a prognostic marker and therapeutic tool [2,3,4]. Mass cytometry (CyTOF) is a high-complexity cytometry technology that has been successfully applied to immune profiling in cancer contexts [5,6,7]. We used mass cytometry to profile the cHL tumor microenvironment in a cohort of eight cHL patients and seven non-malignant lymphoid tissue controls.

**Methods**

Eight viably cryopreserved single-cell suspension samples from tumor biopsies at time of diagnosis were obtained from the prospective lymphoma biobank at the Mayo Clinic, Rochester. Seven similarly prepared de-identified samples from patients with non-malignant diagnoses (four tonsils and three lymph nodes) were used as controls. Samples were stained with a 31-marker surface immunophenotyping panel which was designed to detect all major immune compartments with an emphasis on further characterization T cells. Four cHL and four control samples had additional markers added to their panel (PDL1 and CD30) in order to identify the cHL malignant cells. Samples were acquired using a CyTOF2 instrument. Resulting data was analyzed using the Astrolabe Cytometry Platform (a standardized analysis pipeline) and additional R code, including t-SNE and follow-up statistics.

**Results**

The combination of high-complexity cytometry with a standardized analysis pipeline reveals an accurate profile of the immune system (Figure 1:panels A, B). Cell subset phenotypes and frequencies vary dramatically between the cHL patients and controls (Figure 1:panels C, D). Specifically, the method recapitulated known trends where the B cell compartment shrinks in cHL while the T cell compartment expands [1] (Figure 1:panel E). Subset frequencies between the cHL patients exhibited a higher variation than donors, including three patients whose B cell frequencies were comparable to the non-malignant samples. Further investigation within each compartment identified a five-fold increase in the frequency of EMRA CD4+ T cells and differential expression of CCR4 and CD69 in B cells. Finally, we adapted a method from minimal residual disease (MRD) to correctly detect the Hodgkin lymphoma malignant cells.

**Conclusions**

Our study shows that high-complexity immune profiling of the tumor microenvironment of cHL is feasible using CyTOF. This powerful tool has the potential to be utilized in the evaluation of immune-related prognostic markers and provide insight into targets and mechanisms with therapeutic potential in this disease.

**Acknowledgements**

This study was supported by the Mayo Clinic Cancer Immunology and Immunotherapy Program.

**References**

1. Pileri SA, Ascani S, Leoncini L, Sabattini E, Zinzani PL, Piccaluga PP, Pileri A Jr, Giunti M, Falini B, Bolis GB, Stein H. Hodgkin's lymphoma: the pathologist's viewpoint. J Clin Pathol. 2002 Mar;55(3):162-76.

2. Sánchez-Aguilera A, Montalbán C, de la Cueva P, Sánchez-Verde L, Morente MM, García-Cosío M, García-Laraña J, Bellas C, Provencio M, Romagosa V, de Sevilla AF, Menárguez J, Sabín P, Mestre MJ, Méndez M, Fresno MF, Nicolás C, Piris MA, García JF; Spanish Hodgkin Lymphoma Study Group. Tumor microenvironment and mitotic checkpoint are key factors in the outcome of classic Hodgkin lymphoma. Blood. 2006 Jul 15;108(2):662-8. Epub 2006 Mar 21.

3. Steidl C, Connors JM, Gascoyne RD. Molecular pathogenesis of Hodgkin's lymphoma: increasing evidence of the importance of the microenvironment. J Clin Oncol. 2011 May 10;29(14):1812-26. doi: 10.1200/JCO.2010.32.8401. Epub 2011 Apr 11.

4. Ansell SM, Lesokhin AM, Borrello I, Halwani A, Scott EC, Gutierrez M, Schuster SJ, Millenson MM, Cattry D, Freeman GJ, Rodig SJ, Chapuy B, Ligon AH, Zhu L, Grosso JF, Kim SY, Timmerman JM, Shipp MA, Armand P. PD-1 blockade with nivolumab in relapsed or refractory Hodgkin's lymphoma. N Engl J Med. 2015 Jan 22;372(4):311-9. doi: 10.1056/NEJMoa1411087. Epub 2014 Dec 6.

5. Levine JH, Simonds EF, Bendall SC, Davis KL, Amir ED, Tadmor MD, Litvin O, Fienberg HG, Jager A, Zunder ER, Finck R, Gedman AL, Radtke I, Downing JR, Pe'er D, Nolan GP. Data-Driven Phenotypic Dissection of AML Reveals Progenitor-like Cells that Correlate with Prognosis. Cell. 2015 Jul 2;162(1):184-97. doi: 10.1016/j.cell.2015.05.047. Epub 2015 Jun 18.

6. Greenplate AR, Johnson DB, Ferrell PB Jr, Irish JM. Systems immune monitoring in cancer therapy. Eur J Cancer. 2016 Jul;61:77-84. doi: 10.1016/j.ejca.2016.03.085. Epub 2016 May 4.

7. Lavin Y, Kobayashi S, Leader A, Amir ED, Elefant N, Bigenwald C, Remark R, Sweeney R, Becker CD, Levine JH, Meinhof K, Chow A, Kim-Shulze S, Wolf A7, Medaglia C, Li H, Rytlewski JA, Emerson RO, Solovyov A, Greenbaum BD, Sanders C, Vignali M, Beasley MB, Flores R, Gnjatic S, Pe'er D, Rahman A, Amit I, Merad M. Innate Immune Landscape in Early Lung Adenocarcinoma by Paired Single-Cell Analyses. Cell. 2017 May 4;169(4):750-765.e17. doi: 10.1016/j.cell.2017.04.014.

**Ethics Approval**

Deidentified specimens were retrieved from a prospective lymphoma biobank at the Mayo Clinic, Rochester (IRB# 118-01).

**Fig. 1 (abstract 043).**
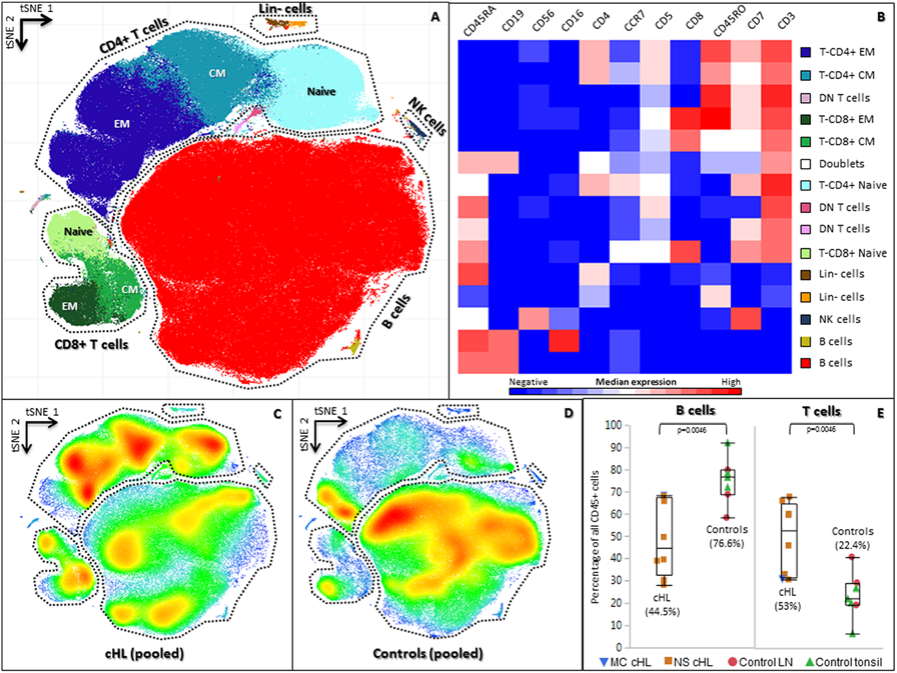
Immune profiling in cHL patients and non-malignant donors.

#### O44 Intratumoral CD163+ DC amplify the type 1 immune response of CD4+CD161+ T cells in HPV16-associated cancers and are associated with better survival

##### Chantal Duurland, PhD , Saskia Santegoets, Nikki Loof, Vanessa van Ham, Ilina Ehsan, Marij Welters, PhD, Sjoerd van der Burg

###### Leiden University Medical Center, Leiden, Netherlands

####### **Correspondence:** Chantal Duurland (c.l.duurland@lumc.nl)

**Background**

High-risk human papilloma virus type 16 (HPV16) induces the majority of cervical and oropharyngeal cancers. Patients with HPV16+ oropharyngeal squamous cell carcinoma (OPSCC) and an intratumoral type 1 T cell response against the viral oncoproteins E6 and E7 (immune response positive (IR+)) have a much better prognosis after (chemo)radiotherapy than HPV16- OPSCC. Analysis of the tumor microenvironment (TME) revealed that HPV16+IR+ OPSCC were highly infiltrated by CD4+CD161+ T cells with an effector memory phenotype and their numbers correlated with improved survival. To understand how CD4+CD161+ T cells contribute to better survival, the aim of this study is to examine the regulation and role of CD4+CD161+ T cells in HPV16+ cancers.

**Methods**

The TME was analysed by multispectral immunofluorescence on archived formalin-fixed paraffin-embedded tumor tissue, and flow cytometry on cryopreserved tumor infiltrating lymphocytes obtained from freshly isolated tumor tissue. DC subtypes were sorted from healthy individuals. HPV16-specific CD4+ T cell clones with or without CD161 expression were derived from OPSCC and cervical cancer patients. Proliferation and cytokine production were analysed by tritium-thymidine incorporation, flow cytometry and/or ELISA.

**Results**

Analysis of myeloid cell populations in the TME revealed a dense infiltration with a fairly unknown population of CD14-CD33-CD163+ myeloid cells and cell numbers correlated with a strong T cell infiltrate and improved survival. A more in-depth analysis of these cells by flow cytometry and multispectral immunofluorescence demonstrated that these cells represent a genuine DC population. In an allogeneic setting, blood-derived CD163- and CD163+ DC stimulated memory T cell proliferation, but IFNγ was mainly produced by CD161+ T cells. Interestingly, activated CD163+ DC produced higher levels of IL-12 and IL-18 than their CD163- counterparts. These cytokines are known inducers of a type 1 immune response and blockade of IL-12 and IL-18 impaired IFNγ production by T cells stimulated with these DC. Furthermore, IFNγ-production by cognate antigen-stimulated patient-derived HPV16-specific CD4+ T cells clones was enhanced by the addition of IL-12 and IL-18. Notably, under suboptimal antigen conditions, HPV16-specific CD4+CD161+ T cells produced more IFNγ in response to IL-12 and IL-18 than their CD4+CD161- counterparts.

**Conclusions**

In conclusion, intratumoral CD163+ DC contribute to a stronger antitumor response through the production of IL-12 and IL-18 which amplifies especially the type 1 response of CD4+CD161+ T cells as these cells are more sensitive to IL-12 and IL-18 than their CD161- counterparts.

**Ethics Approval**

This study was approved by the Leiden University Medical Center (LUMC) Ethics Board, approval number P07-112 and P08-197.

#### O45 Overexpression of CD200 is a stem cell-specific mechanism of T cell evasion in AML

##### Shelley Herbrich, MS, Gheath Alatrash, DO, PhD, Richard Davis, MD, Michael Curran, PhD, Marina Konopleva, MD, PhD

###### The University of Texas MD Anderson Cancer Center, Houston, TX, United States

####### **Correspondence:** Marina Konopleva (mkonopleva@mdanderson.org)

**Background**

Acute myeloid leukemia (AML) stem cells (LSC) are capable of surviving current standard chemotherapy and the likely source of deadly, relapsed disease. To identify potential immunotherapeutic targets, we sourced multiple large, publically available datasets and identified CD200 as a potential stem-cell specific immune checkpoint in AML. CD200 has been shown to have an immunosuppressive effect on macrophages [1] and NK cells [2] and correlates with a high prevalence of FOXP3+ regulatory T cells [3]. Additionally, CD200 has been implicated as a poor prognostic marker in AML [4]. To date, we have screened 40 primary AML patient samples by flow cytometry, 95% of which are positive for CD200.

**Methods**

To study the functional role of CD200 in AML, we generated a CD200 overexpression model in the OCI-AML3 cell line (with no basal expression) and characterized changes in proliferation, survival, and gene expression. To examine the immune function of CD200 in AML in vitro, we performed a series of mixed lymphocyte reactions where normal PBMCs were cultured with the CD200+ or CD200- leukemia cells and measured by flow cytometry for apoptosis, proliferation, or intracellular cytokine production. Finally, we established a humanized-PBMC mouse model in which we tracked the engraftment and overall survival of the CD200+/- OCI-AML3 cells.

**Results**

CD200 overexpression did not affect the proliferation rate or viability of the AML cells. However by RNAseq, CD200+ cells significantly downregulated genes involved in inflammatory response and those regulated by NF-κB in response to TNFα; indicating a potential intrinsic role in suppressing the immune microenvironment of AML LSCs. In vitro, healthy PBMCs significantly reduced the production of inflammatory cytokines (including TNFα and IFNγ; Fig1 A,B) and cytotoxic enzymes (including GzmA, GzmB, Perforin, and sFAS ligand; Fig1 C,D) in the presence of AML cell-bound CD200. Interestingly, this did not translate into direct AML cell killing in this system. In vivo, OCI-AML3 CD200+/- cells showed no difference in engraftment, progression, and overall survival in immunodeficient NSG mice. However, when mice were humanized using healthy PBMCs (Fig 1E), CD200+ leukemia progressed rapidly, escaping elimination by T cells, compared to CD200- control leukemic cells (Fig 1F).

**Conclusions**

We have identified CD200 as a putative stem cell-specific immunomodulatory target that aids in establishing an immunosuppressive microenvironment and allows cells to escape immune detection and destruction resulting in disease progression. These findings indicate a utility of CD200 as a novel immune checkpoint target for the development of therapeutic strategies in AML.

**References**

1. Hoek RM, Ruuls SR, Murphy CA, Wright GJ, Goddard R, Zurawski SM, Blom B, Homola ME, Streit WJ, Brown MH, Barclay AN, Sedgwick JD. Down-regulation of the macrophage lineage through interaction with OX2 (CD200). Science. 2000; 290: 1768-71.

2. Coles SJ, Hills RK, Wang EC, Burnett AK, Man S, Darley RL, Tonks A. Expression of CD200 on AML blasts directly suppresses memory T-cell function. Leukemia. 2012; 26: 2148-51.

3. Coles SJ, Hills RK, Wang EC, Burnett AK, Man S, Darley RL, Tonks A. Increased CD200 expression in acute myeloid leukemia is linked with an increased frequency of FoxP3+ regulatory T cells. Leukemia. 2012; 26: 2146-8.

4. Tonks A, Hills R, White P, Rosie B, Mills KI, Burnett AK, Darley RL. CD200 as a prognostic factor in acute myeloid leukaemia. Leukemia. 2007; 21: 566-8.

**Ethics Approval**

All animal studies were approved by the MD Anderson Cancer Center Institutional Animal Care and Use Committee (Houston, Texas, USA) under protocol 00001146-RN0/3.

**Figure:**
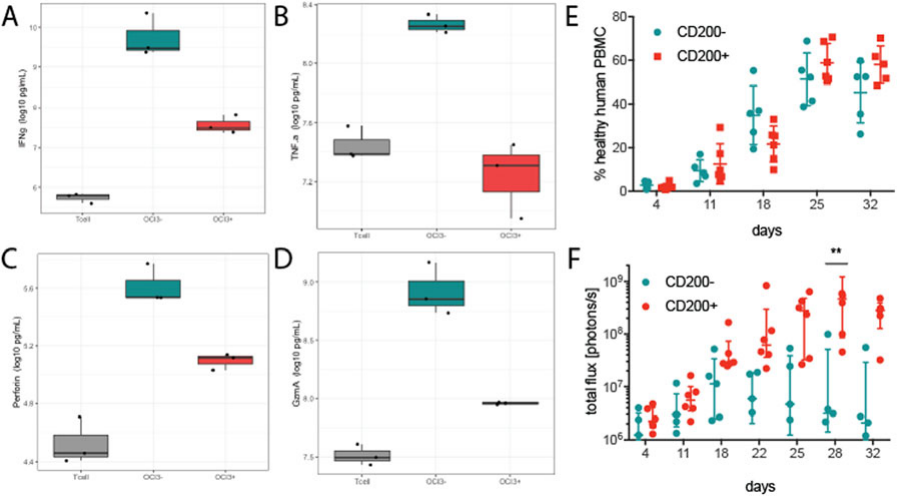
**Fig. 1 (abstract 045).** See text for description

#### O46 Characterization of tumor infiltrating and post HSCT NK and T cells in human cancer patients

##### Sean Judge, MD, MS^1^, Cordelia Dunai^1^, Ian Sturgill^1^, Kevin Stoffel, MS^1^, Patrice Chevallier^2^, Christelle Retière^2^, Arta Monjazeb^1^, Robert Canter, MD, MAS, FACS^1^ , William Murphy, PhD^3^

###### ^*1*^*University of California, Davis, Sacramento, CA, United States* ; ^*2*^*INSERM, Nantes University, Nantes, France* ; ^*3*^*University of California, Sacramento, CA*

####### **Correspondence:** Robert Canter (rjcanter@ucdavis.edu), William Murphy (wmjmurphy@ucdavis.edu)

**Background**

To expand the promise of immunotherapy, anti-PD-1 checkpoint inhibition is being applied to non-T cell-based strategies, including NK cells. Given that expression of PD-1 by NK cells remains controversial, we set out to characterize the phenotype and function of tumor infiltrating NK (and T) populations, including PD-1 and TIGIT, in human cancer patients.

**Methods**

We evaluated peripheral blood and tumor specimens from sarcoma and colon cancer patients and blood from post-allogeneic, T cell replete HSCT patients with diverse hematologic malignancies. We also assessed intra-tumoral lymphocytes from subcutaneous tumor-bearing mice (CT26 colon, 4T1 mammary, B16 melanoma, and 3LL lung) with paired mouse splenocyte populations to validate findings from human specimens. Immune phenotype and function were analyzed by flow cytometry. Cell types were quantified, including expression of PD-1 and TIGIT.

**Results**

Within human tumors, T cells (CD3+CD56-) consistently outnumbered NK cells (CD3-CD56+) within soft tissue sarcomas (2.6 x 10^5±3.9 x 10^5 vs 5.5 x 10^4±8.5 x 10^4 per million live cells), but not within colorectal cancer (2.1 x 10^5 vs 3.6 x 10^5). Intra-tumoral NK and T cells had higher expression of CD69 compared to blood (60% and 22% vs

**Conclusions**

NK cells represent a minor fraction of infiltrating lymphocytes across murine tumor types, whereas NK cell frequencies are variable in human tumors with minimal infiltration in sarcomas but high infiltration in colon cancer. Across human and mouse tumors (and HSCT patients), PD-1 expression on NK cells is negligible indicating that NK cells are unlikely to be a direct mediator of checkpoint blockade therapy. In contrast, TIGIT shows substantial expression on human intra-tumoral NK and T cells, suggesting key immunoregulatory function in the tumor microenvironment and the potential for therapeutic targeting.

**Ethics Approval**

Collection of human blood and tumor specimens was approved by the Institutional Review Board at the University of California, Davis or Ethics Review Board of the Nantes University Hospital, and complied with all ethical regulations.

#### O47 IL-35+ B cells regulates anti-tumor immune response in pancreatic cancer

##### Bhalchandra Mirlekar, PhD, Yuliya Pylayeva-Gupta

###### Lineberger Cancer Center, University of North Carolina at Chapel Hill, Chapel Hill, NC, United States

####### **Correspondence:** Yuliya Pylayeva-Gupta (yuliyap1@email.unc.edu)

**Background**

Pancreatic ductal adenocarcinoma (PDA) is an aggressive malignancy and 3rd-leading cause of cancer-related death with a dismal 5-year survival rate of 7%. Unfortunately, recent efforts towards treatment of pancreatic cancer with immunotherapy have not seen successful. A major barrier for immunotherapeutic approaches in PDA is marked immunosuppression within pancreatic tumor milieu [1,2]. Understanding the mechanisms that drive immunosuppression may help to improve the efficacy of current immunotherapies. Therefore, the comprehensive aim of this research is to reveal cellular and molecular mechanisms by which immune cells promote the growth of tumor cells and thereby facilitate the progression of cancer.

**Methods**

We employ spontaneous and orthotopic mouse models of PDA which summarizes human pancreatic cancer [3,4]. We achieved this goal by using mouse model with a B cell-specific genetic loss of IL-35 combined with orthotopic PDA modeling. Additionally, we assessed the effect of anti-IL-35 blockade in combination with immune checkpoint blockade therapy as a pharmacological approach to provide additional invigoration for T cell-mediated killing of pancreatic cancer cells.

**Results**

Our analysis reveals a specialized subset of B cells, termed regulatory B cells, which promote pancreatic tumorigenesis through production of the cytokine IL-35 (encoded by subunits p35 and Ebi3) which has been previously implicated in suppression of T cell driven autoimmune diseases [5,6]. Our results demonstrate that IL-35, but not IL-10, potentiates PDA growth. This correlates with induction of regulatory T cells and suppression of effector T cells, suggesting that IL-35 controls endogenous anti-tumor immune responses [7]. While IL-35 is expressed by several immune cell types in PDA, we show that IL-35 expression specifically in B cells is essential for suppression of anti-tumor immunity. Importantly, while PDA is typically resistant to anti-PD-1 immunotherapy, we demonstrate robust synergistic reduction in tumor growth when B cell specific IL-35 deficiency is combined with anti-PD-1. Finally, we have identified an IL-35+ B cell subset in PDA patients and demonstrate that the presence of IL-35+ cells in tumor samples predicts increased occurrence of dysfunctional CD8+ T cells.

**Conclusions**

PDA is resistant to immune-checkpoint therapy therefore understanding the mechanism of function of IL-35+ B cells in PDA provide us with novel therapeutic targets that could be used to synergize with existing immunotherapeutic approaches and provide a path to clinical translation. In addition to expanding our basic knowledge of tumor immunology, this research has direct relevance for the design of immune based treatment therapies against patients with pancreatic cancer.

**References**

1. Sideras K, Braat H, Kwekkeboom J, van Eijck CH, Peppelenbosch MP, Sleijfer S et al. Role of the immune system in pancreatic cancer progression and immune modulating treatment strategies. Cancer Treat Rev. 2014;40(4):513-22.

2. Chang JH, Jiang Y, Pillarisetty VG. Role of immune cells in pancreatic cancer from bench to clinical application: An updated review. Medicine (Baltimore). 2016;95(49):e5541.

3. Hingorani SR, Petricoin EF, Maitra A, Rajapakse V, King C, Jacobetz MA, et al. Preinvasive and invasive ductal pancreatic cancer and its early detection in the mouse. Cancer Cell. 2003;4(6):437-50.

4. Tuveson DA1, Hingorani SR. Ductal pancreatic cancer in humans and mice. Cold Spring Harb Symp Quant Biol. 2005;70:65-72.

5. Pylayeva-Gupta Y, Das S, Handler JS, Hajdu CH, Coffre M, Koralov SB, et al. IL35-Producing B Cells Promote the Development of Pancreatic Neoplasia. Cancer Discov. 2016;6(3):247-55.

6. Mauri C, Bosma A. Immune regulatory function of B cells. Annu Rev Immunol. 2012;30:221-41.

7. Mirlekar B, Michaud D, Searcy R, Greene K, Pylayeva-Gupta Y. IL35 Hinders Endogenous Antitumor T-cell Immunity and Responsiveness to Immunotherapy in Pancreatic Cancer. Cancer Immunol Res. 2018;6(9):1014-1024.

**Ethics Approval**

All mouse protocols were reviewed and approved by the Institutional Animal Care and Use Committee of the University of North Carolina at Chapel Hill (NIH/PHS Animal welfare assurance number: D16-00256 and study approval number: 17-064). Animals were maintained in a specific pathogen-free facility.

#### O48 Characterizing the anti-tumor immune response to an array of tumor cell lines expressing unique neoantigens

##### Christopher Copeland, Kim Nguyen, BA, Stefani Spranger, PhD

###### Massachusetts Institute of Technology, Cambridge, MA, United States

####### **Correspondence:** Stefani Spranger (spranger@mit.edu)

**Background**

Recent clinical studies have provided evidence that therapeutic vaccinations against mutationally derived neoantigens can enhance anti-tumor immunity and provide long-term protection. The efficacy of these therapies varies and recent work suggests that the affinity and sequence motifs of the neoantigens can be critical determinants of the magnitude of the induced immune response. However, systemic in vivo studies have thus far been hampered by the lack of appropriate neoantigen-expressing tumor mouse models.

**Methods**

Using a curated list of neoantigens previously identified [1-4] we have established an array of lung cancer cell lines expressing a single neoantigen, with neglectable additional mutational burden. The chosen neoantigens have varying predicted binding affinities to MHCI molecules (NetMHC and Syfpeithi). Each line was used to establish a subcutaneous tumor in immunocompetent or immune-deficient mice. Tumors were harvested at day 9 and day 28 and subjected to flow cytometry analysis to phenotype T cell populations. ELISpot assays were performed using splenocytes harvested from mice to determine the frequency of neoantigen-specific T cells in the periphery following peptide vaccination or tumor implantation. Cross-reactivity to the wildtype peptide sequence was also assessed. Mice were also prophylactically vaccinated with peptides derived from the neoantigen sequences before tumor implantation to study differences in therapeutic efficacies.

**Results**

Peptide vaccination alone was sufficient to induce expansion of neoantigen-specific T cells in the spleen for each neoantigen, though the T cell frequency and cross-reactivity to the non-mutated peptide varied depending on the peptide. The immune response of naïve mice to implanted tumors also varied depending on the peptide, while all tumor cell lines were able to grow with similar kinetics in immunocompetent mice. There was no correlation between response induced by peptide vaccination and response induced by tumor implantation by IFN-gamma ELISpot assays. A trend between anti-tumor immune response and peptide: MHC affinity could be observed however no correlation between affinity and therapeutic efficacy could be detected.

**Conclusions**

Using our reductionist syngeneic mouse model and using for the first time true neoantigens rather than model antigens our results suggest that therapeutic peptide vaccination may not be sufficient to drive tumor control and elimination even with predicted high-affinity peptide-MHC complexes. Instead, it may be the case that potentiating the anti-tumor immune response with checkpoint blockade antibody treatment will segregate high-affinity peptide-MHC complexes as responders and the remaining complexes as non-responders.

**References**

1. Castle JC, Kreiter S, Diekmann J, et al. Exploiting the mutanome for tumor vaccination. Cancer Res. 2012. doi:10.1158/0008-5472.CAN-11-3722

2. Gubin MM, Zhang X, Schuster H, et al. Checkpoint blockade cancer immunotherapy targets tumour-specific mutant antigens. Nature. 2014. doi:10.1038/nature13988

3. Matsushita H, Vesely MD, Koboldt DC, et al. Cancer exome analysis reveals a T-cell-dependent mechanism of cancer immunoediting. Nature. 2012. doi:10.1038/nature10755

4. Yadav M, Jhunjhunwala S, Phung QT, et al. Predicting immunogenic tumour mutations by combining mass spectrometry and exome sequencing. Nature. 2014. doi:10.1038/nature14001

#### O49 Chronic activation under hypoxia drives T cell differentiation to exhaustion

##### Nicole Scharping, PhD, Yiyang Wang, Ashley Menk, MS, Greg Delgoffe, PhD

###### University of Pittsburgh, Pittsburgh, PA, United States

####### **Correspondence:** Greg Delgoffe (delgoffeg@upmc.edu)

**Background**

CD8+ tumor-infiltrating T lymphocytes (TIL) progressively succumb to a dysfunctional state known as exhaustion, characterized by poor effector function and sustained co-inhibitory marker expression, but the factors causing this hyporesponsive phenotype remain unclear. We have previously demonstrated that exhausted TIL also exhibit progressive loss of functional mitochondria, due in part to repression of the transcriptional co-activator PGC1α, resulting in suppressed mitochondrial fusion and biogenesis. Enforcing PGC1α expression in tumor-specific T cells not only led to increased mitochondria, but improved T cell functionality, decreased tumor burden, and increased survival in mouse B16 melanoma. We then hypothesized T cell exhaustion may be driven by metabolic insufficiency and led us to explore the mechanism driving T cell exhaustion.

**Methods**

We developed models to identify how tumor-derived signals – hypoxia and chronic T cell receptor activation – induce metabolic insufficiency and T cell exhaustion in vitro and in vivo.

**Results**

Using several complementary systems, we found T cells experiencing chronic activation or hypoxia alone in vitro could carry out similar or superior effector functions, but experiencing both chronic activation and hypoxia simultaneously generated profound, persistent T cell dysfunction. This dysfunction was characterized by high co-inhibitory and TOX expression, loss of IFNγ production, and decreased mitochondrial oxygen consumption. Terminally exhausted TIL have high expression of the transcriptional repressor Blimp1, which we found to be driven by chronic activation and caused potent repression of PGC1α transcription. This repression resulted in dysfunctional mitochondria and excessive mitochondrial reactive oxygen species (ROS). Inducing mitochondrial ROS alone was sufficient to cause T cell exhaustion, which could be prevented by mitigating ROS with antioxidant co-culture. Consequently, by reducing tumor hypoxia in vivo, we can slow the differentiation to T cell exhaustion, allowing for a greater abundance of functional T cells in the TME and improving responses to checkpoint blockade immunotherapy.

**Conclusions**

Our findings support a model in which chronic activation fundamentally alters how T cells respond to hypoxic conditions, exposing a mechanism of T cell exhaustion in cancer. These data reveal potential metabolic avenues to rescue exhausted T cells and improve immunotherapy.

**Ethics Approval**

The study was approved by Institutional Animal Care and Use Committee of the University of Pittsburgh, approval number 17071235.

#### O50 PD1+ T regulatory cells in metastatic cancer patients co-express CD39 and exhibit attenuated suppressive function due to decreased survival

##### Richard Wu, MD, PhD^1^, Christopher Chuckran, BS^2^, Ashwin Somasundaram, MD^3^, Rajeev Dhupar, MD^1^, Katie Nason, MD^1^, Adam Soloff, PhD^2^, Michael Lotze, MD^1^, Caleb Lampenfeld, BS^2^, Sheryl Kunning^2^, Tullia Bruno, PhD^2^, Dario Vignali, PhD^2^

###### ^*1*^*University of Pittsburgh Medical Center, Pittsburgh, PA, United States* ; ^*2*^*University of Pittsburgh, Pittsburgh, PA, United States* ; ^*3*^*UPMC Cancer Center, Pittsburgh, PA, United States*

####### **Correspondence:** Tullia Bruno (tbruno@pitt.edu), Dario Vignali (dvignali@pitt.edu)

**Background**

While T regulatory cells (Tregs) maintain normal immune homeostasis, they are also potent suppressors of the anti-tumor immune response within the tumor microenvironment (TME). Tregs are associated with poor clinical response in a subset of melanoma and gastric cancer patients treated with anti-PD1 immunotherapy [1]. Currently, it is not known how inhibitory receptors (IRs) impact the survival or suppressive function of tumor-infiltrating Tregs in metastatic cancer patients [2, 3].

**Methods**

Using high-dimensional spectral cytometry (Cytek®), we have undertaken a comprehensive characterization of Treg phenotype and function from metastatic melanoma (n = 10) and malignant pleural effusions (MPE) (n = 27). We analyzed the differences in multiple inhibitory and stimulatory receptors, membrane-bound purinergic receptors (CD39, CD73), as well as differences in proliferation (Ki-67) and survival (Bcl-2) between Treg subsets. Since PD1 and CD39 were two of the highest-expressing inhibitory and purinergic receptors on Tregs in MPEs, we sorted these populations and utilized an allogeneic microsuppression assay to determine the differences in suppressive function between PD1+CD39+, PD1-CD39+, and PD1-CD39- Treg subsets.

**Results**

Our results indicate that Tregs in metastatic cancer patients express significantly higher levels of PD1, CD39, and ICOS, compared to Tregs from patient or healthy donor peripheral blood. We found PD1+ Tregs co-expressed CD39 and ICOS, and expressed higher levels of proliferation (Ki-67) with lower levels of the anti-apoptotic protein Bcl-2, compared to PD1- Tregs. After sorting MPE Tregs by PD1 expression, we observed that the PD1- subset was the most suppressive, while PD1+ subset was the least suppressive. We also found that the anti-PD1 blockade with nivolumab enhanced the suppression of the sorted PD1+ Tregs, while having no significant impact on the sorted PD1- Tregs.

**Conclusions**

Our study indicates that PD1 and CD39-expressing Tregs are enriched in the TME and seem to be more dysfunctional, likely due to local tumor factors such as chronic TCR stimulation, adenosine receptor signaling (known drivers of PD1), or hypoxia (a known driver of CD39). PD1+ Tregs, though co-expressing the activation marker ICOS and membrane-bound CD39, nevertheless lose suppressive function due to susceptibility to apoptosis. Thus, the relative ratios of PD1+ vs. PD1- Tregs in the TME may influence the response of metastatic cancer patients to immunotherapy.

**Acknowledgements**

Funding support from the UPMC Cancer Therapeutics T32 Training Program (PI: Dr. Edward Chu)

**References**

1. Kamada T, Togashi, Y, Tay C, et al. PD-1+ regulatory T cells amplified by PD-1 blockade promote hyperprogression of cancer. Proc Natl Acad Sci. 2019; 116(20): 9999 - 10008.

2. Lowther, D.E., Goods, B.A., Lucca, L.E., et al. PD-1 marks dysfunctional regulatory T cells in malignant gliomas. JCI Insight, 2016. 1(5): e85935.

3. Sim, G.C., Martin-Orozco N, Jin L, et al., IL-2 therapy promotes suppressive ICOS+ Treg expansion in melanoma patients. J Clin Invest, 2014. 124(1): p. 99-110.

**Ethics Approval**

The study was approved by the UPMC Cancer Center’s Institutional Review Board.

#### O51 Sirt2 inhibition enhances anti-tumor immunity by promoting T cell metabolism

##### Imene Hamaidi, PhD^1^, Lin Zhang, MD PhD^2^, Nayoung Kim, PhD^3^, Min-Hsuan Wang^1^, Cristina Iclozan, PhD^1^, Bin Fang, PhD^1^, Min Liu, PhD^1^, John Koomen, PhD^1^, Anders Berglund, PhD^1^, Sean Yoder^1^, Jiqiang Yao, PhD^1^, Ben Creelan, MD, MS^1^, Scott Antonia, MD PhD^4^, Sungjune Kim, MD PhD^1^

###### ^*1*^*Moffitt Cancer Center, Tampa, FL, United States* ; ^*2*^*Tianjin Medical University Cancer Institute, Tianjin, China* ; ^*3*^*Rosetta Exosome Inc., Seoul,, Korea, Democratic People's Republic* ; ^*4*^*Duke University School of Medicine, Durham, NC, United States*

####### **Correspondence:** Sungjune Kim (Sungjune.Kim@moffitt.org)

**Background**

Despite an unprecedented success with immune checkpoint inhibitors and adoptive cell therapies, the majority of patients remain refractory to immunotherapy. Strategies to overcome immune barriers include manipulation of immune metabolism. There is a growing consensus that metabolism is a key driver of T cell functions with a direct impact on anti-tumor immunity. Sirt2 is an (NAD+)-dependent histone deacetylase with conflicting reports on its tumor suppressor or oncogenic roles. In the immune system Sirt2 appears to primarily function as a suppressor of inflammation but its role in T cell biology has not been previously explored.

**Methods**

Sirt2 expression in human and murine T cells was assessed by flow cytometry and Western blot (Wb). The role of Sirt2 in anti-tumor immunity was assessed by in vivo B16F10 tumor challenge, ex vivo cell proliferation (CFSE labeling), IFN-γ ELISpot assay, granzyme B expression and LDH cytotoxicity assay in Wild-type and Sirt2 knockout mice. Phenotypic profiling of peripheral T cells from B16F10 challenged mice was conducted by flow cytometry. Sirt2 interactome and acetylome were identified via mass spectrometry (MS) and immunoprecipitation/Wb analyses. T cells metabolism was assessed by seahorse extracellular flux analyzer and metabolomic MS analyses. Pharmacologic inhibition of Sirt2 in human T cells was achieved using AGK2, a Sirt2 selective inhibitor.

**Results**

Upregulation of Sirt2 expression in human tumor-infiltrating lymphocytes (TILs) compared to peripheral blood mononuclear cells (PBMCs) from patients enrolled into a phase I clinical trial of Nivolumab and TILs in advanced non-small-cell lung cancer (NSCLC) correlated with a poor clinical response to immunotherapy. Sirt2 deficiency in mice led to hyper-reactive T cell phenotype ex vivo with improved capacity to reject tumor challenge in vivo. Sirt2 was found to directly interact with key glycolytic enzymes including PFKP, GAPDH, enolase and aldolase during T cell activation, to modify their acetylation status and enzymatic activity. Consistently, Sirt2-/- T cells exhibited a hyper-metabolic status with a profound upregulation of glycolysis and enhanced proliferation and effector activity in mice. More importantly, pharmacologic inhibition of Sirt2 with AGK2 conferred human TILs with superior metabolic fitness and enhanced effector functions.

**Conclusions**

We demonstrate that Sirt2 is a metabolic immune checkpoint and its abrogation enhances the metabolic and functional fitness of tumor reactive T cells to augment cancer immunotherapy.

**Acknowledgements**

This work was supported in part by K08 CA194273, ACS IRG-17-173-22, NCI Cancer Center Support Grant (P30-CA076292) and the Moffitt Foundation.

**Ethics Approval**

Human studies for collection and expansion of TILs received Institutional Review Board (IRB) approval: #MCC18541 and #MCC19122. Tumor biopsies were procured through Moffitt Cancer Center Hospital. All subjects provided informed consent.

For animal studies, all procedures were approved and performed in accordance with guidelines provided by the Institutional Animal Care and Use Committee: #MIS00004728 and #RIS00005143.

#### O52 ADT-mediated IL-8 promotes myeloid infiltration and prostate cancer progression

##### Zoila Lopez Bujanda, MS^1^, Michael Haffner, MD^1^, Matthew Chaimowitz^2^, Nivedita Chowdhury^1^, Nicholas Venturini^2^, Aleksandar Obradovic^2^, Corey Hansen^2^, Joanna Jacków^2^, Karen Sfanos^1^, Charles Bieberich^3^, Paula Hurley^1^, Mark Selby^4^, Alan Korman^4^, Angela Christiano, PhD^2^, Angelo De Marzo^1^, Charles Drake, MD, PhD^2^

###### ^*1*^*Johns Hopkins University, New York, NY, United States* ; ^*2*^*Columbia University, New York, NY, United States* ; ^*3*^*University of Maryland Baltimore County, Baltimore, MD, United States* ; ^*4*^*Bristol-Myers Squibb, Redwood City, CA, United States*

####### **Correspondence:** Charles Drake (cgd2139@cumc.columbia.edu)

**Background**

Immunotherapy is a treatment for many types of cancer, primarily due to deep and durable clinical responses mediated by immune checkpoint blockade (ICB)[1,2]. Prostate cancer is a notable exception in that it is generally unresponsive to ICB. The standard treatment for advanced prostate cancer (androgen-deprivation therapy; ADT) is initially effective, but over time patients eventually develop castration-resistant prostate cancer (CRPC). Recently, the myeloid-derived suppressor cell (MDSC) subset that most closely relates to neutrophils, called polymorphonuclear (PMN)-MDSCs, were correlated with decreased overall survival (OS), increased levels of IL-8 (also known as CXCL8), immune resistance, and prostate cancer progression from localized to metastatic disease[3, 4, 5].

**Methods**

We evaluated the differential expression profile of tumor epithelial cells isolated from castration-sensitive (CS) and ADT-treated MCRedAL tumor bearing mice. The effect of two transcription factor affected by ADT treatment (p65 subunit of NF-kB and AR) was evaluated by Chip-Seq and Chip-PCR experiments on CS LNCaP cells. Their respective effects on the promoter activity of interlukine-8 (IL-8) was further evaluated by luciferase assays. ADT-mediated IL-8 and Cxcl15 expression in tumor cells was confirmed by RNA-ISH and qPCR in murine and human prostate cancer samples, respectively. Intratumoral infiltration of PMN-MDSCs was evaluated in vitro by transwell assays and in vivo by flow cytometry in a set of murine and human cell lines and xenograph models. Tumor outgrowth of an immunocompetent model of prostate cancer after receiving CXCR2 blockade in combination with ICB + ADT was evaluated and compared to ICB + ADT alone. Finally, polyfunctional CD8 T cells were evaluated by intracellular staining of TNFα, INFγ, Granzyme B, and IL-2 cytokines.

**Results**

ADT regulated gene expression of both IL-8 and it's potential mouse homologue (Cxcl15). These chemokines drove subsequent intra-tumoral infiltration with PMN-MDSCs, promoting tumor progression. PMN-MDSC infiltration was abrogated when IL-8 was deleted from prostate cancer epithelial cells using CRISPR/Cas9, or when PMN-MDSC migration was blocked with antibodies against the IL-8 receptor CXCR2. Blocking PMN-MDSC infiltration in combination with anti-CTLA-4 delayed the onset of castration resistance and increased the density of polyfunctional CD8 T cells in tumors.

**Conclusions**

Taken together, our findings establish ADT-mediated IL-8 secretion and subsequent PMN-MDSC infiltration as a key suppressive mechanism in the progression of prostate cancer. Targeting of the IL-8/CXCR2 axis around the time of ADT, in combination with ICB, represents a novel therapeutic approach to delay prostate cancer progression to advanced disease.

**References**

1 Topalian, S. L., Drake, C. G. & Pardoll, D. M. Immune checkpoint blockade: a common denominator approach to cancer therapy. Cancer Cell 27, 450-461, doi:10.1016/j.ccell.2015.03.001 (2015).

2 Drake, C. G. Prostate cancer as a model for tumour immunotherapy. Nat Rev Immunol 10, 580-593, doi:10.1038/nri2817 (2010).

3. Kawahara, T., et al., Neutrophil-to-lymphocyte ratio predicts prostatic carcinoma in men undergoing needle biopsy. Oncotarget, 2015. 6(31): p. 32169-76.

4. Lopez-Bujanda, Z. and C.G. Drake, Myeloid-derived cells in prostate cancer progression: phenotype and prospective therapies. J Leukoc Biol, 2017. 102(2): p. 393-406.

5. Yin, X., et al., Prognostic Role of Neutrophil-to-Lymphocyte Ratio in Prostate Cancer: A Systematic Review and Meta-analysis. Medicine (Baltimore), 2016. 95(3): p. e2544.

#### O53 Altered T-cell clonotype composition is strongly associated with differential functional orientation of colon cancer microenvironment

##### Jessica Roelands^1^, Peter Kuppen^2^, Lisa Mathew^3^, Amany Awad^3^, Francesco Marincola, MD^3^, Darawan Rinchai, PhD^3^, Davide Bedognetti, MD, PhD^3^, Wouter Hendrickx, PhD^3^

###### ^*1*^*Sidra Medical Research Center, Doha, Qatar* ; ^*2*^*Leiden University Medical Center, Leiden, Zuid-Holland, Netherlands* ; ^*3*^*Sidra Medicine, Doha, Qatar*

####### **Correspondence:** Wouter Hendrickx (whendrickx@sidra.org)

**Background**

An active immune microenvironment characterized by high levels of tumor-infiltrating lymphocytes and cytotoxic activity, has been associated better prognosis of colorectal cancer patients [1]. In addition to cytotoxicity, characteristics of immune-mediated tumor rejection include expression of chemokine-receptor ligands, activation of interferon-stimulated genes and upregulation of immune regulatory genes [2]. The Immunological Constant of Rejection (ICR) gene signature summarizes these aspects and can be used to define tumor immune phenotypes [2-5]. It is increasingly clear that the composition of the T-cell repertoire, defined by T-cell clonality, contains additional valuable information. A high T-cell clonality implies expansion of specific T-cell clones. Until now, the relation between an active immune microenvironment and T-cell clonality in colon cancer remains unexplored. Here, we profiled the T-cell receptor (TCR) of primary colon cancer samples and investigated its relation to the immune contexture.

**Methods**

118 primary colon cancer samples were analyzed for their TCR profile. RNA and DNA were isolated from fresh frozen tissue samples of a cohort of colon cancer patients collected over the last decade at Leiden University Medical Center, the Netherlands. T-cell repertoire profiling of samples was performed using Adaptive immunoSEQ. This assay specifically amplifies and sequences the complementary determining region 3 (CDR3) of the TCR-beta locus. Both the TCR and RNASeq was performed using Illumina sequencing at Sidra Medicine. ICR score was calculated as previously described [2-5].

**Results**

Our multi-modal approach allowed us to investigate the association between T-cell clonality and cancer immune phenotypes. We here report, for the first time, a significant positive correlation between an active Th1 immune phenotype and a higher TCR clonality in colon cancer. T-cell clonality strongly correlated with the expression of the ICR signature (R=0.62, p=4.4e-14) [2–4], and less with T-cell enrichment estimates. Importantly, the clonality of T-cells was highest in tumor pathologic stage I compared with later stages of disease. In-depth analysis of the clonal structure of T-cell infiltrates and correlation with outcome is currently ongoing.

**Conclusions**

We here demonstrated that an active immune tumor microenvironment in colon cancer is positively associated with a more clonal T-cell repertoire. This observation suggests that specific T-cell clones are expanded in colon cancer with a Th1 polarized immune response.

**Acknowledgements**

This research has been supported by Qatar Foundation, Qatar National Research Fund (grant numbers: JSREP07-010-3-005 and NPRP-10-0126-170262 awarded to WH and DB, respectively).

**References**

1. Pagès F, Mlecnik B, Marliot F, Bindea G, Ou F-S, Bifulco C, et al. International validation of the consensus Immunoscore for the classification of colon cancer: a prognostic and accuracy study. The Lancet. 2018 May 26;391(10135):2128–39.

2. Galon J, Angell HK, Bedognetti D, Marincola FM. The Continuum of Cancer Immunosurveillance: Prognostic, Predictive, and Mechanistic Signatures. Immunity. 2013 Jul 25;39(1):11–26.

3. Wang E, Worschech A, Marincola FM. The immunologic constant of rejection. Trends Immunol. 2008 Jan 6;29(6):256–62.

4. Hendrickx W, Simeone I, Anjum S, Mokrab Y, Bertucci F, Finetti P, et al. Identification of genetic determinants of breast cancer immune phenotypes by integrative genome-scale analysis. OncoImmunology. 2017 Feb 6;0(ja):00–00.

5. Roelands J, Hendrickx W, Kuppen PJK, Mall R, Zoppoli G, Saad M, et al. Genomic landscape of tumor-host interactions with differential prognostic and predictive connotations. bioRxiv. 2019 May 8;546069.

**Ethics Approval**

Sidra Medicine IRB approval : #1602002725.

#### O54 Germline genetic variation affects the immune response in cancer

##### Rosalyn Sayaman, PhD^1^, Mohamad Saad, PhD^2^, Vésteinn Thorsson, PhD^3^, Younes Mokrab^4^, Wouter Hendrickx, PhD^4^, Farshad Farshidfar^5^, Tomas Kirchhoff, PhD^6^, Randy Sweis, MD^7^, Najeeb Syed^4^, Oliver Bathe^8^, Eduard Porta-Pardo^9^, Cynthia Stretch^10^, Donglei Hu^11^, Scott Huntsman^11^, Jessica Roelands, Master^4^, Simon Shelley^12^, Denise Wolf^11^, Jerome Galon^13^, Francesco Marincola^14^, Michele Ceccarelli^15^, Elad Ziv^11^, Davide Bedognetti, MD, PhD^4^

###### ^*1*^*City of Hope Comprehensive Cancer Center / University of California, San Francisco, Duarte, CA, United States* ; ^*2*^*Hamad Bin Khalifa University, Doha, Qatar* ; ^*3*^*Institute for Systems Biology, Seattle, WA, United States* ; ^*4*^*Sidra Medicine, Doha, Qatar* ; ^*5*^*University of Calgary / Stanford University, Alberta, Canada* ; ^*6*^*New York University Langone Health New York, New York, NY* ; ^*7*^*University of Chicago, Chicago, IL, United States* ; ^*8*^*Tom Baker Cancer Centre, Alberta, Canada* ; ^*9*^*Barcelona Supercomputing Centre, Barcelona, Spain* ; ^*10*^*University of Calgary, Alberta, Canada* ; ^*11*^*University of California, San Francisco, San Francisco, CA, United States* ; ^*12*^*Leukemia Therapeutics, LLC, Hull, MA, United States* ; ^*13*^*INSERM, Sorbonne Universite ´ , Sorbonne Paris Cite, Universite ´ Paris Descartes, Universite ´ Paris Diderot, Centre de Recherche des Cordeliers, Paris, Paris, France* ; ^*14*^*Refuge Biotechnologies Inc., Menlo Park, CA, United States* ; ^*15*^*AbbVie Inc., Benevento, Italy*

####### **Correspondence:** Elad Ziv (elad.ziv@ucsf.edu), Davide Bedognetti (dbedognetti@sidra.org)

**Background**

Somatic genetic alterations have been associated with differential disposition of the intratumoral immune milieu. In contrast, the role of germline genetics remains largely unknown. The Cancer Genome Atlas (TCGA) Pan-Cancer Immune Response Working Group recently analyzed associations between immunological features of tumor microenvironment, prognosis, and tumor-intrinsic properties (including somatic mutations and copy number aberrations). The study generated a comprehensive set of per-sample immune response signatures, and identified distinct tumor immunological subtypes shared across multiple cancer types [1]. Here, we examined the germline genetic contribution to >100 such immune response signatures, considered as potential traits, in >9,000 study participants across 30 different cancer types in the TCGA.

**Methods**

We used SNP data from Affymetrix 6.0 arrays typed on normal tissue and blood. After stringent quality control, we imputed missing SNPs using the Haplotype Reference Consortium dataset and included SNPs with minor allele frequency >0.005 and imputation quality R2>0.5. We inferred genetic ancestry using principal components analysis. We estimated genome-wide heritability of the immune signatures using the genomic-relatedness-based restricted maximum-likelihood (GREML) approach implemented in genome-wide complex trait analysis (GCTA). We performed genome-wide association studies (GWAS) using linear regression models. All analyses were adjusted for cancer type, age at diagnosis, gender, and genetic ancestry using principal components 1-7. Linear regression was also applied to germline exome sequence data [2] to evaluate rare variant associations.

**Results**

Across different tumor types, we found significant heritability (FDR<0.05) for estimates of innate and adaptive immune cell enrichment including natural killer cells, activated dendritic cells, eosinophils and neutrophils, and T-cell subsets (CD8 Cytotoxic, T-helper, T-follicular helper, T-effector memory, T-central memory, Th1 and Th17 cells) respectively, as well as for antigen-presenting machinery and interferon-related signatures. Furthermore, we found significant interactions between germline modifiers and distinct immune subtypes (FDR<0.05). Through GWAS analysis, we identified several polymorphisms associated with both immune cells and immunomodulatory signaling passing the genome-wide significance threshold (p<5E-8). Two SNPs previously associated with the risk of several auto-immune diseases [3], rs2111485 and rs1990760 mapping to Interferon Induced Helicase C Domain 1 (IFIH1) locus, were significantly correlated with interferon signaling in tumors (Figure 1). Moreover, suggestive associations between rare variants and immune response traits were found.

**Conclusions**

We demonstrated that intratumoral immune disposition is partially under germline control through systematic pan-cancer analysis. Germline variants associated with differential immune response might be used to stratify patients based on likelihood of treatment response and to prioritize targets for development of novel therapies.

**References**

1. Thorsson V, et al. The Immune Landscape of Cancer. Immunity 2018; 48:812–830.

2. Huang KL, et al. Pathogenic Germline Variants in 10,389 Adult Cancers. Cell 2018; 173:355-370

3. Buniello A, et al. The NHGRI-EBI GWAS Catalog of published genome-wide association studies, targeted arrays and summary statistics 2019. Nucleic Acids Res. 2019; 47:D1005-D1012.

**Fig. 1 (abstract 054).**
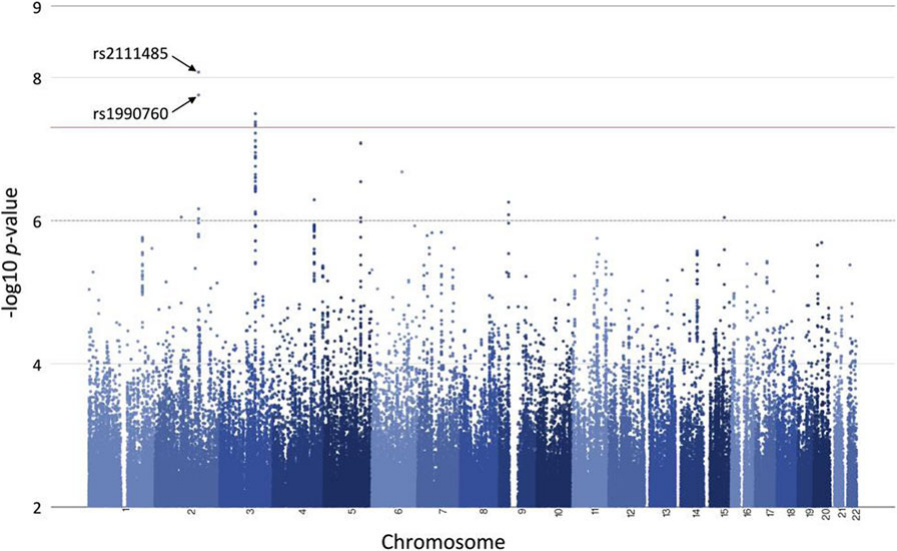
Germline variants associated with IFN signaling

#### O55 Antigen-cross presentation promotes the development of terminally differentiated CD8 T cells in young individuals

##### Ardiana Moustaki^1^, Shanta Alli, PhD^1^, Anthony Zamora^1^, Jeremy Crawford^1^, Paul Thomas^1^, Shannon Boi^1^, Natalie McDonald^2^, Alberto Pappo^1^, Michael Dyer^1^, Elizabeth Stewart^1^, Sara Federico^1^, Ben Youngblood^1^

###### ^*1*^*St Jude Children's Research Hospital, Memphis, TN, United States* ; ^*2*^*UTHSC, Memphis, TN, United States*

####### **Correspondence:** Ben Youngblood (Benjamin.Youngblood@STJUDE.ORG)

**Background**

Recent advances in T cell-based immunotherapies have revolutionized treatment strategies for several types of cancers, however, approaches that rely on endogenous T cell responses have had limited success in pediatric populations, attributed primarily to the low mutational burden of these tumors. Here we characterized CD8 T cells isolated from a wide range of pediatric solid tumor types and observed enrichment of cells with an antigen-experienced phenotype (high PD1 expression). The limited ability of immune checkpoint blockade therapy (ICB) to trigger anti-tumor responses in pediatric populations, despite the presence of activated CD8 T cells in these tumors, prompted us to explore alternative mechanisms restricting the endogenous T cell response.

**Methods**

Here we developed a transplantable mouse tumor model that expresses a well-characterized viral epitope, GP33, coupled to a mCherry marker.

**Results**

Using this new model, we document antigen cross-presentation by tumor-infiltrating myeloid cells as a key factor that drives suppression of CD8 T cell in tumors. Specifically, we show that tumor microenvironment (TME)-mediated suppression of CD8 T cell polyfunctionality occurs even in the absence of direct antigen-presentation by tumor cells, suggesting that antigen cross-presentation is sufficient to enforce the establishment of this dysfunctional state. More importantly, age-related changes in tumor microenvironment had a significant impact on the differentiation of tumor-specific CD8 T cells. Transplanting MHCI-deficient tumor cells in young or adult mice, we report a striking skewing of tumor-specific effector CD8 T cells towards a TCF7lowGzmBhi terminally differentiated state in peripheral lymphoid organs. This phenotype was associated with increased levels of antigen cross-presentation by tumor-infiltrating myeloid cells in young hosts. Consistent with our mouse findings, analysis of immune infiltrates from human pediatric solid tumors revealed a strong correlation between the expression of PDL1 on myeloid cells and enrichment of CD8 TILs with an exhaustion phenotype (PD1hiTim3+).

**Conclusions**

Collectively we report how the “immature/young” microenvironment of an actively developing tissue/individual contributes in the generation of an immune response skewed towards a terminally differentiated state with limited plasticity, thus narrowing the window for immunotherapeutic interventions.

**Acknowledgements**

This study was supported by the DBSTP Garwood Fellowship (SJCRH) to AM, and funding from ALSAC (American Lebanese Syrian Associated Charities) to BY.

**Trial Registration**

ClinicalTrials.gov Identifier: NCT01050296 (Molecular Analysis of Solid Tumors (MAST))

**Ethics Approval**

MAST protocol was approved by the St. Jude Children’s Research Hospital institutional review board (IRB), approval # XPD09-234

Animal protocols were approved on an annual term by the Institutional Animal Care and Use Committee (IACUC) at St. Jude Children’s Research Hospital.

#### O56 Expression of ART1, an extracellular mono ADP-ribosylase, promotes lung cancer growth and dissemination by limiting tumor infiltration of P2X7R+ CD8+ T cells and CD103+ dendritic cells

##### Erik Wennerberg, PhD, Clarey Hung, Amanda Valeta, Timothy McGraw, Sandra Demaria, MD, Brendon Stiles, MD

###### Weill Cornell Medicine, New York, NY, United States

####### **Correspondence:** Erik Wennerberg (erw2010@med.cornell.edu)

**Background**

NAD+ released from stressed and dying cells acts as a proinflammatory mediator [1]. In the presence of mono-ADP-ribosyltransferases (ARTs), extracellular NAD+ can serve as substrate for ADP-ribosylation of P2X7-receptors (P2X7Rs) expressed on immune cells, including CD8+ T cells and CD103+ dendritic cells (DCs) which are essential for immune-mediated tumor rejection, resulting in NAD-induced cell death (NICD) [2]. NICD of P2X7R-expressing tissue-resident lymphocytes was recently described as an important regulator of tissue homeostasis [3]. However, involvement of ART-mediated NICD in cancer and its implications for anti-tumor immunity remain uninvestigated. We hypothesized that ART1-expression in non-small cell lung cancer (NSCLC) would limit immune-mediated tumor control by eliminating tumor-infiltrating P2X7R+ immune cells.

**Methods**

Human NSCLC tumor samples and matched adjacent normal tissue (n=40) were analyzed for ART1 expression by qPCR. In addition, a lung adenocarcinoma tissue microarray (TMA) (n=493) was scored for ART1 expression. The KP1 cell line was derived from lung tumors of KRASG12D/P53-/- mice. KP1-shART1 cells with doxycycline (DOX)-inducible knockdown of ART1 were generated by lentiviral constructs. In an orthotopic lung tumor model, C57BL/6 mice were intravenously injected with KP1-shART1 cells, fed DOX-water ad libitum and sacrificed after 14 days. Lung tumor nodules were enumerated from H&E-stained FFPE lung sections. Alternatively, lung-derived cell suspensions were analyzed for infiltration of T cells and DCs by flow cytometry.

**Results**

ART1 expression in human NSCLC tissue was >2-fold higher compared to normal tissue. TMA analysis revealed that 55% of tumors strongly expressed ART1. ART1-knockdown by DOX-treatment resulted in a 75% decrease in lung metastatic nodules compared to DOX-untreated mice (p<0.001). P2X7R was highly expressed on CD8+ T cells and CD103+ DCs derived from KP1-shART-bearing lungs. Importantly, ART1-knockdown significantly increased the percentage of CD8+ T cells (23.8% in DOX+ vs 12.9% in DOX-, p<0.05) and CD103+ DCs (20.7% in DOX+ vs 12.7% in DOX-, p<0.05) among total T cells and DCs respectively. In vitro, we confirmed that P2X7R+ CD8+ T cells were preferentially susceptible to ADP-ribosylation and NICD compared with P2X7R- CD8+ T cells. In line with our hypothesis that ART1 expression counteracts immune-mediated tumor control, ART1-knockdown significantly delayed growth of subcutaneous tumors in wild type mice while it had markedly reduced differential effect on tumor progression in nude or CD8-depleted mice.

**Conclusions**

Our findings indicate that ART1 tumor expression constitutes a barrier to immune-mediated control of NSCLC tumors. This warrants further studies to determine whether targeted ART1-blockade can potentiate the response to immunotherapy of ART1-expressing NSCLC.

**Acknowledgements**

This work was supported by a Department of Defense, Breast Cancer Research Program Breakthrough Fellowship Award (W81XWH-17-1-0029) and grants from the Lung Cancer Research Foundation (Free to Breathe) and the Graham Foundation of the American Association for Thoracic Surgery. Funding also provided by the Meyer Cancer Center, Pre-SPORE funding mechanism, “Using Radiotherapy to jump-start cancer response to immunotherapy.”

**References**

1. Haag F, Adriouch S, Brass A, Jung C, Moller S, Scheuplein F, et al. Extracellular NAD and ATP: Partners in immune cell modulation. Purinergic Signal 2007;3(1-2):71-81

2. Hildner K, Edelson BT, Purtha WE, Diamond M, Matsushita H, Kohyama M, et al. Batf3 deficiency reveals a critical role for CD8alpha+ dendritic cells in cytotoxic T cell immunity. Science 2008;322(5904):1097-100

3. Stark R, Wesselink TH, Behr FM, Kragten NAM, Arens R, Koch-Nolte F, et al. T RM maintenance is regulated by tissue damage via P2RX7. Sci Immunol 2018;3(30)

**Ethics Approval**

All patient samples were obtained following informed consent at the Cardiothoracic Surgery Department of Weill Cornell Medical College under Biobank protocol 1008011221, which was approved by the Institutional Review Board at Weill Cornell Medical College. All mouse experiments were approved by the Institutional Animal Care and Use Committee (IACUC) at Weill Cornell Medical College.

#### O57 Novel dsRNA-sensing dendritic cells enhance anti-tumor immunity

##### Ellen Duong, ScB, Stefani Spranger, PhD

###### Massachusetts Institute of Technology, Cambridge, MA, United States

####### **Correspondence:** Stefani Spranger (spranger@mit.edu)

**Background**

Myeloid cells can profoundly shape the landscape of tumor-infiltrating T cells. For example, tumor-resident cross-presenting CD103^+^ dendritic cells (DC1) recruit effector T cells into the tumor microenvironment (TME) and provide local T cell stimulation [1-2]. Boosting DC numbers or function enhances responses to checkpoint blockade therapy, suggesting that DC may contribute to the reactivation of exhausted or dysfunctional T cells or even prevent their induction [3]. Elucidating the DC subsets that associate with productive T cell responses in the TME will open new therapeutic opportunities to enhance anti-tumor immunity.

**Methods**

To identify DC subsets that correlate with functional T cell responses, we compared the myeloid infiltrate of an acutely cleared regressor tumor with that of a progressively growing tumor, an approach that was previously used to phenotype dysfunctional T cells in the TME [4]. Murine syngeneic tumor lines expressing SIY were implanted in wild-type, Rag2^-/-^, Batf3^-/-^, Clec9a^-/-^, CD11c-DTR bone marrow chimera, Ifnar1^-/-^, and Sting^-/-^ mice. Flow immunophenotyping was used to profile the myeloid compartment, and ELISpot was performed to quantify antigen-specific T cells. To assess function, sorted myeloid cells were co-cultured with T cells to evaluate their ability to induce T cell proliferation. Single cell RNA-sequencing was performed to profile the immune infiltrate of the regressor tumor.

**Results**

Compared to progressor tumors, regressor tumors showed a higher number of CD103^+^ DC with cell surface markers that phenotypically resembled canonical cross-presenting DC1. Yet, Batf3^-/-^ and Clec9a^-/-^ mice, which lack functional DC1, were still able to mount tumor-reactive T cell responses and eliminate regressor tumors. Functional assays with sorted antigen-presenting cells from regressor tumors in Batf3^-/-^ mice indicated that the CD11c^+^ DC compartment was uniquely able to activate T cells, and specific depletion of CD11c^+^ cells ablated T cell responses. We found that these DC required sensing of type I interferons to mediate anti-tumor immunity but were independent of cytosolic dsDNA sensing via the cGAS-STING pathway. Single-cell RNA sequencing of myeloid cells identified a novel DC subset characterized by interferon stimulated genes (ISG). ISG^+^ DC highly express the cytosolic dsRNA sensors, RIG-I and MDA5, suggesting sensing of tumor-derived dsRNA. T cells primed by ISG^+^ DC in Batf3^-/-^ mice induced protective systemic immunity against progressor tumors.

**Conclusions**

We identified a novel DC subset characterized by an ISG signature that can activate T cells through means independent of canonical cross-presentation, leading to systemic immunity. Engaging these cells provides a therapeutic opportunity to boost anti-tumor immune responses.

**References**

1. Spranger et al. Melanoma-intrinsic β-catenin signaling prevents anti-tumour immunity. Nature. 2015;523(7559):231-5.

2. Broz et al. Dissecting the tumor myeloid compartment reveals rare activating antigen-presenting cells critical for T cell immunity. Cancer Cell. 2014;26(5):638-52.

3. Salmon et al. Expansion and activation of CD103+ dendritic cell progenitors at the tumor site enhances tumor responses to therapeutic PD-L1 and BRAF inhibition. Immunity. 2016;44(4):924-938.

4. Williams et al. The EGR2 targets LAG-3 and 4-1BB describe and regulate dysfunctional antigen-specific CD8+ T cells in the tumor microenvironment. J Exp Med. 2017;214(2):381-400.

#### O58 Human tumor-infiltrating MAIT cells display hallmarks of antigen-recognition that are associated with tumor bacterial infiltration

##### Shamin Li, PhD, Evan Newell, PhD

###### Fred Hutchinson Cancer Research Center, Seattle, WA, United States

####### **Correspondence:** Evan Newell (evannewell@gmail.com)

**Background**

Growing evidence indicates the importance of microbiota in cancer therapy, and bacteria are established to be capable of modulating anti-tumor efficacy [1]. Here, we study Mucosal-associated invariant T (MAIT) cells, a group of innate-like T cells expressing a semi-invariant TCR and recognizing riboflavin metabolites produced by many species of bacteria [2,3]. We explore whether these cells could recognize bacterial antigens in the tumors and whether this response could be microbiome-modulated.

**Methods**

We use mass-cytometry to deeply profile MAIT cells in human colorectal (CRC), lung (NSCLC) and kidney (RCC) cancers. Focusing particularly on colorectal cancer, we investigate the relationship between MAIT cells and the tumor microbiome by estimating the microbial composition in each bulk tumor using whole genome sequencing data.

**Results**

We observe that tumor-infiltrating MAIT cells from CRC are highly enriched for CD39 expression (Figure 1), a marker recently reported to distinguish between bystander and tumor-specific T cells [4], and show that in vitro, CD39 expression is induced in a TCR-dependent manner (Figure 2). We further show a distinct phenotype for MAIT cells from tumor, adjacent tissue and PBMC of CRC patients (Figure 3). Particularly, CD39+ tumor-infiltrating MAIT cells are characterized by an exhaustive phenotype profile as compared to the CD39- subset, with higher expression of the inhibitory receptors CTLA-4 and PD-1 (Figure 4). They are also more proliferative, less apoptotic and polyfunctional than the CD39- MAIT cells. Unexpectedly, we identify a subset of CD39+ MAIT cells expressing CD4 and Foxp3 within the tumors capable of producing pro-inflammatory cytokines. By sequencing CRC tumors, we observe a positive correlation between CD39+ MAIT cell frequency and tumor bacterial load, suggesting a microbiome-influenced tumor microenvironment in shaping the MAIT cell phenotype. This hypothesis is supported by a higher CD39 expression observed on tumor-infiltrating MAIT cells from CRC as compared to NSCLC and RCC, known to be less infiltrated by bacteria (Figure 5). Among the identified bacterial strains, we show that Fusobacteria nucleatum is able to activate MAIT cells in vitro in a TCR-specific way (Figure 6).

**Conclusions**

Our results show unique features of MAIT cells in tumors with evidence of being influenced by TCR-mediated signals, and highlight a bacterial strain that could potentially shape MAIT cell response in CRC. This work will not only provide new understanding in innate-like T cell responses in tumors, but also pave the way for manipulating these cells for cancer prevention and therapeutics.

**References**

1. Helmink BA, Khan MAW, Hermann A, Gopalakrishnan V, Wargo JA. The microbiome, cancer, and cancer therapy. Nat Med. 2019;25(3):377-88.

2. Kjer-Nielsen L, Patel O, Corbett AJ, Le Nours J, Meehan B, Liu L, et al. MR1 presents microbial vitamin B metabolites to MAIT cells. Nature. 2012;491(7426):717-23.

3. Corbett AJ, Eckle SB, Birkinshaw RW, Liu L, Patel O, Mahony J, et al. T-cell activation by transitory neo-antigens derived from distinct microbial pathways. Nature. 2014;509(7500):361-5.

4. Simoni Y, Becht E, Fehlings M, Loh CY, Koo SL, Teng KWW, et al. Bystander CD8(+) T cells are abundant and phenotypically distinct in human tumour infiltrates. Nature. 2018;557(7706):575-9.

**Ethics Approval**

The use of human samples from CRC and NSCLC patients was approved by the appropriate institutional research boards, A*STAR and the Singapore Immunology Network, Singapore. The RCC samples were provided by Northwest Biotrust, under a NWBiospecimens protocol, Seattle.The analysis was performed according to the IRB file/approval number NHS #6007-1061.

**Fig. 1 (abstract 058).**
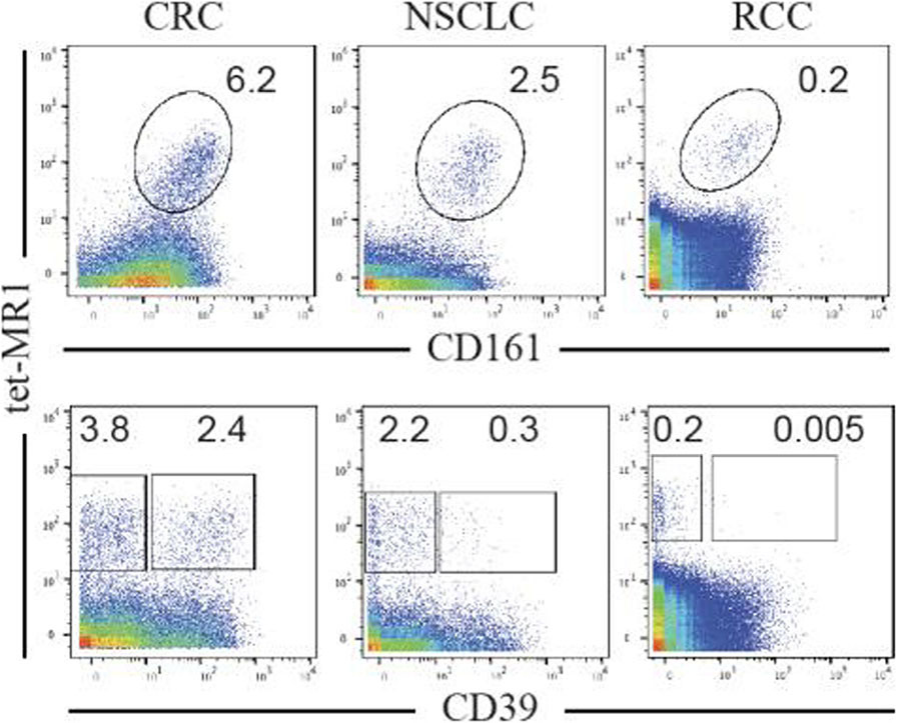
Enriched CD39 expression on MAIT cells from CRC.

**Fig. 2 (abstract 058).**
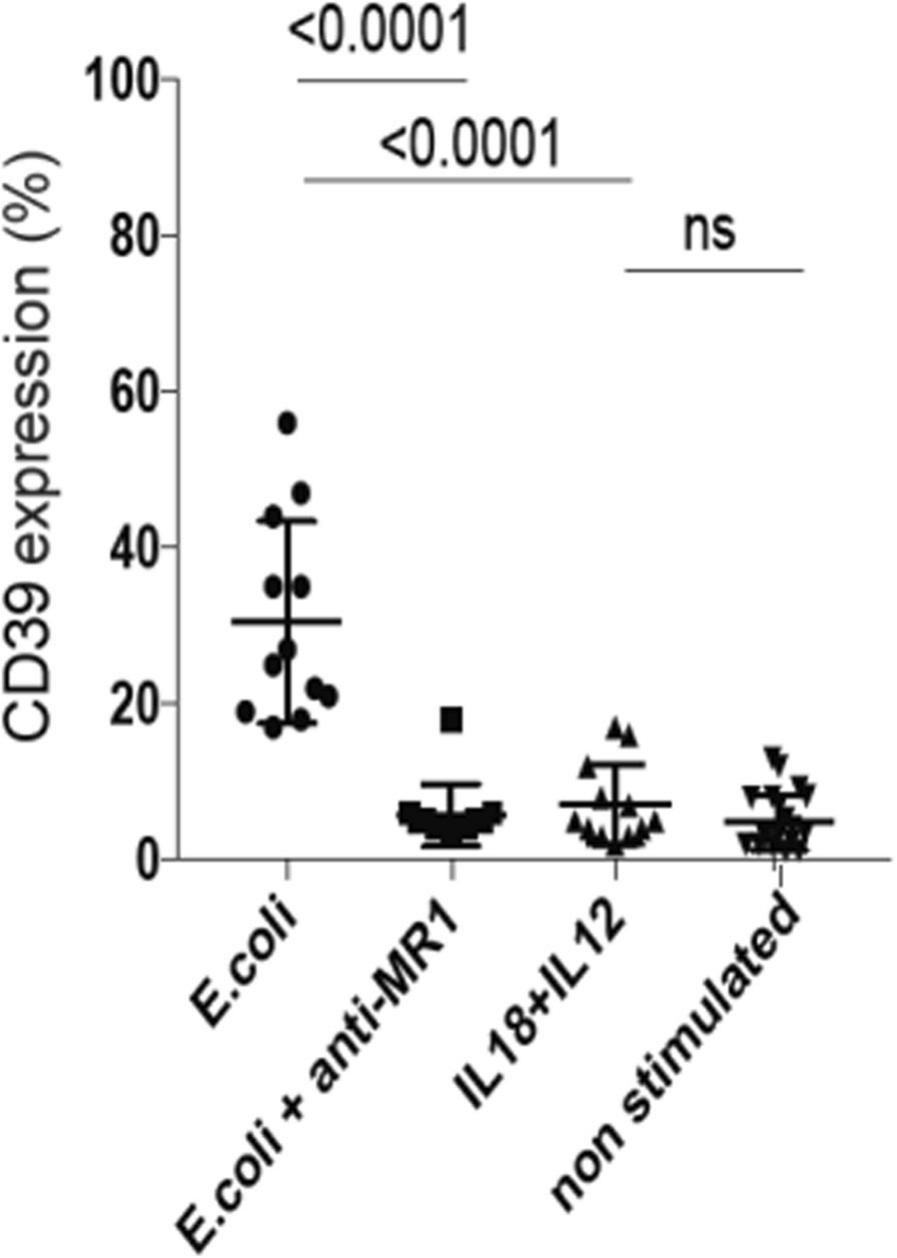
CD39 expression on MAIT cells is TCR-dependent.

**Fig. 3 (abstract 058).**
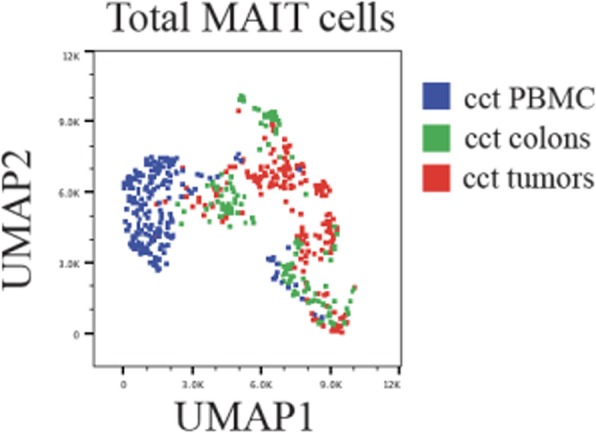
Distinct phenotype of MAIT cells in CRC.

**Fig. 4 (abstract 058).**
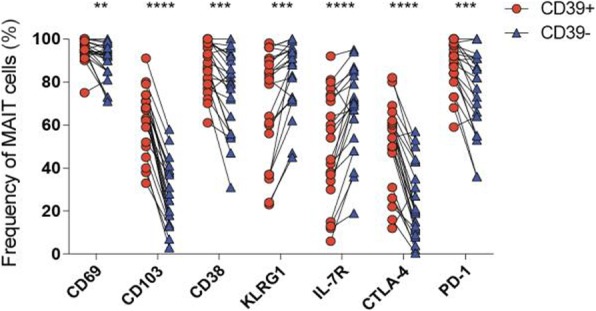
Characterization of CD39+ and CD39- tumor MAIT cells.

**Fig. 5 (abstract 058).**
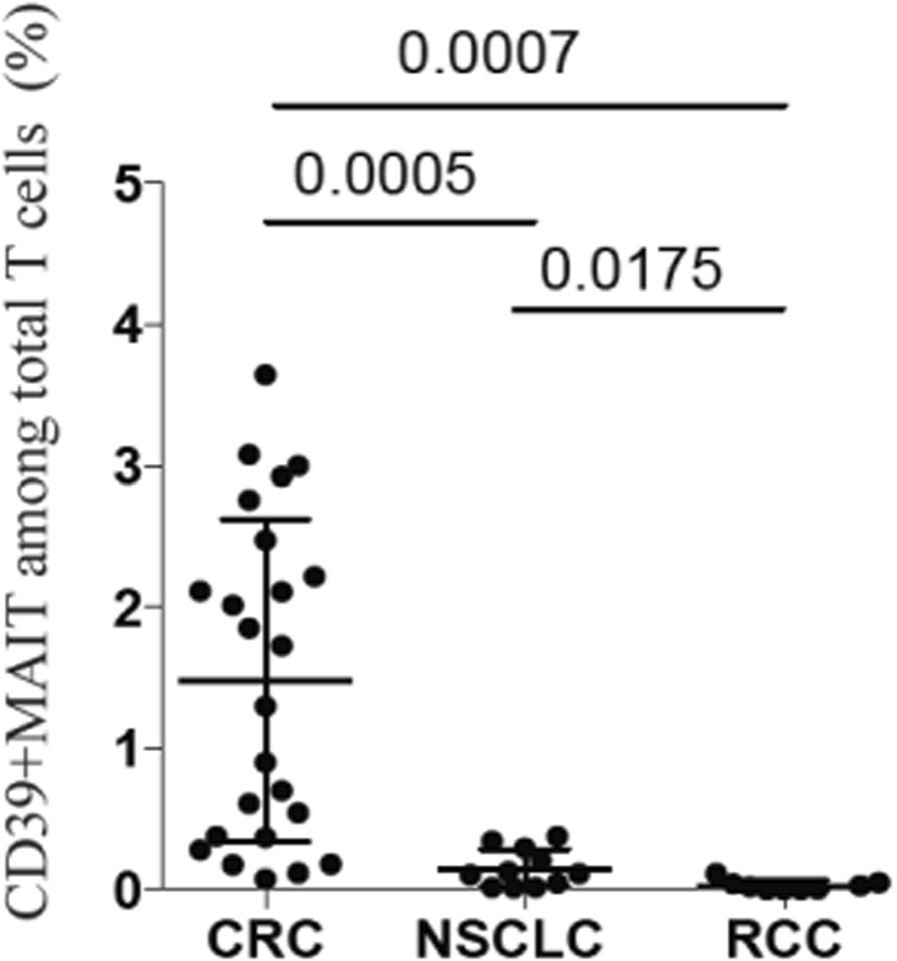
Low expression of CD39 on MAIT cells from NSCLC and RCC.

**Fig. 6 (abstract 058).**
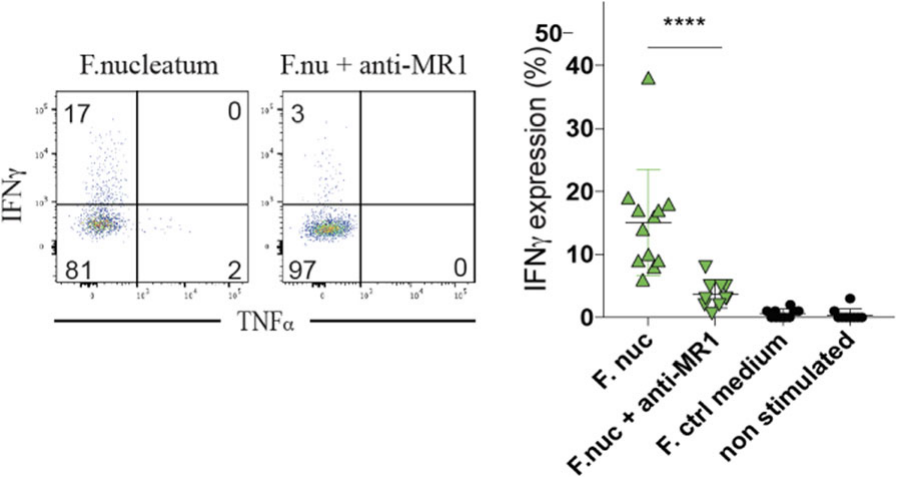
Fusobacteria nucleatum activate MAIT cells in vitro.

#### O59 CD8 T-cell infiltration into renal tumors requires a supportive antigen presenting niche and is independent of PD-L1 status

##### Caroline Jansen, BS^1^ , Nataliya Prokhnevska, BS^1^, Viraj Master, MD, PhD^1^, Jennifer Carlisle, MD^1^, Mehmet Bilen^1^, Maria Cardenas^1^, Scott Wilkinson, PhD^2^, Ross Lake^2^, Adam Sowalsky, PhD^2^, Adeboye Osunkoya, MD^1^, Patrick Mullane, MD^1^, Carla Ellis^1^, Adriana Reyes^1^, Yuan Liu, PhD^1^, Haydn Kissick^1^

###### ^1^Emory University, Decatur, GA, United States; ^2^National Cancer Institute, Bethesda, MD, United States

####### **Correspondence:** Caroline Jansen (carey.jansen@emory.edu)

**Background**

Tumor infiltrating T-cells have a clear prognostic benefit in many tumor types. Immuno-biomarkers have independently improved prognostication in various cancer types, with tumor-infiltrating lymphocytes (TILs) more accurately predicting patient survival than currently employed methods [1-4]. Recent studies have highlighted similar observations in breast cancer, lung cancer, and melanoma [5-7]. These observations raise the question of whether the level of T-cell infiltration into renal tumors predicts patient outcomes, and more fundamentally, why some patients mount a strong immune response to their tumors and others do not.

**Methods**

Tumor tissue was collected from renal cell carcinoma (RCC) patients undergoing surgery at Emory University Hospital. Minimum follow up time was 24 months. Intraoperative tumor samples were analyzed by flow cytometry, immunofluorescence, and immunohistochemistry.

**Results**

The proportion of CD8 TILs, measured by flow cytometry, varied widely in RCC patients (Fig 1A). A greater proportion of tumor CD8 T-cells was associated with improved progression-free survival (PFS) (Fig 1B), and this association was particularly strong in patients with advanced disease.

In examining the phenotype and function of these TILs, the presence of a stem-like CD8 T-cell—that both proliferates and differentiates—was found to be required for the generation of a strong anti-tumor T-cell response. Without stem-like T-cells, the anti-tumor response deteriorates, and patient PFS declines. The presence of dendritic cells in the tumor correlates with T-cell infiltration, and quantitative immunofluorescence revealed that stem-like CD8 T-cells reside in areas of high antigen presenting cell (APC) density (Fig 1C). Tumors lacking in TILs also lack APC density, suggesting that an APC niche is necessary to support the T-cell response. Furthermore, T-cell dense tumors harbor increased vascular and stromal cells, suggesting a potential role for non-hematopoietic cells in the tumor microenvironment in supporting the anti-tumor immune response.

Interestingly, PD-L1 status did not correlate with T-cell infiltration (Fig 1D), and there was no difference in progression free survival between patients with PD-L1+/- tumors (Fig 1E), suggesting that the described immune infiltration and organization may be a prevailing factor in predicting patient outcomes.

**Conclusions**

CD8 T-cell infiltration in RCC predicts PFS, particularly in patients with advanced disease. Improved prognostication is a critical goal for these patients, so these findings represent an opportunity to inform future development of prognostic tools and of reduction/intensification of therapy.

T-cell infiltration is maintained by a population of stem-like T-cells, which require a supportive niche inside the tumor. Without this niche, the immune response declines, resulting in disease progression.

**Acknowledgements**

This work was supported by funding from the Prostate Cancer Foundation, Swim Across America, and NCI grant 1-R00-CA197891. We would like to

acknowledge The Yerkes NHP Genomics Core which is supported in part by NIH P51 OD011132, the Emory Flow Cytometry Core (EFCC) supported by the National Center for Georgia Clinical & Translational Science Alliance of the National Institutes of Health under Award Number UL1TR002378, the Intramural Research Program of the NIH, National Cancer Institute, and the Emory University Integrated Cellular Imaging Microscopy Core of the Winship Cancer Institute of Emory University and NIH/NCI under award number, 2P30CA138292-04.

**References**

1. Galon J, Costes A, Sanchez-Cabo F, Kirilovsky A, Mlecnik B, Lagorce-Pages C, Tosolini M, Camus M, Berger A, Wind P, Zinzindohoue F, Bruneval P, Cugnenc PH, Trajanoski Z, Fridman WH, Pages F. Type, density, and location of immune cells within human colorectal tumors predict clinical outcome. Science. 2006;313(5795):1960-4. Epub 2006/09/30. doi: 10.1126/science.1129139. PubMed PMID: 17008531.

2. Mlecnik B, Bindea G, Angell HK, Maby P, Angelova M, Tougeron D, Church SE, Lafontaine L, Fischer M, Fredriksen T, Sasso M, Bilocq AM, Kirilovsky A, Obenauf AC, Hamieh M, Berger A, Bruneval P, Tuech JJ, Sabourin JC, Le Pessot F, Mauillon J, Rafii A, Laurent-Puig P, Speicher MR, Trajanoski Z, Michel P, Sesboue R, Frebourg T, Pages F, Valge-Archer V, Latouche JB, Galon J. Integrative Analyses of Colorectal Cancer Show Immunoscore Is a Stronger Predictor of Patient Survival Than Microsatellite Instability. Immunity. 2016;44(3):698-711. doi: 10.1016/j.immuni.2016.02.025. PubMed PMID: 26982367.

3. Tosolini M, Kirilovsky A, Mlecnik B, Fredriksen T, Mauger S, Bindea G, Berger A, Bruneval P, Fridman WH, Pages F, Galon J. Clinical impact of different classes of infiltrating T cytotoxic and helper cells (Th1, th2, treg, th17) in patients with colorectal cancer. Cancer research. 2011;71(4):1263-71. doi: 10.1158/0008-5472.CAN-10-2907. PubMed PMID: 21303976.

4. Pages F, Berger A, Camus M, Sanchez-Cabo F, Costes A, Molidor R, Mlecnik B, Kirilovsky A, Nilsson M, Damotte D, Meatchi T, Bruneval P, Cugnenc PH, Trajanoski Z, Fridman WH, Galon J. Effector memory T cells, early metastasis, and survival in colorectal cancer. The New England journal of medicine. 2005;353(25):2654-66. doi: 10.1056/NEJMoa051424. PubMed PMID: 16371631.

5. Savas P, Virassamy B, Ye C, Salim A, Mintoff CP, Caramia F, Salgado R, Byrne DJ, Teo ZL, Dushyanthen S, Byrne A, Wein L, Luen SJ, Poliness C, Nightingale SS, Skandarajah AS, Gyorki DE, Thornton CM, Beavis PA, Fox SB, Kathleen Cuningham Foundation Consortium for Research into Familial Breast C, Darcy PK, Speed TP, Mackay LK, Neeson PJ, Loi S. Single-cell profiling of breast cancer T cells reveals a tissue-resident memory subset associated with improved prognosis. Nature medicine. 2018;24(7):986-93. doi: 10.1038/s41591-018-0078-7. PubMed PMID: 29942092.

6. Peranzoni E, Lemoine J, Vimeux L, Feuillet V, Barrin S, Kantari-Mimoun C, Bercovici N, Guerin M, Biton J, Ouakrim H, Regnier F, Lupo A, Alifano M, Damotte D, Donnadieu E. Macrophages impede CD8 T cells from reaching tumor cells and limit the efficacy of anti-PD-1 treatment. Proceedings of the National Academy of Sciences of the United States of America. 2018;115(17):E4041-E50. doi: 10.1073/pnas.1720948115. PubMed PMID: 29632196; PMCID: 5924916.

7. Azimi F, Scolyer RA, Rumcheva P, Moncrieff M, Murali R, McCarthy SW, Saw RP, Thompson JF. Tumor-infiltrating lymphocyte grade is an independent predictor of sentinel lymph node status and survival in patients with cutaneous melanoma. Journal of clinical oncology : official journal of the American Society of Clinical Oncology. 2012;30(21):2678-83. doi: 10.1200/JCO.2011.37.8539. PubMed PMID: 22711850.

**Ethics Approval**

Samples are collected under an approved IRB protocol (The Urological Satellite Specimen Bank at Emory University, IRB00055316), and all patients provided informed consent.

**Fig. 1 (abstract 059).**
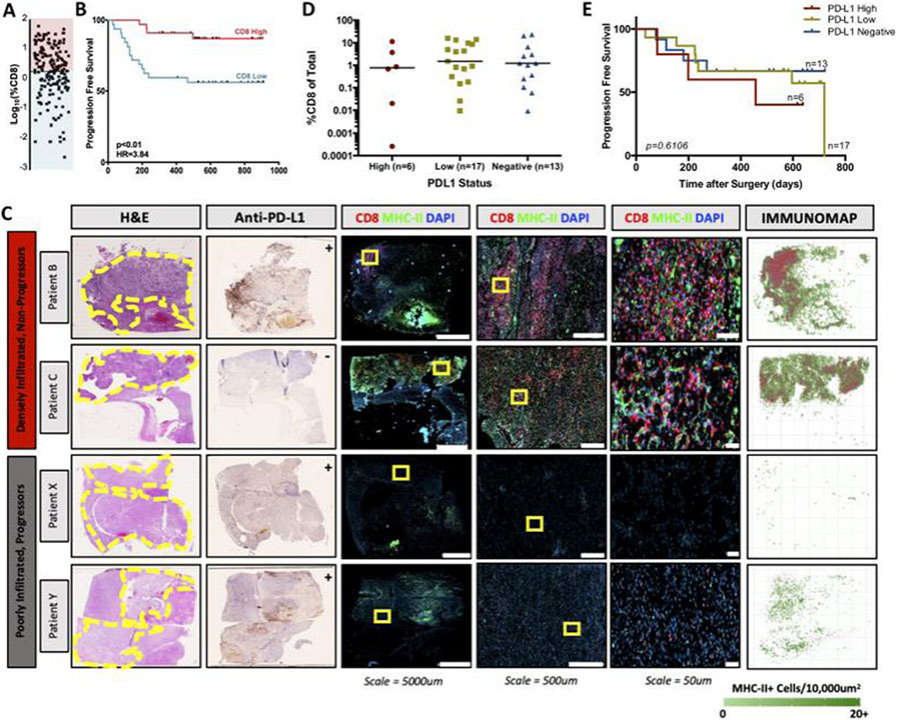
See text for description

### Immune-Stimulants and Immune Modulators

#### O60 Tumor cell-intrinsic defects in STING pathway signaling and IFN-β gene regulation

##### Blake Flood, BS^1^, Leticia Corrales^2^, Thomas Gajewski, MD, PhD^1^

###### ^*1*^*University of Chicago, Chicago, IL, United States* ; ^*2*^*Aduro, Berkeley, CA, United States*

####### **Correspondence:** Thomas Gajewski (tgajewsk@medicine.bsd.uchicago.edu)

**Background**

Our laboratory has previously shown that immunogenic tumors activate the innate immune system through the host STING pathway. The STING pathway senses cytosolic DNA, which activates a signal transduction pathway culminating in IRF3 activation that induces expression of several genes including IFN-β. STING signaling and IFN-β receptor signaling in tumor-infiltrating immune cells, in turn, are required for optimal priming of tumor-specific CD8+ T cells. Consequently, STING agonists have been developed as a pharmacologic approach to activate the pathway. However, the functional role of STING signaling within cancer cells is incompletely understood.

**Methods**

We stimulated tumor cell lines with STING agonists to test their ability to produce IFN-β. Further biochemical techniques including western blotting, immunofluorescent imaging, ChIP assays, and ATAC-sequencing were used to evaluate each step of the STING pathway that might be defective.

**Results**

We observed that most tumor cells themselves fail to produce IFN-β in response to STING agonists or cytoplasmic DNA, arguing loss of this signaling capacity might occur regularly as a component of oncogenesis. Surprisingly, tumor cells retained STING pathway signaling up to and including nuclear translocation of IRF3. ChIP assays in tumor cells demonstrated failure of IRF3 to bind the IFN-β promoter. To test if this was dominant or recessive, we generated hybrid cells between B16 melanoma and Mφs. These hybrids retained nuclear IRF3 translocation but failed to express IFN-β indicating a dominant-negative mechanism. Sequencing revealed no mutations in the IFN-β promoter. However, ATAC-sequencing indicated the IFN-β promoter remained epigenetically closed after STING pathway activation in B16 tumor cells, unlike Mφ controls. NF-κB is an innate immune pathway proposed to control IFN-β locus accessibility. We found B16 tumor cells and hybrids were defective in NF-κB nuclear translocation, unlike Mφ controls. Furthermore, tumor cells expressed normal levels of most NF-κB factors but over expressed the negative regulator IκBα. Combined treatment with cycloheximide and a STING agonist caused IκBα degradation and tumor cell-intrinsic IFN-β expression.

**Conclusions**

We find tumor cells fail to express IFN-β downstream of STING pathway activation. This is likely due to a dominant-negative inhibitor of NF-κB signaling, such as IκBα, which causes the IFN-β locus to remain epigenetically repressed in tumor cells and prevents IRF3 binding. We hypothesize restoring NF-κB signaling in tumor cells will enable tumor cell-intrinsic IFN-β expression and promote tumor rejection in vivo. Therapies based on identifying and reversing tumor-specific defects in IFN-β expression represent a strategy to induce tumor site-specific innate immune activation.

#### O61 Myeloid cell-targeted miR-146a mimic alleviates NF-κB-driven cytokine storm without interfering with CD19-specific CAR T cell activity against B cell lymphoma

##### Marcin Kortylewski, PhD^1^, Yu-Lin Su^2^, Xiuli Wang, PhD^2^, Mati Mann^3^, Dayson Moreira, PhD^2^, Zhuoran Zhnag^2^, Ching Ouyang^2^, Piotr Swiderski^2^, Stephen Forman, MD^2^, David Baltimore, PhD^3^, Ling Li^2^, Guido Marcucci^2^, Mark Boldin^2^

###### ^*1*^*Beckman Research Institute, City of Hope, Duarte, CA, United States* ; ^*2*^*City of Hope*, *Duarte, CA, United States* ; ^*3*^*Caltech, Pasadena, CA, United States*

####### **Correspondence:** Marcin Kortylewski (mkortylewski@coh.org)

**Background**

NF-κB is a key regulator of inflammation, myeloproliferation and cancer progression, with an important role in leukemogenesis. Despite therapeutic potential, targeting NF-κB proved challenging. However, in non-malignant myeloid cells NF-κB activity is tightly regulated through many molecular mechanisms, including miRNA.

**Methods**

Here, we describe an original approach to NF-κB inhibition using miR146a, which targets upstream regulators of NF-κB signaling. We generated a myeloid cell-targeted NF-κB inhibitor by tethering a chemically-modified miR146a mimic oligonucleotide to a scavenger receptor (SR)/Toll-like receptor 9 (TLR9) ligand (C-miR146a).

**Results**

Unlike an unconjugated miR-146a molecule, C-miR146a was rapidly internalized and delivered to cytoplasm of target myeloid cells such as macrophages or myeloid leukemia cells. C-miR146a reduced protein levels of classic miR-146a targets, IRAK1 and TRAF6, thereby efficiently blocking NF-κB activation in target cells. Intravenous injections of C-miR146a mimic to miR-146-deficient mice prevented excessive NF-κB activation in myeloid cells, thereby alleviating myeloproliferation and exaggerated inflammatory responses to bacterial challenge. The NF-κB-driven release of IL-1 and IL-6 from monocytes is known to be responsible for cytokine release syndrome (CRS), which can occur in response to bacterial infections, antibody-based therapies and relatively frequently as a serious adverse effect of chimeric antigen receptor (CAR) T-cell therapies. While low expression of miR146a has not yet been implicated in CRS, C-miR146a treatments did reduce pro-inflammatory activity of human monocytes, at the level of IL-1 and IL-6 production, induced by the CD19-specific but not by the naive CAR T cells in vitro. Repeated systemic administration of C-miR146a oligonucleotide alleviated human monocyte-dependent CRS in xenotransplanted B-cell lymphoma model without impeding the on-target therapeutic effects of CAR T-cells against lymphoma cells.

**Conclusions**

Our results demonstrate potential of using myeloid cell-targeted miR146a mimics for treatment of inflammatory diseases and prevention of potential side effects of immunotherapies. The SR/TLR9-targeted miR-146a mimic design provides an outline for the development of miRNA therapeutics for a variety of myeloid cell-related diseases.

**Acknowledgements**

This work was supported in part by the National Cancer Institute/NIH awards R01CA213131 (to M.K.), Lymphoma SPORE P50CA107399 (to S.F.) and P30CA033572 (to the COH).

### Immuno-Conjugates and Chimeric Molecules

#### O62 Targeting myeloid tumors by Off-the-Shelf NK cells using an NKG2C-IL15-CD33 Trispecific Killer Engager

##### Emily Chiu, BA^1^, Martin Felices^1^, Frank Cichocki, PhD^1^, Zachary Davis, PhD^1^, Hongbo Wang^1^, Katie Tuininga^1^, Daniel Vallera^1^, Tom Lee, PhD^2^, Ryan Bjordahl^2^, Dan Kaufman, CAMD, PhD^3^, Karl Johan Malmberg^4^, Bahram Valamehr^2^, Jeffrey Miller, MD^1^

###### ^*1*^*University of Minnesota, Minneapolis, MN, United States* ; ^*2*^*Fate Theraputics, San Diego, United States* ; ^*3*^*University of California-San Diego, La Jolla, CA, United States* ; ^*4*^*Karolinska, Stockholm, Sweden*

####### **Correspondence:** Jeffrey Miller (mille011@umn.edu)

**Background**

Allogeneic NK cell infusions can achieve remission in 30-50% of patients with relapsed/refractory acute myeloid leukemia (AML). This strategy may be limited by NK cell heterogeneity and lack of specificity. NK cells mediate antibody-dependent cellular cytotoxicity of tumors coated with therapeutic antibodies through the CD16 receptor. While a strong activation receptor, CD16 is also present on neutrophils, which represent a sink for CD16-driven therapies. To bypass this issue, we elected to target NKG2C, an activating receptor with better specificity which is present on more differentiated NK cells. CD94/NKG2C is a heterodimeric receptor that binds to HLA-E and associates with DAP12. NKG2C+ cells are enriched in AML patients who have undergone a hematopoietic stem cell transplant and reactivated CMV. [1] NKG2C+ NK cells are more responsive to AML and patients with higher circulating NKG2C+ cells have improved relapse free survival. [2]

**Methods**

We aimed to direct specific killing and proliferation of the NKG2C+ cells using a NKG2C1533 Tri-specific Killer Engager (TriKE), containing single chain variable fragments specific for NKG2C (on NK cells) and CD33 (on AML cells), and an IL15 moiety (to support NK cell survival and proliferation). An obstacle to utilizing NKG2C is the frequency of NKG2C+ cells, so we genetically modified induced pluripotent stem cells (iPSCs) to stably overexpress NKG2C alone or along with DAP12. We then differentiated these cells to NK (iNK) cells.

**Results**

In the presence of AML cell lines, NKG2C1533 TriKE activated NK cells from transplant patients with CMV reactivation, where NKG2C+ frequency correlated with activation (Figure 1A,B). The NKG2C1533 TriKE also led to preferential expansion of NKG2C+ cells (Figure 1C). We found that the NKG2C1533 TriKE mediated robust activation of NKG2C iNK cells and NKG2C/DAP12 iNK cells leading to THP1 cell killing (Figure 1D). The NKG2C/DAP12 iNKs exhibited the strongest response.

**Conclusions**

Engaging NK cells through NKG2C or genetically modified iNK cells expressing NKG2C, will be more specific than targeting through CD16, which will bind to CD16A on NK cells but also have off-target binding to CD16B on neutrophils. The NKG2C1533 TriKE is an effective way to selectively target NK cells with two applications, one in individuals with high frequencies of NKG2C+ cells and another within the iNK cell platform creating an “off-the-shelf” NK cellular therapy that is targeted, specific and efficacious where TriKE can be co-administered or in the future, secreted by the engineered iNK cell itself.

**References**

1. Foley B, Cooley S, Verneris MR, Pitt M, Curtsinger J, Luo X, et al. Cytomegalovirus reactivation after allogeneic transplantation promotes a lasting increase in educated NKG2C+ natural killer cells with potent function. Blood 2012;119:2665–74.

2. Cichocki F, Cooley S, Davis Z, DeFor TE, Schlums H, Zhang B, et al. CD56dimCD57+NKG2C+ NK cell expansion is associated with reduced leukemia relapse after reduced intensity HCT. Leukemia 2016;30:456–63.

**Ethics Approval**

The study was approved by the University of Minnesota's IRB, approval number 9709M00134

**Fig. 1 (abstract 062).**
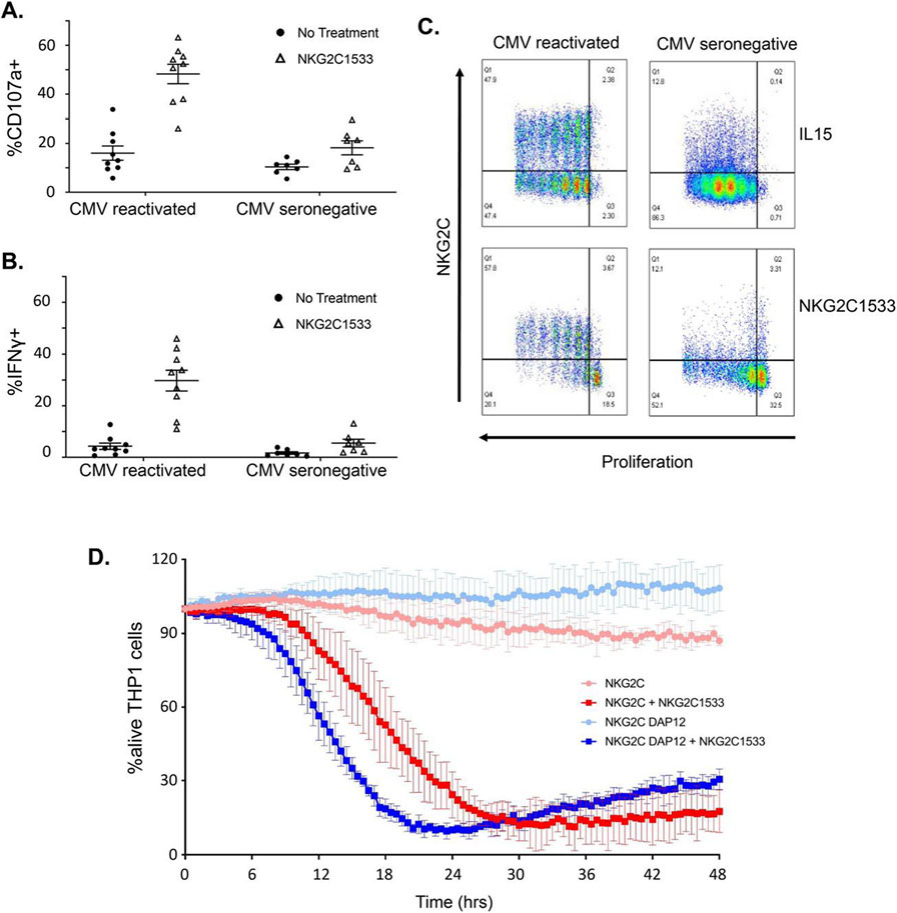
NKG2C1533 specifically activates NKG2C+ cells

### Immunotherapy Toxicities

#### O63 Gastric toxicity associated with PD-1 blockade therapy revealed by Multiplexed Ion Beam Imaging

##### Selena Ferrian, PhD^1^, Theodore Nowicki, MD, PhD^3^, David Dawson^3^, Alex Baranski^2^, John Glaspy, MD^3^, Antoni Ribas, MD, PhD^3^, Michael Angelo, MD, PhD^2^

###### ^*1*^*Stanford University, Palo Alto, CA, United States* ; ^*2*^*Stanford University, Palo Alto, CA, United States* ; ^*3*^*University of California Los Angeles, Los Angeles, CA, United States*

####### **Correspondence:** Michael Angelo (mangelo0@stanford.edu)

**Background**

Immune checkpoint blockade utilizing PD-1 inhibition is an effective approach for the treatment of a wide variety of cancer subtypes (1-3). However, these therapies can be associated with a diverse array of autoimmune side effects, which can affect multiple sites throughout the body (4-8). While lower gastrointestinal (GI) side effects are more common, upper gastrointestinal adverse events are rarely reported (7-9). IFN-γ is the signature cytokine generated by CD4+ Th1 and CD8+ T effector cells, but its exact role in regulating epithelial cell changes is unknown. Epithelial cells are also a source of cytokines that may regulate gastritis, including IFN-γ, IL-1b, IL-6, and IL-33 (10). This is the first comprehensive multiplexed histopathological study of a case nivolumab-associated gastroenteritis reported in the literature.

**Methods**

To elucidate the immunology underlying this condition, we leveraged the ability of Multiplexed Ion Beam Imaging by Time-Of-Flight (MIBI-TOF) to identify the presence and proportion of infiltrating immune cells from a single section of biopsy specimen. This approach circumvents limitations imposed by insufficient tissue for thorough histological and molecular profiling. Using MIBI-TOF we analyzed formalin-fixed, paraffin-embedded (FFPE) human gastric tissue with twenty-seven labels simultaneously.

**Results**

Our analysis revealed a gastritis characterized by severe mucosal injury, interferon gamma (IFN-γ)-producing gastric epithelial cells, and mixed inflammation that included CD8 and CD4 T cell infiltrates lacking or with reduced expression of granzyme B and FoxP3, respectively (Figure 1).

**Conclusions**

Taken together, the salient features in this case study of PD-1 associated gastritis include lamina propria expansion by mononuclear inflammation, epithelial injury from crypt apoptosis, intraepithelial lymphocytic and neutrophilic inflammation, and expression of IFN-γ by epithelial cells, in conjunction with clinical resolution of symptoms upon cessation of nivolumab therapy, are consistent with anti-PD-1-induced gastritis. Prolonged PD-1 blockade may induce gastric epithelial cells to overproduce IFN-γ, which we speculate concomitantly downregulates T-cell activity in gastric-mediated activation and immunopathology. Understanding the landscape in regard to immune cell infiltrates and their interactions with mucosal epithelia may shed light on the underlying mechanisms of anti-PD-1 induced gastritis, further informing future clinical applications of immunotherapeutic agents.

**Acknowledgements**

M.A. and S.C.B. are supported by the NIH 1-DP5-OD019822, 1R01AG056287-01, 1R01AG057915-01, and 1U24CA224309-01. T.S.N. by K12-HD000850, the Tower Cancer Research Foundation, and the Hyundai Hope on Wheels Award. This collaboration was enabled by the Parker Institute for Cancer Immunotherapy.

**References**

1. Buchbinder EI, Desai A. CTLA-4 and PD-1 pathways: similarities, differences, and implications of their inhibition. Am J Clin Oncol 2016;39:98–106.

2. Hofmann L, Forschner A, Loquai C, Goldinger SM, Zimmer L, Ugurel S, et al. Cutaneous, gastrointestinal, hepatic, endocrine, and renal side-effects of anti-PD-1 therapy. Eur J Cancer 2016;60:190–209.

3. Larkin J, Chiarion-Sileni V, Gonzalez R, Grob JJ, Cowey CL, Lao CD, et al. Combined nivolumab and ipilimumab or monotherapy in untreated melanoma. N Engl J Med 2015;373:23–34.

4. McDermott DF, Sosman JA, Sznol M, Massard C, Gordon MS, Hamid O, et al. Atezolizumab, an anti-programmed death-ligand 1 antibody, in metastatic renal cell carcinoma: long-term safety, clinical activity, and immune correlates from a phase IA study. J Clin Oncol 2016;34:833–42.

5. Balar AV, Galsky MD, Rosenberg JE, Powles T, Petrylak DP, Bellmunt J, et al. IMvigor210 Study Group. Atezolizumab as first-line treatment in cisplatin-ineligible patients with locally advanced and metastatic urothelial carcinoma: a single-arm, multicentre, phase 2 trial. Lancet 2017;389:67–76.

6. Collins M, Michot JM, Danlos FX, Mussini C, Soularue E, Mateus C, et al. Inflammatory gastrointestinal diseases associated with PD-1 blockade antibodies. Ann Oncol 2017;28:2860–65.

7. Gonzalez RS, Salaria SN, Bohannon CD, Huber AR, Feely MM, Shi C. PD-1 inhibitor gastroenterocolitis: case series and appraisal of 'immunomodulatory gastroentero-colitis'. Histopathology 2017;70(4):558–67.

8. Lu J, Firpi-Morell RJ, Dang LH, Lai J, Liu X. An unusual case of gastritis in one patient receiving PD-1 blocking therapy: coexisting immune-related gastritis and Cytomegaloviral infection. Gastroenterology Res 2018;11(5):383–7.

9. Boike J, Dejulio T. Severe esophagitis and gastritis from nivolumab therapy. ACG Case Rep J 2017;4:e57.

10. Bockerstett KA, DiPaolo RJ. Regulation of gastric carcinogenesis by inflammatory cytokines. Cell Mol Gastroenterol Hepatol 2017;4(1):47–53.

11. Lee I. Critical pathogenic steps to high risk Helicobacter pylori gastritis and gastric carcinogenesis. World J Gastroenterol 2014;20:6412–9.

**Ethics Approval**

This study was deemed exempt under Stanford IRB-39053.

**Fig. 1 (abstract 063).**
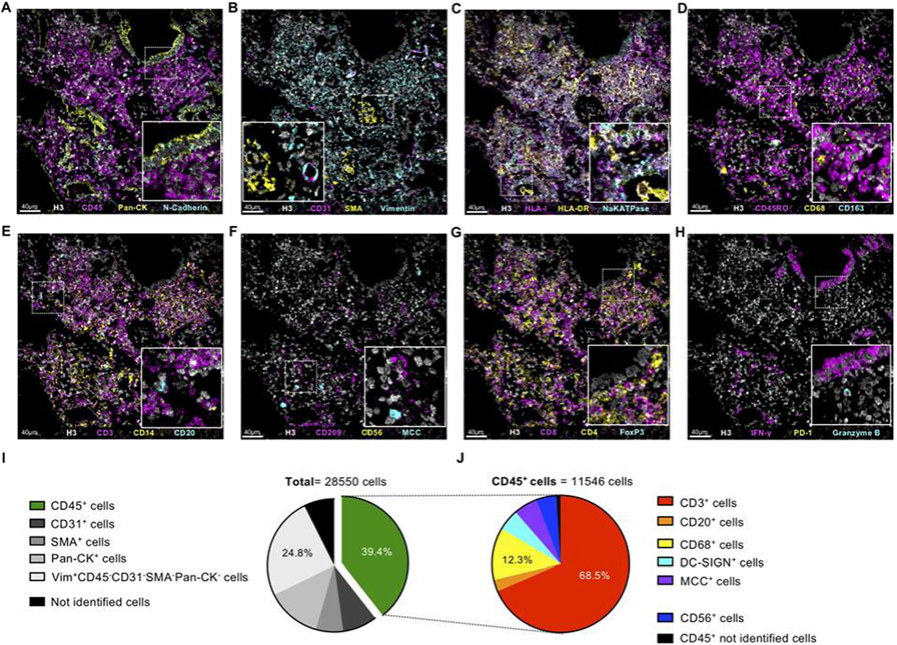
Immune and non-immune cells landscape

#### O64 Immune checkpoint inhibitor therapy in patients with preexisting inflammatory bowel disease

##### Hamzah Abu-Sbeih, MD^1^, David Faleck^2^, Biagio Ricciuti, MD^3^, Robin Mendelsohn^4^, Abdul Rafeh Naqash, MD^5^, Justine Cohen, DO^6^, MacLean Sellars^6^, Aanika Balaji^7^, Guy Ben-betzalel^8^, Ibraheim Hajir^9^, Jiajia Zhang, MD, MPH^7^, Mark Awad, MD PhD^3^, Giulia Leonardi^3^, Douglas Johnson, MD, MSCI^10^, David Pinato, MD, MRes, PhD^11^, Dwight Owen^12^, Sarah Weiss, MD^13^, Giuseppe Lamberti, MD^14^, Mark Lythgoe^11^, Lisa Manuzzi^14^, Christina Arnold^15^, Wei Qiao, PhD^1^, Jarushka Naidoo, MD^7^, Gal Markel^8^, Nick Powell^9^, Sai-Ching Yeung, MD, PhD^1^, Elad Sharon, MD, MPH^16^, Michael Dougan, MD, PhD^6^, Yinghong Wang, MD, PhD^1^

###### ^*1*^*Univ of Texas MD Anderson Cancer Center, Houston, TX, United States* ; ^*2*^*Memorial Sloan Kattering Cancer Center, New York, NY, United States* ; ^*3*^*Dana-Farber Cancer Institute, Boston, MA, United States* ; ^*4*^*Memorial Sloan Kettering Cancer Center, New York, NY, United States* ; ^*5*^*East Carolina Univ, Greenville, NC, United States* ; ^*6*^*Massachusetts General Hospital, Boston, MA* ; ^*7*^*Johns Hopkins University, Baltimore, MD, United States* ; ^*8*^*Sheba Medical Center, Ramat Gan, Israel* ; ^*9*^*King's College London, London, United Kingdom* ; ^*10*^*Vanderbilt University, Nashville, TN, United States* ; ^*11*^*Imperial College London, London, United Kingdom* ; ^*12*^*Ohio State University, Columbus, OH, United States* ; ^*13*^*Yale University, New Haven, CT* ; ^*14*^*Bologna University, Brookline, MA, United States* ; ^*15*^*The Ohio State University, Columbus, United States* ; ^*16*^*National Cancer Institute, Bethesda, MD, United States*

####### **Correspondence:** Yinghong Wang (YWang59@mdanderson.org)

**Background**

The risk of immune checkpoint inhibitor therapy-related gastrointestinal adverse events in cancer patients with inflammatory bowel disease (IBD) has not been well described. We characterized gastrointestinal adverse events in patients with underlying IBD who received immune checkpoint inhibitors.

**Methods**

We performed a multicenter, retrospective study of patients with documented IBD who received immune checkpoint inhibitor therapy between January 2010 and February 2019. Backward selection and multivariate logistic regression were conducted to assess risk of gastrointestinal adverse events.

**Results**

Of the 102 included patients, 17 received therapy targeting cytotoxic T-lymphocyte antigen-4 and 85 received monotherapy targeting programmed death-1 or its ligand. Half the patients had Crohn’s disease and half had ulcerative colitis (Table 1). The median time from last active IBD episode to immunotherapy initiation was 5 years (interquartile range, 3-12). Forty-three patients were not receiving treatment for IBD. Gastrointestinal adverse events occurred in 42 patients (41%) after a median of 62 days (interquartile range, 33-123), a rate higher than that among similar patients without underlying IBD who were treated at centers participating in the study (11%; P<0.001). Gastrointestinal events among patients with IBD included grade 3 or 4 diarrhea in 21 patients (21%). Four patients experienced colonic perforation, 2 of whom required surgery (Table 2). No gastrointestinal adverse event-related deaths were recorded. Anti-cytotoxic T-lymphocyte antign-4 therapy was associated with increased risk of gastrointestinal adverse events on univariate but not multivariate analysis (respectively, odds ratio, 3.19; 95%confidence interval, 1.8-9.48; P=0.037 and odds ratio, 4.72; 95%confidence interval, 0.95-23.53; P=0.058) (Table 3).

**Conclusions**

Preexisting IBD increases the risk of severe gastrointestinal adverse events in patients treated with immune checkpoint inhibitors.

**Ethics Approval**

This study was approved by MD Anderson Cancer Center Institutional Review Board with IRB # PA17-0329.

**Consent**

Written informed consent was waived given retrospective study.

**Table 1 (abstract 064).**
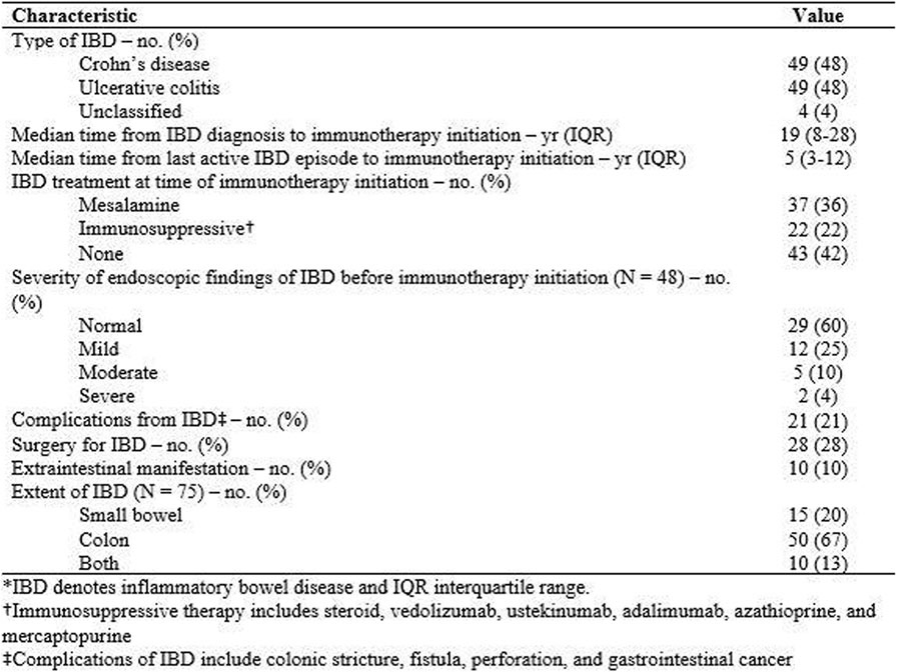
See text for description

**Table 2 (abstract 064).**
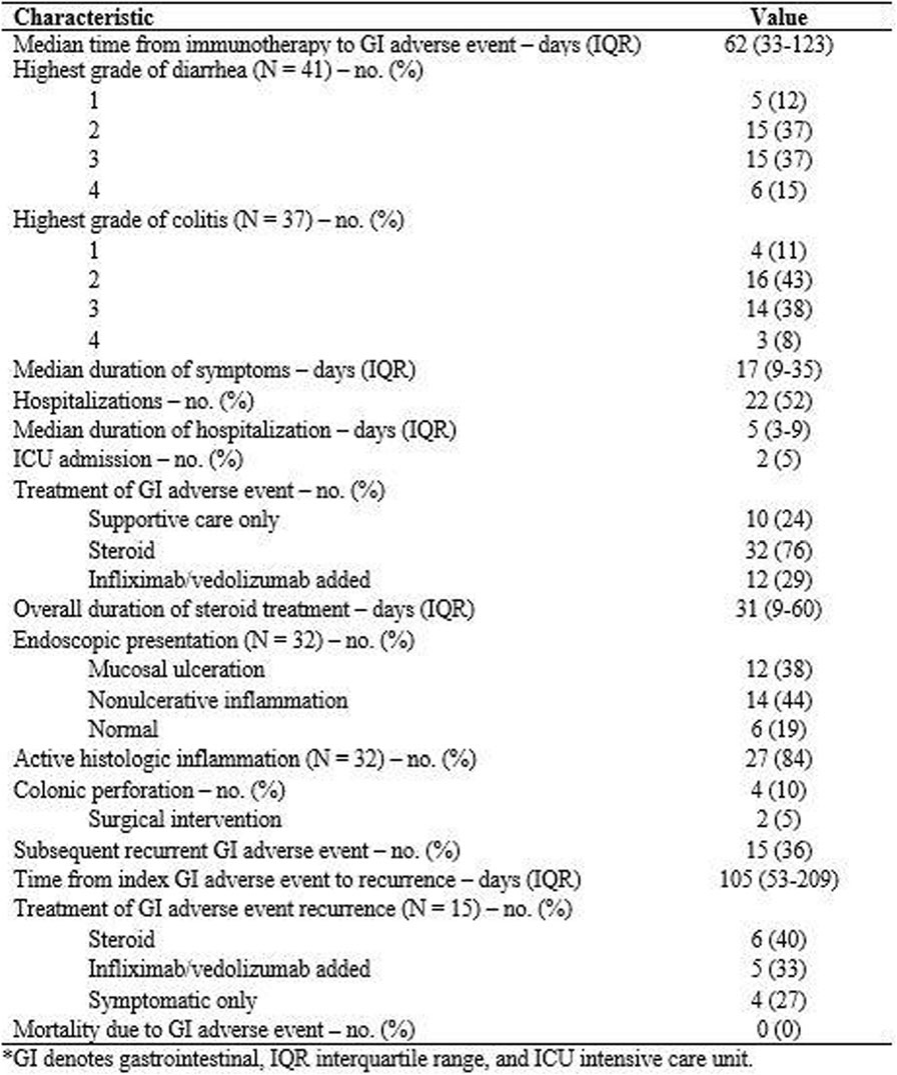
See text for description

**Table 3 (abstract 064).**
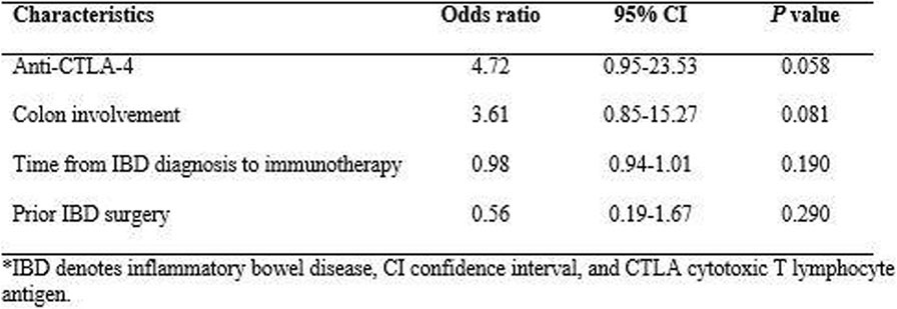
See text for description

### Machine Learning, Artificial Intelligence, and Computational Modeling

#### O65 Artificial intelligence-powered retrospective analysis of PD-L1 expression in nivolumab trials of advanced non-small cell lung cancer

##### Vipul Baxi, MS^1^ , Vipul Baxi, MS^1^, Andrew Beck, MD, PhD^2^, Dimple Pandya^1^, George Lee, PhD^1^, Cyrus Hedvat, MD, PhD^1^, Aditya Khosla^2^, Dayong Wang^2^, Hunter Elliott^2^, Harsha Pokkalla^2^, Abhik Lahiri^2^, Benjamin Glass^2^, Jennifer Kerner^2^, Ilan Wapinski^2^, Scott Chasalow^1^, Han Chang, PhD^1^, Robin Edwards, PhD^1^, Michael Montalto^1^

###### ^*1*^*Bristol-Myers Squibb, Princeton, NJ, United States* ; ^*2*^*PathAI, Inc, Boston, MA, United States*

####### **Correspondence:** Vipul Baxi (Vipul.Baxi@bms.com)

**Background**

Immune-cell PD-L1 expression is an emerging biomarker of response to immunotherapy, but manual pathology assessment is challenging. We developed and validated an artificial intelligence (AI)-powered image analysis algorithm to quantify PD-L1 expression on tumor cells and immune cells, including expression on macrophages and lymphocytes. Statistical analyses assessed concordance between manual and AI-evaluated PD-L1 expression on different cell types and their relationship to efficacy of nivolumab monotherapy in patients with advanced non-small cell lung cancer.

**Methods**

PD-L1 expression on tumor cells, macrophages, and lymphocytes was evaluated using the PD-L1 IHC 28-8 pharmDx assay (Dako, Agilent Technologies Co) and on digital whole-slide images using the PathAI platform (not intended for diagnostic use). Deep-learning models were trained with 250,000+ image annotations to detect and classify cells. Model performance was assessed on annotated frames consisting of 150x150-micron–sized sub-regions extracted from a validation image dataset against a consensus across 5 pathologists as reference. Locked algorithms were applied to PD-L1–stained images from clinical trials of nivolumab (CheckMate 017 [n = 167], CheckMate 026 [n = 502], and CheckMate 057 [n = 410]) [1-3] in an exploratory post-hoc analysis.

**Results**

In the frame-based validation of images selected across trials, AI-powered and manual quantification of PD-L1 was correlated (Pearson coefficients were 0.93, 0.79, 0.84, and 0.83 for PD-L1 on tumor cells, macrophages, lymphocytes, and total immune cells, respectively). Both approaches yielded similar associations between PD-L1 on tumor cells and progression-free survival, with log2(HR) and P values of –0.57 (P=0.033) for AI-based and –0.50 (P=0.025) for manual in CheckMate 017; –0.30 (P=0.070) and –0.27 (P=0.176) in CheckMate 026; –0.56 (P=0.001) and –0.62 (P=0.002) in CheckMate 057. Similar associations with response were observed for tumor cell scoring and AI-powered quantitation of PD-L1 on immune cells. Composite PD-L1 expression on tumor cells plus macrophages did not show a stronger association with response compared with tumor cells alone. C-index values for PD-L1 expression on tumor cells vs tumor cells plus macrophages were 0.610 and 0.586 in CheckMate 017; 0.587 and 0.591 in CheckMate 057; and 0.522 and 0.527 in CheckMate 026.

**Conclusions**

AI-powered analysis provides a robust, scalable approach to quantify PD-L1 expression and is concordant with manual assessment. The model warrants further exploration of PD-L1 expression patterns and their association with response across tumor types.

**Acknowledgements**

Bristol-Myers Squibb and PathAI, Inc. Professional medical writing and editorial assistance were provided by Katerina Pipili, PhD, and Jay Rathi, MA, of Spark Medica Inc, funded by Bristol-Myers Squibb.

**Trial Registration**

NCT01642004, NCT02041533, NCT01673867

**References**

1. Brahmer J, Reckamp K, Baas P, et al. Nivolumab versus docetaxel in advanced squamous-cell non–small-cell lung cancer. N Engl J Med. 2015;373:123-135.

2. Carbone DP, Reck M, Paz-Ares L, et al. First-line nivolumab in stage IV or recurrent non–small-cell lung cancer. N Engl J Med. 2017;376:2415-2426.

3. Borghaei H, Paz-Ares L, Horn L, et al. Nivolumab versus docetaxel in advanced nonsquamous non–small-cell lung cancer. N Engl J Med. 2015;373:1627-1639.

**Ethics Approval**

The protocol was approved by site institutional review boards or independent ethics committees and conducted according to Good Clinical Practice guidelines, per the International Conference on Harmonisation. Patients provided written informed consent based on Declaration of Helsinki principles

### Microbiome and Other Environmental Factors

#### O66 Patient-derived microbiota germ-free mouse model for identifying mechanisms of checkpoint blockade efficacy modulation

##### Vyara Matson, PhD, Jessica Fessler, BS, Yuanyuan Zha, PhD, Thomas Gajewski, MD, PhD

###### University of Chicago, Chicago, IL, United States

####### **Correspondence:** Thomas Gajewski (tgajewsk@medicine.bsd.uchicago.edu)

**Background**

Checkpoint blockade immunotherapy is having a major impact on cancer outcomes. However, a sizable subset of patients still fails to benefit. Along with tumor- and host-intrinsic factors, the gut microbiota has emerged as an important variable leading to inter-patient heterogeneity in responsiveness to PD-1 blockade (1-3). Here, we seek deeper understanding of the underlying mechanisms.

**Methods**

Stool samples were collected from 42 metastatic melanoma patients prior to immunotherapy and microbial composition was found to be associated with responsiveness to anti-PD-1 (1). Reconstitution of germ-free (GF) mice with patient microbiota provides a model for understanding mechanistic details and also for testing therapeutic interventions. GF mice were colonized by oral gavage with stool from 11 responding and 6 non-responding patients and efficacy of anti-PD-L1 against B16.SIY melanoma was evaluated. For a more physiologic system, we generated stable colonies of mice naturally colonized from birth with responder or non-responder patient microbiota through breeding. Interventions were explored to convert non-responder mice into responders, including oral antibiotics (vancomycin, ampicillin, metronidazole, and neomycin) and/or fecal microbiota transfer (FMT) from responders. Fecal 16S rDNA was sequenced to identify microbiome alterations associated with conversion from non-responder to responder phenotype.

**Results**

Transfer of fecal material from patients to GF mice recapitulated the efficacy of PD-1/PD-L1 blockade in over 72% of cases, pointing to mechanistic involvement of gut commensals. The responder microbiota conferred increased BATF3-lineage dendritic cells in the mesenteric lymph nodes and peripheral lymphoid tissues, increased activation of anti-tumor T cells, increased infiltration of tumor antigen-specific CD8+ T cells in the tumor, and higher circulating levels of IFN-γ, IL-12p70, and CXCL5. Antibiotic pre-treatment of non-responder mice partially improved anti-PD-L1 efficacy, suggesting depletion of immune-inhibiting bacteria. Responder FMT also partially improved tumor control, and a combination of antibiotics + FMT completely rescued the anti-PD-L1 efficacy. In contrast, antibiotic pre-treatment + administration of non-responder FMT interfered with immunotherapy efficacy. FMT-induced alterations in microbiome composition are informing ongoing efforts to isolate and culture specific immunomodulatory strains.

**Conclusions**

Gut microbiota modulation shows promise as a potential strategy for improving outcomes with checkpoint blockade therapy. Our results support the existence of both immune-potentiating and immune-inhibiting gut bacteria, and suggest that the effect of oral antibiotics depends on the initial composition of the gut microbiota.

**References**

1. Matson V, Fessler J, Bao R, et al.: The commensal microbiome is associated with anti–PD-1 efficacy in metastatic melanoma patients. Science 2018, 359: 104–108

2. Gopalakrishnan V, Spencer CN, Nezi L, et al.: Gut microbiome modulates response to anti–PD-1 immunotherapy in melanoma patients. Science 2018, 359: 97–103

3. Routy B, Le Chatelier E, Derosa L, et al.: Gut microbiome influences efficacy of PD-1-based immunotherapy against epithelial tumors. Science 2018, 359: 91-97

**Ethics Approval**

This study was approved by The University of Chicago Research Administration, approval number FP069810.

#### O67 Variation in the commensal urinary microbiome is associated with response to Bacillus Calmette-Guérin (BCG) immunotherapy in early stage urothelial bladder cancer

##### Randy Sweis, MD^1^, Randy Sweis, MD^1^ , Ciro Andolfi^1^, Jeffrey Bloodworth^1^, Shay Golan, MD^2^, Nimrod Barashi^3^, Elle Hill^1^, Ryan Werntz^1^, Thomas Gajewski, MD, PhD^1^, Gary Steinberg, MD^4^

###### ^*1*^*University of Chicago, Chicago, IL, United States* ; ^*2*^*Rabin Medical Center, Chicago, IL, United States* ; ^*3*^*University of Illinois, Chicago, IL, United States* ; ^*4*^*New York University, Chicago, IL, United States*

####### **Correspondence:** Randy Sweis (rsweis@uchicago.edu)

**Background**

Intravesical bacillus Calmette-Guérin (BCG) immunotherapy is the standard of care for non-muscle invasive bladder cancer (NMIBC) with high-risk features. Its mechanism of action is pleotropic and involves innate immune activation, enhancement of antigen presentation, and stimulation of cytokine secretion resulting in an adaptive immune response. Despite the efficacy of BCG, up to 50% of patients have recurrent or progressive disease within 5 years. Recent data have indicated that the commensal gut microbiome impacts immunotherapy responsiveness for some advanced cancers. We hypothesized that a commensal urinary microbiome could influence responsiveness to BCG immunotherapy in urothelial bladder cancer.

**Methods**

Patients presenting with a new bladder tumor were enrolled prior to transurethral resection and BCG immunotherapy. Urine samples for microbiome assessment were collected by sterile catheterization to minimize urethral or skin contamination. The microbial composition for each sample was determined using the 16S rRNA gene sequencing-based method and shotgun sequencing. Data were organized by Operational Taxonomic Units (OTUs) and analyzed according to clinical outcome. Urine cytokine analysis (Invitrogen ProcartaPlex immunoassay) was also performed on available samples.

**Results**

We enrolled 31 patients with a median age of 69 years (46-87). The median follow-up was 12 months, and 10 (32%) patients experienced disease recurrence. In the overall population, Actinobacteria, Bacteroidetes, Firmicutes, Proteobacteria, and Tenericutes accounted for > 99% of the detected phyla. Distance matrix calculation indicated a significant difference between patients with and without recurrence (Bonferroni-corrected P = 0.017). The abundance of Proteobacteria was higher in patients with recurrence (P = 0.035), with stronger differences observed for specific taxa, such as the Gammaproteobacteria class (P = 0.0025) and the Enterobacteriacae family (P = 0.001). Firmicutes, in particular Lactobacillales, were more abundant in patients without recurrence (P = 0.049). Cytokine concentrations were measured in urine from a subset of 13 patients, including 6 patients with recurrence. Changes in urinary IFN-γ and TNF-α levels after 6 weekly doses of BCG were calculated, and no differences were observed between patients with and without recurrence (both P > 0.10)(Figure 1).

**Conclusions**

In this study, we uncovered a correlation between the composition of the urine microbiome and clinical responsiveness to BCG immunotherapy in patients with bladder cancer. Further studies to confirm these results are ongoing.

**Ethics Approval**

This study was approved by the University of Chicago institutional review board (IRB approval #161317).

**Consent**

Written informed consent was obtained from all patients on this study.

**Fig. 1 (abstract 067).**
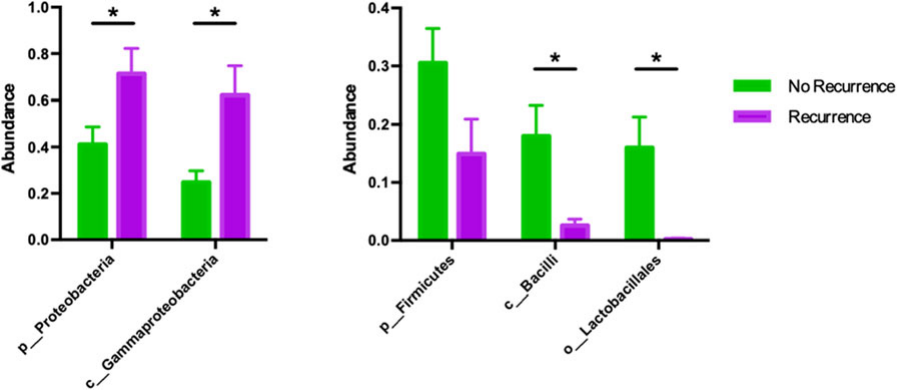
Variation in the urinary microbiome of BCG-treated patients

#### O68 Characteristics of the microbiome form and function of complete responders to Anti-PD1 and healthy individuals implicate donor selection for clinical trial design

##### Md Abdul Wadud Khan, PhD^1^, Jennifer Wargo, MD, MMSc^2^ , Beth Helmink, MD, PhD^2^, Elizabeth Burton^2^, Christine Spencer, PhD^3^, Jennifer McQuade, MD^2^, Miles Andrews, MD, PhD^2^, Alexandria Cogdill^2^, Lauren Haydu, MS, BChe, MIPH^2^, Reetakshi Arora^2^, Angela Harris^2^, Vanessa Jensen, DVM^2^, Adrienne Duran, BAS^2^, Linsey Martin^2^, Elizabeth Sirmans^2^, Sapna Patel, MD^2^, Tim Heffernan^2^, Robert Jenq, MD^2^, Michael Wong, MD PhD FRCPC^2^, Jeffrey Gershenwald, MD^2^, Tawbi Hussein^2^, Isabella Glitza, MD, PhD^2^, Patrick Hwu, MD^2^, Michael Davies, MD, PhD^2^, David Cook^4^, Jennifer Wortman^4^, Theresa LaVallee, PhD^5^, Adi Diab, MD^2^, Vancheswaran Gopalakrishnan^6^

###### ^*1*^*The University of Texas MD Anderson Cancer Center, Houston, TX, United States* ; ^*2*^*University of Texas MD Anderson Cancer C, Houston, TX, United States* ; ^*3*^*Parker Institute for Cancer Immunotherap, New York, NY, United States* ; ^*4*^*Seres Therapeutics, Inc., Cambridge, MA, United States* ; ^*5*^*Parker Institute for Cancer Immunotherapy San Francisco, CA, United States* ; ^*6*^*AstraZeneca, Gaithersburg, MD, United States*

####### **Correspondence:** Jennifer Wargo (JWargo@mdanderson.org)

**Background**

The gut microbiome is being increasingly recognized as an important modulator of immune checkpoint blockade efficacy [1-4]. We have recently identified a ‘Type-I’ signature characterized by higher alpha diversity and enrichment of Ruminococacceae that is associated with complete response to anti-PD1 therapy [1]. Several clinical trials of microbiome modulation to enhance response to immune checkpoint blockade (ICB), many using fecal microbiota transplant (FMT) from healthy donors (HD). Though FMT from HD is effective in treating dysbiotic gastrointestinal diseases such as C. difficile, the optimal donors for FMT to enhance responses to ICB in cancer patients remain unknown.

**Methods**

We characterized the gut microbiome in metastatic melanoma patients undergoing anti-PD1 using 16S rRNA sequencing (n=114) and whole genome shotgun sequencing (n=76). In addition, we compared the taxonomic profiles of microbiomes between complete responders (CR; n=35) and healthy patients (n=194) from American Gut [5]. FMT of CR donors with Type-1 signature versus a known NR and a healthy donor (HD) was then performed into germ-free mice. Mice were implanted with melanoma tumors and then treated with anti-PD-L1. Tumor growth was assessed, and longitudinal microbiome analyses and immune profiling of tumor in mice are performed.

**Results**

Consistent with our prior findings, we found a significantly higher alpha-diversity in responders (n=72) (P=0.03) and contrasting compositional structures at baseline in comparison to non-responders (n=42). Though there was a similar trend in alpha diversity in both CR and healthy cohorts, distinct clustering patterns in principal coordinate analysis (P=0.04) is suggestive of substantial variation in overall microbiome composition between these two groups. Importantly, not all CRs demonstrated a Type-1-like signature (60%, 21 of 35 CRs) nor did healthy controls (43%, 83 of 194). Functional metagenomics revealed differential metabolic capabilities in CR compared to other response categories. Mouse studies demonstrated reduced tumor growth in both CR-FMT and HD-FMT mice vs. NR-FMT mice and CR-FMT outperformed HD-FMT. Immune profiling of human and mouse specimens are being conducted and will be integrated with GM analysis.

**Conclusions**

Together, these studies reveal differences in comparing healthy vs CR microbiome and functional profiles and provide important information about potential donor selection in FMT trials in immunotherapy, warranting additional studies and translational research.

**References**

1. Gopalakrishnan, V., et al. Gut microbiome modulates response to anti-PD-1 immunotherapy in melanoma patients. Science (2017).2. Matson, V., et al. The commensal microbiome is associated with anti-PD-1 efficacy in metastatic melanoma patients. Science 359, 104-108 (2018).3. Frankel, A.E., et al. Metagenomic Shotgun Sequencing and Unbiased Metabolomic Profiling Identify Specific Human Gut Microbiota and Metabolites Associated with Immune Checkpoint Therapy Efficacy in Melanoma Patients(). Neoplasia (New York, N.Y.) 19, 848-855 (2017).4. Routy, B., et al. Gut microbiome influences efficacy of PD-1-based immunotherapy against epithelial tumors. Science (2017).5. McDonald, D., et al. American Gut: an Open Platform for Citizen Science Microbiome Research. mSystems 3(2018).

**Ethics Approval**

The study was approved by the University of Texas MD Anderson Cancer Center and the Protocol Number: PA15-0232.

#### O69 Helicobacter hepaticus remodels the tumor immune microenvironment and reduces colorectal tumor burden

##### Abigail Overacre-Delgoffe, PhD, Hannah Bumgarner, Ansen Burr, Timothy Hand, PhD

###### University of Pittsburgh, Pittsburgh, PA, United States

####### **Correspondence:** Timothy Hand (timothy.hand@chp.edu)

**Background**

Immunotherapy has revolutionized patient treatment in numerous cancers. However, the vast majority of advanced colorectal cancer (CRC) patients are non-responsive and the 5-year survival rate is poor. While select microbes have been associated with progression and immunotherapy responsiveness in other cancers such as melanoma, fundamental mechanistic questions remain, including how the microbiota shapes anti-tumor immunity and therapeutic response in CRC. Our hypothesis is that the intestinal microbiota that live on mucosal surfaces or within colorectal tumors shift immune balance, in part through increased antigen presentation and CD4+ T cell help, resulting in a stronger anti-tumor immune response and tumor reduction.

**Methods**

We utilized the AOM-DSS mouse model to establish colitis-associated CRC. Once tumors were developed (7 weeks), we colonized half of the mice with Helicobacter hepaticus (Hhep) and assessed tumor number 5 weeks later. We isolated lymphocytes from the lamina propria, colonic epithelium, mesenteric lymph node, and tumor for flow cytometry analyses and performed 16S FISH and confocal microscopy of tissue sections to visualize Hhep or immune infiltrate. Lastly, we used 16S sequencing to determine how Hhep colonization affected other populations within the host microbiome.

**Results**

We found that modulating the microbiome of AOM-DSS tumor-bearing mice through the addition of a single bacteria, Hhep, reduces tumor burden, in some cases leading to complete tumor clearance, and significantly enhances survival. Hhep colonization reshapes the tumor microenvironment (TME) through increased infiltration of migratory dendritic cells and Tconv within the tumor core and decreased Tregs. In addition, colonization leads to a substantial increase in organized tertiary lymphoid structures surrounding the tumor and a decrease in T cell exhaustion. FISH revealed that Hhep colonizes the mucosa around the tumor but was also found within the tumor itself, which we believe drives Hhep-specific CD4+ T cell infiltration, providing vital signals to cross-presenting DCs and tumor-specific T cells.

**Conclusions**

We have observed a complete remodeling of the TME after the addition of a single bacteria, Hhep, leading to enhanced anti-tumor immunity and increased survival in CRC. While overall rates of CRC prevalence in the US have declined, incidence among young adults has been steadily rising. Dysbiosis in CRC patients has been previously reported, and whether this has a direct effect on the anti-tumor immune response remains unknown. These studies have the potential to improve our understanding of how microbiota-specific T cells shape tumor progression as well as potentially uncover novel mechanisms of resistance to immunotherapy in CRC.

**Ethics Approval**

IACUC #16099226

### Novel Single-Agent Immunotherapies

#### O70 CD122-selective IL-2 complexes treat ovarian carcinomas, possibly by dysregulating Treg differentiation and reducing suppression, and promoting T cell stem cells

##### Yilun Deng, MD, PhD^1^, Justin Drerup^1^, Xinyue Zhang^2^, Ryan Reyes^1^, Jenny Mendez^1^, Myrna Garcia^1^, Alvaro Padron^1^, Harshita Gupta, PhD^1^, Tyler Curiel, MD^1^

###### ^*1*^*UT Health San Antonio, San Antonio, TX, United States* ; ^*2*^*Sun Yat-Sen University, Guangzhou, China*

####### **Correspondence:** Tyler Curiel (curielt@uthscsa.edu)

**Background**

Ovarian cancer (OC) responds poorly to immunotherapies. Regulatory T cells (Treg) engage IL-2 by high affinity CD25 for differentiation and function [1], and anti-tumor effector T cells (Teff) use intermediate affinity CD122. We studied IL-2 complexes (IL-2c) that selectively activate CD122 (Teff) over CD25 (Tregs). A CD8+CXCR5+TCF-1+ T cell stem cell (TCSC) population reportedly improves immune checkpoint blockade efficacy [2, 3], but IL-2c effects on TCSC are unreported.

**Methods**

Orthotopic ID8agg-luc mouse OC burden was measured by in vivo imaging. Tumor, ascites and draining lymph nodes (TDLN) were analyzed by flow cytometry and tSNE. IL-2c was complexed using 1.5 μg/mouse IL-2 and 7.5 μg/mouse αIL-2 (clone JES6-5H4) before i.p. injection every other day starting at Day 7 for 4 doses. antiPD-L1 was given at 100ug/mouse every 3 days starting from Day 11 for 4 doses. FoxP3-IRES-RFP (FIR) mice [4] were used to sort live Tregs.

**Results**

IL-2c but not antiPD-L1 potently inhibits ID8agg (Figure 1). IL-2c surprisingly increased ascites Treg numbers but decreased Treg functional markers (CD25, CTLA-4, granzymeB) and suppressive function (Figure 2). TDLN Tregs were unaffected. tSNE showed great similarity of TDLN Tregs treated with isotype and IL-2c while ascites Tregs after IL-2c showed skewed differentiation (e.g., increased T-bet, IFNgamma) (Figure 3) which decreases suppression [5, 6]. In B16 melanoma, antiPD-L1 induced CD8+TCF-1+CXCR5+PD-1+ TCSC as reported, while adding IL-2c augmented expansion further (Figure 4). In ID8agg, IL-2c alone induced CD8+TCF-1hi TCSC (Figure 5). However, tSNE revealed that these TCSC differed from those in B16, including distinct IL-2c versus antiPD-L1 induced TCSC. In ID8agg, antiPD-L1-induced TCSC are mostly CXCR5+ and PD1+ while IL-2c-induced TCSC are PD1- (Figure 6), distinct from B16, and express CCR2 and CXCR3.

**Conclusions**

We define two novel IL-2c effects: inducing novel TCSC and blunting Treg suppression through differentiation skewing. Current work tests if effects are related and help efficacy, and mechanisms for IL-2c Treg effects. We also show that elicited TCSC differ by treatment and tumor, requiring additional investigations.

**Acknowledgements**

This work is supported by CPRIT Research Training Award (RP 170345) to YD, RO1 CA205965 and Clayton Foundation for Biomedical Research Grant to TC

**References**

1. Malek TR: The biology of interleukin-2. Annu Rev Immunol 2008, 26:453-479.

2. Brummelman J, Mazza EMC, Alvisi G, Colombo FS, Grilli A, Mikulak J, Mavilio D, Alloisio M, Ferrari F, Lopci E et al: High-dimensional single cell analysis identifies stem-like cytotoxic CD8(+) T cells infiltrating human tumors. J Exp Med 2018.

3. Sade-Feldman M, Yizhak K, Bjorgaard SL, Ray JP, de Boer CG, Jenkins RW, Lieb DJ, Chen JH, Frederick DT, Barzily-Rokni M et al: Defining T Cell States Associated with Response to Checkpoint Immunotherapy in Melanoma. Cell 2018, 175(4):998-1013 e1020.

4. Fantini MC, Dominitzki S, Rizzo A, Neurath MF, Becker C: In vitro generation of CD4+ CD25+ regulatory cells from murine naive T cells. Nat Protoc 2007, 2(7):1789-1794.

5. Levine AG, Mendoza A, Hemmers S, Moltedo B, Niec RE, Schizas M, Hoyos BE, Putintseva EV, Chaudhry A, Dikiy S et al: Stability and function of regulatory T cells expressing the transcription factor T-bet. Nature 2017, 546(7658):421-425.

6. Downs-Canner S, Berkey S, Delgoffe GM, Edwards RP, Curiel T, Odunsi K, Bartlett DL, Obermajer N: Suppressive IL-17A(+)Foxp3(+) and ex-Th17 IL-17A(neg)Foxp3(+) Treg cells are a source of tumour-associated Treg cells. Nat Commun 2017, 8:14649.

**Ethics Approval**

All animal work was done under UTHSA Institutional Animal Care and Use Committee approved studies, approved protocol numbers: 20120071AR, 20170035AR, 20140039AR, in compliance with the Guide for the Care and Use of Laboratory Animal Resources (published by National Research Council of the National Academies), Animal Welfare Act (AWA) (published by USDA), Public Health Service Policy on Humane Care and Use of Laboratory Animals (published by NIH) and US Government Principles for Utilization and Care of Vertebrate Animals Used in Testing, Research, and Training.

**Fig. 1 (abstract 070).**
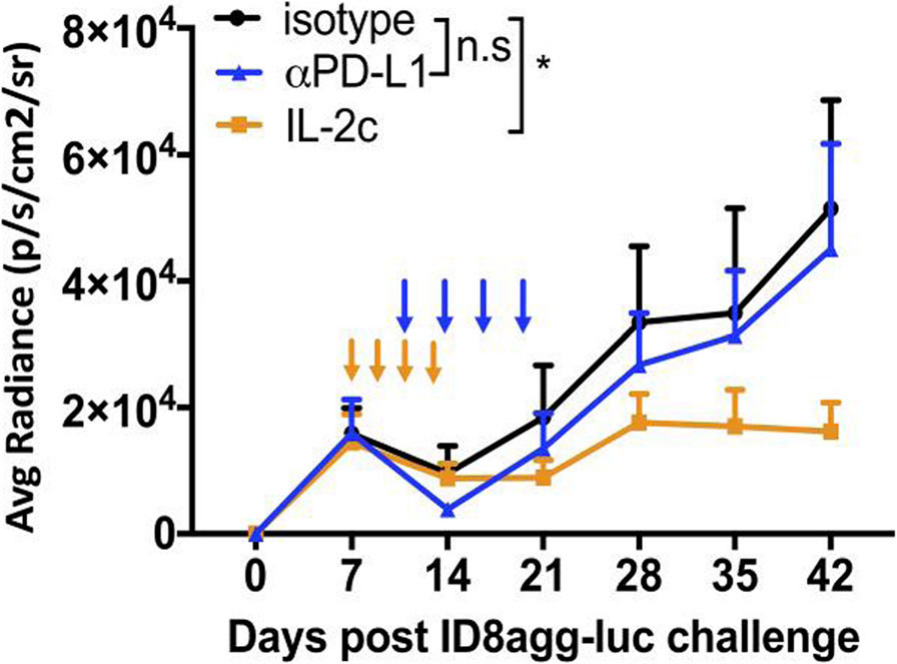
See text for description

**Fig. 2 (abstract 070).**
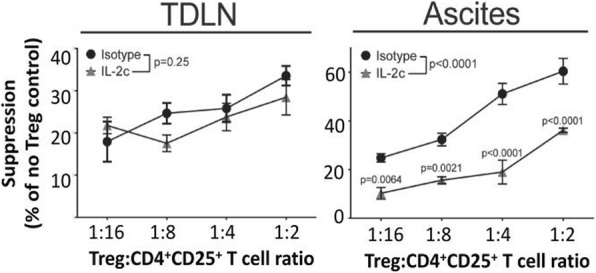
See text for description

**Fig. 3 (abstract 070).**
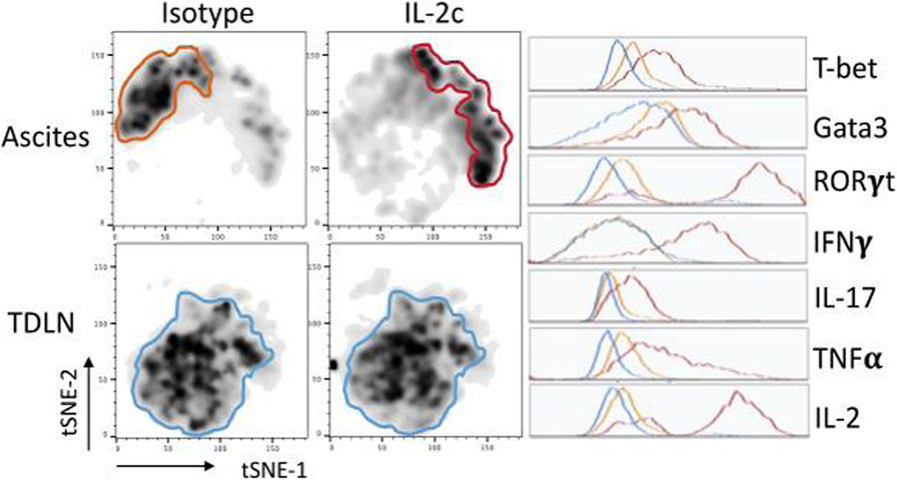
See text for description

**Fig. 4 (abstract 070).**
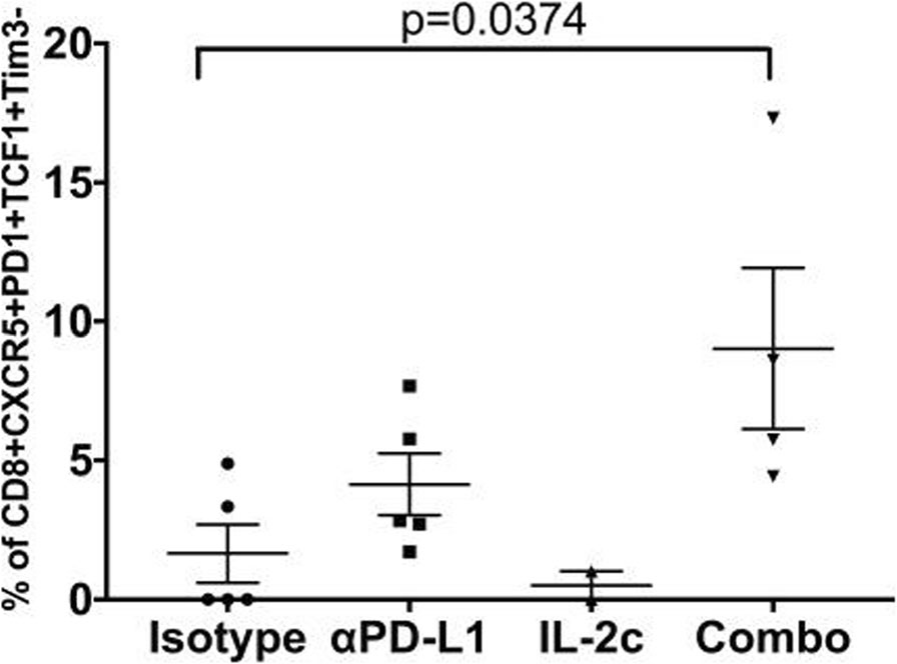
See text for description

**Fig. 5 (abstract 070).**
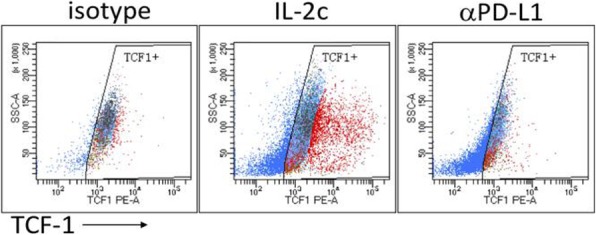
See text for description

**Fig. 6 (abstract 070).**
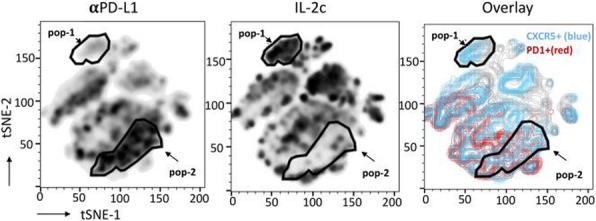
See text for description

#### O71 AMV564, a novel bivalent, bispecific T-cell engager, targets myeloid-derived suppressor cells

##### Sterling Eckard, PhD^1^, Leah Gehrs^2^, Victoria Smith^1^ , Jeanmarie Guenot^1^, John DiPersio^2^, Sheng Wei, MD^3^, Michael Rettig^2^

###### ^*1*^*Amphivena Therapeutics, Inc., South San Francisco, CA, United States* ; ^*2*^*Washington University School of Medicine, St. Louis, MO, United States* ; ^*3*^*Moffitt Cancer Center, Tampa, FL, United States*

####### **Correspondence:** Victoria Smith (vsmith@amphivena.com)

**Background**

Overcoming the suppressive tumor microenvironment is a major challenge in immune therapy, and the critical effectors of the suppressive tumor microenvironment are the myeloid-derived suppressor cells (MDSCs). MDSCs elicit a range of suppressive functions that inhibit normal T cell responses and cause unresponsiveness to immune checkpoint blockade. A dominant function of MDSCs is the suppression of T cell activity in a variety of manners that are pathology and context dependent. AMV564, a bivalent, bispecific antibody that engages both CD3 and CD33 for selective depletion of MDSCs and leukemic blasts, is currently in clinical development in acute myeloid leukemia (AML) and myelodysplastic syndromes (MDS). Here we report ex vivo and translational clinical data that support our proposal to evaluate AMV564 as a selective agent to deplete MDSCs in solid tumors.

**Methods**

Flow cytometry was used to phenotype AML and MDS patient blasts, T cells, and MDSCs in freshly isolated whole blood or bone marrow aspirates. Additionally, reactive oxygen species (ROS) were detected by flow cytometry using the cell permeant dye 2’,7’-dichlorofluorescin diacetate. The numbers of blasts, lymphocytes, neutrophils, and monocytes were evaluated using patient hematology panels.

**Results**

Treatment *ex vivo* of patient-derived bone marrow mononuclear cells results in selective depletion of MDSCs (p < 0.01) and a decrease in the production of ROS (Figure 1). AMV564 induces a significant increase of activated T cells only in the presence of CD33+ target cells, with >2-fold increase in the proliferation of CD4+ and CD8+ T cells. The increase in proliferation is dose-dependent and accompanied by a significant increase in IFNγ production. In the AML clinical trial, rapid depletion of both monocytic and granulocytic MDSCs is apparent with no impact on circulating neutrophil or monocyte populations (Figure 2), and with concomitant T cell redistribution consistent with T cell activation.

**Conclusions**

Treatment with AMV564 selectively depletes MDSCs in a dose-dependent fashion both *ex vivo* and in patients. The ability of AMV564 to deplete MDSCs while activating T cells should lower the threshold necessary for a patient to respond to immunotherapy, and these results indicate potential for benefit in solid tumor indications where elevated MDSCs are associated with poor outcomes including inadequate response to immunotherapy.

**Ethics Approval**

This study was approved by the Institutional Review Board (IRB) or Independent Ethics Committee (IEC) at each participating institution.

**Fig. 1 (abstract 071).**
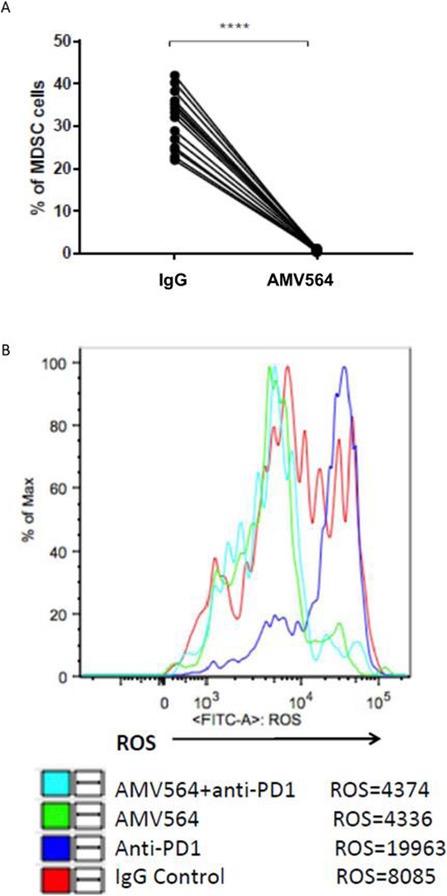
See text for description

**Fig. 2 (abstract 071).**
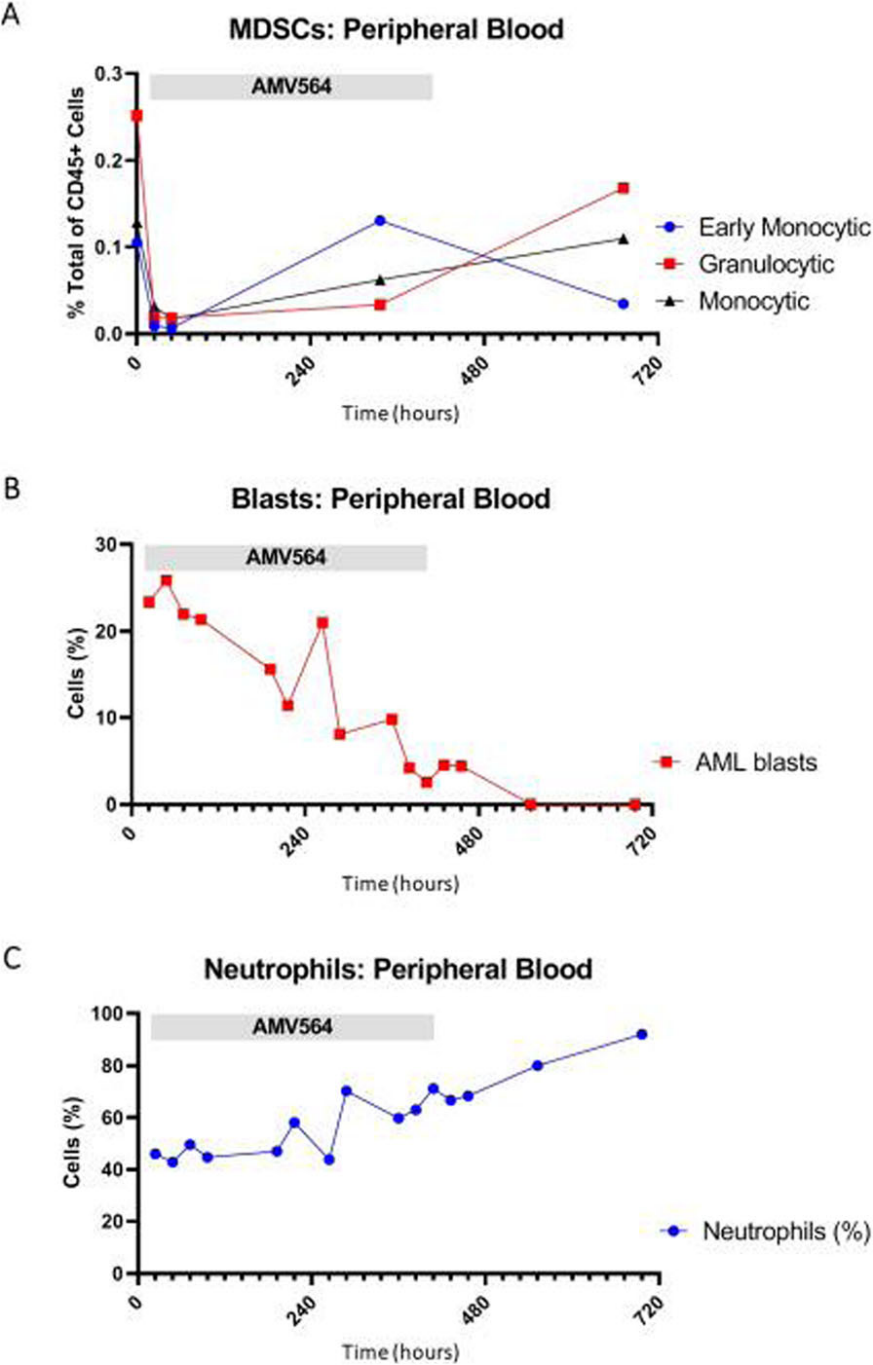
See text for description

#### O72 Genetic programming of macrophages to perform anti-tumor functions using targeted mRNA nanocarriers

##### Fan Zhang, PhD, Neha Parayath, PhD, Michael Coon, MS, Sirkka Stephan, PhD, Matthias Stephan, MD, PhD

###### Fred Hutchinson Cancer Research Center, Seattle, WA, United States

####### **Correspondence:** Matthias Stephan (mstephan@fredhutch.org)

**Background**

Tumor-associated macrophages (TAMs) usually perform immunosuppressive and tumor-promoting functions, expressing what is known as an ‘M2’ phenotype. Reprogramming these TAMs toward an ‘M1’ phenotype could thwart their pro-cancer activities and unleash anti-tumor immunity, but efforts to date have proven nonspecific, eliciting systemic inflammation. Here, we describe an in vitro-transcribed mRNA encoding an M1-polarizing transcription factor, interferon regulatory factor 5 (IRF5). This mRNA can be delivered through targeted nanoparticles (NPs) to reprogram TAMs without causing systemic toxicity (Figure 1).

**Methods**

We first validated that IRF5-NPs can introduce mRNA expression and modify macrophage phenotype in vitro, using RT-PCR and targeted gene sequencing. In our in vivo studies, we evaluated the therapeutic efficacy of IRF5-NPs in three animal models. We then determined the mechanisms associated with the therapeutic efficacy by analyzing immune cells populations (flowcytometry), cytokines release (FACS, Luminex), T-cells infiltration (immunofluorescence), and IRF5 mRNA biodistribution (RT-PCR).

**Results**

Our study on bone marrow-derived macrophages showed IRF5-NPs can induce immediate, transient, robust mRNA expression, and imprint a pro-inflammatory M1-like macrophage. Specifically, IRF5-NPs induced a macrophage gene profile that was similar to the signature M1 phenotype (Figure 2); signature M2 phenotype genes were strongly downregulated (e.g., Serpinb2 and Ccl11, 87-fold and 115-fold, respectively) while key M1-differentiation genes were upregulated (e.g., Ccl5, 190-fold). Infusions of IRF5-NPs reprogrammed the immuno-suppressive TAMs to induce anti-tumor immunity in models of ovarian cancer, melanoma, and glioblastoma. For example, in an ID8 syngeneic mouse model of ovarian cancer, nine doses of intraperitoneal IRF5-NPs regressed tumor growth and significantly extended mouse median survival for more than two folds (Figure 3). IRF5-NPs reduced the percentage of immune-suppressive macrophages for 17-fold. Conversely, the fraction of immune-stimulating M1-like macrophages increased for 20-fold (Figure 4). TAMs in IRF5-NPs treated mice also released higher pro-inflammatory cytokines, including IL-12 (3.4-fold), IFN-g (8.4-fold) and TNF-α (1.5-fold). The IRF5-NPs treatment also elicited significant CD4+ and CD8+ T-cells infiltrates in the mesentery tumor metastatic nodules (Figure 5). A biodistribution and histology-based study further established that these nanoreagents were safe for repeated dosing.

**Conclusions**

Thus, we have developed a novel strategy to genetically reprogramming TAMs, using NPs that carry mRNAs encode for transcriptional factor, IRF5. This new immunotherapy can safely obviate suppressive tumors without systemically disrupting immune homeostasis. Our next step is to clinically translate this technology as a new approach to treating ovarian cancer patients who were not responsive to other therapies (Figure 1).

**Acknowledgements**

The presenting author Fan Zhang was supported by a Research Scholar Grant, BRF1800015 from American Brain Tumor Association

**Fig. 1 (abstract 072).**
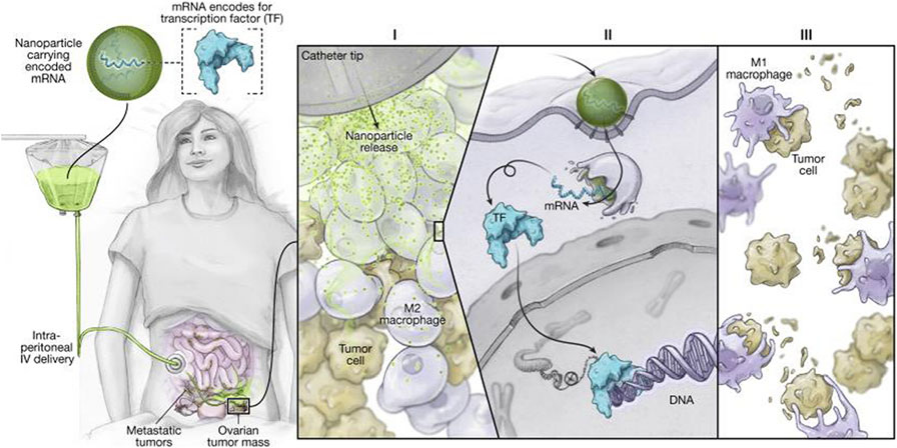
See text for description

**Fig. 2 (abstract 072).**
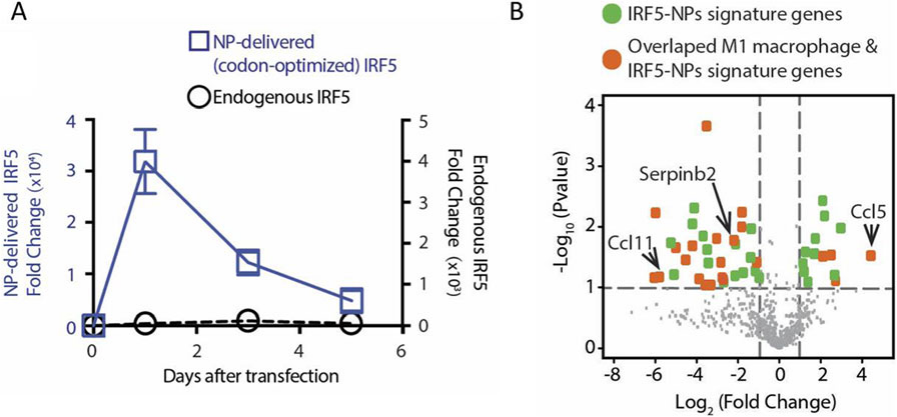
See text for description

**Fig. 3 (abstract 072).**
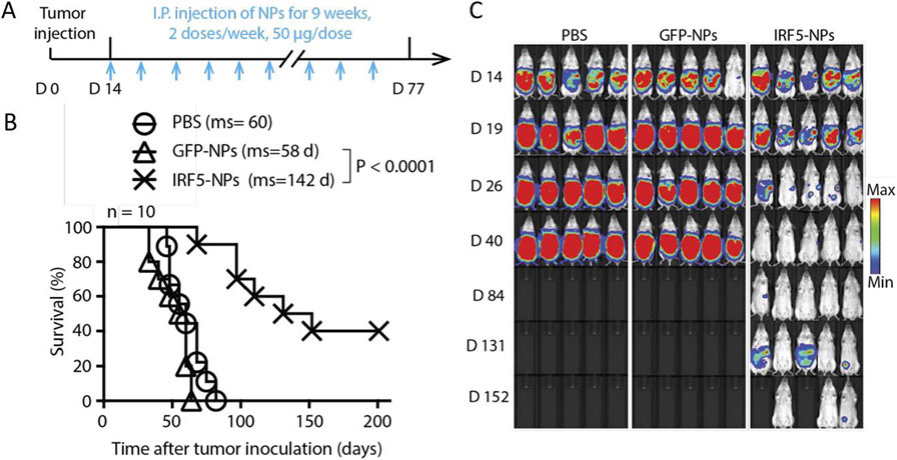
See text for description

**Fig. 4 (abstract 072).**
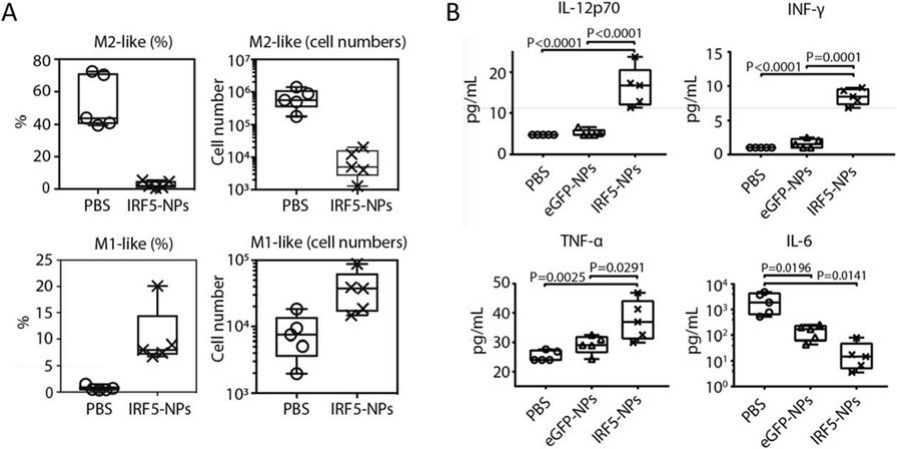
See text for description

**Fig. 5 (abstract 072).**
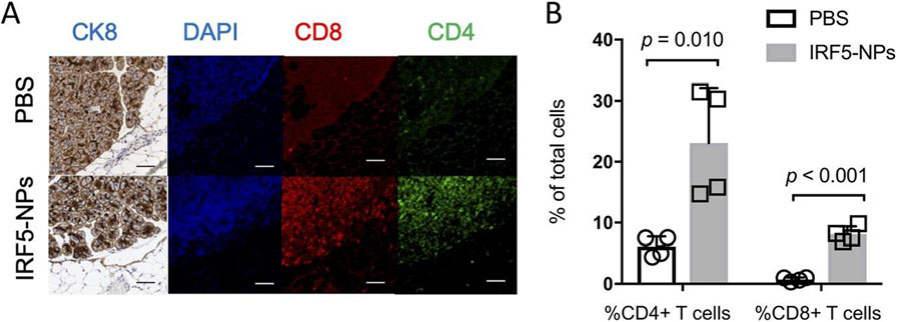
See text for description

### Other

#### O73 Designed improvement to T-cell immunotherapy by integrated single cell profiling.

##### Irfan Bandey, PhD^1^, Jay RT. Adolacion, PhD^1^, Gabrielle Romain, PhD^1^, Melisa Martinez, PhD^1^, An Xingyue, PhD^1^, Arash Saeedi^1^, Rasindu Rajanayke^1^, Ivan Liadi, PhD^1^, Harjeet Singh, PhD^2^, Laurence Cooper, MD, PhD^2^, Navin Varadarajan, PhD^1^

###### ^*1*^*University of Houston, Houston, TX, United States* ; ^*2*^*University of Texas MD Anderson Cancer Center, Houston, TX, United States*

####### **Correspondence:** Navin Varadarajan (nvaradarajan@uh.edu)

**Background**

CD19 specific CAR T-cells have shown promising results against B cell lymphoid malignancies. However, data from these trials have revealed suboptimal responses in some patients. Therefore, besides the need for developing new improved CAR T-cells for the treatment of liquid/solid tumors, a more fundamental insight into the dynamic functionality of CAR T-cells especially at single cell and molecular level is warranted [1-9].

**Methods**

Timing assays, RNAseq, phenotyping, cytokine secretion, cytotoxicity assay and mouse xenograft studies.

**Results**

We were functionally able to categorize CD19-specific CAR T-cells (19-28z) into serial, mono and no-killers. Integrated single-cell functional and transcriptional profiling demonstrated that at the single-cell level, killer T cells showed differential upregulation of transcripts including FASLG, CD2, CD27, HAVCR2, TNFRSF9, and TGFB1 with concomitant upregulation of LEF1, RORA, TCF7, CD69, IL2RA and GZMA transcripts in non-killer T cells. Since the abundances of the cytotoxic proteins were not different between these two subsets of cells, we utilized kinetic modeling of the contact time distributions between the T cells and tumors, and lysosome polarization experiments. These data revealed that the inability of CAR T-cells to kill is due to failures at multiple steps including those involved in lysosomal polarization and degranulation. Since CD137 and TIM3 (HAVCR2) were differentially expressed on killers, we sought to quantify the levels of these proteins using flow cytometry. These experiments revealed that upon interaction with NALM-6, 19-28z T-cells showed significant increase in protein expression of TIM3 and CD137, and these were, higher in degranulating subset (CD107a+) compared to CD107a- cells, indicating induction of CD137 expression in killer T cells upon incubation with target cells. Long-term killing experiments (48-72h) demonstrated that addition of exogenous CD137L enabled sustained killing, confirming that CD137 expression is inducible in killer CAR T cells and receptor ligation promotes sustained killing. RNA-sequencing of CD137+ cells illustrated a specific enrichments of glycolysis, mitochondrial biogenesis and proliferative pathways. Based on these results, we explored the effects of overexpression of co-stimulatory molecule 4-1BBL in 19-28z T-cells. Our mouse xenograft studies show that genetically engineered 19-28z-41BBL T-cells demonstrate better tumor regression in tumor models, at lower doses compared to 19-28z T-cell.

**Conclusions**

We have demonstrated that the failure of CAR T cells to kill is related to the failure in multiple steps leading to degranulation. Our data also shows that CD137 is inducible receptor within killer 19-28z T cells and that triggering upon induction can lead to sustained responses in vitro and in vivo.

**Acknowledgements**

Tiejuan Mi. Research Investigator Division of Pediatrics, University of Texas MD Anderson Cancer Center, for helping us with imaging and injection of CAR T cells in mice.

**References**

1. Sabatino, M. et al. Generation of clinical-grade CD19-specific CAR-modified CD8+ memory stem cells for the treatment of human B-cell malignancies. Blood 128, 519-528, (2016).

2. Singh, N., Perazzelli, J., Grupp, S. A. & Barrett, D. M. Early memory phenotypes drive T cell proliferation in patients with pediatric malignancies. Sci Transl Med 8, 320ra323, (2016).

3. Klebanoff, C. A. et al. Determinants of successful CD8+ T-cell adoptive immunotherapy for large established tumors in mice. Clin Cancer Res 17, 5343-5352, (2011).

4. Mukherjee, M., Mace, E. M., Carisey, A. F., Ahmed, N. & Orange, J. S. Quantitative imaging approaches to study the CAR immunological synapse. Molecular Therapy 25 %6, 1757-1768 %&, (2017).

5. Bailey, R. C., Eadie, G. S. & Schmidt, F. H. Estimation Procedures for Consecutive First Order Irreversible Reactions. Biometrics 30, 67, (1974).

6. Floyd, D. L., Harrison, S. C. & van Oijen, A. M. Analysis of Kinetic Intermediates in Single-Particle Dwell-Time Distributions. Biophysical Journal 99, 360-366, (2010).

7. Subramanian, A. et al. Gene set enrichment analysis: a knowledge-based approach for interpreting genome-wide expression profiles. Proc Natl Acad Sci U S A 102, 15545-15550, (2005).

8. Delignette-Muller, M. L. & Dutang, C. fitdistrplus: AnRPackage for Fitting Distributions. Journal of Statistical Software 64, (2015).

9. Singh, H. et al. Reprogramming CD19-specific T cells with IL-21 signaling can improve adoptive immunotherapy of B-lineage malignancies. Cancer Res 71, 3516-3527, (2011).

### Tumor and Stromal Cell Biology

#### O74 Fibroblast activation protein is expressed by human and murine leukocytes and nonspecific inhibition of FAP enhances anti-PD-1 therapy in murine models of PDAC

##### Louis Weiner, MD, Allison Fitzgerald , Shangzi Wang, PhD

###### Georgetown University, Washington, DC, United States

####### **Correspondence:** Allison Fitzgerald (ao546@georgetown.edu)

**Background**

Immunotherapy has been largely ineffective in pancreatic cancer, partially due to the dense stromal fibrosis surrounding the tumor that creates an immunosuppressive microenvironment. The main cellular components of this fibrosis, activated pancreatic stellate cells (aPSCs), are marked by elevated expression of fibroblast activation protein (FAP). Here we investigate the relationship between aPSCs, human natural killer (NK) cells and FAP.

**Methods**

To assess the relationship between aPSCs and NK cells we used a novel in vitro co-culturing system that utilizes primary donor-derived PSCs and human NK cell lines, NK92, NKL, YT and KHYG-1. We tested the ability of NK cells to kill aPSCs using CytotoxGlo and Annexin V assays. We monitored FAP expression using rt-qPCR, western blot and FAP activity assays.

To assess the effects of FAP inhibition we used a non-specific FAP inhibitor, talabostat, in vivo. Forty female C57BL/6 mice were injected subcutaneously with 1X105 syngeneic MT3-2D cells (Kras+/G12D, p53+/-R172H derived from a PDAC KPC GEMM model [1]). Once tumors reached 40-50 mm3, ten mice per group were given either 30 μg of talabostat per mouse daily by oral gavage, 200 μg of anti-PD-1 per mouse twice a week by i.p., both, or neither. Control mice were treated with PBS. Treatment was terminated after 4 weeks and the mice were monitored, with tumor measurements occurring weekly. Experiment was repeated with 15 mice per group and tumors were collected for flow immunophenotyping and mRNA expression analysis. Blood was collected weekly to monitor cytokine levels.

**Results**

We demonstrate that the human NK cell line (NK92) is activated by and kills aPSCs (Figure 1). Upon direct contact with PSCs, PSCs downregulate FAP expression and NK92 cells upregulate FAP. We further demonstrated that FAP is expressed in a variety of human and murine leukocyte cell lines as well as healthy-donor derived NK cells (Figure 2). The addition of a nonspecific FAP inhibitor, Talabostat, enhanced anti-PD-1 therapy efficacy in murine models of PDAC. Cytokine analysis demonstrated that talabostat treatment enhanced NK activating cytokines. Flow cytometry immunphenotyping showed that combination treatment reduced T cell infiltration but increased NK cell infiltration.

**Conclusions**

This is the first report of FAP expression in multiple human and murine leukocyte lineages. It further suggests that talabostat, a non-specific FAP inhibitor, can enhance NK activating cytokine production and that talabostat enhances anti-PD-1 efficacy through increased recruitment of NK cells to the tumor (Figure 3).

**Acknowledgements**

This work was funded by: P30 CA51008 (LMW), R01 CA50633 (LMW), F30 CA239441 (AO), GESR Nanostring Grant (AO)

**References**

1. Boj SF, Hwang C-I, Baker LA, Chio IIC, Engle DD, Corbo V, Jager M, Ponz-Sarvise M, Tiriac H, Spector MS, et al. Organoid Models of Human and Mouse Ductal Pancreatic Cancer. Cell (2015) 160:324–338.

**Ethics Approval**

This study was approved by Georgetown University’s IACUC, protocol #2016-1254

**Fig. 1 (abstract 074).**
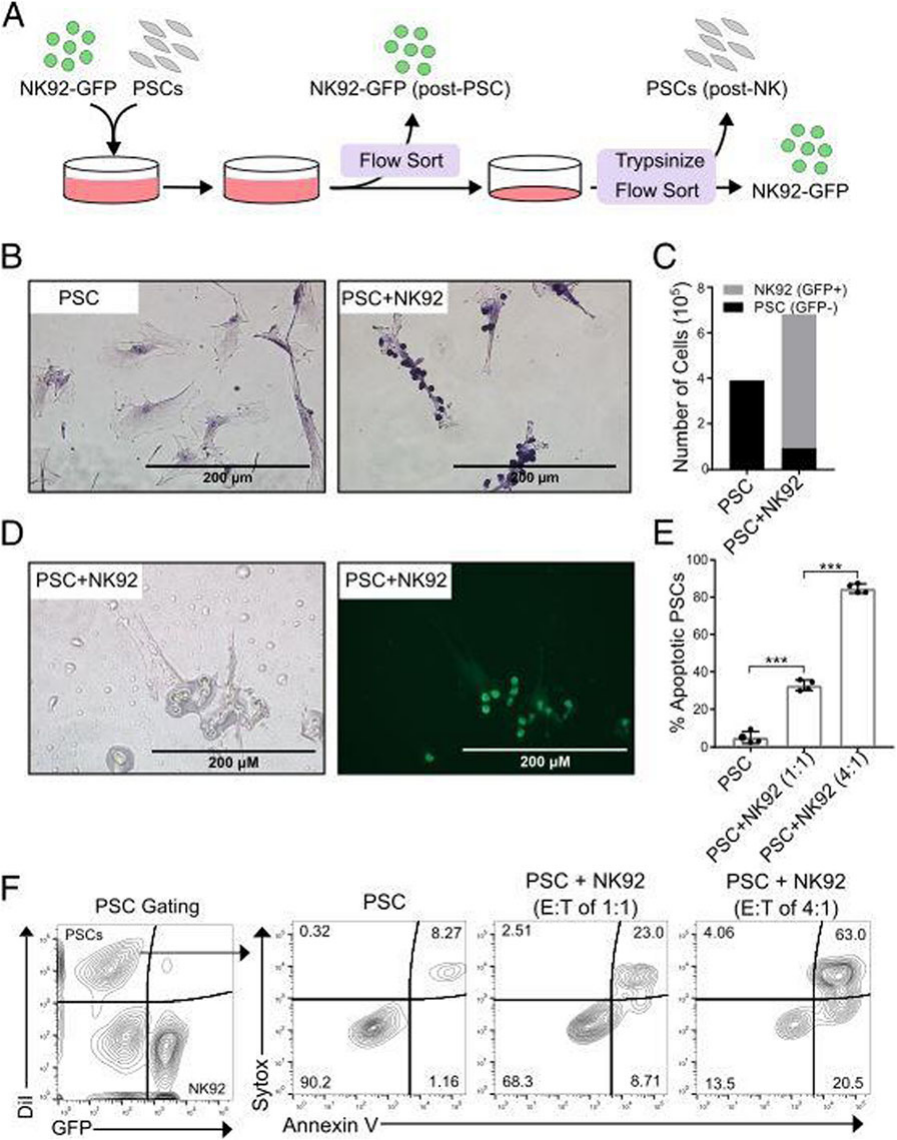
NK92 cells bind to and kill human PSCs.

**Fig. 2 (abstract 074).**
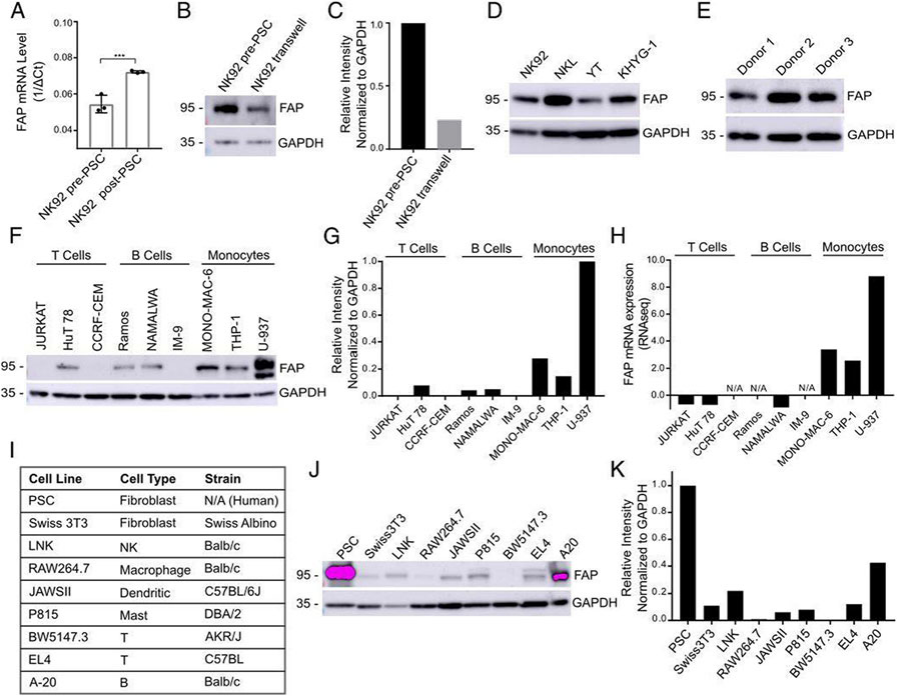
Human and murine leukocytes express FAP.

**Fig. 3 (abstract 074).**
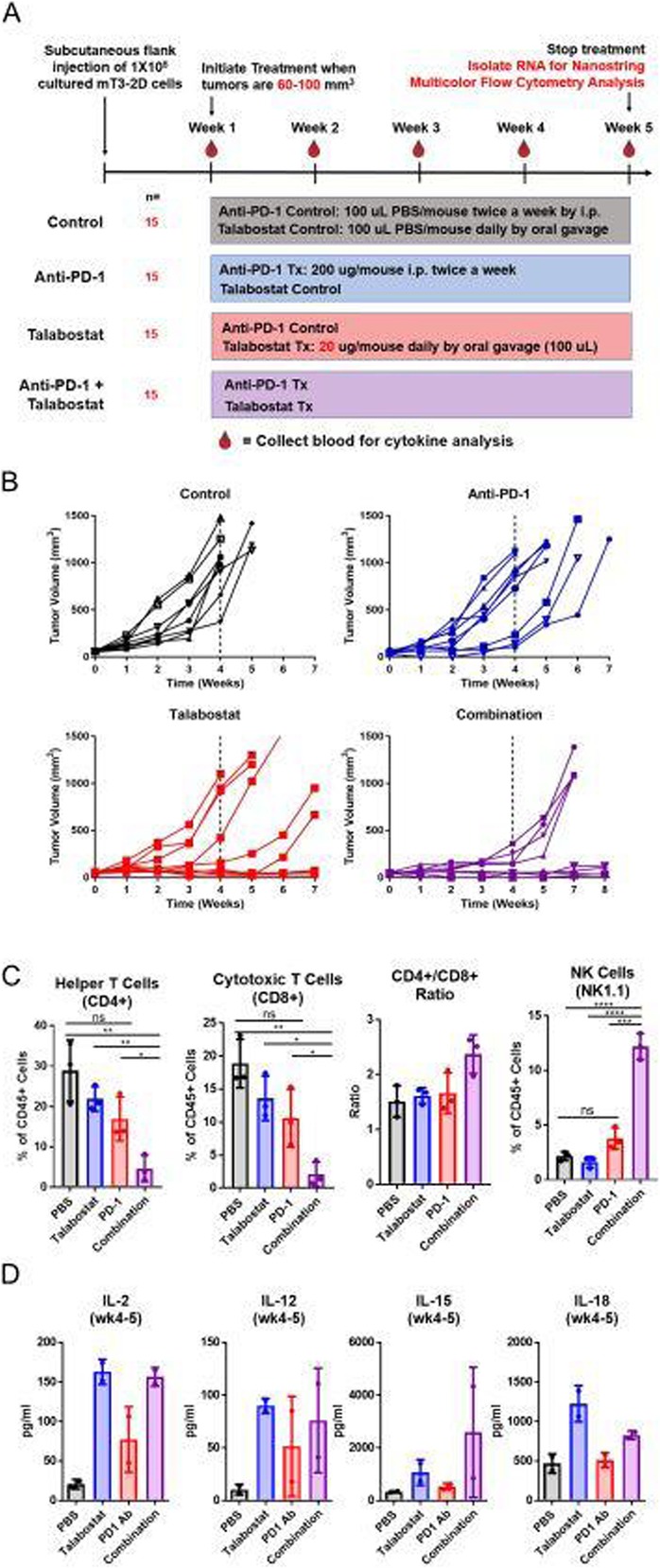
Talabostat enhances anti-PD-1 efficacy

#### O75 Selective induction of S100a8/a9 heterodimer protein in pancreatic cancer in response to immune selection pressure

##### Louis Weiner, MD, Reham Ajina, MS

###### Georgetown University, Washington, DC, United States

####### **Correspondence:** Louis Weiner (weinerl@georgetown.edu)

**Background**

Pancreatic ductal adenocarcinoma (PDAC) is a leading cause of cancer-related deaths worldwide. Host immunity plays a critical role in pancreatic cancer development and progression. However, there is no FDA approved immunotherapy for pancreatic cancer yet. The aim of this study was to investigate the effects of immune selection pressure on pancreatic cancer immunoevasion. S100a8/a9, which belong to the S100 calcium-binding protein family, has been shown to enhance the progression and metastasis of many cancer entities [1], including PDAC [2,3]. While the downstream effects of S100a8/a9 have been extensively studied, the role of immune selection pressure in the regulation of S100a8/a9 expression has never been reported.

**Methods**

1 X105 mT3-2D murine pancreatic cancer cells (Kras+/G12D, p53+/-R172H) [4] were injected subcutaneously into syngeneic C57BL/6J (WT), B6.CB17-Prkdcscid/SzJ (SCID) and T cell-depleted WT mice (10 mice/group). T cell depletion was performed using anti-CD4 (GK 1.5 BioXCell) anti-CD8 (2.43 BioXCell), with a dose of 200ug/mouse, twice weekly. Tumor growth was monitored over time, and 1 cm3 tumors were then harvested and processed for global proteomics profiling using NanoUPLC-MS/MS mass spectrometry (10 tumors/group were combined as one sample with two technical replicates), and transcriptomics profiling using NanoString nCounter (5 samples/group). The results were validated by immunohistochemistry (IHC) and Western blotting. T cell infiltration was evaluated using IHC (5-7 samples/group) and flow cytometry (8 samples/group).

**Results**

mT3-2D tumors engrafted in WT mice grow more slowly as compared with SCID and T cell-depleted tumors. While this observation indicates that the reduced mT3-2D tumor growth rate in WT mice is T cell dependent, it suggests that PDAC can develop immunoevasion mechanisms. T cells moderately infiltrate WT tumors (Among all CD45+ immune cells, 4% are CD4 T cells and 2% are CD8 T cells). Global proteomics profiling determined that S100a8 and S100a9 are selectively induced in WT tumors compared to SCID tumors. Similarly, NanoString nCounter analysis shows that S100a8 is significantly higher in WT tumors compared with SCID tumors (p-value of Kruskal-Wallis test is 0.0091). The induction of S100a8 and S100a9 was further validated by IHC (p-value of Mann Whitney test is 0.0424 and 0.0043, respectively). Interestingly, Western blotting analysis indicates that the S100a8/a9 heterodimer but not S100a8 and S100a9 monomers is selectively expressed in WT tumors.

**Conclusions**

Using the mT3-2D model, T cell immunity incompletely controls PDAC tumor growth. Our data shows that S100a8/a9 heterodimer is selectively induced in response to T cell selection pressure, suggesting its role in PDAC immunoevasion.

**References**

1. Hanahan D, Weinberg RA. Hallmarks of Cancer: The Next Generation. Cell, 2011; 144; 646–674.

2. Srikrishna G. S100A8 and S100A9: New Insights into Their Roles in Malignancy. J Innate Immun., 201; 4(1): 31–40.

3. Gammal A, Sturm JH, Pinnschmidt HO, Hofmann BT, Bellon E, Ghadban T, Melling N, Bachmann K, Izbicki JR, Bockhorn M, Güngör C, R Perez DR . Protein S100A8/A9: A Potential New Biomarker for Pancreatic Diseases. International Journal of Clinical Endocrinology and Metabolism, 2017; 3(1): 023-028.

4. Boj SF, Hwang C-I, Baker LA, Chio IIC, Engle DD, Corbo V, Jager M, Ponz-Sarvise M, Tiriac H, Spector MS, et al. 2015. Organoid models of human and mouse ductal pancreatic cancer. Cell 160:324–338.

**Consent**

No contest is needed. However, I am not sure were to indicate the author names and affiliations:

Reham Ajina1, Annie Zuo1, Sandra A. Jablonski1, Louis M. Weiner1.

1 Department of Oncology and Lombardi Comprehensive Cancer Center, Georgetown University Medical Center, 3970 Reservoir Road NW, Washington DC, 20057, USA

#### O76 Interferon-gamma mediated T cell exhaustion generates distinct tumor microenvironments in synchronous melanoma via PD-1/PD-L1 axis

##### Peter Prieto, MD, MPH^1^ , Shuyang Qin, BS^1^, Alyssa Williams^1^, Booyeon Han, BS^1^, Rachel Jewell^1^, Katherine Jackson, MD^1^, Alexander Chacon, MD^1^, Edward Englerth III, BS^1^, Brian Belt, JD^1^, Goran Micevic, MD, PhD^2^, David Linehan, MD^1^, Scott Gerber, PhD^1^, Alexandre Reuben^3^

###### ^*1*^*University of Rochester Medical Center, Rochester, NY, United States* ; ^*2*^*Yale University School of Medicine, New Haven, CT, United States* ; ^*3*^*University of Texas at MD Anderson, Houston, TX, United States*

####### **Correspondence:** Peter Prieto (peter_prieto@urmc.rochester.edu)

**Background**

Despite recent advances in melanoma immunotherapy the presence of synchronous metastatic lesions portends a poor prognosis. Although responses to immune checkpoint blockade can be durable, only a minority of patients display clinically meaningful responses [1]. Inherent tumor heterogeneity secondary to genomic instability results in distinct immune microenvironments and discordant lesion-specific responses [2]. Interferon-gamma (IFN-γ) has been associated with both anti-tumorigenic and pro-tumorigenic activities in melanoma by promoting apoptosis of melanoma cells in vitro while increasing aggressiveness of human melanoma cells in vivo [3,4]. However, the contribution of IFN-γ to heterogeneous PD-1 inhibitor response in synchronous metastatic melanoma has yet to be investigated.

**Methods**

We generated a murine synchronous metastatic melanoma model using a parental YUMM 1.7 cell line, which contains BrafV600E/Pten-/-/Cdkn2-/- driver mutations, and a UVB-irradiated derivative containing de novo somatic mutations, YUMMER 1.7. We characterized the distinct tumor microenvironments via flow cytometry in wildtype and IFN-γ-/- C57BL/6 mice.

**Results**

YUMMER and YUMM establish separate and distinct tumor microenvironments in a synchronous melanoma model with the former being more immunogenic and sensitive to presence of IFN-γ. Presence of YUMMER tumors did not increase immune infiltration in YUMM (p > 0.9) tumors nor increase its sensitivity to IFN-γ. Despite having increased CD8 immune infiltration (p < 0.01), YUMMER tumors eventually escaped immune surveillance. Furthermore, YUMMER tumors contained elevated levels of PD-1+ CD8 T-cells and of PD-L1+ stromal cells. This upregulation of PD-1/PD-L1 axis is abolished in IFN-γ-/- mice as compared to wildtype mice.

**Conclusions**

IFN-γ blunts anti-tumoral CD8 T-cell response via upregulation of PD-1/PD-L1 axis in a model of synchronous metastatic melanoma. The presence of an immunogenic tumor does not exert an abscopal effect on a synchronous, but genetically dissimilar “cold” tumor. Genomic and immune characterization of this synchronous model could potentially provide mechanistic insight and explain lesion-specific responses to immunotherapy observed in this challenging patient population.

**Acknowledgements**

We would like to thank Dr. Marcus Bosenberg from the Department of Dermatology at Yale University for kindly gifting us with the YUMMER 1.7 murine melanoma cell line.

**References**

1. Schachter, J., et al., Pembrolizumab versus ipilimumab for advanced melanoma: final overall survival results of a multicentre, randomised, open-label phase 3 study (KEYNOTE-006). Lancet. 2017; 390(10105): 1853-1862.

2. Reuben, A., et al., Genomic and immune heterogeneity are associated with differential responses to therapy in melanoma. NPJ Genom Med. 2017; 2.

3. Kortylewski, M., et al., Interferon-gamma-mediated growth regulation of melanoma cells: involvement of STAT1-dependent and STAT1-independent signals. J Invest Dermatol. 2004; 122(2): 414-22.

4. Brocker, E.B., et al., Inflammatory cell infiltrates in human melanoma at different stages of tumor progression. Int J Cancer. 1988; 41(4): 562-7.

**Ethics Approval**

Animal experiments were approved by the University Committee on Animal Resources and performed in accordance with University of Rochester approved guidelines.

#### O77 Clinicopathological and genomic correlates of PD-L1 expression in nonsquamous non-small cell lung cancer

##### Giuseppe Lamberti, MD^1^, Liam Spurr^2^, Yvonne Li^2^, Biagio Ricciuti, MD^1^, Gonzalo Recondo, MD^1^, Renato Umeton^1^, Andrew Cherniak^2^, Matthew Meyerson, MD, PhD^2^, Mark Awad, MD PhD^1^

###### ^*1*^*Dana-Farber Cancer Institute, Brookline, MA, United States* ; ^*2*^*Broad Institute of MIT and Harvard, Boston, MA, United States*

####### **Correspondence:** Mark Awad (Mark_Awad@DFCI.harvard.edu)

**Background**

Programmed death-ligand 1 (PD-L1) expression, reported as tumor proportion score (TPS), is the only clinically-available biomarker of response to immune-checkpoint inhibitors in non-small cell lung cancer (NSCLC), but factors associated with PD-L1 expression are not well understood. We aimed to identify clinical and genomic correlates of PD-L1 expression in nonsquamous NSCLC.

**Methods**

We retrospectively collected clinicopathologic and genomic data from patients with nonsquamous NSCLC whose tumors underwent PD-L1 assessment and targeted next generation sequencing by OncoPanel at the Dana-Farber Cancer Institute. Samples were grouped into three PD-L1 TPS categories: negative (TPS <1%), low (TPS 1-49%) and high (TPS ≥50%). Only genomic alterations with oncogenic potential according to OncoKB were included in the analysis. Permutation testing was performed to correct for mutation enrichment accounting for differences in tumor mutation burden (TMB) among groups. Genomic mutations, copy number gains/losses, and chromosomal arm-level gains/losses were compared among PD-L1 expression groups. A q-value <0.25 was considered significant after correction for multiple comparisons.

**Results**

We identified 909 non-squamous NSCLCs with PD-L1 negative (N=304, 33.4%), low (N=326, 35.9%), and high (N=279, 30.7%) expression. Tumors in the PD-L1 high group were more likely to arise in smokers compared to those in the PD-L1 low and PD-L1 negative groups (86% vs 79% vs 78%, respectively; p=0.04) and to be diagnosed at advanced stage IIIB-IV disease (76% vs 67% vs 61%, respectively; p<0.001) (Table 1). The median TMB was higher in the PD-L1 high than in the PD-L1 low and PD-L1 negative groups (median 12.2 vs 10.6 vs 10.6 mutations/megabase, respectively; p<0.001). Compared to PD-L1 TPS ≥50% tumors, those with TPS <1% were more likely to have mutations in STK11 (19% vs 5%; q=0.001), EGFR (23% vs 12%; q=0.003), and CTNNB1 (4% vs <1%; q=0.036) (Figure1). The most significantly enriched gene copy losses associated with PD-L1 negativity occurred at the 9p24.1 locus, which encodes for the CD274 (PD-L1), PDCD1LG2 (PD-L2), and JAK2 genes (q<0.001). Gene amplification in CD274 and PDCD1LG2 were enriched in tumors with high PD-L1 expression (q<0.25). Chromosome 9p arm loss was significantly more frequent in PD-L1 negative samples than in PD-L1 high tumors (23% vs 8%; q=0.04).

**Conclusions**

High PD-L1 expression in non-squamous NSCLC is associated with smoking status, advanced stage at diagnosis, and high TMB. PD-L1 negative tumors are enriched in mutations in STK11, EGFR and CTNNB1 and copy loss of CD274, PDCD1LG2 and JAK2.

**Ethics Approval**

The study was approved by Dana-Farber Cancer Institute's Ethics Board, approval number DF/HCC #02-180

**Table 1 (abstract 077).**
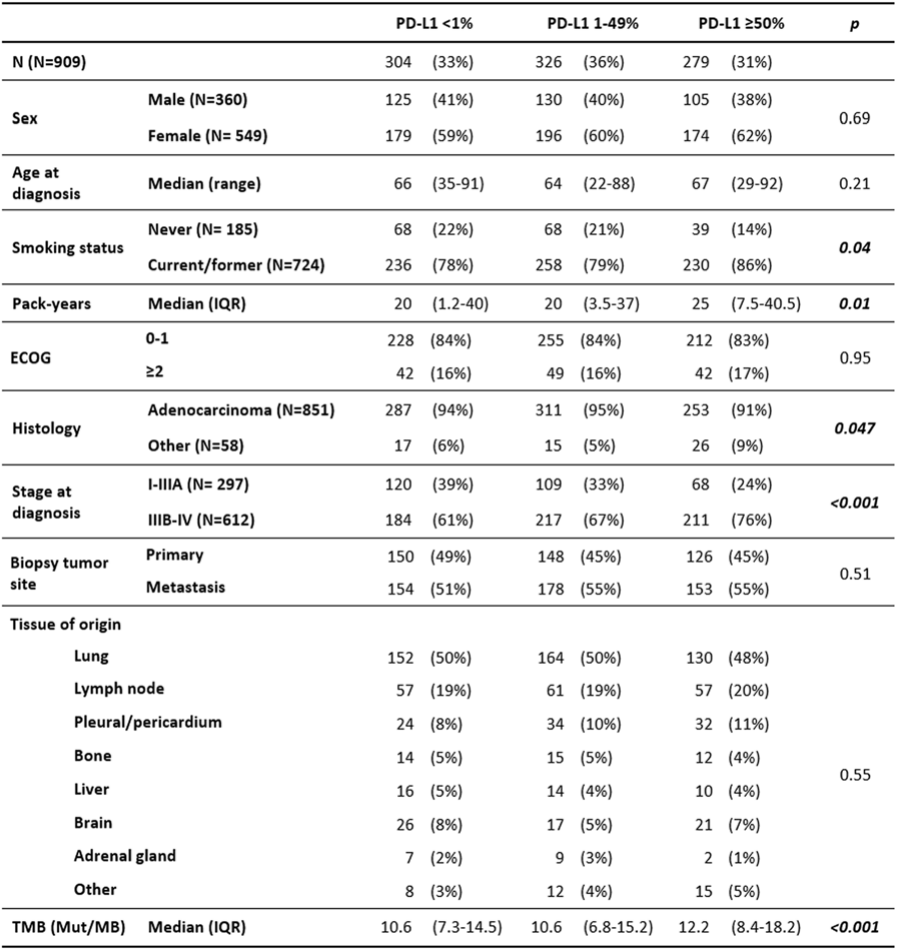
Patient Characteristics

**Fig. 1 (abstract 077).**
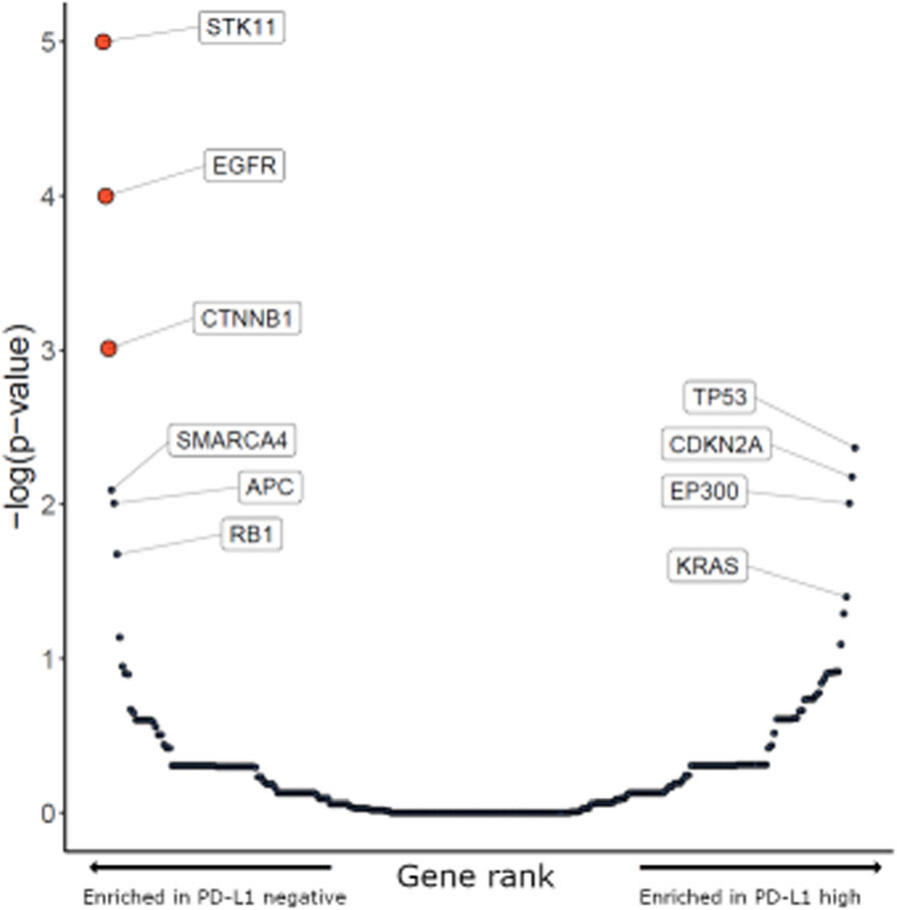
Gene mutations enrichment plot

#### O78 Identification of breast cancer neoantigens exposed by radiation therapy

##### Claire Lhuillier, PhD, Nils Rudqvist, Takahiro Yamazaki, PhD, Lorenzo Galluzzi, Sandra Demaria, MD

###### Weill Cornell Medical College, New-York, NY, United States

####### **Correspondence:** Claire Lhuillier (cfl2002@med.cornell.edu)

**Background**

Recent studies have highlighted the key role of neoantigens generated by somatic non-synonymous mutations in tumor response to immunotherapy [1]. In the BALB/c-derived 4T1 mouse model of immunotherapy-refractory metastatic breast cancer, we have previously shown that tumor-targeted radiation therapy (RT) combined with CTLA4 blockade induces CD8+ T cell-mediated regression of irradiated tumors and inhibits lung metastases [2]. Analysis of the T-cell receptor (TCR) repertoire indicated that unique clonotypes expand in treated tumors, suggesting that tumor rejection involves T cells reactive to a set of tumor antigens that are made available to the immune system by RT [3]. Therefore, we hypothesize that RT increases the expression of genes containing immunogenic mutations and hence promotes priming of neoantigen-specific T cells.

**Methods**

We performed whole-exome sequencing and RNA sequencing of untreated and irradiated (8GyX3) 4T1 cells in vitro to identify tumor-specific neoantigens and determine which ones are upregulated by RT. These mutations were also documented in vivo, in 4T1 tumors harvested before and after treatment (8GyX3 + anti-CTLA4). Dedicated algorithms were used to predict MHC-I and MHC-II-binding epitopes from these mutated genes. Peptides with a predicted affinity

**Results**

Out of 309 total mutations initially identified in 4T1 cancer cells, six peptides able to bind H2Ld or H2Kd in vitro were tested in vaccination experiments, together with two I-Ad-predicted binders. Two MHC-I and one MHC-II neoepitopes were immunogenic, as assessed by IFNγ/TNFα response after T cell re-stimulation. These three neoepitopes were encoded by genes upregulated by RT. Although a vaccine based on these three neoepitopes was not sufficient to inhibit tumor growth, a significant growth delay was seen when vaccination was combined with tumor-targeted RT. TCR repertoire analysis of these tumors revealed significant vaccination-related differences in the frequency of T cell clones, independent of clones specific for the dominant AH1 epitope (derived from the gp70 shared tumor antigen). In addition, in vivo killing experiments demonstrated a potent cytolytic activity of T cells from vaccinated mice towards one of these neoepitopes. Further analyses are ongoing to understand the contribution of these neoantigens to T cell-mediated tumor rejection after targeted RT.

**Conclusions**

These data provide initial proof-of-principle evidence that RT can expose existing neoantigens to the immune system.

**References**

1. Schumacher TN, Schreiber RD. Neoantigens in cancer immunotherapy. Science. 2015; 348(6230):69-74.

2. Demaria S, Kawashima N, Yang AM, Devitt ML, Babb JS, Allison JP, Formenti SC. Immune-mediated inhibition of metastases after treatment with local radiation and CTLA-4 blockade in a mouse model of breast cancer. Clin Cancer Res. 2005: 11(2 Pt 1):728-734.

3. Rudqvist NP, Pilones KA, Lhuillier C, Wennerberg E, Sidhom JW, Emerson RO, Robins HS, Schneck J, Formenti SC, Demaria S. Radiotherapy and CTLA-4 Blockade Shape the TCR Repertoire of Tumor-Infiltrating T Cells. Cancer Immunol Res. 2018: 6(2):139-150.

#### O79 Mapping the spatial architecture of acute myeloid leukemia in the bone marrow microenvironment by multiplexed ion beam imaging

##### Xavier Rovira-Clave, PhD^1^, Sizun Jiang, PhD^1^, Yunhao Bai, BS^1^, Bokai Zhu^1^, Marc Bosse, PhD^1^, Michael Angelo, MD, PhD^1^, Yara Banz, MD, PhD^2^, Christian Schürch, MD, PhD^1^ , Garry Nolan, PhD^1^

###### ^*1*^*Stanford University, Stanford, CA, United States* ; ^*2*^*University of Bern, Bern, Switzerland*

####### **Correspondence:** Christian Schürch (schuerch@stanford.edu), Garry Nolan, PhD (gnolan@stanford.edu)

**Background**

Acute myeloid leukemia (AML) is an aggressive blood cancer that originates from malignantly transformed hematopoietic stem cells in the bone marrow (BM). Many AML patients respond to induction chemotherapy but the majority eventually relapse and succumb to the disease within five years of diagnosis. Multi-parameter single-cell technologies have revealed a high intra- and inter-tumor heterogeneity in AML, and this heterogeneity is in part related to treatment failure and the outgrowth of drug-resistant clones. Therefore, a deeper understanding of the molecular and cellular events that promote heterogeneity and lead to AML relapse is needed.

Emerging evidence indicates that the BM microenvironment plays a crucial role in the pathobiology of AML [1]. This is especially relevant for immunotherapy, which aims to elicit an in situ immune response to eradicate tumors. So far, little is understood about the architecture and spatial cellular composition of the AML BM microenvironment, and how this influences disease initiation, progression, immune responses, and outcome.

**Methods**

We created a tissue microarray (TMA) with formalin-fixed, paraffin-embedded (FFPE) BM samples from 119 AML patients comprising all subtypes of the French-American-British classification. Using an AML-specific metal-conjugated antibody panel, we analyzed the spatial distribution of more than 40 parameters simultaneously by multiplexed ion beam imaging (MIBI) (Figure 1) [2,3].

**Results**

The application of MIBI to study the BM microenvironment identified common immune cell subsets and malignant blasts with immunoprofiles matching the known subtypes of human AML. Unsupervised clustering detected numerous cellular clusters that corresponded to immune and leukemic cell types. Computational deconstruction of these cell clusters and their spatial distribution revealed distinct cellular neighborhoods (CNs, patches of tissue where every cell has a similar surrounding) that were either unique to or shared among specific AML subtypes. Leukemic cells and reactive immune cells expressing checkpoint and activation markers were differentially distributed among the CNs. In addition, CNs with enrichment of stem-like leukemic cells were identified.

**Conclusions**

MIBI enables high-throughput, highly multiplexed analysis of FFPE BM. Mapping the architecture of the BM microenvironment in AML will advance our understanding of this devastating disease and likely provide important information to guide future immunotherapies.

**Acknowledgements**

We thank Matt Newgren for excellent technical support on the MIBI instrument. S.J. was supported by a Stanford Dean’s Fellowship and the Leukemia & Lymphoma Society Career Development Program. X.R.-C. was supported by a long-term EMBO fellowship. B.K. was supported by a Stanford Graduate Student Fellowship. C.M.S. was supported by an Advanced Postdoc Mobility Fellowship from the Swiss National Science Foundation (P300PB_171189 and P400PM_183915), and an International Award for Research in Leukemia from the Lady Tata Memorial Trust, London, UK. This work was further supported by grants from the FDA, the NIH, the Parker Institute for Cancer Immunotherapy, the Bill and Melinda Gates Foundation, and a Rachford and Carlota A. Harris Endowed Professorship to G.P.N.

**Ethics Approval**

The study was approved by the Cantonal Ethics Committee of Bern and was carried out according to national and international guidelines, in accordance with the ethical principles of the Declaration of Helsinki using Good Clinical Practice [4].

**References**

1. van Galen P, Hovestadt V, Wadsworth Ii MH, Hughes TK, Griffin GK, Battaglia S, Verga JA, Stephansky J, Pastika TJ, Lombardi Story J, Pinkus GS, Pozdnyakova O, Galinsky I, Stone RM, Graubert TA, Shalek AK, Aster JC, Lane AA, Bernstein BE. Single-Cell RNA-Seq Reveals AML Hierarchies Relevant to Disease Progression and Immunity. Cell. 2019 Mar 7;176(6):1265-1281

2. Angelo M, Bendall SC, Finck R, Hale MB, Hitzman C, Borowsky AD, Levenson RM, Lowe JB, Liu SD, Zhao S, Natkunam Y, Nolan GP. Multiplexed ion beam imaging of human breast tumors. Nat Med. 2014 Apr;20(4):436-42

3. Keren L, Bosse M, Marquez D, Angoshtari R, Jain S, Varma S, Yang SR, Kurian A, Van Valen D, West R, Bendall SC, Angelo M. A Structured Tumor-Immune Microenvironment in Triple Negative Breast Cancer Revealed by Multiplexed Ion Beam Imaging. Cell. 2018 Sep 6;174(6):1373-1387

4. Galli S, Zlobec I, Schürch C, Perren A, Ochsenbein AF, Banz Y. CD47 protein expression in acute myeloid leukemia: A tissue microarray-based analysis. Leuk Res. 2015 Jul;39(7):749-56

**Fig. 1 (abstract 079).**
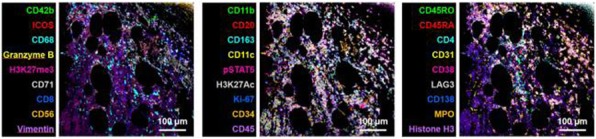
Preliminary MIBI data in diffuse large B-cell lymphoma

#### O80 Atlas of clinically-distinct cell states and cellular ecosystems across human solid tumors

##### Bogdan Luca, Ash Alizadeh, Max Diehn, Aaron Newman , Andrew Gentles, PhD , Chloé Steen

###### Stanford University, Stanford, CA, United States

####### **Correspondence:** Aaron Newman (amnewman@stanford.edu), Andrew Gentles (andrewg@stanford.edu)

**Background**

Tumors are complex ecosystems of malignant, immune, and stromal elements whose dynamic interactions underlie patient survival and response to therapy. A comprehensive understanding of the diversity of cellular states within the tumor microenvironment (TME), and their patterns of co-occurrence, could reveal new diagnostic tools for improved disease management, as well as novel targets for therapeutic intervention.

**Methods**

We applied CIBERSORTx, a recently described “digital cytometry” framework [1], to impute cell type-specific gene expression profiles of cancer cells and 11 major immune and stromal cell types from thousands of solid tumor transcriptomes profiled by The Cancer Genome Atlas (TCGA). We then devised and applied a novel machine learning framework to identify distinct transcriptional states for each cell type, validate them in scRNA-seq data, and uncover co-occurrence patterns between them in order to define tumor cellular ecosystems. We systematically profiled these states across nearly 17,000 solid tumor samples from TCGA, PRECOG [2], in situ transcriptomics arrays, and other publicly available resources, to determined their associations with genomic features, clinical outcomes, and spatial organization.

**Results**

By dissecting cellular heterogeneity across thousands of tumor samples, we defined robust transcriptional states of 12 cell types, including epithelial, fibroblast, endothelial, and 9 immune subsets. These states included both known and novel cellular phenotypes, nearly all of which could be validated in published scRNA-seq tumor atlases. For example, we recapitulated the transcriptional profiles of M1 and M2 polarized macrophages, along with 7 other macrophage states, of which several were strongly associated with overall survival. We found that specific cell states often co-occur in distinct cellular communities with characteristic patterns of ligand-receptor interactions, genomic features, clinical outcomes, and spatial organization. Further analysis of these communities unveiled unique cellular ecosystems that span most solid tumor types. One such “ecotype” defined a normal-like state that was strongly enriched in non-malignant samples. Others delineated novel pro- and anti-tumor inflammatory environments involving specific fibroblast, endothelial, and immune cell transcriptional programs.

**Conclusions**

Large-scale deconvolution of cell type-specific transcriptomes across thousands of solid tumors revealed a comprehensive atlas of cellular states in the TME. The majority were present across multiple tumor types. We found that specific cell states tend to co-occur in a pan-cancer manner, defining robust tumor cellular ecosystems with specific biological features and clinical outcomes. Overall, our results provide a high resolution portrait of cellular heterogeneity in the TME of multiple solid tumor types, with implications for novel diagnostics and immunotherapeutic targets.

**References**

1. Newman, A.M., et al., Determining cell type abundance and expression from bulk tissues with digital cytometry. Nat Biotechnol. 2019; 37(7): 773-782.

2. Gentles, A.J., et al., The prognostic landscape of genes and infiltrating immune cells across human cancers. Nature medicine. 2015; 21(8): 938.

